# Supplementary material for: A temperature-regulated circuit for feeding behavior
Source: Nat Commun. 2022 Jul 22;13:4229. doi: 10.1038/s41467-022-31917-w (PMC9307622; doi:10.1038/s41467-022-31917-w)
Supplement: Supplementary file 1 — Supplementary file [file 41467_2022_31917_MOESM1_ESM.pdf]

Supplementary Information for

A temperature-regulated circuit for feeding behavior

Shaowen Qian[1,2,*], Sumei Yan[1], Ruiqi Pang[1,3], Jing Zhang[4], Kai Liu[2], Zhiyue Shi[1], Zhaoqun Wang[1], Penghui Chen[1], Yanjie Zhang[1], Tiantian Luo[1], Xianli Hu[1], Ying Xiong[1,*], Yi Zhou[1,*]

[1] Department of Neurobiology, Chongqing Key Laboratory of Neurobiology, School of Basic Medicine, Army Medical University, Chongqing, China
[2] Department of Medical Imaging, The 960th Hospital of Joint Logistics Support Force of PLA (Former Jinan Military General Hospital), Jinan, Shandong, China
[3] Advanced Institute for Brain and Intelligence, School of Medicine, Guangxi University, Nanning, Guangxi, China
[4] Department of Radiation Oncology, Tianjin Medical University Cancer Institute and Hospital, National Clinical Research Center for Cancer, Tianjin, China

* Corresponding author:

Yi Zhou, PhD (Lead Contact, E-mail: zhouyisjtu@gmail.com)
Ying Xiong, PhD (E-mail: xiongying2001@163.com)
Shaowen Qian, PhD (E-mail: qianshaowen1110@163.com)

Supplementary Information provided in this file:

Supplementary Figure 1. Food intake and rectal temperature during ambient temperature exposure.
Supplementary Figure 2. C-Fos expressions in multiple preoptic and hypothalamic nuclei in a hunger state.
Supplementary Figure 3. C-Fos expressions in the apMPOA of mice with different dietary states during ambient temperatures.
Supplementary Figure 4. Anterograde tracking of glutamatergic neurons in the apMPOA and the effect of optogenetic terminal activation on food intake and rectal temperature.
Supplementary Figure 5. Feeding behavior under optogenetic activation on the apMPOA-recipient PVH and ARC neurons.
Supplementary Figure 6. Retrograde and anterograde tracing of apMPOA→ARC/PVH pathways for the strategy using high titer AAV1.
Supplementary Figure 7. Effects of the genetic ablation of postsynaptic neurons of the apMPOA→ARC/PVH/DMH pathways on food intake and rectal temperature.
Supplementary Figure 8. Heat maps of GCaMP6s signal changes (ΔF/F) of PVH and

ARC-projecting apMPOA neurons in response to ambient thermal exposure and local stimuli.
Supplementary Figure 9. Anatomical identifications of the apMPOA to ARC/PVH pathways via retrograde labeling.
Supplementary Figure 10. Retrograde labeling for PVH and ARC-projecting apMPOA neurons in vGluT2-cre and GAD2-cre mice.
Supplementary Figure 11. Monosynaptic retrograde RV co-expressed locations for the two subsets of PVH and ARC-projecting apMPOA neurons.
Supplementary Figure 12. Controls for RV experiments.
Supplementary Figure 13. Characterization of Aplnr, GalR1, PVH and ARC-projecting apMPOA neurons that got C-Fos activated by fasting.
Supplementary Table 1. Gene expression values (in FPKM) of ARC and PVH-projecting apMPOA neurons from RNA-seq analysis.
Supplementary Table 2. Differentially expressed genes and their test statistical P values of ARC and PVH-projecting apMPOA neurons from RNA-seq analysis.

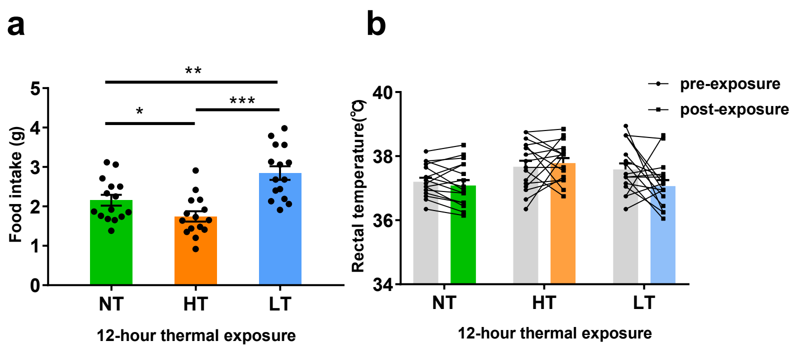

Supplementary Figure 1. Food intake and rectal temperature during ambient temperature exposure

a, Food intake after overnight fasting during three different 12-hour ambient temperature conditions. One-way ANOVA and post hoc least significant difference (LSD) multiple comparison, *p = 0.035, ** p = 0.002, ***p < 0.001, n = 15 animals for each group.

b, Rectal temperature changes after three different 12-hour ambient temperature exposure. Data were statistically analyzed using two-way repeated-measure ANOVA and post hoc LSD multiple comparison, no significance, n = 15 animals for each temperature group. Interaction effect: $F_{2,45}$(temperature × time) = 2.38, p = 0.104; main effect: $F_{2,45}$(temperature) = 4.79, *p = 0.013; $F_{1,45}$(time) = 1.99, p = 0.166.

All error bars show SEM.

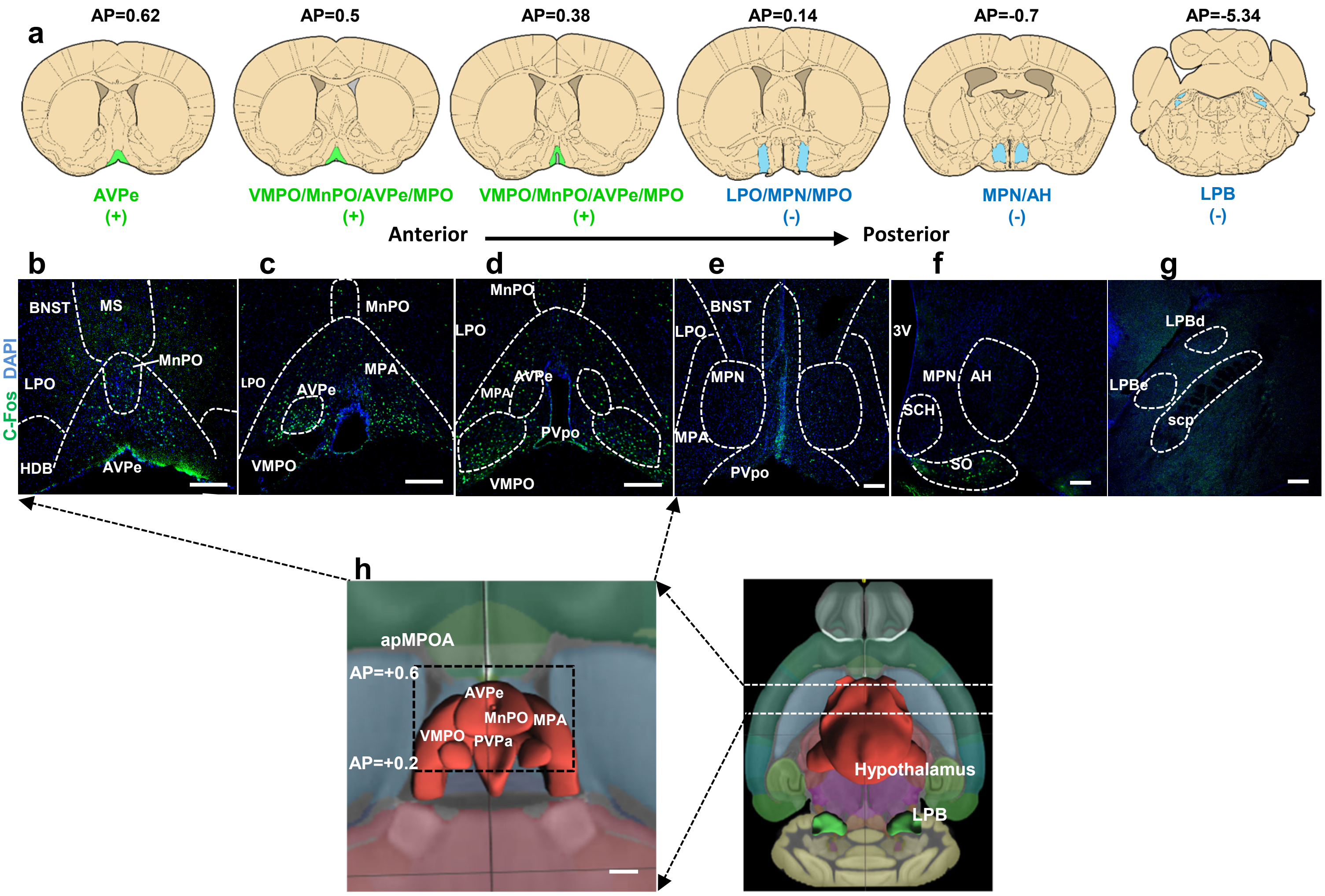

Supplementary Figure 2. C-Fos expressions in multiple preoptic and hypothalamic nuclei in a hunger state

a, Schematic images corresponding to the coronal slices below show C-Fos staining in the hypothalamic nuclei and LPB in a hunger state. The green labeled areas represent C-Fos expression in a hunger state, whereas blue labeled areas do not.

b-d, Multiple hypothalamic nuclei showed C-Fos staining including AVPe, VMPO, anterior MPOA, ventral MnPO, and PVpo. These nuclei are defined as anteroventral and periventricular portions of medial preoptic area (apMPOA) in current study. AVPe, anteroventral periventricular nucleus; VMPO, ventromedial preoptic nucleus; MPOA, medial preoptic area; MnPO, median preoptic nucleus; PVPo, preoptic part of periventricular hypothalamic nucleus; 3V, the 3rd ventricle. The abbreviations can be looked up in Allen Brain Institute Reference Atlas. Image representative of n = 4 mice. Scale bar, 100 µm.

e-g, Other hypothalamic nuclei did not express C-Fos staining including LPO, posterior MPOA, MPN, posterior PVpo, AH, and LPB. LPO, lateral preoptic area; MPN, medial preoptic nucleus; AH, anterior hypothalamic nucleus; LPBe, external part of lateral parabrachial nucleus; LPBd, dorsal part of lateral parabrachial nucleus. Image representative of n = 4 mice. Scale bar, 100 µm.

h, The apMPOA consists of the AVPe, VMPO, anterior MPOA, ventral MnPO, and PVpo in a 3D viewer ranging from approximately AP = + 0.6 mm to AP = + 0.2 mm. Scale bar, 100 µm.

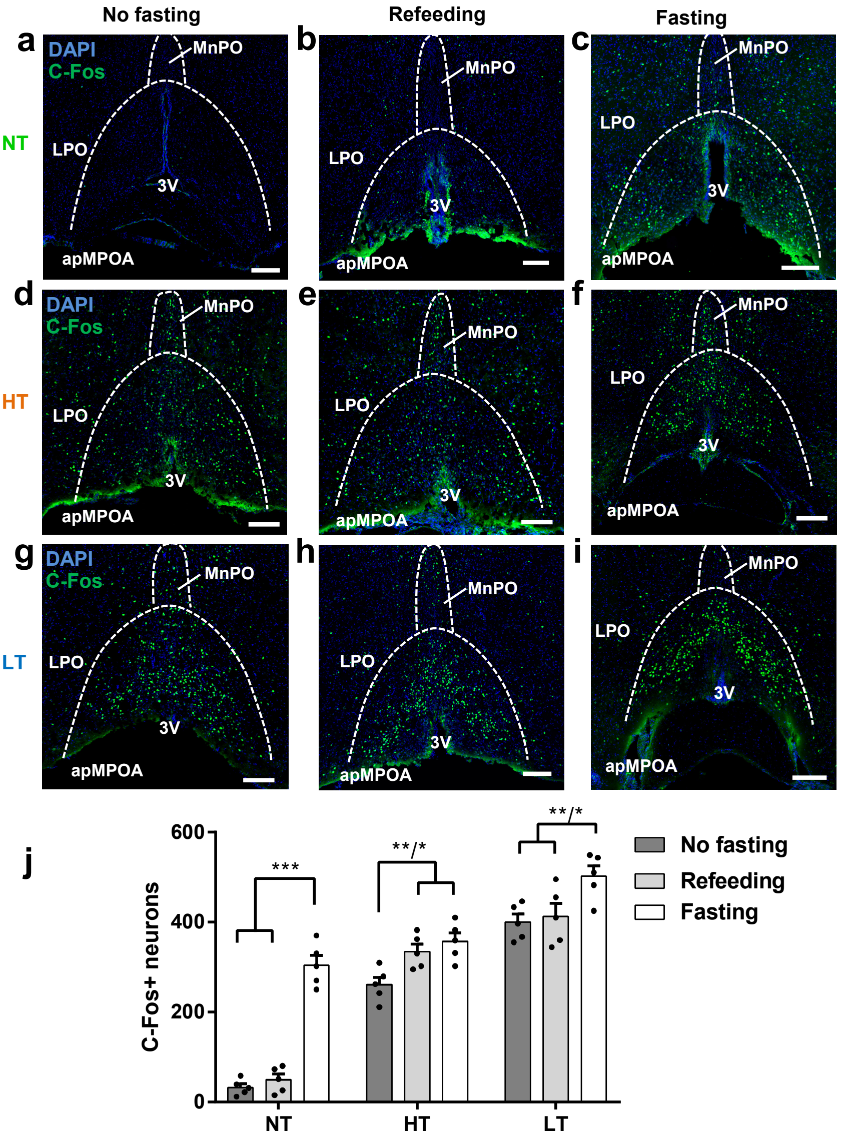

Supplementary Figure 3. C-Fos expressions in the apMPOA of mice with different dietary states during ambient temperatures

a-c, C-Fos expressions in the apMPOA of no fasting (**a**), refeeding (**b**) and fasting (**c**) groups during neutral temperature. No significant C-Fos expressions during neutral temperature for no fasted mice (**a**). Substantial C-Fos expressions in apMPOA were induced by fasting (**c**) but significantly reduced (**b**) after refeeding. Scale bar, 100 μm.

d-f, Substantial C-Fos expressions in the apMPOA during high temperature were found in no fasted (**d**), refed (**e**) and fasted (**f**) mice. Scale bar, 100 μm.

g-i, Substantial C-Fos expressions in the apMPOA during low temperature were found in no fasted (**g**), refed (**h**) and fasted (**i**) mice. Scale bar, 100 μm.

j, Quantitative statistically analysis of C-Fos expressions using two-way ANOVA showed interaction effect of temperature and dietary state: $F(4,24) = 5.25$, $^{**}p = 0.003$; main effect of temperature: $F(2,11) = 219.6$, $^{***}p < 0.001$; main effect of dietary state: $F(2,12) = 67.8$, $^{***}p < 0.001$. Pairwise comparisons showed that more C-Fos expressions in the fasted mice than no fasted and refed mice during all the thermal exposures, especially during the neutral thermal exposure. All error bars show SEM.

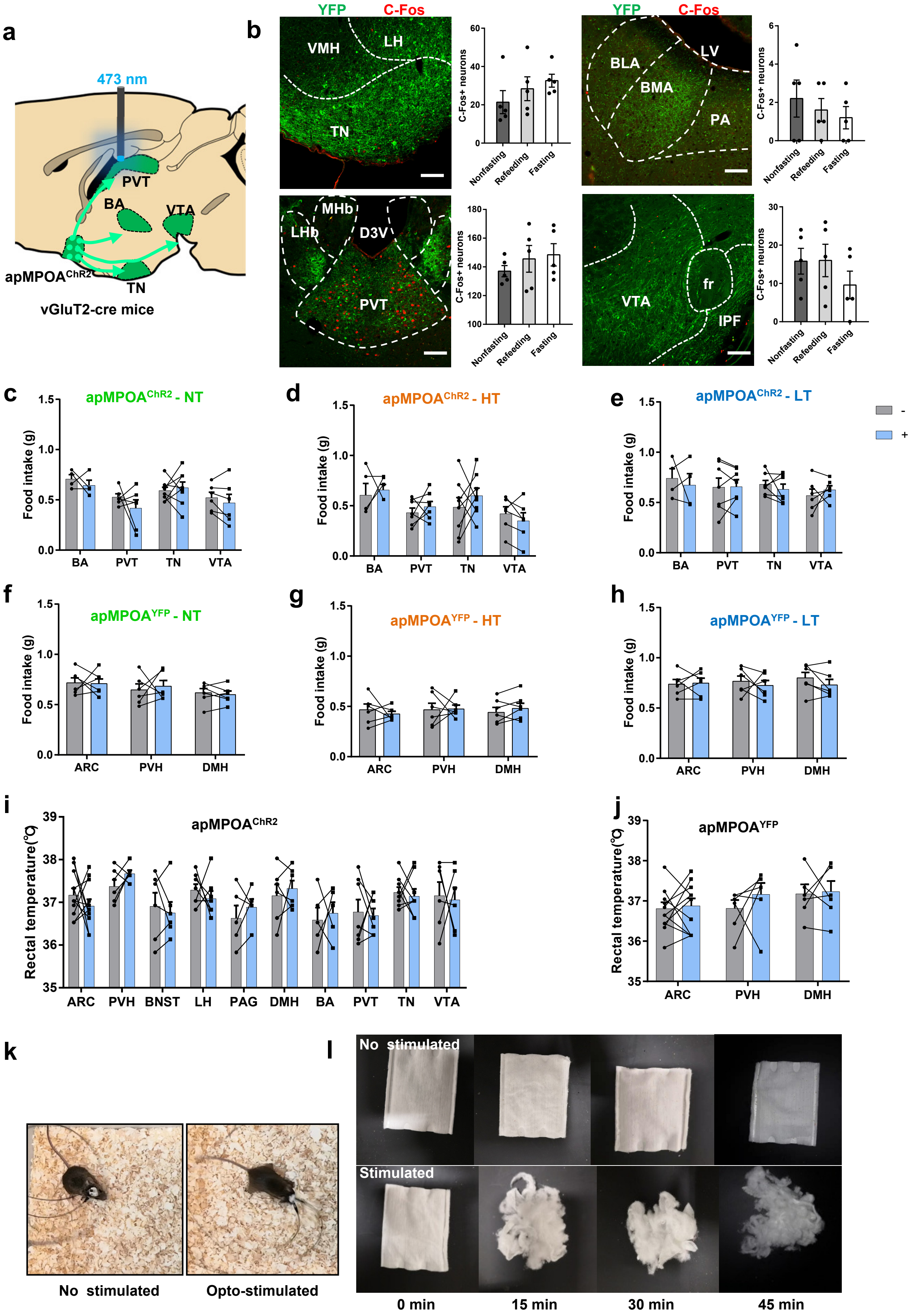

a
473 nm
PVT
BA
VTA
apMPOAChR2
TN
vGluT2-cre mice
b
YFP
C-Fos
VMH
LH
TN
C-Fos+ neurons
Nonfasting
Refeeding
Fasting
BLA
BMA
LV
PA
MHb
LHb
D3V
PVT
VTA
fr
IPF
c
apMPOAChR2 - NT
Food intake (g)
BA
PVT
TN
VTA
d
apMPOAChR2 - HT
e
apMPOAChR2 - LT
-
+
f
apMPOAYFP - NT
ARC
PVH
DMH
g
apMPOAYFP - HT
h
apMPOAYFP - LT
i
apMPOAChR2
Rectal temperature(℃)
ARC
PVH
BNST
LH
PAG
DMH
BA
PVT
TN
VTA
j
apMPOAYFP
k
No stimulated
Opto-stimulated
l
No stimulated
Stimulated
0 min
15 min
30 min
45 min

Supplementary Figure 4. Anterograde tracking of glutamatergic neurons in the apMPOA and the effect of optogenetic terminal activation on food intake and rectal temperature

a, Schematic sagittal image of terminal activation on individual target (PVT shown here), which allows for selective activation on the downstream target of apMPOA.

b, Representative images of target regions innervated by glutamatergic apMPOA neurons (green, ChR2), including the TN, PVT, VTA, and BA, in which no specific fasting-induced C-Fos expression can be observed compared to the re-feeding and no fasting conditions (red, C-Fos). Scale bar, 100 μm.

c-e, Food intake with 2-hour opto-stimulation (+, blue boxes) and without opto-stimulation (-, gray boxes) on the target regions (BA, PVT, TN and VTA) during ambient temperatures. Two-way repeated-measure ANOVA and post hoc LSD multiple comparison, no significance.

f-h, Two-hour food intake with (+, blue boxes) and without (-, gray boxes) opto-stimulation on the PVH, ARC and DMH that expressed controlled YFP during ambient temperatures. Two-way repeated-measure ANOVA and post hoc LSD multiple comparison, no significance.

i, Rectal temperature with opto-stimulation (+, blue boxes) and without opto-stimulation (-, gray boxes) on the downstream terminal fibers of glutamatergic apMPOA neurons expressing ChR2. Paired t tests (two-sided), no significance.

j, Rectal temperature with opto-stimulation (+, blue boxes) and without (-, gray boxes) opto-stimulation on the PVH, ARC and DMH that expressed controlled YFP. Paired t tests (two-sided), no significance.

k, Terminal activation on the DMH induced nest building behavior. Representative photographs of a mouse that is scratching the sawdust in response to opto-stimulation.

l, Representative chewed cotton sheets in response to 45-min opto-stimulation and fresh cotton sheets in no-stimulation condition.

All error bars show SEM.

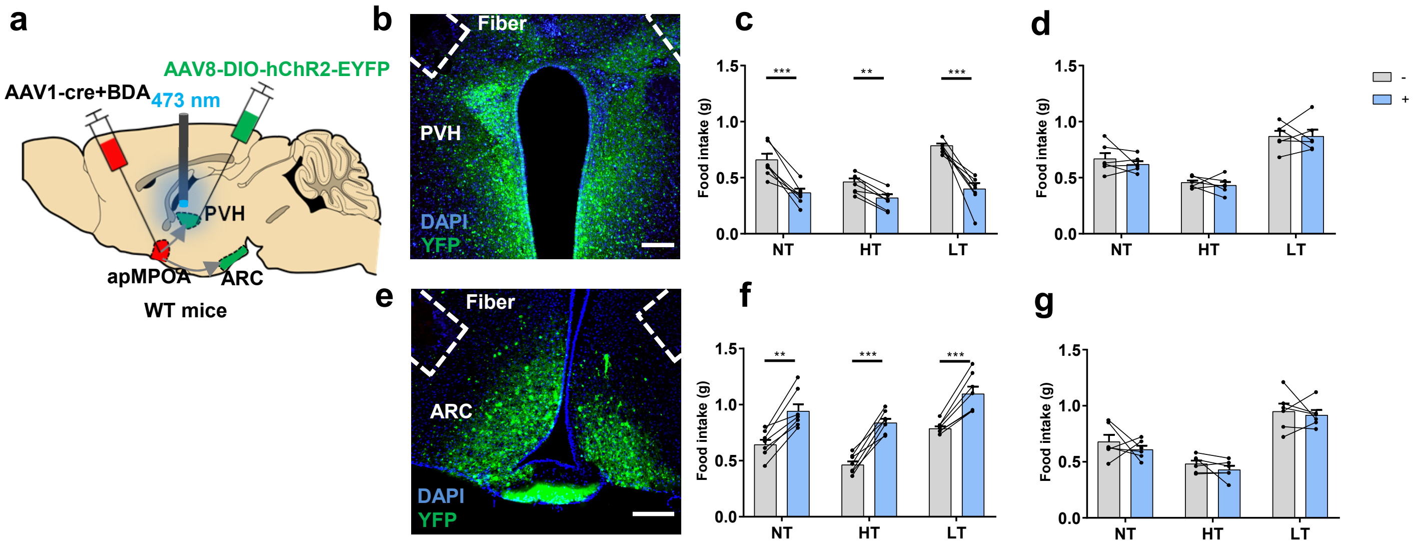

Supplementary Figure 5. Feeding behavior under optogenetic activation on the apMPOA-recipient PVH and ARC neurons

a, Optogenetic activation on apMPOA-recipient PVH and ARC neurons by injecting high titer AAV1-cre into apMPOA and AAV8-DIO-hChR2 into PVH or ARC. BDA was mixed into the virus to indicate injection range.

b, e, Optical fiber implantation, apMPOA-recipient PVH and ARC neurons expressing hChR2-EYFP. Image representative of n = 7 mice. Scale bar, 100 μm.

c, Optogenetic activation on apMPOA-recipient PVH neurons significantly inhibited food intake in all thermal conditions. Interaction effect: $F_{2,11}$ (temperature × treatment) = 7.88, $^{**}p = 0.008$; main effect: $F_{2,11}$(temperature) = 20.8, $^{***}p < 0.001$; $F_{1,12}$(treatment) = 40.1, $^{***}p < 0.001$. Pairwise comparisons showed that optogenetic activating apMPOA-recipient PVH neurons inhibited food intake in all thermal conditions (NT: $^{***}p = 0.001$, HT: $^{**}p = 0.009$, LT: $^{***}p < 0.001$). Within-group tests showed significant simple effect of temperature in control group (F(2,11) = 27.14,$^{***}p < 0.001$), but not in the optogenetic activating group (F(2,11) = 1.54, $p = 0.26$). Two-way repeated-measure ANOVA and post hoc LSD multiple comparison, n = 7.

f, Optogenetic activation on apMPOA-recipient ARC neurons significantly promoted food intake in all thermal conditions. Interaction effect: $F_{2,11}$ (temperature × treatment) = 0.52, $p = 0.61$; main effect: $F_{2,11}$(temperature) = 35.14, $^{***}p < 0.001$; $F_{1,12}$(treatment) = 36.9, $^{***}p < 0.001$. Pairwise comparisons showed that optogenetic activating apMPOA-recipient ARC neurons increased food intake in all thermal conditions (NT: $^{**}p = 0.003$, HT: $^{***}p < 0.001$, LT: $^{***}p = 0.001$). Within-group tests showed significant simple effect of temperature in both of control group (F(2,11) = 20.73,$^{***}p < 0.001$) and optogenetic activating group (F(2,11) = 14.93, $^{***}p < 0.001$). Two-way repeated-measure ANOVA and post hoc LSD multiple comparison, n = 7.

d, g, The controlled groups that expressed YFP in apMPOA-recipient PVH and ARC neurons did not show food intake changes under optogenetic activation. PVH (**d**): interaction effect: $F_{2,9}$(temperature × treatment) = 0.22, $p = 0.81$; main effect: $F_{2,9}$(temperature)=46.83, $^{***}p < 0.001$: $F_{1,10}$(treatment) = 0.34, $p = 0.57$). ARC (**g**): interaction effect: $F_{2,9}$(temperature × treatment) = 0.19, $p = 0.83$; main effect: $F_{2,9}$(temperature) = 82.89, $^{***}p < 0.001$; $F_{1,10}$(treatment) = 0.82, $p = 0.39$. Two-way repeated-measure ANOVA and post hoc LSD multiple comparison, n = 6.

All error bars show SEM.

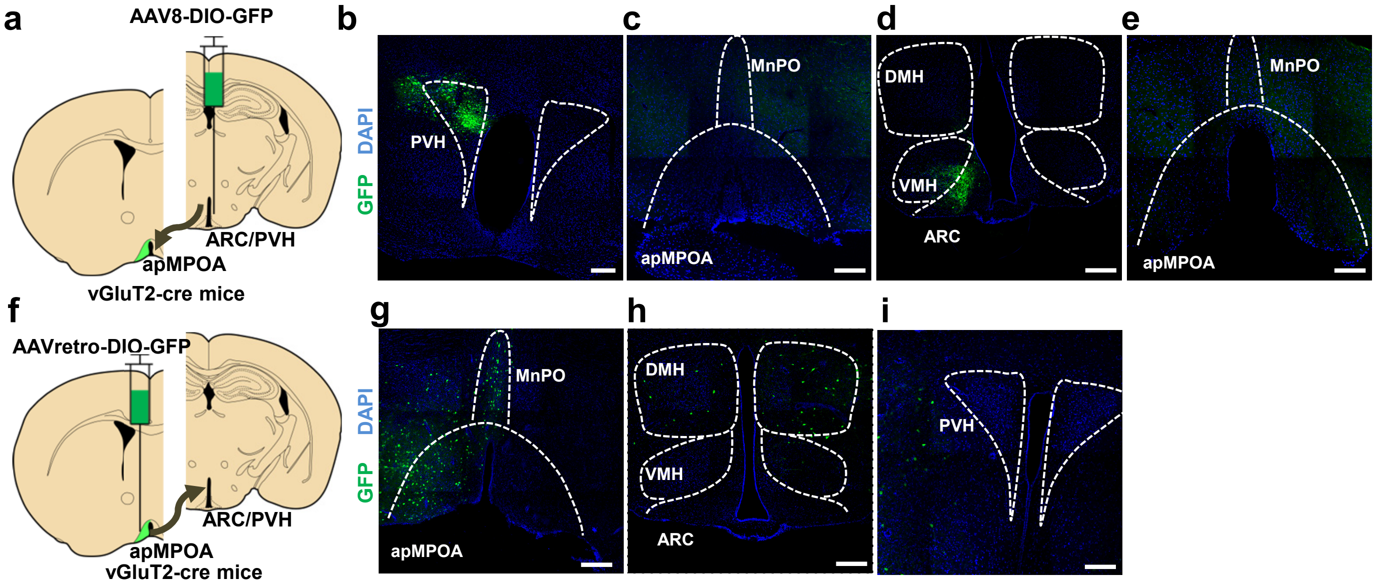

Supplementary Figure 6. Retrograde and anterograde tracing of apMPOA→ARC/PVH pathways for the strategy using high titer AAV1

a, Schematic image of injecting anterograde AAV-syn-GFP into the PVH or ARC to investigate whether there are terminals in the apMPOA innervated by PVH or ARC.

b, d, Schematic coronal images showing neurons expressing GFP in the injection site PVH and ARC. Image representative of n = 4 mice. Scale bar, 100 μm.

c, e, Schematic coronal images showing no projections from PVH or ARC to apMPOA. Image representative of n = 4 mice. Scale bar, 100 μm.

f, Schematic image of injecting retrograde AAVretro-DIO-GFP in the apMPOA of vGluT2-cre mice to investigate whether there are retrogradely labeled neurons in PVH or ARC.

g, Schematic coronal images showing neurons expressing GFP in the injection site apMPOA. Image representative of n = 4 mice. Scale bar, 100 μm.

h, i, Schematic coronal images showing there were no retrograde labelled neurons in the PVH or ARC from the apMPOA. Image representative of n = 4 mice. Scale bar, 100 μm.

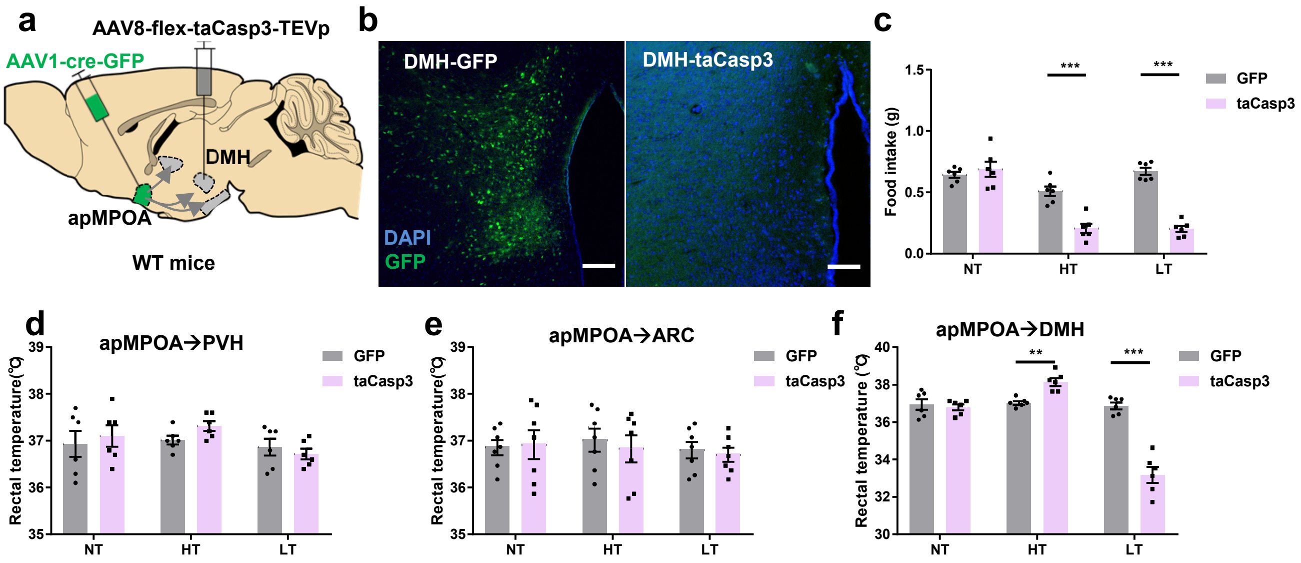

Supplementary Figure 7. Effects of the genetic ablation of postsynaptic neurons of the apMPOA→ARC/PVH/DMH pathways on food intake and rectal temperature

a, Schematic image showing genetic ablation of apMPOA-recipient neurons in ARC/PVH/DMH individually (PVH shown here).

b, Representative images showing preserved transsynaptic neurons in DMH in GFP-injected mice, but ablated transsynaptic neurons in the DMH in taCasp3-injected mice. Image representative of n = 6 mice. Scale bar, 100 μm.

c, The ablation of transsynaptic neurons in DMH did not induce food intake changes under normothermic conditions, but significantly decreased food intake under high and low temperatures. Two-way repeated-measure ANOVA and post hoc LSD multiple comparison. Interaction effect of temperature and treatment group: $^*F(2,9) = 36.29$, $^{***}p < 0.001$; ANOVA main effect of temperature: $F(2,9) = 33.06$, $^{***}p < 0.001$; ANOVA main effect of treatment group: $F(1,10) = 50.33$, $^{***}p < 0.001$. n = 6 mice for each group.

d-e, The genetic ablation of postsynaptic neurons of apMPOA→PVH and ARC pathways had no influence on rectal temperature changes under ambient temperature exposures. Two-way repeated-measure ANOVA and post hoc LSD multiple comparison. **d,** apMPOA→PVH: interaction effect of temperature and treatment group: $^*F(2,9) = 1.76$, $p = 0.227$; main effect of temperature: $F(2,9) = 4.08$, $p = 0.055$; main effect of treatment group: $F(1,10) = 1.15$, $p = 0.309$, n = 6 mice. **e,** apMPOA→ARC: interaction effect of temperature and treatment group: $^*F(2,11) = 0.154$, $p = 0.859$; ANOVA main effect of temperature: $F(2,11) = 0.368$, $p = 0.7$; ANOVA main effect of treatment group: $F(1,12) = 0.126$, $p = 0.729$, n = 7 mice.

f, The genetic ablation of postsynaptic neurons of the apMPOA→DMH pathway resulted in abnormal thermoregulation under ambient temperatures with an increased rectal temperature during high temperature but a decreased rectal temperature during low temperature. Two-way repeated-measure ANOVA and post hoc LSD multiple comparison. Interaction effect of temperature and treatment group: $^*F(2,9) = 37.28$, $^{***}p < 0.001$; ANOVA main effect of temperature: $F(2,9) = 42.43$, $^{***}p < 0.001$; ANOVA main effect of treatment group: $F(1,10) = 25.9$, $^{***}p < 0.001$.

All error bars show SEM.

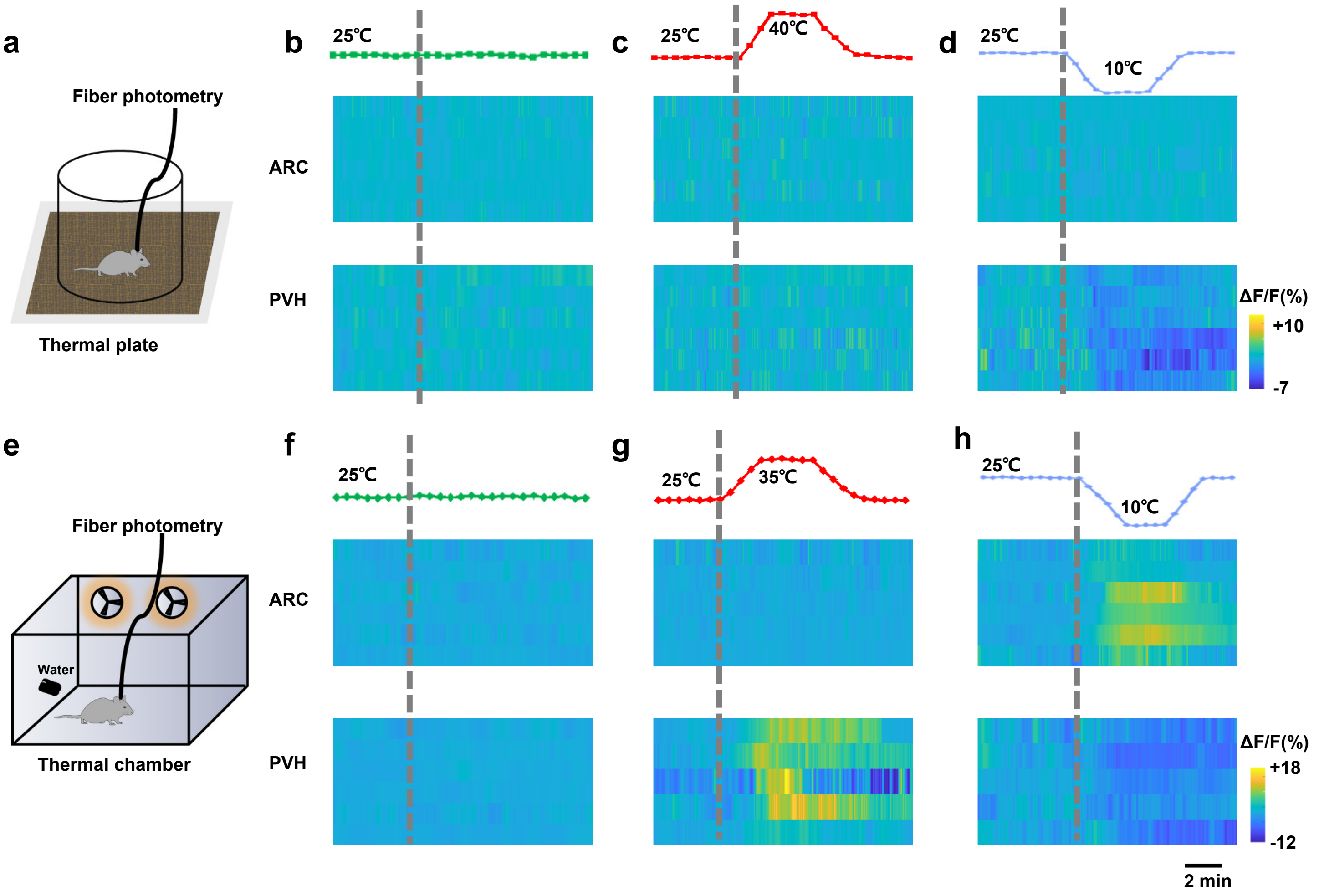

Supplementary Figure 8. Heat maps of GCaMP6s signal changes (ΔF/F) of PVH and ARC-projecting apMPOA neurons in response to ambient thermal exposure and local stimuli

a, Schematic of local thermal exposure on a temperature-controlled plate.

b-d, GcaMP6s signal changes (ΔF/F) of ARC (top) and PVH-projecting (bottom) apMPOA neurons in response to local thermal stimuli: neutral (25 ℃, **b**), hot (25 ℃ →40 ℃, **c**), cold (25 ℃ →10℃, **d**).

e, Schematic of ambient exposure in a temperature-controlled chamber.

f-h, GcaMP6s signal changes (ΔF/F) of ARC (top) and PVH-projecting (bottom) apMPOA neurons in response to ambient exposure: neutral (25° C, **f**), hot (25 ℃ →35 ℃, **g**), cold (25 ℃ →10 ℃, **h**).

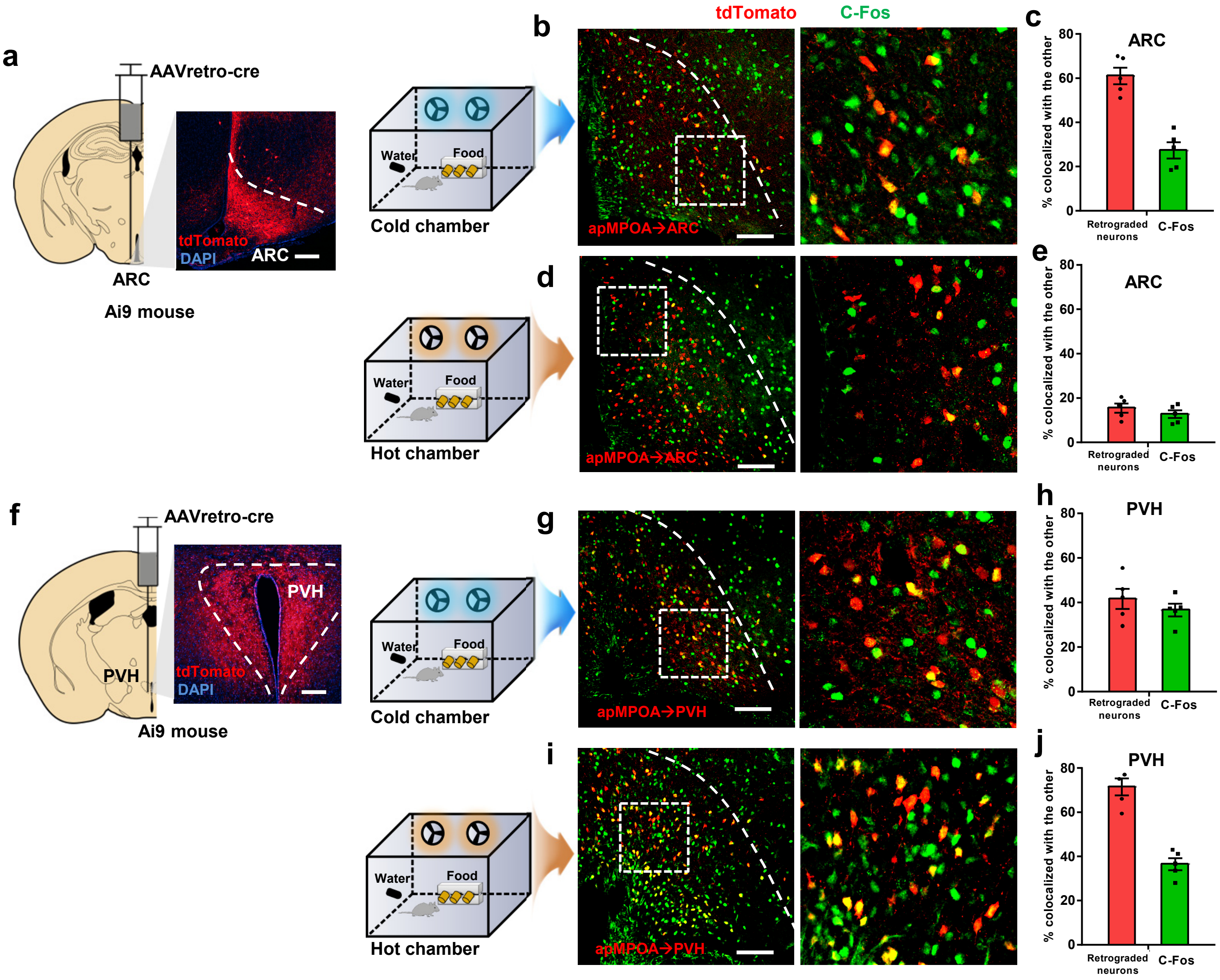

Supplementary Figure 9. Anatomical identifications of the apMPOA to ARC/PVH pathways via retrograde labeling.

a, Schematic and representative coronal images of the injection of AAVretro-cre virus into ARC in Ai9 mice.

b-c, Colocalizations of ambient cooling-induced C-Fos (green) with retrograde-labeled neurons (red) by injecting AAVretro-cre into the ARC of Ai9 mice. The ARC-projecting apMPOA neurons were obviously overlapped with cooling-induced C-Fos (61.2 ± 3.7% ARC-projecting apMPOA neurons expressed C-Fos). Scale bar, 100 μm.

d-e, Colocalizations of ambient hot-induced C-Fos (green) with retrograde-labeled neurons (red) by injecting AAVretro-cre into the ARC of Ai9 mice. The ARC-projecting apMPOA neurons were sparsely overlapped with hot-induced C-Fos (15.7 ± 2.1% ARC-projecting apMPOA neurons expressed C-Fos) Scale bar, 100 μm.

f, Schematic and representative coronal images of the injection of AAVretro-cre virus into the PVH in Ai9 mice.

g-h, Colocalizations of ambient cooling-induced C-Fos (green) with retrograde-labeled neurons (red) by injecting AAVretro-cre into the PVH of Ai9 mice. The PVH-projecting apMPOA neurons were colocalized with the C-Fos induced by low temperature (41.9 ± 4.5% PVH-projecting apMPOA neurons expressed C-Fos). Scale bar, 100 μm.

i-j, Colocalizations of ambient hot-induced C-Fos (green) with retrograde-labeled neurons (red) by injecting AAVretro-cre into the PVH of Ai9 mice. The PVH-projecting apMPOA neurons were obviously colocalized with the C-Fos induced by high temperature (71.7 ± 3.8% PVH-projecting apMPOA neurons expressed C-Fos). Scale bar, 100 μm.

All error bars show SEM.

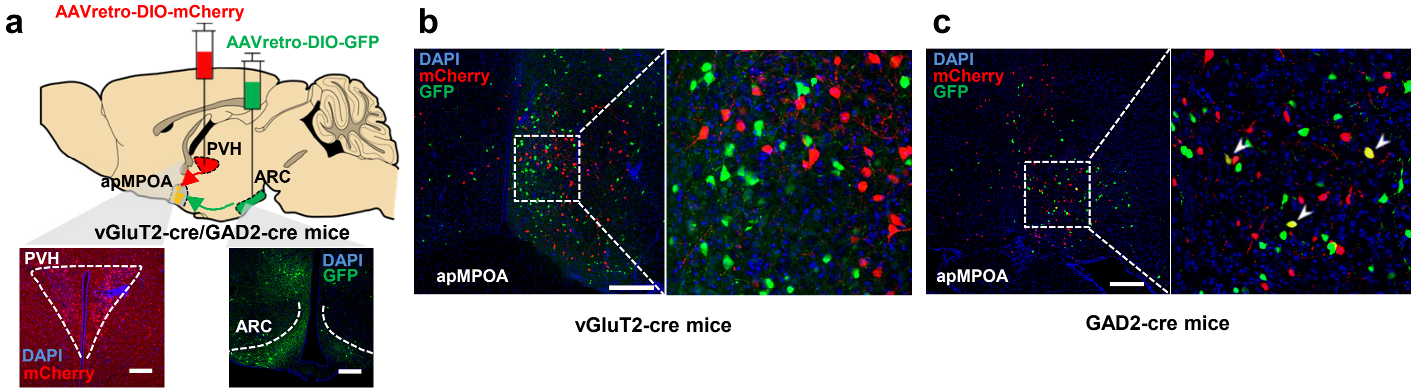

Supplementary Figure 10. Retrograde labeling for PVH and ARC-projecting apMPOA neurons in vGluT2-cre and GAD2-cre mice

a, Schematic and representative coronal images of the injection of AAVretro-DIO-mCherry and AAVretro-DIO-GFP into PVH and ARC of vGluT2-cre and GAD2-cre mice. Scale bar, 100 μm.

b, Retrograde-labeled PVH (red) and ARC (green) projecting neurons in the apMPOA showed no colocalizations in the vGluT2-cre mice. n = 4, Scale bar, 100 μm.

c, Retrograde-labeled PVH (red) and ARC (green) projecting neurons in the apMPOA showed a small fraction of colocalizations in the GAD2-cre mice. n = 4, Scale bar, 100 μm.

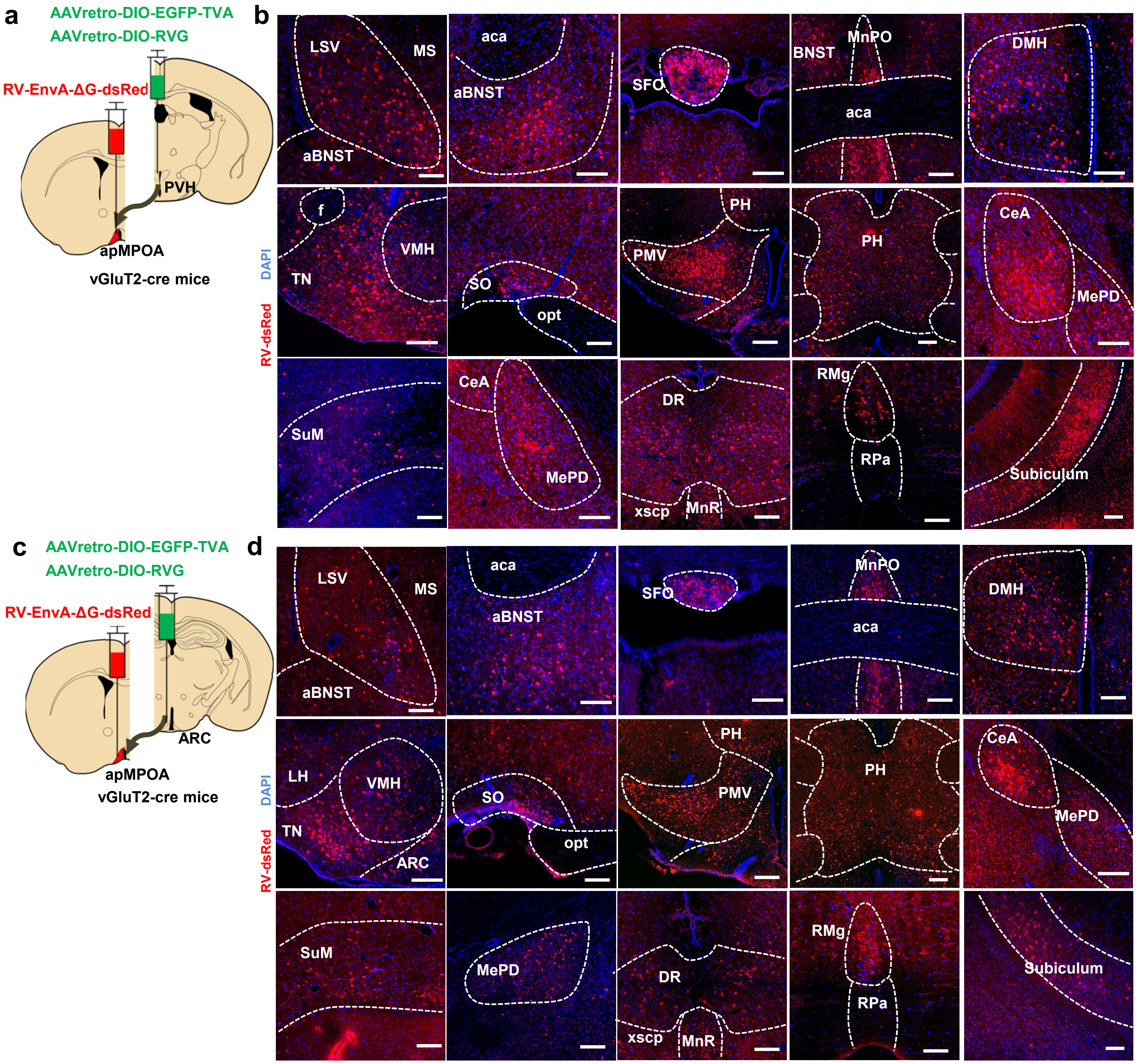

Supplementary Figure 11. Monosynaptic retrograde RV co-expressed locations for the two subsets of PVH and ARC-projecting apMPOA neurons

a, c, Schematic images of the injection of Cre-dependent AAVretro vector expressing RV helpers into the PVH (**a**) or ARC (**c**) on day 1 and RV-EnvA-ΔG-dsRed on day 21 into apMPOA of vGluT2-cre mice

b, d, Retrograde RV co-expressed nuclei for the two subsets of PVH and ARC-projecting apMPOA neurons, including ventral lateral septal nucleus (LSV), anterior BNST (aBNST), subfornical organ (SFO), MnPO, DMH, TN, supraoptic nucleus (SO), ventral premammillary nucleus (PMV), posterior hypothalamus (PH), central amygdaloid nucleus (CeA), supramammillary nucleus (SuM), posterodorsal part of medial amygdaloid nucleus (MePD), dorsal raphe nucleus (DR), raphe magnus nucleus (RMg), and subiculum. MS, medial septal nucleus; aca, anterior part of anterior commissure; f, fornix; opt, optic tract; xscp, decussation of the superior cerebellar peduncle; MnR, median raphe nucleus; RPa, raphe pallidus nucleus. Image representative of n = 4 mice. Scale bar, 100 μm.

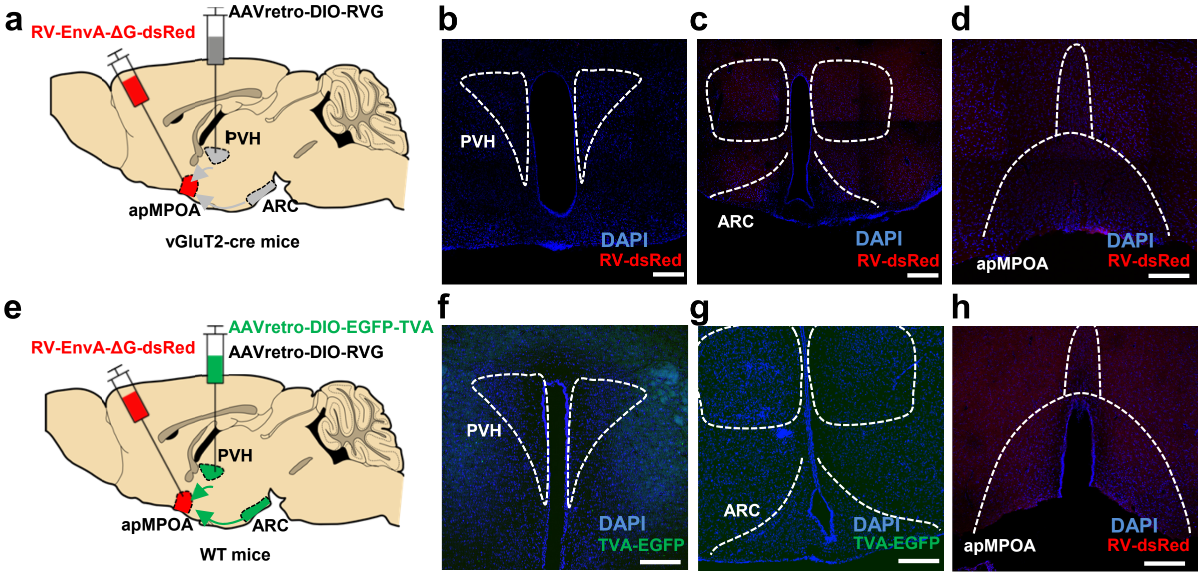

Supplementary Figure 12. Controls for RV experiments

a, Schematic images of injecting AAVretro-DIO-RVG without TVA into the PVH or ARC on day 1 and RV-EnvA-ΔG-dsRed on day 21 into apMPOA of vGluT2-cre mice.

b-d, Coronal images of PVH, ARC and apMPOA showed no dsRed-labeled neurons in the apMPOA or other areas in the brain, indicating the dependence of the RV infection on the TVA expression. Image representative of n = 4 mice for **b** and **c**, and n = 8 mice for **d**. Scale bar, 100 μm.

e, Schematic images of injecting mixed helper viruses with AAVretro-DIO-oRVG and AAVretro-DIO-TVA-EGFP into the PVH or ARC on day 1 and RV-EnvA-ΔG-dsRed on day 21 into apMPOA of wild-type mice.

f-h, Coronal images of PVH, ARC and apMPOA showed no TVA-GFP or RV-dsRed expression in the injection sites or apMPOA, suggesting no leaky expression of the helper viruses or RV. Image representative of n = 4 mice for **f** and **g**, and n = 8 mice for **h**. Scale bar, 100 μm.

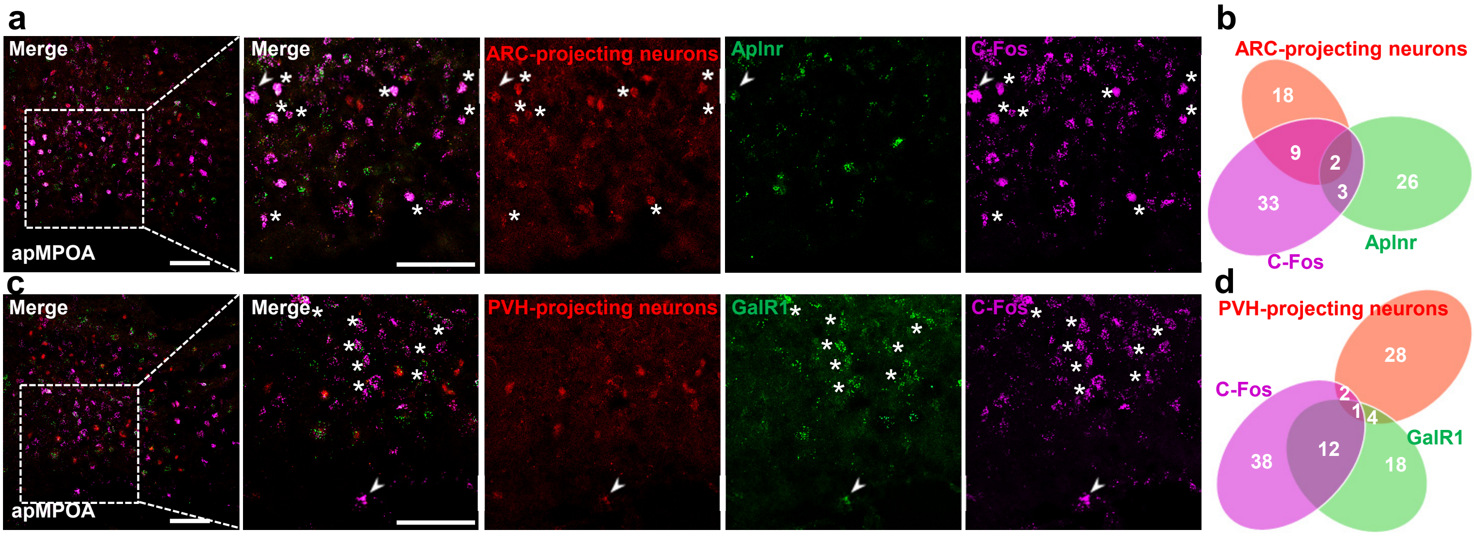

Supplementary Figure 13. Characterization of *Aplnr*, *GalR1*, PVH and ARC-projecting apMPOA neurons that got C-Fos activated by fasting

a, Colocalizations of *Aplnr* (green), ARC-projecting apMPOA neurons (red) and fasting-induced C-Fos (purple). Image representative of n = 5 mice. Scale bar, 100 μm. Arrowhead denotes *Aplnr*+, ARC-projecting+, and C-Fos+ neurons, asterisk denotes ARC-projecting+ and C-Fos+ neurons.

b, Diagram illustrating quantitative counts showed that ARC-projecting neurons present colocalization with fasting-induced C-Fos (approximately 42.5% ARC-projecting neurons), but rarely with apelin receptor (approximately 6.9% ARC-projecting neurons).

c, Colocalizations of *GalR1* (green), PVH-projecting apMPOA neurons (red) and fasting-induced C-Fos (purple). Image representative of n = 5 mice. Scale bar, 100 μm. Arrowhead denotes *GalR1*+, PVH-projecting+, and C-Fos+ neurons, asterisk denotes *GalR1*+ and C-Fos+ neurons.

d, Diagram illustrating quantitative counts showed that PVH-projecting neurons present minor colocalizations with fasting-induced C-Fos (approximately 12.5% PVH-projecting neurons), and galanin receptor 1 (approximately 20% ARC-projecting neurons).

Supplementary Table 1 Gene expression values (in FPKM) of ARC and PVH-projecting apMPOA neurons from RNA-seq analysis. Values from each biological replicate are all included.ARC-projecting apMPOA replicates: A5, A6, A8, A10, A12; PVH-projecting apMPOA replicates: P11, P12, P15, P16, P17

Gene ID	Gene Symbol	A_10	A_12	A_5	A_6	A_8	P_11	P_12	P_15	P_16	P_17
100009600	'Zglp1'	5.17	0.3	0	1.47	0	0	0	0	0.44	0
100009614	'Gm10024'	0	0.19	0	0	0	0	0	0	0	0
100017	'Ldlrap1'	0	0	0	0	0.82	0	0	0	0	0.82
100019	'Mdn1'	1.25	2.58	1.87	2.25	3.13	2.75	2.39	0.21	3.03	1.82
100033459	'Ifi208'	0.13	0.18	0	0.09	0.1	0	0	0	0.01	0.01
100034251	'Wfdc17'	0	0	2.99	0	0	0	0	0	0	0
100034361	'Mfap1b'	75.49	41.7	54	35.3	40.7	39.91	36.6	53.43	33.55	36.55
100034363	'Tmsb15b2'	15.53	8.21	24.1	5.93	10.5	3.27	0.14	6.96	7.06	10.26
100034684	'BC100530'	0	0	0	0.76	0	0	0	0.21	0	0
100036521	'Umad1'	55.47	59.98	54.6	76.5	44.4	60.85	58.7	79	68.03	65.66
100037258	'Dnajc3'	13.63	18.89	13.6	21.7	12.1	25.42	9.84	10.11	19.37	31.45
100037278	'Fam129c'	0.27	0.45	3.36	0	0	0	5.12	1.82	4.82	6.92
100037282	'Rsph3b'	59.44	57.38	30.1	45.5	31.3	35.76	30.5	40.07	27.64	60.44
100037283	'Rnaset2a'	45.74	57.53	52.8	54.9	62.1	63.44	65.9	20.65	32.06	5.96
100038347	'Fam174b'	21.64	19.62	18.5	16.7	22.9	13.75	8.17	27.08	17.44	15.03
100038514	'Gm11837'	5.58	9.32	6.97	20.5	2.43	0.11	0	4.15	3.05	4.68
100038538	'Gm10767'	0.03	6.46	0.29	0	0.12	3.84	3.1	2.41	0	6.29
100038570	'Prcd'	2.57	3.04	1.54	0	11.9	5.66	0	1.59	2.17	3.46
100038725	'Cep85l'	1.82	3.83	2.79	1.6	0.22	2.81	1.37	0.06	2.07	0
100038847	'Gm10406'	15.9	6.82	12.4	2.32	11.4	12.5	4.97	13.11	19.41	8.38
100038860	'Olfr239'	0	0	2.52	0	0	0	0	0	0	0
100038882	'Isg15'	1.74	0	1.62	4.82	0.14	0	0	0.51	4	0.48
100038949	'Gm1979'	0	0.05	0	0	0	0	0.07	0	0	0
100038992	'Gm2001'	0	0	0	0	0	0	1.08	0.67	0	0
100038993	'Gm2002'	0	0.1	0.05	0.34	0.26	0	0	0.64	0.09	0
100039014	'Gm20793'	0	0	0	0	0	0.01	0	0	0	0
100039028	'Mup11'	0	0	0	0	0	0	0	1.75	0	0
100039034	'Gm20795'	0	0	0	0	0	0.01	0	0	0	0
100039042	'Gm2016'	0	0	0	0	0.21	0	0	0	0	0
100039045	'Gm10471'	0.13	0.21	0.03	0	0.75	0.07	0.01	0.08	3.09	0.51
100039053	'Gm2023'	0	0	0	0	0	0	0	0	0	0.48
100039060	'0610010B08Rik'	0	0.15	0.12	0	1.07	0	0.68	0	2.91	0
100039087	'Gm2042'	0	0	0.03	0	0	0	0	0	0	0
100039123	'Gm14295'	175	172.8	149	212	147	205.4	148	169.5	124.6	127.91
100039175	'Gm9780'	0	0	0	0.06	0	0	0	0	0	0
100039192	'Tmem254c'	31.04	6.56	32.1	21.9	10.8	11.97	0	40.26	21.84	53.96
100039227	'Duxbl2'	0.28	0.13	0.56	6.92	0	0.65	0	0.28	0	0.73
100039246	'Plac9b'	0.55	0	3.61	0	0	0	0	3.79	0	0
100039257	'Tmem254b'	30.63	28.04	50.7	70.4	85.7	78.86	86.9	42.19	69.1	58.51
100039284	'Gm2137'	3.09	3.09	2.43	3.07	2.85	1.38	3.76	0.86	9.63	1.88
100039293	'Duxbl3'	0.28	0.42	0.56	4.17	0	0.65	0	0.28	0	0.31
100039441	'Gm2237'	1.15	1.01	0.75	0.22	1.15	0.98	2.1	1.63	4.06	0.27
100039596	'Tcf24'	0	0	0	0.21	0	0	0	0	0	0
100039614	'Gm20809'	0	0	0	0	0	0	0	0	0.65	0
100039707	'Mthfsl'	21.35	16.81	24.4	87.3	22.8	58.19	24.5	21.28	36.78	22.58
100039795	'Ildr2'	6.6	3.14	1.97	5.17	8.72	7.41	0.28	2.59	3.38	8.28
100039815	'Gm2436'	7.49	0	4.19	0.14	4.31	0	0	9.86	0	2.96
100039830	'Gm2446'	0	4.36	0.36	0.14	1.73	0	12.9	0	0	0.75
100039863	'Gm13306'	0	0.98	0.58	6.23	0.33	0.44	0.26	0.96	3.6	0
100039939	'Gm2506'	12.18	5.09	3.48	2.38	6.43	2.12	5.17	6.93	12	6.11
100039968	'Tmem35b'	10.4	5.28	3.6	16	11.2	7.81	10.5	5.32	49.92	16.04
100040048	'Ccl27b'	0	0	0	5.43	0	12.44	0	2.3	7.92	13.74

100040171	'Gm20827'	0	0	0	0	0	0.01	0	0	0	0
100040335	'Gm20836'	0	0	0	0	0	0.01	0	0	0	0
100040462	'Mndal'	0.04	0.12	0.2	0.13	1.44	0.11	0.08	0.04	0.06	0.03
100040500	'Gm2808'	0	0.01	0.02	0.05	0.01	0	0	0	0	0
100040531	'Dynlt1f'	9.33	30.04	32	14	32.2	30.91	8.68	18.45	23.26	18.62
100040545	'Gm2832'	0	0	0	0	1.72	0	0.04	0	0	1.24
100040563	'Dynlt1c'	34.79	52.57	52.9	37.8	76.8	76.46	61.1	54.44	27.85	53.59
100040585	'Gm2854'	0.29	0	0	0	0	0	0	0	0	0
100040591	'Kcnj13'	0	0	0.11	0	0	0.12	0	0.53	0	0
100040599	'Gm15319'	0.01	0.03	0	0	1.15	0.03	0.05	0.27	0	0.01
100040608	'Fancf'	0.05	0	0	0	0.03	1.33	0	0.05	2.51	0
100040657	'Gm2888'	0	0	0	0	0.07	0	0	0	0	0
100040671	'Gm2897'	5.45	8.77	8.63	9.74	5.99	8.63	11	14.89	14.38	5.26
100040697	'Gm10340'	0	0.43	1.31	1.71	0	0	0	1.8	0.33	0
100040722	'Btbd35f13'	0	0	0.89	0	0	0	0	0	0	0
100040732	'Btbd35f14'	0	0	0	0	0.03	0	0	0	0	0
100040766	'Mroh2a'	0	0.01	0	0.06	0	0	0	0	0	0
100040771	'Gm2959'	0	0	0.19	0	0	0.04	0	0	0	0
100040807	'Gm2977'	0.07	0	0.9	0	0.03	0	1.24	2.07	0.47	0
100040870	'Gm3005'	6.77	4.78	4.13	5.17	11.6	2.02	5.8	9.32	12.45	5.85
100040935	'Gm3050'	0.61	0	0.09	0	0.03	0	0.39	1.21	0.38	0
100040944	'Gm3055'	4.34	1.74	4.5	0	1.11	0	1.17	2.47	0	2.39
100040972	'Tceal7'	2.46	0	0	0	5.32	0	0	1.37	4.33	2.92
100041012	'Gm3095'	3.19	3.6	1.69	0.84	0.61	2.42	3.88	4.23	2.89	2.45
100041057	'LOC100041057'	0	0.04	0.05	0	0	0	0.02	0	0	0
100041115	'Gm3147'	0	0	0	0	0	0	0	0	0.04	0
100041151	'Gm3636'	30.53	13.84	13.8	7.3	11.8	5.75	15.6	20.03	29.23	16.2
100041194	'Ahnak2'	0	0	0	0	0.44	0	0.34	0	0	0
100041230	'Hist1h4m'	6.24	2.72	28.1	13.4	1.13	21.14	20	10.47	4.73	12.66
100041256	'Gm20867'	0	0	0	0	0	0	0	0	0.06	0
100041262	'Gm3239'	2.27	1.36	1.1	0.16	1.71	0.29	2.38	2.39	0.44	1.69
100041273	'Ndufb4c'	34.4	31.97	50.3	61.4	36.8	88.95	52.3	36.33	49.86	34.71
100041279	'Gm3248'	4.47	8.39	0.85	0.88	1.42	1.74	0.73	4.28	0.68	3.8
100041294	'Supt4b'	0.24	0.18	0.52	0.29	0.09	0.16	0.06	0.95	0.54	0.31
100041306	'Gm3264'	11.56	7.4	9.25	2.32	10.4	5.15	11.6	17.19	15.56	9.52
100041420	'Gm3325'	1.21	0.11	0.19	0.29	0.28	0.73	5.61	0.24	3.13	0.47
100041504	'LOC100041504'	0	0	0	0	0.23	0.71	0	0	0	0
100041515	'Gm3383'	13.8	22.17	15.1	20.3	42.4	3.69	8.72	37.17	44.82	10.62
100041546	'Ly6c2'	0	0	0	0	0	0	0	0	5.51	0
100041574	'9030025P20Rik'	5.97	2.23	5.99	13	0	0.79	3.49	0.46	8.55	6.71
100041579	'Gm20878'	1.66	2.89	1.05	7.47	2.24	3.4	2.53	2.55	1.84	5.29
100041581	'Zkscan16'	4.4	2.57	9.02	2.87	8.33	11.13	10.5	1.88	5.72	2.59
100041585	'Amd2'	0.11	0.06	0.05	1.37	0.94	0	0.05	0.04	1.74	0.23
100041586	'Gm3417'	0	0	0	0	0	0	0	1.27	0	0
100041593	'LOC100041593'	0	0	0	0	0.28	3.16	0	0	0	0
100041599	'LOC100041599'	1.72	0.03	7.71	4.12	2.85	0	0.25	0.75	1.43	1.34
100041621	'Gm3435'	3.14	4.74	0.08	7.05	10.7	7.23	3.81	5.69	3.41	7.59
100041639	'Gm3448'	0	0	0	0.23	0	0	0	1.27	0	0
100041677	'Zfp984'	1.09	0.02	0.05	13.1	0.07	0.05	0	0	0	0.08
100041678	'Gm3500'	1.09	0.06	0.26	1.08	3.32	0.09	0	4.88	10.97	1.42
100041702	'Gm3476'	0	0	0.52	0	0	0	0.36	0	0.23	0
100041708	'LOC100041708'	0.13	0	0.05	0	0	0	0	0	0	0
100041712	'LOC100041712'	0	0	0.16	0	0	0	0	0	0	0
100041734	'4930522L14Rik'	2.18	0.06	0.1	0.55	5.04	1.14	1.1	0.24	10.6	2.63
100041735	'Gm3488'	7.13	13.54	20.2	2.19	10.4	2.17	7.89	21.24	19.63	8.9
100041774	'Gm10413'	0	0	0.06	0	0	0	0	0	0	0
100041840	'Gm10408'	0.04	0	0.11	0.15	0.22	0.19	0.04	0.13	0.31	0.61
100041874	'Gm3558'	13.35	9.44	10.3	5.01	12.4	9.65	5.73	11.63	10.23	16.21

100041953	'Sap18b'	272.9	292.4	254	294	291	358.4	195	280.5	198.4	233.7
100041958	'Gm3591'	1.04	0.14	0	0	0.12	0	0.34	0.11	0.15	0
100041964	'Gm3594'	0	0	0.14	0	0	0	0	0.06	0	0.06
100041979	'Gm3604'	0	1.3	0.48	0.02	0.16	0.1	0	1.24	0.52	0.14
100042054	'Gm3194'	1.15	0.37	1.17	5.34	0.7	0.03	7.35	1.13	1.09	1.44
100042074	'Gm3650'	0.98	0.5	0	0	0	0	2.17	2.68	1.4	0.16
100042100	'Gm3667'	2.11	2.78	2.87	0	2.2	0.9	1.16	2.85	3.2	3.07
100042149	'Gm3696'	3.71	5.56	4.6	0.17	6.17	7.46	6.69	11.39	6.84	4.98
100042150	'Nrg2'	2.27	0.12	2.74	0	0.17	1.76	2.05	0.63	0.79	0.82
100042165	'BC005561'	2.12	1.65	1.08	3.28	2.54	8.07	1.71	1.52	2.35	2.57
100042268	'Gm20907'	0	0	0.12	0	0	0	0	0	0	0
100042304	'Gm38418'	1.43	0.76	0.92	0	1.28	0.17	1.05	3.04	1.56	1.69
100042450	'Smim17'	3.11	3.15	2.46	0	2.04	8.61	5.94	2.98	0.23	2.41
100042480	'Nhsl2'	1.77	2.38	4.41	1.3	2.86	2.59	8.75	1.8	4.96	2.75
100042493	'Ccl21b'	0	0	0.79	0	7.13	4.14	6.01	0.1	1.97	2.71
100042499	'Vmn2r55'	0	0	0.02	0	0	0	0	0	0	0
100042503	'Ndufb4b'	15.07	9.34	12.1	7.05	29.9	18.33	16.1	16.1	32.54	19.31
100042514	'Sprr2a3'	0	0	0	0	0	0	0	0	6	0
100042555	'Gm13305'	0	0.04	0	0	0	0	0	0	0	0
100042591	'Vmn2r31'	0.02	0.04	0.02	0	0	0	0	0.02	0	0.03
100042636	'Vmn2r34'	0	0	0	0	0	0	0	0	0	0
100042653	'Vmn2r36'	0	0	0	0	0	0	0	0	0	0
100042698	'Gm3973'	1.89	0.06	0	0.07	0.9	0.86	0.3	0.56	0.06	0.71
100042781	'Vmn2r40'	0.02	0	0	0	0.01	0	0	0.01	0	0
100042784	'Prdm11'	0	0	1.94	0	0	0	0	0	0	0
100042786	'Gm16381'	0	0	0	0	0	0	0.11	0.27	0	0
100042807	'Eif3j2'	0	0	0.15	0.06	0	0.06	0	0	0.14	0
100042848	'Vmn2r41'	0	0	0	0	0.01	0	0	0	0	0
100042856	'Gm4070'	0	0	0	0	0.36	0	0	0	0	0
100042960	'Gm4131'	0	0	0	0	0	0	0.06	0	0	0
100043016	'Vmn1r131'	0	0	0.02	0	0	0	0	0	0	0
100043019	'Gm4175'	0	0	0	0.02	0	0	0	0	0	0
100043025	'Gm4177'	0	0	0	0.02	0	0	0	0	0	0
100043029	'Vmn1r138'	0	0	0.02	0	0	0	0	0	0	0
100043034	'Rex2'	0.34	0	0	0	0	0.05	0	0	0	0
100043037	'Gm10666'	0	0	0	0	0	2.64	0	0	0	0
100043079	'Gm4214'	0	0	0	0.02	0	0	0	0	0	0
100043083	'Gm4216'	0	0	0	0.02	0	0	0	0	0	0
100043088	'Vmn1r166'	0	0	0.02	0	0	0	0	0	0	0
100043133	'9130023H24Rik'	0	1.22	0	0	0.54	0.63	5.18	3.85	0	1.31
100043173	'BB114814'	0	0	0.3	0	0	0	0	0	0	0
100043272	'Inafm2'	14.9	17.84	6.69	22.2	17.1	4.67	8.82	6.36	6.01	5.69
100043314	'Tigit'	0	0.1	0	0.06	0.05	0	0	0	1.35	0.05
100043381	'Gm14308'	0.04	0.14	0.21	1.32	1.51	0	0	0.78	0	0
100043387	'Gm14305'	14.73	11.5	18.1	13.5	12.8	15.69	15.4	8.67	10.04	10.97
100043456	'Gm10681'	0	0	0	0	0	0.14	0	0	0	0
100043468	'Zfp955b'	8.88	6.93	1.94	4.62	14.8	4.63	13.1	3.72	7.43	5.86
100043474	'Olfr1564'	0	0	0.06	0	0	0	0	0	0	0
100043497	'Nat8f7'	0.04	0	0	0.05	0	0	0.04	0.03	0	0
100043523	'Gm4498'	0	0	0.02	0	0	0	0	0	0	0
100043597	'Srcap'	1.18	1.95	7.66	3.2	5.8	1.63	3.67	3.77	3.16	0.87
100043604	'Vmn1r132'	0	0	0	0	0	0.05	0	0	0	0
100043617	'Gm4553'	0	0	0	0.02	0	0	0	0	0	0
100043638	'Gm4565'	0	0	0.02	0	0	0	0	0	0.47	0
100043665	'Gm10662'	0.83	0	0	0	0	0.03	0.03	0	0.3	0.02
100043684	'Amy2a4'	0	0.06	0.06	0.07	0.02	0.05	0.01	0.01	0.01	0.04
100043686	'Amy2a3'	0	0.06	0.06	0.07	0.02	0.05	0.01	0.01	0.01	0.04
100043688	'Amy2a2'	0	0.06	0.06	0.07	0.02	0.05	0.01	0.01	0.01	0.04

100043757	'Zfp831'	0.38	0	2.37	0	0	0	0	0.01	0.01	0
100043772	'Zfp850'	0.01	1.37	2.56	0	1.12	0	2.95	1.52	4.67	1.89
100043861	'Klrb1'	0	2.95	0	0	0	0	0	0	0	0
100043914	'Zfp968'	0	0	0.12	0	0	1.14	0	0	0	0
100043915	'Gm4724'	0.58	0.73	1.22	0	1.02	0.13	0.59	0.79	0.47	0.52
100043920	'Fam205a4'	1.79	0	0.43	0.68	0.84	0.34	0.13	0	0	0.09
100044193	'Gm20939'	1.21	0.23	0.25	0	2.52	0.6	0.91	0.45	3.42	1.57
100044509	'Tgfbr3l'	0.04	1.34	4.57	0	2.12	2.9	2.74	4.39	5.41	2.87
100044627	'LOC100044627'	139.1	133.1	149	158	138	185.6	141	102.8	176.7	134.73
100045778	'Rnf223'	0	0	1.14	0	0	0	0	0	0	0
100045792	'Ect2l'	0.73	0.12	0.09	0.41	0.68	0.16	0.03	0.18	0.47	0.8
100048534	'Cfap43'	0	0.1	0.44	0.01	0	0.14	0.6	0	0	0.18
100048644	'Pet117'	0.2	0	0	0	0.29	0	0	0.17	0	0
100072	'Camta1'	17.9	18.84	13.6	29.1	18.5	13.78	16.7	16.78	28.77	14.48
100073351	'Yy2'	1.8	2.29	1.91	4.55	0.6	3.14	0.29	0.98	0	0.63
100087	'Kti12'	16.14	15.11	20.8	0	19.7	17.92	8.85	20.2	1.42	20.1
100088	'Rcc1'	5.66	11.1	3.69	4.62	3.86	0.04	2.88	10.28	4.8	7.65
100090	'Zbtb48'	2.7	3.07	8.39	1.7	8.34	16.16	11.3	8.49	6.08	9.55
100101806	'Srp54c'	58.71	58.21	63.4	61.8	40.1	82.89	53.8	76.81	77.86	77.15
100101807	'1700047I17Rik2'	37.38	39.97	30.8	25.4	27	17.47	25.2	18.62	27.95	16.73
100101919	'Dnah7c'	0.01	0.06	0.3	0	0.01	0.12	0	0	0	0.02
100102	'Pcsk9'	0	0	3.12	0.42	0	0	0	1.07	0	0
100113398	'Adat3'	2.1	0.86	6.09	14.3	1.45	0	3.71	0	0.62	0
100121	'Tdrd7'	1.8	3.46	0.63	6.94	3.14	4	10.5	0.24	4.65	4.56
100125586	'Vmn2r2'	0	0.07	0.35	1.96	0	0.12	0	0	0	0
100126824	'Sco2'	37.96	34.14	26.6	11.8	30.7	38.98	23.5	8.92	63.24	12.89
100129	'Gpr153'	4.76	3.44	4.88	0.52	0.24	2.16	8.22	0.02	0.39	0.78
100141474	'4933428G20Rik'	0	0	0	0	0	0.1	0	0	0	0
100151772	'Srsx'	0	0	0	0	0	0.03	0	0	0	0
100155	'AI481877'	0.58	0	0	0	0.35	0	0	0	0	0
100163	'Pafah2'	0.25	5.2	2.75	0	5.9	0	3.42	3.37	1.19	2.9
100169	'Phactr4'	0.62	0.38	2.05	14.9	1.48	2.54	0.55	0.52	0.68	0.33
100169864	'Gm44504'	0	0.26	0.09	0.07	0	0	0.04	0	1.84	0.11
100169868	'Gm3173'	1.6	1.41	1.28	1.96	3.91	2.59	2.26	2.46	2.76	1.14
100177	'Zmym6'	8.42	5.66	9.47	6.98	8.7	4.65	5.86	4.28	7.96	10.99
100182	'Akna'	0.6	0	0.01	0	1.43	0.18	0.17	0	0.52	0
100188919	'Gm45915'	0.02	0	0.18	0.62	0	0	0.35	0	0.1	0
100198	'H6pd'	1.32	0	0.01	0	2.35	0.17	1.42	0.01	0.02	2.11
100201	'Tmem64'	2.95	2.21	2.92	6.31	0.54	2.07	4.2	3.01	0.61	0.01
100206	'Adprhl2'	12.57	21.57	39.3	3.99	29.2	36.58	26.7	29.96	8.18	23.58
100210	'Gpn2'	4.94	6.96	16.9	0	13.6	20.51	5.7	8.81	5.32	20.68
100213	'Rusc2'	6.85	11.13	8.39	1.66	8.69	4.55	3.87	6.55	3.61	7.97
100216455	'Gm14124'	0.01	0	0	0	0	0	0	0	0	0.01
100216474	'Ttll2'	0	0	1.03	0	0	0	0	0	0	0.02
100226	'Stx12'	71.62	82.32	96.2	67.8	77.9	53.32	84.9	78.29	85.99	64.04
100233175	'Gon7'	21.07	62.22	23	76.4	16.9	39.63	41	15	60.4	51.37
100233207	'Gm17359'	0	2.32	0.03	0	0	0	0	0.55	0	0
100233208	'Gm10778'	0.05	0.11	1.05	0.08	0.09	1.2	0	1.21	0.01	0.76
100270744	'Btbd18'	0.03	0	0.11	0	1.53	0	0.01	0	0	0
100271704	'Gm17660'	9.3	5.16	3.35	9.59	16.2	0	19.8	6.72	0	7.81
100271928	'Gm15881'	7.28	3.09	1.76	0.29	3.57	8.55	0	4.52	4.17	2.5
100273	'Osbpl9'	27.86	19.54	20	26.6	20	18.9	20.9	24.45	22.06	28.39
100294583	'Alkal2'	0.12	10.6	0	0	0	0	0	0	3.96	0
100294660	'Defa2'	0	0	0	0	0	0	0.14	0	0	0
100302688	'Gm17455'	0	2.76	0	0	0	0	4.59	0	5.08	0
100303732	'Zfp967'	2.12	0.31	1.97	1.81	0.95	0.47	0.51	1.04	0.48	1.1
100303744	'Sprr2a2'	0	0.01	0.01	0	0	0	0	0	0	0
100310872	'Dynlt1a'	12.37	8.28	14.9	0	6.1	5.55	4.91	7.68	13.28	6.43

100317	'AU040320'	12.53	22.27	19.8	9.63	8.39	6.02	11.9	11.11	14.27	39.11
100336	'Ppp1r8'	35.8	46.71	36.3	48.2	40.1	15.93	30.3	54.53	56.87	46.14
100340	'Smpdl3b'	11.89	4.66	7.64	0	15.5	11.23	0.03	2.94	0	9.44
100342	'Tent5b'	0	0.77	0	0	0	0	0	0	0	0
100383	'Bsdc1'	36.21	19.93	28.5	38.5	14	13.87	20.8	11.36	14.27	12.7
100384868	'Gm37013'	3.64	0.44	0.8	0.88	0.11	0.06	0.47	5.61	0.96	0.82
100416706	'Zfp729b'	1.95	1.16	1.19	0	5.07	0.01	2.08	0.55	0.76	0
100417514	'Adh6b'	0.79	0	0	0	0	0	0	0	0	0.02
100434	'Slc44a1'	0.02	2.21	4.1	4.1	4.43	1.67	5.08	1.58	0.66	1.27
100463512	'Gm20594'	524.4	378.1	487	376	679	822	507	195	300.8	447.85
100465	'Mob3c'	4.5	5.83	7.1	0.14	0.75	6.42	0	6.7	3.14	6.38
100494	'Zfand2a'	20.93	23.97	28.7	19.4	19.4	12.41	21.4	25.28	10.16	23.27
100502590	'Tex50'	0.96	0.89	1.94	0.25	1.55	1.1	0.44	1.18	0.23	0.42
100502698	'Rubcn'	7.02	6.7	12.9	10.6	7.82	3.26	16.9	11.95	17.2	11.4
100502736	'Gm19345'	13.49	13.46	2.8	0	0	8.67	0	11.38	8.5	19.98
100502766	'Kifc1'	1.73	2.49	0.38	0	0	0.41	2.36	0.03	0.26	0
100502777	'Gm38422'	0	1.41	1.52	0	0.18	0.31	1.15	2.5	5.27	0
100502825	'Rpl37rt'	0.36	0.6	0.61	2.23	3.29	1.54	0.61	0.51	0.38	0.39
100502841	'Epg5'	1.29	1.46	2.96	8.12	0.5	0.29	3.61	1.41	2.8	1.54
100502846	'Gm19410'	0	0	0	0	0	0.02	0	0.03	0.01	0
100502861	'Ccdc13'	1.79	3.35	0.02	0	3.84	3.47	8.03	0	0	1.66
100502876	'Kcnmb3'	0	0.31	0.34	0.76	0.19	0.03	2.3	0	0.98	0.25
100502921	'Gm10142'	0	2.79	0	0	0	0	0	0	0	0
100502940	'Colca2'	0	2.52	0	0	0	0	0	0	0	2.69
100502941	'Gm17353'	0	0.05	0.13	0.03	0	0	0	0.04	0	0
100502950	'Gm3336'	0	0	0	0	0	0	0	0.11	0	0.38
100502955	'Gm19470'	0	0.25	0	0	0	0	0	0	0	0
100502967	'Speer4c'	0.05	0.04	0	0	0.13	0.44	0.09	0.14	0	0
100503041	'Pdzd7'	0.01	0.91	0.04	0	2.89	10.62	2.04	0	0.07	1.04
100503043	'Armcx4'	1.19	2.89	2.5	4.53	2.45	1.72	1.87	3.13	0.91	2.03
100503085	'Klhl3'	1.36	3.92	0.03	1.78	0.19	6.72	2.1	2.05	4.3	2.41
100503185	'Btbd8'	0.72	0	3.36	0.83	0.18	0	0	0	2.42	0
100503240	'Trpc5os'	0	0	0	0	0	0	0	0.45	0	3.15
100503280	'Gm38425'	0.74	0	0	0	0	0	0	0	0	0
100503311	'Pifo'	3.1	1.38	4.7	12.6	50.8	0	0	0	0	25.13
100503353	'Gm14440'	12.53	18.02	16.4	11.3	18.2	20.1	13.5	19.78	10.46	15.31
100503355	'Zfp648'	1.23	1.4	0	0.03	1.37	0	5.72	0.77	0	0
100503361	'Tmem95'	0	1.64	0	0	0	1.32	0	0	0	1.1
100503386	'Tpbgl'	1.65	0.17	0.42	0.54	2.22	0	0	0.2	0	0
100503545	'Nuggc'	0	0	0	0	0	0	4.8	0	0	0
100503549	'Gm10354'	0	0	0.06	0.08	0	0	0.91	0.12	0.14	0
100503572	'Bbip1'	13.89	32.29	17.3	21	28.1	17.15	25.2	24.61	15.15	18.66
100503583	'Fsbp'	0	0	0	0	0.33	0	0	0	0	0.68
100503584	'Zfp534'	0	0	0	0.03	0	0.36	0	0	0	0.03
100503605	'Hbb-bs'	2.02	0	6.17	81.7	0.08	0	7.42	13.75	29.53	6.43
100503659	'Cbarp'	113.4	90.31	69.6	69.8	117	39.51	67.2	140.4	181.1	107.85
100503670	'Rpl5'	149.1	142.2	144	165	176	98.31	172	139.8	135.7	151.59
100503710	'Gm5741'	0.33	0.18	0	1.07	0.18	0	0	0	0.48	0
100503884	'Ccdc149'	1.82	8.46	1.82	8.15	3.08	2.72	6.22	7.37	2.47	6.23
100503890	'Pet100'	16.43	16.05	35.2	33.5	28.4	58.99	24.6	21.65	18.07	21.5
100503915	'Smpd5'	1.07	0	1.59	0	0	0	0	0	0	0
100503949	'Zfp965'	0	0	0	0	0	0.32	0	0	0	0
100504112	'Ccer2'	16.28	4.74	54.7	0	15.4	12.88	0	9.45	32.51	5.59
100504156	'Fam181a'	12.91	17.6	3.37	12.3	5.65	3.64	0.04	5.45	0	0
100504180	'LOC100504180'	0	0	0	0	0	0.78	0	0	0	0
100504183	'Krtap16-1'	0	0.13	0	0	0.18	0	4.86	0	0.02	2.53
100504195	'Micalcl'	0	1.8	0.72	0.31	0	0	0	0	0	0
100504200	'Gm8050'	0	0	0	0	0	0	0	0.21	0	0

100504234	'Ccdc170'	0	0	0	0	1.68	0	0	0	0	0
100504263	'2210418O10Rik'	8.31	4.12	4.68	1.38	1.76	1.13	0.62	2.82	0.43	3.06
100504323	'Gm20172'	1.21	0	0.03	0	2.41	0	1.99	0.31	3.14	0
100504429	'Spin2d'	0.05	0	0	0	0	0	0	0	0	0
100504446	'Prr22'	0	0	2.1	0	5.08	2.07	0	0	1.98	3.15
100504491	'Dcdc2b'	0	1.63	0.2	0.91	3.14	3.07	0	3.17	1.7	0.83
100504518	'3425401B19Rik'	0	0	0	0	0	0	0	0	0.01	0.02
100504608	'Eef1akmt3'	0	0.14	0	0	0	0.07	0	0	0.07	0.52
100504663	'Atg14'	8.17	4.8	8.08	7.02	3.2	2.36	11.6	7.99	2.03	5.49
100504715	'Tmppe'	2.28	2.81	3.4	1.6	1.26	1.74	1.92	0.73	3.61	4.97
100505386	'Iqschfp'	0	2.64	0	0	9.62	9.72	3.52	2.75	0	0
100505397	'Gm20385'	1.94	0	2.28	9.44	1.64	0	0.29	0.75	0.03	1.36
100515	'Zfp518b'	0.61	1.7	3.66	0.98	0.07	0	1.35	1.24	1.22	1.08
100529082	'Gm11127'	3.55	2.44	2.43	1.73	7.18	3.76	2.25	3.24	6.41	3.75
100532	'Rell1'	0.01	0	1.6	0	0.71	0.1	0	0.22	2.65	0
100534273	'Fer1l5'	0	0	0.23	0	0	0	0.25	0.02	0.01	0.32
100561	'Slc15a4'	13.7	9.2	10.1	27.3	10.9	0.37	13.6	3.94	27.27	4.4
100604	'Lrrc8c'	7.42	4.47	4.23	12.3	3.17	3.81	16.6	5.41	0.6	0.18
100608	'Noc4l'	23.35	25.87	29.3	29.2	27.5	18.15	24.7	18.24	21.01	15.79
100609	'Nsun5'	5.8	15.29	9.69	21.1	6.97	5.01	4.14	17.06	11.57	10.24
100637	'N4bp2l1'	46.41	29.26	26.6	37.9	40.6	40.93	24.2	31.21	75.79	72.01
100678	'Psph'	12.35	0.66	13.1	7.4	11.7	13.69	13.3	12.38	16.36	6.2
100683	'Trrap'	3.8	5.19	6.81	3.49	4.83	6.4	3.84	3.25	3.82	6.21
100702	'Gbp6'	0	0.42	2.11	6.23	2.94	0	0	0	1.54	0.09
100705	'Acacb'	0.58	0.48	0.33	0.36	0.9	3.36	2.75	1.78	0.87	1.06
100710	'Pds5b'	9.72	5.35	8.12	11.4	7.8	4.31	8.29	3.98	8.24	7.58
100715	'Tent2'	18.16	10.73	8.26	18	6.74	13.98	5.66	18.44	10.57	2.43
100732	'Mapre3'	258.5	249.7	232	154	266	278.5	272	268.4	219.9	268.69
100737	'Dcun1d4'	49.44	47.41	46.2	28.2	38.8	13.97	51.6	65.87	36.02	31.2
100756	'Usp30'	9.77	6.67	6.24	0.95	3	3.84	0	3.01	8.31	0.75
100763	'Ube3c'	5.99	6.08	9.91	0.73	8.6	3.69	7.27	6.56	1.09	4.25
100764	'Rita1'	6.01	9.97	20.1	0.04	16.8	12.28	9.26	10.57	14.23	5.02
100855	'Tbc1d14'	19.73	15.36	11.8	17.2	18.7	10.86	6.16	6.45	7.97	19.91
100859931	'Gm20604'	23.04	22.65	17.8	23.6	5.84	13.21	13.9	24.58	21.04	21
100861615	'LOC100861615'	4.85	2.86	5.41	4.32	3.19	3.71	3.31	6.49	6.8	3.44
100861668	'Gm21119'	0.05	0	0.23	1.21	0.03	0.03	0.18	0	0	0.01
100861702	'Gm21149'	0.05	0.03	0.08	0	0.04	0.91	4.53	0.05	0	0.03
100861708	'Gm21154'	0	0	0.03	0	0.43	0	0	0	0	0
100861719	'Gm21162'	0	0	0.5	0	0.45	0	0	0	0	0.01
100861753	'Gm21188'	0.08	0	0.03	0.2	0.06	0.08	0.06	0	0	0
100861755	'Gm21190'	0	0	0	0	0	1.39	0.39	0	0	0
100861879	'Gm21292'	0	0	0	0	0	0	0	0	0.38	0
100861899	'Gm21310'	0	0	0	0	0	0.01	0	0	0	0
100861908	'Gm21319'	0.02	0	0	0.1	0	0	0	0	0	0.05
100861969	'LOC100861969'	0	0.04	0	0	0	0	0	0	0	0
100861978	'LOC100861978'	1.13	1.73	4.81	1.95	0	2.98	0	5.11	8.35	4.25
100862043	'LOC100862043'	0	0.06	0	0	0	0	1.3	0	0	0
100862066	'Cfap99'	0.59	0	0.68	3.07	0.03	0.29	0.76	0	0.6	0.18
100862072	'Gm21451'	0	0.07	0	0	0	0	0	0	0	0
100862085	'Gm16867'	2.16	0	0.99	2.52	3.97	2.64	3.02	0.61	0.03	0.24
100862192	'LOC100862192'	0	0	0	0	0	0	0	0	0.04	0
100862203	'Gm6712'	5.45	0.54	0.6	0	7.12	8.5	9.23	1.24	4.34	3.55
100862247	'Gm21586'	1.66	2.89	1.05	7.47	2.24	3.4	2.53	2.55	1.84	5.29
100862261	'Fam205a3'	0.73	0.21	0.05	0.3	0.31	0.36	0.07	0.14	1.72	0.07
100862314	'Gm21637'	0	0	0	0	0	0	0	0.03	0	0
100862324	'LOC100862324'	0.44	0.12	0.25	0	0.29	0.02	0.25	0	1.04	0.23
100862345	'Gm21660'	0	0	0	0	0	0.01	0	0	0	0
100862348	'Gm9295'	1.5	0.98	1.1	1.78	0.87	0.25	2.42	0.72	2.28	0.36

100862349	'Gm21663'	0.17	0.33	0.31	0	0.26	0	0	0.04	0	0
100862359	'Gm21671'	0.03	0.06	0	0	0	0	0	0	0	0.28
100862368	'Gm21680'	0.06	1.6	1.71	0	4.5	1.09	1.11	0.96	1.64	2.12
100862369	'Btbd35f9'	0	0	0.37	0	0	0	0	0	0	0
100862375	'Entpd4b'	24.06	25.76	50.2	10	20.4	40.65	61.3	21.82	38.57	9.79
100862388	'Gm21698'	0.02	0	0.04	0	0	1.48	0	0.19	0	0.13
100862433	'LOC100862433'	0.64	0.31	0.53	0.48	0.24	0.57	0.17	0.14	0.62	0.7
100862446	'LOC100862446'	3.32	5.97	3.97	9.91	3.6	4.79	5.61	4.73	5.57	5.58
100862455	'LOC100862455'	139.1	133.1	149	158	138	185.6	141	102.8	176.7	134.73
100900	'Hscb'	19.03	14.69	7.68	20.8	2.65	21.22	22.7	8.72	11.15	18.16
100910	'Chpf2'	18.79	14.79	22.3	3.99	28.7	5.18	19.5	27.79	6.23	26.1
100929	'Tyw1'	6.73	13.43	22.4	7.51	7.86	1.55	9.09	18.69	10.09	10.19
100952	'Emilin1'	0	0	0	0	4.96	0	0	0	1.88	0
100972	'Rab28'	61.19	64.4	47	51.3	69.4	43.94	59.8	33.61	71.52	38.86
100978	'Nfxl1'	0.39	0	0.85	0.28	0.03	0.01	0	0.06	1.75	0.41
100986	'Akap9'	11.6	12.18	8.79	15.4	12	12.74	14.1	11.98	18.34	15.94
101023	'Zfp513'	2.24	3.54	0.41	0	0.72	1.71	0	0.16	0	1.62
101055630	'101055630'	2.88	29.63	22.3	22.3	35.1	2.85	9.06	7.23	19.1	16.37
101055663	'LOC101055663'	0	1.57	1.4	0	0	0	0	0	0	0
101055671	'Lipo2'	0.18	0.13	0	0	0.09	0	0.03	0.29	0.67	0.09
101055672	'LOC101055672'	0	0	0.04	0.05	0	0	0	0	0	0
101055676	'LOC101055676'	0	0	0.4	0	0	0.47	4.46	1.17	3.47	0.11
101055754	'Gm2974'	1.61	0.12	0.56	0.65	1.38	0.85	0.04	2.16	0.32	1.43
101055758	'LOC101055758'	0	0.9	0.09	0	0	0	0	0	0	0
101055806	'Gm10378'	0	0	0.59	0	0.36	0.66	0	0	0	0.12
101055837	'Gm38434'	0	0.12	0	0	0	0	0	0	0	0
101055854	'Gm29733'	0.59	2.26	3.64	27.1	0	0.05	6.66	1.69	2.47	0
101055864	'LOC101055864'	0	0	0.02	0	0	0	0	0	0.47	0
101055907	'Gm15246'	18.13	5.68	2.58	0	11.4	1.93	5.72	5.9	2.65	7.94
101055987	'Gm29758'	0.37	0.45	2.38	4.27	0.31	0	0.12	0.96	0.57	0.08
101056073	'Zfp990'	0	0	0	0	0	0.14	0	0	0	0
101056102	'Gm29779'	1.47	0.24	2.11	0	0	0	18.3	4.31	0	6.32
101056115	'LOC101056115'	0	0	0	0.01	0	0	0	0	0.01	0
101056159	'LOC101056159'	0	0	0.49	0	1.87	0	0	0.41	0	0
101056205	'Gm29797'	0	0	0	0	0	2.26	0	0	0	0
101056210	'Gm28576'	0	0	0	0	0	0	0	0.23	0	0
101056241	'Gm29808'	0	0.03	0	0	0	0	0	0	0	0
101056296	'LOC101056296'	0	0.04	0	0	0.08	0.03	0.06	0	0	0
101056300	'LOC101056300'	0	0.04	0	0	0.08	0.03	0.06	0	0	0
101056408	'Gm15816'	0	0.16	1.11	0	0	0	0	0.75	0	0
101095	'Zfp282'	4.89	1.16	2.67	3.74	4.05	1.5	3.15	0.99	0	0.73
101100	'Ttll3'	5.88	7.71	9.8	4.02	13.7	8.41	1.35	0	5.53	6.25
101113	'Snx21'	13.5	17.56	11.6	15.4	12.1	5.67	6	16.11	28.2	12.81
101118	'Tmem168'	3.09	2.32	0.85	1.56	5.45	0.23	0	2.77	1.82	7.43
101122	'Rpusd3'	11.21	12.22	10.2	7.25	5.95	17.7	15.8	3.37	21	15.78
101142	'Itfg2'	45.61	22.18	29.7	36.8	25.7	39.82	33	30.67	44.8	35.49
101148	'Bmt2'	4.04	0.98	0.08	1.43	1.91	0.22	0.41	0.13	1.4	2.53
101185	'Pot1a'	3.53	21.54	6.26	2.41	0.96	9.41	4.48	10.82	7.36	11.75
101187	'Parp11'	12.75	8.97	5.25	0.01	8.97	7.47	19.9	9.29	19.9	15.74
101197	'Zfp956'	14.14	5.6	6.4	12.3	2.51	1.59	7.23	27	15.49	10.16
101202	'Hepacam2'	0	0	0	0	0	0.77	0	0.13	0	0.32
101206	'Tada3'	13.24	35.94	15.5	31.8	37.1	20.58	20.7	29.78	20.89	24.11
101214	'Tra2a'	47.29	52.49	45.5	23.8	28.9	50.22	30.7	35.3	45.01	42.99
101240	'Wdr91'	8.62	4.37	3.79	7.16	6.33	7.29	0.09	4.53	6.42	2.97
101314	'Brk1'	263.2	256.1	212	200	231	337.5	223	188.4	160.1	242.67
101351	'Eogt'	7.39	0.4	7.93	6.51	4.53	0.06	0	1.33	3.22	3.15
101358	'Fbxl14'	1.7	0.64	0.62	0	1.34	0.8	1.61	0.6	0.75	0.03
101359	'Prrt4'	0	0.75	2.8	1.24	1.17	0.01	2.91	0.13	0.38	0.49

101401	'Adamts9'	0	0.41	0.02	0.03	0	0.01	0	0.12	0	0
101434	'Ceacam15'	0	0	0.11	0	0	0	0	0	0	0
101437	'Dhx32'	15.34	21.5	8	4.87	11.4	6.95	6.74	13.65	11.54	7.37
101471	'Phrf1'	2.01	4.3	3.71	4.62	8.46	4.66	5.58	2.24	2.53	5.4
101476	'Plekha1'	16.73	9.49	9.13	12.5	4.79	6.61	11.6	10.73	13.25	8.86
101488	'Slco2b1'	0	0	0	0	0	0.8	0.36	0	0.72	0
101488143	'Hbb-bt'	0.16	3.37	0	11.6	0	0.95	0	6.67	0	0
101489	'Ric8a'	33.55	26.76	52.2	18.9	24.5	25.11	25.9	30.53	53.87	37.61
101490	'Inpp5f'	65.72	66.58	111	83.1	60	61.96	69.9	99.72	75.92	59.77
101497	'Plekhg2'	0	0	0	0	0	0	0	0	0	0.01
101502	'Hsd3b7'	6.46	10.78	0	0.03	11.3	12.32	21.2	7.11	15.82	14.71
101513	'Mob2'	57.51	44.48	36	9.74	65	37.94	34.4	33.43	50.42	27.33
101540	'Prkd2'	1.62	0.48	0.05	1.78	0.05	0	7.4	0	4.31	2.43
101543	'Wtip'	0	1.28	0.02	0	0	0	3.02	3.15	1.22	0
101544	'Zfp575'	14.44	15.32	12.4	16.8	8.52	22.63	16.6	17.64	11.09	5.24
101565	'Ccp110'	7.12	5.33	6.08	6.76	3.77	3.73	4.59	6.41	3.18	6.29
101568	'Vrk3'	31.5	21.34	33.6	43.7	28.3	35.77	22.4	22	26.79	12.27
101592	'Efl1'	0.78	7.45	2.02	10.5	6.74	3.32	1.89	4.68	9.44	10.24
101602	'AI467606'	15.69	21.1	3.82	18.2	3.97	11.35	0.05	18.8	0.07	31.64
101604	'E430018J23Rik'	3.17	2.69	0	5.19	1.74	7.42	6.54	5.58	9.83	7.13
101612	'Grwd1'	25.33	29.4	29.9	28.8	32.5	51.24	68	47.38	52.68	21.37
101613	'Nlrp6'	0	0	0.01	0	0	0.49	0	0	0	0
101631	'Pwwp2b'	0.53	0	0	0	8.77	7.65	0	0	4.17	0
101685	'Spty2d1'	3.5	2.69	3.9	7.12	1.39	3.26	3.89	3.08	1.94	2.27
101700	'Trim68'	5.13	0	0	0	2.93	3.49	0	2.84	0	0
101706	'Numa1'	5.38	4.51	6.07	8.62	7.24	7.05	5.52	4.06	4.45	7.43
101739	'Psip1'	19.96	15.43	18.9	30.5	13.5	19.21	29	18.66	31.14	11.31
101744	'Acp7'	0	0	0	0	1.22	0	0	0	0	0
101772	'Ano1'	0.01	0	4.07	0.1	5.52	0.09	0	1.42	2.49	1.92
101809	'Spred3'	5.42	3.12	2.26	10	3.75	4.45	5.38	3.68	5.45	2.22
101831	'Faap24'	2.35	1.43	3	13.6	13.8	4.5	4.28	5.43	17.93	8.58
101835	'AW146154'	5.5	1.92	0.88	0	5.59	0	0	1.14	0.4	2.32
101861	'Ints4'	13.61	17.42	13.7	22.4	11.1	7.19	9.68	12.17	10.21	16.35
101867	'Rrp8'	0	8.06	8.77	10.2	9.36	5.51	3.37	12.42	7.49	2.74
101869	'Unc45a'	17.52	13.72	9.56	28.3	19.9	4.53	13.6	15.23	3.61	15.18
101883	'Igflr1'	4.28	6.72	5.65	6.57	14.1	4.71	4.76	3.33	4.54	5.65
101943	'Sf3b3'	26.33	22.9	26.7	11.6	12.7	9.44	39	23.83	19.38	29.17
101966	'D8Ertd738e'	259.7	185.4	229	362	293	252.4	158	185.2	320.1	220.38
101985	'Usb1'	13.57	8.16	17.2	13.2	14.5	15.09	13.5	8.51	26.43	5.5
101994	'Champ1'	11.01	8.13	10.7	10.4	4.78	2	11.4	5.82	8.52	17.81
102032	'Smim19'	47.17	31.55	27.5	38.6	23.9	74.89	98.9	18.84	31.22	29.83
102058	'Exoc8'	2.64	4.33	1.6	1.29	3.81	1.33	2.97	2.88	2.91	6.6
102060	'Gadd45gip1'	77.07	61.13	79.7	63.4	111	90.18	80.3	83.81	98.42	74.91
102075	'Plekhg4'	3.48	0.48	0.6	0	0	1.66	0	0.11	0	2.54
102093	'Phkb'	7.88	4.98	1.89	5.04	7.05	2.46	5.73	4.55	9.55	2.59
102098	'Arhgef18'	1.61	3.11	9.69	7.4	8.35	3.27	7.16	3.82	6.94	8.41
102103	'Mtus1'	18.3	10.9	3.61	2.93	10.2	1.08	6.31	17.54	6.15	6.33
102115	'Dohh'	82.58	117.2	109	112	146	169.4	150	132.5	122.3	131.81
102122	'Fam192a'	28.55	30.36	32.7	28.7	35.1	26.98	41.6	41.41	55.33	62.44
102124	'Enkd1'	12.36	18.81	13.7	2.67	19.4	4.68	28.1	10.03	7.57	3.79
102141	'Snx25'	2.42	4.07	4.61	1.23	0.62	0	0	1.73	0.41	3.54
102162	'Taf5l'	1.96	3.19	5.75	4.25	3.8	0	4	8.69	5.56	0
102182	'Prmt9'	3.61	1.87	5.83	14.1	7.7	9.17	9.44	9.54	0.37	2.98
102193	'Zdhhc7'	15.83	4.93	22.4	17.1	6.26	3.05	6.37	7.92	7.5	3.89
102209	'Snapc2'	79.96	75.27	67.7	43.4	27.4	26.08	66.8	22.45	40.35	26.14
102216272	'Ak6'	37.11	18.67	29.4	25.8	40.7	37.98	30.7	38.01	15.99	31.18
102247	'Gpat4'	34.93	35.56	34.9	40.4	28.5	26.85	18.8	28.11	30.57	29.01
102278	'Cpne7'	34.43	22.69	27.9	8.57	12.7	44.12	25.8	19.7	45.62	19.61

102294	'Cyp4v3'	3.93	2.24	2.2	0	0	0	3	1.3	0	0
102323	'Dcun1d2'	8.79	18.15	19.3	14.1	9.1	7.31	19	13.47	1.36	1.83
102334	'Ankrd10'	52.39	87.11	42.9	64.6	43.9	65.46	60.2	47.88	40.73	63.75
102339	'Cog4'	21.31	34.77	15.5	45.6	31.4	30.99	29.8	23.5	13.95	30.08
102371	'Myzap'	0.08	0	0.11	0.05	0.04	0.08	0.02	0.02	0.06	0.06
102414	'Clk3'	43.87	32.72	28.5	71	32.5	18.65	10.4	50.21	31.93	30.25
102423	'Hinfp'	0.58	6.58	0.62	0.01	1	5.39	9.3	4.75	0.56	0.28
102436	'Lars2'	8.61	12.73	11.6	4.62	1.45	2.04	0.05	7.21	2.77	8.02
102442	'Dennd4a'	1.12	1.5	1.96	1.23	1.79	2.05	0.23	0.89	1.79	1.26
102443350	'Xndc1'	8.1	3.2	5.85	5.05	5.46	6.12	6.97	2.93	0.22	4.34
102443351	'Xntrpc'	0	0	1.03	0	0	0	0	0.32	0	0
102448	'Xylb'	4.01	1.78	0.09	0.12	0.34	0.12	3.92	4.13	0.02	3.08
102462	'Imp3'	68.01	57.49	49.4	40.3	45.4	82.36	57.7	47.85	74.9	53.15
102502	'Pls1'	4.4	6.77	0.17	0.02	6.45	3.21	0	0.56	1.88	1.48
102545	'Cmtm7'	7.55	0	0	0	0	13.54	0	0.4	0	0
102566	'Ano10'	13.93	11.78	4.97	3.38	18.4	17.81	19.7	16.03	18.8	18.63
102570	'Slc22a13'	0	1.17	0	0	0	0	0	0	0.32	0.53
102580	'Alg9'	11.48	3.85	13.7	2.14	1.59	0.45	7.68	3.63	8.77	2.96
102595	'Plekho2'	3.67	0	2.37	0	1.94	0	0.14	0	0	0
102607	'Snx19'	13.42	10.1	23.5	12.5	3.91	5.56	16.6	9.06	9.64	10.2
102614	'Rpp25'	17.63	8.46	12.6	9.46	17.6	29.39	12.2	12.26	4.81	0
102626	'Mapkapk3'	2.26	2.47	0	0	5.93	6.38	0	0	4.1	3.48
102631585	'LOC102631585'	0.01	0	0	0	0	0	0	0	0.01	0
102631705	'Gm29975'	0.52	1.92	0.27	7.48	1.67	1.24	1.24	0.07	0.72	0.69
102631717	'Gm29986'	0	0	0	0	0.15	0	0	0	0	0
102631730	'4930512M02Rik'	15.33	13.17	19.3	34.6	23.4	19.79	20.8	11.33	26.32	11.93
102631805	'LOC102631805'	0	0	0	0.01	0	0	0	0	0.01	0
102631909	'Gm30124'	0	0.53	0	0.04	0.59	0	0.79	0.16	1	0
102632	'Acad11'	3.03	7.69	0	7.38	5.63	0.82	4.7	11.56	5.13	4.46
102632383	'LOC102632383'	1.5	0.45	0.05	1.88	1.39	3.2	0.24	1.22	4.45	1.24
102632483	'Gm17330'	0.2	0.12	0.68	0.28	0.21	0.16	0.75	0.15	2.43	0.19
102632554	'Gm30599'	0	0	0	0	0.03	0	0.53	0	0	0
102632693	'Gm30698'	0.04	0	0	0	0.26	0.29	0	0	0.19	0.38
102632737	'Gm30732'	0	0	0.02	3.02	0	0	0	0	0.02	0
102632738	'Gm30733'	0	0	0.23	0	0	0	0	0	0	0
102632739	'Gm19774'	0	0	0.34	0.49	0	0	0	0	0	0
102633032	'1700064H15Rik'	0	0.02	0.06	0	0.39	0	0	0	0.29	0
102633100	'Gm7682'	0	0	0	0	0	0	0	0	0.01	0
102633131	'Gm31035'	0	0	0	0	0.22	0	0	0	0	0
102633156	'LOC102633156'	4.74	4.27	1.04	2.36	3.27	2.05	4.03	2.42	2.29	2.2
102633301	'Gm31160'	0.03	0	0	0	0	0	0	0	0	0
102633345	'Gm9922'	0	0	0	0	0	0	0	0	0	0.02
102633458	'Gm11100'	0	0	0	0	0.09	0	0	0.3	0.08	0
102633498	'Gm31309'	0.63	0.03	0	0	0	0	0	0	0	0
102633740	'Gm31493'	0	2.08	0	0	0	0	0	0	0	0
102633750	'Gm10130'	3.81	0.17	1.54	0.11	0.16	0.04	0.11	1.78	0.19	0.09
102633888	'Gm31606'	0.45	0.42	1.47	1.62	2.28	1.07	1.81	1.35	1.32	0.57
102633951	'Gm31649'	0	0	1.03	0	0	0	0	0.26	0	0
102634072	'Gm38457'	0	0	0	0	0	0.11	0	0	0	0
102634078	'LOC102634078'	5.97	2	5.27	6.46	6.13	3.17	4.25	4.63	8.06	6.85
102634296	'Gm4779'	0	0.99	0	2.9	3.35	1.57	0	1.63	0	0
102634333	'1110002E22Rik'	0	0	0	0	0	0	0	0	0	0
102634429	'Gm6569'	0.11	0	0	0	0	0	0	0	0	0.05
102634529	'LOC102634529'	0	0	0	0	0	0	0	0	0.01	0
102634532	'Gm3642'	0	0	0	0.31	0	0	0	0.01	0	0
102634581	'LOC102634581'	0	0.75	0	0	0	2.01	0	0	2.49	0
102634611	'Gm32151'	0.53	0	0	0.13	2.83	0	5.01	1.59	1.08	4.46
102634746	'Gm13201'	0	0	0	0	0.36	0	0	0.03	0	0.02

102634788	'Gm32289'	0	0	1.41	0	0.11	0.85	0	0	1.18	0
102634841	'Gm38469'	0	0	0	3.89	0	0	0	0	0.41	0
102634941	'Gm29376'	0.04	0	0.01	0.02	0.07	0.22	0	0.46	0	0
102635315	'Gm32687'	1.09	3.41	2.67	3.77	3.14	0.78	0	4.73	4.43	0.7
102635360	'Gm32719'	0.23	0	0.19	0	0.08	0.09	0	0.24	1.31	0.5
102635385	'Gm32742'	0	0	0.01	1.31	0	0	0	0	0	0
102635552	'Gm32856'	2.48	0.03	0.53	0.05	0.77	1.28	1.78	2.63	3.43	3.65
102635694	'Gm32966'	0.55	0	0.2	0	0.66	0	0	0	0.63	0.18
102635744	'Gm28729'	0.02	2.36	0	0.02	0.02	0	6.72	0.24	0	1.79
102635781	'Gm38481'	5.19	1.78	6.68	8.69	6.74	1.55	3.66	6.69	2.47	5.57
102635802	'Gm33049'	0.06	0.07	0.2	0	0	0	0.55	0	0.3	0
102635879	'4933407O12Rik'	1.95	0	0	0	0	0	0.23	0	0	1.08
102636251	'Gm33370'	0	0	0	0	0.1	0	0	0	0	0
102636264	'Gm33378'	0	0	0.01	0	0	0	0	0.03	0.01	2.65
102636554	'Als2cr11b'	0.28	0	0	0	0	0.04	0.82	0	0	0
102636690	'Gm33691'	0	0	0.14	3.46	0	0	0	0.99	0	0
102636907	'Gm4275'	2.12	4.13	1.68	0	0.03	0	3.35	0.08	2.88	2.15
102636931	'Gm33869'	0.46	0.06	0.56	0.38	0.73	0	0.2	0.38	0.04	2.26
102637020	'Gm33933'	0.6	0.29	0.37	0	0.56	0.09	0.6	1.03	0.45	0.35
102637087	'Gm33989'	0.42	0.28	0.39	0.05	0.4	0.78	0.04	0.01	1.07	0.14
102637099	'Gm38495'	2.77	2.37	1.19	5.67	1.23	2.79	2.7	2.06	3.79	0.91
102637189	'Gm34066'	0.44	0	0	0	0	0	0	0	0	0
102637366	'Gm10037'	1.14	8.23	0	0	7.22	1.36	0.08	5.28	13.8	3.85
102637400	'Gm34220'	0.45	0.05	0.16	2.75	0.41	0.53	0.04	0.15	1.16	2.38
102637572	'Gm38499'	0	1.04	0.66	0	0	0	0	0	2.36	0
102637593	'Gm34362'	0	0	1.48	0	0	0	0	0	0	0
102637806	'LOC102637806'	0	0	0	0	0.45	0	0	0	0	0
102637808	'LOC102637808'	0.03	0	0	0	0	0	0	0	0	0.03
102637839	'Gm10476'	0.06	0	0.24	0.28	0	0	0.4	0.17	0	0
102638047	'LOC102638047'	0	0.05	0.05	0.15	0.13	0	0.16	0.04	0.07	0.12
102638083	'Ccdc188'	0	0	0	0	0	0	0	0.22	0.02	0
102638132	'Gm34768'	0	0	0.33	0	0	0	0	0	0	0
102638268	'1700014D04Rik'	0	0	0	0	0	0	0	0	0	1.68
102638541	'Gm35078'	0.04	0	0	0	0	0	0	0	0	0
102638555	'Gm38510'	0	0.15	1.44	0.03	0	0	0	0	0	0
102638674	'LOC102638674'	0	0	0	0.31	0.03	0	0	0.03	0	0
102638847	'Gm35315'	0.25	0.34	0.57	0.29	0.4	0.07	0	0.27	0.53	0.52
102638882	'Gm35339'	0	0.05	0.25	0	0	0	0.23	0.56	0	0
102638888	'LOC102638888'	0.8	0.91	0.04	0.67	2	0.99	0.09	1.3	1.55	1.58
102638918	'Gm35364'	0.73	0	2.7	3.02	0.16	3.03	0	0	0	0.28
102639021	'LOC102639021'	1.01	0.62	2.61	0.07	0.99	0	1.27	0	1.61	0.22
102639037	'LOC102639037'	0	0	0.42	0	0.68	0	0	0	0	0
102639132	'Gm21885'	1.03	1.14	0.08	4.12	0.52	0.01	0.14	0.91	0.45	0.02
102639178	'Gm35549'	0	0	0	0	2.15	0	0.04	0	0	2.03
102639229	'Gm35588'	0	0	0.38	0	0	0	0	0	0	0
102639505	'LOC102639505'	0.35	1.36	0.94	2.67	1.04	3.4	0.49	0.96	0.3	1.01
102639543	'Ifi206'	0	0	0	0	0	0	0	0	0	0.23
102639598	'Gm14296'	0	1.6	4.83	1.6	3.22	3.15	3.28	4.06	1.89	2.61
102639650	'Vamp9'	0	0	0.41	0	0.88	1.26	0.73	0.71	0	0
102639653	'LOC102639653'	3.86	1.87	5.21	6.55	1.14	3.65	2.25	2.48	3.4	3.59
102640043	'Gm36210'	0	0.16	0	0	0.06	4.49	0	0	0	1.63
102640263	'Gm36375'	0	0.48	0	0	0	0	0	0	0.79	0
102640268	'Gm2956'	2.03	2.19	5.69	0	4.18	1.85	0.63	3.92	3.92	3.54
102640292	'102640292'	0.43	0.46	0.76	0.72	0.37	0.11	1.03	0.63	0.03	0.15
102640376	'Gm36448'	0	0	0	0	0	0	0	0	1.74	0
102640399	'102640399'	7.21	7.43	8.5	12.5	17.6	5.02	14.6	8.81	3.94	10.18
102640444	'Gm36504'	0	0	0	0	0	0	0	0	0	0.18
102640476	'Gm36527'	0.02	0.07	0.87	0.04	0.03	0.01	0.01	0.01	1.21	0.02

102640673	'LOC102640673'	6.31	6.23	1.19	0.04	8.99	8.39	5.53	5.14	9.91	5.63
102640710	'Gm36712'	1.14	3.65	0	0	0	0	3.14	0	0	0.04
102640809	'Gm36789'	0	0	0	2.2	0	0	0	0	0	0
102640833	'Gm36807'	0	0	0	0	0	0	0	0	0	1.38
102640920	'Pvrig'	0	0	0	0	0	0	0	0	2.23	0
102641028	'LOC102641028'	0	0.16	1.62	1.7	1.93	0.34	0.81	0	0.46	2.05
102641068	'Gm38529'	0.75	0.77	1.51	1.46	0.77	0.3	2.89	0.66	2.01	0
102641467	'Gm20684'	0.29	0	0	0	0	0	0	0	0	0
102641557	'Gm38564'	0	0	0.03	0	0	0	0	0	0.01	0
102642386	'LOC102642386'	0	0.02	0	0	0	0	0	0	0	0
102643088	'Gm38656'	0	0	0	0	0	0	0	0	0.23	0
102643210	'Gm38664'	0.02	0	0.12	0	0	0	0	0	0	0
102644	'Oaf'	0	0.16	0	0	0	0	3.72	0	0	0
102657	'Cd276'	3.21	2.56	6.74	7.1	0.32	7.01	0	0	0.6	4.21
102680	'Slc6a20a'	0	0	0	0	0	0	0.05	0	0	0.11
102693	'Phldb1'	8.46	1.8	4.5	6.15	5.79	5.78	5.97	0.99	0.08	1.17
102747	'Lrrc49'	52.9	44.62	56.4	47.5	39.6	42.55	45.7	43.04	40.64	42.31
102774	'Bbs4'	57.78	61.73	80.8	47	70.1	61.78	62.4	72.05	69.92	94.01
102791	'Tcta'	24.06	44.14	56.5	36.4	55.9	83.1	49.7	64.73	65.05	62.56
102857	'Slc6a8'	0.13	0.81	2.51	4.66	4.65	7.29	0	2.26	3.25	0
102866	'Pls3'	30.29	48.65	22.4	29.1	32.8	17.07	45.9	56.34	34.99	49.05
102871	'D330045A20Rik'	1.06	6.5	3.64	14.5	1.17	6.59	0	1.09	34.4	31.51
102902673	'Gm21992'	2.25	0.31	2.36	1.33	0.18	0.07	0.43	8.92	0	0
102920	'Cenpi'	0	0	0	0	0	0.39	0.67	0	0	0
102954	'Nudt10'	39.38	60.51	18.3	27.6	36.1	34.09	35.1	21.45	43.46	11.93
103080	'Sept10'	2.08	1.82	0.13	0.29	0.13	0.32	0.09	0.06	2.47	0.12
103098	'Slc6a15'	26.09	28.49	21.2	25.1	19.7	2.56	10.5	18.66	7.46	20.43
103135	'Pan2'	14.09	8.09	10.7	5.76	13	5.72	7.93	5.27	9.86	9.29
103136	'Pwp1'	19.77	21.52	14.9	0.02	18.7	14.92	4.19	16.49	8.04	11.44
103140	'Gstt3'	3.42	1.44	5.59	7.91	0.05	0.27	0	0	0.23	3.56
103142	'Rdh9'	0.71	1.27	0.62	1.51	0.42	0.59	0.07	0.18	0.32	0.44
103149	'Upb1'	0	0	0.25	0	0	0	0	0	2.26	1.73
103172	'Chchd10'	566.5	351.1	663	414	566	928.6	544	543.4	580.3	380.55
103199	'Fig4'	16.08	14.81	25.3	10.4	19	17.24	13.8	25.1	14.31	17.58
103213	'Traf3ip2'	10.85	0.85	2.75	0.08	2.25	4.93	0.02	1.83	0.34	2.66
103220	'Ttc41'	6.04	7.59	4.18	5.59	2.59	6.58	3.2	6.13	6.26	8.62
103236	'Csnk1g2'	4.98	10.82	4.83	6.72	4.35	9.56	3.89	4.16	4.68	4.18
103266	'Tmem263'	31.31	30.35	13.8	14	19.7	8.63	16.5	16.97	6.03	17.98
103268	'Cep57l1'	3.72	0.95	1.53	5.56	0	10.4	0	5.06	0.84	1
103284	'Zc3h10'	11.53	14.06	0.13	10.7	11.6	2.97	8.81	13.02	11.83	16.25
103406	'Zfr2'	15.94	13.77	38	7.71	21.5	10.51	27.8	5.9	24.91	6.6
103425	'Ncln'	17.68	16.52	17.7	17.7	22.5	10.68	15.3	14.67	9.81	8.3
103466	'Nt5dc3'	2.18	1.76	1.78	3.39	2.64	0.75	1.29	0.74	4.15	0.16
103468	'Nup107'	2.8	9.94	8.64	14.2	7.77	3.56	7.08	6.74	5.89	8.26
103511	'Calhm5'	14.02	0	3.22	0	0.03	7.79	8.26	6.73	9.08	3.19
103534	'Mgat4b'	1.26	2.26	3.35	1.73	1.43	0.02	4.62	0.13	3.92	0
103537	'Mbtd1'	1.77	3.03	1.28	3.03	1.7	0.85	3.69	2.28	5.75	1.25
103554	'Psme4'	2.09	0.35	1.14	4.17	2.04	0.18	2.2	0.72	2.22	0.34
103573	'Xpo1'	7.7	7.98	9.15	10	4.05	2.27	8.87	4.95	22.3	11.41
103583	'Fbxw11'	21.53	23.4	14.3	29.3	19.6	19.87	12.7	18.6	21.11	15.32
103611158	'Gm38666'	0.16	1.9	0.57	1.27	1.08	0	0	0	2.06	1.61
103611159	'Gm38667'	3.78	2.94	0.57	0	0	7.69	0	1	1.21	4.4
103677	'Smg6'	8.21	5.73	5.9	5.03	5.99	3.77	4.71	6.02	4.54	6.58
103694	'Tmed4'	206.3	235.8	118	103	207	255.2	138	202.3	232.9	312.91
103710	'Slc35e4'	20.96	19.35	32	22.5	10.2	15.84	35.9	5.3	11.48	15.79
103711	'Pnpo'	30.56	65.05	73	42.4	102	105.1	109	76.55	73.98	109.16
103712	'6330403K07Rik'	2128	2232	1902	2417	1864	2283	1669	2821	1733	1767.71
103724	'Tbc1d10a'	14.76	11.9	2.35	32.9	12.2	4.62	33.2	16.8	23.17	10.27

103733	'Tubg1'	95.7	81.97	80.2	50.6	88.3	138.7	65.4	82.56	98.99	79.38
103737	'Pex12'	13.59	8.83	8.98	16.8	9.26	5.99	14.7	3.37	8.6	17.01
103742	'Mien1'	186.6	190.8	125	313	207	244.5	198	212	189.6	199.52
103743	'Tmem98'	4.28	2.59	0	0	4.67	9.65	0	1.67	0	2.21
103765	'Tmem17'	16.74	28.92	16.1	38.1	22	21.07	28.5	10.96	34.84	34.54
103768	'Tubg2'	115.8	110.5	94.5	52.5	112	186.6	166	140.1	102.6	106.61
103775	'Slc25a41'	0.91	0	0	0	0.08	0	0	0	0.31	0
103784	'Wdr92'	13.97	10.2	7.41	5.75	3.49	5.78	10.8	8.09	11.02	7.5
103806	'Maml1'	0.04	0.6	1.51	1.98	0.01	0	1.14	0	0.54	0.02
103836	'Zfp692'	8.41	17.58	30.6	13.3	34.6	30	34.7	23.81	28.67	29.61
103841	'Cuedc1'	1.01	1.61	1.63	6.92	3.86	1.76	0.43	2.35	0.83	1.13
103844	'Inca1'	7.39	7.98	3.84	12.8	4.23	13.05	4.81	1.05	23.92	1.24
103850	'Nt5m'	76.17	22.63	46.1	34.7	38.2	60.65	46.8	40.21	43.18	37.66
103963	'Rpn1'	97.51	104	109	102	77.1	90.14	72.8	88.32	130.6	119.84
103967	'Dnm3'	33.3	24.7	30.1	20.5	29.1	33.57	31	19.82	33.26	16.95
103978	'Gpc5'	17.52	24.62	9.49	47.1	20.5	8.6	10.6	9.58	8.52	3.66
103988	'Gck'	51.48	37.42	7	51.7	41.5	25.71	15.7	41.67	9.71	30.97
104001	'Rtn1'	2015	1769	1793	1658	1607	2163	2174	1868	1602	1622.46
104009	'Qsox1'	23.8	22.66	39	20.6	29.2	15.93	27.1	23.9	28	31.3
104010	'Cdh22'	4.43	7.38	22.2	0.67	0.03	1.05	3.28	4.46	0	0.62
104015	'Synj1'	19.87	16.43	28.1	37.6	17.8	13.99	24.3	24.28	26.18	26.01
104027	'Synpo'	1.08	0.7	0.02	1.52	4.95	0.04	6.25	1.63	3.17	3.23
104069	'Sncb'	77.32	69.28	144	32.7	153	89.79	156	145.4	198.1	92.42
104079	'Nxph3'	0	0.07	0	1.2	0	0	0	0.48	0	0
104080	'Nxph4'	5.01	0.55	0.06	0.65	25.9	102.6	0.03	0	239.2	64.88
104082	'Wdr7'	8.49	7.91	23	10.2	8.48	5.38	19.3	6.94	11.71	15.11
104086	'Cyp27a1'	3.94	2.8	4.37	0	2.84	2.76	0	0	0	0.02
104099	'Itga9'	0	0	1.37	0	0	0	0	0.68	0	0
104111	'Adcy3'	7.11	6.05	5.8	2.59	4.58	2.02	5.81	2.53	9.83	2.71
104112	'Acly'	43.01	36.55	54.7	67	38.7	6.65	56.8	42.96	60.69	84.07
104130	'Ndufb11'	260.4	234.8	212	170	311	437.4	244	248.1	251.7	287.2
104156	'Etv5'	7.21	9.96	8.45	12.1	3.49	5.22	8.3	3.99	14.26	10.27
104158	'Ces1d'	0.04	4.9	0.02	0.06	0	6.14	0	0	0.02	1.69
104174	'Gldc'	0	0.95	1.25	3.42	0	0.02	0	0	0.67	0.5
104175	'Sbk1'	0.02	0	0.01	0	0.08	0	1.45	1.36	0	0.07
104184	'Blmh'	69.42	57.74	51.3	49.5	53.8	73.95	89.3	67.11	58.49	61.98
104215	'Rhoq'	1.05	2.92	0.74	0.01	1.32	0.67	0.13	0	5.81	1.51
104248	'Cabin1'	9.01	5.64	7.81	7.05	8.85	3.46	21.4	11.05	7.36	11.67
104252	'Cdc42ep2'	6.29	7.21	16.3	0	11.1	7.4	0	5.15	4.2	4.2
104263	'Kdm3a'	2.18	5.56	3.28	2.87	2.44	3.2	6.67	5.58	7.88	8.92
104271	'Tex15'	2.33	0	0	0	0	0	0	0	0.02	0
104303	'Arl1'	108.7	104.4	117	90.1	123	121	102	145.7	156.8	113.14
104318	'Csnk1d'	19.27	31.7	26.7	43.3	12.7	21.58	29	26.39	27.01	18.25
104346	'Gas8'	39.19	13.81	13.6	36.6	14.7	50.79	18.9	36.72	28.7	19.33
104348	'Zfp120'	2.72	3.57	1.01	0.01	3.75	0.06	1.99	2.08	1.21	6.07
104349	'Zfp119a'	0	0.14	2.58	0.12	2.4	1.78	0	1.58	4.03	1.02
104362	'Meig1'	16.07	33.09	10.6	8.61	20.1	30.77	16.2	24.82	7.02	61.82
104382	'Barhl2'	0	0	0	0	3.37	3.66	0.15	1.19	0	0.68
104383	'Rcor2'	0	0.74	1.51	0.45	0	1.3	0	2.75	0.13	0
104394	'E2f4'	2.08	5.65	6.5	11.5	4.23	0.09	3.17	2.94	1.49	0.43
104401	'Pcnx3'	1.45	0.64	1.98	1.82	1.44	2.63	1.07	1.68	0.17	0.71
104416	'Bap1'	46.18	40.45	56.6	17.4	34.2	34.16	51.9	55.01	60.65	70.51
104418	'Dgkz'	2.33	1.49	7.76	13.3	9.81	1.92	8.77	1.17	10.14	2.07
104443	'Npffr2'	0	0	0	0	0	0	0	2.1	0	0
104444	'Rexo2'	68.56	44.16	49.9	122	70.4	65.83	43	44.25	86.16	30.41
104445	'Cdc42ep1'	7.41	9.57	2.94	11.9	0.04	26.55	8.48	2.16	3.91	0
104457	'0610010K14Rik'	54.74	84.3	52	36.3	84.2	93.57	76.8	38	55.79	94.96
104458	'Rars'	42.52	32.56	47.4	36.7	36.5	45.98	45.3	30.62	44.92	43.58

104479	'Ccdc117'	3.88	0.45	0.89	7.12	2.05	1.11	2.14	0.02	0.06	0.95
104570	'Ppp4r3b'	7.54	11.53	8.85	7.24	6.8	9.87	6.46	10.34	7.42	11.98
104582	'Rprml'	19.43	11.04	12.7	13	66.1	74.66	19.9	23.76	0.04	0
104601	'Mycbpap'	1.87	5.08	1.16	0	8.18	7.51	2.63	4.04	8.16	7.46
104625	'Cnot6'	1.65	0.73	0.39	4.38	3.06	0.2	0.02	2.81	2.4	1.99
104662	'Tsr1'	5.29	11.43	5.76	6.89	12.5	16.6	6.08	4.8	7.98	7.32
104681	'Slc16a6'	0.57	1.56	1.18	0	0.51	5.38	7.35	0.25	0.4	14.46
104709	'Pik3r6'	0	0	0	0	1.59	0	0	0	0	0
104718	'Ttc7b'	6.19	3.3	10.6	8.81	11.4	6.52	0.78	7.06	7.88	4.51
104721	'Ddx1'	92.05	60.29	104	71	43.2	56.7	98.6	79.51	114.2	121.86
104725	'Sptssa'	63.02	76.72	43.3	129	78.9	55.44	28.2	68.59	33.24	78.45
104732	'Tedc1'	4.91	0	0.25	0	0	0	1.22	0	0	2.43
104759	'Pld4'	0	0	0	0	0.05	13.97	12.9	0	5.17	0
104771	'Jkamp'	163.9	121.1	109	133	149	173.4	122	159.6	182.4	170.56
104776	'Aldh6a1'	32.11	22.07	22.5	17.3	21.4	17.32	39.1	18.36	24.08	16.62
104799	'Vipas39'	39.6	22.94	36.3	20.3	32.8	17.84	8.7	38.88	23.16	22.51
104806	'Fancm'	0.17	1.63	1.23	0	3.72	0.01	4.05	0.14	0.96	0.91
104816	'Aspg'	0	0	0	0	0.05	0	0	0	0	0
104831	'Ptpn23'	11.31	3.73	1.6	5.5	2.03	3.62	1.36	2.49	8.62	5.3
104836	'Cbll1'	4.02	4.2	7.36	0	4.41	4.26	2.64	8.66	10.43	4.01
104859	'Tecpr2'	0.34	1.21	2.98	3.16	3.96	0.83	0	0.32	0.72	0.94
104871	'Spata7'	17.49	9.73	14.9	11.6	10.2	13.17	24	10.91	15.44	4.2
104884	'Tdp1'	12.32	9.06	27.3	22.9	8.57	0.04	16.7	5.96	10.76	12.15
104885	'Tmem179'	265.7	288.8	291	86.2	275	264	316	256.4	214.6	241.38
104886	'Rab15'	11.39	8.87	12.1	27.8	12.2	0	20.6	8.39	17.71	6.2
104910	'Slc25a47'	0	2.12	0.74	0	7.75	3.13	3.36	8.86	0	0.71
104923	'Adi1'	27.28	54.6	54.7	11.9	38.3	43.01	48.4	44.52	15.75	57.94
105000	'Dnal1'	3.92	1.99	3.62	0.05	6.3	5.59	1.16	0.8	4.02	1.81
105005	'Fam84a'	49.54	29.65	27.7	19.5	5.6	16.1	27.8	84.69	16.98	2.7
105014	'Rdh14'	32.29	32.49	34.2	21.9	26.6	32.03	24.2	29.17	40.67	33.32
105083	'Pelo'	3.09	0.12	0.06	0	2.82	5.56	1.32	1.6	4.8	3.54
105148	'Iars'	18.61	11.09	20.1	3.96	7.05	4.15	21	19.75	14.22	14.74
105171	'Arrdc3'	3.64	6.78	6.07	0.11	4.63	5.26	5.24	2.78	1.03	6.79
105180375	'Tmem265'	2	0.36	0.81	0	6.96	2.15	0	1.04	0.5	4.01
105193	'Nhlrc1'	12.44	4.82	5.54	16.2	1.63	0.02	4.94	11.5	15.09	4.07
105203	'Fam208b'	3.92	3.25	4.74	7.91	3.19	2.51	2.39	2.13	1.99	1.5
105239	'Rnf44'	7.73	2.24	8.41	1.14	4.96	3.98	11.1	8.94	9.63	9.81
105242399	'Gm21083'	0	0.29	0	0	0.5	0.55	0.05	0.12	0	0
105242416	'105242416'	0	0.03	0.06	0	0	0.12	0	0	0	0
105242418	'Gm38690'	3.94	1.29	1.8	0	4.34	0.53	0.65	2.79	7.01	4.73
105242430	'Gm38699'	1.21	0.3	0.75	3.19	0.33	0.74	0.43	1.28	1.48	0.22
105242433	'Gm38702'	0.65	0.73	1.92	0.25	0.97	0.37	1.61	0.44	1.31	0.4
105242435	'Gm38704'	0.05	0	0.47	0.14	0.08	0	0.17	0.13	0.25	0.18
105242449	'LOC105242449'	0	0	0	0	0	0	0.08	0	0.06	0
105242453	'LOC105242453'	0.08	0.41	0.13	0	0	0	0	0	0	0
105242736	'LOC105242736'	0.21	0.08	0.72	3.82	0	0.08	0.17	0.47	0.94	0.07
105242930	'Gm39002'	0	0	0.05	0	0	0.17	0	0	0.54	0
105243090	'LOC105243090'	0	0	0	0	0	0	0	0	0	0.53
105243585	'Gm39469'	8.4	8.26	17.8	18.5	17.6	7.15	10.2	20.68	18.68	17.34
105243944	'Gm39653'	0	0	0	0	0	0	0	3.56	0	0
105244006	'Gm39701'	0.25	0.27	0.3	0.87	0.36	0.71	1.72	0.43	0.38	0.92
105244063	'Gm39743'	0	0	0	0	0	0	0	0.04	0	0
105244392	'Gm40011'	0	0	0	0	0	0	0.02	0	0	0
105244402	'LOC105244402'	0.41	0.42	0.04	5.2	0	0	0	1	1.36	0.37
105244808	'Gm40353'	0	0.04	0.08	0	0	0	0	0	0	0
105244826	'Gm40363'	7.91	7.84	13.6	9.98	8.91	4.58	2.39	9.46	7.15	9.15
105244829	'Gm40365'	1.42	0.55	0.49	0	0	0	0.4	0	1.8	0.74
105244832	'Gm40368'	9.18	5.1	8.24	7.62	8.09	12.76	8.98	7.78	14.43	7.34

105244833	'Gm40369'	0.07	0	0	0	0	0	2.78	0.18	0	0
105244844	'Gm40378'	0	0	0	0	0	0	0.08	0	0.06	0
105244925	'Gm40447'	0.02	0	0	0	0	0	0	0	0	0
105244931	'Gm40453'	0.55	0.34	0	0.13	0.55	1.09	0	0	0	0
105244938	'Gm40460'	0	0	0	0	0	0	0	0	0	0.03
105244980	'Gm40498'	0	0.02	0.31	0.01	0	0.76	0	0.04	0	0.13
105244999	'Gm40514'	0	0	0.02	0	0	0.03	0	0	0.03	0
105245	'Txndc5'	20.2	19.62	16.4	46.8	6.94	8.82	39.4	22.62	50.07	8.62
105245043	'LOC105245043'	0	0	0.01	0	0	0	0	0	0	0.85
105245047	'Gm40556'	0.62	0	0.15	0	0	0	0	0	2.18	0
105245097	'Gm40595'	0.03	0.56	0.82	0	0.22	2.25	0.79	0.34	0.74	0.61
105245106	'Gm40600'	0	0	0	0	0	0	0.09	0	0	0
105245236	'LOC105245236'	0	0	0	0	0	0	0.08	0	0	0.29
105245342	'Gm40814'	4.07	4.06	3.38	5.87	3.56	0	3.31	0.46	0.52	3.82
105245353	'Gm40824'	0.57	0.79	1.45	2.49	0.44	0.47	0.41	0.81	0.53	0.79
105245369	'Gm40835'	0.11	0.07	0	0	0.02	0	1.41	0.11	0	0
105245381	'Gm40847'	0.05	0.4	1.31	0.04	0.23	0.44	1.26	0.7	0.75	0.3
105245383	'Gm40848'	1.21	1.97	1.67	1.38	1.94	0.91	4.39	1.42	2.35	1.13
105245389	'Gm40853'	1.79	2.82	3.56	0.93	5.22	0	4.92	2.66	9.13	1.14
105245400	'Gm40862'	0.42	0	0.08	0	0.36	0.12	0.13	0.14	0.39	0.06
105245402	'Gm40864'	0.05	0.02	0.07	0	0	0.05	0.39	0	0.64	0
105245406	'LOC105245406'	0	0.1	0	0	0.12	0	0	0.03	0.93	0.03
105245424	'Gm40881'	0.13	0.05	0.25	0.43	0.07	0	0.21	0.11	0.06	0.04
105245547	'Gm40991'	0	0	0	0	0	0.09	0.13	0	0.04	0
105245580	'A930018O16Rik'	5.06	2.05	0	0	0	0	0	0	0	0
105245604	'Gm41035'	0	0	0	0	0	0.04	0	0.11	0	0
105245668	'Gm26650'	0	0.09	0	0	0	0	0	0.14	1.25	0
105245673	'LOC105245673'	0	0	0	0	0	0	0	0.04	0	0
105245675	'LOC105245675'	0	0	0.23	0	0	0	0	0	0	0
105245682	'Gm41099'	0	0.02	0.13	0	0	0.26	0.18	0	0.17	0
105245737	'LOC105245737'	0	0	0.5	0	0.45	0	0	0	0	0.01
105245911	'Gm41291'	2.02	0.78	3.05	3.14	4.84	0.82	4.46	5.15	0.97	3.32
105246	'Brd9'	14.44	33.64	34.8	18.8	24.5	42.98	16.3	29.9	31.87	30.96
105246138	'Gm41476'	0	0	0.07	0	0	0.05	0	0.19	0	0
105246320	'Gm26637'	0	0.05	0.01	0	0	0	0	0	0	1.14
105246512	'Gm41793'	0	0.2	0	0	0	0	0	0	0	0
105246572	'Gm41844'	2.12	1.54	2.77	1.93	2.02	1.98	2.28	0.62	0.7	2.63
105246807	'Gm42031'	0.05	0	0.82	0	0	0.95	0	0	0.01	0.39
105246811	'105246811'	54.78	67.56	62.7	30.3	45	53.67	46.9	40.24	60.55	83.89
105246828	'Gm42052'	1.23	0.81	1.54	1.68	0.65	2.08	0.71	0.66	4.48	1.35
105246872	'Gm42078'	0	0	0	0.07	0	0	2.23	0	0	0.7
105246961	'AB010352'	0.22	2.56	0.04	0.59	1.05	0.01	2.17	0.03	0.38	2.27
105246978	'Gm42166'	0.16	0.43	0.39	1.2	0.69	0.2	0.25	0.15	0.32	0.15
105247075	'LOC105247075'	0	0	0	0	0	0	0	0	1.83	0
105247125	'105247125'	0	0	0	0	0	0	1.54	0	0	0
105247180	'Gm42323'	0	0.07	0	0	0	0	0	0	0	0
105247240	'Gm42372'	10.72	10.62	12.8	14.1	13.4	13.38	13.7	9.93	3.77	17.61
105247297	'LOC105247297'	0	0	0.06	0	0	0.03	0.03	0	0	0
105278	'Cdk20'	24.59	16.29	28.8	8.94	9.73	41.51	46.6	17.46	5.79	11.93
105298	'Epdr1'	204.1	200.3	165	160	107	153.4	126	108.7	262	110.39
105348	'Golm1'	2.82	1.65	0.91	0	1.46	10	0	0	0.14	0
105351	'AW209491'	34.32	45.07	45.8	40.6	43.8	47.05	40.9	63.9	73.51	72.53
105352	'Dusp22'	21	20.98	18.5	6.9	17.5	6.95	10.5	14.28	10.07	12.2
105372	'Utp15'	8.99	8.89	15.1	19.3	7.38	3.69	8.84	8.87	4.21	13.6
105377	'Slf1'	3.17	1.64	1.14	5.96	3.13	4.3	4.02	5.35	3.12	8.23
105418	'E330034G19Rik'	0.03	0	0.1	0	0	0	0	0	0	0
105428	'Fam149b'	9.01	5.03	0.08	6.38	2.08	9.83	0	1.62	3.44	2.74
105439	'Slain1'	1.82	2.69	0.37	5.85	2.07	0	0	0.1	3.07	2.96

105440	'Kctd9'	0.44	2.83	0.96	2.26	4.09	0.03	0.03	2.59	0	5.58
105445	'Dock9'	3.62	1.94	4.31	6.28	1.07	1.89	4.56	5.77	2.48	0.46
105446	'Gmpr2'	39.34	26.15	25.1	24.9	30	46.51	23.2	30.03	34.18	34.68
105450	'Mmrn2'	0.47	0.05	0	0.95	0	0	0	0.57	0.15	0
105501	'Abhd4'	19.98	13.62	25.6	9.84	5.55	59.4	7.28	12.9	23.02	12.85
105504	'Exoc5'	11.06	6.68	14.4	13.8	5.87	4.89	3.82	9.58	10.83	0.37
105513	'Chmp7'	29.28	19.98	19.8	29.5	15.2	4.32	36.8	12.6	16.58	4.73
105522	'Ankrd28'	0	1.19	1.71	0	2.74	0.03	0.69	0.34	2.83	0.56
105559	'Mbnl2'	72.24	61.09	38	40.2	54.6	48.2	73.3	65.52	59.64	100.71
105590	'Zfp957'	0	0	0.02	0	0	0	0	0	0	0
105638	'Dph3'	19.02	15.31	18.6	17.2	20.2	10.99	31.3	8.47	19.1	19.65
105651	'Ppp1r3e'	0	0	0	0	0.03	1.09	1.85	0	0.09	0
105653	'Phyhip'	119.3	135.4	143	42.9	103	85.57	151	114.6	55.83	94.04
105663	'Thtpa'	10.56	8.95	22.3	14	20.1	17.84	15.8	18.21	13.06	14.85
105670	'Rcbtb2'	7.9	6.14	9.31	16.3	3.18	5.58	4.34	6.2	7.06	8.83
105675	'Ppif'	14.48	16.3	40.5	19	27.6	35.79	23.1	17.16	42.86	9.86
105689	'Mycbp2'	6.72	12.71	11.8	20.8	11.9	9.07	18.5	8.24	8.58	10.47
105704528	'Gm13090'	0.02	0.58	0	0	0	0	0	0	0	0
105722	'Ano6'	4.99	5.82	6.39	2.74	8.19	1.24	0	4.28	4.02	8.84
105727	'Slc38a1'	30.42	36.18	42.8	22.6	36.1	25.82	37.5	29.96	55.28	35.19
105732	'Fam83h'	0	0	0	0	1.04	0	0	0	0	0
105734	'Tigd5'	0.02	0.02	0	2.28	0	0.07	0.04	0.05	0	0.66
105734727	'Gm27021'	3.14	0	6	0	0	4.07	0	5.29	11.88	2.15
105734734	'Gm11715'	0	0	0	0	0	1.65	0	0	0.31	0
105782	'Scrib'	2.64	3.5	0.93	5.22	1.95	0.1	0	3.19	8.42	1.24
105785	'Kdelr3'	0	0	0	0	0	1.12	0	0	0	0
105787	'Prkaa1'	6.53	7.67	12.2	5.53	16.4	2.96	7.94	2.45	4.83	4.62
105827	'Amigo2'	12.1	7.23	13.8	5.7	1.31	3.58	3.6	12.37	16.67	1.09
105833	'Ccdc65'	11.34	14.18	10.3	1.19	17.1	26.82	0	6.38	8.66	16.73
105835	'Sgsm3'	36.11	33.24	29.4	53.8	42.8	35.33	39.2	47.46	31.28	46.21
105837	'Mtbp'	0	0	0	4.87	0	0	0	0	0.44	0
105841	'Dennd3'	1.44	0	0.77	0.05	0.58	2.65	0	0.03	0.02	1.41
105844	'Card10'	0	0.13	1.66	0	0	0	0	0.01	0.61	0
105847	'Lmf2'	13.06	1.72	4.76	17.4	12.2	2.21	9.22	2.62	9.45	9.51
105853	'Mal2'	17.74	7.77	12	0.71	12.7	19.06	23.5	15.74	15.45	3.29
105855	'Nckap1l'	0	0	0	0	1.33	0	0	0	0	0
105859	'Csdc2'	97.95	107.7	138	36.3	111	107.6	115	101.7	152.6	83.35
105886298	'Cmc4'	19.54	13.84	1.2	3.75	14.6	8.02	25.2	13.21	15.88	13.57
105940408	'Gm20498'	46.88	46.77	49.9	49.7	34.7	60.39	32.6	41.44	41.92	22.68
105943584	'Gm45927'	0.82	0.07	0	0	0.97	1.09	0	0.39	0.77	0
105980076	'Gm45929'	0	0.01	0	0	0	0	0	0	0	0
105988	'Espl1'	0	0.09	0	0	0	0	0	0	1.94	0.03
106014	'Fam19a5'	21.6	21.06	15.5	26.2	23.1	7.03	3.64	12.62	8.48	10.59
106021	'Topors'	18.18	17.04	9.37	9.1	4.5	7.06	20.7	14.35	14.87	19.12
106025	'Sharpin'	22.04	24.08	34	25.1	53.8	31.06	39.2	61.17	30.37	39.68
106039	'Gga1'	13.51	14.18	16.7	11.2	12.4	15.66	31.2	15.91	24.68	18.84
106042	'Prickle1'	4.37	3.7	1.19	0	3.86	0.98	2.39	2.37	5.53	2.91
106052	'Fbxo4'	3.2	0.51	2.89	0.7	2.37	0	0.03	0.14	0.05	1.44
106064	'AW549877'	2.5	3.54	8	4.11	4.43	4.01	7.62	3.1	20.83	8.14
106068	'Slc45a4'	1.38	3.38	1.73	2.82	4.53	3.5	0.13	3.75	0.39	2.5
106073	'Mfsd5'	11.18	8.74	11.7	3.13	17.7	17.48	18.2	20.09	16.49	12.79
106143	'Cggbp1'	12.25	5.4	10	12.4	5.37	2.96	5.25	4.23	9.59	8.04
106200	'Txndc11'	0.85	5.96	3.86	4.22	9.37	6.14	8.51	3.16	12.04	10.73
106205	'Zc3h7a'	7.77	10.89	10.4	4.06	18.2	9.44	2.18	8.53	12.56	17.52
106248	'Qtrt2'	6.55	2.85	4.33	3.45	6.44	6.1	4.4	3.11	3.84	4.21
106298	'Rrn3'	35.33	54.74	44.4	22.2	30.3	9.7	30.2	40.03	50.82	44.21
106326	'Osbpl11'	4.35	0.89	1.92	0.3	2.46	0.36	0.13	0.61	1.43	0.62
106338	'Nsun3'	3.51	4.7	3.07	0	10.6	3.79	0.2	4.04	10.86	11.92

106344	'Rfc4'	7.59	2.15	4.86	1.87	10.6	0.04	0	4.29	0.11	13.22
106347	'Ildr1'	0	0.57	0	0	0	0	0	0	0	0
106369	'Ypel1'	1.58	4.41	3.52	0	2.77	5.53	11.1	6.77	0	0
106389	'Eaf2'	1.46	2.23	3.45	3.17	2.9	1.48	1.69	1.35	10.47	2.7
106393	'Srl'	0.04	0.01	0.06	0.02	0	0.01	0.05	0.34	0.02	0.05
106407	'Slc51a'	0	0	0.13	0	0	0	0	0	0	0.45
106489	'Sft2d1'	95.57	53.93	44.7	102	87.8	103.4	61.7	104.7	62.5	115.77
106504	'Stk38'	18.82	5.16	13.2	0.67	5.53	6.27	11.3	11.82	13.05	9.67
106512	'Gpsm3'	0	1.86	0	1.38	0	1.42	0	2.95	2.09	2.17
106522	'Pkdcc'	2.63	2.61	0	3.17	1.5	0	0.04	1.81	1.83	0.04
106529	'Tecr'	1897	1472	1477	1512	1525	1800	1502	2109	1800	1843.64
106564	'Ppcs'	31.7	10.74	22.3	44.8	22.8	18.31	36.4	35.68	18.69	29.73
106565	'Dlk2'	10.32	13.13	8.66	17.1	8.93	12.21	21.5	9.7	8.73	5.07
106572	'Rab31'	7	10.73	15.4	6.23	6.69	14.51	19.2	12.17	2.8	1.6
106581	'Fam234a'	5.32	7.54	12.4	13.6	14.5	12.82	12.4	1.04	2.32	2.57
106582	'Nrm'	0	0.03	0.03	0	3.95	0	8.91	2.25	0	0
106583	'Scaf8'	3.39	1.35	4.44	6.67	0.58	2.19	8.34	0.94	2.93	3.21
106585	'Ankrd12'	11.41	7.01	9.66	12.8	15.5	14.16	11.1	9.14	7.21	8.67
106618	'Wdr90'	2.41	1.13	0.19	1.74	0	0	2.67	0.15	4.56	2.53
106628	'Trip10'	0.25	0	0.02	0.02	0.37	0.02	2.27	8.06	8.26	0
106633	'Ift140'	3.16	2.38	1.56	0.12	2.36	0.78	3.01	6.92	1.79	7.16
106639	'Vmac'	6.26	10.28	6.71	0	31	17.11	5.1	14.83	10.93	10.65
106648	'Cyp4f15'	3.03	1.21	3.7	2.8	0.02	0.02	6.13	0.03	2.28	1.23
106672	'AI413582'	242.4	111.8	157	193	153	211.2	233	207.3	162.8	207.86
106707	'Rpusd1'	70.96	69.21	67.5	45.1	92.1	60.5	54.6	53.82	47.36	56.19
106757	'Catsperd'	0	0	0	0	0	2.61	5.09	1.64	0.1	0.37
106759	'Ticam1'	0	0	0.2	0	0	0	0	0	4.38	2.45
106763	'Ttbk1'	0.43	0.26	0.53	0.04	5.08	0.15	1.17	0.38	0.44	0.12
106766	'Stap2'	0.12	2	0	0	5.09	0.08	0	0.84	0	0
106794	'Dhx57'	6.99	9.69	5.14	16.7	12.3	8.23	10.5	10.14	12.64	14.38
106795	'Tcf19'	0	0	2.52	0	0	0	0	2.04	4.02	0
106821	'Oard1'	18.26	26.92	31.6	55.3	30.9	19.01	33.6	18.15	52.5	18.12
106840	'Unc119b'	7.43	7.05	9.78	0.02	1.73	0	7.59	1.88	2.92	0
106861	'Abhd3'	64.09	28.8	31.7	59.3	16	59.4	14.8	45	48.37	30.89
106869	'Tnfaip8'	4.5	0	0.07	0	0	0.05	4.32	0.02	0	0.86
106877	'Afap1l1'	1.25	2.02	1.41	0	5.39	0	1.63	0.73	2.35	2.71
106878	'Smim3'	0	0.43	4.01	6.9	3.83	0	4.85	6.11	12.38	0
106894	'Hmgxb3'	5.38	1.25	6.87	6.71	5.55	0.15	6.41	0.33	5.73	2.9
106931	'Kctd1'	1.08	0.22	1.99	0	0.95	11	6.25	0.66	1.07	0.07
106947	'Slc39a3'	1.21	2.99	8.67	8.36	0.72	9.58	6.17	7.02	2.89	6.96
106952	'Arap3'	0	0.49	0.28	0	0	0	0	0	0.04	0
106957	'Slc39a6'	35.96	37.43	49.4	44.8	24.7	30.42	40.8	45.95	51.36	30.49
107022	'Gramd3'	4.3	0	0.08	0.26	6.09	0	5.14	3.32	3.07	0
107029	'Me2'	24.75	20.11	6.48	23	16	8.74	6.72	22.6	14.86	16.96
107035	'Fbxo38'	11.48	10.97	12.9	21.8	4.86	4.46	13.8	18.04	21.86	12.26
107045	'Lars'	13.33	10.59	17	5.5	9.8	5.71	15.7	10.9	8.02	11.79
107047	'Psmg2'	31.97	45.58	27.8	17.5	26.3	52.35	42.3	31.86	30.09	56.25
107065	'Lrrtm2'	11.14	9.89	10.6	13.1	5.93	2.86	8.73	4.81	2.56	7.41
107071	'Wdr74'	28.78	49.63	51.3	31.3	58	54.48	20.1	52.78	41.88	39.09
107094	'Rrp12'	6.94	17.64	13.9	16.7	2.08	2.88	6.69	3.15	16.59	6.46
107173	'Gpr137'	51.76	38.48	68.3	4.39	54.2	33.02	79.5	57.82	31.06	46.81
107182	'Btaf1'	1.15	0.46	0.79	2.71	1.23	1.68	0.33	0.14	2.05	0.85
107197	'Uqcc3'	20.83	46.61	57.4	61	84.9	62.59	35.1	63.87	35.92	43.55
107221	'Ffar4'	0	0	0	0	0.11	0	0	0	0	0
107227	'Macrod1'	0.01	4.35	1.37	5.96	2.79	0.11	0.2	1.6	4.06	0.17
107239	'Carns1'	1.71	6.57	5.23	0.09	11.9	7.27	0	2.73	1.92	0.19
107242	'AI837181'	5.12	18.43	2.94	5.16	26.1	29.45	15.7	13.93	4.31	9.46
107250	'Kazald1'	0	0	14.3	3.84	3.51	0.03	5.95	6.05	0	0

107260	'Otub1'	360.6	352.3	404	418	491	446.2	451	570.6	432	371.96
107271	'Yars'	87.11	72.19	70.9	92.3	45	86.42	93.7	77.4	47.4	70.28
107272	'Psat1'	20.42	20.75	35.2	10.3	18.1	24.11	15.8	14.62	14.71	17.2
107303348	'Gm45935'	0	0	0	0	0	0	0	0	0	0.04
107305	'Vps37c'	9.38	11.3	4.38	0.02	19.9	18.66	16.2	16.22	37.12	14.47
107321	'Lpxn'	0	0	0	0	0	12.78	4.83	0	0	0
107328	'Trpt1'	16.94	15.9	2.89	13.4	20.8	20.28	13.5	13.7	7.15	6.29
107338	'Gbf1'	8.39	11.21	10.9	12.2	6.42	8.17	13.2	12.87	18.27	12.93
107351	'Kank1'	0.2	3.54	3.29	0.7	0.14	3.28	16.3	4.41	0.05	0
107358	'Tm9sf3'	5.41	4.18	3.75	3.55	7.46	1.02	6.78	3.02	1.66	4.62
107368	'Pdzd8'	1.12	3.22	2.49	3.64	6.52	1.13	5.43	0.67	2.15	1.49
107371	'Exoc6'	6.28	2.47	6.6	5.71	1.83	0.38	4.93	4.55	9.75	5.7
107373	'Fam111a'	0	0	0.11	0	0	0.45	0	0	1.88	0
107375	'Slc25a45'	0	0	0	0.56	4.43	0	0	1.36	0	0
107392	'Brms1'	38.97	24.82	14.2	16.8	20.7	28.63	0.8	20.22	20.74	25.08
107435	'Hat1'	26.04	20.39	15.6	5.52	14.5	30.96	40.3	15.79	51.35	35.5
107448	'Unc5a'	6.64	5.89	4.98	1.45	7	18.63	11.6	10.14	5.88	6.92
107449	'Unc5b'	0.85	0.13	0	0	4.55	2.84	0.01	0	0.59	2.46
107476	'Acaca'	2.04	0.89	4.76	6.66	2.32	1.33	2.53	2.87	1.5	3.25
107477	'Guca1b'	0.4	0	0.03	0	0	0	0	0.12	0	3.35
107503	'Atf5'	0.03	0.86	0	5.55	5.6	0.61	0	0.23	6.4	0.07
107508	'Eprs'	22.57	21.25	40.3	47	22.2	37.34	31.7	24.73	33.72	46.1
107513	'Ssr1'	80.24	116.6	67.6	117	63.3	92.63	52.5	78.57	108.2	148.14
107515	'Lgr4'	2.23	6.08	2.53	3.33	2.63	1.48	1.16	3.08	4.29	3.21
107522	'Ece2'	33.97	28.57	39.7	18.6	40.6	24.55	28.8	39.48	36.86	51.88
107527	'Il1rl2'	0	0	0	0	0	2.04	0	0	0	0
107528	'Magee1'	137.8	121.9	146	42.8	99.3	76.11	129	110.8	131.8	153.28
107566	'Arl2bp'	9.17	17.2	23	15.6	13	3.34	7.48	11.64	14.2	8.29
107568	'Wwp1'	0.76	1.24	2	1.84	2.36	0.3	1.21	0.24	1.13	0.83
107569	'Nt5c3'	45.46	80.25	62	8.92	45.5	36.66	57.5	55.01	40.67	45.47
107581	'Col16a1'	0.87	2.97	8	4.29	5.77	1.94	12.9	4.05	0	0
107589	'Mylk'	0.04	0.16	0.42	0	0.31	0	0.2	0.32	0.02	0
107605	'Rdh1'	0.51	0.75	1.06	1.15	1.27	0.67	0.93	0.51	1.3	0.55
107607	'Nod1'	0	0	0.09	0	0.87	0	0	0.79	0	0
107650	'Pi4kb'	6.34	6.52	5.13	0	8.17	0.95	2.79	6.96	4.62	2.57
107652	'Uap1'	8.14	11.67	3.57	1.3	7.83	3.72	3.98	6.95	5.07	4.24
107684	'Coro2a'	1.05	0.74	3.35	2.64	7.96	1.51	2.09	3.36	2.6	0.04
107686	'Snrpd2'	202.8	214.6	216	356	201	336.5	138	168.6	311.7	227.29
107701	'Sf3b4'	90.14	69.41	98.7	43	64.1	79.8	71.7	113.2	98.52	93.99
107702	'Rnh1'	92.18	70.92	53.2	47.8	40.6	75.94	77.7	69.03	96.23	87.73
107723	'Slc12a6'	4.83	3.57	4.66	3.55	2.39	1.58	4.46	3.92	4.05	1.08
107732	'Mrpl10'	55.06	72.67	74.9	41	69.9	101.2	131	79.3	48.37	60.85
107733	'Mrpl41'	82.02	64.33	57.3	56.2	49.3	108.2	72	83.58	48.16	77.4
107734	'Mrpl30'	123.4	149	109	224	95.2	149.1	133	139.8	150.3	132.51
107746	'Rapgef1'	0.02	1.42	2.9	9.97	4.79	0.04	2.99	2.34	1.91	3.47
107747	'Aldh1l1'	17.32	2.84	4.4	0.02	2.1	6.16	9.93	2.73	8.7	0.15
107751	'Prrxl1'	0	0	2.09	0	0	0	0	0	0	0
107765	'Ankrd1'	0.28	0.05	1.21	0.23	0.22	0.34	0.54	0.09	0.29	0.29
107766	'Haao'	0	0.36	0	0	0	0.02	0	0	0	0
107767	'Scamp1'	89.48	76.2	77	54.7	77.6	28.17	94.1	86.31	92.14	76.79
107769	'Tm6sf1'	1.76	0.53	1.9	0.29	0.28	9.45	0.04	4.77	0.64	3.32
107770	'Tm6sf2'	0.03	0.67	0	1.28	2	0	0	0	0	0
107771	'Bmyc'	185.3	161.1	174	92.1	184	272.2	206	162.4	171.6	206.32
107815	'Scml2'	0	0.1	0	2.19	0	0	0	0	0	0
107817	'Jmjd6'	11.84	15.25	15.4	33.7	26.3	23.74	10.6	11.97	25.31	21.35
107823	'Nsd2'	5.54	3.32	2.49	4.63	5.05	3.99	7.22	0.83	5.62	4.58
107829	'Thoc5'	6.32	16.36	21.4	0.84	15.6	22.58	15.4	21.48	13.56	5.89
107831	'Adgrb1'	2.87	2.29	1.32	2.67	3.25	2.85	1.37	1.6	3.15	1.54

107869	'Cth'	3.44	6.77	0	0	3.82	10.08	0.07	0	3.23	0
107885	'Mthfs'	3.42	5.09	7.38	24	12.4	13.11	13.1	8.77	4.76	17.47
107895	'Mgat5'	5.22	4.39	2.4	1.4	9.23	4.69	4.24	3.85	8	5.4
107932	'Chd4'	7.38	8.06	5.68	9.48	11	10.73	6.31	6.7	14.04	10.15
107934	'Celsr3'	0.53	1.7	2.82	1.74	1.59	1.04	3.4	1.24	2.48	0.74
107939	'Pom121'	2.54	3.37	2.72	0.5	2.45	0.08	5.41	1.67	1.44	1.22
107951	'Cdk9'	9.48	3.38	6.34	41.2	20	9.25	10.4	8.32	11.34	1.84
107970	'Hist1h1t'	0	0	0	0	0	0	0	0	2.06	0
107971	'Frs3'	7.55	21.24	23.3	0	10.9	23.39	15	23.05	29.51	13.07
107975	'Pacs1'	4.37	5.71	13.5	4.65	4.34	11.24	7.22	10.76	5.34	5.27
107976	'Babam2'	14.96	9.56	16.5	24	10.9	29.65	9.46	15.87	17.89	38.02
107986	'Ddb2'	2.5	10.45	3.84	5.75	0.33	10.67	20.8	10.56	8.54	15.27
107993	'Bfsp2'	0	2.2	3.56	0	0	0	6.09	0	0.03	0
107995	'Cdc20'	0.02	0	0	0	3.97	0	0	0	0	0
107999	'Gtpbp6'	17.54	9.88	16.1	24.3	23	24.05	11	10.28	15.95	11.19
108000	'Cenpf'	0.02	0.64	0.04	0.1	0.03	0.08	1.05	0.19	0.12	0.05
108011	'Ap4e1'	0.62	5	1.63	0.11	3.56	3.12	1.33	1.63	4.23	2.14
108012	'Ap1s2'	13.14	46.54	38.4	48	78	77.77	168	115.1	114.7	182.77
108013	'Celf4'	272.2	262.8	171	229	233	311.9	222	233.8	216.3	287.68
108014	'Srsf9'	11.47	7.63	5.69	24.7	9.43	3.8	3.98	9.18	5.28	7.96
108015	'Chrnb4'	6.62	1.28	0.03	0.05	21.1	0.01	0.03	0	0	3.21
108017	'Fxyd4'	0	0.38	0	0	2.41	0	0.03	1.71	0	0
108030	'Lin7a'	3.42	6.97	5.99	8.14	3.61	10.59	10.6	7.87	5.16	6.26
108037	'Shmt2'	11.14	10.19	5.35	31.6	8.31	15.02	9.75	3.57	12.26	17.22
108043	'Chrnb3'	1.29	0	0	0	0	0	0	0	0	0
108052	'Slc14a1'	3.18	6.62	3.94	0	0.38	0.19	6.16	2.08	3.43	0.93
108058	'Camk2d'	40.28	21.67	31.5	13.3	36.7	27.43	32.1	27.26	25.36	34.39
108062	'Cstf2'	22.9	24.23	26	8.62	15.2	11.3	19.7	22.03	21.77	34.2
108067	'Eif2b3'	20.21	20.6	29.9	68.9	43.9	51.92	28.7	49.8	29.85	47.84
108068	'Grm2'	1.95	0.89	2.06	0	10.8	7.97	6.14	1.88	3.54	8.18
108069	'Grm3'	2.24	5.38	1.33	0.48	0.63	0.13	0	2.15	0.74	1.16
108071	'Grm5'	15.66	5.23	7.53	8.02	10.5	6.91	24.1	5.38	8.11	4.58
108073	'Grm7'	21.06	10.09	18.7	45	12.7	7.23	47.7	17.67	30.33	26.33
108075	'Ltbp4'	3.12	6.39	2.74	5.84	3.46	3.69	3.69	4.51	1.86	8.45
108077	'Skiv2l'	19.43	21.51	25	5.73	25.2	12.72	15.9	8.97	11.37	21.01
108079	'Prkaa2'	3.43	1.93	0.78	2.79	3.96	0.93	0.12	1.62	1.12	3.02
108083	'Pip4k2b'	2.95	3.25	2.26	1.59	1.46	0.67	1.14	2.12	0.45	1.62
108086	'Rnf216'	13.57	10.64	10.6	3.54	10	5.3	6.59	14.23	8.92	6.11
108089	'Rnf144a'	0.95	0.68	0.94	0.36	0.98	0	3	0.98	2.58	0.01
108097	'Prkab2'	7.61	2.11	2.46	1.8	2.11	0.05	0	2.64	0	5.84
108098	'Med21'	91.07	105.5	79.9	137	97.5	82.33	82.6	108.4	104.5	72.35
108099	'Prkag2'	23.85	33.98	19.4	7.24	28.8	11.38	17.8	26.28	19.08	30.36
108100	'Baiap2'	29.28	76.06	34.9	60.4	41.6	16.81	33.7	15.38	16.45	15.28
108101	'Fermt3'	1.38	0	0	2.52	0	3.86	0	2.63	0.1	0
108105	'B3gnt5'	0	0.15	1.58	0	0	0	0	0.22	0	0
108112	'Eif4ebp3'	0	0.69	2.19	0	1.23	1.76	4.69	1.9	0.15	0.95
108114	'Slc22a7'	0	0.02	0	0	0	0.02	0.2	0	0	0.09
108115	'Slco4a1'	1.98	0	4.04	1.09	1.99	0	4.1	0.1	5.04	8.62
108116	'Slco3a1'	5.06	5.83	1.79	7.81	4.16	4.49	20.7	5.06	2.33	3.63
108121	'U2af1'	97.71	82.15	125	93	80.7	138.7	116	130	162.6	98
108123	'Napg'	49.97	60.05	45.5	46.1	43.4	19.65	62.4	54.94	46.48	39.67
108124	'Napa'	233.1	283.7	211	241	292	310.5	241	316	271.6	262.24
108138	'Xrcc4'	4.94	10.68	5.25	1.45	16.8	10.91	12.2	4.07	20.76	6.7
108143	'Taf9'	147.1	167.5	147	264	138	163.7	136	156.3	166.2	120.46
108147	'Atic'	12.88	11.87	9.13	5.38	2.14	16.33	4.48	11.59	17.51	12.58
108148	'Galnt2'	8.91	7.66	5.59	9.62	6	8.04	8.4	11.82	7.45	11.16
108150	'Galnt7'	5.14	5.23	0.47	0.01	0.38	0	0.14	1.9	2.9	2.29
108151	'Sema3d'	1.29	1.72	1.08	0	0.01	1.82	1.6	0.46	1.51	0.19

108153	'Adamts7'	0	0.08	0	0	0	0	0	0.08	0	0
108154	'Adamts6'	0	0.06	7.07	7.3	1.39	0.01	0	0	0.57	0.01
108155	'Ogt'	51.55	46.8	50.3	40.4	43	34.36	56.4	47.31	48.72	59.48
108156	'Mthfd1'	10.07	20.72	35.6	23.9	16.9	8.86	31.8	24.52	20.98	19.93
108159	'Ubxn8'	4.89	10.58	6.74	18.5	11.1	14.67	28.4	12.56	8.01	16.25
108160	'Fam50a'	32.93	30.9	34.6	21.2	40.7	25.26	32.6	44.24	33.04	16.47
108167320	'LOC108167320'	15.92	9.59	10.8	13.5	11	12.42	8.7	6.52	6.87	16.29
108167321	'LOC108167321'	1.76	1.02	2.83	0.56	1.36	0.36	2.56	0.93	3.78	0.51
108167339	'LOC108167339'	0.02	0.11	0.44	0	0.26	0.02	0.73	0.09	0.02	0.41
108167344	'Gm45951'	6.82	7.17	7.72	2.16	5.08	6.71	5.48	3.66	4.97	2.58
108167358	'LOC108167358'	0.03	0.04	0.02	0.31	0.08	0.01	0.02	0.06	0.36	0.06
108167362	'Gm45961'	0.19	0.31	1.27	1.94	0.24	0.13	0.27	0.49	0.64	0.15
108167368	'LOC108167368'	0.66	3.51	2.19	0.89	0.83	0.35	2.03	0.75	1.99	0.75
108167370	'Gm45965'	0	0	0	0	0.04	0	0	0	0	0
108167412	'Gm45975'	0	0	0	0	0	0.02	0	0	0	0
108167434	'Gm45988'	0.79	1.15	0	0	0.23	1.35	0	0	2.02	1.15
108167466	'Gm45618'	0	0.08	0	0	0	0	0	0	0	0.34
108167476	'Gm46008'	0.12	0.11	0.29	0.02	0.11	0	0.01	0.1	0	0
108167482	'Gm46011'	0.1	0.04	0.06	0	0.02	0	0	0.02	0	0.12
108167549	'108167549'	0.33	0.12	0.67	0.34	0.85	1.64	0.29	0.29	0.65	0.08
108167560	'Gm46058'	0	0	2.8	2.83	0	0	0	0	0	0
108167571	'Gm43263'	1.56	1.14	0.56	3.87	2.56	1.04	1.13	0.64	0.44	0
108167576	'Gm46066'	1.16	0.59	0.44	3.77	0.66	1.03	0.46	1.47	0.1	0.72
108167614	'Gm46084'	0	0	0.54	0	0	0	0.67	0	0.03	0
108167694	'LOC108167694'	1.24	1.54	1.95	2.83	1.05	1.69	1.39	0.86	0.46	1.2
108167700	'Gm46139'	0.17	0.02	0.05	0	0.98	0.92	0.61	1.07	0	0.19
108167735	'Gm46164'	0	0.72	0	0	0	0	0	0	0	0
108167752	'Gm46175'	0	0	2.37	0	0.06	0.01	0	0	0	0
108167806	'Gm46221'	0.37	0.36	0.15	0.08	0.53	1.37	0	0.26	0.23	0
108167809	'Gm46223'	0	0.23	0	0	0	0	0.41	0	0	0.9
108167814	'LOC108167814'	0	0	0	0	0	0.02	0	0	0.03	0
108167830	'Gm46240'	0.16	0	0.13	0.21	0.14	0	0.05	0	0	0.08
108167848	'Gm12258'	7.15	3.7	2.62	7.35	2.82	2.39	0.01	4.17	1.18	6.93
108167855	'Gm46255'	0.66	0.33	0.79	0.02	0.03	0	0	0.58	0.3	0
108167858	'Gm46257'	0	0.89	0.22	0	0	5.11	2.12	0.28	1.9	0.63
108167883	'Gm46270'	0.29	0.48	3.12	0.25	0.49	0	0.22	0.52	0.12	1.42
108167886	'Gm46272'	0	0.05	0	0	0	3.06	0	0	0	0
108167918	'Gm46290'	0	0	0	3.45	0.35	0	0	0	2.15	0.12
108167922	'LOC108167922'	0.09	0.16	0.15	0.12	0	0.04	0.12	0.01	0.23	0.07
108167925	'Gm46294'	0	0.51	0	1.7	0.99	1.69	0	0.83	0	0.27
108167946	'Gm46306'	0	0.07	0.06	0.49	0.19	0	0.69	0.1	0.05	0
108167951	'Gm46310'	0.28	0.1	0.85	0.15	0.51	0	0	0	0.14	0
108167961	'Gm46319'	0	0.13	0	0	0	0	0	0	0	0.06
108167963	'Gm46320'	0	0	0	0	0	0.07	0.07	0	0	0
108167986	'Gm46339'	0.43	1.5	1.37	0.81	0.69	0.19	0.44	0.35	1.16	0.27
108167989	'Gm46340'	0.11	0.16	0.87	0.29	0.08	0.02	0.36	0.81	0.28	0.19
108167995	'Gm46345'	0	0	0	0	0	0.37	0	0	0.25	0
108168003	'Gm46353'	0	0	0.4	0	0	0.71	0	0.49	0	0
108168034	'Gm46382'	0	0	0	0.28	0	0	0	0	0	0
108168049	'Gm46390'	0.77	2.45	3.25	0	5.76	0.42	0.12	1.54	2.23	1.26
108168067	'Gm46403'	1.52	1.95	1.71	8.3	1.86	3.97	2.74	1.16	3.69	3.49
108168097	'Gm46426'	0	0.18	0.06	0.07	0.13	0	0	0.1	0	0
108168098	'Gm46427'	0	0	0	0	0	0	0	0.03	0	0
108168101	'Gm46430'	10.15	8.24	5.34	11.1	9.49	0.44	8.6	8.17	2.93	6.86
108168108	'LOC108168108'	0	0	0	0	0	0	0.02	0	0.03	0.7
108168152	'Gm8126'	0	0	0	0	0.05	0	0	0	0	0
108168162	'Gm43305'	1.4	4.29	10.3	10.6	18.6	12	14.7	12.96	7.74	2.97
108168219	'Gm46500'	0.28	0.27	0.37	0	0.45	0.01	1.27	0	0.4	0.03

108168225	'Gm26513'	0	0	0	1.5	0	0	0	0	0	0
108168226	'Gm46504'	0.13	0.14	0.77	0	0	1.2	0	0	0.25	0.19
108168313	'LOC108168313'	0	0	0.16	0.55	0.24	0	0.08	0.13	0	0
108168323	'Gm46580'	0.37	0.33	0.03	0.24	1.23	0	0	0	1.02	0.42
108168336	'Gm46592'	0	0	0	0	0	0	0	0	0.04	0.01
108168358	'Gm46608'	0	0	0.84	0.87	1.11	2.52	0.54	0.86	0.56	0
108168367	'LOC108168367'	0	0	0.99	0	0.46	4.08	0	0	0.12	0
108168395	'Gm45871'	1.99	1.71	2.88	0.42	2.91	0.92	3.47	6.71	6.51	4.79
108168405	'LOC108168405'	0.32	0.04	0.07	0.46	0.3	0.56	0.04	0.36	0.12	0.26
108168534	'Gm46714'	0	0.69	1.02	1.88	0.03	0	0	0.14	0.89	0
108168543	'LOC108168543'	0	0	0.12	0	0	0	0	0	0	0
108168553	'LOC108168553'	0	0	0	0	0	0	0	0.23	0	0
108168572	'Gm46715'	0	0	0	0	0	0	0	0.04	0.02	0
108168573	'Gm46716'	0	0	0	0	0	0	0	0.04	0.02	0
108168574	'LOC108168574'	0	0	0	0	0	0	0	0	0.31	0
108168644	'Gm46717'	0.16	0.2	0.22	0.18	0.24	0	0.04	0.02	0.05	0
108168679	'LOC108168679'	6.61	12.99	13	8.94	0.07	16.61	7.15	4.55	2.92	10.34
108168680	'LOC108168680'	0.01	0	0	0.12	0.01	0.01	0	0	0.02	0
108168683	'LOC108168683'	0	0	0	0	0	0.06	0	0	0	0
108168685	'LOC108168685'	0	0	0	0	0	0.06	0	0	0	0
108168691	'LOC108168691'	0	0	0.55	2.11	0.71	1.08	0	0.45	0.55	0
108168724	'LOC108168724'	0	0	0.08	0	0	0	0.43	0.21	0.45	0.6
108168740	'Gm46731'	2.29	0	0.04	0	0	0	0	0	2.81	0
108168771	'LOC108168771'	1.46	1.2	2.71	3.09	1.41	1.61	1.25	0.96	1.87	2.21
108168884	'Gm37500'	0.03	1.61	0.99	0.55	4.39	0	2.82	0.24	0.54	2.14
108168906	'Gm43707'	0.06	0.01	0.52	0.01	0.01	0.33	0	0	0.27	0
108168936	'LOC108168936'	0	0	0	0	0.15	0.63	0	0.01	0	0
108168952	'108168952'	0.01	0	0	0.12	0.01	0.01	0	0	0.02	0
108168953	'Gm46858'	0	0	0	0	0	0.27	0	0	0	0
108168959	'LOC108168959'	0.13	0.05	0	0	0	0	0	0	0	0.06
108168960	'Gm45533'	0	0	0	0	0	0	0	1.09	0	0
108168962	'LOC108168962'	0	0.26	0.24	0.34	0.29	0	0	0.02	0	0
108169010	'LOC108169010'	0.7	0	0	0.05	0	0.75	0	1.23	0	0
108169023	'Gm46901'	0	1.2	2.29	4.41	5.07	1.03	3.27	2.51	0.4	0.86
108169043	'Gm46911'	3.61	4.26	2.79	24.4	6.56	1.8	0.58	3.59	15.66	6.05
108169060	'LOC108169060'	0.34	0.05	0.07	0.08	0	0.06	0.22	0.06	0.07	0.07
108169061	'Gm46918'	3.62	2.68	1.92	0.19	4.63	11.37	0	0.69	1.21	1.26
108169096	'Gm46933'	0	0.12	2.28	5.38	4.76	3.35	0.46	0.96	1.65	1.91
108169098	'Gm46934'	0	0	0	0	0	0.06	0	0	0	0
108169100	'LOC108169100'	0	0	0	0	0	0.06	0	0	0	0
108169150	'Gm46963'	0	0.09	0.26	0	0	0	0	0	0	0
108169152	'Gm46965'	0	0	2.12	0	0	0	0	0	0	0.52
108169171	'Gm46977'	0	0	0	0	0	0	0	0	0.5	0
108169182	'Gm46982'	0.46	0.12	0	0	0	0.3	0.65	0	0.45	0
108169201	'Gm46988'	0	0	0.02	0	0	0.49	0	0	0	0
108645	'Mat2b'	107.8	73.24	74.8	84.6	102	102.2	145	94.34	112	64.19
108652	'Slc35b3'	5.46	7.91	11.2	12.4	8.4	15.03	1.54	14.3	6.21	11.27
108653	'Rimklb'	18.49	7.14	10.3	10.8	5.85	5.45	18.8	11.75	12.43	7.54
108654	'Fam210a'	1.69	5.14	3.26	0.41	1.02	2.21	3.06	2.86	4.44	1.73
108655	'Foxp1'	3.83	4.5	5.66	6.22	7.28	4.91	11	6.94	8.83	5.18
108657	'Rnpepl1'	0	6.95	5.42	3.34	2.94	1.84	3.98	1.49	0.81	1.54
108660	'Rnf187'	158.2	72.56	149	233	100	35.44	107	24.73	101.1	23.14
108664	'Atp6v1h'	178.8	228.6	215	169	166	202	197	234.4	206.5	209.23
108670	'Epsti1'	4.34	1.94	0	0	0	0	3.09	0.39	0	0
108671	'Dnajc9'	46.62	36.14	56.9	48.1	49.4	80.71	34.9	25.68	70.42	49.82
108672	'Zdhhc15'	0.02	0.9	3.4	0	0.8	0	1.71	0.78	1.44	1.72
108673	'Ccdc86'	9.96	9.57	4.41	4.43	13.9	15.98	12.8	13.92	9.52	12.26
108679	'Cops8'	122.2	131.1	131	84.2	145	218.7	121	140	140.7	110.88

108682	'Gpt2'	9.24	8.41	9.66	9.81	4.81	6.04	23.2	5.03	8.32	0.63
108686	'Ccdc88a'	3.24	4.06	3.34	5.2	5.72	4.06	4.38	3.58	1.99	6.95
108687	'Edem2'	31.1	28.4	26.1	44.6	5.11	21.3	10.4	22.77	28.42	30.02
108689	'Stn1'	5.33	4.51	11.8	15.2	17	8.3	15.2	15.67	9.18	11.91
108699	'Chn1'	87.77	120.7	80	79.6	64.7	80.37	64.9	104.5	41.29	34.95
108705	'Pttg1ip'	50.07	44.49	51.4	13.7	56	32.53	22.2	20.22	53.72	38.86
108707	'Fam207a'	10.7	15.33	25.6	12.6	8.1	19.28	15.5	7.94	32.45	4.96
108723	'Card11'	0	0	0	3.52	0	0	0	0	0	0
108735	'Sft2d2'	0.11	3.24	0.95	0	4.77	1.53	0.01	0.09	0.2	0.54
108737	'Oxsr1'	2.07	0.87	2.74	7.41	4.07	4.64	4.03	3.97	5.21	4.07
108755	'Lyrm2'	32.2	26.12	25.7	53.8	32.6	36.2	31.4	38.67	38.88	31.69
108760	'Galnt16'	92.29	109.6	78.3	53.5	69.5	55.84	91.2	70.69	78.89	180.97
108767	'Pnrc1'	13.47	14.07	6.92	18.5	6.98	13.49	3.44	11.53	9.94	16.99
108797	'Mex3b'	0.61	0.69	0	1.71	0.54	0	5.02	0.08	0	1.01
108800	'Ston2'	1.36	0.84	0.11	0.29	0.52	0.67	0.37	0.07	1.06	0.15
108802	'Calr4'	2.24	0	0	0	0	0	0	0	0	0
108811	'Ccdc122'	0.04	0	0	0	1.08	0.56	0	0	0	0
108829	'Jmjd1c'	7.07	10.38	5.54	30.1	3.22	6.15	5.42	2.39	2.99	4.41
108832	'Tmem74b'	6.9	12.58	11	10.6	7.46	5.42	14.3	17.51	11.44	2.84
108837	'Ibtk'	1.78	1.69	3.15	2.38	1.33	4.46	4.4	2.85	3.2	1.79
108841	'Rdh13'	21.73	21.89	10.8	28.6	14.7	25.29	10.2	27.16	35.52	20.72
108853	'Mtrf1l'	4.02	3.97	6.49	5.83	5.79	2.2	0.68	1.54	3.73	0
108857	'Ankhd1'	2.17	1.83	2.84	4.56	2.64	1.58	3.84	3.13	2.99	2.19
108888	'Atad3a'	20.76	18.79	39.6	36.2	26.9	19.59	17.8	11.93	13.13	1.96
108897	'Aif1l'	1.08	0.98	3.66	0	0	0	0	0	0	0
108899	'2700081O15Rik'	3.08	1.39	1.9	0	3.87	0	3.15	1.85	10.46	0.82
108900	'Fam72a'	0.07	0	2.24	0	0	0	0.11	0.54	0	0
108902	'B4gat1'	159.7	156.2	176	132	144	203.1	135	169.4	156.2	145.86
108903	'Tbcd'	17.18	8.42	15.4	7.21	25.1	9.03	21	8.97	15.22	11.82
108907	'Nusap1'	0.02	1.71	0	0.06	0	0.51	0	0	0	0.04
108909	'Aida'	5.45	7.6	14.5	22.7	8.61	6.21	5.41	8.33	9.73	5.05
108911	'Rcc2'	11.9	13.52	4.35	10.2	11.2	6.9	4.65	4.32	9.85	9.94
108912	'Cdca2'	0	0	0	0	0	0	0	1.26	0	0
108927	'Lhfp'	7.53	18.17	4.24	1.25	18.7	11.19	7.02	8.15	10.37	13.28
108934	'Smim13'	3.39	4.22	3.54	3.66	8.12	2.56	8.27	4.79	11.04	4.57
108937	'Rnf169'	2.11	1.08	0.7	2.47	1.02	0.51	0.01	3.48	0.57	0.23
108943	'Trmt10a'	9.62	4.53	8.66	2.5	2.36	24.98	6.06	11.52	10.39	10.4
108946	'Zzz3'	3.66	0.77	3.23	0.21	3.6	0.92	0.3	2.84	5.42	7.48
108954	'Ppp1r15b'	1.31	7.18	6	6.73	2.77	0.58	2.49	3.97	6.33	4.6
108958	'Miga2'	15.56	25.24	36.7	3.91	39	8.11	30.6	20.83	24.43	28.69
108960	'Irak2'	10.62	10.84	4.71	8.47	15.5	8.42	5.01	7.25	8.66	4.22
108978	'4930555G01Rik'	0.92	1.15	1.67	0.84	0.47	0.4	1.06	0.56	0.41	0.97
108989	'Tpr'	7.14	8.91	8.49	17.3	10.4	13.37	8.36	8.42	10.52	4.49
108995	'Tbc1d10c'	0	0	0	5.73	0	0	0	0	6.26	0
109006	'Ciapin1'	85.69	38.62	74.6	67.5	58.4	76.35	61.6	63.89	61.86	77.51
109019	'Nabp1'	7.7	12.02	5.6	2.05	5.16	0.04	16.3	1.19	0.58	4.73
109032	'Sp110'	0	0.05	0.03	0	0.06	0.03	0	0.05	0.07	6.62
109042	'Cavin3'	27.45	21.68	24.1	7.26	14.1	29.84	18.5	9.56	10.86	20.52
109050	'Inka2'	0.01	0	4.42	0.38	1.85	5.68	1.57	0	2.51	7.46
109054	'Pfdn4'	37.66	59.17	39.2	44.5	36.6	42.68	38.3	39.65	45.29	35.77
109065	'Dnaaf2'	3.56	7.4	10.7	29.5	2.24	6.13	5.41	7.31	3.51	6.11
109075	'Exosc4'	70.11	53.38	44	34.8	55.8	58.15	32.7	48.97	51.42	61.41
109077	'Ints5'	5.19	2.62	3.54	0.02	4.39	3.94	3.64	8.63	0.89	3.42
109079	'Sephs1'	0.25	2.29	3.77	3.65	1.52	5.2	6.14	1.47	4.11	3.95
109082	'Fbxw17'	29.34	26.85	8.81	0.17	11.1	28.04	8.63	9.29	17.89	35.29
109093	'Rars2'	23.14	24.55	21.8	49	23.7	21.83	29.8	48.58	37.76	16.94
109095	'Rbm15b'	2.62	4.17	2.42	8.23	0.11	0.3	4.8	5.47	1.49	1.29
109108	'Slc30a9'	31.83	28.28	31.9	51.9	25.1	18.45	32	20.41	50.76	6.38

109113	'Uhrf2'	8.05	7.94	5.07	0.98	4.34	6.07	4.36	2.74	5.34	5
109115	'Supt3'	6.24	7.97	9.05	5.25	14	4.46	13.6	3.52	7.92	9.65
109129	'Mmadhc'	48.25	78.27	63.4	56.3	70.6	41.8	69.7	48.86	59.64	62.14
109135	'Plekha5'	5.98	5.79	6.16	3.42	10.8	9.8	9.34	9.27	7.32	6.7
109136	'Mmaa'	1.98	14.69	6.57	6.55	10.6	11.53	4.75	7.25	5.76	15.01
109145	'Gins4'	36.6	34.89	2.3	9.58	33.7	44.82	33.4	34.18	32.34	15.09
109151	'Chd9'	4.24	5.25	4.55	3.34	7.7	4.04	3.92	3.85	4.9	3.59
109154	'Mlec'	12.22	8.69	8.38	14.1	10.5	4.94	8.75	7.23	16.78	7.95
109161	'Ube2q2'	14.29	16.09	15.1	19.4	13.9	13.45	18.5	10.95	17.42	17.27
109168	'Atl3'	2.28	0	0.16	0.47	0.62	1.12	0.65	0.7	0.59	0
109169	'Igip'	12.11	4.22	9.26	2.86	7.48	22.73	18.3	1.24	8.75	3.46
109181	'Trip11'	5.64	8.52	8.84	12.9	14.5	14.14	8.89	7.19	11.84	10.59
109205	'Sobp'	4.01	0.67	2.5	7.08	2.05	1.95	1.47	0.58	0.93	3.88
109222	'Rarres1'	0	0	0.03	0	0	5.14	0	0	1.95	0
109229	'Fam118b'	19.93	25.66	15.5	14.5	24.2	6.62	5.26	23.03	15.02	5.8
109232	'Sccpdh'	252.4	220.6	167	309	126	203.6	114	208.6	127.8	120.41
109241	'Mbd5'	7.23	5.89	5.39	5.74	3.52	3.31	10.7	5.89	2.32	4.45
109242	'Kif24'	0.01	0.46	0.17	1.63	0.02	0.08	1.92	0.13	0.03	0.46
109245	'Lrrc39'	0.42	0.04	0.97	0.1	0.04	0.04	0.08	0.19	3.43	3.54
109246	'Tspan9'	5.75	3.27	1.37	0.02	6.21	5.41	0.01	2.8	0	0.02
109263	'Rlf'	0.93	2.26	2.41	5.72	1.85	1.69	1	0.91	4.29	2.14
109264	'Me3'	16.28	4.09	18	19.9	30.5	25.22	9.29	28.65	8.71	12.83
109267	'Ssc4d'	1.68	0	0.11	0	0.29	0	0	0.07	2.42	0.03
109270	'Prr5'	14.26	19.66	26.1	21.6	10.7	8.52	16.4	32.15	21.48	15.29
109275	'Actr5'	0.25	2.23	1.64	0.05	5.09	0	6.67	0	0	2.81
109284	'R3hdm4'	26.97	34.05	55.2	26.2	86.5	36.21	40.5	39.26	10.39	21.19
109294	'Prex2'	1.01	0.64	0.46	0	0	0.53	1.23	1.1	0.71	0.01
109299	'Tmem250-ps'	149.5	163.4	72.8	109	195	243.7	137	189	149.6	231.94
109305	'Orai1'	0.17	0	7.23	0	0	2.28	0	0	0	0
109331	'Rnf20'	6.84	11.19	8.27	4.15	7.45	9.47	10.7	8.4	9.71	7.09
109332	'Cdcp1'	0	0.1	1.63	0	0	0	0	0.22	0.05	3.39
109333	'Pkn2'	0.01	0.11	3.25	1.28	0.07	0.88	1.06	0.04	0.04	1.97
109346	'Ankrd39'	16.25	25.08	36.5	19	31.3	13.89	24	23.27	21.22	53.56
109349	'Fam163b'	19.86	10.3	9.98	6.66	16.2	2.45	8.05	26.43	5.83	9.23
109359	'Abraxas2'	24.88	16.52	11	7.14	6.12	10.68	4.42	16.73	12.57	19.93
109552	'Sri'	37.62	29.48	38.6	14.5	72.1	45.53	66.3	50.42	53.5	34.32
109575	'Tbx10'	0	0	0	0	0	1.94	0.05	0	0	0.02
109593	'Lmo3'	5.24	23.48	5.75	0.06	24.2	7.63	17.8	8.85	0.08	3.24
109594	'Lmo1'	54.19	33.17	4.84	0	0	12.47	14.3	9.62	8.17	18.89
109620	'Dsp'	0.02	0	0.06	0	0.01	0.03	0.03	0.01	0.02	0.01
109624	'Cald1'	1.28	1.09	2.31	3.88	1.26	3.76	3.75	0.77	4.97	6.19
109637	'Upk1a'	0	0.15	0.04	0	2.69	0	0	0	2.51	0
109648	'Npy'	3.78	0	0	0	0	0	0	6.54	0	0
109652	'Acy1'	4.47	15.31	1.79	5.54	3.5	7.51	0	13.13	9.95	6.22
109658	'Txlna'	6.23	17.74	5.77	14.6	5.57	6.06	4.07	12.62	8.86	9.13
109660	'Ctrl'	1.88	0	0.11	8.42	2.03	0	0	5.58	0	2.47
109672	'Cyb5a'	130	160.8	85.5	170	111	183.7	192	103.2	117.8	166.02
109674	'Ampd2'	54.66	52.89	55.7	30.1	40.7	48.09	54.6	60.34	116.9	117.99
109676	'Ank2'	10.61	7.13	19.7	11.5	12.9	14.68	17.9	9.32	9.5	11.58
109685	'Hyal3'	1.43	5.68	3.02	0.45	0.09	0.16	0.21	2.35	8.22	0.26
109689	'Arrb1'	6.57	3.53	2.94	1.13	2.03	4.08	13.3	5.59	8.09	3.15
109711	'Actn1'	0.74	0.67	1.58	0.62	0.58	0.35	1.55	0.16	0.97	0.59
109731	'Maob'	9.53	10.55	17.7	11.1	2.99	32.43	11.4	47.76	26.82	6.21
109754	'Cyb5r3'	113.9	116.8	143	98.4	170	125.4	84.4	108.5	137.4	151.03
109778	'Blvra'	30.71	31.51	39.4	43.9	22.8	30.63	32	40.31	48.57	62.04
109785	'Pgm3'	20.15	17.76	23.3	29.6	3.17	20.68	19.6	29.11	31.9	29.67
109801	'Glo1'	127.9	133.6	65	49.1	111	130.3	75.9	80.19	101.4	119.42
109815	'Selenos'	63.5	54.01	52.7	157	40.5	48.1	81.7	89.7	86.73	72.85

109828	'C7'	0.02	0	0.37	0	0	0.02	0	0	0	0.42
109857	'Cbr3'	0.08	3.72	9.68	0	3.37	8.54	8.93	0	0	0
109880	'Braf'	4.75	6.21	5.69	5.78	6.95	5.37	4.61	4.76	2.55	2.11
109889	'Mzf1'	2.71	4.48	3.1	0.02	7.27	11.12	8.27	7.77	6.17	6.71
109900	'Asl'	25.06	51.79	51.5	67.4	42	66.1	43.8	38.04	123.2	45.69
109901	'Cela1'	1.64	3.13	0	0	5.81	0	0	0	0	0
109904	'Mcf2'	0.07	0	0	1.24	2.73	0.33	3.26	1.35	1.49	0
109905	'Rap1a'	8.2	12.4	11.7	10.2	11.7	18.94	9.07	7.95	5.81	4.38
109910	'Zfp91'	3.98	3.07	3.03	7.93	4.11	2.39	2.49	1.94	1.59	1.06
109929	'Zbtb25'	6.82	5.68	6.33	9.89	6.11	3.24	11.3	12.66	6.48	6.1
109934	'Abr'	39.89	57.31	61.8	40.6	25.1	12.53	50.3	32.28	26.57	32.78
109959	'Amy2a5'	0	0	1.38	0	0	0	0	0	0	0
109978	'Art4'	0	0	0.02	0	0	0	0	0	0	0
109979	'Art3'	0.16	0.04	0.26	0.09	0	0.07	0.19	0.03	0.14	0.1
110006	'Gusb'	13.41	11.79	9.18	19.2	15.1	33.91	24.9	12.26	27.41	23.54
110012	'Tpgs1'	128.3	115.9	129	161	144	147.2	168	179.2	154.5	169.83
110033	'Kif22'	1.64	0.84	0.21	1.96	4.54	2.41	1.31	0.56	0.08	0.02
110052	'Dek'	15.15	14.29	20.4	27.7	16.7	6.96	34	12.63	20.24	7.55
110058	'Syt17'	15.63	2.09	27.8	10.9	23.8	19.69	7.76	9.59	5.18	6.63
110074	'Dut'	22.98	24.73	24.5	17.5	17.9	27.91	26.6	23.32	22.13	25.36
110075	'Bmp3'	0	2.95	5.38	5.31	0.01	0	7.66	0.26	0	0
110078	'Pygb'	68.45	53.53	61.2	23.6	43.3	29.45	73.2	58.82	91.91	54.81
110082	'Dnah5'	0	0.39	0.69	0.34	0	0	0	0.15	0	0.4
110083	'Dnah12'	0.64	0.94	0.69	0.18	0.07	0.01	0.03	0.83	0.03	0.17
110084	'Dnah1'	6.65	13.76	9.07	5.83	16.6	3.72	10.1	6.51	7.15	15.56
110094	'Phka2'	1.94	2.78	6.34	8.53	1.57	9.05	4.11	3.76	2.61	5.48
110095	'Pygl'	2.69	2.62	2.28	0	0	1.01	0	5.52	0	3.85
110109	'Nop2'	23.7	24.97	20	17.3	17.2	23.58	18.7	20.81	18.89	18.27
110119	'Mpi'	78.61	22.73	42.9	110	69.9	93.7	66.7	78.12	53.53	42.12
110147	'Ehmt2'	8.05	13.31	9.88	9.23	18.4	8.8	5.6	4.08	3.54	3.79
110157	'Raf1'	11.27	5.38	25.2	29.4	7.48	4.27	18.5	15	5.59	4.69
110168	'Gpr18'	0	0.86	0	0	0	0	0	0	0	0
110172	'Slc35b1'	76.26	74.49	75.7	61	85.9	109.2	96.5	103.9	72.64	71.87
110173	'Manba'	7.38	0.41	6.9	0.92	0.99	2.27	4.39	3.47	8	20.49
110175	'Ggct'	15.02	23.72	18.6	13.3	16	20.93	13.9	8.07	19.46	8.17
110196	'Fdps'	204.1	142.2	201	326	201	258.9	192	197	157.5	191
110197	'Dgkg'	27.7	29.38	27.2	13.8	13.2	5.37	27.4	31.7	23.17	29
110198	'Akr7a5'	38.4	44.96	44.3	4.86	27.4	63.84	54.6	29.29	40.01	45.82
110208	'Pgd'	24.56	49.4	38.4	39.6	12.3	54.7	47.2	31.53	19.88	26.68
110213	'Tmbim6'	265.5	273.4	285	253	270	412.1	294	301.3	308.7	325.51
110253	'Triobp'	0.93	0.92	0	0	0.04	0	0	2.39	0.05	0.05
110257	'Hba-a2'	19.05	0	23.1	45.4	9.34	14.66	0	14.75	21.29	3.57
110265	'Msra'	21.75	3.38	24.9	9.07	5.03	19.09	35.4	52.15	11.84	10.19
110279	'Bcr'	4.44	1.76	2.65	12.5	5.85	3.01	2.31	2.81	2.35	7.92
110304	'Glra3'	7.25	19.66	1.42	0.04	1.32	2.91	1.83	6.06	5.71	4.57
110323	'Cox6b1'	1219	1097	1389	1389	1165	1729	1334	956.6	1563	1216.78
110326	'Tas1r1'	1.03	3.6	1.37	0	2.75	0	0.02	0.87	0.7	0.02
110332	'Pp2d1'	0.67	0.02	0	0	0.09	0	0.34	1.79	1.15	1.02
110350	'Dync2h1'	10.22	8.34	4.26	10.1	8.48	4.61	4.8	7.63	8.08	18.91
110351	'Rap1gap'	53.05	34.56	77	46.2	37.5	35.35	73.3	42.69	82.45	31.03
110355	'Grk2'	10.23	10.68	7.19	5.51	19.8	19.38	7.12	8.95	19.44	9.13
110379	'Sec13'	92.67	98.6	112	97.3	98	135	119	95.16	100.1	139.74
110380	'Shroom2'	9.59	15	13.6	9.5	10.5	2.55	20.6	18.11	8.04	8.05
110385	'Pde4c'	0.1	0.07	0.17	0.15	0.03	0.11	0.18	0.08	0	0.07
110391	'Qdpr'	282.7	233	207	212	391	498.1	171	222.7	262.8	276.63
110417	'Pigh'	5.18	15.54	3.26	6.76	14.5	3.11	15.3	24.53	10.6	11.99
110446	'Acat1'	66.48	49.45	61	46.8	43.9	65.34	98.4	60.93	71.03	56.06
110454	'Ly6a'	0	0	0	0	0	5.54	0	0	0	0

110460	'Acat2'	82.28	66.31	71.5	85.2	72.3	58.77	86.1	79.65	61.28	51.44
110521	'Hivep1'	0.56	0.75	4.42	0.32	0.31	0.66	0.5	0.77	0.02	0.6
110524	'Dgkq'	12.91	7.4	14.9	18.8	6.43	10.88	14.8	10.81	19.66	3.89
110532	'Adarb1'	6.82	0.7	2.95	3.86	6.41	2.81	6.4	3.99	3.45	3.2
110542	'Amhr2'	0	0.32	0	0.03	0.08	5.3	0	0	0	0.73
110557	'H2-Q6'	0	0	0	0	0	0.14	0	0	0	0
110558	'H2-Q9'	0	0.41	0.03	0.6	0.82	1.03	0	0.43	1.55	0
110593	'Prdm2'	5.4	3.32	7.33	7.01	3.8	4.73	3.02	2.87	4.4	5.91
110595	'Timp4'	37.99	23.24	21.5	3.38	5.31	19.48	31.8	12.93	10.98	12.06
110596	'Arhgef28'	5.39	6.55	5.44	2.37	6.48	6.11	1.41	5.68	4.07	4.44
110606	'Fntb'	24.74	45.45	21.8	21.2	22.1	14.07	12.4	34.86	23.7	26.02
110611	'Hdlbp'	12.93	11.6	18.7	12.4	11.1	1.69	18.5	9.02	5.66	14.83
110616	'Atxn3'	0.14	3.53	3	3.86	6.46	0	3.79	1.28	2.23	4.2
110637	'Grik4'	1.13	6.24	1.07	2.97	7.76	0.64	15.5	2.92	15.41	7.62
110639	'Prps2'	2.54	8.75	17	5.11	7.31	1.12	7.36	4.55	11.36	4.56
110648	'Lmx1a'	0	0	0	0.51	0	0	0	0	0	0.45
110651	'Rps6ka3'	0.01	0.13	0.01	0.62	0.05	0	1.56	0	0.73	0
110695	'Aldh7a1'	29.01	19.66	13.1	5.97	13.8	38.72	21	9.8	17.95	28.56
110749	'Chaf1b'	0	0.63	0.04	0	0	0	0	0	0	0
110750	'Cse1l'	31.7	17.56	26.4	11.1	13.7	10.1	30.3	32.08	16.27	37.79
110751	'Adam33'	0.02	8.26	0	0	0	0	0	0	0.06	3
110784	'Nr3c2'	3.42	1.35	4.57	5.58	0.24	1.97	1.15	1.39	1.39	1.39
110789	'Adgrv1'	0.16	0.18	0	0.01	0.42	0	0	0.01	0	0.03
110796	'Tshz1'	0.04	1	0.09	1.5	1.38	0.17	0.51	0	0.01	1.96
110809	'Srsf1'	7.24	9.43	10.5	14.3	7.88	5.71	9.87	6.98	9.24	7.25
110816	'Pwp2'	5.26	8.37	13.8	15.3	1.95	4.05	6.28	5.23	3.96	10.64
110821	'Pcca'	10.81	13.13	8.65	15.4	19.8	13.7	2.32	15.85	0.78	20.98
110826	'Etfb'	139.2	120.5	134	157	163	210.8	74	147.8	140.5	134.23
110829	'Lims1'	10.19	3.77	7.81	4.24	5.98	7.35	4.52	5.09	6.87	2.81
110834	'Chrna3'	22.84	4.6	18.7	5.12	0.14	0	2.19	1.47	0	6.34
110842	'Etfa'	51.54	42.88	63.7	53.4	60.8	97.03	36	61.32	89.64	51.6
110854	'Ptpa'	132.2	126.4	135	78.1	88.9	113.9	156	130.6	141.5	122.3
110862	'Kcnq3'	1.27	3.55	0.09	0.24	2.54	1.46	0.83	0.31	0.11	2.88
110876	'Scn2a'	14.8	13.77	24.2	17.7	18.3	12.38	27.5	15.91	12.85	23.12
110877	'Slc18a1'	0	0.56	0.97	0	2.67	1.42	0	0	1.46	5.96
110880	'Scn4a'	0.01	0	0	0	0	0.01	0	0	0	0
110886	'Gabra5'	16.21	36.77	32.9	54.2	20.2	10.55	28.9	86.78	29.47	15.06
110891	'Slc8a2'	0.33	0.42	0.6	0.83	0.03	0.26	0.21	1.11	0.04	0
110893	'Slc8a3'	7.03	2.11	9.15	12.6	5.07	9.65	10.7	4.32	2.88	1.3
110902	'Chrna2'	0	0	0	0	0	0	0	0	0.02	0
110911	'Cds2'	15.76	15.89	21.6	23.1	20.5	17.21	26	15.09	17.49	15.03
110920	'Hspa13'	39.21	41.88	30.5	31.8	6.42	15.24	23.7	21.31	26.39	28.65
110935	'Atp6v1b1'	0.44	0	0	0	0	0	0	0	0	0
110948	'Hlcs'	2.4	5.4	4.46	3.61	0.21	3.13	4.67	0.81	2.29	8.45
110954	'Rpl10'	301.4	347.4	298	369	266	287	299	276.1	162.7	325.36
110956	'D17H6S56E-5'	0.25	0.27	2.39	0.73	0.26	0.19	3.54	2.41	0.2	0.52
110957	'D1Pas1'	0	0	0.03	0	0	0.07	0	0.01	0	0
110959	'Nudt19'	50.33	64.56	72.3	68	47.9	73.29	49.6	57.52	65.64	25.51
110960	'Tars'	19.16	32.31	16	36.6	11.5	21.28	14.1	17.73	16.61	38.96
110962	'Mbd6'	1.96	2.69	0.71	1.16	1.86	3.44	0.02	0.24	5.15	0.98
111173	'Erc1'	7.88	9.27	7.94	1.91	10	2.47	8.28	5.78	7.83	6.18
111174	'Taar1'	0	0	0	7.06	0	0	0	0	0	0
111175	'Pecr'	14.12	2.45	3	11.4	15.7	13.68	0	3.13	6.25	6.25
111241	'Hmga1b'	0.07	0	0	0.06	0	0.02	0	0.05	0.13	0.04
112403	'Dxo'	32.37	19.04	12	44.2	25.1	31.68	25.8	15.31	44.39	18.52
112405	'Egln1'	0.29	0	4.72	2.3	5.44	1.76	6.56	2.42	0.06	1.28
112406	'Egln2'	54.48	45.48	55.5	10.3	39.4	22.4	67.2	38.97	78.11	18.68
112407	'Egln3'	2.21	0	2.48	0.02	0	2.37	0	0.54	0	0

112415	'Zfp607b'	1.4	2.25	0.4	5.97	3.72	6.47	6.44	3.33	5.97	2.75
112418	'1700102P08Rik'	0	1.53	3.07	0	0.41	0	0	1.76	1.17	0.12
112419	'Ifit1bl2'	0.01	0	0.03	0.05	0.03	0.03	0.16	0	0.04	0
112422	'Zfp979'	0.48	0	0	0	0	0	0	0	0	0
11287	'Pzp'	0	0.12	0	0	0	0.03	0.01	0.02	0	0
11298	'Aanat'	1.77	0	0	0	0	0	0	1.42	2.97	0
11302	'Aatk'	7.11	16.59	26	17.5	12.2	7.54	17.2	6.15	9.77	7.67
11303	'Abca1'	0.01	0.18	0	1.42	0.67	0.17	0	1.4	0	0
11304	'Abca4'	0.01	0.17	0	0	0	0	0	0	0	0
11305	'Abca2'	8.81	7.02	6.91	6.46	8.94	2.72	7.05	5.15	10.23	9.73
11306	'Abcb7'	0.93	0.71	4.4	5.8	0.69	1.59	0.53	0.28	1.52	7.06
11307	'Abcg1'	37.69	32.57	45.6	29.4	45.6	51.71	35.3	36.12	31.84	35.82
11308	'Abi1'	39.13	38.52	34.6	26	31.6	27.75	58	24.44	37.73	32.55
11350	'Abl1'	2.26	1.03	2.13	0.93	1.16	0.01	1.48	0.7	1.38	1.55
11352	'Abl2'	1.09	1.47	2.53	3.68	3.08	0.24	2.58	1.86	0.62	0.71
11363	'Acadl'	16.35	4.3	8.29	0.18	2.64	28.76	24	2.27	13.49	2.85
11364	'Acadm'	31.44	23.13	24.2	18.5	21.1	20.84	13.9	18.07	21.74	11.89
11370	'Acadvl'	47.98	66.97	55.6	43.1	67.5	51.03	39.6	74.15	65.2	35.04
113868	'Acaa1a'	71.19	77.04	71.3	51.4	79.6	121.5	89	79.07	67.36	92.71
11409	'Acads'	2.67	1.21	5.76	1.9	0.16	2.84	6.29	4.58	4.03	16.02
114128	'Laptm4b'	37.69	76.41	74.2	49.8	39.9	57.63	33.8	64.56	44.22	69.19
114142	'Foxp2'	0.74	0.22	1.93	0	19.5	6.1	0.22	0	0.22	2.41
114143	'Atp6v0b'	703.2	608	650	767	723	875.1	677	702.5	800.6	863.47
11416	'Slc33a1'	12.04	9.48	14.4	3.48	12.6	4.72	13.3	13.89	19.66	21.91
11418	'Asic2'	10.46	26.64	33.2	18.4	14.1	12.85	20.7	9.95	27.09	14.31
11419	'Asic1'	9.31	7.09	5.86	14.1	11.7	11.28	14.8	6.85	2.91	5.68
11421	'Ace'	1.68	0.62	3.81	3.15	1.03	0.79	5.62	1.63	10.48	3.21
114229	'Kiss1r'	2.44	2.46	5.8	0	28.1	7.18	0.04	4.64	0.41	1.18
11423	'Ache'	243.2	220.7	199	48.9	309	252.5	209	289.8	208.8	239.6
114230	'Aipl1'	0	0	0	0	0	0	0	0	0	2.06
114249	'Npnt'	0	0	0.01	0	0	0	2.74	0.01	6.95	0.92
114255	'Dok4'	2.15	5.67	5.52	9.78	0	3.75	5.94	13.31	5.83	0.27
11426	'Macf1'	7.07	7.21	6.76	5.01	5.34	7.42	6.4	4.92	6.2	6.85
11428	'Aco1'	8.24	4.3	4.65	0.01	3.7	4.71	13.8	2.94	3.69	10.53
11429	'Aco2'	123.6	186.6	295	97	172	215.5	165	190.9	97.12	164.33
11430	'Acox1'	25.49	28.45	41.3	18.1	23.2	11.02	34.9	21.61	30.07	41.89
114301	'Palmd'	16.05	3.46	0	8.58	18.9	0.46	5.12	4.35	2.25	2.46
114304	'Slc28a3'	0.14	0.18	0.13	0.3	0.19	0.11	0.09	0.05	0.14	0.08
11431	'Acp1'	66.26	55.23	54.8	62.9	78	103.9	53.1	72.25	77.06	67.87
11432	'Acp2'	23.93	43.06	34.9	43.5	38.1	22.39	30.4	44.45	31	52.81
11434	'Acr'	1.14	0.42	0.13	4.09	0.03	0.07	1.12	1.01	3.31	1.73
11435	'Chrna1'	0.29	1.41	0.95	0.76	0.89	1.13	0.53	0.35	0.63	1.21
11438	'Chrna4'	1.63	0	9.81	0	0.17	4.4	4.91	2.85	1.99	0.76
11440	'Chrna6'	2.36	0	0	0.02	0	0.02	0	0	0	0
11441	'Chrna7'	14.37	3.45	0.81	4.2	0.45	0	8.62	5.25	1.11	8.13
11443	'Chrnb1'	0	0	0	0	0.3	0	0	0	0	0.04
11444	'Chrnb2'	8.17	5.44	13.8	0.69	11.2	9.57	6.6	10.05	4.11	9.9
11447	'Chrnd'	0	0	0.05	0	0	0	0	0	0	0
114479	'Slc5a5'	22.1	19.58	44.5	23.1	10.7	5.46	10.9	75.42	3.33	0.01
11449	'Chrng'	0	0	0.52	0	0	0.02	0	0	0	0
114565	'Zbtb21'	6.5	8.13	7.76	8.19	3.73	8.98	3.6	4.09	8.29	3.81
114584	'Clic1'	11.93	5.49	3.07	9.06	33.1	37.83	5.45	8.43	7.09	37.01
114585	'D17H6S53E'	12.71	8.07	8.07	7.83	27.5	10.99	6.02	8.78	13.98	18.14
11459	'Acta1'	15.27	8.32	15	7.3	6.67	7.45	10.9	10.62	20.25	25.75
114601	'Ehbp1l1'	25.45	34.16	29.3	23.9	27.5	69.55	27.9	25.47	18.54	16.14
114602	'Zmynd10'	2.99	6.68	4.97	11.2	13.5	12.22	10.4	0	4.46	5.11
114604	'Prdm15'	1.15	1.04	1.22	0	0.47	0	0	0	1.52	2.63
114606	'Tle6'	1.66	2.01	0	3.18	4.47	0.02	5.82	2.22	1.42	0.79

11461	'Actb'	1748	1508	1867	1754	1960	1289	1553	1709	1512	1754.8
114615	'Elac1'	12.52	8.02	10.1	8.09	11.1	4.88	5.35	7.84	9.77	15.88
11464	'Actc1'	4.47	0.77	13.6	2.5	2.8	3.94	6.91	1.48	4.47	3.97
114641	'Rpl31'	123	132.1	130	195	114	157.4	117	98.41	136	98.03
114642	'Brdt'	2.19	0.91	0.19	7.37	2.36	1.36	0.1	1.27	1.41	1.33
114643	'Oas1c'	0	3.57	3.3	0	0	6.5	6.05	0	0	0.25
114644	'Slc13a3'	2.25	0.12	2.3	7.89	5.59	1.2	3.23	0.06	3.36	1.8
11465	'Actg1'	846.4	707.6	986	543	790	834.2	963	741.7	761.7	748.97
114652	'Ly6g5c'	0	0	0	0.72	0	0	0	0	0.11	0.43
114654	'Ly6g6d'	3.27	0	0	0	0	0	0	0	0	0
114663	'Impa2'	0	0.39	4.37	0	0	0	0	1.21	0	1.25
114664	'Hsd17b11'	8.84	7.92	6.99	2.4	9.72	7.32	1.72	2.92	9.47	13.25
114674	'Gtf2ird2'	8.77	5.31	2.48	0.02	5.5	0	7.11	5.23	7.12	0.84
114679	'Selenom'	266.5	188.7	350	334	343	399.2	235	297.9	392.3	350.21
11471	'Actl7b'	0	3.43	0	0	0	0	0	0	0	0
114713	'Rasa2'	1.6	3.91	5.78	0	1.56	3.54	6.15	3.33	5.86	2.73
114714	'Rad51c'	6.22	0	1.18	0	0	0.98	0	0.48	4.15	2.08
114715	'Spred1'	2.91	3.13	2.46	4.04	1.15	2.59	7.03	1.4	1.48	5.65
114716	'Spred2'	1.51	3.18	3.52	8.79	1.43	0.92	2.1	1.59	4.08	2.05
11472	'Actn2'	3.74	2.02	0.42	1.3	1.98	0.11	0.98	0.59	4.45	0.17
11474	'Actn3'	0.92	1.17	0	0	0	0	0	0	1.07	0
114741	'Supt16'	21.46	15.81	31.8	33.1	13.5	23.91	21.7	25.44	36.74	33.17
11475	'Acta2'	0.14	0.07	0.04	0.4	0.02	0.02	0	0.02	0.25	0
11477	'Acvr1'	2.18	0.48	0.33	5.37	1.67	0.55	0	2.08	3.15	4.93
11479	'Acvr1b'	7.21	8.65	11.5	0	4.46	5.97	11.5	6.39	0.22	5.6
11480	'Acvr2a'	2.47	4.11	1.17	7.2	1.04	0.85	2.73	1.45	11.85	7.35
11481	'Acvr2b'	1.96	1.39	6.01	3.35	4.53	1.52	0.13	0.71	0.26	3.34
11482	'Acvrl1'	0	0.35	0	0	0	0	0	0	0	0
11484	'Aspa'	20.06	8.56	0.03	0	7.97	7.46	0	5.93	0	0
11486	'Ada'	3.39	0	0.03	0	0.03	5.58	0	0	0	10.07
114863	'Plpbp'	56.91	29.25	30.7	28.6	29.1	37.99	51.4	43.43	32.02	45.89
11487	'Adam10'	9.65	13.35	7.15	12.7	14.4	7.14	5.45	6.69	7.04	9.03
114871	'Psg28'	0.02	0	0	0	0	0.12	0.03	0	0.07	0
114872	'Psg29'	0.03	0.02	0.01	0.21	0.04	0.14	0.01	0.04	0.05	0.02
114873	'Dscaml1'	2.35	0.9	6.82	2.81	1.23	2.59	2.33	0.08	2.91	2.26
114874	'Ddhd1'	3.79	2.34	0.63	7.21	3.15	0.29	0.93	2.19	1.72	1.65
114875	'Plcz1'	0.02	0	0	0	0	4.29	0	1.31	0	2.25
11488	'Adam11'	9.77	0.64	14.6	10.4	20.1	2.9	23.3	10.61	8	1.78
114886	'Cygb'	27.2	30.87	48.5	32.9	36.2	43.95	63.3	37.28	71.54	24.72
11489	'Adam12'	0	1.22	0.23	1.88	0	0	1.8	0.43	0	0.02
114893	'Dcun1d1'	9.41	12.37	9.33	24.4	17.8	11.87	1.97	13.21	10.17	15.82
114896	'Afg3l1'	5.66	5.37	4.9	7.27	5.21	4.47	8.35	3.06	9.85	6.85
11490	'Adam15'	7.57	15.79	27.4	18.4	10.7	6.47	7.28	14.69	10.53	7.26
11491	'Adam17'	0.7	3.89	0.03	0	0.21	0.13	0	0.05	1.71	4.21
11492	'Adam19'	0.42	0.05	2.68	0	0.69	1.65	0.45	0.27	0.45	0.62
11496	'Adam22'	53.63	58.36	77	9.39	36.3	28.93	68.7	28.36	33.99	29.54
11498	'Adam4'	0	0	0	0	0	0	2.22	0	1.71	0
11499	'Adam5'	0	2.49	0	0	0	0.02	0	1.18	0	0
11500	'Adam7'	0.1	0	0	0	0	0	0	0	0	0.81
11501	'Adam8'	0.13	4.57	2.37	2.5	0.02	1.61	0.97	1.78	3.5	2.81
11502	'Adam9'	3.03	2.16	11.4	8.76	0.94	0.01	5.02	1.53	0	2.89
11504	'Adamts1'	0.4	3.74	8.49	2.83	0	5.04	8.41	2.25	0.32	0
11512	'Adcy6'	3.15	7.24	6.55	4.27	5.16	1.51	3.37	2.88	0.32	2.02
11513	'Adcy7'	1.94	1.16	7.82	1.45	5.94	10.73	7.47	3.41	6.04	4.39
11514	'Adcy8'	1.01	1.31	1.86	0	3.8	2.14	0.03	2.48	3.87	0.96
11515	'Adcy9'	1.26	1.19	1.41	2.1	2.6	2.04	4.25	0.32	0.7	1.64
11516	'Adcyap1'	51.17	174.8	86.4	90.9	3.05	22.45	82.1	20.93	42.31	78.59
11517	'Adcyap1r1'	18.47	15.12	40.4	10.7	17.4	4.47	20.7	14.73	30.68	15.33

11518	'Add1'	64.98	36.96	39.7	10	54.6	36.32	37	44.63	50.11	54.23
11519	'Add2'	11.93	7.36	10.2	6.04	17.7	8.95	19.6	6.73	15.31	6.87
11520	'Plin2'	10.48	4.95	2.71	18.9	3.63	8.92	2.05	2.9	38.49	15.49
11529	'Adh7'	0.93	5.72	0.35	0	0	0	0	2.41	0	0
11532	'Adh5'	33.15	55.74	37.4	59.5	83	46.83	29.8	44.97	55.87	58.49
11534	'Adk'	43.33	54.51	34.1	26.3	21.3	41.35	47.1	47.41	42.13	37.16
11535	'Adm'	0	0	0.07	0	0	0	0	0	0.03	1.79
11536	'Gpr182'	0	0	0	0	5.77	0	4.44	0	10.57	2.16
11538	'Adnp'	5.42	6.57	13.1	21	2.37	6.04	9.37	8.17	2.4	8.62
11539	'Adora1'	7.78	2.41	9.47	7.39	11.5	2.32	17.9	6.78	5.15	0.1
11540	'Adora2a'	3.54	0.83	0	0	0.03	3.44	0	0.98	1.68	0
11541	'Adora2b'	0	2.17	0	0	0	0	0	0	0	0
11542	'Adora3'	0	0	0	0	0	0.69	2.42	0	0	0
11544	'Adprh'	113.2	145.7	154	112	107	153.1	86.5	138.6	166.8	116.2
11545	'Parp1'	7.14	2.59	13	1.7	0.38	1.38	15.7	10.36	0.49	3.36
11546	'Parp2'	20.94	19.54	7.72	22.5	19.1	37.82	1.1	28.87	16.99	20.26
11548	'Adra1b'	1.33	0	3.46	2.39	4.12	0.18	0	2.72	1.8	0
11549	'Adra1a'	0.12	4.35	2.31	5	0.39	1.09	0.6	0.12	2.54	2.7
11551	'Adra2a'	0.01	1.57	0	0	0	0	2.64	1.44	0.02	0
11552	'Adra2b'	0	0	0	0	0	0	0	0	2.12	0
11553	'Adra2c'	0	0	0	0	0	1.16	0	0	1.06	0.9
11554	'Adrb1'	0.72	0.02	0.02	0	0.59	0	0	0	0	0.02
11555	'Adrb2'	0	0	0	0	3.72	5.54	0	1.16	0	0
11556	'Adrb3'	3.18	1.34	0	0	0.02	1.81	0	1.06	0.79	0
11564	'Adsl'	22.59	22.46	24.9	12	28.5	21.38	26.1	23	29.28	26.5
11565	'Adssl1'	11.23	9.2	46.9	29.4	14	15.87	8.96	12.83	30.01	6.8
11566	'Adss'	32.02	21.44	26.7	15.4	32.6	12.62	36.4	19.28	18.38	38.91
11567	'Avil'	0	0.28	0	2.34	1.85	0	0	0.12	0.23	0.08
11568	'Aebp1'	0.01	0.05	2.27	0	4.42	0	0	0	0.06	0.36
11569	'Aebp2'	0.31	1.7	1.95	1.61	0.98	0	1.28	1.23	1.35	0.24
11576	'Afp'	0	0	0	0	0	2.54	0	0	0	0
11593	'Aga'	1.99	32.67	6.88	17.2	20.1	23.86	41	8.38	37.01	14.52
11595	'Acan'	0	0	0.13	0	0	0	0.06	0	0	0
11596	'Ager'	0.25	0.26	0	7.46	0	0	0.04	0	0	0
11600	'Angpt1'	0.01	8.77	1.49	0.03	6.1	2.96	6.32	0.07	2.79	0
11601	'Angpt2'	0.43	0	0.79	0	1.28	0	0.18	0	0	2.3
11602	'Angpt4'	0	0	0	0	0	0	0	1.01	0	0
11603	'Agrn'	3.03	5.01	21.9	2.32	6.34	5.55	11	4.73	11.41	5.71
11604	'Agrp'	2.4	0	2	0	3.2	6.7	13.7	0	0	0
11605	'Gla'	0.89	7.63	2.72	5.15	6.18	6.39	0	0.99	5.72	4.95
11606	'Agt'	106.2	139.7	49.6	106	45.5	66.44	72.3	69.42	82.98	12.36
11607	'Agtr1a'	4.5	0	0	0.03	0	8.77	0	0.02	2.93	17.72
11609	'Agtr2'	0	0	5.05	0	0	0	0	0	0	0
11610	'Agtrap'	4.97	5.4	4.71	6.11	9.77	10.01	3.24	11.14	8.39	17.06
11614	'Nr0b1'	0	0	0	0	0	0	0	0	0	3.1
11615	'Gm4737'	0	2.83	0.4	5.32	0	0	0	0.1	0.09	0.1
11622	'Ahr'	0	0	0	0	0	0	0	0	0	0.03
11629	'Aif1'	9.21	14.75	3.97	51.7	17.1	31.61	43.9	9.28	4.64	2
11630	'Crybg1'	0	0	0	0	0	0	1.28	0	0	0
11632	'Aip'	124.9	96.12	145	125	129	245.1	113	160	130.1	175.95
11634	'Aire'	0	0	0	0	0	0	0	0.31	0	0
11636	'Ak1'	65.62	92.42	115	130	138	148.2	127	162.5	88.89	95.8
11637	'Ak2'	34.95	31.03	28.6	69.9	29.6	56.33	20.1	30.02	28.52	43.33
11639	'Ak4'	5.85	7.48	7.99	11.1	7.21	4.16	20.3	14.96	8.84	6.39
11640	'Akap1'	2.25	1.78	0.45	2.07	0.26	0.57	0.01	0.7	0.91	1.4
11641	'Akap2'	4.52	5.36	5.44	3.5	3.27	2.79	3.63	5.96	7.02	4.54
11642	'Akap3'	0	0	0	0	0	0	0	0.58	0	0
11647	'Alpl'	5.05	0	0	8.72	0.41	0	7.12	3.45	0	1.59

11651	'Akt1'	7.82	8.72	13	15.9	10.4	3.29	12	8.3	10.77	7.83
11652	'Akt2'	9.03	5.78	9.43	12.3	4.53	5.83	6.44	6.18	14.49	7.22
11655	'Alas1'	21.74	10.68	19.6	15.7	31.6	20.93	16.5	35.42	19.27	31.18
11656	'Alas2'	0.42	0.65	0.12	0	0	0	0.16	0.5	0	0.24
11658	'Alcam'	43.88	35.14	25	7.53	38	50.39	131	43.27	148.2	159.36
11666	'Abcd1'	4.35	5.73	2.21	6.85	5.28	7.35	8.9	5.02	1.16	6.19
11668	'Aldh1a1'	9.22	1.75	1.73	0.03	19.1	18.69	0	5.47	2.05	5.08
11669	'Aldh2'	15.23	25.71	38.5	15.2	17.9	43.29	24.6	24.67	14.64	12
116701	'Fgfrl1'	0.18	0	6.6	0.02	1.36	7.61	0	3.59	0	0.02
11671	'Aldh3a2'	26.65	6.6	19.2	18.6	5.87	10.13	23.1	19.94	26.96	31.61
116731	'Pcdha1'	0	0	4.45	0	0	0	0	0	0	0.11
116732	'Tsga13'	0	0	0	0	0	0.46	0	0.9	0	0
116733	'Vps4a'	78	67.24	62.5	44.3	53	76.62	78.2	80.76	67.18	107.39
11674	'Aldoa'	2058	1786	2303	1734	2201	2624	2229	2545	2241	2144.97
116748	'Lsm10'	43.05	33.65	11.5	39.8	52.9	25.46	21	46.88	31.55	44.99
11676	'Aldoc'	196.9	241	294	215	69.9	310.7	381	348.5	265.2	177.15
11677	'Akr1b3'	89.6	51.55	87.4	66.9	81.9	125.2	88	67.63	106.8	49.83
11682	'Alk'	0.01	0.87	0.27	0	0	0	1.87	0	2.07	0.18
116837	'Rims1'	1.74	0.64	1.88	0.61	3.54	2.21	2.11	5.22	1.48	3.15
116838	'Rims2'	17.97	10.36	14.4	2.77	15.7	12.08	7.28	12.86	10.31	7.91
116847	'Prelp'	1.17	2.13	0	0	1.93	0	2.09	1.1	3.59	0
116848	'Baz2a'	0.67	5.13	2.97	2.29	3.96	1.46	1.55	2.09	0.73	2.98
11686	'Alox12b'	0	0	0.02	0	0	0.02	13.1	0	0	0
11687	'Alox15'	0	0	1.27	0.2	0	1.75	0.03	0.28	0	0
116870	'Mta1'	9.67	1.3	8.86	7.07	10.3	2.9	2.82	2.98	4.02	2.94
116871	'Mta3'	8.97	25.82	30	5.56	14.6	7.96	24.7	17.42	3.25	16.69
116872	'Serpinb7'	0	0	0	0	0	0	0	1.83	0	0
116873	'Stim2'	6.67	5.99	5.01	6.83	0.91	6.16	7.03	3.19	3.59	6.13
11688	'Alox8'	0.59	0.91	3.76	2.19	3.13	1.67	0.71	0.29	1.14	2.5
116891	'Derl2'	15.38	5.4	4.51	7.96	8.29	6.97	11	9.41	9.68	12.73
11690	'Alox5ap'	9.47	5.2	5.3	68.3	11.2	18.93	0	0.29	8.27	9.4
116904	'Alpk3'	0.01	0	0.02	0	0.02	0.02	0	0.25	0.01	0
116905	'Dph1'	16.65	24.19	18.2	0	14.6	13.28	9.07	10	25.42	9.78
116914	'Slc19a2'	6.24	2.1	0.44	5.07	3.72	2.42	12.1	1.6	4.32	3.32
11692	'Gfer'	18.94	15.49	23.1	18	22.3	11.26	8.01	8.72	12.85	16.31
116939	'Pnpla3'	0.81	3.05	1.91	0.44	1.67	0	4.26	2.99	0.74	0.12
116940	'Tgs1'	15.42	15.77	8.29	8.3	5.8	6.83	16.1	4.57	10.62	8.79
11695	'Alx4'	0	0.05	0	0	0	0	0	0	0	0
116972	'Fam57a'	4.44	11.32	12.9	1.27	4.84	24.2	1.01	12.38	5.6	12.06
11702	'Amd1'	34.56	28.43	28.8	28.5	39.8	18.08	43.4	55.65	36.78	26.22
11704	'Amelx'	0	0	0	0	0	0	0	0	0	0.82
11705	'Amh'	0	0	0	0	0	0	0	0.85	0	0.23
117109	'Pop5'	113.2	94.12	53.7	129	65	61.77	43.1	82.29	59.27	76.63
117146	'Ube3b'	0.73	6.58	8.35	4.8	9.4	3.15	6.27	4.39	0.01	5.79
117147	'Acsm1'	0	0	0	0	0	0	0	0.04	0	0
117148	'Necab2'	27.49	23.52	13.7	23.4	107	24.2	2.54	17.98	6.22	22.01
117149	'Tirap'	1.29	1.38	1.46	0.4	0.29	0.99	0.13	0.1	1.7	1.34
117150	'Pip4k2c'	12.71	10.44	17.4	0	10.8	6.75	15.2	18.26	15.27	7.54
117160	'Ttyh2'	8.12	15.78	9.63	9.47	15.4	17.38	1.17	1.05	7.72	3.22
11717	'Ampd3'	6.67	5.36	16.3	0.25	4.26	0.02	7.02	10.6	11.1	2.5
117171	'1110038F14Rik'	39.94	53.21	22.8	18.2	40.3	26.69	38.5	38.32	30.32	50.12
117197	'Bloc1s4'	2.41	6.49	3.08	26.8	6.61	6.76	1.72	3.38	6.14	3.1
117198	'Ivns1abp'	53.1	47.11	36.4	22.4	42.1	23.33	43.2	41.86	37.69	41.36
11722	'Amy1'	6.95	10.89	22.4	32	6.72	7.43	16.3	19.18	33.57	16.58
117229	'Stk33'	11.8	18.1	6.03	10.8	0	0.02	19.2	2.83	0	9.73
11727	'Ang'	2.57	3.9	5.96	0	0	8.27	0.36	1.09	6.25	2.82
11732	'Ank'	10.16	7.92	15.4	19.4	11.5	16.45	7.48	3.67	3.67	2.37
11733	'Ank1'	1.83	5.45	1.54	2.62	1.71	0.35	0.99	1.4	0.59	1.09

11735	'Ank3'	8.08	7.78	6.7	10.9	8.19	6.65	3.92	6.41	8.25	5.74
11736	'Ankfy1'	3.99	3.91	8.44	6.88	0.61	1.33	1.36	2.81	2.84	4.83
11737	'Anp32a'	26.01	21.27	18.3	17.8	35.6	38.62	23.6	26.75	39.52	26.38
11739	'Slc25a4'	1168	989.1	1204	1016	1078	1215	1024	1441	1029	1208.21
11740	'Slc25a5'	248.7	164.3	380	236	270	393.7	275	288.1	238.2	157.91
11744	'Anxa11'	0.66	0	0	1.61	1.99	0.25	2.89	0	1.91	6.04
11745	'Anxa3'	0	0	0	11.5	0.12	8.38	3.77	5.45	0	0
11746	'Anxa4'	0	0	0	0	0	0	0.93	0	0	0.6
11747	'Anxa5'	68.56	36.67	72.6	173	115	123	25.8	50.89	120.5	117.87
11749	'Anxa6'	76.68	71.59	51.1	66.2	45.1	47.55	33.8	55.99	27.1	43.96
11750	'Anxa7'	26.39	45.48	21.2	24.5	14.6	31.91	30.3	37.07	47.29	30.66
11757	'Prdx3'	77.94	89.67	95.8	62.5	91.2	67.38	118	103.3	73.84	55.07
11758	'Prdx6'	102.1	85.68	56.5	96.1	118	84.74	86.8	63.97	49	51.24
117589	'Asb7'	1.49	0.38	0.62	6.96	1.36	1.32	1.28	3.91	0.05	3.64
117590	'Asb10'	0	0	0.02	0	0.02	0.04	0	0	0.02	0.02
117591	'Slc2a9'	0	0.06	0	0	0	0	0	0	0	0
117592	'B3galt6'	17.08	19.66	16.3	4.57	13.1	16.02	25.8	4.98	32.17	4.27
117599	'Helb'	0	0.03	0.13	0.02	0.98	0.01	0	0.06	0.06	0.02
117600	'Srgap1'	1.21	2.38	1	1.5	2.27	2.77	0.5	0.57	1.07	1.34
117606	'Boc'	3.13	0.42	0.04	0	0.62	2.83	0.4	0	0	0.74
11761	'Aox1'	0.6	0	0	0	0	0	0	0	0.01	0
11764	'Ap1b1'	56.18	50.53	86	18.6	50.3	29.04	73	71.89	39.71	68.04
11765	'Ap1g1'	5.98	1.94	4.63	1.92	7.5	3.07	10.3	2.85	6.28	6.96
11766	'Ap1g2'	0.32	0.38	2.97	0	0.02	5.46	1.85	5.32	0	1.65
11767	'Ap1m1'	93.54	90.48	122	46.1	40.9	105	101	76.71	95.44	137.04
11768	'Ap1m2'	0	0	0	0	0.23	0.04	0	0	0	0
11769	'Ap1s1'	181.4	231.7	180	203	215	196.6	158	228.9	264.9	189.62
11770	'Fabp4'	0	0	0.08	0	10.3	0.08	0.17	0	0.07	0.23
11771	'Ap2a1'	29.5	30.1	68.6	19.3	36.9	13.4	49.5	36.21	42.39	42.05
11772	'Ap2a2'	57.79	46.56	63.6	45.4	41.1	14.79	79	33.84	60.34	51.31
11773	'Ap2m1'	499.3	428.6	459	443	588	689.6	557	581.5	556.4	573.69
11774	'Ap3b1'	11.12	15.72	5.42	5.63	6.89	11.74	27.4	11.78	14.26	19.98
11775	'Ap3b2'	65.07	52.7	70	44.7	46.8	25.99	22.2	44.98	35.34	57.7
11776	'Ap3d1'	33.98	43.88	61.9	30.3	36.5	30.06	60.4	28.41	44.65	42.83
11777	'Ap3s1'	11.46	20.11	24.1	16.6	16.4	26.29	15.8	14.87	38.69	10.75
11778	'Ap3s2'	12.79	16.24	6.2	6.29	11.7	8.97	6.99	12.24	11.29	8.98
11781	'Ap4m1'	1.84	3.05	7.23	2.64	13.4	8.51	11.7	4.16	8.33	7.23
11782	'Ap4s1'	84.95	65.2	76.8	68	53.8	64.95	73.4	69.9	105.9	41.85
11783	'Apaf1'	0.77	0.87	0.31	0.02	1.27	0.43	0.01	0.59	0.1	0.39
11784	'Apba2'	41.76	44.91	87	26.3	46	43.38	27.3	83.19	41.53	31.44
11785	'Apbb1'	229.2	250.2	258	200	300	226.4	202	280.1	299.5	329.76
11787	'Apbb2'	10.34	11.4	10	15.5	13.7	6.95	12.9	3.17	13.89	10.49
11789	'Apc'	7.64	4.9	10.1	5.2	5.17	5.31	6.82	6.96	8.93	15.66
11790	'Speg'	3.73	9.73	2.55	2.16	11.6	8.97	2.57	6.66	0.27	0.73
11792	'Apex1'	122.4	93.93	80	144	105	138.9	112	142.5	101.7	113.21
11793	'Atg5'	22.63	32.66	12	38.6	21.1	37.1	20.9	29.21	32.81	27.84
11796	'Birc3'	0	0	3.03	0	2.16	0	0	0	0	0.93
11797	'Birc2'	19.18	9.5	6.6	24	10.4	7.09	23.6	24.87	14.13	15.39
11798	'Xiap'	1.7	2.81	0.82	2.28	3.98	1.5	3.9	0.82	1.89	1.16
11799	'Birc5'	2.52	0.35	0.26	0.27	0.21	0.14	0.06	0.09	3.2	4.8
11800	'Api5'	25.8	26.04	33.2	11.7	27.7	11.34	48.4	33.55	21.75	42.42
11801	'Cd5l'	0.02	0	0	0	0	0	0	0	0	0
11803	'Aplp1'	176.1	190.1	139	203	153	194.6	177	193.6	211	125.18
11804	'Aplp2'	140.2	96.82	222	83.9	130	99.22	240	157.3	175.6	130.26
11806	'Apoa1'	2.83	0.81	1.62	7.74	0.98	7.56	2.73	4.6	0.59	0
11807	'Apoa2'	13.59	9.58	6.87	20.8	5.01	0	17.2	13.82	9.47	22.33
11808	'Apoa4'	0	0.67	0	0	0	0	0	0	0	0
11810	'Apobec1'	0	0	0	0	0	0	0	2.63	3.76	7.61

11811	'Apobec2'	0.11	2.62	0.12	0	0	0.08	0.04	0	0.11	5.81
11812	'Apoc1'	6.01	9.34	8.6	18.6	0	10.32	0	2.02	0	2.28
11814	'Apoc3'	45.45	83.22	18.9	143	0	6.97	14.1	21.49	26.77	0
11815	'Apod'	15.9	26.54	0.87	3.56	45.3	24.53	0.03	1.26	4.18	1.81
11816	'Apoe'	1568	1109	1006	1351	826	1371	1178	785.4	758.3	528.18
11818	'Apoh'	0	0.25	3.24	0	0	7.01	0	0	0	0
11819	'Nr2f2'	0.64	17.57	7.1	6.68	0.02	0.29	20.3	14.83	1.29	0
11820	'App'	155.3	215.5	154	246	133	103.9	218	198.7	153.9	101.49
11821	'Aprt'	63.6	96.71	102	114	60.9	99.25	73.7	88	108.6	90.15
11826	'Aqp1'	0	0	0	4.87	0.6	0	0	0	0	0.02
11829	'Aqp4'	5.5	6.92	1.18	22.5	5.33	8.65	0	2.81	7.17	2.76
11830	'Aqp5'	0	0	2.41	0.04	0	0	0	0	0	0
11831	'Aqp6'	0	0.57	0	0	0	1.86	0	0	0	0
11832	'Aqp7'	0.09	0.22	0.67	3.75	0.48	0.25	0.3	0.93	0.19	0.2
11834	'Aqr'	14.89	6.77	8.36	8.53	6.59	9.52	3.88	5.75	11.15	5.28
11835	'Ar'	10.68	14.58	6.39	10.8	2.31	3.12	6.14	17.52	1.91	8.87
11836	'Araf'	565.9	513.4	476	498	437	581.5	460	566.2	549	507.72
11837	'Rplp0'	191	233.2	160	230	163	191.5	199	188.6	225.2	197.41
11838	'Arc'	7.11	39.28	41.5	37.1	4.26	22.38	16.2	20.1	2.38	52.74
11840	'Arf1'	531.3	553.6	467	410	536	599.5	545	555.7	456.1	600.62
11841	'Arf2'	34.86	25.9	46.2	63.5	47.1	23.37	26.7	50.68	52.45	71.3
11842	'Arf3'	44.88	38.43	31.7	47.3	52.9	37.08	38.4	55.47	53.41	35.2
11843	'Arf4'	110.4	117.3	118	123	79.3	108.5	110	102.9	115.8	91.66
11844	'Arf5'	35.16	36.76	69.6	60	55.3	40.75	37.3	26.51	51.73	29.13
118445	'Klf16'	0.59	1.98	2.38	16.8	6.51	0.51	2.42	3.26	5.52	5.41
118446	'Gjc3'	4.53	3.28	19.1	8.21	10.2	3.09	11.1	3.08	3.46	2.92
118449	'Synpo2'	0.02	0.26	0.05	0	0.23	0.03	0	0.74	2.09	1.09
11845	'Arf6'	3.05	4.38	5.12	2.06	5.66	2.91	3.24	1.59	2.76	4.9
118451	'Mrps2'	9.18	24.8	24.8	27.6	24.4	14.76	19	20.83	9.18	17.36
118452	'Baalc'	11.13	4.88	5.73	0.72	3.82	14.9	14	17.9	13.54	5.37
118453	'Mmp28'	0	0	0	0	1.13	0	0	0	0	0
118454	'Gjc2'	0.28	0	0	0.03	3.37	7.47	0.02	0.26	0	0
11846	'Arg1'	0	0	3.07	0	30	0	0	0	0.91	0.7
11847	'Arg2'	5.83	10.05	12.7	0.25	4.21	7.8	12.2	6.41	0	0.37
11848	'Rhoa'	94.6	79.92	48	64.9	89.2	82.58	83	42.71	64.41	51
11852	'Rhob'	12.86	18.32	15	28.5	9.93	10.55	19.1	16.18	14.83	6.6
11853	'Rhoc'	16.68	4.51	41.6	16.5	0	13.9	13.2	5.75	19.58	8.07
11855	'Arhgap5'	5.67	4	6.99	4.76	6.27	6.7	0.42	4.41	2.71	6.64
11856	'Arhgap6'	8.44	3.52	0	0	7.16	11.64	2.46	7.79	5.81	6.35
11857	'Arhgdib'	2.06	0.05	4.05	18.3	7.41	42.25	0	13.22	7.53	0
11858	'Rnd2'	19.65	57.5	41.4	49.9	55.5	46.56	40.4	36.78	50.31	23.77
11861	'Arl4a'	6.41	12.05	7.36	32.4	2.41	3.65	25.2	5.02	18.33	10.46
11863	'Arnt'	2.32	5.11	5.43	1.02	8.23	1.39	2.86	4.56	1.97	1.93
11864	'Arnt2'	5.55	3.56	4.11	3.4	2.46	5.75	18.1	4.55	7.16	1.01
11865	'Arntl'	7.58	21.76	8.83	4.08	9.76	4.11	3.42	7.62	5.8	5.47
11867	'Arpc1b'	7.51	8.55	0.13	9.3	9.82	35.18	9.04	10.96	36.62	64.34
11870	'Art1'	0.06	1.17	0.17	0.04	0	0	0.14	0	4.53	0.09
11875	'Art5'	0	0	0.09	0	0	0.04	2.72	0	0	0
11876	'Artn'	2.48	1.94	0	0	0	1.73	2.68	2.85	1.78	0.68
11877	'Arvcf'	2	8.8	8.55	10.5	8.28	7.83	10.1	4.14	6.99	3
11878	'Arx'	0	1.16	1.51	0	6.7	0.26	3.29	0	0	0.28
11881	'Arsb'	20.58	23.6	22.5	5.66	12.2	1.01	13.4	26.4	13.29	24.39
11883	'Arsa'	26.97	28.18	27.9	25.9	30.9	19.12	34.5	25.79	48.56	34.38
11886	'Asah1'	62.66	49.92	61.2	53.9	27.8	37.07	30.6	51.75	41.59	54.24
11889	'Asgr1'	1.28	1.44	0.05	4.73	0	0	0.85	0.27	0.77	1.73
11891	'Rab27a'	5.96	6.7	3.86	9.9	6.99	10.04	0.18	3.07	0.63	4.17
11898	'Ass1'	275.1	213	194	123	303	324	250	193.6	289.1	304.82
11899	'Astn1'	19.91	13.41	36.1	21.6	10.1	2.84	22.3	9.46	15.91	20.2

11905	'Serpinc1'	0	0	0.27	0	0	0	0	0	0	0
11906	'Zfhx3'	1.08	2.14	2.77	2.01	1.78	0.92	3.53	1.77	2.86	1.29
11907	'Ate1'	92.79	85.4	86.4	61.6	91.4	90.05	98.3	53.24	80.06	79.3
11908	'Atf1'	2.75	4.87	1.11	7.27	0.02	0	3.02	4.42	8.62	1.24
11909	'Atf2'	39.59	26.6	41.7	30.9	36.4	10.82	34.7	28.84	42.4	51.93
11910	'Atf3'	0	16.92	5.61	63.9	0.02	86.51	4.97	0	0.02	0
11911	'Atf4'	61.7	55.88	120	123	53.6	24.62	46.6	66.1	45.22	44.8
11920	'Atm'	0.75	0.71	0.77	4.69	1.8	0.09	3.2	0.04	0.39	0.05
11927	'Atox1'	202.4	167.1	177	220	156	272.2	189	147.7	215.9	210.42
11928	'Atp1a1'	56.22	33.46	31.8	55.4	33.8	21.64	40.6	33	51.9	36.98
11931	'Atp1b1'	822.5	683.2	804	487	725	711.4	683	642.2	749.4	463.28
11932	'Atp1b2'	170.1	109.3	115	61.6	105	133.2	195	165.3	206.9	138.47
11933	'Atp1b3'	123.3	161.2	87	145	90.9	179.1	140	117.5	116.3	169.18
11936	'Fxyd2'	13.17	57.6	10.1	42	15	16.15	25.2	7.27	8.68	10.79
11937	'Atp2a1'	0	0	0	0.41	0	0	0	0	0.65	0
11938	'Atp2a2'	18.3	11.41	18.4	16.5	17.6	9.29	18.7	13.16	9.84	13.34
11941	'Atp2b2'	17.48	12.73	18.2	11.8	27.6	7.02	19.8	10.36	17.78	15.51
11944	'Atp4a'	0.03	0	1.18	0	0	3.76	0	1.73	0	1.91
11945	'Atp4b'	0	0	1.22	0	0	0	0	0.4	0	0
11946	'Atp5a1'	409.5	358.1	625	416	444	404.5	436	421	355.3	382.03
11947	'Atp5b'	941.9	906.6	1516	687	990	1184	1178	1002	921.4	998.33
11949	'Atp5c1'	437.8	431.2	496	307	476	516.5	537	509.2	399.6	422.46
11950	'Atp5f1'	256.8	239.2	295	262	255	332.9	285	327.6	301.7	297.19
11951	'Atp5g1'	652.4	589.9	727	813	741	694	554	742.2	580.5	538.46
11957	'Atp5j'	760.9	632.1	836	791	705	920.3	756	569.4	672.5	679.86
11958	'Atp5k'	155.1	200.9	254	413	146	528.6	173	96.69	297.2	127
11964	'Atp6v1a'	159.7	146.6	169	149	135	112.4	145	165.1	126.9	145.74
11966	'Atp6v1b2'	464.1	505.1	522	502	587	453.4	631	661.4	587.3	524.02
11972	'Atp6v0d1'	194.7	165.2	233	167	188	220.6	230	216	325.6	264.25
11973	'Atp6v1e1'	446.4	535.1	526	406	464	692.9	586	585.6	492.3	420.29
11974	'Atp6v0e'	70.23	38.93	26.6	40.9	45.7	87.15	71.6	20.01	34.75	30.96
11975	'Atp6v0a1'	101	98.68	118	74.4	89.4	30.06	82.5	117.1	102.4	123.03
11977	'Atp7a'	4.39	0.76	0.65	1.23	0.76	0.56	0	0.91	0	0.85
11979	'Atp7b'	0.38	0.77	1	4.88	2.17	0.26	1.48	0.42	1.9	0.72
11980	'Atp8a1'	13.15	12.49	11.3	14.6	12.3	9.51	12.8	8.34	12.32	8.43
11981	'Atp9a'	31.72	50.74	35.7	45.9	80.5	61.4	44.2	35.03	32.67	63.75
11982	'Atp10a'	1	0.41	2.65	0.01	0.14	0	0	0.09	0	0.1
11983	'Atpif1'	303.5	283.8	316	340	338	433.8	298	253.4	321.7	307.24
11984	'Atp6v0c'	2562	2245	2245	2518	2702	3031	2391	2445	2123	2666.18
11987	'Slc7a1'	3.1	4.6	8.15	5.59	4.18	0.59	1.96	1.91	2.54	3.75
11988	'Slc7a2'	0.53	0.04	0.02	0.03	0.02	0.02	0.01	0.04	0.02	0.01
11989	'Slc7a3'	38.27	76.29	46.9	13.9	33.5	59.12	76.7	93.33	82.9	30.82
11990	'Atrn'	3.98	2.08	5.05	4.81	5.92	4.23	5.08	4.96	4.36	3.5
11991	'Hnrnpd'	2.69	2.66	7.12	12.6	11.3	4.87	4.51	2.37	5.04	2.65
11992	'Auh'	61.81	71.85	95.7	51.7	83.7	88.83	71.7	94.81	82.05	83.13
11993	'Aup1'	98.33	65.96	77.7	54	68.8	99.46	86.3	59.44	86.43	64.06
11994	'Pcdh15'	0.73	0.32	6.93	4.89	1.75	1.6	9.06	2.94	1.57	0
11997	'Akr1b7'	0	0	4.21	0	0	0	0	0	0	0
11998	'Avp'	0	4.51	0.09	0	0	0	0	0	0	0.46
12005	'Axin1'	7.6	1.92	0.48	7.63	3.74	6.42	9.93	1.48	3.68	0
12006	'Axin2'	0.27	0.16	0.86	0.77	0.43	0.88	0.02	0	0.02	3.46
12009	'Cep131'	3.85	0.49	5.78	9.14	2.8	3.07	2.25	3.17	5.12	3.28
12010	'B2m'	111.1	93.11	91.4	156	72.3	450.5	108	53.48	208.3	248.57
12013	'Bach1'	0.16	0.38	0.57	2.79	1.64	0	0	0.74	0.45	0.01
12014	'Bach2'	0.01	0.91	0.18	0.81	0.65	0.21	0.13	0.26	0.03	0.9
12015	'Bad'	91.95	35.11	49.2	43.8	81.2	70.8	74.5	61.39	51.14	42.79
12017	'Bag1'	16.49	16.53	15.4	35.5	16.7	23.9	11.6	10.08	38.98	4.86
12018	'Bak1'	0.57	0.89	3.23	0.24	0	0	0.4	0.56	2.95	0.41

12021	'Bard1'	0	0.1	0	0	0.6	0	0.01	0	0.82	0
12023	'Barx2'	0	2.16	0.54	0.19	0.66	0	0	1.17	2.43	0
12028	'Bax'	78.18	54.7	47.6	152	108	66.75	73.4	65.91	86.49	70.04
12029	'Bcl6b'	0	0	2.63	0	0	0	0	0	0	0
12032	'Bcan'	23.19	33.71	73.1	43	16.7	38.1	83.4	28.82	41.66	4.58
12033	'Bcap29'	52.75	55.33	49.3	65.8	39	67.17	41.2	52.01	73.39	66.41
12034	'Phb2'	90.97	90.97	84.8	138	72.4	121.5	68.6	110.7	98.53	50.23
12035	'Bcat1'	22.42	24.57	28.2	21.8	24.6	32.08	28.6	21.5	5.83	24.19
12036	'Bcat2'	8.11	21.15	0.95	0	10.9	9.47	11.9	9.95	13.29	0
12038	'Bche'	0	0	0	0.01	0.02	0	0	0	0.01	0
12039	'Bckdha'	34.29	34.31	43.7	43.4	59	53.11	28.2	24.23	40.39	24.98
12040	'Bckdhb'	21.78	21.99	36.6	24.1	11.2	21.35	11.6	12.67	37.99	0.03
12041	'Bckdk'	38.07	34.19	25.8	37.5	39.9	46.06	58.3	42.28	39.47	48.01
12042	'Bcl10'	3.92	18.36	12	18.7	4.56	5.35	21.5	15.48	0.86	16.79
12043	'Bcl2'	2.07	2.73	2.79	2.81	1.57	1.57	2.29	4.06	3.96	1.05
12044	'Bcl2a1a'	1.41	0	0	0	0	4.73	0	0	0	0
12045	'Bcl2a1b'	0	2.37	0	8.05	0	15.34	0	0	0	6.21
12047	'Bcl2a1d'	0	0.28	0	1.26	0.46	5.8	0	0	0	0.59
12048	'Bcl2l1'	89.11	98.77	108	52.3	124	80.11	81.4	91.02	112.3	95.59
12050	'Bcl2l2'	3.55	2.62	0.76	0.23	1.81	0.04	2.63	0.54	3.16	1.91
12051	'Bcl3'	1.24	0.53	0.95	0	0	3.33	0	0	0.43	1.47
12053	'Bcl6'	3.49	3.22	0.62	4.7	1.57	3.83	0	1.16	2.82	1.14
12054	'Bcl7b'	46.83	64.31	48.7	18.9	68.9	40.74	58.3	63.08	64.97	42.64
12055	'Bcl7c'	0.99	0.57	2.43	0.01	0.59	2.1	0.74	0.13	0.46	1.08
12057	'Opn1sw'	0.56	0	1.39	0	0	0	0	0.65	0	0.02
12061	'Bdkrb1'	0	0	1.28	0.04	0	0	0	0	0	0
12062	'Bdkrb2'	0	0	0	0	0	0	0	0	0	1.46
12064	'Bdnf'	9.64	68.7	19.5	34.1	24.2	45.62	6.26	14.24	51.36	62.09
12068	'Bet1'	9.42	16.22	14.8	15.5	20.7	20.67	16.4	16.97	26.01	47.76
12069	'Bex2'	1796	1962	1521	2848	1822	2068	1396	1974	1711	2038.57
12070	'Bex3'	899.6	877.5	843	1162	848	1227	789	836.9	869.8	1206.79
12075	'Bfsp1'	0.02	2.81	0	12.1	3.46	0	6.3	0	0	0
12091	'Glb1'	23.06	35.34	13.6	28.1	15.6	34.83	35.3	32.41	40.86	39.33
12095	'Bglap3'	0	0	0	0	0	0	0	0	0.29	0
121021	'Cspg4'	0	0	11.2	1.76	1.14	0.73	9.61	0.38	0.01	0.01
121022	'Mrps6'	133.7	62.34	79.5	69.6	63.5	57.69	86.5	68.98	70.62	75.06
12116	'Bhmt'	0	0	0	0	0	0.02	0	0	0	0
12121	'Bicd1'	19.96	17.96	23.4	8.84	19.6	18.39	12.7	14.43	18.57	22.07
12122	'Bid'	27.65	27.55	41.1	32.1	12.6	42.8	16.6	28.89	46.6	37.98
12123	'Hrk'	0.51	1.79	0	2.36	0.03	1.81	0.82	0.16	1.38	0.73
12125	'Bcl2l11'	0	1.07	0.02	0.2	0.03	0	0.01	0	0.01	0
12140	'Fabp7'	33.32	43.88	26.9	34.3	22.1	46.91	89	29.02	17.05	42.95
12142	'Prdm1'	0	0	1.49	0	0	0	0	0	0	0
12144	'Blm'	0	0	1.14	0	0	1.53	0	0.93	1.39	1.93
12145	'Cxcr5'	0	0	0	0	0	0	0	0	3.74	0
12151	'Bmi1'	12.29	3.67	2.77	1.58	1.56	0	1.56	1.86	2.46	2.77
12153	'Bmp1'	7.95	3.17	4.67	6.55	0.73	4.76	0.03	4.1	0.01	2.29
12156	'Bmp2'	0	0	1.12	0	0.79	0	0	0	0	0
12159	'Bmp4'	0	0	8.41	0	0	0	0	0	0	0
12161	'Bmp6'	0.48	0	0	0.02	0	0	0	0	0	0
12162	'Bmp7'	0	0.14	7.83	0.11	5.02	0	2.45	1.23	0	0
12163	'Bmp8a'	0	0	0	4.08	0	0	0	0	0	0
12164	'Bmp8b'	0	0	0	0	0	0	0	0	1.99	2.5
12166	'Bmpr1a'	2.18	1.35	1.47	1.89	1.13	1.33	2.85	1.12	1.47	2.95
12167	'Bmpr1b'	3.18	0.1	0	0.01	0.38	1.92	0	0.02	1.35	0.32
12168	'Bmpr2'	2.1	2.83	2.41	5.05	4.18	2.12	5.09	1.06	0.18	1.13
12175	'Bnip2'	15.78	18.12	12.6	25.3	10.5	6.17	10.3	10.77	11.86	15.26
12176	'Bnip3'	184	115.5	139	122	183	192.4	117	200.6	115.7	133.56

12177	'Bnip3l'	58.96	62.01	33.9	49.1	81.1	59.02	54.2	58.21	51.16	56.41
12180	'Smyd1'	1.01	0.01	0.01	0	0	1.78	0	0	0.01	0
12181	'Bop1'	21.45	28.21	23.2	13.6	18.1	17.84	15.4	31.32	9.51	30.53
12183	'Bpgm'	33.1	26.43	43.3	63.4	39.2	39.71	16.3	49.96	16.81	57.82
12189	'Brca1'	0	0	1.59	0	0	0	0	0	0.11	0
12190	'Brca2'	1.59	0.3	2.47	2.67	0.16	0.84	1.85	0.07	1.64	0.81
12192	'Zfp36l1'	0.99	6.88	11.6	18.1	3.37	8.65	12.5	2.69	5.16	0.61
12193	'Zfp36l2'	1.5	1.05	0.6	2.81	0.03	0.28	5.24	1.92	0.67	0.9
12209	'Brs3'	7.89	16.63	8.61	0.02	0.02	0.03	0.02	34.03	56.16	110.34
12211	'Birc6'	4.85	5.08	5.17	10.2	6.77	3.34	4.48	4.51	6.12	5.53
12212	'Chic1'	13.88	7.94	30.2	0.75	3.06	6.24	19.7	4.36	7.3	9.75
12215	'Bsg'	863.9	1092	797	914	869	992.5	784	1056	1011	1182.12
12217	'Bsn'	0.78	0.95	2.05	0	2.92	0.21	2.19	1.04	2.01	1.42
12223	'Btc'	0.91	0.7	0.23	3.63	0	0	0	0	0	0.04
12224	'Klf5'	1.49	5.26	0	14.5	0.17	0.03	2.16	0	0	0
12226	'Btg1'	5.11	5.7	1.81	19.6	3.64	2.34	1.64	4.25	5.34	1.12
12227	'Btg2'	12.33	23.34	14.8	46.5	10.7	20.65	5.61	3.33	20.86	32.39
12228	'Btg3'	12.35	8.51	1.41	11.2	11.4	0.19	0	0.37	8.28	5.31
12229	'Btk'	0	0.17	0	0.27	0	0	0	0	0	0
12231	'Btn1a1'	0	0	0	0	0	0	0	0.24	0	0
12234	'Btrc'	42.13	25.74	27.3	18.6	15.3	38.68	26.2	20.61	32.19	37.44
12235	'Bub1'	0.02	0	0	0	0	0	0	0	0	0
12236	'Bub1b'	0	0.9	0	0	2.5	0	0	0	1.76	0
12237	'Bub3'	138.9	109.8	182	210	104	147.7	109	270.9	191.4	148.33
12238	'Commd3'	126	119.8	136	131	140	189.7	75.1	113.7	91.47	109.78
12257	'Tspo'	0.38	0.18	4.65	7.57	0	31.14	0	3.45	0.05	8.44
12258	'Serping1'	0	3.9	7.62	9.47	5.63	0.03	0	4.98	5.54	8.8
12259	'C1qa'	0	22.78	9.16	35.1	45.7	219.4	37	4.32	16.08	3.62
12260	'C1qb'	3.82	45.54	27.5	48.7	42.8	444.1	40	24.47	5.27	21.31
12261	'C1qbp'	101.2	90.77	111	70.4	84	104.6	80.5	120.4	93.68	81.09
12262	'C1qc'	8.31	8.98	2.92	44.9	29.9	50.07	0.05	1.22	0.04	0
12263	'C2'	1.62	0.02	0	0	0	0	0	0	0	0
12265	'Ciita'	0	0	0	0	0.25	0	0	0	0	0.01
12266	'C3'	0	0	0	0	1.08	0	4.48	2.15	0	0
12267	'C3ar1'	0.46	0.93	0	0	0	4.18	0	0	0	0
12268	'C4b'	0	0	3.43	5.43	24.3	0	0.01	0	0.01	0.01
12274	'C6'	0	0	0	0.08	0.07	0	0	0	0	0.03
12282	'Hyou1'	16.81	10.48	15	5.13	7.05	1.85	8.16	9.36	38.79	15.92
12283	'Cab39'	6.39	4.83	9.83	34	10.9	0.52	5.09	10.6	6.66	7.78
12286	'Cacna1a'	0.63	0.33	2.58	1.96	1.07	1.23	0.77	0.09	0.13	0.64
12287	'Cacna1b'	5.28	10.14	8.91	5.27	3.96	6.17	7.22	7.16	13	7.45
12288	'Cacna1c'	0.65	1.34	0.16	0.73	0.79	0.01	1.36	0.47	0.38	0.54
12289	'Cacna1d'	2.25	2.1	1.93	1.33	1.85	0	1.2	1.01	2.54	1.55
12290	'Cacna1e'	10.56	8.1	17.5	4.89	13.8	7.8	10.1	9.56	1.97	12.31
12291	'Cacna1g'	3.04	4.73	1.99	9.3	9.24	1.12	4.38	2.88	3.91	1.73
12292	'Cacna1s'	0.24	0.44	0	0	0	0	0	0	0	0
12293	'Cacna2d1'	5.29	15.08	14.1	23	14.7	8.92	19.9	3.37	14.6	26.86
12294	'Cacna2d3'	4.14	9.35	0.15	0.45	0.03	0	0	1.97	5.39	0
12295	'Cacnb1'	15.54	9.46	8.28	2.14	10.7	11.38	8.56	8.2	13.75	5.64
12296	'Cacnb2'	1.37	0.52	1.66	0.86	1.96	2.92	2.91	1.36	3.66	1.55
12297	'Cacnb3'	15.76	36.36	34.3	10.2	22.4	32.98	39.9	31.95	38.08	22.75
12298	'Cacnb4'	11.46	6.73	11.6	10.1	8.12	4.06	7.11	4.23	8.92	5.39
12299	'Cacng1'	0	0	0	0	0	0	0	1.98	0	0
12300	'Cacng2'	8.43	5.43	18	16.1	11.2	10.67	12	8.76	9.63	4.04
12301	'Cacybp'	183.6	186.4	143	171	106	120.5	105	141.1	154.7	179.34
12304	'Pdia4'	37.11	91.93	37.1	14.5	48.4	41.96	20.7	52.45	66.11	78.31
12305	'Ddr1'	5.41	5.57	3.11	3.68	13.3	2.91	1.5	5.2	4.5	4.5
12306	'Anxa2'	0	0	0.04	0	7.7	2.24	0	0	0.22	0

12307	'Calb1'	101.8	221.7	33	146	161	47.77	99.4	117.2	74.68	128.99
12308	'Calb2'	238	269.1	228	23.4	441	296.1	436	314.8	498.1	365.63
12309	'S100g'	0	2.23	1.35	0	0	0	0	0	0	0
12310	'Calca'	0	0	0	0	0	20.55	0	0	0	11.28
12311	'Calcr'	30	61.08	68.4	12.1	0.84	0.47	126	23.74	46.35	1.86
12313	'Calm1'	664.6	535.4	465	725	701	733.9	552	702.1	499.8	562.54
12314	'Calm2'	2975	2797	2122	3724	3591	3574	2877	3643	2719	2965
12315	'Calm3'	267	183.4	262	202	295	199.4	319	313.9	299.8	237.49
12316	'Aspm'	0	0	0	0.02	0	0	1.78	0	0	0
12317	'Calr'	132.6	135.4	114	94.4	66.4	91.06	120	69.33	84.23	104.8
12319	'Car8'	0.01	0.23	4.85	6.46	3.99	1.4	1.39	1.68	2.81	1.6
12321	'Calu'	17.99	13.78	36.9	12.3	17.4	11.71	20.7	19	30.75	28.31
12322	'Camk2a'	74.51	104.1	81	44	52.6	36.44	76.4	36.98	4.66	56.53
12323	'Camk2b'	146.5	167.9	162	101	141	105.4	210	169.5	173.6	131.52
12325	'Camk2g'	84.12	106.4	115	103	88.6	77.4	97.9	99.71	57.77	80.33
12326	'Camk4'	2.93	4.37	1.91	0.75	3.23	2.11	3.87	3.94	2.47	1.67
12328	'Caml'	49.61	48.33	61.4	40.7	78.3	55.64	71.9	48.52	35.9	55.09
12330	'Canx'	207.7	206.3	208	210	168	119.6	181	189	249	291.32
12331	'Cap1'	17.18	19.31	8.37	1.88	23.8	16.42	13.5	16.06	68.54	37.67
12332	'Capg'	0	0	2.32	13.7	11	5.88	0.04	2.71	0	0
12333	'Capn1'	0	0	0	0	0	0	2.85	0.23	0	1.13
12334	'Capn2'	25.39	6.1	7.54	2.74	5.02	10.27	8.32	2.28	19.19	15.39
12336	'Capns1'	32.99	40.25	24.9	39.8	60.7	67.13	28.5	19.89	42.37	35.96
12337	'Capn5'	5.22	5.02	10.7	3.86	16.1	3.88	9.23	2.97	17.51	12.41
12338	'Capn6'	0.53	0.12	0	0.02	0	0	3.78	0	0	0.02
12339	'Capn7'	14.75	9.62	9.6	6.43	8.41	4.47	18.9	18.98	20.23	14.46
12340	'Capza1'	38.21	33.86	45.8	44	33.2	27.53	32.5	45.47	61.95	54.76
12343	'Capza2'	188.5	123.6	158	146	173	170	132	172.7	169.7	173.61
12345	'Capzb'	296	277.3	243	196	239	390.5	215	354.7	244	306.79
12348	'Car11'	20.71	20.82	18.6	26.3	56.2	66.73	33.3	24.68	26.11	6.21
12349	'Car2'	55.1	81.37	9.98	21.3	25.6	144.9	3.26	16.18	15.7	6.47
12351	'Car4'	3.29	7.95	0	0	1.31	0.14	0	0	0	4.62
12352	'Car5a'	0.07	0.12	0.06	0.23	0.29	0.04	0.08	0.16	0.22	0
12354	'Car7'	0	2.05	0	0	2.92	0	3.41	0	0.03	2.99
12355	'Nr1i3'	0	0	0.7	0	0	0	0	0	0	0
12359	'Cat'	10.33	11.01	6.22	12.6	11.9	23.52	25.1	18.41	8.37	3.65
12361	'Cask'	46.71	31.76	19.2	13.9	31.8	39.33	23.8	46.05	43.49	41.19
12362	'Casp1'	0	0	0	0	0.69	0	0	0	0	0
12363	'Casp4'	0	0	0	0	0	0.29	0	0	0	0
12366	'Casp2'	0	3.47	5.71	0	3.05	0.03	0	1.31	5.46	4.48
12367	'Casp3'	5.98	8.19	5.41	4.86	3.4	4.98	4.36	9.15	1.32	2.15
12368	'Casp6'	4.91	2.48	0.66	0.24	5.41	0	0	1.02	0	3.84
12369	'Casp7'	2.22	0	2.41	0	0	5.11	0.02	1.43	1.69	0
12370	'Casp8'	0	0.34	0	0	2.05	6.64	0.58	0.23	0.49	0.16
12371	'Casp9'	0.85	2.49	7.73	0	1.35	0	0	0	2.79	0
12372	'Casq1'	0	0.1	0	0	0	0	0	0	0	1.03
12373	'Casq2'	0.12	0.02	0.32	0.77	0.02	0.11	0.1	0.03	0.12	0.08
12374	'Casr'	0	0.17	1.23	0.14	0.02	0	0	0	0	0
12380	'Cast'	4.87	0.59	1.22	0.63	2.97	3.81	0.24	3.29	7.69	4.57
12385	'Ctnna1'	10.54	8.67	15	35	14.8	7.78	14.3	11.23	1.8	10.34
12386	'Ctnna2'	71.66	35.55	31.4	22.7	28.2	15.27	47.7	45.89	24.71	18.14
12387	'Ctnnb1'	30.32	37.75	27.9	52.7	37.2	19.59	46	35.51	28.49	34.48
12388	'Ctnnd1'	0.85	0.58	3.54	0	1.02	1.84	0.19	1.35	2.17	1.63
12389	'Cav1'	1.32	0	7.67	8.7	3.78	2.56	19.6	0.85	0.02	0.02
12390	'Cav2'	6.17	1.63	9.92	4.28	0	0.08	11.4	3.28	4.07	2.32
12391	'Cav3'	0.04	0	0.09	0.05	0	0	0	0	0	0
12393	'Runx2'	0	0.01	1.01	4.47	0.27	0.01	0.01	0.01	0.01	0
12394	'Runx1'	0.02	0	0	0	0	0	0	0	0	0

12395	'Runx1t1'	1.64	3.88	6.71	1.83	5.58	3.71	1.32	2.56	0.54	4.46
12396	'Cbfa2t2'	15.28	16.15	10.3	8.19	13	13.38	10.9	12.18	11.78	14.29
12398	'Cbfa2t3'	0.01	0.34	0.01	0.13	1.07	0	0	0	0	0.94
12399	'Runx3'	0.01	0	0	0.02	0.02	0	0.01	0.01	0.01	0
12400	'Cbfb'	3.2	7.09	2.25	9.67	3.91	1.44	1.98	8.36	12.5	10.25
12402	'Cbl'	1.52	2.2	6.89	5.46	1.95	0.91	0.09	1.52	2.45	3.33
12404	'Cbln1'	0.02	19.98	4.6	0	1.11	5.04	11.5	0	0.33	3.6
12405	'Cbln2'	16.26	112.8	51.8	130	7.75	51.91	5.47	11.58	305.6	75.39
12406	'Serpinh1'	2.58	0	0.74	0	0.11	0	0	0.52	1.94	1.56
12408	'Cbr1'	146.5	133.5	134	117	184	181.4	198	179.6	134.7	133.61
12411	'Cbs'	2.82	10.71	1.93	16.4	1.83	5.7	13.5	1.31	1.09	1.4
12412	'Cbx1'	32.05	23.06	16.9	32.8	43.1	7.76	25.2	30.94	20.64	33.33
12416	'Cbx2'	0.81	0	0.14	0	0.01	0.29	3.6	0	1.06	0
12417	'Cbx3'	202.3	206	184	329	239	105.2	156	202.5	160.9	190.64
12418	'Cbx4'	3.17	4.87	6.29	9.29	0.16	0.75	1.98	1.72	4.76	2.99
12419	'Cbx5'	110.8	68.51	54.9	73.7	105	76.93	104	100.6	69.22	86.47
12421	'Rb1cc1'	4.91	3.57	4.61	17.3	7.4	2.66	3.96	4.71	8.22	5.86
12424	'Cck'	0	3.19	246	188	62.1	73.22	0	1.06	83.84	96.2
12425	'Cckar'	21.54	5.79	4.36	0	4.55	8.44	1.85	11.24	0.03	0.01
12426	'Cckbr'	3.02	4.38	2.45	0	0	0	4.93	7.94	0.02	1.39
12427	'Ccna1'	0.03	0.31	3.19	0	0	0	0	2.92	5.8	0
12428	'Ccna2'	1.91	2.96	0.05	0.21	0.15	2.37	0.59	0.83	0.32	1.37
12442	'Ccnb2'	2.23	0	2.27	0	0	0	0	0	0	1.74
12443	'Ccnd1'	0.13	17.87	28.4	0	15.1	6.16	16.2	3.89	17.96	29.76
12444	'Ccnd2'	0.03	4.75	5.08	0.04	3.39	1.41	4.61	4.85	4.77	3.95
12445	'Ccnd3'	27.78	30.19	30.2	12.8	23.9	15.4	21.3	8.04	31.15	47.53
12447	'Ccne1'	12.69	8.71	5.25	13.4	5.17	12.77	0.03	17.05	0	3.58
12448	'Ccne2'	3.98	0.15	0.14	6.95	3.56	3.88	0.18	1.58	0.41	0
12449	'Ccnf'	0	2.39	2.73	0	3.98	2.53	0	0.01	1.18	0.01
12450	'Ccng1'	15.45	10.21	8.36	9.78	29.6	4.61	9.16	14.13	13.41	8.86
12452	'Ccng2'	0	5.29	0.27	0.45	1.73	0	0	6.09	4.94	2.23
12453	'Ccni'	6.96	1.95	0.34	17.1	7.79	3.56	0.02	2.11	5.46	5.87
12454	'Ccnk'	2.33	1.93	2.45	0.63	0.06	4.77	5.86	1.97	0.38	2.04
12455	'Ccnt1'	23.78	21.95	18.2	18.2	21.2	14.39	24.7	19.13	14.27	20.51
12457	'Noct'	3.77	0.98	7.66	8.26	4.76	0.38	2.38	2.33	3.16	0.3
12460	'Ccs'	25.09	31.79	57.2	84.1	26.7	26.65	45.4	79.68	48.13	45.55
12461	'Cct2'	67.84	125.5	75	97.9	66.2	90.88	122	81.11	65.32	74.55
12462	'Cct3'	230.8	225.5	193	228	157	231.8	218	164.3	214.6	229.35
12464	'Cct4'	145.4	143.2	106	65	74.7	134.8	88.5	144.3	157.5	157.21
12465	'Cct5'	204.7	200.1	221	213	170	262.5	237	226.6	290.5	267.16
12466	'Cct6a'	155.5	187.3	141	156	136	183.9	198	192	154.8	157.7
12467	'Cct6b'	0	0	0.05	0	0	0	0	0	0	0
12468	'Cct7'	360.8	312.3	294	440	293	382.2	343	298.8	356.7	413.1
12469	'Cct8'	246.1	282.7	266	229	160	255.9	282	258.7	227.6	268.98
12475	'Cd14'	0	0	0	0	3.05	20.37	0	0	0.03	0
12476	'Cd151'	27.27	24.56	21.1	49.6	37.4	37.48	22	29.88	11.56	40.09
12479	'Cd1d1'	0	6.77	0.65	0	0.03	12.38	11.4	4.69	55.75	77.25
12483	'Cd22'	0.02	0.01	0.02	0	0.06	0.03	0	0.02	0	0
12484	'Cd24a'	3.32	50.72	44.5	28.2	1.63	0.03	0.03	21.32	3.94	4.79
12488	'Cd2ap'	1.95	1.25	0.72	0.59	1.91	0.84	0.37	1.89	1.78	1.11
12489	'Cd33'	0	0	0	0	4.65	0.31	0	0	0	0.02
12490	'Cd34'	0	0	0	75.8	0	4.04	4.97	2.73	0	0
12491	'Cd36'	15.2	9.56	0.6	5.67	0.18	4.24	0.18	4.87	0.51	2.66
12492	'Scarb2'	16.88	19.17	14.3	7.19	10.2	8.82	19.1	18.22	22.22	17.66
12493	'Cd37'	0	0	0	0	0	25.75	0	0	0	4.31
12494	'Cd38'	10.11	5.8	0.02	3.97	0.12	2.85	0.02	3.21	3.17	3.04
12495	'Entpd1'	0	0	0	0	1.71	6.46	3.32	1.49	0	1.04
12496	'Entpd2'	4.04	12.51	0	15	3.93	12.81	0	5.02	5.82	3.97

12497	'Entpd6'	78.9	92.95	80.4	53.9	64	70.02	68.5	90.39	102.1	124.44
12499	'Entpd5'	6.77	12.15	6.02	7.46	8.07	6.64	8.77	4.91	11.93	10.01
12503	'Cd247'	0	0	0	0	0.41	0	1	0	0	0.14
12504	'Cd4'	0	0	0	0	0	0	0	0.65	0	0
12505	'Cd44'	0.01	2.04	0	0	0.26	0	0.01	0	0.01	2.66
12507	'Cd5'	0	3.06	0.02	0	0.02	0	7.49	0	0	0.02
12508	'Cd53'	0	0	0.05	0	0	10.14	3.49	0.55	0	2.53
12509	'Cd59a'	31.22	16.24	17.5	39.5	10.5	12.27	8.21	21.36	6.15	9.41
12511	'Cd6'	0	0	0	2.17	0	0	0	0	0	0
12512	'Cd63'	117	91.11	101	141	104	123.1	50.7	65.33	78.27	118.04
12514	'Cd68'	0.94	8.54	3.75	12.4	6.57	15.77	2.26	0.03	0.92	9.03
12515	'Cd69'	0	0	0.09	0	0	0	0	0	0	0
12517	'Cd72'	0	0.04	0.08	0	0	1.27	0	0	0	0
12518	'Cd79a'	0	0	0	0	0	0	2.57	0	0	0
12519	'Cd80'	0	0.08	0	0	0	0.16	0	0	0	0.03
12520	'Cd81'	699.7	688.8	672	681	478	761.3	613	643.8	633	624.73
12521	'Cd82'	17.26	26.39	11.6	13.8	5.08	54.1	0	23.1	12.24	5.38
12522	'Cd83'	66.78	56.5	82	29.7	75.6	115.9	97.4	103.4	103.1	54.67
12523	'Cd84'	0.75	0.38	1.14	0.06	0.64	3.85	1.17	0.4	0.93	1.6
12524	'Cd86'	1.32	0	0	2.17	0	0.02	0	0	0	0
12525	'Cd8a'	0	0	0.4	0	0.03	0	0	0	0.05	0
12526	'Cd8b1'	0.03	0.03	0	0	0.04	0.07	0	0.21	0	0.03
12527	'Cd9'	17.39	18.87	91.3	28.4	8.93	118	86.7	26.64	2.37	6.24
12530	'Cdc25a'	4.18	6.36	1.61	18.5	2.48	4.5	6.98	3.28	5.83	0.12
12531	'Cdc25b'	0.78	0.83	0.63	0	0.56	0	0	3.08	2.52	2.33
12532	'Cdc25c'	0	0	0	0	0	5.2	0.03	0	0	0
12537	'Cdk11b'	13.86	18	34.3	18.3	28.7	22.18	7.34	17.89	20.31	16.88
12539	'Cdc37'	99.76	90.3	110	105	97.3	87.76	82.2	103.6	106.2	91.8
12540	'Cdc42'	438.7	410.2	409	430	300	488	362	392.3	320.8	367.58
12544	'Cdc45'	3.82	0	2.63	0	0	0	4.53	4.48	0	0
12545	'Cdc7'	15.49	29.11	13.2	0	19.2	3.66	31.3	20.52	31.68	13.96
12549	'Arhgap31'	0	0	0.42	0.18	0	0	2.15	0	0	0.04
12550	'Cdh1'	0	0	0.01	0	0	0	0	0	0	0
12552	'Cdh11'	7.08	13.36	14.4	6.33	9.89	8.27	14.4	12.13	12.34	9.12
12554	'Cdh13'	97.15	67.79	93.5	71	73.3	190.8	75.3	92.63	40.26	30.57
12555	'Cdh15'	1.38	5.56	7.57	18	4.31	7.89	1.83	1.94	2.99	4.45
12556	'Cdh16'	0	0.02	0	0.19	0.03	0	0.02	0.08	0.06	0.03
12557	'Cdh17'	0	0	0.01	0	1.9	0.83	0	0	0	0
12558	'Cdh2'	19.9	30.88	24.5	20.1	13.3	11.26	17	11.73	15.74	10.46
12561	'Cdh4'	5.11	7.28	7.31	2.94	0.8	0.01	11.5	4.99	1.53	6.36
12562	'Cdh5'	0	0	0.01	0	0	0	0	0	0	0
12563	'Cdh6'	2.12	1.4	1.64	3.37	2.55	0.15	0.76	4.49	0	0.3
12564	'Cdh8'	9.71	8.93	18.2	1.09	9.98	1.2	21.9	25.36	6.8	3.61
12565	'Cdh9'	2.95	0	0.19	0	1.69	6.14	0.05	1.37	0.01	12.1
12566	'Cdk2'	0	0	0	0	0	0	4.69	0	0	0
12567	'Cdk4'	53.06	65.4	53.2	40.8	82.1	55.02	86.4	100.4	51.02	48.71
12568	'Cdk5'	144.8	118.3	130	157	103	169.2	110	182.2	93.62	90.38
12569	'Cdk5r1'	3.74	1.24	4.33	6.87	7.4	4.29	2.75	2.77	8.28	3.27
12570	'Cdk5r2'	3.9	1.08	1.27	3.93	4.81	0.87	1.95	3.99	2.6	3.2
12571	'Cdk6'	0	0.35	0	0.01	0.01	0.73	0	0.01	0.01	0.01
12572	'Cdk7'	5.89	4.59	6.75	2.77	6.55	5.13	0.96	6.23	5.8	7.05
12575	'Cdkn1a'	2.08	3.8	5.45	24	2.25	10.44	0.34	1.53	13.25	13.7
12576	'Cdkn1b'	9.59	8.06	6.29	7.19	9.67	7.54	2.55	4.25	12.69	11.45
12577	'Cdkn1c'	0	0	0	0	0.93	0	1.21	0	0	0
12580	'Cdkn2c'	0	0	0	0	0.23	0	0	0	0	0
12581	'Cdkn2d'	166.6	153.6	132	136	163	235.9	136	186.8	164.1	130.95
12583	'Cdo1'	34.85	36.51	68.7	33.5	63.2	34.89	47.4	34.67	23.42	22.84
12585	'Cdr2'	3.89	1.32	0	0	2.04	0	0	0	3.41	0

12587	'Mia'	0	1.85	0.09	0	0	0	7.52	2.18	0	0.5
12589	'Ift81'	22.71	24.67	23.4	37.9	32.9	35.15	18.9	26.2	25.35	23.16
12593	'Cdyl'	0	0	0	0	0	0	0.23	1.1	0.54	0
12606	'Cebpa'	0	0	0	0	0	0.34	0	0	0	0
12607	'Cebpz'	8.21	10.2	6.75	12.9	5.99	5.77	10.3	8.86	5.73	5.56
12608	'Cebpb'	0.18	0.13	0	9.44	0	0	0	0	0	0.06
12609	'Cebpd'	0.65	1.56	1.47	8.72	1.09	0	0	0	0	0
12611	'Cebpg'	1.12	2.56	1.29	4.6	3.01	1.66	0.05	0.84	0.65	2.48
12614	'Celsr1'	0.04	0.1	0.07	0	0.05	1.32	0	0	0.03	0.07
12616	'Cenpb'	2.06	0	2.25	1	4.98	1.87	2.36	0	2.03	0.02
12617	'Cenpc1'	11.62	16.8	13.1	9.32	15	12.56	11.7	15.6	13.21	14.17
12626	'Cetn3'	120.3	98.13	114	158	114	100.5	59	110	121.6	97.18
12628	'Cfh'	0	1.26	0	0	0	3.74	0	1.34	0	1.27
12631	'Cfl1'	1455	1425	1276	1794	1487	1676	1465	1543	1192	1270.66
12632	'Cfl2'	51.86	42.64	54.5	29.1	42.4	38.57	60.6	52.43	69.68	41.65
12633	'Cflar'	4.95	7.36	5.93	15.5	8.55	5.93	4.1	3.66	5.45	3.6
12638	'Cftr'	0	0.14	0.35	0	0.64	0.02	0	0.09	1.14	0.87
12642	'Ch25h'	0.07	0	0	0	0	0	6.66	3.8	2.3	0
12647	'Chat'	0	0	0	0	0.38	0	0	0	0	0.02
12648	'Chd1'	0.93	1.65	0.12	3.24	0.64	0.68	0.72	0.12	1.29	0.45
12649	'Chek1'	0.32	0.61	0.01	5.58	0.21	0.01	0.94	0	1.24	0
12651	'Chkb'	29.17	36.7	16.9	27.5	36.7	41.8	52.6	57.79	22.05	30.94
12652	'Chga'	107.2	159.8	182	23.7	195	105.5	113	75.31	99.17	105.54
12653	'Chgb'	175.6	163.7	139	158	123	156	99.7	162	203.4	249.03
12654	'Chil1'	3.89	1.24	0	0	0	1.74	18.1	10.59	0	0
12659	'Ovgp1'	3.71	31	5.29	4.17	4.38	11.01	12.4	3.71	3.51	8.12
12660	'Chka'	25.6	35.58	17.2	23.1	13.8	32.56	42.8	17.56	21.72	9.31
12661	'Chl1'	33.42	18.9	13	17.3	8.05	16.25	22.1	15.22	8.06	17.54
12662	'Chm'	7.16	4.54	3.5	4.03	5.13	3.59	3.7	4.61	5.5	6.2
12663	'Chml'	0.69	0.1	0	1.57	0	0.02	0.53	1.3	0.94	1.2
12667	'Chrd'	0.51	2.18	3.11	0	0.55	0	5.41	2.93	4.59	0
12669	'Chrm1'	0.34	1.34	3.97	2.6	0.72	0.01	0.03	2.92	0.01	0.03
12671	'Chrm3'	0.98	0.2	1.53	0.08	0.47	0.09	0.25	0.01	0	0.71
12672	'Chrm4'	1.47	0.56	0.05	0.1	0.02	0	0	0	0	0
12675	'Chuk'	5.38	6.44	1.57	13.8	7.29	4.36	4.51	10.09	15.41	7.5
12677	'Vsx2'	0	0	0	0	2.71	0	0	0	0	0
12683	'Cidea'	12.5	20.79	13.2	21.1	0	18.06	0.04	1.24	26.06	21.7
12684	'Cideb'	0.04	3.14	0	0	0	2.1	0	0	5.37	0
12686	'Elovl3'	0	0	2.41	0	0	0	0	0	0	0.02
12695	'Patj'	0.26	0.61	10.3	2.12	15.4	17.9	3.45	1.97	0.06	3.93
12696	'Cirbp'	171.3	220.1	339	368	317	284.3	217	275.8	236.5	268.59
12700	'Cish'	0.07	0	9.75	0.02	0.02	1.65	0	6.33	0.78	4.18
12702	'Socs3'	2.37	33.61	54.3	96.3	0.05	10.7	45.3	7.06	7.78	46.31
12703	'Socs1'	3.58	10.51	7.14	38.6	0	3.3	0	1.91	2.31	0.93
12704	'Cit'	22.01	22.13	24	39.6	30.5	24.8	23	22.2	26.35	21.88
12705	'Cited1'	58.2	55.1	37.2	44.1	33.5	39.87	8.2	110.5	128.6	29.61
12709	'Ckb'	1446	1151	1406	918	1100	1536	1293	1207	990.9	1003.34
12715	'Ckm'	0.51	0.07	1.93	0.45	0.25	0.62	0.5	0.2	0.89	0.4
12716	'Ckmt1'	106.4	126	189	126	168	89.52	189	181.6	137.3	102.53
12721	'Coro1a'	36.34	49.73	55.6	50.9	52.3	83.46	64.9	70.13	70.61	64.59
12722	'Clca3a1'	1.63	7.99	4.22	15.6	2.44	0.92	1.76	5.48	0.92	0.63
12723	'Clcn1'	0	0.08	0	0	0	0	2.33	0	0	0.09
12724	'Clcn2'	4.36	4.35	0.25	3.33	6.74	0.23	12.2	6.53	3.98	0.16
12725	'Clcn3'	44.99	46.6	52.2	29	32.3	17.06	38.7	38.1	33.11	35.36
12727	'Clcn4'	53.52	50.25	60.1	37.3	48.8	55.73	85.6	50.32	57.92	61.07
12728	'Clcn5'	2	3.63	5.47	0.64	3.28	1.26	2.29	6.01	1.33	2.63
12729	'Clns1a'	71.92	69.93	44.7	60.3	53	98.91	96.1	85.77	62.97	63.22
12733	'Clcnka'	0.07	0	0	0	0	0	0	0	0	0

12737	'Cldn1'	1.06	0	0	0	0	0.01	4.18	0	0	0
12738	'Cldn2'	0	0.76	0	0	0	0	0	0	0	0
12739	'Cldn3'	0	0	0	0	12.5	0	12.4	0	0	2.11
12741	'Cldn5'	0	0	0	0	0	0	0	0	0.49	0
12745	'Clgn'	0	2.87	6.01	0.14	3.98	0	4.44	1.49	0	0.44
12747	'Clk1'	92.96	63.32	78.1	98.9	56.3	72.14	82.5	85.13	57.4	75.68
12748	'Clk2'	3.38	0.06	0	0.27	4.29	1.82	0	3.12	0.27	2.53
12750	'Clk4'	54.85	50.77	51.8	20.1	52.3	31.36	41.8	29.04	20.62	57.87
12751	'Tpp1'	18.16	21.45	21.6	9	23	25.15	33.1	14.99	8.02	17.92
12752	'Cln3'	4.69	12.44	9.86	6.15	4.55	10.67	17.3	11.52	0.34	4.19
12753	'Clock'	1.85	1.14	1.03	3.81	1.55	1.38	1.46	1.43	1.23	2.74
12757	'Clta'	171.8	195	218	169	132	252.9	151	175.5	156.9	118.01
12759	'Clu'	419.7	538.2	470	676	318	516.8	363	419.6	540.7	519.2
12763	'Cmah'	1.07	1.84	1.13	2.01	1.42	0.61	1.08	0.65	1.13	1.08
12764	'Cmas'	32.28	21.04	18.7	27.5	17	38.57	51.7	15.13	28.9	20.5
12765	'Cxcr2'	1.66	1.62	1.49	4.02	2.6	2.45	2.39	0.76	2.69	1.61
12767	'Cxcr4'	0	0	0	0	2.41	0	0	0	0	0
12769	'Ccr9'	0	0	0	0.01	0	0.01	0	0	0	0
12773	'Ccr4'	0	0	0	0	0	0.02	0	0	0.02	0
12777	'Ccr10'	0.64	0	0	0.03	0	0	5.03	2.5	0.03	3.16
12778	'Ackr3'	0.02	3.33	3.17	0.03	0	0.43	0	8.68	0	0
12780	'Abcc2'	0.01	0	0.13	0.85	0	0	0	0	0	0
12785	'Cnbp'	412.9	534.6	392	526	367	515.1	440	474.6	399.3	424.66
12789	'Cnga2'	0	0	0	0	0	0.51	0	0	0	0
12790	'Cnga3'	3.11	6.87	2.69	8.43	4.22	14.76	5.56	0.17	0.53	25.42
12793	'Cnih1'	83.4	112.4	62.3	52.5	76.7	110.7	63.9	91.13	76.01	114.95
12794	'Cnih2'	23.42	20.26	14.7	31	36.1	21.44	22.2	20.33	29.36	14.27
12795	'Plk3'	3.06	1.33	6.75	20.9	0	2.41	6.86	0.02	4.69	0
12797	'Cnn1'	1.42	0	0	0	3.86	0	0	0	0	0
12798	'Cnn2'	0.02	0	8.63	11.5	0	0	0.03	0.02	0	0
12799	'Cnp'	90.8	107.8	97	23.3	87.2	297.8	23.8	86.09	39.38	50.79
12801	'Cnr1'	5.17	5.91	12.8	2.07	8.75	8.57	19.3	13.59	19.53	22.33
12802	'Cnr2'	0.22	0.24	0.27	1.05	0.27	1.41	0.21	0.18	0.3	0.39
12803	'Cntf'	0	1.27	0	0	0.13	0	0	0	0	0
12804	'Cntfr'	14.24	11.1	17.5	6.84	24	21.53	17	11.81	39.58	16.62
12805	'Cntn1'	83.13	46.2	104	60.5	61.6	46.28	100	78.97	66.46	82.91
12807	'Hps3'	0.58	2.04	7.91	2.34	0.51	0.79	3.21	3.46	2.25	4.96
12808	'Cobl'	2.48	2.41	5.62	4.14	3.33	2.14	2.18	4.72	1.57	3.45
12810	'Coch'	45.24	17.18	2.27	0.73	3.87	13.32	22.5	32.32	3.91	0.22
12812	'Coil'	1.73	1.36	3.87	4.75	5.43	15.23	8.59	11.01	8.2	0.54
12813	'Col10a1'	0	0	0	0	0.02	0	1.11	0	0	0
12814	'Col11a1'	0.48	1.44	10.6	6.72	1.95	6.93	2.75	1.09	1.46	1.28
12815	'Col11a2'	0.63	0.47	1.76	0.01	2.4	2.27	1.78	0.01	0	0.01
12816	'Col12a1'	0.13	0.62	0	0	0.58	0	0.73	0.28	0.16	0.02
12819	'Col15a1'	0.03	0	0.08	0	0.27	0.01	0.38	0.07	0.4	0.64
12821	'Col17a1'	0	0	0.31	0	0	0	0	1.15	0	0
12822	'Col18a1'	4.93	14.3	0.02	7.01	9.98	2.48	3.47	2.65	4.5	11.42
12823	'Col19a1'	0.01	0.72	4.78	2.35	0.69	0.26	0.44	1.86	0.61	0.57
12824	'Col2a1'	0	1.12	1.66	1.28	0	0	0	0.15	0.78	4.11
12825	'Col3a1'	0	0	0	0	0	0	0	0	0.24	0
12826	'Col4a1'	0.74	2.07	3.47	11.4	0.37	1.9	1.33	1.41	2.27	0.4
12827	'Col4a2'	4.84	2.84	3.89	2.87	8.3	2.59	7.56	3.13	2.81	4.04
12828	'Col4a3'	3.19	0	0	0	0.01	1.01	0.42	0	8.06	0
12829	'Col4a4'	2.55	0	0	0.47	0	0	0	0	1.77	0
12830	'Col4a5'	1.55	0.17	0.08	3.04	0.13	0	0	0	0.27	0
12831	'Col5a1'	0	1.46	0	0	4.84	0.01	1.21	0.56	0	0
12832	'Col5a2'	0	0	0	0	0	0.12	0	0	0	0
12833	'Col6a1'	0	1.86	0	0	0	0	0	0	0.82	0

12834	'Col6a2'	0	4.36	0	0	2.67	0.29	0	0	0.01	0
12835	'Col6a3'	0	0	0.16	0	0	0	0	0.59	0	0
12836	'Col7a1'	0	0.19	0.03	0.16	0.14	0	1.37	0.48	0	0.16
12837	'Col8a1'	0	0	0.06	0	0	0	0	0.95	0	0
12839	'Col9a1'	0	1.14	2.24	1.46	7.1	3.07	0	1.54	0	2.01
12840	'Col9a2'	2.85	2.85	11.4	8.11	6.78	5.73	4.94	4.42	11.01	4.59
12841	'Col9a3'	6.16	4.91	6.11	0	1.84	3.76	6.75	2.07	3.15	3.38
12842	'Col1a1'	0	0.02	0	0	0	0	0	0	0.06	0
12843	'Col1a2'	0	0	0	0	0.02	0	2.63	0	0	0.87
12845	'Comp'	0	0.1	0	0	0	0	0.29	0.02	0	0
12846	'Comt'	93.43	70.94	84.5	34.5	69.8	133.2	83.9	84.91	75.35	83.23
12847	'Copa'	26.18	47.81	45.3	62.5	32.4	32.12	15.8	31.01	41.71	37.09
12848	'Cops2'	26.53	41.73	26.9	30.7	32.1	17.92	27.7	30.35	19.62	40.07
12850	'Coq7'	105	112.1	206	212	155	173.4	133	183.2	132.1	156.57
12854	'Cort'	0	0	1.07	0	0	0	0	0	0	0
12856	'Cox17'	209.3	277.1	302	455	150	331	228	125.9	370.6	175.59
12857	'Cox4i1'	1452	1159	1344	1278	1703	1990	1741	1423	1262	1691.11
12858	'Cox5a'	617	484.7	662	561	565	813.4	682	663	560	515.39
12859	'Cox5b'	722.4	648.7	985	606	837	1015	893	681.7	739.8	608.93
12861	'Cox6a1'	818.3	674.2	893	839	818	981.6	890	674.7	742.1	688.26
12862	'Cox6a2'	2.75	0.7	16.2	31	5.68	22.35	2.94	13.85	5.79	3.85
12864	'Cox6c'	572	513.5	627	616	513	853.5	565	353.2	463.1	455.59
12865	'Cox7a1'	4.61	0.31	11.9	2.6	3.77	9.93	8.97	4.94	2.53	3.06
12866	'Cox7a2'	405.1	363.1	508	448	397	502.6	430	301.9	472.4	382.36
12867	'Cox7c'	362.8	442.1	472	459	350	536.9	449	223.9	462.4	320.08
12868	'Cox8a'	1286	1202	1358	1260	1124	1707	1441	984.1	1087	1184.36
12869	'Cox8b'	1.6	0.62	6.55	2.44	1.11	2.89	2.75	1.05	1	1.83
12870	'Cp'	0.01	0.11	4.47	0.25	0.25	0	5.82	1.13	0	0
12874	'Cpd'	0.72	0.36	1.41	0.12	0.34	0.52	1.44	0.44	0.69	0.34
12876	'Cpe'	257.3	222.2	142	594	169	208.5	194	213.9	428.8	156.55
12877	'Cpeb1'	18.36	12.7	23.4	5.4	13.9	19.87	21	24.84	11.06	14.16
12879	'Cys1'	2.01	0	0.25	0.26	2.04	2.19	0.4	0	0	0
12889	'Cplx1'	52.65	36.03	295	14.9	154	33.74	136	176.9	23.55	17.51
12890	'Cplx2'	33.68	33.89	49.9	35.3	64.1	24.37	29.3	56.04	48.88	28.15
12891	'Cpne6'	131.8	95.64	120	128	116	90.07	99.3	178.8	105.4	112.16
12892	'Cpox'	2.4	2.23	0.57	3.36	7.2	1.48	2.75	2.07	6.11	2.27
12894	'Cpt1a'	0.01	4.51	0	0	0.05	0	0	0.25	0.01	0
12895	'Cpt1b'	0.78	10.48	0.16	0.21	0.05	0.05	4.08	0.03	0.01	0.09
12896	'Cpt2'	18.54	7.64	12	26.5	18.9	17.49	1.74	7.85	8.29	21.39
12902	'Cr2'	0	0	0	0.03	0.02	0	0	0	0.01	0
12903	'Crabp1'	0	6.31	0	0	3.68	0	0	2.64	0	0
12904	'Crabp2'	0	0	0	0	0	0	5.57	0	5.15	0
12905	'Cradd'	6.15	11.49	12.7	2.08	3.08	25.7	8.81	14.91	5.24	12.51
12908	'Crat'	7.24	2.03	9.04	3.52	4.57	16.52	25.2	10.91	3.99	5.29
12909	'Crcp'	43.29	47	45	31.2	83.7	89.89	64.5	71.28	32.52	85.41
12912	'Creb1'	2.79	2.15	4.04	2.33	1.75	4.92	1.57	2.74	2.45	2.69
12913	'Creb3'	93.79	82.83	122	159	64.3	106.9	94.1	101.3	138.8	158.51
12914	'Crebbp'	8.15	8.15	6.94	14.8	10.9	4.95	7.2	5.02	5.96	6.63
12915	'Atf6b'	12.74	16.29	20.4	6.97	14.2	5.88	11	5.84	11.77	23.72
12916	'Crem'	16.29	15.37	2.46	10.1	11.3	4.92	2.83	7.53	34.18	39.71
12918	'Crh'	0	7.79	0.37	7.45	0.15	0	131	16.7	2.75	0
12919	'Crhbp'	0.03	5.42	0.03	0	15.8	28.25	1.26	0.02	3.4	25.9
12921	'Crhr1'	0	1.51	2.07	2.68	1.39	0	3.92	0.03	0	0
12922	'Crhr2'	95.98	120.6	86.8	50	0	0	0	0	0	0
12925	'Crip1'	3.39	4	0.49	16.8	14.4	10.61	0	1.84	0	2.73
12927	'Bcar1'	5.49	10.4	5.63	21	2.56	0	3.8	9.45	0	11.21
12928	'Crk'	7.74	4.49	4.26	10.5	7.83	8.25	13.9	4.53	9.37	11.25
12929	'Crkl'	10.69	13.43	25.5	12.7	10.9	11.94	26.2	11.1	10.73	27.23

12931	'Crlf1'	0	0	3.05	0	0	0	0	0	0	0
12933	'Crmp1'	70.36	75.43	63.3	114	65.7	29.67	79.3	61.94	41.11	34.94
12934	'Dpysl2'	627.7	545.7	406	365	599	571	369	566.1	478.2	540.46
12936	'Pcdha4'	0.68	0.23	2.31	0.1	0	0	2.93	1.38	1.69	0.35
12937	'Pcdha6'	0	0.12	0.26	0	0.22	0	0	0	1.37	0
12939	'Pcdha7'	2.39	0.26	0	0	0	1.11	0	0.23	0	0
12941	'Pcdha5'	0.38	1.77	0.03	0	4.59	0.24	11.3	1.13	1.55	1.61
12942	'Pcdha11'	1.82	1.4	1.08	0	8.93	0.37	0.84	0.96	5.2	1.08
12943	'Pcdha10'	0.87	1.85	1.34	0	2.03	0.36	0	0.03	2.8	3.3
12946	'Cr1l'	18.23	19.2	29.6	23.3	8.03	15.12	20.5	13.29	12.8	27.62
12950	'Hapln1'	0	0	0.94	0.01	0	1.41	0	0	0	0
12951	'Crx'	0.06	0.09	0.16	0.2	0.07	0.08	0.1	0.04	0.12	0.04
12952	'Cry1'	9.78	11.36	12.6	20	11.4	1.83	25.8	18	12.55	5.16
12953	'Cry2'	26.1	32.81	41.4	44.3	16.2	14.56	44.1	27.76	15.96	10.81
12955	'Cryab'	62.5	100.6	54.8	31.1	161	222	73.7	38.86	19.04	22.69
12959	'Cryba4'	0	0	4.2	0	0	0	0	0	0	0
12960	'Crybb1'	0	2.81	1.89	0	4.46	0.22	3.94	0	0	8.53
12962	'Crybb3'	0.06	6.6	0.1	2.3	0	0.33	0	1.33	0.06	0
12964	'Cryga'	1.17	0	0	0	0	0	0	0	0	2.95
12971	'Crym'	8.44	0	0	0	10	0	0	0	0	4.66
12972	'Cryz'	23.71	5.82	6.61	5.43	4.23	10.89	11.1	5.43	3.77	4.95
12974	'Cs'	89.21	88.36	114	55.7	67.1	79.12	94	134.3	92.41	59.71
12977	'Csf1'	5.75	1.81	2.73	1.46	1.46	1.24	5.4	1.2	0.08	2.01
12978	'Csf1r'	0.47	0.17	1.15	0	4.08	61.79	8.84	1.66	1.89	2.3
12982	'Csf2ra'	6.84	12.01	10.9	8.42	9.28	3.34	0.62	1.94	5.3	20.77
12983	'Csf2rb'	0	0	0.01	0	0	0	0	0	0	0
12984	'Csf2rb2'	0.01	0	0.01	0.08	0.03	0.01	0.01	0.01	0.01	0.04
12985	'Csf3'	0	0	0	6.73	0	0	0	0	0	0
12986	'Csf3r'	2.96	0.68	0.77	1.26	3.29	8.63	0.37	0.23	0.74	0.66
12988	'Csk'	0.25	6.24	4.57	2.68	1.66	1.84	5.35	2.96	0.02	0.8
12995	'Csnk2a1'	68.49	61.07	52.3	39.3	62.8	46.16	29.9	60.79	58.03	54.85
13000	'Csnk2a2'	1.44	0.44	5.02	6.56	2.55	4.1	1.63	0.29	4.48	6.22
13001	'Csnk2b'	133.3	101.1	164	185	66.8	113.6	81.8	110.8	94.99	64.41
13002	'Dnajc5'	91.96	73.38	114	51.7	63.1	45.14	128	102.3	111.1	81.89
13003	'Vcan'	0.02	1.53	4.76	2.94	2.5	0.14	9.21	0.76	0	2.7
13004	'Ncan'	20.95	33.23	37.9	37.2	16.9	18.82	23.2	27.86	23.26	17.16
13006	'Smc3'	19.56	31.97	16.5	13.9	28.6	23.04	22.4	14.86	19.91	23.53
13007	'Csrp1'	13.25	8.6	2.35	29.7	3.41	14.98	9.62	15.06	4.73	9.55
13008	'Csrp2'	12.02	22.52	11.9	40.6	15.4	0.05	13.2	13.94	5.98	2.57
13009	'Csrp3'	1.75	0.28	5.43	0.8	1.14	1.71	1.81	3	1.21	4.68
13010	'Cst3'	2944	2478	1784	3554	1719	5485	1948	1576	2321	2494.52
13011	'Cst7'	0	0	0	0	0	0	0.16	0	0	0
13013	'Cst9'	0	0	0	0	0	0	15.4	0	0	0
13014	'Cstb'	103.8	102.7	140	251	104	185.8	165	83.27	154.7	168.42
13016	'Ctbp1'	20.44	22.64	9.44	21.1	21	23.02	52.9	19.67	24.2	23.48
13017	'Ctbp2'	6.78	9.39	2.08	13.4	4.74	10.19	3.37	6.67	7.01	8.85
13018	'Ctcf'	3.52	4.11	1.67	7.3	6.06	2.48	10.1	4.11	0.23	1.19
13024	'Ctla2a'	6.57	5.28	7.19	9.84	10.4	6.5	6.17	3.7	9.36	4.67
13025	'Ctla2b'	0	0	0	12.5	0	0	0	0	0	0
13026	'Pcyt1a'	2.83	3.97	6.27	4.18	4.16	0.35	2.84	2.09	5.24	1.41
13030	'Ctsb'	591.5	587.5	695	501	616	737.8	696	573.9	779.5	679.88
13032	'Ctsc'	3.7	0	0	0	3.05	2.29	0.01	0	1.79	0.08
13033	'Ctsd'	268.5	350.1	403	258	296	699.5	294	354.4	438.3	513.94
13036	'Ctsh'	0.35	4.87	4.19	0.04	11.9	3.47	10.2	0.38	0.36	4.64
13038	'Ctsk'	0.22	1.93	0	0	3.83	0.22	0	1.59	0	0
13039	'Ctsl'	142.2	127.2	108	173	149	186.9	129	128.7	201.3	174.24
13040	'Ctss'	25.14	22.83	13.5	69.3	62.3	445.6	45	12.1	17.63	14.98
13041	'Ctsw'	0	0	1.05	0	0	0	0	0	3.91	0

13043	'Cttn'	54.05	55.97	66.4	85.3	61.5	59.04	75.6	69.71	73.71	74.1
13046	'Celf1'	11	9.74	9.37	11	11.3	2.02	6.85	7.39	9.57	6.26
13047	'Cux1'	13.37	4.85	3.93	11.6	10	9.8	12.8	2.31	25.66	8.02
13048	'Cux2'	6.03	2.81	7.76	7.56	6.09	5.33	14.1	5.87	10.16	4.66
13051	'Cx3cr1'	0	2.01	0.03	0	0.21	17.12	11	0	2.73	1.6
13052	'Cxadr'	0.52	0	5.71	4.42	0.1	4.62	8.89	1.02	1.6	1.35
13056	'Cyb561'	85.87	107.7	95.3	85.1	93.9	105.5	100	72.95	129.1	195.54
13057	'Cyba'	4.83	6.9	11.6	22.3	0.08	10.67	0	4.49	0	0.12
13058	'Cybb'	0	0	0	0	0	0	0	0	0	0.03
13063	'Cycs'	128.4	119.8	142	147	158	144.6	113	150.6	139.4	144.16
13067	'Cyct'	0	0	0	11.5	0	0	0	0	0	0
13070	'Cyp11a1'	1.82	0.38	0	0	4.95	0.33	0	3.84	0	0.15
13075	'Cyp19a1'	0.02	4.87	0	0.17	0	0.2	0.02	0.03	0	0
13076	'Cyp1a1'	0	0	0.41	0	0	0	0	0	0	0
13078	'Cyp1b1'	0	1	0	0	0	0	0.09	0	0	0
13079	'Cyp21a1'	0	0	0	4.25	0	0.02	0	0	0	0
13087	'Cyp2a5'	0	2	0	0	0	0	0	0.02	0	0.13
13089	'Cyp2b13'	0	0	0	0	4.31	0	0	0	0	0
13090	'Cyp2b19'	0	0	0	0	0	0.36	0	0	0.16	0
13101	'Cyp2d10'	0.06	0	0	0	0	0	0	0	0	0
13105	'Cyp2d9'	0.11	0	0	0	0	0	0	0	0	0
13106	'Cyp2e1'	0.4	0.38	0.92	0.83	0.84	1.02	0.89	0.32	0.37	0.41
13109	'Cyp2j5'	0	0	0	0.03	0	0.01	0	0	0	0
13110	'Cyp2j6'	3.49	5.95	3.41	7.13	0.07	5.64	11	2.1	0.04	1.48
13116	'Cyp46a1'	73.05	71.3	117	53.4	98.5	131	131	125.7	96.42	78.74
13120	'Cyp4b1'	0	0	0	0	0	0.02	0	0	0.02	0
13121	'Cyp51'	26.86	11.1	30	30.4	10.9	21.96	15.8	30.25	17.51	34.43
13122	'Cyp7a1'	0	0.19	0	0	0	0	0	0	0	0
13123	'Cyp7b1'	1.38	2.08	0	0	3.91	5.01	1.64	1.42	4.86	5.4
13131	'Dab1'	29.61	12.73	36.5	20.8	14.3	12.6	18	10.48	13.72	14.85
13132	'Dab2'	4.14	1.91	3.43	0.13	0	0.38	2.42	0.77	2.04	0
13134	'Dach1'	0.44	0	0	0	0	0	0	0	0.12	1.27
13135	'Dad1'	458.1	465.3	326	632	333	389.3	357	444.8	355.2	488.76
13136	'Cd55'	5.44	3.49	3.28	1	4.64	12.73	0.25	13.26	10.3	12.11
13138	'Dag1'	9.97	2.27	12.8	2.95	4.53	1.8	4.44	3.17	7.27	2.59
13139	'Dgka'	3.36	6.37	8.11	0.02	3.75	9.5	0	5.11	3.81	16.06
13142	'Dao'	0	0.19	0	0	0	2.78	0	0	0	0
13143	'Dapk2'	0	0	0	0	3.56	0	0.04	0	0	0
13144	'Dapk3'	4.45	4.93	3.3	14.4	4.29	0.93	3.3	0.13	1.01	0.42
13163	'Daxx'	21.38	3.83	12.1	13.9	13.2	4.41	38.4	15.08	10.38	20.32
13164	'Dazl'	0	0	0	0	0	0	0	0.6	0	0
13166	'Dbh'	1.96	0	0.02	0	1.03	0	0	0	0	7.89
13167	'Dbi'	442.4	302.4	503	353	378	570	434	248.6	254.2	213.18
13168	'Dbil5'	0	1.46	0.91	0	2.14	0	0	0	2.14	1.06
13169	'Dbnl'	81.61	93.35	60.7	38.5	67.1	80.54	87.5	69.68	113.4	77.28
13170	'Dbp'	7.57	4.16	1.59	25.8	17.5	16.53	18.4	15.39	6.93	15.55
13171	'Dbt'	4.49	4.91	8.98	7.59	0	3.04	1.89	0.73	4.94	5.96
13175	'Dclk1'	29.3	29.23	33.5	20.4	30.4	27.3	31.2	26.42	38.37	22.67
13176	'Dcc'	1.41	1.09	3.16	0.86	2.31	0.13	0.83	2.11	0.67	3.36
13177	'Eci1'	26.29	20.68	19.1	4.37	30.3	41.26	26.5	17.48	12.6	9.12
13178	'Dck'	16.7	17.31	9.46	0	5.63	10.04	4.13	16.02	7.05	8.56
13179	'Dcn'	0	0	0	0	3.04	0	0	0	0	0
13180	'Pcbd1'	222.6	150	203	224	151	210.2	49.4	227.1	132.5	242.29
13185	'Vps26c'	38.45	30.3	30	44.6	30.6	51.66	59	60.94	30.1	36.93
13190	'Dct'	0	0	0.02	0	8.89	2.13	3.55	0	0	0
13191	'Dctn1'	89.92	80.91	108	33	76.3	61.17	120	70.93	102.4	86.08
13193	'Dcx'	2.7	1.09	1.22	1.16	2.22	1.46	1.44	0.5	1.15	1.1
13194	'Ddb1'	52.43	47.14	89.7	24.1	35.8	19.28	67.2	40.33	83.99	80.54

13195	'Ddc'	117.7	81.17	29.8	94.9	31.2	12.03	4.26	12.82	16.34	7.03
13196	'Asap1'	5.59	3.94	7.57	5.71	9.15	2.19	9.84	4.5	7.45	6.85
13197	'Gadd45a'	4.91	11.64	6.47	12.8	6.58	7.43	4.56	4.87	4.49	3.5
13198	'Ddit3'	24.85	27.43	48.7	39.5	58.9	86.25	55.4	25.88	90.38	89.98
13199	'Ddn'	0.67	0.86	1.05	1.73	4.38	0.92	0.01	0.57	0	0
13200	'Ddost'	138.7	274.6	195	206	155	195.2	147	188.9	263.6	263.16
13202	'Ddt'	86.14	58.82	60.4	26.1	62.3	73.89	64.5	58.31	121.5	74.89
13204	'Dhx15'	24.76	16.31	20.8	13.1	14.8	5.34	24.8	14.75	13.45	30.98
13205	'Ddx3x'	22.4	12.64	31.7	45.8	12.1	9.69	16.7	14.03	16.86	10.06
13206	'Ddx4'	0	2.27	2.77	0	0	0	0	0	0	0.02
13207	'Ddx5'	388.9	512.4	356	534	278	160.8	412	320.6	261.4	448.03
13209	'Ddx6'	29.66	21.53	22.3	11	41.4	29.37	30.8	38.14	17.23	26.17
13211	'Dhx9'	7.31	5.7	5.37	5.11	10.4	6.82	2.17	4.78	2.42	3.72
13214	'Defb1'	0	0	0	0	0	0	35.1	0	0	0
13218	'Defa29'	0	0	0	0	0	0	6.37	4.07	0	0
13237	'Defa3'	0	0	0.13	0	0	0	0	0	0	0
13244	'Degs1'	106.7	70.73	50.3	39.9	63.8	98.39	92.8	103	76.35	109.55
13340	'Slc29a2'	5.48	2.44	7.3	8.73	1.85	9.74	3.47	8.08	19.09	5.99
13345	'Twist2'	0	6.85	0	0	0.75	5.27	0	0	2.85	0
13346	'Des'	0.1	0	0.22	0	0.05	0.03	0	0.05	0.07	0.04
13347	'Dffa'	4.17	7.92	24	18.3	9.07	10.33	5.69	15.2	14.54	14.37
13349	'Ackr1'	7.36	0.17	0.6	8.36	13	0.2	21.7	4.09	9.46	10.83
13350	'Dgat1'	7.99	20.33	26.9	4.77	20.2	2.75	29.3	14.03	18.01	2.94
13353	'Dgcr6'	63.73	97.21	50.6	51	89.5	126.5	62.1	87.34	90.3	80.53
13356	'Dgcr2'	91.69	103.1	127	72.5	71.8	115	117	105.8	111.7	100.05
13358	'Slc25a1'	80.25	92.83	90.4	74.2	75.3	98.25	113	45.45	87.72	78.39
13360	'Dhcr7'	18.94	28.31	44.5	18.3	13.7	33.59	23.5	23.04	20.85	14.57
13361	'Dhfr'	0.66	0.51	0.01	0	1.05	2.39	0	0.83	1.4	0
13363	'Dhh'	0	0	0	0	0.42	0	0	0	0	0
13367	'Diaph1'	1.9	4.57	2.5	1.51	0.58	3.59	3.28	2.36	2.12	3.62
13368	'Dffb'	0.27	12.25	2.47	22.1	0	4.75	9.18	3.89	11.68	1.01
13370	'Dio1'	0	0	0	0	0	0	0	0.05	0	0
13371	'Dio2'	0.17	0.01	0	1.42	0	0	0	0	0	0.98
13382	'Dld'	33.91	76.19	32.4	43.4	34.4	51.75	24.7	59.74	38.45	44.67
13383	'Dlg1'	11.32	12.41	18.7	17.8	8.31	4.49	10.6	7.66	9.62	13.62
13384	'Mpp3'	8.59	22.41	19.7	9.66	10.7	17.11	31.2	18.07	34.88	43.8
13385	'Dlg4'	14.51	9.38	6.88	5.36	16	10.58	3.9	10.72	25.36	21.82
13386	'Dlk1'	20.92	18.49	0.33	6.22	30.6	29.39	8.24	12.46	18.49	51.22
13388	'Dll1'	3.97	2.05	10.5	3.59	0	0.45	7.21	0	6.07	4.72
13389	'Dll3'	0.33	1.46	4.71	0	0	1.29	5.83	2.44	1.01	4.31
13390	'Dlx1'	46.03	20.55	20.3	31.5	0.95	4.18	6.09	39.83	15.26	1.85
13392	'Dlx2'	19.95	7.29	8.21	5.13	0.02	0.11	0.5	3.85	4.02	0
13393	'Dlx3'	0	0	0	0	0.11	0	0	0	0	0
13394	'Dlx4'	0	0	0	0	2.09	0	0	0	0	0.16
13395	'Dlx5'	15.76	13.57	6.06	7.56	53.3	18.89	0.13	20.23	23.87	0
13396	'Dlx6'	17.79	9.66	9.26	7.93	16.3	12.32	2.6	20	14.97	0.74
13400	'Dmpk'	1.23	2.53	1.83	8.5	1.54	2.65	2.03	1.03	0	2.39
13401	'Dmwd'	17.63	8.23	10.6	10.8	8.36	13.23	10.1	8.81	2.32	10.96
13405	'Dmd'	6.07	11.21	3.23	11.9	12.5	21.67	11	9.39	13.18	12.16
13411	'Dnah11'	1.95	0	0.3	0	0	0	0	0	0	0
13417	'Dnah8'	0.28	1.12	0	0	0	0	0.23	0	0	0.21
13418	'Dnajc1'	2.94	2.41	3.57	3.91	2.84	0.34	8.5	3.75	4.12	3.02
13419	'Dnase1'	0.57	0.79	0.61	0.82	1.69	0	0.52	0.26	0.1	0.58
13421	'Dnase1l3'	1.36	0	2	0	0	0	0	0	0	1.52
13423	'Dnase2a'	0.46	0	0	0.04	0	4.49	0	0	0	0.05
13424	'Dync1h1'	7	4.86	9.17	9.07	7.82	7.1	13.2	5.12	7.26	7.91
13426	'Dync1i1'	167.3	143	137	106	106	76.72	57	202.9	116.8	97.94
13427	'Dync1i2'	78.73	84.13	61.6	103	54.4	81.97	120	84.01	97.2	101.82

13429	'Dnm1'	41.35	35.97	38.5	10.2	56.2	50.83	59.3	42.15	36.76	33.77
13430	'Dnm2'	18.1	16.08	23.9	13.9	32.9	8.17	9.07	15.79	23.98	17.05
13433	'Dnmt1'	6.85	12.65	14.6	3.93	2.53	5.15	8.15	6.12	15.93	21.21
13434	'Trdmt1'	0.51	2.31	0.04	6.67	5.36	0.03	0	2.51	3.47	8.65
13435	'Dnmt3a'	4.68	5.32	5.92	3.85	8.94	2.26	3.11	3.65	4.37	1.36
13437	'Dnpep'	36.39	58.43	29	21.8	72.9	60.26	39.1	72.39	69.06	66.59
13445	'Cdk2ap1'	21.66	14.96	18.5	24.8	26.5	13.26	23	11.29	17.55	22.21
13446	'Doc2a'	19.05	14.66	6.89	11.1	13.8	11.64	18.9	12.22	7.67	4.53
13447	'Doc2b'	1.4	2.59	1.16	7.42	2.63	1.61	0.98	0.61	1.33	0.74
13449	'Dok2'	0.05	0	0.04	0.06	1.79	0	0.2	1.12	0	0
13476	'Reep5'	239.5	199.6	180	163	204	204.7	236	148.9	154.8	218.96
13478	'Dpagt1'	11.15	21.53	22.3	0.5	25.6	6.69	16.6	9.97	15.46	20.75
13480	'Dpm1'	28.73	28.5	22	50.8	32.7	28.12	35.3	30.32	40.79	37.29
13481	'Dpm2'	88.25	125.5	50.9	126	130	115.8	93.6	86.84	96.31	90.67
13482	'Dpp4'	0.15	0.4	0.46	0.89	0.36	1.15	0.17	0.31	0.96	0.24
13483	'Dpp6'	114.7	62.54	102	84.6	96.7	61.18	106	106.5	88.11	124.99
13486	'Dr1'	7.64	2.32	10.6	10.7	8.69	4.04	2.72	10.72	6.62	4.8
13488	'Drd1'	0.07	0	0.01	0	2.36	0	0	0	0	4.17
13489	'Drd2'	3.03	0	5.18	0	0	0	0.02	0	0.26	1.03
13490	'Drd3'	36.3	0	64	0	40.5	190.2	0	114.3	154.3	267.35
13492	'Drd5'	0	0.76	1.6	0.77	5.84	5.69	0	0	8.13	2.25
13494	'Drg1'	183.2	133.7	142	208	113	131.8	147	180.4	115.5	182.09
13495	'Drg2'	85	78.47	106	152	44.3	96.98	69.1	83.73	87.76	44.8
13496	'Arid3a'	0.17	0.81	0.04	0	0.87	0.03	0	0.68	0.15	0.07
13497	'Drp2'	14.2	4.8	2.25	0.01	1.45	0.34	2.09	5.58	2.75	0.95
13498	'Atn1'	7.99	7.19	13.8	5.98	10.7	9.13	10.8	5.8	12.64	8.47
13506	'Dsc2'	1.7	2.62	0.08	0	0.78	0.07	0	1.94	0.01	0.01
13508	'Dscam'	3.04	1.41	6.26	3.1	5.72	1.43	9.15	3.82	6.36	3.3
13511	'Dsg2'	0	0	0	3.42	0.64	0	0.02	0.37	0	0.01
13518	'Dst'	5.93	3.4	7.96	8.36	6.59	5.86	8.32	3.09	8.69	7.57
13521	'Slc26a2'	0.07	1.13	0	3.86	0.01	0.57	0	0.61	0.02	2.72
13522	'Adam28'	0.27	0.21	0.46	0.76	0.31	0.26	0.25	0.21	0.31	0.24
13524	'Adam18'	0	0	0	0	0	0	0.91	0	0	0
13527	'Dtna'	10.82	7.51	14	4.89	10.5	8.01	28.6	8.72	6.6	15.84
13528	'Dtnb'	12.22	13.16	16.2	4.43	14.6	10.86	20.8	5.38	21.5	13.01
13531	'Usp17la'	0.08	0.05	0.02	0.19	0.12	0.05	0.11	0.03	0.03	0.06
13537	'Dusp2'	0	0	2.64	5.94	1.2	0	0	0	0	0
13542	'Dvl1'	3.49	4.15	2.28	6.61	9.85	7.47	6.94	9.43	7.61	10.33
13543	'Dvl2'	2.36	0.39	1.66	0.33	1.01	1.25	1.69	0.82	5.9	0.95
13544	'Dvl3'	4.37	5.35	6.04	7.44	3.34	6.65	11.6	3.48	6.47	0.01
13548	'Dyrk1a'	8.89	10.7	7.34	12.1	8.06	3.32	8.29	7.2	7.28	7.35
13549	'Dyrk1b'	2.86	1.94	3.52	0.01	1.34	0.24	2.13	2.16	0.81	7.81
13555	'E2f1'	0	1.26	0	0	0	0.02	0.07	0	0	0
13557	'E2f3'	0.02	1.36	0.31	2.64	1.53	0.77	0.78	1.48	0	0.04
13559	'E2f5'	2.56	0.28	4.66	0.67	1.5	4.58	6.06	0.99	1.68	0.72
13560	'E4f1'	11.58	19.08	15.3	0.9	8.96	0.73	9.37	16.52	15.49	19.24
13589	'Mapre1'	17.46	20.59	16.1	25.6	23.2	10.44	22.2	16.78	20.98	20.37
13590	'Lefty1'	0	2.48	0.03	0	0	0	0	0	0	0
13591	'Ebf1'	0.02	31.04	1.76	3.82	2.15	0	7.83	0	2	0
13592	'Ebf2'	0	2.05	0.77	0	0.58	0	0.01	0.33	2.79	1.22
13593	'Ebf3'	0	0	0	0	0.97	0.87	0	0	0	0
13595	'Ebp'	15.68	17.92	5.5	0.03	22.7	27.02	16.2	24.96	37.96	16.91
13599	'Ecel1'	366.7	166.3	178	45.2	104	52.98	144	281.6	78.75	72.52
13601	'Ecm1'	0	3.25	0	0	0	5.46	0	0.02	2.45	0
13602	'Sparcl1'	290.6	222.5	170	219	138	220.9	236	144.7	207.5	145.11
13603	'Opn3'	5.16	3.87	0.07	28.1	2.29	0.03	0.09	0.56	6.26	2.08
13605	'Ect2'	0	0	0	0	0	0	0	1.46	0	0
13607	'Eda'	0.71	0.07	0	0.03	0	0.01	0	0.12	0.74	0.78

13608	'Edar'	2.35	0	0	1.22	0	0	0	0	0	0
13609	'S1pr1'	28.64	21.3	2.59	24.2	6.96	22.28	10.1	15.87	40.7	0.93
13610	'S1pr3'	2.26	2.74	0	0.09	2.19	0	0.01	1.61	5.25	0
13611	'S1pr4'	0	0.3	1.32	0	0	0	0	0	0	0
13612	'Edil3'	21.46	15.05	56.9	28.6	28.8	7.17	19.8	20.76	73.6	56.54
13616	'Edn3'	0	0	0	0.02	1.36	0	0	0	0	1.68
13617	'Ednra'	0	0	0.01	0	0	0	0	0	0	0
13618	'Ednrb'	38.72	21.55	11.7	27.3	2.02	9.54	15.1	15.49	20.4	4.2
13619	'Phc1'	4.57	2.32	4.79	9.88	1.34	0.01	3.51	1.8	0.57	6.96
13626	'Eed'	3.4	2.16	3.6	20.2	0	7.51	3.21	2.01	3.08	1.24
13627	'Eef1a1'	645.6	840.5	714	819	563	644.5	671	475.1	359.4	430.89
13628	'Eef1a2'	10.04	3.81	4.38	0.14	4.05	3.42	5.09	6.76	7.07	3.19
13629	'Eef2'	72.9	76.94	81.1	79.7	91	76	65.4	65.98	63.3	75.96
13631	'Eef2k'	3.59	3.58	8.66	4.36	4.36	9.28	6.02	4.17	5.43	2.51
13636	'Efna1'	0	2.63	2.99	0	0	10.81	0	3.56	0	0
13637	'Efna2'	4.15	1.04	0.09	1.39	2.45	0	0	2.82	3.32	0.82
13638	'Efna3'	14.47	3.95	14.9	11.3	15.8	7.39	5.03	3.5	15.43	3.64
13639	'Efna4'	0	0	1.92	0	0	0	0	0	0	0
13640	'Efna5'	41.49	47.47	29.8	57.3	14.5	11.25	30.2	25.98	22.8	27.59
13641	'Efnb1'	1.24	0.31	2.59	0.23	2.26	0.55	0.47	2.79	2.92	1.43
13642	'Efnb2'	0.66	1.96	0.7	1.09	1.48	2.55	1.47	2.77	0	0.94
13643	'Efnb3'	64.05	88.45	96.9	33.2	88.2	67.82	91.4	45.34	87.53	51.58
13644	'Efs'	3.55	3.23	4.65	0	0	0	2.75	2.35	0.4	2.47
13645	'Egf'	0	1.96	0	10.4	1.96	0	0	1.34	0	0
13649	'Egfr'	0	0	0	0.34	0.04	4.68	0	0	0.11	0
13650	'Rhbdf1'	2.23	0.58	1.44	0	1.47	5.15	3.79	0	0.13	0.76
13653	'Egr1'	111	232.8	223	292	38.5	452.9	194	94	28.15	190.09
13654	'Egr2'	4.81	3.69	2.22	19.7	0.21	2.39	0.02	0	0	0.2
13655	'Egr3'	0	2.36	0	3.54	0	0	0	1.17	0.04	0.02
13656	'Egr4'	0	1.33	1.05	20.1	0	2.98	5.91	0	0	0.79
13660	'Ehd1'	13.79	9.67	2.89	0.02	8.04	14.28	1.63	8.53	9.97	4.64
13663	'Ei24'	7.45	16.39	6.77	11.6	16.4	11.38	3.42	11.81	28.67	8.58
13664	'Eif1a'	49.96	59.53	59.7	98	42.9	35.74	64	58.26	74.77	46.94
13665	'Eif2s1'	33.26	33.95	17.3	33	22.6	32.31	21.5	31.54	28.47	27.05
13666	'Eif2ak3'	2.2	0.4	2.87	4.62	3.06	0.06	0	0.01	0	0.85
13667	'Eif2b4'	34.19	14.91	32	21.7	39.8	28.84	36.9	35.02	36.66	32.5
13669	'Eif3a'	32.04	22.53	17.4	23.5	29.6	20.75	21.1	17.37	17.62	22.88
13680	'Ddx19a'	1.05	3.65	2.32	0.02	0.76	0.68	2.52	1.67	2.6	5.09
13681	'Eif4a1'	352.7	379.9	234	263	268	333.5	377	396.9	279.6	374.79
13682	'Eif4a2'	319.7	372.8	310	383	255	264.9	321	261.2	220.1	236
13684	'Eif4e'	190.9	256.6	145	175	166	194.7	141	219.2	181.7	243.43
13685	'Eif4ebp1'	0.65	0	0.1	0	5.36	5.78	0.16	0	0.05	0
13688	'Eif4ebp2'	6.53	8.6	1.39	2.05	4.02	0.03	1.14	0.79	2.41	8.09
13690	'Eif4g2'	40.04	25.59	36.3	35.6	35.7	13.45	29.7	32.81	53.31	58.85
13706	'Cela2a'	0	0.93	0	0	0	0	0	0	0	0
13709	'Elf1'	0	0.27	0.09	1.1	0.1	3.46	0.07	0.67	0.31	0.03
13710	'Elf3'	0	0	0.19	3.39	2.07	0	0	0	0	0
13712	'Elk1'	4.97	3.49	5.03	8.39	3.28	6.03	16	9.02	2.97	5.47
13713	'Elk3'	1.02	3.52	1.02	1.32	0.95	0.3	3.15	1.77	0	1.54
13714	'Elk4'	1.45	3.51	1.23	2.66	0.71	5.07	0.28	0.56	2.78	0
13716	'Ell'	1.36	8.34	4.61	15.4	10.8	1.49	5.65	6.55	8.35	3.88
13717	'Eln'	0	1.08	1.83	0	0.98	0	3.81	0.97	0	0.22
13722	'Aimp1'	46.37	52.16	36.1	41.3	35.8	13.74	44.4	51.93	31.52	35.88
13723	'Emb'	77.99	70.72	25.6	70.3	53.4	70.24	55.6	34.24	96.47	101.25
13726	'Emd'	38.54	40.04	60.6	38.3	52.9	45.67	60.8	54.14	42.39	78.35
13728	'Mark2'	11.71	10.26	7.03	8.89	13.7	5.56	10.8	10.09	7.37	11.08
13730	'Emp1'	0	0	0.04	1.56	0	0	0	0	0	0
13731	'Emp2'	0.72	0.8	2.59	0	3.21	2.24	0	0	3.66	5.41

13732	'Emp3'	0	0.19	0	0	0	0	0	0	0	0
13733	'Adgre1'	0	2.84	0.25	0	4.56	2.78	0	0	0.71	0
13796	'Emx1'	0	0	0	0	6.88	10.14	0	0	0	2.28
13797	'Emx2'	0	0	0	0	0	3.04	0	0	11.93	3.61
13800	'Enah'	9.7	3.76	7.33	12.9	6.63	4.1	5.65	7.05	1.1	3.09
13803	'Enc1'	46.94	44	19.8	4.73	39.4	15.51	67.5	41.29	23.83	16.62
13804	'Endog'	7.76	6.76	9.77	9.86	12.9	12.41	0	4.23	11.87	4.82
13805	'Eng'	0	0	0.01	0	0	0	0	0	0.01	0.86
13806	'Eno1'	1030	928.3	1000	851	876	1503	1320	1291	1390	1215.65
13807	'Eno2'	243.5	243.3	335	137	261	221.2	248	253.1	150.3	239.73
13808	'Eno3'	8.23	7.01	8.47	18.7	10.3	9.08	6.97	6.76	1.65	11.63
13813	'Eomes'	0	0.9	10.9	6.62	0	0	2.82	2.33	0.01	0
13819	'Epas1'	0.85	2.01	8.9	4.98	0.45	4.23	4.54	1.58	2.59	1.92
13821	'Epb41l1'	15.72	12.59	12.2	18.8	19.9	6.04	18	15.93	8.12	12.52
13822	'Epb41l2'	6.91	4.71	5.85	10.1	2.77	13.11	5.47	1.74	5.72	4.68
13823	'Epb41l3'	41.31	50.59	48.6	24.6	43.7	31.4	40.2	57.78	28.75	46.91
13824	'Epb41l4a'	3.57	8.35	5.1	0	3.95	0.03	1.23	7.19	1.39	8.18
13829	'Dmtn'	218.3	283	156	119	194	213.5	192	257.2	240	252.08
13830	'Stom'	0	0	0	4.05	0.4	0	2.52	1.93	0	0
13831	'Epc1'	7.71	15.48	5.62	37	8.13	15.18	2.81	7.58	10.62	6.15
13835	'Epha1'	0	0	0.05	0.31	0.15	0.11	0	0	0	0
13837	'Epha3'	0	1.06	0.42	0	0	0	0	0.06	0	1.92
13838	'Epha4'	3.97	3.28	1.4	3.07	2.26	1.29	1.13	5.57	2.67	2.06
13839	'Epha5'	9.56	13.21	5.43	6.44	3.94	11.91	14	12.24	16.39	4.18
13840	'Epha6'	2.99	4.89	7.49	0.6	3.63	1.43	14.3	3.05	1.13	1.18
13841	'Epha7'	2.96	2.49	1.89	0	7.95	2.79	11.5	4.98	16.98	4.91
13842	'Epha8'	0.05	0	0	0	1.58	1.14	0	0	0	0
13844	'Ephb2'	0	0.02	0.02	0	0.54	0	0.65	0.77	0	0.72
13845	'Ephb3'	1.69	0	0.02	0	0.47	0.09	2.81	0	0	0
13846	'Ephb4'	0	0	0	1.51	0	0	0	0	1.5	0
13848	'Ephb6'	4	5.01	11	0	13.5	8.79	19.4	5.69	26.84	6.11
13849	'Ephx1'	25.54	2.89	5.76	0.17	0.94	15.4	2.4	15.9	4.05	18.83
13850	'Ephx2'	3.57	3.71	5.39	11.8	7.6	5.27	5.66	2.12	0.02	0.56
13852	'Stx2'	0.76	7.33	8.37	9.45	6.64	1.6	1.2	1.65	0.65	8.4
13853	'Epm2a'	0.97	0	0	0	0.05	0	0	1.57	0	0
13854	'Epn1'	3.45	5.11	11.9	0.18	4.04	3.98	6.14	3.64	6.13	3.46
13855	'Epn2'	15.03	8.02	20.3	14.2	9.48	11.93	32.2	16.24	12.74	3.87
13857	'Epor'	1.82	0	3.57	0	0	3.33	0	2.35	0	0
13858	'Eps15'	15.25	24.16	12.5	8.48	11	10.46	14.1	15.49	13.43	15.14
13859	'Eps15l1'	14.41	26.25	19.3	7.41	15	19.76	33.1	35.46	28.59	29.44
13860	'Eps8'	4.69	6.87	5.67	3.44	2.4	4.82	5.89	3.04	3.52	0.04
13861	'Epx'	0	2.06	0	0	0	0	0	0	0	0
13864	'Nr2f6'	8.81	2.47	1.24	7.75	5.38	2.9	10.8	0.53	1.13	2.94
13865	'Nr2f1'	1.8	6.5	0.79	3.57	1.39	0	4.05	0.05	0	0
13866	'Erbb2'	0	0	0	0	0	0	0.01	0.05	2.4	0
13867	'Erbb3'	0.17	0	0.75	0	0	0	0	0	0	0
13869	'Erbb4'	2.45	0.48	1.77	0.12	1.34	1.65	0.15	0.65	1.94	0.01
13870	'Ercc1'	33.8	22.54	24.5	14.3	31.7	26.5	36.5	31.58	44.75	26.77
13871	'Ercc2'	0.84	2.49	1.21	1.23	4.21	0.93	2.38	0.14	0.26	4.53
13872	'Ercc3'	12.2	6.26	9.02	13.7	9.9	14.74	13.1	12.74	15.69	8.48
13875	'Erf'	0	0	0.66	0.1	0.31	0	0	0	0.08	0.02
13877	'Erh'	305.1	287	224	449	201	381.5	224	340.2	239.7	328.31
13885	'Esd'	90.21	102	107	79.2	73.1	162.6	88.9	95.33	109.7	119.02
13924	'Ptprv'	0	0	0	0	0.01	0	0	0	0.12	0
13929	'Amz2'	15.66	19.21	11.1	0.02	8.78	24.71	15.8	6.25	13.39	15.91
13972	'Gnb1l'	1.12	1.89	4.7	0.02	7.29	0	9.25	5.47	3.01	0
13982	'Esr1'	26.19	11.6	6.7	30.2	2.73	1.65	4.89	20.49	0.02	2.95
13983	'Esr2'	2.38	0.11	0.01	0	0	0.01	0	0	2.67	0

13990	'Smarcad1'	5.45	0.59	3.44	6.81	1.61	5.79	3.75	1.67	1.96	2.39
13992	'Khdrbs3'	27.51	11.36	9.61	36.3	7.54	4.72	15.5	11.44	16.34	15.27
13998	'Fgd6'	0.11	0.04	0	0	0	0	0.46	0.6	0	0
13999	'Gm14288'	38.81	65.13	46.9	56.4	56	62.04	40.9	34.67	37.05	39.55
14000	'Drosha'	9.17	15.3	15.4	20.5	13.6	4.06	17.7	15.61	20.86	27.51
14004	'Chchd2'	1103	967.8	911	798	1117	1563	1008	924.7	1014	1117.67
14007	'Celf2'	8.67	8.82	4.56	14.1	11.8	11.67	14.3	8.66	3.51	4.9
14009	'Etv1'	11.23	5.85	9.51	1.03	12.7	37.56	4.77	2.89	25.8	61.93
14011	'Etv6'	1.81	0.67	0.93	0.23	0.57	0	0	0.19	0.86	1.69
14013	'Mecom'	0	0.01	0	0	0	0	0	0	0	0
14017	'Evi2a'	5.28	16.78	14.5	0.59	21.8	34.48	19.3	3.29	3.61	5.62
14020	'Evi5'	6.2	1.65	5.61	15.4	7.12	6.67	7.3	4.08	5.84	3.58
14025	'Bcl11a'	5.9	6.39	26.7	12.4	16.5	4.51	0	16.17	11.86	3.13
14026	'Evl'	9.69	10.32	13.3	23	13.4	8.38	8.82	7.72	14.3	4.21
14027	'Evpl'	3.92	3.18	1.01	4.42	0.1	2.31	0.57	0.86	0.28	3.19
14030	'Ewsr1'	160.1	204.4	151	180	193	172.8	124	230.6	249.6	199.13
14038	'Wfdc18'	0	3.68	0	0	1.69	3	0	2.85	0	0
14042	'Ext1'	0.2	0.78	3.25	4.17	5.58	2.55	1.5	0.7	1.98	2.37
14043	'Ext2'	32.51	39.33	29	23.5	12.8	10.1	51.2	13.7	50	19.97
140476	'Strc'	0	0	0	0	0	0	0.48	0	0.07	0
140477	'Dmbx1'	0	0	0	0	0.01	0	0	0	0	0
14048	'Eya1'	1.47	0.13	4.18	8.97	0	0	8.16	0.18	0	1.79
140481	'Man2a2'	9.87	3.57	3.24	21.6	13.3	9.29	3.08	5.69	12.46	2.47
140482	'Zfp358'	6.67	2.36	13.5	6.88	10.5	12.12	10.3	6.11	15.02	17.14
140483	'Hnmt'	39.74	21.07	22.9	1.65	20.1	59.94	29.5	23.58	41.45	23.3
140484	'Pofut1'	0.45	2.45	0.67	1.16	1.34	2.8	0.49	0.69	1.72	1.29
140488	'Igf2bp3'	0.12	0.08	0.18	0.37	0.2	0.55	0	0.08	0	0.26
140489	'Bhlhe23'	0	1.42	0	0.02	0	0	0.02	0	0	0
14049	'Eya2'	0	0	0	0	0.72	0.02	0	0	0	0
140491	'Ppp1r3a'	0.01	0	0.03	0.01	0	0	0.01	0	0.01	0.01
140492	'Kcnn2'	3.4	2.46	11.9	0.03	8.01	13.21	13	1.5	1.46	1.44
140493	'Kcnn3'	3.08	0.91	4.46	3.47	1.42	2.87	4.31	0.74	0.36	1.1
140494	'Atp6v0a4'	0	1.02	0	0	0	1.75	0	0.64	0	0
140497	'Cd300c2'	0	0	0.04	0	0	13.93	0	0	0	0
140498	'Rxfp2'	1.97	0	0.03	0	0	4.46	0	0	0	1.77
140499	'Ube2j2'	11.06	9	8.52	9.99	7.61	5.92	7.47	9.94	15.32	8.12
14050	'Eya3'	2.22	5.65	2.52	0	0.86	0.02	5.98	1.87	0	1.02
140500	'Acap3'	9.34	5.08	16.5	5.21	9.25	9.01	7.46	15.51	16.66	6.07
14051	'Eya4'	0	0	0	0	0	0.01	0	0	0	0
140546	'Eri3'	163.7	183.5	165	345	198	187.3	111	216.4	227.2	162.75
14055	'Ezh1'	15.96	16.26	18	10.2	10.7	17.68	25.5	15.16	26.29	19.24
140557	'Smc1b'	0.76	0	0	0.03	0	0	0.04	0	0	0.28
140559	'Igsf8'	64.96	72.83	103	38.6	135	51.98	133	107.8	128.8	81.66
14056	'Ezh2'	0	0	0	0	2.27	0.11	0	0	0	0
14057	'Sfxn1'	76.44	78.71	53.7	64.3	83.3	101.7	57.1	70.8	49.95	80.41
140570	'Plxnb2'	2.28	3.91	8.17	8.47	4.25	2.08	5.5	1.96	7.21	5.91
140571	'Plxnb3'	2.44	0	4.01	0.09	0	0.16	0.04	0	0	0
140577	'Ankrd6'	14.99	11.23	11.4	23.8	15.1	12.99	24.9	18.05	13.25	18.57
140579	'Elmo2'	24.76	51.15	22.7	22.1	41	26.41	40.7	55.1	54.77	29.43
14058	'F10'	0	0	0	0	0.02	0.02	0	0	0	1.47
140580	'Elmo1'	51.91	31.72	61.6	36.4	50.5	31.46	20.6	40	67.41	25.78
14061	'F2'	0	0	0	0.39	0	0	0	0.1	0.5	0.13
14062	'F2r'	7.19	10.62	14.8	0	0	0.55	4.28	7.74	5.52	3.91
140629	'Ubox5'	1.43	0	3.6	6.5	5.12	0	4.89	3.67	3.55	1.74
14063	'F2rl1'	0	0	1.4	0	0	0	0	0	0	0
140630	'Ube4a'	3.34	2.12	3.39	4.97	2.66	0.34	8.45	0.87	3.74	3.37
14064	'F2rl2'	12.18	3.26	1.88	0	0	3.43	0	0	0.05	6.42
14065	'F2rl3'	0	1.15	0	0	0	2.45	0	0	2.25	2.3

14066	'F3'	41.03	36.53	16.9	61.6	7.67	38.31	16.2	24.62	49.85	3.17
14069	'F8'	0.45	0	0.43	0	0	0.04	0	0.82	1.13	0.81
14070	'F8a'	14.57	29.05	33.7	46.4	16.7	23.18	36.5	24.69	18.73	17.15
140703	'Emid1'	0	4.2	4.35	0	0	0	8.38	3.93	4.64	0
140709	'Col26a1'	0	0.29	0.74	0	0.8	0.02	0.07	0	0	0.05
140721	'Caskin2'	0.06	0.01	3.92	0	0.11	0.01	2.85	1.38	0.88	0.24
140723	'Cacng5'	31.24	2.04	27.8	10.4	15.7	20.65	2.95	12.23	69.11	109.68
14073	'Faah'	25.88	15.8	23.6	13.9	8.97	4.25	11.9	10.24	15.23	19.04
140740	'Sec63'	3.21	0.41	1.58	2.14	3.84	2.97	5.66	0.45	1.74	3.14
140741	'Gpr6'	25.67	3.77	3.23	29.6	0	10.24	0.02	12.55	14.06	11.35
140742	'Sesn1'	30.47	22.14	19.7	20.6	23.8	14.5	13.3	24	22.82	14.01
140743	'Rem2'	15	34.06	17.4	31.9	20.7	0.95	1.86	15.44	1.51	13.22
14077	'Fabp3'	252.5	156.9	186	250	122	252.6	152	203.2	336	306.3
140780	'Bmp2k'	0	0.12	0.14	0	0.01	1.14	0	0	1.2	0
140781	'Myh7'	4.89	8.85	4.75	8.04	3.29	4.83	9.88	2.86	2.79	5.27
140792	'Colec12'	2.87	4.75	4.06	11.7	4.62	2.81	3.82	2.36	5.7	4.92
140795	'P2ry14'	2.05	0	0.09	3.98	0.03	0	0.55	0.31	2.43	0
140806	'Il25'	0	0	0	0	0	0	0	0	0	3.06
14081	'Acsl1'	11.86	15.12	16.3	7.05	12.6	5.68	15.5	16.83	10.02	23.21
140810	'Ttbk2'	1.81	3.21	1.91	2.07	4.87	0.36	2.09	1.61	2.97	1.92
14082	'Fadd'	0.67	0.07	3.13	0.14	1.76	0.93	0.04	0.28	2.21	4.23
14083	'Ptk2'	8.08	7.15	4.31	6.1	4.87	3.05	10.1	6.52	9.07	7.01
14084	'Faf1'	10.82	4.07	2.55	5.72	5.62	3.79	9.76	1.68	4.96	1.53
14085	'Fah'	11.44	4.32	10.1	12.6	12.9	18.74	14.8	4.12	25.37	17.86
140858	'Wdr5'	1.44	20.32	9.9	41.8	23.4	3.88	21	9.16	4.44	6.5
140859	'Nek8'	0	5.76	0.14	0.57	4.37	8.72	0.73	6.32	3.54	0.02
14086	'Fscn1'	35.75	36.67	43.5	28.3	36	26.03	40.1	38.85	37.19	43.53
14087	'Fanca'	0.03	0.01	0	0	0	0	0	0	0	0
14088	'Fancc'	4.02	5.58	7.48	6.67	8.96	0.11	6.77	0.16	5.67	9.31
140887	'Lnx2'	0.81	1.32	0.29	3.02	0.93	0.01	0	0.78	0.01	2.45
14089	'Fap'	0	0	0	0	0	0	2.91	0	0	0
140904	'Caln1'	11.95	15.21	13.8	8.6	28.5	13.72	13.9	16.79	6.9	3.69
140917	'Dclre1b'	3.65	1.16	1.73	6.29	0.36	1.64	4.43	1.8	6.18	1.2
140919	'Slc17a6'	39.26	68.01	110	104	78.4	83.55	155	67.8	72.68	134.26
14102	'Fas'	0	0	0	0	0	0	3.1	0	0.06	0
14104	'Fasn'	6.76	8.91	11.7	10.1	10.9	10.52	9.79	6.21	12.83	10.28
14105	'Srsf10'	40.01	30.63	30.7	31.7	28.1	24.3	30.2	25.32	36.74	19.85
14106	'Foxh1'	0.04	0.06	0.03	0.06	0.04	0.04	0.13	0.03	0	0.11
14107	'Fat1'	0.88	1.16	0.76	0.29	0.48	0	0	0.02	0.01	0.11
14109	'Fau'	486.2	450.8	698	682	444	569.1	514	247.6	493.5	460.07
14113	'Fbl'	27.27	25.81	31.6	57.5	30.7	15.09	28.6	13.86	42.11	39.18
14114	'Fbln1'	6.69	1.33	0.02	0	1.03	0	12.7	2.72	2.43	0.65
14115	'Fbln2'	0	0.58	0.01	3.42	0	0	7.06	0.76	0	0
14118	'Fbn1'	0	0	0	0.07	0.27	0.16	0	0.03	0	0
14119	'Fbn2'	2.16	1.41	1.14	0	1.38	0	0.01	0.03	0.14	0
14120	'Fbp2'	0	0	0.11	0.09	0	0.04	0	0	0.03	0
14121	'Fbp1'	0	0	6.3	0	0	0	0	0	0.03	0
14123	'Fbrs'	6.72	8.43	8.6	9.71	0.59	3.66	6.06	1.07	2.84	2.7
14127	'Fcer1g'	18.63	16.77	10.5	47.9	15.7	176.3	28.6	7.3	11.6	17.04
14128	'Fcer2a'	0.01	0.36	0.12	0.88	0.01	0.69	0.05	0	0	0.18
14129	'Fcgr1'	0	0	0	0	0	6.74	0	1.46	0	0.74
14130	'Fcgr2b'	0	0	0	0	6.73	0	0	0	0	0.06
14131	'Fcgr3'	13.59	2.67	3.87	18.9	3.91	100.8	6.74	2.78	0	3.37
14132	'Fcgrt'	2.99	8.26	0.03	0	0	8.55	0	6.55	0	0
14137	'Fdft1'	62.94	53.02	72.3	45.4	81.7	36.94	75.2	71.35	56.79	58.49
14148	'Fdx1'	13.55	14.59	22.4	16.2	26.7	23.14	22.3	10.89	8.34	7.85
14149	'Fdxr'	3.38	18.35	10	0	7.37	16.05	4.51	6.79	5.17	12.38
14151	'Fech'	39.18	43.2	34.1	21.4	31	24.19	13.8	32	32.6	51.3

14154	'Fem1a'	1.5	5.7	4.98	10.5	1.58	0.44	0.01	3.5	2.35	2.41
14155	'Fem1b'	8.89	6.84	6.59	16	1.86	5.52	8.01	3.75	8.14	2.96
14156	'Fen1'	3.53	6.95	6.37	17.8	23.4	24.77	0.07	8.41	5.7	31.33
14158	'Fer'	14.65	8.07	9.3	5.43	10.9	11.27	16.6	6.15	11.79	15.59
14159	'Fes'	0	0	0.59	3.05	0.07	0	1.76	0	4.5	6.07
14160	'Lgr5'	0.03	0.03	1.3	1.54	0.55	0.06	0.09	1.38	0.11	0.03
14163	'Fgd1'	0	0.63	0	0.71	0.02	0.54	0	1.45	0	0
14164	'Fgf1'	3.24	12.35	14	3.77	14.7	7.02	9.14	9.12	20.42	11.38
14165	'Fgf10'	0.01	0.06	0	0	0.35	0	0	0	0.47	1.58
14166	'Fgf11'	0.96	0	0.69	0	4.28	0	1.61	2.06	0	0.48
14167	'Fgf12'	44.6	47.94	40.2	46.8	29	36.39	52	49.74	31.3	32.94
14168	'Fgf13'	41.1	28.87	25	14.9	51.7	43.03	93.9	80.55	31.77	50.24
14169	'Fgf14'	12.96	10.16	18.7	17.1	24.5	12.57	7.49	13.55	6.08	16.23
14170	'Fgf15'	0	0	0	0	0	0	0.33	0	0	0
14171	'Fgf17'	2.16	2.46	1.32	0	14.3	26.9	1.75	0.03	21.94	33.82
14172	'Fgf18'	0	0	0.06	0	1.19	0	0.36	0	0	0
14178	'Fgf7'	0	1.05	0	0	0	1.07	0	0	0	0.98
14180	'Fgf9'	5.99	7.51	8.93	8.12	10.3	3.82	1.42	6.4	4.41	0.07
14182	'Fgfr1'	12.28	12.3	5.57	25.7	7.78	4.77	7.87	11.25	10.73	3.34
14183	'Fgfr2'	1.47	0.75	3.48	0	0.34	1.77	0	0.82	0.05	0
14184	'Fgfr3'	5.94	0.85	5.04	6.4	2.16	0.46	0	3.24	7.69	0.15
14187	'Akr1b8'	0	0	0.04	0	0	0	0	0	0	0
14190	'Fgl2'	0	0.91	0	0	0.61	0	0	0	0.91	0
14191	'Fgr'	0	0.14	0	0	0.68	0	0	0	0	0
14194	'Fh1'	96.05	95.23	84.9	60.6	89	127.8	143	109.1	94.48	86.29
14198	'Fhit'	4.65	0	14	1.05	20.3	2.01	3.65	13.1	8.06	7.88
14199	'Fhl1'	84.71	107.9	98.4	69.1	76.3	77.6	92.5	120	59.19	62.48
14200	'Fhl2'	7.48	8.46	14.5	0.41	0.55	6.85	25.8	8.35	0.43	8.87
14201	'Fhl3'	0	0.09	0	0	0.3	0	0	0	0	0
14202	'Fhl4'	0	4.24	0	0	0	0.07	0	1.76	0.02	0
14204	'Il4i1'	0	2.87	3.69	2.45	1.18	0	0	0.35	0.02	0
14205	'Vegfd'	0	0	0	0	0	4.26	0	0.23	0.28	0
14208	'Ppm1g'	78.06	54.16	65.1	56.6	59.8	39.36	83.2	43.79	55.73	67.05
14211	'Smc2'	2.74	1.98	3.01	1.28	1.29	1.05	13.2	0.87	2.65	4.92
14218	'Sh3pxd2a'	0.06	0.08	1.45	0.01	0.28	0	0	0.08	0	0
14219	'Ctgf'	0	0	3.45	0.71	0	0	0.02	0	0	0.35
14221	'Fjx1'	0.34	0.47	0	3.44	0.73	1.22	2.09	0.02	2.18	0
14225	'Fkbp1a'	504.8	517.8	395	441	574	597.2	437	671.4	397.1	439.62
14226	'Fkbp1b'	33	4.37	17.6	3.67	8.81	17.62	9.27	27.63	9.99	14
14227	'Fkbp2'	541.3	474.6	476	412	538	716.8	436	474.2	657.7	673.05
14228	'Fkbp4'	150.8	168.3	139	156	134	167.3	144	160.7	244.8	169.94
14229	'Fkbp5'	1.27	0.59	2.4	13	0.05	1.25	0.03	5.89	2.53	1.94
14230	'Fkbp10'	0	1.5	0.72	0.33	1.05	0	0.15	0	1.13	0
14231	'Fkbp7'	4.54	5.69	4.45	12.6	0.08	5.53	0	8.79	0	2.49
14232	'Fkbp8'	88.48	115.9	127	115	118	155	127	108	119.4	130.13
14233	'Foxi1'	0	0	0	0	0	1.64	0	0	0	0
14234	'Foxc2'	0	0	0	0	0	0	0	0	0	0.02
14235	'Foxm1'	0.03	0.04	0.28	0	0	0.01	0	0.12	0	0.01
14236	'Foxn2'	2.08	1.08	0.94	4.41	0.71	0.56	0	0.53	0.59	0.48
14241	'Foxl1'	0.03	0	0	0	0.02	0	0	0	0.01	0
14245	'Lpin1'	11.1	10	7.67	7.47	18	1.57	9.2	16.86	9.01	13.7
14247	'Fli1'	0	0	0	3.91	0	0.25	0	0	0	0
14248	'Flii'	23.52	14.35	23.3	13.6	14.8	11.62	18.7	12.57	31.27	27.13
14251	'Flot1'	92.13	112.3	125	125	127	137.3	140	123.7	106.6	115.16
14252	'Flot2'	76.64	62.9	62.6	111	51.9	64.42	54.6	72.49	63.6	57.67
14254	'Flt1'	0	0	0	0	0.56	0	0	0	0	0
14255	'Flt3'	4.35	7.2	14.8	5.13	3.69	0.01	11	5.33	2.33	0
14256	'Flt3l'	0	7.68	0	0	0	4.36	0	0	5.24	13.42

14260	'Fmn1'	0.36	0.46	0.9	0	1.06	2	0.22	0.46	1.76	0.16
14261	'Fmo1'	0	0.69	3.45	0	0	0.06	0	2.71	0.75	0
14263	'Fmo5'	1.87	1.16	0	0	1.25	0	0	0	0	2.13
14264	'Fmod'	0	0	0	0	0	0	1.93	0	0	0
14265	'Fmr1'	1.46	0.41	1.77	1.56	3.04	2.25	4.2	2.47	0.09	0.01
14266	'Aff2'	2.83	0.35	0.71	0.21	0.8	0.84	2.29	2.57	2	1.57
14268	'Fn1'	0	0.04	0	0	1.6	0	0	0	0.01	0
142681	'Slc34a3'	0	0	0	0	1.97	0	0	0	0	0
142682	'Zcchc14'	2.45	3.86	3.51	6.24	2.51	0.73	4.79	5.19	2.5	1.87
142687	'Asb14'	0	0.24	0.24	0	0.03	0	0.03	0	0	0.21
142688	'Asb13'	18.36	17.35	37.3	17	28.2	28.92	11.9	31.95	19.6	20.49
14269	'Fnbp1'	10.96	10.93	16	7.79	8.65	6.55	10.3	4.12	8.99	9.25
14270	'Srgap2'	5.72	4.2	4.08	6.34	2.53	6.45	4.08	5.68	2.54	9.22
14272	'Fnta'	14.62	15.92	17.5	44.1	2.29	17.79	8.07	37.82	31.82	7.03
14281	'Fos'	166.7	604.7	453	1226	12.1	367.2	336	82	39.46	122.88
14282	'Fosb'	2.45	30.72	52	77.7	0.04	5.97	43.3	3.55	1.93	30.82
14283	'Fosl1'	0	1.22	0	5.76	0.03	0	1.08	0	0	1.45
14284	'Fosl2'	0	1.16	0.5	0	0.07	1.25	0	0	1.35	1.2
14287	'Fpgs'	4.95	4.91	9.15	7.97	6.73	7.68	4.52	5.51	6.77	4.15
14289	'Fpr2'	0	0	0	0	0	0	0	0	1.29	0
14293	'Fpr1'	0.03	2.15	0	0	0	0	0	0	0	0
14296	'Frat1'	43.64	49.12	27.1	34.9	47.4	48.75	67.8	52.28	23.14	37.86
14297	'Fxn'	30.89	27.01	19.9	51.2	24.9	14.89	1.21	16.21	42.91	16.19
142980	'Tlr3'	0	0.45	0	0	0.12	0	0	1.58	0	1.15
14299	'Ncs1'	128.7	107.8	90.7	110	98.1	139.5	92.2	127	68.83	102.01
14300	'Frg1'	44.71	28.93	40.8	65.5	59.6	80.5	49.2	50.82	53.81	64.16
14312	'Brd2'	28.02	37.97	35.1	70.7	29.3	36.17	37.5	20.25	37.8	32.69
14313	'Fst'	0	0	0	0	0	0	0	0	0	1.06
14314	'Fstl1'	5.44	1.68	3.09	0	5.81	2.06	6.19	7.86	2.34	4.7
14317	'Ftcd'	0	0.38	0	0	0	0	0	0	0.26	0
14319	'Fth1'	1945	2060	1704	2059	9095	2966	2461	2095	1358	1916.33
14325	'Ftl1'	778.6	1024	635	1795	875	1294	674	879.7	802.6	1075.28
14339	'Aktip'	124.7	130.9	137	103	146	129.5	140	105.5	158.9	120.24
14345	'Fut4'	0	0	1.9	0	0.13	0	2.26	1	0	0
14348	'Fut9'	4.24	2.72	2.18	1.8	4.85	2.12	2.71	2.07	1.9	2.87
14349	'Fv1'	7.54	3.82	2.62	0	0	0.54	0	0	0.09	0
14356	'Timm10b'	43.09	47.66	73.8	94.2	60.3	62.71	52.3	64.33	47.98	54.48
14357	'Dtx1'	8.92	9.1	9.07	10.8	6.35	2.41	7.96	6.97	15.11	1.96
14359	'Fxr1'	31.49	39.17	9.88	37.1	21	38.38	28.7	27.33	36.12	19.85
14360	'Fyn'	8.05	14.03	18.9	11.4	10.8	8.45	8.26	5.87	7.38	2.87
14362	'Fzd1'	0.01	3.7	0.01	0	1.2	0	0	0	0.87	0
14365	'Fzd3'	2.89	2.77	3.94	1.47	2.22	2.03	0.89	1.43	0.44	2.49
14367	'Fzd5'	0.21	0	0.01	0	0	0	0	0	0	1.81
14368	'Fzd6'	0	0	1.16	0	0.04	0	0	0	1.75	0
14369	'Fzd7'	0.01	0	0	0	0	0	0	0	0	0
14370	'Fzd8'	0.64	0	0	0	0	0	0	0.21	0.04	0
14371	'Fzd9'	3.44	0.52	0	9.29	1.87	1.77	4.98	0.04	0.02	0
14373	'G0s2'	7.28	9.14	5.39	27.1	3.43	0.11	19.5	10.36	0.05	2.29
14375	'Xrcc6'	20.94	20.68	7.33	21.6	21	8.48	2.3	15.91	22.34	25.03
14376	'Ganab'	33.62	22.62	21.4	23.4	28.6	17.74	23.5	27.21	27.84	27.86
14381	'G6pdx'	22.77	35.45	27.3	23.8	11.4	26.71	1.58	20.97	10.02	17.37
14385	'Slc37a4'	7.74	10.9	11.5	10.8	3.39	19.58	14.1	10.37	21.87	4.27
14387	'Gaa'	964.3	1181	985	598	735	588.4	773	984.9	1024	1131.14
14388	'Gab1'	4.25	0.49	3.55	0	0.5	0	0	0.41	3.04	0.01
14389	'Gab2'	5.38	1.36	2.86	8.96	3.83	0.01	3.94	1.72	2.71	0
14390	'Gabpa'	7.83	2.6	2.59	0	1.88	2.39	3.05	1.3	4.01	7.14
14391	'Gabpb1'	17	16.58	15.4	3.47	10.7	18.4	6.27	12.8	5.74	10.61
14394	'Gabra1'	74.93	41.94	186	5.76	87.9	11.67	142	56.18	72.32	33.67

14395	'Gabra2'	60.23	75.01	29.2	11.5	9.36	27.61	43	37.51	39.05	48.73
14396	'Gabra3'	68.45	46.27	41.4	38.1	23.1	21.3	49	52.38	25.57	71.73
14397	'Gabra4'	16.76	11.49	14.9	21.6	7.81	9.56	11.5	19.51	10.81	23.21
14400	'Gabrb1'	134.4	131.1	103	58.4	61.1	89.48	139	175.6	84.49	83.69
14401	'Gabrb2'	13.88	9.95	30.7	4.25	23	3.97	12.9	9.54	10.82	6.28
14402	'Gabrb3'	12.54	9.38	7.79	11.9	10	4.9	5.29	5.33	5.88	6.5
14403	'Gabrd'	0.92	2.64	0	0	0	0	0	0	1.12	0
14404	'Gabre'	26.13	19.9	2.9	18.8	3.02	6.93	11.7	2.35	16.53	16.24
14405	'Gabrg1'	69.81	35.7	11	3.13	11.3	14.98	27.1	13.43	31.84	45.46
14406	'Gabrg2'	130.3	126.3	138	69.9	88.8	54.86	161	129.8	95.35	108.31
14407	'Gabrg3'	4.89	5.53	6.27	1.83	3.63	10.21	18.4	3.01	0.88	11.92
14409	'Gabrr2'	0	3.24	0	0	11.5	4.04	0	1.15	10.38	12.52
14415	'Gad1'	277.7	114.7	236	44.3	104	23.54	74.6	285.4	106.6	11.21
14417	'Gad2'	121.6	30.41	53.6	22.2	64.3	32.43	41.4	88.46	109.5	101.13
14419	'Gal'	1348	1976	1191	904	182	115.3	151	1404	446.6	240.42
14420	'Galc'	1.14	3.14	1.18	0.75	3.23	0.05	2.87	1.23	2.96	3.26
14421	'B4galnt1'	41.01	58.94	43.3	14.8	26.2	48.2	45.6	24.6	67.52	32.97
14422	'B4galnt2'	0.19	0.23	0.3	0.45	0.47	0.32	0.73	0.13	0.24	0.5
14423	'Galnt1'	5.95	3.79	4.16	6.39	5.5	1.45	11.4	1.72	6.87	9.72
14425	'Galnt3'	0	0	0	0	0	0	0.15	0	0	0
14426	'Galnt4'	0.7	0	1.2	0	0	0	0	0	0	0
14427	'Galr1'	8.2	18.8	23.8	0	0	75.9	45.5	51.7	49.6	60.3
14428	'Galr2'	0	2.4	17.5	0	0	0	0	0	9.4	0
14429	'Galr3'	0	0	0	13.6	0	0	0	0	0	0.9
14430	'Galt'	37.71	35.09	27.9	26.8	35.6	35.48	29.8	25.94	48.08	36.71
14431	'Gamt'	42.1	11.87	30.4	0	20	51.67	5.85	13.98	7.19	8.84
14432	'Gap43'	1080	1066	746	1410	1153	1539	817	1582	1141	1302.51
14433	'Gapdh'	2194	2252	2798	1387	2162	2788	2613	2251	1862	2094.59
14447	'Gapdhs'	0.09	0	0	0	2.07	0.03	0	1.38	0.03	1.21
14450	'Gart'	21.13	20.77	20.6	32	7.26	16.09	7.58	16.46	23.04	20.29
14451	'Gas1'	0.47	0	0.73	0	0.51	0	0.4	0	1.1	0
14453	'Gas2'	7.31	8.33	10.7	17.4	8.84	3.56	0.62	13.43	10.87	9.74
14456	'Gas6'	38.44	130.4	41.4	53.1	29.5	29.64	115	59.92	54.28	31.57
14457	'Gas7'	62.16	81.66	30.9	28.3	123	92.28	28	65.27	28.08	71.78
14459	'Gast'	21.7	172.8	0	47.3	3.59	15.57	0.12	33.26	6.71	11.71
14461	'Gata2'	0	0	0.01	0	0	0	0	0	0	0
14462	'Gata3'	0	0	0.04	0	0	0	0	0	0	0.09
14463	'Gata4'	0.01	0	0	0	0	0	0	0	0	0.01
14465	'Gata6'	0.01	0	0	0.02	0	0	0	0	0	0
14466	'Gba'	30.64	32.44	33.8	0.11	16.3	20.83	20.7	34.37	35.05	47.8
14467	'Nipsnap2'	66.85	41.97	82.1	68	71.1	49.43	47.3	56.36	65.37	84.61
14469	'Gbp2'	0	0	1.09	0	0	4.81	0	0	0	0
14470	'Rabac1'	500.3	427.1	532	564	601	723.2	603	589.8	504.4	587.72
14472	'Gbx2'	0	0	0	0	0	0	0	1.49	0	0
14473	'Gc'	0	0.03	0	0	0	0.08	0	0	0.02	0
14479	'Usp15'	37.16	32.23	35.5	16.8	43.9	58.07	44.6	43.12	37.59	42.95
14489	'Mtpn'	47.95	57.55	49	59.6	67	18.67	54.4	52.43	44.24	63.55
14528	'Gch1'	1.41	0	0	1.47	0	0	4.71	0.16	4.92	0.61
14531	'Gcm1'	0.07	0.07	0.02	0.06	0.05	2.52	3.47	0.08	0.04	0
14533	'Bloc1s1'	108.6	127.1	193	209	164	151.6	143	142.1	139.7	183.97
14534	'Kat2a'	22.47	18.9	31.7	13.2	13.5	10.96	35.5	13.93	35.55	5.63
14536	'Nr6a1'	0	0	1.04	2.01	1.27	0	0	0	0	1.61
14537	'Gcnt1'	1.43	0	0	0	0	0.27	0	0	0	0
14538	'Gcnt2'	0	1.01	2.92	0	2.24	0.01	0.55	0	0.15	0
14539	'Opn1mw'	1.96	0	0	0	0	8.33	0	0	0	4.26
14544	'Gda'	30.55	22.64	16.8	28.6	9	1.44	14.3	12.44	2.69	7.28
14545	'Gdap1'	47.34	60.31	47.6	26	40	34.57	58.2	43.49	47.34	41.88
14547	'Gdap2'	22.66	28.39	15.2	8.29	14.9	5.25	12.3	19.94	8.89	18.64

14548	'Mrps33'	167.8	167.2	155	202	215	220.7	130	167.5	195.8	209.18
14555	'Gpd1'	0.45	0.51	6.8	10.1	0.19	0.03	0	0.09	0.15	0.02
14559	'Gdf1'	21.23	0	34.1	27.5	0	0.01	0	9.67	0	0.01
14560	'Gdf10'	0	0	0.55	0	0	2.6	0	0	0	0
14561	'Gdf11'	1.49	0	0.05	0	2.98	0	0	0.67	0	1.73
14562	'Gdf3'	0.02	0	0	0	0.02	0	0	0	4.64	17.18
14566	'Gdf9'	0	4.34	2.78	0.51	0	1.22	0	0	3.37	9.1
14567	'Gdi1'	623.8	612.3	594	625	704	642.5	598	647.6	646.7	631.57
14569	'Gdi2'	76.92	77.23	103	91.1	84.7	88.38	112	81.54	91.56	90.59
14570	'Arhgdig'	160	183.6	202	292	143	173.8	119	252.1	236.3	100.2
14571	'Gpd2'	16.96	23.08	24.4	32.3	17.3	9.89	17.5	27.06	7.7	22.85
14579	'Gem'	7.03	14.4	2.64	27.3	2.18	5.55	0.04	0.43	0	2.52
14580	'Gfap'	23.87	3.15	3.32	8.85	256	21.34	4.9	6.12	13.52	22.89
14583	'Gfpt1'	3.07	1.36	1.03	0.31	1.17	1.72	1.64	2.36	1.21	1.86
14584	'Gfpt2'	0.58	3.48	2.09	0.4	0.02	0	4.77	0	0	5.78
14585	'Gfra1'	33.49	15.91	40.6	15.3	18.8	10.49	16.4	21.07	33.14	15.35
14586	'Gfra2'	5.48	33.94	58.1	36.4	12.6	8.2	1.24	30.88	3.08	3.25
14587	'Gfra3'	1.94	0	0	0	0	0	0	0	0	0
14588	'Gfra4'	46.46	38.39	50.5	50.7	32.4	42.79	52.9	35.72	71.29	17
14590	'Ggh'	16.26	33.84	18.9	23.5	14.9	18.33	32.8	17.82	14.62	17.98
14593	'Ggps1'	29.13	37.83	19.7	20.8	41.5	36.4	17.6	26.94	50.32	31.61
14594	'Ggta1'	0.79	2.89	0	0	0	0	0	0	0	0
14595	'B4galt1'	3.21	1.12	1.12	0.25	1.1	8.09	3.24	1.71	2.49	2.82
14600	'Ghr'	7.66	3.68	1.17	0.01	2.1	0.14	0.52	3.49	0.05	2.78
14601	'Ghrh'	8.82	102.5	1.5	259	0.51	5.7	0.49	22.61	0.03	0.07
14605	'Tsc22d3'	88.18	49.72	89.1	46.3	91.1	78.64	50.6	84.87	32.77	34.74
14608	'Gpr83'	5.41	1.55	33.2	0.02	2.73	10.58	47.2	38.8	0	0.01
14609	'Gja1'	36.21	60.04	2.35	54.7	14.5	21.46	0.08	27.82	27.98	14.15
14612	'Gja4'	3.58	0	0	0	0	0	6.67	6.38	0	0
14615	'Gjc1'	0.02	0	0.02	0	0	0	0	0	0	0
14616	'Gja8'	0	0.15	0.27	0	0	0	0	0	0	0
14617	'Gjd2'	3.56	2.22	0.46	0	1.38	0	0	1.89	0.25	1.19
14618	'Gjb1'	6.06	8.15	0	0	3.76	8.49	0	0	0	0.14
14619	'Gjb2'	0	0	0	0	0.8	0	0	1.01	0	0
14623	'Gjb6'	31.04	37.21	12.9	0	0.39	37.52	15.4	27.35	7.38	0
14628	'Ostm1'	33.86	31.97	23.4	16.9	22.9	22.61	11.9	37.98	38.03	57.09
14629	'Gclc'	4.17	1.3	8.57	16.4	4.45	4.05	0.53	7.18	3.65	2.48
14630	'Gclm'	4.47	6.12	2.93	4.32	4.78	1.39	3.5	1.12	2.92	0.82
14632	'Gli1'	0	0.63	0.66	0.03	0	0.74	0	0	0	0.07
14633	'Gli2'	0.01	0.03	0	0.01	0	0	0.06	0	0.05	0
14634	'Gli3'	0.93	0	0	0.18	0.64	0.96	0.01	0	1.1	0.21
14635	'Galk1'	12.49	12.08	7.02	29.7	21.8	26.88	27.5	16.26	12.04	31.47
14645	'Glul'	114.9	81.61	39.3	58.4	51.9	165	82.8	63.29	76.68	41.72
14651	'Hagh'	221.7	227.3	253	181	163	259.7	163	226.9	240.9	296.5
14652	'Glp1r'	0.76	0.69	0	0	0	0.09	0	0	0	0
14654	'Glra1'	0.4	1.06	0.4	0	0.16	0.17	0.83	0.27	0	0.07
14657	'Glra4'	0.03	0	2.85	0	0.93	0.01	0	0	0	0
14658	'Glrb'	47.91	41.55	94.9	33.3	33.2	27.65	45.9	48.19	92	46.85
14660	'Gls'	10.91	5.02	14.2	17.1	14	10.74	24.2	10.75	16.04	10.22
14661	'Glud1'	38.22	48.96	55.8	48.7	20.7	26.74	59.2	6.9	50.06	20.36
14664	'Slc6a9'	14.31	17.67	4.39	10.4	8.65	27.65	16.3	17.83	12.12	3.43
14667	'Gm2a'	17.02	12.28	40.6	19.5	9.87	14.02	43.1	19.32	6.99	7.69
14670	'Gnl1'	34.42	37.58	23.6	58.5	44.3	24.18	25.5	21.47	20.96	26.67
14672	'Gna11'	47.26	62.16	63.8	97.9	67	55.92	54.4	88.12	43.85	48.69
14673	'Gna12'	5.03	0	2.18	6.33	3.79	0.02	0.08	0	0	0.71
14674	'Gna13'	4.42	0.37	3.22	10.2	2.63	5.67	2.57	2.84	2.6	1.24
14675	'Gna14'	0	2.18	0.01	0	0	0	0	3.05	0	0
14676	'Gna15'	0	0	0	0	0.05	3.91	0	0	0	0

14677	'Gnai1'	32.55	31.04	8.72	25.5	17.2	13.27	30.9	15.58	69.38	12.35
14678	'Gnai2'	45.34	31.32	30.7	52.6	45.3	49.02	43	49.98	46.37	51.7
14679	'Gnai3'	9.53	17.3	13	7.74	13.7	0.01	7.67	9.47	5.97	13.63
14680	'Gnal'	12.89	9.85	5.8	8.24	6.82	3.65	10.5	1.93	7.39	6.67
14681	'Gnao1'	66.16	64.44	67.4	49.9	71.2	32.3	73.9	47.45	58.42	47.03
14682	'Gnaq'	44.29	51.25	27.5	48.8	39.6	37.2	31.4	31.34	28.12	32.61
14683	'Gnas'	773.3	644.3	555	630	701	817.7	664	574.2	785.5	745.97
14686	'Gnat2'	0	0	0	0	1.02	0	0	0	0	0
14687	'Gnaz'	45.4	58.85	37.1	98.5	39.2	24.69	51.4	38.43	58.3	18.62
14688	'Gnb1'	507.9	485.6	378	548	627	541.5	404	580.8	599.3	600.8
14693	'Gnb2'	38.8	29.13	33.8	62.3	56.8	26.16	44.3	39.27	55.45	43.35
14694	'Rack1'	97.93	131.6	86.1	136	88.3	83.44	80.2	110.3	105.5	113.34
14695	'Gnb3'	1.01	0	7.69	0	0	0	0.44	0.11	0	2.27
14696	'Gnb4'	5.77	28.75	17.4	5.16	17.8	3.44	20.6	11.68	52.92	37.39
14697	'Gnb5'	99.52	79.04	122	101	96.9	122.9	98.6	90.8	102.9	91.21
14699	'Gngt1'	0	0	0	0	0	0	0	0	0.05	0
14700	'Gng10'	70.48	140.1	49	231	58.6	109.9	68.3	94.91	23.48	81.87
14701	'Gng12'	2.35	2.96	5.94	11.1	1.19	1.53	4.85	3.18	3.15	1.05
14702	'Gng2'	74.96	68.38	53.9	46.4	61.6	61.02	49.5	72.74	69.21	65.8
14704	'Gng3'	586.7	512.1	509	754	526	637.4	528	585.9	475	543.11
14705	'Bscl2'	195.5	213.5	134	205	118	222.6	179	219.5	248.4	238.7
14706	'Gng4'	51.32	36.14	30.6	39.9	20.6	16.64	3.59	18.98	31.69	22.35
14707	'Gng5'	68.77	54.99	53	51.8	30.9	122.4	90	33.81	68.74	61.92
14708	'Gng7'	11.45	14.4	4.76	0.18	2.29	1.68	6.06	7.25	6.54	4.62
14709	'Gng8'	2.14	0	0	13.9	68.3	92.18	1.29	0.05	38.5	84.92
14710	'Gngt2'	8.51	12.6	0	33.6	13.5	21.6	7.08	0.09	0	5.78
14711	'Gnmt'	0	0	8.8	0	4.88	0	13	0.73	0	3.33
14712	'Gnpat'	31.13	16.89	26.1	28.8	28.5	21.26	34.7	35.71	36.34	26.85
14714	'Gnrh1'	0.94	0.28	4.52	0.99	####	84.26	9.39	1.7	1.58	1836.64
14718	'Got1'	225.5	195.4	304	155	204	433	352	323.3	259.6	251.77
14719	'Got2'	124	149.7	211	106	145	173.2	237	176.3	148.9	104
14723	'Gp1ba'	0.86	0	0.4	0	0	0	0	0	0	0.31
14724	'Gp1bb'	0.35	0	0.89	5.33	0	0	0	0.67	0.5	0.04
14725	'Lrp2'	0	0.22	0	0	0	0	0	0.01	0	0
14726	'Pdpn'	6.5	5.27	8.49	22.1	7.22	4.86	9.98	3.48	0.74	0.05
14728	'Lilrb4a'	0	0	0.03	0	0	0	0	0	0	0
14729	'Gp5'	0.92	0.44	0	0	0	4.14	1.45	1.36	6.99	0.7
14731	'Gpaa1'	44.94	34.56	52	27.3	45.8	25.61	10.3	34.49	17.69	31.57
14732	'Gpam'	2.44	2.21	2.16	5.72	0.78	1.75	2.41	2.65	4.86	2.37
14733	'Gpc1'	3.56	4.02	13.7	8.45	8.75	8.77	11.8	5.74	8.04	8.66
14734	'Gpc3'	14.91	20	49	61.5	11.6	0.04	34	46.25	2.88	14.25
14735	'Gpc4'	2.84	0.75	13.5	0.54	0.01	4.05	0	9.9	6.52	0.14
14738	'Gpr12'	3.43	1.06	6.52	0	10.3	9.45	2.83	9.34	7.6	3.88
14739	'S1pr2'	0	0.49	5.66	0	0	0	0	2.84	0	0
14744	'Gpr65'	0	0	0	0	0	0	0	0	0	0.02
14745	'Lpar1'	4.38	2.11	1.76	0	20.4	1.71	7.66	1.78	1.75	2.65
14747	'Cmklr1'	0	0	0	0	0	0.13	0	0.22	0	0
14748	'Gpr3'	0.02	1.34	12.8	0	0	0	0.16	0	0.68	3.79
14751	'Gpi1'	194	181.4	343	120	204	232.6	294	243	240.4	275.24
14755	'Pigq'	30.84	17.85	22.8	15.4	20.9	15.61	16.3	13.78	12.87	32.09
14756	'Gpld1'	32.34	18.94	22	51.6	5.52	14.5	11.9	16.19	20.98	15.18
14758	'Gpm6b'	79.31	60.21	54.9	63.7	102	97.07	93	52.18	42.15	48.58
14760	'Gpr19'	31.43	41.43	20.4	64.4	15.9	26.57	30	33.95	16.04	45.36
14761	'Gpr27'	0.68	0.91	0.17	0.41	0.62	0	0	0.11	0.35	0.12
14763	'Gpr37'	8.35	21.87	1.97	0.63	0.15	19.53	1.25	5.43	0.3	0.01
14765	'Gpr50'	0	0	0.02	32.2	0	0	0	0	0	0
14766	'Adgrg1'	33.13	58.3	44.8	24.1	52.3	29.77	43.5	33.45	32.86	64.51
14768	'Lancl1'	55.55	46.45	68.7	59.5	50	82.35	69.6	62.99	48.79	56.54

14772	'Grk4'	13.18	16.68	16.7	29.3	20.7	20.08	17.9	10.44	15.2	16.03
14773	'Grk5'	0.01	1.64	0.33	0	0	0	2	0.01	0.95	0
14775	'Gpx1'	218.6	179.2	208	212	259	242.9	211	200.3	242.9	314.26
14776	'Gpx2'	0	0	0	4.14	0	0.18	0	0	0	0
14778	'Gpx3'	973.3	1167	589	814	602	1059	595	929.7	1050	1290.07
14780	'Gpx5'	0	0	0	0	0	0	0	0	0.13	0
14782	'Gsr'	5.71	6.8	4.93	8.66	6.28	4.61	8.24	6.05	9.52	4.64
14783	'Grb10'	28.6	36.92	38.7	12.5	22	11.65	49.6	24.32	31.59	38.33
14784	'Grb2'	30.86	31.98	19.5	37.9	22.5	17.52	22.7	19.92	35.25	29.91
14786	'Grb7'	0	0.02	0	0	0	0	0	0	0	0
14787	'Rhpn1'	11.28	7.89	8.97	5.85	7.42	6.07	0.02	8.51	7.26	9.84
14788	'Gpr162'	0.72	0.66	1	1.61	2.16	0.17	1.45	1.18	5.96	1.03
14789	'P3h3'	0.37	3.6	2.93	2.64	5.84	2.17	14.2	9.33	5.21	11.52
14790	'Grcc10'	638.4	654.3	524	595	671	746	625	458.5	615.2	655.86
14791	'Emg1'	50.37	38.75	49.9	24.2	41.7	82.92	50.6	36.85	38.37	36.17
14792	'Lpcat3'	7.94	13.8	13.8	0.03	7.34	21.41	3.59	6.17	5.54	7.05
14793	'Cdca3'	0	0	0	0.12	0	0	0	0	0.06	1.82
14794	'Spsb2'	9.14	6.97	3.64	3.58	7.91	26.56	9.13	2.21	12.78	30.15
14797	'Aes'	45.74	91.2	122	73.4	93.5	76.6	156	83.34	69.81	54.74
14799	'Gria1'	33.89	48.14	44.6	31.7	31.3	20.84	31.2	39.52	59.13	43.85
14800	'Gria2'	166.8	147.7	104	94.1	162	77.3	161	146.7	185.6	174.72
14802	'Gria4'	11.88	9.32	25.4	9.9	19.6	17.93	29.1	12.52	16.61	15.85
14803	'Grid1'	9.06	7.33	13	20.4	4.84	8.3	6.51	4.06	7.52	9.69
14804	'Grid2'	3.48	2.53	7.98	0.62	4.48	5.33	3.9	1.94	3.13	1.91
14805	'Grik1'	40.4	23.7	9.88	9.59	7.49	14.2	16.3	30.54	0.04	22.58
14806	'Grik2'	5.95	8.88	6.22	0.01	7.07	2.62	6.38	7.76	7.86	1.06
14807	'Grik3'	2.01	3.42	0.54	0.68	1.45	0.14	0.01	0.8	0	0.01
14809	'Grik5'	3.41	2.98	1.71	0.18	3.05	3.98	0.95	1.31	2.04	3.41
14810	'Grin1'	99.54	65.27	138	16.4	64.5	45.53	109	61.58	91.21	36.78
14811	'Grin2a'	2.29	1.66	4.29	0	0.73	0.43	0.03	0	0.3	0.55
14812	'Grin2b'	7.39	6.28	11.4	11.2	6.12	14.15	9.37	5.17	7.07	8
14813	'Grin2c'	0	0.44	3.12	0	0	0.33	0	0	1.6	0.01
14814	'Grin2d'	0.11	0.24	0.76	1.25	0.54	0.25	0.85	0.16	1.49	0.24
14815	'Nr3c1'	15.26	7.26	16.5	16.8	5.48	5.19	5.24	7.05	8.26	7.4
14816	'Grm1'	1.31	3.4	9.23	1.01	8.83	8.68	33.2	9.85	15.8	1.37
14823	'Grm8'	0.55	4.49	8.21	1.72	3.04	5.74	7.89	14.72	0.01	1.15
14824	'Grn'	19.66	33.81	31.5	18.4	20.6	55.3	14.2	42.41	25.07	21.55
14825	'Cxcl1'	0	0	0	8.72	0	0	0.05	0	0	0
14827	'Pdia3'	245.5	302.7	155	271	180	191.4	166	168.4	259.3	312.03
14828	'Hspa5'	421.1	625	286	502	251	381.4	265	282.2	493	655.53
14829	'Grpr'	0.92	0	3.85	0	0.85	0	7.9	0.02	0	0
14840	'Gsg1'	0	0	0.08	0	0	3.76	0.04	0.7	0	0.48
14842	'Gsx1'	0	0	3.41	0	0.03	0	4.91	0	0	0
14843	'Gsx2'	0	0	0	0	2.18	0	0.03	0	0	0
14852	'Gspt1'	5.63	1.62	2.61	0.6	1.2	2.71	1.65	2.85	1.4	0.63
14853	'Gspt2'	19.2	20.93	12.3	13.5	3.83	22.43	31.8	11.09	14.75	6.61
14854	'Gss'	19.8	22.31	13.9	8.9	28.9	24.15	5.32	11.89	20.11	22.37
14857	'Gsta1'	0	0	0	0	0	0	0.06	0	0	0
14859	'Gsta3'	0	0	1.85	4.86	0.15	4.4	0	0	0.35	1.87
14860	'Gsta4'	16.41	61.56	43.6	15.5	35.1	90.76	30.9	46.03	13.65	26.46
14862	'Gstm1'	257.6	157.4	164	311	101	441.7	277	178.9	152.5	154.2
14863	'Gstm2'	0.04	6.58	0.09	5.64	0	0.14	21.2	1.94	0.04	4.18
14864	'Gstm3'	0.04	4.67	0	0	0	0	0	0	0	0
14865	'Gstm4'	22.74	29.16	23.7	50	22.9	37.93	24.6	44.3	38.8	32.34
14866	'Gstm5'	297.6	359.7	288	381	271	414.6	323	421.8	247.7	253.04
14867	'Gstm6'	142.6	138.7	159	262	97.3	111.7	64.4	139.1	83.97	71.42
14869	'Gstp2'	19.77	13.9	1.46	0.31	0.7	3.87	0.11	3.53	0	1.89
14870	'Gstp1'	1307	1327	1001	1022	1335	1747	1219	1221	1082	1368.34

14871	'Gstt1'	19.59	34.03	38.5	29.5	8.38	19.11	20.7	8.93	16.86	12.14
14872	'Gstt2'	9.08	12.35	6.78	16.2	5.88	39.35	28.7	9.83	19.1	36.51
14873	'Gsto1'	85.09	102.7	124	47.7	35.7	91.58	184	123.1	131.4	66.66
14874	'Gstz1'	40.49	50.56	45.7	47.1	29.2	72.48	9.39	51.84	50.92	28.65
14884	'Gtf2h1'	16	19.53	11.2	25.1	6.16	11.18	19.3	15.67	11.48	19.1
14885	'Gtf2h4'	21.57	8.39	19.7	0	22.2	20.25	24.6	12.66	11.14	10.51
14886	'Gtf2i'	28.8	22.32	21.3	36.4	23.1	17.19	23.8	14.84	30.61	20.69
14894	'Cfap20'	131.2	155.5	129	195	124	109.2	81.7	125.5	77.42	108.74
14897	'Trip12'	3.93	6.64	9.28	11.6	4.62	4.52	12.1	4.52	4.92	4.68
14904	'Gtpbp1'	11.29	2.07	4.4	0	3.62	1.08	6.31	3.18	4.11	3.51
14911	'Thumpd3'	26.8	25.98	40.6	28.1	19.8	6.43	16.8	26.97	27.09	24.94
14912	'Nkx6-2'	9.2	3.56	0.19	0.18	0.88	10.65	4.34	0.5	1.91	0
14913	'Guca1a'	0	0.88	0	0	0	0	0	0	0	0
14919	'Gucy2e'	0.78	0.21	0.97	0	0.79	0	0.23	0.3	0.49	0
14923	'Guk1'	283.5	289.9	291	275	310	361.3	219	317.5	216.6	303.34
14924	'Magi1'	20.09	36.77	17.1	22.6	18.8	11.19	28.2	23.14	25.61	17.66
14933	'Gk'	6.29	4.59	11	5.53	3.37	0.69	4.71	6.07	0	4.56
14934	'Gypa'	0	0.95	0	0	0	0	0	0	0	0
14936	'Gys1'	8.65	6.01	8.4	3.86	0.04	5.84	1.89	1.78	1.55	6.06
14938	'Gzma'	0	0	0	0	0	0	0	0	0.05	0
14945	'Gzmk'	0	2.17	0	0	0	0	0	0	0	0.23
14950	'H13'	133.8	122.8	63.3	98.4	81.4	121.9	115	110.7	132.9	154.06
14957	'Hist1h1d'	0	0	0	0	0	3.92	0	0	0	0
14958	'H1f0'	19.92	28.06	31.4	34.6	41.1	35.92	24.3	38.45	39.93	23.25
14960	'H2-Aa'	0	0	0	0	2.28	0	0	0	0	0
14961	'H2-Ab1'	0	0	0	0	0	9.44	0	0	0	0
14962	'Cfb'	0	0	0	0.82	0.11	2.7	0	0	0	0.2
14963	'H2-Bl'	0.12	0	3.73	4.03	7.81	0	0	0	1.78	0
14964	'H2-D1'	70.01	41.7	82.2	133	64.5	223.8	83.5	34.4	339.7	362.98
14969	'H2-Eb1'	0.05	0.03	0	6.03	0.45	0.03	0	2.18	6.79	0
14972	'H2-K1'	4.29	1.69	9.03	22.3	9.37	31.34	6.82	0.34	14.37	25.17
14976	'Pfdn6'	187.3	100.3	147	168	240	139.6	214	254.6	196.2	123.24
14977	'Slc39a7'	50	98.05	109	67.5	74.9	105.4	87.7	85.4	100.2	125.56
14979	'H2-Ke6'	10.25	4.91	9.69	32.9	19.8	25.04	12.5	18.31	24.94	11.57
14991	'H2-M3'	19.49	3.56	0	13.3	2.28	12.16	0	3.6	5.57	14.87
14998	'H2-DMa'	3.83	8.75	9.81	28.3	5.01	26.24	1.34	11.95	3.76	11.7
14999	'H2-DMb1'	0.35	0	2.52	0	0	7.06	6.25	5.36	0	0
15000	'H2-DMb2'	0	0	10.7	0	0	0	0	0	0	0
15006	'H2-Q1'	0.09	0	0.08	0.25	0.09	0.27	0.15	0.07	0.26	0.4
15007	'H2-Q10'	0	0	0	0	0	0	0	0	0.06	0
15013	'H2-Q2'	31.12	19.62	24.8	50.9	10.7	10.36	0.05	14.21	17.94	11.18
15015	'H2-Q4'	16.97	18.92	18.7	20	26.6	20.32	18.9	12.23	29.57	17.52
15018	'H2-Q7'	0	0	0	0	0	0.11	0	0.95	0.03	1.18
15019	'H2-Q8'	2.86	0	0	0	0	0	0	0.25	0	0
15024	'H2-T10'	0.92	0.22	0.95	2.56	1.3	0.94	0.47	1.45	3.15	0.99
15033	'H2-T18'	0.04	0.05	0.07	0.3	0.01	0.13	0.02	0	0.08	0.02
15039	'H2-T22'	6.47	8.05	13.9	38.1	31.2	0.06	12.9	4.42	12.04	23.61
15040	'H2-T23'	18.86	35.6	47.3	133	21.4	70.14	37.2	26.98	52.45	24.95
15042	'H2-T24'	0.89	4.6	3.38	0.79	1.85	2.67	3.57	1.49	1.58	0.86
15064	'Mr1'	0	3.94	0	0	0	6.03	0	0	1.39	1.45
15077	'Hist2h3c1'	0	0	0	0	0	0	0	0	1.04	0
15078	'H3f3a'	144.6	191.2	111	157	147	125.5	174	149.5	62.64	124.33
15081	'H3f3b'	254.5	364.1	345	508	386	430.6	292	341.4	411.8	349.91
15107	'Hadh'	8.65	0	5.84	11.7	9.8	14.25	0.03	3.18	5.43	5.15
15108	'Hsd17b10'	63.95	66.07	33.9	84.9	60.3	45.35	82.6	76.86	53.97	55.43
15109	'Hal'	0	0	0	1.67	0	0	0	0	0	0
15114	'Hap1'	225.1	282.2	282	111	209	98.42	226	354.8	345.6	272.72
15115	'Hars'	89.35	87.11	102	111	84.5	160.1	114	87.85	109.8	107.18

15116	'Has1'	0	0.11	1.06	0	0.25	0.83	4.16	0.04	6.46	2.24
15117	'Has2'	0	0	0	0	0	0	0.39	0	0	0
15118	'Has3'	0	0	0.43	0.06	0	0.34	0	1.03	0	0
15122	'Hba-a1'	6.17	0	19.9	79.1	7.89	12.27	7.05	35.78	16.83	8.37
15129	'Hbb-b1'	0	0	0	0.23	0	0	0	0	0	0.09
15132	'Hbb-bh1'	0	0.09	0	0	0	0	0	0	0	0
15139	'Hc'	0	0	0	0	0	0	0	0.34	0	0.53
15159	'Hccs'	3.01	13.66	13.5	6.92	7.45	6.37	14	5.14	9.37	7.23
15160	'Serpind1'	0	2.09	0	0	0.85	0	0	0.02	0	0
15161	'Hcfc1'	12.88	18.48	21.8	21.6	15.2	15.13	24.3	16.71	13.83	23.75
15162	'Hck'	3.07	0	1.95	0	0	4.67	5.22	2.61	0	0
15163	'Hcls1'	0	0	0	0	0	0.72	0	0	0.26	0
15165	'Hcn1'	0.96	2.59	0.78	1.07	0.91	0.43	0	0.65	1.36	0.38
15166	'Hcn2'	0.01	0	0.06	0.32	0.5	0	0.76	0	0.32	0.3
15168	'Hcn3'	7.03	6.9	5.41	3.09	2.27	0.79	2.4	3.79	3.14	0.94
15170	'Ptpn6'	1.94	0	0	5.28	0	10.82	0	0.09	0	0.11
15171	'Hcrt'	0.25	1.61	0	0.96	0	0.95	0.09	0	0	0
15182	'Hdac2'	16.14	9.2	11.3	22.6	11.7	18.37	15.1	21.11	20.23	5.86
15183	'Hdac3'	85.27	106.1	104	85.8	103	56.63	77.7	131	104.4	87.82
15184	'Hdac5'	3.07	3.55	12.7	6.54	4.74	9.79	0.8	6.99	2.69	7.23
15185	'Hdac6'	10.2	11.85	17.9	0.93	16.1	6.34	27.9	16.25	24.98	19.63
15186	'Hdc'	0	0	0.73	0	2.31	0	0	0	0	0.1
15191	'Hdgf'	48.73	64.39	42.7	44.4	59.4	59.01	85.1	47.02	59.4	57.49
15193	'Hdgfl2'	24.82	55.81	50	36.4	38.6	37.96	44.9	27.19	35.96	37.28
15194	'Htt'	1.56	1.8	0.92	0.52	1.12	3.44	2.35	1.47	3.23	0.88
15199	'Hebp1'	26.53	35.73	31.6	27.9	18.2	40.33	27.9	58.87	54.58	18.26
15200	'Hbegf'	6.58	11.39	4.06	22.2	1.69	0.04	1.93	9.13	8.92	3.7
15201	'Hells'	0.18	0.79	0.31	9.23	0.3	0.31	1.74	0.37	0.51	0.11
15202	'Gml2'	0	0	0.04	0	0	0	0	0	0	0
15204	'Herc2'	4.41	3.66	7.56	3.77	4.63	1.33	2.15	3.07	6.35	2.26
15205	'Hes1'	2.63	1.51	0.61	40.5	0	0.13	0.13	0	0.06	1.27
15207	'Hes3'	0	0	0	0	0	0	5.21	0	5.17	0.89
15208	'Hes5'	21.93	7.28	6.46	4.81	3.67	5.23	8.64	8.62	27.98	0.03
15211	'Hexa'	78	83.52	52.6	76.4	26.7	106.3	113	70.36	106.1	73.12
15212	'Hexb'	20.42	47.15	21.8	4.29	47	321.1	55.9	34.37	27.33	28.54
15213	'Hey1'	13.44	10.03	3.35	10.9	8.36	9.13	7.44	7.57	4.78	3.01
15214	'Hey2'	0	0	0	0	0	0	0	0	4.16	0
15216	'Hfe'	1.17	1.94	2	13.8	4.83	1.18	1.38	3.3	1.89	1.3
15220	'Foxq1'	0	0	0.02	0	0	0	0	0	0	0
15223	'Foxj1'	0.53	0.87	0.35	4.06	1.99	1.62	0.04	1.98	0	1.22
15227	'Foxf1'	0	0	0.02	0	0	0	0	0	0	0
15228	'Foxg1'	7.55	6.96	7.69	25.6	1.92	0	0	3.27	4.03	2.88
15233	'Hgd'	0.26	0	0	0	0	2.37	0	0	2.05	0
15234	'Hgf'	0	0	0	0	0	0.94	0	0	0	0.29
15239	'Hgs'	47.49	55.9	68.6	42.6	71.8	42.93	62.5	37.74	73.5	94.96
15245	'Hhip'	1.03	0.01	0	0	0	0	0	0.31	0.01	0
15247	'Mfsd14a'	35.67	46.46	21.6	11.7	34.6	32.11	21.7	38.64	44.25	26.88
15248	'Hic1'	0	0	0	0.77	0	0	0	0.14	0	0
15251	'Hif1a'	11.06	19.81	19.5	19.4	12.4	3.77	13.7	18.25	6.29	13.14
15254	'Hint1'	582.3	594	645	667	472	597.1	622	471.9	574.5	525.7
15257	'Hipk1'	3.17	2.66	4.88	22	0.3	2.7	6.51	0.89	1.95	4.11
15258	'Hipk2'	12.5	6.13	4.05	4.68	9.87	1.7	4.93	4.34	4.73	6.65
15259	'Hipk3'	1.48	2.82	2.19	6.53	3.89	1.07	1.66	1.8	0.13	1.31
15260	'Hira'	4.95	0.42	5.73	2.51	0.06	2.49	0.1	1.79	1.5	1.84
15267	'Hist2h2aa1'	36.69	33.84	26	52	73.4	67.92	38.8	26.11	11.04	31.62
15270	'H2afx'	76.31	66.91	49.4	44.8	37.5	45.21	68.7	69.75	53.16	69.03
15273	'Hivep2'	2.39	1.58	1.51	1.56	4.26	0.71	4.78	1.73	3.69	2.42
15275	'Hk1'	33.74	40.47	62.6	24.1	37.2	25.52	47.3	47.68	30.59	29.59

15277	'Hk2'	0.05	9.13	0.61	3.23	0.95	2.66	0.02	1.24	0.03	1.41
15278	'Tfb2m'	16.55	12.7	8.44	21.3	18	7.53	13.4	17.42	11.49	16.23
15288	'Hmbs'	41.95	24.41	10.7	30	40.6	29.42	13.6	26.62	36.74	22.59
15289	'Hmgb1'	106.3	124	131	213	143	140.8	127	149.7	133.7	135.73
15312	'Hmgn1'	148.5	120.3	74.7	118	104	81.42	85.4	91.42	96.13	98.67
15331	'Hmgn2'	239	343.9	236	588	318	284.2	168	251.3	289	298.51
15353	'Hmg20b'	2.08	1.28	3.38	3.84	4.22	3.63	1.73	1.94	2.66	2.58
15354	'Hmgb3'	39.54	56.77	90.9	110	26.7	31.24	50.1	81.63	14.91	30.43
15356	'Hmgcl'	39.99	45.81	33.6	26.1	48.5	54.02	87.9	47.37	62.52	43.53
15357	'Hmgcr'	18.19	17.35	19.8	16.7	12.9	7.43	16.5	24.97	25.71	32.33
15360	'Hmgcs2'	4.74	1.69	0	0	0	0	2.51	0	2.99	1.8
15361	'Hmga1'	64.81	19.48	59.6	42	38.3	47.51	19.7	49.32	66.38	65.44
15364	'Hmga2'	0.03	0.27	0.09	0.13	0.15	0.06	0.22	0.09	0.09	0.11
15366	'Hmmr'	0.36	0.32	0.45	0.56	0.7	0.33	0.55	0.35	0.33	0.3
15368	'Hmox1'	6.53	0.34	3.95	18.6	0	0	0	0	1.87	3.75
15369	'Hmox2'	135.3	111.7	126	130	149	144.2	146	81.63	139.8	109.54
15370	'Nr4a1'	5.11	51.75	38.4	114	2.35	9.67	19.4	7.86	10.11	25.35
15371	'Hmx1'	0	0	0	0.03	0	0	0	0	0	0
15372	'Hmx2'	4.48	1.73	7.26	0.21	0	5.44	0	5.93	1.61	1.13
15373	'Hmx3'	0.03	0.09	10.8	0.12	0	0	0	1.46	0	0.19
15374	'Jpt1'	161	162.7	151	144	148	162.7	125	188.6	161.1	185.81
15379	'Onecut1'	0	0	0	0	0.79	4.88	0	0	0.02	2.96
15381	'Hnrnpc'	175.5	173.7	114	129	89.4	170.3	138	126.1	109.5	146.9
15382	'Hnrnpa1'	108.4	178.8	143	159	88.4	129	104	138.9	136.3	121.14
15384	'Hnrnpab'	8.36	7.18	5.27	16.5	7.67	6.94	15.8	12.84	13.58	7.39
15387	'Hnrnpk'	361.5	392.8	407	457	371	377.2	333	439	352.8	361.59
15388	'Hnrnpl'	6.9	8.46	7.34	12.4	10.3	6.2	4.59	5.37	16.54	9.39
15400	'Hoxa3'	0	0	0	0	0	0.02	0	0	0	0
15425	'Hoxc6'	0	0	0	0.01	0	0	0	0	0	0
15431	'Hoxd11'	0	0	0	0	0	0	0	0.01	0	0
15436	'Hoxd4'	0	0	0	0	0	0	0	0	0.33	0
15441	'Hp1bp3'	60.99	69.41	54.2	61.5	71.4	58.37	78.9	51.03	55.83	66.99
15442	'Hpse'	0	0	0	0	0	0	0	0.3	0	0
15444	'Hpca'	22.93	14.12	62.7	20	27.2	18.7	20	82.41	14.23	1.95
15446	'Hpgd'	1.16	8.61	1.78	0	1.82	6.48	0	1.08	1.71	0
15450	'Lipc'	0	0	0	0	3	0	0	0	0	0
15452	'Hprt'	414.8	498.6	348	432	408	550.1	313	474.4	392.6	413.83
15458	'Hpx'	0.21	0	0	0	0	0	0	0	0	0
15460	'Hr'	1.19	0.01	0.5	0	0	0.03	0	2.13	0	0
15461	'Hras'	36.04	11.88	32.8	42.9	35.4	70.54	43	20.31	18.62	17.11
15463	'Agfg1'	1.79	1.79	0	0.08	1.08	0.08	2.55	0.08	2.87	2.46
15464	'Hrc'	0.02	0	0.12	0.05	0.02	0.02	0.04	0	0.02	0
15465	'Hrh1'	10.67	20.02	11.8	10.4	8.39	6.6	23.6	16.22	18.84	10.71
15466	'Hrh2'	0	0	0	3.04	0.35	0	0	2.17	1.56	2.79
15467	'Eif2ak1'	6.86	23.25	28.9	9.79	21.6	8.88	19.4	26.39	21.8	9.68
15468	'Prmt2'	339	386.1	351	365	421	525.5	307	404.8	520.8	549.6
15469	'Prmt1'	28.05	24.69	29.8	33.8	19.7	16.79	30.3	28.53	60.15	35.91
15473	'Rida'	58.15	41.71	48.9	69.8	49.9	37.91	58.7	41.64	60.57	48.75
15476	'Hs3st1'	5.14	13.48	5.26	0	26.1	47.48	2.75	37.16	5.01	13.14
15478	'Hs3st3a1'	0.01	0	0	0	0	0	0	0.04	0	0
15481	'Hspa8'	2342	2151	1859	1956	1452	1653	1935	2135	2266	2498.84
15482	'Hspa1l'	1.49	1.75	3.24	0	0	0	4.54	0	2.3	0
15483	'Hsd11b1'	10.67	5.47	0	0	0.04	0	0	1.61	0	0
15485	'Hsd17b1'	0	0	0	0	0	0	0	0	1.63	0
15486	'Hsd17b2'	0	0	0	0	0	0	0	4.32	0	0
15488	'Hsd17b4'	24.48	22.36	13.3	9.46	11.5	12.83	20.1	20.91	31.58	11.29
15490	'Hsd17b7'	1.66	0.76	2.18	3.22	2.57	0	8.29	4.17	0.63	1.47
15494	'Hsd3b3'	0	0.23	0.03	0.03	0.05	0	0.21	1.21	0.01	0.03

15496	'Hsd3b5'	0	0	0	0	0	0.2	0	0	0	0
15499	'Hsf1'	40.68	34.99	28.5	58.5	41.1	56.24	44.8	31.39	50.29	37.19
15500	'Hsf2'	22.45	33.84	18.5	39.4	30.2	11.92	8.45	24.64	18.89	23.8
15502	'Dnaja1'	194.4	221.2	156	114	147	150.4	144	185.5	181.5	174.81
15504	'Dnajb3'	5.76	4.4	3.48	14.1	9.41	10.37	7.99	7.69	6.93	2.7
15505	'Hsph1'	181.7	172.5	70.5	114	87.6	62.11	177	133.2	117	170.43
15507	'Hspb1'	18.52	0	5.5	12.8	0.17	37.5	0.46	0.14	17.15	33.99
15510	'Hspd1'	212.4	172.9	130	190	144	209.5	134	165.4	170.8	197.9
15511	'Hspa1b'	0	0	0.04	0	0	0	0	0.25	0.02	0.63
15512	'Hspa2'	0.6	8.05	2.2	33.7	9.7	15.94	4.53	3.54	2.57	10.23
15516	'Hsp90ab1'	1054	1033	742	733	942	1090	917	890	996	1153.26
15519	'Hsp90aa1'	866.3	825.4	710	855	498	719.5	568	530.7	482	674.8
15525	'Hspa4'	14.51	18.81	14.2	17.6	11.5	19.97	16	19.32	21.71	8.02
15526	'Hspa9'	72.89	80.54	102	84.1	65.1	95.98	102	107.6	94.96	103
15528	'Hspe1'	300.7	238.7	299	329	185	329.9	250	238.8	308.5	299.68
15529	'Sdc2'	32.34	65.33	46.7	52.5	24.2	22.32	47	35.49	25.55	44.7
15530	'Hspg2'	0	0.06	0.75	0	0	0	1.18	0	0	0.01
15531	'Ndst1'	4.08	1.33	4.74	8.72	1.88	0.86	4.68	1.96	3.21	4.9
15547	'Trmt2a'	51.16	34.17	41.4	47.2	40.6	58.64	26	62.46	32.2	36.54
15550	'Htr1a'	3.4	41.4	10.7	0	72.9	2.2	57.2	27.4	32.1	14.1
15551	'Htr1b'	40.6	1.3	51.9	0	79.2	44.2	41.1	40.1	87.5	34.4
15552	'Htr1d'	0	0	0.2	0	29	0	0	0	0	18.4
15557	'Htr1f'	54.4	33.2	0	74	0	0	0	0	0	0
15558	'Htr2a'	39.8	0.3	42.3	0	9.7	0	0	0	16.6	38
15559	'Htr2b'	0	4.9	0	0	0	0	0	0	0	0
15560	'Htr2c'	53.8	39.4	94.8	0	164	16.7	77.1	25.5	46.7	34.5
15561	'Htr3a'	0	0	98.3	0	88.5	0	3.4	0	0	0
15562	'Htr4'	0.1	9.8	54	0	4.9	0	3.5	9.3	0	0
15563	'Htr5a'	0	1.4	27	0	12	0.3	24.1	8.6	2.2	0.1
15564	'Htr5b'	18.4	0	0	0	116	109	45.4	0.2	0	24.5
15565	'Htr6'	0	0	0	0	12.1	0	7	0	39.8	0
15566	'Htr7'	151.1	132.9	112	0	145	98.7	1.9	106.4	34	75.8
15567	'Slc6a4'	0	1.94	0	0.04	0	0	0	0.09	0	0
15568	'Elavl1'	4.22	2.74	10.4	3.36	3.33	1.91	2.03	2.41	2.22	2.8
15569	'Elavl2'	31.91	21.59	41.8	12.5	20.8	12.64	40.6	15.93	45.28	25.9
15571	'Elavl3'	29.3	12.55	14.1	18.7	38	15.82	14.3	13.46	20.05	18.81
15572	'Elavl4'	61.74	51.92	29.7	73.8	42.4	67.34	41.3	58.47	54.66	42.35
15574	'Hus1'	4.19	1.14	0.02	4.39	0.1	6.57	4.56	0	0.95	0
15586	'Hyal1'	6.5	0	0	0	0	0	0	0	2.15	0.02
15587	'Hyal2'	17.57	17.84	3.92	0	13	17.02	29	23.83	20.41	19.29
15874	'Iapp'	0	0	0	0	0	0	0	2.59	0	0
15893	'Ica1'	59.91	50.96	55.5	62	61.2	39.53	60.7	60.62	62.25	64.21
15894	'Icam1'	0	0	0	0	0	6.16	0	0	0	0
15896	'Icam2'	0	0	0.04	0	0	0	0	0	0	0
15898	'Icam5'	6.74	10.41	4.68	16.5	8.33	0.17	7	7.44	3.29	4.68
15900	'Irf8'	0.08	0	0	5.14	0	22.15	0	0	0	0.01
15901	'Id1'	5.57	5.57	4.07	40.3	3.56	0.05	0	0.76	0	0
15902	'Id2'	56.95	119.4	72.8	189	27.3	191	48.5	37.56	29.13	51.62
15903	'Id3'	8.78	36.07	2.78	68.3	22	30.14	0	6.58	12.41	0.05
15904	'Id4'	7.93	2.8	10.9	21.7	7.51	17.8	11	7.92	5.87	5.54
15925	'Ide'	6.86	8.5	11.6	5.56	3.08	6.37	19.7	5.36	5.22	11.75
15926	'Idh1'	51.48	40.7	46	51.5	49.8	20.8	23.3	47.99	26.73	47.84
15929	'Idh3g'	296.7	264	285	321	306	506.2	315	268.2	303.1	293.4
15930	'Ido1'	1.63	4.26	3.1	0	0	17.36	3	2.73	8.25	5.03
15931	'Ids'	1.87	3.89	2.76	5.05	5.61	7.26	12.3	5.43	4.7	3.07
15932	'Idua'	8.93	11.59	19	19.4	17	13.15	20.8	13.04	15.85	10.8
15936	'Ier2'	1.64	40.98	42.3	120	0.79	14.6	32.7	4.21	0	5.28
15937	'Ier3'	3.13	26.46	29.8	28.8	17.5	20.11	19.1	11.96	7.81	6.46

15939	'Ier5'	0.78	0.81	0.12	0.89	1.31	4.26	8.08	0.94	0.01	0.41
15944	'Irgm1'	11.46	7.54	13.5	24.2	0.04	13.47	27.1	6.02	13.77	0.91
15945	'Cxcl10'	0	0	0.04	13.3	5.7	0	0	0	0	0
15950	'Ifi203'	0.01	0	0.01	0	0	0.03	0.01	0	0	0.64
15951	'Ifi204'	0	0	0	0	0	0	0	0	0	0.07
15953	'Ifi47'	0	0	0	13	0.6	0	0	0	0	0
15957	'Ifit1'	2.82	0	17.3	29.9	0	0	0	0	7.1	4.59
15958	'Ifit2'	0.72	1.71	1.52	2.71	1.4	0.24	7.95	0	2.47	2.91
15959	'Ifit3'	8.3	2.97	35	134	6.27	11.05	10.1	2.18	2.32	4.25
15975	'Ifnar1'	3.47	11.01	8.38	14	7.62	6.2	7.58	9.25	17.8	11.63
15976	'Ifnar2'	20.64	32.29	9.77	17.5	12.4	22.71	20.1	12.35	24.26	31.61
15979	'Ifngr1'	10.37	9.11	9.35	13.7	6.1	10.07	16.6	10.71	7.19	11.79
15980	'Ifngr2'	17.84	26.93	20.9	26.2	14.4	8.54	4.19	27.31	20.12	16.52
15982	'Ifrd1'	11.41	23.61	9.71	32	13.2	0.88	6.79	4.19	4.56	0.5
15983	'Ifrd2'	3.66	1.77	1.44	5.08	4.51	3.16	6.03	3.33	6.49	6.06
16000	'Igf1'	0.43	0	0.44	0	0	1.82	0	0.71	0	0.25
16001	'Igf1r'	0.06	2.34	1.01	0.91	3.07	0.95	0.69	0.26	1.31	2.48
16002	'Igf2'	0.01	0	0	0	0	0	0	0	0	0
16004	'Igf2r'	1.6	1.63	3.38	4.16	2.23	1.4	0.17	0.63	0.45	2.3
16006	'Igfbp1'	0	0	0	1.44	0	0	0	0	0	0
16007	'Cyr61'	2.54	38.51	6.17	86.6	3.43	9.64	13	2.97	0.08	0.06
16008	'Igfbp2'	0	0	3.34	0	0.05	0	0	0	2.88	0
16009	'Igfbp3'	3.47	2.37	4.84	0	3.21	3.92	5.55	3	0.05	4.47
16010	'Igfbp4'	0.02	1.56	7.88	0	2.9	0	0	2.17	0	0
16011	'Igfbp5'	2.34	0.56	12.6	11.5	7.73	6.35	0.8	2.59	5.23	1.66
16012	'Igfbp6'	0	3.19	0	0	0	0.11	0	0	1.26	0
16068	'Il18bp'	2.65	1.39	7.7	0	4.75	0	6.57	2.33	17.6	12.22
16069	'Jchain'	0.98	0.02	0.02	0.03	0	0	0	0	0	0.02
16145	'Igtp'	2.59	0	0.15	31.3	4.14	0	0.02	0	0	0
16149	'Cd74'	0.07	0	0.04	0	0	1.95	0	0.69	0	3.19
16150	'Ikbkb'	15.21	7.07	5.45	3.83	7.16	8.03	16.4	8.24	7.24	16.46
16151	'Ikbkg'	4.09	8.03	6.65	0.31	4.26	2.8	9.02	7.1	6.43	8.89
16154	'Il10ra'	0	2.15	0	3.69	0.9	8.06	0	0	0	0
16155	'Il10rb'	8.68	0	0.03	16.7	1.95	1.36	4.27	2.39	3.89	0.05
16156	'Il11'	0	1.88	0.04	1.35	0.04	0	0.04	0	0	0
16157	'Il11ra1'	8.91	23.14	17.1	30.7	34.3	51.79	11.2	0.72	22.31	53.9
16158	'Il11ra2'	0	0.14	0.05	0.34	0.26	0	0	0.64	0.09	0
16159	'Il12a'	0	2.82	0	0	4.97	0	0	0	0	0
16162	'Il12rb2'	0.7	0	1.15	5.78	0	0	0	0	0.5	0.34
16164	'Il13ra1'	7.69	13.78	4.06	12.6	5.22	3.53	7.72	5.06	6.42	7.55
16168	'Il15'	0	0	0	0	0	0	0.57	0	0	0
16169	'Il15ra'	4.36	6.05	2.86	4.57	0.13	1.6	3.82	3.88	2.25	9.47
16170	'Il16'	0	0.35	0	0	0	7.73	0	0.97	0	0
16172	'Il17ra'	0	0	0	0	0	1.51	0.01	0	5.92	4.38
16173	'Il18'	8.62	25.58	23.9	5.33	18.9	26.16	35.9	10.86	17.44	35.64
16175	'Il1a'	7.18	0.12	0	13.9	6.87	8.94	0	0	0.22	0
16177	'Il1r1'	0.92	0	0	0	0	0	0	0	0	0
16178	'Il1r2'	0	3.97	4.52	0	0	0	0	0	0	0
16179	'Irak1'	14.2	7.8	8.2	11.4	8.2	9.53	10	3.35	11.07	9.6
16180	'Il1rap'	8.89	5.11	6.23	15	9.47	3.42	5.91	14.16	2.65	4.66
16181	'Il1rn'	0	0	0.02	0	0	0	0	0	0	0
16184	'Il2ra'	0.08	0.01	0.06	0.04	0.1	0.1	0.06	0.02	0.1	0.05
16188	'Il3ra'	0	1.8	0	5.14	2.62	0	0.15	1.72	2.59	1.04
16190	'Il4ra'	0.54	3.37	2.59	0.56	0	3.98	0	0	0.01	4.27
16192	'Il5ra'	0.13	0.07	0.21	0.25	0.33	0.11	0.24	0.08	0.23	0.08
16194	'Il6ra'	0	1.03	0	0	0.14	2.33	0	0	0	2.14
16195	'Il6st'	7.1	7.29	4.06	0	3.09	4.82	5.26	1.44	7.77	2.79
16196	'Il7'	0	0	0	0	3.73	0	0.02	0	0	1.64

16197	'Il7r'	0.46	0.12	0	0	0	0.58	0	0	0.03	0
16199	'Il9r'	0	0	0.03	0.01	0	0.01	0.01	0.02	0.03	0.01
16201	'Ilf3'	55.02	64.61	42	23.1	53.5	42.61	62.4	48.85	64.3	60.37
16202	'Ilk'	24.14	25.4	38.7	34	36	22.5	41.3	30.92	32.77	16.21
16205	'Gimap1'	1.41	1.51	1.72	2.35	2.64	1.2	1.99	1.68	1.1	0.89
16206	'Lrig1'	1.16	3.43	0.01	3.43	2.18	2.79	1.26	4.05	2.02	1.57
16210	'Impact'	398.1	451.6	490	307	308	202.6	411	444.3	499.9	481.41
16211	'Kpnb1'	9.94	10.78	9.57	16.4	10.2	14.71	7.63	9.3	10.59	8.09
16319	'Incenp'	3.84	1.31	4.32	8.56	1.38	6.94	6.4	0.59	8.95	0
16322	'Inha'	2.44	12.46	8.53	16.3	17.9	6.97	9.51	5.17	3.63	11.89
16323	'Inhba'	2.89	0.84	0.53	1.24	0	0	0.22	0.17	0	0
16324	'Inhbb'	0	0	0.17	0	0	0	0	0	1.17	0
16325	'Inhbc'	0	0	0	0	0	0	0	0	0	0.41
16326	'Inhbe'	0	0.04	0	0	0	0.24	0	0	0	0
16328	'Cep250'	1.37	1.69	4.81	3.06	2.59	0.33	1.35	3.53	4.72	4.37
16329	'Inpp1'	26.93	25.77	26.9	9.14	22	32.89	15.6	24.97	25.33	14.89
16330	'Inpp5b'	9.38	6.99	11.6	12.3	1.69	1.99	4.5	6.82	3.93	14.6
16331	'Inpp5d'	0	0	2.05	0	0	1.24	0	0	0	0
16332	'Inppl1'	0.15	0	0.73	0.91	0	0	0	1.1	0	0.49
16334	'Ins2'	0	0	0	0	0	0	0	1.74	0	0
16336	'Insl3'	0	0	0	0	0	0	2.4	0	0	0
16337	'Insr'	1.52	9.53	3.94	2.56	0.75	3.01	8.92	1.94	1.84	1.16
16341	'Eif3e'	66.76	77.6	45.8	90.8	42.1	86.18	38.2	82.15	53.7	74.74
16348	'Invs'	0.91	0.8	0.75	0.87	2.3	0.16	0	0.55	1.12	5.46
16351	'Ipp'	2.91	3.22	6.31	10.5	1.08	19.93	10	4.64	7.89	15.29
16362	'Irf1'	12.94	6.78	15.5	32.1	0	4.57	4.62	3.13	8.24	5.1
16363	'Irf2'	6.46	4.3	2.61	9.78	8.65	0.98	2.37	6.68	7.91	3.11
16367	'Irs1'	0.23	0.25	2.08	0.79	0.1	0.62	0	0.06	0	0.88
16370	'Irs4'	20.45	17.07	17.1	34.2	3.89	1.32	18.8	20.96	15.23	23.55
16373	'Irx3'	0	0	0.04	0	0	0	0	0	0	0
16391	'Irf9'	1.16	6.62	14.5	4.24	5.13	7.09	11.1	4.02	3.19	8.82
16392	'Isl1'	24.99	22.03	28.5	49.1	28.2	5.16	3.56	7.47	27.56	2.96
16396	'Itch'	4.29	2.13	7.04	0.56	4.14	3.46	1.01	4.97	9.45	3.33
16398	'Itga2'	0	0	0.85	0	0	0	0	0	0	0
16399	'Itga2b'	1.11	0.36	0.94	4.53	2.01	4.58	0.65	0.68	0.48	1.93
16400	'Itga3'	3.11	11.43	9.07	14	8.54	6.33	6.45	9.35	26.69	17.42
16401	'Itga4'	0.18	0.3	0.41	0.4	0.38	0.21	0.23	0.79	0.29	0.32
16402	'Itga5'	0	0.16	0.01	0.01	0.07	0.14	0	0	0	0.01
16403	'Itga6'	0.14	0.6	0.92	3.85	0.84	0.03	0	0.16	0.1	0.83
16404	'Itga7'	0.02	0.01	0.04	0	0.14	0.02	4	0.01	0.86	1.1
16409	'Itgam'	0	0	1.23	0	0	0.23	0	0	0	1.32
16410	'Itgav'	0.07	0.43	3.3	1.86	0.48	0.02	1.1	0.09	0.15	0
16412	'Itgb1'	12.35	2.48	7.54	10.7	14.5	3.01	6.86	6.96	7.39	10.39
16413	'Itgb1bp1'	33.64	21.72	20.6	82.3	37.1	29.83	78.4	32.23	16.9	21.76
16414	'Itgb2'	0	0	0	0	0	5.89	0	0	0	0.08
16416	'Itgb3'	0	0	1.16	0	0	0	0	0	0	0
16418	'Eif6'	96.33	85.53	77.8	91.4	86.2	114.6	78.8	76.07	79.54	78
16419	'Itgb5'	20.01	5.2	10.1	0	30.8	58.03	80.8	101.2	50.03	40.01
16420	'Itgb6'	0	0	0	0	0.01	0	0	0	0	0
16421	'Itgb7'	0	0	0	0	0	0	0.49	0	0	0
16423	'Cd47'	135.5	152.9	120	72.6	114	103.4	154	118.1	148.3	129.94
16425	'Itih2'	0	0	0.05	0	0	0	0	0	0	0
16426	'Itih3'	151.8	154.1	44.4	135	13	148.7	41	43.26	91.23	48.84
16428	'Itk'	0	0	1.18	5.43	0	0.04	0	0	0	0
16430	'Stt3a'	16.22	20.38	22.1	38.5	24.7	7.93	18	18.1	27.34	20.62
16431	'Itm2a'	12.24	1.07	7.52	27.8	11	18.37	36.1	11.68	6.37	7.49
16432	'Itm2b'	698.3	918.6	742	864	884	961.6	742	792.9	736.2	951.29
16433	'Cuzd1'	0	0	0	0	0	0	0	0.05	0	0

16434	'Itpa'	92.05	60.99	90.5	76.9	72.6	73.06	89.8	71.82	61.7	101.6
16438	'Itpr1'	5.5	5.55	6.05	6.88	3.3	1.84	1.9	0.26	2.39	2.11
16439	'Itpr2'	0.28	1.45	0.82	2.01	0	0.59	1.63	0.59	1.48	0.03
16440	'Itpr3'	0	0	0	1.33	0	0	0	0	0	0.18
16443	'Itsn1'	8.14	6.35	4.2	3.03	3.72	3.15	4.05	3.62	5.84	3.14
16449	'Jag1'	0.06	0.31	2.1	1.96	0.14	0	0.48	0.08	0.24	0
16450	'Jag2'	3	8.12	14	12.4	2.37	7.96	8.25	3.05	5.91	0.02
16451	'Jak1'	27.41	25.94	31.3	27.4	17.9	12.9	55.2	23.74	22.37	45.33
16452	'Jak2'	0.9	3.1	6.03	10.2	0.21	5.2	3.76	1.34	1.92	1.99
16453	'Jak3'	1	1.7	0	0	0.96	0	0.01	0.64	0	0.05
16456	'F11r'	0.46	0	0.04	0	35.2	23.24	0.02	0	0	5.69
16467	'Atcay'	85.62	76.83	93.9	62.3	96.8	82.21	115	117.6	86.35	87.49
16468	'Jarid2'	1.4	2.79	4.57	7.73	2.15	2.2	0.71	2.74	5.04	4.54
16469	'Jrk'	2.28	0.9	0.96	0.01	2.78	1.42	1.77	1.36	0	1.86
16470	'Ush1g'	1.04	0	0	0	0	0	0	0	0	0
16475	'Ajuba'	0.05	0.01	0.07	0	0.33	0	0	0	0.02	0
16476	'Jun'	83.55	162.4	259	163	65.8	180.1	179	98.2	61.77	106.84
16477	'Junb'	100.9	281.6	143	311	10.2	150.6	121	87.83	65.88	194.1
16478	'Jund'	5.05	3.76	4.56	12.5	3.19	5.97	6	3.09	3.98	4.63
16480	'Jup'	8.99	10.54	3.19	6.67	7.48	0.73	2.43	2.95	4.75	13.87
16485	'Kcna1'	3.21	1.01	3.07	3.3	9.19	0.97	0.47	1.25	0	0.01
16490	'Kcna2'	1.36	1.33	2.54	0	4.26	0.92	1.91	1.21	1.09	1.33
16491	'Kcna3'	3.41	0.66	0.73	0	2.11	0	1.81	0.8	3.49	7.31
16492	'Kcna4'	1.25	2.89	0.19	0.32	3.07	1.71	1.97	2.75	4.77	2.73
16493	'Kcna5'	6.69	5.7	1.7	3.79	5.59	2.13	0.03	6.14	1.44	9.79
16494	'Kcna6'	6.91	3.26	15.1	2.81	8.18	5.79	15.7	7.08	13.23	1.79
16497	'Kcnab1'	21.68	20.91	47.7	23	15.6	14.51	41.4	23.33	16.03	7.02
16498	'Kcnab2'	13.45	7.11	33.9	6.23	28.6	7.68	25.9	20.21	6.14	8.24
16499	'Kcnab3'	4.72	0.25	5.41	0	15.7	0.23	0.04	0	0.24	0.17
16500	'Kcnb1'	0.86	0.8	1.51	0.08	5.34	2.67	0.38	1.67	0.41	1.3
16502	'Kcnc1'	3.51	4.38	6.19	15.7	18.2	9.36	14.5	9.24	5.22	3.35
16504	'Kcnc3'	0.54	0.04	0.42	1.25	1	0.71	0.65	0.02	0.54	0.11
16506	'Kcnd1'	0.72	3.1	1.43	0.56	1.67	4.47	5.28	2.12	0.98	2.58
16508	'Kcnd2'	16.2	5.87	8.89	12.9	4.71	6.24	9.46	9.19	1.82	6.7
16509	'Kcne1'	0	0	0	0	0	0	0	0	0.01	0
16510	'Kcnh1'	2.29	0.91	2.41	0	1.72	2.91	4.44	4.46	3.22	1.3
16511	'Kcnh2'	3.27	1.71	2.7	1.52	9.17	5.21	0.03	0.31	2.4	5.34
16513	'Kcnj10'	3.57	1.39	0.02	3.65	1.69	0.42	0.07	2.5	1.54	0.14
16514	'Kcnj11'	0.07	0	0.02	5.98	3.82	4.73	0.47	0.92	4.21	0
16515	'Kcnj12'	1.26	0	2.16	0	1.06	0.02	0.01	0.4	0	0
16517	'Kcnj16'	7.86	0.17	8.18	0.09	0.32	5.8	0	5.34	4.42	11.15
16518	'Kcnj2'	1	0	0	0	0	0	0	0	0	0
16519	'Kcnj3'	8.67	7	5.37	11.4	3.46	1.19	4.13	5.25	3.51	4.92
16520	'Kcnj4'	0.02	0	0	0	1.92	0	0	0	0	1.03
16521	'Kcnj5'	0	3.62	0.03	0	0	4.45	1.41	0	2	5.72
16522	'Kcnj6'	4.1	9.29	1.6	9.91	3.9	15.03	4.74	3.68	17.18	0.82
16523	'Kcnj8'	0.02	0	0.02	0	0	0	0	0	0	0
16524	'Kcnj9'	17.51	18.83	26.6	2.72	15.5	26.98	24.4	16.8	22.86	32.75
16525	'Kcnk1'	31.34	30.31	36.8	11.9	44	28.64	28.1	52.04	24.7	30.75
16526	'Kcnk2'	5.98	14.73	7.78	7.97	2.13	1.99	17.1	11.91	6.99	3.95
16527	'Kcnk3'	0.13	1.18	1.54	0.04	7.59	3.51	5.89	2.36	5.46	3.36
16528	'Kcnk4'	0	0.55	0	0	2.32	2.03	5.67	3.89	3.83	1.57
16530	'Kcnk7'	0	1.58	0	0	0	0	0	0	0	0
16531	'Kcnma1'	1.77	9.42	7.75	13.1	4.5	6.9	11	4.81	3.83	3.39
16532	'Kcnu1'	0	3.42	0	0	2.54	1.9	0	1.17	2.01	1.56
16535	'Kcnq1'	0	1.18	0	0	0	0	0	0	0	0.05
16536	'Kcnq2'	6.04	6.65	5.31	15.6	10.1	7.4	7.19	4.66	7.85	3.61
16538	'Kcns1'	0	2.43	4.01	0.04	4.71	4.39	1.39	0	12.03	7.22

16539	'Kcns2'	0.79	2.08	9.15	3.37	0.37	0	3.28	3.2	0	3.34
16541	'Napsa'	0	0	0.26	0	0	0.09	0.82	0	0.44	0.47
16542	'Kdr'	0	0	0	0	0	0	0	0	1.05	0
16543	'Mdfic'	0	0	0	0	0	0.92	0	0.63	0	0
16545	'Kera'	0	0	0	0	0	1.13	0	0	0	0
16548	'Khk'	9.57	21.23	9.34	40.5	13.5	16.57	10.9	34.47	18.23	15.83
16549	'Khsrp'	1.78	0.85	1.99	2.28	4.02	1.76	4.24	1.65	2.34	1.94
16551	'Kif11'	0.76	0.1	1.99	2.51	0.45	0.1	0	0.33	0.29	0.4
16553	'Kif13a'	0.19	2.02	0.71	2.97	1.31	0.01	0.01	0.34	1.09	0.13
16554	'Kif13b'	1.01	1.42	0.31	0	2.16	0.81	0.51	1.01	0.35	0.95
16558	'Kif16b'	0.31	0.61	0.15	0	0.76	0.01	0	1.89	0.01	0.01
16559	'Kif17'	2.84	7.73	7.52	0.04	8.9	6.16	7.86	2.77	6.76	8.98
16560	'Kif1a'	56.3	49.14	72.1	56	53.7	44.29	57.2	31.74	35.55	66.09
16561	'Kif1b'	18.29	11.89	20.2	33.2	19.7	11.4	16	8.23	11.12	11.94
16562	'Kif1c'	0.56	0.56	0.65	0	0.23	1.06	1.26	0.28	2.28	0.33
16563	'Kif2a'	4.68	8.46	4.38	10.2	10.8	8.43	9.91	7.21	5.61	12.23
16564	'Kif21a'	36.49	48.76	35.6	39.3	37.8	45.76	43.3	37.38	28.17	31.64
16565	'Kif21b'	1.41	2.25	4.37	3.07	0.69	5.91	0.62	1.29	1.52	1.23
16568	'Kif3a'	73.07	71.92	71.2	61.7	66.9	75.33	75.3	86.3	56.74	82.84
16569	'Kif3b'	2.99	4.61	2.72	2.46	4.38	9.31	5.02	4.33	1.6	1.65
16570	'Kif3c'	15.94	12.24	31.2	15.7	6.91	4.63	8.75	15.97	20.33	12.98
16571	'Kif4'	0	0.02	0	0	0	0	0	0	0	0
16572	'Kif5a'	27.59	20.08	25.5	12.2	32.8	26.36	37.3	27.09	45.29	21.7
16573	'Kif5b'	35.27	35.76	21.6	42.9	57.9	28.64	31.1	27.73	32.98	36.16
16574	'Kif5c'	28.37	35.22	30	21.8	36.7	19.4	26.6	26.14	24.2	16.57
16576	'Kif7'	0	0.45	0	0	0.63	0.33	0.14	1.09	0.88	0
16578	'Kif9'	1.69	4.63	2.75	0	0.45	6.25	0	0.95	0.02	8.98
16579	'Kifap3'	62.46	50.98	77.1	31.6	47.9	51.88	84.3	65.82	62.05	55.16
16580	'Kifc5b'	3.8	0	0.35	0	0.91	4.2	0	2.56	0.53	0.02
16581	'Kifc2'	37.73	44.65	87.1	28.7	65.7	38.06	53.3	66.31	51.94	35.41
16582	'Kifc3'	11.7	3.93	8.73	4.09	4.99	4.43	21.4	4.18	8.71	4.56
16588	'Kin'	17.38	12.9	8.42	13.6	18.5	8.84	2.17	8.66	6.05	10.15
16589	'Uhmk1'	1.83	0.14	1.9	0.58	0	0	0	1.65	0	1.07
16590	'Kit'	0.02	3.23	3.86	0	3.89	0.38	3.78	0.49	6.38	3.65
16591	'Kl'	1.53	1.42	0	2.03	0.01	0.02	0.01	0	0	1.4
16592	'Fabp5'	127.5	113	158	222	97.8	144.3	127	176.8	100.9	69.73
16593	'Klc1'	314.7	291.7	317	313	279	226.8	386	261.6	248.7	175.6
16594	'Klc2'	6.7	4.55	9.31	3.81	11.7	1.35	7.54	7.81	16.48	6.07
16596	'Klf1'	0	0	0	0	3.57	0	0	0.9	0	0
16597	'Klf12'	4.81	2.26	7.27	2.8	4.83	7.25	15.2	4.78	3.24	6.77
16598	'Klf2'	0	3.16	2.13	0.06	0	0.03	0.03	0	0	0
16599	'Klf3'	0.11	0.88	4.09	0	0.01	3.16	0.02	0.05	1.47	2.72
16600	'Klf4'	0	2.3	2.69	13.7	0.42	6.61	0.91	0.85	0	3.59
16601	'Klf9'	6.04	3.86	5.29	5.57	5.22	0.57	4.69	4	0.03	0.25
16612	'Klk1'	4.48	0	0	0	0	0	0	0	0	0
16618	'Klk1b26'	0	0	0.05	0	0	0	0	0	0.05	0
16619	'Klk1b27'	0	0	0	0	0	0	0	0	0	3.52
16625	'Serpina3c'	0	0	0.19	0	0	0	0	0.07	0	0
16633	'Klra2'	1.43	0.23	0	0	0	0	0	0	0	0
16646	'Kpna1'	20.06	10.75	22.4	27	16.3	3.62	15.8	18.8	20.16	18.1
16647	'Kpna2'	56.36	16.26	61.8	42.1	25.3	64.41	20.3	53.03	31.53	57.47
16648	'Kpna3'	34.59	23.56	21.8	14.7	16.4	7.19	17.8	25	14.89	15.63
16649	'Kpna4'	1.29	4.04	4.99	7.68	3.85	1.99	3.38	3.89	7.33	1.84
16650	'Kpna6'	5.7	4.7	0.97	2.75	4.89	1.44	0.52	5.27	1.54	3.87
16651	'Sspn'	3.44	3.99	1.88	0.01	3.46	1.77	0.02	1.59	2.42	3.65
16653	'Kras'	5.5	7.69	5.74	13.3	6.13	5.12	3.22	2.31	2.68	3.41
16656	'Hivep3'	9.4	14.15	3.79	18.2	6.05	7.56	9.69	2.03	17.26	4.61
16658	'Mafb'	0.46	0	0.03	3.77	0.73	4.24	2.8	1.4	0	0.02

16661	'Krt10'	4.07	6.81	1.31	0	5.19	10.8	8.47	2.35	4.28	7.56
16667	'Krt17'	0	9.98	0.03	0	0	3.42	0.2	2.61	0	4.56
16668	'Krt18'	0	5.23	0	0	0	0	0	1.46	5.36	0
16669	'Krt19'	0.05	2.79	0	0	0	7.2	0	0	0	3.83
16673	'Krt36'	0.1	0	0	0	0	0	0	0	0	0
16675	'Krt27'	0	0	0	0	10.3	0	0	0	0	0
16678	'Krt1'	6.61	4.12	0.88	13.8	2.28	6.51	1.65	7.4	5.28	4.8
16680	'Krt84'	0	0	0	0	0	0	0	0	0.18	0
16681	'Krt2'	0	0	0	0	0	0	3.27	0	0	0
16691	'Krt8'	0	0	0.03	0.03	24.3	0	0	0	0	0.07
16694	'Krtap12-1'	0	0.08	0	0	0	0	0	0	0	0
16706	'Ksr1'	1.88	1.11	0	3.38	1.49	0	1.96	0.1	2.03	0.26
16709	'Ktn1'	10.64	14.11	17.1	16.5	12.1	9.91	7.56	8.39	12.55	12.59
16716	'Ky'	0	0	0	0	0	0	0	0	0	1.08
16728	'L1cam'	24.05	18.29	44.9	6.97	32.3	6.61	37.4	26.18	27.5	45.9
16763	'Lad1'	0	0	2.01	0	0	0	0.02	0	0	0
16764	'Aff3'	1.26	0.82	1	0.18	3.34	0	0.66	1.46	1.74	1.08
16765	'Stmn1'	1226	931.3	993	1077	1525	1982	1264	1157	1240	1341.45
16768	'Lag3'	0.77	0.67	2.16	0	6.38	0	0	0.02	0	0
16772	'Lama1'	0.04	0	0	0	0	0	0	0.51	0	0
16773	'Lama2'	0.23	0.01	0	1.01	0	1.91	0	2.07	0.49	0
16774	'Lama3'	0.61	0	0	0.16	0	0	0.09	0.35	0	0
16775	'Lama4'	0	0	0.75	0.01	0	0	0	0	0	0
16776	'Lama5'	0	0.17	0.07	0	0.31	0.04	0	0	0	0
16777	'Lamb1'	0.94	3.13	1.95	1.95	0.83	1.44	0	1.3	0	2.67
16779	'Lamb2'	0.87	0.49	0.01	0	0.76	0.83	0.01	0	0.33	0.36
16780	'Lamb3'	8.37	60.5	1.54	30.3	1.02	9.02	0.05	3.64	0.03	3.06
16782	'Lamc2'	0	0.2	0.01	4.15	4.11	0	0.51	0.01	0	0.07
16783	'Lamp1'	70.76	29.41	65.2	186	73.7	24.4	59.8	12.38	37.26	16.81
16784	'Lamp2'	34.09	29.66	29.2	40.7	28.1	31.09	37	16.67	16.48	41.66
16785	'Rpsa'	145.4	102.6	146	76.1	173	165.5	111	125.4	103.1	90.91
16792	'Laptm5'	2.33	7.36	2.5	5.32	31.2	46.48	0	2.87	0	0
16795	'Large1'	1.96	6.47	6.86	5.5	2.31	0.42	3.69	4.71	0.01	4.93
16796	'Lasp1'	23.48	27.6	34.3	14.4	29.8	21.23	31.1	12.29	34.57	26.95
16797	'Lat'	0	0	0	1.83	0	0	2.11	0	1.42	0
16798	'Lats1'	0.4	1.2	0.07	1.52	1.26	1.51	0	0.75	1.94	1.07
16800	'Arhgef2'	8.06	3.91	11.3	7.99	11.2	5.43	6.89	3.21	8.63	15.07
16801	'Arhgef1'	6.25	10.54	6.61	12	9.05	7.39	4.4	4.14	8.12	7.15
16803	'Lbp'	0	0	0	0	0	0	0	0.03	0	0
16816	'Lcat'	8.08	4.27	2.55	8.57	0	4.72	0	0	6.61	6.7
16818	'Lck'	0.35	2.93	1.97	0	4.36	0.75	1.4	2.47	0.36	2.09
16822	'Lcp2'	2.22	2.84	3.46	6.01	0	6.78	2.54	0.4	0.04	1.22
16825	'Ldb1'	8.72	18.19	13.2	13.8	16.7	7.48	25.4	18.73	13.75	20.97
16826	'Ldb2'	34.69	19.72	26.3	11.5	44.9	16.47	37.6	28.19	5.94	11.4
16828	'Ldha'	410.9	462.9	443	381	441	543.1	510	475	420.6	612.17
16832	'Ldhb'	630.5	489.8	819	806	774	1101	714	843.6	756.4	612.38
16833	'Ldhc'	0	0	0	0	0	0	0	0	0	0.04
16834	'Cog1'	34.53	27.34	29.1	1.78	20.5	12.77	28.4	15.15	30.48	7.5
16835	'Ldlr'	1.6	4.5	8.19	8.97	1.32	0.03	2.63	2.47	0	1.26
16840	'Cnmd'	3.42	0	0	0	5.51	0.21	7.03	0	3.14	4.29
16841	'Lect2'	0	0.52	0	2.35	0.65	0	0	0	0.02	0.34
16842	'Lef1'	0	0	0	0	0	0	0	0.02	0	0
16847	'Lepr'	1.1	2.1	1.8	0	2.6	4.3	3.6	1.9	7.7	7.1
16848	'Lfng'	2.58	0.36	0.04	0.1	0	0.16	0	0.24	0.88	0
16852	'Lgals1'	0.35	4.81	2.98	11.5	7.13	5.34	0.2	1.33	1.08	1.81
16854	'Lgals3'	0	1.63	0	0	4.41	0	0.04	2.46	3.14	0
16855	'Lgals4'	0.44	13.28	3.32	1.29	0.03	0.03	0	0	0.61	5.28
16858	'Lgals7'	0	0	0	10.4	0	0	0	0	0	0

16859	'Lgals9'	0	0	0.03	27.9	9.82	33.64	0	0	0.06	3.3
16865	'Eif2d'	21.2	18.73	33.1	19.7	14.9	27.39	52.4	27.44	26.74	17.86
16867	'Lhcgr'	0	0	0	0	0	0	0	0	0.97	0
16869	'Lhx1'	1.33	0	0	0	0.25	3.01	3.4	0.1	2.04	3.95
16870	'Lhx2'	1.67	1.41	3.53	0.03	4	13.13	0.06	0	3.34	5.33
16872	'Lhx4'	0	0.01	0.4	0	0	0	0	0.29	0	0
16873	'Lhx5'	7.15	1.85	10.3	8.47	3.25	18.41	4.34	5.94	11.13	8.96
16874	'Lhx6'	0	10.37	0	0	4.73	0.2	7.99	3.58	0	0.01
16875	'Lhx8'	0.02	0	0	0	31.5	6.08	7.16	0	0	0
16876	'Lhx9'	0.88	1.93	6.04	8.75	12.8	28.41	4.45	0.33	7.65	21.13
16880	'Lifr'	10.85	3.2	3.89	3.98	2.55	1.19	4.27	2.73	5.61	3.55
16881	'Lig1'	9.05	6.89	14.3	0.51	5.81	9.23	7.79	5.32	8.93	9.77
16882	'Lig3'	3.8	1.26	5.27	1.12	7.88	0	1.65	2.68	5.92	4.01
16885	'Limk1'	3.37	5.78	3.62	0	2.07	0.84	1.23	6.14	0.03	0.98
16886	'Limk2'	47.7	51.19	42.2	13.2	39.7	25.7	61.3	57.68	50.99	31.92
16889	'Lipa'	5.72	9.5	3.05	0	2.24	5.74	6.91	13.91	11.91	8.64
16890	'Lipe'	1.04	3.23	0.96	0.59	8.46	10.6	12.3	6.75	5.07	4.59
16891	'Lipg'	3.06	0.24	0.01	0	0	0	0	0	0	0
16897	'Llgl1'	13.64	3.52	3.26	6.41	2.43	3.69	0.54	1.53	6.96	2.4
16898	'Rps2'	104	161.9	187	250	129	53.15	113	64.17	126.2	47.51
16904	'Gzmm'	0	1.9	0.98	2.32	0	0	0	1.13	6.63	0.79
16905	'Lmna'	45.65	48.91	53.1	99.6	31.3	34.42	20.2	32.51	40.83	44.43
16906	'Lmnb1'	0.57	2.78	3.93	1.07	2.22	0	0	1.45	0	2.88
16907	'Lmnb2'	1.71	6.97	11.7	6.44	6.63	0.59	6.59	6.03	1.58	2.03
16909	'Lmo2'	1.32	0	3.88	16.6	1.61	4.08	4.84	0	3.18	0.2
16911	'Lmo4'	9.33	11.17	17.6	11.9	31.9	27.65	30.6	35	7.81	17.47
16912	'Psmb9'	0	0	0	13.2	5.29	10.23	0	0	4.52	0.06
16913	'Psmb8'	3.25	2.49	3.18	5.35	0	1.46	0.04	10.08	8.35	6.54
16918	'Mycl'	2.71	5.61	5.87	18.4	2.22	2.8	0.08	6.18	1.95	5.87
16922	'Phyh'	137.6	141.9	125	142	178	199.6	143	144.2	122.9	199.79
16923	'Sh2b3'	0.9	1.27	0	4.41	0.06	0	0	0.18	0.04	3.99
16924	'Lnx1'	16.73	24.31	18	10.2	12.2	15.51	50.9	18.25	23.17	24.73
16939	'Lor'	0.77	0.81	0.38	8.99	1.56	3.87	2.72	1.07	0.15	0.7
16949	'Loxl1'	2.64	0.26	6.07	6.02	8.77	25.04	0.01	7.79	17.82	6.14
16950	'Loxl3'	0.35	0	1.89	0	1.75	0.46	0.01	0.32	0.33	0
16956	'Lpl'	0.22	1.12	0.85	0.14	1.87	0.25	0.16	0.24	0.23	0.28
16969	'Zbtb7a'	7.43	3.79	6.13	5.91	7.96	0.95	6.22	2.77	4.66	3.39
16971	'Lrp1'	9.05	6.93	19	9.16	9.01	5.81	15.1	7.67	8.51	5.98
16973	'Lrp5'	0	0	0.04	0	0.01	0	0	0	0	0
16974	'Lrp6'	0.61	0.21	0.13	0.68	0.1	0.1	2.51	1.04	0.48	0
16975	'Lrp8'	0.77	5.33	1.28	8.16	1.65	3.17	3.25	1.63	3.33	1.78
16976	'Lrpap1'	287.3	434.8	218	378	261	426.1	277	365.8	501.9	558.69
16977	'Lrrc23'	0.03	5.45	0.82	0	0.42	0.74	0	4.72	0.11	6.64
16978	'Lrrfip1'	30.57	19.38	17.3	63.5	27.3	6.81	31.5	20.79	24.54	14.97
16979	'Lrrn1'	7.06	9.61	16.4	7.44	4.19	0.92	36.1	6.37	3.97	4.16
16980	'Lrrn2'	33.55	33.86	46.5	17.3	39.1	26.43	30.8	53.13	54.71	27
16981	'Lrrn3'	43.54	25.96	14.5	15.9	27.3	13.23	21.8	37.33	11.73	43.72
16985	'Lsp1'	0	0	7.93	0	4.09	0.97	0	0	4.72	1.81
16987	'Lss'	8.22	3.5	10.6	18.7	7.33	1.51	0.03	0.27	6.33	0.46
16988	'Lst1'	0	1.45	2.38	0	3.78	1.67	20.6	0.22	0	2.03
16992	'Lta'	3.59	0.04	3.91	0	0	0	5.64	0	0	0
16993	'Lta4h'	39.53	44.11	67	61.6	25.1	42.44	23.4	33.77	50.25	56.22
16994	'Ltb'	0	0	0.05	0	0	0	3.81	0	0	2.61
16997	'Ltbp2'	0	0	3.15	1.42	0	0	0	0.37	0	0
16998	'Ltbp3'	4.86	10.79	3.9	8.02	3.23	0.71	5.93	2.8	5.36	2.73
17000	'Ltbr'	0	0	6.07	0.46	0	0.04	0	0	0.02	4.16
17001	'Ltc4s'	3.61	0	0	0	10.2	8.19	0	3.54	0	11.18
17005	'Ltk'	0	0	0	0	2.86	0	0.18	0	0	0

17025	'Alad'	53.11	52.84	67.9	63	57.8	65.4	66.4	68.28	80.88	49.91
17035	'Lxn'	64.82	51.55	20.8	40.6	18	84.44	68.1	66.86	60.23	69.31
170439	'Elovl6'	8.09	8.87	8.76	6.88	7.21	12.26	9.54	15.02	19.09	7.58
170441	'Slc2a10'	0	0	0	0	0.01	0	0	0	0	0
170442	'Bbox1'	3.28	5.01	0.03	7.4	0	1.73	6	0	0	2.14
170459	'Stard4'	12.05	13.31	5.58	7.71	14.8	5.5	23.6	13.56	4.21	7.04
170460	'Stard5'	0.2	1.89	0.85	0.07	4.77	0	0	1.25	0	0.03
170461	'Stard6'	0.03	3.83	0	0	0	0	0.11	0.32	0.03	0
170472	'Recql5'	14.65	13.64	10.4	4.5	4.91	0.06	13.7	4.23	4.05	16.17
170483	'Grin3b'	0	0	0.31	0	0	0	0	0	0	0
170484	'Nphs2'	0	0	0	3.61	0	0	0	0	0	0
170571	'Cntnap4'	40.62	21.19	16	11.5	21.2	7.02	30	30.93	36.69	32.17
170574	'Sp7'	0	0	0	2.04	0	0	0	0	0	0
17060	'Blnk'	0	0	0	0	0	2.31	0.02	2.58	0	0
170625	'Snx18'	0.6	0.96	1.38	2.3	1.94	0.6	0.45	1.17	0	1.61
170638	'Hpcal4'	110.5	143.7	60.5	79.7	67.9	92.18	87.5	112.7	46.9	80.93
17064	'Cd93'	0	0	0.01	0.02	0.02	0.01	0	0	0.01	0.11
170643	'Kirrel'	0	0.06	0	0	0	0	0.03	0.12	0.17	0
170644	'Ubn1'	1.58	0.56	2.21	6.46	2.69	0.92	0.91	0.21	1.37	2.71
17067	'Ly6c1'	0.05	0	0.12	0	0.91	0	0.06	0	0	0
170676	'Peg10'	2.95	1.72	1.58	0.07	0.2	2.24	7.16	3.79	10.58	13.92
170677	'Cdhr1'	0	0.53	2.37	0	1.52	0.01	0.01	0	4.33	2.17
17069	'Ly6e'	164.8	150.7	116	193	104	272.7	118	151.2	176.9	166.71
170706	'Tmem37'	0.04	0	0.05	0	0	0	0	0	0	0
170707	'Usp48'	6.46	10.2	10.8	15.8	15	3.94	9.96	4.55	11.11	4.96
170711	'Otud7a'	0	0.21	0.01	1.21	0.35	2	1.44	0.69	0.17	0.74
170716	'Cyp4f13'	0.39	0	4.15	6.12	4.11	0	12	4.73	4.93	11.95
170718	'Idh3b'	271.6	233.3	422	221	336	348.4	401	325.2	304.7	312.7
170719	'Oxr1'	31.08	21.05	48.4	38.2	35	41.48	43.3	41.69	40.4	55.26
170721	'Papln'	0	0	0.19	1.69	0.35	0	0	0.26	0.6	1.03
170728	'Rtn4ip1'	13.99	5.36	9.58	5.62	15.4	9.62	3.18	11.43	5.2	19.96
170729	'Scrt1'	0	0	0.08	0	0	0.01	0	0.06	0.73	0
170731	'Mfn2'	25.34	29.83	66.6	18.6	25.5	8.02	61.8	42.51	52.54	37.32
170732	'Trhr2'	0	0	0	0	0	0	0	2.34	0	0
170734	'Zscan5b'	0.02	0.02	0	0	0.1	0	0	0	0.02	0.02
170735	'Arr3'	0	0	0	0	0	0	0	0	0	2.64
170736	'Parvb'	0.07	1.61	0.24	2.86	1.06	0	0	1.33	2.42	3.46
170737	'Znrf1'	12.07	13.14	13.2	15.9	11.7	15.19	13.3	10.35	8.33	9.93
170738	'Kcnh7'	6.09	2.03	1.97	5.03	2.4	3.45	3.85	3.32	2.21	10.83
170740	'Zfp287'	2.25	3.88	2.49	1.61	2.42	3.86	11	4.09	2.07	10.3
170741	'Pilrb1'	0	0	0	0	6.39	0	0	0	0	0
170742	'Sertad3'	0	0	9.89	7.29	5.52	5.14	6.1	3	2.8	0.97
170743	'Tlr7'	0	0	0	0	0	0.05	0	0	0	0
170748	'Smco4'	2.36	3.14	0.05	0	0	4.85	13.6	4.38	2.87	3.59
170749	'Mtmr4'	11.06	4.02	12.1	7.17	3.92	2.86	3.15	7.12	7.02	6.62
17075	'Epcam'	0	0	2.03	0	22.2	0	0	0	0	0.04
170750	'Xpnpep1'	20.02	17.75	10.1	31.1	15.7	9.66	16.7	37.61	38.06	41.09
170752	'Bco2'	0	1.62	0.05	0	0	0	0	0	0	0
170753	'Zfp704'	0.73	0.52	0.83	1.83	0.87	0.75	1.42	0.24	1.73	1.89
170755	'Sgk3'	0.44	0.14	0.07	0.09	0.02	0.04	0.04	0.3	0	0.02
170756	'Slc8b1'	0.64	0.96	0.15	0.12	0.59	5.83	2.47	0.03	0.02	0.09
170757	'Adgrl4'	0	0	0	0	0	0	0	0	0.6	0
170758	'Rac3'	239.9	107.2	230	154	134	146.1	246	83.96	122.2	64.76
170759	'Atp13a1'	22.63	17.02	23.8	37.1	14.4	14.39	34.2	31.67	36.12	33.12
170760	'Acbd3'	4.47	3.61	2.27	12.7	7.39	6.49	4.1	2.54	1.3	2.8
170761	'Pdzd3'	1.21	5.48	2.97	10.6	7.4	0	0	1.89	4.83	1.27
170762	'Nup155'	2.24	0.23	4.59	0	1.15	0	0.06	2.33	1.27	0.41
170763	'Zfp87'	9.26	5.99	2.68	0.29	10.9	9.35	16.1	12.74	0.38	5.01

170765	'Ripply3'	1.6	1.17	1.18	2.71	1.91	0.47	0.63	0.6	1.82	1.05
170767	'Rfxap'	2.61	1.35	2.78	6.64	2.64	2.78	0	1.74	6.46	0
170768	'Pfkfb3'	9.83	3.98	6.74	5.43	2.35	1.12	7.05	6.27	3.22	8.36
170770	'Bbc3'	1.04	1.19	0.67	6.66	0	0.09	0	1.68	1.63	0.49
170771	'Khdrbs2'	23.52	23.46	43.8	18.3	54.5	38.61	38	23.75	11.61	30.76
170772	'Glcci1'	2.58	2.93	3.17	0.62	3.3	0.94	1.14	0.43	0.01	3.06
170776	'Cd209c'	5.98	8.9	9.77	16.9	7.39	11.81	10.9	9.07	9.21	6.88
170787	'Hdac10'	7.22	11.18	17.5	0.38	18.1	9.13	4.43	8.66	17.78	17.74
170788	'Crb1'	1.54	3.3	0	0	0.7	0.04	0	1.41	0.01	4
170789	'Acot8'	33.36	31.7	36.2	31	13.5	22.38	16.7	42.1	36.43	30.14
17079	'Cd180'	0	0	0	0	0	0.22	0	0	0.17	0
170790	'Mlc1'	59.63	33.48	30.6	65.6	35.1	68.56	27.9	15.25	45.66	31.45
170791	'Rbm39'	79.55	120.3	87.5	147	79	107.7	89.5	78.78	70.9	80.17
170799	'Rtkn2'	0.08	0.01	0.05	0	0.79	0.83	0.01	1.38	0	0
170813	'Ms4a3'	0	0	0.15	0	0	0	0	0	0	0
17082	'Il1rl1'	0	0	0.02	0.01	0.03	0.02	0	0.01	0.01	0.01
170822	'Usp33'	35.42	49.98	50	50.2	25.5	17.61	28.8	37.78	50.94	50.94
170823	'Glmn'	6.03	11.84	8.82	6.37	3.72	8.96	11.8	18.81	9.82	6.35
170826	'Ppargc1b'	4.26	4.02	8.83	3.48	3.11	1.78	8.47	8.21	1.07	2.77
170829	'Tram2'	0.03	0	0.04	0	0	0	0	0	0	0.25
17083	'Tmed1'	42.92	46.26	44	32.6	90.8	35.76	36.9	46.23	59.33	84.77
170833	'Hook2'	37.16	14.69	19.5	46.8	34.1	15.93	9.33	18.84	26.8	22.83
170835	'Inpp5j'	4.9	5.61	20.4	6.1	5.03	5.45	0.78	4.17	6.01	1.98
17084	'Ly86'	3.26	11.13	1.05	11.8	12.4	69.04	5.67	9.56	2.6	2.36
17087	'Ly96'	0.09	0	0	0	0	0	0	0	0.96	9.28
17089	'Lyar'	6.04	11.44	4.9	29.8	8.71	15.62	18.9	17.21	18.32	18.98
170930	'Sumo2'	322.7	311.3	289	431	395	262.7	300	321.5	219	313.79
170935	'Grid2ip'	0	0.76	1.85	0	0.31	0.01	0	0	0	0
170936	'Zfp369'	0.42	0	2.04	0	0.22	0	0	0.18	1.61	2.1
170938	'Zfp617'	16.17	13.18	11.6	8.05	11.6	9.4	15.5	21.09	5.16	7.88
170947	'Myoz3'	0.93	0.01	0.01	0.03	0.01	0.01	0	0	0.02	0.01
17095	'Lyl1'	0	0	0.08	0	4.93	6.57	0	0	0	0.02
170952	'Prima1'	0	0.14	2.19	0	2.7	6.6	0	0	0	0.01
17096	'Lyn'	1.31	0	0.01	0	0	1.29	0	0	2.63	4.78
17101	'Lyst'	1.2	2.2	1.79	2.41	1.23	2.26	1.84	1.47	2.18	1.21
17105	'Lyz2'	0	3.61	6.44	0	0	8.92	0	0	0	6.51
171095	'Il17rc'	1.14	4.72	3.11	0	0.48	5.85	0.9	1.02	0.32	1.43
17110	'Lyz1'	0	0.2	0.5	0	0	0.48	0	0	0	0.34
17113	'M6pr'	85.61	90.7	91.6	82	78.6	108.4	66.7	96.93	136.3	132.64
17116	'Mab21l1'	0	0	0.02	0	28.4	25.21	0.05	7.21	4.87	3
171166	'Mcoln3'	0	0	0	0	1.99	3.73	0	0	0	0
171167	'Fut10'	0	1.5	0.02	0	5.71	3.58	4.75	3.08	2.61	0
171168	'Acer1'	0.11	0.1	0.33	0.14	0.13	0.16	0.07	0.07	0.22	0.04
17117	'Amacr'	33.28	9.24	27	19.8	17.5	23.55	6.36	27.53	25.76	17.3
171170	'Mbnl3'	0.29	0	0.01	0	0.13	0	0	0	0	0
171171	'Ntng2'	0	0	0	0.2	0	0	0	0	0	0
17118	'Marcks'	32.91	25.92	26.4	16	24.7	16.38	23.2	15.09	13.81	24.45
171180	'Syt12'	6.93	3.98	12	0.1	3.08	5.07	22.3	10.66	5.07	8.44
17119	'Mxd1'	5.29	7.34	0.87	0.3	2.95	1.67	6.86	5.05	0.64	2.29
17120	'Mad1l1'	10.71	23.97	14.6	0.03	12.4	9.71	5.74	11.65	9.65	3.57
171207	'Arhgap4'	0	0.14	0.11	0	0	5.12	4.75	1.51	0.87	2.38
171209	'Asic3'	0.14	0.16	1.41	0.23	2.24	0.25	0.11	0.12	0.11	0.53
17121	'Mxd3'	0	0	0	0	5.16	0	0	0	0	0
171210	'Acot2'	2.54	13.3	5.53	6.43	12.5	8	5.02	5.25	6.52	15.37
171211	'Edaradd'	0	0	0.94	0	0	0	0	0	1.41	0
171212	'Galnt10'	3.32	1.46	0.1	2.89	0.89	0.35	3.05	0.84	0.27	0.87
17122	'Mxd4'	2.19	2.76	2.97	0	0.04	0	0	2.44	10.48	4.35
17123	'Madcam1'	0	0	4.67	0	0.22	9.78	0	0	0.29	0.49

17125	'Smad1'	4.02	4.1	5.62	7.62	1.31	3.08	2.2	5.74	2.74	0.03
17126	'Smad2'	2.77	4.46	3.4	3.33	1.81	2.97	3.22	5.7	5.81	6.15
17127	'Smad3'	1.68	2.13	4.09	0	0.01	0.31	0	0.24	2.35	0.01
171270	'Vmn1r203'	0	0	0.11	0	0	0	0	0	0	0
17128	'Smad4'	1.54	2.15	2.5	12.5	5.07	2.12	0.38	1.5	2.53	0.88
171281	'Acot3'	1.28	4.45	5.51	0.2	1.14	3.54	8.93	0.12	0.32	1.34
171282	'Acot4'	1.49	0	0.29	0	2.34	0.1	0	0.78	2.81	0
171283	'Havcr1'	0	0.08	0	0	0	0	0	0	0	0
171285	'Havcr2'	0	0	0	0	0	8.3	0	0	4.65	0
171286	'Slc12a8'	0.1	0.28	0.2	0.4	1.91	0.22	0.05	3.92	0.15	0.18
17129	'Smad5'	0.82	5.15	6.9	0.26	1.38	0.05	0	0.92	6.55	1.04
17130	'Smad6'	0.59	0.17	0	0	0	0	0	0.69	0	0
17131	'Smad7'	1.13	2.27	2.33	3.63	0.02	0.06	2.27	0.03	0.97	0
17132	'Maf'	0	0	0.78	0	0.53	0	0.01	1.12	0	0.16
17133	'Maff'	0	1.19	0.03	14	0.31	0.16	0	0.62	0.04	0.66
17134	'Mafg'	8.55	6.36	9.67	12.2	7.98	8.09	15.3	8.35	4.94	8.52
17135	'Mafk'	11.11	15.28	16.2	7.62	18.3	0.06	10.7	6.5	8.18	16.13
17136	'Mag'	22.88	12.51	5.12	10.2	19.9	58.17	0.76	13.2	12.39	19.28
171382	'Trpm8'	0.02	0	0	0	0	0	0	0.05	0	0
171388	'Bnipl'	0.45	0	0	0	0	0	0	1.47	0	0
171395	'Pkd1l1'	0	0	0.03	0	0	0.41	0	0	0	0
171429	'Slc26a6'	4.6	0.67	0	3.79	2.38	5.07	2.24	0	5.41	0
171463	'Il17rd'	0.57	0.16	0	1.8	0	0	0.01	0.01	0.3	1.27
171469	'Gpr37l1'	69.69	59.47	99.1	64.6	29.5	131.5	58.1	58.03	62.23	12.49
171486	'Cd99l2'	53.95	56.55	67.4	34.6	60.9	64.25	35.7	49.15	45.7	78.64
17149	'Magoh'	33.86	39.28	38.1	72.5	66.9	51.08	46.9	56.89	57.28	40.83
17150	'Mfap2'	15.9	20.87	20.9	44.8	17.7	3.68	11.6	9.08	5.75	18.03
171504	'Apobr'	0	0	0	0	0	3.12	0	1.47	0	0.02
171508	'Creld1'	81.16	112.8	65.8	30.5	62.5	50.93	75.6	90.86	84.55	92.02
17151	'Ccndbp1'	55.14	53.81	82.7	114	41.3	77.97	58.6	71.98	68.67	70.49
17152	'Mak'	0.04	5.57	2.49	2.64	0.93	0.94	0.02	1.99	0.05	0
17153	'Mal'	15.92	22.19	1.94	0	25.4	57.14	0.04	11.25	0.07	1.44
171531	'Mlph'	0.02	0	0	0.01	0.02	0.01	0.02	0.01	0	0.01
171543	'Bmf'	0.01	0	0	0	0.01	0.03	0	0	0	0
17155	'Man1a'	1.79	0	0.78	0	0	1.1	0	0.82	0.11	0
17156	'Man1a2'	7.63	11.39	7.48	12.8	7.4	1	8.55	4.05	6.06	3.5
171567	'Nme7'	2.79	5.86	6.31	0.02	14.1	5.28	10.5	15.35	12.73	9.08
17158	'Man2a1'	0	0	0.05	0.83	0	0.01	0	0	0.34	0
171580	'Mical1'	4.92	0.73	0	4.17	0	0	0	0	0	0.24
17159	'Man2b1'	5.31	12.78	19.7	11.2	11.4	11.87	9.48	24.2	29.17	12.38
17160	'Man2b2'	3.6	5.24	15.7	8.3	15	3.55	16.7	9.92	36.01	30.66
17161	'Maoa'	10.48	17.06	11.7	1.16	9.14	2.69	8.79	18.13	10.43	7.75
17164	'Mapkapk2'	0.68	1.99	0.1	6.43	1.05	0.02	8.02	0.73	0.35	0
17165	'Mapkapk5'	1.59	7.69	5.05	8.81	11.3	6.93	10.1	9.77	13.2	1.6
17168	'Nprl3'	23.91	30.69	21.7	6.1	23.1	14.88	25.7	21.37	23.48	37.07
17169	'Mark3'	1.78	3.05	2.14	4.08	5.04	4.38	2.86	1.83	3.33	5.53
17171	'Mas1'	0.03	0.01	0.01	0.04	0.14	0.01	0	0.02	0.1	0.01
17172	'Ascl1'	1.19	1.36	2.19	8.5	0	0	2.23	0.02	0	0.32
17173	'Ascl2'	0	0.57	0	0	0	0	0	0	0	0
17174	'Masp1'	0.07	1.72	2.71	0	0.17	0.32	4.63	0.12	3.78	0.67
17175	'Masp2'	1.37	0.02	0	0	0.77	0	0	0	0	0
17179	'Matk'	191.3	147.2	133	126	270	307.4	225	187.1	142	210.35
17180	'Matn1'	0	0	0.48	0	0	0	0	0	0	0
17181	'Matn2'	2.81	7	16.2	1.47	0.39	7.71	0.49	3.95	1.15	0.16
17183	'Matn4'	0.02	0	18.3	2	1.5	0	6.49	0.34	0	0.08
17184	'Matr3'	99.4	98.52	111	73.2	55.8	54.65	126	82.18	99.13	112.4
17187	'Max'	68.63	55.89	53	33.8	43.6	48.18	62.4	90.99	85	73.64
17188	'Maz'	7.5	5.64	5.44	9.42	8.81	0.69	9.44	2.96	2.61	4.26

17189	'Mb'	6.88	1.12	26.4	4.1	6.58	8.17	7.08	5.6	5.86	7.29
17190	'Mbd1'	11.2	5.28	8.28	8.12	17.9	10.98	11.1	5.08	13.18	7.57
17191	'Mbd2'	6.94	0.99	0	2.72	0.02	0	4.4	2.15	1.84	1.85
17192	'Mbd3'	16.49	13.24	11.9	50.7	25.9	13.12	27.4	29.71	31.77	15.12
17193	'Mbd4'	4.64	10.23	5.73	2.49	1.01	0.97	0.58	5.8	3.45	2.41
17196	'Mbp'	431.4	211.9	150	104	####	1136	130	249.7	246.9	483.5
17199	'Mc1r'	0.4	2.7	2.1	0	0.4	1.1	0.1	0.5	20.9	0.6
17201	'Mc3r'	52.9	103.4	0	0	0.2	0	487	66.6	0	0.2
17202	'Mc4r'	54.3	36.3	23.3	0	0	69.9	0.2	145.2	36	36.3
17203	'Mc5r'	1.5	12.5	10	0	0.4	0	0	0	5.3	0
17207	'Mcf2l'	6.15	12.38	7.02	10.4	4.94	4.96	15.8	9.7	13.32	10.95
17210	'Mcl1'	5.69	7.11	2.94	22.7	2.22	4.28	1.03	3.41	4.77	4.6
17215	'Mcm3'	0	2.82	0	0	0	0	0	0	4.35	0
17216	'Mcm2'	0	0	3.06	0	0	0.01	0	1.6	4.18	2.81
17217	'Mcm4'	7.09	9.78	10	11.7	1.33	0.76	8.29	4.24	4.85	5.27
17218	'Mcm5'	0	0.03	3.55	0.02	0.45	0.04	0	0.58	0	0.02
17219	'Mcm6'	7.14	12.28	5.62	4.58	6.71	15.13	18.8	12	7.37	12.84
17220	'Mcm7'	5.58	4.43	4.72	8.45	14.7	11.31	13.3	12.11	2.7	1.65
17221	'Cd46'	1.28	0.73	1.58	1.57	0.6	0.65	0.52	0.65	1.07	2.29
17222	'Anapc1'	2.79	2.1	9.3	2.31	5.06	4.25	2.52	1.42	3.44	0.93
17229	'Tpsb2'	0	0	0.68	0	0	0	0	0	0	0
17237	'Mgrn1'	52.77	43.48	73.1	50.1	55.3	42.58	79.1	73.04	88.11	82.21
17242	'Mdk'	28.71	13.81	12.7	27	9.29	29.13	30.7	15.1	19.73	6
17245	'Mdm1'	6.7	3.17	2.94	4.05	13.5	3.17	2.88	2.26	0.55	4.15
17246	'Mdm2'	9.8	12.26	13.9	33.2	20.1	7.52	10.3	13.83	5.43	15.18
17248	'Mdm4'	8.92	5.33	2.76	7.57	4.39	11.23	6.92	3.07	3.76	7.75
17250	'Abcc1'	7.17	1.08	0.16	5.46	1.21	2.44	0	7.55	2.41	2.8
17252	'Rdh11'	14.19	14.65	18.6	27.5	5.75	20.9	0.03	21.45	1.41	10.22
17254	'Slc3a2'	55.27	92.18	84.8	99.9	60	116	45.7	55.23	64.72	75.48
17256	'Mea1'	318.7	231.2	267	266	274	340.7	199	251.4	199.6	209.49
17257	'Mecp2'	1.57	1.64	1.77	0.14	3.01	1.54	1.36	2.48	1.93	1.4
17258	'Mef2a'	3.22	3.77	5.61	10.7	5.89	2.27	2.34	2.04	5.21	0.19
17259	'Mef2b'	0	0	0	0	0	0	0	1.54	0	0
17260	'Mef2c'	12.37	10.75	2.74	14.4	19.7	18.09	7.42	5.34	21.84	6.2
17261	'Mef2d'	13.51	10.01	12.1	12.2	12.5	5.79	3.89	4.32	10.38	6.56
17268	'Meis1'	2.1	0.41	0.03	0.07	16.5	0	0.05	0	2.39	0
17274	'Rab8a'	58.56	62.29	53.5	58.9	43.3	55.39	43.2	65.19	62.3	75.54
17281	'Fyco1'	2.58	0.57	4.75	1.01	2.7	5.63	0.81	1.31	2.03	3.32
17283	'Men1'	35.53	17.85	22.4	28.6	33.2	36.85	33.8	28.4	36.3	30.59
17285	'Meox1'	1.69	3.61	0	13	0	0	0.02	2.07	0	6.82
17286	'Meox2'	5.59	0	0	0	0	0	0	0	0	0
17288	'Mep1b'	0	0	0	0	0	0	0	0	0	1.8
17289	'Mertk'	0	0.03	0	2.01	0.92	1.68	0.01	2.39	0.19	0
17293	'Mesp2'	0	0	0	0	0	1.51	0	0	4.02	0
17294	'Mest'	86.76	113.5	112	83.4	136	97.62	92.6	85.65	101.2	122.2
17295	'Met'	0	0	0	0	0.01	0	0	0	0	0.17
17299	'Mettl1'	41.67	28.73	20.1	17.1	37.3	14.25	12.2	38.47	40.88	30.81
17300	'Foxc1'	0	0	0	0	0	0	0	0	0.05	0
17301	'Foxd2'	0	0	0	0	0	0	0	0.02	0	0
17304	'Mfge8'	48.73	56.54	15.5	9.63	17.1	48.23	21.2	21.23	29.05	56.16
17305	'Mfng'	4.56	1.31	6.95	15.2	0	7.67	0	0	0	24.59
17306	'Sypl2'	0	0	0	0	0	0	0	0	0	0.16
17308	'Mgat1'	2.94	2.91	0.7	25.5	8.97	9.21	15.7	4.91	12.64	3.55
17309	'Mgat3'	3.97	5.56	5.58	10.3	7.69	1.59	5.46	4.49	3.64	2.25
17311	'Kitl'	0.75	0	0.01	0	0.43	0.59	3.31	0.23	0	0.29
17313	'Mgp'	0	0	0	6.04	0.8	0	0	2.48	13.29	0
17314	'Mgmt'	4.72	0.07	0.07	0.84	0	0	12.3	0	3.55	0.06
17318	'Mid1'	2.32	2.97	1.86	1.95	1.63	0.02	1.23	1.06	3.57	2.04

17319	'Mif'	935.8	976.4	1408	1418	744	1242	1004	991.8	895.1	979.75
17329	'Cxcl9'	0	0	0	0.02	0	0	0	0	0	0
17330	'Minpp1'	53.63	44.12	58.4	33.6	28	36.82	54.2	27.09	55.44	23.93
17341	'Bhlha15'	0	0.03	0	5.66	0	0.01	0	0	0	0
17342	'Mitf'	0.78	0.69	0.33	2.4	0.2	2.98	0	0	0	0.02
17344	'Pias2'	6.15	8.02	3.94	11.7	13.7	5.12	6.44	4.34	14.1	5.51
17345	'Mki67'	0	0.02	0.01	0.01	0.01	0	0.3	0	0.03	0.01
17346	'Mknk1'	6.96	6.4	3.04	10.6	11.7	5.37	4.63	7.24	9.4	6.27
17347	'Mknk2'	0.59	0.63	1.94	0.29	3.08	0.01	0.03	1.85	1.68	1.11
17349	'Mlf1'	0.2	3.58	5.26	91.6	3.4	6.25	0.75	0.07	0.04	5.13
17350	'Mlh1'	14.37	11.03	9.16	0	15.4	11.78	5.27	6	12.96	11.3
17354	'Mllt10'	5.78	6.75	6.09	8.2	8.16	2.88	5.09	2.69	8.61	6.84
17355	'Aff1'	0.24	0.55	0.47	0.51	0	0.33	0	0.44	0.43	0.03
17356	'Afdn'	4.72	3.16	3.64	5.36	4.96	1.64	2.99	0.92	4.19	2.29
17357	'Marcksl1'	129.2	71.66	54.9	223	75.7	24.05	48.6	72.68	77.68	43.48
17380	'Mme'	0.11	0.01	0	0	1.47	0	1.29	0.1	0.84	0
17385	'Mmp11'	0.02	1.3	2.16	0.09	3.55	5.28	0.03	4.14	0	1.36
17387	'Mmp14'	2.77	1.11	2.81	1.86	1.7	0.2	0	1.68	2.04	0
17388	'Mmp15'	1.01	1.08	2.34	0	4.38	0.03	2.13	0.16	2.18	0.4
17389	'Mmp16'	4.5	2.12	3.93	1.13	8.4	5.64	8.53	4.51	5.89	3.75
17390	'Mmp2'	2.98	1.64	11.3	4.07	0	0	7.18	0	1.26	0.01
17391	'Mmp24'	22	15.86	13.1	7.63	12.5	25.12	21	18.34	24.43	16.97
17392	'Mmp3'	0	0	0	0	0.03	0	0	0	0	0
17394	'Mmp8'	0	0	0	0	0	0.06	0	0	0	0
17395	'Mmp9'	0	0.01	0	0	0.91	0	0.02	0	0	0.01
17420	'Mnat1'	18.52	28.49	28.7	18.6	10.8	34.46	10	11.41	8.92	20.3
17423	'Ndst2'	1.18	3.55	0.27	0.22	0.69	1.37	1.62	0.56	3.22	2.07
17425	'Foxk1'	0	0	1.18	0	0.63	0.39	0.63	0.02	0.01	0
17427	'Mns1'	0.43	0	0.13	4	0.2	3.83	0.79	0.77	0	0.18
17428	'Mnt'	1.09	0.85	0.57	8.32	2.83	0.03	2.21	1.11	3.98	1.06
17433	'Mobp'	101.8	51.9	6.67	8.32	3102	175.7	48.4	51.3	29.72	68.78
17434	'Mocs2'	40.23	41.86	44.7	48.3	38.9	65.41	25.4	39.43	61.72	37.94
17436	'Me1'	15.72	9.71	15	13.8	23.7	9.87	10.8	13.56	14.38	7.71
17441	'Mog'	21.14	19.67	0	0	18	84.91	0.05	15.19	0	0.6
17444	'Grap2'	0	0	0	0	0	0.49	0	0	0	0
17448	'Mdh2'	707.1	570.2	806	765	845	1083	843	1087	847.4	804.8
17449	'Mdh1'	756.9	673.5	1066	711	820	1153	1000	1062	847.3	795.35
17454	'Mov10'	8.85	10.34	7.94	0	12.1	1.9	3.45	6.76	5.91	4.97
17463	'Psmd7'	170.3	140.2	156	152	135	142.2	106	188.3	119.7	131.55
17470	'Cd200'	285.5	392.9	266	205	241	239.6	213	367.4	343.6	318.46
17475	'Mpdz'	0.41	0.66	1.52	6.43	0.5	4.65	3.16	0.33	0.97	2.5
17476	'Mpeg1'	1.55	0	0	0	1.91	4.9	0	0	0	0
17480	'Mpl'	0	0	0	0	0	0.19	0.02	0	0	0
17523	'Mpo'	0	0.02	0.49	0	0	0	0	1.11	0	0
17524	'Mpp1'	31.68	44.89	42.6	57.7	33.2	36.54	21.1	47.72	32.51	36.79
17527	'Mpv17'	48.28	85.42	52.8	66.3	65.7	107.6	78.5	110.3	110.5	97.87
17532	'Mras'	8.92	8.89	11.6	1.39	8.27	2.12	10.1	5.96	7.34	6.88
17534	'Mrc2'	0	0.2	0	0.25	0	0	0	0	0	0
17535	'Mre11a'	1.77	1.67	0.12	1.99	0.92	1.38	1.06	1.39	0.23	3.02
17536	'Meis2'	0.2	0	0	0	6.19	3.45	0	3.19	0.26	0
17537	'Meis3'	25.65	17.63	39.7	37	20.7	25.28	29.9	29.34	30.48	26.42
17540	'Mrvi1'	0	0	0	0	0	0.35	0	0	0	0
17681	'Msc'	0	0.08	1.04	0	0	0	0	0	3.69	1.4
17684	'Cited2'	4.17	6.33	2.18	4.82	3.23	3.07	21.5	1.49	12.18	3.04
17685	'Msh2'	171.8	224.6	256	0	184	175.8	90.7	327.8	292.4	278.4
17686	'Msh3'	57.6	75.4	82.6	0	19.8	2.6	63.8	23.9	69.3	38.5
17687	'Msh5'	0	0	0.06	0	0	0	0	0	0	0.09
17688	'Msh6'	65.4	21.2	22.9	0	12.7	1.9	35.1	12.8	18.3	66.8

17690	'Msi1'	1.47	0.69	4.31	3.08	2.3	0.2	1.32	0.5	1.34	0.76
17691	'Sik1'	0.88	3.89	3.27	7.02	0.19	2.16	2.26	0	1.02	0.89
17692	'Msl3'	2.1	5.15	3.26	7.49	2.18	5.23	0.02	0.81	0.45	3.54
17698	'Msn'	1.15	0	2.49	0	2.1	2.68	5.67	1.07	2.05	1.92
17701	'Msx1'	0	0	0	0	0.08	0	0	0	0	0
17702	'Msx2'	0	0	0	0	0.07	0	0	0	0	0
17713	'Grpel1'	35.71	31.74	55	41	39.2	36.92	48.9	47.28	37.16	28.31
17714	'Grpel2'	13.4	13.28	4.59	13.2	8.32	18.21	5.91	12.09	11.76	5.2
17748	'Mt1'	136.8	161.1	267	297	96.7	281.4	176	107.8	140	68.24
17749	'Polr2k'	74.26	144.3	94	160	110	85.97	88.9	67.24	114.9	130.36
17750	'Mt2'	30.97	45.47	86.7	78.8	36.9	96.16	62.7	27.03	5.39	8.99
17751	'Mt3'	348.5	470.6	531	415	501	968.4	495	303.5	389.6	349
17754	'Map1a'	5.23	5.5	12.8	11.3	15.1	10.63	8.56	6.39	5.57	9.57
17755	'Map1b'	144.5	159.5	139	149	155	161.3	103	187.5	126.2	156.49
17756	'Map2'	17.88	18.09	12.8	17.8	12.2	10.64	12.9	10.29	14.41	17.53
17758	'Map4'	77.12	67.29	74.5	61.4	98.3	41.52	64.5	84.72	84.52	77.88
17760	'Map6'	60.23	47.14	66.1	30.5	50.3	31.61	47.8	37.94	34.66	33.57
17761	'Map7'	11.33	11.39	2.65	20.5	10.7	7.24	13.3	5.52	2.43	11.06
17762	'Mapt'	109.6	91.51	110	53.5	95	113.8	82	109.6	84.5	77.96
17763	'Mtcp1'	3.13	2.52	0.04	0	0	0	0	3.99	3.49	0
17764	'Mtf1'	2.11	3.06	1.1	7.42	2.19	2.59	2.25	1.33	0.95	2.08
17765	'Mtf2'	23.79	9.78	11.9	33.1	28.6	26.15	21.8	19.83	30.38	23.35
17766	'Nudt1'	10.93	9.52	4.18	0.06	8.75	37.4	0	6.42	7.4	8.83
17768	'Mthfd2'	11.07	24.09	13.2	2.8	15	18.75	20.5	16.87	3.4	28.9
17769	'Mthfr'	0.01	3.4	7.1	0	2.25	2.2	6.43	1.41	4.61	2.72
17771	'Tesmin'	0.04	0	0.02	0	0.37	0.02	0	0.93	0	0
17772	'Mtm1'	1.08	0.61	2.91	4.93	1.79	2.01	5.58	1.1	9.9	3.09
17773	'Mtnr1a'	0	0	0	0	0	0	0	2.2	0	0
17775	'Laptm4a'	169.8	157.9	94.3	145	112	161.4	125	122.6	170.1	169.84
17776	'Mast2'	8.33	7.7	3.98	4.46	0.55	6.42	18.1	6.23	7.13	3.42
17777	'Mttp'	4.61	1.6	11.4	6.91	2.35	0.29	9.01	4.28	5.96	10.01
17826	'Fam89b'	28.46	31.15	23.3	36.8	20.8	17.34	38.3	33.62	19.59	28.47
17827	'Mtx1'	23.67	27.38	39.6	60.1	37.6	68.06	45.1	38.41	35.07	28.43
17828	'Bloc1s5'	44.33	21.23	28.3	10.4	18.1	19.82	6.32	29.82	35.73	9.31
17829	'Muc1'	0	0	0.17	1.39	0	0	0	0.07	0.38	0.09
17831	'Muc2'	0	0	0	0	0	0	0	0	0	0.57
17833	'Muc5ac'	0	0	0.01	0	0	0	0	0	0	0
17837	'Mug2'	0	0	0.02	0	0	0	0	0	0	0
17841	'Mup2'	0	0	0	0	0	0	0	1.14	0	0
17843	'Mup4'	0	0	0	0	0	0	0	0.1	0	0
17844	'Mup5'	0	0	0	0	0	0	0	0.1	0	0
17846	'Commd1'	79.98	85.02	28.5	113	121	118.1	110	83.34	62.01	84.06
17847	'Usp34'	4.37	3.64	6.9	10.5	1.62	3.24	5.66	3.11	7.68	4.68
17850	'Mut'	9.09	10.05	13.5	0.48	4.89	5.4	12.6	4.87	10.29	9.68
17855	'Mvk'	26.51	17.87	15	19.5	16.9	18.98	11.6	11.79	11.13	10.37
17859	'Mxi1'	4.73	4.9	3.3	7.98	7.37	12	12.7	2.88	7.43	3.22
17863	'Myb'	3.31	0.03	0	0	0	0	0	0.95	0	0.09
17864	'Mybl1'	0.01	0.07	0.22	0	1.05	0.01	0.21	0.04	0.31	1.11
17865	'Mybl2'	0.65	0	0	0	0.01	0	0	0.06	0	0.02
17868	'Mybpc3'	0.12	0	0.39	0.6	0.05	0.11	0.04	0.04	0.12	0.13
17869	'Myc'	0.51	13.95	4.51	34.9	0.04	0	19.6	3.69	2.6	2.48
17872	'Ppp1r15a'	35.39	100.8	64.4	239	27.9	72.49	61.9	19.98	21.26	86.89
17873	'Gadd45b'	1.64	25.65	9.89	9.12	4.92	3.73	6.78	4.76	4.61	3.76
17874	'Myd88'	2.16	3.44	3.27	6.87	2.72	11.13	4.64	2.28	0.5	3.88
17876	'Myef2'	44.77	45.27	34.6	34.4	36.7	34.79	27	41.44	53.86	28.38
17880	'Myh11'	0	0.67	0	0	0	0	0	0	0	0
17882	'Myh2'	0	0	1.01	5.32	0	0	0	0	0	0
17883	'Myh3'	0	0	0	0.1	0	0	0	0	0	0.59

17884	'Myh4'	0	0	0.66	1.45	0	0	0	0.2	0	0
17885	'Myh8'	0	0.03	0	0	0	0	0	0.24	0	0
17886	'Myh9'	0.24	0.16	0.58	0.01	1.35	0	0.02	1.62	0.81	0.78
17888	'Myh6'	0.62	0.08	1.93	0.45	0.4	0.55	0.37	0.17	0.5	0.49
17896	'Myl4'	5.56	11.75	0.06	0	10.3	6.66	24	5.65	9.48	0.2
17897	'Myl3'	4.37	0.73	14.9	3.33	3.37	4.92	3.67	1.87	3.56	4.63
17898	'Myl7'	0.08	1.86	0.27	8.16	0.09	0	0	0	2.16	0
17901	'Myl1'	0.06	0.07	0.25	0.66	0.39	0.17	0	0	0.06	0.11
17904	'Myl6'	681.7	559.6	724	678	882	824.3	680	684.3	669	603.05
17906	'Myl2'	8.13	1.14	29.6	6.52	6.25	12.61	8.34	3.59	8.41	12.83
17907	'Mylpf'	0	0	0.15	0	0	0	0.62	0	0	0
17909	'Myo10'	6.32	6.61	7.63	10.1	2.33	1.17	2.8	3.63	2.78	5.57
17910	'Myo15'	0.27	0.17	1.03	0	0	0	0	0	0	0
17912	'Myo1b'	2.77	3.23	1.54	2.06	4.98	0.1	0.11	0.88	0.21	1.99
17913	'Myo1c'	0.01	0.12	4.97	2.82	1.07	1.52	5.7	2.86	2.52	5.93
17916	'Myo1f'	0	0	0	0	0	3.36	0	0	0	0
17918	'Myo5a'	7.8	7.25	14	9.96	5.48	7.12	10.9	6	6.99	5.55
17919	'Myo5b'	0.58	0	2.73	1.8	0.01	0.01	1.84	0.35	0	0
17920	'Myo6'	6.45	8.92	2.35	9.09	6.47	8.67	4.66	1.47	3.44	6.98
17921	'Myo7a'	0.28	3.67	0.96	1.59	1.84	0.76	1.62	0.41	0.94	1.29
17922	'Myo7b'	0	0	0	0.05	0	0	0	0	0	0
17925	'Myo9b'	4.93	2.44	0.76	8.2	7.25	1.09	5.81	2.9	7.18	4.26
17926	'Myoc'	2.8	0	0	0	0	0	0	0	0	0
17927	'Myod1'	0	1.87	0	0	0	0	0	0	0	0
17929	'Myom1'	0.01	0.01	0.22	0.04	0.01	1.6	0.01	0	0.01	0.05
17930	'Myom2'	0.02	0	1.22	6.04	0	0.06	0.02	3.06	0.03	0.03
17931	'Ppp1r12a'	15.14	14.45	5.79	19.9	19	15.37	17.3	20.17	17.98	21.87
17932	'Myt1'	0.13	1.33	3.76	3.61	1.03	1.22	2.41	0.86	0.26	4.26
17933	'Myt1l'	37.78	43.35	30.8	24.4	35.4	30.32	28.4	37.06	19.55	25.17
17936	'Nab1'	2.18	1.67	0.35	0	1.54	2.81	0.11	3.78	3.53	0.16
17937	'Nab2'	0.61	1.21	1.69	3.33	8.54	0.1	0.02	0.03	0.07	0
17938	'Naca'	298.9	298.1	257	307	260	364.8	396	303.1	239.5	250.57
17939	'Naga'	7.96	28.68	23.9	7.73	21.4	37.68	23.4	27.41	66.12	52.08
17948	'Naip2'	0.01	0.05	0.1	0.16	0.06	1.46	0	0.29	0.05	0
17951	'Naip5'	0.42	2.85	1.33	2.16	1.53	0.99	2.08	0.54	1.63	0.64
17954	'Nap1l2'	55.53	88.8	97.6	47.9	56.1	48.41	96	82	52.18	44.93
17955	'Nap1l4'	141.2	152.9	133	200	138	165.6	157	122.7	134.1	126.48
17957	'Napb'	120.9	144	179	189	95.7	52.05	190	172	109.7	90.42
17960	'Nat1'	0	0.91	0	0	0	0	0	0	0	1.97
17961	'Nat2'	0	0	0	0	0.03	0	0	0	0.1	0
17965	'Nbl1'	0.05	2.08	0.06	0	20.7	0	3.09	2.12	0.02	0.15
17966	'Nbr1'	16.08	13.26	25.2	8.32	9.43	5.27	19.5	16.33	19.64	23.74
17967	'Ncam1'	137.5	165	129	100	96.8	55.11	136	154.5	144.4	154.54
17968	'Ncam2'	4.77	4.48	3.86	1.9	2.21	2.49	6.68	6.3	2.15	0.8
17969	'Ncf1'	0	0.05	0.05	0.04	0.25	7.55	0.07	1.52	0.02	0.02
17970	'Ncf2'	0.02	0	2.72	0	0	2.41	0	0.05	0	0.01
17972	'Ncf4'	0	1.32	0	0	0	1.69	0	0	0	0
17973	'Nck1'	21.5	18.69	13.9	6.5	12.3	24.86	10.3	18.69	8.58	3.39
17974	'Nck2'	0.83	0.22	0.53	3.75	6.76	0.16	1.32	3.05	3.91	4.9
17975	'Ncl'	28.96	25.43	29.9	51.1	31.1	30.02	25.1	33.35	33.52	24.94
17977	'Ncoa1'	7.83	6.33	5.96	6.9	4.34	4.44	3.21	3.82	5.19	4.3
17978	'Ncoa2'	2.25	3.55	6.61	0	0.88	1.64	6.26	1.51	1.18	5.72
17979	'Ncoa3'	6.96	7.32	8.83	18.1	6.69	5.79	5.88	3.64	6.62	5.96
17984	'Ndn'	1802	1607	1305	944	1295	1442	1404	609.2	1525	1780.72
17986	'Ndp'	2.83	1.94	0	6.04	3.23	0	0.28	0	3.35	1.67
17988	'Ndrg1'	45.51	38.54	46.9	45.2	37.4	72.8	41.2	27.11	15.37	6.05
17991	'Ndufa2'	448.7	394.7	560	446	383	767.6	448	286.2	540.4	381.56
17992	'Ndufa4'	1027	991	1199	958	962	1161	1125	1000	1019	997.95

17993	'Ndufs4'	73.02	70.08	91.5	63.8	75.8	85.11	97.3	75.21	50.85	75.38
17995	'Ndufv1'	172.4	185.9	256	197	226	308.1	251	249.2	174.7	229.62
17996	'Neb'	0.49	0.79	0	0.01	0.01	0	0	0	0	0
17997	'Nedd1'	1.87	2.48	2.32	1.7	0.52	0	0.15	0	3.24	0.04
17999	'Nedd4'	78.41	60.03	58.5	60.2	33.1	40.55	48.4	52.28	55.82	70.97
18000	'Sept2'	21.61	28.29	26.6	29.4	42	28.24	21.4	36.51	41.08	26.97
18002	'Nedd8'	539	445.9	474	442	504	630.7	514	473.6	544.4	563.86
18003	'Nedd9'	1.15	2.24	3.13	10.4	0.57	2.05	0.78	4.61	0.01	1.17
18004	'Nek1'	8.94	8.29	12.6	15.3	16.7	12.57	12.3	10.42	3.84	13.17
18005	'Nek2'	0	0	0.1	0	0.02	0	3.93	0	0	1.42
18007	'Neo1'	5.61	2.56	3.45	5.9	3.2	0.77	4.68	2.51	0.02	1.68
18008	'Nes'	0	0	0	0	0.03	0	0	0	0	0
18010	'Neu1'	15.78	26.56	35.3	20.2	20.2	19.76	25	24.97	20.27	21.09
18011	'Neurl1a'	6.85	3.52	6.58	3.86	6.47	1.82	16.6	1.76	9.59	3.54
18012	'Neurod1'	0	0.42	0.88	4.27	6.33	0	7.87	0.8	1.47	7.87
18013	'Neurod2'	0	0	0	0	0	0	1.9	0.52	0	0
18014	'Neurog1'	0	0	0	0	0	0	0	0	0	0.03
18015	'Nf1'	0.41	2.53	1.46	4.77	2.98	1.57	1.73	0.43	0.75	1.22
18016	'Nf2'	3.69	2.55	6.33	4.8	3.84	8.11	4	3.69	7.51	3.12
18018	'Nfatc1'	0.18	1.07	0.11	1.2	1.16	0	2.96	0.57	0	0.01
18019	'Nfatc2'	5.31	8.99	17.7	12.8	6.92	18.08	0	15.75	3.01	5.04
18020	'Nfatc2ip'	0.01	0.25	0.01	0	0.42	1.3	0.03	0.51	0.01	0
18021	'Nfatc3'	0.03	2.51	0.34	1.16	0.68	0.01	0.02	0.23	0.01	0.85
18022	'Nfe2'	0	0	0	0	0.03	0	0	0.22	0	0
18023	'Nfe2l1'	22.44	17.52	41.2	13.6	33.5	14.59	40	16	37.69	31.01
18024	'Nfe2l2'	1	1.63	0	0	2.57	7.63	0.06	0	0	1.49
18025	'Nfe2l3'	0.26	0.09	0.02	0	2.5	0	3.6	5.12	0	0
18027	'Nfia'	0.41	1.27	5.47	4.8	1.72	2.31	2.78	0.82	1.56	1.55
18028	'Nfib'	0.53	2.63	3.34	4.76	3.99	0.83	5.4	0.93	0.64	1.38
18029	'Nfic'	4.43	2.6	5.01	2.38	3.64	1.84	4.68	2.63	2.44	4.1
18030	'Nfil3'	6.65	12.93	12.3	19.1	2.45	1.65	3.83	3.07	9.92	6.13
18032	'Nfix'	2.15	2.26	3.02	5.16	2.78	3.12	11.9	1.89	0.02	2.81
18033	'Nfkb1'	1.33	0.83	0.39	3.46	3.95	0	0	1.94	0.02	0.45
18034	'Nfkb2'	0.37	0.93	0	3.11	0	0.02	0.15	0.56	0	2.9
18035	'Nfkbia'	12.95	34.8	24.7	183	17.8	194.7	8.68	38.86	14.81	34.21
18036	'Nfkbib'	32.91	23.37	19.2	35.9	46.1	48.21	32.1	56.49	31.6	25.57
18037	'Nfkbie'	3.02	0.5	0.81	0	0	6.87	0	0	0.02	0
18038	'Nfkbil1'	5	11.24	10.5	8.72	4.54	4.7	4.83	14.06	18.01	2.38
18039	'Nefl'	82.3	67.48	84	55.3	142	123.1	111	158.6	117.1	105.8
18040	'Nefm'	13.56	10.83	14.1	7.2	69.6	38.87	5.24	27.1	9.44	13.66
18041	'Nfs1'	33.35	34.03	33.9	19.5	62.3	27.95	45.7	48.91	24.2	51.02
18044	'Nfya'	0	0.07	0.08	0	0.04	0	0.04	1.35	0.24	0.05
18045	'Nfyb'	19.31	26.09	7.7	15.4	14.4	9.4	7.8	17.19	16.25	13.66
18046	'Nfyc'	19.8	25.93	39.8	3.69	29.7	33.4	6.38	29.57	9.87	14.8
18049	'Ngf'	0	0.42	0	0	0	0	0	0	0	1.07
18053	'Ngfr'	0.01	11.75	0.01	7.11	1.06	0.3	0	0	0	0
18054	'Ngp'	0	0	0.47	0	0	0.12	0	0	0.11	0
18071	'Nhlh1'	0	0	0	0	0	0	0	0	0	0.02
18072	'Nhlh2'	0	4.72	5.35	0.02	11.2	7.08	6.06	1.74	8.8	6.76
18073	'Nid1'	0	0	0	0	1.05	0	0	0.28	0	0
18074	'Nid2'	0.11	0	0	0.35	0.81	0	0	0	0.11	0
18080	'Nin'	0.79	1.22	2.28	2.27	2.05	0.92	2.86	0.34	0.52	1.34
18081	'Ninj1'	34.06	19.88	20.4	55.6	56.2	33.51	66	31.35	40.02	24.64
18082	'Nipsnap1'	109.3	93.79	108	77.8	99.1	65.9	96	99.5	88.53	102.23
18087	'Nktr'	7.73	9.98	10.8	17.9	10.3	7.42	11.8	8.46	7.65	10.42
18088	'Nkx2-2'	1.76	1.2	4.41	0.3	0.51	2.32	2.03	0.08	0.06	0
18091	'Nkx2-5'	0	0	0	0	0	0.03	0	0	0	0
18096	'Nkx6-1'	0	0	0	0	0	0	0	0	0.02	0

18099	'Nlk'	6.87	2.43	1.76	2.43	5.8	1.57	4.24	1.28	0.32	2.04
18100	'Mrpl40'	97.8	71.7	103	73.8	82.4	125.5	113	139.1	115	122.96
18101	'Nmbr'	0	13.77	0	0	1.59	0	0	1.58	0	0.86
18102	'Nme1'	631.5	641	565	842	561	928.3	541	838.9	780.6	663.07
18103	'Nme2'	241.7	242	193	261	155	291	239	278.7	266.5	260.55
18104	'Nqo1'	0.29	2.82	1.41	0.04	0.38	11.32	0	2.64	0.03	3.92
18105	'Nqo2'	7.71	2.73	3.84	6.86	3.02	4.55	6.09	1.95	0.68	1.78
18107	'Nmt1'	40.68	30.41	26.2	42.4	39.6	23.71	48.9	25.02	36.46	42.61
18108	'Nmt2'	6.34	6.3	4.54	0.88	4.62	3.41	4.08	2.25	1.41	7.92
18109	'Mycn'	6.02	7.58	2.1	14.9	0	0	7.17	7.24	6.09	0
18111	'Nnat'	829.8	1300	752	728	958	1587	670	1013	757.9	1700.99
18113	'Nnmt'	5.39	2.1	3.61	13.3	0	0	0	16.04	0	1.49
18114	'Rrp1'	391.5	352	402	252	415	512.9	390	376.7	397	400.55
18115	'Nnt'	7.29	8.69	1.76	15	9.09	3.76	13	7.34	7.52	4.4
18117	'Emc8'	13.98	23.95	19.6	5.25	12.7	9.3	25.9	13.17	29.69	12.72
18119	'Nodal'	0	0	0	5.58	0	11.29	0	0	10.96	0.1
18120	'Mrpl49'	48.38	52.7	44.5	94.4	62.2	84.17	53.7	57.99	31.42	68.92
18121	'Nog'	0	0	0.08	1.67	0.08	0	0	0.65	0	0
18124	'Nr4a3'	0	0.85	0.09	0.46	0	0.06	0.02	0	0.06	0.01
18125	'Nos1'	17.72	8.89	1.45	3.45	5.85	15.42	2.05	3.19	11.6	13.24
18127	'Nos3'	1.05	0.63	2.57	0.62	0.61	2.07	0.78	1.45	1.36	2.75
18128	'Notch1'	0.99	0	0.02	2.67	1.25	0.76	1.27	0.81	1.84	0.3
18129	'Notch2'	1.39	0.46	1.97	1	2.36	0.42	0.33	1.33	1.56	1.8
18130	'Ints6'	2.77	1.04	4.13	1.09	0.52	1.59	5.31	2.56	5.05	3.56
18131	'Notch3'	1.07	0.72	0.08	1.19	0.02	0.06	0.74	0.12	0.07	0.23
18132	'Notch4'	0.21	0.36	0.19	0.27	0.11	0	0.07	0.14	0.76	0.2
18133	'Nov'	0	0	0	0	0	0.5	4.62	0.1	0.51	2.87
18139	'Zfp638'	9.83	9.42	9.4	7.09	5.03	5.31	12.4	12.12	10.92	13.82
18140	'Uhrf1'	0	0.01	0	0	0.01	2.42	0	0.01	0	0
18141	'Nup50'	4.96	9.91	11.3	11.4	6.36	5.58	7.34	11.88	12.51	8.46
18142	'Npas1'	0	0	0.39	0	0	0	0	0	0	0
18143	'Npas2'	0	3.14	0	4.85	0.67	0	7.78	0.02	0	2.16
18145	'Npc1'	3.24	1.89	11.2	1.65	6.83	5.06	3.75	4.94	1.94	3.62
18146	'Npdc1'	328.6	377.2	401	241	238	413.6	383	424.5	357.9	468.8
18148	'Npm1'	142.3	173.5	142	211	144	157.7	118	147.8	152.1	131.76
18150	'Npm3'	4.64	4.5	6.62	8.65	0	16.47	5.73	16.21	7.94	10.54
18155	'Pnoc'	103.6	80.23	77.5	105	84	99.73	15.9	159.9	97.42	19.31
18158	'Nppb'	0.3	0	0.39	0.16	0.25	0.38	0.17	0	0.07	0.06
18159	'Nppc'	5.3	4.67	5.44	5.5	2.78	3.41	10.4	10.24	5.21	8.14
18160	'Npr1'	0.29	0	0	0	0	0	0	0	2.98	10.95
18163	'Ctnnd2'	10.74	8.59	11.4	7.87	15.9	13.24	5.68	3.44	10.53	6.92
18164	'Nptx1'	13.16	2.64	6.03	3.74	6.92	2.51	7.79	4.31	3.91	6.91
18166	'Npy1r'	110.1	43	41.6	0	5.6	71.9	296	255.4	40.7	176.9
18167	'Npy2r'	322.4	72.8	218	0	216	13	639	81.4	21.6	40
18168	'Npy5r'	9.9	0	22.1	0	19.7	40.5	105	86.8	26.8	28.6
18173	'Slc11a1'	0.02	0	0	0	0	7.17	0	0	0.02	0
18174	'Slc11a2'	6.82	12.82	7.75	1.39	6.85	9.42	16.3	17.42	17.66	5.08
18175	'Nrap'	0.02	0	0.04	3.94	0.01	0.01	0.02	0.69	0.02	0.01
18176	'Nras'	7.53	9.63	5.08	9.14	11.1	8.95	3.48	4.91	8.12	5.36
18181	'Nrf1'	5.96	2.45	9.07	6.87	6.96	8.86	0.07	5.75	12.48	13.49
18183	'Nrg3'	1.48	0.02	1.24	2.39	1.9	2.71	0	1.34	1.43	0.78
18185	'Nrl'	0.8	1.44	0	6.58	0	4.42	0	0.03	5.41	0.04
18186	'Nrp1'	31.04	13.1	10.9	1.87	16.7	11.11	30.2	15.59	3.47	20.41
18187	'Nrp2'	18.43	20.09	5.45	2.2	20.4	1.34	9.11	4.64	4.81	5.91
18188	'Nrtn'	0	0	0.07	0	1.14	0	0	0	0	0
18189	'Nrxn1'	57.12	47.66	37.2	42.7	39.2	58.39	72.1	40.31	34.74	27.91
18190	'Nrxn2'	11.38	11.96	15.8	16.6	22.5	15.41	14.3	12.3	9.94	11.98
18191	'Nrxn3'	76.65	67.46	48.4	36.4	48.3	43.78	48.7	70.69	40.35	15.64

18193	'Nsd1'	5.51	3.14	2.77	6.36	6.18	4.47	3.53	3.72	7.73	5.72
18194	'Nsdhl'	18.24	11.91	10.2	32.2	30.4	49.49	17.4	26.8	17.5	22.16
18195	'Nsf'	252.1	240.5	321	169	241	243.4	389	329.4	334.4	242.53
18196	'Nsg1'	497.9	482.9	477	384	471	440.4	486	618.1	447.7	433.66
18197	'Nsg2'	240.7	225.5	281	123	268	206.3	263	256.7	310.9	291.41
18201	'Nsmaf'	2.98	6.88	6.19	1.11	5.55	2.01	13.8	2.01	13.35	6.45
18203	'Ntan1'	55.04	63.71	61.8	34.7	49.7	44.09	36	60.18	46.1	56.84
18205	'Ntf3'	0	0	0	0	0	0	0	1.86	0	0
18207	'Nthl1'	0	4.67	0.78	0.05	6.48	9.9	0	3.21	2.51	2.29
18208	'Ntn1'	0	0	3.61	0.02	0.03	3.18	0	0	0	0.98
18209	'Ntn3'	2.28	1.33	1.49	1.66	0.6	0.64	0.97	1.84	2.51	0.99
18212	'Ntrk2'	42.84	49.94	34.5	35.2	24.2	39.68	46.6	33.25	43.15	37.45
18213	'Ntrk3'	31.17	22.06	20	10.7	20.8	28.28	18.8	17.37	15.99	16.12
18214	'Ddr2'	0	0	1.7	0	0.33	0.98	2.38	0	0	0
18216	'Ntsr1'	0.33	0.15	2.55	0	0	0	0	2.51	7.13	2.11
18217	'Ntsr2'	121	126.3	50.9	43.9	24.2	179.2	77.3	56.21	99.3	37.86
18218	'Dusp8'	31.06	26.13	53.7	31.1	50.5	49.66	46.1	47.65	31.51	38.12
18220	'Nucb1'	58.86	68.88	67.7	41	118	92.42	86.2	93.49	151.1	147.24
18221	'Nudc'	282.7	278.2	234	198	281	310	319	274.2	331.4	278.91
18222	'Numb'	2.65	6.51	1.52	6.65	3.83	1.22	7.61	4.46	2.32	5.18
18223	'Numbl'	6.57	5.22	2.33	3.31	3.76	4.49	10	6.82	1.91	1.14
18226	'Nup62'	2.48	5.63	7.33	10.2	13.4	8.18	7.58	6.5	7.53	10.46
18227	'Nr4a2'	7.45	3.28	6.8	0.79	4.98	5.07	0	6.99	0.01	7.87
18230	'Nxn'	0.94	3.44	2.21	0	1.51	0	1.18	0	0	0
18231	'Nxph1'	20.85	8.39	17.7	37	52.6	30.3	57.2	20.34	3.27	11.54
18242	'Oat'	52.46	62.28	85.2	56.8	77.5	125.2	72.2	85.9	131.9	123.71
18245	'Oaz1'	1018	860.2	731	903	1001	1327	985	936.7	823.7	963.91
18247	'Oaz2'	393.4	369.4	323	422	321	485.8	427	384.4	325.3	366.77
18260	'Ocln'	1.06	0	0	0	0	3.82	0	0	1.62	2.86
18261	'Ocm'	0	0.1	0	0	0	0	0	0	0	0
18263	'Odc1'	11.57	18.89	12.2	49.3	14.9	15.01	16.4	8.16	3.76	4.57
18286	'Odf2'	28.49	23.3	34.3	36.5	22.2	57.38	32	19.94	19.87	42.81
18291	'Nobox'	0	0	0	0	3.63	0	0	0	0	0
18293	'Ogdh'	50.07	55.04	91.6	43.4	63.5	51.42	73.9	56.56	82.91	86.65
18294	'Ogg1'	2.1	2.2	1.73	2.76	9.29	1.41	1.02	2.84	4.72	2.12
18295	'Ogn'	0	0	0.09	0	0	0	0	0	2.28	0.05
18301	'Fxyd5'	4.1	5.02	0	0	3.08	0	0	1.37	3.3	1.38
18302	'Oit3'	0	0	0	0	0	0	0	0	0.05	0
18307	'Olfr10'	0	0	0	0.06	0	0	0	0	0	0
18323	'Olfr25'	2.18	0	0	0	0	0	0	0	0	0
18330	'Olfr31'	0	0	0.05	0	0	0	0	0	0	0
18341	'Olfr263'	0	0	0.05	0	0	0	0	0	0	0
18359	'Olfr59'	0	0.04	0	0	0	0	0	0	0	0
18377	'Omg'	106.5	102.4	95	105	72.5	145	132	167	85.54	69.21
18378	'Omp'	0.02	22.45	9.95	0	0	0	0	0	0.02	0
18383	'Tnfrsf11b'	2.39	0	0	5.17	0	1.12	0	0	0	0
18386	'Oprd1'	0.83	0.86	0	3.55	0.04	0	0.01	0	0	0
18387	'Oprk1'	3.21	21.54	16.9	7.55	2.51	5.01	7.73	2.92	7.34	1.89
18389	'Oprl1'	64.73	79.73	57.8	93.2	41.9	63.58	90.5	92.11	55.15	89.73
18390	'Oprm1'	8.91	5.27	2.99	0	0	6.91	6.6	3.14	0	2.54
18391	'Sigmar1'	40.98	44.13	39	15.8	56.7	29.94	28.8	69.12	54.47	81.62
18392	'Orc1'	0	0.02	0	0	0.02	0	0	0	0.21	0.21
18393	'Orc2'	10.95	15.28	9.19	10.5	13.2	9.05	4.9	9.94	16.12	4.95
18407	'Orm3'	0	1.8	0	0	0	0.02	0	0	0	0
18408	'Slc25a15'	0	0	1.75	0	1.76	0	4.95	3.04	7.77	0
18412	'Sqstm1'	575.6	568.8	707	748	373	348.7	558	480.3	502.5	464.84
18414	'Osmr'	0	0	0	0	0.05	0	0	0	0	0
18415	'Hspa4l'	46.54	37.49	41.4	38.6	28	53.51	60.7	42.15	58.92	54.97

18417	'Cldn11'	28.55	52.55	2.91	0.03	58.4	94.95	0.03	24.05	0.9	1.9
18419	'Otog'	0	0	0.62	0	0	0	0	0	0	0
18420	'Otp'	0	7.37	0	0	0.03	0	23	0	0	0
18423	'Otx1'	0	0	0.02	0	31.1	3.73	0	0	0	0
18424	'Otx2'	0	2.34	3.8	0.05	1.28	0.02	0	0	0.02	8.62
18429	'Oxt'	1.54	0.2	2.15	5.18	0	6.88	3.96	1.04	14.34	0
18430	'Oxtr'	0.26	0	3.85	1.08	0.15	1.33	0	0.72	0	0
18431	'Oca2'	1.9	1.05	0	2.22	0	0	0	1.05	0	0
18432	'Mybbp1a'	14.57	13.81	17.8	0.39	7.71	8.49	20.9	11.33	18.7	18.39
18436	'P2rx1'	0	0	0	0	0	0	0.66	0	0	0
18438	'P2rx4'	4.36	12.87	0.09	0.07	8.2	13.9	11.6	9.91	5.76	2.32
18439	'P2rx7'	0	2.43	6.62	0	0	1.15	4.61	0	0	0
18440	'P2rx6'	0	1.33	2.88	0	0.37	0	0.87	0	0	8.13
18441	'P2ry1'	1.47	3.61	1.1	0	4.51	6.24	2.43	2.49	6.96	1.07
18442	'P2ry2'	0	0	0	0	0	0	0.02	0	2.07	0
18451	'P4ha1'	31.56	41.1	18.2	21.4	8.67	20.09	41.6	15.56	21.26	30.65
18452	'P4ha2'	23.23	17.21	12.7	13.5	10.1	24.66	5.35	22.07	19.02	20
18453	'P4hb'	118.8	119.4	102	105	87.2	85.97	89	95.37	124.7	114.21
18457	'Bloc1s6'	38.85	31.04	28.1	5.95	33.9	32.84	33.4	28.33	34.11	22.08
18458	'Pabpc1'	17	15.64	14.4	22.4	7.55	12.8	8.11	13.65	14.88	16.78
18472	'Pafah1b1'	16.38	16.85	11.4	14.4	15.8	6.69	15.3	13.67	19.02	14.29
18475	'Pafah1b2'	103.4	74.38	72.6	57	59.1	69.82	62.8	80.4	87.61	82.03
18476	'Pafah1b3'	101.3	97.99	145	115	107	110.3	100	143.4	165.4	169.74
18477	'Prdx1'	361.3	315.8	328	281	395	480.9	295	363.6	345.3	446.42
18479	'Pak1'	64.53	117.2	96.1	32.8	69.9	46.27	93.1	80.21	85.79	56.37
18481	'Pak3'	70.18	77.35	41.9	67.7	30.4	53.27	35	59.3	51.8	52.68
18483	'Palm'	27.68	22.58	15.9	16.6	30.4	15.01	14.2	11.24	20.7	10.03
18484	'Pam'	62.38	32.44	60.2	93.1	52.7	17.19	36.1	14.82	62.18	54.38
18488	'Cntn3'	0.05	0	0	1.4	3.1	2.38	2.19	0.39	0	0
18491	'Pappa'	0	0.83	0	0	0	0	0	1.1	0	0.02
18504	'Pax2'	0	0	0.02	0	0.08	0	0	0	0.87	0
18506	'Pax4'	0	0	0	0.1	0	0	0	0	0	0
18508	'Pax6'	0	0	0.72	0	0.03	0	4.5	0	0.04	0
18511	'Pax9'	0	0	0.01	0	0.08	0.01	0	0	0	0
18514	'Pbx1'	4.44	10.15	3.91	5.64	12.3	5.48	4.38	6.68	2.43	4.31
18515	'Pbx2'	1.15	0.7	1.07	4.15	1.27	0.69	2.11	1.35	2.29	0.75
18516	'Pbx3'	6.39	0.01	2.56	0	16	0.62	8.21	8.06	0	1.79
18518	'Igbp1'	6.71	8.74	8.08	8.03	8.63	6.62	7.68	7.11	12.74	10.13
18519	'Kat2b'	1.06	0.62	0.42	2.33	1.65	0.02	0	0.11	0	0.01
18521	'Pcbp2'	21.93	46.01	26.1	43.2	55.7	81.81	29.4	19.04	35.15	63.57
18526	'Pcdh10'	5.37	6.18	12.8	6.31	7.83	7.96	4.03	7.31	11.32	8.02
18530	'Pcdh8'	0.01	5.89	6.08	0	10.3	4.47	8.93	7.09	4.48	3.52
18536	'Pcm1'	12.25	11.79	15	16.1	13.7	11.62	9.98	9.26	4.77	8.69
18537	'Pcmt1'	214	204.1	180	186	210	209.4	200	222.2	212.2	218.02
18538	'Pcna'	43.81	46.03	39.4	37.8	39.1	42.29	21	29.45	87.82	74.16
18541	'Pcnt'	1.89	2.42	0.59	1.57	1.87	1.91	0.01	2.63	2.09	0.91
18542	'Pcolce'	0	0	0	0	1.42	0	0	0.84	0	0
18546	'Pcp4'	2156	653.1	1614	3263	1144	1412	1302	1380	523.2	849.72
18548	'Pcsk1'	16.46	31.46	30.5	16.9	7.1	28.98	0.1	9.97	81.23	93.43
18549	'Pcsk2'	27.67	62.43	61.7	66.3	53.7	52.96	134	17.79	42.59	96.7
18550	'Furin'	0.07	0.16	0.4	2.42	0.16	0.72	0	0.33	0	0.28
18551	'Pcsk4'	6.25	5.79	7.68	7.38	2.62	13.42	3.32	7.67	8.78	7.29
18552	'Pcsk5'	5.93	3.29	0.37	13.2	3.57	7.62	36.5	1.4	21.85	3.19
18553	'Pcsk6'	0	0	0	0	0.02	0	0	2.53	0	0
18554	'Pcsk7'	10	1.53	5.71	8.6	9.07	0.53	5.32	4.01	4.35	6.93
18555	'Cdk16'	19.71	8.74	10.1	12.4	18.3	19.38	33.3	9.22	37.74	2
18557	'Cdk18'	11.54	19	25.6	22.3	30.3	27.89	23.8	25.87	24.5	23.72
18559	'Pctp'	0	0	1.73	0.04	5.71	0	0	0	0	0.09

18563	'Pcx'	3.39	6.7	8.54	12.1	12.9	6.22	27.5	6.1	2.53	14.53
18567	'Pdcd2'	25.97	33.51	42	17.3	23	3.83	44.9	11.98	50.47	1.81
18569	'Pdcd4'	69.03	75.98	65	156	78	25.27	30.5	58.97	92.46	77.32
18570	'Pdcd6'	52.39	52.98	80.9	95.8	89.4	88.18	79.2	72.36	64.02	75.34
18571	'Pdcd6ip'	12.45	10.51	18.8	10.7	6.49	5.37	17.1	5.84	17.39	12.74
18572	'Pdcd11'	2.1	2.65	4.93	6.52	0.51	1.26	1.4	1.82	1.79	7.19
18573	'Pde1a'	10.45	41.79	6.21	0.06	16.5	12.95	17.8	18.76	24.67	38.03
18574	'Pde1b'	22.22	14.49	42.4	6.58	14.5	20.66	56	30.03	14.27	3.6
18575	'Pde1c'	0.54	8.89	0.48	16	4.52	0.35	0.19	7.94	0	0.97
18576	'Pde3b'	0.15	0.16	0.3	2.43	0.83	0.11	1.92	0.68	0.32	1.94
18577	'Pde4a'	14.88	15.03	27.8	8.89	24	12.38	32.4	19.9	23.64	27.46
18578	'Pde4b'	26.79	30.5	34.6	16.5	24.9	36.96	20.9	41.48	12.15	24.57
18582	'Pde6d'	103.6	70.05	41.1	93.1	78.4	80.61	42.7	70.16	85.06	26.39
18583	'Pde7a'	6	1.82	0.11	4.69	0.23	0.09	0	0.08	5.51	0.01
18584	'Pde8a'	0.21	0.57	0	0.06	4.01	0	0	0	0	0
18585	'Pde9a'	8.16	13.29	6.14	15.6	8.58	12.5	19.3	32.23	24.94	11.31
18587	'Pde6b'	0	0	0	0	0	0.05	0	0	0	0.01
18590	'Pdgfa'	31.79	40.91	40.6	36.7	58.2	31.16	18.8	33.53	30.88	13.35
18591	'Pdgfb'	2.01	2.17	3.16	1.33	4.01	0.52	4.29	3.39	1.35	0.27
18595	'Pdgfra'	0	0	16.3	7.8	0.38	0	14.4	2.29	0	0
18596	'Pdgfrb'	0.74	0	1.26	0	0	1.34	0	0.68	1.97	0.16
18597	'Pdha1'	74.81	100.3	127	76.5	83.9	59.55	173	103.2	111.2	91.09
18599	'Padi1'	0	0	0	0	0	0.38	0	0	0	0
18600	'Padi2'	1.31	1.55	0.24	1.21	5.57	3.41	3.56	1.19	1.51	1.58
18602	'Padi4'	0	0.04	0.02	0.03	0	0	0	0	0	0.02
18604	'Pdk2'	27.51	47.4	59.4	22.3	31.7	51.67	71.5	67.77	31.39	36.92
18605	'Enpp1'	0	0	0	0	0.57	0	0	0.01	0	0.22
18606	'Enpp2'	46.27	90.39	21.3	34.6	7.55	61.6	29.9	27.24	62.26	60.06
18607	'Pdpk1'	14.78	14.58	27.7	9.73	6.39	27.34	15.4	14.48	21.2	7.16
18610	'Pdyn'	2.03	53.01	0.04	97.2	0.04	27.79	0.11	83.95	69.76	17.56
18611	'Pea15a'	379.6	426.7	258	417	386	402.8	269	341.7	442.6	408.03
18612	'Etv4'	0.56	2.05	5.24	1.72	0	0	5.24	1.01	1.03	0.35
18613	'Pecam1'	0	0	0	4.53	0	1.95	0	0	0	0
18616	'Peg3'	114.9	64.48	145	111	67.1	51.99	115	96.39	124.3	157.94
18617	'Rhox5'	0	0	0	0	0	0	0	2.26	0	0
18618	'Pemt'	4.87	21.67	10.8	10.6	1.32	3.71	0	7.21	6.88	20.29
18619	'Penk'	117.3	55.18	94.2	562	303	27.84	174	59.71	22.3	75.81
18624	'Pepd'	68.66	71.15	67.7	55.4	64.6	92.22	85.9	72.98	65.47	70.27
18626	'Per1'	1.2	6.53	2.99	24.2	3.08	1.7	3.2	2.22	1.54	9.12
18627	'Per2'	2.97	3.48	3.84	14.9	1.41	1.2	4.96	1.43	6.34	5.54
18628	'Per3'	16.08	7.58	7.9	23.5	16.8	10.82	5.11	11.17	11.61	15.15
18631	'Pex11a'	5.42	5.17	6.53	10.9	6.12	14.6	4.84	12.71	5.19	8.67
18632	'Pex11b'	68.69	50.22	41.7	58.9	45.7	68.48	60.3	45.45	54.6	65.92
18633	'Pex16'	49.41	25.18	37.3	20.4	21.6	44.16	39.3	42.54	42.4	36.78
18634	'Pex7'	39.34	22.24	39.4	27.6	19.3	45.8	28.2	32.09	17.95	27.75
18636	'Cfp'	5.71	7.44	4.69	1.2	4.22	0.03	5.22	2.6	6.65	0
18637	'Pfdn2'	279.6	194.2	229	320	292	358.4	166	230.8	297.8	255.78
18639	'Pfkfb1'	0.06	2.22	0.03	0	0	8.19	0	2.62	0	0
18640	'Pfkfb2'	6.11	4.21	9.58	0.1	6.5	1.74	9.13	3.89	5.4	3.63
18641	'Pfkl'	68.93	99.23	80.4	106	81.6	90.55	92.1	96.63	96.5	91.12
18642	'Pfkm'	91.8	88.96	145	54.8	72.9	126.6	97.7	92.87	74.54	107.55
18643	'Pfn1'	348.3	372.5	206	251	311	428.4	384	450.9	455	463.77
18645	'Pfn2'	183.8	188.5	196	195	258	242.5	285	226.6	148.1	184.47
18647	'Cdk14'	12.7	2.56	20.8	13.1	21.3	9.16	25.4	6.22	10.39	17.37
18648	'Pgam1'	545.4	612.9	676	434	613	802.8	704	655.9	524.4	599.53
18654	'Pgf'	0	6.99	8.51	11.2	2.78	0	3.75	14.77	31.13	15.89
18655	'Pgk1'	264	227.3	282	227	271	265	267	298	196.6	220.28
18667	'Pgr'	3.49	7.13	9.49	5.54	2.46	1	1.56	6.4	3.72	1.15

18669	'Abcb1b'	0.86	0	0.54	0.04	0	0.05	0.03	0.09	0.01	0
18670	'Abcb4'	1.98	0.01	0.6	0.05	0	1.56	0	0	0	0.11
18671	'Abcb1a'	0.01	0	0	0.01	0	0	0	0.02	0.1	0
18673	'Phb'	144.5	165.2	172	164	97.9	185.5	151	141.1	168.8	157.56
18674	'Slc25a3'	556.7	473.1	665	520	580	645.4	655	661.5	603.1	604.06
18675	'Phex'	0	0.02	0.04	0.04	0.03	0	0.05	0.03	0.02	0.02
18676	'Phf2'	1.82	2.38	0.87	2.95	0.07	0.04	2.68	0.25	0.55	1.17
18679	'Phka1'	6.8	7.94	5.59	6.1	1.75	3.29	1.39	1.68	2.41	6.43
18682	'Phkg1'	4.55	5.77	0.05	8.38	0	12.48	2.16	0.02	4.39	2.5
18685	'Phtf1'	4.25	4.7	5.52	3.28	6.99	10.32	3.28	2.83	1.15	12.22
18693	'Pick1'	48.75	51.67	38.1	51.5	75.7	75.45	21.6	44.88	57.19	76.64
18700	'Piga'	0	2.26	2.16	0	5.27	0	0	0	0	0
18701	'Pigf'	24.12	29.37	40.7	53.7	16.5	43.58	26.1	27.82	28.96	9.98
18703	'Pigr'	0.01	0.02	0.05	0.01	0.03	0.02	0.01	0.01	0.04	0.01
18704	'Pik3c2a'	0.92	1.6	1.06	0.52	1.53	1.04	1.5	2.46	1.31	3.09
18706	'Pik3ca'	2.5	0.65	1.18	2.59	2.25	2.5	1.96	1.36	4.47	3.82
18707	'Pik3cd'	1.07	0.26	0.39	0.01	1.35	0.06	6.44	1.84	2.59	1.37
18708	'Pik3r1'	6.52	5.33	5.49	1.93	4.16	8.67	8.21	1.73	6.09	10.65
18709	'Pik3r2'	25.13	11.26	37.3	4.23	16.7	4.01	45	28.83	12.55	21.51
18710	'Pik3r3'	5.06	3.95	6.17	15.7	4.19	3.03	3.39	8.85	13.34	13.5
18711	'Pikfyve'	0.12	0.76	2.09	0.66	0.53	0.15	1.23	0.5	1.69	1.03
18712	'Pim1'	0	0.6	0.17	1.7	0	1.18	0	0	0	0
18715	'Pim2'	56.36	82.72	61.9	53.4	47	84.53	53.5	65.16	79.66	75.58
18717	'Pip5k1c'	7.1	9.13	22.8	13.4	9.7	5.61	11.7	10.4	4.73	4.26
18718	'Pip4k2a'	11.28	2.28	0	1.59	9.35	1.81	0.14	2.72	1.27	3.41
18719	'Pip5k1b'	11.22	11.99	7.54	2.18	0.12	0.11	5.34	1.26	2.53	2.38
18720	'Pip5k1a'	13.18	19.81	24.9	14.7	21.3	19.17	6.78	13.84	13.44	11.47
18722	'Pira1'	0	0	0.2	0.13	0	0	0	0	0	0
18733	'Pirb'	0	0	0.09	0	0	0.47	0.14	0	0.06	0
18736	'Pou1f1'	0	0.11	0	0	0	0	0	0	0	0
18738	'Pitpna'	5.75	6.58	19.6	21.4	18.5	2.45	17.7	10.42	18.4	7.81
18739	'Pitpnm1'	42.64	36.85	47.9	24.8	36.2	16.89	27.7	30.56	39.38	56.18
18744	'Pja1'	255.2	363.6	255	336	166	282.8	369	286.4	341.7	262.77
18746	'Pkm'	961.4	1007	1350	973	975	831.7	876	1200	878.9	803.57
18747	'Prkaca'	68.7	48.37	68.5	73.7	64.5	38.65	43.3	36.62	54.26	43.09
18749	'Prkacb'	225.4	177.7	127	131	100	28.25	140	125.3	118.9	172.73
18750	'Prkca'	3.05	3.37	3.05	8.69	2.24	0.67	2.55	1.05	0.89	1.53
18751	'Prkcb'	9.42	7.77	7.91	9.15	21.7	6.45	26.8	7.72	9.33	6.59
18752	'Prkcg'	23.29	13.86	25.4	12.1	38.2	35.26	19.7	22.09	5.63	12.54
18753	'Prkcd'	1.28	0	5.04	0	8.42	1.49	0	0.02	9.52	5.28
18754	'Prkce'	44.84	31.24	16.6	51.7	25.4	19.92	29.5	35.39	24.73	37.41
18755	'Prkch'	0.16	0	3.52	2.6	0.03	0	0	0.02	1.48	0
18759	'Prkci'	2.74	0.64	1.13	7.54	3.02	0.03	1.89	2.04	3.92	1.41
18760	'Prkd1'	1.4	0.07	0.03	4.36	0.25	0	1.66	2.66	0.08	0.03
18761	'Prkcq'	0.71	1.81	8.21	3.29	3.34	2.62	0.34	5.22	0	0
18762	'Prkcz'	24.9	33.04	35.5	29.3	33.7	33.07	22.1	31.73	30.49	22.32
18763	'Pkd1'	5.45	3.53	10	8.22	7.21	2.49	7.9	3.97	8.23	3.57
18764	'Pkd2'	1.99	0.04	0.04	0.98	1	0.45	1.31	0.45	0.28	1.27
18766	'Pkdrej'	0	0	0.11	0	0	0	0	0	0	0
18767	'Pkia'	58.8	96.94	71.2	41.1	52.2	15.96	103	40.46	32.06	22.65
18768	'Pkib'	12.72	20.64	8.31	0.37	15.4	11.31	0	13.13	11.4	15.12
18769	'Pkig'	60.73	67.47	69	207	75	54.77	38.2	60.18	42.43	77.19
18770	'Pklr'	0.15	0	0.16	0.36	0.22	0	0	0	0.08	0
18771	'Pknox1'	2.3	2.91	0.26	5.65	1.7	0	1.23	5.32	2.83	0.47
18772	'Pkp1'	0	0	1.79	0	0	0	0	0	0	0
18777	'Lypla1'	33.25	22.07	15.7	2.54	3.83	11	21.8	15.03	11.85	17.3
18778	'Pla2g1b'	0	0	0	0	0	0	0	0	0	0.43
18781	'Pla2g2c'	1.51	0.48	0.24	0	0	0	0	0.95	0	3.09

18782	'Pla2g2d'	0	0.04	0.08	0.13	0.07	5.26	0.07	0.02	0.08	0.04
18783	'Pla2g4a'	0.02	0	0.2	0	0.24	0	0	0	0	0
18784	'Pla2g5'	0	0	0	0	4.29	0	0	0	0	0
18786	'Plaa'	8.73	8.11	8.15	6.07	2.66	6.76	9.88	8.51	11.8	7.49
18787	'Serpine1'	2.05	0.66	0.16	1.17	0	0	0.51	0.08	0.38	0.56
18789	'Papola'	25.01	31.97	26.1	27.6	26.4	19.68	33.1	24.49	35.7	23.85
18791	'Plat'	13.51	13.75	4.29	7.03	15.7	5.22	4.7	16.61	3.8	16.2
18793	'Plaur'	0	0	0	0	0	1.75	0	0	1.47	0.69
18795	'Plcb1'	3.39	4.04	7.36	2.98	1.74	5.1	6.74	0.26	1.42	0.15
18796	'Plcb2'	0	0.74	0.04	0	0	0	0	0	0	0
18797	'Plcb3'	1.41	1.72	0	0	0.33	0.16	0	0.02	0	0
18798	'Plcb4'	3.07	12.46	5.61	28	12.6	7.59	6.77	9.13	4.9	6.61
18799	'Plcd1'	0	3.3	1.87	8.22	3.92	13.32	0	4.64	0	3.8
18802	'Plcd4'	8.09	23.08	14	22.7	6.4	18.47	0.75	9.73	8.79	12.84
18803	'Plcg1'	0.89	2.07	1.51	2.95	1.77	0.09	4.14	0.37	2.99	0.84
18805	'Pld1'	0	0.02	0.26	0	0	0.9	2.29	1.16	0.1	1.1
18806	'Pld2'	0	0.58	0.1	1.93	1.03	1.46	0.01	0	4.93	0.02
18807	'Pld3'	625.7	663.7	505	592	502	549	305	601.1	433.8	674.14
18810	'Plec'	1.26	2.09	4.53	3.33	2.78	4.48	4.41	3.77	1.09	1.89
18813	'Pa2g4'	40.57	43.45	39.8	26.9	38.4	36.08	44.1	43.04	16.07	42.21
18817	'Plk1'	0	0	0.37	0	0	0	2.75	0	0.58	0
18821	'Pln'	0.76	0.18	3.13	0.8	0.69	1.1	0.91	1.15	0.78	0.82
18822	'Plod1'	2.73	10.17	7.59	6.65	6.65	2.23	0	3.93	3.57	3.99
18823	'Plp1'	190.3	328.7	93.6	2.98	341	614.8	18.8	129	7.21	32.06
18824	'Plp2'	7.56	6.98	16.3	20.2	20	6.79	0.04	10.47	0	5.84
18826	'Lcp1'	0	1.31	0.61	0	0.01	7.45	0	0.01	0	0
18828	'Plscr2'	0	1.25	0.15	7.02	0	2.64	0	0	0	0
18829	'Ccl21a'	0	0	0	0	0.42	0	0	0	0	0
18830	'Pltp'	4.12	10.38	3.23	0	0.49	3.4	0	4.39	1.54	2.1
18844	'Plxna1'	0.89	5.91	2.04	9.17	6.68	3.42	5.54	3.59	2.5	2.29
18845	'Plxna2'	4.79	0.76	4.56	7.6	2.31	2.1	6.46	2.62	2.55	1.88
18846	'Plxna3'	1.11	2.82	2.7	2.75	0.42	2.18	4.44	2.84	7.05	5.44
18854	'Pml'	0	0.65	0.53	0.03	0.31	0	0	0	0	0
18858	'Pmp22'	5.21	7.05	0	1.08	7.79	26.14	24	4.29	4.3	0.03
18861	'Pms2'	5.93	8.63	3.79	5.65	14	1.07	12.5	8.64	11.83	6.28
18933	'Prrx1'	0	0.01	0	0	0	0.97	0.95	0.91	0.38	0.01
18938	'Ppp1r14b'	0.35	4.41	0.05	5.78	2.34	3.13	2.04	5.56	0.05	1.07
18948	'Pnmt'	0.05	0	0	0	0	0	0	0	0	0
18949	'Pnn'	23.15	21.59	28	22.7	46.7	31.58	29.3	27.44	26.11	27.68
18950	'Pnp'	8.59	4.29	11	20.6	14.9	11.42	7.67	6.53	17.21	12.5
18951	'Sept5'	77.81	68.16	53.4	20.9	47.7	23.08	53	54.8	76.26	62.56
18952	'Sept4'	65.13	66.65	81.3	80.5	163	290.4	58.4	46.64	36.22	28.77
18968	'Pola1'	0.01	0.59	3.7	5.62	0	0	1.04	2.36	0.06	1.45
18969	'Pola2'	5.02	4.82	3.87	15.3	11.5	3.92	15.9	5.13	0.66	11.83
18970	'Polb'	83.02	46.72	50.5	60.9	73.9	61.35	96.6	36.6	43.84	54.39
18971	'Pold1'	2.72	1.15	0	0.02	4.05	2.09	0	0.43	2.95	0.01
18972	'Pold2'	5.45	25.01	25.9	8.69	22.3	16.03	56.5	25.28	50.32	25.76
18973	'Pole'	0	0.02	0	0.53	0	0	0	0.02	0	0
18974	'Pole2'	0	1.14	0.24	0	0	0.08	0.47	0.03	0	0
18975	'Polg'	3.39	4.37	11.5	0	6.51	10.02	5.57	4.24	2.34	5.65
18976	'Pomc'	149	109	53.4	0	120	151.3	227	57	116.8	79.5
18979	'Pon1'	0	0.87	0	0	0	0	0	0	0	0
18983	'Cnot7'	49.96	41.34	38.1	36.7	18.5	23.32	29.2	42.62	48.41	40.92
18984	'Por'	110	83.34	89.4	51.3	89.4	85.76	92.1	94.68	108	103.04
18985	'Pou2af1'	0.02	0.5	0	1.59	0.99	0.95	0	0	0	2
18986	'Pou2f1'	2.54	1.52	0.29	1.79	2.31	0.05	0.77	1.16	1.52	1.88
18987	'Pou2f2'	8.09	3.59	1.99	3.57	1.74	2.77	3.16	3.77	2.14	3.94
18991	'Pou3f1'	0	0	1.87	0.07	3.83	0	0	0	0	0.06

18992	'Pou3f2'	0.84	1.1	0.82	1.62	0.12	0.66	0.84	0.65	1.38	0.76
18993	'Pou3f3'	0.65	1.58	2.59	0.96	0.73	1.3	0.21	0.25	1.65	0.69
18994	'Pou3f4'	0.05	0.31	0.05	0	1.06	0	0	1.67	2.95	0
18996	'Pou4f1'	0	0	0	0	0.08	0	0	0	0	0
19009	'Pou6f1'	1.71	1.53	1.27	3.15	7.84	1.16	3.28	0.58	0.01	0.03
19011	'Endou'	0	2.27	0	0	0	0	0	0	0.02	0
19012	'Plpp1'	48.92	78.73	40.2	46.9	47.3	83.02	99.2	72.44	100.2	101.09
19013	'Ppara'	0	0	0	0.5	0	0	0	0	0	0
19014	'Med1'	8.78	9.8	11.9	0.78	8.95	5.66	4.74	17.98	8.79	10.89
19015	'Ppard'	0	0	0.24	3.46	0.59	0	0	0.65	0.01	0.55
19016	'Pparg'	0	0	0.03	0	0	0	0	0	0	0
19017	'Ppargc1a'	2.18	4.79	3.67	3.02	1.1	0.02	3.02	0.12	0.54	0.84
19018	'Scand1'	15.85	10.7	12.8	30.9	11.1	7.58	7.88	9.5	14.3	1.43
19023	'Ppef2'	0	1.42	0.75	0	1.98	0	1.13	0.63	4.31	0.67
19024	'Ppfibp2'	1.93	1.78	0.86	5.72	0.15	1.73	2.39	0.8	0.88	4.01
19025	'Ctsa'	42.2	39.82	44.3	53.6	38.9	93.16	48.1	61.66	57.81	60.69
19027	'Sypl'	4.73	11.08	5.7	7.5	8.43	7.1	0.79	5.82	7.1	13.99
19035	'Ppib'	82.36	94.72	144	61.7	127	134.5	154	86.14	102.2	48.51
19039	'Lgals3bp'	44.4	76.11	26.6	88.6	48.8	107.4	8.97	48.32	116.5	105.26
19041	'Ppl'	0	0	0	0	0.11	0	0	0	0	0
19042	'Ppm1a'	11.55	10.85	8.44	15.1	22.3	7.32	12	6.33	6.05	8.43
19043	'Ppm1b'	11.49	8.33	11.7	2.43	10.9	10.18	2.84	10.26	28.31	1.92
19044	'Ppox'	21.19	52.02	18.9	11.5	41	36.75	36.8	36.85	31.73	37.71
19045	'Ppp1ca'	240.6	224.1	246	303	190	313.2	257	275.1	190	256.26
19046	'Ppp1cb'	54.73	34.85	29.6	45.7	49.5	31.23	29.9	38.12	65.73	43.71
19047	'Ppp1cc'	12.96	8.09	10.6	10.2	17.3	13.22	24.3	19.29	24.06	17.52
19049	'Ppp1r1b'	12.89	8.32	3.74	0	4.43	26.85	0	50.65	16.75	0
19051	'Ppp1r17'	80.09	79.1	18.8	106	21.6	0.05	0.06	11.54	0.07	9.8
19052	'Ppp2ca'	30.65	29.4	16.5	73	48.1	46.31	31.6	13.56	10.43	33.64
19053	'Ppp2cb'	29.17	30.71	20.2	49.3	28.1	17.77	27.3	21.33	24.38	25.51
19054	'Ppp2r3d'	5.92	3.89	2.67	11.5	5.32	2.9	3.44	1.16	2.01	2.54
19055	'Ppp3ca'	40.6	58.49	32	41.9	58.9	47.73	41.6	54.85	19.88	38.18
19056	'Ppp3cb'	7.8	7.37	11.7	15.9	21.6	2.97	15.3	11.7	6.97	7.88
19057	'Ppp3cc'	0.07	4.57	0	0	5.29	6.04	0	1.54	7.66	0.52
19058	'Ppp3r1'	267	277.2	268	218	146	150.8	327	298.6	235.3	230.48
19059	'Ppp3r2'	2.04	0	0	0	0	0	0	0	0	0
19060	'Ppp5c'	92.08	87.55	103	83.2	114	129.4	117	119.5	137.6	136.8
19062	'Inpp5k'	21.25	37.51	23.9	26.4	28.7	54.15	15.7	28.27	30.17	29.3
19063	'Ppt1'	101.1	98.45	120	60.2	70.4	69.28	96.9	123.9	75.99	103.7
19065	'Npy4r'	0	2.28	0	0	0	0.02	0	0	0	0
19069	'Nup88'	99.08	55.67	55.4	12.7	70.4	63	62.1	86.56	101.5	78.66
19070	'Mob4'	49.55	48.68	40	19.2	43.9	58.29	57.1	40.91	34.21	54.23
19072	'Prep'	3.92	3.53	14.9	0	8.84	3.01	12.6	10.94	11.81	8.43
19073	'Srgn'	0.08	0.05	0.16	0.16	1.53	16.26	0.07	0	0.1	1.42
19075	'Prim1'	13.74	8.62	0.13	34.6	7.34	0	9.88	3.31	13.8	9.43
19076	'Prim2'	10.57	7.24	3.63	5.64	6.22	4.21	6.02	3.11	2.72	1.71
19079	'Prkab1'	3.16	19.47	7.93	8.08	12.5	11.13	18.4	7.6	0.99	6.51
19082	'Prkag1'	76.43	53.35	39.9	31.4	52.8	22.34	63.4	64.37	44.9	38.77
19084	'Prkar1a'	615.1	786.4	594	579	506	513.9	647	682.6	720.2	843.32
19085	'Prkar1b'	355.4	367.1	423	95.6	240	314	403	338.9	291.8	335.01
19087	'Prkar2a'	10.32	5.7	4.56	13.3	3.43	5.74	7.37	5.85	12.73	6.42
19088	'Prkar2b'	8	7.01	3.2	5.81	1.54	5.4	7.37	7.06	3.8	2.23
19089	'Prkcsh'	78.56	102.2	49.4	24.4	51.2	45.27	99.5	57.5	64.45	68.93
19090	'Prkdc'	1.45	1.6	4.09	0.75	0.76	1.92	5.06	1.16	3.76	0.68
19091	'Prkg1'	5.8	6.74	0.09	3.36	4.57	2.71	8.18	2.67	1.73	0.35
19092	'Prkg2'	2.04	0.86	0.42	0.07	0.07	0	0	1.35	4.89	0
19094	'Mapk11'	18.67	11.41	14.7	12.3	10.7	14.29	14.1	20.02	38.84	15.65
19099	'Mapk8ip1'	9.14	10.78	12.3	6.57	11	9.64	15.7	6.78	11.33	9

19106	'Eif2ak2'	2.05	0.96	4.13	9	2.72	1.6	4.06	0.26	0.58	8.74
19108	'Prkx'	0.24	1.92	6.38	7.18	1.41	0	1.83	1.26	4.24	5.32
19116	'Prlr'	64.1	77.86	39.9	13.1	3.92	24.6	26.5	71.97	73.01	42.27
19118	'Prm1'	0	1.11	0.69	0	0	0	0	0.47	0	0
19122	'Prnp'	860.1	1149	690	840	716	846.3	687	906.4	765.8	914.56
19125	'Prodh'	6.31	1.45	0.63	0.02	2.04	10.56	2.67	1.31	8.63	1.36
19126	'Prom1'	2.08	0	0	0	0	0	0	0	0	0
19128	'Pros1'	2.44	6.03	3.13	9.27	1.23	8.24	8.22	2.08	0.62	0
19130	'Prox1'	2.1	1.32	2.3	3.45	0	0.07	0.01	0	0	0.01
19134	'Prpf4b'	9.67	14.66	14	12.7	10.3	9.94	6.15	14.31	14.09	13.82
19139	'Prps1'	179.7	195.1	124	198	183	319.4	177	178.8	170.6	138.5
19141	'Lgmn'	141.1	178.7	176	99.9	206	348	119	189.9	198.7	177.48
19142	'Prss12'	0	0	3.52	0	0	0	0	0.02	0.11	2.56
19143	'St14'	0	0	0	0.01	0	0	0	0	0	0.01
19144	'Klk6'	0	1.45	0	0	17.5	0	0	0	0	0
19152	'Prtn3'	6.47	8.41	3.44	8.19	9.14	5.69	0	3.05	6.86	0
19155	'Npepps'	27.2	15.76	18.7	28.6	31.1	20.84	31	13.04	24.68	23.16
19156	'Psap'	893.5	904	917	379	807	965.8	1115	817.7	827.8	741.47
19157	'Cyth1'	6.69	19.64	17.2	28.4	16.1	8.99	10.8	9.86	23.48	2.05
191578	'Helq'	3.03	2.05	5.82	10.2	2.69	1.91	0.17	1.77	0	2.49
19158	'Cyth2'	31.25	22.3	30.8	30.7	24.3	16.9	28.4	12.36	11.48	4.2
19159	'Cyth3'	13.02	9.16	9.9	8.08	3.96	4.25	12.8	6.9	4.99	7.13
19164	'Psen1'	27.51	23.21	37.6	21.3	17.8	22.05	27	24.88	33.47	50.73
19165	'Psen2'	30.39	14.17	28.7	10.9	25.1	23.68	22.6	20.03	34.96	17.13
19166	'Psma2'	396.3	328	342	306	360	442.1	240	419.1	360	376.96
19167	'Psma3'	281	246.7	251	330	304	288	257	326.7	251.7	283.89
19170	'Psmb1'	415.4	436.5	390	466	347	397.7	372	457.1	401.8	412.66
19171	'Psmb10'	56.49	53.78	96	98.8	68.6	113.5	80.3	80.16	64.57	89.64
19172	'Psmb4'	294.8	302.4	424	576	289	372.1	279	303.8	328.7	295.68
19173	'Psmb5'	383.5	269.7	345	388	393	337	350	408.5	311.9	366.24
19175	'Psmb6'	441	393.3	338	421	472	652.2	364	488.1	430.7	478.28
19177	'Psmb7'	234.3	297.9	286	414	348	345.3	340	387.2	336.1	306.02
19179	'Psmc1'	245.6	244.3	239	311	276	296.1	258	332.3	366.2	306.06
19181	'Psmc2'	152.9	147.9	174	206	119	181	138	139.4	142.4	165.82
19182	'Psmc3'	156.4	163.7	190	213	181	190.5	153	187.9	166.4	148.19
19183	'Psmc3ip'	3.16	0	6.52	0	4.36	0.05	0	4.89	2.72	3.38
19184	'Psmc5'	354.2	350	399	381	374	560.3	349	461.3	383.9	401.47
19185	'Psmd4'	273.7	237.2	265	306	250	377.2	289	253	216	276.78
19186	'Psme1'	159.6	156.9	163	216	120	147.5	182	124.3	142.3	207.48
19188	'Psme2'	118.6	114.9	96.9	304	134	188.5	119	123.4	106	147.98
19192	'Psme3'	7.61	3.72	2.74	32.6	14.7	16.5	6.06	18.82	23.71	8.12
19193	'Pipox'	4.75	4.68	0	0	0	1.76	0	0	9.44	0.87
19197	'Pspn'	4.01	1.81	5.23	11.7	0	0	3.43	0	0.82	2.14
19200	'Pstpip1'	1.06	5.31	0	0	4.97	8.08	0	2.71	0	0
19201	'Pstpip2'	2.41	0.83	1.63	14.8	2.45	0.14	0.02	5.41	0	0.02
19204	'Ptafr'	0	0.09	0.09	0.08	0.07	1.94	0.07	0.03	0.03	0.06
19205	'Ptbp1'	9.53	18.68	14.8	41.2	19.9	7.5	9.64	10.26	7.93	6.79
19206	'Ptch1'	6.41	0.48	0.46	18.4	0.1	4.06	1.42	2.48	2.8	0.1
19207	'Ptch2'	2.11	0.35	2.43	0	0	0	0	0.62	1.62	0
19208	'Ptcra'	0.57	4.26	0.6	1.25	1.06	2.8	1.37	1.19	0.7	3.82
19210	'Ptdss1'	38.82	30.48	37.6	35.1	34.2	37.04	82.6	44.71	49.96	46.18
19211	'Pten'	2.08	2.28	1.02	4.92	2.73	2.59	1.84	1.47	0.5	1.52
192119	'Dicer1'	0.73	1.66	0.31	6.05	2.73	0.46	0.63	1.21	0.96	0.53
19212	'Pter'	0.14	2.3	1.41	2.38	0.27	1.25	0.16	0.12	3.21	0
192120	'Bspry'	0.66	0	0	0	0	0	0	0	0	0
192136	'Sugct'	0	3.13	7.28	0	4.62	0	7.19	0	3.22	0
19214	'Ptgdr'	0	0	0.31	0	0	0	0	0	0	0
192140	'Tmc2'	0.02	0.45	0	0.02	0.01	0	0.04	0.01	0.01	0.01

19215	'Ptgds'	175.3	194	11.1	12.8	62.7	738.6	22.7	27.55	95.88	35.16
192156	'Mvd'	5.72	19.9	24.5	32.5	12.4	44.14	18.7	19.45	9.88	7.53
192157	'Socs7'	2.75	2.46	1.7	5.26	3.74	0.49	1.83	1.66	1.56	0.44
192159	'Prpf8'	7.24	6.55	11.2	6.32	3.46	1.81	11	5.71	2.83	9.63
19216	'Ptger1'	0	0	0	0	0	1.8	0	0	0	0
192160	'Casc3'	7.22	4.34	5.06	16.9	3.16	6.13	4.71	5.74	14.01	3.6
192161	'Pcdha9'	0.66	5.83	1.99	0.02	2.16	0	1.22	0.93	3.6	2.53
192163	'Pcdha3'	1.34	0.67	2.18	0	0.02	0	3.68	0.66	0.74	3.86
192164	'Pcdha12'	10.15	2.05	2.16	0	0	7.29	4.03	1.49	4.17	2.7
192166	'Sardh'	1.29	0	2.35	0	1.16	0	0	0	3.52	0
192167	'Nlgn1'	8.23	11.26	12.3	7.71	18.1	14.51	20.4	9.9	12.74	9.79
192169	'Ufsp2'	51.61	53.79	63	37.8	41.9	63.92	48.3	78.66	41.78	58.56
19217	'Ptger2'	0.15	0	0.4	0	0	2.09	3.03	0.21	1.48	0
192170	'Eif4a3'	61.91	78.9	73.9	71.6	64.8	47.9	51.5	96.37	83.37	62.37
192173	'Mcrip1'	132.3	110.6	186	272	134	171.1	190	144.5	257.8	107.71
192174	'Rwdd4a'	7.89	10.05	6.93	19.5	3.39	9.06	7.55	9.99	7.58	8.56
192176	'Flna'	1.87	3.41	6.61	4.74	2.34	1.37	8.9	4.08	3.1	6.4
19218	'Ptger3'	1.14	1.63	1.07	0.03	0.33	19.02	2.94	0	2.95	11.84
192185	'Nadk'	17.39	14.27	11	20.4	6.32	16.51	16.8	34.84	11.81	15.3
192187	'Stab1'	0	0	0.01	0	0	0.84	0	0	0	0
192188	'Stab2'	0	0.59	0	0	0	0	0.01	0.12	0	0
19219	'Ptger4'	0	1.37	0	0	0.13	0	1	1.92	0.15	4.83
192190	'Pkhd1l1'	0.21	0	0	0	0.01	0	0	0	0	0.02
192191	'Med9'	16.71	23.6	15.1	11.1	16.7	9.5	22.6	24.25	16.58	21.23
192192	'Shkbp1'	3.98	9.26	7.21	2.98	9.91	16.93	5.16	7.18	5.22	10.39
192193	'Edem1'	0.24	1.04	0.5	0.12	0.69	0.2	1.41	0.97	0.09	0.27
192194	'Btnl10'	0.01	0	0	0.98	0.01	0	0	0.01	0	0
192195	'Ash1l'	5.09	3.54	3.93	15.7	6.99	6	6.99	5.94	4.93	7.3
192196	'Luc7l2'	12.18	12.23	17.7	11.6	12.6	9.85	9.88	7.33	13.56	11.87
192197	'Bcas3'	14.97	12.8	17.3	33.2	12.8	18.11	21	6.1	19.47	17.36
192198	'Lrrc4'	3.45	0.58	3.92	1.35	4.03	1.89	0	7.43	2.88	0.78
192199	'Rspo1'	3.52	2.42	1.5	0.87	4.94	13.21	5.57	9.3	3.01	0.31
19220	'Ptgfr'	19.8	0	0		40.4	70.4	31.8	0	21	55.4
19221	'Ptgfrn'	0	0	0.01	0	0.01	0.48	0	0	0	0
192212	'Prom2'	0	0.03	0	0	0	0	0	0	0	0
192216	'Tmem47'	21.68	40.81	17	27	24.9	9.24	26.5	19.07	19.31	41.02
19222	'Ptgir'	0	0	0.01	0	0	0.01	1.16	0	0	0
19223	'Ptgis'	0	3.73	0.28	0	0	0.08	0	2.49	5.82	1.79
192231	'Hexim1'	5.83	6.91	3.31	0.81	2.89	9.05	7.93	13.64	5.47	9.02
192232	'Hps4'	0.8	4.54	8.3	7.36	8.99	9.85	7.79	8.39	3.85	5.74
192236	'Hps1'	8.61	7.83	5.75	12.4	7.96	4.67	0.01	10.04	6.07	8.5
19224	'Ptgs1'	2.4	0	0	5.59	0.02	33.59	5.63	5.65	0.03	0
19225	'Ptgs2'	0	0	0.13	0	0	0	0	0	0	0
19227	'Pthlh'	6.31	3.01	1.76	0	0	0	0	1.84	0	5.08
19228	'Pth1r'	4.28	3.31	7.15	0	7.39	6.19	0.27	4.98	3.54	8.38
192285	'Phf21a'	9.15	4.33	3.88	4.58	4.65	6.66	6.41	6.46	3.91	7.14
192287	'Slc25a36'	15.87	17.09	25.3	15.4	23	15.55	23.1	24.92	25.61	24.9
192289	'Tmlhe'	0	0.26	0.02	0	0.89	0.38	0	0.89	0	0
19229	'Ptk2b'	3.44	5.72	30.5	55.7	5.29	5.5	2.61	5.52	15.82	32.22
192292	'Nrbp1'	82.13	96.86	102	95.1	60.2	88.08	80.6	60.53	110.2	99.96
19230	'Twf1'	48.2	52.25	36.3	47.9	52.1	36.57	22.8	45.88	50.87	49
19231	'Ptma'	144.1	207.7	153	214	147	122.9	211	120.5	130.5	202.28
19240	'Tmsb10'	1434	1278	1202	1591	1207	1274	1200	1119	1438	1195.29
19241	'Tmsb4x'	1477	1679	1307	1966	1937	2059	1765	1458	1226	1461.94
19242	'Ptn'	52.2	74.98	120	61.9	82.3	54.95	141	54.3	124.9	68.59
19243	'Ptp4a1'	62.33	94.83	57.3	158	26.1	22.44	41.9	36.49	30.77	12.57
19244	'Ptp4a2'	56.04	55.61	49.9	50.6	59.4	52.16	39	39.71	30.56	37.2
19245	'Ptp4a3'	21.57	13.9	20.8	13.5	21.3	16.9	2.92	13.16	11.97	23.23

19246	'Ptpn1'	7.31	5.35	11.5	20.7	6.78	6.22	9.83	7.52	3.71	8.71
19247	'Ptpn11'	11.63	10.07	19.9	10.9	21.6	10.75	18.5	6.55	22.95	9.27
19248	'Ptpn12'	4.22	4.4	2.45	1.92	10.1	0.19	0.15	1.34	3.92	1.18
19249	'Ptpn13'	1.26	0.22	0.01	0.05	1.77	0.11	0	0.01	0	0
19250	'Ptpn14'	0	0	0.01	3.27	0.02	0	0	0	0	0
19252	'Dusp1'	78.91	222.2	290	534	121	371.9	81.2	87.2	68.22	266.95
19253	'Ptpn18'	0.06	0	0	4	0	1.71	2.52	0	0.03	0.03
19255	'Ptpn2'	20.63	11.31	15.4	5.28	13.6	15.81	21.1	17.33	22.44	35.51
19256	'Ptpn20'	0	0	0	0	0	0.79	0	0	0	3.52
19258	'Ptpn4'	5.75	0.91	3.24	1.62	1.76	1.19	0.09	1.96	4.61	1.35
19259	'Ptpn5'	89.15	119.2	80	141	51.6	53.78	103	98.05	96.12	69.9
19260	'Ptpn22'	0	0	0	0.02	0	0	5.59	0	0	0
19261	'Sirpa'	33.83	19.41	15.1	11.4	24.7	23.99	23.5	24.42	22.12	21.27
19262	'Ptpra'	18.17	24.3	20.5	40.5	9	23.29	33	18.52	19.98	16.6
19263	'Ptprb'	0.03	0	0.78	0.33	0	0	0	0	0.41	0.47
192650	'Cabp7'	0	0	0	0	0.98	0	0	0	0	0
192651	'Zfp286'	8.32	7.36	7.28	7.76	4.44	12.3	3.97	5.97	7.91	3.41
192652	'Wdr81'	1.03	0.88	2.98	2.31	0.52	3.43	0	2.15	0.87	0.67
192653	'Ttc36'	0.68	0.93	0	1.83	0	0	0	0.06	0	0.18
192654	'Pla2g15'	13.84	7.57	19.5	14.3	0.34	17.49	0.02	9.86	1.12	23.28
192656	'Ripk2'	0.1	0.32	0	0	0	4.65	0	0.04	0.09	0
192657	'Ell2'	0	2.74	0.61	1.34	1.98	1.24	2.8	0	0.35	0
192658	'Rfpl4'	0	0.37	0.1	0	0	0	0	0	0	0.06
19266	'Ptprd'	13.94	4.32	2.82	0.88	10.3	5.11	8.74	6.47	6.49	6.06
192662	'Arhgdia'	155.9	152.4	195	112	210	149.5	249	140.7	150	166.06
192663	'Abcg4'	8.73	15.71	30.9	5.06	16.6	2.9	9.86	24.32	10.31	6.86
19267	'Ptpre'	3.43	3.52	2.73	4.79	5.41	8.37	12	4.37	9.65	1.07
192678	'Rassf3'	0.01	0	0	0.02	0.36	0.36	0	0	1.61	0.01
19268	'Ptprf'	10.9	10.94	8.24	10.9	9.45	5.59	8.1	10.07	8.26	4.55
19270	'Ptprg'	0.57	3.12	3.8	6.54	2.5	0.02	2.48	1.89	0	0.36
19271	'Ptprj'	0.85	1.49	2.3	1.32	2.32	0.56	3.66	1.53	1.4	0.98
19272	'Ptprk'	8.66	5.05	2.12	0.78	11	3.6	7.53	8.46	0.29	12.23
19273	'Ptpru'	3.69	2.77	8.96	1.96	1.24	0.89	8.62	0.78	7.28	2.69
192734	'Lrrc75b'	1.87	2.16	2.41	0.07	1.47	5.26	4.79	0.62	6.65	3.98
19274	'Ptprm'	7.66	3.36	16.6	8.33	2.27	5.35	6.35	6.04	5.3	12.76
19275	'Ptprn'	48.79	69.76	54.3	157	66.6	58.15	36.9	53.72	86.54	93.07
19276	'Ptprn2'	15.85	11.98	14.3	9.27	18.8	11.39	4.68	17.22	30.32	18.31
19277	'Ptpro'	26.78	34.28	16.8	13.5	11.5	4.49	12.5	20.64	3.75	20.19
192775	'Kcnh6'	19.67	21.06	42.9	42.3	21.1	56.48	31.9	14.21	47.11	36.01
192786	'Rapgef6'	14.01	10.43	3.6	25.9	7.56	8.45	7.67	11.48	11.52	16.59
19279	'Ptprr'	14.99	11.21	49.4	70.9	16.9	5.49	0.14	16.29	5.35	3.1
19280	'Ptprs'	34.49	30.29	61.5	18.5	53.8	20.16	45.9	42.88	49.02	54.03
19281	'Ptprt'	0.5	0.93	2.78	1.41	1.42	4.47	2.13	0.99	4.18	2.36
19283	'Ptprz1'	18.22	11.57	27.1	19.8	3.89	7.11	24.4	8.28	25.27	8.82
19285	'Cavin1'	1.34	3.1	0	4.39	0.74	0	0	0	0.01	0
192852	'Lrrc3c'	0	0	0	0	0	0.05	0	0	0	0
19286	'Pts'	77.89	99.31	61.9	76.7	74.7	65.82	61.4	72.02	101.4	61.09
19288	'Ptx3'	0	0	0	0	0	0	0	1.17	0	0
19289	'Igdcc3'	1.97	0	0	0	0	0	0	0	0	0
192897	'Itgb4'	1.94	0.95	0.01	0.04	0.72	0.01	0.39	0	0.03	0.08
19290	'Pura'	15.24	10.18	12.1	12.9	26.2	12.74	14.1	17.94	11.77	12.45
19291	'Purb'	6.34	6.74	8.55	9.71	5.64	6.42	14.1	4.56	4.29	5.73
19293	'Pvalb'	5.28	0	71.7	0.07	6.26	8.7	0	0.05	0.06	5.09
19294	'Nectin2'	13.87	11.79	18	7.9	14	28.88	23.3	8.33	13.11	14.27
192950	'Nacad'	8.98	12.59	16.7	2.53	10.2	19.45	12.3	10.57	14.17	15.78
192970	'Dhrs11'	2.24	1.96	13.3	0	2.4	0.07	10.5	5.27	5.48	1.16
192976	'Lrrc75a'	0	1.72	0	0	4.11	9.03	7.26	0.02	1.47	2.41
19298	'Pex19'	28.3	41.87	45.9	31.2	55.4	32.88	41.9	49.97	32.57	28.91

192986	'Cyb5d2'	13.22	8.64	2.66	9.22	7.62	4.05	19.1	11.29	21.65	15.59
19299	'Abcd3'	28.85	26.66	36.2	18.3	14.9	6.23	48.2	23.16	27.25	19.28
19300	'Abcd4'	6	7.03	7.86	14.7	11.8	15.45	5.77	1.09	5.36	15.75
193003	'Pirt'	0	0	0	0	8.14	0.01	0	0	0	0
19301	'Pxmp2'	5.41	10.95	3.19	1.24	0.07	8.11	0.07	2.65	0.22	0.12
19302	'Pex2'	16.56	24.32	22	6.86	18.2	12.77	14.3	25.64	18.97	13.96
19303	'Pxn'	0	0.73	2.64	1.95	1.14	0.02	4.93	2.24	1.46	0.51
193043	'Zfp3'	0.75	0.03	5.91	7.51	17.7	16.09	22.4	8.02	6.69	19.15
19305	'Pex5'	16.7	18.35	16.7	51.4	21	27.09	10	34.63	11.61	28.24
19309	'Pygm'	3.44	12.3	12.7	7.26	9.17	19.79	5.79	10.13	8.92	5.76
193116	'Slu7'	26.67	18.34	33.3	24.7	40.5	25.11	20.2	19.33	9.2	33.22
19317	'Qk'	14.22	23.68	16.2	17.4	14.1	13.95	13.5	3.52	3.19	6.09
19324	'Rab1a'	128.1	125.7	122	182	102	146	170	132.2	141.9	120.65
19325	'Rab10'	10.1	6.56	7.24	14.6	7.2	7.71	8.05	10.74	15.22	7.21
19326	'Rab11b'	233.2	236.3	268	205	244	236.7	204	314.5	276	293.49
19328	'Rab12'	6.04	8.87	11.3	22	7.66	5.91	17.1	6.39	4.39	6.24
193286	'BC049762'	0	0.21	1.1	0	0.07	0	0	0.05	0	3.62
19330	'Rab18'	84.06	86.07	86.2	78.1	80.7	41	57.3	71.94	81.05	56.91
19331	'Rab19'	0	0	0	0	0	0	0	0	0	6.29
19332	'Rab20'	0	0	3.38	0.05	0	0.07	0	0	4.2	0.03
19334	'Rab22a'	1.27	3.96	0.73	0.6	6.65	0.07	8.47	1.43	13.61	2.87
19335	'Rab23'	5.59	10.43	4.59	6.65	2.74	2.66	16.7	6.47	5.24	2.39
19336	'Rab24'	7.12	10.34	7.11	85.4	13.3	13.92	34.7	16.71	54.85	9.02
19337	'Rab33a'	35.06	43.74	25.1	54.4	12.3	20.81	19.1	21.9	13.82	18.72
19338	'Rab33b'	24.3	20.54	16.8	29.1	14.8	13.98	27.1	25.02	20.81	15.24
193385	'Ripor2'	7.71	6.19	11.4	2.74	7.28	5.5	2.65	7.6	10.65	5.59
19339	'Rab3a'	948.3	796.3	1081	903	836	959.8	945	1124	846.9	878.72
19340	'Rab3d'	3.38	1.36	2.46	0	2.55	0.31	0	3.87	0	0
19341	'Rab4a'	78.11	96.85	77	84.8	61.6	67.11	81.5	76.16	74.15	56.45
19342	'Rab4b'	107.9	98.78	138	101	104	110.1	97.2	189.6	138.9	166.06
19344	'Rab5b'	32.43	42.89	50.6	20.4	49.3	40.12	26.8	42.06	58.12	27.91
19345	'Rab5c'	58.71	40.11	65	36.5	58.1	76.59	72.1	59.56	76	64.56
193452	'Zfp184'	0	3.72	0	9.16	2.01	2.96	5.29	3.05	2.32	4.76
19346	'Rab6a'	162.9	199.4	195	207	126	110.4	218	106.7	218.9	52.02
19347	'Dennd5a'	7.81	13.65	4.55	8.81	15.2	5.81	5.76	6.84	5.96	7.17
19348	'Kif20a'	0.01	0.1	0.03	0.03	0.05	0.07	0	0	2.28	0.89
19349	'Rab7'	247.6	238.3	258	287	237	266.6	251	309.2	242.9	291.02
19352	'Rabggtb'	59.84	61.65	54.6	99	73	60.03	47.2	62.91	61.73	51.96
19353	'Rac1'	51.27	29.12	38	184	58.7	14.88	57.9	19.75	34.48	16.18
19354	'Rac2'	2.11	0.06	0.06	3.52	1.47	0.02	0.45	0.09	0.08	0.04
19355	'Rad1'	24.2	10.62	4.8	7.99	19.8	13.45	12.7	26.83	35.12	16.44
19356	'Rad17'	12.51	12.48	6.6	5.78	11.2	2.2	5.26	8.01	3.17	6.02
19357	'Rad21'	7.25	7.73	5.46	11.5	4.07	8.5	0.12	8.21	1.85	3.33
19358	'Rad23a'	77.32	69.71	61.8	98.4	80.2	87.94	74.2	85.09	63.82	78.89
19359	'Rad23b'	4.86	13.67	9.02	17.7	9.39	1.89	12.2	10.9	16.12	5.65
19360	'Rad50'	3.69	1.36	1.73	8.63	1.55	8.16	2.58	3.8	5.55	2.89
19361	'Rad51'	7.31	5.03	1.37	0	11.7	4.16	0	5.99	0.02	0
19362	'Rad51ap1'	0.05	0.51	0	0	7.51	0	5.61	0	0.61	2.26
19363	'Rad51b'	0	3.29	0	0	0	0.05	0	0	0.04	0
19364	'Rad51d'	23.93	17.69	12.7	5.89	28.2	8.09	29.1	22.73	22.87	12.58
19365	'Rad52'	13.92	8.47	10	14.6	11.5	1.26	3.46	2.78	11.69	4.48
19366	'Rad54l'	0	0.2	0.13	0	0.87	2.13	0	0.05	0.39	0
19367	'Rad9a'	13.62	20.11	6.82	11.9	20	19.25	7.43	19.3	13.36	13.5
193670	'Rnf185'	20.29	27.8	8.31	32.1	11.1	5.71	38.8	12.12	14.74	27.91
19373	'Rag1'	0	0	0	0	0	0	0	0	0.01	0.27
193736	'Zbtb12'	0	0	0	0	0	1.98	0	0.2	2.07	0.4
19374	'Rag2'	0	0	0	0	0.01	0	0	0	0	0
193740	'Hspa1a'	11.04	80.67	77.7	40.7	18.6	59.05	5.2	13.56	30.64	24.88

193742	'Abhd16a'	90.84	98.15	118	61.7	83.5	108.2	159	135.1	102.2	105.49
19376	'Rab34'	20.07	12.98	18.9	16.9	17.2	15.41	60.6	26.13	16.43	19.79
19377	'Rai1'	2.57	2.37	3.99	2.84	1.55	4.27	4.45	4.6	1.58	3.57
19378	'Aldh1a2'	0	0	0	0	0	0	0	0	0.02	0
193796	'Kdm4b'	2.88	3.67	1.82	4.35	5.29	1.18	0.19	1.15	2.99	0.72
193813	'Mcfd2'	261.8	242.4	138	218	148	280.1	173	184.8	262.8	263.49
19383	'Raly'	21.43	24.41	32.4	41.9	25.5	53.33	48.1	51	44.54	46.5
193838	'Eme2'	30.14	37.46	35.5	48.8	39.8	25.08	49.8	34.22	50.85	40.37
19384	'Ran'	315.2	291.5	264	247	221	303.6	324	280.2	234.3	316.65
19385	'Ranbp1'	228.7	168.7	237	249	186	280.2	233	228.2	157	136.63
19386	'Ranbp2'	1.7	2.11	1.89	3.56	0.37	0.28	0.55	0.91	1.73	2.99
19387	'Rangap1'	162.2	170.1	123	72.9	81	132.6	119	131.5	106.8	158.62
19395	'Rasgrp2'	12.81	7.03	19.2	31.3	10.8	20.98	11.1	19.22	23.7	1.69
19400	'Rapsn'	0	0	0	0	0.15	0	0	0	0	0.11
19401	'Rara'	4.24	1.65	1.45	0.15	1.75	0	0	2.29	2.37	2.21
19411	'Rarg'	0	0.33	3.48	8.56	3.36	0.12	2.24	0.04	1.07	0
194126	'Mtmr11'	4.06	4.11	8.97	7.11	4.22	0	1.64	5.87	4	2.08
19414	'Rasa3'	4.73	19.22	15.9	12.2	13.3	2.48	28.5	17.08	3.78	9.29
19415	'Rasal1'	8.99	45.69	12	2.75	8.92	11.26	0.99	17.82	1.97	0.92
19416	'Rasd1'	25.65	47.57	43.5	79.5	12.5	22	2.16	27.27	25.21	52.07
19417	'Rasgrf1'	264.4	160.5	198	59.3	118	98.82	233	244.7	138.3	194.4
19418	'Rasgrf2'	4.62	5.3	7.63	17.8	2.64	6.02	7.92	5.96	5.65	3.92
19419	'Rasgrp1'	14.11	5.56	21.1	17.5	45.6	16.81	6.13	3.41	17.75	47.32
194231	'Cnksr1'	0	0.03	0	0	0	0.03	0	0	0	0
194237	'Rimkla'	1	4.99	4.48	0	4.81	0	0	1.92	2.88	0
194268	'9930104L06Rik'	6.72	10.11	3.56	7.43	7.75	0.46	10.3	8.63	12.69	2.86
19428	'Rasl2-9'	0	0	0	0	0	0	0	0	0	1.76
194309	'Vps37d'	2.17	0.57	1.69	7.05	0.55	0	3.09	0.03	5.29	0.03
194388	'Tet3'	0.66	1.99	1.47	2.52	0.54	1.45	0.43	1.61	1.08	0.87
194401	'Mical3'	2.4	4.35	3.34	7.75	3.11	5.12	5.26	2.79	4.53	7
194590	'Reps2'	0.77	2.77	2.91	4.27	4.47	1.69	2.26	1.65	4.93	5.43
194655	'Klf11'	1.4	10.68	2.84	22.5	1.54	1.94	0.33	1.33	0	1.68
194744	'Slc25a43'	0	0.39	0	0	0	0	0	0	0	2.08
194908	'Pld6'	7.85	0.05	12.9	0	18.5	1.56	0.26	11.27	8.61	2.59
194952	'Jmjd4'	5.2	8.47	5.64	1.52	7.7	6.08	14.3	4.44	8.88	8.77
194974	'Sun3'	0	0	0	0	0	0.04	0	0	0	0
195018	'Zzef1'	3.8	5.07	5.62	10.8	2.72	4.37	3.93	1.81	2.03	4.75
195040	'Tmem199'	135.9	122.1	110	120	170	217.4	85.4	150	174.3	111.01
195046	'Nlrp1a'	0.27	0.13	0.23	0.33	0.27	0.32	0.26	0.17	0.11	0.2
195208	'Dcdc2a'	10.57	3.46	1.57	0	10.7	2.39	0	6.99	4.81	0.32
195209	'Zfp469'	0	0.02	0.28	0	0	0	0	0	0	0
195236	'Pom121l2'	0	0	0	0	0	0	0	0	0	0.47
195434	'Utp14b'	1.49	0.88	4.25	2.95	0.33	2.51	0	0.02	0.01	0
195522	'Zfp691'	1.83	1.75	2.48	4.29	2.17	1.78	1.88	3.3	7.5	1.9
195531	'Zfp982'	1.11	1.13	1.33	0	0	0	0	0	0	0
195646	'Hs3st2'	0	1.35	2.25	30.9	8.18	0	0	25.23	3.63	2.81
195726	'Scml1'	0	0.07	0.69	0	0	0	0	0	0	0
195727	'Nhs'	1.27	0.5	1.16	0.85	1.32	0.34	0.84	1.64	0.36	0.24
195733	'Grhl1'	14.47	21.18	5.75	14.3	8.42	1.19	12.3	10.23	1.47	3.15
19645	'Rb1'	0	0	3.89	2.59	0.3	1.33	0	0.06	0.11	0.67
19646	'Rbbp4'	85.74	80.2	84.3	45.6	80.1	76.08	83.1	84.44	96.99	90.3
19647	'Rbbp6'	9.76	9.09	9.63	17	7.04	1.64	6.55	9.21	7.1	7.7
19649	'Robo3'	0.29	0	0	0	0.38	0	0	0.17	0.13	0
19650	'Rbl1'	0.01	0	1.78	2.29	0.02	0.08	0	0.26	0	0
19651	'Rbl2'	6.95	10.88	5.7	0.19	1.23	2.99	2.28	3.69	22.54	8.82
19652	'Rbm3'	33.99	42.64	151	256	86.2	45.37	37.2	123.3	47.98	53.56
19653	'Rbm4'	17.87	13.93	13.2	11.7	22.1	9.67	13	24.82	23.72	13.09
19654	'Rbm6'	21.81	29.34	18.5	18.5	18.3	12.52	16.7	23.71	30.64	13.48

19655	'Rbmx'	17.86	21.25	17.9	9.48	34.4	18.27	41.7	31.01	27.83	15.85
19656	'Rbmxl1'	62.63	69.86	73.1	69.2	38.4	92.67	67.9	63.95	59.98	60.34
19659	'Rbp1'	5.31	3.92	0.75	6.35	5.96	0.95	0	9.83	1.55	0.98
19660	'Rbp2'	2.34	0	0	0	0	0	0	0	0	0
19661	'Rbp3'	0	0	0	0	0	0	0	2.98	0	0.01
19662	'Rbp4'	7.65	14.2	3.61	8.98	3.3	2.21	2.35	2.57	5.75	0.1
19663	'Rbpms'	2.81	19.73	1.78	0.07	2.61	8.09	1.91	0.05	0	1.36
19664	'Rbpj'	8.86	4.58	4.26	0.98	8.59	1.92	1.68	4.12	5.04	1.17
19668	'Rbpjl'	0	0	2.09	0	0.1	0.02	6.21	0	0	0.09
19671	'Rce1'	27.45	26.28	34.6	6.1	20.9	15.9	16.1	31.91	18.18	23.82
19672	'Rcn1'	36.45	46.84	43.5	88.4	27.8	54.25	67.1	40.93	44.58	58.08
19674	'Rcvrn'	0	0	0	0.06	0	0	0	0	0	0
19679	'Pitpnm2'	1.57	2.48	2.31	1.51	2.67	1.35	0.4	2.93	1.44	0.53
19682	'Rdh5'	8.37	6.27	0	2.27	2.67	0	0.04	4.52	6.91	2.92
19684	'Rdx'	29.97	30.49	29.1	28.5	17.7	7.27	37.1	32.21	27.85	26.54
19687	'Rfc1'	6.53	3.97	7.44	8.85	9.57	1.82	3.02	6.52	3.97	6.55
19691	'Recql'	4.87	1.78	0	7.08	7.52	7.01	2.32	5.53	4.97	3.61
19696	'Rel'	0	1.02	0.13	0.2	0	4.37	0	0	0	0
19697	'Rela'	12.8	4.58	2.98	18.7	1.79	3.21	11.7	3.89	8.95	0
19698	'Relb'	0	0.68	0	0	0	0	0	0	0	0
19699	'Reln'	0.92	0.62	5.55	1.09	7.66	6.08	3.91	2.98	2.69	3.76
19700	'Rem1'	0	1.54	0	0	0	0	0	3.51	0	2.55
19703	'Renbp'	0	5.54	10.4	32.3	6.52	0	14.9	6.58	25.77	4.91
19704	'Upf1'	1.51	2.35	1.13	1.13	1.12	0.43	1.31	0.38	0.5	4.72
19707	'Reps1'	1.67	3.3	4.75	4.65	2.32	9.99	15.5	7.36	11.15	4.26
19708	'Dpf2'	27.11	20.52	25.1	39.9	34.4	11.95	35.5	23.89	27.36	32.2
19711	'Resp18'	4177	4287	2047	4052	2718	3665	1926	3619	3638	4770.5
19712	'Rest'	0	0.51	0	0	0.01	0.38	3.63	1.02	0	0
19713	'Ret'	0.56	0.63	0	1.71	0.51	0	0.66	0	0.01	1.94
19714	'Rev3l'	2.93	1.76	3.77	8.85	2.11	4.65	4.36	1.48	4.24	3.03
19716	'Bex1'	430	338.4	429	443	425	590.7	437	330.7	291.2	344.2
19718	'Rfc2'	45.15	43.21	34.4	95.2	38.8	48.82	61.4	59.67	51.41	44.78
19719	'Rfng'	23.48	26.21	33	19.5	22.1	20.06	27.8	26.48	26.35	6.17
19720	'Trim27'	8.93	7.9	3.19	17.4	19.4	5.01	0.32	3.02	6.76	14.35
19724	'Rfx1'	2.85	2.61	2.41	1.01	1.33	0.55	1.1	4.37	0.66	3.21
19725	'Rfx2'	0.08	3.35	0	0	3.16	0.01	0	2.46	0	1.67
19726	'Rfx3'	1.83	2.53	4.69	2.58	1.35	3.34	0.67	2.19	0.62	2.98
19727	'Rfxank'	1.04	0.79	5.74	6.72	2.21	11.73	10.9	6.88	7.53	9.7
19729	'Slc50a1'	62.23	51.59	30.5	52.5	49.1	62.64	66.7	50.81	39.4	56.62
19730	'Ralgds'	6.11	6.6	14	16.7	2.28	8.24	11	2.29	3.23	2.5
19731	'Rgl1'	25.15	24.48	28.4	30.8	29.1	10.98	5.78	21.35	20.33	14.92
19732	'Rgl2'	9.33	15.95	11.1	12.1	10.4	13.85	8.56	7.57	12.81	2.71
19733	'Rgn'	3.26	0	0	0	0	0	0	0	0	0
19734	'Rgs16'	20.04	20.69	16.9	21.5	27.5	6.99	5.58	31.9	25.96	1.84
19735	'Rgs2'	33.66	27.39	47.4	20.4	17.7	59.21	22.6	46.27	29.94	31.94
19736	'Rgs4'	151.8	185.5	143	193	57.8	92.36	24	115.1	160.2	151.98
19737	'Rgs5'	0	0	0	0	0	0	0.01	0	0	4.26
19739	'Rgs9'	19.85	18.32	19.3	25	14.7	20.98	43.1	26.48	33.73	29.9
19743	'Rhag'	0.02	0	0.04	0.02	0	0	0	0	1.08	0
19744	'Rheb'	156.1	168.6	133	174	138	150	99.4	172.8	166.9	129.21
19746	'Rhd'	1.09	3.12	0.06	0	0	0	0	0	0	3.77
19752	'Rnase1'	0	0	0	0	0	13.17	0	0	0	0
19762	'Rit2'	310.3	187.2	192	273	301	336.9	247	192.3	252.5	351.47
19763	'Ring1'	2.99	2.08	2.58	9.62	14.3	1.8	2.09	2.31	9.26	1.78
19765	'Ralbp1'	11.66	10.24	9.34	23.2	9.86	15.98	14.6	4.18	11.86	11.88
19766	'Ripk1'	0.04	0.58	0.25	9.93	0.54	0.17	1.18	0.01	0.13	0.02
19769	'Rit1'	41.6	51.32	38.3	21.6	48.5	47.86	39.1	25.9	32.07	44.77
19771	'Rlbp1'	0	0	16.1	5.56	0.02	0	16	0	0	0.02

19773	'Rln1'	3.94	7.72	4.27	6.24	5.5	6.43	0.08	8.82	4.57	12.56
19775	'Xpr1'	8.77	9.68	7.21	10.1	3.69	6.87	4.68	5.17	12.31	7.86
19777	'Uri1'	14.66	8.44	6.9	8.78	19.1	5.28	7.38	6.52	11.44	6.13
19819	'Rnaseh1'	18.04	6.18	7.87	12.9	11.1	15.13	12.4	18.52	20.7	6.71
19820	'Rlim'	7	10.37	15	9.85	6.39	3.09	3.5	4.53	4.23	6.92
19821	'Rnf2'	17.45	12.1	13.4	10.2	11.6	1.52	22	16.41	10.32	5.28
19822	'Rnf4'	56.09	49.42	38.1	58.7	36.1	28.46	50.6	46.97	38.49	37.47
19823	'Rnf7'	160.3	162.4	107	100	182	160.3	156	94.47	127.6	158.68
19824	'Trim10'	0	0	1.84	0	0	0	0	0	0	0
19826	'Rnps1'	154.1	153.6	123	170	120	146.9	162	160.1	210	152.17
19876	'Robo1'	2.27	0.25	2.19	0.24	2.55	1.42	1.41	0.57	3.47	2.2
19877	'Rock1'	6.21	1.71	1.95	7.9	2.09	4.37	2.36	2.45	3.48	0.63
19878	'Rock2'	4.07	2.62	5.13	9.74	2.73	6.7	0.77	2.48	5.25	5.16
19879	'Slc22a8'	0	0	0	0	0.04	0	0	0	0	0
19881	'Rom1'	7.25	0	0.29	0	8.95	0	0	1.01	10.68	15.42
19882	'Mst1r'	0	2.12	0	0	0	0	0	0	0	2.54
19883	'Rora'	2.19	3.11	1.17	8.04	1.12	0.97	6.18	1.21	0.32	2.47
19885	'Rorc'	0	0	0.02	0	0.03	0	0.03	2.31	0	0.02
19886	'Ros1'	0	0	0	0.01	0	0	0	0	0	0
19889	'Rp2'	1.5	1.22	0.03	0.03	4.21	8.96	0.23	2.27	0.72	3.26
19891	'Rpa2'	9.55	16.89	3.77	12.9	10.4	8.64	17.7	7.63	12.59	22.13
19893	'Rpgr'	5.07	5.97	4.64	8.2	13.6	10.13	8.78	7.87	10.97	12.89
19894	'Rph3a'	20.43	21.51	29.7	4.55	45.4	3.41	35.4	20.66	15.12	10.42
19895	'Rpia'	0.03	0.05	2.02	0	0.61	0	0	0.8	1.49	0
19896	'Rpl10a'	182.6	166	177	205	119	199.9	118	154.1	123.8	139.21
19899	'Rpl18'	293.5	206.5	251	295	199	330.2	336	222.9	250.1	302.37
19921	'Rpl19'	578.6	477.9	705	907	657	670.4	749	624.1	673.9	818.62
19933	'Rpl21'	143.1	137.2	152	161	107	163.9	157	108.2	130.6	124.53
19934	'Rpl22'	5.48	6.16	6.54	3.73	6.67	3.18	8.32	5.47	1.55	10.62
19935	'Mrpl23'	225.2	209.8	187	171	177	243.9	106	156	197.6	245.45
19941	'Rpl26'	338.2	323.8	350	361	288	421.7	339	310.1	363.6	247.85
19942	'Rpl27'	196	176.4	283	223	181	238.8	199	147.8	173.2	170.43
19943	'Rpl28'	299.6	229.6	361	257	255	392.9	356	268.2	343.3	352.62
19944	'Rpl29'	137.3	122.1	120	87.2	130	197.1	171	147.9	129	149.24
19946	'Rpl30'	180.5	202.5	209	231	118	235.1	155	148.8	159.4	139.86
19951	'Rpl32'	348.9	334.5	333	365	233	284	262	209.3	235.4	213.87
19981	'Rpl37a'	206.2	276.2	341	418	148	386	184	117.7	294.6	166.45
19982	'Rpl36a'	71.64	109.4	108	165	69.8	128.1	99.5	53.45	79.6	81.98
19988	'Rpl6'	402.6	366.3	406	502	326	426.2	388	319.3	311.1	330.96
19989	'Rpl7'	169.9	212.6	205	163	222	162.6	225	146.5	140.9	178.6
20005	'Rpl9'	507.4	402.8	359	689	409	452.8	284	366	291.3	282.74
20014	'Rpn2'	153.4	156.5	155	130	147	136.7	113	152.6	212	212.53
20016	'Polr1c'	37.87	38.24	19.2	20.8	28.4	48.9	50.5	28.97	42.49	29.02
20017	'Polr1b'	3.11	7.34	6.46	18.9	7.85	0.81	9.59	0.98	2.62	9.72
20018	'Polr1d'	92.19	73.21	85.8	113	89.3	79.15	45.7	87.6	76.37	90.15
20019	'Polr1a'	6.32	2.45	6.18	7.53	1.48	4.82	11.9	6.4	2.75	4.45
20020	'Polr2a'	2.33	2.09	4.95	1.03	3.4	2.58	2.28	3.17	4.2	2.51
20021	'Polr2c'	148.7	133.6	136	187	113	152.5	104	140.2	129.2	135.69
20022	'Polr2j'	108.2	142.4	139	129	142	127	151	156.8	122.6	105.8
20024	'Sub1'	189.2	194.7	143	264	142	150.3	145	218.9	185	177.97
20042	'Rps12'	54.16	68.52	78.1	111	86	94.06	50.9	62.68	70.24	37.83
20044	'Rps14'	447.3	380.7	326	682	406	583	374	290.6	339.6	371.73
20054	'Rps15'	297.7	268.7	333	429	301	350.9	400	247.1	325.8	337.25
20055	'Rps16'	301.8	266.2	281	263	203	304.1	276	228.8	227.2	232.46
20068	'Rps17'	383	318.8	457	553	203	378.8	333	202.9	258.8	222.91
20084	'Rps18'	208.4	229.4	200	357	213	286.8	211	151.9	128.9	162.49
20085	'Rps19'	327.3	405.8	427	554	324	345.5	302	227.9	254.2	258.23
20088	'Rps24'	208	199.5	234	242	161	229.3	254	167.1	176	168.44

20090	'Rps29'	144.9	288.2	324	418	131	465.6	215	69	314.2	106.79
20091	'Rps3a1'	194.1	180.6	253	213	211	228.1	324	191.5	146.2	232.94
20102	'Rps4x'	197.9	172.9	152	284	197	148.1	172	121.2	85.78	142.64
20103	'Rps5'	184.2	230.5	245	285	242	270.1	208	193.8	153.8	173.83
20104	'Rps6'	22.7	43.75	15.5	22.1	20.6	18.15	18.3	24.97	10.78	17.14
20111	'Rps6ka1'	5.05	0.06	0.05	0.02	2.3	19.26	0.02	0.33	5.1	2.85
20112	'Rps6ka2'	6.7	11.36	8.2	6.63	9.61	4.8	8.44	13.42	8.34	20.34
20115	'Rps7'	162	159.1	155	169	174	201.9	157	141.5	115.6	164.9
20116	'Rps8'	283.6	264.5	358	486	246	341.8	253	251.4	231.3	232.16
20128	'Trim30a'	0	0	2.33	0	0	4.81	0	0	0	0.01
20130	'Rras'	0.68	0	0	0	0	0	0	3.31	0	3.53
20133	'Rrm1'	3.75	5.23	4.44	8.16	7.89	6.26	5.95	2.38	6.73	4.16
20135	'Rrm2'	0	0.31	0.02	0	2.66	0	0	0	0.08	0
20147	'Rs1'	0	0.04	0	0	0.12	0	0	0.26	0	0
20148	'Dhrs3'	13.25	14.43	4.36	10.1	0	15.18	11.6	7.15	8.72	7.62
20163	'Rsu1'	9.94	0	12	14.9	13.4	19.33	7.82	11.97	7.66	13.16
20166	'Rtkn'	8.92	9.15	30.4	2.92	5.44	17.72	7.58	12.97	17.81	3.51
20167	'Rtn2'	3.72	6.1	2.16	12.2	1.97	11.51	15.8	9.2	7.96	8.95
20168	'Rtn3'	518.2	522.7	573	294	427	445.9	630	544.2	422.1	437.74
20170	'Hps6'	2.01	10.71	2.14	14.2	4.57	5.89	0.02	2.3	12.4	0.02
20174	'Ruvbl2'	27.97	40.48	56.1	44.5	54.2	52.97	59.4	67.45	50.53	33.95
20181	'Rxra'	2.77	0.2	1.23	2.01	1.52	0.89	2.5	4.47	5.26	0.79
20182	'Rxrb'	6.6	5.46	3.74	4.46	13.4	1.41	0	5.15	2.32	5.3
20183	'Rxrg'	20.34	39.93	42.4	2.51	28	57.19	21.2	83.42	49.81	49.91
20184	'Uimc1'	16.36	16.23	7.43	7.32	7.29	7.42	2.11	6.92	2.56	3.1
20185	'Ncor1'	6.26	8.77	6.32	11.8	11.2	10.13	9.37	5.02	4.86	8.86
20186	'Nr1h4'	0	0	0	0.08	0	0	0	0	0	0
20187	'Ryk'	2.12	1.62	0	0.73	2.56	1.8	0	1.54	1.08	1.24
20190	'Ryr1'	0.81	0.22	0.15	0.02	0.48	2.41	0.85	0.38	0.02	0.68
20191	'Ryr2'	0.44	0.54	2.88	0.33	2.19	0.2	2.46	0.8	0.44	1.4
20192	'Ryr3'	1.27	1.29	1.31	0.71	2.65	3.44	0.36	2.1	3.47	2.2
20193	'S100a1'	47.8	44.59	151	107	137	138	98.7	51.43	58.31	47.06
20194	'S100a10'	81.37	24.85	78.2	13.4	88	66.42	19.8	66.87	28.1	59.28
20195	'S100a11'	9.43	0.32	31.9	0	9.84	0	0	2.31	3.65	12.92
20196	'S100a13'	12.95	6.29	16.7	6.07	8.57	23.9	20.6	4.08	3.09	3.07
20197	'S100a3'	0	0	1.79	0	0	0	0	0	0	0
20198	'S100a4'	0	0	0	0.13	13.6	0	15.1	0	0	3.08
20200	'S100a6'	3.35	0.64	7.62	0	13.4	0.07	4.44	0	0	9.08
20201	'S100a8'	0	0	5.12	0	0	0	0	0	0	0
20203	'S100b'	13.78	15.87	21.4	36.9	116	28.47	31.5	14.14	7.38	6.65
20208	'Saa1'	0	24.99	0	0	0	0	0	5.8	6.43	0
20209	'Saa2'	0	3.15	0.09	0	0	0	0	20.14	0	0
20215	'Sag'	2.95	7.01	0.94	3.38	0.11	0	1.13	4.18	0	1.8
20216	'Acsm3'	0	0	0	0	0.11	0	0	0	0	0
20218	'Khdrbs1'	13.89	10.6	17.6	20.4	10.3	1.12	17.7	11.19	12.08	11.89
20220	'Sap18'	41.51	55.87	45.3	51.2	38.1	39.19	25.6	31.23	31.26	43.31
20222	'Sf3a2'	5.43	6.73	9.85	4.23	13.1	1.95	2.22	8.49	25.26	15.91
20224	'Sar1a'	143.4	122.2	113	105	88.7	85.23	108	111.8	118.7	147.47
20226	'Sars'	70.65	50.9	73.8	40.5	69.7	74.07	71.6	78.24	65.05	57.69
20227	'Sart1'	2.77	7.8	4.29	7.78	1.08	5.72	4.87	7.81	4.77	3.31
20229	'Sat1'	43.19	96.63	45.9	154	33.3	117.8	40.5	44.25	86.39	62.05
20230	'Satb1'	0.38	2.07	2.83	0	1.35	2.96	3.25	2.1	0.22	0.48
20238	'Atxn1'	8.74	8.78	7.12	6.15	11.9	9.13	9.75	10.5	9.95	2.58
20239	'Atxn2'	6.57	5.22	6.22	15.5	8.95	4.53	7.66	7.84	4.5	10.43
20249	'Scd1'	2.23	2.3	3.29	7.47	11.9	0.77	8.65	6.02	4.85	5.78
20250	'Scd2'	67.99	45.35	72.7	46.5	40.8	23.11	54.1	32.63	46.84	55.67
20254	'Scg2'	1962	1985	717	2180	411	911.1	773	1300	1337	1307.36
20255	'Scg3'	102.3	126	92.1	142	69.4	73.74	62.1	87.26	75.29	79.31

20256	'Clec11a'	3.5	0	8.08	0.2	7.77	2.36	0.03	2.24	3.91	8.78
20257	'Stmn2'	302.7	261.4	294	233	363	299.8	218	324.9	294.3	298.82
20262	'Stmn3'	1380	1364	1655	1186	1498	1957	1592	1418	1079	1298.67
20265	'Scn1a'	11.62	2.54	22.7	12	13.8	2.63	6.07	6.5	6.69	4.69
20266	'Scn1b'	15.74	8.82	23.4	12.7	37	7.41	18.8	23.76	12.19	9.44
20269	'Scn3a'	9.91	6.52	10.8	20.9	8.71	5.07	9.5	8.07	12.09	16.91
20271	'Scn5a'	1.78	0.32	0.97	0.01	2.88	0	0.05	1.24	0.68	1.17
20272	'Scn7a'	0	0	0	0	0	0	0.4	0	0	0.05
20273	'Scn8a'	3.89	1.25	5.33	10.5	6.9	1.03	2.72	3.23	5.74	3.23
20274	'Scn9a'	25.45	8.58	6.36	18.7	10.2	11.63	4.46	7.13	16.27	13.63
20276	'Scnn1a'	0	0	0	7.07	0	0	0.04	1.28	3.71	2.24
20277	'Scnn1b'	0	6.09	0	0	0	0	0	0	0	0
20280	'Scp2'	55.31	48.05	47.5	45.3	54.5	74.22	63.2	54.79	60.77	60.92
20284	'Scrg1'	18.69	17.56	46	79.6	24.9	31.4	59.7	17.39	16.26	14.07
20286	'Zc3h7b'	4.39	3.55	0.84	0.11	3.24	1.96	0.24	1.57	1.94	2.76
20287	'Sct'	0	6.45	0	0	0	2.51	0	0	0	17.81
20289	'Scx'	0.16	0	1.09	0	0	2.36	2.54	0.94	0	1.89
20293	'Ccl12'	1.37	0	0	38.6	0.1	12.77	5.14	0	0	7.34
20295	'Ccl17'	1.92	0	13.8	8.69	6.34	0	0.11	1.22	0	4.74
20296	'Ccl2'	22.82	6.27	0	13.4	0	0	0	0	1.81	0
20297	'Ccl20'	0	0	0	0	0	0	0	0	0	3.94
20299	'Ccl22'	5.89	0	0	0	0	0	0	0	0	0
20300	'Ccl25'	3.1	13.78	7.56	0.06	0	14.56	1.79	4.86	11.98	8.35
20301	'Ccl27a'	132.1	133.1	112	146	144	126.8	88.3	141.4	214.7	201.65
20302	'Ccl3'	5.8	0	0	7.42	0	89.05	0	0	5.94	2.4
20303	'Ccl4'	46.75	0	0	0	0	91.37	6.43	0.33	3.59	2.59
20305	'Ccl6'	1.65	4.58	0	0	2.82	3.39	7.5	3.43	0	1.27
20306	'Ccl7'	4.37	0	0	0	0	15.2	0	0	0	0
20307	'Ccl8'	0	0	0	0	0	0	0	0	0.09	0
20308	'Ccl9'	1.3	0.13	0.18	0.12	0.05	0.09	0.13	0.01	0.04	0.17
20309	'Cxcl15'	0.04	0	0	0.03	0	0	0	0	0.02	0
20311	'Cxcl5'	0	0	0	0	0	1.81	0	0	0	1.92
20312	'Cx3cl1'	148.3	132	137	34	114	94.51	128	120.6	105.9	92.46
20315	'Cxcl12'	0	0.13	0	0	0.05	0	0.03	0	0	5.38
20316	'Sdf2'	186.3	196.6	117	229	151	195.3	140	148.5	195.8	223.05
20317	'Serpinf1'	9.99	0	0.07	0.7	48.7	166.8	11.5	10.25	100.3	99.58
20318	'Sdf4'	81.52	98.53	87	98.1	58.9	72.96	79	67.02	93.3	104.8
20319	'Sfrp2'	0.84	1.59	4.43	0	4.71	3.91	6.01	2.81	31.24	22.4
20320	'Nptn'	179.2	189.9	178	90.5	229	165.4	187	277.7	252.4	278.4
20321	'Frrs1'	1.62	0.64	0.55	0.01	0.21	1.45	0	0	2.14	0.96
20322	'Sord'	4.78	17.46	8.99	9.59	12.7	16	11.1	9.86	7.59	17.23
20324	'Cavin2'	0.07	5.89	3.35	2.2	0	3.35	4.75	1.67	0.14	0.57
20333	'Sec22b'	57.18	57.14	55.1	64.7	31.5	74.21	56.4	49.5	41.43	52.21
20334	'Sec23a'	33.25	35.26	40.7	30.5	34.8	4.55	41.1	30.26	41.54	43.7
20335	'Sec61g'	111.3	136.2	143	191	96	210.1	92	71	145.3	109.31
20336	'Exoc4'	13.32	11.65	17.5	6.41	17.3	5.75	20.7	10.01	13.48	12.96
20338	'Sel1l'	41.41	36.08	35.8	30	26.7	21.12	34.4	33.1	42.21	49.74
20340	'Glg1'	15.56	14.33	14.8	27.2	16	16.01	21.5	9.47	29.45	20.82
20341	'Selenbp1'	1.29	5.33	0.03	17.1	6.8	8.55	10.5	7.31	4.36	7.13
20342	'Selenbp2'	0	0.33	0	0.47	0	0.23	0.25	0.13	0.11	0.16
20344	'Selp'	0	0.1	0	0	0	0	0	0	0	0
20345	'Selplg'	0.67	5.47	0.05	0.03	4.55	117.2	7.52	2.76	11.4	5.19
20346	'Sema3a'	2.59	8.72	2.63	4.68	0.47	0	0.04	1.61	1.5	1.7
20347	'Sema3b'	2.42	0	0	0	1.87	0	4.36	0	2.27	0
20348	'Sema3c'	0.63	0	2.01	0	5.48	1.65	0	1.07	10.62	4.99
20349	'Sema3e'	2.06	2.69	3.74	4.78	1.16	0	0.01	1.85	0.94	0.63
20350	'Sema3f'	2.31	0	4.83	0	1.71	0.01	0	0.48	5.49	1.97
20351	'Sema4a'	88.36	60.04	38.7	20	38.8	64.1	69	85.55	56.16	88.37

20352	'Sema4b'	1.47	2.68	3.41	7.96	2.74	1.37	2.51	8.05	5.1	4.03
20353	'Sema4c'	0.36	0.18	0.04	0.51	0	0	0.31	0	1.33	0.09
20354	'Sema4d'	5.43	0.96	3.72	4.91	8.85	9.89	6.18	8.47	7.61	2.52
20355	'Sema4f'	2.69	4.44	8.13	3.84	3.6	2.38	14.5	2.2	5.39	4.94
20356	'Sema5a'	2.8	0.86	4.18	2.21	3.17	1.29	3.37	0.27	0.51	3.66
20357	'Sema5b'	0.8	0.52	6.88	5.88	0.12	3.11	1.69	0.35	9.87	8.14
20358	'Sema6a'	9.56	12.54	8.86	2.67	5.48	3.05	12.2	7.72	9.87	18.1
20359	'Sema6b'	2.16	1.58	2.65	2.28	2.67	0.12	3.37	1.38	3.49	0.29
20360	'Sema6c'	0.02	0.05	0.22	0.88	0.23	0.01	0.48	1.09	1.85	0.06
20361	'Sema7a'	0	0.04	1.31	0	0.3	0.04	0.65	0.52	0.36	0
20362	'Sept8'	42.7	37.92	43.4	37.1	29.3	47.95	48.3	31.62	56.41	6.54
20363	'Selenop'	151.1	83.97	29.7	76.4	54.9	64.13	74.7	34.85	31.42	27.63
20364	'Selenow'	1132	1054	893	1879	1634	1281	931	1380	1149	1025.19
20365	'Serf1'	29.41	46.08	32.5	30.2	30.8	22.48	47.2	23.76	46.73	34.72
20370	'Sez6'	19.31	10.43	41.2	11.4	7.01	35.65	44.5	28.04	33.65	19
20375	'Spi1'	0.46	0	0	0	0	15.43	5.07	2.1	0	0
20377	'Sfrp1'	0.02	0.37	0	0.77	0.02	0	0	1.19	0	0
20378	'Frzb'	0	0	0.41	0	0.03	0	4.37	0	0.01	0
20379	'Sfrp4'	0	2.28	3.9	0	0	0	11	0	0	0
20382	'Srsf2'	54.73	31.65	49.1	91.2	122	64.63	62.1	75.67	86.87	70.81
20383	'Srsf3'	62.38	81.18	66.7	41	69.1	78.12	61	60.73	56.62	78.61
20384	'Srsf5'	204.9	267.2	239	293	310	316.4	327	250	305.8	296.91
20388	'Sftpb'	0	1.24	0	0	0	0	0	0	0	0
20389	'Sftpc'	0	0	0	0	0	0	6.1	0.47	0	0
20391	'Sgca'	0	0	0.14	0.04	0	0.03	0.04	0.03	0	0.03
20392	'Sgce'	45.55	23.3	29.4	11.8	11.4	16.44	22.9	36.19	35.46	33.02
20393	'Sgk1'	16.16	7.94	21.8	8.16	5.71	58.34	9.55	5.05	17.68	6.92
20394	'Scg5'	516.2	598.9	514	737	665	657.3	540	584.5	606	674.64
20397	'Sgpl1'	1.96	4.94	14.2	12.1	1.25	0.39	6.19	4.38	6.06	1.17
20399	'Sh2b1'	31.87	16.05	21.7	8.52	31.5	39.98	31.5	22.63	13.05	33.63
20400	'Sh2d1a'	0	0	0	0	0	0	0.62	0	0	0.3
20401	'Sh3bp1'	0	2.26	5.36	1.38	1.69	4.8	13.4	4.22	3.71	0.63
20402	'Zfp106'	3.8	1.82	5.31	7.02	7.03	2.31	6.28	3.31	7.49	3.42
20403	'Itsn2'	8.18	0.56	10.2	0.72	4.13	6.25	8.54	2.98	9.42	2.85
20404	'Sh3gl2'	148.5	97.54	150	46.2	90.3	119.3	151	134.8	85.49	90.7
20405	'Sh3gl1'	42.48	26.34	21.7	16	19.9	34.52	23.4	30.45	33.32	21.97
20408	'Sh3gl3'	15.47	36.66	5.22	5.34	19.6	14.46	10.2	5.23	11.64	12.72
20409	'Ostf1'	9.27	6.75	9.58	6.49	7.64	22.38	15	13.72	16.72	13.55
20410	'Sorbs3'	2.01	3.07	9.93	3.24	9.19	0.84	0	0.02	0	3.94
20411	'Sorbs1'	28.37	20.29	17.7	18.6	21.5	22.44	11.5	30.77	18.81	13.73
20416	'Shc1'	0.24	4.31	2.31	9.86	3.17	0.01	0	0	1.4	2.13
20418	'Shc3'	2.38	0.76	0	11.6	1.78	0.45	0	0.05	0.08	0
20419	'Shcbp1'	0	0.65	0.86	0	0	0	0	0	0	0
20420	'Shd'	49.94	38.16	84.8	52.5	93.9	47.31	82.4	41.08	28.89	34.86
20422	'Sem1'	257.9	233.9	268	373	268	327.1	211	173.6	304.7	206.08
20423	'Shh'	3.9	0	6.41	1.78	1.1	0.05	0	0.51	1.88	0.06
20425	'Shmt1'	0	0	3.17	0.12	0	0.1	5.55	0	0	0
20429	'Shox2'	0	0	0	0	1.13	0	0	0	0	0.01
20430	'Cyfip1'	8.21	5.92	17.2	14.7	7.7	12.09	21.6	11.57	21.07	8.67
20431	'Pmel'	0	0	1.57	0	0.07	0	0	2.96	0	0.28
20437	'Siah1a'	0.06	3.99	0.3	0.46	0.68	0.39	2.38	0.86	1.59	0.89
20438	'Siah1b'	0	2.06	5.78	10.5	5.72	0	4.28	5.98	2.1	5.32
20439	'Siah2'	1.46	1.65	1.33	8.82	0.53	2.9	3.08	0	0	3.53
20440	'St6gal1'	0	1.09	3.9	0	1.82	0.71	2.43	2.07	2.82	0.14
20441	'St3gal3'	7.22	6.55	13.2	10	0.39	2.5	6.04	7.72	5.94	5.48
20442	'St3gal1'	1.57	0	0.03	3.17	0.02	0	0	1.27	0	0.63
20443	'St3gal4'	8.36	20.05	9.15	37.2	4.54	3.38	8.33	11.1	7.36	9.47
20444	'St3gal2'	3.37	2.36	5.37	1	0.48	1.47	3.37	2.61	4.46	4.38

20445	'St6galnac1'	0	0	0.62	0	0	0.78	0	0	0	0
20446	'St6galnac2'	0	0	0	0	0	0	0	0.06	0	0
20447	'St6galnac3'	0.23	1.26	1	0	0.12	1.14	0.66	0.97	0.56	0
20448	'St6galnac4'	16.21	9.85	4.99	1.47	15.3	9.18	4.52	4.1	15.02	9.68
20449	'St8sia1'	0.01	0.43	0.61	1.03	0.41	0	2.73	0	2.98	1.28
20450	'St8sia2'	0	2.1	3.51	0.82	2.98	1.38	6.58	0	0.5	3.51
20451	'St8sia3'	26.36	23.85	20	16.5	17.2	31.37	28	20.33	20.13	34.59
20452	'St8sia4'	0.12	1.23	1.47	0	3.85	0.03	1.08	0.12	0.6	2.57
20454	'St3gal5'	21.56	14.59	4.41	76.5	19.6	19.24	15.1	15.51	23.42	12.49
20459	'Ptk6'	0	0	0	0.05	0	0	0	0	0	0
20460	'Stil'	0	0	0	0	0.07	0	3	0	0	0
20462	'Tra2b'	110.3	131.9	90.1	183	75.7	105.3	66.8	104.1	122.4	121.08
20463	'Cox7a2l'	244.4	96.73	150	167	159	199.2	138	175	154.9	132.11
20464	'Sim1'	0.14	2	0	0	0.01	0	3.37	0	0	0
20465	'Sim2'	0	0.51	0	0	0	0	0	0.03	0	0
20466	'Sin3a'	4.06	4.14	7.03	1.71	3.31	0.6	6.61	2.95	13.36	4.39
20467	'Sin3b'	38.49	78.96	72	58.8	49.4	68.5	71.6	56.6	75.01	65.93
20469	'Sipa1'	5.64	1.47	7.96	10.2	6.56	19.18	7.14	4.37	9.27	1.38
20471	'Six1'	0	0	0	0	1.68	0	0.02	0	0	0.46
20473	'Six3'	0.81	5.6	3.57	5.03	11.6	7.68	1.17	1.15	4.11	14.02
20474	'Six4'	0	0	0.47	0	0	0	0	0	0	0
20476	'Six6'	0	0	0	0	18.8	0	0.01	0	0	0.06
20479	'Vps4b'	22.73	28.49	25.2	11.4	13.4	14.44	19.6	17.59	28.42	48.3
20480	'Clpb'	8.94	14.97	12.9	5.06	14.1	11.26	16.6	17.65	21.97	22.15
20481	'Ski'	2.05	2.66	2.27	9.48	3.2	0.63	4.27	1.98	1.51	3.25
20482	'Skil'	6.45	6.32	3.98	11.5	6.38	6.58	3.96	2.62	5.08	10.04
20491	'Sla'	0	0	0.05	0	0	0	0	0	0	0
20492	'Slbp'	82.44	94.17	78.9	93.8	62	73.77	73.5	82.1	66.95	57.87
20493	'Slc10a1'	0.06	0.1	0.1	0.31	0.23	0.06	0.13	0.1	1.35	1.33
20494	'Slc10a2'	0	0.01	0	0	0.01	0	0	0	0	0
20495	'Slc12a1'	0	0	0	0	0	0	0.89	0	0	0
20496	'Slc12a2'	0.03	0.04	1.42	6.14	1.25	0.4	0.71	0	1.39	1.08
20497	'Slc12a3'	0	0	0	0	0.04	1.45	0	0.15	0	0
20498	'Slc12a4'	2.18	0.67	10.5	7.64	0.01	0.02	8.81	2.85	4.97	0
20499	'Slc12a7'	1.02	0.01	0.01	0.51	9.52	3.72	0.01	0	0	10.58
20501	'Slc16a1'	5.83	6.49	7.31	6.23	7.35	1.68	0.02	0.73	7.66	1.29
20502	'Slc16a2'	0	5.78	2.48	8.14	3.09	0.86	2.69	1.99	5.33	3.32
20503	'Slc16a7'	1.18	0.38	1.58	1.24	0.12	0.09	5.48	1.37	0.12	1.66
20509	'Slc19a1'	15.01	12.4	32.1	0	9.53	23.4	15.4	12.02	18.44	14.44
20510	'Slc1a1'	23.53	13.01	31.8	15.9	11.9	12.75	5.51	13.59	11.17	11.11
20511	'Slc1a2'	10.31	8.83	6.79	7.74	6.39	6.17	16.9	3.14	7.92	6.39
20512	'Slc1a3'	87.98	66.67	39.7	101	22.5	45.73	41.7	29.26	43.22	39.61
20513	'Slc1a6'	9.16	4.49	6.95	7.41	6.11	7.08	3.11	11.95	10.27	1.92
20514	'Slc1a5'	0	0	0.53	0	1.58	1.37	0	1.31	3.64	0
20515	'Slc20a1'	13.51	17.1	22	28.7	8.55	11.68	5.37	19.51	22.63	25.21
20516	'Slc20a2'	12.89	14.58	13.8	6.45	6.64	8.97	14.7	8.67	13.12	15.75
20517	'Slc22a1'	0	0	0	0	0	1.01	0	0	0	0
20518	'Slc22a2'	0	0.38	0	1.17	0	0	0	0	0	0.34
20519	'Slc22a3'	0	4.23	2.89	0	0.03	0	0	1.15	0.63	0
20520	'Slc22a5'	2.92	2.59	0	1.31	0.09	0.05	1.94	4.52	7.51	10.88
20521	'Slc22a12'	0.16	0	0	0	0	0	0	0	0	0
20522	'Slc23a1'	0.92	0.22	0.19	0.14	0.13	0.06	0.19	0.08	0.2	0.19
20523	'Slc25a14'	117.6	93.99	71.9	85	67.6	49.55	83.4	76.75	125.4	108.02
20524	'Slc25a17'	58.08	65.61	47.8	39.3	59.3	36.46	50.1	83.26	52.91	21.38
20525	'Slc2a1'	21.72	34.64	33.5	4.91	26.7	30.59	26.3	19.04	0.02	23.43
20526	'Slc2a2'	0	0	0	0.02	0	0	2.32	0	0	0
20527	'Slc2a3'	18.49	18.97	43.7	21.5	23.4	16.85	9.52	20.01	37.7	47.08
20528	'Slc2a4'	0	0	0.07	0.05	0.02	0.02	0.02	0	0.02	0.04

20529	'Slc31a1'	9.57	12.73	13.4	6.67	7.83	9.73	0	6.44	11.02	8.11
20530	'Slc31a2'	6.27	6.71	7.64	0.62	0.03	4.89	4.48	12.22	4.56	15.51
20532	'Slc3a1'	0	0	0	0	0	0	0.06	0	0	0.05
20534	'Slc4a1ap'	26.35	18.13	14.1	2.93	11.9	25.26	18.6	16.07	11.97	23.7
20535	'Slc4a2'	3.16	1.76	0.83	0	1.37	1.22	2.8	0.42	3.64	3.77
20536	'Slc4a3'	16.8	16.76	23.8	3.62	21.9	10.26	17.4	9.91	6.63	3.55
20538	'Slc6a2'	0.01	0	0.01	0.01	0.01	0	0	0	0.01	0
20539	'Slc7a5'	1.09	0.36	1.3	2.67	0.58	2.51	4.53	2.39	6.52	0.88
20540	'Slc7a7'	0	3.37	0.24	0	0	5.68	0	2.26	0	0
20541	'Slc8a1'	4.91	2.97	4.85	2.24	10.3	5.84	5.06	4.09	4.88	0.99
20544	'Slc9a1'	1.97	3.46	8.02	5.48	1.86	3.93	3.38	4.38	0.56	2.89
20556	'Slfn2'	1.07	0	0.03	0	0	0	0	0	0	0
20562	'Slit1'	15.94	29.39	9.46	0.04	12.3	13.03	15.1	18.42	30.16	28.84
20563	'Slit2'	0.4	0.05	1.2	1.91	2.38	0.58	1.11	1.02	3.39	1.64
20564	'Slit3'	1.24	1.07	0.22	0.35	1.93	0	0.37	2.08	0.02	0.3
20583	'Snai2'	0.02	3.06	0.22	5.5	0	0.3	0	0	1.3	0
20585	'Hltf'	4.08	2.1	2.47	1.35	1.62	2.07	5.02	6.26	7.3	6.9
20586	'Smarca4'	13.82	23.41	19.8	11.6	34.2	22.55	30.1	24.38	24.07	26.68
20587	'Smarcb1'	89.34	93.08	124	118	68.2	97.89	79.5	125.5	74.56	83.83
20588	'Smarcc1'	1.07	3.25	2.6	5.53	1.79	3.28	1.96	3.89	6.2	2.18
20589	'Ighmbp2'	4.68	2.19	6.09	0.23	0.02	9.12	9.13	6.64	0.04	1.54
20591	'Kdm5c'	1.38	3.5	3.61	10.8	3.75	1.19	2.45	1.8	6.66	5.32
20592	'Kdm5d'	2.34	1.55	0.02	0	2.04	0.42	9.69	1.85	0.9	2.76
20595	'Smn1'	18.83	10.01	21.2	25.7	14.5	13.4	18.2	17.29	18.81	16.69
20597	'Smpd1'	123.7	107.3	79.9	67.4	101	133	142	97.35	112.5	111.23
20598	'Smpd2'	29.36	30.94	22.2	29.7	0.18	46.37	12.9	30.69	26.48	46.37
20602	'Ncor2'	4.45	3.12	7.43	6.58	5.7	2.65	3.53	2.99	3.89	3.05
20603	'Sms'	22.96	16.86	56.1	37.6	25.2	6.46	34.7	28.29	29.68	19.61
20604	'Sst'	0.24	0	0.17	0.62	8.1	0.08	428	5.11	2.12	17.23
20605	'Sstr1'	5.99	15.78	16.3	3.7	6.48	3.91	28.2	15.07	5.59	5.31
20606	'Sstr2'	7.96	22.2	15	10.1	0.04	6.78	5.85	6.69	20.23	3.28
20607	'Sstr3'	1.19	0	0	3.17	1	0.35	3.68	0	0	0
20608	'Sstr4'	0	0	1.02	0	0	0.07	0	0	1.51	0
20609	'Sstr5'	5.77	0	6.39	7.85	2.9	2.78	0.04	0	0	2.52
20610	'Sumo3'	132.1	116.3	158	182	132	166.7	124	113.9	137.5	133.66
20611	'Ssty1'	0	0	0.19	0	0	0	0	0	0	0
20613	'Snai1'	0	0	0	5.61	0	0	0	0	1.66	0.5
20614	'Snap25'	802	656.4	810	563	1257	907.8	865	1180	877.3	861.84
20615	'Snapin'	65.22	53.87	38.9	60.9	65.5	82.48	61.7	45.09	61.05	54.47
20616	'Snap91'	50.52	39.68	34.2	25.8	40.3	24.3	52.1	44.96	38.05	23.33
20617	'Snca'	88.22	194.6	120	45.4	285	151.1	219	276.5	92.42	55.63
20618	'Sncg'	15.78	9.06	14.8	9.99	21.3	129.9	78.6	0	54.18	104.13
20619	'Snap23'	4.89	6	9.8	0.06	1.65	6.8	4.72	1.88	0.84	0.75
20620	'Plk2'	27.19	35.11	47.9	137	14.5	37.8	36.6	30.56	44.08	40.73
20621	'Snn'	10.39	9.1	14.3	11.5	4.59	5.6	21.2	8.23	14.5	4.07
20623	'Snrk'	31.36	42.08	48.9	21.3	21.8	18.74	55.8	26.16	44.66	14.08
20624	'Eftud2'	34.95	32.21	29.9	24.5	26.8	14.97	33.9	30.06	26.72	40.28
20630	'Snrpc'	99.07	63.38	98.1	132	93.1	83.19	94.7	53.75	76.68	95.87
20637	'Snrnp70'	72.96	85.49	71.2	66	85.3	65.44	61.8	61.97	78.26	83.6
20638	'Snrpb'	138.4	113.2	136	209	186	203.8	152	129.2	131.3	115.68
20639	'Snrpb2'	18.95	18.45	24.7	30.3	31.1	17.41	27.1	32.58	37.66	26.36
20641	'Snrpd1'	145.1	148.6	140	157	113	172.8	140	138	150	122.91
20643	'Snrpe'	216	166.9	167	189	146	239.9	177	139.6	102.6	139.73
20646	'Snrpn'	1409	1302	1144	1605	1764	1798	1351	1781	1407	1473.64
20648	'Snta1'	3.16	3.75	3.93	7.19	7.75	7.44	5.61	2.63	0.79	0.14
20649	'Sntb1'	0	0.56	0	0	0	0	0	0	0	0
20650	'Sntb2'	0.01	0.31	1.63	0	0.08	0.01	2.64	0.12	0	0
20652	'Soat1'	2.95	1.81	0.1	1.19	0.46	0.45	0.43	0.1	0.57	0.43

20655	'Sod1'	1268	1150	1457	1762	1601	1054	1064	969.1	1196	1212.54
20656	'Sod2'	61.16	62.17	62.7	69.9	68.8	73.76	55.4	53.96	47.54	45.62
20657	'Sod3'	2.89	1.88	2.17	0	0	0	0	0	0	0
20658	'Son'	18.27	19.32	19	45.9	24.2	38.07	20.8	9.57	23.88	15.1
20660	'Sorl1'	4	2.07	4.45	6.12	3.29	1.27	3.7	2.7	3.8	0.52
20661	'Sort1'	4.69	0.61	2.41	2.48	8.56	2.53	7.28	2.46	1.25	2.34
20662	'Sos1'	2.18	1.58	2.21	8.61	1.72	3.16	1.62	1.82	2.7	2.62
20663	'Sos2'	1.49	1.73	2.01	7.93	0.56	1.97	1.05	3.46	0.48	2.68
20664	'Sox1'	0.76	0	0.62	0.01	0.79	0.23	0.04	0	0	0
20665	'Sox10'	0	4.66	1.62	0	0.34	0	7.57	3.09	0	0.08
20666	'Sox11'	7.64	6.5	7.53	10.3	4.68	7.78	6.99	15.71	5.2	8.51
20667	'Sox12'	1.28	0.04	0.03	1.4	0.03	0.02	1.15	0.71	0.29	1.43
20668	'Sox13'	0	0.91	1.1	0.06	1	1.8	2.83	0.59	0.7	3.74
20669	'Sox14'	0	0	0	0	0	0	0	3.09	0	0.02
20672	'Sox18'	0.66	0	0	0	0.03	0	0	0	0	0
20674	'Sox2'	2.06	5.56	1.79	7.56	1.67	1.18	2.24	7.73	0.43	3.13
20675	'Sox3'	1.43	0.77	2.05	0	0.93	2.76	0	0.95	1.64	0.5
20677	'Sox4'	1.87	1.28	0.45	7.47	0.94	0.59	2.76	1.6	0.46	0.81
20678	'Sox5'	4.76	0.41	8.48	4.17	0.14	0.77	1.06	2.74	1.37	5.4
20679	'Sox6'	1.11	2.46	2.7	0.58	0.96	0.98	5.65	1.45	0	0.07
20680	'Sox7'	0	0.7	0	2.72	0	0	0	0	0	0
20681	'Sox8'	13.24	5.17	27.4	6.45	2.88	16.6	49.2	13.1	0	3.68
20682	'Sox9'	15.61	12.59	1.72	20.7	3.17	14.48	4.9	3.53	2.15	5.73
20683	'Sp1'	0.76	0.19	0.84	0.52	0.02	0.02	0.01	0.09	0.73	0.78
20684	'Sp100'	0	0.63	0.02	0.03	0	0	0.07	0	0.04	0
20686	'Spa17'	10.89	8.6	2.34	7.57	32.6	13.65	6.3	8.4	11.8	8.09
20687	'Sp3'	5.53	7.27	3.18	0.87	3.21	0.27	5.69	1.18	4.37	1.93
20688	'Sp4'	0.21	0.9	1.42	8.35	2.15	0.54	5.02	1.01	0.86	3.42
20689	'Sall3'	0	0.12	2.09	0.29	0	0.42	0.04	0.13	0.01	0
20692	'Sparc'	258.7	153.6	146	192	154	335.7	211	116	113	132.06
20698	'Sphk1'	0	0.43	1.08	0	0	0	0	0	0	1.04
20700	'Serpina1a'	2.66	0	0	0	0	0	0	0	0	0
20703	'Serpina1d'	4.49	0	0	0	0	0	0	0	0	0
20708	'Serpinb6b'	0	0	0.03	0	0	0	0.06	0	0.02	2.49
20713	'Serpini1'	136.4	91.6	39.6	46.5	69.8	33.14	113	58.3	53.27	81.25
20715	'Serpina3g'	0	0	0.27	0	0	0	0	0	0	0
20716	'Serpina3n'	26.03	5.45	11.7	0	22.3	3.14	4.23	44.3	19.73	4.58
207165	'Bptf'	5.43	5.11	3.6	11	6.18	4.18	6.67	3.02	3.2	8.08
207175	'Cetn4'	16.54	9.87	11.2	29	24.7	9.25	11.2	11.98	10.79	15.96
207181	'Rbms3'	27.41	5.93	9.11	8.96	12.1	5.26	4.59	6.3	10.2	18.36
207182	'Ggt7'	59.14	45.86	115	47.5	64.1	65.12	71.3	102.3	61.49	67.78
20719	'Serpinb6a'	34.13	28.14	33.2	53.5	46.7	44.24	20.7	30.99	42.79	25.96
20720	'Serpine2'	113	141.6	241	139	28.9	139.1	197	204.7	113.1	46.24
207212	'Arhgef17'	3.41	3.35	3.74	13.2	6.03	3.39	5.72	5.33	4.85	5.94
207214	'Larp4'	3.56	3.9	6.78	3.61	3.74	0.79	3.77	2.99	3.84	4.62
207215	'Fbxo40'	0.02	0	0.03	0	0	0.01	0	1.08	0	0.01
207227	'Stxbp5l'	3.35	2.16	2.44	3.44	3.16	1.95	3.8	2.29	4.97	3.57
20723	'Serpinb9'	0	5.61	3.85	7.77	7.42	0	0.04	8.07	0	2.62
207259	'Zbtb7c'	10.8	1.02	1.01	0.91	3.48	1.31	2.22	0.99	0.01	1.43
207278	'Fchsd2'	9.09	13.44	6.85	16.7	7.4	5.52	5.87	8.69	2.21	4.33
20729	'Spin1'	42.12	26.97	36.9	51.1	23.6	17.81	21.5	25.74	35.95	18.65
207304	'Hectd1'	4.25	5.89	4.31	8.24	5.43	3.53	4.3	4.98	4.86	3.45
20732	'Spint1'	6.53	5.22	0.66	6.67	24.5	30.53	5.82	1.04	26.8	4.43
20733	'Spint2'	280.4	443.6	230	680	262	314.2	144	227.1	466	365
207352	'Sec23ip'	10.95	13.88	15.3	12.4	13	3.02	12.2	11.77	5.49	10.4
207375	'Fam120c'	2.89	2.46	2.28	2.32	1.93	0.15	2.51	1.88	6.89	2.98
207393	'Elfn2'	0.47	0.42	3.23	0.57	3.89	2.46	0.71	1.13	1.1	3.6
20740	'Sptan1'	9.3	14.36	23.1	21.8	15.4	7.1	19.3	13.39	10.78	17.67

20741	'Sptb'	1.67	1.3	2.76	0.56	0.08	1.05	2.96	1.51	1.94	0.83
20742	'Sptbn1'	11.44	11.63	8.06	19	22.8	13.91	10.5	13.8	15.21	11.75
207425	'Wdr11'	5.59	7.36	9.48	9.32	11.2	2.42	9.09	7.17	12.8	9.32
20743	'Sptbn2'	3.17	4.76	3.71	3.2	4.18	3.73	5.17	1.49	0.98	7.85
20744	'Strbp'	3.89	5.35	6.62	7.07	6.7	11.87	5.17	3.9	11.43	3.29
20745	'Spock1'	40.72	36.73	63.7	42.7	68.2	32.1	102	110.5	97.73	25.09
20747	'Spop'	61.67	81.83	55	96.3	98.3	55.36	67.2	104	56.79	73.61
207474	'Kctd12b'	0	0	0	0	4.01	2.87	3.67	0.71	0	0
207495	'Baiap2l2'	0	0	1.61	0	0	2.07	0	0	0.22	0.8
20750	'Spp1'	0	0	13.2	0	0	0	0	0.57	5.78	0
20751	'Spr'	59.66	62.16	108	110	100	135.9	141	103.5	98.24	145.85
207521	'Dtx4'	1.67	1.64	1.23	0.02	4.17	3.7	5.08	1.71	1.45	1.01
20755	'Sprr2a1'	0	0.01	0.01	0	0	0	0	0	0	0
207565	'Camkk2'	4.82	8.36	7.84	1.75	10.1	1.87	2.25	9.8	14.17	0.24
207592	'Tbc1d16'	0.9	1.39	0.93	3.17	0.01	0	0.98	0.54	1	0
207596	'Thsd4'	0	0	0.01	0.06	0	0	0	0	0	0.23
207607	'Ccdc40'	1.03	1.92	2.73	5.36	2.5	3.96	1	1.72	4.01	4.48
207615	'Wdr37'	15.73	18.17	20.1	28.5	12	9.59	7.98	17.89	16.28	19.35
207618	'Zfp804b'	0.86	2.21	2.28	3.03	0.61	0	0	1.17	0	1.06
20768	'Sephs2'	23.94	58.63	53.4	21.3	28.2	78.41	62.5	72.69	55.52	32.93
207683	'Igsf11'	0.46	0.06	0	0.59	1.31	0.22	0.09	0.21	0.67	0.46
207686	'Cfap69'	4.44	5	4.79	3.76	2.49	5.83	6.4	5.47	3.35	3.76
207704	'Gtpbp10'	1.77	1.19	1.35	1.04	5.92	2.75	0	1.58	1.68	4.11
207728	'Pde2a'	47.45	36.75	17.4	24	38.4	7.18	33.2	34.59	42.5	32.96
20773	'Sptlc2'	0.98	3.19	6.59	6.39	3.26	7.15	5.53	3.16	1.58	3.4
207740	'Ubald1'	73.54	99.91	70.5	35.2	90	112.4	80	111	82.14	75.51
207742	'Rnf43'	0	0	1.19	0	0	0	0	0	0	0
20775	'Sqle'	48.58	57.32	56.7	33.5	48.7	42.99	52.6	70.04	37.55	41.39
20776	'Tmie'	39.24	35.03	30.7	14.3	10.7	25	16.5	27.12	36.28	19.63
207777	'Tspoap1'	6.31	5.71	21.2	26	18.4	7.79	10.5	8.21	11.04	6.75
20778	'Scarb1'	7.58	19.52	11.4	2.28	12.1	5.48	16.4	7.42	17.76	12.51
207781	'C2cd2'	3.19	1.5	0.79	0	2.04	1.94	0.3	1.91	0.93	1.06
207785	'Csrnp2'	1.39	1.66	1.15	1.43	7.43	1.23	6.54	4.14	2.22	1.1
20779	'Src'	2.6	5.5	6.67	11.4	4.67	2.98	6.87	2.21	0.11	3.59
207792	'BC034090'	0.01	0.67	0.01	0.03	0.81	2.35	2.34	0	0	0
207798	'Gramd1c'	0.74	0.12	0.45	2.34	2.24	1.14	0.28	0.06	0.24	0.03
207806	'Usf3'	0.58	0.06	0.01	1.94	0.22	0	0	0.04	0	0.02
207819	'4930539E08Rik'	1.89	1.71	1.34	0.04	0.07	2.17	0.06	0.03	0.03	1.81
207839	'Galnt6'	0.11	1.37	0	0	0.02	2.04	0	0	0	0
207854	'Fmr1nb'	0	0	0	0	0	0	0	0.36	0	0
20787	'Srebf1'	9.19	13.9	6.97	3.42	4.45	9.86	7.22	2.63	8.87	7.78
20788	'Srebf2'	40.34	26.84	53.8	21.7	21	12.29	43.7	34.56	40.21	48.5
207911	'Mchr1'	26.7	8.64	5.16	20.2	19.7	19.36	5.62	1.96	4.83	10.88
207920	'Esrp1'	0	0	0	0.01	0	0	0	0	0.02	0
207921	'Fam228b'	4.48	3.08	0.24	0.93	0.14	8.89	0.27	1.97	0.02	3.02
207932	'Urb1'	1.18	2.92	0.99	0	0	0.88	0.37	1.56	1.04	0.78
207952	'Klhl25'	0	2.94	4.06	0	3.97	0	0	0	2.28	0
207958	'Alg11'	11.57	18.02	10.7	7.99	24	8.52	12.1	19.02	16.92	15.31
207965	'Vcpkmt'	25.91	1.01	14.2	32.6	4.45	11.4	5.65	22.84	21.28	13.34
208043	'Setd1b'	0.84	1.26	1.33	3.93	1.03	0.76	0.22	0.89	0.44	1.23
20807	'Srf'	5.29	4.63	8.47	16.6	2.55	14.71	5.52	3.43	3.84	9.02
208076	'Pknox2'	8.04	3.37	16.9	2.25	4.21	9.12	12.8	3.37	14.42	14.93
208080	'Ubap1l'	5.01	5.86	7.2	3.58	6.43	7.42	6.16	2.18	6.72	2.46
208084	'Pif1'	0.01	0	0	0	0	0	0	0.01	0	0
208092	'Chmp6'	53.39	47.93	37.3	33.7	83.4	30.16	25.6	54.28	59.38	41.94
20810	'Srm'	52.38	19.82	48.2	62.6	20.8	27.88	45.9	50.82	29.71	10.87
208104	'Mlxip'	1.73	4.31	1.6	4.89	3.2	1.81	1.43	2.34	3.83	2.15
208111	'Zfp976'	3.52	0.34	2.16	0.02	1.69	0.22	0.02	0.31	4.8	0.1

208117	'Aph1b'	7.92	5.82	7.11	7.04	3.75	0.7	9.34	6.84	14.71	12.05
20813	'Srp14'	422.7	410.5	368	489	368	463.2	386	395.7	369.2	482.2
208144	'Dhx37'	1.56	2.71	2.01	5.92	3.81	0.89	0.91	5.34	1.54	5.16
208146	'Yeats2'	11.21	6.19	10.2	4.27	3.04	7.95	10.6	7.95	10.78	9.83
20815	'Srpk1'	19.51	25.74	15.9	8.69	16.8	35.48	41.5	18.53	30.21	36.28
208151	'Tmem132b'	1.3	2.48	1.79	6.33	0.85	1.45	6.69	1.62	2.12	2.12
208154	'Btla'	0	0	0	0	0.32	0	0	1.43	0	0
208158	'Map6d1'	1.93	10.04	10.9	13.6	4.2	2.67	7.73	4.05	3.58	0
208169	'Slc9c1'	0.02	0	0	0	0	0	0	0	0	0
20817	'Srpk2'	57.12	38.84	44	60.9	41.5	50.72	36.8	37.15	44.57	41.06
208171	'Tmprss7'	0	0.11	0	0.12	0.04	0	0.6	0	0	0.03
208177	'Phldb2'	0	0.23	0	0	0	0	0	0.01	0	0
20818	'Srprb'	51.81	41.93	25.8	34.6	31.2	34.14	36.8	32.21	45.96	60.36
208188	'Ghsr'	4.8	4.6	5	0	0.2	0	14.6	16.7	0	0
208194	'Exog'	18.83	11.67	10.5	0.03	10.1	12.45	10.5	17.54	16.39	9.46
208198	'Btbd2'	10.65	4.58	1.72	10.4	23.4	10.19	1.33	8.46	5.75	7.11
208211	'Alg1'	47.18	55.22	34.9	41.8	37.9	66.76	48.1	40.97	39.06	34.75
208213	'Tmem132c'	0.02	0.99	0.78	1.86	0.02	1.73	1.99	1.83	0.01	0.24
20822	'Trove2'	10.52	6	4.39	11.7	5.57	4.73	6.23	10.69	6.54	4.05
208228	'Mob3a'	15.17	17.33	19.2	17.6	12.1	15.32	16.7	19.29	10.32	30.11
20823	'Ssb'	107.8	86.57	84.3	120	81.2	103.4	108	84.67	85.52	70.19
208258	'Ankrd33'	0	0	0	0	0	0.54	0	0.67	0	0
20826	'Snu13'	342.8	334.4	286	248	262	287.1	268	284.1	290	327.05
208263	'Tor1aip1'	2.91	7.55	4.03	4.61	6.96	0.97	5.63	5.7	0.85	2.83
208266	'Dot1l'	2.7	1.03	2.29	6.72	1.65	3.15	1.25	0.37	2.46	1.56
208285	'Cyp4f17'	7.52	0.84	5.58	2.74	3.92	4.11	6.14	8.52	5.12	8.01
208292	'Zfp871'	6.24	4.06	5.83	1	5.8	3.46	6.82	2.45	6.71	5.26
20832	'Ssr4'	193.3	318.7	184	281	130	428.8	161	191.7	241.7	429.06
20833	'Ssrp1'	24.24	27.59	40	52.6	36	20.19	55.4	41.09	39.78	33.65
20834	'Znrf4'	0	0	0	0.05	0	0	0	0	0	0
208366	'Rpp40'	0.05	8.52	3.78	3.68	8.24	0.23	0	9.02	1.8	3.71
208372	'Asb18'	0.99	0	0	0	0	0	0	0	0	0
20840	'Stac'	0.02	2.71	7.41	0	1.98	0	0	3.85	10.37	0
20841	'Zfp143'	3.83	1.95	3.38	4.79	4.19	1.38	6.79	2.36	5.01	0.83
20842	'Stag1'	1.4	2.17	3.53	1.8	0.99	0.72	2.3	3.6	3.91	4.43
208426	'Iqcj'	0	0	0	0	0.67	1	5.25	0	0	0
20843	'Stag2'	1.33	1.01	1.6	4.32	0.46	2.37	3.57	1.58	1.33	9.94
208431	'Shroom4'	0	0	0.2	0	0	0	0	0	0	0
208439	'Klhl29'	1.76	1.07	0.28	0.19	2.91	2.36	1.83	2.73	3.67	1.34
20844	'Stam'	23.89	32.04	32.5	13.5	22	9.23	26.2	32.15	33.81	23.72
208440	'Dip2c'	2.1	0.37	1.08	8.5	2.37	1.34	0.61	1.54	1.36	2.03
208449	'Sgms1'	4.26	6.14	4.92	3.76	1.73	4.64	5.44	3.99	7.85	5.77
20845	'Star'	1.55	2.09	0.44	1.04	1.42	0.09	2.39	0.5	0.36	0
20846	'Stat1'	15.34	5.55	10.7	45.3	10.8	12	9.58	7.27	12.82	15.62
20847	'Stat2'	4.92	1.21	3.98	11.7	1.59	4.33	2.68	2.11	8.5	2.47
20848	'Stat3'	23.18	19.13	20.6	31.3	26.7	17.51	27.3	15.29	41	27.95
20849	'Stat4'	0	0	0	0	0	0	0	0.02	0.02	0
20850	'Stat5a'	2.2	4.78	0.69	6.28	0.03	1.09	0	0	6.13	1.51
208501	'Ndufaf8'	76.83	30.6	29.4	62.1	87	138	70	47.63	71.78	50.86
20851	'Stat5b'	6.29	7.11	9.64	1.85	13.7	9.25	18.2	11.17	9.25	8.13
208518	'Cep78'	1.15	3.81	2.96	6.72	0.48	1.44	5.1	1.87	4.29	2.31
20852	'Stat6'	0	0	0.83	0.01	5.3	4.41	0	0	15.98	4.56
20853	'Stau1'	8.72	18.36	20	8.78	25.7	6.65	18	15.94	22.03	15.78
20855	'Stc1'	4.96	12.48	14.2	10.4	5.74	4.68	5.32	13.17	1.66	5.73
20856	'Stc2'	0	0	0.03	11.6	0	0	0	1.1	0	0
208583	'Nek11'	0	1.45	3.36	0	5.28	0	0	0	0	0.37
208595	'Mterf1b'	0	0	0	0	0	11.24	0	0	2.52	0
208606	'Rsrc2'	50.47	48.25	40.2	61.8	50	42.71	45.1	42.59	53.79	40.47

20861	'Stfa1'	0	0	0	7.58	0	0	0	2.88	0	0
208613	'Tmem212'	0	0	0	0	0	0	0	0	0.64	3.63
208618	'Etl4'	0.35	1.33	6.63	0.34	1.02	1.43	0.12	0.68	5.42	0.92
208624	'Alg3'	17.97	17.89	5.78	8.62	31	57.93	11	14.64	3.83	10.01
208628	'Kntc1'	1.56	0.02	0	0.76	0	0	0	0	0	0
208638	'Slc25a38'	34.6	16.63	46.6	79.2	24.5	23.55	17	19.3	23.73	19.01
208643	'Eif4g1'	11.72	11.54	16.6	35.3	10.9	16.37	15.2	10.62	17.38	14.14
208647	'Creb3l2'	1.41	1.93	0.24	7.58	3.6	0.01	0.04	0.9	0.03	2.59
208650	'Cblb'	0.95	1.07	2.47	1.29	2.29	0.22	2.63	2.32	1.42	1.16
208659	'Fam20a'	5.75	2.98	16	0.8	4.27	11.3	5.3	7.31	8.79	0
20866	'Stim1'	26.6	23.66	26.6	8.79	13.8	13.41	8.28	12.99	12.51	11.44
208665	'Akr1d1'	0	0	0	0	0.02	0	0	0	0	0.02
208666	'Diras1'	15.24	11.42	24.8	1.66	19.2	18.55	19	28.78	18	12.93
20867	'Stip1'	279	238.9	180	149	160	237	294	215.3	270.6	331.96
208677	'Creb3l3'	0	1.95	5.23	0	0	0	0	0	0	0
20868	'Stk10'	0	0.44	0	2.36	0	0	0	0	0	0
20869	'Stk11'	9.17	9.7	5.01	6.74	11.4	0.4	3.07	1.94	12.72	4.89
208691	'Eif5a2'	2.01	5.32	12.9	0	3.25	4.54	3.42	2.91	9.24	2.52
20871	'Aurkc'	0	0	0	0	0	0	1.78	2.25	0	0
208715	'Hmgcs1'	68.85	72.83	73.2	30.4	75.4	34.34	71	106.7	59	75.77
208718	'Dis3l2'	3.1	17.51	15.1	12.7	0.97	8.07	0.47	13.45	9.36	13.8
20872	'Stk16'	54.09	46.96	55.2	45.5	69.1	80.41	21.9	52.99	33.11	65.98
208727	'Hdac4'	8.92	11.84	7.66	8.6	14.4	7.09	7.13	7.84	6.45	11.04
20873	'Plk4'	0	0	0.19	0	0.25	0	0.7	2.64	4.01	0
20874	'Slk'	1	1.97	0.03	1.02	1.2	1.14	0.02	1.02	0.5	0.36
208748	'Prrg3'	2.45	2.54	1.75	2.01	3.69	3.27	4.11	2	1.62	1.13
208760	'Aqp12'	0	0	3.28	0	0	0	0	0	0	0
208768	'Sde2'	10.52	12.43	13.3	13.1	18.6	10.09	15	15.26	19.02	12.01
208777	'Sned1'	1.64	0.76	1.88	0	1.01	3.05	0.56	0	0.24	1.12
20878	'Aurka'	0	1.52	0	0	2.77	0	0.21	0	0	0
208795	'Tmem63a'	2.3	6	0	0	2.66	6.93	0.03	2.36	0	0
208820	'Triqk'	17.17	14.76	15.5	11.3	19.7	23.49	12.4	9.92	17.32	14.93
208836	'Fanci'	0	0.07	0.44	0.11	0.02	0.43	0	0	0	0
208846	'Daam1'	8.9	8.82	4.75	5.81	4.33	11.79	3.46	5.35	4.55	5.32
208869	'Dock3'	1.55	2.94	2.17	5	6.15	4.13	2.76	3.04	3	1.8
20887	'Sult1a1'	3.35	0.88	3.5	8.28	0	0	3.1	0	0	0
208884	'Zdhhc9'	1.35	1.26	1.32	0.19	9.17	0.12	0.81	6.58	0.03	6.36
208898	'Unc13c'	1.23	4.56	1.83	0.94	1.14	2.23	9.11	0.7	2.74	0.34
208908	'Ccdc62'	0.49	1.38	0	1.74	2.16	8.12	6.73	0.77	2.59	2.38
20892	'Cenpx'	70.71	24.22	63.4	47.2	22.3	52.55	98.3	40.01	51.66	61.5
208922	'Cpeb3'	2.21	6.21	13.5	3.38	2.98	1.09	5.3	1.64	6.37	4.29
20893	'Bhlhe40'	11.85	27.12	46.5	95.3	20.1	18.33	8.63	6.86	8.6	9.51
208936	'Adamts18'	1.29	0.26	0.27	0	0.69	0	0.74	1.61	0	0
208943	'Myo5c'	0	0	0.14	0.02	0	0.83	0	0.01	0.33	0.03
208967	'Thnsl1'	15.26	15.33	2.94	22.6	15.3	8.18	6.06	17.95	13.83	17.17
208968	'Zfp280c'	2.87	1.39	2.29	0	3.7	0.43	1.47	1.6	0.93	1.37
20897	'Stra6'	0.05	0	0	0	0	0	0.16	0	0	0
208982	'Hmgcll1'	15.16	8.96	9.88	6.92	8.33	2.48	17.2	8.83	4.87	4.11
208990	'Npb'	2.2	2.82	0	0	5.42	10.76	1.72	0.66	0	0
208994	'Fam83b'	0.35	0	0	0	0	0	0	0	0	0
209003	'Rbmx2'	8.15	4.16	8.19	19.4	10.8	8.36	7.28	7.02	9.86	5
20901	'Strap'	108.9	101.6	69.8	90.5	61.5	89.79	111	98.32	81.01	83.99
209011	'Sirt7'	13.88	24.93	18.5	45.7	41.4	70.19	32.6	32.23	32.73	23.19
209012	'Ulk4'	5.26	0.6	2.41	2.75	7.26	0.28	3	0.73	2.67	11.2
209018	'Vps8'	3.05	2.21	13.6	0.91	4.98	3.83	6.16	8.39	9.59	20.15
209027	'Pycr1'	0	3.37	6.04	0	7.53	0	0	2.36	1.17	0
209032	'Zc3hav1l'	1.09	0.6	0.05	0.03	0.02	0.27	0	0.04	1.09	0
209039	'Tns2'	0	0.13	3.73	0	0	0	0.01	0.01	0.68	0

20907	'Stx1a'	35.26	59.64	23.8	32.4	37.6	34.94	49.4	51.23	36.93	20.79
20908	'Stx3'	13.51	16.86	17.8	8.87	16.6	3.64	9.54	4.38	11.01	12.74
209086	'Samd9l'	0	0.19	1.34	0.03	0	0	0	0	0.01	0
20909	'Stx4a'	11.25	14.78	23.1	22	19.3	29.24	30.9	21.46	39.93	26.92
20910	'Stxbp1'	198.3	200.9	314	196	134	104	190	204	213.8	138.74
20911	'Stxbp2'	14.28	43	26	47.6	38.7	75.39	6.12	21.99	77.9	77.68
20912	'Stxbp3'	13.09	9.53	2.43	5.05	3.79	5.57	5.14	0.05	6.46	5.61
20913	'Stxbp4'	12.95	4.79	3.53	0.82	1.12	14.01	4.45	10.15	7.52	18.21
209131	'Snx30'	3.41	1.71	2.42	1.59	2.52	2.95	1.06	1.53	6.46	2.08
20916	'Sucla2'	171.4	150.6	160	149	146	199.5	125	139.5	129	173.84
20917	'Suclg2'	0.05	1.27	0.03	2.39	4.33	0.01	0	0	3.64	2.01
209176	'Ido2'	0.05	0.64	0.65	3.84	0	0.56	1.63	0.6	0	1.16
20918	'Eif1'	327.4	381.2	480	533	433	201.8	368	386.6	404.9	502.94
209186	'Acnat2'	0.07	0	0	0	0	0	0	0	0.28	0
209195	'Clic6'	2.67	1.99	3.52	0	1.04	4.36	0	0.32	5.78	1.37
209200	'Dtx3l'	0.99	1.34	1.37	0.92	0.38	0.17	0.17	0.03	0.42	3.78
209212	'Osgin2'	5.28	0.17	4.56	16.5	2.34	4.12	2.88	3.42	0	0.49
20922	'Supt4a'	142.6	124	143	104	176	233	123	173.6	148.3	121.13
209224	'Enox2'	3.87	10.15	4.11	9.23	1.59	2.38	0	10.34	1.71	1.66
209225	'Zfp710'	2.12	0.73	0.81	0.06	1.46	4.13	0.03	0.08	1.09	0.2
209239	'Gan'	0	0.25	0	0	1.22	0	0	0.02	0	4.69
20924	'Supt5'	33.77	58.29	35.7	42.6	63.9	13.07	49.7	36.17	49.33	44.69
20926	'Supt6'	16.42	11	22.7	16.3	13.9	7.49	19.8	9.1	13.51	12.2
209268	'Igsf1'	75.7	60.44	24.5	17.1	5.22	13.11	3.54	29.06	13.96	24.35
20927	'Abcc8'	3.1	3.79	5.96	0.01	3.6	0.64	9.29	5.74	3.77	0.01
20928	'Abcc9'	0.07	0.15	0.15	0.18	0.21	0.2	0.1	0.09	0.18	0.11
209294	'Csta1'	0	0	0	1.78	0	0	0	0	0	0
20930	'Surf1'	94.52	77.36	117	122	70.8	146.4	102	92.18	107.6	165.95
20931	'Surf2'	53.7	26.46	36.7	30.1	49.9	57.42	14.5	21.25	45.14	30.79
209318	'Gps1'	177.2	118.4	178	44.7	137	151.3	153	176.3	128.1	164.12
20932	'Surf4'	74.08	64.69	52.2	47.5	34.1	56.96	50.8	43.44	52.01	56.08
20933	'Med22'	30.69	45.81	36.8	73.3	70.2	42.89	51.8	36.42	41.59	27.7
209334	'Gen1'	0.6	0.02	0.07	0.04	1.72	0.05	2.29	0.85	0.04	0.07
20935	'Surf6'	10.23	6.79	8.21	21	9.21	0.65	13.9	9.99	15.57	6.46
209354	'Eif2b1'	35.65	46.09	21.7	58.4	20.2	42.58	30.4	50.28	37.43	27.66
209357	'Gtf2h3'	7.5	7.95	8.27	0.02	13.7	6.15	8.34	8.2	2.94	3
209361	'Taf3'	9.28	6.83	3.75	8.15	6.92	5.08	2.8	10.23	3.67	9.08
20937	'Suv39h1'	21.76	41.95	25.5	7.03	21.7	13.12	34.7	26.99	11.43	24.63
209378	'Itih5'	0.01	0.73	0.43	0.6	0.04	2.16	0	0.7	0	0
209387	'Trim30d'	0	0	0.01	0.21	0	0	0	0	0	0
209416	'Gpkow'	15.87	10.87	30.1	9.21	20.2	7.52	24.1	20.99	24.17	16.26
209446	'Tfe3'	18.16	11.77	13.2	10.7	29.1	9.9	16.7	20.26	3.32	11.84
209456	'Trp53bp2'	5.6	2.3	4.41	3.45	1.54	1.65	0.12	1.18	4.27	1.64
209462	'Hace1'	4.15	5.58	1.39	14	6.23	3.75	0.06	2.99	7.26	2.21
20947	'Swap70'	0.9	0	0	0	0	0.38	3.92	0	3.45	0
209478	'Tbc1d12'	0	2.12	0.96	1.06	0.77	2.59	0.55	1.08	7.01	0.83
209488	'Hsh2d'	0	0.12	0	0	0	0	0	0	0	0
209497	'Tmem164'	1.4	0.03	0.36	0.45	4.68	5.42	0.01	0.03	6.82	6.18
209540	'Rtl9'	0.66	0.06	1.05	5.56	0.32	0.92	0.01	0.03	4.3	0.06
20955	'Vamp7'	17.65	32.26	24	21	19.9	10.86	28.2	20.42	30.55	33.56
209550	'Rad51ap2'	0	0.67	0.03	0	0.87	1.05	0	0	0.99	1.14
209558	'Enpp3'	0.11	0	0	0	0	0	0	0.42	0	0
209584	'Tyw3'	4.02	1.91	3.03	2.68	1.84	4.76	3.22	3.57	2.04	3.54
209586	'Nudcd3'	21.12	40.31	44.4	45	37.4	40.95	57.1	36.13	52.42	19.18
209601	'Erich3'	3.36	4	3.09	5.82	3.44	1.09	2.67	0.92	2.67	0.19
20962	'Sycp3'	11.95	0	0	0	0.09	5.92	0	0	0	3.21
20963	'Syk'	0	0.2	0.01	0	0	1.92	0.02	0	0	0
209630	'Frmd4a'	4.89	2.96	4.23	7.71	5.47	6	4.93	4.25	8.52	5.88

20964	'Syn1'	139.5	146.9	186	112	112	63.16	273	136.6	140.7	52.79
209645	'Bend7'	4.82	0	0	1.35	0.01	0	0	0.77	1.87	0.47
20965	'Syn2'	32.53	24.73	26.6	54.1	41.2	27.2	42.6	44.34	29.15	23.62
209683	'Ttc28'	0	0.61	0.55	0.68	1.3	0.44	2.37	0.13	1.25	0.23
20969	'Sdc1'	1.64	2.35	2.12	0	0	6.63	2	1.49	0	2.48
209692	'Dhtkd1'	0.85	1.71	3.63	2.19	5.26	5.14	1.93	3.03	3.11	1.59
20970	'Sdc3'	1.58	10.51	13.4	11.3	15.3	3.02	8.77	0.31	2.03	1.78
209707	'Lcorl'	3.54	7.87	5.36	4.72	4.55	1.44	1.58	2	2.23	2.34
20971	'Sdc4'	29.69	8.92	13.6	42	23.7	25.48	10.6	24.54	10.42	4.24
20972	'Syngr1'	69.3	40.48	83.8	38.1	60.2	76.84	44.1	57.16	97.9	65.13
20973	'Syngr2'	11.66	14.51	1.5	7.24	21	43.58	7.68	3.52	0.31	3.43
209737	'Kif15'	0.26	0.04	0.03	0	0.01	0	0.23	0.09	1.87	0
20974	'Syngr3'	471.4	387.5	387	342	381	613	591	517.1	455.1	555.9
209743	'AF529169'	3.11	0	0.01	0	5.7	1.59	0	0	0.22	0
20975	'Synj2'	1.89	1.71	0.93	0.79	3.58	2.88	5.71	2.25	15.83	4.72
209760	'Tmc7'	0	1.64	3.02	4.86	0.16	0.07	0.13	1.4	5.52	0
20977	'Syp'	393.8	330	467	289	353	202.1	375	365.2	388.7	381.41
209773	'Dennd2a'	1.79	0.39	0.05	1.42	0.12	0.01	1.76	0.35	0.08	0.47
209776	'Gpr139'	3.39	0	1.25	0	0	0	0	4.12	0	0
20979	'Syt1'	56.26	81.22	167	78.3	160	53.4	199	104.8	103.9	82.8
20980	'Syt2'	0	4.65	5.1	0	0.84	0.01	0	0.12	5.59	2.46
20981	'Syt3'	38.62	37.59	56.1	19.8	26.4	26.33	39.6	66.99	36.79	14.28
209815	'Tbc1d25'	5.73	11.6	15.3	9.08	15.6	1.76	1.55	6.86	4.55	8.5
20983	'Syt4'	173	114.1	100	106	91.3	87.6	92.8	96.63	119.8	116.63
209966	'Pgbd5'	3.9	9.27	1.88	9.16	3.06	0.42	8.76	1.91	0.02	4.25
210004	'B3gntl1'	7.73	1.86	2.37	0	5.22	0	0	4.9	7.79	11.01
210009	'Mtrr'	4.48	5.33	6.54	6.29	5.41	1.94	2.26	5.94	0	13.07
210027	'Slc35f3'	3.12	1.89	2.79	14	4.91	5.25	2.54	5.52	3.66	2.5
210029	'Metrnl'	0.02	3.49	1.55	4.27	1.51	5.13	0.02	0.97	2.23	0
210035	'Nemp1'	0	0	3.17	0	1.11	0	0.1	0	0	0
210044	'Adcy2'	10.98	3.75	0.02	1.81	11.3	13.04	0.23	9.29	17.77	2.84
210094	'Iglon5'	0	0.5	0	0	0	0	0	0.86	1.6	0.18
210104	'Zfp658'	5.65	0	0.08	6.6	3.1	0	1.08	5.67	1.21	2.33
210105	'Zfp719'	1.39	8.58	6.6	2.52	6.88	0.13	0	3.42	0.6	2.86
210106	'Tent4a'	2.6	3.6	1.24	1.82	0.55	0	2.01	2.69	0.01	0.22
210108	'D130043K22Rik'	15.11	14.07	22.4	4.42	17.1	7.19	11.4	9.38	12.97	13.95
210126	'Lpp'	2.02	1.62	0.24	1.86	0.03	0.62	3.77	0.88	7.33	3.06
210135	'Zfp180'	11.51	16.88	6.67	9.97	3.4	1.65	4.22	16.3	6.14	10.11
210146	'Irgq'	5.38	2.8	3.7	19.3	2.47	0.79	5.75	3.23	4.64	2.85
210148	'Slc30a6'	24.46	13.29	10.3	21.6	12.8	7.4	13.1	10.16	5.47	9.38
210162	'Zkscan2'	1.54	0.13	3.39	0	0.65	0.32	0.91	0.95	4.59	1.56
210172	'Zfp526'	0.03	0	1.51	0	0.04	0.29	2.16	0.38	0	0.35
210274	'Shank2'	3.55	4.11	2.73	3.85	0.74	3.78	1.54	1.74	1.18	1.58
210293	'Dock10'	1.05	1.18	0.64	0.01	1.26	1.82	0.37	0	1.21	1.1
210297	'Lrch2'	2.15	2.32	0.18	1.42	1.32	1.59	1.64	0.78	2.25	1.95
210356	'Nckap5'	1.57	1.75	3.59	3.12	0.03	0.25	3.16	1.2	1.46	0.52
210376	'Mtmr9'	6.06	3.44	10	0.02	4.54	6.23	15.2	3.4	0.62	14.53
210417	'Thsd7b'	1.1	0.76	0.06	3.07	5.7	0.13	4.96	1.62	1.86	7.89
210503	'Zfp677'	0.39	1.44	0.03	0	0.74	3.06	9.97	1.5	0	5.29
210510	'Tdrd6'	0	0	0	0	0	0	0	0	0	0.08
210529	'Mettl14'	13.06	8.44	9.72	10.8	13.1	4.31	13.6	7.2	12.99	8.4
210530	'P3h2'	0.02	0.06	0	0	0	0	0	0	0	0
210544	'Tbc1d31'	1.3	3.21	4.72	1.41	1.01	1.49	0.43	1.85	5.27	0.91
210573	'Tmem151b'	13.39	15.16	19.2	8.1	15.6	19.07	14.1	12.52	4.47	9.14
210582	'Coq10a'	5.52	21.97	8.83	16.1	23.8	21.4	9.27	21.44	8.28	18.37
210583	'Gm4767'	0	2.19	2.17	0	4.73	13.37	0	4.02	0.02	8.47
210622	'Pamr1'	0.46	0	9.19	0	4.02	0	0	0.63	0.21	0
210673	'Prrt3'	9.17	3.58	20.3	2.65	9.15	5.67	26.5	7.62	16.11	3.59

210710	'Gab3'	3.6	1.74	7.43	0	2.38	3.79	3.9	0.81	1.63	0.38
210711	'Mcmbp'	11.07	11.39	9.35	13	18.2	17.71	9.94	8.27	14.35	11.33
210719	'Mkx'	3.1	2.84	1.43	0	0.29	0.49	0	1.72	0	0
210741	'Kcnk12'	0.14	0.89	0.47	0	0.63	0	2.26	0	0	0
210762	'Ppp1r36'	3.36	0	0	0	0	0	0	2.1	0	0
210766	'Brcc3'	84.41	77.07	59.1	72.8	51.3	101	43.3	87.8	67.11	102.87
210789	'Tbc1d4'	0.02	7.76	4.68	5.59	3.14	2.41	6.58	0.98	0.04	0.5
210801	'Unc5d'	7.29	3.46	3.6	3.58	2.69	1.77	5.62	2.91	1.15	3.22
210808	'Lacc1'	0	0	0	2.32	0	0.08	0	0	0	0
210853	'Zfp947'	3.47	1.22	2.15	2.87	1.32	2.63	2.08	5.47	1.16	1.54
210925	'Ints9'	7.77	7.58	6.53	8.58	4.59	2.66	12.8	5.41	13.22	23.88
210933	'Adgrb3'	26.38	16.33	14.4	10.4	12.3	7.04	20.7	13.11	11.17	13.04
210973	'Kbtbd2'	22.28	10.06	13.9	17	18.6	4.35	20.9	12.56	12.15	15.42
210982	'Bicral'	1.5	2.6	2.94	0.82	1.4	0.46	6.63	4.77	7.56	3.67
210992	'Lpcat1'	15.97	7.35	13.7	16.1	9.75	13.58	12.6	17.12	25.47	6.27
210998	'Fam91a1'	2.54	1.3	3.74	3.55	3.61	3.55	2.72	3.67	5.22	4.7
211006	'Sepsecs'	4.38	4.79	1.98	0.43	2.65	8.46	3.79	1.86	2.78	1.87
211007	'Trim41'	0.33	3.26	1.47	1.63	9.04	0.29	2.91	0.77	8.66	5.32
211064	'Alkbh1'	8.77	13.17	24.7	15.6	6.7	5.19	17.9	7.71	8.56	19.86
211134	'Lzts1'	18.86	15.08	4.23	7.63	27.9	9.32	5.12	30.91	10.6	10.12
211135	'D130040H23Rik'	2.96	1.75	2.01	0.1	0.59	4.03	5.1	1.46	0.45	1.85
211147	'March11'	2.31	2.17	0	6.11	0	0	0	1.38	0	0.9
211151	'Churc1'	340.2	161.6	306	297	206	291	298	231.5	230.2	228.89
211187	'Lrtm2'	14.21	11.04	20.3	0	15.6	6.02	17.3	14.96	8.35	14.56
211223	'Vmn2r15'	0	0	0	0	0	0	0	0.09	0	0
211228	'Lrrc25'	0	0	0	0	0	0.55	0	3.61	0	0
211232	'Cpne9'	17.15	0.91	14.2	6.05	0.02	7.78	0	1.77	1.91	0.05
211253	'Mtrf1'	5.57	9.53	13.7	6.28	5.32	17.84	4.47	17.97	13.57	5.31
211255	'Kbtbd7'	7.83	3.34	1.09	14.2	2.72	1.8	6.56	6.07	5.39	1.78
211286	'Cln5'	7.95	4.65	5.09	22.8	8.96	0.63	1.12	3.08	10.01	0.81
211323	'Nrg1'	0.86	4.35	1.45	5.71	4.76	0	4.12	2.88	3.59	8.5
211329	'Ncoa7'	5.78	10.01	10.1	5.36	11.4	6.79	9.21	8.93	12.33	21.61
211347	'Pank3'	1.17	0.21	3	0.33	2.33	0.91	0.28	1.1	1.58	0.16
211378	'6720489N17Rik'	1.75	1.18	3.06	0.65	1.96	0.97	0.02	0.16	0.35	1.31
211383	'Amer3'	6.62	3.19	4.18	0	3.49	6.51	2.25	7.36	14.1	3.59
211389	'Suox'	13.64	4	3.96	0	7.21	4.05	0.02	7.33	7.6	3.16
211401	'Mtss1'	9.36	11.77	8.72	18.9	3.76	8.51	4.28	8.37	7.19	8.27
211429	'Pla2g4b'	0.05	1.85	1.66	0.47	2.46	1.88	0.1	0.35	0.4	2.29
211446	'Exoc3'	33.34	22.8	36.6	36.8	24.7	23.95	28.5	19.97	43.37	31.98
211468	'Kcnh8'	3.25	0.78	0.59	0.01	0.66	1.69	0	1.96	1.44	1.6
211480	'Kcnj14'	5.44	7.31	3.95	1.54	3.88	0	5.81	4.87	1.4	15.45
211482	'Efhb'	3.91	2.24	0	4.55	4.06	0	3.78	0	2.53	2.46
211484	'Tsga10'	0.59	2.3	3.05	0.92	2.64	1.2	4.34	0.51	3.09	5.2
211488	'Ado'	14.57	11.43	9	8.59	11.9	4.31	4.67	4.3	4.1	1.74
211499	'Tmem87a'	6.29	1.88	2.34	5.86	7.13	9.59	4.51	4.96	12.56	2.17
211535	'Ccdc114'	0	0.17	0	0	0.02	0	2.03	0	0	0.29
211548	'Nomo1'	15.84	17.02	19	5.56	5.99	6.93	23.7	13.76	14.43	21.25
211550	'Tifa'	0	0	0.02	4.1	2.81	0.06	0	0.11	0	0
211556	'Ap1ar'	1.77	4.1	5.79	12.4	8.6	0.77	4.41	2.05	2.82	2.55
211577	'Mrgprf'	0	0	0	0	0.23	0	0	0	0	0
211586	'Tfdp2'	4.42	0.99	2.69	2.58	2.63	2.17	3.97	0.42	0.7	2.86
211612	'Ptchd1'	10.25	3.68	11.6	7.31	17.2	18.03	5.05	14.23	3.82	10.43
211623	'Plac9a'	5.03	0	19.8	0	0	0	0	8.1	0	0
211651	'Fancd2'	0	0.26	4.73	0	0.01	0.51	0	0.86	0	0
211652	'Wwc1'	1.94	1.66	0.36	4.55	2.03	0	0.01	1.12	0.14	1.1
211660	'Cspp1'	11.22	6.63	5.54	5.02	6.09	18	9.22	3.5	6.22	11.04
211666	'Mgst2'	0	0.85	2.36	0	0	0	0	0	0	0
211673	'Arfgef1'	3.96	1.83	1.8	2.58	5.63	0.73	4.78	1.35	0.06	3.76

211712	'Pcdh9'	96.97	87.64	50.2	16.6	42.1	68.53	88.4	48.15	90	84.87
211739	'Vstm2a'	100.3	67.71	32.1	18.3	53.7	33.8	101	68.1	70.53	65.01
211770	'Trib1'	0	0.49	4.64	14.1	0.02	4.57	10.8	0.45	0.1	0.59
211798	'Mfsd9'	0	1.76	5.03	0	0.91	2.22	1.74	1.71	1.73	0
211896	'Depdc7'	0	2.72	0.48	0	0.03	0	0	0	4.61	0
211914	'Asap2'	1.42	0.76	1.51	1.68	1.49	1.86	0.5	0.27	2.86	2.84
211922	'Dennd6a'	2.05	5.93	1.4	4.3	2.33	1.24	1.14	1.57	12.49	3.31
211936	'Ccdc73'	0.27	1.58	1.27	0	1.7	0.51	1.53	0.32	1.18	2.43
211945	'Plekhh1'	0.49	0.02	1.04	0.68	0.52	1.48	1.01	0	0.1	0
211948	'Pde12'	16.3	15.18	11.9	27.3	5.97	8.72	9.3	4.2	13.72	3.83
211949	'Spsb4'	2.91	4.3	5.14	0	0.26	4.65	1.99	3.87	2.42	2.27
211961	'Asxl3'	1.2	0.15	0.47	0.02	0.19	0.02	0	0.56	0	0
211978	'Zfyve26'	1.8	0	0.9	1.64	0	1.12	1.03	0.85	3.04	1.97
211986	'Tmem18'	18.59	11.15	13.9	5.8	3.1	14.41	18.7	16.02	37.17	7.77
212032	'Hk3'	1.21	0.26	0.05	0.02	2.48	0.06	0	1.31	0	3.26
212090	'Tmem60'	81.52	57.73	65.1	43.5	92.4	136.6	65.3	60.93	113	112.38
212111	'Inpp5a'	4.84	0.11	3.1	11.3	2.89	4.64	4.86	2.07	2.68	0.41
212114	'Nhlrc3'	0.09	0.47	0	0	0.89	0	0	0	2.67	2.29
212123	'Dcaf15'	8.4	4.59	2.57	12.7	4.74	10.12	6.46	0.41	3.61	2.68
212124	'Cfap46'	1.04	3.52	4.43	1.95	2.68	6.06	4.57	2.86	1.18	4.39
212127	'Proser1'	0.99	0.37	2.41	1.97	3.48	0.01	2.61	0.5	0.64	1.02
212139	'Cc2d1a'	18.83	14.98	23.4	1.51	10.4	14.06	11.9	15.84	23.99	19.26
212153	'Ccdc191'	1.76	2.06	1.25	4.66	2.18	0	3.03	0.28	3.79	2.57
212163	'8030462N17Rik'	10.68	7.46	11.7	8.15	9.44	6.07	8.37	6.37	13.06	9.6
212167	'Gsap'	2.95	0.07	8.01	12	3.72	0.95	2.86	1.7	3.77	2.65
212168	'Zswim4'	3.12	1.7	2.61	2.64	0.33	0.72	0	1.41	0	2.68
212190	'Ubxn10'	3.9	3.1	2.89	0	8.67	0	0	3.32	3.08	4.94
212198	'Wdr25'	2.52	2.78	5.03	0	0.01	3.99	0.25	12.46	3.78	0.08
212276	'Zfp748'	2.49	1.95	1.2	0.36	1.95	0.02	2.68	3.82	6.05	2.73
212281	'Zfp729a'	0.06	0.14	0.06	3.64	0.12	2.14	0.01	0	1.1	0.04
212285	'Arap2'	1.07	0.77	0.03	0	1.6	2.78	1.83	0	0.92	0.01
212307	'Mapre2'	81.51	91.45	79.7	74.3	67.3	47.3	67.6	77.86	92.1	105.38
212326	'Fam149a'	9.75	6.54	6.1	21.7	6.83	6.44	4.44	9.53	3.94	7.06
212377	'Mms22l'	0	0	0	0.25	1.09	0	0.98	0.03	0	0
212390	'Klhl32'	9.85	11.57	22.3	0.12	24.1	17.16	1.76	25.01	13.67	17.57
212391	'Lcor'	1.85	2.07	5.59	7.2	2.81	3.85	1.89	1.62	0.34	2.54
212392	'Ccdc110'	0	0	0	0	0	0	0	0	0	0.43
212398	'Frat2'	0.02	0	1.13	1.76	0	0	1.78	3.56	3.59	0
212439	'AA986860'	1.16	0	0.07	1.22	0.12	0.79	0	0	0.02	0
212442	'Lactb2'	0.02	0.87	3.22	3.61	8.05	2.53	4.16	5.42	4.24	6.53
212448	'9330159F19Rik'	13.2	14.53	10.9	14.2	7.2	10.3	14.9	9.69	6.87	10.85
212483	'Fam193b'	1.14	3.65	4.64	19.1	14.5	13.04	8.56	7.31	4.67	2.99
212503	'Paox'	1.79	4.85	2.52	12.7	6.72	0.04	11.4	0	0.02	0
212508	'Mtg1'	18.35	47.99	45.8	9.3	49	44.67	52.9	31.33	29.49	45.31
212514	'Spice1'	7.25	6.88	5.71	0	5.6	1.94	4.94	2.1	2.8	12.94
212516	'Efcab12'	4.02	1.37	4.49	0.48	9.21	9.85	13.5	2.12	2.58	14.28
212517	'Cfap44'	2.76	2.86	1.22	2.5	3.68	1.23	0.01	0.22	1.2	5.19
212518	'Sprn'	10.76	12.83	19.4	7.46	18.9	5.99	12.4	9.53	7.5	8.51
212528	'Trmt1'	33	43.38	29.9	20.1	46.4	74.77	25.5	18.95	20.39	34.75
212531	'Sh3bgrl2'	4.79	1.73	1.28	0	0.03	0.17	3.22	1.05	4.54	2.42
212539	'Gm266'	2.27	0	1.99	0	0	0	6.11	0	0	0
212541	'Rho'	0	1.37	0	0	0	0	0	0	0	0
212547	'Nepro'	0.72	0.02	0.58	0	1.64	0.34	1.66	0.72	0.44	0.52
212555	'Pqlc2'	8.27	6.2	8.57	2.88	16	0	8.2	4.98	15.13	1.62
212569	'Zfp273'	4.77	0	0	6.8	2.03	1.49	0	2.36	0	5.3
212627	'Prpsap2'	32.69	33.43	49.7	7.14	25.4	38.92	14.6	39	35.44	52.92
212632	'Iffo2'	0.19	1.88	0.3	16.4	0.63	0.01	0.38	1.4	0.02	0.16
212647	'Aldh4a1'	19.54	13.12	12.4	26.3	15.2	3.57	15.1	13.88	12.17	16.83

212670	'Catsper2'	4	0.71	0	0	1.41	0.1	0.01	0	2.04	1.35
212679	'Mars2'	3.72	0	1.69	0	0.05	6.48	1.46	3.69	6.5	0
212706	'N4bp3'	14.56	2.36	16	0	0	4.95	0	0.39	5.48	1.66
212712	'Satb2'	0	0	0.01	0	0	0	0	0	0	0
212728	'Tarbp1'	0.18	0.33	0.07	0.01	2.01	0.38	0.19	0.02	0.64	1.2
212733	'Bicdl2'	0	0	0	0	0	0.07	0	0	0	0
212772	'Arl14ep'	8.68	12.81	11.9	16.4	7.93	8.36	4.3	12.39	11.19	12.89
212862	'Chpt1'	5.38	2.55	4.51	5.82	3.58	5.39	0.48	2.16	2.97	3.56
212880	'Ddx46'	14.85	11.16	12.7	17.3	5.25	16.29	8.83	7.54	13.54	5.37
212892	'Rsph4a'	7.82	6.13	10.8	32.6	10.4	1.59	1.98	0.87	1.68	15.11
212898	'Dse'	2.91	0.05	1.2	0	0	0	0	0.11	0	0.17
212919	'Kctd7'	7.24	7.1	9.77	8.5	4.24	3.76	3.27	3.88	1.13	6.8
212933	'Pm20d1'	0	0	1.63	1.59	0	0.02	0	0	1.99	0
212937	'Tifab'	0	1.64	0	0	0	6.17	0	2.21	0	0
212943	'Tent5a'	0.42	9.58	1.24	14.9	0.01	1.7	1.66	2.66	11.71	4.38
212974	'Pgghg'	0	0	3.03	0	0.02	0	0	0	0.04	4.05
212980	'Slc45a3'	0	0	0	9.09	0	0	0	0	0	0
212986	'Scfd2'	10.71	4.72	0.77	14.7	6.22	10.35	7.64	9.8	3.92	2.61
212989	'Best2'	0	0	0	0	0	0	0	0.02	0	2.76
212996	'Wbscr17'	1.63	2.18	1.09	1	3.62	2.46	3.76	3.35	1.72	3.33
212999	'Tnpo2'	2.23	2.63	4.87	4.22	8.88	4.14	2.65	3.46	9.83	3.52
213002	'Ifitm6'	0	5.66	0	0	1.3	0	0	0	0	0
213006	'Mfsd4a'	19.96	13.91	27.7	28.4	31.5	16.24	20.7	15.79	20.04	23.76
213011	'Zfp583'	6.66	2.61	0.31	0.24	0.11	4.86	2.3	0.13	4.61	3.11
213012	'Abhd10'	46.1	65.2	57.5	52.9	58	47.04	91.4	54.89	67.9	73.92
213019	'Pdlim2'	0	11.06	0	0	43.5	35.02	0.04	3.42	0	3.64
213027	'Evi5l'	20.38	18.89	18.2	23.8	23.3	15.33	22.7	20.6	16.61	11.38
213053	'Slc39a14'	1.48	0.71	1.22	1.83	1.18	0.89	0.01	0.44	0	1.58
213054	'Gabpb2'	1.12	0.28	0.17	0.35	2.14	1.03	0.21	0.09	1.34	0.76
213056	'Fam126b'	10.39	4.09	12	10.1	8.47	2.33	14	7.13	12.95	7
213068	'Tmem71'	0	0	0	4.95	0	0	0	0.03	0	0
213081	'Wdr19'	6.89	1.83	1.47	3.94	7.3	2.03	10.7	0.93	1.04	2.57
213084	'Cdkl3'	1.41	14.79	7.35	20.8	16.1	13.3	1.93	13.7	9.99	4.66
213109	'Phf3'	2.24	2.36	3.53	3.46	1.43	2.81	4.79	2.28	1.13	2.41
213119	'Itga10'	0	0	0.12	0.1	0.05	0	0	0.16	0	0.7
213121	'Ankrd35'	12.37	10.93	16.8	12.1	2.07	9.74	10	11.54	12.43	16.32
213171	'Prss27'	0.04	0	1.42	0	0	0	0	0.22	0	0
213208	'Il20rb'	1.84	0	1.91	0	0	0	0	0.02	0.24	0
213211	'Rnf26'	0.89	0.02	0.63	0	3.44	6.21	5.67	13.75	0	0.02
213233	'Tapbpl'	12.05	22.39	12.9	37.1	16.6	7.75	8.33	23.49	27.96	16.79
213234	'Zbbx'	1.31	2.74	0	0.1	0.7	3.65	1.94	1.2	1.97	8.35
213236	'Dnd1'	0	0.36	0.07	0	0	0	0.03	0.5	0	0
213262	'Fstl5'	103.1	55.51	17.4	47.8	6.59	3.96	55.2	34.63	19.48	33.52
213272	'Txndc2'	0	0	0	0	0	0.13	0	0.23	0	0
21331	'T2'	4.02	1.96	0	0	2.88	0.05	0.09	0	0	0
213311	'Fbxl21'	11.93	4.22	6.93	4.19	7.15	3.12	11.6	10.23	21.68	12.95
213326	'Scyl2'	5.83	8.03	3.42	10	4.3	6.19	6.72	5.99	20.2	7.01
21333	'Tac1'	0.04	2.45	47.4	5.76	18.5	6.66	8.65	24.39	0	1.91
21334	'Tac2'	0	0	4.76	0	0	0	0	35.45	5.85	0
21335	'Tacc3'	6.99	0.37	3.77	9.24	3.33	0	0	2.84	6.19	0
213350	'Gatd1'	29.34	31.3	35.4	10.7	36.5	29.09	70.6	41.98	34.87	41.74
21336	'Tacr1'	0.6	9.53	3.37	6.04	3.21	1.11	3.46	13.83	15.45	7.43
21337	'Tacr2'	0	0	0	0	0	0	0	0	2.82	0
21338	'Tacr3'	0	0	2.49	3.8	0	0	0	0	0	5.39
213389	'Prdm9'	1.88	0.22	0.11	1.3	3.22	0.06	0.09	0.05	0.06	0.69
21339	'Taf1a'	13.15	9.16	5.7	6.78	9.62	0.15	2.47	10.04	8.05	10.78
213391	'Rassf4'	0.52	0.29	0.59	0	2.75	1.05	0.62	0.49	0.31	0.98
213393	'Depp1'	0.03	0.04	0.04	0.04	0	0	0	0	0	0

21340	'Taf1b'	20.06	14.06	27.6	22.1	15.4	7.61	19	23.08	16.65	19.84
213402	'Armc2'	1.29	0.06	0.61	1.63	0.03	0.36	3.12	2.13	0.45	2.4
213409	'Lemd1'	0.52	0	0	0.3	2.23	15.08	6.08	2.99	25.89	11.09
21341	'Taf1c'	3.03	1.05	3.83	0	6.58	1.46	2.07	6.17	0	2.53
213417	'Klhdc8a'	5.26	1.67	4.04	0.02	7.01	0	5.16	5.29	0.7	9.82
21343	'Taf6'	18.39	30.27	31.7	50.1	25.1	36.57	13.1	42.6	31.9	47.59
213435	'Mylk3'	0.03	0	0.13	0.06	0.02	0.04	0.08	0.02	0	0.01
213438	'P2ry10b'	0.01	0	0	0	0	0	0	1.21	0	0
21345	'Tagln'	0	0.03	0	0.04	0	0	0.26	0	0	0.04
213452	'Dstyk'	4.51	2.96	4.1	1.49	4.04	2.49	6.84	5.71	7.79	4.32
21346	'Tagln2'	6.95	7.6	16.5	0	1.52	11.18	18.3	2.92	36.36	35.61
213464	'Rbbp5'	13.69	8.6	28.6	21.8	6.78	7.88	17.3	22.28	18.98	17.28
213469	'Lgi3'	30.09	17.08	47.4	10.2	24.2	17.7	27.1	16.98	13.33	15.51
213484	'Nudt18'	34.6	26.72	37.3	13.2	28.8	60.31	51.2	29.8	32.38	2.63
213491	'Szrd1'	3.13	7.91	10.3	3.89	5.11	4.2	4.69	6.46	2.8	1.25
213498	'Arhgef11'	1.75	0.81	2.9	2.08	2.06	0	2.35	2.05	1.9	3.86
213499	'Fbxo42'	9.9	7.24	22	9.05	1.61	1.47	12	10.29	13.65	13.38
21350	'Tal2'	0	0	0	5.08	0	0	0	0	0	0
21351	'Taldo1'	94.48	97.29	96.1	107	143	162.3	89.6	100.8	92.57	75.98
213522	'Plekhg6'	0	0	0	0	0.11	0	0	0	0	0
213527	'Pth2r'	0	1.5	3.22		8.12	0	0	0	0	0
21353	'Tank'	24.66	18.8	12.2	32.6	11.7	14.18	9.77	10.25	17.26	19.05
213539	'Bag2'	24.45	10.19	15.7	18.5	7.85	0.38	9.76	37.8	7.91	24.97
21354	'Tap1'	0	3.34	0	6.72	0.1	0	4.46	3.58	1.35	1.56
213541	'Ythdf2'	26.3	22.29	24.9	36.9	11.7	13.72	13.6	28.22	24.15	32.24
21355	'Tap2'	3.38	7.66	9.44	15.5	1.81	10.92	2.6	2.83	21.81	6.62
213550	'Dis3l'	2.19	5.67	10.7	9.51	6.58	5.37	5.64	2.83	7.3	6.56
213556	'Plekhh2'	0.46	0.01	0.79	0.03	0.03	0	1.13	0.01	0.02	0.15
21356	'Tapbp'	16.77	23.38	18.7	41.7	60.3	34.92	32.3	18.44	84.7	74.43
21357	'Tarbp2'	14.26	6.37	19.1	23.3	6.67	15.78	25.8	31.4	18.92	17.32
213573	'Cracr2b'	0.44	0.03	0.09	0.08	0.06	0.03	0.04	0.03	0.05	0.05
213575	'Dync2li1'	60.69	50.17	49.9	10.2	22.8	66.75	57	31.1	33.05	51.57
213582	'Map9'	22.31	20.83	15.9	19	25.4	11.2	12	15.24	9.92	21.47
213603	'Slc44a3'	0	0	0	0	0	0	0	1.84	0.02	0.36
213649	'Arhgef19'	0.01	0	0	0	2.33	0.16	0	0	0	0
21366	'Slc6a6'	6.07	17	14.4	17.7	18	12.77	12.9	27.45	23.94	28.64
21367	'Cntn2'	4.62	3.9	13.7	8.38	7.32	6.8	2.15	1.3	4.89	4.5
213673	'9530068E07Rik'	83.81	92.13	60.1	46.8	60.2	81.57	70.5	86.97	118.7	123.85
213696	'Duoxa1'	0	0	3.8	0	0	0	0	0	0	0
21371	'Tbca'	192.6	191.5	246	201	86.2	160.1	358	156.7	186.7	188.94
21372	'Tbl1x'	19.1	21.66	21	14.1	35.6	10.02	8.48	32.22	31.44	29.54
21374	'Tbp'	14.68	7.96	12.8	12.8	21.3	9.89	4.45	25.05	24.21	13.81
21375	'Tbr1'	0	0	0.01	0	0.68	11.58	0.04	0.27	3.3	3.53
213753	'Zfp598'	1.13	5.36	2.71	2.66	1.3	2.45	1.25	2.92	1.06	0.35
21376	'Tbrg1'	34.33	63.52	67	112	74.7	95.89	46.3	49.74	104.2	78.86
213760	'Prepl'	206.5	172.9	243	201	134	51.94	225	170	174.5	239.24
213773	'Tbl3'	6.09	25.69	14.8	18.2	32.3	28.43	9.82	31.74	21.42	12.41
213783	'Plekhg1'	1.48	0	0	2.13	0.05	0.15	0	0.6	0.01	0
213788	'Chrm5'	2.34	3.91	0	0	5.56	0	0	0	5.04	3.52
21379	'Tbrg4'	10.83	10.42	14.8	2.35	1.02	7.78	14.7	8.04	12.31	4.79
213819	'Casd1'	16.47	15.82	10.8	19.2	14.8	5.87	16	14.74	21.14	19.58
213827	'Arcn1'	55.09	52.88	64.5	54.8	36.9	21.02	52.1	60.42	48.87	48.85
21385	'Tbx2'	0	0	0.53	0	0	0	0	0	0	0
21388	'Tbx5'	0	0	0	0	0	0	0	0	0.01	0
21389	'Tbx6'	0	0	0	0	0	0	1.82	0	0	0.33
213895	'Bms1'	3.74	6.72	3.68	6.23	6.48	4.74	15.9	4.54	9.26	4.77
21390	'Tbxa2r'	0	0	0	0.03	0	0	0	0	0	0
21391	'Tbxas1'	0	0.92	0	0	0	3.1	0	0	0	3.64

21393	'Tcap'	1.5	0.26	9.23	1.44	1.4	2.92	1.32	0.66	1.61	1.22
213945	'Col28a1'	0	0.8	0	0	0.31	0	0	0	0	0
213948	'Atg9b'	1.46	5.46	4.5	1.65	3.57	6.98	12.7	3.38	7.56	3.63
213980	'Fbxw10'	0	0	1.39	0	3.8	0	0	0.46	1.73	0
213988	'Tnrc6b'	3.02	1.73	3.95	4.22	3.53	3.21	1.85	2.08	4.02	2.08
213989	'Tmem82'	0.37	0.27	0.3	0.44	0.64	0.48	0.33	0.14	0.65	0.61
21399	'Tcea1'	55.35	49.88	58.4	49.2	39	36.79	26.5	50.71	54.86	33.93
213990	'Agap3'	18.68	14.53	26.6	28.3	19.1	24.61	23.8	8.23	22.68	19.43
213993	'Ccdc186'	9.52	10.34	8.3	7.51	14.6	10.56	5.72	11.51	5.56	9.97
21400	'Tcea2'	102.9	88.65	79.5	116	74.3	82.23	93.7	129.8	103.7	119.51
21401	'Tcea3'	0.29	1.93	0.08	0.05	0.85	0	0.04	1.35	0.46	0.14
21402	'Skp1a'	868.6	1026	735	597	966	1160	905	1181	871.3	1010.73
214048	'Larp1b'	1.46	0.48	0.08	0.03	2.78	0.23	0.58	0.58	1.54	2.25
21405	'Hnf1a'	0	0.42	1.3	0	0	0	0	0	0.88	0
214058	'Megf11'	6.8	3.2	30.2	2.55	12.5	4.41	2.17	4.1	0.97	2.04
21406	'Tcf12'	1.45	2.31	2.21	0.43	0.14	0.01	1.05	0.73	1.81	1.45
214063	'Dnajc16'	10.49	3.84	9.1	8.38	6.28	0.46	0.55	16.49	4.83	15
21408	'Zfp354a'	1.91	1.08	0	5.62	6.18	0	2.48	1.72	0.3	1.49
214084	'Slc18a2'	8.15	30.92	0.31	20.1	4.27	1.65	0.25	2.67	9.54	0.79
21410	'Hnf1b'	5.63	9.55	8.86	4.03	3.6	2.7	0	12.41	9.51	3.38
214106	'4933430I17Rik'	0	0	2.44	0.23	0.13	0	0	0	0	1.16
21411	'Tcf20'	7.35	6.26	7.91	8.26	10.3	4.83	4.24	4.82	5.1	7.54
214111	'Slc24a1'	0.25	1.13	1.25	2.16	1.58	0	0	0	0.01	0
214112	'Nipal4'	0	0.89	0	0	0	2.94	0	1.3	0	0
21413	'Tcf4'	8.98	7.69	5.25	13.5	6.79	12.7	8.27	7.9	1.97	4.72
214133	'Tet2'	1.55	3.55	1.88	3.5	0.57	2.58	5.09	2.12	1.65	0.41
214137	'Arhgap29'	0.47	0.05	0.02	0	0	0.53	0	0	1.18	0
21414	'Tcf7'	0	0	0	0	0	0.02	0	0.2	0	0
21415	'Tcf7l1'	0.06	0.7	0	0	0	0	0.05	0	0	0
214150	'Ago3'	6.17	6.62	3.5	11.5	2.45	2.12	6.77	1.72	6.93	3.77
21416	'Tcf7l2'	12.11	4.15	3.35	4.55	1.1	1.54	2.61	4.71	7.04	0.36
214162	'Kmt2a'	5.39	3.36	4.26	7.61	3.8	6.85	7.43	3	5.3	4.05
21417	'Zeb1'	13.32	9.05	9.43	12.4	9.52	6.37	18.7	9.2	7.13	7.88
21418	'Tfap2a'	0	0	0	0	0	0	0	0	0.01	0.01
214189	'Scgn'	6.28	18.22	0.03	0.7	0.07	0.03	0	1.82	0	0
214191	'Ttc24'	0	1.15	0	0	0	0	0	0	0	0
21422	'Tfcp2'	11.1	7.79	5.99	11.3	7.67	4.29	12.4	6.4	11.07	13.5
21423	'Tcf3'	1.58	2.25	0.13	0.17	0.11	2.42	0	3.71	3.7	3.52
214230	'Pak6'	8.26	18.57	15.3	25.4	7.54	10.57	19.4	7.54	9.83	14.23
214239	'Ccdc9b'	0.32	0.28	1.18	0	0	0	1.59	1.36	0	0
214240	'Disp2'	39.95	40.36	100	30	33.9	23.23	64.4	39.53	47.71	54.95
21425	'Tfeb'	0	0	0.02	0	0.72	1.38	0	0	0	0
214253	'Etnk2'	9.37	2.82	10.1	11.1	10.6	9.93	6.25	14.61	12.78	4.92
214254	'Nudt15'	6.13	1.86	2.24	0	2.76	4.71	3.95	0.51	0.02	2.45
21427	'Vps72'	54.24	55.51	44	37.9	54.9	91.86	30.8	60.34	55.16	52.81
21428	'Mlx'	58.47	54.43	33	45.1	23.6	25.06	65.6	49.96	35.39	30.38
21429	'Ubtf'	15.35	8.09	15.5	3.71	16.8	25.29	23	3.32	12.65	12.91
214290	'Tut7'	3.35	3	4.7	2.17	2.98	1.33	6.71	3.28	1.75	2.54
214292	'Syna'	0	1.34	1.5	0	0	0	0	0	2.76	0
214301	'Crygn'	0.4	0.09	0	0	0.26	0.25	0.27	0	0.55	0.23
214305	'Hhipl1'	0.55	0	0.02	0	0.41	0	1.32	0	0	0.03
214321	'Gm4787'	0.28	0	3.35	0	0.41	0	0	1.17	0	0.65
214345	'Lrrc1'	0	1.02	0.03	0.06	5.39	0.66	0.02	2.1	2.84	0.26
214359	'Tmem51'	4.86	2.57	0.03	10.2	4.35	0	0	0	0	0
214384	'Myocd'	0	0.01	2.06	0.01	0	0	1.28	0.01	0.01	0.01
214424	'Parp16'	6.9	7.46	4.77	7.03	4.01	3.7	4.55	0.77	2.63	2.25
214425	'Cilp'	0	0	0	0	0	0	0	0	0	1.69
214444	'Cdk5rap2'	0	0.15	0	0.68	0.12	0	0.79	0.07	0.87	0.17

214459	'Fnbp1l'	12.34	16.15	13.1	9.51	12.7	16.04	18	18.59	16.11	21.78
214469	'Fam168b'	3.62	5.21	7.91	20.2	14.6	11.68	13.5	4.04	10.39	1.84
214489	'BC003965'	18.68	19.63	17	17.4	13.8	21.68	15.3	22.95	13.52	7.86
214498	'Cdc73'	2.9	0.99	1.3	2.46	2	1.25	1.64	1.28	1.15	3.01
214505	'Gnptg'	182.7	253	155	146	162	272.6	188	137.2	213.9	308.82
21452	'Tcn2'	3.99	3.34	6.28	20.4	0.03	38.62	13.5	10.05	9.93	14.64
21453	'Tcof1'	6.78	2.28	4.58	7.58	0.99	1.03	9.8	9.32	4.7	4.81
21454	'Tcp1'	147.9	167.9	91.6	91.6	111	167.1	129	134.9	107.5	133.73
214552	'Cep164'	2.33	2.28	4.7	14.9	4.41	5.95	1.71	3.1	2.94	1.17
214568	'Gm136'	0	0	0	4.51	0	0	0	0	0	0
214572	'Prmt7'	26.77	65.66	36.7	66.7	66	93.31	43.8	70.55	55.9	50.37
214575	'Tdrd5'	0.22	0.68	2.99	0.59	0.38	1.1	0	2.02	0.12	5.7
214579	'Aldh5a1'	4.98	2.97	5.64	5.4	6.63	2.22	9.48	2.89	5.19	1.96
214580	'Pstk'	23	33.66	31.9	15	39.2	29.39	23.8	22.32	6.89	17.49
214585	'Spg11'	0.35	0.38	0	0.79	2.2	0.11	0.64	1.1	3.13	0.52
214597	'Sidt2'	2	4.06	8.6	7.14	9.47	2.67	10.1	6.92	8.15	3.02
214601	'Slc10a3'	6.78	7.69	3.87	0.03	0	3.21	0	3.23	8.41	13.99
21461	'Tcp10a'	0	0.03	0	0	0	0	0	0.07	0	0
214616	'Spata5l1'	0.54	0.35	0	5.91	1.32	1.13	0	0	0	1.1
21462	'Tcp10b'	0	0.3	0	0	0	0	0	0.68	0	0
214627	'Tent4b'	0.74	0.78	5.39	6.16	1.98	2.36	3.56	0.9	0.19	4.2
21463	'Tcp11'	0.33	3.47	0.55	3.25	0	0	0	0.23	0.24	0.27
214642	'Cped1'	0	0	0.1	0.21	0	0.89	0.9	0	1.98	0.07
214663	'Slc25a29'	17.6	27.14	20.6	15.2	25.3	10.2	30.4	7.87	31.8	9.68
214669	'L3mbtl2'	12.41	21.5	21.6	15.4	26.9	16.37	8.07	24.48	24.42	25.76
214685	'Chadl'	3.16	4.97	4.2	9.76	9.14	4.25	7.89	11.08	9.1	11.39
214704	'Iqub'	2.44	1.4	0.73	0	1.03	4.6	0.02	0.89	1.21	2.45
214742	'Rcor3'	10.75	11.9	12.4	16.2	11.4	8.85	4.38	9.8	6.77	14
214763	'Cgas'	0.64	0	0.1	0.03	1.28	0.02	0.02	0.06	0	0.92
214764	'Edrf1'	3.14	3.12	0.58	10.2	0.11	1.16	6.9	2.15	3.28	0.17
214766	'Mmp21'	0	0	0.21	0	0	0.38	0	0	0	0
214779	'Zfp879'	0.12	0	0	0	0.12	0	5.48	0.5	3.26	0
214791	'Sertad4'	3.27	1.35	0.77	7.09	8	1.44	0	2.48	6.42	3.9
214804	'Syde2'	0.22	0	0.01	0	0.26	0	0.01	0	0	0.06
214812	'Zfp609'	1.06	1.06	1.15	0	0.89	0.29	0.01	1.04	2.05	0.98
214854	'Neurl3'	0.03	3.09	0.14	0	0.02	0	0.02	0.02	0.02	0
214855	'Arid5a'	1.37	2.86	3.6	0.02	0.97	1.56	0	0	1.51	3.01
214895	'Lman2l'	76.61	62.39	37.2	26.4	71.1	76.49	59.3	48.63	68.08	117.91
214897	'Csnk1g1'	5.53	2.44	2.07	2.96	0.98	1.79	4.15	0.87	6.45	3.31
214899	'Kdm5a'	8.95	8.01	12.2	10.6	3.31	10.34	10.5	12.05	9.81	8.46
214901	'Chtf18'	0	0	0	0	0	0.1	0	0	0	0
214917	'Fam173a'	212	190.4	213	135	285	261.6	295	217.7	161.4	225.98
214922	'Slc39a2'	2.29	1.67	0	0	0.15	0	0	0	0	2.24
214931	'Fbxl16'	36.2	24.57	30.3	10.3	26.7	18.82	28.7	27.59	22.17	16.04
214932	'Hdhd5'	13.27	9.18	13.4	34.2	15	18.8	19.1	12.55	23.06	5.61
214944	'Mob3b'	2.32	0.68	0.03	0.01	0.92	0.02	0.01	2.5	0.01	0.01
214951	'Rhbdl1'	19.46	27.84	15.6	38.2	22.8	19.76	15.2	53.75	77.26	52.21
214952	'Rhot2'	24.57	23.24	15.8	36.1	16.1	31.65	10.5	20.15	22.73	27.84
214968	'Sema6d'	5.82	5.82	5.88	0.01	11.2	6.22	1.72	3.65	9.07	8.28
214987	'Chtf8'	10.71	21.47	15.2	6.99	13.9	6.73	13	18.27	14.86	23.32
215001	'Wfikkn1'	0	1.47	3	1.76	0	2.09	0	0	2.67	0
215008	'Vezt'	7.55	4.6	4.2	4.24	4.04	4.82	3.63	4.51	2.94	3.28
215015	'Fam20b'	8.84	13.39	7.44	11.5	12.6	4.7	2.76	6.01	23.14	13.03
215051	'Bud13'	5.16	2.54	1.15	5.02	8.53	1.91	9.74	3.13	6.32	6.66
215085	'Slc35f1'	4.91	2.63	9.7	9.37	11.1	1.7	7.43	4.54	6.02	8.86
215090	'Maneal'	4.71	6.05	18.7	2.65	15.6	5.29	14.3	13	1.5	1.33
215113	'Slc43a2'	5.94	7.73	2.62	11.2	5.15	4.97	11.1	6.71	6.51	3.42
215114	'Hip1'	1.45	1.05	2.17	1.75	0.26	2.37	0.45	2.54	1.14	0.1

215160	'Rhbdd2'	219.5	167	232	144	193	249.3	252	228.1	226.2	316.25
215193	'Diexf'	1.37	4.93	1.21	5.01	8.11	0	15.7	5.97	5.29	4.34
215194	'Kri1'	15.71	16.12	8.53	14.2	8.33	12.76	5.12	5.81	4.09	12.82
215201	'Trmt2b'	2.29	3.77	4.03	4.2	4.12	0	0	8.84	20.98	13.04
215210	'Tmem120a'	32.65	37.79	11.1	16.6	12.6	35.29	11	29.97	16.35	5.53
215243	'Traf3ip3'	0	0	0	0	0.02	4.2	0	0	0	0
215257	'Il1f9'	1.62	1.53	1.71	2.34	2.2	1.68	0.58	0.98	2.31	1.46
215280	'Wipf1'	0	0	0.64	0.98	0.15	1.2	0.01	0.3	0	0.05
215303	'Camk1g'	46.91	36.08	41.1	13	25.5	60.81	50.9	45.14	40.74	36.56
215335	'Slc36a1'	1.35	0	2	0.88	0.01	1.67	0	2.14	2.01	1.83
215351	'Senp6'	1.21	3.31	3.25	11	6.98	4.99	1.89	2.48	2.06	4.05
215378	'Brinp3'	20.15	40.67	22	0.8	5.47	29.62	37.9	26.12	34.15	43.95
215387	'Ncaph'	0	0.11	2.57	0.02	0	2.76	0	0.92	0.05	0
215418	'Csrnp1'	0.55	9.97	8.91	24.7	0.07	7.08	9.1	0	5.08	5.08
215436	'Slc35e3'	3.34	6.4	7.77	8.44	6.12	0.63	3.61	4.43	13.58	9.06
215445	'Rab11fip3'	5.28	4.95	3.18	7.12	4.46	3.02	5.27	6.44	2.01	0.56
215446	'Entpd3'	7.03	2.27	13.1	1.97	2.32	2.5	0	7.91	9.33	1.59
215449	'Rap1b'	15.67	6.81	7.64	16.8	9.86	16.33	5.96	6.85	25.91	10.79
215456	'Gpat2'	0	0	0	0	0.32	0	0	0	0	0
215467	'Gm4791'	0.45	0.57	1.24	1.81	1.2	1.02	1.49	0.33	0.92	1.04
215474	'Sec22c'	7.53	12.25	4.98	6.97	0.93	6.45	3.3	0.32	10.46	4.52
215476	'Prr14l'	3.13	7.55	5.84	4.89	2.24	3.23	6.82	5.45	3.73	3.88
215493	'A3galt2'	0	0.15	0.01	0	3.82	0	0	0	0.01	0
215494	'Pomgnt2'	109.1	93.47	103	63.2	58.1	102.9	56.8	120.1	67.51	121.06
215512	'Fam117a'	0.13	0.02	0.68	0.42	6.15	0.39	0.02	0.03	0	0.09
215615	'Rnpep'	28.81	29.46	15.1	2.97	17.7	56.16	16.7	27.05	26.72	30.29
215627	'Zbtb8b'	2.1	0.51	2.59	0.83	2.39	0.41	0.63	1.36	0.52	0.55
215632	'Psd4'	0	0	0	0	2.17	0	0	0	0	0
215653	'Rassf2'	2.92	3.29	3.95	0	7.64	0.12	8.66	1.34	0.67	3.27
215654	'Cdh12'	2.01	0.81	1.06	3.18	0.48	1.33	2.37	6.61	11.07	4.13
215690	'Nav1'	6.2	7.27	5.57	12	5.32	5.78	9.38	5.99	4.05	4.53
215693	'Zmat1'	18.87	14.19	16.5	12.9	7.57	20.46	7.96	26.86	18.59	20
215705	'Arrdc1'	9.72	17.07	12.2	26.6	12.5	16.02	3.82	17.67	26.23	23.43
215707	'Ccdc92'	58.14	26.29	49.1	64.7	18.7	26.3	62.7	40.42	48.54	11.83
215708	'Miga1'	4.86	2.35	4.28	8.65	5.01	6.94	6.79	6.88	5.6	9.94
215748	'Cnksr3'	0	0	0	0	0.03	0	0.01	0	0.58	0
215751	'Ginm1'	41.7	50.95	38.2	64.6	23.1	21.58	22.1	38.16	33.2	26.93
215772	'Adgb'	1.03	0	0.01	3.53	0	0	0	1.15	0	0
215789	'Phactr2'	13.95	10.7	8.37	11.4	21.3	29.37	3	18.14	4.17	16.6
215814	'Ccdc28a'	95.97	42.3	54.9	56.2	67.1	83.8	54.9	92.6	65.28	40.72
215819	'Nhsl1'	0.36	1.29	2.94	7.95	0.02	0.27	0.01	0.6	0.35	0.82
215821	'Arfgef3'	6.56	5.29	8.06	4.45	4.55	1.55	3.29	3.53	12.3	7.51
215890	'Clvs2'	9.77	17.96	27.6	6.09	12.6	16.04	16.7	28.76	20.37	15.21
215900	'Calhm6'	0.04	0	0	0	0	5.5	0	0	0	0
215928	'Mfsd4b5'	0.17	0.41	0.16	0.02	0.09	0.41	0.39	0.05	0.12	0.06
215929	'Mfsd4b1'	0.33	0.2	0.11	0	0.05	4.26	3.35	1.45	2.38	0.03
215951	'Afg1l'	7.41	9.64	5.26	11.8	10.9	11.2	6.3	10.23	2.47	11.86
215999	'Mcu'	11.01	6.93	20.1	9.45	17.6	3.19	8.3	10.69	7.36	0.06
216001	'Micu1'	33.79	32.48	42.5	17.3	26.9	27.43	22	23.86	10.51	17.93
216011	'Lrrc20'	5.86	2.2	3.52	0.05	11	4.7	1.5	8.24	4.97	3.29
216019	'Hkdc1'	0	0	0	0	0	0	0.35	0	0	0
216021	'Stox1'	2.54	0.54	0.96	1.63	2.55	1.97	0.03	0	1.94	1.43
216028	'Lrrtm3'	25.57	31.64	33.3	36.8	20.7	15.24	52	25.07	22.56	18.51
216033	'Ctnna3'	1.62	0	0.64	0	0.48	0.02	0	0	0.01	0
216049	'Zfp365'	20.96	17.2	11.3	9.18	29	8.23	9.44	7.07	11.86	8.89
216080	'Ube2d1'	27.5	16.39	27.3	8.78	20.3	14.57	32.6	15.14	22.67	9.54
216119	'Ybey'	2.6	3.22	1.17	6.08	5.09	0.13	5.05	4.93	1.28	2.66
216131	'Trappc10'	2.21	2.4	1.77	1.77	2.4	8.6	9.95	4.17	0.99	2.86

216134	'Pdxk'	66.37	49.76	79.2	17.9	52.4	46.89	63.4	47.43	67.19	58.11
216136	'Ilvbl'	39.65	22.73	30.3	7.52	18.5	26.46	11.5	19.47	19.48	32.4
216148	'Shc2'	1.6	5.33	4.44	0.59	0.64	0.02	0.88	1.8	5.46	2.72
216150	'Cdc34'	40.4	31.75	28.4	7.13	35.5	27.99	7.74	20.92	24.1	12.58
216151	'Polrmt'	9.76	7.7	10.3	3.77	11.3	6.11	8.61	6.71	9.32	6.46
216152	'Plppr3'	78.51	71.31	80.7	44.6	63.8	50.55	84.4	83.96	92.82	104.45
216154	'Med16'	3.35	10.46	7.94	7.89	9.85	12.42	4.58	4.15	2.67	8.03
216156	'Wdr18'	66.74	68.14	46.8	56.6	66.1	134.2	78.3	98.09	90.89	75.18
216157	'Tmem259'	3.03	0.84	1.47	6.96	5.6	1.33	0.65	2.92	3.26	2.19
216161	'Sbno2'	2.27	0.89	2.24	0.39	2.25	0	3.66	0.21	0.36	1.37
216166	'Plk5'	3.96	5.97	0.05	0.3	57.8	6.09	24.4	8	0.02	16.72
216169	'Abhd17a'	5.12	5.76	6.03	8.09	14.7	10.54	9.92	2.54	18.39	13.24
216177	'AU041133'	7.77	0.05	0.54	1.68	0.69	2.91	0	0.82	2.83	9.44
216188	'Aldh1l2'	1.92	1.89	0.02	0	3.25	0	4.53	3.34	2.6	1.67
216190	'Appl2'	1.9	14.86	12.9	6.15	4.96	6.05	4.57	7.71	2.57	5.74
216197	'Ckap4'	4.88	2.61	8.78	0	7.65	3.12	1.19	4.79	2.46	3.75
216198	'Tcp11l2'	24.48	22.91	9.3	20.5	22.8	24.51	18.1	19.97	11.03	23.05
216225	'Slc5a8'	0	0	0.73	0	0	0	0	0	0	0
216227	'Slc17a8'	0.02	2.63	0.02	0.12	0.09	0.02	0.02	0.02	0.08	0.11
216233	'Socs2'	5.75	6.54	8.01	20.5	19.5	6.65	5.41	6.79	4.02	19.62
216238	'Eea1'	3.76	2.51	3.88	8.7	3.4	1.86	3.19	2.93	3.38	3.84
216274	'Cep290'	3.03	2.78	3.01	1.87	3.1	2.53	3.23	0.93	3.37	4.98
216292	'Mettl25'	6.12	10.07	8.76	4.01	7.91	8.41	7.43	6.11	13.11	11.35
216343	'Tph2'	0	0	0	0	1.42	0	0	0	0	7.94
216344	'Rab21'	6.46	6.12	3.1	13	4.5	0.03	3.67	1.62	2.84	8.55
216345	'Zfc3h1'	1.86	2.63	1.36	2.17	1.69	1.34	1.46	2.06	2.11	1.37
216363	'Rab3ip'	5.8	10.56	11.4	10.7	12.3	5.09	10.6	13.45	13.95	2.14
216393	'D930020B18Rik'	0.11	1.37	0.21	22.7	6.48	2.8	2.42	0.45	0.92	5.08
216395	'Tmem5'	35.62	35.65	30.2	10.3	30.1	35	4.39	20.18	33.02	15.28
216438	'March9'	0.8	0	0	2.89	0	0	0	0	0	1.62
216439	'Agap2'	2.41	1.98	0.32	0.08	0.11	1.41	5.35	0.34	2.75	1.34
216440	'Os9'	49.77	87.71	53.6	34.8	41.1	56.13	53.1	67.93	95.18	57.38
216441	'Slc26a10'	2.71	0	0.15	0	0.42	0.1	0.02	0	0.26	0
216443	'Mars'	29.31	60.92	19.5	34.5	28.7	45.54	48.9	29.01	39.88	31.04
216445	'Arhgap9'	0.52	0	3.44	0.16	0	0	0	0	0	0
21645	'Tcte1'	0	4.75	0	0.57	0.05	0	0.07	0.28	11.12	0
216456	'Gls2'	10.91	15.03	11.5	0.07	2.16	0.19	11.3	5.06	13.58	4.96
216459	'Myl6b'	1.3	0.39	0.05	0.92	0.64	1.67	0.62	0.17	0.65	0.43
21646	'Tcte2'	0.03	3.85	0	0	0	0	0	0	0	2.83
21647	'Tcte3'	0	0	0	0	0	0.26	0	0	0	0
21648	'Dynlt1b'	65.65	66.41	20.4	26	89.1	34.8	41.7	45.88	55.46	58.39
216505	'Pik3ip1'	37.57	24.1	17.2	25.2	47.2	19.04	36.5	20.56	21.49	6.74
216516	'Ccdc157'	3.17	6.48	8.96	7.44	3.76	4.62	3.38	0.79	5.98	2.6
21652	'Phf1'	32.2	40.07	38.3	17.6	32	27.37	22.9	16.8	16.14	13.48
216527	'Ccm2'	21.73	16.51	14.5	5.31	6.13	11.18	4.14	14.06	33.04	14
216543	'Cep68'	2.23	3.6	1.83	0	2.59	1.43	1.82	5.46	2.04	1.16
216549	'Aftph'	8.98	10.35	6.06	1.93	5.04	6.04	3.05	3.92	11.19	7.78
216551	'Lgalsl'	15.7	20.18	11.8	9.87	9.76	2.57	13.5	16.3	10.97	16.99
216558	'Ugp2'	16.7	10.03	14	19.5	29.9	16.11	12.8	16.22	25.78	17.03
216560	'Wdpcp'	0.02	7.21	1.37	1.94	5.74	14.19	0.02	3.88	1.28	16.8
216565	'Ehbp1'	3.61	3.21	3.83	10	5.96	6.17	2.86	2.97	6.06	3.38
216578	'Papolg'	0.65	4.81	6.28	5.53	3.59	2.1	2.36	12.48	7.37	4.91
216613	'Ccdc85a'	16.24	16.06	21.1	8.83	5.85	0.99	8.04	7.53	6.94	7.23
216616	'Efemp1'	0	3.35	0	9.32	0	0	0	2.13	0	1.16
216618	'Cfap36'	80.17	86.1	101	104	115	94.31	96.8	88.51	78.94	88.88
216622	'4931440F15Rik'	0	4.82	0.01	5.14	0.01	0	3.01	0	1.72	5.32
216635	'Hbq1a'	2.48	2.58	2.76	0	12.3	24.08	17	11.22	13.87	5.56
21664	'Phlda1'	20.01	15.98	10	8.83	8.97	8.62	41.1	15.16	7.77	1.49

21665	'Tdg'	11.72	13.7	8.79	9.59	9.51	6.65	18.1	11.18	7.24	4.74
21667	'Tdgf1'	0	0	0	0.09	0	0	0	0	0	0
216705	'Clint1'	6.04	5.11	4.2	10.8	2.56	0.54	3.56	0.52	4.12	6.05
21672	'Prdx2'	403.2	370	335	389	460	537.4	230	402.5	292	341.9
216724	'Rufy1'	15.59	15.49	9.94	14.2	12	4.57	1.52	16.47	5.94	18.96
216725	'Adamts2'	0.43	1.57	1.28	0.04	0.06	0.15	4.72	0.02	4.03	1.17
216739	'Acsl6'	30.95	28.87	31.6	30.9	14.2	23.41	15.8	19.78	30.57	9.04
216742	'Fnip1'	0.73	4.13	2.13	4.55	2.63	0.23	2.72	1.61	4.4	2.26
216749	'Nmur2'	1.12	0	0.01	0	0	1.22	0	0	6.28	0
21676	'Tead1'	1.36	1.51	0.3	2.99	0.01	0.31	0.35	0.22	1.2	3.36
216760	'Mfap3'	8.13	9.66	1.78	7.38	8.67	8.81	7.32	8.07	11.78	11.05
216766	'Gemin5'	3.42	1.86	7.54	13.1	1.17	1.01	5.32	1.77	0.17	4.03
216767	'Mrpl22'	78.89	57.22	33.5	27.8	55.4	92.44	93.7	81.67	55.8	80.22
21677	'Tead2'	0.59	0	0	0	0	0	0	0	0	0
21678	'Tead3'	0.04	0	0	0	0.52	0.57	1.32	0	0	0.26
216781	'Trim58'	0	1.49	0.02	0	0	0	0	0	0	0.02
21679	'Tead4'	0	0	0	0	0	0	0	0	0.01	0.01
216792	'Iba57'	1.79	5.55	0	0	1.04	2.35	0	1.08	3.38	0
216797	'Prss38'	0	0	0	0.03	0	0	0	0	0	0
216799	'Nlrp3'	0	0.28	0	0	0	0.06	0	0	0	0
216805	'Flcn'	24.23	19.7	19	2.15	23.7	13.41	31.9	24.3	32.54	41.24
21681	'Alyref'	0.8	3.31	0.09	5.24	3.35	0.17	0.41	2.08	1.02	1.82
216810	'Tom1l2'	29.26	21.32	48	12.9	34.4	11.38	42.4	27.55	48.58	20.35
21682	'Tec'	0	0	0	0	0.04	0	0	0	0	0
216820	'Dhrs7b'	18.35	46.87	18	26	23.8	15.66	30.6	35.56	37.52	32.5
216821	'Tmem11'	52.43	53.8	83.7	67.2	56.7	67.47	39	69.22	45.31	36.43
216825	'Usp22'	97.8	124.1	121	97.8	60.8	33.25	139	113	98.67	103.72
216829	'Mmgt2'	18.19	4.27	5.22	10.2	18.2	26.75	0.04	2.77	34.75	25.45
21683	'Tecta'	0	0	0.06	0.11	0	0	1.45	0	0	0
216831	'Arhgap44'	19.1	22.52	24.5	22	21.3	26.2	22.3	27.68	22.2	22.78
216835	'Usp43'	0	0	0	0	0	0	0	0.1	0	0
216846	'Cntrob'	0	8.15	4.02	1.46	2.62	2.46	3.87	0.18	7.41	1.15
216848	'Chd3'	9.94	9.59	10.4	16.4	12.8	11.71	16.3	10.47	6.63	9.8
21685	'Tef'	18.74	13.1	12.8	34.6	19.6	7.07	15.9	18.47	9.7	18.33
216850	'Kdm6b'	3.79	5.23	3.86	19.3	4.54	1.78	4.2	3.22	3.36	5.28
216853	'Wrap53'	2.67	4.17	6.39	1.98	8.51	13.76	10.4	5.83	2.86	4.84
216856	'Nlgn2'	12.3	9.51	16.3	18.1	16.3	6.37	9.11	9.26	6.83	4.6
216858	'Kctd11'	0	0	1.3	0	3.99	0.07	0.02	0	0	0.22
216860	'Neurl4'	1.38	2.05	11.2	2.19	9.87	5.47	4.83	5.73	2.42	4.15
216864	'Mgl2'	0.03	2.49	0	0	0.03	0.03	0	0	0	0
216867	'Slc16a11'	6.78	10.86	15.9	2.39	30.9	15.06	24.3	18.5	16.31	5.53
216869	'Arrb2'	12.12	14.47	14.5	22.3	21.2	23.03	19.5	12.69	35.57	8.77
216871	'Gltpd2'	0	0	0	0	0	0	0	0	0	0.65
216873	'Spag7'	164	122.7	139	241	268	244.8	179	164.1	150.1	190.66
216874	'Camta2'	12.39	11.66	19.6	17	13.9	6.7	32.2	13.09	9.91	12.11
216877	'Dhx33'	2.01	4.82	2.11	4.66	5.03	0.25	5.47	0	2.97	0.05
216881	'Wscd1'	35.07	56.73	72.7	21.1	23.3	25.16	10.2	24.05	38.63	8.76
21689	'Tekt1'	13.09	45.29	0	67.7	5.69	0.48	8.64	14.38	3.56	13.42
216892	'Spns2'	4.39	2.12	1.44	0	13.3	2.22	4.71	3.54	6.03	3.64
216961	'Coro6'	9.9	1.79	32.3	0	18.5	2.18	0.38	23.56	3.87	0.52
216963	'Git1'	5.45	9.27	7.75	26.1	19.7	11.05	7.47	14.21	7.37	11.83
216964	'Trp53i13'	25.72	12.82	7.5	26.4	14.7	28.2	27.4	25.77	21.47	14.22
216965	'Taok1'	7.76	15.2	6.13	4.07	11.7	9.03	4.19	5.03	7.2	8.13
216971	'Fam222b'	11.85	8.53	8.28	14.8	10.2	9.06	14.3	9.67	10.03	9.53
216974	'Proca1'	3.82	3.82	2.57	2.4	2.91	12.05	9.31	1.69	3.24	2.12
216976	'BC030499'	9.34	4.67	3.46	1.49	5.12	13.02	0.08	1.86	0	4.44
216984	'Evi2b'	0	0.01	0.03	0.02	0.17	1.11	0.66	0	0.01	1.31
216987	'Utp6'	8.98	13.69	3.82	4.69	2.27	4.52	3.48	4.93	12.16	6.1

216991	'Adap2'	1.54	0	0	0	0	2.17	5.87	0.03	0	0
217011	'Nle1'	1.73	3.06	1.31	3.32	16.6	8.48	27.2	6.24	5.08	17.09
217012	'Unc45b'	0	0.03	0.08	0.02	0.01	0	0	0	0.02	0.01
217026	'Heatr6'	3.47	5.41	6.63	8.5	3.42	9.26	11.5	8.45	2.85	3.66
217030	'Synrg'	5.15	4.78	2.45	9.19	3.28	3.64	4.77	2.43	3.88	3.84
217031	'Tada2a'	12.54	10.1	22	11.5	20.2	23.49	12.5	24.72	24.14	29.12
217038	'Mrm1'	10.15	10.98	8.87	5.81	5.16	10.52	16.1	15	15.91	6.14
217039	'Ggnbp2'	46.43	42.99	46	57.6	40.4	34.98	45.5	38.57	51.01	18.49
217057	'Ptrh2'	14.44	11.06	11	7.79	16.5	18.12	6.54	10.19	21.24	17.36
217069	'Trim25'	0.24	0.96	0.37	1.38	0.21	0	0.04	0	0	1.08
217082	'Hlf'	5.79	5.7	4.26	4.89	3.37	4.71	0.45	5.16	2.63	1.83
217109	'Utp18'	6.56	4.79	3.64	8.75	5.17	3.66	3.77	2.72	3.54	5.8
217119	'Xylt2'	5.14	0	0.74	15.7	5.66	1.7	0	1.63	9.4	5.93
217124	'Ppp1r9b'	4.61	4.16	6.74	7.17	5.18	5.21	8.5	2.55	7.93	5.34
217125	'Samd14'	14.61	10.8	16.2	10.2	27.2	28.3	33.8	20.16	14.26	23.72
217127	'Kat7'	42.86	54.38	49	32.8	14.6	24.89	53.4	32.54	55.9	78.66
217140	'Scrn2'	0	7.98	2.82	0	10.2	25.89	4.73	23.03	9.85	4.78
217143	'Gpr179'	0	0	0	4.9	0	0	0	0	0	0
217149	'Cisd3'	95.72	70.66	79.5	166	114	135	65.7	101.8	62.15	85.5
217151	'Arl5c'	0	0	0	3.32	0	0	0	0	0	0
217154	'Stac2'	1.49	0	1.17	0.17	0	0	1.56	2.46	4.39	0.29
217166	'Nr1d1'	19.21	55.89	28.9	99.7	13.2	13.88	14.5	15.82	11.4	26.44
217169	'Tns4'	0	0.01	0.02	0.02	0.01	0.01	0	0	0.02	0
217194	'Klhl11'	2.63	5.07	2.83	0	2.38	7.08	12.6	4.33	3.52	6.31
217198	'Plekhh3'	0	1.06	2.05	0.98	1.17	1.92	1.27	0.72	0	0.34
217201	'Rundc1'	11.28	20.35	17.3	12	2.83	8.42	8.62	10.93	22.83	13.1
217203	'Tmem106a'	9.67	0	4.75	6.17	2.4	0.02	0	4.27	3.68	9.79
217207	'Dhx8'	2.85	3.74	12.3	0.49	3.6	6.85	0.56	8.1	9.57	1.43
217214	'Nags'	0	0	0	2.34	0	0	0	0	0	0
217216	'BC030867'	0	0	3.6	0	0	0	0	0	0	0
217217	'Asb16'	0.13	0	0	0	0	0	0	0	0	0.22
217218	'Atxn7l3'	2.61	2.72	3.95	0	5.77	4.5	5.22	2.17	3.15	1.16
217219	'Fam171a2'	0	0.14	0.38	1.11	0.96	0.41	0.18	1.6	0.4	0.04
217232	'Cdc27'	7.52	7.98	4.65	23.1	3.7	10.16	8.89	4.7	4.98	2.03
217246	'Ace3'	0	0.02	0	0	0	0	0	0	0	0
217262	'Abca9'	0.84	0	0	0	0	0	0	0	0	0
217265	'Abca5'	3.35	1.16	2.36	1.72	2.36	0.27	5.59	0.18	1.35	2.74
217303	'Cd300a'	0.21	2.2	2.61	1.68	0.58	0.57	0.36	3.33	1.71	0.37
217304	'Cd300lb'	0	0	0	0	0	0	0	0	0	2.4
217310	'Hid1'	56.66	60.79	56.2	44.5	55.6	61.3	65.9	62.22	83.06	70.21
217325	'Llgl2'	0	0	0	0	1.39	0.01	0	0	0	0
217331	'Unk'	7.83	11.92	7.6	4.1	4.03	7.34	11.3	9.95	7.02	14.31
217333	'Trim47'	0	0	0	4.1	1.48	0	0	0	0	0
217335	'Fbf1'	3.06	6.6	9.47	9.43	4.19	6.75	8.86	8.7	12.1	5.5
217337	'Srp68'	54.2	59.07	50.5	64	73.4	75.19	71.4	51.79	84.64	51.24
217340	'Rnf157'	9.26	4.25	2.38	0.98	11.1	8.83	14.8	4.2	1.41	3.91
217341	'Qrich2'	0	0	0	0	0.09	0	0	0	0	0
217342	'Ube2o'	8.02	7.63	12.4	7.23	19.1	8.18	5.32	11.55	5.3	6.8
217344	'Rhbdf2'	0	0	0	0	0.29	0	0	0.58	0	0
217351	'Tnrc6c'	7.11	6.3	6.11	6.03	8.81	5.49	5.23	4.87	7.28	7.07
217353	'Tmc6'	0.16	0.07	0	0	1.33	0	0	0	0	0.5
217356	'Tmc8'	0	0	0	1.25	0	0	1.09	0	0.08	0
217364	'Engase'	2.08	1.8	2.16	4.49	0.72	0.45	0	0	0	1.65
217365	'Nploc4'	1.97	1.54	3.36	1.22	3.22	3.21	3.73	3.59	7.57	1.95
217366	'Lrrc45'	43.78	45.68	71.2	62.2	59.7	87.94	52.2	61.97	54.6	65.95
217369	'Uts2r'	40.2	77.2	11.5		59.3	216.1	195	134.2	168.3	153.83
217370	'Cybc1'	22.32	22.03	26.5	8.1	19.3	25.12	5.46	24.54	18.9	12.67
217371	'Rab40b'	7.92	23.87	2.54	24.9	10.2	6.24	6.84	7.32	6.44	8.19

217378	'Dnajc27'	19.07	17.56	43.4	18.5	23.3	11.82	26.2	28.29	18.08	22.9
217379	'Ubxn2a'	17.56	14.58	23.1	10.7	12.6	14.65	19.5	14.23	12.61	7.79
217410	'Trib2'	0.96	13.14	5.89	6.82	4.4	3.84	17.4	9.39	2.56	3.89
217430	'Pqlc3'	0	2.38	0.85	0	0	0	0	0.53	1.58	0.55
217431	'Nol10'	9.73	13.12	12.7	13.8	21	10.31	18.1	14.94	21.19	11.1
217449	'Trappc12'	29.36	34.55	35.5	8.63	27.3	15.69	31.2	29.41	14.52	24.54
21745	'Tep1'	0.12	0.03	0.05	0.02	0.33	0.91	0.1	0.06	0.11	0.11
217463	'Snx13'	11.29	6.31	12.7	4.11	10	6.83	2.43	6.8	13.32	10.14
217473	'Ankmy2'	30.07	24.09	25.2	20.2	26.7	33.04	17.9	24.67	30.97	15.82
217480	'Dgkb'	14.99	22.67	13	38.5	4.88	11.04	9.22	14.69	7.8	8.89
21749	'Terf1'	8.8	12.4	10.5	11.4	17	5.66	8.01	11.45	24.71	15.69
21750	'Terf2'	11.57	6.18	6.58	4.43	5	7.31	4.91	2.58	11.25	3.71
217517	'Stxbp6'	0.96	1.41	0	0.97	8.25	2.16	11.5	7.4	0.68	0.02
21752	'Tert'	0.73	1.57	1.38	0.85	0.42	0.49	2.3	1.59	1.65	2.02
21753	'Tes'	0	1.68	0.33	0	3.67	0.56	0	1.91	3.07	0.54
21754	'Tesk1'	5.05	6.17	6.87	15.1	6.78	1.92	7	3.41	3.78	9.12
217558	'G2e3'	1.2	0.62	0.55	0.02	0.05	0	1.78	1.25	0.88	0.99
217578	'Baz1a'	0	0	0	0	0	0.01	0	0	0	0
217588	'Mbip'	11.82	12.53	19.1	16.5	25	27.36	16.1	12.18	21.21	17.73
21761	'Morf4l1'	281.5	290.5	318	449	316	346.5	254	420.3	452.9	359.18
217615	'217615'	4.32	0.94	5.66	3.77	4.11	2.43	5.54	2.19	9.49	1.85
21762	'Psmd2'	131.8	95.7	124	181	87.6	123.3	97.3	117.8	110	138.51
21763	'Tex2'	20	8.65	7.61	4.73	8.48	3.9	11.8	10.85	10.22	10.62
21766	'Tex261'	24.37	25.12	23.4	25.2	52.8	38.62	44.1	37.08	26.55	21.71
217664	'Mgat2'	11.89	8.72	3.69	32.1	8.59	11	3.21	11.87	7.65	10.12
217666	'L2hgdh'	10.76	5.28	6.37	18.5	8.17	4.01	13.7	13.21	15.89	10.71
21767	'Tex264'	44.55	56.54	56	26.7	68.2	31.82	81.7	59.82	37.15	38.48
217682	'Plekhd1'	0	0.46	0	0	1.88	1.02	0	0	0	0.8
217684	'Susd6'	1.73	0.24	0.5	3.75	1.58	0.01	3.5	0	0.01	0
21769	'Zfand3'	2.77	12.96	13.6	6.96	11.5	1.44	14.1	4.49	7.45	11.31
217692	'Sipa1l1'	5.53	6.07	7.09	1.71	4.19	5.92	2.63	4.05	4.29	7.84
217695	'Zfyve1'	9.51	13.98	10.8	10.7	8.9	3.77	2.64	20.91	3.36	12.36
217698	'Acot5'	0	0	2.25	0	2.39	0	0	0	0	0
21770	'Ppp2r5d'	17.36	20.78	32.7	34.3	28	6.23	30.1	39.57	39.45	13.71
217700	'Acot6'	0.32	0.56	0.8	0	0	0	0	0	2.86	0.92
217705	'Fam161b'	2.59	10.29	9.14	9.52	8.3	2.45	12.3	6.35	2.48	2.71
217707	'Coq6'	28.52	30.32	36.5	53.6	37.7	60.41	21.5	37.72	16.37	56.58
217708	'Lin52'	46.36	35.68	24.2	24.4	35	42.77	25.7	48.77	49.66	47.06
21771	'Utp4'	16.22	30.36	19.4	5.46	15.5	15.47	26.4	25.78	33.93	27.56
217715	'Eif2b2'	36.57	54.64	47.7	62.4	49	51.25	26.5	44.86	40.39	46.56
217716	'Mlh3'	4.83	2.96	1.87	1.93	5.33	3.46	1.55	3.17	0.71	7.03
217718	'Nek9'	1.86	2.25	5.23	7.61	8.35	0.73	6.84	1.41	1.16	0.83
217732	'Cipc'	12.96	15.52	20.5	12.2	10.6	7.51	4.96	11.38	4.04	12.27
217733	'Tmem63c'	15.5	8.88	10.6	0.02	0.98	8.5	8.57	12.73	6.13	12.12
217734	'Pomt2'	6.64	2.79	7.64	0.01	10.2	1.24	9.8	4.79	3.33	5.77
217737	'Ahsa1'	212.5	235.6	134	134	140	131.6	109	155.6	180.2	200.72
217738	'Ism2'	0	0.97	0	0.12	0	0	0	0	0	0
217779	'Lysmd1'	7.23	11.28	22.1	0	16.7	12.15	13.6	8.21	2.67	16.36
21778	'Tex9'	10.74	7.31	5.96	14.5	8.24	8.5	4.44	6.11	2.92	11.43
21780	'Tfam'	14.71	9.99	15.6	15.2	12.2	10.65	8.66	9.94	7.72	12.43
21781	'Tfdp1'	8.99	16.68	8.72	5.87	24.4	10.99	9.79	12.79	5.41	8.51
217826	'Kcnk13'	1.67	0.33	0.58	11.3	0.08	4.1	2.59	0	6.59	3.03
217827	'Nrde2'	2.59	7.31	10.9	3.31	1.53	2.92	8.79	3.68	0.82	8.35
217830	'Dglucy'	12.08	11.56	3.9	0.02	9.08	7.63	4.8	4.56	0	7.79
217837	'Itpk1'	18.31	25.46	15.1	0.02	17.9	15.53	8.95	2.89	14.96	20.12
217843	'Unc79'	3.75	1.36	1.53	5.01	2.82	0.62	5.02	1.63	3.14	1.04
217845	'Ifi27l2b'	0.04	0.3	0	0.05	0	0	0	0	0	0.08
21785	'Tff2'	0	0	0	0	0	0.27	0	0	0	0

217864	'Rcor1'	0.83	1.17	1.45	0.46	0.91	0	2.19	0.02	2.79	0.47
217866	'Cdc42bpb'	6.58	2.95	4.15	3.41	6.4	4.51	1.72	3.23	1.79	9.73
217869	'Eif5'	81.83	71.13	63.7	102	80.9	78.34	88.4	105	100.5	88.89
21787	'Tfg'	86.45	112.8	101	60.2	55.3	93.52	75	70.96	85.8	107.7
21788	'Tfpi'	0.01	0	0.02	0	0	0	0.04	0	0	0
217882	'Cep170b'	5.72	6.93	3.43	13.5	13.8	7.68	12	1.64	9.91	8.09
217887	'Clba1'	14.1	19.1	23.2	19.9	8	15.26	13	10.98	13.97	7.81
21789	'Tfpi2'	0	0	0	0	0	0	0	0.03	0	0
217893	'Pacs2'	2.94	3.5	2.95	0.58	7.52	4.45	1.48	3.27	0.39	2.58
217935	'Wdr60'	8.8	8.86	4.22	7.13	6.19	10.51	7.87	3.07	12.47	9.94
217944	'Rapgef5'	2.28	3.36	4.42	1.16	0.92	1.57	5.23	4.96	1.54	3.79
217946	'Cdca7l'	1.35	0	0	0	0	0	0	1.27	0	0
217951	'Tmem196'	11.44	6.22	6.09	11	15.1	0.23	4.63	5.44	1.87	4.15
217980	'Larp4b'	8.32	10.28	12.1	17.9	13.9	10.38	10.3	7.98	11.89	10.64
217995	'Heatr1'	1.09	1.92	1.84	1.07	0.83	1.44	1.66	0.01	1.88	0.59
21802	'Tgfa'	1.85	3.42	11.9	0	1.2	0	16	2.24	0.02	0
21803	'Tgfb1'	0	0	0	0	0	0	0.39	0	0.1	0.08
218030	'Pou6f2'	0	6.81	0.02	0	0	0	0.07	8.77	0.83	0
218035	'Vps41'	66.6	83.13	101	101	104	84.01	122	110.2	116.4	114.93
218038	'Amph'	27.03	18.54	33.9	15	43.7	35.95	54.1	22.98	14.65	23.37
21804	'Tgfb1i1'	8.83	0.03	2.35	0.95	7.82	4.1	11.5	1.31	4.39	4.88
21807	'Tsc22d1'	454.9	440.7	505	828	430	470.6	673	613	360.8	429.45
21808	'Tgfb2'	2.09	3.27	0.75	0.02	9.64	2.34	2.76	3.54	2.62	1.71
21809	'Tgfb3'	2.98	0.62	0	0.78	2.83	6.01	1.55	3.71	1.44	0
21810	'Tgfbi'	0.02	0.69	0	0.02	0	5.95	0	0	0	0
218100	'Zfp322a'	6.57	4.81	2.97	3.35	6.6	1.91	6.74	7.92	4.9	5.89
21812	'Tgfbr1'	0.41	0.08	0.33	2.59	1.19	3.02	1.6	0.19	2.03	0.05
218121	'Mboat1'	0	0.77	0	0	1.66	4.5	0	0	0	0
21813	'Tgfbr2'	0	0	0	0	0	0.01	0	0	0.15	0
218138	'Gmds'	13.21	33.36	19.4	3.48	28.7	9.35	17.5	27.21	6.94	9.41
21814	'Tgfbr3'	1.23	0.33	2.51	0	2.08	0.96	1.54	0.67	1.92	0.6
21815	'Tgif1'	0	0	0	0	0	5.03	0	3.95	0	0
21817	'Tgm2'	0	0	1.63	0	0	0.44	0	0.01	0	0.01
21818	'Tgm3'	0	0.13	0	0	0	0.16	0	0	0.02	0
218194	'Phactr1'	18.81	27.83	16.5	29.9	25.6	26.23	16.5	23.4	9.14	28.55
218203	'Mylip'	3.62	6.26	2.44	2.7	0.74	1.85	6.37	0.7	0.01	0.06
21821	'Ift88'	3.54	5.48	7.82	4.1	9.65	7.72	14.4	5.71	9.43	9.05
218210	'Nup153'	0.05	2.03	0.22	0.56	0.21	2.69	2.96	0.68	0.03	0.98
218214	'Kdm1b'	0.06	1.79	1.42	0.16	0.68	0	1.61	0.37	1.25	0.09
218215	'Rnf144b'	2.29	0.05	1.98	1.17	0.77	0.64	0.05	2.07	0.17	0.32
21823	'Th'	52.1	157.9	4.08	263	5.95	0.1	0.2	0.07	55.1	0.12
218232	'Ptpdc1'	1.33	11.58	1.91	16.4	5.98	5.11	4.42	4.6	5.9	2.84
218236	'Fam120a'	8.14	11.56	5.92	11.9	8.47	2.08	5.67	7.24	10.18	11.28
21824	'Thbd'	0	0	0	0	0	3.82	0	0	0.4	0
21825	'Thbs1'	0	0.4	0	0	0	0	0	0	0	1.82
21826	'Thbs2'	0	1.08	0	0.02	0	0.03	0	0	7.74	5
21827	'Thbs3'	6.48	5.23	3.76	4.69	3.87	0.21	11	3.87	4.95	0.37
218271	'B4galt7'	25.72	16.73	22	16.9	20.8	7.17	28.1	20.16	12.79	16.77
21828	'Thbs4'	18.57	0	0	2.95	0	0	0	0	0	0
218294	'Cdc14b'	3.19	6.49	1.59	3.16	2.21	1.03	4.4	1.47	1.84	3.26
218304	'Prss47'	0	0	0	2.14	0	0	0	0	0	0
218311	'Zfp455'	1.74	4.31	2.29	0	0	0.02	0	0.01	0.19	0
218314	'Zfp595'	0	0	0.04	0	1.83	0	0	0	0	0
21832	'Thpo'	1.46	0	0	0.03	3.94	0	10.9	4.52	0.02	0.83
21833	'Thra'	44.02	30.14	37.7	79.9	51.7	27.92	52.9	31.24	29.37	27.64
218333	'Ice1'	1.08	1.96	1.52	3.95	4.42	4.68	2.99	1.22	3.67	1.96
218335	'Clptm1l'	19.46	13.69	9.55	41.2	20.6	19.49	18.3	12.77	14.09	4.76
21834	'Thrb'	5.74	4.65	5.3	4.93	3.09	3.86	8.03	1.52	1.98	4.85

218341	'Rfesd'	4.3	12.78	10.5	5.83	13.9	18.37	9.32	15	12.43	9.66
218343	'Ttc37'	1.35	1.17	2.72	0.63	0.05	0.52	0.91	0.99	2.86	0.59
21835	'Thrsp'	15.48	13.68	0	0.05	1.7	40.78	33.5	4.69	23.61	40.29
21838	'Thy1'	45.19	30.33	77.4	78.4	67.1	35.34	55.8	71.1	53.21	17.54
218397	'Rasa1'	0.38	7.99	3.74	2.79	1.71	4.13	2.22	1.9	0.13	3.09
21841	'Tia1'	26.06	36.04	32.5	11.4	30.6	26.72	30.3	32.79	40.91	40.91
21843	'Tial1'	22.52	13.46	14.3	30.6	23.6	14.27	7.6	18.38	26.03	20.48
21844	'Tiam1'	3.64	3.74	5.03	3.12	2.36	1.51	3.57	0.9	1.75	0.71
218440	'Ankrd34b'	3.49	4.11	8.05	0.34	4	5.13	3.53	4.72	5.04	3.81
218441	'Zfyve16'	4.62	4.15	3.44	3.45	2.73	4.78	5.81	5.05	3.79	2.63
218442	'Serinc5'	4.39	2.77	10.6	0.19	0.17	0.24	6.15	4.41	0.15	0.09
218454	'Lhfpl2'	5.32	1.61	0.6	0	4.34	0.7	0.62	0	0.01	4.72
218460	'Wdr41'	11.85	18.44	27.4	7.96	19	27.09	24.9	8.76	13.45	9.27
218461	'Pde8b'	8.18	9.37	6.46	5.29	9.67	14.63	8.44	11.19	13.41	17.96
21847	'Klf10'	4.22	1.93	2.37	11.6	3.81	0.13	0.02	2.57	0	2.28
218476	'Gcnt4'	0	0.09	0	0	0	0	0	0	0	0
21848	'Trim24'	0.03	0.12	1.58	3.27	0.34	0.05	3.44	0.7	0.51	0.61
21849	'Trim28'	19.5	13.6	30	42.9	17.5	11.73	19	17.63	25.75	6.87
218490	'Btf3'	165.9	208.5	254	158	158	201.6	236	156.6	148.4	190.72
218503	'Fcho2'	2.47	0.89	0.53	0.09	0.03	0.66	1.62	0.82	0.83	2.45
218506	'Mrps27'	36.13	24.83	26.7	14.8	24.5	27.22	34.4	30.78	40.9	43.61
218518	'Marveld2'	2.54	0	0	6.17	0	0	0	0.43	1.69	4.57
21853	'Timeless'	0.03	0.03	1.26	0.02	0.12	0.03	0.04	0.05	0.05	0.91
21854	'Timm17a'	251.7	228.5	231	211	272	370.6	217	268.4	213.5	267.59
218543	'Srek1'	21.95	28.73	13.7	24.7	22.2	24.23	20.8	22.74	22.78	22.3
218544	'Sgtb'	11.29	16.46	21.9	22.1	16.2	10.89	20.2	10.56	37.21	14.7
21855	'Timm17b'	34.3	76.4	52.5	17.5	57.9	37.13	26.3	41.74	51.15	57.83
21856	'Timm44'	27.04	23.42	49.2	46	59.6	44.99	18.9	40.57	38.12	40.56
21858	'Timp2'	62.25	47.17	46.5	55.4	67.1	51.76	107	37.1	65.2	50.77
21859	'Timp3'	12.54	20.47	12.6	24.2	3.25	7.54	40.6	9.6	21.46	2.18
218613	'Mier3'	3.49	3.01	3.42	3.68	1.99	0.5	2.02	1.25	1.46	3.34
218624	'Il31ra'	0.2	0.17	0.23	0.25	0.16	0.13	0.13	0.08	0.19	0.18
218629	'Dhx29'	6.46	4.82	3.66	4.62	5.54	5.59	5.32	6.61	7.02	9.74
218630	'Ccno'	0	0	1.6	0	0	0	0	0	0	0
218639	'Arl15'	8.77	4.02	4.28	2.81	10.8	0.67	6.65	3.44	2.01	9.25
21869	'Nkx2-1'	2.95	2.1	0.95	13.2	0.17	0.06	0	0.72	0.09	0.06
218693	'Paip1'	76.19	65.26	46.7	54	73	51.28	63	69.34	76.99	82.09
218695	'Gm10044'	0.76	0.75	0.31	1.41	0.04	0.01	0.62	0.47	0.82	0.45
218699	'Pxk'	21.92	12.62	24.9	16.3	19.6	21.01	4.43	24.7	23.23	9.3
21871	'Atp6v0a2'	6.36	6.51	16.9	13.8	3.83	3.22	8.43	8.5	15.16	4.62
21872	'Tjp1'	1.09	0.8	1.86	3.06	0.97	1.63	0.78	1.11	1.29	0.53
21873	'Tjp2'	3.29	1.31	1.52	6.72	1.21	1.05	1.18	0.04	0.28	0.01
218734	'3830406C13Rik'	13.09	15.62	10	18.7	19.3	10.53	16.7	19.29	22.07	15.79
218739	'Sntn'	0	0	0	0	0	0	0	0.63	0	0
218756	'Slc4a7'	4.88	1.39	4.23	5.98	2.44	0.39	0.3	5.42	7.71	4.26
218763	'Lrrc3b'	25.67	10.28	17.3	20.4	33	46.43	24.3	33.45	27.96	9.51
21877	'Tk1'	0	2.68	0	0	0.53	0	0	0.06	0	0
218772	'Rarb'	1.05	0.8	3.41	0	0.05	0	0	3.95	0	0
218793	'Ube2e2'	20.95	36.89	46.3	110	59.6	39.46	37.9	40.85	46.25	31.54
21881	'Tkt'	93.21	106	142	68.7	74.9	131.6	105	128.6	103.7	153.55
218811	'Sec24c'	15.9	12.78	16.7	0.23	13	7.89	17.3	20.2	7.51	12.35
218820	'Zfp503'	0	0	0	0	0.14	0	0	0	0	0
218832	'Polr3a'	8.97	4.69	6.79	6.85	8.87	4.74	3.48	2.04	6.06	11.21
21885	'Tle1'	0.77	5.39	3.85	5.1	6.48	5.49	0	0.17	3.1	9.89
218850	'Fam208a'	2.11	2.14	0.24	2.93	1.9	2.02	1.47	1.59	1.86	1.88
21886	'Tle2'	1.26	13.36	18.8	10.1	4.18	0.31	12	17.33	19.45	0.02
218865	'Chdh'	0	0.19	0.05	0	0	0	0	0	0	0.4
21887	'Tle3'	0.62	0.21	2.08	0.74	5.3	0.3	2.11	2.11	1.48	0.01

218877	'Sema3g'	0	0	0	0	0	0	0	0	3.07	0
21888	'Tle4'	11.71	13.96	5.31	2.13	9.27	12.38	4.66	3.22	14.83	14.63
218885	'Oxnad1'	9.8	15.32	14	7.02	11.1	0.03	2.89	10.85	0	16.81
218914	'Wapl'	4.76	7.36	3.41	11.7	7.98	4.67	3.99	6.16	5.77	4.49
21892	'Tll1'	0	0.18	0	0	0	0	0	0	0	0
21894	'Tln1'	1.27	1.17	0.19	1.25	1.2	0.04	0.85	0.53	0.41	1.37
218952	'Fermt2'	6.13	7.49	9.44	32.6	4.12	10.64	10.1	4.22	12.8	6.4
21897	'Tlr1'	0.01	0	0	0.02	0.01	0.01	0.01	0	0	0
218973	'Wdhd1'	1.58	1.47	0.05	7.22	0	2.23	2.38	2.54	0.12	0.09
218975	'Mapk1ip1l'	9.87	8.94	4.28	17	8.25	1.76	14.2	12.69	10.37	13.71
218977	'Dlgap5'	0.03	0.02	1.27	0.04	0.92	0.06	0	0.01	0	0.22
218989	'Tmem260'	2.74	2.26	3.2	5.99	2.64	1.91	0.12	1.39	1.57	1.38
219022	'Ttc5'	32.35	27.32	23.7	64.4	65.3	82.8	53.6	67.44	35.3	44.2
219024	'Pip4p1'	18.55	21.41	19.5	56.9	19	35.89	18.4	26.58	62.59	8.11
21907	'Nr2e1'	0.5	0.06	0	2.61	0	3.44	0	1.29	0	2.73
219072	'Haus4'	4.36	10.56	3.06	5.5	2.6	1.52	6.92	3.45	3.83	3.94
219094	'Khnyn'	4.58	10.54	8.77	8.26	6.82	9.57	2.63	5.66	17.1	17.55
219103	'Cenpj'	0.55	1.57	0.99	0	0.97	0	0	0.01	0	0
219105	'Zmym5'	18.67	16.86	6.91	16.4	3.06	13.19	14.3	16.23	23.41	25
219114	'Ska3'	0	3.86	1.24	0.13	0	2.58	0	0.02	0	1.12
21912	'Tspan7'	1163	817.5	1200	1080	964	917.4	1702	1151	633.2	827.79
219131	'Phf11a'	0	0	0	0	0.21	1.05	0	0	0	0
219132	'Phf11d'	0.05	0.02	0.02	0	2.09	2.45	0.02	0	0	0.05
219134	'Shisa2'	0.14	0	0	2.52	0	0	1.83	0	1.89	0
219135	'Mtmr6'	66.86	57.84	42.8	39.5	56.3	19.72	59.1	63.39	56.12	80.37
219140	'Spata13'	0.01	1.4	1.7	1.06	2.09	1.91	1.14	0.06	0.01	2.9
219148	'Fam167a'	0	0.28	3.28	0.29	0.55	0	0	2	0.08	0
219149	'Xkr6'	0.83	1.15	0.28	0	3.57	0.59	9.02	0.44	0.81	4.4
21915	'Dtymk'	45.66	56.26	33.1	79.8	56.7	38.6	85.8	15.59	35.35	69.42
219150	'Hmbox1'	1.45	0.18	2.31	10.4	0.97	1.55	4.33	3.37	4.83	1.89
219151	'Scara3'	0.06	0	0	0	0.44	0	0	0	6.11	0
219158	'Ccar2'	38.73	21.29	44.9	7.82	26.9	10.19	22.4	32.84	44.56	29.53
21916	'Tmod1'	7.4	15.68	4.44	13.7	4.73	1.38	1.59	2.98	4.06	0.8
21917	'Tmpo'	3.47	1.88	2.93	5.64	0.58	2.6	4.72	2.31	4.57	0.74
219170	'Fam216b'	0	0	0	0	0	0	0	0.05	0	0
219181	'Akap11'	11.57	9.82	17.5	9.49	10.2	8.9	11.7	5.11	12.92	6.31
219189	'Vwa8'	4.16	1.75	8.02	8.63	2.35	0.06	0.63	3.02	2.92	0.25
21922	'Clec3b'	0	0.65	2.26	0	0.36	0	0	0	0	0
219228	'Pcdh17'	23.49	20.99	21.4	19	6.55	13.62	11.3	35.82	23.64	28.03
21924	'Tnnc1'	12.73	12.31	15.6	2.18	33.2	11.27	19.7	13.26	18.52	16.67
219249	'Tdrd3'	6.32	0.98	0.66	0.59	5.81	3.35	8.12	5.96	0.59	0.58
219257	'Pcdh20'	39.4	15.06	5.36	8.79	0.36	2.71	4.36	38.81	13.94	10.39
21926	'Tnf'	2.42	0	0	0	0	64.14	6.11	0	0	0
21927	'Tnfaip1'	15.14	19.94	21.1	25.9	8.23	32.99	9.04	24.92	17.6	22.92
21928	'Tnfaip2'	0.02	0	0	0	0.94	0	0	0	0.05	0
21929	'Tnfaip3'	0	0	0	0	0	0.6	0	1.35	0	0
21930	'Tnfaip6'	6.15	17.21	3.79	20.4	0.34	7.76	0	2.96	0.08	0
21933	'Tnfrsf10b'	1.7	2.27	3.44	3.29	2.87	3.13	4.37	1.4	3.27	1.82
21934	'Tnfrsf11a'	0.47	0.01	0.04	0.01	0	0	0	0.02	0.01	0
21935	'Tnfrsf17'	0	3.36	0	0	0	6.03	0	0	0	0
21936	'Tnfrsf18'	0.85	1.09	0.35	2.31	1.08	2.82	0.97	0.1	0	1.31
21937	'Tnfrsf1a'	3	0	3.52	0	0	0	0	3.03	0	2.33
21938	'Tnfrsf1b'	1.13	2.27	4.54	0	0	1.24	0	0.82	0.02	0
21939	'Cd40'	0	5.96	0	0	0	0	0	5.25	0	0
21940	'Cd27'	0	0	0	0	0	0	0	0.26	0	0.17
21941	'Tnfrsf8'	6.11	10.11	3.33	0	8.15	0	2.02	15.39	5.98	5.61
21942	'Tnfrsf9'	1.3	0	0	0.03	0	6.82	0	0	0	0
21944	'Tnfsf12'	0.87	0	1.64	0	0	0	0	0	0	0.15

21945	'Dedd'	19.42	16.03	17.7	16.9	11	25.52	16.1	15.14	14.79	8.62
21946	'Pglyrp1'	0	0.3	0	0	0	0	0	0.44	0	2.23
21948	'Cd70'	0	0.78	3	0	1.59	8.94	7.3	0.9	11.68	2.19
21950	'Tnfsf9'	2.49	0	1.88	18.4	0	5.14	0	0	4.4	13.75
21951	'Tnks'	1.18	0.61	1.53	5.4	0.35	0.82	0.36	0.53	2.19	0.42
21952	'Tnni1'	0.61	0.23	0	0	2.29	1.12	0	0	0	0
21954	'Tnni3'	5.37	0.92	18.3	4.33	4.39	5.78	5.99	2	5.67	9.84
21955	'Tnnt1'	0	0	0.1	0	0	0	0	1.21	0	0
21956	'Tnnt2'	6.22	9.91	32.2	7.41	4.63	5.74	1.96	17.3	42.47	17.6
21960	'Tnr'	4.55	2.33	8.72	7.65	5.22	1.67	1.35	6.44	12.59	6.66
21961	'Tns1'	0.01	1.49	0.85	0.02	0.86	0.01	1.19	0.16	0.47	0.08
21968	'Tom1'	8.74	26.51	43.6	12.4	34.4	30.24	45	41.18	46.09	39.92
21969	'Top1'	16.67	19.74	12.1	24.1	16	9.22	12.3	10.92	15.29	12.43
21973	'Top2a'	0	0	0	0	0	0	0	0	0.02	0
21974	'Top2b'	4.33	3.94	1.34	5.74	2.48	0.04	4.28	2.94	5.81	1.74
21975	'Top3a'	1.3	0	0.41	0.03	4.58	2.49	0	0	4.76	10.24
21976	'Top3b'	18.92	22.88	26.2	16.4	13.9	24.47	20.7	16.89	19.81	20.88
21981	'Ppp1r13b'	6.61	4.11	3.87	4.55	4.19	7.23	4.03	2.64	3.38	3.96
21982	'Tmem165'	3.31	1.88	4.23	0	6.49	0	13	2.74	1.63	0.88
21983	'Tpbg'	0.88	2.8	0.01	1.89	0.76	3.82	0	1.75	4.07	1.39
21985	'Tpd52'	32.77	38.37	24.9	80.8	45.3	31.27	13.5	24.78	51.47	22.5
21987	'Tpd52l1'	62.27	58.75	72.3	98.1	85.2	141.8	58.1	79.91	60.99	91.81
21991	'Tpi1'	705.1	695.6	810	389	622	759.2	1059	773.1	542	549.94
22003	'Tpm1'	76.26	69.18	78.2	54	46.6	61.82	76.2	71.59	58.8	46.62
22004	'Tpm2'	0	0	0	0	1.31	0	0	0.07	0	0
22017	'Tpmt'	8.86	2.93	2.31	2	3.03	7.73	5.39	6.17	0.18	2.67
22018	'Tpo'	0	1.41	0.01	0.07	5.83	0.01	0	2.33	3.26	0.18
22019	'Tpp2'	14.71	5.12	17.5	2.67	4.86	5.93	13.8	12.92	17.16	12.04
22021	'Tpst1'	9.48	9.89	10.1	9.02	12.9	5.05	4.89	8.18	3.31	0.73
22022	'Tpst2'	23.31	22.3	2.88	6.51	10.8	19.6	26.9	22.79	30.88	10.07
22025	'Nr2c1'	4.75	0.26	1.9	0	2.35	0.03	0	2.94	4.75	4.19
22026	'Nr2c2'	2.56	2.42	2.87	7.09	4.33	5.02	0.94	3.9	2.3	3.89
22027	'Hsp90b1'	271.7	258.4	151	181	202	193.6	190	209	316.7	342.96
22029	'Traf1'	0	0	0	1.27	3.19	0	0	0	3.57	0
22030	'Traf2'	7.21	7.72	3.9	0	6.09	3.63	0.02	11.08	5.53	13.72
22031	'Traf3'	2.26	2.51	1.53	4.51	6.78	3.27	0.76	0.01	3.02	2.55
22032	'Traf4'	1.52	0.93	9.96	6.26	0.1	0.01	16.5	0.76	4.72	0
22034	'Traf6'	0.04	0.7	2.3	3.13	1.97	0.01	0.91	0	2.05	2.33
22035	'Tnfsf10'	0.19	0.42	0.4	0.39	0.14	0.34	0.91	0.16	1.73	0.44
22036	'Traip'	14.98	12.44	7.28	5.77	13.4	30.2	22.9	18.84	2.73	12.83
22038	'Plscr1'	1.51	7.54	0	21.4	4.65	4.29	0.03	7.22	12.34	7.44
22040	'Trex1'	5.54	13.92	28.1	52.7	41.1	45.73	6.24	23.06	23.42	8.91
22041	'Trf'	86.56	158	2.92	21.3	119	365.1	5.78	15.05	0.29	37.16
22042	'Tfrc'	3.94	3.14	10.1	9.57	7.61	3.94	9.59	12.79	13.43	1.86
22044	'Trh'	23.27	1782	5.16	0.38	4.19	41.18	910	4.77	1.26	240.48
22045	'Trhr'	6.12	14.31	12.2	0	9.96	2.96	5.36	4.57	7.9	3.87
22051	'Trip6'	0	0	1.7	0	0	0	0.06	2.38	0.17	3.61
22057	'Tob1'	12.34	18.83	19.6	23.8	8.03	2.8	7.14	6.46	3.33	7.69
22059	'Trp53'	14.11	19.46	21.3	32	12.2	10.37	25.5	25.9	23.46	12.94
22061	'Trp63'	0	0	0.16	0	0	0	0	0	0	1.22
22062	'Trp73'	0	0	0	0	0	0.82	0	0	0	0
22063	'Trpc1'	1.95	1.39	1.38	6.18	0.01	0	1.06	1.06	3.16	0.01
22065	'Trpc3'	0	0.03	0.06	0	0	1.17	0	1.37	1.19	0.12
22066	'Trpc4'	3.71	5.45	3.21	0.16	3.51	4.61	0.02	2.67	0	0
22067	'Trpc5'	2.57	2.84	6.38	1.57	5.31	3.23	7.37	4.15	9.56	7.35
22068	'Trpc6'	0.65	0.28	0.2	0.3	0.31	0.39	0.15	0.59	3.15	1.96
22070	'Tpt1'	422.6	386	324	285	348	492	350	328.7	363.2	364.86
22083	'Ctr9'	11.8	15.81	18.3	37.4	15.7	11.28	21	13.81	6.19	13.69

22084	'Tsc2'	4.04	6.54	12.7	7.63	5.16	3.19	11.1	7.75	6.62	11.66
22088	'Tsg101'	143	131.3	127	88.3	87.6	93.38	122	125	154.9	118.1
22092	'Rsph1'	25.19	19.14	0.04	15.9	75.5	10.3	12.7	12.64	18.81	26.18
22094	'Tshb'	6.25	7.05	9.74	11.8	11.5	8.42	7.89	5.45	9.64	6.48
22099	'Tsn'	69.61	78.32	74.4	67.1	77.8	73.64	51	83.48	58.85	64.72
22110	'Tspyl1'	140.2	169.5	132	90.4	123	73.58	88.2	115.5	125.6	91.23
22116	'Tsks'	0.22	0	0.16	0	1.11	0	0.28	0	1.79	0
22117	'Tst'	9.55	16.61	13.9	14.3	9.34	52.44	23.2	9.51	5.46	10.56
22121	'Rpl13a'	227.3	238	217	364	286	233.4	183	144.1	130.5	192.39
22122	'Tsta3'	40	44.22	58.2	38.2	46.1	56.17	91.8	42.77	58.11	60.2
22123	'Psmd3'	43.89	46.33	87.2	44.9	46.1	37.23	59.6	34.67	43.94	10.67
22129	'Ttc3'	212.6	213.4	250	188	185	140.4	251	186.5	263.6	203.4
22130	'Ttf1'	3.62	4.27	3.33	5.51	5.7	5.62	5.5	3.06	4.77	4.31
22134	'Tgoln1'	78.44	70.81	74.8	75.9	40.1	26.35	55	71.55	80.77	90.09
22137	'Ttk'	0.02	0	0	0	0	0	0	0	0	0
22138	'Ttn'	0.03	0	0.26	0.11	0.02	0.04	0.03	0.03	0.03	0.03
22139	'Ttr'	0	0	0	11.5	0	0	0	3.7	7.97	0.14
22141	'Tub'	9.43	11.75	13.8	19.7	12.7	5.11	8.37	14.19	5.2	10.18
22142	'Tuba1a'	1635	1632	2231	1538	1928	1981	1484	1896	1502	1901.53
22143	'Tuba1b'	2256	1823	2086	1181	1897	2540	2464	2400	1982	2270.08
22145	'Tuba4a'	191.8	165	318	106	163	304.8	209	216	156.2	188.42
22146	'Tuba1c'	0.06	1.95	0.23	0.54	0.22	0.05	0.21	0.12	0.05	0.15
22151	'Tubb2a'	872.3	700.4	817	504	879	942.2	800	878	593.1	680.74
22152	'Tubb3'	209.1	191.1	376	143	313	352.9	239	288.6	107.5	182.08
22153	'Tubb4a'	551.8	522.5	576	333	587	895.2	509	928.1	541.6	558.28
22154	'Tubb5'	288.5	250.3	304	225	336	307.5	303	318.1	278	242.37
22156	'Tuft1'	0	0.91	7.02	7.39	0	1.81	0	0	0	0.33
22158	'Tulp3'	0.75	1.43	2.92	4.17	5.24	2.29	0.26	0.29	6.39	2.51
22166	'Txn1'	160.4	219.4	200	211	158	273.5	162	177.6	200.9	216.22
22169	'Cmpk2'	15.09	12.16	25.1	34.2	21.2	12.06	28.4	27.18	17.99	15.17
22171	'Tyms'	16.04	18.09	22	29.5	23.6	23.44	20.5	14.45	30.7	18.18
22173	'Tyr'	2.51	3.63	2.25	5.14	2.44	3.2	2.88	1.51	2.35	2.21
22174	'Tyro3'	3.06	2.36	2.6	0	4.58	3.3	1.51	7.98	9.01	1.39
22177	'Tyrobp'	25.29	22.48	21.6	44.1	55	205.2	42.4	13.24	14.17	38.92
22178	'Tyrp1'	0	0	0	0	0	0	0	0	0	4.12
22183	'Zrsr1'	0.88	1.89	1.33	2.89	6.79	1.77	1.47	3.33	3.03	2.61
22184	'Zrsr2'	11.08	15.4	17.9	16.4	12.5	15.92	24.6	13.22	6.55	16.95
22185	'U2af2'	18.56	19.61	17.8	11.6	26.9	12.47	26.7	26.09	39.66	34.4
22186	'Uba52'	334.8	377.1	399	386	304	441.6	511	242.6	306.2	288.06
22187	'Ubb'	8536	8746	7058	####	9628	13192	7930	11020	10273	11620.2
22190	'Ubc'	1270	1393	1572	1345	1109	1688	1400	1253	1036	1516.2
22192	'Ube2m'	8.61	14.12	15.5	30.1	18.4	15.01	9.44	20.03	14.48	17.33
22193	'Ube2e3'	64.19	46.09	35.5	42	52.4	71.77	37.7	46.64	102.5	52.66
22194	'Ube2e1'	179.7	162.6	102	111	106	188.7	132	118.2	181.4	151.16
22195	'Ube2l3'	120.1	117.3	108	115	71.7	116.2	86.1	97.21	126	104.92
22196	'Ube2i'	149.8	146.7	101	97.3	112	142.8	178	137.1	179.2	156.12
22200	'Uba3'	33.55	30.8	50	61	67.4	45.58	38.2	68.85	35.03	47.11
22201	'Uba1'	89.27	105.1	123	71.4	65.6	47.41	82	74.09	85.72	83.58
22202	'Uba1y'	0.24	0.11	0.45	0.57	0.46	0.35	0.33	2.66	0.23	0.24
22209	'Ube2a'	48.63	41.47	38	17.3	36.1	32.72	14.2	47.89	17.11	29.39
22210	'Ube2b'	191.7	278.6	158	265	149	194.2	179	179.4	147.5	158.17
22213	'Ube2g2'	46.45	37.79	48.7	50.6	27.9	51.37	39.7	40.32	50.86	41.55
22214	'Ube2h'	12.48	23.21	15.5	26	12.5	4.4	9.05	16.84	13.25	13.69
22215	'Ube3a'	19.41	24.08	19.8	18	24.4	19.44	21.1	21.28	28.36	13.09
22217	'Usp12'	1.28	1.62	1.99	1.03	5.21	3.45	3.64	1.68	3.06	1.38
22218	'Sumo1'	283.8	328.4	200	363	275	260.9	388	275.1	266.8	301.6
22221	'Ubp1'	6.64	9.89	5.55	24.5	5.81	10.71	12.4	4.28	2.69	6.24
22222	'Ubr1'	8.31	8.14	5.71	5.33	6.96	1.55	19.1	16.69	11.52	16.88

22223	'Uchl1'	1076	1067	1067	777	1138	1303	1088	891.4	615.4	1016.81
22224	'Usp10'	31.54	33.43	29.2	31.1	23.5	15.01	55.3	24.18	38.18	27.45
22225	'Usp5'	150.1	159.6	180	107	135	142.2	162	168.5	165.5	158.47
22227	'Ucp1'	0	0	0	4.2	0	0	0	0	0	0
22228	'Ucp2'	44.06	40.07	28.4	72.5	49	60.83	51.2	13.4	16.41	93.96
22229	'Ucp3'	5.14	0	0.02	0	0	1.72	0	0	0	0
22230	'Ufd1'	116.6	96.42	105	160	120	137.1	121	112.6	118.7	139.6
22232	'Slc35a2'	31.94	25.42	15.9	58.6	25.8	33.41	25	24.74	38.02	36.34
22234	'Ugcg'	3.38	4.91	6.48	6.55	8.05	0.87	4.42	4.27	2.15	4.91
22235	'Ugdh'	15.48	2.77	24.9	21.3	2.97	4.01	18.6	13.94	18.65	6.79
22239	'Ugt8a'	2.72	3.52	3.11	0	3.26	4.23	2.94	1.61	1.08	0
22240	'Dpysl3'	20.21	12.87	18.6	7.57	16.6	20.89	22.8	16.11	12.34	7.61
22241	'Ulk1'	8.09	10.19	7.38	14	7.63	7.26	18.2	9.45	5.95	8.49
22242	'Umod'	0	0	0	0	0	0	0	0	6.3	0
22245	'Uck1'	78.13	70.35	66.8	39.2	68.8	107.1	72.1	57.03	74.58	90.04
22247	'Umps'	12.46	7.7	4.19	21.3	7.61	13.45	12.2	10.39	11.31	14.21
22248	'Unc119'	16.03	3.14	16.3	7.46	6	34.73	21.6	14.84	9.3	6.7
22249	'Unc13b'	1.51	1.44	0.04	1.61	2.34	0	0	0.66	0.01	1.2
22253	'Unc5c'	3.68	3.83	5.62	3.12	1.28	0.91	3.41	2.22	1.94	0.88
22255	'Uncx'	0.08	0.09	0	0	0.56	1.92	1.22	0	0	0
22256	'Ung'	0.11	0.12	2.4	0.26	0.05	0	0	0	4.7	0
22258	'Usp4'	40.5	33.31	24.3	57.1	26	8.75	35.7	29.56	57.58	33.57
22259	'Nr1h3'	0	0	2.17	0	3.26	0	0	0	0	0
22260	'Nr1h2'	16.07	17.49	24.5	11.5	20.4	24.96	26.9	18.11	13.63	19.02
22262	'Uox'	2.95	0	0	0	0	0	0	0	0	0
22264	'Prap1'	0	0.17	0	0	0	0	0	0	0	0.07
22268	'Upk1b'	1.9	0	0	0	2.72	0	0	0	0	0
22269	'Upk2'	3.55	0	0	0	0	0	0	0	0	0
22270	'Upk3a'	0	8.8	4.52	11.4	13.7	0	0	0	17.71	18.85
22271	'Upp1'	8.48	0	0	0	0	0	8.96	2.81	0.04	0
22272	'Uqcrq'	182.3	140.6	167	199	171	268.1	158	154.7	177.2	128.68
22273	'Uqcrc1'	481.9	373	669	547	531	751.7	704	568.1	555.8	585
22275	'Urod'	45.32	64.13	43.9	44.8	57	55.14	56.5	59.98	82.51	27.75
22276	'Uros'	54.53	49.55	29.9	11.9	37.8	68.34	38.4	54.36	47.1	70.53
22278	'Usf1'	49.49	56.41	77	40.1	95.5	49.39	62.7	91.74	61.05	49.48
22282	'Usf2'	5.29	5.21	14.3	0.02	6.32	2.1	19.5	3.45	4.48	2.97
22283	'Ush2a'	0	0	0	0	0	0.02	0	0	0	0
22284	'Usp9x'	15.74	11.53	9.32	12.8	16	10.25	9.48	14.11	10.48	14.61
22286	'Utf1'	0	0	0	0	0	0	0.04	0	0	0
22287	'Scgb1a1'	0	0	4.39	0	0	0	0	0	0	0
22288	'Utrn'	9.11	2.37	1.08	1.91	2.88	4.18	1.7	4.47	0	1.7
22289	'Kdm6a'	0.36	1.11	1.03	1.43	1.19	0.19	2.23	0.88	3.8	6.51
22290	'Uty'	3.54	3.7	0	0	0.14	0.43	3.7	2.76	2.36	6.36
22294	'Uxt'	5.19	3.53	38.9	0.11	6.3	14.36	2.02	6.37	4.5	9.8
22295	'Cdh23'	0.02	0	0	0.07	3.74	0.87	0	0	0.37	0
22305	'Vmn2r37'	0	0	0	0	0	0	0	0	0.01	0
22313	'Vmn2r104'	0	0.06	0	0	0	0	0	0	0	0
22317	'Vamp1'	38.31	16.15	102	15.4	49.8	65.2	116	68.84	26.16	19.33
22318	'Vamp2'	638.6	599.6	487	204	645	566.6	629	891	719.5	751.69
22319	'Vamp3'	8.1	5.47	15.9	49.5	6.96	11.18	14.7	9.57	10.11	8.86
22320	'Vamp8'	0.54	12.44	0	46	13.1	61.1	12.5	7.08	4.47	20.78
22321	'Vars'	12.59	6.02	19.1	9.64	11.9	3.62	10.1	9.37	22.41	6.44
223227	'Sox21'	0	0	0	0	0.12	0	0	0	0.01	0
22323	'Vasp'	1.64	3.85	3.9	3.71	8.6	4.03	3.64	5.63	18.8	9.66
22324	'Vav1'	0.04	0	0.01	0.03	0.02	1.41	3.84	0.03	0.02	0
22325	'Vav2'	0	0.97	2.2	0	0.33	0.16	0	0.02	0	0.08
223254	'Farp1'	1.91	3.38	7.25	10.1	3.24	4.74	0.11	2.41	5.78	3.67
223255	'Stk24'	3.97	8.56	6.03	18.8	4.16	3.15	12.3	2.48	15.83	7.95

22326	'Vax1'	0.79	0.39	0.71	0	2.24	0.57	0.91	0.44	0	1.64
223267	'Ggact'	31.36	33.77	31.7	10.4	17.7	19.25	22	49.82	24.46	14.54
22327	'Vbp1'	150.4	190.2	105	101	135	139.7	123	145.9	90.98	155.11
223272	'Itgbl1'	0	0	0.51	1.1	0	0	0	0.6	2.64	0.33
22329	'Vcam1'	7.12	0.54	2.13	2.75	1.45	10.46	0	2.61	0	0
22330	'Vcl'	2.52	0.51	1.83	3.63	2.67	0	1.77	2.82	2.12	3.11
22333	'Vdac1'	272.8	203.5	294	145	296	301.1	352	241.3	222	166.46
223332	'Ranbp3l'	1.1	0.12	0.23	0.18	0.16	0.16	0.13	0.22	0.18	0.12
22334	'Vdac2'	181.5	205.9	137	180	191	191.9	209	224.9	213.9	211.14
22335	'Vdac3'	458.7	391.3	340	352	378	538.6	428	452.3	338.1	339.21
22337	'Vdr'	0	0	0	0.41	0	0	0	0	0	0
22339	'Vegfa'	0.21	0.49	0.69	3.11	2.84	1.63	1.09	0.95	0.11	1.1
22340	'Vegfb'	18.21	8.61	7.61	4.26	30.4	5.6	10.9	13.99	10.25	12.35
22341	'Vegfc'	0	0.1	0.18	0	0	0	0	0	0	0
22342	'Lin7b'	124.8	69.67	139	216	102	147.2	69.7	103.4	102.5	91.77
22343	'Lin7c'	17.86	14.94	12.1	11.4	11.6	1.09	14	8.7	14.13	22.43
223433	'Otulinl'	2.92	3.45	1.79	0	9.54	2.86	0.02	4.84	0	1.31
223435	'Trio'	0.85	1.2	0.25	2.11	1.38	0.34	0.54	1.61	1.01	1.67
22344	'Vezf1'	6.24	6.78	12	9.13	5.19	6.07	8.58	4.82	3.96	4.8
223453	'Dap'	1.9	3.52	3.72	7.65	6	24.32	0.72	0.03	24.1	12.32
223455	'March6'	8.69	6.69	12.7	11.3	8.33	7.07	20.6	8.12	11.34	19.53
22346	'Vhl'	26.09	10.15	11.7	31.6	14.6	28.03	14.1	12.25	29.28	6.47
223473	'Nipal2'	5	3.1	4.64	7.56	6.3	1.22	8.02	12.07	4.18	3.65
22348	'Slc32a1'	5.76	1.07	2.81	0.08	11.6	1.92	0.16	2.88	3.36	1.16
22349	'Vil1'	0	0.05	0.02	0	0	0	0	0	0.23	0
223499	'Dcaf13'	8.59	26.63	32.8	24.2	29.1	32.37	28.4	31.73	23.83	34.56
22350	'Ezr'	12.02	6.54	0.09	18.2	13.3	3.23	2.07	0.05	0.59	2.15
22351	'Vill'	0	2.02	0	0	7.64	0	0	4.53	3.51	0.02
223513	'Abra'	0.03	0	0.08	0.03	0	0.03	0	0.05	0	0.02
22352	'Vim'	3.96	0	0.13	0.03	32.8	0	3.29	1.56	0.42	7.71
223527	'Eny2'	42.99	36.83	34.7	11.8	22.2	29.48	23.4	29.34	25.43	32.45
22353	'Vip'	0	0	1.79	0	0	0.42	4.6	85.08	9.61	0.03
22354	'Vipr1'	0	0	0	0	0	0	0	0	0.21	0
22355	'Vipr2'	4.43	0	0.1	0	0	1.75	0	0	0.03	0
22359	'Vldlr'	6.15	12.78	13.2	7.09	8.79	3.59	3.76	5.36	4.96	3.98
223593	'Washc5'	4.61	11.94	12	9.14	15.1	1.68	7.18	10.61	14.04	8.85
22360	'Nrsn1'	471.1	279.2	223	424	256	234.3	298	387.9	147.9	236.03
223601	'Fam49b'	26.36	16.71	28.9	20.9	22.2	14.41	30.6	26.05	33.17	21.38
223604	'Kcnk9'	0.37	0	2.28	0.97	1.79	0.56	0.76	0.41	3.35	1.96
22361	'Vnn1'	0	0	2.58	0	0	0	0	0	0	0
223626	'Them6'	0	1.18	0.16	0	2.88	3.27	4.57	0.41	2.19	0.23
22364	'Vpreb3'	0	0	0	0	0.97	0	0	0	0	1.88
223642	'Zc3h3'	19.78	8.43	11.9	2.69	7.32	5.73	9.69	7.23	4.11	21.92
223646	'Naprt'	4.66	5.56	6.23	19.5	4.92	11.95	6.22	1.85	7.79	0
223648	'Ccdc166'	9.48	20.25	17.6	21.2	15.1	10.31	18.1	18.83	8.63	16.36
223649	'Nrbp2'	18.37	8.47	5.52	23.8	27.4	4.89	7.34	3.16	4.19	2.6
22365	'Vps45'	42.92	35.73	28	25.4	28.9	33.87	45.8	21.93	35.79	25.02
223650	'Eppk1'	0.26	0.21	0	0	0	0	0	0.06	0	0.08
223658	'Mroh1'	5.44	4.19	12.4	8.04	15.1	3.55	10.2	6.09	9.32	9.02
223664	'Lrrc14'	30.17	20.96	15.2	4.74	20	23.58	37.9	12.95	10.51	4.75
223665	'C030006K11Rik'	3.9	2.71	9.5	0.38	18.8	9.07	12.1	4.14	4.83	5.07
223666	'Arhgap39'	0.27	1.83	3.29	2.92	2.9	0.28	0.38	0.11	4.04	0.55
223669	'Zfp7'	1.19	0.43	9.72	0	12.7	3.36	2.4	5.49	4.04	0.05
22367	'Vrk1'	2.4	7.94	16.8	7.88	5.4	15.96	14.7	8.82	2.83	3.19
223672	'Apol9a'	0	2.38	0.03	0	0	0	0	3.92	1.48	4.64
22368	'Trpv2'	16.78	31.48	22.9	16.6	11.7	22.94	15.4	20.88	16.96	26.95
223690	'Ankrd54'	37.42	31.82	31.5	61.2	17.3	13.65	40.9	19.56	33.69	13.32
223691	'Eif3l'	38.84	74.89	60.9	63.9	61.6	58.27	67.3	84.38	57.62	87.28

223693	'Tmem184b'	11.27	15.97	24.5	5.29	12.1	6.48	12.8	8.7	15.17	9.66
223696	'Tomm22'	115.2	89.68	93.4	92	70.2	87.98	93	100.6	87.86	112.53
223697	'Sun2'	1.74	1.17	0.09	0	0.21	1.37	0	0.06	0	0
22370	'Vtn'	0	0	0	0	0	0	0	0	0.5	0
223701	'Mkl1'	0.3	0.58	6.47	5.47	3.96	5.91	5.05	2.51	6.05	2.31
223722	'Mcat'	1.98	4.71	5.02	0	2.22	4.94	0.26	9.71	9.78	0
223723	'Ttll12'	10.2	3.87	7.77	1.15	4.64	5.91	6.82	5.19	7.58	12.04
223726	'Mpped1'	16.8	2.96	4.09	6.73	20.9	8.51	24.6	5.52	0.03	29.95
223732	'Rtl6'	17.71	12.93	18.5	3.12	16.5	6.07	15.6	17.44	13	20.87
223739	'5031439G07Rik'	12.2	13.4	12.4	1.83	29.8	22.82	14.2	20.14	7.01	9.08
22375	'Wars'	57.24	57.16	80	9.79	64.5	43.1	66.2	66.49	35.44	37.39
223752	'Gramd4'	1.82	2.15	0.8	7.43	0	5.68	0	0.75	0	0.45
223753	'Cerk'	0.78	3.51	4.27	0.3	2.05	2.92	3.32	0.37	0.01	0.46
223754	'Tbc1d22a'	11.81	5.89	6.73	6.07	9.59	5.41	3.44	5.65	4.68	7.62
22376	'Was'	0.02	0	0	0.05	0.42	0	0	0	0.04	0
22377	'Wbp1'	30.32	9.24	13.9	13.6	55.2	9.63	9.27	10.8	22.2	9.46
223770	'Brd1'	6.04	4.63	5.59	5.87	8.17	3.19	3.11	4.24	5.37	7.22
223773	'Zbed4'	0	0.97	2.2	1.89	0	0	5.87	1.54	2.67	0.01
223774	'Alg12'	29.61	19.61	14	13.6	0	5.29	6.76	19.07	13.73	7.63
223775	'Pim3'	20.75	44.5	33.3	67.1	11.6	20.18	33.6	10.62	25.31	7.5
223776	'Selenoo'	5.43	2.23	10.3	15.3	7.04	0.96	4.8	4.76	4.53	0.67
22378	'Wbp2'	308.5	347.6	420	283	375	408.6	375	349.3	449.7	292.23
22379	'Fmnl3'	0	0	0.01	0	0	0.7	0.64	0.59	0	0.29
22380	'Wbp4'	11.62	15.74	11.3	22.8	15.1	15.71	19.8	22.74	19.52	14.82
22381	'Tceal9'	377.8	507.3	321	632	444	475	276	427.8	384.9	496.41
223827	'Gxylt1'	0.57	0.85	0.03	4.94	0.58	0.36	1.54	0.49	0.6	0.99
223828	'Pphln1'	23.93	9.81	6.8	28.6	7.38	1.67	12.3	15.78	25.4	14.03
223838	'Adamts20'	2.12	0.02	0.17	0.94	0.01	0	0	0	0.82	0
22384	'Eif4h'	207.9	217.1	224	173	173	212.7	272	199.4	191	190.16
223843	'Dbx2'	12.34	4.58	1.12	4.75	0.02	0.02	4.51	3.05	1.69	1.57
22385	'Baz1b'	7.61	10.9	8.81	15.6	7.66	4.86	5.21	5.05	6.16	4.99
223864	'Rapgef3'	25.31	17	21.5	0.16	14.2	12.52	16.4	10.65	8.39	13.96
223870	'Senp1'	0.24	1.99	1.32	9.43	3.27	2.34	2.17	1.63	0.33	2.41
22388	'Wdr1'	149.6	151.2	193	81.6	117	96.74	181	175.2	212.3	142.9
223881	'Rnd1'	31.12	20.12	23.3	33.4	24.4	35.18	32.8	28.22	12.15	9.71
22390	'Wee1'	2.92	6.45	3.34	4.09	5.35	0.16	0.89	4.01	2.88	10.25
223915	'Krt73'	1.04	2.09	2.57	9.38	0.02	0	0.02	0	0	0
223917	'Krt79'	0	0	0	0	0	0	0.07	0	0	0
223918	'Spryd3'	77.93	69.67	96.1	51.7	96.2	57.41	111	119	170	134.93
223920	'Soat2'	1.2	1.56	3.04	3.11	2.16	1.88	3.07	0.65	2.54	1.15
223921	'Aaas'	29.71	10.79	14.1	0	12.9	26.11	13.8	15.8	20.56	10.19
223922	'Atf7'	1.94	0.08	3.97	0.85	0.26	3.05	5.14	0.39	10.57	0.3
22393	'Wfs1'	39.57	58.9	29.2	40.7	11.5	19.33	31.4	25.74	9.84	27.64
223970	'Rmi2'	0.17	0.14	0.16	0.34	0.48	0.11	0.22	2.14	0.25	0.2
223978	'Cpped1'	7.84	6.88	10.4	0.83	0.83	7.89	0.21	3.8	18.84	5.42
223989	'Marf1'	37.38	25.6	16.7	12.3	41.3	29.65	22.8	46.46	50.51	41.38
224008	'Spidr'	0.34	0.63	1.72	0.2	0	0.63	0	0	0	0
22401	'Zmat3'	2.09	3.54	3.47	4.34	5.66	0.21	5.23	1.69	2.3	1.87
224014	'Fgd4'	6.87	5.83	4.24	5.33	4.99	0.48	1.75	0.82	4.96	4.07
224019	'Tmem191c'	63.63	51.49	62.9	77.9	84.7	88.66	43.6	62.43	60.85	54.72
22402	'Wisp1'	0	0	0.3	0	0	0	0	0	0	0
224020	'Pi4ka'	10.67	8.22	7.07	32.1	13.2	16.85	4.73	10.18	12.72	11.4
224022	'Slc7a4'	79.24	83.24	40.8	32	69.8	86.34	79.4	68.98	60.39	101.53
224023	'Klhl22'	54.62	45.38	46.2	43.2	33.5	18.02	92.6	62.03	73.9	46.5
224024	'Scarf2'	0.01	0.01	0.23	1.6	0.5	0	0	0	1.26	0
22404	'Wiz'	0.43	1.17	1.88	0.27	3.11	0.01	7.98	1.26	4.86	2.03
224045	'Eif2b5'	2.47	4.65	16.1	13.8	7.93	7.27	3.37	7.85	31.41	1.62
224055	'Rtp2'	0	2.17	0	0	5.01	0	0	1.79	0	2.38

224079	'Atp13a4'	5.37	3.38	3.35	0	0	0	5.51	1.23	5.56	0
22408	'Wnt1'	0	0	0	0	0	0.02	0	0	0	0
224088	'Atp13a3'	0.23	1.25	0.2	3.67	1.27	0.62	1.41	0.68	0.71	0.93
22409	'Wnt10a'	1.55	0	0	0	0	0	0	2.23	0	0
224090	'Tmem44'	4.43	2.08	4.44	6.3	3.34	4.8	7.76	4.13	2.13	0
224092	'Lsg1'	12.76	22.01	18.6	3.9	29.4	10.11	14.9	23.47	7.08	12.3
224093	'Fam43a'	3.23	1.52	4.06	2.2	0	0	0	1.93	0.03	0
224105	'Pak2'	4.98	5.74	8.1	6.09	4.18	2.33	2.89	1.39	4.77	8.73
224109	'Nrros'	2.74	2.21	0.02	10.8	0	0	0	0	0	2.41
224111	'Ubxn7'	2.49	3.23	3.93	0.61	1.56	0.61	5.04	4.33	5.04	7.81
224116	'Muc20'	0	0	0	0	0	0	0	0	0.01	0
224129	'Adcy5'	4.44	3.25	6.23	0.5	2.52	4.96	1.85	0.92	3.57	2.35
22413	'Wnt2'	0	1.29	0	0	1.49	0	0	0.15	0	0
224132	'Dirc2'	1.88	2.01	2.6	9.14	0.11	0.46	0.02	2.9	1.7	2.66
224139	'Golgb1'	9.14	9.86	8.09	12.7	10.4	10.04	10	6.54	9.04	11.44
22414	'Wnt2b'	0.19	0	0.01	0	0.05	0	0	0.23	0	0
224143	'Poglut1'	58.96	54.34	81.3	50.7	46.9	51.18	76.8	43.93	103.8	49.42
22415	'Wnt3'	0	0.09	1.81	2.57	3.78	9.87	11	0.71	0.08	7.58
22417	'Wnt4'	4.42	4.47	0.73	0.15	0.29	4.1	0.12	3.45	2.23	2.01
224170	'Dzip3'	32.8	20.52	18.9	20.9	17.6	18.27	25.4	26.62	19.06	27.66
224171	'Cip2a'	1.84	0	0.02	0.46	0	0	0	0.43	0.02	0
22418	'Wnt5a'	0	2.1	0.03	0	3.2	0	0	0	0	1.18
22419	'Wnt5b'	1.79	0	0	0	8.44	0	4.56	0.02	0	5.43
22420	'Wnt6'	0	0.44	0	0	0	0	1.11	0	0	0.04
22421	'Wnt7a'	0.41	2.65	3.82	11.2	0	0	1.78	1.3	0	0
22422	'Wnt7b'	0	3.14	4.76	3.58	0.01	4.69	4.51	2.43	3.47	2.41
224224	'Impg2'	0	0.16	0	0	0	0	0	0.02	0.06	1.21
224250	'Cldnd1'	52.3	61.3	47.7	54.6	86.4	111.9	72.4	76.15	59.54	72.58
22427	'Wrn'	0.66	2.03	0.83	0	0.02	2.2	1.47	1.34	0.76	2.59
224273	'Crybg3'	0.53	0.67	0.62	0	0.53	0.06	0	0.43	0.32	0.39
22428	'Dctn6'	124.9	117.4	148	116	182	173.7	127	164.9	101.4	139.24
22433	'Xbp1'	121.2	124.5	98.3	156	128	155.7	135	83.88	200.1	83.91
224344	'Rbm11'	5.74	2.8	10.7	0.02	5.03	18.85	5.76	9.7	4.95	5.96
22437	'Xirp1'	0	0	0.03	0.01	0	0.01	0.01	0	0	0.01
22439	'Xk'	4.59	0.22	3.12	0	0.41	0	4.95	1.69	1.53	3.24
224405	'Cyyr1'	0	0	0.01	0	0	0	0	0	0	0
22441	'Xlr'	0	0.06	0	0.21	0	5.94	0	1.96	0	0
224432	'Scaf4'	3.72	2.9	5.51	6.17	1.96	2.73	4.34	3.02	2.1	2.81
224440	'Setd4'	10.07	7.4	3.06	2.39	6.83	1.39	3.51	6.49	3.84	4.61
22445	'Xlr3a'	5.64	23.73	7.59	0	5.19	0	17.4	5.39	0.04	0
224454	'Zdhhc14'	10.06	12.43	2.61	16.9	4.21	7.51	11.8	8.7	3.94	7.65
22446	'Xlr3c'	0.11	0	0.04	0	0	0	0	0	0.03	0.05
224480	'Nox3'	0	0	0	0	0.41	0	0	0	0	0
224481	'Tfb1m'	7.45	6.22	4.49	18.1	24.9	8.6	6.12	2.03	4.44	18.02
224530	'Acat3'	2.87	0.14	0.07	0.05	2.61	0	0.02	0.03	0	0.98
224576	'Vmn2r106'	0	0	0	0	0	0	0	0	0	0.11
224585	'Zfp160'	2.07	3.43	2.06	0	1.82	2.9	9.4	3.34	8.23	6.21
224598	'Zfp758'	4.22	3.63	2.96	0.19	1	2.79	3.64	1.31	2.81	0
224613	'Flywch1'	247.5	210	297	178	201	256.2	288	196.1	287.5	178.78
224617	'Tbc1d24'	3.2	2.96	2.75	3.92	4.25	1.62	1.53	3.36	3.89	1.95
224619	'Traf7'	14.86	14.01	20.3	12.3	14.3	5.27	23.1	11.72	10.43	7.7
224624	'Rab40c'	0.09	2.79	4.23	8.6	0.42	13.82	5.62	0.02	4.29	5.52
224630	'Bnip1'	41.98	20.23	27.1	28	17.3	46.61	50.2	34.02	15.74	26.76
224640	'Lemd2'	4.08	1.38	4.86	0	7.37	3.02	2.03	2.19	4.53	1.84
224647	'D17Wsu92e'	9.05	6.4	6.26	8.27	6.15	11.2	3.15	8.04	8	7
224648	'Uhrf1bp1'	1.22	0.53	0	0.38	2.74	3.28	0.04	1.51	1.74	0.41
224650	'Anks1'	2.72	0.74	2.48	0.54	1.58	1.53	4.88	2.97	0.06	0.54
224656	'Zfp523'	16.58	9.83	17.7	17	25	13.01	17.7	5.88	18.21	11.47

224661	'Slc26a8'	0.04	0.43	0.7	7.15	0.43	0	0	0	0	0.72
224671	'Btbd9'	10.14	8.48	13.1	18.8	13.3	9.77	20	9.11	10.33	7.7
224674	'Slc37a1'	1.91	5.73	5.04	0	3.67	3.94	0.05	2.49	10.16	15.1
224691	'Zfp472'	0.24	0	3.36	0	0	0	0	0.72	3.53	4.67
224694	'Zfp81'	3.3	6.43	6.97	3.13	8.22	0.61	0.02	1.28	1.2	0.09
224697	'Adamts10'	4.6	3.05	14.8	4.25	7.19	2.72	7.08	3.17	11.05	17.98
224703	'March2'	14.57	7.95	8.68	19.7	24.7	9.03	13.9	17.66	9.63	5.67
224705	'Vps52'	13.96	13.81	19.8	11.9	27.8	19.15	21.8	33.79	31.47	13.7
224727	'Bag6'	13.46	12.75	8.53	6.4	10.7	12.08	8.77	16.31	23.81	11.88
224742	'Abcf1'	21.6	21.22	20.2	45.9	28	25.22	36.4	24.6	21.59	23.94
224792	'Adgrf5'	0.01	0.67	0	0	0.2	1.32	0	0	0	0.61
224794	'Enpp4'	4.96	3.91	1.1	9.72	2.84	2.45	2.54	5.88	2.32	5.96
224796	'Clic5'	0.01	0	0.02	0	0.56	0.02	0.01	0.03	0.01	0.01
224805	'Aars2'	8.02	8.27	11.1	0.07	1.86	7.21	9.07	7.08	10.07	8.56
224807	'Tmem63b'	25.77	18.56	29.7	5.52	24.9	15.51	28.5	13.3	14.47	17.27
224813	'Lrrc73'	6.26	3.67	4.84	26.5	3.69	8.71	4.49	1.41	9.03	2.41
224814	'Abcc10'	3.55	3.52	9.85	9.55	3.39	4.41	6.5	3.98	6.58	2.06
224823	'Rrp36'	26.25	34.39	20.5	33.6	35.2	44.26	31.6	41.74	13.38	41.64
224824	'Pex6'	3.9	10.8	3.45	6.18	2.95	3.44	7.85	0.01	5.2	2.54
224826	'Ubr2'	1.38	5.66	3.49	7.47	2.78	3.45	2.72	1.75	8.56	3.22
224829	'Trerf1'	1.39	2.66	4.68	0.88	3.28	1.48	7.73	2.17	0.97	2.26
224836	'Usp49'	1.66	0.23	0.33	1.24	0.6	0.91	1.54	0.46	1.22	2.16
224840	'Treml4'	0.02	0	0	0	0.12	0	0	0	0	0
224860	'Plcl2'	5.66	0.88	0.3	0.5	1.6	1.93	4.25	3.79	7.14	1.3
224893	'Zfp959'	0.44	0.97	0.12	12.2	0.94	0.74	4.39	4.29	4.85	6.25
224897	'Dpp9'	11.88	4.8	9.15	7.1	5.79	8.94	11.2	7.12	15.07	10.52
224902	'Safb2'	23.98	15.46	16.4	33.3	30.9	16.26	25.5	8.95	19.22	17.89
224903	'Safb'	36.89	47.99	37.2	34.1	43.8	41.47	56.9	34	40.48	47.55
224904	'2410015M20Rik'	233.7	242.2	220	276	224	344.9	248	188.4	221.6	228.64
224907	'Dus3l'	52.79	44.37	44	2.74	83.1	72.26	72.5	57.68	36.23	48.58
224912	'Crb3'	0	2.05	0	0	7.76	1.88	1.36	0	0.66	0
224916	'Vmn2r120'	0	0.02	0	0	0.02	0	0	0	0	0
224938	'Pja2'	160.6	151.9	142	98	141	78.35	137	167.7	168.5	182.44
224997	'Dlgap1'	18.19	28.08	14.4	14.2	16.2	6.73	15.1	9.75	5.37	8.34
225010	'Lclat1'	10.34	3.81	5.62	0.54	3.64	1.47	6.37	5.4	7.81	2.77
225020	'Fez2'	20.97	34.43	34.3	58.9	26.6	21.87	44.9	22.82	14.48	19.05
225027	'Srsf7'	135.5	134.3	104	80.5	116	114.6	120	122.1	137	173.69
225028	'Map4k3'	4.42	4.28	1.23	0.01	1.03	0.37	3.39	4.8	0.51	3.2
225049	'Ttc7'	0.22	0	0.72	1.67	0.25	0.37	0.84	0	2.5	2.04
225055	'Fbxo11'	12.91	8.31	7.2	14.2	14.1	11	16.6	12.59	10.63	15.61
225115	'Svil'	0	0.35	0.44	0.96	0.02	0	0.79	1.45	0.72	0.17
225131	'Wac'	18.02	15.43	20.7	16.4	15.1	15.3	23.2	13.97	22.71	21.67
225160	'Thoc1'	15.94	20.05	25	13.5	15.2	16.07	4.59	17.68	13.47	22.2
225164	'Mib1'	3.21	3.21	2.76	4.76	2.85	1.79	1.06	3.86	3.04	2.54
225182	'Rbbp8'	1.5	1.35	0.67	1.96	0.06	1	2.9	1.7	2.03	1.67
225187	'Ankrd29'	2.04	0.2	11.5	1.86	0.82	7.65	4.07	5.03	6.97	3.68
225192	'Hrh4'	0	0	2.06	0	0	0	0	0	0.56	0
225207	'Zfp521'	3.9	2.94	0.15	0.02	7.76	2.5	11.7	0.2	2.63	9.29
225215	'Rsl24d1'	41.8	51.21	28.8	73.5	72.9	87.43	72.6	53.46	52.32	51.27
225266	'Klhl14'	0.06	0	2.3	0	0	0	0	0	0	1.91
225280	'Ino80c'	13.7	4.67	25.4	22.7	10.5	20.44	27.3	18.53	15.67	12.28
225283	'Rprd1a'	10.72	7.11	8.37	37.3	12.6	10.72	12.2	11.96	9.1	21.67
225288	'Fhod3'	1.46	0.57	1.45	5.63	0.55	0.18	3.06	0.65	0.47	0.27
225289	'AW554918'	1.01	0.03	1.61	0.21	0.01	2.63	0.03	1.18	2.49	0.77
225326	'Pik3c3'	14.91	15.6	10.5	13.8	15.1	11.3	16.1	12.66	16.77	19.74
225339	'Ammecr1l'	12	4.22	5.6	13.9	7.94	5.94	3.02	10.22	8.68	9.34
225341	'Lims2'	4.05	2.24	23.7	8.24	15.4	6.07	28.2	4.55	0	0.05
225348	'Wdr36'	13.38	13.47	13.4	3.77	5.38	9.68	12.7	21.47	5.5	19.89

225358	'Fam13b'	20	10.73	17.4	11.1	6.89	13.82	25	10.7	28.16	16.13
225362	'Reep2'	104.1	117.1	116	48.3	133	93.51	95.5	106.9	68.14	83.09
225363	'Etf1'	11.9	17.42	13.1	21.7	14	17	7.96	13.46	24.72	14.57
225372	'Apbb3'	59.39	40.52	39.1	52.9	34.8	62.84	13	53.47	37.9	16.64
225392	'Rell2'	1.23	6.32	15.3	6.24	14.8	2.25	14.1	10.36	29.48	6.25
225432	'Rbm27'	3.14	4.3	3.6	9.08	3.7	5.35	4.72	3.93	6.49	5.12
225467	'Pggt1b'	2.49	10.03	3.55	2.36	0.47	7.06	1.83	5.45	3.7	2.14
225471	'Ticam2'	0	0	0	0	0	0	0	1.66	0	0
225523	'Cep120'	9.14	4.41	10.2	10.8	4.87	1.22	8.25	4.2	16.41	13.94
225583	'A730017C20Rik'	345.5	344	228	305	202	419.1	248	331.2	309.9	381.34
225594	'Gm4841'	0	0.1	0.03	0.06	0.19	0.02	0.05	0.07	0.07	0.04
225600	'Pde6a'	0	0	0	0	0	0	0	0.37	0	0
225608	'Sh3tc2'	0	0	0	0	0.8	1	0	0	0	0
225631	'Onecut2'	0.14	0.13	0	0.07	1.91	1	1.07	0.27	0.86	0.93
225638	'Alpk2'	0.01	0	0.01	0	0	0	0.01	0.01	0.01	0.01
225642	'Grp'	14.62	0	4.64	0	9.38	67.09	2.53	0	0	54.95
225651	'Mppe1'	9.94	9.06	1.97	3.68	12.4	0.56	13	25.17	11.42	6.38
225655	'Prelid3a'	24.16	42.48	19.6	14.8	40	31.95	44.7	71.18	31.59	18.98
225659	'Cep76'	4.06	0.93	2.11	0.01	0.28	1.27	0.58	0.12	0.14	0
225724	'Mapk4'	8.77	12.67	13.2	5.24	9.61	5.54	3.17	0.03	13.92	1.92
225742	'St8sia5'	3.83	11.42	10.3	10.1	6.91	5.79	6.17	1.07	4.59	4.87
225743	'Rnf165'	2.12	1.32	1.77	4.31	0.74	0.89	1.44	2.73	1.45	0.21
225745	'Haus1'	9.91	9.42	4.82	23.1	22.8	5.75	0.03	3.93	12.98	13.18
225791	'Zadh2'	3.72	4.72	0.23	7.98	3.75	0.04	0.04	0	7.28	0.02
225825	'Cd226'	0	0	0	0	0	0	0	0	0.12	0
225845	'Pla2g16'	8.68	20	8.46	0.09	1.38	25.84	27.1	8.2	4.97	3.05
225849	'Ppp2r5b'	35.91	43.74	86.9	13.3	37.8	30.26	43.9	52.59	77.95	23.27
225861	'Snx32'	101.2	109	102	116	175	191.8	172	115.4	105.2	101.47
225870	'Rin1'	0.64	3.74	0.28	1.38	2.05	0.38	0.07	0.04	0.22	4.04
225872	'Npas4'	15.07	15.19	44.7	13	6.7	28.34	4.5	24.14	12.22	40.44
225875	'Lrfn4'	0.21	1.34	0.15	1.03	1.3	0.4	1.64	0.76	2.46	0.03
225876	'Kdm2a'	3.95	8.51	5.81	14.4	6.23	3.45	4.45	4.59	7.05	10.43
225887	'Ndufs8'	288.8	250.2	353	292	417	567.2	303	377.6	353.7	272.26
225888	'Kmt5b'	5.31	4.38	3.22	3.04	3.87	1.22	3.44	1.8	7.66	2.68
22589	'Atrx'	8.59	15.52	12.7	18.9	13.1	14.25	11.4	14.53	12.52	10.52
225895	'Taf6l'	8.83	13.73	33	0	17.1	18.8	35.6	16.62	26.05	23.21
225896	'Ubxn1'	55.21	80.83	92	123	116	99.08	71.4	61.27	72.81	75.07
225898	'Eml3'	0.89	1.25	1.88	0	4.88	1.19	5.98	2.78	20.26	11.56
22590	'Xpa'	8.67	14.15	21.1	2.16	16.5	28.23	8.97	7.88	0.54	11.86
225908	'Myrf'	0.19	0.53	0.38	0	0.05	0.57	1.44	0	0	0.01
22591	'Xpc'	4.24	3.59	4.64	6.91	1.67	0.01	17.2	11.19	7.44	3.66
225912	'Cyb561a3'	9.11	11.44	1.15	0.39	10.2	19.57	1.26	3.42	10.12	3.83
225913	'Tkfc'	12.66	9.42	9.43	0.05	2.78	1.56	2.74	7.76	12.27	8.46
22592	'Ercc5'	6.49	4.71	11	1.75	4.9	4.03	2.34	4.9	10.62	2.73
225922	'Oosp2'	1.27	0	0	0	0	0	0	0	0	0
225929	'Patl1'	2.67	0.65	5.59	1.07	2.76	3.53	2.72	3.5	6.3	5.99
22594	'Xrcc1'	11.39	23.36	12.8	14.6	20.6	23.57	23.7	20.44	13.5	9.03
22596	'Xrcc5'	20.07	12.99	13.4	19.9	20.4	15.63	10.1	10.47	6.87	13.22
22598	'Slc6a18'	0.12	0.17	0.14	0.61	0.15	0.19	0.29	0.11	0.25	0.12
22599	'Slc6a20b'	0.04	0.04	0	0	0	0	0	0.02	1	0
225994	'Nmrk1'	9.1	19.81	14.2	25.9	19.1	29.44	33.6	8.15	18.86	11.92
225995	'D030056L22Rik'	8.93	11.31	3.75	20.2	4.29	0.03	8.11	2.36	5.25	0
225997	'Trpm6'	0	1.17	0	2.07	0.3	0	0.7	0.76	0.63	0.82
225998	'Rorb'	15.97	10.89	4.76	10.2	0.7	4.32	0.61	8.75	13.87	7.55
22601	'Yap1'	0	2.45	0	0	0.02	0	0	0.98	0	0.58
226016	'Abhd17b'	0.64	4.31	1.99	9.55	4.95	0.02	2.03	0.19	1.6	1.85
226025	'Trpm3'	3.83	4.75	0.04	0.02	1.98	2.05	4.18	2.59	1.08	3.58
226026	'Smc5'	7.99	5.33	8.9	8.74	7.48	13.13	0.15	6.58	10.78	6.79

226040	'Tmem252'	0	0	1.48	0	0	0	0	0	0	0.02
226041	'Pgm5'	0.02	0	0.37	0	0	0	0.01	0	0.03	0
226043	'Cbwd1'	3.95	8.47	0.42	3.67	11.6	8.22	0.62	5.02	0.53	9.32
226075	'Glis3'	0	0.73	0	0	0	0.1	0	0	0	0.46
22608	'Ybx1'	15.55	14.36	11.5	57.2	12.2	8.11	13.2	6.48	11.71	5.11
226089	'Ric1'	0	0	1.97	0	0	0	0	1.04	0	0.23
226090	'Ermp1'	6.61	2.59	2.13	1.71	1.13	1.22	5.4	2.49	3.16	0.7
226098	'Hectd2'	0	0	1.26	1.68	0.01	0.79	0.44	0.89	1.98	1
226101	'Myof'	1.02	0	0	0	0	0.74	0	0	0	0
226115	'Opalin'	11.46	5.96	0	0	5.93	66.48	0.06	19.35	0	0
22612	'Yes1'	2.66	0.37	0.01	1.59	2.61	0.01	0	0.21	0.21	0
226122	'Ubtd1'	3.1	0	5.48	11.8	0.03	0	3.55	2.37	6.19	3.33
226123	'Morn4'	94.02	84.19	86	102	99.7	112.1	79.9	112.9	78.8	106.2
226139	'Cox15'	14.44	11.08	9.16	5.93	5.63	1.63	6.05	9.05	8.56	6.23
226143	'Cyp2c23'	0	0	0	0	0	0	0	0.57	0	0
226144	'Erlin1'	6.15	2.61	1.64	9.2	2.76	4.21	2.2	1.81	4.44	6.64
226151	'Slf2'	14.89	13.5	17.8	14	11	21.28	7.94	9.24	16.08	23.52
226153	'Twnk'	7.29	2.59	2.23	19.7	3.82	2.45	2.3	10.55	5.94	7.39
226154	'Lzts2'	1.62	2.37	9	6.11	7.82	0.54	5.36	2.04	8.14	0.03
226162	'Dpcd'	207.1	174.9	179	224	159	262.5	141	170.6	185.3	234.16
226169	'Pprc1'	4.32	2.82	2.18	8.56	0.66	0.78	6.02	4.22	3.23	1.1
226178	'Wbp1l'	6.19	8.34	4.31	0	12.6	1.14	0.05	8.7	15.61	7.11
226180	'Ina'	42.16	56.47	35.6	66.5	39.9	58.18	94.1	48.39	36.45	37.11
226182	'Taf5'	0.07	0.06	0.07	0	0.78	0.22	0.64	0.88	0	0.96
22619	'Siae'	10.06	5.14	4.18	7.14	6.22	8.68	8.12	11.28	22.32	7.81
22625	'Map3k19'	1.27	1.6	0.25	5.19	0.25	1.33	0.46	0.08	0.96	0.11
226250	'Afap1l2'	0	1.98	0.54	4.17	0.02	0	8	1.86	0	0.02
226251	'Ablim1'	3.11	3.66	7.08	3.87	7.31	6.41	9.99	7.48	3.31	1.61
226252	'Fam160b1'	0.01	0.46	0.95	5.67	6.34	1.04	0	1.4	1.04	3.82
226255	'Atrnl1'	1.42	0.54	0.9	0	0.02	0	1.01	0.99	0.04	0
22626	'Slc23a3'	0.01	0	0	0	0	0	0	0	0	0
226265	'Eno4'	0.56	4.58	2.39	3.03	9.3	1.94	2.31	1.57	1.63	0.87
22627	'Ywhae'	455.6	555.6	418	394	540	494.9	525	525	552.1	673.75
226278	'Prlhr'	0	2.19	0	0	0	0	0.03	1.01	3.81	0
22628	'Ywhag'	190	172.8	196	107	122	62.71	193	172.6	225.9	137.22
22629	'Ywhah'	715.2	601.2	520	811	579	684.3	578	598.8	720.1	475.9
22630	'Ywhaq'	631.8	672	532	583	655	698.2	504	691.8	635.2	659.96
226304	'Npbwr1'	1.72	3.51	3.53	4.18	0.01	0	0	2.69	7.81	0
22631	'Ywhaz'	593.6	644.4	503	410	579	446.1	500	620	537.2	644.33
22632	'Yy1'	1.78	3.81	3.67	10.5	3.06	0.77	0	1.93	1.03	3.86
22634	'Plagl1'	3.66	3.29	1.79	7.58	3.95	1.82	3.11	3.33	4.22	8.39
22635	'Zan'	0	0	1.11	0	0	0	0	0.68	0	0
226351	'Tmem185b'	11.29	23.07	11.9	2.12	6.8	14.6	4.95	17.04	0.9	16.1
226352	'Epb41l5'	5.09	2.19	2.94	5.65	3.91	0.54	2.23	3.35	5.93	6.79
226356	'Cfap221'	0	0	0	0	0	0	0	0	0	0.01
226359	'C1ql2'	11.27	0	187	266	0.05	0.05	15.3	26.23	0	14.32
22637	'Zap70'	0.74	9.21	0	0	6.63	9.03	9.03	3.23	3.45	7.46
22640	'Zfp1'	10.42	7.21	6.68	5.69	3.96	6.52	5.27	10.9	5.11	13.01
226407	'Rab3gap1'	30.55	37.05	20.7	14.4	26.9	9.7	33.3	21.97	44.84	45.9
226409	'Zranb3'	0.27	0.57	1.05	4.55	3.64	8.06	0.12	4.91	1.94	1.94
226412	'R3hdm1'	18.99	21.41	19.1	27.2	29.1	22.67	32.7	18.08	21.13	28.72
226414	'Dars'	72.73	61.43	34.2	42	35.4	21.36	50.4	65.57	37.95	53.78
226418	'Yod1'	4.34	3.34	1.17	3.3	1.98	0.04	2.84	1.47	0	0
226419	'Dyrk3'	2.55	3.05	3.92	47	0	0	0	0	3.2	0.94
22642	'Zbtb17'	3.9	9.79	9.62	15.8	6.24	8.44	4.41	10.33	11.07	4.11
226421	'Rab7b'	0	0	0	0	1.56	0	0	0	0	0
226422	'Rab29'	4.15	5.24	7.26	0	9.51	0	5.96	5.16	0	0
22643	'Zfp101'	0.02	0.94	1.8	0	0.13	0	0	2.7	0.9	1.61

226432	'Ipo9'	21.43	26.03	42.4	8.11	24.6	9.4	29.4	33.33	40.69	27.33
226438	'Igfn1'	0	0	0.02	0	0	0	0	0	0	0
226439	'Ascl5'	0	0.09	0	0	1.42	0	0	0	0	0
22644	'Rnf103'	6.37	7.15	2.89	9.86	4	4.37	0.77	1.56	3.23	2.31
226442	'Zfp281'	2.67	3.28	8.5	7.27	4.15	1.9	8.61	4.57	5.24	0.05
22646	'Zfp105'	6.32	8.64	16.9	2.35	0.34	32.52	0.05	5.41	2.44	7.02
226470	'Zbtb41'	1.76	0	0.01	0	0.33	0.08	0.13	0.39	1.22	0.24
22648	'Zfp11'	1.76	1.76	0.17	0.29	3.14	0.1	2.68	1.87	3.71	2.76
226499	'Odr4'	19.69	14.55	18.2	8.72	19.1	14.69	10.2	13.21	22.42	18.43
226517	'Smg7'	4.51	4.34	11.9	7.35	4.3	4.16	9.75	3.56	9.27	4.74
226518	'Nmnat2'	31.35	35.24	40.9	13.2	29.5	30.34	41.8	40.4	66.99	28.77
226519	'Lamc1'	0	0	0.72	1.84	0.03	0	0.12	0	1.39	0.5
226525	'Rasal2'	1.9	0.98	2.18	3.85	5.23	1.74	1.01	3.19	2.28	3.03
226527	'Cryzl2'	3.74	2.27	3.37	21.5	1.18	10.22	20.8	2.47	1.15	3.92
226539	'Dars2'	10.25	3.92	7.2	0.02	1.38	2.23	8.08	4.62	8.36	10.07
22654	'Zfp13'	14.32	11.97	9.84	0	2.73	1.6	0	15.96	14.24	12.22
226541	'Klhl20'	3.15	4.74	3.65	3.44	0.84	1.57	4.68	4.79	0	3.96
226548	'Aph1a'	26.56	14.03	10.1	0.08	10.3	25.96	15.9	20.68	14.88	6.49
226551	'Suco'	2.5	4.53	7.24	8.17	2.95	4.01	6.45	0.43	5.85	2.73
226562	'Prrc2c'	23.63	23.73	19.4	45.2	19	24.15	12.8	22.35	15.03	30.68
226564	'Fmo4'	0	0	0	0	2.02	0	0	0	0	0
22658	'Pcgf2'	0.9	2.38	3.02	2.89	2.88	0.14	2.51	0.89	2.91	1.68
226591	'Tiprl'	70.09	90.1	74.1	84	61.7	90.16	61.1	91.97	50.45	49.88
226594	'Rcsd1'	0	0	0	0	0	0	0	0	0.76	0
226601	'Gm4846'	0	0	0	0	0	0	0	0	0.02	0
22661	'Zfp148'	11.73	12.61	5.49	7.2	12.6	6.53	15.4	12.09	10.6	12.42
226610	'Fam78b'	0	0	3.36	2.56	1.39	0	0.38	0.61	0	0.08
226641	'Atf6'	0.97	1.76	4.67	6.8	4.26	2.42	1.04	2.68	1.79	2.88
226646	'Ndufs2'	224.1	154.6	196	197	203	248.3	265	197.3	189.3	175.57
226652	'Arhgap30'	0	0	0	3.59	0	0	0	0	0	0.27
226654	'Tstd1'	0.89	0	0.57	2.41	117	0	0.2	1.2	0.95	3.26
22666	'Zbtb14'	8.48	8.2	10.5	5.69	6.39	4.7	3.99	6.3	12.66	13.52
22668	'Sf1'	20.86	31.19	27.6	14.1	23.2	27.21	25.5	20.74	43.38	30.27
22670	'Trim26'	13.57	7.9	5.78	14.7	20.4	11.27	19.3	14.88	16.55	8.43
22671	'Rnf112'	21.07	43.63	66.4	10.3	27.1	16.34	42.2	24.27	29.48	15.26
226744	'Cnst'	7.05	6.74	6.09	1.88	1.56	4.21	6.37	10.63	8.32	1.29
226747	'Ahctf1'	2.11	0.75	1.66	4.37	3.33	0.59	1.98	1.61	1.02	1.95
226751	'Cdc42bpa'	5.35	5.39	5.02	14.1	11.3	6.64	11.5	7.39	5.57	8.63
226757	'Wdr26'	2.43	4.45	3.73	5.4	2.78	4.04	3.84	2.46	2.05	2.29
226777	'C130074G19Rik'	1.09	0	0	0	0.6	0	0	0.25	0	0
226778	'Mark1'	4.91	1.71	5.54	3.55	4.93	3.37	1.61	2.43	5.71	4.52
22678	'Zfp2'	10.71	6.85	6.75	3.07	12.8	5.87	15.7	14.02	9.56	11.94
226781	'Slc30a10'	4.23	4.04	2.81	0.48	2.09	4.81	1.38	1.73	2.07	3.41
226791	'Lyplal1'	12.38	9.3	12.5	0	12.9	22.32	18.2	14.7	4.04	2.98
22680	'Zfp207'	90.98	69.59	41.5	40.7	56.6	86.23	66	64.88	69.85	80.48
22682	'Zfand5'	17.41	19.54	8.89	47.6	15.8	15.4	6.49	8.33	12.96	6.74
226823	'Kctd3'	2.29	11.36	7.07	36.7	11.8	0.1	0.92	4.8	0.47	6.73
226830	'Smyd2'	73.15	74.88	91.4	104	87.8	77.39	67.2	86.41	89.97	50.52
226841	'Vash2'	0	1.07	0.05	8.82	1.44	4.14	1.37	2.39	0	1.16
226844	'Flvcr1'	0.01	2.31	0.06	7.65	1.02	0.49	3.94	0.5	0	0.98
226849	'Ppp2r5a'	2.44	0.12	0.02	0.39	0.99	0.01	2.33	0.12	1.97	2.96
22685	'Zfp239'	19.08	19.07	14.2	5.48	17.8	18.47	15.6	18.53	14.11	10.81
226856	'Lpgat1'	5.63	7.1	7.03	2.01	7.12	8.88	2.04	3.14	3.24	4.46
226861	'Hhat'	1.65	2.45	2.71	0	3.15	6.7	5.13	1.23	0.12	0.86
226866	'Sbspon'	0	1.41	0	0	2.49	0	0	0	1.47	0.22
22687	'Zpr1'	25.11	23.7	33.6	24.6	15.4	30.65	23.7	26.69	21.67	18.23
22688	'Zfp26'	1.74	0.41	2.07	7.39	0.54	0.14	2.33	0.87	1.09	0.53
22689	'Zfp27'	1.99	0.01	1.29	10	1.79	0.86	0.31	2.83	0.46	5.16

22690	'Zfp28'	9.55	2.72	6.52	11.1	9.07	10.18	4.94	9.95	12.89	4.67
22691	'Zscan2'	0.02	0.28	0.61	0.1	0.13	0.01	0.02	0.19	0.01	0.22
226922	'Kcnq5'	0	0.21	2.46	0	1.71	0	3.11	0.01	1.88	0.49
22693	'Zfp30'	1.97	0.48	9.91	13.3	8.84	6.26	8.65	14.31	10.93	8.75
22694	'Zfp35'	11.75	10.04	8.17	17.5	13.9	0.52	1.57	26.86	3.87	6.82
22695	'Zfp36'	29.07	39.13	5.07	29.1	12.6	133.1	0	0	3.33	1.41
22696	'Zfp37'	5.1	8.4	4.18	17.7	5.65	4.85	8.25	9.13	2.89	8.37
22697	'Zscan21'	12.48	16.26	0.85	9.06	18	11.32	21.7	11.6	32.39	16.17
226970	'Arhgef4'	122.6	64.42	81.4	41.8	69.7	75.52	117	115.8	90.38	106.79
226971	'Plekhb2'	256	204.2	224	125	188	168.1	226	268.9	299.7	301.15
226976	'Kansl3'	17.97	9.45	21.1	10.2	14.4	9.39	25.5	15.68	37.58	13.96
226977	'Actr1b'	121.5	125.4	144	99.8	141	121.7	202	167.6	72.43	134.85
22698	'Zfp39'	1.04	4.48	3.65	0	0.56	0.07	4.63	2.4	3.71	0.02
226982	'Eif5b'	11.81	7.69	6.58	12.8	9.56	9.54	7.07	7.19	5.28	6.35
226999	'Slc9a2'	0	0	0	0.03	0	0	0	0	0	0
22700	'Zfp40'	1.93	4.18	3.36	2.16	2.82	3.41	5.48	5.08	5.19	3.76
22701	'Zfp41'	0.97	4.35	2.49	6.28	3.26	0.01	0.77	2.78	3.37	2.71
22704	'Zfp46'	2.97	3.46	8.7	3.86	3.94	0.01	6.84	6.11	8.05	4.13
227058	'Dnah7b'	0.47	0.65	0.37	0.15	1.16	0.1	1.63	0	0.8	0.02
227059	'Slc39a10'	8.48	9.12	17.7	0.24	12.4	6.61	19.1	11.62	15.74	24.44
22709	'Zfp51'	0.47	7.65	0.19	5.67	2.1	6.47	1.01	4.12	6.67	4.32
227094	'Nemp2'	0	0.01	3.72	0	0.01	0.31	0	0.5	2.42	0
227095	'Hibch'	10.77	13.81	7.75	46.1	23.3	20.57	27.3	16.08	21.9	8.97
227099	'Pms1'	0.13	3.48	2.11	0	0.1	3.61	0	1.23	1.32	5.21
22710	'Zfp52'	1.79	6.61	3.87	6.45	0.91	3.16	2.99	3.22	2.85	3.79
227102	'Ormdl1'	34.48	31.08	28.3	47.3	32.6	59.71	12.9	21.51	40.01	36.13
22712	'Zfp54'	2.3	0.49	2.89	0	0.02	3.31	0	1.74	3.33	4.71
227120	'Plcl1'	5.31	0.47	2.33	0.72	1.57	0.48	1.08	2.29	1	0.99
22715	'Zfp57'	8.52	8.43	20	9.72	5.16	7.47	17.7	33.1	16.45	15.16
227154	'Stradb'	16.39	15.6	4.2	0	17.8	19.44	18.9	25.83	9.79	7.34
227157	'Mpp4'	0	0	0.02	0	0	0	0.09	0.03	0	0.42
22717	'Zfp59'	1.55	2.24	1.74	0.03	0.03	0	3.65	2.39	0.86	1.27
22718	'Zfp60'	15.74	11.7	12.5	13.8	12.6	8.06	20.3	15.89	14.93	9.56
22719	'Zfp61'	19.88	16.39	11.3	34.3	5.42	14.17	16.5	17.74	16.28	8.73
227195	'Ino80d'	1.01	0	0.86	0.05	0.08	3.25	5.11	1.27	2.39	3.15
227197	'Ndufs1'	100.8	94.95	104	163	71.5	81.88	149	101.5	82.85	109.15
22720	'Zfp62'	8.31	5.85	9.56	9.88	1.18	1.98	4.02	4.38	3.79	4.87
227210	'Ccnyl1'	0	0	0.03	0	0	0	0	2.1	4.26	0
22722	'Zfp64'	5.32	9.2	5.53	0	12.2	1.76	6.62	10.74	7.88	5.45
227231	'Cps1'	0	0	0	0	0	2	0	0	0	0.02
22724	'Zbtb7b'	0.12	0.08	0	0	2.6	0.04	0	0.42	0.02	0
227289	'Gpbar1'	0	0	0	0	0	0	0	0	0	1.57
227290	'Aamp'	266.7	305.8	188	275	239	365.4	275	331.9	296.8	291.17
227292	'Ctdsp1'	4.69	2.75	3.46	8.91	4.49	4.42	0.02	0.01	0.02	2.5
227298	'Retreg2'	43.95	19.71	16.2	53.3	41.8	36.78	54	47.9	27.41	24.13
227325	'Dner'	168.4	215.7	156	132	92.9	84.22	93.2	158.7	147.8	149.12
227326	'Gpr55'	1.64	4.36	0.72	3.36	0.87	0	0	0.38	3.17	0
227327	'B3gnt7'	2.82	3.29	0.35	1.37	6.02	0.21	5.79	2.82	4.99	2.71
227331	'Gigyf2'	8.36	6.83	8.19	6.48	5.43	2.22	7.16	3.73	5.76	5.94
227333	'Dgkd'	3.83	1.49	7.66	10.2	2.97	2.46	4.91	2.43	0.58	5.1
227334	'Usp40'	1.91	0.76	3.03	3.74	2.24	2.06	0.71	5.25	3.65	4.25
227357	'Espnl'	0	0	0	0	0	0.01	0.03	0	0	0
227358	'Erfe'	0	0	0	0	0	0	0	0	4.65	0
227377	'Farp2'	0.76	0.17	5.39	0.77	1.74	0.24	0	1.7	7.5	3.35
227394	'Slco4c1'	0.66	0.24	0	0	1.16	2.94	0	0.15	0.07	0.02
227399	'Ppip5k2'	9.47	3.58	8.72	11.1	5.89	2.35	2.32	7.26	7.3	10.18
227446	'Relch'	9.07	9.65	12	13.7	12.3	1.63	12.7	3.37	8.95	5.58
227449	'Zcchc2'	1.03	0.17	0.22	6.26	0.01	0.02	1.08	0.96	4.35	1.1

22746	'Zfp85'	0	0	0	0	2.79	0	0	3.94	4.51	1.75
227485	'Cdh19'	0	0	0	2.27	0.01	0	2.13	0.32	0	0
22750	'Zfp9'	6.62	1.69	9.1	10.2	5.52	4.95	12.8	1.62	4.92	3.04
22751	'Zfp90'	5.27	9.5	8.32	4.56	13.2	0.02	14	10.32	5.87	10.68
227522	'Rpp38'	73.24	74.03	73.9	67.8	101	145.3	87.2	126.6	100.7	103.57
227525	'Dclre1c'	7.64	9.34	13.6	25.6	11.2	14.38	11	9.74	21.19	11.39
227526	'Cdnf'	0	0	0.03	0.04	0	0.03	0.07	0	0.39	0.06
22754	'Zfp92'	11.84	6.51	3.66	1.39	6.02	0.35	2.86	8.97	7.57	3.92
227541	'Camk1d'	0.68	0.32	1.2	1.82	2.49	2.85	4.95	0.79	0.36	0.85
227545	'Proser2'	1.52	2.46	1.14	1.3	2.34	2.66	0.01	2.23	0.62	1.68
22755	'Zfp93'	0	2.12	0.11	0.01	3.18	0.7	1.19	4.32	2.44	1.3
22756	'Zfp94'	5.51	1.76	4.76	1.79	3.66	9.11	13.5	3.94	1.06	8.53
22757	'Zkscan5'	4.29	4.82	5.04	0	2.27	1.04	8.43	3.78	2.66	2.61
22758	'Zscan12'	1.41	0	0.94	0.16	1.32	0.45	0.07	0.11	0.01	1.23
227580	'C1ql3'	1.41	0	1.21	4.3	2	0.76	2.76	3.8	5.2	0.97
22759	'Zfp97'	2.93	2.26	0.06	0.04	10	4.85	4.11	0.56	1.62	1.33
22761	'Zfpm1'	0	0.17	0	0.01	0	0	0.24	0	1.64	0
227612	'Tor4a'	2.15	2.35	3.8	5.18	2.82	6.1	2.25	1.44	2.78	3.76
227613	'Tubb4b'	374.1	341.2	428	228	414	608.1	323	498.6	285.5	384.04
227615	'Tmem203'	7.83	13.31	8.45	7.05	19.6	17.09	7.19	9.91	19.49	12.65
227619	'Man1b1'	17.53	10.78	24	5.2	25	9.6	12.1	14.33	28.64	27.56
22762	'Zfpm2'	1.63	1.92	0.71	3.34	0.09	0.35	0.96	0.51	0	0
227620	'Uap1l1'	11.1	27.41	44.9	43.3	36.3	53.53	22.3	22.35	65.46	22.38
227622	'Paxx'	45.74	40.5	61.2	70.7	67.3	67.6	35.9	54.66	62.05	49.79
227624	'Rabl6'	18.7	21.87	46.7	29	24.5	16.21	25.1	16.42	22.31	13.55
22763	'Zfr'	93.77	87.83	66.7	70.6	68.4	44.29	83.4	60.15	106.5	88.73
227632	'Kcnt1'	2.96	5.17	8.89	5.54	7.23	10.13	18.1	3.69	8.68	4.85
227634	'Camsap1'	3.04	3.9	4.64	11.1	5.65	5.17	4.89	4.03	2.13	2.49
227638	'Qsox2'	9.91	12.25	30	16.3	6.67	6.96	0.41	5.96	12.43	1.96
22764	'Zfx'	1.36	0.09	0.54	0.78	1.15	1.65	2.64	0.93	0.98	2.8
227644	'Snapc4'	3.16	5.87	8.94	8.33	10.5	3.29	6.39	7.17	6.51	2.81
227648	'Sec16a'	1.34	2.09	11.2	4.91	3.59	1.91	6.43	1.37	6.82	4.25
227656	'Rexo4'	27.63	15.57	24.5	21.2	19.9	43.52	17.8	30.75	17.62	24.82
227659	'Slc2a6'	10.84	13.42	9.04	19.1	12.2	6.84	32.7	31.64	12.7	3.93
227671	'Gbgt1'	0	2.68	0.87	1.48	0	0.02	1.67	2.08	0.51	1.33
227674	'Ddx31'	13.39	7.24	7.05	15.5	1.83	4.42	0.04	8.94	4.4	6.52
227682	'Trub2'	2.87	5.92	2.98	0.19	2.97	6.74	0.6	4.09	12.5	7.58
227683	'Coq4'	19.3	18.97	39.6	40.7	37.3	43.86	42.9	25.75	8.3	17.66
227693	'Zer1'	4.75	6.45	5.73	14.1	3.75	6.52	12.9	4.15	6.45	11.14
227695	'Spout1'	16.23	31.13	48.5	50.3	58.8	17.68	58.9	23.15	46.42	37.75
227696	'Phyhd1'	8.35	2.68	0	8.38	7.53	28.92	12.8	6.05	0.54	2.22
227697	'Dolk'	32.7	28.73	29.9	0.05	28.8	14.08	22.7	34.18	20.54	5.99
227699	'Nup188'	1.35	2.29	0.69	4.17	0.93	0.6	2.81	0.01	0.92	2.93
22770	'Zhx1'	14.53	9.78	12.6	14.2	6.84	8.24	7.32	13.25	7.15	11.12
227700	'Sh3glb2'	90.13	129.4	109	101	154	140.4	154	132.8	117	121.87
227707	'BC005624'	125.6	117.3	67.9	149	127	169.1	74.8	146.4	105.4	149.46
22771	'Zic1'	4.35	5.16	5.23	2.04	6.08	12.71	17.2	1.16	9.11	7.16
227715	'Exosc2'	28.64	48.92	11	89.3	26.9	20.34	5.19	41.37	19.83	22.26
227717	'Qrfp'	0	0.33	0	0	0	0	0.36	0	0	0
22772	'Zic2'	0.28	0.43	0.37	0.02	1.88	0.05	0.18	0	0.01	3.55
227720	'Nup214'	8.03	6.3	6.87	3.33	4.63	5.13	3.14	2.67	5.32	2.78
227721	'Plpp7'	18.52	25.89	12.8	0.06	34.4	32.76	29.5	37.85	14.19	23.9
227723	'Prrc2b'	13.4	12.56	13.7	18.5	13.6	9.37	16.9	3.88	6.26	10.44
22773	'Zic3'	2.9	0.4	6.39	0	4.19	0.76	9.33	2.2	2.03	7.69
227731	'Slc25a25'	31.26	29.81	72.2	25.5	27.9	19.82	43.4	27.73	24.82	18.2
227733	'Pip5kl1'	1.82	1.36	3.71	0	1.32	6.14	0.07	4.56	2.66	0
227736	'Cfap157'	0.21	0	0.93	0	0.51	0	0	0.05	0	0.31
227737	'Fam129b'	1.46	3.34	2.98	0	5.63	0	0	0.75	0	0.06

227738	'Lrsam1'	16.21	11.63	23.6	13.7	13.6	3.13	19.1	7.78	11.19	8.9
22774	'Zic4'	6.22	9.03	4.48	0	11	15.79	16.5	0	3.76	10
227743	'Mapkap1'	7.33	2.64	7.6	24.4	7.96	12.89	15.1	7.79	13.53	5.05
227746	'Rabepk'	27.32	8.61	30	39.4	16.1	42.79	19.5	15.97	38.04	31.19
22775	'Zik1'	3.68	2.14	3.2	11	1.97	1.6	4.42	4.8	4.01	5.7
227753	'Gsn'	10.69	15.4	8.49	9.41	38.2	8.08	21	25.25	6.31	10.32
22776	'Zim1'	1.61	5.74	4.32	2.64	0.43	0.03	0.19	4.57	0.81	0
22779	'Ikzf2'	2.17	0.36	0.62	1.06	0.01	0	0.21	0	0	0
22780	'Ikzf3'	0	0	0	0.08	2.18	0	0.01	0	0	0
227800	'Rabgap1'	20.84	9.75	21.2	18	10.5	16.87	14.4	14.91	23.31	19.21
227801	'Dennd1a'	6.48	4.42	4.98	12.7	2.39	1.26	6.53	0.5	1.28	2.93
22781	'Ikzf4'	2.25	2.49	4.74	5.1	3.6	2.59	1.85	1.86	1.8	6.72
22782	'Slc30a1'	0.85	0.56	0.17	2.77	0.76	0.03	0.21	0.77	0.21	0
227835	'Gtdc1'	5.74	2.63	3.63	5.04	4.93	1.51	5.34	7.91	7.88	5.93
22784	'Slc30a3'	0	0	0.03	0	0	0	0	0	0.18	0
22785	'Slc30a4'	0.06	1.48	1.52	3.73	1.04	0.01	2.59	0	1.97	0
227867	'Epc2'	2.54	2.03	1.46	11.2	5.15	1.18	3.48	0.83	1.58	1.15
22788	'Zp3'	0	0	0	0	0	0	0	0.28	0	0
22791	'Dnajc2'	20.5	24.08	14.6	14.5	28.2	28.62	16.9	16.43	24.76	10.89
22793	'Zyx'	8.91	5.85	4.45	0	8.89	2.53	14.4	6.35	21.75	13.54
227933	'Ccdc148'	13.06	7.03	7.17	9.14	14	9.39	8.85	8.43	9.78	14.57
227937	'Pkp4'	15.79	12.17	13.6	26.1	62.2	6.77	25.4	10.72	18.01	5.28
227960	'Gca'	4.06	4.72	2.24	4.26	1.93	0.78	0	3.2	4.69	4.87
228003	'Klhl41'	3.07	0.57	0.57	13.8	1.9	0.55	0.2	0.31	0.58	0.45
228005	'Ppig'	10.59	9.44	10.4	21.3	12.1	11.49	9.57	12.87	11.07	13.89
228012	'Tlk1'	1.93	5.4	2.73	1.79	6.57	5.36	5.62	8.51	5.88	2.86
228019	'Mettl8'	5.37	11.32	5.29	12.9	1.89	7.71	0	8.02	4.83	8.33
228026	'Pdk1'	11.41	6.14	8.27	0.77	7.57	4.09	2.26	4.88	7.2	7.76
228033	'Atp5g3'	412.5	329	572	288	480	546	402	410.8	256.2	345.64
228061	'Agps'	0.07	0.85	0.58	0.01	1	0.04	0	1.31	0.26	0.98
228071	'Sestd1'	1.61	2.53	3.97	1.75	2.23	1.2	1.5	4.6	2.1	3.16
228094	'Cerkl'	0	0.51	0	0	0	0.03	3	0	2.17	7.55
228136	'Zdhhc5'	2.56	2.51	3.16	5.53	2.08	0.01	8.4	0.99	5.42	2.7
228139	'P2rx3'	0	0	0	0	0	0	2.6	0.81	1.78	0
228140	'Tnks1bp1'	2.31	0.31	2.55	2.41	2.7	0.97	0.02	0.45	0	1.74
228355	'Madd'	8.97	9.17	18.6	13.8	10.9	10.69	7.86	8.04	13.47	13.38
228356	'1110051M20Rik'	174.2	218.4	187	207	186	234.7	259	214.9	205.9	221.77
228357	'Lrp4'	0.61	2.19	0.37	1.15	0.01	3.13	1.93	1.57	0.35	0.87
228359	'Arhgap1'	59.36	70.64	55	46.5	61.3	61.93	46.4	62.7	51.55	55.84
228361	'Ambra1'	4.39	0.56	6.51	0.07	2.55	3.41	6.72	4.65	12.07	4.72
228368	'Slc35c1'	2.66	3.91	8.13	0	2.97	0	0.32	2.26	5.63	10.64
228410	'Cstf3'	39.45	36.89	10.4	18.9	52.7	38.53	19.2	33.23	31.96	33.07
228413	'Prrg4'	0	0.15	2.41	0	0	2.81	0	0	0.15	1.08
228421	'Kif18a'	0	0.72	0.03	0	0	0	0	0	0	1.52
228432	'Ano3'	3.63	12.05	0.9	0.8	1.97	6.88	2.25	4.59	0	0
228482	'Arhgap11a'	0	0	0	1.75	0	0	0	0	0	0
228491	'Zfp770'	0	0.72	0.06	0	1.27	2.55	1.14	1.37	0.01	3.34
228536	'Bahd1'	2.69	2.65	3.81	0.08	0.61	0	2.04	0.46	0.85	0.01
228543	'Rhov'	13.01	9.56	14.7	10.2	25.7	26.9	23.5	37.34	3.87	6.9
228545	'Vps18'	14.14	7.85	10.6	3.62	19.5	6.8	14.7	7.03	16.1	7.44
228550	'Itpka'	0.54	2.56	0	7.32	0	0	0	0.2	0	0
228564	'Frmd5'	9.39	3.69	0.73	3.87	4.16	1.95	7.71	2.47	0.34	1.98
228598	'Ebf4'	0.02	3.5	5.91	19.1	11.2	7.4	3.11	3.54	3.96	0.21
228602	'4930402H24Rik'	12.6	16.18	11.1	22.1	11.1	11.23	15.3	10.14	13.88	13.13
228607	'Mavs'	11.25	14.85	9.53	2.08	3.33	14.1	7.58	4.62	5.47	14.71
228608	'Smox'	6.83	10.35	21.2	20.4	8.48	6.59	3.89	8.35	14.93	6.29
228662	'Btbd3'	4.29	5.71	11.6	3.22	13.1	10.06	2.93	6.06	10.9	15.26
228677	'Sptlc3'	0	0.06	0	0	0	0	0	0	0	0

228714	'Kat14'	6.43	2.12	2.13	8.79	6.5	0.06	1.79	4.75	7.12	4.19
228715	'Smim26'	73.11	53.42	54.4	85.7	43.1	112	51.9	43.25	48.5	43.14
228730	'Kiz'	9.63	16.45	7.2	21.1	19.2	32.73	52.8	16.98	7.74	13.12
228756	'Cstl1'	0	2.11	0	0	0	0	0	0	0	0
228765	'Sdcbp2'	2.93	0	1.54	0	0	0	0	0	0	0
228769	'Psmf1'	24.58	34.74	25.1	16.7	20.2	38.01	22.2	23.22	34.81	46.11
228770	'Rspo4'	1.2	0	0	0	0.13	0	0	0	0	0
228775	'Trib3'	0.47	0	0	0	0	0	0	0	0	0
228777	'Nrsn2'	757.8	920.5	857	863	632	854.7	824	892.7	894.2	1181.82
228778	'6820408C15Rik'	2.06	5.12	3.94	1.3	2.87	9.56	4.83	1.91	1.1	5.18
228787	'Xkr7'	0.4	0	0.94	0.7	0	0	0	0	0	0
228788	'Ccm2l'	0	1.19	0	0	0	0	0	0.31	0	0.17
228790	'Asxl1'	0.01	2.28	6.66	4.59	6.92	1.89	3	3.15	0.01	5.33
228801	'Bpifb1'	10.82	0	0.86	7.67	0	0	0	1.14	0	3.87
228802	'Bpifb5'	0	1.18	0	0	0	0	0	0	0	0
228807	'Zfp341'	0.52	0	1.11	5.05	1.42	0.02	2.3	0	0.33	0
228812	'Pigu'	53.13	32.61	43.5	0	39.8	58.75	42.7	59.01	60	54.87
228829	'Phf20'	23.79	14.54	17.9	29.3	27.7	14.52	21.9	19.93	13.84	19.55
228836	'Dlgap4'	22.21	24.19	21.1	32	20.7	15.41	27.4	14.41	14.45	7.76
228839	'Tgif2'	1.67	1.94	1.47	3.94	1.05	0.15	0	2.64	0.28	4.01
228846	'D630003M21Rik'	0	1.37	0.04	0	0	2.51	0	0.01	0	0
228850	'Ralgapb'	1.56	2.68	1.28	2.33	3.44	1.68	0.22	1.13	1.67	4.12
228852	'Ppp1r16b'	20.64	17.27	13.1	20.8	26.7	29.89	12.6	13.99	13.18	14.46
228858	'Gdap1l1'	81.38	75.84	68.8	52.7	43.3	59.53	66.7	77.64	77.47	44.94
228859	'Fitm2'	11.32	10.47	16.6	9.74	2.36	14.51	11.2	15.66	14.23	3.17
228866	'Pcif1'	14.76	12.7	18.1	25.2	23.8	8.47	22.3	15.87	24.04	15.81
228869	'Ncoa5'	2.16	7.39	1.69	2.98	8.05	0.44	0.02	2.98	1.33	0.57
228875	'Slc35c2'	29.5	27.2	58.9	0	51.1	35.92	46.8	34.12	28.89	22.72
228876	'Zfp334'	0.99	1.39	3.67	6.12	0.2	1.13	3.21	1.79	0	3.97
228880	'Zmynd8'	31.85	51.21	41.4	67.5	52.4	39.47	16.8	31.47	62.26	66.89
228889	'Ddx27'	10.7	19.41	20.6	12.2	14	12.15	15.9	7.58	14.94	14.05
228911	'Tshz2'	7.47	9.04	8.25	10.6	9.12	8.51	16.3	8.42	12.32	6.31
228913	'Zfp217'	0	0	0	0	0.03	0	0	0.03	0	0.05
228942	'Cbln4'	12.25	3.7	17.3	12.3	26.5	24.72	72.2	11.29	5.74	23.43
228960	'Stx16'	6.52	8.32	14.4	13	21.4	12.22	5.45	15.01	31.62	10.25
228961	'Npepl1'	5.97	1.35	0	0.11	0	0.02	0.02	0	1.76	2.36
228966	'Ppp1r3d'	0	3.46	0	0	0	0	1.08	2.16	1.47	0
228980	'Taf4'	0.69	1.79	2.93	0	1.38	0	0.83	0.66	0	2.06
228983	'Osbpl2'	27	55.61	45.6	43.1	54.4	32.06	8.58	33.91	40.58	49.37
228993	'Slc17a9'	0	0	0	0	0	3.64	0	0	0	0
228994	'Ythdf1'	1.44	1.6	3.14	4.23	3	3.67	0.14	2.85	2.07	0.6
228998	'Arfgap1'	57.88	34.48	39.4	56.1	69.5	59.08	87.9	61.59	65.48	42.18
229003	'Helz2'	0	0	0	0	0	0	0	0	0	0.84
229004	'Gmeb2'	2.08	2.91	0	0	0.4	1.6	0.01	2.3	1.51	1.41
229007	'Zgpat'	38.25	28.89	35.8	0.97	35.7	19.16	55.4	22.14	23.94	12.09
229011	'Samd10'	10.53	19.23	23.9	2.01	34.1	8.01	22.3	12.04	32.55	12.44
229055	'Zbtb10'	0.2	0.21	0.76	2.36	0.03	0	0.01	0.01	0.02	0.01
229096	'Ythdf3'	5.61	6.08	6.94	4.47	6.09	1.13	3.14	1.84	3.77	3.24
229211	'Acad9'	17.25	20.39	17.7	7.8	18.3	24.5	23.2	31.86	16.65	17.27
229214	'Qrfpr'	0	0	1.13	0	0	5.48	1.76	0.22	2.2	0
229227	'4932438A13Rik'	3.46	3.3	6.19	9.38	5.69	3.04	6.98	3.4	9.86	10.95
229228	'Nudt6'	4.26	3.18	0.13	32.5	2.9	4.41	6.83	0.32	7.67	2.88
229279	'Hnrnpa3'	30.39	44.17	25.6	16.8	39	25.17	45.3	32.1	45.97	41.11
229285	'Spg20'	16.53	15.33	9.71	5.98	14	8.26	7.5	12.91	11.55	28.87
229302	'Tm4sf4'	2.31	0	0	0	0	0	0	0	0	0
229317	'Eif2a'	7.29	6.25	8.78	14.6	6.94	12.86	3.45	9.89	13.04	18.55
229320	'Clrn1'	0	3.48	0.07	3.23	0	0	0.07	2.99	0.01	0.01
229323	'Gpr171'	1.9	0	0.3	10.7	1.22	1.68	0	0	2.9	0

229357	'Gpr149'	7.29	6.44	0.89	0	4.62	2.97	4.96	4.2	0.19	1.33
229363	'Gmps'	18.95	22.3	13.5	25.8	26	20.27	24.1	12.19	15.52	41.68
229445	'Ctso'	7.89	5.41	13.7	17.1	3.83	14.71	7.16	8.95	6.53	19.39
229473	'Tmem131l'	3.86	6.65	0.97	1.37	14.1	5.93	4.24	5.72	7.87	10.17
229474	'Fhdc1'	0	0	0.26	1.24	0	0	0	0	0	0
229487	'Gatb'	18.07	6.03	22.4	0.03	21	22.98	6.21	38.86	22.13	14.13
229488	'Fam160a1'	0	0.75	0	0	0	0	0.5	0.63	0	0
229499	'Fcrl1'	0	0	0	0	0	6.99	0	0	0	0
229503	'Rrnad1'	15.99	18.46	23.3	9.95	25.9	3.44	18.8	15.06	22.07	27.41
229504	'Isg20l2'	2.91	0.26	2.82	0	0.08	0.69	0	3.38	13.13	0.83
229512	'Smg5'	19.25	11.06	19.6	18.5	17.6	14.1	5.62	10.59	17.41	10.32
229517	'Slc25a44'	20.48	10.97	23.5	17.3	21.2	13.49	9.24	31.31	16.44	13.57
229521	'Syt11'	313.4	279.8	294	195	252	359.4	298	381.5	282.4	259.93
229524	'Msto1'	32.99	34.01	26.8	12.3	53.7	19.23	49.8	37.6	24.15	50.13
229534	'Pbxip1'	12.64	17.42	12.7	15.2	15.1	9.88	1.89	10.33	7.83	16.91
229541	'Dennd4b'	0.09	4.03	3.02	4.86	1.18	0	2.07	1.42	3.28	1.69
229542	'Gatad2b'	3.79	5.83	2.71	4.58	3.18	2.54	1.93	1.54	1.67	3.09
229543	'Ints3'	7.98	7.86	7.39	6.11	2.56	1.03	16.4	3.09	6.44	3.41
229574	'Flg2'	0.01	0	0	0	0	0.01	0	0	0	0
229584	'Pogz'	3.85	2.03	5.1	6.98	2.34	0	3.76	5.71	4.55	8.29
229588	'Gm128'	0	0.03	0	0	0	0	6.59	0	0	0
229589	'Prune1'	11.44	12.13	11.7	18.8	15.9	13.7	15.5	14.07	21.52	25.13
229593	'Golph3l'	9.88	7.9	8.07	0.11	10.5	1.15	4.31	7.49	10.73	11.01
229595	'Adamtsl4'	0	0	0	0	0.49	2.43	0.02	0.88	1.22	0.02
229599	'Ciart'	22.09	23.45	22.6	88.8	12.5	19.99	19.7	20.03	5.75	9.8
229600	'BC028528'	1.25	1.51	8.44	8.29	1.14	2.05	17.5	1.52	1.92	2.47
229603	'Otud7b'	1.26	0.91	1.86	1.16	1.47	2.04	0.66	0.11	2.93	0.81
229615	'Pias3'	18.68	19.56	14.8	3.44	15.8	11.54	30.3	30.19	13.85	16.74
229644	'Trim45'	9.72	7.8	2.43	17.1	15.4	5.83	15.9	6.14	5.72	8.88
229658	'Vangl1'	0.33	1.15	1.49	11.7	1.69	0	0.01	0	0	0.93
229663	'Csde1'	54.85	64.87	63.5	53.5	36.8	23.44	48.4	42.01	54.11	72.51
229665	'Ampd1'	0.04	0	0.02	0	0	0	0	0	0	0
229672	'Bcl2l15'	0	0	0	0	0	0	0	0	0.06	0
229675	'Rsbn1'	6.61	4.57	6.33	12.1	5.25	4.21	10.1	3.61	7.78	1.32
229681	'St7l'	11.3	10.17	8.2	13.3	7.65	9.9	12.7	3.09	11.04	18.01
229687	'Chil5'	0	1.57	0.54	1.44	1.02	0.75	0.89	0.21	0	0.16
229699	'Slc16a4'	0.05	0	0	0	0	0	0.11	0	0	0
229700	'Rbm15'	2.61	0.68	1.36	1.58	0.91	0.65	1.34	0.41	0.61	0.91
229706	'Slc6a17'	8.85	4.19	9.83	7.69	13.3	14.57	0.87	6.14	8.61	7.69
229707	'Strip1'	50.66	47.33	35.6	56.4	36.2	20.72	53.3	38.32	46.23	33.97
229709	'Ahcyl1'	24.04	16.41	21.5	8.62	19.3	10.42	26.7	12.35	19.51	9.07
229714	'Gpr61'	12.24	10.03	8.01	0	9.48	1.7	7.47	10.29	18	4.53
229715	'Amigo1'	1.82	3.25	4.19	1.59	4.68	4.5	2.8	1.3	3.66	0.83
229722	'5330417C22Rik'	14.19	28.61	19.5	13.2	25.1	9.34	7.52	19.58	36.03	33.42
229725	'Clcc1'	8.39	25.86	21.2	9.81	17.9	15.54	19.2	19.32	17.94	21.51
229731	'Slc25a24'	0	0	0.01	0	1.76	0	0	1.04	0.89	1.94
229759	'Olfm3'	38.94	16.74	7.78	14	3.62	1.37	15.2	10.46	12.51	5.6
229776	'Cdc14a'	0.01	0	0.01	7.6	0	0	1.11	0	0	0
229780	'Trmt13'	3.38	3.93	3.1	7.52	5.38	5.13	10.8	7.24	6.3	10.03
229782	'Slc35a3'	0.15	1.19	1.97	2.89	2.12	0.91	0.01	0.07	2.41	1.32
229791	'Plppr4'	34.91	28.58	11.1	30.1	26.9	12.75	15.7	22.85	21.5	22.65
229801	'Tram1l1'	137.7	124.7	71.8	68.1	84.2	90.48	94.8	117.4	85.85	125.73
229841	'Cenpe'	0.01	0.01	0.01	0	0.43	0.13	0.01	0.89	0	0
229877	'Rap1gds1'	25.43	24.38	37.1	31.6	16.5	6.22	17.7	32.61	8.28	18.45
229898	'Gbp5'	0.32	0	0	0	0	0	0	0.25	0	0
229900	'Gbp7'	1.18	0.42	1.63	3.09	0.41	0	0	0.03	1.27	2.58
229905	'Kyat3'	0.02	2.53	3.99	0	1.58	0.03	0.25	0	3.06	0
229906	'Gtf2b'	37.99	33.96	37.6	60.3	48	82.88	41	42.85	58.11	44.58

229927	'Clca3b'	0	0	0	0	0	0	0	0.01	0	0
229937	'Znhit6'	18.55	27.51	18.2	5.04	33.5	39.89	26.5	10.52	19.67	10.35
229949	'Ak5'	5.27	6.73	16.7	18	3.44	0	10.3	9.7	34.51	7.49
230025	'Prdm13'	0	0.45	0	0	0	0	0	0	0	0
230027	'Coq3'	25	9.68	17	8.44	15	15.33	10.3	13	12.57	18.96
230073	'Ddx58'	0.02	0.62	0.46	7.35	0.76	1.01	0	0.47	0.93	2.47
230075	'Ndufb6'	424.7	356.3	484	384	362	449.3	482	410.7	429.9	385.08
230082	'Nol6'	14.79	25.17	40.6	6.25	16	6.98	40.5	17.35	20.53	16.17
230085	'Phf24'	10.96	5.99	17	4.85	8.65	5.22	7.11	8.1	3.19	7.4
230088	'Fam214b'	10.53	18.76	11	15.7	16.1	21.11	31.4	3.59	21.93	14.1
230098	'Arhgef39'	0	0.93	0	0	0	1.85	0	0.25	0	0
230099	'Car9'	0	0	0	2.11	0	0	0	0	0.02	0
230101	'Gba2'	5.15	8.32	11.9	16.1	16.8	10.15	18.6	13.93	20.9	11.06
230103	'Npr2'	8.13	11.94	5.46	19.4	1.58	7.25	13.4	3.36	5.88	6.69
230119	'Zbtb5'	5.96	1.19	3.73	2.22	3.91	1.01	0	0.01	2.28	1.28
230125	'Slc25a51'	34.97	17.35	27.6	20.1	18.8	18.63	25.8	21.21	30.12	34.47
230126	'Shb'	0.14	0	0.02	0	9.51	0	0.78	1.58	2.91	5.33
230145	'Galnt12'	0.97	0	0	0	0	4.23	0	0.03	6.64	2.42
230157	'Tmeff1'	4.95	4.54	10.8	20.6	11.6	12.27	10.8	4.68	10.87	3.33
230161	'Acnat1'	0.03	0.08	0.08	0.17	0.04	0.09	0.1	0.02	0.09	0.04
230162	'Zfp189'	4.64	3.89	5.14	4.1	1.5	0	0.02	2.97	4.78	4.65
230163	'Aldob'	0.04	0.86	0	3.22	5.89	0	0	3.8	0.89	1.04
230233	'Elp1'	7.64	13.59	26.9	2.6	19.2	6.69	9.15	7.52	11.44	7.96
230234	'Fam206a'	2.67	6.39	5.51	7.22	3.39	1.11	1.55	2.42	6.84	0.52
230235	'Frrs1l'	8.84	10.59	7.56	5.11	18.1	12.4	4.42	10.83	5.69	5.27
230249	'Ecpas'	5.9	4.22	8.88	10	3.88	3	3.7	3.13	7.16	11.62
230257	'Ptbp3'	1.12	1.63	0.25	0.06	0.96	0.59	0	0	1.39	0.08
230259	'E130308A19Rik'	4	0	1.32	0	6.85	3.88	0.24	1.01	0	1.98
230279	'Tmem268'	2.91	0.91	6.53	0	10.7	9.4	8.1	12.13	4.58	5.67
230316	'Megf9'	6.72	0.03	10.3	13.2	2.45	0	4.23	0.04	4.93	1.13
230376	'Haus6'	0.66	0	2.16	1.03	3.83	1.26	0	1.04	4.38	1.28
230379	'Acer2'	7.5	6.8	4.49	5.53	4.86	18.72	4.71	4.74	2.56	11.2
230393	'Focad'	2.93	4.66	9.19	21.4	3.69	5.34	6.66	0.83	7.58	5.76
230459	'Cyp2j13'	0.01	0.01	0	0	0.04	0.02	0.01	0	0.08	0
230484	'Usp1'	0	1	2.81	12	3.31	0.01	0.03	1.52	1.99	0.08
230500	'Efcab7'	0.94	4.05	0	0	0.03	4.08	0	0.07	1.52	0.05
230514	'Leprot'	28.74	6.21	12.3	27.8	21.5	41.29	5.75	18.41	15.76	18.99
230576	'Ttc22'	5.27	3.71	0	0	0	13.22	6.04	3.08	15.37	8.8
230577	'Pars2'	3.41	6.8	0.04	0.03	2.3	0.37	11.7	3.67	10.22	2.25
230579	'Fam151a'	0	3.58	0	0	0	0	0	0	0	0
230582	'Cyb5rl'	9.81	0	2.98	11	11.5	12.05	0.2	0.67	3.23	14.73
230584	'Yipf1'	58.14	43.44	31.6	68	51.2	31.04	53.3	61.63	73.88	63.19
230587	'Glis1'	0	1.29	0	0.24	0.17	0	0	0	0	0
230594	'Tut4'	2.38	3.76	1.31	5.07	3.91	4.3	3.54	1.82	4.26	2.93
230596	'Prpf38a'	11.02	10.21	3.91	25.8	16.4	9.05	8.04	24.56	18.4	26.35
230597	'Zfyve9'	0.39	5.16	0.78	2.36	5.31	0.71	4.43	2.3	7.55	0.71
230598	'Nrd1'	21.3	24.28	22.4	9.04	19.7	20.44	25.3	17.95	30.27	17.04
230603	'Ttc39a'	8.18	4.59	9.36	4.1	8.3	4.77	1.95	0	0.82	4.75
230648	'Efcab14'	9.53	11.23	4.22	4.38	16.1	7.05	9.76	6.22	2.98	8.42
230649	'Atpaf1'	9.98	16.53	9.94	19.1	9.27	22.19	6.66	13.8	16.12	7.02
230654	'Lrrc41'	30.75	37.45	52.8	21.8	29.3	37.39	17	29.02	35.26	37.31
230657	'Tmem69'	2.03	1.22	7.37	11.5	16.9	2.97	6.73	2.03	5.34	6.01
230661	'Tesk2'	0	6.49	2.73	6.23	0.06	0	0.02	1.45	0	2.48
230673	'Ipo13'	13.93	15.05	13.3	24.1	18.6	12.75	23.9	6.94	16.56	8.21
230674	'Kdm4a'	9.38	4.03	3.56	3.24	4.43	5.44	4.94	9.84	1.38	4.15
230676	'Szt2'	2.2	0.95	2.82	0.19	0.89	1.08	2.08	1.28	0.07	0.91
230678	'Tmem125'	5.46	3.23	0	0	3.51	1.57	0	0	0	0
230696	'AU022252'	10.29	6.35	12.9	5.98	6.89	14.63	3.86	15.01	5.12	3.84

230700	'Foxj3'	10.63	14.81	15.8	26.7	11.2	12.89	10.5	4.34	18.81	11.81
230709	'Zmpste24'	5.75	19.01	6.8	5.28	5.16	1.12	10.5	10.02	9.78	7.92
230718	'Nt5c1a'	1.26	0.96	3.32	0	1.26	0.14	5.16	2.55	1.67	0.56
230721	'Pabpc4'	3.72	2.65	2.35	2.66	0.49	2.8	3.29	3.81	4.09	7.72
230726	'Rhbdl2'	0.07	0.12	0.09	0.16	0.33	0	0.11	0.1	0.18	0.07
230734	'Yrdc'	6.03	7.1	8.14	19.2	5.64	2.44	0.29	3.37	1.73	2.55
230735	'Epha10'	5.51	0.09	6.37	1	12.1	8.9	1.26	5.45	1.11	0
230737	'Gnl2'	48.28	40.78	47.5	66.3	32	48.99	40.7	57.73	45.6	50.76
230738	'Zc3h12a'	0	0.02	0.01	0	0.03	0.16	0	0	0	0.02
230751	'Oscp1'	28.82	25.91	32.3	18.3	40.6	43.38	56.2	34.82	20.51	37.04
230752	'Eva1b'	0	0.89	0.47	0	0	0	0	0.6	0	0
230753	'Thrap3'	24.24	26.64	20	34.4	27.3	24.46	22	22.31	38.27	30.78
230757	'5730409E04Rik'	24.29	22.84	13.8	9.37	12.9	7.81	20.1	22.55	6.04	14.7
230761	'Zfp362'	2.29	5.41	5.24	7.85	4.69	3.67	8.8	3.72	6.67	3.69
230767	'Iqcc'	15.81	11.14	0.48	9.2	9.74	11.16	11.3	11.73	1.74	5.57
230770	'Tmem39b'	10.64	12.7	15.7	1.49	0.95	7	23.6	13.52	29.24	22.81
230775	'Adgrb2'	4.12	2.39	1.08	1.34	5.1	0.03	4.17	0.01	1.6	0
230777	'Hcrtr1'	9	27.15	4.91	0	1.39	11.72	11.5	22.58	20.18	0
230779	'Serinc2'	2.79	0.32	0.11	0.2	0.13	0.14	0.06	0.12	2.42	0.08
230784	'Sesn2'	20.32	14.89	14.8	1.72	8.32	3.71	8.68	19.81	7.84	1.91
230789	'Fam76a'	36.93	29.08	47.3	31.3	26.8	24.33	28.7	21.07	31.96	21.36
230793	'Ahdc1'	0.17	2.97	1.31	3.38	1.7	1.11	1	1.03	0.75	0.95
230796	'Wdtc1'	1.4	1.37	0.76	2.82	4.06	1.78	8.1	4.16	6.56	0.98
230801	'Pigv'	6.53	7.53	3.46	10.4	21.1	3.97	4.67	5.01	1.23	19.92
230806	'Crybg2'	0.51	0	0	0	0.24	0.01	0	0	1.81	0
230809	'Pdik1l'	2.77	6.76	5.12	0.06	8.94	3.23	3.46	0.89	9.95	8.2
230815	'Man1c1'	0	1.27	2.43	0	2.65	1.23	0.05	0.18	4.13	0.15
230822	'Ncmap'	0	4.9	5.46	0.76	7.79	0	8.28	1.94	16.14	3.86
230824	'Grhl3'	2.73	0	0	4.35	0	0	0	0	0	0
230837	'Asap3'	0	2.11	1.5	2.13	0.21	1.91	2.39	1.42	0	0.04
230848	'Zbtb40'	1.36	0	0.7	0	0.09	0.85	0	0.13	0.03	0.09
230857	'Ece1'	18.22	28.54	19.2	47.9	14.6	20.58	18.8	21.95	25.71	30.94
230861	'Eif4g3'	12.11	8.77	12.3	9.5	8.62	6.79	4.2	7.64	3.18	1.6
230863	'Sh2d5'	13.13	16.64	13.7	11	23.5	15.85	18.3	9.95	25.38	20.99
230866	'Emc1'	33.55	38.75	45.3	32.9	23.7	10.76	35.9	27.92	36.48	25.4
230868	'Igsf21'	14.89	12.88	26.6	26.8	41.7	2.43	21.6	35.56	0	2.56
230872	'Crocc'	2.29	3.84	3.02	5.55	3.93	5.74	2.53	2.6	6.32	2.24
230895	'Vps13d'	1.68	0.63	1.19	1.45	1.45	0.77	2.59	0.95	2.03	0.45
230899	'Nppa'	0.11	1.87	8.26	19.3	1.08	0.22	0	0	0	0.99
230903	'Fbxo44'	117.1	114.5	152	137	169	180.1	86	174.8	140.2	116.51
230904	'Fbxo2'	21.82	11.67	21.3	65.7	44.8	10.68	28.8	41.55	11.66	7.79
230908	'Tardbp'	21.93	18.66	16.4	27.5	24	12.2	24.6	18.15	22.03	14.82
230917	'Tmem201'	27.17	33.58	30.3	0.91	31.3	25.67	23.7	25.21	25.77	11.28
230935	'Dnajc11'	40.59	38.8	21.8	36.9	56.9	37.4	32.5	39.66	39.81	26.29
230936	'Phf13'	5.61	6.67	1.6	16.9	5	0	0.03	2.86	3.58	1.97
230959	'Ajap1'	0.01	0.28	0.64	0.99	0.3	4.46	1.57	0.62	2.61	1.1
230967	'Cep104'	6.27	9.24	5.94	8.56	4.14	2.13	8.47	5	5.38	7.84
230971	'Megf6'	0.48	4.08	3.37	9.5	3.1	0.2	0.2	1.85	0	2.77
230972	'Arhgef16'	0	0	3.08	0	0	5.33	0	0	0.19	0.07
230979	'Tnfrsf14'	0.15	0	0.11	0.12	0.22	0.13	0.03	0.02	0.07	0.1
230991	'Fndc10'	13.49	3.83	25.7	0.48	20.3	18.7	39.4	15.77	12.09	7.61
230996	'9430015G10Rik'	18.84	2.12	7.99	17.7	6.74	7.68	10.2	5.88	4.33	8.48
231002	'Plekhn1'	2.69	0	3.63	0	3.98	0	2.71	0	5.25	0.85
231003	'Klhl17'	2.23	4.14	2.13	5.99	7.53	6.98	3.11	3.66	3.49	6.49
231004	'Samd11'	3.14	7.4	8.01	4.68	4.12	20.15	15.7	7.42	8.93	17.28
231014	'9330182L06Rik'	5.22	4.6	14.8	11.4	3.29	1.11	4.85	9.78	10.15	12.25
231042	'Nupl2'	2.3	5.29	1.73	3.12	5.49	7.61	7.08	6.84	1.02	4.6
231050	'Galnt11'	19.42	28.43	35	31.2	28	13.29	36	31.53	30.77	21.57

231051	'Kmt2c'	1.6	3.35	2.15	4.82	2.84	1.41	2.81	1.4	2.82	2.89
231070	'Insig1'	39.96	38.74	55.2	47.1	42.6	14.4	36.7	33.68	78.22	24.63
231086	'Hadhb'	116.8	69.23	63.9	52.6	87.4	122.7	73.8	32.26	80.16	71.05
231093	'Agbl5'	6.33	3.83	4.33	0	3.01	2.71	9.98	2.12	6.51	1.11
231103	'Gckr'	2.17	0	0	0	0.15	0	0	0	0	0
231123	'Haus3'	0.99	3.3	1.95	0.12	12.1	8.06	13.5	1.46	7	7.87
231125	'Zfyve28'	2.76	2.17	2	10.1	3.93	7.25	3.12	2.64	3.9	6.84
231128	'Fam193a'	4.77	4.99	3.38	11.9	1.74	6.23	3.77	4.33	2.15	1.89
231130	'Tnip2'	5.43	10.8	27	0	5.35	12.52	4.87	30.57	37.65	17.1
231134	'Dok7'	4.23	6.45	8.22	1.42	10.6	0.19	2.7	4.3	11.33	0.99
231148	'Ablim2'	23.7	35.15	31.7	8.14	29.3	24.52	38.1	22.33	18.91	31.93
231151	'Tada2b'	0.72	0	0	4.51	0.51	0.72	0	0.18	0	0
231201	'AF366264'	0	0	0.02	0	0	0	0	0	0	0.01
231207	'Cpeb2'	0.53	0.95	1.56	2.68	0.97	0.82	4.19	1.74	0.46	1.43
231214	'Cc2d2a'	1.63	2.25	0.83	7.18	0.23	1.14	2.87	1.68	1.66	8.18
231225	'Tapt1'	6.86	0.01	1.38	4.23	1.98	2.07	7.38	4.57	1.28	0.88
231238	'Sel1l3'	1.17	0	1.17	4.12	2.27	0.47	0	0.29	2.47	0
231252	'Chrna9'	0	0.38	0	0	0	0.16	0	0	0	0
231279	'Guf1'	1.92	1.66	1.68	0.89	4.82	5.89	3.89	2.94	4.02	2.1
231290	'Slc10a4'	0	0	0	0	4.3	0	0	2.85	0	1.33
231293	'Cwh43'	0	0	0	0	0.02	0	0	0.52	0.08	0
231296	'Lrrc66'	0	0	0.79	0	0	0	0	0	0	0
231326	'Aasdh'	5.28	4.01	1.77	3.04	6.03	2.82	12.9	3.46	2.34	4.5
231327	'Ppat'	1.68	6.15	3.93	6.37	4.67	4.09	5.16	1.86	2.15	0.01
231329	'Polr2b'	38.99	19.41	22.7	43.4	20.6	8.33	39.4	29.16	41.06	49.3
231380	'Uba6'	7.33	2.24	5.1	0.37	0.49	1.29	0.14	1.13	4.63	6.41
231386	'Ythdc1'	6.72	4.66	5.83	23.3	6.04	5.71	6.26	4.53	5.83	3.71
231413	'Grsf1'	34.73	17.87	48.8	64.8	22.6	11.84	49.6	27.49	28.41	34.16
231430	'Cox18'	24.29	14.67	17.6	31.1	15.6	44.47	18.2	16.98	10.12	25.67
231440	'Parm1'	61.83	30.4	13.2	34.7	95.3	30.43	32	25.95	7.62	26.96
231452	'Sdad1'	1.28	4.6	10.2	7.48	2.11	8.6	9.93	5.15	7.95	8.62
231464	'Cnot6l'	1.78	2.86	1.83	1.85	1.1	0.86	1.43	0.48	1.51	0.82
231470	'Fras1'	0	0	0	0.01	0.53	0	0	0.05	0	0.25
231474	'Paqr3'	13.54	20.23	10.8	18.7	14.8	9.36	8.16	9.45	19.06	8.79
231503	'Tmem150c'	9.4	10.13	9.93	8.16	10.9	16.82	12.4	7.54	19.61	15.52
231506	'Lin54'	1.03	0	3.19	0	0.03	0	1.3	0.7	0	1.36
231507	'Plac8'	0	0	0	0	1.64	0	0	0	0	0
231510	'Gpat3'	1.87	0	3.5	2.29	3.89	1.41	0	3.58	8.61	2.35
231532	'Arhgap24'	24.57	12.92	16.1	4.65	20.4	59.84	15.5	20.48	13.03	47.29
231549	'Lrrc8d'	4.24	11.28	5.23	5.38	7.76	0.01	0.11	2.95	10.35	7.56
231570	'A830010M20Rik'	9.75	8.48	10.2	15.5	8.53	4.38	5.82	9.41	10.07	11.94
231571	'Rpap2'	5.03	16.84	3.18	16.9	16.6	10.48	6.74	21.42	8.72	11.7
231580	'Gak'	16.98	13.42	24.6	17	22.9	10.56	28.4	27.13	29.21	14.66
231583	'Slc26a1'	1.29	0.44	0	0	0	0	0	2.28	0.77	1.97
231600	'Chfr'	12.31	10.71	10.7	13.8	6.29	3.63	9.57	10.09	9.73	12.84
231602	'P2rx2'	1.98	4.57	0.03	0	0.03	0	0	2.82	0	10.14
231605	'Galnt9'	3.05	2.93	3.3	1.93	6.31	10.24	0	12.16	7.82	0
231630	'Ficd'	12.7	11.05	12.3	2.75	8.86	23.01	8.22	13.8	14.56	26.08
231633	'Tmem119'	0	1.91	0	0.03	1.06	75.29	0	1.79	0	0
231637	'Ssh1'	2.39	1.26	3.08	1.86	0.61	1	4.94	1.16	0	0.15
231642	'Alkbh2'	9.3	15.63	21.7	6.06	16.6	16.14	2.43	8.33	10.92	22.93
231646	'Myo1h'	2.98	3.78	0.39	0.1	0.12	5.88	4.7	1.73	1.43	4.84
231659	'Gcn1l1'	7.36	5.23	10.3	1.15	11.4	4.52	10.6	4.42	1.14	3.64
231668	'Vsig10'	0	1.45	0.28	0	0	0	0	0	0	3.7
231670	'Fbxo21'	25.89	36.56	81.4	39.2	38.2	18.71	79.7	40.6	35.55	25.1
231672	'Fbxw8'	2.93	3.41	0.42	1.07	0.91	0.15	0.32	1.2	0	0.43
231712	'Trafd1'	22.88	27.47	36.7	32.4	27.7	30.2	33	32.05	26.43	16.14
231713	'Naa25'	22.71	21.53	23.5	2.61	17.6	18.16	36.9	13.74	18.24	13.45

231717	'Pheta1'	0.02	5.39	8	0	4.67	3.18	4.99	0	5.5	2.28
231724	'Rad9b'	1.98	7.1	14.7	12.5	3.45	0.03	8.46	17.06	0.11	7.43
231727	'B3gnt4'	6.75	1.67	0	5.54	0	0.1	0	2.64	0.02	1.18
231760	'Rimbp2'	1.1	0.65	1.24	2.15	3.22	3.25	0.55	0.78	0.14	0.57
231769	'Sfswap'	5.3	6.27	10.7	5.26	12.3	10.59	7.44	6.55	6.48	3.12
231798	'Lrch4'	5.47	12.2	13.6	0	23.3	13.52	2.06	17.82	20.58	10.54
231801	'Agfg2'	12.69	4.72	4.02	3.3	2.53	3.31	2.64	7.66	9.82	1.81
231803	'Mepce'	3.82	0.18	5	7.35	3.71	5.07	1.61	5.46	6.02	2.17
231805	'Pilra'	0	0.53	0.02	0.04	0.09	3.08	0	0.46	0	0.02
231807	'BC037034'	0.1	0	0	1.35	0.83	0	1.64	1.16	2.01	0
231821	'Adap1'	15.56	16.48	24.2	17.1	29	12.29	10.5	14.34	8.76	10.65
231830	'Micall2'	0.94	3	0.95	0	0.44	0.38	1.49	0.33	3.38	0.96
231832	'Tmem184a'	0	5.71	0	0	7.64	0	0.05	0	0	0
231834	'Snx8'	11.82	29.79	29.1	0.58	28.9	12.06	20.9	19.9	6	3.32
231841	'Brat1'	16.6	7.66	11	0	7.19	15.76	2.93	11.17	1.73	4.25
231842	'Amz1'	15.84	17.43	11	5.71	4.61	3.2	5.02	10.83	13.8	7.34
231855	'Ap5z1'	8.47	8.27	7.38	7.72	6.89	6.94	0	5.38	8.31	4.55
231858	'Radil'	8.5	12.99	13	0.92	13	4.79	9.8	20.08	12.28	4.42
231861	'Tnrc18'	0.25	0.9	1.64	0.2	0.41	0.06	0.48	0.08	0.2	1.06
231863	'Fbxl18'	0.03	1.65	0	5.92	1.52	5.17	0.97	1.21	3.08	0
231866	'Zfp12'	4.24	2.04	4.26	5.5	6.68	2.93	3.5	1.11	6.15	2.12
231868	'E130309D02Rik'	12.52	6.03	7.17	10	15	3.47	13.1	0.66	23.06	10.54
231871	'Daglb'	19.58	11.11	18	10.3	19.8	24.07	15.5	12.29	10.51	27.58
231872	'Aimp2'	41.33	52.82	78.4	76.7	79.6	49.25	46	49.21	76.5	58.62
231874	'Ccz1'	3.61	0	1.61	22	0.68	4.19	4.81	2.95	2.91	0
231876	'Lmtk2'	0.33	0.46	1.44	0.02	0.14	1.05	3.27	2.11	1.88	0.62
231887	'Pdap1'	29.76	20.32	36.1	24.7	39.3	23.76	28.8	28.35	39.32	26.28
231889	'Bud31'	37.15	63.68	61.4	79.2	41.9	52.38	53.8	59.24	21.44	42.87
231912	'Katnal1'	16.91	17.15	25.1	16.7	23.4	3	7.2	15.23	17.92	13.85
231915	'Uspl1'	12.22	12.3	18.6	19.8	14.2	3.88	6.7	14.31	2.78	15.32
231931	'Gimap6'	0	14.95	0	0	0	0.34	0	1.86	0	0
231946	'Fam221a'	3.93	3.14	2.27	0	0	2.06	0	1.98	0	1.83
231986	'Jazf1'	16	21.34	17.7	9.68	9.05	4.59	9.45	8.17	8.33	3.93
231991	'Creb5'	0	1.75	3.48	0	0.76	2.28	4.12	0.84	0	0.67
231997	'Fkbp14'	3.08	18.41	6.45	7.21	10.4	7.02	20.5	11.36	17.21	24.11
231999	'Plekha8'	2.06	0.85	0.16	2.59	0	2.29	0	0.98	0	2.17
232016	'Itprid1'	0	0	0	0.01	0	0	0	0	0	0
232023	'Vopp1'	37.67	34.48	29.8	9.94	17.7	5.51	27.9	20.68	19.93	40.53
232035	'Ccser1'	1.29	0.94	0.24	1.95	0.01	0	1.7	0.22	0.6	2.37
232078	'Thnsl2'	2.08	0	6.56	0.03	9.16	0.02	0	2.51	5.79	3.43
232086	'Tmem150a'	3.49	2.06	2.19	0	3.6	3.43	0.03	0.3	0.72	0.51
232087	'Mat2a'	131.7	134.4	111	105	146	133	95.8	135.2	113.9	95.1
232089	'Elmod3'	2.59	13.05	2.14	3.64	3.96	14.73	12.3	7.19	6.42	5.51
232146	'Eva1a'	6.57	5.3	0	0	2.75	0	0	4.65	0	1.33
232156	'Slc4a5'	0	0	0	0	0	0	0	0.01	0	0
232157	'Mob1a'	2.87	0.51	1.98	0	0.03	0.56	0	0	0	0
232164	'Paip2b'	23.67	8.91	13.7	9.64	7.03	20.84	6.21	13.86	28.08	19.61
232174	'Cyp26b1'	0	0	0.01	0	0	0	0	0	0	0
232187	'Smyd5'	30.01	30.3	59	10.3	25.2	30.85	31.9	30.49	27.19	26.26
232196	'C87436'	19.15	15.88	22.1	31.2	18	15.94	3.72	10	16.08	26.85
232201	'Arhgap25'	0.01	0	0	0.02	0	2.04	0	0	0	0
232210	'Hmces'	37.11	34.93	11.3	52.7	16.5	32.59	33.4	53.25	33.4	42.36
232223	'Txnrd3'	9.2	2.54	11.4	0.02	4.14	0	12	0.97	2.61	0.05
232227	'Iqsec1'	7.1	14.25	14.3	18	14.9	4.9	6.59	5.88	9.01	15.42
232232	'Hdac11'	131.3	122.1	138	76.6	122	122.4	111	144.2	149.6	179.43
232236	'Ccdc174'	16.53	12.18	18.7	24.5	4	37.81	11.3	13.29	31.39	27.68
232237	'Fgd5'	3.43	0.75	1.11	1.35	2.68	2.42	1.65	0	1.28	0.06
232286	'Tmf1'	11.68	10.82	8.26	12.1	11.8	9.1	12.9	8.94	10.22	10.36

232288	'Frmd4b'	3.88	0.38	3.55	8.23	0.93	2.7	1.71	0	1.57	2
232314	'Ppp4r2'	1.85	4.62	1.73	1.59	2.13	1.99	0.5	2.76	4.65	2.88
232333	'Slc6a1'	89.51	74.65	111	56.1	47.9	15.3	106	41.96	94.41	25.17
232334	'Vgll4'	2.82	5.24	5.36	0	8.2	6.54	2.21	1.93	3.27	2.19
232337	'Zfp637'	41.21	67.1	59.2	21.7	69.2	84.78	74.5	114.2	65.24	91.28
232339	'Ankrd26'	4.62	7.11	3.85	9.75	8.93	18.77	9.02	6.58	7.82	12.15
232341	'Wnk1'	4.65	3.59	1.35	4.62	4.15	0.79	4.12	2.43	2.23	1.72
232345	'A2m'	0	0	0	0	1.31	0	0	0	0	0
232370	'Clstn3'	75.88	143.8	177	35.7	133	78.32	130	133.4	225.1	97.22
232400	'A2ml1'	1.32	2.77	4.52	0.56	1.03	1.15	0.88	4.04	0.89	0.88
232406	'BC035044'	0	0	0	0	0	7.48	0	0	2.95	0.02
232408	'Klrb1f'	0	0	0	0	0	0	0	0.12	0	0
232430	'Crebl2'	12.67	5.76	7.78	3.76	7.01	4.65	6.29	9.51	11.5	12.24
232431	'Gprc5a'	0	0	0	0	5.94	0	0	0	0	0
232440	'H2afj'	27.92	15.24	23.1	30.8	28.9	26.68	24	28.6	17.41	17.61
232441	'Rerg'	27.53	45.22	4.68	3.66	14.2	20.76	9.57	37.73	5.79	5.05
232449	'Dera'	0.34	0	3.02	23	0	4.2	0	2.8	0	2.01
232491	'Pyroxd1'	9.97	14.37	13.8	22.2	12.9	15.89	12.4	8.96	10.95	15.74
232493	'Gys2'	0	0	1.11	0	0	0	0	0	0	0
232533	'Stk38l'	5.91	5.52	6.62	7.71	8.25	3.77	2.84	6.06	11.81	7.98
232536	'Mrps35'	48.44	30.96	32.7	12.2	25.5	51.53	12.9	19.31	12.21	29.72
232539	'Klhl42'	2.91	1.85	1.5	0.22	1	1.64	1.63	1.23	1.3	0.38
232560	'Caprin2'	0.14	1.41	1.44	1.83	5.82	5.23	5.6	1.44	1.4	7.57
232566	'Amn1'	45.98	39.73	16.5	25.1	21	20.45	24.8	22.12	22.37	29.29
232664	'Ccdc136'	8.46	12.5	6.72	37.7	14.6	11.85	2.42	12.27	19.32	4.52
232670	'Tspan33'	6.18	5.95	6.16	7.53	1.65	2.75	9.6	7.22	11.53	1.97
232679	'Zc3hc1'	36.18	24.71	23.4	28.3	33	21.17	36.8	21.64	41.15	23.91
232680	'Cpa2'	22.41	12.94	6.44	35.9	21	12.15	15.1	20.33	6.03	27.37
232748	'Tcaf2'	0	0.06	0	0	0	0	0	0	0	0
232784	'Zfp212'	1.49	2.15	0.16	1.59	8.61	10.38	0.02	4.27	3.67	4.87
232790	'Oscar'	0	0	1.66	0	0	0	0	0	0.57	0
232791	'Cnot3'	4.89	10.08	9.92	2.44	8.47	7.32	13.4	11.6	19.09	13.36
232798	'Leng8'	21.67	15.31	19.9	2.88	17.5	10.55	17.8	11.73	15.17	10.52
232807	'Ppp1r12c'	6.73	4.11	2.16	30.9	6.09	7.43	6.13	1.77	6.5	2.39
232811	'Kmt5c'	8.99	7.77	5.66	0	2.51	13.86	11.1	3.69	16.9	10.44
232813	'Shisa7'	3.52	2.09	3.43	3.35	0.48	0.16	1.51	0.61	2.23	1.57
232816	'Zfp628'	0.8	0.6	0.01	0	0	1.3	1.8	0.59	0.31	0
232821	'Ccdc106'	37.4	27.09	27.5	0	26.5	34.66	23.8	46.12	20.72	45.2
232827	'Nlrp2'	0	0	0.21	0	0.34	0	0	0.28	0.2	0.28
232853	'Zfp954'	10.24	3.23	13.1	1.79	15.7	0.04	20.2	12.82	18.42	7.39
232854	'Zfp418'	0	0.09	0	0	2.63	0	0.77	3.08	0	2.11
232855	'Zfp772'	3.61	6.36	2.15	27.1	0	1.6	2.28	5.59	3.39	9.8
232875	'Zscan18'	24.26	20.76	24.2	8.77	20	10.59	15.9	17.62	20.2	25.82
232878	'Zscan22'	9.75	10.31	4.58	0.09	0.02	0.88	0.03	3.07	16.98	1.51
232879	'Zbtb45'	3.39	3.19	4.92	10.2	9.54	2.6	5.73	4.44	11.12	8.79
232889	'Pla2g4c'	0	0	0	0	0.53	0	0	0	0.01	0.01
232906	'Arhgap35'	3.84	3.57	4.9	1.65	5.06	0.29	3.94	2.79	4.65	1.7
232910	'Ap2s1'	482	429.3	532	630	445	786.3	557	574	462.8	443.69
232933	'Ccdc61'	9.95	13.4	5.48	0.26	4.67	21	11	11.5	13.64	8.26
232934	'Mypop'	1.48	3.09	2.28	2.54	2.08	1.41	8.31	1.69	7.61	2.53
232941	'Ppm1n'	0	0.16	0	0	0	3.22	0	0	3.85	5.72
232943	'Klc3'	3.36	2.55	0.67	10.7	19.8	0	0.06	0	7.93	10.47
232944	'Mark4'	1.27	2.2	2.89	4.07	4.14	0	5.54	1.9	0.04	1.52
232946	'Bloc1s3'	4.92	1.13	1.73	6.03	3.26	0.19	3.23	0.01	0.56	0.32
232947	'Ppp1r37'	3.74	5.19	3.66	3.13	2.02	0	9.62	3.81	11.66	4.97
232966	'Zfp114'	0	0.18	1.43	0.11	0	0.06	3.93	1.15	0.01	0
232969	'Zfp428'	80.85	64.4	51.8	85.1	82.2	98.07	63.5	37.73	68.33	52.18
232970	'Phldb3'	0	0	0	0	0	0	0	0	0.02	0

232973	'Lypd4'	0	0	0	0	0	0.43	0	0	0	0
232974	'Gm4881'	0	0	0	0	0	0	19.2	0	0	0
232975	'Atp1a3'	572.8	460.9	911	144	560	281.8	726	677.8	435.5	578.99
232976	'Zfp574'	4.19	6.08	8.96	13.9	7.48	3.33	6.64	4.98	2.83	4.92
232983	'Cxcl17'	0	0	0.6	0	0	0	0	0	1.08	0
232984	'B3gnt8'	0	0	0	0.77	0.03	0	0	0	0	0
232987	'B9d2'	70.72	51.17	39.4	70.2	80	110.6	11.9	57.26	44.1	59.84
232989	'Hnrnpul1'	7.31	4.37	3.37	5.12	3.22	1.42	6.4	2.95	10.53	3.17
233011	'Itpkc'	0.01	2.35	0	19.7	0	2.34	3.72	0.28	0	5.38
233016	'Blvrb'	8.31	2.21	9.9	0	12	19.32	0	5.65	14.52	15.55
233020	'Hipk4'	0.44	0.35	0.29	5.14	0.35	3.18	6.72	1.38	2.63	0
233033	'Samd4b'	1.11	2.28	4.2	4.35	0.15	3.17	6.51	2.82	4.37	1.67
233040	'Fbxo27'	0	8.33	3.9	5.29	4.31	1.36	10.9	10.71	5.81	11.68
233046	'Rasgrp4'	0.08	1.13	1.01	0.02	0.27	1.02	1.06	0.64	1.3	0.37
233056	'Zfp790'	6.69	5.95	5.6	5.1	4.92	1.2	7.34	1.23	3.78	12.01
233057	'Zfp940'	8.11	6.45	7.68	0	5.4	0.2	7.37	7.28	4.83	6.86
233058	'Zfp420'	6.3	9.01	0.29	0	4.34	2.95	4.6	8.55	4.78	1.28
233060	'Zfp382'	3.21	4.47	2.86	0.16	1.57	1.37	0.13	4.92	0.92	0.25
233064	'Wdr62'	0	0.02	0.02	0	0	0	0.03	0	0.01	0
233065	'Alkbh6'	90.48	109.7	98.4	139	144	123.1	81.2	145.1	144.9	132.17
233066	'Syne4'	4.17	0.63	0.47	0	6.88	1.79	1.17	0.15	8.68	5.62
233067	'Lrfn3'	1.98	2.75	4.55	3.26	5.74	1.6	2.27	0.06	1.34	2.09
233071	'Arhgap33'	8.26	3.57	7.44	0.07	11.2	7.54	10.6	5.49	5.42	13.89
233073	'U2af1l4'	43.96	53.37	66.8	67.9	108	58.53	36.8	58.98	77.37	84.15
233079	'Ffar2'	0	0	0.02	0	0	0	0	0	0	0
233103	'4931406P16Rik'	2.44	0.87	7.07	2.99	1.55	2.65	3.72	0.59	3.8	4.3
233107	'Kctd15'	5.65	3.31	0.07	0	1.91	0	6.42	0.02	2.38	2.18
233115	'Dpy19l3'	0.8	3.59	1.69	2.77	0.04	0.65	2.42	2.34	3.5	4.74
233147	'Zfp939'	1.98	0.73	1.07	0	5.96	3.04	0.03	7.68	7.03	6.52
233168	'AI987944'	7.59	8.05	2.16	0	8.04	5.78	2.18	4.65	9.09	6.98
233189	'Ctu1'	5.81	7.85	0	3.23	7.3	0	0.83	5.63	0	4.26
233204	'Tbc1d17'	41.89	39.12	57.2	29.2	39	38.73	27.1	40	60.5	29.79
233208	'Scaf1'	3.34	4.06	12.4	0.84	2.94	1.3	2.85	4.43	3.68	7.05
233210	'Prr12'	1.16	0.84	2.62	3.49	0.7	1.88	2.41	1.08	0.87	2.24
233231	'Mrgprb1'	0	0	0	0.03	0.02	0.02	0.01	0	0	0
233246	'Ano5'	0.23	1.85	0.73	2.67	2.59	2.18	1.01	0.21	0.43	1.44
233271	'Luzp2'	12.5	5.97	14.4	3.48	28	9.23	13.4	7.59	3.07	9.83
233274	'Siglech'	0	1.07	0	16.7	10.7	26.69	3.78	0.45	4.32	0
233276	'Tubgcp5'	21.34	9.88	16.2	4.18	17.3	3.97	13.8	12.24	18.82	7.18
233280	'Nipa1'	6.66	5.19	2.41	4.64	1.55	0.68	6.02	9.07	3.93	3.63
233315	'Mtmr10'	2.05	0.66	0.6	2.5	0.06	1.39	6.24	0	4.52	1.33
233332	'Adamts17'	0.19	0.01	0.04	0	1.05	0	1.71	0.16	0	0.84
233335	'Synm'	1.34	1.7	1.44	4.4	5.75	0.19	1.53	0.82	0	0
233405	'Vps33b'	12.4	18.97	11.7	12.8	6.01	12.56	23.4	16.91	11.11	13.45
233406	'Prc1'	0	0.06	0.23	0	0.6	0	0	0.4	4.34	0
233410	'Zfp592'	0.8	0.95	1.15	2	0.69	0.07	5.28	0.16	3.55	0.86
233424	'Tmc3'	0.97	0	0.01	0	1.8	0.03	0	1.46	0	0.02
233489	'Picalm'	8.87	6.29	4.87	20.4	5.64	9.41	3.77	3.23	3.38	4.84
233490	'Crebzf'	3.75	3.29	1.35	0.14	3.64	4.62	2.77	1.01	2.24	1.9
233529	'Kctd14'	0.02	0.12	0	0	0	0.04	0	0.04	0	0
233532	'Rsf1'	5.24	0.81	2.63	5.49	4.51	3.05	3.93	3.89	3.58	1.42
233537	'Gdpd4'	0.01	0	0	0	0	0	0	0	0	0
233545	'Emsy'	4.18	11.86	7.73	4.4	4.6	5.5	14.6	4.79	5.67	8.05
233552	'Gdpd5'	2.47	1.42	1.91	1.07	2.07	0	2.99	0	4.67	0.01
233571	'P2ry6'	0.02	0	0.03	0	0	13.5	0	0	0	0
233575	'Pgap2'	6.92	4.5	5.93	2.4	14	4.58	9.13	5.83	9.7	10.57
233649	'Cnga4'	0.58	0.36	0	0	3.62	0.88	0	0	3.84	1.12
233651	'Dchs1'	0.84	1.13	0	0.74	1.06	0.67	2.17	0.92	1.21	0.95

233724	'Tmem41b'	14.84	16.65	24.7	30.9	13	23.57	20.5	14.42	18.63	16.98
233726	'Ipo7'	8.78	2.73	14.8	10.5	7.36	5.57	13.1	6.44	10.24	11.58
233733	'Galnt18'	17.53	29.88	6.28	25.4	29.9	10.74	12.5	11.24	3.44	40.32
233744	'Spon1'	58.7	9	14.4	8.52	15.3	5.46	9.94	59.53	15.72	12.33
233752	'Insc'	0	0	0	0	0.15	3.57	4.37	0	0	0
233765	'Plekha7'	3.97	7.97	6.05	2.02	5.35	1.49	4.89	2.48	2.26	4.04
233781	'Xylt1'	0	0	1.13	0	0	0	0	0	0.01	0
233789	'Smg1'	1.85	1.85	2.85	3.34	3.02	2.13	3.92	0.83	1.52	1.66
233799	'Acsm2'	0	0	0.01	0	0	0	0	0	0	0
233802	'Thumpd1'	66.33	57.86	52.1	86.7	39.8	78.65	36.6	43.76	52.6	57.55
233805	'Dcun1d3'	3.18	1.95	1.07	4.75	0.22	0.03	2.95	1.55	5.6	3.22
233806	'Tmem159'	17.06	13.9	11.8	15.7	13	22.43	6.85	19.67	42.45	33.24
233812	'Mosmo'	4.92	4.29	4.94	5.19	6.58	1.35	0.26	3.27	2.54	8.05
233813	'Vwa3a'	8.69	2.49	11	1.57	4.84	0.02	0.01	2.53	0	7.54
233824	'Cog7'	17.96	46.36	47.7	48.1	45.2	38.12	35.3	32.85	26.77	49.41
233826	'Palb2'	0	0	0.03	0	0	0.32	0.35	0.13	0	0
233833	'Tnrc6a'	3.02	7.38	7.34	11.3	11.9	7.42	8.23	6.96	8.72	6.16
233836	'Slc5a11'	3.07	0.5	0	0	0.06	0	0	0	0	2.8
233863	'Gtf3c1'	8.99	7.93	19.6	16	7.8	8.86	8.46	6.05	11.12	7.25
233865	'D430042O09Rik'	1.9	2.33	2.04	4.43	0.14	1.61	0.34	1.26	3.95	3.01
233870	'Tufm'	56.76	40.34	82.5	48.6	65.5	60.75	66.5	60.89	88.71	53.17
233871	'Atxn2l'	9.87	7.79	11.3	19.1	8.62	4.14	7.3	9.43	11.6	4.25
233875	'Ino80e'	48.33	37.35	43.9	51.3	28.6	61.57	42.8	46.24	61.52	69.49
233876	'Hirip3'	9.06	8.6	10.2	8.8	4.02	17.94	5.23	10.06	12.34	8.28
233877	'Kctd13'	56.27	51	49.7	40.2	58.7	28.67	32.3	34.24	52.73	25.12
233878	'Sez6l2'	42.7	42.71	44.7	66.4	29.4	34.46	30.6	51.16	43.43	33.51
233879	'Asphd1'	16.3	7.11	17.3	11.9	10.3	5.21	11.6	13.72	8.8	15.49
233887	'Zfp553'	11.94	4.03	8.8	0.93	9.97	1.97	16.2	7.37	5.04	2.62
233890	'Zfp768'	0.04	0.11	2.65	0	0	3.95	0	4.88	0	4.9
233893	'Zfp764'	3.19	2.12	2.49	1.47	0.16	9.9	0.08	2.46	5.25	0.29
233895	'Prr14'	11.33	15.53	13.2	24.9	11.3	13.03	14.5	18.45	16.59	16.33
233899	'Ccdc189'	10.63	15.68	11	18.2	10.7	12.82	3.84	9.39	13.93	14.91
233900	'Rnf40'	6.07	10.41	3.97	15.6	8.83	1.95	8.38	6.76	9.14	15.06
233902	'Fbxl19'	1.75	0.71	3.16	0	3.6	5.49	1.63	0.86	0.14	3.38
233904	'Setd1a'	1.64	2.42	4.85	9.94	0.43	1.88	3	2.82	3.62	4.09
233905	'Zfp646'	0.31	0.61	0.02	1.92	1.38	0	0	1.25	0.38	1.47
233908	'Fus'	76.52	62.17	124	72.3	83.7	40.17	89.5	68.07	105.8	28.86
233912	'Armc5'	1.25	0.24	5.39	0	3.31	2.08	3.9	2.12	0	3.78
233913	'BC017158'	17.44	17.6	13.9	26.1	12.5	19.3	38.8	17.39	10.09	9.16
233919	'Gpr26'	4.76	2.03	0.51	0.02	0.84	5.78	0.02	0.02	0	2.78
233977	'Ppfia1'	0.03	2.75	2.79	1.19	4.36	2.95	0.54	5.53	3.97	3.42
233979	'Tpcn2'	0	5.03	1.92	0	2.14	3.05	0	3.3	0.3	0
233987	'Zfp958'	3.89	2.07	6.9	0	3.97	6.67	0	4.26	1.57	5.59
234023	'Arglu1'	93.85	86.98	102	125	169	33.16	61.1	63.94	102.9	117.71
234069	'Pcid2'	11.6	23.03	14	8.06	5.94	28.9	8.01	31.24	6.79	14.26
234072	'Adprhl1'	0.45	0.52	1.09	1.08	1.53	1.26	0.47	1.19	2.32	1.2
234076	'Tmco3'	13.46	29.52	32	35.4	12.1	25.5	38.9	27.93	27.51	17.3
234086	'Erich1'	10.62	10.92	5.76	24.3	14.5	16.23	5.94	5.17	14.16	6.16
234094	'Arhgef10'	1.58	0.55	0.54	0.66	1.99	3.37	0.29	0	0.45	0.72
234129	'Tpte'	0	0	0.03	0	0	0	0	0	0	0
234130	'Dkk4'	0	0	0.52	0	0	0	0	0	0	0.1
234135	'Nsd3'	30.19	31.45	11.2	40.4	33.4	26.64	18.2	25.31	27.6	30.02
234138	'Tti2'	7.56	9.04	14.2	14.2	3.36	7.58	12.6	8.26	16.21	14.15
234155	'Mboat4'	1.62	0.08	0	0	2.02	0	0.11	0	2.35	5.74
234199	'Fgl1'	0	6.36	1.68	16.2	0	11.28	0	0	0	3.03
234214	'Sorbs2'	8.4	4.65	13.9	6.87	7.2	9.23	10.4	11.24	5.24	18.17
234219	'Helt'	0	0	0.11	0	0	0	0	0	0	0
234267	'Gpm6a'	430.5	507.2	397	335	300	276.6	526	451	310.6	391.33

234290	'BC030500'	18.89	5.2	3.72	11.3	6.47	8.27	16.1	18.61	3.51	22.2
234309	'Cbr4'	22.1	14.89	5.01	14	15.7	12.69	0.09	17.34	8.42	6.74
234311	'Ddx60'	0	0	0	16.6	2.69	0	0	0	0.01	0
234344	'Naf1'	0.06	0.64	0.03	0.79	1.93	0.03	0.1	0.41	0.1	0.04
234353	'Psd3'	4.51	1.37	5.24	3.33	4.51	1.8	1.51	2.95	3.5	2.08
234356	'Csgalnact1'	4.59	10.13	5.35	22.2	1.09	7.4	1.4	6.55	10.02	6.57
234358	'Zfp930'	10.68	7.64	4.37	5.06	1.17	2.88	1.57	2.71	4.43	10.03
234362	'Zfp868'	12.96	6.47	14.3	3.09	5.51	28	5.8	21.4	0.02	12.8
234366	'Gatad2a'	3.92	4.68	3.5	13.5	3.26	3.08	0.03	3.58	4	8.37
234371	'Tmem161a'	32.61	31.84	32.9	34.7	25.8	33.23	10.7	49.69	15.67	22.05
234373	'Sugp2'	17.98	13.52	26.5	14.7	11.3	5.93	30.7	17.16	18.4	10.71
234374	'Ddx49'	19.82	8.79	21.5	23.8	22.3	22.51	17.3	11.9	7.71	14.98
234378	'Klhl26'	29.5	26.77	26.3	22	31.5	9.96	43.2	34.56	13.56	28.61
234384	'Mpv17l2'	32.12	27.16	23.6	0.08	17.8	38.19	17.8	8.92	23.55	55.26
234388	'Ccdc124'	179.8	143.8	148	112	285	267.4	266	183.1	150.8	214.58
234395	'Ushbp1'	0	0	0.12	0	0	0	0	0	0	0
234404	'Nxnl1'	0	0	1.07	0	0	0.83	0	0	0	0
234407	'Colgalt1'	19.49	23.32	30.4	45.4	16.7	3.37	27.1	10.45	36.7	8.96
234413	'Zfp961'	4.92	8.84	6.14	4.66	2.5	0.83	4.28	4.74	1.92	6.6
234463	'Tmem184c'	31.21	35.64	29	27.3	30.7	44.5	32.6	37.16	35.82	46.29
234515	'Inpp4b'	0.37	7.44	2.01	10.5	0.78	0.02	4.09	12.36	0.01	2.89
234542	'Rtbdn'	0.03	0.65	3.12	0	4.87	0.03	0	1.82	4.31	0.74
234549	'Heatr3'	6.19	14.89	21.7	24.4	14.5	4.69	16	9.51	4.2	8.51
234577	'Cpne2'	22.15	18.96	20.2	9.23	22.7	15.32	13.4	23.09	16.96	6.68
234582	'Ccdc102a'	2.36	0	0	0	0	0	0	0	0	0
234593	'Ndrg4'	709.7	803.6	1004	467	703	881.4	685	1105	725.7	761.57
234594	'Cnot1'	2.1	2.83	4.29	3.67	3.54	1.14	5.95	4.33	1.68	5.01
234595	'Slc38a7'	16.1	14.69	9.72	11.2	12.9	9.46	7.09	23.5	17.77	10.63
234663	'Dync1li2'	32.01	21.37	40.5	27.8	33.4	15.84	24.8	21.72	20.52	12.04
234664	'Nae1'	45.5	45.41	70.4	51.7	39.2	49.26	73.3	49.25	88.19	59.91
234669	'Ces2b'	9.35	9.36	13	21.3	14.5	12.8	12.4	7.51	14.18	9.67
234677	'Ces4a'	0	0.29	0	0	0	0	0	0	0.07	0
234678	'D230025D16Rik'	16.9	24.5	31.1	13.4	26.4	34.82	47.1	25	22.78	15.22
234683	'Elmo3'	2.18	2.12	10.2	0	3.52	6.88	0.18	1.62	3.08	3.27
234684	'Lrrc29'	0.02	0	1.2	0.06	1.39	0.02	0.05	4.32	0	7.4
234686	'Fhod1'	0.08	0.01	1.46	0	0	0	0	0.04	1.83	0
234695	'Carmil2'	12.04	5.98	9.01	12	6.48	2.13	7.96	11.12	8.08	3.76
234699	'Edc4'	12.64	8.5	29.4	33.3	21.7	3.39	14.7	18.89	14.66	8.65
234700	'Nrn1l'	0	13.81	1.69	27.5	8.96	12.89	0	2.63	0.08	11.18
234723	'Txnl4b'	26.08	10.68	9.88	34.3	42.4	34.34	8.77	28.05	14.41	31.5
234724	'Tat'	0	1.59	0	0	0	0	0	0	0	0
234725	'Zfp612'	40.23	39.15	36	30.5	46.1	52.74	25.9	49.96	30.77	50.36
234728	'Cmtr2'	2.23	0.61	3.91	0	2.04	2.39	3.81	3.38	2.89	5.41
234729	'Vac14'	1.98	5.26	2.61	9.56	5.59	0.03	4.68	2.55	0	1.4
234730	'Fuk'	0.85	1.03	5.31	0	0.02	4.61	0	1.38	3.25	0
234733	'Ddx19b'	0	1.03	0.04	2.78	0.09	2.97	7.77	0	1.04	0.1
234734	'Aars'	53.94	60.97	65.5	39.4	59.8	36.55	63	65.1	96.35	49.52
234736	'Rfwd3'	4.39	2.93	6.18	3.1	5.29	0.06	1.61	5.16	1.56	3.77
234740	'Tmem231'	12.17	8.86	2.94	0.86	5.44	2.79	15.4	11.28	16.07	4.86
234776	'Atmin'	5.01	6.05	1.7	11.9	9.94	6.06	4.24	3.14	9.38	4.68
234779	'Plcg2'	0.23	0.14	1.36	0.93	0	0	0.34	0.02	0.01	0
234788	'Slc38a8'	0	0.04	0	0	0	0.02	0	0.06	0	0
234796	'Klhl36'	4.52	9.64	6.37	0.05	7.11	11.44	0	8.73	4.35	9.97
234797	'6430548M08Rik'	7.91	10.37	18.7	12	8.49	3.27	18.5	2.76	11.69	10.63
234814	'Mthfsd'	27.55	22.98	23.9	20.7	36.9	25.64	32.5	36.34	37.17	33.06
234825	'Klhdc4'	38.26	12.02	15.8	9.48	18.3	39.52	14.4	20.32	28.93	7.54
234836	'Il17c'	0	2.37	0	11.3	0	0	0	0	0	0
234839	'Piezo1'	0	0.02	0	0	0	0	0	0	0	0.01

234847	'Spg7'	7.31	5.17	8.47	8.97	13.9	2.91	17.4	5.59	10.31	1.9
234852	'Chmp1a'	88.7	66.87	85.8	36.8	61.2	59.95	66	76.26	70.6	83.7
234854	'Cdk10'	53.74	44.14	44.5	55.5	35.9	50.53	41.8	59.42	38.31	66.09
234857	'Spire2'	0.02	0.1	4.17	0	8.96	0.43	0	0.74	0.05	0.38
234865	'Nup133'	8.46	6.64	12.6	2.11	5.21	3.99	14.1	5.09	4.78	10.85
234875	'Ttc13'	10.36	21.42	26.4	11.7	7.48	6.9	25.6	13.01	34.87	21.11
234878	'Map3k21'	0	0	0	0.01	0.03	0	0.01	0	0	0
234889	'Gucy1a2'	0.53	0.44	2.08	2.64	0.23	2.97	3.45	0.84	2.49	0.92
234912	'Cfap300'	16.33	9.34	2.36	0.6	6.36	0.24	0.97	8.78	7.24	11.22
234915	'Cep126'	0.42	0.38	0.04	3.57	3.32	1.78	0	0.37	1.48	1.79
234959	'Med17'	0.52	8.63	5.96	6.79	3.24	1.3	7.83	6.11	7.61	6.31
234964	'Deup1'	2.02	2.33	0.73	0	0.15	2.15	0.48	1.88	8.74	2.63
234967	'Slc36a4'	16.98	6.17	16.1	15.3	3.29	11.96	35.6	13.09	18.45	11.85
235028	'Zfp426'	14.68	3.97	10.3	5.44	15.3	0.8	4.64	11.8	7.86	9.06
235036	'Ppan'	21.07	14.2	17.1	29.3	2.5	15.55	18	10.23	26.29	25.01
235040	'Atg4d'	23.56	24.59	21.6	6.78	19.8	12.17	8.02	34.05	21.9	12.64
235041	'Kank2'	0	0.34	0.02	0	0	0	1.9	1.43	0	0
235043	'Tmem205'	74.73	47.15	59.5	13.5	44.3	66.78	29.3	62.05	38.47	65.39
235044	'Plppr2'	36.84	45.45	63.7	34.9	25.1	32.56	41.2	29.51	29.47	18.5
235047	'Zfp809'	10.44	2.68	1.51	7.61	3.47	9.24	6.5	2.88	0.44	5.99
235048	'Zfp599'	3.36	1.23	1.76	0.12	1.48	0.03	3.56	2.75	4.34	2
235050	'Zfp810'	1.55	1.47	5.64	0	3.71	1.25	5.52	4.51	1.26	4.68
235072	'Sept7'	139	180.4	109	99.1	139	151.6	158	136.2	154.1	171.28
235086	'Igsf9b'	4.25	3.55	5.12	4.56	8.77	7.72	1.64	5.02	7.79	5.33
235106	'Ntm'	94.58	128.3	160	68	146	130.5	99.7	105.3	72.98	95.29
235130	'Adamts15'	0	1.77	0.01	0	0	0	0	0.64	1.03	0.52
235132	'Zbtb44'	0.3	0.75	0.71	0.09	0.34	0.39	0.9	0.82	0.35	0.33
235134	'Nfrkb'	6.48	4.15	10.6	2.52	9.26	3.98	6.42	4.05	5.32	7
235135	'Tmem45b'	0	0	5.4	0	20.5	0	0	0	0.04	0
235169	'Foxred1'	22.11	33.31	41.6	40.8	28.5	22.92	35	47.13	28.75	17.65
235180	'Fez1'	204.8	211	197	228	209	239.3	303	291	198	188.35
235184	'Msantd2'	2.83	6.8	2.14	3.79	4.56	0.2	1.36	2.29	0.79	4.03
235281	'Scn3b'	41.03	40.69	27.9	19.3	46.5	6.79	54.3	20.26	33.24	23.25
235283	'Gramd1b'	3.94	5.38	2.44	2.29	0.81	2.5	5.03	4.01	4.15	3.32
235293	'Sc5d'	33.35	27.83	46	32.6	32.8	35.43	62	62.14	56.54	51.61
235300	'Tmem136'	4.46	5.24	2.47	2.11	3.37	0.82	0.61	1	1.86	2.46
235302	'D630033O11Rik'	0	0.02	0	0	0	0	0	0	0	0
235312	'C1qtnf5'	6.19	3.93	0	18.8	1.56	0	0.03	0.26	0	2.13
235315	'Rnf214'	16.94	22.34	23.2	23.8	15.8	13.93	13.8	16.1	10.05	14.42
235320	'Zbtb16'	0.02	4.73	2.96	0.06	0.36	1.85	2.29	0.96	0.01	0.12
235323	'Usp28'	4.82	3.65	2.82	5.85	0.56	0.06	3.57	3.48	7.48	0.41
235330	'Ttc12'	1.25	1.27	0.52	0	0	0	0.8	2.67	0.02	0
235339	'Dlat'	37.06	36.52	47.1	22.3	37.9	25.11	51.2	51.68	33.38	46.63
235344	'Sik2'	2.42	2.42	5	1.66	0.09	0.63	4.08	1.76	1.91	0.6
235345	'4833427G06Rik'	2.6	0	0	0.08	0	0	0	1.43	0	0
235379	'Gldn'	0.69	4.93	3.03	10.6	1.11	1.86	0.11	0.38	0	8.1
235380	'Dmxl2'	5.27	4.99	8.97	14.8	7.41	4.46	4.7	2.64	4.59	3.12
235386	'Hykk'	0.11	1.36	0.6	0.05	1.3	0.02	0.03	0.12	0.03	1.71
235402	'Lingo1'	14.12	7.5	6.17	9.49	7.23	6.56	10.2	8.88	2.5	10.17
235406	'Snx33'	0	3.3	0	0.01	0	0.01	0	0.09	0	0
235415	'Cplx3'	0	0	5.3	0	0.02	0	0	0	0	0
235416	'Lman1l'	0	0.28	0	0	0	0	0	0	0	0
235431	'Coro2b'	9.29	15.38	14.8	9.54	8.61	1.4	5.42	8.33	12.95	8.77
235439	'Herc1'	6.71	5.18	8.09	15.1	7.79	7.98	7.95	4.3	7.28	9.53
235441	'Usp3'	3.46	7.08	3.33	8.72	3.57	4.56	11.2	10.62	5.93	7.27
235442	'Rab8b'	9.29	7.06	8.91	17.8	7.61	1.25	13.1	6.35	4.24	13.11
235459	'Gtf2a2'	78.9	67.66	117	139	82.7	68.97	50.1	82.12	49.75	81.32
235461	'Mindy2'	3.98	2.91	2.27	4.49	3.22	2.73	1.77	0.8	2.35	1.52

235469	'Zfp280d'	6.91	7.04	3.55	7.56	4.39	6.44	4.32	3.8	8.31	7.96
235472	'Prtg'	0.5	1.1	0.34	0	0.4	0	1.27	0.12	0.4	0
235493	'Fam214a'	4.05	7.58	5.16	19.8	1.74	1.05	5.12	13.09	4.75	5.2
235497	'Leo1'	11.52	38.12	22.9	58.6	31.8	36.37	32.5	41.44	37.78	27.45
235504	'Slc17a5'	11.72	15.35	9.54	10.8	3.19	11.56	2.71	18.62	14.3	10.73
235527	'Plscr4'	0	3.91	0.97	0	0	3.18	0.23	3.3	0	0
235533	'Gk5'	1.43	0.97	1.34	4.71	0	0	0	0.89	2.11	0.05
235534	'Pxylp1'	11.68	20.38	17.9	5.42	5.68	0.83	4.99	5.59	10.57	4.48
235542	'Ppp2r3a'	2.34	2.52	7.01	1.95	4.6	1.85	0.49	2.01	3.93	5.36
235559	'Topbp1'	0	0.02	0.04	0	0.01	0.02	0	0.01	0.02	0.02
235567	'Dnajc13'	0.41	0.57	0.61	2.01	1.48	1	1.42	1.31	0.78	1.74
235574	'Atp2c1'	21.32	21.14	15	7.55	14.3	6.18	7.75	10.73	26.66	3.96
235582	'Glyctk'	0	0.69	1.16	3.96	0	0.69	0	0.89	0.37	0
235584	'Dusp7'	1.22	0.02	0.21	0.18	7.58	0	9.95	1.52	2.71	0
235587	'Parp3'	0	0	0	6.43	0	3.41	2.57	1.06	0.02	3.1
235599	'6430571L13Rik'	0	1.65	0	0.02	5.07	6.68	8.82	4.26	0.38	0.43
235604	'Camkv'	111.1	100.1	92.8	29.6	73.3	69.76	47.6	76.88	45.55	26.27
235606	'Apeh'	35.84	29.47	25.9	0.02	34	25.91	31.5	12.78	47.35	28.07
235610	'Atrip'	6.08	8.4	11.4	13.7	13.1	8.86	17.4	8.22	20.18	8.2
235611	'Plxnb1'	1.08	3.97	5.07	4.19	4.24	2.51	3.04	1.33	3.57	1.89
235623	'Scap'	10.33	11.45	7.58	21	18.6	5.61	13.5	7.1	19.57	24.75
235626	'Setd2'	1.82	5.16	4.67	8.41	5.79	1.17	4.51	1.65	2.17	3.85
235627	'Nbeal2'	0.76	2.54	1.01	0	1.15	0.01	0	0.94	1.64	0.89
235631	'Prss50'	0	0	5.94	0	0	0	0	0.42	0.24	0
235633	'Als2cl'	0.01	0.07	4.59	0.64	0.01	0.07	0.01	2.21	4.14	0.97
235636	'Rtp3'	0	0	0	0.02	0	0	0	0.02	0.02	0
235661	'Dync1li1'	40.17	67.28	50.2	36.9	27.9	22.89	68.4	42.03	42.68	45.6
235674	'Acaa1b'	0.36	0	0	0	0	5.56	0	2.24	0	0.04
235682	'Zfp445'	11.79	9.27	10.3	17.3	11	5.24	12.4	6.29	3.17	13.72
235907	'Zfp65'	8.76	8.33	11.5	21.3	15.8	10.66	11.3	10.98	12.89	8.98
235956	'Zfp825'	15.46	9.19	8.51	12.8	11.4	8.55	7.63	17.06	7.42	10.6
236082	'Dhrsx'	1.12	1.42	3.44	2.59	1.64	2.32	0.31	2.54	6.49	0.42
236193	'Zfp709'	0	0	1.78	0	0.1	0	0	0.12	2.45	1.78
236266	'Alms1'	0.35	0.21	3.62	0.82	0.35	0.01	0.92	0.07	0.45	1.05
236285	'Lancl3'	4.73	0	0	0	3.18	0.16	0.48	4.14	7.92	0
236293	'Slc22a29'	0	0	0.04	0	0	0	0	0	0	0
236366	'5730507C01Rik'	0.05	0.08	0.41	2.58	0.52	1.2	0.43	0.06	1.5	0.69
236451	'Phf11b'	0	0	0	0	4.56	1.64	0	0	0	0.07
236511	'Ago1'	0.66	1.45	3.92	1.61	0.42	1.83	0.24	0.4	2.22	0.37
236539	'Phgdh'	54.66	28.28	27.4	26.6	9.27	94.83	14.2	28.2	35.54	10.59
236573	'Gbp9'	0	0	0	0	3.16	0	0	0	0	0
236574	'Smok2b'	0	0	0.28	1.48	0.29	0	0	0	0	0
236576	'Spry3'	3.83	1.49	2.56	0.23	0.17	0.02	8.11	1.49	1.89	0.02
236643	'Sytl5'	0	1.95	0.97	0.12	1.33	0	1.15	0.07	0	0
236690	'Nyx'	0.04	0.05	0.07	0.02	0.02	0	0.03	0	0	0.12
236727	'Slc9a7'	10.21	11.25	9.2	17	4.33	10.31	9.7	5.12	5.56	5.89
236732	'Rbm10'	24.51	21.36	19.1	9.65	27.2	17.85	34.4	21.91	19.96	31.07
236733	'Usp11'	150	151.1	171	94.3	96.2	91.58	125	114.2	167.2	146.7
236790	'Ints6l'	8.44	3.74	14.2	6.01	6.2	7.54	17.8	7.81	17.52	14.19
236792	'Mmgt1'	9.79	4.82	5.42	0	4.59	2	4.2	4.61	0.35	6.15
236794	'Slc9a6'	17.46	20.36	22	19.8	13.3	10.55	35.9	18.83	13.87	17.13
236798	'Adgrg4'	0	0.2	0	0	0	0	0	0	0	0
236848	'Tmem185a'	39.3	18.66	16.8	22.7	31.4	22.85	16	23.97	25.19	55.29
236899	'Pcyt1b'	3.3	4.56	4.37	0	5.12	1.09	7.67	2.88	3.35	1.01
236900	'Pdk3'	18.49	9.41	22.1	0	16.9	28.74	21.9	6.24	15.94	15.45
236904	'Klhl15'	1.59	0.55	2.41	3.73	1.32	0.06	4.11	1.75	0.03	1.63
236915	'Arhgef9'	32.68	15.94	30	23.4	22	12.15	14.1	16.74	23.13	14.96
236920	'Stard8'	0.75	0.68	0	0	0	0	0	0	0	0

236930	'Ercc6l'	0	0	0	0	0.14	0	0	0	0	0
237010	'Klhl4'	2.84	4.66	1.88	0	0	1.74	0	2.72	2.43	3.34
237029	'4932411N23Rik'	0.01	0	0.01	0.02	0.01	0	0	0	0.03	0
237038	'Nox1'	0	0	0	0	0.02	0	0	0	0	0.24
237052	'Tceal1'	72.9	94.35	61.5	102	65	76.93	55.8	84.48	57.94	91.12
237073	'Rbm41'	0.03	0.01	0.04	0.11	0.67	0.36	0	0.01	0.92	0.39
237082	'Nxt2'	18.15	12.81	23.3	6.59	15.8	15.42	19.2	18.09	24.43	17.38
237107	'Gnl3l'	117.6	115.9	123	106	76.7	75.91	116	108.1	87.08	96.28
237175	'Adgrg2'	0	0	0.85	1.84	0.11	3.01	1.51	0.87	1.42	0
237178	'Ppef1'	0.05	0.04	0	2.1	0	0	0	3.69	0	0
237211	'Fancb'	0	0	0	0	0	0.07	0	1.26	0.7	0.2
237213	'Glra2'	32.74	11.99	16.2	12.7	27.9	11.81	26.3	49.75	8.53	10.61
237221	'Gemin8'	15.9	10.31	12.6	10.6	4.03	15.64	3.34	7.16	3.12	13.8
237222	'Ofd1'	1.32	1.29	0.64	2.45	1.33	3.78	0	0.7	6.21	1.16
237253	'Lrp11'	19.85	17.39	15.1	23.3	17.6	9.79	15.9	10.94	29.87	6.93
237300	'Gm4922'	0	0	1.45	0	0	0.04	0	0.02	0	0.29
237320	'Aldh8a1'	0	0	0	2.05	0	0	0	0	0	0.02
237336	'Tbpl1'	39.7	41.73	42.8	30.6	46.5	22.01	35.2	17.37	43.95	27.71
237339	'L3mbtl3'	2.82	7.97	1.26	2.05	1.44	0.01	3.27	6.55	2.54	3.66
237353	'Sh3rf3'	0.2	0.98	0	0.73	0	0	0.01	0.04	0.09	0
237360	'Adamts14'	0	0	0	0	0	0.07	0	0	0	0
237362	'Npffr1'	0.09	4.92	3.43	0.27	11.3	0.07	2.3	4.19	0.04	2.33
237387	'Lrrc3'	1.82	1.85	5.06	1.33	0.22	3.75	2.46	0.02	0.25	0.19
237397	'C2cd4c'	0.01	1.77	0.29	0.5	0.01	0	2.52	1.77	0.02	0.17
237400	'Mex3d'	1.48	0.22	1.66	11.7	1.35	0	0	0.21	0.01	0
237403	'Lingo3'	6.45	3.67	9.31	0	12.8	1.16	14.6	5.41	5.72	0.29
237411	'Zfp938'	5.39	12.89	5.9	4.55	15.8	8.11	9.63	12.42	11.78	16.43
237412	'Gm4924'	4.62	8.34	1.91	0.09	5.57	0.08	3.38	3.7	3.46	1.72
237422	'Ric8b'	18.18	24.08	19.4	26.3	7.28	4.28	13.7	17.98	17.81	13.06
237433	'Gm4925'	0	0	0	0	0.29	0.38	0	1.97	0	0
237436	'Gas2l3'	0.45	0.36	0.88	1.52	1.62	0.56	0.33	0.34	1.07	0.48
237459	'Cdk17'	4.39	2.2	1.72	0.51	4.85	5.31	3.02	2.7	8.58	8
237465	'Ccdc38'	0.08	0.2	0.23	0	0.37	0.06	0.14	0.19	0.28	1.28
237500	'Tmtc3'	4.02	0.5	2.29	3.74	5.03	5.32	0.3	0.34	4.83	3.77
237504	'Rassf9'	0	0	0	0	0	0	0	0	0	0.12
237523	'Ptprq'	0.81	0	0	0	0	0	0	0	0	0
237542	'Osbpl8'	3.03	7.08	4.18	3.97	1.78	3.32	5.56	6.22	6.3	5.16
237553	'Trhde'	1.5	1.66	0.21	1.98	1.41	1.24	0.93	0	2.98	2.04
237558	'Myrfl'	0	0	0	0	0	0	0	0	0	0.01
237560	'Lrrc10'	0	1.2	0	0.08	0.03	0.07	0.04	0	0.03	0.06
237611	'Stac3'	0.97	2.88	0	2.09	0.66	3.08	1.71	0	10.89	11.69
237615	'Ankrd52'	2.02	1.34	1.29	2.39	0.66	1.16	1.61	0.21	2.66	1.35
237625	'Pla2g3'	1.98	0.64	5.21	0	0	0	0	0	0.81	1.42
237711	'Eml6'	3.33	2.79	4.91	6.01	1.65	0.84	2.91	1.92	3.25	2.52
237716	'Gpr75'	3.96	2.99	6.46	4.8	5.29	0.01	4.89	1.67	2.61	2.72
237730	'Fbll1'	6.11	4.07	4.73	13	7.98	12.15	16.6	3.78	16.21	3.85
237758	'Zfp454'	0.89	1.28	6.21	0	3.24	0	0	1.59	4.64	0.14
237759	'Col23a1'	0	0.28	0	0.45	3.64	0.02	0.21	0	0	1.55
237761	'Sowaha'	7.79	1.97	4.53	3.96	7.28	4.26	4.81	0.02	0.01	1.06
237775	'Zfp867'	4	2.56	1.92	5.52	2.36	0.3	7.97	6.55	7.57	3.93
237781	'Mief2'	3.25	8.74	14.6	16.2	12.6	12.36	8.43	8.75	14.19	9.34
237782	'Smcr8'	0.01	0.78	2.67	4.17	0.11	0.01	0.15	0.02	0.75	1.06
237806	'Dnah9'	0.99	0.3	0.08	0.48	0.31	0.05	0.16	0.07	0.83	0.14
237823	'Pfas'	14.73	16.73	9.53	12.4	13.9	10.68	7.55	15.35	11.15	11.92
237831	'Slc13a5'	2.7	0	2.75	0	0	0	4.5	0	2.95	1.96
237847	'Rtn4rl1'	0.01	1.36	9.78	0	5.04	8.58	0	1.69	8.03	17.48
237858	'Trarg1'	2.74	0	0	0	1.67	0	0.02	0.01	0	0
237859	'Nsrp1'	11.28	13.49	9.44	5.6	15.8	8.03	6.55	8.83	7.82	12.75

237860	'Ssh2'	1.99	2.56	0.93	0.1	2.62	1.94	1.86	1.2	1.97	0.75
237868	'Sarm1'	8.96	8.37	8.07	7.53	8.35	5.53	6.38	6.37	3.24	7.04
237877	'Atad5'	1.43	2.72	0.14	1.9	0.5	0.37	0.01	2.18	0.39	2.5
237880	'1700071K01Rik'	0	0	3.54	0	0	0	0	0.96	0	0
237886	'Slfn9'	0.35	0.41	0.41	2.43	0.62	0.53	0.53	0.28	0.46	0.32
23789	'Coro1b'	38.18	60.42	33.2	28.3	44.5	38.96	41.1	43.04	38.81	29.69
237891	'Gas2l2'	1.21	1.26	0.44	0	0.18	0	0	0	0	0.44
237898	'Usp32'	0.89	3.86	2.23	8.97	2.31	0.05	1.56	3.09	3.62	3.55
23790	'Coro1c'	27.39	31.83	50.4	52.9	39.4	39.3	43.1	45.87	41.95	34.25
237911	'Brip1'	0.27	0.4	0.55	0.3	0.4	0.34	0.35	0.33	0.33	0.42
23792	'Adam23'	3.12	1.74	2.06	1.08	5.15	0.88	3.96	2.16	7.73	1.71
237926	'Rsad1'	2.93	2.79	1.99	0	4.84	0	9.48	1.51	7.48	2.25
237928	'Phospho1'	0	0	0	0	0.03	0	0	0.04	0	0
23794	'Adamts5'	0.73	0	0	0	0	0.24	0.04	0	0	0
237940	'Aoc2'	0.12	0.09	0.13	3.99	1.14	0.11	0.04	0.08	0.18	0.02
237943	'Gpatch8'	10.73	5.77	7.22	8.05	10.5	6.76	7.99	6.65	5.53	5.24
23795	'Agr2'	0	0.4	0	0	0	0	0	0	0	0
23796	'Aplnr'	21.2	11.4	30.5	25.2	13.1	151.1	113	252.8	102.1	233.41
23797	'Akt3'	4.72	2.75	4.33	4.13	6.71	2.34	2.71	0.38	5.14	0.91
237979	'Sdk2'	1.51	1.37	0.06	2.07	0.47	0.01	0	0.46	0.61	0.34
237988	'Cdr2l'	1.88	1.41	6.09	0	2.05	1.5	4.67	1.31	4.44	1.16
23801	'Aloxe3'	4.46	7.01	3.33	3.01	2.99	4.9	7.37	1.9	6.9	14.89
23802	'Amfr'	15.13	13.89	20.2	23.3	15	12.66	25.3	3.85	13.59	12.07
238021	'Fscn2'	0.75	0.45	0	5.21	0	0	0	0.47	0	0.32
238023	'Hexdc'	2.66	15.53	14.9	24	5.4	12.32	19.3	15.66	2.58	5.43
238024	'Fn3krp'	4.16	5.91	6.44	2.05	3.19	6.71	3.55	1.73	3.99	3.9
238037	'Wdcp'	5.37	4.73	7.22	7.64	2.21	3.73	1.63	5.67	5.26	3.37
23805	'Apc2'	8.61	7.44	12.1	9.64	8.11	3.28	4.87	7.79	7.44	15
23806	'Arih1'	1.03	3.24	5.24	4.81	0.61	1.88	4.02	2.84	0.48	2.31
23807	'Arih2'	16.61	12.98	14.8	13.7	21.2	9.19	14	12.7	13.23	16.45
238076	'Kcns3'	0.02	2.66	0.51	0	0.03	5.84	6.35	3.42	0	0
23808	'Ash2l'	11.5	4.05	7.56	42.5	6.56	11	12.2	8.82	10.7	11.33
238123	'Cog5'	8.64	6.8	14.9	15.5	0.51	6.86	2.76	12.11	13.93	4.15
238130	'Dock4'	2.58	2.09	0.66	0.12	1.46	0.2	1.29	0.2	0.33	0.29
238161	'Akap6'	14.06	11.94	15.8	12.5	11.4	9.16	11.6	9.97	12.29	14.53
238205	'Lrfn5'	24.78	18.83	27	18.9	10.2	9.35	26.6	21.42	15.93	21.56
23821	'Bace1'	16.59	14.34	14.4	9.13	6.86	10.65	14.7	7.31	12.33	10.78
238247	'Arid4a'	24.98	21.12	13.3	47.7	26.7	27.35	36.2	22.81	22.44	26.85
23825	'Banf1'	143.5	139.5	109	149	121	114	119	146.3	102.2	117.83
238252	'Gpr135'	2.68	12.37	3.31	5.28	2.08	14.46	20.7	6.24	3.94	16.52
238257	'Tmem30b'	0	0	0	0	1.54	0	0	0	0	0
238266	'Syt16'	3.52	28.66	28.2	13.3	7.63	7.99	29.9	35.41	8.35	7.61
23827	'Bpnt1'	50.66	62.88	37.1	29.7	33.7	38.57	29.8	57	25.82	50.8
238271	'Kcnh5'	1.94	2.8	3.99	0.01	5.6	0.65	1.8	2.52	6.21	4.11
238276	'Akap5'	2.21	1.8	1.11	4.77	0.77	2.89	6.19	2.43	2.62	0.79
23828	'Bves'	0	0	1.43	0	0	0	0	0.35	0	0
23829	'C1ql1'	1.23	0	0	0	3.79	9.54	0	0	5.93	3.96
23830	'Capn10'	13.15	18.66	33.3	19.1	17.1	29.52	17.9	8.09	20.69	4.02
23831	'Car14'	2.18	4.57	0.09	0.04	0.09	7.37	0	5.07	0.05	0.05
238317	'Elmsan1'	0.79	3.91	1.66	0.01	0.76	0.47	1.46	0.03	0.21	0.29
23832	'Xcr1'	0.55	0.14	0.08	0.16	0.1	0.09	0.06	0.06	0.03	0.08
238323	'Rps6kl1'	31.68	44.21	39.2	21	33	25.64	24	37.79	45.33	54.91
238328	'Vash1'	0.16	1.11	0.54	2.94	4.46	2.38	3.65	1.87	1.43	0.74
23833	'Cd52'	12.51	1.27	2.55	19.6	4.55	33.03	0	1.87	0	0
238330	'Irf2bpl'	10.35	10.01	12.6	6.53	5.97	9.33	5.56	8.11	7.05	8.79
238331	'Zdhhc22'	34.5	54.28	23.3	50.1	54.3	28.33	64.5	47.33	7.74	5.71
238333	'Samd15'	0.64	2.63	0.11	1.33	0.26	0.03	1.17	0.44	2.54	2.73
23834	'Cdc6'	0	0	0	0	0.02	0	4.94	0	0	0.02

23836	'Cdh20'	4.7	8.65	0.03	0.35	0	0.58	3.75	1.21	4.66	6.41
23837	'Cfdp1'	204.2	127.7	136	179	127	161.7	135	158.9	129.1	140.85
238377	'Gpr68'	3.51	0.94	10.5	0.1	2.83	10.55	0.07	1.98	4.78	4.42
238384	'Slc24a4'	0	0	0	1.37	2.02	0	0.04	0	0	0.01
238386	'Btbd7'	0.63	0.72	1.94	5.88	0.88	2.24	0.01	0.66	0.75	0.24
238393	'Serpina3f'	0.02	1.91	0.09	0	0	0	0	0	0	0
23844	'Clca1'	0	0	0	0	0.78	0	0	0	0	0
23845	'Clec5a'	0	0.72	0	0	3.9	0.55	2.83	0.02	0	0
23849	'Klf6'	8.33	29.5	28.9	88	15.9	17.18	20.5	16.09	17.6	18.37
23850	'Pappa2'	0	0	0.17	0	0	0	0	0	0	0
238505	'Mtr'	2.16	0.8	0.02	0.01	1.75	0.56	4.1	0	3.02	0.53
23853	'Def6'	2.55	1.62	0	0	0	5.32	0	0	2.23	5.24
23854	'Def8'	19.42	23.13	30.3	3.73	19.5	8.42	11.2	8.1	18.89	13.61
23855	'Defa17'	0	0	0	0	0	0	34.3	0	0	0
238555	'Btn2a2'	0	0	0	0	0	0	0	0	0.87	0
23856	'Dido1'	6.93	4.99	2.62	9.48	8.85	7.15	2.16	4.91	11.84	8.28
238564	'Mylk4'	0.03	0	0.1	0.05	0.04	0.02	0.01	0	0.02	0.01
23857	'Dmtf1'	12.55	10.22	20.8	17	23.1	8.74	7.16	15.72	12.12	26.76
23859	'Dlg2'	9.42	8.11	8.18	6.06	9.78	14.32	5.25	7.1	13.17	19.14
23863	'Dand5'	2.05	0.95	6.87	0	5.78	11.11	5.3	15.15	13.31	20.22
238663	'Spata31d1d'	0	0	0	0	0	1.64	0	0	0	0
238673	'Zfp367'	5.3	5.67	4.89	0.68	7.01	3.69	4.41	2.18	0.85	4.45
238680	'Cntnap3'	0	0.14	1.92	0	1.18	0	0.23	0	0	0
238690	'Zfp458'	1.91	1.43	0.34	0.26	0.84	0.55	2.27	0.09	1.42	0.77
238692	'Zfp874a'	4.77	2.82	3.43	0	1.92	4.73	0	4.48	0	6.54
238693	'Zfp58'	0	7.73	1.1	0	6.35	3.56	3.7	2	2.72	5.04
23871	'Ets1'	0	0	0	0	0.01	0	2.02	0	0	0
23872	'Ets2'	40.49	54.69	35	27.3	21.9	23.4	42.2	50.1	18.63	37.36
238722	'Zfp72'	3.15	2.92	4.02	0	2.78	1.19	4.59	4.22	6.6	3.57
238725	'Gpr150'	0.57	1.77	2.67	0	3.33	0.98	0	2.13	1.62	2.68
238726	'Fam81b'	1.56	0	0	0	0	0	0	0	0	0
23873	'Faim'	44.72	76.73	53.2	66	64.7	72.23	62.6	23.67	56.54	52.13
23874	'Farsb'	117.1	95.54	73.1	67.8	47.6	83.09	106	73.83	82.5	87.2
23876	'Fbln5'	15.37	6.25	0.02	0.02	0	5.23	12.9	1.95	0	0
23877	'Fiz1'	9.4	2.27	7.49	4.23	11.8	7.52	2.34	2.21	1.57	5.01
23879	'Fxr2'	14.15	17.95	10.2	0	22.6	4.99	5.54	13.21	11.07	8.19
238799	'Tnpo1'	4.91	4.84	5.07	7.56	1.8	2.16	3.59	3.55	4.38	5.08
23880	'Fyb'	0	0	1.25	0.63	1.66	9.52	0.01	1.54	0	0
23881	'G3bp2'	157.2	151	118	74.1	159	146.8	167	157.3	154	166.39
23882	'Gadd45g'	93.13	305	263	490	58	133.7	22.5	141.6	82.93	147.39
238831	'Ppwd1'	2.33	2.33	2.35	0.99	5.93	2.46	2.21	1.5	3.41	5.13
23885	'Gmcl1'	7.71	0.72	2.56	6.09	7.89	1.91	2.16	4.8	5.45	3
23886	'Gdf15'	0	3.01	0	0	0.98	0	0	0	0	0
23887	'Ggt5'	0.31	6.45	0	0	0.03	0	0	1.65	0	0
238871	'Pde4d'	2.28	3.35	5.1	7.53	2.91	3.78	7.87	3.36	5.35	1.84
238875	'Gapt'	0	0	0	0	0.07	0	0	0	0	0
23888	'Gpc6'	0.45	0.39	1.03	0.02	1.58	1.04	0.51	0.66	0.09	0.38
238896	'Cdc20b'	0	0.02	0	0	0	0	0	0	0	0
23890	'Gpr34'	0.23	0.25	0	7.71	6.77	19.41	0	0.04	0	1.76
23892	'Grem1'	0	2.99	4.12	9.66	0	8.69	0	0	5.68	0
23893	'Grem2'	0.22	0.57	1.42	0.56	3.37	0.6	3.13	0.87	3.35	1.18
23894	'Gtf2h2'	23.02	26.3	18.3	60.8	23	55.58	24.7	28.77	64.8	39.22
23897	'Hax1'	101.3	145.2	108	129	104	210.9	158	128.8	105.7	122.09
238988	'Erc2'	9.01	10.94	8.93	6.73	12.6	11.06	13.8	8.61	15.36	7.02
239017	'Ogdhl'	16.75	20.19	52.9	17.5	14.3	6.57	27.8	14.61	33.56	19.13
239027	'Arhgap22'	3.78	0.44	4.68	6.1	1.07	4.92	6.01	0	7.47	0.67
23908	'Hs2st1'	5.31	8.72	2.37	10.6	1.45	0	0.01	2.96	0.99	0.43
239096	'Cdh24'	0.27	1.69	0.07	0	0	0.11	2.08	0	0	0

239099	'Homez'	2.45	2.41	0.04	4.57	3.41	0.08	3.56	1.73	3.16	2.56
239102	'Zfhx2'	3.12	4.87	1.95	1.96	3.69	1.57	2.81	3.04	5.27	1.1
239114	'Il17d'	1	1.73	1.97	0	2.55	0	0	0.64	0	0
23912	'Rhof'	46.42	35.96	34.3	17	25.2	34.09	54.7	39.39	29.21	26.24
239122	'Setdb2'	0	0.46	1.85	0	2.54	0	0.08	0.88	2.26	3.53
239133	'Dleu7'	4.49	7.6	2.8	7.33	3.88	15.51	8.92	10.68	3.68	5.22
239134	'Gucy1b2'	0.23	0.07	0.02	0	0	0	1.95	0	0	0
239157	'Pnma2'	35.88	38.32	42.4	8.86	36.3	34.4	35	32.07	29.94	26.19
23917	'Impdh1'	15.72	22.85	23.1	6.33	17.2	14.07	16.4	14.78	23.35	15.7
239170	'Fam160b2'	2.83	2.76	3.25	5.26	4.32	2	3.19	1.82	1.03	1.54
23918	'Impdh2'	54.21	71.86	43.8	58	54.3	34.34	49.8	47.79	43.11	52.58
239188	'Enox1'	4.75	5.82	5.73	2.12	4.58	3.89	13.1	3.94	5.28	0.44
23919	'Insl5'	2.66	0.76	2.5	0	0	8.94	0.24	0	5.84	0
23920	'Insrr'	0	0	0.1	0	0	2.01	0	0	0	0
23921	'Sh2b2'	6.02	0.83	0	0	3.8	1.8	0	2.94	0	0
239217	'Kctd12'	0	0.31	0.82	1.56	1.8	0	0	0.34	1.73	0.5
23922	'Jtb'	73.7	65.44	64.4	60.4	61.9	70.27	60.8	78.11	77.02	97.02
23923	'Aadat'	0	0	0	0	0	0	0	0	0	5.37
23924	'Katna1'	20.52	12.39	5.54	8.04	15.2	9.08	7.18	11.59	13.98	10.94
239250	'Slitrk6'	0.38	3.03	0	1.08	0.09	0	0	3.2	0	0
239273	'Abcc4'	0.13	0.57	0	0.04	0.17	0.16	2.36	0.01	0	0.95
23928	'Lamc3'	0.04	0	0	0	0	0	0	0	0	0
239318	'Plcxd3'	82.48	98.67	52.5	92	83.8	122.1	58.9	135	176.2	91.17
239319	'Card6'	0	0.06	0	0	0	0	0	0	0	1.5
239336	'Rxfp3'	1.4	6.12	0.27	15.2	0	0	0	1.25	0	13.48
239337	'Adamts12'	0	0	0.31	0	0	0	0	0	0	0.13
23934	'Ly6h'	1273	1611	843	1317	927	1029	660	1150	1363	1296.41
23936	'Lynx1'	37.17	38.8	38.3	7.26	55.8	26.54	62.4	53.52	76.68	60.9
239364	'Tspyl5'	11.45	12.23	6.39	19.9	13.1	4.32	10.7	9.56	8.18	6.44
239368	'Erich5'	0.02	0	0	0.02	0	0.01	0	0	0	0
23937	'Mab21l2'	0.02	16.94	4.45	0	0	2.31	13	0.9	0	0.02
23938	'Map2k5'	6.75	8.1	9.28	4.15	21.3	18.62	0.04	5.79	16.77	12.97
23939	'Mapk7'	5.82	9.43	8.92	0.53	5.09	0	12.5	0.01	12.62	0.48
239393	'Lrp12'	3.68	3.09	6.46	2.83	7.04	0.34	2.52	0.87	4.5	9.67
239408	'Tmem74'	0	2.81	3.97	0	2.82	0	0	3.88	1.09	3.73
23942	'Mta2'	6.42	26.72	15.2	14.9	14.3	10.54	34.3	15.64	6.82	18.06
239420	'Csmd3'	5.05	5.68	5.34	5.09	7.18	12.87	3.87	3.26	5.02	4.5
23943	'Esyt1'	0	0.33	0.17	0	0.07	0.01	0	1.31	0	0.01
239435	'Aard'	1.33	0.98	2.54	6.64	0.33	3.8	7.18	2.73	9.09	1.71
239436	'Slc30a8'	1	0	0	0	0	2.33	0	0	0	0
23945	'Mgll'	6.32	0	4.7	0	4.28	9.89	6.35	3.74	1.56	1.27
239463	'Fam83a'	0.31	0.48	0.41	2.14	0.33	0.78	0.36	0.49	0.74	0.36
23947	'Mid2'	0.44	0.37	1.86	0	2.07	0.04	2.16	1.62	2.59	3.76
23948	'Mmp17'	0.94	0.99	5.34	6.64	14	7.99	3.1	3.69	3.13	1.62
23950	'Dnajb6'	171.2	193.8	149	244	154	196.1	137	155.8	182.6	171.53
239510	'Phf20l1'	3.5	6.22	4.23	9.93	5.54	0.71	7.37	1.42	8.05	0.71
239528	'Ago2'	1.59	1.38	5.63	2.34	2.55	1.5	5.91	0.37	2.43	2.85
239530	'Gpr20'	0.1	0.02	0.02	0.02	0.01	0.14	0.01	0	0.29	0.03
23954	'Nek3'	3.97	0.97	1.21	0	2.46	0	0	0.4	0	4.84
239546	'Zfp647'	0.52	0.28	3.84	0.67	0	4.69	1.31	5.03	6.09	0.54
23955	'Nek4'	4.89	9.32	5.76	25.9	4.56	15.62	10.2	5.15	12.83	12.45
239552	'Apol8'	0	0	3.47	0	0	0	0	1.78	0	0.11
239554	'Foxred2'	6.28	3.3	6.99	5.01	1.91	0.37	10.1	4.82	5.09	4.59
239555	'Mief1'	5.17	3.76	2.5	0.05	0.98	3.29	4.08	8.61	4.1	2.64
239556	'Cacna1i'	0.93	2.32	0.83	0	1.26	1.44	0.64	1.2	0.22	0.03
23956	'Neu2'	0	2.41	0	0	1.57	0	0	0	0	3.77
239570	'Ttc38'	1.65	0.68	2.17	0	0	6.05	0.02	0.71	2.26	3.63
23958	'Nr2e3'	0.23	0	0	0	0	0	0	0	0	0

23959	'Nt5e'	0	0.05	0	0.61	0	0	4.35	1.6	0	0
239591	'Ttll8'	0.13	0.12	0.31	0.02	0	0	0	0.01	0.52	0.83
23960	'Oas1g'	0	0	0	1.96	0	0	0	0	0	0
239606	'Slc2a13'	1.33	1.27	4.89	0.03	0.94	2.32	2.81	0.63	1.87	2.78
239611	'Muc19'	0	0.01	0	0	0	0	0	0	0	0
239618	'Pdzrn4'	13.9	28.93	8.98	5.06	6.94	4.43	14	14.46	4.43	2.67
23962	'Oasl2'	0.02	3.18	7.26	86.5	0	0.02	6.41	2.3	17.93	10.9
23963	'Tenm1'	10.82	6.69	9.55	5.14	10.3	7.7	11.7	8.92	11.41	9.35
23964	'Tenm2'	8.06	3.54	7.44	2.31	16	3.93	7.01	6.95	0.94	1.27
239647	'Pced1b'	7.84	2.64	2.74	0.31	0.19	4.18	2.89	4	0.29	2.23
23965	'Tenm3'	2.04	1.27	1.86	7.44	3.07	1.61	7.46	0.82	0.48	1.24
239650	'Ccdc184'	33.32	34.98	80.8	93	31.6	28.76	44.3	52.58	34.21	32.89
239652	'Zfp641'	1.71	1.96	5.37	0	2.29	1.11	3.24	3.19	0.13	2.76
23966	'Tenm4'	1.9	5.99	6.18	3.18	7.66	4.34	9.14	3.64	4.93	2.48
239667	'Dip2b'	1.72	0.71	3.34	9.17	1.64	1.62	3.33	1.5	2	1
23967	'Osr1'	0.02	0	0	0	0	0	0	0	0	0
239673	'Krt90'	11.18	59.19	0.02	24.8	0.04	0	0	26.18	0.07	9.78
23968	'Nlrp5'	0	0	0.1	0	0	0	0	0	0	0
23969	'Pacsin1'	85.08	65.44	76.8	29.8	113	87.09	63.9	81.3	60.3	78.75
23970	'Pacsin2'	4.57	6.33	30.3	25.6	22.6	13.93	26	9.12	29.91	12.29
239706	'Mettl22'	27.9	41.27	49.9	12.6	34.9	73.51	31.1	64.52	56.39	41.27
23971	'Papss1'	19.31	32.86	36.3	8.85	22.5	26.28	36.6	17.29	23.07	43.38
239719	'Mkl2'	26.1	17.95	17	35.9	24.9	12.61	22.8	17.1	17.67	19.66
23972	'Papss2'	3.84	1.07	2.49	0	0.11	4.13	0.8	3.09	1.47	0
239731	'Rimbp3'	1.36	1.78	0	0	0.01	0	0	0	0.8	0
239739	'Lamp3'	0	0.04	0	0	0	0	0	0	0.03	0.04
239743	'Klhl6'	0	0	0.02	0	2.65	0.68	0	0	0	0
239759	'Liph'	1.53	0	0	0	0	0	0	0	0	1.48
239766	'Rtp1'	1.56	3.95	0	0	0.03	3.67	3.92	5.64	0	0
239789	'Gmnc'	0	0	0	0	0	0	0	0.03	0	0
239796	'Mb21d2'	11.5	4.68	1.87	0	0.55	6.42	3.6	5.56	5.12	2.89
23980	'Pebp1'	739.5	772.6	774	641	812	1061	983	794.9	591	680.63
239827	'Pigz'	14.29	3.84	7.79	1.69	2.73	8.35	11.9	12.13	18.68	9.68
23983	'Pcbp1'	102	104.8	79.9	84.2	86.3	103.8	87.7	78.72	64.95	100.21
239833	'Lmln'	0.29	0.01	0.41	2.15	1.03	2.77	3.22	0.55	1.19	1.79
239839	'Ccdc14'	0.65	0.24	0	8.21	0.45	0	1.97	1.59	0	0.38
23984	'Pde10a'	4.12	2.03	3.44	5.95	2.86	3.47	1.48	1.87	11.42	2.13
239845	'Gpr156'	0	0	1.24	0	0	0	0	0	0	0
23985	'Slc26a4'	0	0	2.73	0	0	0	0	0.9	0	0.01
239857	'Cadm2'	3.46	2.17	3.09	2.48	3.02	0.13	7.82	2.58	1.16	4.02
23986	'Eci2'	20.23	40.78	50.7	57.7	23.2	52.48	51.4	35.58	24.31	19.82
23988	'Pin1'	67.89	55.9	58.5	86.7	90.6	86.1	58.7	91.77	71.89	84.73
23989	'Med24'	15.72	31.8	27.7	19.3	13.8	8.32	37.7	26.46	11.07	28.98
23991	'Cib1'	7.87	2.07	5.26	0.07	10.1	16.7	5.88	5.77	13.58	4.64
23992	'Prkra'	13.42	38.12	36	24.3	14.6	30.24	33.8	23.1	20.78	27.35
23994	'Dazap2'	71.96	52.56	81.4	126	66.3	82.62	120	78	53.05	58.97
23996	'Psmc4'	156.2	187.6	182	208	163	223.6	142	241.6	233.2	192.39
23997	'Psmd13'	112.5	114.7	130	152	120	103.6	112	134.6	87.21	119.44
239985	'Arid1b'	2.35	1.65	0.39	1.93	0.46	0.43	0.56	0.32	2.47	1.33
23999	'Twf2'	12.99	33.61	40	12.3	34.4	22.65	20.3	22.92	24.81	39.17
24000	'Ptpn21'	0.46	0.11	0.18	1.13	0	0.9	1.71	0.42	1.19	0.24
24001	'Tiam2'	1.45	1.28	1.68	3.29	0.11	0	0.06	1.88	0	1.71
240023	'Pnldc1'	0.03	0.21	0	0.13	0.09	0.11	0.68	0.03	0.09	0
240025	'Dact2'	9.44	4.58	1.68	0.02	2.01	0	1.8	1.98	7.02	0.03
240028	'Lnpep'	0.02	1.62	1.61	0	0	4.53	1.11	1.4	3.22	0.69
240034	'Zfp760'	1.99	1.9	0.66	0.01	3.21	0.01	0	0.71	2.16	0.01
240038	'Zfp994'	2.46	3.81	3.85	2.48	1.08	2.06	5.99	3.24	1.46	1.73
24004	'Rai2'	1.39	0.84	2.63	11	8.02	2.72	12.7	5.42	13.22	9.66

240041	'Zfp945'	1.69	0.77	2.39	3.03	2.06	1.28	1.5	1.36	2.64	2.28
240047	'Mmp25'	0	0.03	0	0.02	0	0	0	0	0	0.02
240055	'Neurl1b'	0.17	0	0.92	4.14	0.25	0	0	0	0	0.01
240057	'Syngap1'	0.78	0.3	1.47	0.76	0.53	2.97	0.77	1.4	1.13	0.67
240058	'Cpne5'	9.35	7.83	7.55	25.2	16.6	22.68	11.7	14.32	7.01	23.53
240063	'Zfp811'	6.8	11.12	4.57	0.22	3.3	5.5	11.6	13.33	3.33	8.01
240064	'Zfp799'	5.34	4.59	4.27	6.47	3.68	3.97	3.27	2.13	1.78	11.44
240066	'Zfp870'	0.14	0.06	3.8	0	0	1.58	0.01	1.57	4.38	3.22
240067	'Zfp952'	12.25	4.13	7.33	18.8	14.9	3.42	7.5	10.47	10.29	2.51
240068	'Zfp563'	4.77	1.84	0.85	0	1.38	1.18	3.06	1.37	4.48	2.48
240069	'Morc2b'	1.58	0.6	2.39	0	0.02	0.93	2.46	0.34	0	0.45
240084	'Cchcr1'	1.63	0	0.25	0	0	0	0	0	0	1.34
240087	'Mdc1'	0.29	0.61	1.23	0.58	2.58	1.48	0.97	0.2	0.79	4.21
240095	'H2-M5'	1.32	0	0	2.35	0	0	0.09	0.21	0	0
24010	'Ik'	97.61	69.62	96.8	143	68.2	50.03	78.6	80.7	66.52	82.44
240119	'St6gal2'	1.66	0.4	12.7	0	0.29	12.07	1.36	10.04	0.02	0
24012	'Rgs7'	17.72	5.65	14.9	10.1	8.07	7.36	11.3	9.98	15.47	12.2
240120	'Zfp119b'	2.43	0	5.83	0.13	0	0	0.03	0.02	3.34	0.09
240121	'Fsd1'	116.7	134.7	97.3	119	117	82.29	99.5	91.29	107.2	115.91
24013	'Grk1'	2.42	2.46	3.76	2.69	4.13	2.91	3.23	2.46	4.79	2.27
24014	'Rnasel'	0.13	0.1	0.11	0.21	6.01	0.07	2.98	0.09	5.82	6.8
24015	'Abce1'	17.98	14.49	14.5	26	11.6	7.69	9.11	15.93	19.25	10.87
240168	'Rasgrp3'	3.9	0.54	0	0.01	1.91	2.44	0	0	0	2.11
24017	'Rnf13'	83.23	57.98	92.3	62.2	60.3	76.03	50.1	38.52	56.6	64.34
240174	'Thada'	3.63	0.86	2.21	0.02	2.32	0.46	5.09	0.38	0.25	2.42
24018	'Rngtt'	19.57	18.39	5.01	21.7	8.72	5.28	14	11.71	18.12	29.15
240185	'Jcad'	0.2	0	0	0	0.77	0	0	0.01	0	3.18
240186	'Zfp438'	1.32	0.95	0	0.08	0.11	5.97	1.88	2.12	7.5	0
240216	'E230025N22Rik'	0	0	0.32	0	0.03	11.18	3.73	0	0	0
240239	'Gpr151'	0	9.47	0	0	0.03	0.03	0	0	0	0
240255	'Ythdc2'	0.27	0.46	1.01	1.91	0.84	0.01	5.22	0.85	0.75	0.12
240261	'Ccdc112'	0.28	1.31	2.11	2.14	1.77	0.98	3.05	1.82	1.1	1.21
240263	'Fem1c'	4.8	2.28	1.9	6.89	9.87	2.29	3.95	4.2	1.94	2.95
240283	'Dmxl1'	0.72	0.51	0.65	2.27	0.63	0.58	1.52	0.06	1.06	0.51
24030	'Mrps12'	230	161.8	202	243	245	347.7	252	307.1	231.3	272.84
240322	'Adamts19'	0	0	0	0.16	0	0	0	0	0	0.01
240327	'Gm4951'	0.02	0.02	1.18	0	0	0.04	0	0	1.22	0.21
240328	'F830016B08Rik'	4.69	3.61	5.22	11.5	7.37	5.43	4.15	2.21	4.38	4.37
240332	'Slc6a7'	10.41	7.43	10.9	7.17	9.32	7.83	10.4	4.87	3.47	2.06
240334	'Pcyox1l'	23.87	34.38	21.4	13.7	26	25.75	28	40.7	42.92	35.28
240354	'Malt1'	0.99	0.35	1.47	2.53	0.82	0.18	2.99	0.19	1.19	0.65
240396	'Mex3c'	1.23	0.88	0.33	0.01	0.67	0	1.44	1.26	0	0.03
240427	'Setbp1'	0.49	1.46	0.41	0.76	0	0.54	0.07	0.07	0.82	0.8
24044	'Scamp2'	5.7	16.59	17.3	5.77	1.68	20.13	20.6	5.08	3.67	4.77
240442	'Adnp2'	6.24	0.29	1.68	9.64	0.8	0.04	3.2	3.97	1.86	2.45
240444	'Kcng2'	0.35	2.65	0.04	3.74	4.93	4.44	1.78	2.8	2.36	4.71
24045	'Scamp3'	83.62	73.69	73.7	85.5	86.8	83.9	79.7	91.07	95.74	61.37
24046	'Scn11a'	1.41	0	0.01	1.1	2.78	0.57	0	0	0	1.08
24047	'Ccl19'	0	0	0	0	0	0	0	0	0.52	0
240476	'Zfp407'	1.46	0.56	1.12	3.73	2.24	1.2	0	0.43	0.09	1.05
240479	'Fam69c'	0	0	5.16	2.61	0	3	0	3.06	0	0
24050	'Sept3'	50.97	41.86	54.7	52.3	50.4	50.33	32.9	54.03	29.99	40.22
240505	'Cdc42bpg'	0.62	0	0	0	0	0	0	1.05	0	0
24051	'Sgcb'	17.16	33.08	29.2	15.5	8.79	25.45	51.2	33.61	20.36	36.79
240514	'Ccdc85b'	7.41	8.43	14.8	5.67	16.9	5.28	10.2	4.97	9.84	0.93
240518	'Peli3'	21	8.97	14.8	8.77	19.3	3.57	24.8	6.94	33.07	20.37
24052	'Sgcd'	2.84	0.05	0.84	0.11	1.18	0.1	1.62	0.47	0.75	4.79
24053	'Sgcg'	0	0.01	0.09	0	0.04	0.03	0	0.01	0.02	0.02

240549	'Gm4952'	4.01	0	0.03	0	0	0	0	0	0.03	0
24055	'Sh3bp2'	0	0.13	0	0	4.43	3.57	1.79	0	0.31	0.74
24056	'Sh3bp5'	25.3	28.24	15.5	6.82	22.7	19.71	34.6	18.97	12.91	13.25
24057	'Sh3yl1'	0	12.51	18.4	22.7	13.5	7.45	12.5	8.04	18.17	10.54
24059	'Slco2a1'	0	0.04	0	0	0.09	0	0	0	0	0
24060	'Slc35a1'	9.15	20.99	20.1	35.1	22.9	30.39	12.1	22.65	18.46	24.01
24061	'Smc1a'	20.84	31.86	30.3	29.8	19.4	23.39	25.8	24.38	23.04	23.15
240613	'9930021J03Rik'	3.18	0.34	3.48	8.2	2.02	2.57	2.6	0.6	5.63	0.99
240614	'Ranbp6'	18.68	13.18	9.59	9.37	14.1	5.8	27.6	8.27	16.28	13.71
24063	'Spry1'	4.49	8.43	19	21.2	1.18	0	4.1	2.6	0	2.03
240638	'Slc16a12'	0.04	0	0	0	0	0	3.5	0	0	0
24064	'Spry2'	13.76	20.61	16.6	51.4	5.29	12.29	8.68	15.67	18.19	18.15
240641	'Kif20b'	0	0	0.33	0	0	0.15	0	0	0	0
24066	'Spry4'	0.79	2.31	1.95	0.6	1.15	0.22	7.93	2.76	0.43	2.53
240660	'Slc35g1'	2.76	0.07	0.12	0.08	0.11	0.03	0.03	0.03	0.11	1.39
240665	'Ccnj'	1.28	1.05	1.63	3.05	0.05	0	0	0.34	0.94	0
240667	'Sec31b'	0	0	0.08	0	0	0	0.49	0.34	1.28	0.19
24067	'Srp54a'	30.53	29.55	40.9	31.2	28	33.7	25.9	38.97	41.38	34.02
240672	'Dusp5'	0	0.38	3.63	7.06	0	0	0.04	0	0.42	0.19
240675	'Vwa2'	0	0.19	0	4.41	1.26	0	0	0	0.01	0
24068	'Sra1'	87.73	57.98	68.7	125	105	97.6	20.5	59.94	77.04	109.39
24069	'Sufu'	2.61	4.04	2.5	7.55	3.44	2.34	2.72	2.09	0.3	1.78
240690	'St18'	0.06	0.12	0.01	0.01	0.84	1.3	0.71	0	0.01	0.57
240697	'Mcmdc2'	8.71	5.79	7.38	11	9.59	7.94	7.13	7.08	11.36	7.44
24070	'Mpdu1'	34.98	43.51	62	62.3	51.8	51.75	44.7	65.74	46.54	45.47
24071	'Synj2bp'	15.22	17.14	14.3	14.8	16.7	11.99	14.5	15.45	9.27	13.41
240725	'Sulf1'	11.59	4.2	12.9	5.86	9.07	1.17	18.5	3.19	24.2	22.48
240726	'Slco5a1'	0	0.29	0.01	1.24	0.76	0	0.88	0.28	0.01	0.02
24074	'Taf7'	12.38	11.8	15.1	33.7	4.19	7.26	13.1	6.69	12.38	13.45
24075	'Taf10'	8	4.43	24.9	9.79	7.15	4.45	6.51	0.06	5.93	1.26
240752	'Pik3c2b'	0.77	1.9	0.36	2.91	1.9	0.33	0.1	0.54	0.68	1.72
240753	'Plekha6'	4.33	4.32	5.31	1.29	3.76	3.9	5.79	3.9	3.96	4.5
240754	'Lax1'	1.29	0	0	0	0	0	0	0	0	0
240756	'Klhl12'	31.03	15.28	23.7	12.8	5.02	12.98	24	24.44	8.65	18.19
240776	'Kcnt2'	0.03	0	2.19	1.47	3.47	2.24	0.28	2.11	5.01	7.02
240816	'Rgsl1'	0	0	0.07	0	0	0	0	0	0	0
24083	'Natd1'	1.38	2.35	3.53	3.31	0.28	5.87	0	0.01	0	0.66
240832	'Tor1aip2'	34.12	19.08	30.2	57.1	15.2	18.05	22.6	28.69	10.52	32.39
24084	'Tekt2'	7.72	36.28	6.79	13.1	51.9	25.96	9.84	6.19	16.9	40.59
240843	'Brinp2'	35.89	33.95	27.9	42.2	6.09	4.6	14.5	35.54	25.55	31.49
24086	'Tlk2'	1.63	4.25	6.63	7.9	12.4	4.32	8.82	4.42	11.55	4.47
240869	'Zbtb37'	0.58	0.84	3.73	0	1.57	4.04	2.76	1.47	0	0.32
24087	'Tll2'	0.01	0.49	0	2.54	0.99	0.63	0	0	0.67	0
240879	'Mettl11b'	0	0	0	0	0	0	1.08	0	0	0
24088	'Tlr2'	0	0	0	0	0	2.98	0	0	0	1.92
240880	'Scyl3'	3.14	1.44	5.13	3.59	0.42	0.79	5.95	2	1.84	0.02
240888	'Gpr161'	0.24	0.67	0.01	0.02	0	0.01	0	0.47	1.45	0.08
240892	'Dusp27'	0	0	0	0.01	0	0	0	0	0	0
240913	'Adamts4'	1.41	5.68	0.12	0.02	1.23	0.27	3.23	0	0.01	0
240916	'Vsig8'	0.37	0	1.8	0	0	0	0	0.03	0.07	1.24
240960	'Dnah14'	0.01	0	0	0	0	0	0	0	0	0
24099	'Tnfsf13b'	1.83	0	1.48	0	2.31	3.71	0.07	3.07	2.31	0
24100	'Tpra1'	67.48	76.38	47	29.5	68.5	40.34	71.6	68.2	68.69	99.56
24102	'Trex2'	0	3.33	0	0	0	0	0	0	0	0
24105	'Rbck1'	62.34	63.74	84.8	51.8	53.2	79.1	126	87.12	40.29	110.45
241062	'Pgap1'	2.63	3.12	4.77	3.68	1.66	1.64	4.54	1.9	0.82	4.66
241066	'Carf'	5.21	2.87	3.85	2.28	2.61	1.27	0.14	1.98	4.28	1.61
241070	'Gpr1'	2.58	8.97	6.01	10.1	1.64	9.54	0.03	0.02	0.77	0.14

241075	'Plekhm3'	0.48	1.66	0.37	1.12	0.88	0.83	3.97	1.87	2.05	1.92
24109	'Ubl3'	60.36	48.29	67.5	67	57.3	90.63	57.4	97.49	76.74	55.18
24110	'Usp18'	0	2.67	2.81	31.6	2.88	0	6.6	0	0	0.02
241112	'Catip'	0	2.77	0	0	0	0	0	0.72	0	0
241113	'Prkag3'	0	0	0	0	0	0.02	0	0	0	0
241116	'Cfap65'	0.4	3.14	0.01	0.01	0.75	0.02	0	1.71	3.05	0.81
241118	'Asic4'	0.02	36.64	2.15	0	0	0.02	0.51	0	0.03	0
241134	'Nyap2'	3.01	4.65	2.84	1.96	1.38	0.64	1.99	4.83	1.52	2.68
24115	'Best1'	12.21	3.76	5.89	5.34	12.9	18.46	6.16	2.68	27.81	10.93
241158	'Ankmy1'	0	0	0.18	0	0	2.78	0.09	0	0.07	2.34
241159	'Neu4'	0	0	7.42	2.89	1.18	0	25.4	3.97	0	0
24116	'Nelfa'	25.73	26.86	17.3	7.35	19.9	19.72	20.5	21.33	26.57	10.47
24117	'Wif1'	0	3.42	0	0	0.02	1.32	0	3.19	7.25	0
241175	'Cntnap5b'	1.5	0.04	0.02	0	1.3	1.12	1.49	2.47	0.23	1.76
241201	'Cdh7'	2.66	6.34	4.92	0.02	1.25	0.55	0.38	7.05	2.95	4
241226	'Itga8'	0	1.21	0.55	0	0	0.01	0.53	0	1.19	0.89
241230	'St8sia6'	0	0	1.54	0.01	0.26	0	0	4.1	0.4	0.01
241263	'Gpr158'	1.98	3.92	10.4	3.45	9.32	2.22	7.26	2.82	5.51	4.57
24127	'Xrn1'	2.94	1.16	2.56	10.3	2.15	5.41	3.58	2.65	3.54	1.62
241274	'Pnpla7'	0.43	2.53	0.07	5.39	0.59	3.06	3.93	0.23	0	1.25
24128	'Xrn2'	15.79	11	22.8	27.8	12.7	13.23	9.94	15.97	17.14	18.06
241289	'Ppp1r26'	3.46	2.89	3.84	4.48	1.89	0.05	1.11	1.49	2.29	0.96
241296	'Lrrc8a'	32.48	23.8	30.5	28.8	41.7	35.01	30.2	56.07	76.97	37.03
241303	'Fam78a'	0	0.02	0	0.01	0	0	0	0	0	0
241308	'Ralgps1'	2.02	3.3	3.52	10.7	2.08	1.95	8.53	2.1	2.24	4.48
24131	'Ldb3'	6.7	4.69	0.95	0.21	11.9	0.23	0.19	5.57	0.16	5.35
241311	'Zbtb34'	1.19	0	0	0	0	0	0	0.73	0	0.25
24132	'Zfp53'	10.95	6.59	5.4	0.84	5.48	3.34	4.86	4.41	2.16	10.03
241322	'Zbtb6'	1.86	0.25	0.47	3.7	0.07	0.23	0.26	0.75	0.02	1.19
241324	'Crb2'	0	0	0.01	0	0	0	0	0	0.03	0.89
241327	'Olfml2a'	0	0	0	0	0	0	0	0	2.94	0
24135	'Zfp68'	6.64	7.06	12.7	5.68	5.9	7.72	10.9	14.82	9.52	13.44
24136	'Zeb2'	0.64	0.01	0.02	0	0.77	1.89	3.55	0.14	1.88	0.24
241431	'Xirp2'	0.01	0	0.04	0	0	0.01	0.02	0	0.01	0.01
241447	'Cers6'	3.32	3.43	2.82	3.69	3.43	0.02	2.77	3.13	2.44	4.09
241489	'Pde11a'	0	0.36	4.36	0.01	3.96	0.27	0.3	1.29	0.01	0.93
241490	'Rbm45'	2.7	2.29	0.99	1.02	3.67	0.31	0.98	1.67	1.88	0.59
241494	'Zfp385b'	1.24	9.73	8.63	9.07	16.3	1.82	10.3	16.52	1.67	12.73
241514	'Zfp804a'	1.9	3.74	0.68	3.95	3.55	1.55	2.28	1.21	1.49	1.07
241516	'Fsip2'	0	0	0	0	0	0	0	0.01	0	0
241520	'Fam171b'	54.99	96.78	57.9	41	29.9	39.32	60.5	55.29	58.98	51.66
241525	'Ypel4'	32.96	25.96	45.6	6.19	26.6	19.89	32.9	26.88	50.15	58.71
241528	'Lrrc55'	10.61	1.19	8.82	3.38	4.39	0.15	2.34	4.2	5.04	3.58
241547	'Harbi1'	4.95	11.06	6.17	0	3.35	0.77	1.04	6.81	6.11	5.37
241556	'Tspan18'	5.1	3.18	7.38	0	3.62	0.41	0.22	5.76	0	7.2
241568	'Lrrc4c'	23.8	21.35	31.9	23.2	37.4	32.51	27.1	32.94	44.94	24.03
241576	'Ldlrad3'	0	0.29	1.59	0.06	0	0.13	3.02	1.47	0.33	0.08
241589	'D430041D05Rik'	2.13	0.99	2.19	2.64	1.73	3.06	2.34	3.33	2.38	2.31
241612	'Slc5a12'	0.02	0	0	0	0	0	0	0	0	0
241624	'Exd1'	0.01	0.27	1.02	0	1.33	0.06	0.02	0.02	0.01	0
241627	'Wdr76'	0.94	0.15	0.45	0	1.01	0.12	1.25	0.1	0	0.09
241633	'Atp8b4'	0	0.89	0	0	0	0	0	0	0	0
241636	'Tgm6'	0	0	0.45	0	0.02	0	0	0	0	0
241638	'Lzts3'	1.63	1.78	10	2.14	2.64	1.94	5.17	3.12	9.1	3.66
241639	'Fermt1'	0	0	0	0	0	0.29	0	1.18	0	0
241656	'Pak7'	8.92	9.74	3.91	15.7	2.56	0.04	3.08	4	4.38	5.11
241688	'Dzank1'	18.02	11.41	40.1	16.7	12.1	14.22	27.8	16.62	16.07	18.08
241694	'Ralgapa2'	1.95	1.73	2.56	5.99	0.73	3.4	0.04	0.78	2.43	0.78

241727	'Snph'	9.05	13.78	30.9	3.69	3.61	10.46	12.7	16.5	3.29	11.45
241732	'Tspyl3'	40.85	37.99	37.2	7.72	24	37.98	21.5	57.44	42.22	28.28
241764	'L3mbtl1'	0.32	2.26	9.2	0.71	1.24	2.28	2.32	4.44	0.45	3.4
241770	'Rims4'	1.02	2.16	1.4	0.01	3.04	2.75	5.12	0.61	1.82	1.98
241794	'Kcng1'	0	0	6.85	0	0	0	0	0	0	0.83
241846	'Lsm14b'	4.6	3.73	3.8	3.55	6.03	1.14	2.18	0.96	7.22	2.28
241850	'Abhd16b'	0.02	0	0	0.06	0	0	0	0	0	0
241915	'Phc3'	1.3	1.65	4.85	8.18	1.85	4.41	4.95	0.95	2.31	1.19
241919	'Slc7a14'	11.97	8.55	12	13.6	7.31	7.35	6.81	9.99	17.08	9.43
241943	'Ccdc144b'	0	0.25	0	0	0.06	0.05	0	0.08	0	0.05
241944	'D3Ertd254e'	2.76	3.74	3.69	6.77	8.08	1.15	1.89	3.4	1.42	6.95
241950	'Bbs12'	2.11	0.92	3.41	0	3.01	0	0.78	0.31	3.95	6.27
241989	'Pabpc4l'	0	0	0	0	0	0	0	0.44	0	0
242022	'Frem2'	0.41	0	0	0	0	0	0	0	0	0
242037	'Ankub1'	1.99	0	0	0	0	0.39	0	0	0	0
242050	'Igsf10'	0.01	0.55	0.52	0	1.77	0.08	0.01	0.17	1.62	0.01
242083	'Ppm1l'	16.02	7.89	11.6	18.9	16.2	6.19	31.6	13.32	11.88	7.93
242109	'Zfp697'	1.61	2.51	1.94	1.31	1.74	2.53	0.54	0.25	0.3	0.37
242125	'Mab21l3'	0	0	0	0	0	0	0	0	0.85	0
242126	'Slc22a15'	0.46	0.22	0.01	0	0	0.04	0.01	0	0.21	0.52
242253	'Wdr63'	0	0	0	0	0.2	0	0	0	0	0
242259	'Slc44a5'	0.99	1.15	1.66	1.99	2.04	0	0.77	0.91	0.38	0
242274	'Lrrc7'	3.66	0.79	2.56	1.15	2.98	2.16	1.86	2.08	1.45	0.86
242291	'Impad1'	3.43	4.33	4.04	5.23	8.42	2.13	6.84	3.42	8.87	3.19
242297	'Fam110b'	17.2	17.96	50.3	10	2.29	20.65	26.4	25.75	32.53	20.27
242362	'Manea'	3.42	2.23	3.81	6.14	3.99	2.81	4.34	3.23	2.24	0.87
242377	'Pm20d2'	0.56	0.91	1.15	2.35	0.43	0.02	0.99	0.24	1.44	0.25
242384	'Lingo2'	17.09	4.76	20.8	11	4.68	5.66	22.2	12.65	12.81	8.69
242406	'Rgp1'	2.87	1.63	8.7	1.41	4.02	3.3	1.76	0.02	0.11	4.51
242408	'Fam221b'	0	0.6	0	0.03	0	1.72	0	0.07	2.03	1.58
242409	'Tmem8b'	4.72	3.53	4.28	1.68	4.61	2.82	4.45	5.84	12.57	4.79
242418	'Dcaf10'	3.11	2.72	4.14	18.6	2.73	2.48	2.02	1.27	3.34	1.85
242425	'Gabbr2'	5.18	4.9	2.62	4.79	9.53	9.69	3.05	3.13	0.39	3.15
242443	'Grin3a'	2.05	5.02	3.95	2.35	7.16	0.84	9.49	3.5	5.56	6.1
242466	'Zfp462'	1.41	1.98	3.71	3.52	2.68	0.37	1.12	4.09	2.01	1.16
242474	'Tmem245'	1.66	2.81	0.6	2.5	1.47	0.39	0.85	1.72	0.67	0.97
242481	'Palm2'	3.87	3.29	3.05	0.64	2.76	1.38	0.45	1.38	0.19	1.9
242505	'Rasef'	0	0	0	0	0	0.6	0.88	0.01	0.31	0.46
242506	'Frmd3'	5.39	6.99	13.7	0.91	5.98	16.68	11.3	2.54	9.29	1.06
242509	'Bnc2'	0	0	0	2.12	0	0	0	0	0	0
242521	'Klhl9'	20.35	20.68	41.2	33	24.5	13.33	18.5	22.48	21.96	16.36
242523	'Dmrta1'	0	1.28	0.7	0	0	0	0	0	0	0
242546	'Cyp2j12'	0	1.02	0	0	5.5	4.74	0	0	0	0
242553	'Kank4'	0.01	1.22	0.23	0	0.85	0	4.3	0	0.32	0.1
242557	'Atg4c'	6.95	16.51	13.2	15.6	6.91	8.68	3.12	11.35	16.93	15.43
242570	'Raver2'	0.6	1.05	0	0	0	0.08	0.02	0	0	0.01
242584	'Wdr78'	3.76	5.42	3.94	0	7.53	11.15	8.76	4.9	7.04	6.05
242585	'Slc35d1'	0.02	2.65	0.62	5.44	2.5	3.07	1.06	0.88	1.19	0.03
242594	'Fyb2'	0.75	0.45	0	0	0	0	0.07	0.13	0	0.05
242602	'Lexm'	0	0.19	0	0	0	0	0	1.06	0	0.07
242603	'Cdcp2'	0	0	0	0	0	0.19	1.31	0	0	0.39
242607	'Slc1a7'	0	0	0.03	0.03	0.08	0	0	0	0	0
242620	'Dmrta2'	0.32	0	0	0	0	0.49	0	0	0	0.11
242642	'Hpdl'	1.22	0	0.61	0	0	0	0.03	0	0	2.04
242646	'Tctex1d4'	0	0	0	0	0	0	0	7.89	0	0
242653	'Cldn19'	0	0.01	0	0	0	0.02	0	0	0	0
242662	'Rims3'	17.54	8.05	17.9	11.5	6.8	7.39	18.6	10.25	12.79	9.37
242667	'Dlgap3'	2.66	1.2	4.64	7.09	5.69	2.45	5.54	1.32	1.77	0.72

242669	'Azin2'	1.12	5.39	2.27	9.63	0.18	13.15	10.5	6.11	14.25	1.12
242681	'Rab42'	4.14	1.68	3.77	1	7.44	7.13	8.47	5.15	5.96	0.13
242687	'Wasf2'	1.71	3.37	2.67	2	4.34	7.73	0	1.86	0.69	4.37
242691	'Gpatch3'	1.67	0	3.73	0.97	0	0	0	2.99	0	0
242700	'Ifnlr1'	0	0	0.01	0	0	0	0	0	0	0
242702	'Myom3'	0.76	0	0	0	0	0	0	0	0	0
242705	'E2f2'	0	0	0	0	0	0.13	0.01	0	0	1.64
242707	'Lactbl1'	0	0	0	0	0	0	0	0	0	0.15
242721	'Klhdc7a'	0	0	0	0	0.01	0	0.14	0	0.04	0
242726	'Padi6'	0	0	0	0	0	0	0	0	0.79	0
242735	'Lrrc38'	1.3	5.55	1.78	4.21	0.99	1.49	1.66	4.37	2.02	0.83
242736	'Pramef8'	9.11	0.33	4.45	9.22	0.64	0	6.5	2.75	6.36	4.39
242747	'Zfp933'	2.38	1.08	1	0.04	2.71	0.02	0.07	3.06	1.55	0.02
242748	'Disp3'	10.54	6.89	14.9	5.28	3.47	0.96	7.34	8.7	6.64	3.59
242773	'Slc45a1'	27.78	23	33.3	8.84	20.6	27.13	25.1	29.47	49.98	15.38
242785	'Klhl21'	1.37	2.12	4.86	3.27	1.47	0.53	2.27	1.61	0.11	3.01
242800	'Ttc34'	0.04	1.95	1.75	0.85	0.14	0	1.27	2.22	0.03	0.61
242819	'Rundc3b'	3.6	3.77	2.77	4.78	3.04	3.35	2.61	5.18	5.84	5.65
242838	'Lrrd1'	0.38	0	0	0	0	0	0	0.63	1.67	0.04
242860	'Rsbn1l'	1.03	0.56	2.07	4.63	0.92	0.52	0.19	0.21	0.09	0.29
242864	'Napepld'	3.27	0.89	0.01	1.07	2.78	0.37	4.16	2.26	2.03	2.36
242891	'Cct8l1'	0	0	0	0	0.2	0	0	0	0	0
242894	'Actr3b'	45.44	54.01	49.4	52	42.1	32.27	30.5	40.13	13.6	27.37
242915	'Garem2'	0.41	1.15	3.29	0	1.31	0	2.73	0	0.01	0
242960	'Fbxl5'	47.84	45.92	26.6	60.2	38.2	29.04	69.9	30.06	49.13	61.35
243025	'Tmem156'	0	0	0	0	0	0	0	0.11	0	0
243043	'Kctd8'	4.58	6.71	11.2	3.32	7.74	2.06	3.8	4.38	9.08	0.01
243078	'Tecrl'	0.02	0	0.08	0	0	0	0	0	0	0.02
243168	'Hsd17b13'	0	0	0	0	0.2	0	0	0	0	0
243197	'Mfsd7a'	0	0	0	0	0	0	0	0	0	0.02
243219	'2900026A02Rik'	2.91	4.48	3.28	3.53	1.26	2.34	5.23	1.2	0.01	1.15
243272	'Sbno1'	10.35	9.35	14	7.15	10.7	8.12	9.49	7.75	13.61	12.18
243274	'Tmem132d'	0	1.44	0	6.91	0.43	0	0.02	0	1.76	0.02
243277	'Adgrd1'	0.86	0	2.7	0	0	0	0.02	0.16	0	0
243300	'Nyap1'	5.08	6.07	3.37	0.57	4.54	4.36	4.7	1.99	1.45	7.77
243308	'A430033K04Rik'	9	3.89	16.4	4.82	5.68	7.3	3.31	7.7	5.86	8.95
243312	'Elfn1'	0.81	7.35	3.24	3.51	7.66	1.6	2.19	0.08	0.01	1.17
243328	'Slc29a4'	4.44	0.5	12.8	12.1	0.02	4.12	5.2	1.75	3.38	2.4
243339	'Tmem130'	1239	1349	1242	939	705	1176	904	1146	1197	1408.06
243362	'Stard13'	0	0.08	1.06	0	0.06	0.18	3.58	0	0	1
243369	'Sspo'	0.28	0.21	0	0	0	0	0	0	0.08	0
243371	'Lrrc61'	25.85	11.96	27.8	16.1	19.7	12.11	13.6	25.13	17.13	27.08
243372	'Zfp775'	6.61	5.42	8.87	0	2.21	4.06	0	3.46	9.1	3.81
243374	'Gimap8'	0.5	0.27	0.99	0.48	0.4	0.34	0.05	0.09	0.17	0.08
243382	'Ppm1k'	12.09	5.89	10.4	3.58	9.98	14.64	9	7.27	8.21	8.51
243385	'Gprin3'	7.04	4.8	8.47	1.91	6.63	0.13	9.42	1.67	3.95	0
243407	'C130060K24Rik'	2.01	0.84	0	0	0.76	0	1.44	0	0	0
243499	'Lrrtm4'	9.27	6.05	10.3	0.81	14	8.22	9.32	11.83	7.05	8.47
243510	'Ccdc142'	2.09	0.78	3.38	0	0.12	4.09	4.34	3.14	3.36	3.42
243529	'H1fx'	0.43	0.14	0.09	0.44	0.14	0.04	0.39	0.12	0	0.37
243537	'Uroc1'	0.07	1.57	0	0.14	0	0	0	0.09	0	0
243538	'Cfap100'	1.14	4.07	0.11	7.6	0.03	2.87	0.31	0.14	0	0
243547	'Grip2'	1.83	2.05	0.91	2.15	2.04	1.06	1.7	2.93	2.67	1.59
243548	'Prickle2'	6.15	4.41	5.64	8.43	5.2	5.91	7.16	5.43	3.83	3.86
243574	'Kbtbd8'	1.42	0	1.78	0.01	0	0.01	0	0.98	1.53	3.15
243616	'Slc6a11'	23.04	16.25	8.65	8.24	12.3	10.35	9.37	8.51	5.2	6.37
243621	'Iqsec3'	3.42	7.22	4.73	8.67	5.28	3.82	0.28	4.22	2.83	0.56
243634	'Ano2'	0	0	0	0	1.26	0	0	0.76	0	0

243653	'Clec1a'	0	0	2.13	0	0	0	0	0	0	0
243659	'Styk1'	4.07	0	0	0	0	3.16	0	1.24	10.96	5.76
243725	'Ppp1r9a'	12.78	11.1	9.12	9.83	8.59	11.34	17	12.54	19.63	19.55
243743	'Plxna4'	4.76	2.83	11.1	9.91	7.43	0.54	3.1	4.85	0.91	3.24
243753	'Slc23a4'	0.02	0.14	4.87	0	2.61	0	0.02	0	0.57	0
243755	'Slc13a4'	0	1.32	0	0	2.39	0	0.01	0	0	0
243764	'Chrm2'	0.26	1.25	2.17	0.01	3.79	0	25.9	3.09	2.97	0.89
243771	'Parp12'	1.16	0.18	1.3	6.9	1.78	0.11	4.68	0	0	1.24
243780	'E330009J07Rik'	0.9	0.37	1.75	0.33	2.19	0.11	1.37	0.24	1.63	0.93
243813	'Leng9'	3.39	11	4.3	8.84	11.2	4.69	26	12.67	7.24	6.31
243816	'Gp6'	0.04	0.02	0.41	0.07	0.06	0.11	0.02	0.02	0.03	0.02
243819	'Ppp6r1'	1.41	0.39	1.31	0.65	0.5	4.89	0.14	0.07	0.09	1.58
243833	'Zfp128'	0	1.45	0.01	0.01	4.38	0	4.13	2.4	3.47	1.83
243834	'Zfp324'	0.86	0	0.09	0.04	4.46	0.09	0.36	0	2.33	4.47
243842	'Bicra'	0.12	0.45	1.19	0	1.09	0.01	0.94	0.97	0.77	0.09
243846	'Ccdc9'	0.8	0.61	0.69	0	4.34	4.65	0.07	1.68	6.78	1.41
243853	'Fkrp'	21.28	33.91	18.2	12.9	5.54	13.96	26.3	19.15	14.6	29.01
243864	'Mill2'	0.03	0.31	0.06	0.35	5.78	6.21	0.48	1.75	5.11	10.68
243867	'Fbxo46'	8.27	3.57	5.26	0.34	1.4	0.58	3.52	2.4	2.22	6.59
243874	'Nlrp9b'	0	0	0	0	0	0	0	0	0.04	0
243880	'Nlrp4a'	0	0	0	0	0.01	0.02	0	0	0	0
243897	'Ggn'	0	0	0	0	0.27	0	0	0	0	0.48
243905	'Zfp568'	0.4	0	0.01	0.06	0	0.07	0	0	2.7	0.64
243906	'Zfp14'	8.77	2.65	4.39	5.81	7.85	8.8	3.9	8.25	7.37	7.66
243910	'Nfkbid'	2.09	4.33	1.41	0	6.54	2.22	0.03	3.3	0	0
243911	'Kirrel2'	0	1.57	0	12.7	0	0	0	0	0	0
243912	'Hspb6'	10.11	24.12	19.7	32	0.15	26.82	20.3	24.7	24.27	14.94
243914	'Lgi4'	1.33	0	3.17	0	0.15	2.05	3.57	0.61	1.69	0
243923	'Rgs9bp'	0	0	0.01	0	0	1.5	0	0	0	1.13
243931	'Tshz3'	0.09	0.14	0.32	0.03	2.64	0	0	0	0	0.08
243937	'Zfp536'	0	1.41	2.85	0	1.7	0.63	0	0.55	0	1.14
243961	'Shank1'	4.42	4.23	4.47	3.77	8.47	3.58	2.07	3.16	4.9	2.62
243963	'Zfp473'	0.03	0	0	0.73	0.13	0	0.01	0.01	0	0.19
243967	'Ntn5'	0	0	0	0	0.03	0	0	0	0	0
243983	'Zdhhc13'	9.36	5.88	7.09	22.5	8.95	19	5.81	15.82	4.11	13.14
244058	'Rgma'	0.13	0.47	0.75	0.29	0.07	0.11	0.55	0.05	1.83	0.01
244059	'Chd2'	4.62	6.43	3.38	9.59	5.72	8.43	0.91	4.5	6.71	7.9
244091	'Fsd2'	1.79	0	0.05	0	0.02	0.02	0	0.01	0.01	0.03
244141	'Nars2'	13.9	22.6	14.9	1.11	8.91	13.04	29.9	15.64	12.85	14.42
244144	'Usp35'	1.45	2	7.71	0.03	5.29	0.9	0	2.64	1.88	0.98
244152	'Tsku'	0	0.02	0.02	0	0.32	0	0	0.06	0	0
244198	'Olfml1'	8.45	1.69	0	0	5.81	12.97	0.03	0	3.14	0
244202	'Nlrp10'	0	0	0.01	0	0	0	0	0	0	0
244216	'Zfp771'	34.29	34.32	24.1	16.8	25.1	21.94	56.4	26.78	37.05	16.03
244219	'Zfp668'	6.02	5.61	7.89	1.02	4.85	1.32	3.22	5.83	6.05	5.85
244233	'Cd163l1'	0	0	0	0	0	0	0	0	0.56	0
244237	'Tnfrsf26'	0	0	0	0	0.16	0	0	0	0	0
244238	'Mrgpre'	16.95	8.58	27.2	12.8	11.7	8.76	26.7	18.72	27.23	24.16
244281	'Myo16'	0.25	0.78	0.67	0.73	0.36	0.87	1.22	0.15	0.47	1.39
244310	'Dlgap2'	8.48	1.91	9.26	1.89	1.7	3.55	6.31	5.75	11.52	4.47
244329	'Mcph1'	6.82	2.76	2.32	1.06	3.24	6.86	3.86	1.08	4.14	7.93
244349	'Kat6a'	1.88	2.4	2.55	1.73	0.66	1.83	0.5	1.28	2.76	2.84
244373	'Erlin2'	26.47	20.75	29.6	24.8	33.3	13.39	17	25.48	55.95	35.53
244416	'Ppp1r3b'	0	0	0.01	0	0	0	0	0	0	0
244418	'Prag1'	3.64	7.25	2.7	0.07	0.64	0.07	2.07	1.23	0.01	1.32
244421	'Lonrf1'	0.99	2.47	2.34	5.58	0	0	1.43	1.08	0	0
244431	'Sgcz'	1.99	3.21	0	3.79	1.83	1.75	2.41	8.77	0.86	3.43
244484	'Wdr17'	6.7	6.65	6.12	11.2	9.96	5.53	2.51	8.23	8.68	3.44

244495	'Sgo2b'	0	0	0	0	0.43	0	0	0	0	0
244548	'Elmod2'	2.02	0.9	4.21	3.63	0.98	3.72	7.22	3.09	9.99	7.69
244550	'Podnl1'	0.19	0	1.93	1.4	0	0.85	0	0.02	0	0
244551	'Nanos3'	0.04	0.74	1.46	5.84	1.72	0	0.87	0.59	1.86	0
244556	'Zfp791'	3.87	4.55	4.19	0.07	3.74	0.72	0.09	0.04	6.52	2.69
244562	'Abcc12'	0	0	0	0	0	0.12	0	0	0	1.14
244579	'Tox3'	0.5	0	0	0	1.49	1.43	0.7	0.39	0	0
244585	'Rpgrip1l'	7.04	3.61	3	0.71	9.13	9.91	4.36	1.74	5.93	8.42
244595	'Ces1a'	0	0	0	0	0	0	0	0.03	0	0
244608	'Ccdc113'	6.3	16	7.47	12.8	3.72	8.42	3.17	4.49	0.66	14.57
244631	'Pskh1'	0	2.49	6.34	0	7.47	0	4.39	0.68	0	8.93
244646	'Pkd1l3'	0.01	0.09	0.07	0.01	0	0	0	0.03	0.03	0
244650	'Phlpp2'	2.47	2.09	2.21	4.67	3.88	5.32	3.16	2.45	1.07	3.76
244653	'Hydin'	0	0.39	0.01	0	0.02	0.06	0	0	0	0.01
244654	'Mtss1l'	5.59	8.57	9.62	1.98	11.8	6.97	10	5.49	2.91	8.2
244666	'Sprtn'	0.07	0.97	9.35	0.94	0	0.22	0	0.7	4.09	2.05
244667	'Disc1'	0.55	0.1	0.88	2.3	0	0.01	2.42	1.32	0.01	1.76
244668	'Sipa1l2'	0	0	0.74	0	0.01	0	0	1	2.66	0.17
244672	'Cwf19l2'	1.09	5.98	4.46	7	1.23	9.42	2.63	1.21	1.62	3.43
244682	'Cntn5'	3.57	1.34	4.83	0.59	14.8	7.49	13.3	6.27	0	5.89
244694	'Kdm4d'	0.05	1.17	0.39	8.81	3.55	0.4	0	7.04	2.88	0.02
244713	'Zfp317'	9.96	5.09	7.47	5.01	4.64	2.24	5.37	10.59	8.23	6.91
244721	'Zfp846'	9.49	4.62	9.01	0.88	5.92	2.89	10.4	7.36	5.55	9.81
244723	'Olfm2'	80.25	118.9	66.3	92.3	108	37.46	73.2	96.24	26.94	37.34
244745	'Dpy19l1'	3.39	6.73	15.7	16.6	11.2	7.77	11.2	5.99	3.76	1.79
244757	'Glb1l2'	0.73	0.05	0.01	0	8.77	0	0	0	0	4.74
244810	'AW551984'	195.3	232.6	125	119	84.5	97.4	193	133.9	133.2	209.95
244813	'Bsx'	0	0	0	0	5.48	0	0	0	0	0
244853	'Nxpe4'	2.2	0.59	4.3	0.3	0.15	0	0.02	0	0	1.63
244864	'Layn'	0.73	1.27	1.26	1.44	2.72	0.94	4.23	0.41	4.57	0.63
244867	'Arhgap20'	3.14	2.8	3.01	3.82	3.36	1.23	0.5	0.25	1.05	4.24
244871	'Zc3h12c'	3.15	3.83	1.81	4.68	3.07	0.67	2.29	1.89	2.4	1.15
244879	'Npat'	5.31	1.89	2.58	10.7	0.61	1.08	4.25	2.18	4.36	5.55
244882	'Tnfaip8l3'	0	5.13	1.92	0	0	4.97	1.39	7.26	9.96	2.77
244885	'Sh2d7'	0.28	0.37	0.04	0	0.05	0.44	0.33	0.19	0.17	0.06
244886	'Tmem266'	7.99	7.38	3.62	14.1	3.38	0	3.77	6.12	0	0.04
244891	'Scaper'	9.97	9.88	9.51	5.69	4.05	10.17	9.59	6.7	9.96	7.67
244895	'Peak1'	0.04	0.02	0.16	0.9	0.35	0.25	0.24	0.75	1.13	0.32
244923	'Klhl31'	0.01	0.01	0.02	0	0	0.02	0.33	0.01	0.02	0.11
244954	'Prss35'	3.61	2.82	0.8	1.13	0.63	1.04	0.21	4.38	0.57	0.5
244958	'Mrap2'	306.7	90.7	85.5	76.2	80.2	161.5	96.1	199.8	175.1	194.54
244962	'Snx14'	24.99	24.76	25.5	22.6	17.9	18.58	38.6	19.01	38.5	28.59
245000	'Atr'	0.13	0.92	0.15	0.16	0.02	0	0.11	1.55	1.35	1.91
245007	'Zbtb38'	3.57	5.33	7.44	10.4	2.55	6.51	10.5	2.66	6.44	1.64
245020	'Slc35g2'	91.98	84.94	64.8	0.55	76.4	138.7	101	82.3	134.2	113.82
245026	'Col6a6'	0	0	0	0	0	0	0	0	1.81	0.45
245038	'Dclk3'	9.65	17.16	1.49	0.05	9.98	19.08	15	5.18	9.07	3.21
245049	'Myrip'	2.73	0.14	0.84	0.03	2.07	1.08	1.33	1.52	1.64	1.66
245050	'Fam198a'	1.98	4.93	1.33	0	1.21	0	0	0.04	2.79	0
245126	'Tarm1'	0	0	0	0	0.16	0	0	0	0	0
245174	'Zfp937'	3.6	5.44	3.82	4.07	8.2	2.23	7.1	1.24	8.69	8.2
245269	'Nim1k'	7.45	7.73	8.16	11.5	2.53	1.25	0.42	10.63	0.92	7.73
245308	'Zdhhc19'	0	0	0	0	2.36	0	0	0	0	0
245350	'AA414768'	13.72	28.67	12.9	23	17.5	18.99	9.92	15.96	18.85	7.93
245368	'Zfp300'	3.6	2.6	0.23	0.52	3.03	0.26	1.94	0.21	2.27	1.67
245386	'Tmem255a'	171.9	132.4	103	93.9	140	96.83	102	127.7	97.88	136.39
245403	'Dcaf12l2'	3.01	1.36	0.89	0.04	1.07	0.06	0	1.47	0.01	0.32
245404	'Dcaf12l1'	58.11	48.23	45.4	27	55.7	77.44	68.1	64.41	48.5	60.7

245423	'Gm364'	0	0	0	0	0	0	0	0	0	0.94
245424	'Gpr101'	20.87	7.67	13.2	0	14.5	21.75	35.6	44.21	156.2	88.09
245440	'Gm4988'	37.62	31.74	27.6	15.6	24.8	27.93	29.7	29.22	31.55	27.55
245446	'Slitrk4'	8.25	5.43	7.97	0	14.5	5.37	15.6	12.78	1.24	3.59
245450	'Slitrk2'	4.82	2.03	2.24	3.16	1.52	0.75	1.68	1.72	3.22	1.09
245468	'Pnma3'	8.65	6.11	3.03	4.77	11.4	8.09	6.74	4.8	7.66	15.69
245469	'Pdzd4'	3.9	4.1	1.56	0	2.82	1.75	7.01	1.78	3.55	2.47
245474	'Dkc1'	21.38	13.19	13.2	16.7	6.59	26.47	16.2	18.44	20.11	28.96
245522	'Zc4h2'	2.55	6.38	6.41	15	10.7	12.2	10.7	8.2	8.55	5.2
245526	'Pgr15l'	0.04	0.69	3.85	0.49	0.04	0.63	0	0.61	0.96	1.12
245527	'Eda2r'	0	0	0	0	0	0	0.01	0	0	0.16
245532	'Awat2'	0	0	2.72	0	0	0	0	0	0	0
245537	'Nlgn3'	20.06	26.03	47.6	8.8	21.9	11.19	44.1	16.09	18.2	27.78
245555	'Nexmif'	2.67	2.47	2.53	5.46	1.75	0.25	1.6	2.56	1.05	0.85
245578	'Pcdh11x'	15.46	11.65	17	1.91	6.77	7.62	4.17	12.22	4.15	5.77
245595	'Zfp711'	0.92	7.07	3.76	11.8	5.85	0.32	9.27	6.31	4.53	3.31
245596	'Hdx'	0.82	1	1.13	2.56	1.8	3.47	2.25	1.3	1.28	0
245607	'Gprasp2'	247	227	265	244	200	91.94	291	239.8	289.3	320.09
245610	'Nxf3'	0.72	0	0	0	0	0	0	0	1.83	1.18
245615	'Kir3dl2'	3.76	1.67	0	0	0	0.27	0	5.24	0	0.04
245616	'Kir3dl1'	0	0	0	0	0	0	0	0.08	0	0
245622	'Fam199x'	0.85	0.1	2.84	1.54	0.37	0.49	1.95	0.92	2.21	1.43
245631	'Mum1l1'	2.01	3.54	4.54	3.39	3.43	1.54	4.71	2.12	4.27	0.04
245638	'Tbc1d8b'	0.01	0	0.06	0.02	0.16	0.01	0.01	0.01	0.02	0.01
245643	'Frmpd3'	1.87	7.68	4.34	7.95	10.4	7.46	12.3	1.6	12.89	3.99
245650	'Gucy2f'	0	0.1	0.11	0	0	0	0.58	0.01	0	0
245666	'Iqsec2'	0.86	1.21	2.44	2.85	2.27	1.07	1.19	1.21	0.95	0.28
245670	'Rragb'	8.95	8.22	11.3	9.52	6.46	9.16	13.2	11.98	12.63	9.21
245671	'Klf8'	0	0.03	0.02	0.02	0	0.35	0.02	1.57	5.11	1.22
245683	'Klhl34'	0.98	1.45	0	0.06	0	0	0	0	0	0
245684	'Cnksr2'	1.79	3.96	5.12	6.1	1.28	3.56	3.4	0.67	0.78	1.75
245688	'Rbbp7'	168.2	196.9	133	353	137	117	109	167.7	129.5	164.13
245695	'Tceanc'	2.2	1.06	0.97	0.17	0.57	0	0.01	0.08	0.08	0.03
245828	'Trappc1'	99.65	104.6	82.6	91.9	117	125.1	59.4	124.8	119.5	96.62
245839	'Gzmn'	0	0	0.69	0	0	0	0	0	0	0
245841	'Polr2h'	45.46	31.29	57.5	60.2	53.7	58.37	62.3	51.7	33.5	55.58
245847	'Amdhd2'	19.87	18.48	24.1	4.03	26.3	46.7	14.6	40.9	20.34	35.96
245857	'Ssh3'	16.52	26.5	9.8	13.3	23.3	0.54	30.9	27.9	14.97	35.57
245860	'Atg9a'	22.74	26.5	27	9.15	25.8	9.51	33.8	22.38	39.23	33.4
245865	'Spag4'	1.7	3.3	2.21	0	10.3	5.21	8.03	5.65	30.51	9.66
245866	'Ift52'	30.19	20.92	27.8	33.7	39.1	14.68	21.1	25.91	14.74	42.41
245867	'Pcmtd2'	17.78	19.11	15.4	18.9	12.4	3.79	8.34	19.23	7.08	19.37
245877	'Map7d1'	22.86	22.73	16.8	31.4	27.2	9.72	11.5	12.18	16.63	11.17
245880	'Wasf3'	4.4	6.79	10.5	6.9	14.2	11.43	6.47	5.09	9.68	4.4
245884	'Fam71f2'	0	2.54	1.77	0	0	0	0	0	0.13	0
245886	'Ankrd27'	22.79	10.04	3.9	13.1	6.95	8.36	4.77	10.3	10.38	9.06
245902	'Ccdc15'	2.09	0	1.9	1.29	0.99	1.53	0	0	1.2	0.89
245944	'Vps54'	2.52	0.25	1.05	9.26	0.48	0.41	1.26	2.2	0.04	0.06
245945	'Rbm47'	0	0	0	0	0	0.02	0.28	0	0	0
246048	'Chodl'	55.56	84.1	0.04	54.4	8.65	1.51	0.07	11.81	3.15	0.03
246049	'Slc36a2'	0	0	0.04	0	0	0	0	0.02	0	0
246086	'Onecut3'	0	0.24	0	0	0.19	0.46	0	0	0.34	3.56
246102	'Rttn'	1.02	0	0	2.33	0.09	0.12	0.11	0.9	0.15	0
246103	'Atxn7'	1.26	1.16	1.01	1.16	2.65	0.02	1.72	0.58	0.06	1.23
246104	'Rhbdl3'	0	0	1.15	0.01	0.32	0	0	0.67	0.11	1.48
246133	'Kcne2'	0	0	0	7.77	0.03	0	0	0	0	0
246154	'Vasn'	5.23	6.35	6.16	5.35	0	0.01	2.48	1.01	0	0.01
246179	'Fktn'	21.55	7.61	9.05	11.6	5.38	3.94	9.75	16.59	5.13	8.24

246190	'Otoa'	0.58	0	0	0	0	0	0	0.63	0	0
246196	'Zfp277'	18.35	23.26	25.2	40.3	27.1	22.41	19.7	13.85	37.71	20.73
246198	'Mllt6'	1.51	5.18	2.91	0	5.52	4.71	3.87	3.49	3.78	1.59
246221	'Mpst'	3.52	3.15	0	0	2.91	0	0	2.76	7.71	0.07
246228	'Vwa1'	7.74	1.9	13.9	6.98	7.72	16.11	26.1	10.97	31.77	20.77
246229	'Bivm'	3	4.76	0.24	3.99	1.14	0.88	1.61	5.91	2.56	7.11
246257	'Ovca2'	2.5	0.69	10.7	6.7	2.58	4.7	1.35	2.64	1.72	3.7
246277	'Csad'	16.1	9.79	5.02	17.9	23.9	17.68	17.2	0.23	5.66	14.32
246293	'Klhl8'	3.26	10.61	6.12	0.61	9.29	7.67	3.22	9.38	4.46	0.01
246313	'Prokr2'	0	0	5.68	0	2.36	18.26	0	0	2.55	11.66
246316	'Lgi2'	0.81	0.76	0.03	0	2.36	0	1.3	0.01	0	0.84
246317	'Neto1'	1.82	3.27	0.67	0.92	4.41	7.08	2.5	0.62	0.97	0
246691	'Prok1'	0	0	0	0.71	0	0	0	0	0	0
246694	'Hps5'	0.01	2.1	2.33	0.3	6.54	1.12	1.6	1.62	1.64	1.66
246696	'Slc25a28'	3.1	2.9	4.87	57.2	0.88	2.59	21.4	3.76	19.97	8.43
246703	'Naxe'	207.9	170.4	239	120	286	280.5	309	255	216.1	235.26
246707	'Emilin2'	0	0.17	0.6	0.87	0.05	0	0	0	0	0
246710	'Rhobtb2'	3.07	1.69	1.13	0.23	3.74	3.67	2.8	1.26	5.23	2.22
246727	'Oas3'	0.25	3.01	0.38	0.77	0.32	0.31	0.37	0.24	0.19	0.43
246728	'Oas2'	0	0	0.02	0	0	0	0	0	0	0
246730	'Oas1a'	0	0	0	0	0.86	0.03	0	0	0	4.1
246735	'AY074887'	0.07	0.07	0.29	0.34	0.07	9.12	0.15	0.18	0.32	0
246738	'Dnajc28'	5.1	5.42	4.69	0.02	0	11.99	2.1	8.21	4.87	11.59
246746	'Cd300lf'	0.1	0.01	0.14	0.12	0.07	0.03	0.17	0.03	0.07	0.08
246747	'Adig'	0.61	0	1.64	0	0	0	0	0	0	0
246782	'Atpaf2'	11.7	41.68	34.7	34.1	38.9	30.8	50.1	26.41	39.05	54.4
246787	'Slc5a2'	0	0.38	0	0	0	0	0	0	0	0
246788	'Trpv3'	0	0	0	0	0	0	0	0	0.15	0
252830	'Obox6'	0	1.79	0	0	0	0	0	0	0	0
252837	'Ackr4'	0.04	0	0.09	0	0	0	0.07	0	0	0
252838	'Tox'	7.18	34.04	12.2	27.1	18.2	9.27	17	1.33	15.19	14.82
252864	'Dusp15'	8.73	7.42	5.5	0	6.64	8	9.68	4.78	3.4	8.29
252868	'Odf4'	0.08	0	0.63	0.21	0	4.59	0.46	0	0.09	0
252870	'Usp7'	2.04	2.04	5.46	3.23	5.82	11.37	1.87	1.76	4.92	1.52
252875	'Mios'	1.72	7.42	2.22	4.68	1.17	4.05	9.32	3.45	5.91	0.84
252876	'Gin1'	2.89	1.12	7	1.49	6.13	18.49	13.6	5.95	0	7.72
252903	'Ap1s3'	0.76	0.63	0.78	1.14	2	0.7	0.78	0.86	3	2.92
252966	'Cables2'	1.54	2.22	0.07	6.14	2.87	1	3.78	2.15	0.12	0.03
252967	'Ropn1l'	0.2	2.31	0.17	1.31	0	0.21	0.75	4.33	0	3.48
252972	'Tpcn1'	2.5	0.41	2.44	7.11	0.77	1.41	0.04	0	5.53	0.44
252973	'Grhl2'	0	0.31	0	1.21	0	0	0.18	0.11	0	0
252974	'Tspear'	0	2.4	2.04	5.8	8.47	0	0.02	0	2.45	3.57
257630	'Il17f'	0.02	0.38	0	0	0	0	0	0	0	0
257632	'Nod2'	0	0	0	1.97	0.02	0	0	0.2	0	0
257633	'Acsf3'	11.26	22.92	30	5.89	17.2	22.56	17.7	16.11	23.91	23.05
257635	'Sdsl'	7.63	4.39	2.65	1.89	5.12	10.38	4.66	4.69	1.31	6.04
257882	'Olfr1344'	2.9	0	7.4	0	0	0	0	0	0.42	0.05
257884	'Olfr744'	0	0	0	0	0.05	0	0	0	0	0
257891	'Olfr479'	0	0	0	0	0	0.05	0	0	0	0
257892	'Olfr324'	0	0	4.72	0	0	0	0	2.72	0	0
257926	'Olfr544'	0	0	0	0	0.18	0	0	0	0	0
257932	'Olfr332'	0.45	0.82	2.79	0.29	0	0	0	0	0	1.64
257947	'Olfr543'	0	0	0	0	0	0	0	0	3.33	0
258008	'Olfr1513'	0	0	0.05	0	0	0	0	0	0	0
258019	'Olfr212'	0	0.05	0	0	0	0.02	0	0	0	0
258020	'Olfr213'	0	0	0	0.06	0	0	0	0	0	0
258028	'Olfr901'	0	0	0	0	0	0	0	0	0	3.38
258064	'Olfr316'	0	0	0.05	0	0	0	0	0	0	0.02

258068	'Olfr804'	0	0	0	0	0	0	0	0	0.05	0
258148	'Olfr789'	0	0	0	0	0	0	0	0	0	0.05
258364	'Olfr976'	0.11	0.1	0.3	0.48	0.29	0.28	0.08	0.22	0.18	0.05
258380	'Olfr461'	0	0	0	6.95	0	0	0	0	0	0
258423	'Olfr1510'	0	0	0	0	0	0.05	0	0	0	0
258436	'Olfr458'	0	0	0	0	0	0	0	0.53	0	0
258462	'Olfr1392'	0	0	0	0	0	0	0	1.74	0	0
258470	'Olfr91'	0	0	0	4.75	0	4.22	0	0	0	0
258494	'Olfr318'	0	0	2.86	0	0	0	0	0	0	0
258521	'Olfr860'	0.15	0	0	0	0	0	0	0	0	0
258540	'Olfr771'	0	0	0	0	0	0	0	0	0	0.05
258571	'Olfr1033'	0.26	0.22	0.01	0.2	0.15	0.14	0.33	0	0.66	0.13
258678	'Olfr1441'	0	0	1.15	0	0	0	0	0	0	0
258731	'Olfr491'	0	0	0	0	0	0	0	0	0	0.05
258737	'Olfr1316'	0	0	0	0	0	0.05	0	0	0	0
258745	'Olfr689'	0	0	0	0	3.24	0	0	0	0	0
258783	'Olfr920'	0	0	0	0	0	0	0	0	0	0.85
258819	'Olfr640'	0	0	0.11	0	0	0	0	0	0	0
258837	'Olfr545'	0	0	0	0	2.19	0	0	0	0	0
258854	'Olfr985'	0	0	0	0	0	0	0	0	0	2.8
258858	'Olfr371'	0	0	0.69	0	0	0	0	0	0	0
258871	'Olfr898'	0	0	0	0	0	0	0	0	0	2.67
258896	'Olfr1206'	0	0.11	0	0	0	0	0	0	0	0
258922	'Olfr267'	0.44	0	0	0	0	0	0	0	0	1.7
258938	'Olfr1417'	0	0	0	0	0	0	6.81	0	0	0
259009	'Olfr394'	0	0	0.05	0	0	0	0	0	0	0
259023	'Olfr1055'	0	0	0	0.1	0	0.05	0	0	0.04	0.04
259051	'Olfr658'	0	0	0	0	0	3.1	0	0	0	0.06
259063	'Olfr691'	0	0	1.55	0	0	0	3.38	0	5.22	0
259087	'Olfr622'	0	0	0	0	0	0	0.82	0	0	0
259104	'Olfr613'	7.05	7.49	11.3	10.7	10.5	8.28	7.56	5.04	11.29	7.83
259116	'Olfr559'	0	0	0	0.93	0	0	0	0	0	0
259122	'Olfr635'	0	0.11	0	0	0	0	0	0	0	0
259124	'Olfr638'	0	0.36	0	0	0	0	0	0	0	0
259144	'Olfr456'	0	0	0	0	0	0	0	0	0.13	0
259148	'Olfr329-ps'	0	0	0.05	0	0	0	0	0	0	5.75
259172	'Mfrp'	0.15	0.05	0	0.44	0	0	0.03	0	0	0.05
259277	'Klk8'	0.44	0	0	11.1	0	0	0	0	0	0.07
259279	'Tubgcp3'	18.59	20.68	24	17.7	19.7	4.22	22.1	20.31	20.04	23.57
259300	'Ehd2'	0	0	0.4	0	0	0	0	0	0	0
259302	'Srgap3'	5.99	7.47	8.24	8.94	9.92	4.42	5.19	3.26	5.34	3.54
260296	'Trim61'	0	0.89	0	0	0	0	0	0	0	2.31
260297	'Prrt1'	7.66	8.09	4.31	12.8	9.65	4.45	10.4	6.49	5.2	9.87
260299	'Cadm4'	6.82	4.95	8.34	4.78	5.8	4.38	12.7	7.08	4.93	4.03
260302	'Gga3'	9.66	15.29	19.7	6.07	15.6	8.66	15.1	16.68	31.87	16.22
260305	'Nphp4'	1.91	2.36	0.79	1.28	1.93	3.51	0.86	2.75	1.37	1.59
260315	'Nav3'	1.66	3.88	4.56	0.61	3.73	3.68	2.34	1.86	2.54	3.1
260408	'Prss45'	0	0	0	0	0	0	0	0	2.96	0
260409	'Cdc42ep3'	15.19	15.03	12.6	4.96	11.3	26.27	25.4	16.28	11.84	16.11
260423	'Hist1h3f'	0	2.66	0	0	0	0.79	1.91	0	1.05	2.07
263406	'Plekhg3'	0.61	0.62	0	0	0.46	0	0	0	0	0
26356	'Ing1'	19	14.85	9.29	17.7	8.35	15.5	12.3	7.58	16.05	19.42
26357	'Abcg2'	0.02	9.64	0.69	0.03	7.56	0	9.36	6.81	13.95	12.77
26358	'Aldh1a7'	0	0	0	0	0	0.09	0	0	0	0
26360	'Angptl2'	0	0	0.01	0	0.03	0	0	0	0	0
26362	'Axl'	2.77	0	0.8	0.01	3.41	0.47	10.3	1.05	2.16	5.55
26363	'Btd'	6.61	1.24	5.88	21.8	2.67	10.8	15.6	3.7	9.5	2.97
26364	'Adgre5'	0	2.08	0	0	0	0	0	2.26	2.73	0.01

26365	'Ceacam1'	0	0	0	0	1.15	0	0.02	0	0	0
26366	'Ceacam10'	0	0	0	0	0	0	0	6.72	0	0
26368	'Ceacam9'	0	0	0	0	0	0	2.93	0.65	0	0.47
26370	'Cetn2'	122.8	111.8	53.2	93.7	85.1	108.8	50.9	126.4	73.18	153.45
26371	'Ciao1'	27.27	22.08	31.3	19.7	22.2	26.57	29.3	36.23	38.54	32.73
26372	'Clcn6'	9.28	11.3	9.35	4.34	9.13	9.1	12	8.72	9.02	5.43
26373	'Clcn7'	16.64	20.77	23.1	19.2	25.8	19.8	20.1	30.53	20.39	30.01
26374	'Cop1'	1.38	2.6	1.4	6.24	0.91	2.65	0.2	1.56	4.53	1.49
263764	'Creg2'	16.28	2.62	19.4	9.16	12	3.48	12.3	3.35	20.42	4.46
26377	'Dapp1'	0	0.11	0	0	6.78	3.51	0	0	0	0
26378	'Decr2'	9.07	12.38	18.1	12.5	12.3	33.58	21.5	17.15	11.05	23.6
26379	'Esrra'	0.88	0.68	0.18	2.5	3.56	0.08	1.74	0.83	2.29	0.15
26380	'Esrrb'	0	0	0.44	0	0	0.01	0	0.01	0	0.99
26381	'Esrrg'	0.81	0.02	4.02	2.19	1.31	0.06	0.12	0	0.35	0.37
26382	'Fgd2'	0	0	0.03	7.77	21.4	10.3	0	0	0.13	0.37
26383	'Fto'	44.3	61.08	78.1	29.8	58.4	22.28	52.1	87.51	115.2	108.82
26384	'Gnpda1'	46.66	28.08	32.3	28.1	60.6	28.21	34.8	64.14	41.55	52.34
26385	'Grk6'	2.69	5.7	30	2.14	8.16	7.98	4.8	11.27	19.29	7.48
26386	'Hsf4'	3.66	5.85	20.2	9.59	23.6	12.68	34.8	17.22	21.23	21.32
263876	'Spata2'	3.13	11.27	16.6	22.4	2.26	0.01	9.47	3.22	6.99	5.4
26390	'Mapkbp1'	1.18	1.92	3.7	3.67	2.63	1.42	6.95	0.23	0.65	1.49
26394	'Lypla2'	107.1	87.39	75.9	59.8	95.2	85.4	104	100.1	136.8	117.6
26395	'Map2k1'	293.9	254	202	233	154	158.8	284	200.8	199.2	199.63
26396	'Map2k2'	48.59	42.72	65.8	23.9	22.1	37.68	48.5	20.9	47.87	13.44
26397	'Map2k3'	4.85	9.03	11.9	5.78	14.1	3.18	16.7	6.62	8.62	13.89
26398	'Map2k4'	48.61	55.09	36.6	75	53.2	49.48	47	63.41	75.74	56.94
26399	'Map2k6'	1.01	1.79	3.61	6.12	4.21	13.08	3.11	3.36	0	7.31
26400	'Map2k7'	43.42	26.54	35.8	8.63	17.9	24.64	20.4	25.98	52.27	23.1
26401	'Map3k1'	1.9	2.27	2.33	2.01	1.29	2.22	3.19	3.86	3.57	6.89
26403	'Map3k11'	4.03	4.42	6.69	0.02	0	3.93	1.71	4.22	1.48	0.2
26404	'Map3k12'	34.19	18.36	42.1	16.7	35	11.82	29	20.75	19.59	24.19
26405	'Map3k2'	0.61	0.56	0.81	2.79	0.61	0.56	0.72	0.25	1.54	0.52
26406	'Map3k3'	2.28	2.41	2.98	0.64	3.85	4.62	0.56	1.37	8.3	1.13
264064	'Cdk8'	3.05	1.79	9.58	12.8	6.97	1.95	0.52	6.08	1.73	2.73
26407	'Map3k4'	8	6.59	4.74	6.66	4.87	4.84	9.09	6.43	7.16	9.36
26408	'Map3k5'	2.26	5.28	3.39	4.73	2.61	2.39	1.16	2.27	3.87	3.25
26409	'Map3k7'	0.2	1.25	0.76	1.4	0.82	1.15	0.39	0.74	0	2.13
26410	'Map3k8'	0	0.66	0.58	6.13	0.03	1.81	4.86	0	0	0.91
26411	'Map4k1'	2.75	1.6	0	5.41	2.46	0	0.05	0	8.64	0.02
26412	'Map4k2'	10.24	14.41	20.2	6.03	15.1	7.37	10.6	13.48	12.92	9.12
26413	'Mapk1'	53.11	52.94	58	125	55.8	79.79	63.8	60.83	61.19	41
264134	'Ttc26'	0.74	0.07	0.58	8.18	0.08	2.61	3.38	0.06	2.62	1.56
26414	'Mapk10'	71.65	92.91	84.1	59.3	72.3	128	115	62.57	85.18	91.06
26416	'Mapk14'	5.32	2.2	12.1	3.52	5.79	1.37	0.02	1.26	9	4.19
26417	'Mapk3'	24.99	25.36	14.5	41.3	50	26.48	29.1	22.39	13.1	28.82
26419	'Mapk8'	1.81	6.48	3.92	6.78	5.59	2.48	13.9	3.29	2.47	4.5
26420	'Mapk9'	19.99	14.03	21	17.6	14	4.74	17.7	19.35	21.41	12.1
26422	'Nbea'	6.96	2.94	3.36	3.68	2.52	5.01	3.99	3.05	5.73	2.88
26424	'Nr5a2'	0	0.18	0.01	0	0	0	0	0.03	0	0.02
26425	'Nubp1'	1.35	0.23	5.67	6.67	2.78	1.11	0	2.98	11.12	2.07
26426	'Nubp2'	40.82	42.2	29.4	84.8	37.5	36.72	16.2	36.99	64.96	47.3
26427	'Creb3l1'	1.32	0	0	19.4	3.16	0	0	1.18	0.04	0.13
26428	'Orc4'	43.17	36.83	35.1	45.1	29.2	37.27	32.3	31.11	37.15	44.61
26429	'Orc5'	26.04	16.98	43.8	31	22.2	40.14	37.9	26.92	37.28	40.58
26430	'Parg'	4.77	2.37	4.78	0	3.25	3.25	0	4.31	4.36	0.36
26431	'Git2'	7.09	9.97	10	7.82	4.71	7.87	5.58	2.65	2.72	1.86
26432	'Plod2'	0.32	0	0.25	0.17	0.52	2.49	0	0.68	0	0.05
26433	'Plod3'	12.33	9.57	5.25	16.5	22.4	2.14	6.07	8.2	0.16	4.24

26434	'Prnd'	0	0	0	3.58	0	0	3.72	0	0.87	0
26436	'Psg16'	23.25	12.46	8.49	19.3	10.6	8.56	20.1	13.3	7.11	13.1
26440	'Psma1'	218.8	256.4	229	195	180	298	229	231.9	161.4	249.72
26441	'Psma4'	130.7	69.7	78.5	149	98.4	153.8	108	160.8	134	133.12
26442	'Psma5'	272.8	166.4	242	323	236	243.8	181	268.9	222	220.46
26443	'Psma6'	244.5	287.1	292	380	192	290.4	250	286.3	272.4	268.62
26444	'Psma7'	228.7	282.8	369	344	362	435	297	443.8	373.3	364.11
26445	'Psmb2'	385.3	320.1	367	471	421	451.8	339	512.2	390.3	386.75
26446	'Psmb3'	348.9	307	454	685	442	485.2	380	483.5	210.3	386.01
26447	'Poli'	8.19	7.22	5.6	0.32	10.9	2.91	7.66	7.19	8.48	11.33
26448	'Mok'	3.39	16.34	6.74	0.46	18.5	30.55	1.92	18.35	8.7	20.34
26450	'Rbbp9'	21.82	49.42	20	12.8	25.6	19.78	21.7	25.08	13.38	25.83
26451	'Rpl27a'	89.13	102	98.3	85.8	92.5	87.43	96.4	52.84	66.04	57.18
26456	'Sema4g'	6.82	0.47	5.89	1.57	4.74	8.24	3.15	3.88	5.42	1.83
26457	'Slc27a1'	23.05	24.47	23.5	22.3	21	12.92	12.3	25.37	10.67	15.76
26458	'Slc27a2'	26.26	6.12	19.6	0	8.64	4.47	16.9	8.96	14.32	8.45
26459	'Slc27a5'	0	0	0	0	0.88	0	0	0.14	0	0
26462	'Txnrd2'	13.79	18	25.8	1.52	22.9	24.46	25.1	15.58	29.51	26.51
26465	'Zfp146'	4.29	9.47	0.07	3.82	16	14.43	0.02	11.13	8.65	4.15
26466	'Zfp260'	15.95	12.5	12.1	9.78	15	9.36	26.2	19.24	25.28	21.23
264895	'Acsf2'	0.06	0.08	0.04	0	0	0	0	0.03	0.25	0
26549	'Itgb1bp2'	0.1	0.53	0.19	0	0	0.07	0.12	0	0.03	0.07
26554	'Cul3'	9.61	8.3	5.76	14.9	12.3	7.14	4.98	5.15	1.91	9.23
26556	'Homer1'	1.45	2.74	1.99	6.01	2.15	0.63	1.68	1.7	5.99	2.16
26557	'Homer2'	0.51	0.06	0.8	1.13	0.42	0.25	0.09	0.53	1.09	1.95
26558	'Homer3'	0.93	0	1.01	6.22	0	0.58	0	0	0.02	0
26559	'Hunk'	0	4.01	4.4	0	1.31	3.06	1.5	6.54	3.9	1.71
26561	'Mmp23'	1.15	6.9	1.43	0	0.46	6.41	3.87	2.6	11.67	0.08
26562	'Ncdn'	171.3	127.3	195	37.1	194	119.5	298	223.6	141.6	105.24
26563	'Ror1'	0	0	0	0	0.39	0	0	0	0	0
26564	'Ror2'	0	0	0	0	0	0	0	0	0.01	0
26565	'Pla2g10'	0	0	0	3.41	0	0	0	0	0	0
26568	'Slc27a3'	0.35	0	2.71	0	2.61	4.3	5.95	6.4	9.73	11.5
26569	'Slc27a4'	9.24	6.53	11.3	9.27	8.95	8.37	3.49	4.52	18.58	6.26
26570	'Slc7a11'	0.34	0.01	0.13	0	0	0.03	2.24	0.03	0.01	0.92
26572	'Cops3'	137.1	107.8	132	99	141	204.4	119	167.7	123.1	177.03
26611	'Rcn2'	378.2	322	246	414	273	547.7	270	324.1	410.6	421.77
266614	'Ly6g5b'	0	0	0	0	0	0	0	0	1.39	0
266632	'Irak4'	69.3	20.5	50.3	0	12.5	399.5	0	301.1	222.6	195.02
266645	'Acmsd'	0.21	0.11	0.23	0.34	0.27	0.13	0.16	0.07	0.19	0.06
266690	'Cyb5r4'	2	2.53	8.52	2.55	0.73	3.2	7.47	4.41	11.87	0
266692	'Cpne1'	21.83	12.61	16.2	8.64	18.4	20.92	21	18.33	26.15	16.82
266781	'Snx17'	109.7	112.4	106	110	94.8	152.7	126	106.7	83.05	140.27
267019	'Rps15a'	10.32	12.06	16.2	16.2	10.3	6.48	16.3	11.64	7.18	8.14
26754	'Cops5'	70.33	43.94	89.8	73.6	102	82.14	76.4	78.71	39.29	66.11
26757	'Dpysl4'	9.59	17.8	24.5	24.5	18.8	5.14	31.2	30.74	21.45	16.46
268281	'Shprh'	2.38	7.73	1.77	5.89	5.91	0.89	2.25	2.62	3.95	1.03
268288	'Samd3'	8.9	3.2	10.4	0	5.24	87.08	3.24	4.25	56.66	81.98
268291	'Rnf217'	0	0	0	0.39	0	0	0.68	0.85	0.3	0.53
268294	'Zbtb24'	12.31	12.83	8.61	6.05	7.46	7.24	6.59	7.98	14.12	7.14
268297	'Scml4'	16.13	4.03	2.28	4.21	1.45	0.36	5.84	5.06	4.35	4.18
268301	'Sowahc'	3.86	1.86	8.36	3.52	0.86	8.47	1.44	3.02	1.25	2.12
268345	'Kcnc2'	7.18	10.93	5.14	4.23	4.3	8.93	7.37	2.62	7.89	7.4
268354	'Fam19a2'	11.33	4.71	17.6	0.02	7.29	7.24	14.3	8.62	10.43	10.66
268373	'Ppia'	4144	3845	3653	3215	3743	4789	4447	4678	3177	4043.98
268379	'Abca13'	0	0	0	0	0	0	1.23	0	0	0
268390	'Ahsa2'	52.61	29.03	8.96	14.2	22.9	11.65	26.2	38.03	12.94	24.67
268395	'Mpg'	27.51	21.96	27.5	19.1	22.8	35.89	41.3	41.47	38.36	25.12

268396	'Sh3pxd2b'	0.27	0.45	0.1	0	0.33	0	0.86	0.64	0.01	0
268417	'Zkscan17'	0.98	0.09	2.33	0.01	0.08	1.78	0.26	0.53	0.25	0.03
268420	'Alkbh5'	16.52	17.39	15.8	19.9	11.8	5.44	9.53	10.53	13.68	17.34
268445	'Ankrd13b'	3.51	5.72	1.36	29	2.2	0.95	3.11	1.69	3.14	0.03
268448	'Phf12'	4.65	2.92	2.02	10.9	8.24	0.44	2.04	1.47	2.81	3.36
268449	'Rpl23a'	147.7	236.9	251	335	202	353.5	320	191.2	226	202.98
268451	'Rab11fip4'	13.02	10.93	7.61	10.4	8.59	5.72	7.24	4.85	8.35	4.84
268465	'Eme1'	0	0	0	0	0	0	0.6	0.03	0	0
268469	'Zfp652'	0.33	3.94	0.02	0.53	0	0	0	0	0	0.25
268470	'Ube2z'	16.96	4.91	10.9	2.96	10.3	6.69	2.4	8.62	4.29	6.82
268480	'Rapgefl1'	4.39	1.81	2.37	0.02	5.43	0.51	2.66	3.56	5.9	1.08
268481	'Krt222'	8.6	9.89	15.1	18.1	10.4	4.53	10.2	17.25	4.67	19.28
268482	'Krt12'	0	2.47	0	0	0	0	4.16	1.61	0	0
268490	'Lsm12'	13.33	15.34	10.8	28.7	19.2	10.8	18.4	20.66	22.87	12.56
268491	'Meioc'	0.13	0	0	0	0.06	0	0	0	0	0
268510	'Mgat5b'	1.38	0	2.95	0.02	0.73	0	10.4	1.77	4.19	4.25
268512	'Slc26a11'	0.04	15.33	0.02	0	7.47	36.27	4.67	13.28	44.5	14.82
268515	'Bahcc1'	0.33	1.13	0.76	0.01	0	0.26	0	0.15	1.28	0.92
268527	'Greb1'	1.83	2.8	0	2.48	0.02	0	0	0.54	0	0
268534	'Sntg2'	3.08	3.44	2.93	0	0.03	7.17	4.63	9.11	9.75	11.76
268564	'Zbtb1'	3.05	3.39	2.22	0	0.01	0.34	0	1.99	2.34	0
268566	'Gphn'	26.24	29.22	43.7	57.8	34.1	22.4	20.7	24.88	33.6	19.76
268567	'Tmem229b'	10.9	0.23	0.01	0	9.13	0.66	8.78	0.81	0.08	2.19
268595	'D430019H16Rik'	4.36	5.07	5.09	3.77	6.27	2.86	11.5	4.75	5.19	7.1
268656	'Sptlc1'	11.03	7.86	15.9	10.6	10.2	6.16	12.5	1.41	18.22	9.97
268663	'Cdhr2'	2.14	6.79	5.81	1.6	0	4.93	0.01	3.73	9.19	2.06
268670	'Zfp759'	0.56	0.01	2.4	4.49	0	0.31	0	0	2.36	0.21
268697	'Ccnb1'	0	0	1.97	0.02	0	0	0	0	0	3.21
268706	'Slc38a9'	2.18	2.34	0.21	2.3	0.34	4.4	2.95	6.48	2.98	2.95
268709	'Fam107a'	5.43	3.02	0.86	9.53	0.03	0.1	0	9.56	0	0.01
268721	'Zswim8'	6.99	7.63	2.48	11.2	7.29	5.67	4.69	5.31	6.32	3.4
268729	'Frmpd2'	0	0	0.21	0	0	0	0	0	0	0
268739	'Arhgef40'	1.47	6.31	7.93	11.6	5.93	4.89	0.43	2.3	4.22	11.19
26874	'Abcd2'	2.57	2.65	0.27	0.04	0.03	0.73	0.06	1.72	2.57	3.93
268741	'Tox4'	15.3	20.16	11.9	0.95	8.14	14.45	16.9	13.14	12.8	11.01
268747	'Carmil3'	2.08	2.83	2.17	6.82	4.75	2.2	3.54	0.38	6.67	7.97
268749	'Rnf31'	3.61	6.32	14.1	12.1	2.59	11.54	8.14	0.95	1.16	11.37
26875	'Pclo'	5.36	2.98	5.47	4.8	3.13	2.9	7.23	4.04	3.79	2.95
268752	'Wdfy2'	0.92	1.83	2.17	2.4	4.05	1.03	0.9	1.76	1.95	2.69
268756	'Gulo'	0	0	0.43	0	0	0	0	0	0	0
268759	'9930012K11Rik'	3.76	9.22	2.75	0	0.02	0	0	0	4.33	0
26877	'B3galt1'	29.44	8.24	12.4	13.6	2.95	0.02	1.24	10.87	1.8	1.74
26878	'B3galt2'	8.85	7.54	1.84	2.3	0.37	2.87	6.78	2.4	2.85	1.63
268780	'Egflam'	0	0	0.01	0	0	0	0	0.12	1.83	0
268782	'Agxt2'	0	0.11	0	0	0	0	0	0	0	0
268783	'Mtmr12'	4.62	0.61	2.71	8.09	0.01	0	3.59	1.52	3.71	0.22
26879	'B3galnt1'	116.5	76.05	106	88.3	55.8	49.89	111	102.5	153.4	81.57
268807	'Klhl38'	0	0	0	0	0	0	0	0.02	0	0.02
268822	'Adck5'	16.34	21.13	13.8	0.76	21.6	14.33	10.9	15.52	8.33	20.48
26885	'Casp8ap2'	0.09	1.83	1.8	7.45	0.52	0.25	1.83	0.92	2.6	0.99
268857	'Nlrc3'	3	3.11	1.74	2.07	2.22	2.61	1.03	2.15	3.48	0.97
268859	'Rbfox1'	52.54	70.11	75.2	69.8	57.2	75.22	103	68.36	44.31	37.1
26886	'Cenph'	0	1.03	1.61	0	3.09	0	0	0	0	0
268860	'Abat'	101.1	74.61	100	71.4	60.8	34.75	69.7	101.9	40.25	57.55
268878	'Atp13a5'	1.6	0	0	0	0	2.09	0.51	2.18	0	0.2
26888	'Clec4a2'	0.85	0.75	1.21	1.53	1.65	2.5	1.29	0.4	0.82	0.98
268880	'Xxylt1'	4.36	3.98	0.61	0	0.14	0	6.8	1.55	6.05	1.58
268882	'Fbxo45'	1.84	2.66	1.6	2.42	2.84	2.63	2.6	0.26	0.9	0.41

26889	'Cln8'	9.4	6.7	14.8	0.83	1.45	14.27	9.71	14.43	13.02	7.75
268890	'Lsamp'	70.68	99.38	64.2	89.5	45.1	85.69	62.2	48.46	66.23	60.96
268902	'Robo2'	3.82	0.33	0.01	0.01	0.72	0.4	5.1	2.46	0.66	0.95
268903	'Nrip1'	3.56	4.38	6.54	4.35	1.34	0.59	0.24	4.48	2.72	1.17
26891	'Cops4'	187.5	164	121	180	157	196	125	183.8	175.7	183.01
26893	'Cops6'	286.8	235.5	278	287	351	380.4	298	430.6	339.7	317.88
268930	'Pkmyt1'	1.54	1.64	5.02	0	7.86	0	0	4.66	17.05	1.48
268932	'Caskin1'	4.2	4.42	12.4	8.88	9.9	8.44	2.98	7.91	2.25	3.69
268933	'Wdr24'	19.58	7.81	7.11	2.22	3.54	1.86	6.73	8.03	3.28	5.95
268934	'Grm4'	1.61	1.38	0	0	0.12	0.01	0.03	0.03	0.61	2.21
268935	'Scube3'	1.31	0.86	0.01	0.02	0.48	0.02	0.01	0.08	0	0
268936	'Brpf3'	0	0.09	0.39	2.83	0.03	1.52	3.95	1.31	0.04	0.99
26894	'Cops7a'	186.8	183.5	146	153	87.7	169.6	153	193.5	93.69	162.6
268949	'Mucl3'	0	0	0	0	0	0.16	0	0	0	0
26895	'Cops7b'	22.6	9.27	14.3	0	31.5	0.06	15.4	22.45	22.62	15.43
268958	'Capn11'	0	0.96	0	1.19	0	0	0	0	1.03	2.79
26896	'Med14'	2.97	1.52	2	5.09	5.64	0.99	0.3	3.06	6.28	1.03
26897	'Acot1'	2.71	20.25	2.49	8.55	13.7	11.13	0	14	16.98	7.18
268977	'Ltbp1'	0	0.23	0	0.93	0.73	0.37	0	0	0	1.26
268980	'Strn'	4.59	1.76	1.9	2.75	0.8	1.29	0.08	1.2	2.23	1.46
268996	'Ss18'	2.1	4.78	9.25	3.46	5.13	2.89	17	2.32	9.97	12.67
26900	'Ddx3y'	32.48	29.77	0.05	0.01	21.1	9.08	21	25.91	24.53	25.14
269003	'Sap130'	6.42	16.62	21.7	7.8	16.2	6.91	23.3	15.16	24.3	14.23
26901	'Ss18l2'	94.91	151	141	130	128	136.5	110	89.08	62.58	120.57
269019	'Stk32a'	13.2	8	10.9	21.5	1.97	2.74	0.04	15.17	11.51	6.71
269023	'Zfp608'	2.43	3.01	0.37	2.35	2.52	2.02	1.56	2.33	3.93	4.27
26903	'Dysf'	0	0.23	0.04	0.01	0.19	0.91	0	0	0.36	0.74
269033	'4930503L19Rik'	4.76	1.48	3.82	0	3.01	0	20	3.8	5.38	1.88
269037	'Ctif'	1.91	4.25	1.3	3.17	3.33	2.94	2.68	3	0.7	0.66
26904	'Sh2d1b1'	0	0.41	0	0	0	0	0	0	0	0
26905	'Eif2s3x'	19.47	22.91	45.3	23.7	13.4	6.82	24.9	20.18	23.48	27.07
269053	'Gpr152'	0	0	0	0	0	0	0	0	0	1.08
269060	'Dagla'	0.91	0.93	4.18	0.08	2.84	3.02	2.34	0.28	1.38	0.78
269061	'Cpsf7'	9.56	14.37	19.5	17.4	12.1	4.76	18.8	10.65	26.73	19.45
26908	'Eif2s3y'	20.79	15.41	0.07	1.56	27	28	31.7	27.13	36.35	42.74
269109	'Dpp10'	11.31	10.99	8.66	7.85	26.7	15.94	15.1	18.65	14.69	19.01
269113	'Nup54'	6.55	9.79	4.89	14.3	2.65	13.61	7.26	7.4	9.47	12.64
269116	'Nfasc'	21.4	15.73	22.3	24.2	11.3	9.98	19.9	9.55	12.52	16.25
26912	'Gcat'	8.41	4.54	7.49	7.37	7.86	14.23	16.5	3.84	23.05	7.34
269120	'Optc'	0.12	1.7	2.86	0	21	38.01	6.96	6.18	4.29	7.08
26913	'Gprin1'	3.04	7.23	1.56	5.01	3.54	0.08	2.05	5.45	2	2.28
269132	'Colgalt2'	0	0.08	0.88	0	0	0	0	0.02	2.77	0.19
26914	'H2afy'	39.81	44.58	43.2	50	45.4	41.2	49	51.56	53.87	31.81
269152	'Kif26b'	0.12	0.01	1.79	0.02	0.42	1.35	1.75	0.26	0	0.08
26918	'Ern2'	3.6	0	0.37	0	0.02	0.02	0.73	6.64	2.91	0.11
269180	'Inpp4a'	99.19	100	61.4	112	124	93.95	54.6	116	141.5	114.36
269181	'Mgat4a'	3.09	6.57	3.97	2.04	3.18	6.95	5.08	3.51	3.12	3.19
26919	'Zfp346'	0.58	3.57	0.03	0	11.2	9.15	16.8	1.48	1.07	0.05
269198	'Nbeal1'	1.52	1.56	1.89	0	1.96	0.29	1.33	0.61	1.41	1.96
26920	'Cntrl'	3.34	2.69	0.77	4.28	8.56	1.34	1.29	0.36	2.67	2.46
269209	'Stk36'	0.11	2.61	1.3	1.25	0.71	0.08	0	0	0	2.55
26921	'Map4k4'	1.31	2.49	0.31	0.78	0.93	0.13	0.81	0.1	0.25	1.43
26922	'Mecr'	16.32	22.6	9.32	19.7	21.9	16.52	2.26	28.75	21.49	29.79
269224	'Pask'	0.01	0.4	1.04	0.16	0	0	0	0	0	0.54
269233	'Fam171a1'	2.54	13.45	7.51	2.23	4.26	8.27	10.2	4.82	2.77	13.1
269252	'Gtf3c4'	1.26	0.95	0.77	3.96	2.25	0.72	0.15	1.1	1.24	1.05
269254	'Setx'	2.64	1.18	2.98	5.68	2.63	0.58	1.5	3.12	2.46	3.99
26926	'Aifm1'	16.52	14.91	7.41	0	11.2	16.09	10.8	18.08	15.03	7.85

269261	'Rpl12'	202.2	155.8	141	250	151	162.8	137	166.9	108.7	184.89
269275	'Acvr1c'	0.65	0.66	0	0.35	0	0	0.1	0.55	0.01	0.12
26931	'Ppp2r5c'	33.03	29.77	34.7	79.1	45.9	49.19	48.2	41.87	32.65	43.97
26932	'Ppp2r5e'	5.98	5.62	6.89	13.7	5.27	1.06	3.82	3.28	3.59	2.36
269336	'Ccdc32'	89.51	61.44	64.5	38.1	64.1	104.2	76.5	82.94	66.42	99.13
269338	'Vps39'	23.27	21.77	34.1	42.7	26	19.93	13.8	24.38	34.32	38.87
26934	'Racgap1'	4.18	10.43	11.5	12.5	4.54	0	11.1	1.83	6.39	3.81
269344	'Ell3'	1.91	3.24	3.07	6.42	3.32	2.2	0.72	1.81	2.97	4.03
269346	'Slc28a2'	0.02	0	0.17	0	0	0	0.02	0	0	0
269356	'Slc4a11'	0.02	2.23	0	0	0.02	0	0	1.33	0	0
26936	'Mprip'	5.01	7.18	6.22	9.52	6.3	6.05	6.92	9.92	7.64	6.46
269378	'Ahcy'	22.09	47.66	35.3	35.1	30.4	16.96	44	37.94	30.17	28.4
26938	'St6galnac5'	21.05	8.64	17.2	0	18.7	13.95	7.57	19.85	7.95	2.3
269389	'Tox2'	52.22	41.66	86.8	25.2	48.1	25.37	94.3	56.34	56.51	38.07
26939	'Polr3e'	14.07	15.56	18.8	16.5	17.6	11.11	16.8	16.75	10.18	6.7
269397	'Ss18l1'	11.25	7.56	13.6	3.51	13.2	4.13	6.46	13.81	12.89	6.69
26940	'Ecsit'	16.7	46.14	49	71.2	53.2	61.41	63.5	40.95	50.39	39.73
269400	'Rtel1'	3.31	6.66	8.48	4.52	5.98	4.45	9.7	2.58	3.36	4.55
269401	'Zfp512b'	9.73	13.68	14	20.1	11.4	8.91	12.9	9.84	7.42	7.54
26941	'Slc9a3r1'	1.82	9.38	7.98	4.4	8.84	9.7	25.9	5.33	5.48	2.45
26942	'Spag1'	1.55	1.65	7.01	2.09	3.18	2.46	5.25	1.77	4.24	1.03
269423	'Abhd18'	4.66	3.7	3.38	3.46	8.3	5.07	6.65	4.23	2.77	1.15
269424	'Jade1'	6.89	2.23	4.26	12.2	1.71	0.37	3.51	3.34	2.26	2.47
26943	'Serinc3'	66.15	41.54	57.9	42.6	45.3	19.99	40.4	35.69	41.89	53.52
269437	'Plch1'	1.42	0.35	3.81	0	1.98	0.44	1.12	0.23	0	0
26944	'Tinag'	0	0	0	0	0	0	0	0	0	0.33
26945	'Tpsg1'	0	0	0.05	0.15	0	0	0	0	0.04	0
26946	'Trpc7'	11.71	7.84	5.48	8.87	14.1	22.1	16.1	15.74	11.74	15.77
269470	'Wdr3'	10.76	10.56	2.57	1.39	4.13	4.4	12.5	8.78	10.24	5.36
269473	'Lrig2'	7.84	2.34	8.41	11.5	6.42	3.2	6.63	6.32	3.24	4.25
26949	'Vat1'	39.31	33.29	25.6	26.6	38.7	23.19	29	37	56.88	54.51
26950	'Vsnl1'	271.8	270.2	269	101	316	396.7	266	420.2	593.6	455.84
26951	'Zw10'	10.57	7.37	9.62	9.93	3.74	13.48	11.4	9.15	8.88	10.32
269513	'Nkain3'	1.72	8.93	5.75	4.43	1.93	0.65	3.18	3.93	0.1	5.55
269514	'Fbxl4'	12.17	19.74	16.5	16.5	4.92	21.97	3.03	26.53	28.39	31.05
269523	'Vcp'	182.4	192.8	157	142	154	198.1	192	160.6	174	258.84
269529	'Fbxo10'	16.96	17.36	37.2	18	6.97	7.99	22	20.96	18.46	22.66
269536	'Tex10'	25.54	14.55	24.5	5.47	7.33	12.62	4.89	16.94	26.89	15.21
269582	'Clspn'	1.12	0	0.02	0.04	0	0	0	0.01	0.04	0
269585	'Zscan20'	1.41	0.09	0.1	0.05	0.65	1.16	0.02	0.54	0.19	1.37
269587	'Epb41'	3	1.88	2.97	6.46	2.21	2.6	1.56	1.59	0.6	3.96
269589	'Sytl1'	0	0.05	0	0.65	0	0	0	0	0	0
269593	'Luzp1'	2.75	4.68	6.71	8.55	8.97	3.56	9.43	4.82	6.87	3.57
269604	'Gpr157'	0.4	0.39	0.56	0.87	0.68	0.76	0.95	0.67	2.26	0.99
269608	'Plekhg5'	9.49	6.69	8.28	7.22	1.02	0.03	8.72	7.86	7.96	6.59
26961	'Rpl8'	165.8	212.5	250	173	170	298	199	151.6	137.8	234.88
269610	'Chd5'	1.97	3.98	6	2.47	5.15	6.13	4.94	3.83	4.92	5.2
269614	'Pank4'	31.55	36.19	20.2	21.8	32.4	17.63	84	36.86	33.46	44.44
269615	'Plch2'	0.33	0	4.45	1.35	1.49	5.71	1.8	4.18	0	2.62
269623	'Rbm48'	7.4	2.57	8.7	16.2	10.9	4.62	5.23	10.29	10.53	10.05
269629	'Lhfpl3'	5.91	23.44	37.9	10.2	11.9	4.14	17.6	19.12	15.33	11.47
269633	'Wdr86'	1.77	0.06	0.03	1.83	0.09	0	0.34	0.55	0.01	0.01
269639	'Zfp512'	23.64	42.63	60.4	40.1	42.3	48.39	28	45.74	64.48	43.46
269642	'Nat8l'	7.08	6.32	5.44	8.7	16.2	2.7	15.4	3.18	1.3	0.76
269643	'Ppp2r2c'	9.48	13.77	13.4	11.5	21	8.48	21.3	12.73	12.59	10.54
26965	'Cul1'	33.15	45.48	56.2	45.4	35	44.66	62.1	30.02	42.05	49.09
26968	'Islr'	0	0	0	0.03	10.3	0	15.9	0	0	0.02
269682	'Golga3'	2.8	8.02	4.02	7.71	5.99	0.29	4.92	10.18	10.47	1.18

269693	'Ccdc60'	0	0.2	0	0	2.32	0	0	1	0	0.39
269695	'Rnft2'	6.1	7.55	6.38	6.64	6.22	2.11	3.72	1.17	2.55	6.11
269700	'Hectd4'	4.09	5.24	8.72	5.64	4.98	3.64	9.15	5.61	5.17	6.46
269701	'Wdr66'	0.07	2.93	0.36	2.42	1.35	0.3	0	0.3	0	1.09
269702	'Mphosph9'	2.74	7.3	2.4	1.82	6.74	5.91	5.26	4.85	4.58	2.43
269704	'Zfp664'	16.32	8.4	12.6	5.44	6.35	3.19	11.8	12.55	12.67	0.87
269713	'Clip2'	0.48	1.25	1.11	1.11	1.34	0.16	0.22	0.51	1.55	1.39
269717	'Orai2'	6.75	6.37	22.3	0.9	10.8	1.31	10.7	8.52	5.58	5.39
26972	'Spo11'	0	0	0	0	0	0	0	0	0	0.85
269774	'Aak1'	3.17	8.75	5.47	7	6.33	2.63	7.5	6.56	3.38	7
269784	'Cntn4'	8.34	0.68	8.32	11	13.3	14.11	11.7	6.8	19.27	15.39
269788	'Lhfpl4'	12.86	12.27	15.9	6.11	11.3	8.04	15	10.54	11.69	6.77
269800	'Zfp384'	5.74	2.56	6.54	2.63	5.4	3.93	2.04	0.48	2.46	2.11
269823	'Pon3'	0.02	0	0.03	0	0	0	0	0	0.08	0.02
269831	'Tspan12'	9.18	13.18	16	4.68	9.91	28.43	10.8	14.76	26.32	11.69
269854	'Nat14'	147.4	132.7	109	114	142	197	99.9	181	117	178.64
269855	'Ssc5d'	0.06	0	0	0	0	0	0	0	0.17	0.6
26987	'Eif4e2'	13.58	14.88	11	31.7	21.1	5.61	12.2	16.16	8.3	16.66
269870	'Zfp446'	5.25	6.46	6.16	2.86	4.98	1.38	2.14	6.08	4.2	4.56
269878	'Megf8'	3.21	5.57	12.8	0.03	10.8	6.72	8.31	2.68	5.09	6.06
269881	'Map3k10'	0.51	0.41	2.99	1.65	4.06	0.97	1.08	0.82	0.92	1.48
269902	'Vmn2r57'	0	0	0	0	0	0	0.04	0	0	0.05
26992	'Brd7'	13.34	14.38	12.5	2.09	8.64	10.41	6.48	6.53	10.16	14.59
269941	'Chsy1'	1.33	2.4	2	7.46	0.66	1.3	0.44	2.37	0.5	0
269951	'Idh2'	69.57	66.15	68.2	70.9	96.1	109.9	87.8	71.82	63.59	70.26
269952	'Gdpgp1'	0.92	2.8	1.93	0.11	5.01	7.58	2.72	0.74	1.95	0.24
269954	'Ttll13'	0.07	0.45	0.2	0	0	0	0	0	0.78	1.24
269955	'Rccd1'	2.48	1.7	2.1	1.8	3.7	0.58	2.35	4.99	4.91	1
269959	'Adamtsl3'	0	0	0	0	0.1	0	0	0.49	0	0
269966	'Nup98'	7.82	2.54	6.73	17.7	5.18	5.15	2.82	2.67	5.8	7.67
269994	'Gsg1l'	2.81	1.76	0	6.16	3.43	4.59	4.74	1.82	0	0
269997	'Zfp747'	0	1.91	0	1.61	3.59	5.26	0	2.73	7.67	9.1
269999	'Orai3'	15.19	16.45	10.6	0	6.99	1.32	5.06	10.99	5.04	5.58
270028	'Fam155a'	24.22	26.78	31.2	47.3	50.7	40.54	30	25.99	38.14	23.83
270035	'Letm2'	7.86	10.35	5.38	17.5	10.6	19.89	6.32	7.91	8.83	15.64
270049	'Galntl6'	8.11	1.76	0.05	10.1	9.59	4.44	4.16	6.13	1.47	5.18
270058	'Map1s'	19.39	26.87	25.2	6.19	19.4	25.88	39.3	36.34	20.05	49.2
270066	'Slc35e1'	0.64	1.31	0.91	0.11	2.35	0.01	1.9	0.36	0.02	1.53
270076	'Gcdh'	28.32	27.28	34.1	33.6	23.2	7.74	11.8	28.85	34.63	31.12
27008	'Micall1'	1.07	1.87	5.01	0.34	0.94	6.23	0.05	0.32	6.47	0.52
270084	'Lpcat2'	1.2	2.92	9.58	4.51	5.51	30.16	4.17	7.52	2.04	6.96
270086	'Ogfod1'	18.4	14.4	25.4	11	18.2	17.75	26.7	14.73	34.05	14.99
270096	'Mon1b'	3.4	3.6	6.49	8.7	6.75	1.89	5.65	4.71	7.55	2.39
270097	'Vat1l'	218.6	218.8	62.6	16.5	109	95.95	221	207.7	204.1	185.63
270106	'Rpl13'	435.6	510.4	591	694	426	470.7	562	371	281.4	392.96
270109	'Pcnx2'	0.08	1.38	1.67	2.88	3.44	1.31	6.47	0.52	0.09	0.11
270110	'Irf2bp2'	3.13	4.43	3.87	4.58	3.29	2.08	3.61	0.94	2.86	1.97
270118	'Maml2'	1.61	1.89	1.35	10.7	4.95	4.12	0.14	3.24	2.12	1.52
270120	'Fat3'	1.71	0.32	1.74	2.53	1.48	0.34	1.25	0.52	0	0.68
27015	'Polk'	1.74	0.93	2.48	1.66	2.26	0.45	1.42	3.35	1.73	1.82
270150	'Ccdc153'	5.33	0	0	0	6.75	5.31	0	14.51	0	6.84
270151	'Nlrx1'	2.97	0.49	0.29	3.46	0.56	0	0	0	0	0.43
270156	'Nkapd1'	2.6	3.02	1.3	8.73	0.68	0	1.95	2.38	1.48	4.4
270160	'Rab39'	1.75	0	0	0	0.28	0.32	0	0.21	0	2.33
270162	'Elmod1'	5.43	9.28	7.75	5.04	24.2	17.94	10.6	10.19	16.02	14.09
270163	'Myo9a'	6.98	5.55	4.06	7.43	2.98	2.56	8.64	4.61	3.21	2.67
270166	'Clpx'	18.84	16.1	18	0.6	17.1	18.18	17.5	21.85	25.63	20.31
270190	'Ephb1'	3.59	0.32	3.16	1.67	1.62	1.97	6.02	4.43	7.32	1.62

270192	'Rab6b'	21.73	17.38	29.9	12.5	26.7	14.75	30.3	16.55	28.21	16.58
270198	'Pfkfb4'	11.38	7.74	11.8	8.55	7.41	8.46	12.8	7	5.31	6.18
270201	'Klhl18'	5.7	5.4	5.35	6.87	10.4	5.69	14.9	6.71	6.58	8.34
270210	'Zfp651'	3.63	3.79	3.43	3.14	0.97	4.58	1.24	3.83	0.23	0.64
27028	'Ermap'	0.15	0.02	1.7	0.05	0.05	0.07	0.01	0.04	0.04	1.07
27029	'Sgsh'	6.98	13.39	13.2	0	11.3	9.5	2.47	4.44	16.42	16.5
270328	'Gsdmc3'	0.06	0	0	0.05	0.02	0	0	0	0	0
27041	'G3bp1'	74.47	93.07	73.4	46	75	63.89	84.4	77.52	75.69	80.29
27045	'Nit1'	48.49	42.22	51.3	35.6	22.3	29.41	27.7	38.63	25.92	45.04
27047	'Omd'	6.91	6.24	7.38	10.8	9.74	7.61	3.69	4.33	9.03	5.89
27049	'Etv3'	0.05	2.71	3.5	9.53	5.47	0.56	2.94	0.58	3.2	1.26
27050	'Rps3'	180.1	199	185	311	198	173.7	164	192.6	194	164.05
27053	'Asns'	117.9	161	136	104	111	185.4	126	144.6	168.3	140
27054	'Sec23b'	38.47	33.86	35.9	41.3	22.4	14.32	32.6	32.71	38.17	45.8
27055	'Fkbp9'	0	4.22	2.93	3.75	0.23	3.63	3.69	0.01	0.01	0.11
27056	'Irf5'	0.05	0	0.84	0.14	3.48	6.06	0	0.08	1.04	0.03
27057	'Ncoa4'	43.76	59.01	52.7	44.2	34.3	14.87	89.9	47.89	32.55	35.99
27058	'Srp9'	138.1	155.9	116	153	139	172.3	138	156.3	127.3	177.2
27059	'Sh3d19'	0	1.36	0	0	1.33	0	0.86	1.15	0.1	0
270599	'Gm648'	0	0	0	0	0	0	0	3.02	0	0
27060	'Tcirg1'	3.26	9.33	8.59	0	9.06	3.29	32.4	0.25	13.14	12
27061	'Bcap31'	197.4	243.6	201	165	119	276.7	151	182.8	200.3	293.35
27062	'Cadps'	11.62	10	12.1	12.3	16.6	5.62	10.4	8.46	11.35	10.97
270624	'Spin4'	0	0.22	0.07	1.17	0	0.05	0	0.07	2.98	0
270627	'Taf1'	5.03	5.4	4.81	1.8	2.58	6.09	5.57	5.16	4.54	7.09
270669	'Mbtps2'	3.64	2.12	3.85	0.98	3.71	0.61	3.2	2.72	0.01	5.78
270672	'Map3k15'	0	2.83	0.94	2.86	0	0.72	11.4	0.42	4.82	2.42
270685	'Mthfd1l'	2.85	6.38	2.01	15.9	5.69	0.68	4.91	2.05	4.79	5.05
270757	'Bpifc'	1	0	2.88	0	0	0	0	2.02	0	0.57
27078	'B9d1'	5.31	41.16	29	16.3	72.7	35.88	32.4	30.2	45.14	40.48
270802	'BC048403'	1.84	1.36	0.55	0	0.19	0.12	0.03	1.18	0.83	0.62
27081	'Zfp275'	4.76	3.59	7.25	8.6	2.72	2.62	0.53	3.59	3.07	5.62
27083	'Xlr4b'	4.81	1.58	0	0	1.02	0	0	0	8.08	2.38
270893	'Tmem132e'	1.17	1.49	6.4	3.51	3.08	0.01	3.6	1.42	1.79	1.72
270906	'Prr11'	0.43	0.69	0.48	2.59	0.61	0.61	1.28	0.56	1.1	0.61
27096	'Trappc3'	103.1	111.5	118	90.6	109	164.2	130	159.5	152.7	102.03
271005	'Klhdc1'	4.36	6.83	1.25	7.93	9.83	0.9	10.2	17.54	9.86	8.76
27103	'Eif2ak4'	2.33	2.66	0.76	2.47	4.38	2	4.35	0.69	3.7	3.14
271047	'Serpina3b'	0	0	0	0.03	0	0	0	0	0	0
271127	'Adamts16'	0	2.38	2.55	0	1.12	0	0.01	0.63	0	0
271221	'Rubcnl'	0	0	0	0	0.34	0	0	0	0	0
271278	'BC024139'	3.58	0.34	3.02	0	0	0	0	0.74	4.3	4.51
271305	'Phf21b'	0	1.46	0.04	0	0.79	0	0	0	0	0
271377	'Zbtb11'	17.64	13.5	18.7	22.4	15	10.26	21.3	7.4	13.72	19.28
271424	'Ip6k3'	0	0	2.33	0	0	0	0	0	0	0
271457	'Rab5a'	16.88	14.88	10.6	35.2	21.2	14.56	17.3	21.15	32.26	12.08
271508	'4933408B17Rik'	1.5	3.4	2.36	2.98	3	0.27	2.92	0.06	0.76	2.04
271564	'Vps13a'	3.38	4.8	3.35	1.73	2.61	2.68	2.93	2.15	1.96	3.55
271639	'Adcy10'	0	0.07	0	0	0	0	0	0	0.06	0
271697	'Cdk15'	0.03	0	0	0	0	0	0	0	0	3.21
271711	'Tmem169'	12.34	3.79	6.42	5.9	2.99	2.72	0.4	7.35	5.86	9.28
27176	'Rpl7a'	157.7	144.1	145	259	147	169.1	93.3	180.8	87.43	98.66
271786	'Galnt13'	8.69	7.72	17.7	7	2.34	2.45	4.39	1.66	8.98	3.33
271813	'Agbl2'	0	0	0.03	0	0	0	0	4.45	0	0.18
271842	'Rpusd2'	3.92	1.65	0.34	4.1	2.82	1.14	4.27	1.56	2.76	0.62
271849	'Shc4'	0.92	0	3.16	0.71	0	0	1.92	0.01	1.17	0
271944	'C2cd4d'	0	1.08	0	0	0	0	0	0	0	0
271970	'Arsj'	2.21	0	0	0	0	0	0	0	0	4

271981	'Tbck'	18.47	18.02	13.2	19.9	17.1	19.4	15.7	19.72	21.66	11.51
272009	'Srsf12'	23.22	16.59	18.3	12.7	21.8	5.69	8.3	14.35	22.33	20.55
272027	'Tstd2'	2.72	6.22	8.64	8.44	4.11	3.1	6.73	5.33	3.28	5.25
272031	'Plppr1'	13.63	8.4	6.11	1.64	9.69	24.84	11.3	10.86	4.86	4.07
27204	'Syn3'	2.66	6.65	7.24	9.9	8.49	6.56	22	7.28	6.74	6.28
27205	'Podxl'	1.14	0.71	1.1	0	0.15	0.01	3.6	0.87	0	0
27207	'Rps11'	478.5	323.2	447	423	387	443.4	478	305.7	440.3	431.48
27214	'Dbf4'	0	0.2	1.12	0	0	0	0	0	0.12	0
27215	'Azi2'	28.62	29.87	23.5	14.1	17	20.91	29.9	34.09	24.22	26.82
272158	'Poln'	0	0.03	0	0.04	0	0.02	0.14	0.08	0.03	0.01
27217	'Mixl1'	0	0	0	0.02	0	0	0	0	0	0
27218	'Slamf1'	0	1.78	0	0	0	0	0	0	0	2.95
27219	'Sgk2'	1.4	2.04	0	0	0	0	0	0	0	0
27220	'Cartpt'	341.4	616.6	449	260	153	26.1	8.57	28.84	331.5	74.49
27221	'Chaf1a'	0	2.11	0.49	2.09	2.02	0	0	1.88	0.01	3.52
27222	'Atp1a4'	0	0	0	0	0	0	0	0	0	0.22
27223	'Trp53bp1'	17.06	11.34	20.7	16.4	9.7	9.76	15.3	12.21	19.26	29.42
27224	'Eloa'	10.92	11.19	4.75	9.58	6.32	4.59	6.36	9.59	2.71	11.44
27225	'Ddx24'	158.8	158.9	143	120	139	142.9	155	138.8	121.4	139.04
27226	'Pla2g7'	42.22	46.31	23.3	74.1	17	34.47	44.1	28.37	63.11	44.58
272322	'Arntl2'	1.35	12.23	7.71	0.07	1.11	2.05	4.18	8.44	0.64	2.19
272347	'Zfp398'	3.74	5.73	5.05	1.27	2.84	6.19	2.57	8.2	5.02	7.13
272359	'Irf2bp1'	12.37	2.16	6.58	4.19	5.55	9.17	7.46	5.41	5.82	8.54
272381	'Lrrc4b'	3.81	2.48	1.09	4.35	5.22	3.64	3.26	1.97	2.82	3.27
272382	'Spib'	0.03	0	0.37	0.04	0	0	0	0.01	0	0
272396	'Tarsl2'	2.19	3.11	3.81	3.24	4.09	3.34	4.03	3.75	0	2.77
272411	'B3gnt6'	0	0	6.03	0	0	0	0	4.05	0	0
272465	'Tmem255b'	0	0	8.43	18.4	0	0	2.3	2.35	0	0
272538	'Tango6'	1.92	0.31	1.87	0	0.1	0	2.62	1.03	0.02	0
272551	'Gins2'	0	2.87	3.5	0	0	0	4.28	0	2.49	3.96
272589	'Tbcel'	3.17	0.82	2.38	0.23	1.85	2.37	1.37	1.75	0.13	1.5
27260	'Plek2'	6.26	4.86	2.98	10.7	0.03	11.3	5.16	4.93	0.73	0
27261	'Dok3'	0.29	0	4.28	18.1	5.15	0	1.81	1.5	0.08	0.67
27263	'Smok2a'	0	0	0.72	3.15	0.53	0	0	0	0	0
272636	'Esyt3'	12.48	10.69	6.79	7.64	28.4	0.2	1.46	3.56	21.83	9.25
272643	'Prss43'	0	0	0	0	0	0	0	0	0.09	0
27267	'Cars'	26.2	27.69	35.2	42.9	38.7	48.73	22	31.65	22.41	40.98
27273	'Pdk4'	0.63	1.17	0.11	0	0	2.49	1.06	0	0.65	0.41
27274	'Zfp354b'	0.54	3.15	0	0	8.4	0.6	0	1.43	2.98	3.21
27275	'Nufip1'	8.64	15.1	10.7	14	7.78	4.89	22.3	7.91	11.67	9.68
27276	'Plekhb1'	59.63	119.6	114	125	780	136.8	86.3	127.2	88.67	69.31
27277	'Golga5'	12.11	20.77	6.83	17.1	10.2	11.42	12.9	8.1	10.24	14.26
27278	'Clnk'	0	0	0	0	0	0	0	0.08	0	0
27279	'Tnfrsf12a'	3.33	9.66	20.6	0	4.29	35.83	13.7	8.52	6.17	20.35
272790	'Magee2'	41.85	36.36	29.5	6.67	5.32	9.17	33.4	16.06	20.17	24.66
27280	'Phlda3'	28.32	8.9	22.6	42.9	23.9	24.21	46	23.65	32.24	12.15
27281	'Hrasls'	28.46	14.63	24.5	34.5	18.5	37.68	45.2	34	34.48	31.55
27354	'Nbn'	4.67	5.18	13.7	5.98	10.3	1.42	6.76	9.83	19.51	8.4
27355	'Pald1'	1.9	0.8	4.3	0	0	0	0	0.33	0.65	0.02
27356	'Insl6'	4.91	5.7	0	0	6.31	1.88	7.57	0	0	0
27357	'Gyg'	29.25	30.78	35	5.09	20.7	26.03	17.7	34.98	50.46	23.13
27359	'Sytl4'	2.7	12.72	4.73	12.8	0.03	0.02	5.77	2.38	0.01	14.16
27360	'Add3'	10.44	24.86	7.67	9.27	11.2	8.35	13.5	13.71	2.71	12.06
27361	'Msrb1'	5.01	4.02	6.07	6.96	5.45	11.66	9.39	7.16	10.13	5.56
27362	'Dnajb9'	76.93	84.8	47.4	59	70.8	66.82	29.2	39.79	76.44	47.31
27364	'Srr'	18.77	19.09	9.51	20.4	23	12.22	10.7	21.3	10.39	21.4
27366	'Txnl4a'	354.6	302.5	388	427	423	415.1	241	358.6	456.8	360.83
27367	'Rpl3'	313.3	378.8	426	517	323	327.8	399	316.2	232.5	190.61

27368	'Tbl2'	7.81	6.44	9.91	0	5.19	2.39	7.92	3.42	3.5	6.78
27369	'Dguok'	50.49	21.14	33.7	62.1	34.6	28.22	32.2	47.41	34.04	39.57
27370	'Rps26'	222.7	249.2	337	436	165	270.5	270	120.5	212.9	158.93
27373	'Csnk1e'	7.44	3.99	16.2	29.9	6.85	9.52	14.4	5.97	0.77	9.96
27374	'Prmt5'	51	56.76	41.5	18.2	26.9	52.29	34.7	34.35	27.94	34.86
27375	'Tjp3'	0	0	0	0	0	0	0	0	0.84	0
27376	'Slc25a10'	2.8	9.03	9.81	3.57	12.9	4.64	9.17	10.94	9.42	4.58
27377	'Yme1l1'	30.67	34.81	22.5	30	23.8	28.74	36.3	28.12	30.75	45.08
27385	'Magel2'	11.17	16.73	29.2	6.16	6.78	10.42	21.1	23.65	13.5	13.88
27386	'Npas3'	1.44	0.87	4.28	4.52	0.03	0.68	0.54	1.02	1.03	1.35
27387	'Sh2d3c'	21.47	7.76	30.5	37.4	8.81	12.71	17.1	14.97	17.48	17.71
27388	'Ptdss2'	39.97	33.07	40.3	14.8	52.3	58.81	19.6	46.73	47.69	30.19
27389	'Dusp13'	0	2.26	0	0	0	0	0	0	0	0
27390	'Mmel1'	0	0.16	0	0.02	3.22	0	8.18	0.45	0	0
27392	'Pign'	0.01	4.12	3.26	0.35	2.67	0.44	4.02	2.35	3.57	1.01
27393	'Mrpl39'	62.35	49.15	43.7	46.6	62.5	61.31	44.6	62.9	69.21	58.24
27395	'Mrpl15'	27.38	15.27	20.7	15.5	24	26.61	23.1	27.17	24.24	25.97
27397	'Mrpl17'	13.41	12.12	8.6	8.37	16.4	16.32	20	17.18	11.07	17.01
27398	'Mrpl2'	78.27	75.47	85.7	37.6	59.7	119.2	117	72.72	38.44	119.34
27399	'Ip6k1'	20.3	27.16	26.3	24	47.6	20.14	26.6	25.33	31.79	22.86
27401	'Skp2'	0.69	1.13	1.56	0.07	0.06	0	6.49	1.63	0	0.16
27402	'Pdhx'	20.49	25.24	34.6	6.91	54.6	31.59	40.1	33.62	17.56	27.35
27403	'Abca7'	0.11	0.23	0.79	2.36	1.95	0.43	1.87	0.31	0.4	1.3
27404	'Abca8b'	0.23	1.11	4.97	0.01	2.56	3.2	0.93	0.74	3.03	1.8
27405	'Abcg3'	0	0	0	0	0	0	0	0.01	0	0
27406	'Abcf3'	64.05	63.9	42.1	67.3	56.5	56.37	83.2	77.27	96.28	85.25
27407	'Abcf2'	97.35	74.23	60.8	62	67	63.71	71	73.55	63.35	77.53
27409	'Abcg5'	0	0	0.02	0	0	0	0	0	0	0
27410	'Abca3'	5.41	2.31	12.6	1.1	5.63	6.69	5.85	7.14	3.61	8.4
27411	'Slc14a2'	0	0	0	0	0	0	0	0	0	8.34
27412	'Peg12'	0	0.04	0	0	0	0	2.3	0	0	0
27414	'Sergef'	39.59	32.43	18.8	27.5	39.2	19.65	8.79	43.49	36.71	19.97
27416	'Abcc5'	91.63	78.59	62.5	83.9	70.4	58.73	53.3	63.67	89.03	95.71
27418	'Mkln1'	8.03	13.07	9.67	6.23	5.99	4.5	10.9	15.76	15.62	24.34
27419	'Naglu'	7.69	4.59	3.51	9.32	8.23	1.48	0.34	9.94	8.73	3.02
27421	'Abcc6'	0	0	0	0	0	0	0	0	0.01	0
27425	'Atp5l'	548.3	570.7	601	846	581	913.8	574	390.4	565.7	492.87
27426	'Nagpa'	4.25	0.31	0	0	0.21	11.17	2.84	3.55	0.36	7.26
27428	'Shroom3'	0	0	0.02	0	0	0.01	0	0	0	0
27494	'Amot'	0.25	1.02	0	0.96	0	0.01	0.67	0	0	1.73
27528	'Nrep'	5.13	9.54	8.08	5.41	11.1	7.84	18.8	12.23	1.23	6.22
27632	'Nelfe'	54.65	28.08	42	77.1	36.2	31.01	47	28.74	37.19	51.24
27643	'Ubl4a'	28.52	32.74	39.6	79.6	50.6	71.55	73.1	63.66	74.66	60.6
27660	'1700088E04Rik'	11.39	4.14	10.9	9.69	14.6	11.27	8.1	7.37	8.5	8.76
276770	'Eif5a'	581.6	713	553	776	721	734.8	687	733.8	590.4	613.62
27681	'Snf8'	3.62	9.39	15.4	14.3	9.5	36.89	17.4	19.53	23.71	15.53
276829	'Smtnl2'	0.09	3	4.67	7.02	1.72	0.34	7.83	0.09	0.29	0.12
276846	'Pigs'	62.06	82.47	70.4	63.2	81.2	103.1	84.3	83.21	121.9	80.95
276852	'D11Wsu47e'	9.27	9.17	7.42	20.3	7.32	3.53	0.02	2.21	11	7.09
276905	'Armc7'	16.06	5.19	10.1	0.03	10.4	0	4.69	2.93	26.23	15.53
276919	'Gemin4'	4.04	7.61	11.8	0	3.95	4.32	4.14	3.71	25.52	9.71
276920	'Ccdc42'	0	1.81	0	0	0	0	0	1.9	0	0
276950	'Slfn8'	0.01	0	0.17	4.85	0.05	0.03	0.1	0.03	0.05	0.03
276952	'Rasl10b'	5.31	6.66	0.95	0.09	7.63	2.29	12.6	3.46	7.25	7.64
277010	'Marveld1'	0.31	0	2.43	0	0	0	0	0.01	0	1.89
277154	'Nynrin'	1.25	0.46	5.93	2.67	0.45	0.8	1.68	1.54	2.91	1.15
277250	'Kdm3b'	4.16	0.9	2.22	4.46	2.13	0.31	2.4	0.67	1.58	1.4
277328	'Trpa1'	0	0	0	0	0	0	0	0	0	0.16

277345	'Wfdc16'	0	0.09	0	0	0	0	0	0	0	0
277360	'Prex1'	1.55	0.49	3.81	11	2.31	0.01	0.85	1.04	0.92	0.01
277396	'Klhl23'	7.41	8.96	14.8	8.42	7.39	2.41	3.08	7.72	13.11	5.53
277414	'Trp53i11'	50.31	85.75	41.5	51.7	129	32.91	10.9	56.55	51.95	25.77
277432	'Vstm2l'	0.59	0.22	0.25	1.7	0.96	0.58	0.56	0.21	0.13	0.13
277463	'Gpr107'	14.46	4.65	19	0.78	4.97	1.28	9.45	14.68	18.07	8.03
277468	'Slc39a12'	10.12	16.83	4.73	4.48	0.69	19.1	7.41	9.36	2.73	4.51
27756	'Lsm2'	41.55	25.31	30.3	16.3	19.2	35.08	3.45	17.84	44.82	24.44
27762	'Vwa7'	0	0	0.46	0	0	0.35	0	0	0	0
277743	'Fam131c'	0.86	0.37	5.98	3.8	1.3	0.13	0	0.11	0.03	0.5
277773	'Fam205c'	0	0	0	6.43	1.12	0	0	0.76	0	0
27784	'Commd8'	28.38	30.96	26.1	36.8	30.5	14.97	32.5	27.2	28.7	34.94
277854	'Depdc5'	2.92	2.81	5.2	0.1	1.85	1.47	7.87	1.78	4.23	1.52
277939	'C2cd3'	1.35	1.91	3.71	0	2.95	1.45	0.01	1.35	0.48	1.56
277973	'Slc9a5'	3.1	1.32	14.4	0.02	2.25	4.93	0.46	8.21	10.78	9.19
277978	'Exoc3l'	0.05	0.18	1.01	0	0.14	7.87	0	0.17	0.61	3.61
27801	'Zdhhc8'	3.63	5.04	6.49	2.18	7.44	0.28	0.36	2.8	1.93	4.38
278097	'Armcx6'	25.72	8.73	25.7	15.8	11.7	11.68	74.8	4.75	41.1	20.44
278174	'Ssxb3'	0	0	0	0	0	0	0	0	0	0.11
278240	'Spin2c'	17.31	14.47	20.8	7	29.3	21.33	33.1	30.29	36.38	22.06
278279	'Tmtc2'	6.5	0.33	0.12	0.67	1.23	0.55	0.27	0.06	0.85	2.52
278304	'Zfp385c'	0.04	0.07	0.12	0.09	0.05	0.36	0.06	0.03	0.04	0.02
278507	'Wfikkn2'	0	0	0	0	0	0.01	0	0	0.01	0
278672	'Duxbl1'	0.62	0.89	0.2	4.38	0.05	1.8	0	0	0	1.12
278679	'Apol7b'	0.1	0.02	0	0.03	0.02	0	0.09	0	0	0.01
27878	'Tada1'	34.7	36.05	39.7	17.1	36.4	20.58	30.6	53.01	29.25	33.45
278795	'Lrrc10b'	1.27	0.37	3.27	3.6	0	0	0	0.54	3.39	4.68
27883	'Tango2'	104.4	113.6	173	126	175	273.4	250	249.1	247.8	178.71
27886	'Ess2'	10.86	22.23	23.4	23.3	29	4.88	3.99	32.14	10.91	39.21
279028	'Adamts13'	0.92	1.3	0.63	2.48	0.9	0.85	1.17	0.71	1.62	0.91
279029	'Stkld1'	0	0	0	0	0	0.84	0	1.22	0	0
279185	'Gm13083'	0	0	0	0	0	0	0	0.03	0.03	0
279499	'Kctd19'	0	0	0	0	0	0	0	0	0	0.01
279561	'Wnk3'	1.46	0.96	1.1	2.59	2.64	1.53	3.62	2.02	1.99	2.39
279572	'Tlr13'	0	0	0.01	4.26	0	0.19	0	0	0	0
279618	'Gm715'	0	0	0.45	0	0	0	0	1.19	0	0.36
27965	'Spg21'	39.4	56.69	45.4	44.7	45.9	65.23	59.8	43.29	63.01	48.63
279653	'Pcdh19'	2.02	4.09	7.18	8.88	4.44	0.87	3.14	2.52	9.39	4.48
27966	'Rrp9'	34.55	26.83	16.8	49.7	29.6	22.85	39.3	38.4	51.57	40.63
27967	'Cherp'	10.91	14.5	30.6	6.82	13.5	5.46	15.1	15.94	9.48	9.87
279706	'Nup62cl'	0	0	0	21.4	0	0	0	0	0	31.05
27973	'Vkorc1'	47.84	105.8	58.2	64.3	38.3	70.03	35.7	31.38	67.3	104.52
279766	'Rhbdd3'	32.22	35.78	27.2	22.2	21.8	52.97	41.5	27.88	23.28	26.13
27979	'Eif3b'	5.97	8.43	3.82	8.2	13.6	6.18	13.4	2.42	16.15	6.58
27981	'Rsrp1'	179	196.6	173	217	365	227.4	157	299.2	207.1	236.87
27984	'Efhd2'	19.27	19.88	8.21	31.5	16.4	35.4	32.2	14.2	73.66	20.55
27993	'Imp4'	14.32	40.35	17.5	56	26.3	41.04	30.3	16.34	30.11	16.06
27998	'Exosc5'	20.17	21.28	28.3	4.24	47.3	68.7	21.7	24.57	22.68	20.8
27999	'Fam3c'	16.4	21.14	18.3	19.8	18	9.42	11.9	7.17	23.29	6.77
28000	'Prpf19'	6.47	8.14	11.2	9.54	7.36	10.7	12	10.23	13.41	10.12
28006	'Washc2'	7.2	15.28	23.8	20.7	6.84	3.11	17.4	11.41	20.74	13.76
28010	'Miip'	8.21	3.47	14.1	0	11.6	8.55	0.19	1.58	17.14	8.01
28015	'Polr2m'	126.9	105.4	144	158	89.6	86.09	198	63	166.3	55.41
28018	'Ubfd1'	8.8	3	11	14.7	6.12	5.63	12.3	9.42	11.58	3.69
28019	'Ing4'	33.97	26.21	24.8	0.04	36.3	42.04	35.6	30.85	29.89	19.01
28028	'Mrpl50'	29.61	19.65	29.3	19.7	30.6	17.14	13.8	25.78	34.15	25.89
280287	'Kiss1'	26.22	2.68	3.34	2484	0.22	0.31	0.22	0.88	0.09	1.36
28030	'Gfm1'	17.85	5.15	9.4	1.7	5.05	16.83	4.46	11.33	15.59	10.65

28035	'Usp39'	10.85	22.57	25.8	7.1	11.8	19.39	5.92	10.26	12.72	3.68
28036	'Larp7'	8.62	13.31	16.4	29.2	9.96	13.25	7.13	11.71	13.3	11.55
28040	'D6Wsu163e'	9.15	13.35	26.3	17.4	4.94	3.34	14.6	12.17	14.01	10.47
280408	'Rilp'	1.92	2.16	0	0	3.45	0.5	0	3.36	7.92	0.06
280411	'Lix1l'	0.56	0	0	0	1.87	0	0	0.71	0	0
28042	'Selenoi'	5.37	8.18	5.12	4.52	7.17	2.95	4.46	4.59	7.31	3.8
280621	'Selenov'	1.9	0.04	5.51	0	0.04	7.29	1.18	0.85	0	1.01
28064	'Yipf3'	54.26	40.05	67.9	12.1	36.4	45.87	36.3	52.4	58.8	54.17
280645	'B3gat2'	2.03	3.97	1.44	5.28	0.77	2.32	2.16	3.54	2.02	2.07
280667	'Adam1b'	0	0	0	0.02	0	0	0	0	0	0
280668	'Adam1a'	2.77	1.17	1.97	0	5.36	0	2.56	0.71	2.48	0.04
28071	'Twistnb'	21.95	29.14	9.96	20	11.8	10.41	17.5	15.93	22.76	11.86
28075	'Desi1'	7.46	20.29	5.09	5.45	18.3	3.37	10.7	6.96	0.42	2.24
28077	'Med10'	47.91	63.18	51.5	87.3	64.5	78.12	79.7	75	64.82	101.8
28080	'Atp5o'	514.9	425	717	444	607	789.3	744	640.6	510.6	546.26
28081	'Fam104a'	21.73	25	19.8	26	22.2	11.05	13.6	22.71	17.69	8.46
28084	'Vps25'	65.85	118.2	59.5	84.4	112	81.81	165	106.3	106	98.42
28088	'Rtcb'	118.9	134.6	166	136	120	182.1	133	134.7	155.4	183.9
28105	'Trim36'	0.9	1.81	0.35	0.3	1.87	0.16	0.2	1.99	2.11	0.22
28106	'Mydgf'	151.7	121.4	103	112	123	183.6	132	145.8	172.2	159.68
28109	'D10Wsu102e'	10.14	11.06	8.54	14.3	5.02	2.18	16.1	8.37	16.2	14.32
28113	'Tinf2'	3.82	3.08	9.75	13.4	3.01	16.44	0.64	10.47	10.51	10.7
28114	'Nsun2'	9.1	10.36	8.24	29.5	6.82	2.42	21.8	14.2	13.52	3.98
28126	'Nop16'	63.67	45.18	34.5	62.7	13.8	63.82	40.3	48.28	61.72	44.33
28135	'Cep63'	14.53	11.21	21.9	17.6	28	18.78	8.76	12.71	8.76	11.72
28146	'Serp1'	16.2	17.64	10.1	41.7	27.3	31.4	23.5	19.12	18.85	13.92
28169	'Agpat3'	6.75	5.92	11.6	14	8.44	10.6	0.87	7.45	5.84	2.42
28185	'Tomm70a'	12.6	12.53	16.9	34.3	12	11.03	22	11.59	15.54	2.2
28193	'Reep3'	1.05	1.77	5.21	2.64	1.59	2.04	4.42	4.27	1.8	0.94
28199	'Dcaf11'	72.03	56.91	42.1	67.1	59.6	56.65	62.6	57.57	74.97	56.25
28200	'Dhrs4'	16.08	4.41	9.92	0.13	10.4	35.98	0.07	3.28	0	6.54
28240	'Trpm2'	8.07	10.95	9.81	10.3	5.9	3.03	15.1	7.04	4.39	3.48
28250	'Slco1a4'	0	0	0	0	0.06	0	0	0	0	0.01
282619	'Sbsn'	6.02	6.46	6.91	13.9	5.85	9.78	7.12	9.52	1.82	4.99
282663	'Serpinb1b'	8.99	8.31	4.65	0	15.7	56.5	47.6	5.48	19.68	6
28295	'D10Jhu81e'	90.43	100.3	124	28.2	89.9	97.67	112	117.6	124.4	105.88
286940	'Flnb'	0.68	1.52	6.44	5.14	2.4	0.17	1.91	2.91	0.84	1.6
286942	'Kif19a'	0.1	0	1.19	0.21	0	0	0	0	0.26	0.7
29805	'Znhit2'	44.06	42.53	38.8	42.2	34.4	41.89	62.1	53.54	37.24	23.68
29806	'Limd1'	1.43	3.05	2.13	4.7	2.03	0.01	3.08	1.75	4.8	1.83
29807	'Tpk1'	0.02	0.02	0.25	0	0.47	0	0	1.62	0	3.43
29808	'Mga'	2.69	2.38	3.59	6.02	3.74	4.28	6.69	5.12	3.19	10.44
29809	'Rabgap1l'	2.85	9.56	5.66	9.53	8.48	4.87	6.57	3.96	4.29	4.65
29810	'Bag3'	9.51	2.21	0.74	19	0.06	0.02	2.51	0.02	0.02	2.82
29811	'Ndrg2'	432.7	234.5	265	351	132	622.8	486	232.2	217.2	311.57
29812	'Ndrg3'	187.4	146.5	204	156	135	154.8	336	200.1	200.5	162.56
29813	'Zfp385a'	2.9	1.8	5.72	2.46	3.8	9.59	2.71	5.15	3.82	1.63
29815	'Bcar3'	4.75	12.65	3.94	6.69	3.72	29.04	11.3	5.96	15.02	13.4
29816	'Hip1r'	2.67	6.77	19.3	5.5	5.24	1.79	10.2	5.18	8.44	5.82
29817	'Igfbp7'	0.69	0	0	0	0.09	0	0	0	0	0
29818	'Hspb7'	0.06	0.02	0.1	0.08	0.09	0.03	0.02	0.01	0.03	0.05
29819	'Stau2'	31.59	37.34	35	42.3	21.8	27.16	33.7	27.35	41.43	35
29820	'Tnfrsf19'	4.47	6.53	3.69	2.15	2.4	0.44	0.01	7.92	1	7.8
29856	'Smtn'	0.14	0.31	0	0	0	0.01	0.3	0.61	0	0.09
29857	'Mapk12'	5.24	0	7.31	0	5.68	0	0.36	1.62	3.3	0
29858	'Pmm1'	218.2	213.1	184	119	206	228.2	214	199.5	252.3	220.71
29859	'Sult4a1'	133.6	118.5	173	88.4	127	66.68	137	123.2	111.1	57.97
29861	'Dpf1'	15.21	18.56	16.2	12.9	10.9	15.58	13.5	16.17	9.76	15.94

29862	'Ninj2'	0	0	3.69	0	5.79	9.07	13.4	3.24	0	0
29863	'Pde7b'	0.78	1.36	2.69	0.05	0.34	0.26	4.16	1.63	3.53	1.51
29864	'Rnf11'	15.07	11.69	11.4	31.2	14.3	16.35	15.2	9.11	7.07	4.91
29867	'Cabp1'	0	3.13	5.28	2.49	0.61	5.56	0	1.75	3.57	4.91
29869	'Ulk2'	0.16	1.4	0.95	5.18	1.99	3.01	2.56	1.11	2.8	2.89
29870	'Gtse1'	0	0	0	8.11	2.14	0	0	0	0	0
29871	'Scmh1'	1.65	9.34	5.7	1.79	12.7	1.23	13	7.54	10.73	11.12
29873	'Cspg5'	16.68	21.4	29.3	17.6	12.2	14.43	43.8	12.17	15.01	5.37
29875	'Iqgap1'	0.92	2.34	7.98	7.86	2.4	1.28	1.02	1.27	2.79	9.71
29876	'Clic4'	11.96	12.12	8.19	1.63	5.03	9.96	17.3	12.64	0.06	5.28
29877	'Hdgfl3'	9.74	6.76	10.9	5.5	8.51	15.56	13.7	6.35	14.22	8.45
30044	'Opn4'	0	0	0	0	1.61	0.02	0	0.37	0	0
30045	'Dnajc12'	39.03	66.07	66.5	118	57.3	107.7	99.6	60.71	105.2	54.96
30046	'Zfp292'	2.92	1	3.24	5.9	3.69	1.76	2.16	1.75	3.6	1.13
30049	'Scd3'	0.06	0.11	1.14	2.93	0.08	3.07	0.09	1.81	1.54	1.21
30050	'Fbxw2'	47.5	57.75	77.8	37.5	41.9	77.86	87.4	51.3	51.05	16.74
30051	'Spdef'	4.9	0.08	5.51	0.18	0.02	4.29	0.02	0.97	0	24.19
30052	'Pcsk1n'	261	276.3	485	494	221	249.4	296	164.3	182.8	145.7
30054	'Rnf17'	0.02	0	0	0	0	0	0	0	1.9	0
30055	'Timm13'	160.4	133.5	147	141	172	222.4	141	135.5	138.6	155.54
30056	'Timm9'	89.68	67.44	96.7	108	56.3	77.08	131	90.72	69.94	79.97
30057	'Timm8b'	439.8	434.5	418	578	401	600.2	346	428.5	419.6	416.14
30058	'Timm8a1'	26.05	13.55	20.7	31.2	13.8	6.46	5.18	28	9.51	10.48
30059	'Timm10'	117.1	89.13	109	33.6	66.7	50.13	108	118	73.39	67.24
30060	'Meltf'	0	0.13	0.06	2.28	0.02	0.13	0	0.08	0	0.07
30785	'Cttnbp2'	5.55	7.49	2.56	3.35	4.44	2.34	6.71	3.75	3.17	4.27
30791	'Slc39a1'	11.1	14.18	28.6	31.4	26.9	8.37	12.8	8.01	36.53	26.53
30794	'Pdlim4'	7.3	8.64	3.08	0	10.4	25.45	0.06	2.08	0	2.99
30795	'Fkbp3'	382.1	358.1	406	414	262	520	426	482.9	460.8	322.06
30805	'Slc22a4'	5.52	3.8	11.3	7.57	8.06	2.49	6.77	12.03	2.53	7.41
30806	'Adamts8'	0	0	0	0	0	0	0	0	0	0.94
30838	'Fbxw4'	2.46	5.02	1	3.99	1.55	0.05	5.78	1.53	4	1.12
30839	'Fbxw5'	71.31	102	49.3	78.5	81.2	99.3	128	113	70.94	90.86
30840	'Fbxl6'	24.07	15.82	16.9	7.52	39.4	16.81	14.3	7.19	18.71	28.7
30841	'Kdm2b'	2.83	3.72	5.67	4.22	1.73	0.57	11.8	1.88	4.18	2.87
30843	'Fbxl12'	8.26	5.25	3.38	7.38	7.73	2.04	10.4	7.33	1.01	4.67
30853	'Mlf2'	228.2	230	202	309	263	254	225	220.7	244.3	112.15
30877	'Gnl3'	11.84	25.99	20.5	15.1	6.26	15.17	17.1	17.37	24.43	20.7
30878	'Apln'	1.12	2.9	2.78	0	3.41	0	0	0	0	0
30924	'Angptl3'	0	0	0	0	0	0	0	0	0	1.47
30925	'Slamf6'	0	0.01	0.02	0	0	6.28	0	0	0	0
30926	'Glrx3'	101.4	107.7	97.3	113	99.5	91.85	81.7	116.1	118.1	95.4
30927	'Snai3'	0	0	0	0	0	0	0	0	0.47	0
30928	'Zbtb18'	0.42	0.81	0.16	0.56	0.19	0.66	0.04	0.91	1.18	0.5
30930	'Vps26a'	19.7	23.02	12.4	19.3	25.1	18.59	38.2	15.85	23.39	20.61
30931	'Tor1a'	49.53	58.28	62.2	59.8	38.9	68.86	37.3	61.04	43.16	59.53
30932	'Zfp330'	23.16	25.53	25.7	40.5	44.1	37.91	24.9	31.39	41.75	35.32
30933	'Tor2a'	29.13	21.99	19.4	5.07	27.6	5.81	21	31.38	24.58	32.17
30934	'Tor1b'	11.11	27.83	3.56	10.8	17.1	7.23	16.4	15.36	3.84	17.39
30935	'Tor3a'	2.1	3.47	1.54	5.01	0.73	1.42	0.53	1.24	6.04	6.28
30936	'Slc46a2'	0	0	0	0	0	0	0	0	0.46	2.24
30937	'Lmcd1'	0	2.46	20.1	6.11	0	0	7.52	0	0	2.61
30938	'Fgd3'	3.23	3.03	0	1.09	3.04	2.68	0	0	0	1.08
30939	'Pttg1'	23.76	45.89	82.8	128	125	33.42	44.2	86.4	36.47	38.61
30940	'Usp25'	0.19	0	2	1.94	0.68	0	0	0.04	0.09	0.02
30941	'Usp21'	54.05	31.8	25.3	5.05	38.1	16.96	18.4	23.96	15.34	12.79
30943	'Prss30'	0.43	2.77	3.24	0	0	0	0	2.13	0	0
30944	'Zfp354c'	3.6	1.03	4.62	0.11	1.68	1.18	4.04	4.83	7.99	3.52

30945	'Rnf19a'	3.63	3.32	8.57	8	4.34	1.33	4.46	7.46	5.54	4.75
30946	'Abt1'	3.99	4.15	8.66	18.3	8.31	6.19	3.88	8.68	5.05	9.06
30947	'Adat1'	4.05	4.43	0.23	0.29	3.26	0.31	0.08	2.95	5.91	4.88
30948	'Bin1'	81.82	73.2	71.1	57.8	73.9	70.7	108	40.77	60.33	38.99
30949	'Lcmt1'	84.91	109.1	98.9	94.2	81.7	107.6	105	88.81	90.69	71.14
30951	'Cbx8'	20.57	14.02	3.3	19.2	29.7	0	4.56	7.32	8.78	20.62
30953	'Schip1'	65.68	67.33	59.3	28.6	107	84.49	68.7	67.7	121.3	75.95
30954	'Siva1'	31.07	22.37	30.2	20.7	20.1	57.63	19.2	34.39	36.52	53.95
30955	'Pik3cg'	0.91	0	0.19	0	0	0	0	0	0	0
30956	'Aass'	1.08	0	0	0	0	2.62	0.01	0	0	0
30957	'Mapk8ip3'	42.14	52.55	80.5	39.9	45.1	37.42	65.1	44.87	66.23	54.5
30959	'Ddx25'	75.27	60.89	112	94.4	80.7	104.7	127	139.5	81.56	104.63
30960	'Vapa'	90.51	86.33	113	202	88.5	75.62	105	66.74	85.73	65.76
30963	'Hacd1'	13.24	2.32	0.11	0	33.1	0.05	10.1	14.62	16.7	2.73
317652	'Klk15'	0	0	0	0	0	0	0	0	0.06	0
317653	'Klk14'	0	0.06	0.08	0.2	0.02	0.03	0.04	0	0.14	0.03
317717	'Sec22a'	19.02	19.3	26.6	36.7	18.4	31.2	36.3	35.47	26.77	27.67
317750	'Slc24a5'	0	0.49	0	20.3	2.33	7.72	0	1.02	0	6.47
317755	'Zar1'	2.83	0.78	0	0	0.63	0	0	0	2.62	0.55
317757	'Gimap5'	0	0	0	0	0	0	0	0	0	3.52
319146	'Ifnz'	0	0	0	0	0	0	0	0	0.31	0
319149	'Hist1h3d'	0	0.42	5.9	0	0	5.74	0	0	0	0
319150	'Hist1h3b'	0	0	0	0	0.12	0	3.02	0	0	0
319151	'Hist1h3e'	0	4.22	6.37	9.09	0.12	25.27	0.75	3.28	6.23	0
319152	'Hist1h3h'	0.93	0	0.13	0	0	0	0	0	0	0
319153	'Hist1h3i'	0	0	0.16	0.4	0	2.35	0	0.19	0.23	0.21
319154	'Hist2h3b'	0	3.08	0.29	0	3.22	0	0	0.58	3.1	2.67
319155	'Hist1h4c'	4.47	7.28	4.29	32.8	2.89	7.33	16.8	1.32	7.67	7.46
319156	'Hist1h4d'	9.44	8.55	9.63	31.2	1.09	24.36	6.37	2.07	7.83	4.42
319157	'Hist1h4f'	0	0	1.34	0	0	0	0	0	0	0
319158	'Hist1h4i'	3.02	2.35	12.5	10.2	0	6	21	6.15	15.81	7.27
319160	'Hist1h4k'	0	0.79	0	0	0	0	0	0	0	0
319162	'Hist3h2a'	43.54	44.83	11.6	37.3	53.8	56.28	42.6	40.95	52.46	48.77
319164	'Hist1h2ac'	0	0.96	0	0	0	0	0.16	0	0	0.16
319165	'Hist1h2ad'	0.23	0.21	0	0	0	0.35	3.69	0	0.39	0
319166	'Hist1h2ae'	11.56	5.03	4.46	9.86	6.2	8.91	17.2	6.91	3.19	5.38
319167	'Hist1h2ag'	0	3.16	0	0	0	3.54	0	0	3.58	0.75
319169	'Hist1h2ak'	0	0	0.41	0	0	0	1.23	0	0	2.06
319171	'Hist1h2ap'	3.2	5.33	9.1	32.1	2.09	18.9	12.2	2.03	9.32	4
319172	'Hist1h2ab'	0	3.23	0	0	0	0	3.5	0.59	0	0
319173	'Hist1h2af'	0	0	0	0.09	0	0.08	0	0	0	0.13
319176	'Hist2h2ac'	0	2.08	0	0	2.01	0	0	0	0	1.98
319178	'Hist1h2bb'	2.06	0	0	0	0	0	0	0	0	0
319179	'Hist1h2be'	3.41	1	0.02	6.27	0	7.47	2.61	1.93	0.11	2.29
319181	'Hist1h2bg'	0	7.54	0.49	0	4.23	1.8	9.14	1.2	0	4.77
319183	'Hist1h2bj'	0	0	0	0	0	0	4.9	0	0	0.58
319184	'Hist1h2bk'	0	0	0	0	0	0	0.46	0	0	0
319186	'Hist1h2bm'	0	0	0	0	0	4.09	0	0	0	0
319187	'Hist1h2bn'	0	1.17	0	0	0	0	2.62	0	0	0
319188	'Hist1h2bp'	0	0	0	0	0	4.7	0	0	0	0
319189	'Hist2h2bb'	0	0.33	1.96	0	3.99	0.95	5.76	1.83	1.97	0
319190	'Hist2h2be'	0	0	0	0	1.74	0	1.53	0	3.67	0
319191	'Hist1h2ai'	1.05	0	2.4	7.96	0	0	0	1.12	0	6
319192	'Hist2h2aa2'	0	0	5.83	0	4.76	0	0	0	0	6
319195	'Rpl17'	167.9	159.5	221	237	106	193	147	139.7	151.2	171.58
319196	'Ankef1'	0.16	4.71	4.31	0	2.48	0	1.35	0	5.95	1.74
319197	'Gpr4'	0	0	0.76	0	0	0	0	0	1.24	1.56
319200	'Gpr82'	0	0.64	0.92	0.01	0	0.02	0	0	0	1.12

319207	'Pgbd1'	1.23	5.29	0.02	0	0.59	0	0	2.23	0.59	1.3
319211	'Nol4'	23.98	20.24	25	30	25.9	12.91	18.8	26.22	20.16	24
319229	'Sctr'	0	0	0	0	0.29	0	0	0	0	0
319236	'Trim12c'	0	0	1	0	0.04	0.06	0	0	0.01	0.02
319239	'Npsr1'	0.91	0	0.08	0	0.03	5.54	11.1	0	0	0
319259	'Bricd5'	0	0	0	0	0	0	13.8	0	0	0
319262	'Fchsd1'	5.3	5.8	5.39	6.93	2.06	0.03	2.89	1.37	6.78	5.77
319263	'Pcmtd1'	7.53	14.57	19.3	11.2	19.6	4.36	11.1	12.52	16.84	10.42
319266	'A130010J15Rik'	0.11	5.78	7.68	5.42	7.58	2.44	0.92	1.44	5.64	3.32
319277	'Washc4'	19.66	21.7	22	63.2	19.6	16.54	16.4	11.42	22.01	12.58
319278	'A230050P20Rik'	100.4	118	153	120	105	75.96	103	125.7	158.7	48.48
319322	'Sf3b2'	123.7	112	133	119	135	139.2	101	121	179.9	160.74
319352	'Pianp'	76.65	76.01	148	65.8	93.6	50.15	93.3	95.1	52.21	42.33
319370	'Ubald2'	4.04	1.68	4.01	1.44	2.66	0.38	1.95	2.8	6.61	2.74
319387	'Adgrl3'	1.06	3.11	3.56	4.48	3.25	5.09	1.42	4.38	2.29	2.37
319415	'Hs3st5'	22.43	10.27	0.18	19.8	1.2	0.79	7.72	4.53	0	1.07
319430	'C5ar2'	0.03	0.03	0	0	0.03	0.03	0.01	0.03	0.03	0.12
319433	'Serpine3'	0	0	0	0	0	0	0.04	0	3.64	0
319446	'Dpep2'	0.35	0	0	0	0.25	0	0	0.27	0	0.28
319448	'Fndc3a'	10.17	11.34	11.6	21.3	13.8	4.98	10.7	4.52	22.61	14.71
319455	'Pld5'	2.8	6	8.2	5.54	23.7	2.58	0.05	7.15	3.91	10.36
319468	'Ppm1h'	33.39	13.59	27.6	16.4	31.8	35.9	9.23	33.68	34.45	17.82
319475	'Zfp672'	15.66	4.54	9.38	3.92	10.2	5.42	13.8	11.79	31.25	11.64
319476	'Lrtm1'	0.01	0.01	0.01	0	0.01	0.01	0.01	0	0.02	0.01
319477	'Insyn1'	3.62	4.04	4.11	3.03	12.7	19.06	3.32	8.76	15.84	17.4
319478	'Cxxc4'	4.38	5.82	4.94	8.08	3.88	1.45	5.39	4.76	4.48	5.6
319480	'Itga11'	0	0.12	0	0.09	0.01	0	0	0	0	1.73
319481	'Wdr59'	0.31	0.7	2.61	4.05	4.4	0.02	3.75	2.27	4.71	2.72
319482	'9530053A07Rik'	0	0.01	0	0	0	0	0	0	0	0
319493	'A430078G23Rik'	0.84	0.78	0.57	1.94	1.02	0.91	1.24	0.75	0.96	1.56
319504	'Nrcam'	16.22	7.24	9.73	10.8	16.4	4.28	25.2	16.52	22.23	23.4
319508	'Syt15'	0.07	0.04	0.24	0.25	0.08	0.07	0.04	0.03	0.04	0.04
319513	'Pced1a'	5.95	6.03	2.42	4.98	10	5	3.1	2.44	11.66	11.63
319518	'Pdpr'	1.65	4.27	2.83	0.64	3.43	1.99	9.87	5.13	0.23	0.24
319520	'Dusp4'	0.6	2.27	1.97	0	2.12	0.49	0.02	1.05	2.5	2.01
319530	'Zfp750'	0	0	1.61	0	0	0	0	0.88	0.07	0
319535	'Zfp182'	1.13	1.39	1.64	0.91	1.9	2.62	1.71	0.83	0.93	1.11
319552	'Spx'	0	2.11	0.04	0	0.03	0	0	0.02	4.49	0.08
319554	'Idi1'	22.88	10.88	16.9	12.8	7.98	4.99	11.2	20.59	14.35	11.51
319555	'Nwd1'	0.01	1.02	1.98	1.85	1.44	0.33	0.03	1.3	0.33	0.45
319565	'Syne2'	1.53	1.35	0.41	1.54	0.75	0.72	1.19	0.78	0.85	0.6
319582	'Trmt9b'	0	0	2.28	13.2	2.02	2.57	6.77	3.42	2.61	3.24
319583	'Lig4'	3.01	1.16	3.59	0	4.4	0.3	0	1.68	4.64	1.64
319586	'Celf5'	7.19	7.18	7.07	6.89	8.81	6.58	5.59	7.09	10.7	8.31
319594	'Hif1an'	0.15	2.05	1.96	3.33	2.91	3.17	1.59	3.18	7.18	0.29
319601	'Zfp653'	4.84	3.77	0.98	1.95	2.57	0.03	3.79	12.65	2.28	2.72
319604	'Fam168a'	10.39	9.59	13.9	15.9	22.9	9.9	30.8	10.71	5.57	14
319613	'Sybu'	69.75	78.86	48.2	43.4	42.4	56.15	57.4	53.38	53.82	30.37
319615	'Zfp944'	9.36	2.11	12.9	6.34	9.95	3.37	7.65	2.88	8.14	9.56
319618	'Dcp1b'	7.09	5.27	2.6	14.6	5.34	0.8	6.25	8.25	3.5	6.68
319622	'Itpripl2'	0	0	0	0	0	0.01	0	0.3	0	0
319625	'Galm'	0	0	0.3	0.06	0	0	0.02	0	0	0
319634	'Efcab5'	0	0.11	0	1.52	0.57	0.01	0	0	0.37	0.61
319636	'Fsd1l'	7.31	5.14	3.44	2.26	3.74	7.83	8.58	5.24	6.83	6.99
319638	'Nt5dc1'	0.82	2.05	2.67	0	4.13	3.6	10.2	0.02	3.71	3.16
319642	'Rab9b'	35.98	52.22	32.9	31.8	30.1	11.19	62.1	33.3	31.77	56.83
319651	'Usp37'	11.59	7.68	6.71	17.6	11.8	5.63	3.34	8.97	8.49	5.33
319653	'Slc25a40'	7.71	12.64	2.5	0	6.92	0.63	9.92	1.83	6.51	8.98

319655	'Podxl2'	149.3	137.4	343	96.3	90.4	69	214	52.62	245.8	32.14
319660	'Agmo'	3.08	0	0	0.04	0	0	0	0	0	2.77
319670	'Eml5'	2.59	3.01	2.92	11.5	4.9	4.2	7.39	3.57	7.28	4.97
319675	'Cep295'	0.72	1.01	0.72	2.6	3.46	1.55	1.23	1.58	2.43	1.68
319695	'Ankar'	0	0.13	0.14	0	0	0	0	0	0.01	0
319710	'Frmd6'	1.76	5.82	0	0	0.54	0.58	0.01	0.51	1.61	5.26
319713	'Ablim3'	9.25	7.63	5.19	10.6	6.12	0.5	3.72	6.55	10.7	16.05
319719	'Simc1'	9.74	8.79	16.2	8.78	0.96	1.46	12.1	3.09	6.32	6.36
319734	'Cacna2d4'	0.01	0	0	1.5	0.82	1.18	1.47	0.89	0	0
319740	'Zfyve27'	17.85	14.4	15.3	13.8	12.4	12.09	15.2	10.35	21.22	14.71
319742	'Mpzl3'	0	0	0	0	1.67	0	5.9	0	0.77	2.39
319748	'Zfp865'	0.07	1.45	0.49	0	0.17	0	0.6	0.71	0	0.46
319757	'Smo'	1.69	3.45	0	0	2.72	2.82	0.02	0	8.94	0
319758	'Rfx7'	1.42	0.87	1.51	6.99	0.88	1.46	0.75	0.32	3.25	0.84
319765	'Igf2bp2'	0.32	0	0	1.25	0.01	0.18	0.66	0.55	0	1.4
319767	'Atp10b'	0.18	0.1	0	0	0.02	0.07	0	0.01	0.31	0.45
319772	'C130050O18Rik'	0	0	0	0	0	8.19	0	1.43	0	0.19
319776	'Tmem72'	0	0	0	0	3.09	0	0	0	0	0
319801	'Tigar'	1.22	1.57	2.56	5.79	2.7	2.07	0	3.33	3.19	0.44
319804	'Glt1d1'	3.23	2.38	1.21	7.54	1.88	0.74	8.9	1.75	6.21	0.64
319807	'Nwd2'	0.42	0	1.21	0	1.52	0.16	2.31	0.09	0.49	4.73
319817	'Rc3h2'	1.94	1.96	1.99	1.83	2.54	1.93	1.12	1.33	0.94	0.88
319822	'Smyd4'	17.48	5.15	6.1	0.96	16	4.61	4.75	6.56	1.85	13.29
319832	'Tmem229a'	1.34	2.74	0.27	3.88	0.76	0.65	0.03	0.99	0.14	0.13
319845	'Bbs9'	11.32	14.36	15.8	10.9	4.67	10.36	5.17	9	6.69	10.07
319876	'Cobll1'	0.03	0	1.45	1.66	0.23	0	0.32	0	0.62	0.63
319880	'Tmcc3'	16.56	10.96	5.8	21.1	7.07	3.5	6.83	3.49	1.42	2.11
319885	'Zcchc7'	23.47	21.45	42.6	39.5	19.1	22.01	20.1	12.11	32.62	19.05
319887	'E030030I06Rik'	2.59	3.11	4.33	3.77	4.1	4.82	3.35	3.49	2.72	2.06
319888	'Oacyl'	0	0	0	0	0.9	0	0	0	0.05	0
319899	'Dock6'	1.78	0.02	7.38	1.4	1.9	0.84	0	2.37	3.51	2.42
319901	'Dsel'	4.09	0.77	6.73	2.99	1.89	1.93	9.61	1.34	4.19	1.95
319909	'Ism1'	0.01	0	1.03	6.53	2.99	0	0	0.72	0	2.08
319922	'Vwc2'	0.01	0.02	1.11	0	1.05	5.75	5.28	4.43	3.37	0
319924	'Apba1'	2.55	2.09	1.47	2.43	0.65	0.42	0.57	1.59	2.93	3.8
319934	'Sbf2'	0.73	1.05	0.86	1.94	1.97	1.21	1.04	2.72	5.13	1.2
319939	'Tns3'	1.32	0.58	0.7	0	0.7	0.18	0	0.19	0.62	0.21
319942	'A530016L24Rik'	0	0	0.9	0	0.02	0	0.99	0	2.25	0.02
319944	'Taf2'	1.26	0.63	3.15	5.31	2.35	0.02	3.05	2.75	4.51	1.89
319945	'Flad1'	25.61	25.66	57.5	21.1	25.7	30.81	29.7	46.46	36.92	38.77
319953	'Ttll1'	81.34	85.59	67.3	113	85.4	114.5	73.3	90.69	107.2	89.62
319955	'Ercc6'	3.82	2.87	4.03	2.22	2.63	2.78	11.6	4.42	10.26	4.24
319965	'Cc2d1b'	5.03	9.59	10.4	2.23	14.5	8	5.14	13.56	19.37	18.77
319974	'Auts2'	4.35	2.25	2.66	0.17	2.57	5.54	1.26	2.56	6	1.99
319984	'Jph4'	7.95	8.45	4.86	12.8	18.2	7.08	5.69	7.62	9.87	9.23
319991	'Kif6'	0.2	0.92	0.2	0.99	2.99	0	0	1.73	0	0.21
319996	'Casc4'	10.51	15.93	19.6	50.3	17.1	19.06	17.1	11.51	15.71	16.78
319998	'Tmem198'	24.74	23.54	8.44	16.1	19.1	15.21	8.12	27.84	11.89	9.66
320007	'Sidt1'	14.11	4.58	4.54	8.85	15.9	4.78	10.1	6.28	16.07	17.66
320011	'Uggt1'	6.38	6.94	9.4	2.84	1.6	6.51	11	7.66	8.45	6.24
320022	'Terb1'	0	1.47	0	0	0	0.04	0	0	2.19	0
320024	'Nceh1'	24.76	29.83	34	12	28.5	31.3	40.9	27.63	40.75	19.89
320027	'Fstl4'	14.19	6.66	5.31	4.35	0.03	7.26	9.65	7.08	14.44	3.09
320051	'Exph5'	0.21	0	0.12	0	0.29	0	0	0	0	0
320078	'Olfml2b'	0	1.95	0.02	0	0	0	0.34	2.39	0	0
320080	'Zbtb39'	0	0	0.02	0.02	0	0	0	0	0.01	0.01
320082	'Fbxw21'	0	0	0	0	0.02	0.02	0.02	0	0	0
320091	'Ano4'	1.04	1.24	5.16	0	4.94	0	1.25	2.36	0.54	3.69

320095	'6430550D23Rik'	0	1.43	3.76	0	0	0	0	0	0	0
320100	'Relt'	35.85	39.61	19.5	61.8	23.5	26.55	11.2	11.33	48.41	19.07
320106	'Slc38a11'	0	0	0	0	0	1.24	0	0	0	0.08
320111	'Prr18'	2.88	3.48	0	0	8.04	1.81	2.69	0.23	0	3.33
320116	'Fndc9'	62.13	75.66	74.2	201	37.3	23.9	22.3	72.82	48.81	52.83
320118	'Fbxl13'	0	0	0.09	5.79	0	0	0	0	0	0
320119	'Rps6kc1'	6.69	8.04	19	24.5	22	3.3	6.13	12.34	15.25	5.65
320127	'Dgki'	1.33	1.25	1.09	0.55	1.38	0.6	0.1	0.99	0.7	0.35
320129	'Grk3'	3.07	4.37	6.93	0.98	5.92	0.15	1.91	2.47	4.51	4.37
320135	'BC049715'	0.43	3.98	3.69	0.17	0.3	0	0.97	0	0.76	0.04
320139	'Ptpn7'	0.15	0.09	0.05	0.17	0.09	0.26	0.17	0.02	0.2	0.13
320145	'Sp8'	0	0.47	0	0	0.87	0	0	0	0	0
320148	'B430306N03Rik'	0	0	0	0	0	0.01	0	0	0	0
320150	'Zdhhc17'	27.64	20.83	25	10.8	15.8	5.12	13.2	20.82	27.82	16.74
320158	'Zmat4'	5.83	12.49	6.1	13.4	9.2	1.8	15.6	17.33	5.49	0.62
320159	'Togaram2'	1.69	1.04	0.01	1.69	0.69	0	0	9.6	2.25	1.57
320162	'Cep95'	0.89	2.22	4.07	0.91	2.56	0.18	3.12	0.15	4.58	5.48
320165	'Tacc1'	12.05	21.43	13.8	8.42	13.7	10.39	11	19.73	19.11	18.17
320183	'Msrb3'	0.02	0	0.04	0	0	0	0	0.01	0.02	0
320184	'Lrrc58'	6.9	10.09	12.1	10.4	7.46	2.61	8.84	3.52	7.42	7.72
320191	'Hook3'	8.99	10.89	7.53	3.45	3.75	14.9	17.6	8.33	13.66	10.86
320204	'Etfbkmt'	40.25	8.57	9.97	15.6	1.4	3.84	3.21	22.76	14.55	7.32
320207	'Pik3r5'	0.43	2.95	0.04	0.01	0.02	0.04	0	0.01	0.03	0
320208	'Tmem91'	266.1	169.1	228	235	261	339.4	204	246.3	223.8	187.08
320209	'Ddx11'	1.16	1.07	0.03	6.43	2	0.01	7.44	1.9	2.58	0.08
320213	'Senp5'	6.75	8.59	7.57	0.95	6.7	4.28	16	6.49	1.31	8.79
320214	'Maats1'	0.51	2.26	1.42	0.2	1.15	2.16	5.01	0.14	2.74	1.41
320225	'Catsperg1'	3.95	1	2.1	1.29	0.84	4.77	0.23	1	0	1.62
320226	'Ccdc171'	1.67	0.12	0	5.83	0.01	0.01	1.25	0	0.53	1.08
320234	'Ccdc66'	7.26	10.3	5.11	0	6.88	14.31	4.98	17.26	3.63	10.29
320244	'Ttll5'	5.98	5.01	11.1	9.71	4.8	7.06	10.1	9.74	0.34	7.6
320253	'March3'	0.43	2.56	0.94	1.84	0.92	0	0.08	0.51	0.47	0.18
320256	'Dlec1'	1.13	3.11	1.48	0	0.38	0.35	1.05	3.66	1.13	0.57
320265	'Fam19a1'	5.14	7.21	8.82	16	4.93	20.88	1.19	7.33	9.68	20.96
320267	'Fubp3'	4.62	5.52	9.31	2.85	10.1	2.81	9	3.9	3.01	5.36
320271	'Scai'	1.64	5.93	3.27	4.2	2.4	9.99	3.63	3.99	6.12	3.98
320277	'Spef2'	0	0.36	0	3.13	0.15	0	0	0	0	0.05
320292	'Rasgef1b'	0.36	4.69	5.28	1.79	1.84	1.82	6.51	17.6	1.51	3.53
320299	'Iqcb1'	37.2	41.45	55.8	51.7	50.2	44.01	55.9	49.01	43.22	42.97
320302	'Glt28d2'	0	2.3	0.02	3.78	0	0	0	0.51	0	0.02
320309	'1520401A03Rik'	0	0	0	0	0	2.59	0	0	0	0
320311	'Rnf152'	0.55	1.4	0.39	0.11	1.43	0.55	9.78	0.43	0.84	3.16
320332	'Hist4h4'	0	0	0	0	6.97	0.6	0	3.35	3.95	7.26
320333	'D830030K20Rik'	10.75	8.21	9.72	24.9	19.8	18.38	5.52	22.67	15.7	11.7
320343	'Lypd6'	1.54	3.37	0.04	0	5.98	5.84	0.01	9.4	6.37	6
320351	'Tmem251'	11.06	31.58	44.9	49	27.7	25.46	60	15.17	38.39	10.31
320352	'Lrrc31'	0.25	0.29	0.41	1.08	0.23	0.41	0.42	0.3	0.29	0.32
320354	'Grin1os'	0	0	1.47	0	0.28	0	0	0.08	0.05	0.46
320360	'Ric3'	22.51	20.73	31	23.5	12.6	23.05	22.1	25.6	35.45	28.86
320365	'Fry'	3.36	2.75	5.75	6.37	2.48	5.58	3.31	2.96	7.66	5.22
320376	'Bcorl1'	0	0.05	2.12	0	0.01	0.71	1.09	0.05	0	1.15
320394	'Cenpt'	10.64	7.37	16.7	14.7	10.6	21.3	4.87	11.68	0.11	20.08
320398	'Lrig3'	0	0	0	0	0	0.17	0	0	0	0.17
320404	'Itpkb'	1.31	2.15	2.76	0.74	0.52	0.46	3.11	3.3	5.51	2.02
320405	'Cadps2'	0.79	4.31	7.35	6.29	1.28	19.2	17.6	6.97	1.61	2.1
320407	'Klri2'	0.03	0	0.01	0	0.04	0.01	0	0	0	0
320415	'Gchfr'	6.1	12.11	0.25	16.1	45.4	38.87	0	4.08	17.56	16.37
320429	'Trank1'	4.59	3.91	4.95	9.06	9.98	6.01	7.51	6.09	5.13	7.45

320435	'Rinl'	0	0	0	0.02	1.92	5.85	2.12	1.59	0	3.8
320438	'Alg6'	9.99	6.1	5.37	2.5	5	3.97	5.54	6.93	1.77	6.5
320452	'P4ha3'	1.77	1.62	0	7.65	0	3.23	0.27	0.04	0	1.1
320454	'Tmprss11g'	0	0.02	0.02	0.02	0	0.02	0.05	0	0.01	0.03
320460	'Vwc2l'	2.07	7.98	10.2	9.67	0	0.32	0.01	2.95	7.49	1.7
320472	'Ppm1e'	1.87	0.23	0.01	2.05	1.51	1.57	1.84	0.25	0	0.46
320473	'Heatr5b'	3.14	0.2	1.76	0	0.31	4.88	0.33	1.57	2.58	4.75
320484	'Rasal3'	0	0	1.48	0	0	2.82	0	0.2	0	0
320487	'Heatr5a'	0.1	0.09	0.51	0.68	0.74	0	0.03	0.56	1.22	0.04
320492	'A830018L16Rik'	5.7	0.92	2.34	6.67	3.67	12.95	5.3	8.09	23.89	5.36
320495	'Ipcef1'	4.4	1.58	1.09	0	0.45	1.24	0.02	0.91	4.37	0
320500	'Tmem215'	27.27	45.16	14.5	5.92	10.2	22.71	12.2	30.21	5.45	4.67
320502	'Lmod3'	0.02	0	0.05	0.03	0	0.02	0.02	0	0.37	0.04
320506	'Lmbrd2'	2.48	4.19	3.17	0.04	4.62	7.18	2.25	5.53	2.82	1.22
320508	'Cachd1'	0.57	2.08	5.03	0.64	0.03	1.12	6.21	2.14	0	2.84
320522	'Bhlha9'	0	7.46	0	0	0	3.23	0.13	0	0	13.08
320528	'Vps13c'	1.54	3.1	1.78	6.2	1.57	2.63	2.61	2.29	2.65	3.07
320534	'Tmem104'	8.42	4.43	5.72	7.63	1.19	4.62	7.01	3.85	6.23	5.59
320538	'Ubn2'	2.4	1.65	2.47	8.4	3.52	2.46	2.6	2.52	2.99	3.89
320541	'Slc35e2'	3.73	4.78	7.49	3.19	6.55	1.81	3.23	2.05	3.34	4.55
320554	'Tcp11l1'	0.31	0.16	1.12	2.18	0.45	1.91	0	0.02	3.89	0.47
320557	'Fam169a'	6.03	7.93	11.8	11	5.78	1.31	5.53	5.12	13.15	5.64
320558	'Sycp2'	0.04	0.8	0.05	2.18	0.16	0	0.01	0.02	0.24	0.03
320560	'Dennd5b'	5.75	4.12	4.24	4.09	2.52	2.58	1.62	1.83	4.66	3.41
320563	'Islr2'	2.12	0.94	2.41	0	7.48	3.1	7.29	3.26	1.83	2.27
320571	'Atp8b5'	0.56	0	0	0	2.42	0.25	0.01	0.11	0	1.33
320587	'Tmem88b'	1.85	4.83	1.04	2.7	10.4	3.93	1.02	3.41	0.78	0.89
320590	'Svopl'	0	0.33	0	0	0.25	0	0.02	0	0	0
320595	'Phf8'	2.82	3.13	5.88	2.07	0.65	3.1	0.21	2.5	3.27	2.18
320604	'Ccdc169'	0	0.04	0	0.04	0	0	0	0	0	0
320609	'Strip2'	6.21	6.66	2.94	5.59	0.94	0.02	0.15	17.62	0.42	0.4
320615	'Dop1a'	4.34	5.09	4.4	0.01	3.02	5.22	2.42	3.79	4.67	5.22
320632	'Snrnp200'	8.53	3.81	7.66	3.56	3.1	0.26	1.58	8.31	9.86	10.34
320633	'Zbtb26'	0.01	0.19	1.18	2.13	0.61	1.4	0.62	0.04	0.43	0.48
320634	'Ocrl'	15.77	6.34	21.6	6.61	4.26	0.36	20.3	13.11	15.32	10.54
320635	'Cyb5r2'	0.03	2.35	0.82	0	0	13.24	0	0.37	3.82	1.42
320640	'Skint4'	0	0.06	0.03	0.12	0.06	0.03	0.07	0.02	0.09	0.01
320655	'Pgap3'	2.68	2.01	0.09	0	0.83	0.02	0	1.2	0	0
320661	'D5Ertd579e'	4.54	2.28	5.71	7.09	4.31	5.11	2.21	2.15	2.01	3.99
320662	'Casc1'	0.02	0	0	0	0.79	0	0.02	0.01	0	0
320664	'Cass4'	0	0.41	0	0	0	0	0	0	1.4	0
320678	'Iffo1'	3.48	14.59	23.4	8.74	16.8	17.66	15.1	13.34	7.08	11.62
320679	'Samd12'	14.82	9.66	8.51	1.18	21.5	4.83	21.8	12.98	10	24.47
320683	'Zfp629'	1.28	0.22	2.24	0	2.06	0.02	7.05	0.15	5.04	0.69
320685	'Dctd'	0.62	3.12	8.09	0	3.64	0	2.83	1.69	2.06	0
320696	'Ccdc158'	0	0.07	0	0.07	0	0	0	0	0	0
320701	'Fam19a4'	0	12.71	10.8	0	0	0	0.02	0	0.02	0
320705	'Bend6'	12.1	19.79	25.6	15.5	16.6	25.85	21.4	25.64	53.25	9.42
320706	'Soga1'	1.77	2.05	1.9	2.88	0.56	0.8	0.76	1.36	0.06	0.62
320707	'Atp2b3'	6.2	3.68	13.5	2.92	7.66	9.48	6.23	6.6	11.49	11.28
320709	'Tmem117'	1.02	1.66	4.26	0	3.18	1.38	2.5	0.47	2.7	1.38
320712	'Abi3bp'	0.26	0.02	0.06	0.11	0	0	0	0	0	0
320713	'Mysm1'	4.44	2.61	5.21	7.35	15.7	13.64	1.54	10.96	7.23	24.12
320714	'Trappc11'	13.04	10.82	12.6	3.32	9.1	4.59	14.2	13.13	26.27	22.43
320717	'Pptc7'	8.28	3.23	4.37	13.5	11	5.16	7.95	6.83	6.47	5.08
320720	'Fastkd1'	1.65	3.7	4.24	0.04	4	4.79	3.35	5.53	0	4
320722	'Akain1'	1.87	0	14.2	0	1.09	0	5.03	1.81	1.47	4.24
320727	'Ipo8'	10.09	8.84	4.74	7.5	5.65	1.88	7.65	1.64	18.71	6.17

320736	'Vstm4'	2.53	0	0	1.15	0	0	0	0	2.51	0
320747	'Lingo4'	0	1.46	1.26	0	2.45	0	0.02	0	0	0
320752	'Dpy19l2'	0.89	0	0	0	0	0.67	0	0.35	0	0
320772	'Mdga2'	7.41	3.57	6.34	3.92	8.28	2.09	20.4	6.34	9.85	8.32
320782	'Tmem154'	1.31	0	0	0	0	0	0	0	3.8	0
320790	'Chd7'	0.47	0.62	1.07	0.48	0.98	0.8	2.63	0.67	0.8	0.84
320795	'Pkn1'	5.02	2.12	1.72	1.28	5.16	0.33	1.37	0	1.43	4.58
320799	'Zhx3'	1.32	2.06	1.83	4.12	0.02	0	0.08	0.98	1.49	0
320802	'Ifitm10'	29.57	14.67	47.1	12.5	26	23.78	21.9	30.57	32.23	11.83
320806	'Gfm2'	3.71	12.19	10.3	9.12	11.3	7.4	3.14	7.9	17.27	6.53
320808	'Dcaf5'	6.32	1.91	4.44	0.87	3.17	0.45	5.52	3.33	2.41	3.85
320816	'Ankrd16'	24.67	8.34	17.2	26.6	13.4	5.7	12	17.94	9.82	8.24
320817	'Atad2b'	0.75	0.93	0.3	1.36	0.27	0.73	1.84	0.57	1.06	0.59
320825	'Samd5'	3.45	0.3	6.67	4.37	2.07	5.1	0	0.64	3.25	2.52
320827	'C530008M17Rik'	4.42	4.55	6.38	0.91	3.95	2.25	3.64	2.01	3.68	6.18
320840	'Negr1'	22.38	44.44	41.8	53	10.8	20.61	24.6	11.88	22.2	11.69
320844	'Amigo3'	0	0.49	0	0	0.48	0.02	0	0	4.05	1.37
320858	'L3mbtl4'	0	0	0.23	0	0	0	0	0	0	0
320865	'Cdh18'	8.01	6.15	10.5	2.26	10.1	3.66	24.5	7.35	31	10.67
320869	'Spata33'	11.14	9.79	4.93	0.02	2.59	7.01	2.52	3.04	0	9.64
320873	'Cdh10'	10.76	5.31	7.21	0.14	22	9.81	17.6	4.03	12.16	22.96
320878	'Mical2'	7.75	7.51	9.34	11.2	7.27	5.13	3.86	6.11	9.65	12.3
320910	'Itgb8'	1.29	0.45	1.51	0	2.33	0.12	1.66	2.1	0	2.33
320916	'Wscd2'	6.07	7.19	3.56	3.01	7.71	5.48	7.72	12.36	0.59	2.95
320923	'Map7d3'	0.03	0	0	0	0	0	0	0	0	0
320924	'Ccbe1'	0.01	0	0.77	0	0.74	0	0	0.03	0	0
320938	'Tnpo3'	14.2	26.48	14.3	30.5	8.07	8.63	12.5	17.88	24.52	29.07
320940	'Atp11c'	1.7	0.19	0.68	0	1.47	0	0.63	0.22	1.54	2.01
320951	'Pisd'	66.16	83.9	73.9	79	97.8	195.6	127	92.21	91.16	90.52
320974	'Lrrn4'	0	0.07	4.24	0	0	3.04	1.45	3.2	1.67	0.01
320981	'Enpp6'	0	0	0	0	5.12	0	0	0	0	0
320982	'Arl4c'	12.66	10.96	12.2	7.84	9.96	7.75	2.46	10.02	7.19	8.46
321000	'Lrif1'	18.74	12.46	10	7.38	8.97	6.38	16.6	9.74	17.39	16.24
321003	'Xpnpep3'	2.02	0.02	2.03	0.17	2.23	5.06	1.04	1.38	3.76	2.04
321006	'Dcaf1'	2.22	5.03	6.96	0.58	3.21	4.58	6.99	3.18	4.48	3.03
321007	'Serac1'	4.04	9.63	5.41	0	3.09	9.24	0.42	3.4	3.04	5.7
321008	'Zswim9'	3.69	4.26	1.65	0.03	3.23	0	0	6.44	0.01	1.78
321019	'Gpr183'	0.33	0	0	0	0	0	0	0	0	0
321022	'Cdv3'	124.7	135.8	73.3	44.4	105	135.5	95.1	113	86.38	107.62
326618	'Tpm4'	8.02	6.86	21.5	7.23	6.46	6.75	24.1	7.24	5.62	5.51
326622	'Upf2'	4.77	2.76	4.01	7.9	1.65	3.11	4.89	3.82	10.56	7.6
327655	'Ppip5k1'	11.06	11.17	14.8	19.7	9.96	4.82	11.8	5.5	15.98	13.31
327743	'Wisp3'	0	0.03	0	0	0	0	0	0	0.03	0
327747	'Mettl24'	2.66	1.83	3.51	0	7.7	0.9	0	0.81	4.08	1.86
327762	'Dna2'	0.01	0	0.63	0	0	0	1.03	0.54	0.59	0.54
327766	'Tmem26'	1.85	0.06	0.6	0	4.18	6.64	0.01	3.59	0.12	1.52
327799	'Usp44'	0.28	0	0	0.02	0	0	0.43	0	0	0
327814	'Ppfia2'	9.24	10.9	8.07	13.1	15.9	25.21	18.6	11.42	12.48	22.56
327826	'Frs2'	2.65	0.65	6.68	6.58	0.08	1.22	0.25	1.17	6.09	4.96
327900	'Ubtd2'	0.17	0.78	5.76	4.03	2.25	0	0	1.62	0	2.63
327942	'Pigl'	1.69	9.08	9.63	5.07	1.68	0	3.22	0.85	0.13	8.71
327951	'Cyb5d1'	12.51	12.61	17.5	1.49	10.1	7.77	17.9	15.88	14.75	15.91
327954	'Dnah2'	0.09	0	0.31	0	0	0.81	0	0.07	0.39	1.35
327956	'Vmo1'	0	0	0	0	0	0	0	0	0	0.96
327958	'Pitpnm3'	3.33	1.57	3.38	0.88	2.21	2.03	0.15	0.42	1.28	2.47
327959	'Xaf1'	4.88	2.24	2.29	9.22	3.82	3.26	0.54	0.66	5.82	4.52
327978	'Slfn5'	0.97	2.03	1.52	3.32	1.56	1.72	1.02	0.72	5.75	1.25
327987	'Med13'	1.04	0.68	1.25	3.25	1.1	1.7	0.95	0.23	0.05	0.55

327992	'Hsf5'	2.17	0.75	2.13	1.39	0.77	0	3.05	2.6	2.4	0.99
328019	'Spata32'	0	1.47	0	0	0.36	0	0.88	0	0	0
328035	'Fads6'	0.63	0.35	2.04	0	5.68	2.04	2.44	4.48	0	4.02
328092	'Dtd2'	33.7	31.85	33.3	33.7	41.3	26.83	31.5	49.53	37.19	31.85
328099	'Prps1l3'	15.01	7.41	5.71	3.88	4.88	2.41	2.43	9.47	12.69	12.31
328108	'Togaram1'	6.11	6.15	6.67	10.2	4.24	1.52	4.83	5.08	5.92	1.31
328110	'Prpf39'	16.68	21.93	18.7	10.3	19	20.85	34.9	22.89	18.23	52.6
328133	'Slc39a9'	9.51	10.67	17	5.75	12.4	8.45	7.14	8.83	15.52	18.12
328162	'Trmt61a'	6.27	6.47	7.46	0.03	8.97	9.39	6.7	8.14	16.43	3.92
328232	'Gfod1'	1.6	0	0.01	0.03	0.12	0	1.65	0.21	2.06	0.27
328234	'Rnf182'	1.82	5.11	3.39	0	1.56	5.55	6.77	4.02	2.91	2.3
328258	'Slc25a48'	0	0	0	0	0	0	0	0	0	3.23
328274	'Zfp459'	0.09	0.17	0.91	0.31	0.37	0.21	0.29	0.17	0.12	1.01
328329	'Mast4'	5.65	3.03	3.39	11.7	2.76	4.28	2.4	1.92	2.19	4.18
328330	'D130037M23Rik'	0	0	0	0	0.75	0	0	0	0	0
328365	'Zmiz1'	1.37	5.25	3.85	15.7	2.6	3.84	5.94	4.21	2.36	3.05
328370	'Rft1'	8.11	2.77	6.15	6.27	9.33	0.43	13.7	7.59	9.59	4.13
328381	'Sh2d4b'	0	0.36	0.11	0	0	0	0	0	0	0
328417	'Parp4'	0.2	0	0	4.06	0.04	0.84	0.35	0	0.18	1.47
328424	'Kcnrg'	0.87	2.19	0	0	0	0	0	0	0	0
328440	'Npm2'	0.44	3.53	0	0.75	2.21	0	4.69	0.47	0.82	1.56
328561	'Apol10b'	0	3	0	0	0	0	0	0	2.42	0
328572	'Ep300'	3.11	2.46	2.79	0.01	1.84	2.1	3.05	1.34	0.91	1.9
328580	'Tubgcp6'	1.61	1.42	3.31	2.17	2.69	2.49	1	2.06	2.65	1.75
328643	'Vwa5b2'	6.9	7.38	15.6	0.97	13.1	2.22	3.66	5.78	4.61	2
328778	'Rab26'	105.7	61.56	47.8	143	114	125.2	28.9	120	102	113.05
328779	'Hs3st6'	3.53	0	0	0	0	0	0	0	0	0
328783	'Mslnl'	0	0	0.23	0	0	0	0	0	0	0
328789	'Lhfpl5'	87.27	49.43	84.1	73.5	72.1	191.1	103	87.64	86.8	63.41
328801	'Zfp414'	36.47	48.97	37.2	67.4	56.2	92.15	77.3	66.64	51.58	47.34
328829	'9830107B12Rik'	0.02	0.02	0	0.04	0.02	0	0	0.01	0	0
328830	'A530064D06Rik'	0	0	0	0.03	0	0	0	0	0.08	0
328845	'Acsbg2'	0	0	0	0.06	0	0	0	0	0	0
328918	'Zscan30'	2.05	0.23	1.98	0	0	0.96	0	2.11	0.41	0.45
328949	'Mcc'	0.8	0.38	1.22	3.85	0.52	0.21	0.07	0.19	0.43	0.13
328967	'Arhgef37'	2.13	1.74	2.86	3.3	2.11	1.26	0.92	0.94	1.36	1.16
328971	'Spink10'	1.28	9.22	0	0.12	5.22	0.04	2.76	0.07	0	0.38
328977	'Zfp532'	6.57	4.37	6.82	7.95	7.69	3.94	5.67	4.39	8.14	7.68
329002	'Zfp236'	1.27	1.66	2.4	2.44	2.03	2.63	4.45	2.89	3.43	0.37
329003	'Zfp516'	0.8	0.72	0.33	0	0.82	0.99	0.32	1.59	0.39	0.4
329015	'Atg2a'	4.4	4.86	5.16	10.3	5.71	10.04	6.26	2.06	7.31	4.91
329064	'Pkd2l1'	1.15	0	0	0	0	0	0	0	0	0
329065	'Scd4'	0.16	0.1	0.11	0.03	0.22	0.1	0.05	0.05	0.08	0.08
329152	'Hecw2'	0.16	0.83	3.41	0.8	2.24	0.35	0.33	0.02	2.19	1.58
329154	'Ankrd44'	0.97	0.9	2.59	3.17	0.08	0.01	4.6	0.04	0.03	0.78
329165	'Abi2'	14.31	13.63	18.1	10.2	15.2	9.46	23.7	9.21	9.34	15.32
329178	'Unc80'	3.45	3.79	2.96	2.86	4.54	3.72	6.69	3.3	3.99	3.59
329251	'Ppp1r12b'	5.82	2.29	4.72	9.7	2.35	3.17	1.26	2.68	1.2	4.54
329252	'Lgr6'	0	3.19	0.49	0.02	1.91	5	0	0.01	0.01	0.76
329260	'Dennd1b'	0.06	0.98	0.75	0	1.46	0.01	0	0.63	3.61	2.02
329274	'Fam163a'	0	0	0	0	6.5	0	0.01	0	0	0.17
329324	'Syt14'	1.07	3.29	2.76	0.69	2.48	3.86	0.7	0.88	1.94	0.83
329366	'Ccdc187'	3.43	0.08	1.31	0	3.08	0	0	0.83	0	0.75
329375	'Cfap77'	0.08	0.38	0.62	0	0	0	0	0	0	1.22
329384	'Ptrh1'	17.97	1.96	1.23	13.5	4.2	10.27	22.3	12.04	0	12.17
329416	'Nostrin'	0.15	0.05	0	0	0	6.15	0	3.63	1.03	0
329421	'Myo3b'	0	0.01	0	0	0.41	0	0	0	0	0
329470	'Accs'	3.66	1.52	4.7	10.7	1.24	0	5.51	2.4	0.89	0.91

329482	'Dcdc5'	0	0	0.06	0	0	0	0	0	0	0
329502	'Pla2g4e'	3.01	1.2	5.12	1.86	0.14	4.98	2.88	6.16	2.35	6.24
329504	'Lcmt2'	7.05	21.06	7.05	26.4	4.39	7.83	19	6	6.9	14.02
329506	'Ctdspl2'	3.3	5.83	8.96	23.2	12.2	5.99	7.87	5.13	0.86	5.25
329509	'1810024B03Rik'	3.88	0	0	0	3.06	0	0.04	0	0	0
329540	'Nol4l'	1.94	2.45	2.72	0.2	0.57	4.62	2.07	0.27	1.93	0.09
329554	'Gm826'	0.04	0	0.06	0	0	0	0	0	0	0
329559	'Zfp335'	3.76	3.02	3.9	7.55	5.22	2.2	7.8	1.33	4.37	2.24
329575	'Gm14325'	2.55	1.81	3.37	1.18	2.52	2.05	0.89	2.69	1.75	3.5
329628	'Fat4'	0.54	0.1	0	0	0	0	0	0	0.08	0
329641	'Sertm1'	0.95	0	1.67	10.1	6.4	0	0	3.31	0.01	0
329650	'Med12l'	2.25	3.13	0.98	1.51	3.18	2.04	2.74	1.8	0.45	0.63
329659	'E130311K13Rik'	4.93	8.17	6.79	10.3	9.93	9.76	5.19	6.24	7.95	7.81
329679	'Fnip2'	0.49	0.29	0.39	1.79	0.31	0.04	2.22	0.38	1.34	0.08
329693	'Fcrl5'	0	0	0	0	0	0	0.12	0.02	0.05	0
329702	'Dcst2'	0	0	0	0	0.29	0	0	0.06	0	0
329731	'Fam19a3'	0	0.11	0	0	0	0	0	0	0	0.01
329738	'Aknad1'	0	2.89	0	0	0	0	0	0	0	0
329739	'Fam102b'	1	1.55	1.11	0.24	0.52	2.15	0.49	1.64	0.01	0.89
329777	'Pigk'	33.71	31.51	44.1	29.4	23.1	32.67	33.6	40.01	35.81	58.84
329795	'Tmem67'	3.71	13.11	7.5	13	15.9	3.26	9.77	7.95	1.81	11.52
329828	'AI464131'	7.07	4.71	5.41	0.5	0.02	4.33	0	0.99	2.36	0.69
329831	'Fam166b'	0	0	0	7.25	0	0	0	4.22	0	0.09
329872	'Frem1'	0	0	0.75	0	1.8	0	0	0	0	0.05
329877	'Dennd4c'	0.25	0.94	1.19	2.04	0.2	0	0	0.23	1.48	0.58
329908	'Usp24'	1.47	1.04	2.88	0.57	2.34	0.75	2.67	2.95	0.46	1.94
329910	'Acot11'	4.06	1.82	2.62	1.23	3.06	0.02	0.17	3.67	3.73	8.58
329934	'Foxo6'	0	1.82	0	1.12	2.92	0	0	1.16	0	0
329942	'Csmd2'	1.6	1.18	2.64	1.17	1.04	0.16	2.03	1.79	1.68	2.42
329954	'Catsper4'	0	0	0	0	0	0	0	0	0.03	1.06
329972	'Spata21'	0.03	0	0	0	0.1	0	0	0	0	0
329977	'Fhad1'	10.98	11.45	8.79	15.7	5.41	11.82	5.75	8.08	2.38	7.87
330010	'Ttll10'	0	0	0	0	0	0	0	0	0	0.98
330050	'Fam185a'	20.46	15.79	15	15.2	9.61	5.7	8.69	5.32	6.8	7.33
330064	'Slc5a6'	18.49	6.18	29.1	7.62	8.57	34.73	3.36	11.02	27.33	18.79
330097	'Gm5108'	0.18	0.19	0.29	0	0.6	0	0	0	0.35	0
330119	'Adamts3'	1.71	1.6	0.85	1.74	2.01	1.24	0.01	0.02	0	0
330149	'Hfm1'	0	3.1	0.02	0	3.06	0.17	0.7	2.46	3.52	0.56
330171	'Kctd10'	46.24	71.91	25.6	40.8	37.3	50.48	82.8	42.99	63.44	41.44
330173	'2610524H06Rik'	48.94	32.58	39	71.8	59.3	147.2	68.1	54.2	97.92	76.77
330177	'Taok3'	16.39	35.35	22.9	37.4	21.4	15.69	17.9	19.8	23.23	29.18
330188	'Ccdc63'	0	0	0	0	0.07	0	0	0	0	0
330189	'Tmem120b'	0.66	5.55	8.47	0.07	10.6	4.2	5.78	10.48	8.34	2.42
330192	'Vps37b'	4.1	15.85	8.51	16	5.38	9.63	2.87	1.43	6.84	3.93
330216	'Mblac1'	15.76	8.8	2.74	0.93	4.37	0	2.46	4.54	1.98	4.63
330217	'Gal3st4'	2.8	4	0.95	7.65	8.18	18.34	0	4.86	0.57	3.82
330222	'Sdk1'	0.57	0.89	0.51	1.73	0.9	0.01	0	0.33	1.36	0.02
330228	'Spdye4b'	0	0	0	0	0	0	0	0	0.69	0
330230	'Zfp853'	0	0	0	0	0	0	0	0.01	0	0.11
330260	'Pon2'	10.96	13.74	3.08	9.84	7.3	6.12	24.5	9.38	6.68	13.26
330267	'Thsd7a'	3.37	1.97	1.99	1.51	3.91	4.41	1.1	1.85	2.76	0.01
330286	'D630045J12Rik'	1.11	2.16	2.04	2.07	2.32	1.25	3.19	1.26	1.19	0.53
330301	'Zfp786'	2.5	3.74	0	0.02	2	0	0	2.54	0	0
330305	'Gm5111'	0	0	0.62	0	3.97	4.75	0	0	0	0
330319	'Wipf3'	2.88	1.01	0.05	0.52	0.44	1.4	0.01	0.14	1.5	0.01
330323	'Mindy4'	0.02	7.38	1.37	0.01	7.82	5.37	0.06	4.95	2.16	3.86
330355	'Dnah6'	0	0	0.03	0	0.22	2.02	0	0	0	0
330361	'Gcfc2'	0.84	0	0.02	0	1.56	0	1.02	0	3.64	1.51

330369	'Fbxo41'	1.34	3.63	1.89	4.46	3.94	3.91	0.67	4.22	2.23	2.68
330401	'Tmcc1'	3.67	4.87	2.66	9.72	4.24	3.44	1.87	2.87	1.41	5.83
330406	'B4galnt3'	0	0	0	0	0.49	0	0.02	0	0	0
330409	'Cecr2'	0	0.52	0.04	0	0.29	0	0	0	0	0
330440	'Sult6b2'	0	0	0	1.88	0	0	0	0	0	0
330450	'Far2'	19.03	9.12	17.2	14	6.35	6.45	11	11.92	16.95	8.18
330460	'Tmem150b'	0	0	0	0	0	0	0	0	0	1.12
330463	'Zfp78'	4.44	0.28	0.41	0.03	1.86	0.47	0.9	7.68	9.05	3.01
330474	'Zc3h4'	0.71	0.7	0.42	0.71	1.37	0.02	0.74	0.26	1.36	0.74
330483	'Ceacam16'	0.52	0	0	0	0	0	0	0.32	0	0
330485	'Tmem145'	17.08	17.61	32.2	14	23	8.18	29.9	18.37	51.53	4.57
330502	'Zfp82'	11.75	0.8	0.81	3.05	7.78	3.81	0	5.34	0	7.67
330554	'Fan1'	0	5.94	1.98	2.38	1.75	8.78	2.1	2.08	0.13	4.75
330577	'Saxo2'	5.9	7.49	0.91	5.46	3.07	3.72	0	2.89	4.93	3.49
330627	'Trim66'	2.64	1.42	0.58	0	4.26	1.65	1.42	1.06	1.9	0.72
330657	'Prss53'	1.19	0.8	0.36	0	0.26	0	1.48	0.02	0	3.67
330660	'Btbd16'	1.95	0.33	0.18	0.39	0.33	0.11	0.2	0.13	0.26	0.1
330662	'Dock1'	0.68	0.63	0.77	1.59	2	0.6	0.93	0.17	1.42	0.17
330671	'B4galnt4'	7.36	4.33	9.01	10.6	4.79	9.3	4.42	7.29	13.01	4.82
330695	'Ctxn1'	431.3	345.7	293	265	327	445.4	280	453.4	312.7	397.03
330721	'Nek5'	1.17	0	0	0	0	0	0	0.05	0	0
330723	'Htra4'	0	0	0	0	0	0.02	0	0	0	0
330788	'Zfp866'	2.39	0.48	1.08	1.98	9.8	3.38	3.64	5.08	3.11	8.69
330790	'Hapln4'	35.27	46.47	66.5	25.4	19.2	22.28	66.2	35.21	48.9	21.77
330812	'Rnf150'	1.06	1.39	1.25	0.47	0.89	0.23	0.21	0.81	1.52	0.25
330814	'Adgrl1'	9.81	10.95	13.5	18	17.3	8.98	13.1	10.98	17.32	5.01
330817	'Dhps'	78.96	38.2	94.8	30.1	80.1	103.8	26.7	63.31	60.25	71.29
330820	'4933402J07Rik'	0	1.8	0	0	0	0	0	0	0	0
330830	'Drc7'	0.95	4.36	1.31	3.84	2.54	1.87	0	1.65	0	0
330836	'Slc7a6'	6.96	10.19	17.5	8.64	7.95	2.73	8.25	15.87	10.76	15.12
330863	'Trim67'	0	0.36	0.69	0	0	0	0	0	0	0.98
330908	'Opcml'	13.42	20.2	38.6	21.6	30	19.67	22.6	11.7	13.38	8.34
330914	'Arhgap32'	1.83	4.49	0.53	1.25	1.18	2.87	2.01	1.9	1.15	1.09
330921	'Pate2'	0	0	0	0	2.4	0	0	0	0	0.98
330938	'Dixdc1'	10.92	8.68	6.96	9.26	6.16	5.72	13.5	6.36	3.79	4.3
330941	'AI593442'	36.99	11.81	11.5	25.1	30.9	13.51	14	21.26	45.52	29.29
330953	'Hcn4'	0.22	1.26	1.25	0	1.18	2.12	0	0	0	0
330959	'Snapc5'	35.47	25.85	24.8	37.7	29.2	55.31	42.3	38.24	61.4	48.15
330963	'Ankdd1a'	1.88	2.3	3.81	4.49	4.69	5.09	3.84	2.6	1.03	2.19
330998	'Ankrd34c'	3.03	0.9	4.01	0	4.48	1.22	0	4.74	0.48	2.24
331004	'Slc9a9'	1.65	0	2.72	0.29	7.59	1.22	0	2.62	2.18	1.43
331026	'Gmppb'	18.93	17.43	3.08	15.6	23.6	4.58	13.1	7.22	6.15	11.37
331046	'Tgm4'	0	0.04	0.03	0	0	0	0	0	0	0
331063	'Gsdmc2'	0	0	0.23	0	0	0.07	0	0	0	0
331188	'Zfp781'	8.82	5.54	6.59	5.35	2.53	6.57	6.01	4.83	1.34	5.6
331374	'Dgkk'	4.22	1.27	1.62	1.22	0.63	0.42	1.03	1.58	1.91	0.7
331401	'Thoc2'	11.17	6.65	8.95	10.2	7.78	4.33	14.6	7.47	8.38	9.77
331461	'Il1rapl1'	0.22	0.73	2.27	2.56	0.29	1.11	1.18	2.34	1.75	1.71
331474	'Rtl5'	5.75	4.07	3.75	1.07	6.25	8.52	1.75	1.52	8.1	7.75
331487	'Uprt'	0	0.58	3.61	0	0	0	0	0	0	0
331524	'Xkrx'	0.49	0.25	0.81	0.31	1.68	1.15	0.46	0.09	1.51	1.03
331532	'Tceal5'	57.91	58.48	71.3	34.8	42.4	30.39	42.4	46.67	55.36	32.5
331623	'Bend3'	0.39	0.02	0.02	0.17	0.03	0.03	0	0.02	0.73	1.48
332110	'Mapk15'	3.18	0.86	4.95	5.84	0	1.52	0	3.57	0	1.72
332131	'Krt78'	0	0	0	0	0	0	0	0	0	0.02
332175	'Zdhhc23'	0.67	0.49	0.56	2.14	0.1	0.13	2.26	0.92	0	0.24
332221	'Zscan10'	0	0	0	0	0	0	0	0	0	0.02
332359	'Tigd3'	0.01	0	2.23	0.19	3.06	2.76	0.17	1.95	5.11	0

332396	'Kcnk18'	0	0	0	0	0	0	0	0	0	0.35
332397	'Nanos1'	1.06	1.33	0.68	1.06	4.18	3.68	3.64	1.57	2.42	3.22
332579	'Card9'	0.05	0	4.14	0	1.44	3.75	0	3.1	0	0.28
332713	'Fndc11'	4.21	6.05	4.62	0	0	0	0	0	4.84	3.91
332934	'Zmynd12'	0.59	0	0	0	0.37	0.13	0	0	0	0.28
332937	'Tfap2e'	0	0	0.05	0	0	0	0	0	0	0
333048	'Tmem211'	0.01	0	0.01	0	3.14	0	0	0	0	0
333050	'Ksr2'	1.69	1.47	3.07	2.1	0.79	2.1	1.75	0.85	2.63	1.23
333088	'Kcp'	0	0	0.46	0	0.26	0.08	1.49	0.32	0.85	0.71
333182	'Cox6b2'	2.93	0	3.24	0	3.58	18.97	6.15	0	3.63	4.7
333193	'Proser3'	2.73	2.97	0.73	1.18	0.02	2.26	3.94	0	4.05	0.56
333315	'Frem3'	0	1.08	0	1.87	0	0	0	1.75	0	0
333329	'Cngb1'	3.91	2.91	0.97	0	3.74	1.35	0.03	4.05	3.25	9.04
333433	'Gpd1l'	10.96	17.55	20.7	8.88	11.4	4.98	26.5	16.06	12.13	6.88
333605	'Frmpd4'	0.51	0.64	3.32	1.82	0.51	2.26	0.31	1.42	2.85	0.65
333639	'Mamld1'	10.36	9.34	10.6	10.4	1.75	0.99	5.89	1.88	5.09	6.12
333654	'Ppp1r13l'	0.38	0.02	0.07	3.56	0.02	0	0.24	0.29	0	0.4
333669	'Gm5134'	0	0	0	0	2.29	0	0	0	0	0
333670	'Gm867'	0.32	0	0	0.67	0.36	0.46	0	2.92	0.12	0
333715	'H2-M10.2'	0.83	0.3	0	0	0	0	0	0	0	0
333789	'N4bp2'	5.8	2.79	2.36	5.31	0.5	0.01	1.54	0.97	1.98	4.71
333883	'Cd59b'	2.51	0.44	1.42	2.56	0.77	3.4	0.04	3.29	0.56	0.94
338337	'Cog3'	6.1	5.2	1.4	2.34	3.6	0	17.6	3.02	6	6.27
338346	'Gpr21'	0	0.81	0	0	0.02	0	5.22	0	2.05	1.07
338348	'Ttc16'	0.59	1.41	0.38	0	0	0	1.08	1.15	0	0
338349	'Cntln'	0.43	1.01	0.12	5.65	1.47	0.09	2.96	0.36	1.57	1.15
338350	'Acad12'	0	0	0	0	0.5	0	0	0	0.02	0
338351	'Akap17b'	8.22	6.08	6.13	11.9	7.8	3.46	6.24	9.62	2.08	10.38
338352	'Nell1'	76.4	10.24	40.1	41.6	16.4	13.38	10.8	93.88	101.4	79.97
338354	'Zfp780b'	4.4	5.56	4.35	1.19	6.71	2.59	5.55	5.5	4.24	10.03
338355	'Fkbp15'	5.24	2.95	17.9	2.74	6.46	0.38	2.14	2.9	7.19	4.52
338359	'Supv3l1'	5.85	7.4	6.89	2.48	5.03	4.4	6.1	7.88	3.95	12.48
338362	'Ust'	0	0.99	0.08	0	0.01	0	8.73	1.65	0.09	0
338363	'Tmem241'	6.36	14.5	13.8	8.33	3.83	14.73	11.7	8.71	16.33	18.84
338364	'Trim65'	0.67	1.15	3.4	0.18	0	4.37	5.92	0	0	0.58
338365	'Slc41a2'	11.53	2.86	2.76	2.28	2.67	2.34	0.04	5.44	6.85	1.47
338366	'Mia3'	8.53	11.56	16	14.5	13.4	9.21	8.74	9.06	11.05	16.57
338367	'Myo1d'	0.03	0.03	0.06	0	2.68	1.98	5.4	1.62	0	1.28
338368	'Pheta2'	0	0	0	0	0	0	0	0	0	0.02
338369	'Tmem220'	0	0	0	0	0	0	0	0	0	0.27
338370	'Nalcn'	4.42	8.64	12.7	2.98	7.64	0.47	5.35	6.96	10.44	9.06
338371	'Endov'	6.12	2.08	2.97	20.3	10.3	10.47	0.13	10.57	9.93	7.91
338372	'Map3k9'	0.11	0.23	0.9	0	1.03	0.25	0.64	0.59	0.64	2.04
338403	'Cndp1'	0	0	1.6	0	0	0	0	0	0	0
338417	'Scgb1c1'	0.12	0	0	0	0	0.12	0	0	0.11	0
338467	'Morc3'	12.35	11.64	6.24	2.09	9.31	1.96	5.83	7.53	3.04	9.92
338521	'Fa2h'	3.08	7.79	3.9	0	4.09	16.83	7.92	2.85	2.48	0
338523	'Kdm7a'	0.17	0.86	1	2.92	0.65	0.32	1.2	0.88	0.41	0.4
347710	'Pramel4'	0.03	0	0.06	0	0.06	0	0	0.02	0.03	0
347722	'Agap1'	2.11	5.27	3.26	5.81	4.23	4.98	2.55	2.38	3.26	4.55
353047	'Plekhm1'	2.19	0	0.5	0	3.64	0.08	3.71	3.04	1.62	1.59
353155	'Gjd3'	0	0.24	1.81	0	1.3	1.39	0	0.5	1.14	0
353156	'Egfl7'	35.5	47.17	31.7	82.9	39.6	38.15	2.41	66.35	54.93	39.2
353169	'Slc2a12'	0	1.06	2.78	0	0	0.12	0.07	1.62	0.01	0.02
353170	'Txlng'	9.18	10.47	6.7	9.37	5.83	4.24	5.12	6.12	4.71	14.57
353172	'Gars'	68.82	53.31	94.2	39.8	42.4	57.66	111	83.85	69.69	72.42
353187	'Nr1d2'	19.07	8.5	21.6	14.8	11.2	2.41	25.5	5.7	17.06	12.72
353190	'Edc3'	17.95	3.73	16.6	1.88	16.8	5.66	6.44	6.51	10.58	15.14

353208	'Zfp931'	1.55	3.51	0.82	1.14	3.36	2.1	0.69	3.15	0.54	1.94
353211	'Prune2'	35.18	21.7	22.4	48.8	52.7	37.86	16.4	37.74	29.97	50.88
353234	'Pcdha2'	1.98	4.93	6.68	0.13	0.08	0.01	0	3.42	0	0.03
353235	'Pcdha8'	0	0	2.34	8	0	0	0	0.31	0	0
353236	'Pcdhac1'	0.6	2.19	0.02	0.13	0.51	0	0	0	0	0.27
353237	'Pcdhac2'	1.67	4.95	2.24	11	3.76	5.26	6.53	2.85	1.71	0
353242	'Mrpl21'	62.04	59.96	58.3	51.6	74.3	78.09	88.5	74.48	84.06	68.92
353258	'Ltv1'	24.56	11.42	7.55	19.2	9.2	19.76	11.1	17.75	14.02	23.2
353282	'Sfmbt2'	0.27	1.95	1.28	0	1.92	5.3	0.04	0.19	0.04	0.01
353287	'Clec18a'	0.02	0	0.48	0	0	0	32.6	0	0	4.76
353310	'Zfp703'	1.47	1.69	0.57	1.63	0	0	0	0.17	0	0
353326	'Rtl1'	3.92	1.15	1.6	2.28	4.38	1.48	2.24	1.72	5.95	3.25
353328	'Muc6'	0	0.32	0	0	0	0	0	0	0	0.81
353344	'Opn5'	0	0	0.03	0	0	0	0	0.02	24.95	22.81
353371	'Oxct2b'	0.96	1.64	0	0	0.39	0.5	1.19	0.88	1.84	2.18
353499	'Tmc4'	6.53	3	6.87	5.42	0.36	1.09	0.68	4.31	2	3.53
353502	'Hcfc1r1'	136.5	125.6	153	125	154	145.2	92	83.37	94.6	119.6
360013	'Myo18a'	5.15	5.63	5.35	6.69	2.51	3.83	7.6	1.87	2.37	4.2
360198	'Hist1h3a'	0.1	1.93	0.12	0	0	6.14	0	0	0	0
360213	'Trim46'	24.84	31.33	33.7	15.7	23.5	28.2	35.9	17.19	10.34	15.23
360216	'Zranb1'	8.58	12.41	7.13	22.2	6.36	8.43	3.05	8.19	3.33	6.69
360220	'Speer4d'	0	0	0	0	0	0.17	0	0	3.69	0
368203	'Gm5136'	0	0	0	0	11.9	0	6.18	3.25	0	0
373864	'Col27a1'	0	0	0.02	0.09	0	0	0	0	0	0
378430	'Nanos2'	0	0	1.56	0	0	0	0	6.25	0	0
378431	'Txlnb'	0.02	0	0.02	0	0.01	0.01	0	0.02	0.02	0.02
378460	'Pram1'	0	0.04	0	0	0.09	0.02	0	0	0	0
378462	'Morn2'	80.09	133.9	55.9	75.1	126	94.86	40.4	79.54	86.55	84.18
378700	'Bpifb3'	0	0	0.03	0	0.03	0	0	1.54	0	0.57
378702	'Serf2'	41.41	43.82	46	44.4	62.5	72.96	56.3	38.32	33.54	56.05
378937	'Lrrc24'	45.55	30.2	47.5	18.7	44	27	58.7	63.24	28.05	29.08
380601	'Fastkd5'	7.17	4.36	0	0.11	3.45	0	4.06	2.4	2.32	2.54
380608	'Tagap1'	38	47.44	32	46.9	36.2	18.08	27	22.56	22.44	36.91
380614	'Intu'	2	2.87	2.11	5.08	5.12	2.1	3.55	2.54	3.5	3.09
380629	'Heca'	0.25	1.36	0.62	0	0	0.22	3.58	0.03	0.04	2.81
380654	'Cfap54'	0.87	0.39	1.83	0.08	1.29	1.13	0.34	0.47	0.63	0.6
380660	'Acss3'	3.58	0	0	0	0	0	0	0	0	1.24
380664	'Lemd3'	0.58	0.61	2.42	1.96	0.22	0.83	0	0.34	1.45	1.72
380669	'Lin28b'	1.21	0	0.07	0.04	0.84	1.96	2.01	0.72	3.12	0.74
380683	'Sec14l3'	3.36	5.83	4.26	15.2	3.6	2.18	0.6	0.24	0.01	3.22
380684	'Nefh'	1.77	0	5.62	13.9	11.8	1.29	5.14	1.85	4.04	3.24
380686	'Cnrip1'	147.6	113.1	95.5	182	90.6	125.6	87.7	119.7	117.3	128.32
380694	'Ccnjl'	0	3.47	1.87	0	0	0.02	0.2	0	0	0
380698	'Obscn'	0.01	0	0.02	0	0.01	0.01	0.01	0	0.01	0.01
380702	'Shisa6'	1.22	0	0.62	0	0.01	0	0	0.11	1.05	0.23
380705	'Tmem102'	0	0	0	0	1.02	0	0	0	0	0
380711	'Rap1gap2'	5.08	7.85	19.1	2.5	1.6	6.07	8.53	3.88	10.08	2.2
380712	'Tlcd2'	0	1.89	0	4.45	0	0	0	0	0	0
380713	'Scarf1'	0	0	0	0	0	0	0	0	0.18	0
380714	'Rph3al'	2.9	9.21	1.78	6.89	7.64	4.39	5.11	5.09	0.02	1.1
380718	'Mks1'	5.18	8.11	14.2	2.64	7.8	3.82	0.23	10.74	3.86	11.96
380728	'Kcnh4'	1.63	0.46	0.13	0.18	0.08	0.02	0.14	0	2.1	0.01
380732	'Milr1'	0	0	0.6	0	0.07	0.03	1.96	0.38	0.55	0
380752	'Eipr1'	78.44	63.47	58.3	62.3	57.3	64.71	27.6	74.37	85.17	38.48
380753	'Atxn7l1'	43.28	38.38	28.8	18.4	9.71	54.25	19	46.29	24.61	28.8
380755	'Lsmem1'	0	0	0.93	8.63	0.04	6.34	0	0	0	0.07
380768	'Ccdc177'	0.43	1.7	0	0	0.55	0.01	0	0.69	0	1.17
380773	'Slirp'	209.2	277.5	271	295	231	351.9	237	180	403.8	201.06

380785	'Begain'	7.81	12.13	21.2	30.2	12.3	18.62	18.8	11.71	24.92	9.81
380787	'Lbhd2'	91.32	93.31	138	155	79.6	142.4	21.5	160.7	128.4	222.05
380836	'Mrs2'	3.48	5.1	16	5.28	3.98	5.19	4.17	1.42	8.26	2.29
380840	'Lyrm4'	99.42	109.9	119	221	139	128.8	149	90.35	125.5	127.58
380850	'Gm5141'	1.82	1.04	4.64	0.1	0.05	0.01	0	1.89	4.51	1.64
380855	'Rsl1'	0.95	0	2.64	0	0	7.44	0	0	7.8	0.25
380882	'Gm906'	0	0.02	0	0	0	0	0	0	0	0
380912	'Zfp395'	1.89	0.78	2.17	4.29	2.91	3.72	4.01	1.09	1.16	1.1
380916	'Lrch1'	9.64	9.31	13	16.2	6.84	1.47	6.15	4.47	10.85	2.32
380918	'Siah3'	0.61	0.78	5.55	13	0.17	0	0	0.37	0	0
380921	'Dgkh'	3.56	3.72	2.34	5.04	1.28	1.49	8.2	2.8	0.61	1.78
380924	'Olfm4'	0	0	4.66	0	0	0	0	0	0	0
380928	'Lmo7'	0.62	0.65	0.21	0.01	0.09	0.02	0.01	0.07	0.74	0.01
380959	'Alg10b'	5.83	5.74	1.93	7.08	3.68	0.46	6.05	3.62	11.19	0.35
380967	'Tmem106c'	88.81	92.32	98.8	91.2	73.2	83.89	76	81.42	102.5	110.54
380969	'Nckap5l'	1.78	1.67	2.88	0	0.03	1.4	1.96	0.34	0.8	2.23
380993	'Zfat'	0	1.4	0.41	1.62	0	0.03	0.29	0.41	0.06	0.08
380997	'Cyp2d12'	0.1	0	0	0	0	0	0	0	0	0
381022	'Kmt2d'	5.12	4.16	3.55	2.65	7.55	1.94	3.93	4.45	7.27	6.64
381038	'Parl'	31.88	56.65	44.4	104	53	43.86	18.1	45.88	56.07	46.55
381045	'Ccdc58'	6.64	8.44	14.3	1.84	7.95	8.04	6.23	7.13	1.94	8.75
381058	'Unc93a'	0	0.05	0	0	0	0	0	0	0	0
381059	'Gm1604b'	10.31	11.36	13.1	8.06	22.7	10.34	11.6	14.8	3.92	16.64
381062	'Ermard'	14.28	11.18	13.3	3.07	8.2	9.25	13.4	13.91	3.94	18.23
381066	'Zfp948'	28.26	18.2	7.42	6.02	24.5	24.12	16.7	13.73	21.96	4.64
381067	'Zfp229'	2.61	0.21	0.03	2.61	0.31	1.14	0	0.5	2.98	0.18
381072	'Abca17'	0.54	0.15	0	0	0	0	0	0.22	1.58	0
381077	'Ccdc78'	0.6	0.32	1.7	0.38	0.12	0.16	0.89	0.08	0.11	0.16
381085	'Tbc1d22b'	1.06	2.6	3.65	8.56	2.88	3.86	2.32	5.8	5.25	3.54
381101	'Dnph1'	4.52	14.26	8.82	17	5.36	0	12.9	8.26	5.41	2.59
381104	'Prickle4'	5.03	5.54	5.14	5.57	5.22	2.65	1.21	4.81	1.34	3.69
381107	'Tmem232'	0.38	0	0	0	0	0	0	0	0	0
381110	'Rmdn2'	8.73	4.5	0.74	14.1	6.98	2.22	4.48	2.75	0.01	2.67
381112	'Arhgef33'	0.11	0.16	0.47	0	0.45	1.01	0	0.92	0.3	0
381113	'Cdkl4'	16.2	19.28	2.25	40.7	9.77	10.94	8.55	5.33	0.03	8.05
381122	'Capn13'	0	0	0	0	0	0	0	1.29	0	0
381126	'Garem1'	2.36	1.09	0.42	0.86	1.61	0.66	0	0.03	2.75	1.9
381142	'Arl14epl'	0	0	0.15	0.05	0	0	0	0	0	0
381148	'Prob1'	0	0	0.75	0	0.2	1.72	0	0.01	0	0
381157	'Greb1l'	4.69	1.88	2.55	0.14	0.62	0.04	0.21	1.23	0.04	0.15
381196	'Gm960'	5.39	0.56	0.2	0	1.95	0	1.9	2.14	0	0.16
381199	'Tmem151a'	36.32	32.69	33.9	22.7	21.3	16.29	19.9	23.78	22.95	11.6
381201	'Ap5b1'	0.02	0	0	0	3.48	0	0	0	0	0
381204	'Naaladl1'	0	0	0.19	0	0	0.04	0	0	0	0
381217	'Fam189a2'	4.66	4.8	0	2.49	6.7	0.34	0	3.18	0.03	0
381218	'4430402I18Rik'	2.28	0.04	4.12	0	3.56	0	0.51	0	0.02	2.14
381229	'Cfap58'	0	0	0.03	0	2.45	0	0	0	0	0.33
381236	'Lipo3'	6.85	4.35	1.16	2.73	6.77	7.27	10.2	7.37	6.33	9.38
381259	'Tmem237'	44.68	57.68	26.5	46.4	30.5	36.78	19.3	42.68	51.7	40.32
381260	'Gm973'	5.94	1.9	1.3	17.8	1.47	3.88	0.14	4.54	0.28	0.83
381269	'Mreg'	3.48	1.11	1.34	0	2.67	0.24	0.72	3.36	0.84	1.27
381270	'March4'	0.02	0.86	1.42	8.14	1.18	2.14	4.72	0	1.87	6.25
381272	'A630095N17Rik'	0	0	0	0	0	1.02	1.74	0	1.26	0
381280	'Hjurp'	14.25	12.57	6.77	9.96	3.8	10.16	25.9	17.13	19.48	18.04
381284	'Crocc2'	0	0.26	0.04	0	1.08	0.29	0	0	0	0.75
381290	'Atp2b4'	1.98	3.41	4	4.16	3.39	2.3	2.74	1.83	0.68	1.26
381293	'Kif14'	0	0	0	0	0	0	0	0	0.53	0
381305	'Rc3h1'	3.19	1.34	1.69	1.38	1.52	2.04	0.46	1.1	1.76	0.29

381306	'BC055324'	2.1	0.07	0.02	0	0.05	0	0	0	2.67	0
381308	'Ifi211'	0.06	0.07	0.18	0.08	0.12	0.08	0.1	0.03	0.09	0.09
381310	'Stum'	0.18	2.04	3.55	4.09	0.63	2.67	1.86	1.68	0	0
381314	'Iars2'	6.76	5.09	9.32	2.7	9.73	0.18	13.2	5.47	0.02	3.67
381318	'Nsl1'	0.42	0.58	0.71	0.83	0.51	0.66	0.39	0.38	0.54	0.49
381319	'Batf3'	0	0	0	0	0	6.21	0	0	0	0
381334	'Gal3st2'	0	1	0	8.97	0	0	0	0	0	0.18
381338	'Lonrf2'	18.34	15.78	20	30.7	8.43	6.47	14.6	13.5	16.97	17.78
381339	'Tmem182'	0.02	0	0.08	0.05	0	0.02	0	0	0.04	0.03
381350	'Spag6'	2.93	0	0	0	0.52	0	0	0.03	0	0
381352	'Mamdc4'	0.18	0	0	0	0	2.29	0	0	0	0
381353	'Ajm1'	7.43	8.37	8.56	2.89	6.71	4.09	10.8	6.55	0.45	13.82
381356	'Cacfd1'	57.87	71.35	82.3	46.1	82.3	73.17	71.9	59.65	95.47	74.19
381359	'Prdm12'	0.46	0	0.6	0.01	0	0	0	0	0	0
381373	'Sp9'	0	0.07	0	0	0.92	0.16	0	0	0	0
381375	'Pjvk'	0	3.11	0	16.4	0	0	0	1.65	0	0
381379	'Med19'	13.91	17.28	17	11	29.8	18.7	29.5	22.29	19.41	16.33
381399	'Bpifb4'	0	0	0	0	0.63	0	0	0	0	0
381404	'Pabpc1l'	0	0.67	0.45	0.57	0	0	0	0.16	0.02	0
381406	'Trp53rka'	6.35	2.85	19	9.71	5.13	0	3.27	2.96	17.68	4.97
381410	'Zfp408'	9.3	3.05	6.36	0.55	3.06	6.28	3.18	2.19	2.6	0.1
381411	'Accsl'	0	0	0.28	0	0	0	0	0	0	0
381413	'Gpr176'	4.21	3.29	12.6	7.7	5.1	9.13	4.54	7.71	5.64	13.42
381418	'Ctxn2'	128.9	107.7	151	71.7	193	264.9	246	157.5	188.8	145.07
381438	'Gm5148'	17.95	21.73	7.35	9.71	20.9	0.86	12.4	19.42	7.76	11.18
381463	'Nr1h5'	0	0.05	0.02	0.04	0.06	0.03	0	0.03	0	0
381489	'Rxfp1'	2.56	0	0	0.25	0	0	0	0.98	0	12.73
381510	'Dpy19l4'	3.04	3.02	2.76	7.3	0.53	2.02	3.94	1.36	0.16	1.66
381511	'Pdp1'	11.63	11.31	7.93	8.81	13.2	7.79	8.65	18.24	18.33	5.57
381522	'Ccdc180'	0	0	0	0	0	0	0	0	0	0.01
381534	'Ube2u'	0	0.42	0.22	0	0	0	0	0	0	0
381538	'Mroh7'	1.4	1.17	0.47	3.35	0.01	0	1.71	0.82	0.87	0.29
381544	'Armh1'	0	0	0	0	0	0	0	0.48	0	0
381546	'Ccdc24'	0	0	0	7.87	0	0	0	0	0	3.5
381549	'Zfp69'	0.6	0	0	0	0.48	0	0	0	0	0.97
381560	'Xkr8'	1.08	2.56	2.25	0	5.37	3.85	8.05	4.28	0.02	6.03
381582	'Tmem240'	7.16	2.1	14.6	0.16	20.9	7.83	9.53	6.4	14.1	5.26
381591	'L1td1'	0	0	0	0.04	0	0	0	0	0	0
381605	'Tbc1d2'	1.72	0	0	0	0.34	0	0	0	5.56	1.28
381622	'5031410I06Rik'	0	0	0.13	0	0.02	0	0.02	0.13	0.17	0.02
381626	'Rbm33'	0.17	1.01	0.05	4.84	1.12	3	1.97	0.52	4.19	0.75
381628	'Adgrf3'	0.34	0	0	1.57	0	0	2.46	0	0.41	0
381629	'Atraid'	192.6	286.9	245	375	245	298.5	220	282	317.3	321.62
381633	'Gm1673'	253.4	276.3	214	328	181	309	303	171.3	434.4	290.65
381634	'Gm1043'	1.47	0.8	0.63	3.09	3.04	1.44	1.75	0.63	0.35	1.59
381644	'Cep135'	1.38	0.85	1.11	2.16	2.07	0.76	1.8	0.6	0.45	2.01
381651	'Odaph'	0	1.94	0	0	0	0	0	0	0	0
381668	'Fbrsl1'	3.5	0.67	1.33	11.8	1.38	3.7	4.7	0.56	6.81	0.41
381673	'A330070K13Rik'	0.98	0	0	0	0	0.55	0	0	0	0
381677	'Vgf'	33.16	69.68	36.6	9.94	13.3	41.99	10.7	38.11	83.11	34.85
381678	'Zcwpw1'	0	4.44	0.98	0.04	1.29	9.15	0	2.33	3.64	5.59
381693	'Wdr95'	3.61	0.31	0.31	0.05	0	0.18	0.68	0.82	0	0.93
381694	'B3glct'	1.42	0.66	2.46	0.01	1.65	0.18	2.07	1.57	0.99	1.52
381695	'N4bp2l2'	7.26	8.53	9.22	12.7	8.79	10.39	9.68	6.4	6.07	14.64
381714	'Gm9758'	0.22	0.27	0	0	0	0.54	0.17	0.09	0.14	0
381716	'1700015F17Rik'	0	0	0	0	0	0	0	1.39	0	0
381738	'Drc1'	21.4	14.58	20.6	25	11.9	19.95	22.4	12.99	9.34	13.51
381741	'Lrrc43'	0	4.51	0	0	2.84	1.53	0	5.4	2.07	4.78

381759	'Wee2'	0.13	0	0	0	0.24	0	0	0	0.02	0.79
381760	'Ssbp1'	38.75	28.72	36	40.6	52.8	47.35	16.8	55.02	80.57	51.81
381798	'4930590J08Rik'	0	0.61	0.02	0.27	0	0	0	0	0.09	0
381801	'Tatdn2'	0	0.99	0	0.17	0.02	0.87	0	0	0	0
381802	'Tsen2'	8.72	5.18	13.6	9.02	10.1	0	33.7	19.24	9.71	31.12
381810	'Lpar5'	0	0	0	0	0	0	2.05	0	0	0
381812	'Cracr2a'	0.34	2.24	0.85	0.66	0.22	0.12	0.09	3.08	0.6	0.13
381813	'Prmt8'	3.92	0	4.2	2.7	15	10.88	3.53	12.84	10.63	1.42
381820	'Smim10l1'	61.95	101.7	58.2	61.5	117	43.1	61.8	62.82	57.75	61.65
381823	'Apold1'	2.41	0.98	0	0	1.5	9.64	2.29	1.8	6.51	7.84
381835	'Sbk3'	0.07	0	0	0	0	0	0	0	0	0
381838	'Vmn2r43'	0	0	0	0	0.01	0	0	0	0	0
381845	'Rnf225'	0.01	0.01	0	0.03	0.03	0.03	0	0.01	0.46	0
381853	'Gipr'	0.95	0.28	0.87	0.3	0.12	0.08	0.12	0.03	1.03	0.11
381884	'Slc6a16'	6.72	3.24	2.9	1.8	4.9	6.87	2.05	5.36	3.78	10.46
381903	'Alg8'	18.62	1.8	19.1	16.2	18.1	6.7	0.06	6.57	8.27	12.07
381914	'B230311B06Rik'	3.18	0	5.93	0	0	3.62	0.5	0	0	0
381917	'Dnah3'	0.01	0	0	0	0	0	0	0	0	0
381921	'Taok2'	7.54	6.06	9.79	4.45	5.78	5.13	1.27	8.95	1.75	11.23
381925	'Plpp4'	5.49	0	9.44	0	20.5	46.69	22.2	1.95	7.7	9.85
381933	'6430531B16Rik'	0.15	0	2.34	0	0	0	0	0.09	0.39	0.1
381979	'Brsk1'	1.33	0.04	0.02	0.06	0	0.03	4.12	0.12	0.37	0.27
381983	'Lmtk3'	0.27	0.06	0.34	0.6	0.49	0.07	0.05	0.16	0.1	0.14
381990	'Zbtb2'	3.2	0.03	7.18	7.36	0.9	3.32	0.02	7.2	0.27	0.31
381994	'E030018B13Rik'	0	0.2	0	0	0.55	0	0	0	0	0
382010	'Cep44'	4.78	3.2	2.38	0.82	4.66	6.09	14.3	3.42	1.5	10
382014	'Ano8'	0	0	0	0	0.2	0	0.11	0.05	0.02	0.01
382018	'Unc13a'	1.54	4.5	4.71	1.63	5.1	1.79	6.89	4.06	9.53	11.28
382019	'Zfp882'	2.71	0.78	0.04	0.1	4.38	0.07	0.67	1.22	0.04	3.73
382030	'Cnep1r1'	17.56	33.82	18.1	16.2	55.5	8.97	39.8	22.26	12	19.38
382034	'Gse1'	7.71	2.18	5.59	7.66	6.43	5.09	4.15	1.36	0.39	1.28
382038	'Urb2'	0	0.02	0	0	0	0	0	0.1	0	0
382051	'Pdp2'	8.37	6.14	6.64	22.8	13.5	5.55	6.13	10.17	8.48	7.3
382056	'Crtc1'	11.21	26.54	25.1	13.3	20.4	16.62	38.9	27.2	32.1	37.21
382066	'Prdm10'	0.57	1.69	1.33	0.01	0.98	1.25	0.55	0.65	1.87	1.37
382073	'Ccdc84'	18.4	14.26	40.3	19.6	13	9.14	11.4	29.52	13.32	10.42
382074	'Foxr1'	0.13	0.25	0.09	0.07	0.15	0.03	0.09	0.03	0.08	0.05
382075	'Odf3l1'	0	0	0.18	0	0	0	0	0	0	0
382077	'Ccdc33'	0	0	0	0	0	0	0	0	0.02	0
382083	'Snx22'	0.53	0.06	6	9.08	13.1	4.56	12.4	7.72	0.96	1.58
382089	'Ripply2'	5.57	9.99	2.04	4.51	12.3	10.13	10.3	16.99	8.69	10.87
382090	'Cep162'	8.45	4.8	4.69	1.25	11.9	3.3	5.08	3.79	4.16	9.66
382097	'Gm1123'	0.03	19.61	0	0	0.04	0.17	0	0	0	0
382111	'Susd5'	1.89	0	11.4	0	0.05	0	26.5	0	0	0.01
382113	'Slc22a14'	0	0	0	0	0	0	0	0	0.02	0
382117	'Tcaim'	3.55	4.92	8.66	1.35	1.69	5.16	9.91	9.58	6.67	8.43
382118	'Zkscan7'	0.97	0.23	3.28	0.06	0.33	0.01	0.25	0.56	0.17	0.16
382137	'Fdxacb1'	2.21	4.6	1.29	4.04	10.4	3.56	5.32	3.07	9.34	8.09
382206	'Ssx9'	0	0	0	0	0.04	0	0	0	1.29	0
382207	'Jade3'	2.66	0.34	0.1	0.02	0	0	0	1.59	1.47	0.35
382236	'Brwd3'	0.09	0	0.75	0	0.4	0.01	0.01	0	1.03	0.85
382244	'Gm15091'	0	0	0	0.1	0	0	0	0	0	0
382245	'Tmem29'	39.96	54.15	34.4	66.6	39.3	41.77	59.8	65.21	32.68	80.44
382252	'Bclaf3'	5.03	8.92	0.86	6.61	8.07	6.82	4.43	3.26	9.77	8.19
382253	'Cdkl5'	4.29	0.2	2.62	6.85	0.11	0	0.17	3.09	0	4.34
382265	'Cldn34c3'	0.4	0.09	0	0	0.3	0	0	0	0	0.52
382384	'Odf3l2'	0	0	0	0	2.74	5.25	0	6.67	4.97	2.8
382395	'Gm5174'	3.17	3.22	3.24	0.95	0.24	0	2.41	3.89	0.04	3.25

382406	'Poc1b'	1.47	0.26	0	9.37	1.07	1.71	0.21	0	0	1.63
382423	'Atxn7l3b'	48.82	73.82	62.2	52	70.2	74.58	66.8	76.73	77.19	58.79
382427	'Best3'	0	0.02	0	0	1.19	0	0	0	0	1.35
382523	'Gm12260'	0	0	2.43	0	0	0	0	0	0	2.26
382543	'Ankfn1'	0.04	1.19	4.28	0.99	0.48	0	3.56	0.52	0	0.05
382562	'Pfn4'	10.53	7.25	8.02	40.3	2.22	3.38	5.2	11.19	0.92	5.1
382571	'Kcnf1'	9.33	14.04	3.86	9.23	7.71	1.75	2.23	7.23	9.51	6.67
382620	'Tmed8'	0.36	0.81	0.25	0.75	0.45	0.28	2.51	0.45	0.18	2.98
382639	'Zbtb42'	0	0	0.01	0.93	0	0	0	0	0	0
382793	'Mtx3'	3.12	2.67	0.91	0	5.62	0.1	5.86	0.31	2.39	1.05
382864	'Colq'	0	0	0	0	0	0	0	0.08	0	0.02
382867	'Zfp488'	47.09	52.61	77.2	80.1	83.4	62.88	65.5	44.01	88.32	61.34
382913	'Neil2'	0.52	0.06	0.41	0	2.85	0.05	0.01	1.55	2.58	0.55
382985	'Rrm2b'	3.58	8.48	14	6.25	9.26	5.94	8.21	5.12	5.32	10.41
383103	'Tvp23a'	20.08	12.51	21.1	4.95	12.3	16.18	25.8	18.03	9.05	8.16
383295	'Ypel5'	97.3	113.1	101	109	93.4	109.5	88.7	121.7	97.63	125.9
383348	'Kctd16'	9.76	8.66	2.53	0	20.6	15.39	13.1	22.13	4.13	0
383563	'Gpr25'	0.11	0.1	0.17	0	0.3	1.94	0.18	0.1	0.42	0.17
383592	'Kif28'	0	0	0	1.18	0	0	0	0	0	0
383619	'Aim2'	0.3	0.28	0.79	0.37	0.78	2.33	4.82	0.41	0.34	1.46
383678	'Obp2b'	0	0	0	0	0	0	0	0	0	1.62
383766	'Tldc2'	0.03	1.82	0.04	0.09	0.1	0.08	0.55	0.04	0.07	0.02
383787	'Ankrd63'	0	0	0	0	0.24	0	0	0.01	0	0
384009	'Glipr2'	3.23	2.19	0	0	2.86	0.02	5.11	0	0	0
384061	'Fndc5'	2.86	4.86	5.09	0	6.01	5.97	4.89	3.87	7.36	6.09
384071	'Slc25a34'	0	0	0	0	0	0	0	0	0	3.31
384185	'Arl9'	0	0	0	0	0	0	0	0	0	1.43
384198	'Fam47e'	0	0	0	0	0	0	0	0	3.76	0
384214	'Ephx4'	8.74	6.09	8.21	10.6	1.23	4.97	0	0.11	0.72	4.74
384281	'Gatc'	32.98	34.45	26.4	21.8	43.5	36.79	53.9	33.15	26.33	27.97
384309	'Trim56'	3.23	0.38	0.57	0.65	0.54	0.36	0.49	0.05	0.16	0.65
384534	'Vmn2r52'	0	0	0.02	0	0	0	0	0	0	0
384569	'Nova2'	2.09	1.41	2.41	1.47	1.02	2.54	0.7	1.17	0.16	1.85
384605	'Wdr88'	0	0.03	0	0	0	0	0	0.14	0	0
384619	'Ccdc155'	0	0.37	0	0	5.95	0.23	0	0.17	0	0
384701	'Usp17ld'	0	0.03	0	0	0	0	0	0	0	0
384724	'Cyp2t4'	0	0.03	0	0	0	0	0	0	0	0
384763	'Zfp667'	6.6	7.57	4.56	19.9	6.93	6.41	4.02	15.82	11.12	7.03
384783	'Irs2'	5.73	13.25	9.83	12.1	1.95	7.48	3.3	5.65	0.81	7.5
384806	'Adam20'	0	0	0	0	0	0	0	0	0	0.02
385024	'Gm5373'	0.05	0	0	0	0	0	0	0	0	0
385312	'Ssxb10'	0	0	0	0	0.23	0	0	0	0	0.07
385354	'Frmd7'	0	0	0	0	0	0	1.67	0	0	0
385377	'Pnma5'	0.06	0.02	0	0.02	0.02	0.31	0	0.09	0.32	0.13
385658	'Nxpe3'	5	4.75	2.11	0.6	0.33	6.23	2.82	4.55	5.37	2.41
385668	'Lca5l'	1.02	0.05	2.42	0.6	1.08	2.09	0	0.25	0.98	0.32
385674	'Zfp174'	3.84	0.22	0.16	0	1.3	0.01	0	0.41	0.01	0
386454	'Rnf39'	0	0.03	0.44	0	1.28	0	0.04	0.79	1.99	0
386612	'Thoc6'	3.38	13.17	3.45	15.9	26.2	21.85	9.83	17.46	17.32	15.66
386649	'Nsfl1c'	176.5	163.6	184	259	150	196.3	169	155.8	181	185.48
386655	'Eid2'	6.05	2.85	6.44	7.51	3.59	1.85	1.07	10.3	7.6	4.84
386750	'Slitrk3'	13.57	6.11	11.3	6.75	5.98	2.56	6.59	1.98	8.55	0.89
386753	'Dbpht2'	41.21	72.72	34.4	107	67.7	14.74	29.3	23.38	15.07	27.99
387285	'Hcrtr2'	2.34	7.42	3.64	4.32	2.67	2.27	4.34	4.12	14.95	2.14
387314	'Tmtc1'	3.58	3	1.49	1.21	3.43	0.19	1.85	3.39	0.64	2.61
387510	'Ifnk'	0	0	0	0	0	0	0	0.1	0	0
387524	'Znrf2'	0.02	2.1	2.01	0.09	0.02	0.71	1.35	1.05	0	0.36
387586	'Ssxb5'	0.08	0	0	0	0.43	0	0	0	0	0.13

387609	'Zhx2'	0.37	1.76	1.72	2.97	2.42	0.27	2.78	0.4	1.17	0.48
394436	'Ugt1a1'	0	0	0	0.47	0	0	0	0	0	0
396184	'Flrt1'	0.56	4.75	4.59	2.71	6.03	4.92	2.24	1.12	3.15	4.18
399510	'Map4k5'	0.71	4.58	0.06	0.64	1.61	1.06	4.39	3.1	1.66	0.06
399548	'Scn4b'	0	0	2.88	0.01	2.55	0.02	0.01	3.2	1.75	0.35
399558	'Flrt2'	1.81	0.69	2.65	2.58	0	1.54	0	3	0.06	1.18
399566	'Btbd6'	4.86	12.36	25.3	6.62	10.2	26.26	1.82	33.9	19.24	18.37
399568	'BC052040'	5.96	0	4.79	3.96	6.48	5.84	0.68	6.41	3.87	7.29
399591	'Tmsb15l'	5.44	3.84	4	9.87	11.4	1.11	3.36	5.87	0.46	4.92
399599	'Ccdc87'	0	0	0	0	0	2.35	0	1.11	0	0
399603	'Fam84b'	0.76	1.14	0	0	2.5	0.38	0	0.03	0	3.71
403174	'Msantd1'	4.78	3.63	5.65	18.1	0.02	7.16	9.21	4.07	5.65	5.26
403175	'Tigd4'	0	0.01	0	0	0	0	0.01	0	0	0
403178	'Plcxd1'	12.69	8.1	5.56	0	42	28.94	10.4	12.31	24.6	21.43
403180	'Ccdc121'	0	0	0	0	0	0	0	0.02	0	5.82
403183	'Mettl21e'	0.01	0	0	0	0	0	0	0	0.01	0
403185	'Cfap97d2'	0	2.01	0	0	13.5	0	0.83	3.16	5.32	6.41
403187	'Opa3'	19.91	20.76	17.9	18.4	33	36.78	17.5	28.32	25.47	20.74
403205	'Agr3'	0	0	0	0	0	0	0	3.62	0	0
404290	'Vmn1r93'	0	0	0	0	0	2.64	0	0	0	0
404312	'Olfr250'	0	0	0	0	0	0	0	0	0	1.56
404545	'Ano7'	5.43	3.19	0.01	0	1.92	9.35	3.19	3.32	2.2	3.2
404634	'H2afy2'	23.83	17.91	19.4	15.4	20.3	22.31	18.4	16.44	19.67	6.27
406217	'Bex4'	69.66	111.4	91.6	155	79.6	117.1	103	76.45	92.32	76.2
406218	'Panx2'	3.7	6.3	4.82	0	7.14	5.56	6.83	3.07	12.37	8.18
406219	'Krt87'	0	0	0	0	0	0	0	0	4.67	0
406220	'Krt77'	9.44	6.13	11.2	1.24	2.4	3.17	0	0.53	0	0
406221	'Krt40'	0	0	0	0	0	0	0.48	0	0	0
407243	'Tmem189'	2.33	0.51	0.77	11.5	2.25	1.25	2.05	0	3.59	2.68
407785	'Ndufs6'	311.6	294.3	349	390	274	381.2	289	252.7	418.8	265.86
407786	'Taf9b'	33.99	51.58	37.7	14	25.6	23.75	43	33.42	30.16	50
407788	'BC051142'	1.62	1.44	3.8	0.13	2.53	0	0	0.2	2.51	7.93
407800	'Ecm2'	0	0	0	0	0.01	0	0	0	0	0.01
407812	'Zfp941'	24.48	26.6	28.1	9.4	40.9	24.95	24.6	18.85	32.5	42.63
407814	'BC053393'	0.04	0	0	0	0	0	0.02	0.1	0	0
407819	'BC031181'	272.2	252.5	221	305	246	273.2	287	321.3	232.6	287.11
407821	'Znrf3'	5.77	4	1.99	8.45	3.87	4.55	3.07	6.08	6.57	3.75
407823	'Baz2b'	2.16	6.62	2.82	6.43	2.8	4.78	2.31	3.73	7.46	1.81
407831	'Tmem204'	0	1.93	0	0	0	3.23	0	0.17	0	0
408022	'Primpol'	1.43	0.98	4.45	0	0.43	0.12	5.84	0.51	0.98	0.31
408059	'BC049352'	0	5.32	0.06	0	0	0	0	0	0	0
408062	'Zfp873'	10.08	1.7	0.39	0	4.04	0	13.3	2.5	11.85	4.49
408065	'Zfp456'	0	0	0.25	0	0.15	0	0	0	0.26	0.04
408067	'Zfp874b'	1.75	0.89	1.4	0.78	0.11	1.98	0.9	1.04	4.01	0.01
408068	'Zfp738'	3.63	2.74	6.68	0.39	6.9	2.36	3.02	2.38	2.45	2.95
408191	'Gm5415'	0.93	0.06	0.19	0.24	0.36	1.44	0.29	0.06	0.35	0.2
414069	'BC024978'	2.03	0.12	3.74	0.05	7.96	4	0	0	0.02	1.4
414077	'Wdr83os'	107.4	129.7	72.9	140	106	167.2	76.6	145.8	97.28	104.42
414758	'Zfp950'	16.2	17.81	11.3	35.4	20.3	34.74	22.3	16.92	19.73	12.37
414801	'Itprip'	0	0	2.72	0	0	0	0	0	0	0.02
414872	'Zyg11b'	4.68	3.58	8.59	6.1	5.73	3.13	5.87	3.54	8.68	3.65
415115	'Neurl2'	13.94	5.21	6.98	0.06	14.6	7.49	7.15	6.49	5.25	3.87
431706	'Zfp457'	0.02	0.05	0	0	0.19	0.04	0.02	0	0.12	0.05
432442	'Akap7'	8.4	11.31	6.95	35.8	12.3	11.21	4.47	14.24	5.08	21.75
432450	'Nkain2'	2.27	1.21	1.8	9.51	9.1	0.04	4.95	2.53	0.01	0.45
432467	'Hnrnph3'	83.88	84.59	66.2	39.3	91.5	79.85	93.6	92.03	127.2	97.04
432478	'Tmprss9'	0.19	0	0	0	0	0	0	0	0	0
432479	'4930404N11Rik'	0	0.04	0	0	0	0	0	0	0	0.24

432486	'Gnptab'	7.25	1.89	2.54	2.89	2.65	0.17	0.02	1.91	8.35	3.64
432508	'Cpsf6'	5.42	13.58	8.48	11.1	11.4	4.66	9.75	5.44	7.46	9.83
432516	'Myo1a'	0.08	0	0	0	0	0	0	0	0	0
432530	'Adcy1'	0.58	1.89	1.72	0.39	0.5	0.26	1.21	0.67	0	0.03
432552	'Fam71b'	0.06	0	0	0	0	0	0	0	0	0
432555	'Gm5431'	0.23	0.13	0.08	0.28	0.13	0.31	0.02	0.01	0.59	0.06
432572	'Specc1'	12.63	6.22	2.63	3.41	7.78	3.41	6.52	5.97	4.03	5.33
432582	'Ccdc92b'	0	1.24	0.79	0	4.1	0	0	3.1	2.66	0
432611	'Dnaic2'	2.94	2.47	2.91	4.9	1.19	8.15	0	0.71	0.93	0.35
432628	'Mfsd2b'	0	0	0.44	0	0	0.01	0	0.71	1.94	1
432677	'Vrtn'	0	0	0	0	2.79	0	0	0	0	0
432731	'Zscan26'	79.86	56.61	47.1	79.3	84.6	58.36	48.6	70.32	93.19	66.16
432763	'Prr7'	4.46	5.8	4.18	11.6	0.33	3.18	1.61	3.03	3.38	2.78
432769	'Zfp708'	0.07	2.99	1.27	6.13	1.85	0	0	0.35	7.94	0.11
432770	'Rslcan18'	0	0	0.02	0	0	0	0	0	0	0
432779	'Lrrc14b'	0.63	0.26	2.92	0.21	0.16	0.02	0.04	1.01	0.55	0.08
432825	'Gm5458'	0.21	0	0	0	0	0	0.08	0.06	0.07	0
432839	'Gprin2'	0.14	0.34	1.85	0	0	1.27	0.46	0.21	1.47	2.41
432860	'B020004C17Rik'	0	0	0.02	0	0	0	0	0	0.04	0
432940	'Otulin'	0	1.02	2.76	5.54	0.07	5.38	0	0	3.79	1.49
432964	'K230010J24Rik'	0	0	0	0	0	0	0	0.23	0	0
432995	'Smim22'	0.14	0.58	1.94	0	17.4	36.62	0	1.19	24.66	28.63
433022	'Plcxd2'	2.12	1.94	5.27	0.03	1.25	1.61	5.37	2.25	1.33	4.6
433091	'Pnpla1'	0.6	0.04	0.29	0.45	0.09	0.1	0.06	0.05	0.08	0.12
433099	'Ly6g6f'	0	0	0	2.37	0	0	0	0	0	0
433182	'Eno1b'	0.06	0.16	0.06	0	0	0.13	0	0.05	0.24	0.15
433215	'Tmem262'	0	0	0.38	0.36	0	0	0	0.08	0	0
433256	'Acsl5'	15.45	18.93	22.2	11.9	8.57	28.4	7.35	21.73	32.63	31.25
433287	'Gm15455'	0	0.32	0.02	0	0	0	0	0	0.61	0
433292	'Nms'	87.63	0	0	0	0	0	6.27	0	0	6.53
433294	'Mettl21c'	0.04	0	0	0.05	0.33	0	0	0	0.34	0
433323	'Sgpp2'	5.34	3.74	6.24	2.4	16	2.18	8.92	3.54	11.33	1.68
433365	'Teddm1b'	0.58	2.1	0.75	1.6	0.42	0.68	0.48	0.41	0.87	0.45
433375	'Creg1'	48.55	60.2	41.9	20.5	60	111.6	56.1	58.25	68.89	74.44
433415	'Gm13420'	0	0.77	2.25	0.84	0	0	2.69	0.06	3.34	0
433416	'Gm13547'	0	0	0.46	0	0	0	0	0.09	0	0
433466	'Jmjd7'	7.17	12.92	7.92	8.1	3.87	12.11	29.5	7.7	4.01	16.6
433470	'AA467197'	0	0	2.83	0	0	0	0	0	3.65	0
433485	'Syndig1'	33.64	53.34	24.7	18.2	18	16.6	13.3	71.07	22.53	14.25
433586	'Maml3'	5.44	1.69	0.44	7.13	6.48	0.01	4.18	0.83	3.14	0.33
433638	'I830077J02Rik'	0	0.04	0	0	0	3.51	0	0	0	0
433653	'Gimd1'	0.01	0	0	0.01	0	0	0	0	0	0
433667	'Ankrd13c'	3.47	1.64	6.09	0.77	2.03	0.31	7.11	3.04	4.21	1.63
433693	'Akirin2'	13.02	6.45	3.33	4.8	8.43	1.98	4.91	2.56	6.97	2.77
433698	'Fam205a1'	1.21	0.13	0.42	0.25	0.78	0.09	0.83	0.17	0.07	1.39
433700	'Spag8'	0	3.93	0.83	0	0	5.34	0	0	0	0
433702	'Ncbp1'	29.81	19.43	19.4	27.7	20.7	3.44	9.07	11.25	13.68	21.45
433752	'Frg2f1'	7.69	2.25	3.66	0	2.61	1.66	5.37	5.1	7.87	6.27
433759	'Hdac1'	19.8	32.73	30.6	29	12.7	25.32	23.5	22.56	17.27	28.82
433766	'Trim63'	0.02	0	0.1	0	0.03	0	0	0	0.02	0
433771	'Minos1'	107.9	95.88	85	170	81.9	96.35	77.1	88.13	57.28	78.74
433791	'Zfp992'	0.03	0	0.03	0	0.4	0	0.01	0	1.15	0.43
433801	'Gm13212'	0.2	0	0.08	0.37	0.02	0.01	0	0	0	0.03
433804	'Zfp985'	0.01	0.02	0	0	0	0.12	0	0	0.01	0
433809	'Rnf207'	1.62	2.59	1.7	2.09	3.92	3.66	7.88	7.9	0.72	2.11
433813	'Pusl1'	20.15	30.29	25.7	29.9	33.3	28.09	25.7	37.33	13.33	24.6
433864	'Nom1'	14.36	10.14	13.7	11.9	14.8	9.59	15.9	7.8	18.42	18.68
433868	'3110082J24Rik'	0	0	0	0	0	2.48	0	0.89	0	1.12

433904	'Ociad2'	23.01	31.82	21.4	26.8	31.5	30.44	40.2	34.99	32.74	43.09
433926	'Lrrc8b'	0.47	0.95	0.23	0.01	0.6	0.03	0.09	0	0	0.05
433931	'Pigg'	6.07	4.04	1.66	0	4.41	1.36	8.08	4.29	6.01	5.48
433938	'Mn1'	5.25	3.62	1.4	15.8	8.75	0.01	1.93	6.52	5.51	1.88
433940	'Fam222a'	1.4	1.72	0	0.06	2.63	0	0.39	0	0	0.14
433956	'Dnaaf5'	2.1	1.99	0.04	3.49	2.3	0	0.48	0	0.06	0
434008	'Tmem178b'	2.76	5.44	3.66	0.64	2.92	1.55	2.74	2.25	0.25	0.95
434113	'Vmn2r44'	0	0	0	0	0.02	0	0	0	0	0
434117	'Vmn2r50'	0	0	0	0	0.01	0	0	0	0	0
434128	'Pnmal2'	155.8	156.3	171	146	185	127.8	130	141.4	153.8	162.1
434130	'Ccdc8'	0	0	0	0	0	0	0	2.42	2.15	0.02
434156	'Eid2b'	13.55	21.43	15.3	13.5	13.1	9.12	14.9	3.95	19.87	4.58
434178	'Zfp141'	3.49	5.22	5.74	7.69	2.33	1.52	1.94	3.65	8.67	4.21
434179	'Zfp975'	4.4	1.45	3.62	0	3.05	0.02	0	3.87	0.02	8.43
434197	'Fam169b'	0.17	0.4	0.27	0.41	0.36	0.18	0.39	0.12	0.16	0.26
434204	'Whamm'	2.98	1.36	4.21	13.4	0.72	6.21	0.92	0.94	4.8	3.1
434215	'Lrrc32'	0	0	0	0	0	0	0	0	3.77	0
434218	'Trim34b'	0	0	0	0	0	0	0.03	0	0	0
434223	'Gm1966'	0	0	0	0	0.03	0.18	0	0	0.1	0
434232	'Iqck'	4.09	1.93	8.36	0.52	2.66	0	1.22	5.67	5.05	5.15
434233	'Ppp1ccb'	0	0	0	0.09	0.8	4.58	1.45	0.07	0.15	0
434234	'Rexo5'	1.9	3.97	0.05	0	2.7	1.35	0.15	1.04	1.59	0.05
434246	'Trim72'	0	0.1	0.14	0.07	0	0.31	1.61	0.02	0.17	0.57
434341	'Nlrc5'	0	0	0	3.4	0.1	0	0	0	0	0
434377	'Zfp560'	3.28	2.55	3.66	2.81	4.2	2.72	3.15	2.05	3.71	3.13
434402	'Gm5617'	11.13	14.56	11.2	21.6	16.6	35.99	17.7	11.81	15.22	22.85
434423	'Dppa5a'	2.84	4.76	6.39	0	2.31	0.08	0	2.37	11.07	11.63
434436	'Lsmem2'	0	0	0.04	0	0	0	0	0	0	0
434437	'Amt'	48.73	61.47	23.2	35	58.6	40.52	33.6	24.88	26.83	39.37
434438	'Ccdc36'	1.76	7.18	1.87	12.9	0.02	0.38	0.33	0.17	0.03	0.95
434484	'Sp140'	0	0	0	0	0	0	0	0	0	0.07
434689	'Gm10220'	0.6	1.26	1.28	0.02	3.34	0	0	0	0.97	0.1
434756	'Akap14'	0.07	0.54	0	0	0	0	0	0	0	0
434768	'Rhox8'	0.3	0.42	1.22	1.03	0.06	0.55	0.72	0.14	1.46	0.07
434778	'Ccdc160'	11.03	10.41	5.92	17.9	8.56	4.78	4.02	11.77	9.42	24.69
434784	'Ldoc1'	0	3.08	6.83	0	3	0	10.9	0	0	0
434794	'Xlr4a'	0.04	0.05	1.56	0	0	0	0	0	0	0
434797	'Gm5640'	0	0.05	0	0	0	0	7.73	0	0	2.81
434800	'Smim9'	0	0	0	0	0	0	0	0.37	0	0
435145	'Shisa8'	0	0	0.03	0	0	0	0	0	0	0
435337	'Gm5662'	0	0	0	0	0.95	0	0	0	0	0
435376	'Atp6ap1l'	0	6.51	10.6	0	0	0	0	0	0.1	0
435391	'Dupd1'	0	0	0	0.07	0	0	0	0	0	0
435529	'Adgrf2'	0	0	0	0	0	0	2.05	0	1.19	0
435653	'Fcrlb'	3.64	2.5	3.18	0	0.2	7.37	6.17	0	0.19	3.66
435684	'Shf'	0.34	0.49	0	0	0.1	0	1.57	0	0	1.83
435766	'Tnni3k'	0	0	0.04	0	0	0.01	4.07	0.02	0	0.04
435811	'Ldlrad2'	0.65	2.8	0.29	7.17	0.33	0.04	0.08	2.3	0.15	3.17
435965	'Lrp3'	1.76	3.71	1.96	0	14.5	6.8	4.18	2.42	2.83	7.63
436008	'Gm5737'	0.49	0.34	0.38	0.02	0	0	0	0	0	1.41
436022	'Dnaaf3'	0	0	2.58	0	0.52	3.19	0	0	0	0
436062	'Fam92b'	16.14	6.89	4.55	25.1	18.7	0	0.06	5.39	3.11	6.54
436090	'Gpr62'	13.38	7.16	16.5	11.7	0.15	10.03	8.08	9.24	7.29	7.15
436240	'Foxr2'	1.47	2.55	6.64	0.02	0.87	1.51	5.09	1.74	12.82	2.51
436336	'Gm5767'	0	0	0	0	0	0	0	0.99	0	0
436440	'Gpr31b'	0	0	0	0	0	0	0	0	0	0.05
442801	'Arhgef15'	7.7	8.53	14.9	9.39	5.2	15.42	7.62	14.23	12.53	13.09
442829	'Ccin'	0	0	0	0	0	0.02	0	0	0	0

442834	'D830031N03Rik'	0.18	0.16	0.11	0	0.59	0	1.78	0	0.28	0
445007	'Nup85'	31.27	33.48	39	44.5	36.5	43.93	41.1	27.29	34.75	44.95
446099	'Nlrp4e'	0	0	0	0	0	0	0	0	0.58	0
446101	'Xrra1'	2.91	0.03	0.81	0.12	0	0.09	7.95	0	1.55	1.33
448850	'Znhit3'	70.24	77.38	93.7	95.6	79.5	111.8	65	53.71	86.02	87.34
448987	'Fbxl7'	0	2.26	0	0	0	0	0	0	0	0.03
449000	'Zfp960'	8.24	4.28	1.55	0	1.13	0.34	0.96	3.9	2.74	2.93
449521	'Zfp213'	7.14	8.67	6.97	0	2.93	3.13	14.8	7.45	4.66	8.87
450219	'Gsdma3'	0	0	0.22	0	0	0	0	0	0	0
474156	'Zbtb9'	0	1.28	5.02	0	2.49	11.39	0	0	3.83	1.88
494448	'Cbx6'	10.51	15.3	8.96	6.68	12.7	10.08	22.1	10.57	14.09	18.14
494468	'Armcx5'	11.18	32.11	23.4	56.4	26.1	9.85	26.3	15.97	12.22	11.54
497097	'Xkr4'	1.82	2.45	1.79	4.4	1.56	1.66	3.11	1.73	0.02	1.93
497114	'Defa23'	0	0	0	0	0	0	0.55	0	0	0
497652	'Acd'	68.95	62.63	59.5	65.3	48.2	130.7	31.3	53.17	40.37	50.3
503491	'Defa24'	0	0	0	0	0	0	43.5	0	0	0
503610	'Zdhhc18'	1.03	1.49	3.7	8.71	5.28	2.71	0.2	3.86	4.35	0.04
503692	'Aym1'	0	0	0	0	0.82	0	0	0	0.82	0
504186	'Chrna10'	0	0	0	0	0	0	0	0	0.02	0
504193	'Npcd'	13.63	17.05	8.95	27.6	7.07	6.74	17.8	12.22	4.81	8.86
50490	'Nox4'	2.63	0	0.73	0	0.18	0.49	0	0	0	0
50492	'Thop1'	29.99	35.53	39	0.02	15.9	21.53	24.8	22.09	20.97	32.42
50493	'Txnrd1'	11.14	8.36	19	18.7	8.11	5.36	16.7	10.62	22.83	7.61
50496	'E2f6'	12.97	4.23	0.04	3.85	19.1	9.14	4.89	9.42	29.98	9.63
50497	'Hspa14'	20.09	14.99	31.9	26.3	37.4	51.48	25.6	43.03	43.85	34.5
50498	'Ebi3'	1.53	1.04	0.29	7.1	2.07	3.17	0	0	0	0
50500	'Ttpa'	0.59	2.14	0.52	0	0.02	0	2.76	1.26	2.03	0
50501	'Prok2'	10.15	17.27	5.35	15.9	0	0	1.19	11.25	1.89	0
50505	'Ercc4'	5.22	8.53	4.84	2.64	3.42	4.93	5.64	8.77	11.18	10.71
50518	'a'	0.11	0.07	0.03	0.02	0.02	0.01	0.11	0.68	0.96	0.07
50523	'Lats2'	0.38	0	0.59	0	0	0	0	0.2	0	0
50524	'Sall2'	8.08	8.78	13.8	2.74	6.6	5.36	3.86	4.98	5.13	3.09
50525	'Spag6l'	6.31	4.29	4.62	14.7	3.14	0.04	0.04	4.66	6.8	7.1
50527	'Ero1l'	16.53	8.48	14	8.83	7.75	8.47	8.47	7.9	10.91	6.19
50529	'Mrps7'	51.39	41.91	45.4	58.9	73.1	52.21	58.4	72.26	48.2	67.19
50530	'Mfap5'	0	0	0	0	1.99	0	0	0	0	0
50540	'Igbp1b'	2.68	4.94	0	6.73	0	0	0	3.16	0	0
50702	'Cfhr1'	0	0.03	0	0	0	0	0	0	0	0
50706	'Postn'	0	0	0	0	0	0	0	0	7.12	0
50708	'Hist1h1c'	0.96	3.03	6.76	0	9.43	3.88	3.38	2.48	2.34	5.54
50709	'Hist1h1e'	8.93	8.16	11	0	4.33	19.6	0	7.88	11.8	15.06
50720	'Sacs'	2.25	2.34	1.2	2.81	4.5	1.46	0.81	1.65	1.65	1.82
50721	'Sirt6'	14.25	15.69	28.2	35.2	22.7	19.77	28	17.79	24.92	20.98
50722	'Dkkl1'	3.38	7.53	1.89	0	0	0	0	5.16	21.63	3.07
50723	'Icosl'	0	1.04	0	0	0	0.05	0	0	0	0.38
50724	'Sap30l'	1.04	1.66	7.47	3.72	4.6	0.06	0	0.65	10.12	2.12
50753	'Fbxo8'	16.98	6.19	7.43	13.6	8.84	18.61	17.8	8.22	11.62	14.62
50754	'Fbxw7'	13.6	24.41	16.1	11.6	20.9	19.13	14.7	16.04	12.69	9.63
50755	'Fbh1'	32.05	22.06	32.8	39.4	26.9	6.54	31.2	30.41	15.39	28.77
50758	'Fbxl17'	1.6	2.7	2.27	3.49	2.32	0.57	1.36	1.78	1.34	1.89
50759	'Fbxo16'	2.34	9.14	15.6	14.4	23	33.04	10.4	13.63	6.44	12.02
50760	'Fbxo17'	6.11	0.18	6.71	9.54	1.88	7.11	4.16	0	5.88	12.92
50762	'Fbxo6'	13.55	48.04	17.9	6.12	49.5	35.58	65.1	33.48	27.82	32.59
50764	'Fbxo15'	0.05	0	0	0	0	0	0	0	0	0
50765	'Tfr2'	5.63	7.16	5.88	6.15	4.11	14.56	11.1	9.43	8.52	4.11
50766	'Crim1'	8.11	19.48	3.21	36.1	6.74	4.08	14.8	16.7	5.94	5.38
50767	'Pnpla6'	13.82	21.63	13.1	8.66	12	5.42	21.6	16.06	37.44	23.35
50768	'Dlc1'	2.33	0.35	3.23	2.47	1.28	0.87	2.36	2.28	2.62	1.03

50769	'Atp8a2'	15.24	21.31	12.5	3.02	15.3	10.21	26.6	13.66	17.28	18.91
50770	'Atp11a'	2.22	3.07	1.17	4.9	5.45	3.38	3.61	1.25	6.12	2.55
50771	'Atp9b'	5.48	3.49	19.7	0.11	14	3.98	15.3	8.38	14.86	11.55
50772	'Mapk6'	6.3	11.72	12.3	15.3	5.27	6.27	27.2	10.1	14.4	16.94
50773	'Nt5c'	103.5	99.81	79.6	53.2	175	171.5	92.5	102.7	73.15	122.19
50776	'Polg2'	2.88	4.4	0.74	1.24	1.36	10.67	5.49	1.49	0.61	4.89
50778	'Rgs1'	0	1.77	0	1.22	0	25.49	0	0	0	0
50779	'Rgs6'	0.57	0	0.13	1.26	2.29	3.6	0	0	3.25	0
50780	'Rgs3'	12.04	20.89	21.1	12.3	8.67	18.14	7.11	9.4	16.11	18.71
50781	'Dkk3'	5.06	13.73	13	2.99	6.62	1.73	5.43	17.81	26.06	26.58
50782	'Rgs11'	31.86	24.28	21.1	7.41	42	26.47	9.84	41.78	11.7	17.71
50783	'Lsm4'	98.77	55.26	93.3	266	78.9	24.11	52.9	50.01	84.76	26.9
50784	'Plpp2'	17.29	13.51	21.8	30.1	26.3	8.16	15	20.3	3.69	20.23
50785	'Hs6st1'	1.6	0.6	2.81	0.23	2.25	0	1.9	1.33	0.18	1.82
50786	'Hs6st2'	9.22	18.44	30.6	18.7	19.8	3.27	25.9	18.8	25.39	19.46
50787	'Hs6st3'	0	1.27	0.37	0	0.33	0	4.54	1.04	0	0
50788	'Fbxl8'	1.01	0	3.23	0	2.57	6.67	0	0.71	0	0
50789	'Fbxl3'	5.5	4.59	1.77	0.21	4.71	2.81	3.47	0.94	3.06	2.08
50790	'Acsl4'	9.56	7.96	8.74	5.86	7.29	4.95	3.22	10.55	14.43	7.98
50791	'Magi2'	2.56	3.98	4.01	9.12	8.77	5.54	8.18	4.85	4.2	5.23
50793	'Orc3'	34.92	33.58	23.2	3.21	11.5	26.67	25.6	24.96	18.25	17.41
50794	'Klf13'	19.24	23.85	12.8	37.8	31.3	21.07	20.4	29.3	14.82	19.07
50795	'Sh3bgr'	0.65	1.81	0.31	0	0	0.1	5.93	1.17	0.04	0.09
50797	'Copb2'	27.18	24.52	49.4	26.9	40.6	19.8	44.3	30.99	46.52	45.77
50798	'Gne'	14.49	12.25	13.3	10.9	16.3	12.05	13.3	26.05	28.87	13.51
50799	'Slc25a13'	2.5	0.17	1.95	0.76	5.63	0	0.08	0.04	0.03	2.74
50817	'Capn15'	4.01	4.52	8.26	5.28	0.84	5.02	8.18	0.65	6.96	5.93
50849	'Rnf10'	11.2	19.94	21.8	16.2	12	17.39	13.3	10.97	7.9	2.95
50850	'Spast'	0.89	0.54	2.24	5.57	3.3	2.23	1.83	3.08	1.3	4.27
50868	'Keap1'	29.47	30.99	33.8	22.5	42.4	7.24	16.4	36	27.04	39.43
50873	'Prkn'	23.32	28.31	33.8	48.3	41.9	17.95	17.3	14.99	31.23	20.12
50874	'Tmod4'	0.13	0.27	0.16	0.12	0.1	0.09	0.07	1.19	0.05	0.07
50875	'Tmod3'	13.06	9.67	7.73	7.61	7.62	8.14	6.83	9.76	16.98	3.05
50876	'Tmod2'	22.08	22.09	17.7	17.4	19.4	10.7	21	18.97	17.29	13.58
50877	'Neu3'	2.45	2.29	4.52	4.4	5.44	2.31	4.21	3.41	2.56	2.1
50878	'Stag3'	0	0	1.46	0.92	0	0	0	1.17	0	0
50880	'Scly'	5.18	6.76	5.91	2.99	0.83	6.93	1.43	2.7	4.33	3.7
50883	'Chek2'	2	2.95	0.21	3.79	0.09	0.25	0.21	0.05	0.07	0.13
50884	'Nckap1'	9.94	20.26	12.8	25.3	17.2	11.9	15.6	11.62	11.27	14.12
50887	'Hmgn5'	3.31	3.34	4.68	6.54	1.17	3.22	4.35	1.43	4.26	4.55
50905	'Il17rb'	0	2.12	2.26	0	0	0	0	0	0	0.02
50907	'Preb'	30.45	31.65	29.2	29.6	25.5	38.19	41.3	25	30.86	24.52
50908	'C1s1'	0	0	0	0	0	0	0	0	0	0.63
50909	'C1ra'	0	0	0	0	0	0	0	0.01	0	0
50911	'Exosc9'	41.74	31.29	40.5	44.3	41	55.36	34.4	39.71	68.11	37.48
50912	'Exosc10'	33.27	26.09	31.9	38.8	42.4	20.92	22.2	24.5	19.37	12.52
50913	'Olig2'	0.52	0	8.06	4.84	0	0	1.98	0.18	0	0
50914	'Olig1'	34.59	28.07	163	29.7	16.2	48.15	256	52.04	7.26	2.37
50915	'Grb14'	13.66	13.89	4.26	0.03	31	27.13	32.1	14.86	37.02	10.57
50916	'Irx4'	0	0	0	0	0	0.02	0	0	0	0
50917	'Galns'	12.5	10.78	21	0	10.9	30.57	6.54	11.27	19.02	10.06
50918	'Myadm'	29.95	19.76	20.9	15.9	32.4	31.69	13.3	21.34	26.61	26.3
50926	'Hnrnpdl'	42.22	41.96	50.4	45.6	49.9	35.67	54.7	38.33	40.19	22.81
50927	'Nasp'	8.06	6.31	8.08	1.09	3.14	8.06	4.23	1.53	4.22	0.3
50928	'Klrg1'	0	0	0	0	3.48	0	0	0	7.14	0
50932	'Mink1'	1.65	2.87	0.81	1.02	2.84	1.7	2.74	3.13	1.94	1.52
50933	'Uchl3'	106.1	129.2	70.1	89.2	87.1	108.7	74.5	123	107.5	94.15
50934	'Slc7a8'	8.96	5.64	7.71	1.49	1.51	3.66	3.91	3.36	17.53	11.68

50935	'St6galnac6'	8.75	16.19	2.91	5.62	19.5	7.8	10.2	20.87	17.31	2.32
50995	'Uba2'	73.92	95.91	48.2	85	54.7	52.92	107	53.12	64.28	94.82
50996	'Pdcd7'	11.66	1.13	0.68	3.46	4.79	4.83	3.02	5.2	3.08	4.47
50997	'Mpp2'	9.54	11.54	7.7	9.81	12.4	8.86	5.73	6.85	24.1	10.46
51786	'Cpsf2'	4.45	6.35	12.4	12.6	9.2	2.51	1.74	5.84	6.79	12.48
51788	'H2afz'	394.7	358.2	318	437	479	551.1	495	458.3	300.4	372.5
51789	'Tnk2'	5.38	5.98	12.1	2.55	9.26	7.53	17.4	5.3	8.66	11.81
51791	'Rgs14'	9.61	8.76	7.2	9.25	9.32	10.68	11.9	21.09	9.67	8.52
51792	'Ppp2r1a'	500.9	460.7	555	350	404	505.2	552	567.7	471.1	565.93
51793	'Ddah2'	6.39	9.13	20.7	0.04	5.23	17.19	23.2	5.06	0	8.47
51795	'Srpx'	0	0	0	0	0	0	0	0	0	0.05
51796	'Srrm1'	24.68	28.38	32	54.8	32.3	31.58	28.9	27.37	34	32.52
51797	'Ctps'	32.47	21.37	28.9	36.7	14.5	23.24	18	40.3	24.19	17.66
51798	'Ech1'	93.42	70.07	80.6	184	124	95.23	54.5	50.36	60.81	55.44
51799	'Rundc3a'	17.14	16.57	9.86	36.3	37.7	12.17	21.8	30.72	26.05	19.98
51800	'Bok'	20.25	31.93	18.1	1.7	24.6	43.47	46.5	13.92	12.37	15.96
51801	'Ramp1'	47.67	18.69	29.2	22.1	14.7	37.44	30.3	41.15	16.77	14.84
51810	'Hnrnpu'	29.23	26.38	17.6	88.7	40.1	21.14	17	14.66	29.52	22.29
51812	'Mcrs1'	36.1	41.19	41.6	49.4	31.9	31.87	40.6	49.4	22.9	16.66
51813	'Ccnc'	38.09	42.81	49.3	33	18	61.07	29.3	32.5	20.59	27.4
51869	'Rif1'	1.47	0.76	0.19	4.87	1.83	1.19	1.58	1.63	0.5	2.07
51875	'Tmem141'	42.83	48.14	39.2	24.3	31.3	40.8	49.8	50.82	33.39	32.93
51885	'Tubgcp4'	9.42	13.04	6.29	0.94	3.65	7.02	7.75	6.42	15.98	9.94
51886	'Fubp1'	25.37	40.28	19.2	24.2	54.2	27.73	53.2	44.41	28.45	35.36
51897	'Atg13'	41.82	49.83	34.5	18.2	22.1	44.52	16.8	34.49	51.99	40.04
51902	'Rnf24'	5.13	0.37	3.02	4	0.72	4.66	4.72	4.09	5.93	2.54
51938	'Ccdc39'	10.88	12.31	6.74	11.1	16.9	6.4	9.17	8.58	5.53	8.96
51944	'Knstrn'	0	0	0.45	0	0.34	0	0	0	0	0
51960	'Kctd18'	3.26	2.98	0.37	0.75	1.01	2.07	5.39	6.71	10.26	3.75
52004	'Cdk2ap2'	73.02	121.8	52	141	93.3	116.1	48.4	77.86	52.68	127.75
52009	'Jpt2'	43.83	47.09	12.5	39.4	26.7	16.19	6.97	9.75	43.36	34.55
52013	'R3hcc1l'	4.14	2.44	5.45	5.16	1.07	5.62	3.83	8.43	8.4	6.42
52014	'Nus1'	5.41	6.97	5.75	12.4	0.71	6.49	1.62	4.31	5.2	4.58
52020	'Umodl1'	0.68	0	0	0	0	0	0	0	0	0
52023	'Pibf1'	4.53	6.53	5.54	4.07	4.59	7.72	7.79	5.67	6.22	8.18
52028	'Bbs1'	4.59	2.79	0.99	2.91	2.03	0.57	5.28	3.82	5.06	1.92
52036	'Ppp6r3'	8.35	4.88	7.28	9.67	8.07	3.19	12.5	3.09	8.92	6
52040	'Ppp1r10'	10.88	14.31	17.1	15.5	26.5	17.44	22.5	9.96	14.08	37.12
52055	'Rab11fip5'	3.1	10.57	11	10.8	6.25	8.74	5.17	14.47	12.25	4.23
52064	'Coq5'	45.7	17.76	27.8	42	82.7	21.41	56.3	39.38	42.59	26.4
52065	'Mfhas1'	2.11	0.54	2.53	3.06	4.78	5.56	4.17	1.94	0.65	0.1
52076	'Tmem38b'	1.72	2.12	0.48	0	1.22	1.69	0	1.3	1.25	0.99
52118	'Pvr'	1.9	0.91	6.54	3.77	0	0	0	2.35	0	0.18
52120	'Hgsnat'	15.4	9.1	21.9	22.1	16.1	8.01	32.1	10.97	19.99	5.4
52123	'Agpat5'	6.45	13.29	9.03	12.7	7.34	16.93	21.7	4.8	9.29	7.73
52132	'Ccdc97'	12.91	16.53	12.4	5.91	24.1	9.2	17.3	19.44	23.73	35.37
52150	'Kcnk6'	0.22	0.47	0.05	0.05	0.95	0.62	0.25	0	1.16	1.66
52163	'Camk1'	101	75.64	108	130	114	90.92	65.5	86.46	135	32.38
52174	'Tmem222'	195.5	183.4	151	129	152	212.4	187	162.2	159	218.7
52184	'Odf2l'	2.28	3.4	4.66	12.3	0.23	9.38	6.38	5.1	4.52	8.23
52187	'Rragd'	2.21	5.43	5.37	7.17	4.9	1.97	3.78	3.04	1.01	2.05
52202	'Rbm34'	18.02	9.75	10.6	26	22.8	18.88	37.9	14.76	7.58	20.97
52206	'Anapc4'	56.78	41.57	57.1	52.5	32.7	46.03	36.5	45.49	72.27	55.36
52231	'Ankzf1'	16.47	14.03	12.6	0	9.47	37.32	13.3	9.61	9.99	7.93
52245	'Commd2'	12.22	10.64	6.5	13.2	13	4.62	5.73	15.43	9.33	9.13
52250	'Reep1'	35.59	44.81	63.5	29.5	56.7	20.28	48.8	44.23	64.38	22.9
52276	'Cdca8'	1.23	0	2.57	0	1.1	0	0	0	0	0
52323	'Klhl7'	58.9	75.19	59.7	53.2	45.5	38.39	74	66.69	53.53	76.77

52331	'Stbd1'	5.4	1.12	0	0	0	0	0	5.1	4.76	2.52
52335	'Atxn1l'	0.01	1.14	0.28	0.04	1.01	0.94	0.01	0.1	0.63	0.01
52348	'Vps37a'	3.46	4.47	2.94	8.79	2.23	0.79	2.81	2.06	9.14	3.79
52357	'Wwc2'	0	2.58	0.66	0	3.6	1.56	2.81	1.14	4.79	2.43
52372	'D6Ertd527e'	0.49	1.42	0	3.02	0.11	4.51	0	0.14	0	0
52377	'Rcn3'	3.22	2.93	9.88	2.02	0	0	11.5	0.08	3.69	6.68
52389	'Adgra1'	15.23	23.11	30	24.8	23.6	20.94	25.6	22.4	13.35	10
52392	'D1Ertd622e'	17.12	11.07	4.17	5.05	9.75	4.79	5.04	7.32	9.26	10.63
52397	'Zfp644'	6.08	3.02	5.91	1.99	3.93	2.39	9.12	2.05	1.43	7.2
52398	'Sept11'	28.99	18.58	23.5	49.3	20.5	19.43	17.5	14.1	21.05	9.67
52428	'Rhpn2'	0.56	2.21	0	0	0.06	2.64	0.28	0.03	0	0
52430	'Echdc2'	4.16	9.48	1.77	0	1.28	13.1	10.5	6.28	25.87	13.38
52432	'Ppp2r2d'	5.44	11.74	8.85	35.9	19.9	2.83	2.34	18.29	5.37	18.62
52440	'Tax1bp1'	59.02	62.72	42.6	81.4	61.9	53.22	85.1	63.5	56.49	64.01
52443	'Mrpl48'	114.4	100.2	89.5	107	83.2	126.4	91.1	120.1	66.67	131.17
52463	'Tet1'	2.22	4.02	5.75	5.36	2.31	0.97	2.31	1.26	3.07	0.57
52466	'Slc46a1'	13.69	4.1	12.2	15.6	9.07	24.22	8.66	12.66	15	13.63
52468	'Ctdsp2'	0.01	0.71	4.45	1.91	0.21	0	1.51	0.7	0.49	0.23
52469	'Coa3'	262	406.2	277	463	337	368.9	399	447.4	416.2	415.14
52477	'Angel2'	14.97	18.52	14.5	24.8	12.9	21.38	14.7	15.04	22.86	9.38
52502	'Carhsp1'	18.78	12.88	7.12	3.21	31.4	6.95	8.03	18.57	9.42	9.83
52504	'Cenpo'	2.68	1.67	2.62	6.76	1.63	2.31	4.14	2.97	4.28	7.43
52513	'Ddx56'	45.76	40.82	35.8	64	31.8	48.74	53.1	75.74	55.27	49.81
52521	'Zfp622'	5.89	1.63	3	13.8	2.16	2.81	9.75	3.4	7.02	1.85
52530	'Nhp2'	34.35	16.5	41.5	15.2	56.7	58.15	36.5	51.64	59.65	53.88
52535	'Mettl17'	16.48	25.93	18.4	8.34	14.8	43.12	15.3	13.32	14.39	18.43
52538	'Acaa2'	20.73	22.28	3.12	18	5.65	12.82	13.9	5.36	4.21	7.46
52551	'Sgta'	137.3	156.9	153	201	137	184.1	248	229.6	163.2	234.13
52552	'Parp8'	6.13	17.31	3.37	11.9	1.32	3.05	8.87	7.76	13.05	9.36
52563	'Cdc23'	20.76	21.41	19	16.3	22.4	12.62	11.5	22.65	31.68	22.14
52570	'Ccdc69'	0	0	3.24	0	0	0	0	0	0	0
52575	'Trmt10c'	14.31	12.75	10.2	25.2	2.84	6.54	0.08	10.27	19.14	13.27
52585	'Dhrs1'	52.32	62.69	63.5	52.9	31.5	66.63	78.9	48.54	51.21	47.64
52588	'Tspan14'	2.48	4.03	0	6.09	0	10.47	5.95	17.56	6.66	8.54
52589	'Ncald'	104.3	210.7	129	113	124	88.64	179	116.2	70.57	59.83
52592	'Brms1l'	17.46	18.26	20.5	30.6	33.2	27.91	32.5	33.09	40.15	34.05
52609	'Cbx7'	11.73	2.52	10.1	10.4	18	20.15	22.7	7.69	9.28	1.99
52615	'Suz12'	4.66	0	1.91	5.91	2.5	1.93	0	1.03	0.21	2.66
52626	'Cdkn2aipnl'	46.47	43.83	45.6	19.8	67.1	121.6	68.7	53.94	117.7	55.19
52633	'Nit2'	25.92	26.41	19.3	0.14	13.3	36.33	6.95	34.46	34.63	16.2
52635	'Esyt2'	1.83	1.98	1.01	2.47	2.21	1.95	3.77	1	3.78	3.06
52637	'Cisd1'	331.3	355.1	303	229	303	539.6	269	316.4	321.4	361.07
52639	'Wipi1'	6.12	8.45	1.81	0.57	3.19	2.22	12.9	3.3	8.78	20.44
52653	'Nudcd2'	21.65	18.83	14.9	0	15	10.94	19.9	18.48	18.21	16.76
52662	'Ldlrad4'	3.02	1.96	2.13	0.02	0.69	3.15	3	0.35	18.85	7.63
52665	'Echdc1'	7.43	11.18	7.55	24.2	5.19	8.92	13.2	8.73	6.85	4.12
52666	'Arhgef25'	7.4	3.07	6.09	10.6	8.04	23.97	8.17	8.19	14.3	11.42
52668	'Ifi27'	54.21	89.8	76.8	90	40.2	56.23	89.5	23.67	32.04	83.7
52670	'Cpsf4l'	0.1	0.11	0.07	0	0.22	0	0.08	0	0.09	0.01
52679	'E2f7'	0	0	0.07	3.03	0	0.01	0	0	0	0
52683	'Ncaph2'	36.32	46.44	66.1	57.9	77	71.35	103	87.08	40.56	71.77
52685	'Cd300lg'	0.65	2.83	0.92	0.24	0.36	0.39	0	0.29	0.49	0.92
52686	'Mettl2'	9.81	16.15	22.8	15	11.4	14.3	21.2	17.54	17.7	12.45
52690	'Setd3'	45.07	32.62	44.9	42.8	28.3	32.31	51.8	32.63	19.38	39.73
52696	'Zwint'	2240	2084	1980	2406	1999	3004	2491	2838	2435	2082.48
52700	'Txndc17'	89.55	89.1	76.5	78.3	83	80.11	63.9	69.66	88.83	89.97
52705	'Krr1'	8.52	10.72	5.34	12.1	8.1	10.57	7.65	6.61	4.32	6.18
52708	'Zfp410'	38.2	23.13	30.3	2.12	11.3	6.52	15.3	20.72	25.35	7.89

52710	'Slc52a2'	8.38	4.8	6.95	4.65	8.2	6.82	3.77	2.98	7.38	9.81
52712	'Zkscan6'	14.34	5.03	13.1	6.63	3.68	13.23	6.73	7.75	18.25	12.83
52713	'Ccdc59'	31.83	24.16	22.4	61.3	28.4	36.93	31.4	39.52	39.9	38.02
52715	'Ccdc43'	4.49	10.73	5.07	22.2	17.2	9.58	6.07	11.62	27	11.96
52717	'Anapc16'	44.42	78.84	31.9	27.8	63.7	90.53	69.6	57.3	48.83	88.47
52808	'Tspyl2'	97.12	104	99.8	97.6	89.5	98.78	116	99.55	145.5	93.14
52815	'Ldhd'	11.3	8.79	16.5	0	9.3	0	3.35	15.29	7.46	10.28
52822	'Rufy3'	89.08	96.44	62	94.6	45.4	84.92	86.2	91.32	71.86	73.92
52829	'Lurap1l'	13.99	10.2	0.97	0	21.6	20.95	12	9.63	10.56	6.98
52830	'Pnrc2'	38.19	6.27	15.1	64	45.4	38.89	18.2	29.63	37.09	46.12
52837	'Tmx4'	99.88	72.23	162	103	64.8	55.96	100	84.78	101.7	119.65
52838	'Dnlz'	8.75	8.3	12	6.14	10.3	14.17	10.6	10.1	11.57	6.66
52840	'Dbndd2'	22.63	24.38	6.16	6.83	117	29.2	19.7	9.07	10.08	9.09
52846	'Cnot11'	2.62	0.42	0.08	2.9	4.29	0	0.27	4.52	2.55	0.01
52850	'Sgsm1'	47.8	57.99	50.3	41	39.1	18.78	28.7	46.67	31.7	80.19
52855	'Lair1'	3.65	0.75	0	0.28	3.61	6.28	1.98	0	0	0
52856	'Mtg2'	27.81	14.13	8.05	34.9	10.5	17.57	7.48	22.21	33.51	8.16
52857	'Gramd1a'	22.8	24.22	25.6	17.9	29.7	28.33	12.2	19.15	25.83	11.32
52858	'Cdipt'	125.5	90.29	81.6	78.9	117	124.8	147	104.8	82.08	139.76
52864	'Slx4'	2.5	0.54	2.2	0.01	0.19	0.61	4.85	2.12	4.72	4.68
52874	'Pum3'	18.54	15.33	16.2	15.7	7.63	17.6	10.9	10.55	8.34	20.93
52882	'Rgs7bp'	8.26	4.51	9.51	5.07	9.13	7.48	6.64	10.88	6.93	4.67
52892	'Sco1'	1.61	3.77	4.27	0.73	4.02	9.06	5.54	3.55	7.94	0.39
52897	'Rbfox3'	37.37	15.47	18.6	23.4	23.1	26.51	22.7	18.46	22.78	15.5
52898	'Rnasek'	879.1	1226	784	714	760	903.3	768	754.6	745.6	954.55
52906	'Ahi1'	290.3	261.6	275	228	202	226.4	343	241	328	321.75
52915	'Zmiz2'	7.8	2.81	7.02	2.45	16.3	1.34	4.03	7.23	14.17	1.58
53310	'Dlg3'	0.37	1.5	2.38	2.3	4.09	0	0.27	0.91	1.31	2.84
53312	'Nub1'	27.59	25.63	41.6	43.8	25.6	16.81	30	31.81	43.39	41.15
53313	'Atp2a3'	0	0	0	0	0	0	0.01	0	0	0
53317	'Plrg1'	43.13	27.89	52.2	41.8	34.2	43.72	37.8	72.99	67.49	80.73
53318	'Pdlim3'	0	0.03	1.74	0	0	0	0	0.72	0	0
53319	'Nxf1'	71.61	86.08	69.4	103	54.3	53.02	53	70.54	89.61	87.23
53320	'Folh1'	4.22	2.52	0	2.5	0.4	2.45	4.84	1.16	0	0
53321	'Cntnap1'	5.27	3.49	5.5	1.83	10.1	2.67	15.8	7.94	11.53	8.36
53322	'Nucb2'	34.2	36.94	10.7	73.7	20.6	15.42	37.2	17.66	40.4	48.18
53323	'Ube2k'	20.14	21.1	23.2	32.8	25.8	29.4	22.8	30.07	30.55	27.06
53324	'Nptx2'	0	0.81	2.27	4.06	0	0	0	1.56	3.16	0.53
53325	'Banp'	24.97	29.07	20	6.65	14.5	27.91	4.08	26.52	13.9	13.52
53328	'Pgrmc1'	810.5	1100	679	595	735	753	997	845.9	617.9	812.9
53330	'Vamp4'	36.46	35.17	33.2	54.1	39.3	34.68	24.7	39.67	60.54	53.11
53331	'Stx7'	134	117.7	95.7	117	112	124.7	134	101.7	101.2	88.39
53332	'Mtmr1'	0.02	2.41	0.23	1.92	0.06	1.19	0	1.86	2.03	1.33
53333	'Tomm40'	107.5	84.03	154	104	115	177.7	109	100.1	157.4	56.76
53334	'Gosr1'	24.03	14.43	21.3	15.2	13.7	13.83	22.6	19.59	14.9	32.25
53356	'Eif3g'	188	118.6	152	144	152	169	133	161.8	139.9	144.94
53357	'Pla2g6'	17.04	13.48	5.78	0.96	20.2	10.69	17.1	10.14	29.12	9.94
53374	'Chst3'	0	0.01	1.43	1.15	0.27	0.02	0.01	0	0.01	0.01
53375	'Mtx2'	67.41	73.44	60.4	99.3	54.4	65.9	70.4	47.92	100.3	40.89
53376	'Usp2'	7.21	10.5	22.9	20.1	5.84	17.02	8.13	4.27	6.84	9.27
53378	'Sdcbp'	272.2	424.5	150	341	284	269.5	141	235.5	271.3	293.09
53379	'Hnrnpa2b1'	205.7	253.9	142	133	233	196.6	168	192.2	185.5	210.13
53380	'Psmd10'	53.38	39.98	43.4	35.4	41.7	67.56	35.8	42.11	48.53	56.11
53381	'Prdx4'	30.38	53.86	46.5	46	21.3	59.59	63.7	43.2	55.84	64.33
53382	'Txnl1'	65.16	84.96	92.3	56.9	64.7	68.51	79.3	79	69.88	74.68
53404	'Atoh7'	0	0	3.74	0.77	2.62	4.79	5.47	3.69	1.77	0
53412	'Ppp1r3c'	7.11	2.67	0.11	13.8	0.04	2.59	6.17	4.49	4.28	0
53413	'Exoc7'	42.35	41.24	34.1	49.6	49.3	30.77	72.4	44.91	61.01	61.63

53414	'Bysl'	7.27	7.51	9.84	24.2	7.11	9.53	15.4	14.99	4.98	11.64
53415	'Htatip2'	25.2	20.58	16.1	42.8	1.31	24.09	3.75	25.15	25.01	35.87
53416	'Stk39'	4.98	3.42	5.28	6.35	9.77	3.23	4.06	4.21	2.17	2.16
53417	'Hif3a'	1.18	0	0	0	0	0	0	0.01	0	0
53418	'B4galt2'	2.71	0.06	0	3.54	2.38	0.02	3.56	2.26	2.79	1.76
53419	'Corin'	0.03	3.01	0.02	0	0.01	0.01	0	0	0.01	0
53420	'Syt5'	312.4	424.7	334	274	355	283	177	373	399.6	562.34
53421	'Sec61a1'	13.35	13.07	21.7	14.7	7.64	18.52	11.8	6.69	33.86	17.24
53422	'Ybx2'	0	0	0	0	0	4.11	0	0.56	0	0
53424	'Tsnax'	146.6	137.4	149	150	138	115.6	141	182.2	125.5	188.18
53598	'Dctn3'	376.4	356.4	301	306	280	429.3	317	399.9	364.8	403.53
53599	'Cd164'	28.58	50.25	28.9	14.5	20.5	20.1	17.4	34.55	61.84	61.62
53600	'Timm23'	138.8	202.2	183	204	156	285	206	249.3	182.1	161.16
53602	'Hpcal1'	258.9	309.8	344	816	147	329	164	300.7	326	391.85
53603	'Tslp'	0	0	0.04	0	0	0	0	0	0	0
53604	'Zpbp'	0	0	0.99	0	0	1.7	0	0.05	0	0
53605	'Nap1l1'	83.94	119.6	61.1	104	68.9	84.49	116	81.34	93.12	70.24
53607	'Snrpa'	135.1	79.64	77.9	76.4	119	133.3	103	105.3	134.3	110.72
53608	'Map3k6'	1.11	0	0.73	3.09	0	0	1.65	0	0	1.04
53609	'Clasrp'	25.93	24.16	47.4	30	24.3	28.29	26.5	18.95	27.48	11.39
53610	'Nono'	58.54	82.7	71.4	52.2	50	60.33	92.1	58	50.32	60.6
53611	'Vti1a'	6.2	8.78	7.49	9.29	12.1	5.21	13.4	11.22	15.65	10.96
53612	'Vti1b'	238.4	272.6	175	275	267	318	248	244.2	287	376.48
53614	'Reck'	0.02	0.53	0.01	3.78	3.06	0	0	0	0.95	0
53618	'Fut8'	64.04	36.67	41.2	47.6	31.8	40.24	47.5	40.8	47.81	17.91
53619	'Blcap'	312.6	295	270	206	282	283.1	318	309.7	270.4	393.66
53620	'Vamp5'	0	1.56	0.09	0	3.62	0.06	2.57	0	0.03	0
53621	'Cnot4'	23.43	20.13	16.1	11.7	8.21	8.18	10.1	13.94	5.82	17.85
53623	'Gria3'	10.68	30.5	18.9	12.8	16.7	12.88	17.1	17.7	6.6	26.6
53624	'Cldn7'	0	0	0	0.06	12.4	5.34	0.84	0	0.59	2.06
53625	'B3gnt2'	21.52	18.6	7.69	27.9	31.1	4.01	0.06	8.27	1.27	2.94
53626	'Insm1'	0.4	0	0.05	0	0	0.57	0.07	0.05	0	0.15
53627	'Porcn'	31.51	34.41	47.8	7.33	30	18.18	52.6	45.63	1.69	14.05
53761	'Prrc2a'	6.74	7.77	4.76	8.34	8.77	2.3	9.46	4.8	10.9	6.47
53814	'Oaz3'	0.15	0.11	0	0	0	0.6	0.55	0.23	0	0
53817	'Ddx39b'	116.5	110	126	110	96.7	183.7	103	155.6	129.5	116.83
53857	'Tuba8'	10.93	15.12	2.95	44.6	3.09	0	0.1	16.91	5.78	9.78
53858	'Rwdd2b'	4.48	13.93	2.83	0	7.72	5.08	16.1	2.9	0	6.22
53859	'Map3k14'	0.01	3.42	0	0	0.37	0	3.35	0.44	1.02	0.26
53860	'Sept9'	7.34	10.5	10.8	7.7	12.3	14.5	24	15.59	18.03	12.69
53861	'Zranb2'	50.54	39.93	34	47.9	52.6	56.54	51.1	49.62	49.8	45.11
53867	'Col5a3'	0	0	1.67	0	0	0	0	0	0	0
53868	'Rab25'	0	0	0	0	16.8	0	0	0	0	0
53869	'Rab11a'	161.6	166.8	164	96	126	196.4	153	182.1	191.2	185.16
53870	'Cntn6'	21.93	3.01	4.87	9.07	11.4	15.9	21.4	8.81	19.73	22.38
53871	'Pkd2l2'	0	0.22	0.02	3.06	0.09	0.02	0	0	0	2.39
53872	'Caprin1'	30.05	44.65	48.4	28.6	15.1	11.78	34.7	33.52	38.74	36.41
53881	'Slc5a3'	3.09	2.22	1.95	5.28	3.87	1.67	5.82	4.82	5.46	4.81
53883	'Celsr2'	6.69	5.8	12.8	12.9	10.9	10.54	9.96	7.89	4.04	6.27
53885	'Nphp1'	10.62	17.39	15.8	34	9.32	8.16	19.9	16.01	9.12	7.92
53886	'Cdkl2'	27.41	26.63	4.73	19.7	32.2	8.41	4.33	20.15	23.28	14.06
53890	'Sart3'	3.32	0.62	4.2	14.6	12	9.49	2.55	0.36	11.97	4.7
53892	'Ppm1d'	0.87	0.54	0.99	1.66	4.25	0	0	0.08	3.32	0
53893	'Nudt5'	6.83	11.21	4.12	4.64	3.06	8.61	0.22	8.58	10.28	7.64
53895	'Clpp'	161.6	164.9	150	192	179	223.7	168	213.4	223.4	136.19
53896	'Slc7a10'	24.56	16.77	10.5	21.7	6.03	8.39	5.98	5.25	15.89	0.02
53897	'Gal3st1'	1.37	0.11	6.53	0	3.24	7.62	1.1	5.11	3.94	0.94
53901	'Rcan2'	89.71	58.15	86.5	28.7	44.6	25.91	125	84.09	60.24	32.92

53902	'Rcan3'	5.75	4.02	14.7	2.76	7.13	6.51	11	4.07	9.85	3.99
53945	'Slc40a1'	0	0	0.01	0	0.03	0.12	3.31	0	1.83	1.94
53951	'Gpatch11'	13.61	8.98	7.54	9.04	3.76	6.54	2.67	6.05	12.9	5.51
53970	'Rfx5'	0.75	0.18	0.18	3.19	1.74	2.63	2.5	2.53	2.45	2.51
53972	'Ngef'	42.73	44.05	44.7	35.9	22.4	52.94	31.4	43.46	25.37	35.42
53975	'Ddx20'	4.02	5.79	7.39	0.05	3.29	11.56	4.13	11.17	11.49	14.96
53978	'Lpar2'	0	0.02	0.01	0	0	0	2.12	0	1.15	0
54003	'Nell2'	64.82	44.64	40.1	141	22	25.9	87	63.91	48.42	21.69
54004	'Diaph2'	0.12	0.38	0.1	0.03	0	0.32	0.07	0.05	0	0.38
54006	'Deaf1'	32.11	22.84	64.9	27.6	34.6	28.95	26.8	23.68	33.5	6.95
54120	'Gipc2'	4.68	3.38	9.76	5.31	2.86	11.8	10.2	5.73	2.58	5.2
54122	'Uevld'	3.35	6.39	4.72	4.14	0.04	4.92	3.17	2.35	11.4	6.13
54123	'Irf7'	0	1.91	5.94	16.3	7.87	2.31	0	0.3	6.05	1.62
54124	'Cks1b'	3.23	12.96	0.27	14.1	6.19	6.7	0.07	0.06	0	9.3
54125	'Polm'	2.72	5.74	2.25	0	0	0	2.29	5.12	4.61	1.57
54126	'Arhgef7'	8.38	15.32	13.7	9.53	6.9	9.94	6.27	10.72	16.38	24.29
54127	'Rps28'	103.4	186.1	240	279	83.7	329.4	171	67.33	166.1	77.63
54128	'Pmm2'	5	20.52	12.8	12.3	10.7	19.62	19.5	10.05	0.13	5.6
54130	'Actr1a'	126.5	101.1	131	97.6	77.6	89.68	149	125.8	114.2	183.2
54131	'Irf3'	16.55	12.74	14.7	10.6	31.8	2.1	0.07	9.16	8.88	23.09
54132	'Pdlim1'	0	0.03	0.03	0	4.01	0	0	0	0	0
54135	'Lsr'	0	0.05	0	0	5.7	4.43	0.03	0	0.02	0
54137	'Acrbp'	2.83	4.74	5.7	0	11.8	3.53	13.2	0	8.58	16.06
54138	'Atxn10'	218	243.5	301	297	231	222.2	321	259	251.3	274.5
54139	'Irf6'	0	0.02	0	0	0	0	0	0	0	0
54140	'Avpr1a'	0.88	0	0	0	0.02	1.25	19.7	0	3.63	2.14
54141	'Spag5'	2.2	0.08	0	2.34	1.56	0	0	0	0	0
54151	'Cyhr1'	49.76	66.82	56.3	6.62	64.6	54.4	59.4	36.29	50.85	50.78
54152	'Dnal4'	37.03	39.27	64.6	18.4	75.6	33.86	39.9	60.79	101.3	57.9
54153	'Rasa4'	7.64	11.79	8.25	7.54	9.97	10.48	1.87	6.42	14.5	7.36
54156	'Egfl6'	4.52	1.86	8.03	0	0.16	1.34	0	1.21	0	0
54160	'Copg2'	29.21	13.97	28.6	5.62	18.2	22.77	43.5	34.6	58.5	37.32
54161	'Copg1'	188.2	228.3	249	128	178	143.1	238	208.6	202.9	230.63
541610	'Trcg1'	0	0	0	0	0	0	0	0	0	0.08
54169	'Kat6b'	4.14	7.69	3.16	4.69	3.37	1.32	5.38	1.21	1.78	2.49
54170	'Rragc'	13.98	4.18	17.7	21.4	3.25	8.89	15.1	11.15	19.26	6.25
54188	'Cpsf4'	16.01	8.37	8.39	5.09	8.79	3.4	9.61	9.72	10.34	3.4
54189	'Rabep1'	13.98	11.87	15.7	18.4	9.08	5.97	19.8	15.66	13.58	9.47
54194	'Akap8l'	110	147.6	106	150	127	141.6	134	128.2	137.1	158.65
54195	'Gucy1b1'	62.91	61.75	35.4	30.9	69.1	40.4	83.7	75.32	78.33	76.17
54196	'Pabpn1'	27.05	18.24	18.5	22.7	31.3	51.69	28.1	40.35	21.71	22.91
54197	'Rnf5'	113.4	128	122	128	161	94.37	173	177.7	93.11	130.3
54198	'Snx3'	332	236.9	216	214	179	342.7	385	240.3	245.6	207.32
54199	'Ccrl2'	0.03	0	0	0	0	18.05	0	0.02	0	0
54200	'Sult2b1'	4.25	2.01	10.1	9.01	10.3	14.56	9.12	12.06	0	17.02
54201	'Zfp316'	1.56	2.2	3.73	3.11	2.91	2.42	1.29	2.2	1.37	0.97
54204	'Sept1'	2.32	0.66	1.01	0	0.41	1.84	0.11	0	0.84	0.45
54208	'Arl6ip1'	362.6	353.9	347	339	314	358.6	439	328.1	298.4	329.91
54214	'Golga4'	8.02	8.52	5.05	9.47	7.98	7.35	4.24	2.38	2.68	5.36
54215	'Cd160'	0	0.37	0	3.04	0	0	0.2	0	0.36	0
54216	'Pcdh7'	10.16	10.08	6.68	7.08	7.77	11.29	15.9	11.18	5.83	5.66
54217	'Rpl36'	262	294.9	447	437	270	396.8	282	131.8	291.4	228.98
54218	'B3galt4'	0	5.69	1.91	0	3.34	2.2	0	4.11	6.52	0
54219	'Cd320'	27.39	26.45	6.91	52.7	13.3	35.44	22.7	25.41	31.17	37.34
54324	'Arhgef5'	0	0	0	0	0.01	0.01	0	0	0	0
54325	'Elovl1'	12.25	6.19	4.06	13.7	6.42	17.74	9.72	5.5	9.31	6.79
54326	'Elovl2'	0.61	1.46	0	2.59	0.01	0.64	0	0	3.03	0
54338	'Slc23a2'	39.99	26.04	42.5	8.05	33.5	14.02	31.3	15.66	30.49	15.23

54342	'Gnpnat1'	13.67	4.85	11.7	10	19	15.66	3.54	12.85	10.54	15.45
54343	'Atf7ip'	2.5	2.1	2.4	1.79	3.45	4.91	2.16	2.61	7.94	5.08
54351	'Elp5'	109.9	75.47	113	86.5	69.6	120.8	86.8	60.64	101.6	67.58
54352	'Irx5'	0	0	0	0.02	0	0	0	1.13	0	0
54353	'Skap2'	2.96	4.14	1.75	6.25	2.12	5.49	16.2	0.79	6	7.35
54354	'Rassf5'	2.6	3.43	12	0	4.16	6.55	11.2	5.6	1.19	1.99
54357	'Epb41l4b'	1.06	4.45	5.31	4.57	4.35	5.9	4.22	2.5	1.77	7.13
54364	'Rpp30'	26.74	32.47	16.6	22.8	31.8	58.64	47.5	40.65	34.83	29.19
54366	'Ctnnal1'	3.57	3.2	1.72	2.8	3.9	0.41	9.02	2.79	4.37	0.61
54367	'Zfp326'	17.93	10.49	15.5	28	11.4	12.04	13.5	7.49	12.54	14.28
54368	'Gp9'	0	1.12	0.52	0	0	0	0	0	0	0
54369	'Nme6'	29.99	19.63	12.9	11.2	7.29	26.33	9.92	6.16	32	31.72
54371	'Chst2'	3.55	7.06	8.84	5.09	6.07	4.18	6.03	8.16	6.08	4.2
54375	'Azin1'	17.73	19.94	25.6	25.1	17.4	12.26	8.63	10.5	14.05	16.22
54376	'Cacng3'	19.06	26.94	45.7	17.8	7.74	44.06	15.2	45.2	18.73	0
54377	'Cacng4'	0.7	10.73	10.8	7.54	4.2	0.34	9.49	9.53	5.54	4.24
54378	'Cacng6'	0.04	0	0	3.87	1.16	0	0	0	0	0
54380	'Smarcal1'	7.63	9.89	20.8	6.07	7.58	5.5	11.3	6.9	9.15	9.83
54381	'Cpq'	16.87	21.35	14.1	29.1	15.2	7.38	37.8	12.67	0.06	25.17
54383	'Phc2'	8.33	6.89	4.6	8.58	2.39	3.36	17.4	10.87	18.1	8.7
54384	'Mtmr7'	43.27	25.13	33.6	8.66	22	28.73	35.2	39.39	29.09	31.59
54387	'Mcm3ap'	7.93	3.23	3.99	4.61	3.91	4.79	2.35	2.21	5.51	5.7
54390	'Sit1'	0	0	0	0	0	0	0	0	0	2.19
54391	'Rfk'	69.17	67.65	61.6	74.7	52.6	54.91	71.6	34.63	59.82	16.82
54392	'Ncapg'	0	0	0	0.75	0	0	0.05	0	0	0
54393	'Gabbr1'	44.32	45.4	49.9	42	38.9	26.06	46.6	40.73	54.12	57.77
54394	'Crlf3'	23.05	6.96	11	0.09	5.4	0.34	6.9	1.22	7.29	8.13
54396	'Irgm2'	0	0	0.04	0	0.07	0	0	0	0	0.01
54397	'Ppt2'	4.05	6.61	0.96	2.72	12.1	1.76	7.58	9.7	10.14	11.47
54399	'Bet1l'	19.34	24.85	14.6	13.4	49.8	64.14	42.3	35.67	39.17	32.55
54401	'Ywhab'	411	355.7	369	284	273	314.9	387	405	371.8	281
54402	'Stk19'	81.62	67.55	63.2	78.9	56	46.23	36.7	70.79	49.87	58.67
54403	'Slc4a4'	1.65	1.86	1.35	4.76	2.29	3.13	1.43	0	1.44	0.42
54405	'Ndufa1'	348.6	380.5	329	529	332	604.4	399	249.4	505.2	382.21
54409	'Ramp2'	3.93	3.11	14.4	25.1	4.96	0	9.97	13.27	6.63	3.29
54411	'Atp6ap1'	323.6	370.6	333	374	297	317.8	368	368.9	340.3	313.24
54418	'Fmn2'	2.78	1.1	2.54	3.03	0.27	1.59	1.58	0.46	0.91	1.54
54422	'Barhl1'	0	0	0	0	0	0.89	0	0	0	0
54445	'Unc93b1'	3.18	0	0	0.03	0.05	10.75	0	0	3.23	3.66
54446	'Nfat5'	0.92	1.03	0.89	0.78	0.71	2.97	0.01	0.55	0.27	1.63
54447	'Asah2'	0.01	0	3.86	0	1.55	0	0	0.97	1.59	0.1
54451	'Cpsf3'	42.17	69.16	57.6	45.2	61.5	26.96	77.9	55.39	49.71	31.74
544678	'Cfap74'	9.29	6.37	6.31	17.2	10.7	16.36	9.82	10.58	6.23	20.66
544696	'Tbc1d32'	1.38	1.64	0.54	1.47	0.39	1.76	2.95	0.85	3.07	1.1
544717	'1190007I07Rik'	19.8	20.25	18	13.8	17.7	23.57	20.7	8.54	13.95	19.3
54473	'Tollip'	50.22	77.14	71.5	35.4	79.8	26.12	50.3	81.68	86.42	73.03
544736	'Glipr1l3'	0	0	0	0	0	0	4.43	0	0	0
544763	'Hbq1b'	0	0	0	0	0.03	0.03	0	0	0	0
544791	'Myh13'	0	0.01	0	0	0	0	0	0	0	0
544817	'Arhgap27'	5.92	2.41	13.7	15.5	3.17	3.02	6.46	5.47	4.38	7.94
54484	'Mkrn1'	35.62	28.21	30.2	39.1	21.8	37.01	68.7	37.91	40.6	32.68
54485	'Dll4'	0	0	0	0	0	0	0	0	2.23	0
54486	'Hpgds'	4.52	16.73	0.18	13.6	2.32	0.63	0.41	2.93	1.63	2.32
544864	'Gm5785'	0.07	0.12	0.13	1.5	0.13	0.61	0.1	0.05	0.18	0.11
544922	'Zkscan4'	2.71	0.8	4.25	7.98	4.59	0.02	6.87	0.73	3.77	2.93
544963	'Iqgap2'	0.79	0.52	0	0	0.91	0	0.02	0.23	0.15	0.5
544971	'Bdp1'	2.02	0.86	1.83	7.64	2.42	1.51	3.73	1.47	4.33	1.33
544990	'Gm5795'	0.28	0	1.85	0	0	0	2.84	1.67	0.75	0

545007	'Gm5796'	1.7	2.7	2.63	1.52	4.06	0.19	1.61	3.09	2.06	1.45
545055	'Cma2'	4.03	0	0	0	0	0	0	0	0	0
545085	'Wdr70'	7.02	9.34	6.68	0.74	7.53	5.55	6.77	6.83	14.5	11.42
545124	'Tdg-ps'	0	0	0	0	0	0	0	0	0	0.01
545136	'Fam186b'	0.87	1.87	0	0.4	0	0	2.03	0.3	0.17	0
545140	'Olfr288'	0.13	0	0.1	0	0	0.04	0	2.36	0	0
545156	'Kalrn'	3.7	2.89	5.37	3.51	6.58	2.87	3.98	5.29	4.22	1.53
54519	'Apbb1ip'	2.34	1.3	0.02	0	1.41	11.48	3.74	0	0	3.25
545192	'Baiap3'	172	148.5	163	98	107	86.53	116	99.49	123.6	142.95
54524	'Syt6'	36.05	3.89	0.24	29	0.54	0	0.09	4.57	0	0.03
54525	'Syt7'	2.55	1.1	3.56	3.12	1.92	0.83	0.59	1.46	2.59	1.48
545253	'Gm5820'	0.76	1.14	2.96	2.24	4.23	1.29	0	1.61	6.23	6.19
54526	'Syt10'	13.85	8.47	12	29.2	10.9	2.28	1.2	7.83	14.64	27.65
545276	'Gal3st3'	17.54	13.1	15	10	12.1	17.95	29.7	13.36	18.56	5.05
545291	'Hpse2'	0.07	0	0	0	0	0	0.02	3.24	0	0
545370	'Hmcn1'	0	0	0	0	0	0	0.19	0	0	0
545389	'Cep170'	8.21	10.26	7.92	13.5	6.27	4.06	5.48	6.02	13.69	11.09
545391	'Catspere2'	1.37	0.47	2.21	1.13	0.72	1.86	1.24	1.09	3.04	0.13
545428	'Ccdc141'	1.64	4.04	0.03	0	0.3	0.02	2.8	0	0	0.02
545471	'Zfp345'	0	0	0	0.02	0	0.01	0	0	0	0
545474	'Scrt2'	0	0	0	0.02	1.03	0	0	0	0	0.15
545481	'Arhgap40'	0	1.19	0.12	0	0	0	0	0.1	0	1.09
545490	'Zfp973'	1	1.68	2.94	0.98	2.89	0.71	3.1	2.24	1.31	0.68
545527	'Erich6'	0.33	0.04	0	0	0	0	0	0	0	0
545554	'Ankrd34a'	19.56	32.07	12.3	3.44	25.9	10.23	17.2	9.55	41.85	7.23
545562	'Amy2b'	0	0	0.04	0	0	0	0	0	0	0
54561	'Nap1l3'	55.02	68.27	60.9	37.4	51.4	58.72	72.2	48.23	40.87	41.52
545611	'Fam205a2'	2.06	1.76	1.28	0.89	2.74	1.13	0.24	1.03	1.86	0.03
54562	'Lrrc6'	12.05	4.29	3.05	15.6	1.73	0.02	1.47	7.62	6.91	3.54
545622	'Ptpn3'	0	0.25	0.24	0	0	0	0.12	0.42	0.38	0
54563	'Nup210'	1.16	0.59	1.11	1.6	5.63	0.62	2.17	1.77	1.24	1.45
545645	'Gm13283'	0	0	0	0	0	0	0	0	3.62	0
545649	'Gm13276'	0	0	0	0	0	0	0	0	0.51	0
545651	'Gm13278'	0	0	0	0	0	0	0	0	0.21	0
545652	'Gm13275'	0	0	0.19	0.07	0	0	0	0	0.17	0
545662	'B020004J07Rik'	0	0.07	0	0	0	0	0	0	0.02	0
545667	'Shisal2a'	5.44	7.19	13.4	0	0	23.07	0	5.37	3.01	6.61
545725	'Mterf1a'	5.5	1.53	0.04	0	3.84	0.09	0	2.83	1.81	4.26
545814	'Smok3a'	0	0	0.04	0.87	0.18	0	0	0	0	0
545817	'Cyp2w1'	0	0	0	0	0	0	0	0.01	0	0
545824	'Zar1l'	0	0	0	1.92	0	0	0	0.14	0	0
545893	'Mansc4'	0	3.69	0	0	0	7.01	0	0	0	0
545902	'Ptprh'	0	0.07	1.01	0.39	0	0	0	0	0	0
545929	'Gm5891'	0	0	0	0	0	0	0	0	0	0
545938	'Zfp607a'	0.1	0.03	0.02	0.02	0	0	0.03	0.02	0	0
545975	'Cers3'	0	0	0	0	0	0.35	0	0.01	0.08	0
54598	'Calcrl'	0	0.18	1.08	0	0	0	1.48	0.16	0	0
54601	'Foxo4'	0	0.18	0	0	0	0	0	0	0	0.43
546024	'Crxos'	2.33	0	0.02	0	0	0	0.06	0.04	0.05	0.08
54604	'Pcnx'	0.51	3.41	1.94	7.22	1.52	0.03	6.49	0.93	2.07	1.84
546049	'C330021F23Rik'	0.14	0.04	0	0.09	0.02	0	0.04	0.03	0.12	0
546061	'LOC546061'	0	0	0	0	0.02	0	0	0	0.02	0
54607	'Socs6'	2.33	1.19	3.25	1.6	2.92	0.02	0.02	2.73	2.19	3.96
546071	'Mast3'	12.59	7.33	19.7	17	10.9	17.72	8.68	9.83	11.35	7.85
54608	'Abhd2'	1.61	0.02	11	0	0	1.65	4.21	0	0	5.39
54609	'Ubqln2'	4.49	5.77	8.46	13.8	5.93	9.11	11.4	4.48	15.72	4.42
54610	'Tbc1d8'	8.06	4.95	5.07	3.66	6.53	2.45	12.7	4.53	13.03	8.76
54611	'Pde3a'	0.01	0.24	0.23	0.02	0.35	0	0	0	0	0.3

546118	'Ubtfl1'	0	0	0	0	0	0	0.02	0	0	0
54612	'Sfrp5'	6.82	0	7.92	2.56	0	0	8.72	2.1	3.42	0.02
54613	'St3gal6'	3.26	0	0	0.38	6.12	0.37	0.77	3.05	3.07	0.51
546134	'Gramd2'	2.69	1.94	0.95	1.26	5.94	0	0	3.83	3.34	0.05
54614	'Prpf40b'	20.76	17.85	25.1	38.7	30.8	21.84	21.5	19.49	22.09	24.61
546143	'Ccpg1os'	13.88	16.96	9.73	0	5.32	15.95	36.3	30.33	42.19	22.45
54615	'Npff'	0	0	0	0	3.23	0	3.04	0	0.11	1.64
54616	'Extl3'	3.27	6.21	10.9	4.49	5.83	7.48	6.89	1.1	7.35	6.57
54624	'Paf1'	51.99	64.97	44.9	32.4	57	104.6	132	69.39	97.58	77.19
54630	'Prickle3'	0	0.12	0	0	0	0	0	0	0.09	0
54631	'Nphs1'	0.2	0.18	0.22	0.36	0.32	0.13	0.25	0.16	0.2	0.15
54632	'Ftsj1'	9.29	11.24	11	4.93	9.34	16.66	14.9	6.16	12.67	18.82
546325	'Gm5936'	0	3.33	0	0.13	4.32	0	0.05	0	0.03	3.31
54633	'Pqbp1'	144.9	167.9	152	247	132	212.1	152	166.4	155.7	171.1
546336	'Prrg1'	0	0.45	0.45	0	0.46	0.01	0.43	0.01	1.08	0
54635	'Pdgfc'	0.49	0.18	1.82	0.03	0.35	0	0	1.24	0	0
54636	'Wdr45'	44.49	51.58	31.3	31	51.3	66.13	65.4	52.33	61.38	38.86
54637	'Praf2'	118.6	132.3	152	135	146	162.3	224	207.2	93.65	118.43
54638	'Ccdc22'	23.2	20.23	14.6	2.27	26.2	10.13	18.1	20.81	16.61	48.04
54644	'Otud5'	4.79	3.1	6.4	17.6	5.33	5.66	1.62	3.8	4.88	0.13
54645	'Gripap1'	46.55	45.47	50.6	22.7	57.3	38.41	29.8	37.81	27.29	41.11
54646	'Ppp1r3f'	3.51	1.43	5.66	2.83	3.69	0.12	0	3.98	3.26	1.08
54648	'Ccdc120'	0.66	1.89	3.06	2.53	0	1.43	0	1.68	2.6	1.99
54650	'Sfmbt1'	2.44	4.61	0.48	5.61	4.17	2.46	4.95	1.45	3.62	3.23
54651	'Usp27x'	3.63	3.93	1.7	5.46	3.64	0.46	0	0	0.47	4.6
546519	'Tmem235'	0	0	0	0	0	0	0	0	4.9	2.49
54652	'Cacna1f'	0	0	0.01	0	0	0	1.35	0	0	0
546611	'Klhl33'	0.53	0.71	3.52	0.14	0.44	0.64	5.31	0.07	5.48	3.54
546648	'Klhdc7b'	0	0	0.11	0	0	0	0	0	0	0
54667	'Atp8b2'	4.15	5.38	9.57	13.7	6.83	2.35	7.39	6.95	4.2	6.45
54670	'Atp8b1'	0	0.03	0	0	0	0	0	0	0	0
54672	'Adgrg3'	0	0.75	0	0	0	0	0	0	0	0
54673	'Sh3glb1'	4.39	5.45	4.74	39.8	6.2	6.2	7.51	12.19	4.72	4.2
54678	'Zfp108'	3.47	2.31	8.14	0	5.25	9.88	19.6	2.32	0.35	3.06
54683	'Prdx5'	514.2	273.1	499	470	501	546.4	615	486.8	424.3	377.8
546840	'Ldlrad1'	0	0.4	1.15	0	0	0	0.01	0	0	0.51
546886	'Cfap73'	0	0.92	0	0	0.67	2.13	0	0	0	8.52
546983	'Vmn2r77'	0	0	0	0	0	0	0	0.08	0	0
54709	'Eif3i'	146.5	87.79	156	101	138	244.1	175	167.5	138.7	155.87
547109	'Trim43a'	1.16	0	0	0	1.03	0	0.03	2.65	0	0
54711	'Plagl2'	0.46	0.65	0	0	0.98	0.01	0	0	0.66	0.8
54712	'Plxnc1'	6.48	7.44	3.87	5.67	4.86	2.88	3.31	2.99	3.01	4.02
54713	'Fezf2'	1.3	0	0	0	2.07	0	0	0	0	0
547176	'Zc3h12b'	1.15	3.25	2.65	1.16	4.95	1.5	0.41	2.15	1.03	1.88
54720	'Rcan1'	7.94	4.42	12.8	16.7	3.47	12.37	10.2	16.23	4.39	13.1
54721	'Tyk2'	0	4.6	4.1	3.64	0.8	1.7	0	1.42	1.76	3.25
54722	'Gsdme'	14.1	19.38	12	0.03	14.3	24.47	19	33.91	22.37	24.13
54723	'Tfip11'	9.4	11.54	14.8	12.5	11.7	5.79	16.3	6.29	16.5	8.33
54725	'Cadm1'	169.6	213.5	114	213	150	113.2	229	193	123.8	122.49
547253	'Parp14'	0	0	0.03	1.12	0.01	0	0	0.03	0	0
550619	'Arid3c'	0	0	0	0	0.14	6.77	0	0	0	0
553127	'Rtl8b'	124.8	103.9	121	115	110	93.02	111	123.5	184.9	143.17
554327	'2610042L04Rik'	14.52	9.52	10.6	3.2	12.1	3.36	7.34	20.63	19.7	4.03
55927	'Hes6'	39.1	62.29	58.8	88.1	19.4	71.52	30.8	28.89	102.4	87.3
55932	'Gbp3'	10.37	0	2.96	16.5	0	3.48	0	0.54	0	4.36
55934	'Rp9'	16.77	31.53	61.4	45.4	23.9	39.21	21.7	17.16	60.9	15.22
55935	'Fnbp4'	11.12	12.16	13.9	17.1	9.31	10.85	19.8	13.07	19.81	14.69
55936	'Ctps2'	17.64	23.99	22.2	13.2	27.2	27.39	10.7	20.82	22.42	35.37

55942	'Sertad1'	4.04	6.88	4.11	10.8	5.22	25.03	0	0	0	5.76
55943	'Stx8'	5.64	9.63	8.93	21.5	16.5	9.84	10.7	7.98	9.51	7.95
55944	'Eif3d'	65.92	70.13	75.4	53.4	35	62.73	51.3	56.56	60.31	30.41
55946	'Ap3m1'	17.74	7.65	17.3	8.22	11.4	11.22	12	7.6	24.7	23.17
55947	'Dclre1a'	1.55	1.07	0.9	1.21	0.81	0.22	0	0	0.81	2.66
55948	'Sfn'	0	2.47	0.09	0	0	0	0	0	0.03	0.85
55949	'Eef1b2'	90.14	107.1	92.1	110	130	125.1	111	95.68	96.56	105.5
55950	'Bri3'	8.73	9.06	4.1	26.2	8.58	0.06	0.05	6.12	10.65	0.69
55951	'Mpc1'	225.5	135.3	251	292	304	321.4	250	233.3	223.1	181.08
55960	'Ebag9'	18.91	10.44	5.19	14.1	27.2	3.43	3.95	15.42	9.78	13.76
55961	'Slc13a1'	0	0.29	0	0	0	0	0	0	0	0
55963	'Slc1a4'	29.14	27.98	32.6	11.4	6.26	8.13	25.8	31.83	28.13	19.46
55978	'Ift20'	59.04	39.42	60.1	90.1	93.6	80.26	97.2	63.96	52.39	36.72
55979	'Agpat1'	61.41	86.3	77.5	8.82	63.5	80.32	65.3	82.47	116.4	79.58
55980	'Impa1'	38.24	38.1	54.7	44.5	44.4	80.3	43.9	47.54	27.87	49.27
55981	'Pigb'	2.13	3.88	8.4	0.43	3.85	6.97	2.48	3.9	7.93	6.84
55982	'Paxip1'	10.05	7.88	5.52	3.46	1.78	5.56	5.46	9.17	4.5	6.46
55983	'Pdzrn3'	2.96	0	0	0	0	0	0.76	0	3	0
55984	'Camkk1'	24.03	37.5	57.3	22.9	23.5	18.26	41.1	48.11	50.7	17.49
55987	'Cpxm2'	0	0	0.02	0	0.85	0.02	0.02	1.35	0	3.3
55988	'Snx12'	24.43	15.69	21	31.1	17.3	25.15	19.9	15.6	34.54	29.01
55989	'Nop58'	28.33	33.03	31.3	25.1	38.2	37.02	34.8	42.53	44.34	38.05
55991	'Panx1'	6.16	8.31	5.01	7.8	12.8	9	5.96	16.95	11.2	2.01
55992	'Trim3'	15.11	29.16	30.4	18.5	25.8	50.21	39.1	19.8	47.49	19.69
55993	'Msh4'	0	0	0	0	0.06	0.03	0	0	0	0
55994	'Smad9'	0.2	0.3	0.66	0.01	0.12	0.06	2.1	0.83	2.83	1.59
56009	'Alyref2'	4.36	14.91	7.79	0.18	3.67	8.52	9.35	8.66	17	9.86
56012	'Pgam2'	0.61	0.33	5.76	0.4	0.51	14.24	19.8	4.77	0.5	4.44
56013	'Srcin1'	1.89	1.39	1.02	1.32	4.36	2.05	2.44	1.49	2.02	2.03
56015	'Olfr71'	0	1.81	0	0	0	0	0	0	0	0
56016	'Hebp2'	18.07	15.54	6.01	52.1	14	13.45	5.36	14.13	16.38	11.62
56017	'Slc2a8'	16.27	23.61	18.1	0	12.2	17.24	15.8	16.39	11.88	22.81
56018	'Stard10'	7.63	14.87	5.76	15.2	18	11.09	2.95	8.44	33.77	20.57
56030	'Tmem131'	1.53	1.49	1.81	6.44	2.41	0.26	1	2.35	1.46	1.65
56031	'Ppie'	16.4	38.11	39.3	115	43.3	44.68	34.8	42.36	21.62	23.19
56032	'Nprl2'	82.31	67.73	70.3	57.3	99.2	119.3	61.9	95.66	41.93	109.65
56036	'Ccnl2'	21.98	34.48	30.8	61.8	27.1	18.17	15	31.2	40.37	22.88
56040	'Rplp1'	556.9	491.6	704	595	459	802.5	618	354.3	565.2	545.17
56041	'Uso1'	0.63	2.19	3.83	5.83	4.27	4.58	5.06	5.56	9.93	0.57
56043	'Akr1e1'	24.86	28.33	31.6	36.5	15.8	56.23	13.3	24.96	12.57	34.2
56044	'Rala'	34.34	27.4	36.7	46.4	23.7	18.19	15.2	25.46	42.42	5.77
56045	'Samhd1'	2.65	5.18	5.78	2.8	3.4	2.65	4.38	1.09	0	2.4
56046	'Uqcc1'	78.61	71.04	73.1	51.1	66.2	88.4	70.2	75.42	78.4	77.88
56047	'Msln'	2.4	6.82	0	9.62	4.24	0	0	0.42	0	0
56048	'Lgals8'	25.95	31.07	53.5	32	38.2	34.94	36.8	30.89	28.13	59.57
56050	'Cyp39a1'	0	0	0	0.64	0	0	0	1.03	0	0
56055	'Gtpbp2'	9.66	2.36	2.59	16.5	5.16	1.25	4.07	1.1	7.51	2.03
56057	'Btg4'	0	0	0	0	0	1.83	0	0	0	0
56068	'Ammecr1'	0.06	0.55	1.7	0.4	0	0.03	0.59	0	0	0
56069	'Il17b'	0.21	0	0	0	0.68	0.07	0.08	0.37	0.8	0.2
56070	'Tcerg1'	27.9	16.52	31.8	38.2	19.1	15.98	27.4	18.2	24.5	16.55
56072	'Lgals12'	0	0	0	0	0	0	1.69	0	0	0
56075	'Pdss1'	5.45	6.96	9.77	8.08	6.38	13.53	9.04	6.9	11.59	8.15
56077	'Dgke'	4.57	6.45	17.5	21.8	14	1.67	8.25	8.11	6.69	6.23
56078	'Car5b'	0.13	1.75	0.2	0.5	0.38	0.84	0.31	0.15	0.22	0.09
56079	'Astn2'	0.01	0.66	0.01	0.2	1.48	0	0.44	1.51	0.52	0.02
56085	'Ubqln1'	21.16	15.23	15.5	37.2	30.1	13.91	14.2	6.05	33.2	10.07
56086	'Set'	92.31	107.9	111	201	121	52.87	108	86	108.3	98.24

56087	'Dnah10'	0.5	0	0.03	0.26	0.02	0.12	0.12	0.02	0.42	0.33
56088	'Psmg1'	69.9	62.18	55.2	61.7	61.8	84.93	63.1	73.1	57.49	79.09
56089	'Ramp3'	6.92	82.08	0.12	4.5	4.62	0.08	0.04	6.66	0	1.75
56094	'Cts8'	0	0	0	0.06	0	0.02	0	0	0.02	0.04
56095	'Ftsj3'	10.07	7.78	15.4	33.7	12.7	14.89	5.56	13.61	15.36	12.1
56149	'Grasp'	0	0.24	1.03	0	0	0	2.51	0.02	3.22	0.59
56150	'Mad2l1'	15.02	6.66	5.01	15.8	1.13	4.16	0	7.05	0.43	0
56173	'Cldn14'	0	4.19	0	0	0	0	0	2.73	0	0
56174	'Nagk'	134.2	116.1	110	131	144	104	83.6	126.1	133.2	97.24
56175	'Bace2'	1.37	0	0	0	0	0	0	0.92	0	0
56176	'Pigp'	81.81	136.6	46	87.6	99.8	112	70.2	109.6	80.46	126.6
56177	'Olfm1'	94.47	104.6	95.5	213	116	83	113	48.64	82.55	70.92
56183	'Nmu'	0	0	0	0	0	0	0	17.87	0	0
56187	'Rabggta'	37.2	14.31	44.7	13.3	25.2	41.05	56.3	35.77	53.54	37.26
56188	'Fxyd1'	40.54	15.59	21.6	8.13	64	38.13	15	10.26	22.5	22.88
56189	'Prodh2'	0.61	0	0	0	0	0	0	0	0	0
56190	'Rbm38'	3.54	6.61	2.41	5.68	0.03	9.7	2.36	5.63	0.44	0
56191	'Tro'	50.73	31.36	50.8	65.1	36.9	44.36	60	47.58	64.03	59.25
56193	'Plek'	0	2.8	3.92	0.57	0.18	6.98	1.46	0.91	1.47	1.25
56194	'Prpf40a'	13.42	19.91	16.8	15.1	25.3	32.12	11.7	26.62	23.29	22.54
56195	'Ptbp2'	41.07	38.4	37.7	23.1	21.7	16.07	36	24.61	27.19	46.78
56196	'Tdp2'	5.85	7.5	7.29	3.99	12.8	0	8.19	12.47	12.22	8.62
56198	'Heyl'	0.84	1.16	2.67	0.73	0	0.14	1.88	0	1.33	0
56199	'Abcb10'	1.54	7.13	11.3	16.4	7.35	4.17	4.05	4.78	1.91	1.08
56200	'Ddx21'	2.39	2.48	2.25	3.64	0.32	1.46	6.45	2.19	0.05	0.86
56205	'Ensa'	193.6	282.6	231	259	316	176.7	237	226.6	138.2	229.59
56207	'Uchl5'	59.58	41.28	54	25.6	78.7	24.59	59.7	60.84	86.89	76.29
56208	'Becn1'	114.2	91.93	70.6	126	72	121.1	129	156.4	96.45	118.45
56209	'Gde1'	222.1	229.3	226	166	225	271.6	247	251.7	180.6	242.79
56210	'Rev1'	3.38	4.73	5.16	15.5	2.45	0.07	1.4	4.57	5.08	1.5
56212	'Rhog'	3.14	19.37	26.9	0	17.9	1.22	0.04	1.36	3.61	3.42
56213	'Htra1'	34.9	30.66	28.3	9.62	8.75	24.02	19.5	9.56	24.14	2.36
56214	'Scamp4'	110.5	107.7	72	120	95.8	81.54	86.9	90.33	89.02	84.47
56215	'Acin1'	17.58	16.14	8.25	10.5	16.3	15.48	12.9	13.96	18.39	13.07
56216	'Stx1b'	28.58	21.41	52.4	9.12	36.6	22.3	44.8	37.18	29.13	23.73
56217	'Mpp5'	14.78	14.47	11.4	16.8	10.5	14.17	12.7	7.21	12.26	15.62
56218	'Patz1'	4.39	8.72	8.01	15.3	3.96	3.41	12.1	5.27	7.81	1.31
56219	'Extl1'	9.64	2.7	10.9	3.08	7.03	0.02	8.05	6.69	5.96	2.2
56220	'Zfp386'	7.13	10.3	5.91	6.41	2.92	10.14	7.53	5.91	18	10.33
56222	'Cited4'	3.26	0.7	1.48	7.23	1	6.1	0	0.85	8.38	0
56223	'Fscn3'	0	0	1.22	0	0	0	0	0	0	0.29
56224	'Tspan5'	3.18	1.64	2.8	7.97	3.61	2.67	7.85	2.62	5.67	2.97
56226	'Espn'	0	0	1.76	0	0.09	0	1.56	0.96	0.43	0
56228	'Ube2j1'	25.52	19.44	21.2	48.4	19.8	10.24	26.9	24.62	21.19	18.24
56229	'Thsd1'	0	0	0.01	4.61	0	0	0.01	0	0	0
56233	'Hdac7'	27.05	42.91	16.5	13.2	16.1	13.25	27.1	14.33	2.94	35.98
56248	'Ak3'	11.97	17.84	18.6	16.2	16.1	6.18	6.19	8.61	8.52	1.69
56249	'Actr8'	58.3	71.64	69	8.73	72.6	102.1	42.1	62.89	77.64	77.93
56258	'Hnrnph2'	86.2	117.7	122	118	96.5	64.86	129	90.3	71.05	105.63
56264	'Cpxm1'	5.81	4.51	11.2	8.13	0	4.41	7.52	0.02	2.2	0.96
56273	'Pex14'	21.95	52.23	55.6	27	33.4	23.29	48.6	44.86	35.54	36.3
56274	'Stk3'	12.33	10.98	2.85	0.22	6.75	2.96	7.72	8.91	4.5	6.25
56275	'Rbm14'	17.9	23.9	10.1	0	7.75	16.37	16.7	7.94	18.29	14.09
56278	'Gkap1'	13.67	7.85	4.19	17.3	3.73	7.5	5.44	6.32	7.85	9.39
56279	'Fam69b'	16.08	15.25	15.2	29.2	23	29.9	20.5	19.34	57.28	12.87
56280	'Mrpl37'	32.29	44.95	54.8	45.7	65.5	48.83	65.8	67.79	23.82	41.11
56282	'Mrpl12'	116.8	88.86	118	131	135	203	134	131.4	204.6	114.03
56284	'Mrpl19'	13.96	7.49	6.87	7.78	12.9	9.79	4.79	8.84	9.71	10.15

56289	'Rassf1'	30.77	43.06	36.6	44.7	39.3	27.09	35.3	32.78	23.8	39.35
56291	'Styx'	4.21	2.3	1.47	7.23	1.56	2.19	3.66	3.09	1.72	1.51
56292	'Naa10'	89.48	90.74	81.2	83	103	46.06	95.8	88.28	98.18	69.72
56293	'Slc35g3'	0.21	0	0	0	0	0	0	0	0	0.03
56294	'Ptpn9'	11.59	4.76	9.41	17.6	5.35	3.37	1.52	6.65	4.78	1.9
56295	'Higd1a'	130.9	112.6	150	106	185	218.9	112	105.2	121.2	91.05
56296	'Dmrtb1'	4.58	1.61	2.32	0	10.1	8.57	4.04	0	11.87	2.36
56297	'Arl6'	48.77	79.26	64.2	67.3	53.8	86.89	65	52.55	70.47	98.4
56298	'Atl2'	17.91	7.53	4.1	12.3	7.17	5.28	6.73	6.89	7.08	8.11
56299	'Fkbpl'	4.03	8.35	4.39	4.32	14.1	0.04	8.82	20.69	12.16	12.92
56305	'Pitpnb'	72.45	64.09	34.8	31.4	27.8	46.16	30.8	56.71	34.53	65.38
56306	'Sinhcaf'	0	2.66	0.52	3.6	5.47	0	9.52	0.52	0	0.03
56307	'Metap2'	49.64	68.06	51.1	71.9	52	64.18	58.3	68	46.66	54.27
56309	'Mycbp'	8.79	2.79	9.32	23.8	8.06	17.51	5.19	4.98	5.86	11.03
56310	'Gps2'	12.61	17.02	11.8	29.2	26.5	30.15	17.1	23.99	24.66	28.14
56312	'Nupr1'	0	0	0	0	17	0	0	2.36	0	0
56314	'Zfp113'	1.01	1.37	0.9	3	2.18	3.75	2.03	0.89	6.08	0.7
56315	'Rhcg'	3.13	3.85	0.59	0	2.08	0	0.7	0	0	0
56316	'Ggcx'	11.44	15.24	8.29	18.8	10.1	12.32	0.61	1.33	4.63	21.07
56317	'Anapc7'	34.23	15.5	26.3	30.7	8.25	15.39	7.08	24.8	10.94	23.62
56318	'Acpp'	0	0	0	0	2.03	0	0	0	0	0
56320	'Dbn1'	65.5	77.94	46.9	61.9	75.3	51.88	21.6	49.27	43.98	70.78
56321	'Aatf'	14.94	17.08	17.2	28	12.5	9.78	16.3	21.36	15.56	21.78
56322	'Timm22'	29.53	61.15	31.3	60.6	53.1	48.88	24.8	38.27	54.48	55.21
56323	'Dnajb5'	19.66	12.32	5.3	9.13	16.4	5.58	8.31	19.57	35.15	9.11
56324	'Stam2'	14.58	9.28	1.58	3.83	2.53	0.23	9.69	7.3	9.88	11.19
56325	'Abcb9'	8.19	19.09	16.9	0.88	2.1	8.78	16.7	8.71	6.02	2.36
56327	'Arl2'	339.4	262.8	326	282	323	399.9	320	391.9	375.7	324.96
56330	'Pdcd5'	164.8	197.4	235	206	192	258.4	187	178.8	227.2	147.75
56332	'Amotl2'	1.27	6.24	0.71	9.57	0.06	0.82	14.9	3.2	2.36	0.01
56334	'Tmed2'	96.48	101	112	184	61.9	67.57	73	69.27	159.3	48.23
56335	'Mettl3'	8.85	12.25	6.52	2.68	25.4	9.46	9.88	15.18	18.86	7.65
56336	'B4galt5'	2.68	3.13	6.03	0.83	7.76	7.06	1.71	5.32	6.43	2.8
56338	'Txnip'	2.28	3.85	0.1	5.79	0.02	3.94	0.05	1.65	0.03	0.05
56347	'Eif3c'	61.69	90.65	84.3	77.5	54.8	42.07	70.3	61.37	72.23	68.25
56348	'Hsd17b12'	99.37	102.5	92	27.2	57.6	103.6	66.9	68.82	116.8	126.94
56349	'Net1'	0.35	0.58	0.77	9.5	0.09	3.19	0.01	1.33	2.38	0
56350	'Arl3'	221.6	280.8	211	313	295	275.1	254	215.3	234.4	240.48
56351	'Ptges3'	147.9	162.4	130	87.2	135	135.3	105	118.2	156.3	119.34
56353	'Rybp'	5.16	2.94	2.24	10.1	2.38	3.09	1.53	2.34	0.87	5.67
56354	'Dnajc7'	113.3	143.3	177	78.4	141	144.1	155	199.3	125.2	179.39
56356	'Gltp'	11.01	6.1	17	17.1	44.7	24.99	11.8	4.64	9.2	12.06
56357	'Ivd'	46.39	55.58	44.7	47.8	46.5	80.11	61.7	62.9	50.01	57.98
56358	'Copz2'	3.47	0	0	0	3.14	0	0	2.43	0	0
56360	'Acot9'	22.36	17.93	32.8	31.1	28.6	25.17	12	16.03	22.45	24.07
56361	'Pus1'	12.78	15.56	13.4	15.3	4.04	12.35	9.32	13.12	10.22	3.91
56363	'Tmeff2'	92.76	88.61	33.5	31.4	21	24.8	54.5	59.77	4.32	18.88
56364	'Zmym3'	22.84	22.4	21.7	29.2	43.5	28.42	39.5	23.31	37.91	26.96
56367	'Scoc'	266.5	240.6	180	168	169	233.3	215	218.5	182.2	180.54
56368	'Cyb561d2'	39.73	39.95	44.1	30.7	27	9.18	68.4	39.05	55.76	49.82
56369	'Apip'	47.1	30.81	13.2	12.4	16.7	46.09	23.1	26.51	19.43	20.78
56370	'Tagln3'	587.3	531.4	447	800	618	598	574	650.9	445.4	481.28
56371	'Fzr1'	35.05	13.29	17.7	47.9	47.2	30.14	25.3	32.12	30.53	21.85
56372	'1110004F10Rik'	255.6	212.5	201	266	234	173.2	208	238.6	256.3	233.16
56373	'Cpb2'	0	0	0	0	0	0.16	0	0.14	0	0
56374	'Tmem59'	479.6	431.9	336	406	368	582.5	434	477.4	523.8	598.52
56375	'B4galt4'	13.14	16.09	13.1	0.03	9.51	12.38	11.5	17.85	16.1	7.32
56376	'Pdlim5'	0.11	0.16	5.06	0.13	7.92	4.5	3.44	1.88	18.5	5.23

56378	'Arpc3'	165.5	174.2	96.1	82.1	167	148.1	129	186	205.3	148.06
56379	'Kcnj1'	0	0	0.02	0	0	0	0	0	0	0
56380	'Arid3b'	0.93	0	0.39	0.18	1.55	0	0	1.25	0	1.62
56381	'Spen'	1.55	0.38	0.61	1.78	0.97	1.38	2.27	1.45	1.9	1.32
56382	'Rab9'	25.41	32.66	37.9	28.9	49.3	19.95	14.9	32.94	16.45	25.57
56384	'Letm1'	28.97	36.12	35.8	58.7	20.3	15.68	27.6	16.42	56.28	15.62
56386	'B4galt6'	9.29	3.43	6.23	8.27	9.64	4.42	5.89	3.81	9.27	4.34
56389	'Stx5a'	61.24	44.58	29.8	34.6	29.8	38.59	38	33.57	47.23	38.09
56390	'Sssca1'	125.5	105.9	129	152	96.1	129.4	103	117.2	103	104.48
56392	'Shoc2'	9.65	8.45	8.99	15.3	9.88	5.85	7.69	9.79	18.04	11.05
56395	'Tmem115'	27.32	28.59	25.9	20.1	34.7	29.86	21.1	22.34	16.15	20.78
56397	'Morf4l2'	316.5	388.4	313	388	230	310.1	228	275.6	322.6	252.61
56398	'Chp1'	63.92	58.79	74.9	36.4	63.9	72.5	79.6	66.37	69.53	64.82
56399	'Akap8'	22.93	23.78	21.2	22.8	15.3	20.74	41.7	14.68	34.96	32.52
56401	'P3h1'	3.71	2.21	5.71	4.19	0.64	0.11	2.82	1.76	1.08	0.07
56403	'Syncrip'	7.75	15.74	4.7	17	13	12.35	28.3	11.49	15.71	15.81
56404	'Trip4'	3.73	4.75	13.3	7.38	3.25	5.26	1.89	2.04	8.19	5.11
56405	'Dusp14'	22.05	26.17	9.33	70.6	23.4	7.78	26.7	21.23	15.88	22.85
56406	'Ncoa6'	1.28	1.33	1.31	0.26	3.7	0.71	0	2.73	1.03	2.64
56407	'Trpc4ap'	21.45	14.19	15	6.8	19.7	14.49	6.3	14.53	35	14.51
56409	'Nudt3'	60.17	39.06	29.9	64.3	61.3	39.35	49.1	42.09	61.81	32.36
56410	'Cbln3'	0.02	0.12	0.2	0.11	0.11	0.23	0.08	0.06	0.12	0.12
56412	'Noa1'	10.39	13.16	7.39	9.6	2.95	4.26	8.43	3.94	9.99	10.8
56417	'Adar'	16.6	16.75	37.5	14.4	13.5	7.14	30.8	17.4	15.81	18.09
56418	'Ykt6'	21.07	24	24.6	15.8	24.4	32.68	33.1	23.62	26.35	25.16
56419	'Diaph3'	1.52	2.34	0.36	3.2	1.74	0.01	0	2.44	1.64	0.73
56420	'Ppp4c'	33.25	18.99	26.3	31.3	43.9	16.93	19.9	31.61	12.71	26.61
56421	'Pfkp'	114.3	72.5	81.9	49.8	68.2	46.39	124	129	98.01	95.52
56422	'Hbs1l'	23.97	31.8	25.4	29.8	37.4	16.1	44.4	22.75	18.51	37.73
56424	'Stub1'	338.6	293.5	320	367	308	411.1	339	320.4	353.1	157.32
56426	'Pdcd10'	47.96	45.15	45.4	25.5	52.6	66.18	58.8	54.19	55.32	72.71
56427	'Tubd1'	11.49	0	13.5	8.5	6.81	7	18.8	2.88	9.31	4.41
56428	'Mtch2'	129.4	73.85	117	53.1	111	134.7	119	124.8	106.9	111.11
56429	'Dpt'	0.98	0	0.3	0	0	0	0	0.37	0	0
56430	'Clip1'	14.12	29.44	16.6	26.3	18.8	29.95	19.7	17.01	18.97	25.18
56431	'Dstn'	147.2	150.3	104	152	128	114.3	140	88.58	92.94	147.67
56433	'Vps29'	246.9	234.8	221	309	284	270.4	215	322.9	246.8	218.3
56434	'Tspan3'	561.7	609.5	628	567	394	618.7	473	595.6	529.2	524.7
56436	'Adrm1'	119.5	120.7	181	212	141	159.5	188	161.4	166.3	121.08
56437	'Rrad'	10.9	28.82	27.1	93.6	17.2	1.18	6.55	1.47	13.91	7.88
56438	'Rbx1'	211.3	202.9	216	288	206	295.3	191	196.9	268.2	219.48
56440	'Snx1'	38.98	36.31	65.4	49.6	52.2	54.46	79.8	47.38	62.7	54.63
56441	'Naa80'	19.99	16.16	12.9	33.6	15.1	22.98	22.3	14.75	33.6	18.65
56442	'Serinc1'	324.8	399.1	355	289	273	207.7	344	358.1	373.1	260.62
56443	'Arpc1a'	129.4	171.6	165	70.1	183	205.9	205	175.3	241.4	260.38
56444	'Actr10'	52.4	57.41	57.3	71.7	50.4	67.9	54.6	32.86	59.08	36.38
56445	'Dnaja2'	89.49	69.52	82.5	94.4	63.1	77.6	71.7	86.5	65.46	88.81
56447	'Copz1'	138.4	130	136	147	151	207.9	199	180.7	163.9	160.99
56448	'Cyp2d22'	0.76	3.62	0	0	0.12	11.04	0	4.84	3.07	3.45
56449	'Ybx3'	2.76	1.93	1.11	0	0.78	3.28	0	0.24	0	0.26
56451	'Suclg1'	159.6	186.6	186	207	193	358.2	287	220	206.4	212.13
56452	'Orc6'	16.94	27.14	23.8	12.1	20.8	33.6	11.2	26.89	14.59	17.14
56453	'Mbtps1'	18.81	17.78	18.4	17.1	36.3	4.3	34.3	25.94	24.23	36.06
56454	'Aldh18a1'	11.86	11.86	4.29	11.7	12.4	8.76	8.49	9.19	10.15	6.63
56455	'Dynll1'	251.4	242.2	229	188	255	271.7	189	215.8	174.7	234.11
56456	'Actl6a'	5.63	4.55	8.96	1.65	11	8.96	5.69	10.39	6.85	14.16
56457	'Clptm1'	47.8	34.52	84.5	103	41.7	19	59.6	47.93	61.91	10.76
56458	'Foxo1'	0.3	2.76	0	1.64	4.07	5.14	0	1.72	0.39	3.07

56459	'Sae1'	119.1	145.7	94.5	73.6	117	127.2	132	130.6	117.5	132.35
56460	'Pkp3'	0	0	0	0	0	0	0	0	2.51	5.03
56461	'Kcnip3'	19.3	13.63	59.1	10.3	0.32	29.23	57.4	17.14	11.9	31.4
56462	'Mtch1'	132.6	101.4	130	126	164	164.6	176	102.7	92.62	96.52
56463	'Snd1'	42.2	40.64	36.6	60.5	20.4	30.29	20.2	30.19	27.68	55.5
56464	'Ctsf'	46.49	78.02	78.4	35.4	64.9	78.87	48.2	58.2	86.1	71.8
56468	'Socs5'	10.45	13.59	6.46	8.04	6.87	4.02	1.9	4.43	10.5	5.8
56469	'Pias1'	14.39	32.85	16.4	9.65	25.9	14.7	28.7	23.1	33.87	27.08
56470	'Rgs19'	49.19	41.09	40.9	37	23.1	45.3	52.5	64.54	57.59	79.29
56471	'Stmn4'	166.2	125.7	77.6	155	185	128.5	91.3	122.3	82.36	110.6
56473	'Fads2'	33.05	40.45	24.3	14.4	11.3	34.7	31.1	17.45	47.24	4.04
56480	'Tbk1'	34.1	12.64	22.6	35.9	20.2	18.86	27.6	24.99	22.92	40.56
56484	'Foxo3'	0.4	1.34	1.98	1.14	0.99	0.48	2.84	1.4	2.87	1.96
56485	'Slc2a5'	0	0	0	0	0	6.24	0	0	2.72	1.71
56486	'Gabarap'	287.9	251.1	249	243	253	270.8	187	254.2	265.9	277.6
56488	'Nxt1'	17.26	9.86	8.49	33.9	20.9	4.25	10.8	17.11	20.58	10.53
56489	'Ikbke'	0	2.15	0	0	0	0	0	0	0.74	0.49
56490	'Zbtb20'	69.24	48.78	38.2	52	14.9	13.41	13.8	23.41	18.07	25.46
56491	'Vapb'	6.12	14.92	6.91	10.2	6.63	6.56	9.13	5.2	6.14	5.27
56494	'Gosr2'	52.7	67.81	57	54.5	62.7	31.56	61.7	52.02	35.47	84.34
56495	'Asna1'	70.36	69.11	88.7	58.8	114	56.06	52.3	88.76	95.98	65.9
56496	'Tspan6'	68.23	67.97	64.5	62.6	27.8	70.11	45.3	60.04	38.92	81.08
56501	'Elf4'	0	0.63	0	0.01	0	0	0	0	1.44	0.48
56503	'Ankrd49'	6.21	0.63	4.39	0.62	5.31	7.53	9.68	5.18	6.87	3.19
56504	'Srpk3'	3.28	2.31	3.11	0.09	4.46	3.35	0	0	13.29	0.02
56505	'Ruvbl1'	72.54	88.99	62	68.1	63.3	79.74	66.9	53.69	84.89	79.99
56506	'Cib2'	81.28	128	79.4	83.7	42.1	89.13	134	43.74	61.22	34.79
56508	'Rapgef4'	26.32	17.49	13.8	29.3	20	16.64	7.71	22.43	10.4	29.27
56513	'Pard6a'	20.46	25.58	46.5	21.3	9.93	17.57	40.5	27.59	47.55	48.06
56515	'Rnf138'	11.29	8.54	6.35	18.6	3.18	1.55	9.44	4.42	15.34	9.42
56516	'Rbms2'	0.3	0	1.15	0	0.52	0	0.03	0	0	0.25
56517	'Slc22a21'	1.51	3.81	3.15	7.52	6.32	2.71	4.9	3.7	11.73	3.24
56520	'Nme4'	0.11	11.28	3.79	10.8	3.22	14.27	0	4.1	13.62	27.72
56523	'Pmfbp1'	0.01	0.28	0.03	0.03	0.04	0.32	0.29	0.01	1.28	0.04
56524	'Mpp6'	2.1	5.01	2.2	3.96	1.78	2	1.17	2.49	0.63	1.79
56525	'Zfp235'	6.61	0.91	3.82	5.85	5.98	2.84	8.45	6.67	5.81	3.3
56526	'Sept6'	12.8	12.84	30.8	25.1	23.5	5.15	9.27	11.25	10.76	14.55
56527	'Mast1'	13.24	18.1	31.1	9.85	14	6.22	14.7	15.34	26.2	11.67
56529	'Sec11a'	114.3	91.22	52.3	156	83.2	110.4	53.6	73.54	104.5	73.33
56530	'Cnpy2'	181.1	285.6	169	141	152	241.3	161	194.3	265.1	214.4
56531	'Ylpm1'	6.88	9.15	8	13.4	17	9.27	16.8	6.06	12.97	7.35
56533	'Rgs17'	17.63	15.56	14	11.6	14.3	14.65	10.1	15.05	12.26	9.08
56534	'Hspb3'	0	20	0.13	0	1.66	0	0	0.05	0	3.88
56535	'Pex3'	20.19	15.77	20	3.01	9.9	27.07	14.2	13.87	17.54	28.07
56541	'Habp4'	35.45	21.08	9.13	104	20.6	23.91	45.4	19.97	44.97	18.65
56542	'Ick'	0.23	1.15	1.54	0	0.77	0.17	0.27	0.27	4.21	0
56543	'Kcnd3'	1.66	2.8	5.93	10.4	2.31	2.23	8.78	3.12	2.34	1.54
56544	'Vmn2r1'	0.01	0.15	0.19	0	0	0.19	0	0	1.25	0
56550	'Ube2d2a'	63.69	67.76	58.1	81.8	64.6	40.65	43.9	70.32	74.66	50.28
56551	'Txn2'	210.4	231.3	184	339	245	301	197	177.1	269.6	312.18
56612	'Pfdn5'	277.9	201.2	292	312	205	290.6	212	212.4	169.8	219.82
56613	'Rps6ka4'	6.27	16.26	10.5	0.02	5.62	11.79	4.05	6.82	10.77	20.03
56615	'Mgst1'	14.2	12.45	0.32	9.85	8.14	18.09	21.8	10.6	13.7	3.54
56620	'Clec4n'	0.04	0	0	0	0	6.47	0	0	0	0
56622	'Adam21'	0	0	0	0	1.71	0.25	0	0	1.42	0
56626	'Poll'	14.96	15.33	1.72	0	13.2	9.4	8.18	3.3	15.78	8.81
56631	'Trim17'	6.65	10.51	11.9	11.2	14.8	8.41	7.11	8.59	11.64	10.78
56632	'Sphk2'	6.02	8.83	11.7	0.68	6.49	4.61	6.86	3.45	7.18	6.43

56636	'Fgf21'	0	0	0	0	0	0.05	0	0	0	0.05
56637	'Gsk3b'	75.32	87.73	45.3	96.2	90.3	68.64	56.8	93.97	69.21	77.49
56642	'Ankrd2'	1.65	6.52	21	0	22.2	16.09	0	1.84	25.46	6.48
56643	'Slc15a1'	0	0	0	0	0	0	0	1.07	0	0
56690	'Mlycd'	1.93	1.96	8.82	4.73	5.46	5.14	3.5	8.84	11.34	2.09
56692	'Lamtor3'	100.8	44.28	70.6	67	53	81.11	75.7	68.07	93.72	79.85
56693	'Crtap'	4.74	10.02	1.89	7.47	7.93	4.62	0.03	7.42	15.91	0
56695	'Pnkd'	82.52	93.7	128	122	105	71.58	100	98.94	123.2	83.66
56697	'Akap10'	2.03	5.28	3.43	6.26	1.77	0.5	2.86	3.49	7.45	0.59
56698	'Phax'	46.93	33.44	51.5	22.6	54.4	51.56	34.7	50.12	48.91	34.56
56699	'Cdc42ep4'	5.96	14.39	7.76	16.4	8.63	13.72	9.95	16.87	3.32	6.64
56700	'Glmp'	34.2	24.54	29.3	10.9	23.4	42.26	12.8	42.08	56.28	33.58
56702	'Hist1h1b'	0	0	0.13	0	0	0	0.27	0	0	0
56703	'Pigo'	12.21	11.86	7.98	2.1	12.9	4.45	5.99	6.27	6.24	5.7
56705	'Ranbp9'	0.27	4.07	3.3	3.94	4.48	0.47	2.94	0.01	8.6	5.45
56706	'Ccnl1'	23.99	37.9	40.8	63	14.8	39.28	23.1	22.73	14.63	22.69
56707	'Zfp111'	0.14	1.97	0.23	2.06	0.39	0.35	1.89	0.39	3.29	2.97
56709	'Dnajb12'	7.12	16.15	14.4	0.06	9.69	17.2	14.7	13.25	11.07	10.15
56710	'Brinp1'	22.25	17.75	56.5	25.4	48.4	55.67	73	40.08	103.4	64.21
56711	'Plag1'	0	0	0.02	0	0.03	0	0.07	0.15	0.3	0.12
56715	'Rabgef1'	17.4	19.44	16.1	14.7	17.8	9.02	7.24	19.7	16.92	28.17
56716	'Mlst8'	9.16	15.15	6.8	2.98	10.8	2.88	13.1	11.14	12.43	12.79
56717	'Mtor'	2.44	3.09	10.8	4.21	2.53	2.14	4.93	2.82	2.45	2.4
56720	'Tdo2'	0	0	0	0	0	0	0	0	3.23	0
56722	'Litaf'	8.49	10.16	5.33	0	4.75	33.71	0.02	1.38	0.02	0.82
56724	'Cript'	107.2	95.2	60	100	59.3	101.9	47.2	86.94	59.36	100.94
56726	'Sh3bgrl'	21.05	22.11	35.6	8.22	30.7	14.97	18.2	27.34	43.5	29.8
56734	'Tulp2'	0	0.34	0	0	0.77	0.19	1.56	0	0.08	0.17
56735	'Krt71'	0	0	0	0	0	0	9.5	0	0	0
56736	'Rnf14'	175	187.5	227	104	149	145.7	185	218.9	212.5	210.71
56737	'Alg2'	164.6	227.8	230	185	178	148.8	199	250.2	186.2	282.06
56738	'Mocs1'	1.36	7.6	3.17	0	6.52	3.49	0	4.78	8.69	3.23
56739	'Rec8'	16.31	12.86	14.9	11.5	13	3.58	9.7	2.31	3.5	13.01
56741	'Igdcc4'	6.15	2.43	4.45	4.01	4.07	1.94	3.06	1.94	4.32	4.53
56742	'Psrc1'	0	0	0	0	0	0.16	0	0	0	0
56743	'Lat2'	0.15	3.15	1.54	0	2.4	33.59	13.3	7.65	5.19	0
56744	'Pf4'	0	0	0	0	3	0	0	0	0	0
56745	'C1qtnf1'	0	1.05	0.07	0	0	0	0	1.14	0.01	0
56746	'Tex101'	0	0	0	0	0	0.04	0	0	0	0
56747	'Sez6l'	15.44	28.37	39	43.2	6.81	6.44	17.7	28.97	2.92	5.22
56748	'Nfu1'	217.5	201.9	158	248	171	169.8	199	236.7	161.8	192.24
56749	'Dhodh'	29.25	25.01	16.2	7.6	27.3	24.83	30.5	18.93	24.91	17.39
56752	'Aldh9a1'	52.34	21.74	37.5	30	24.7	21.11	17.9	23.21	27.95	21.33
56753	'Tacstd2'	0	5.21	0	0	0	0	0	0	13.46	1.97
56758	'Mbnl1'	16.22	17.57	12.8	21.9	21.2	12.1	27.3	15.38	13.97	18.36
56771	'Med20'	41.39	22.42	27.9	41.9	20.1	54.16	43.6	29.48	47.7	46.79
56772	'Mllt11'	243.3	212.8	173	264	196	316.1	192	348.7	227.3	235.81
56773	'Chst5'	2.03	0	1.4	10.1	0.64	4.52	0	6.98	8.07	2.32
56784	'Ralgapa1'	2.75	3.7	5.23	8.71	5.28	3.02	1.48	2.01	3.11	4.75
56786	'Tmem9b'	109.6	141.8	139	90.1	109	173.9	178	166.8	149.8	94.52
56788	'Scube2'	0	2.14	0.03	7.43	0.4	0	6.52	0.98	0.01	0.97
56790	'Supt20'	24.35	17.36	8.17	24.6	9.81	4.32	9.09	10.07	14.74	18.38
56791	'Ube2l6'	3.28	3.13	8.53	45.7	7.65	4.13	0.07	4.09	4.69	4.73
56792	'Stap1'	0.1	0	0	0	0.81	0.31	1.36	0	0.01	0
56794	'Hacl1'	4.41	1.97	0.02	15.9	7.35	3.96	7.39	0	5.07	1.63
56795	'Arl10'	70.79	56.27	110	41.8	20.2	41.1	89.9	29.44	46	13
56805	'Zbtb33'	0.75	0.05	0.88	0	0.47	0	0	0.47	0	2.11
56807	'Scamp5'	124.5	104.9	176	39.4	82.6	85.78	150	97.78	91.42	130.79

56808	'Cacna2d2'	2.06	0.9	1.89	1.58	5.89	3.41	2.73	1.46	6.06	2.79
56809	'Gmeb1'	5.81	3.25	5.14	3.22	6.69	4.06	8.81	1.85	6.5	6.37
56811	'Dkk2'	0	0	0	0	0	0	0	0	1.61	1.77
56812	'Dnajb2'	45.41	54.2	45.5	36.3	37.3	38.61	25.6	27.04	32.1	32.8
56838	'Ccl28'	0	0	0	0	0.07	0	0	0	0.3	0
56839	'Lgi1'	13.98	22.45	9.9	0	11.2	22.97	31.1	21.33	9.67	11.82
56844	'Tssc4'	90.51	55.4	81.6	105	71	105.6	54	82.73	95.1	90.91
56846	'Necab3'	14.07	20.6	52.7	8.76	26.1	27.54	19	38.63	14.65	13.03
56856	'Insm2'	1.36	2.12	0.17	4.01	0	2.3	0	0	2.21	11.04
56857	'Slc37a2'	0	0	0	0	0.63	0	0	0	0.01	3.18
56863	'Cldn9'	0	0	0.03	0	34.1	0	0	3.5	0	0
56868	'Psg23'	0.16	0.35	0.49	0.94	0.23	0.15	0.28	0.14	0.14	0.37
56869	'Zfp109'	0.51	7.76	0	4.79	0	0.49	0	0	0	0.02
56872	'Pate4'	0.72	0	0	0	0	0	0	0	0	0.13
56873	'Lmbr1'	10.3	13.7	29.5	4.67	18.2	18.26	22	19.37	17.5	15.86
56874	'Rnf32'	10.45	6	6.07	22.1	9.37	13.68	23.8	10.44	6.39	7.52
56876	'Nsmf'	12.85	8.57	18.8	5.33	11.1	5.27	17.8	12.11	12.47	9.13
56878	'Rbms1'	0	3.28	0.74	10.3	15.7	6.73	13.6	0.41	10.03	7.18
57014	'Htr3b'	0	1.17	0	0	0	0	0	0	0	0
57028	'Pdxp'	88.33	77.93	47.5	172	96.4	154.4	94.2	51.94	57.7	65.31
57080	'Gtf2ird1'	8.18	3.89	6.56	6.98	7.87	7.55	1.43	6.4	13.94	12.75
57138	'Slc12a5'	34.94	14.21	38.7	11	24.4	17.18	36.9	22.38	22.89	8.77
57170	'Dolpp1'	22.67	21.47	13	29.6	10.7	20.87	11.9	26.1	24.51	15.8
57230	'Sap30bp'	27.66	43.6	25.6	17.9	23.6	35.14	33.3	26.45	33.02	31.77
57246	'Tbx20'	0	0	0	0.01	0	0	0	0	0	0
57247	'Zfp276'	5.6	5.64	2.04	0	9.3	6.51	0	1.97	0	2.86
57249	'Gabrq'	31.64	16.21	20.8	15	9.21	8.01	2.41	19.73	18.78	25.64
57253	'Tas2r108'	0	0	0	0	0	0	0	0	3.28	0
57257	'Vav3'	0	0.2	0	0.03	0.76	1.43	0.04	0	3.28	0.01
57258	'Xpo4'	2.04	1.05	0.66	0.78	2.56	3.24	3.87	0.43	0.52	1.29
57259	'Tob2'	1.62	3.28	2.22	8.07	4.23	1.68	2.85	4.38	1.86	6.9
57260	'Ltb4r2'	0.01	0.01	0.03	0.03	0.1	1.39	0.2	1.24	0.18	0.15
57261	'Brd4'	12.74	11.02	12.5	26.9	22.1	21.24	11.6	14.1	16.45	18.02
57264	'Retn'	0	0	0	0	0	0.09	0	1.89	1.3	1.45
57265	'Fzd2'	0	1.87	0.31	0	0	0.9	0	0.8	0.36	0
57266	'Cxcl14'	15.85	7.6	6.93	23.4	87	11.26	18.5	5.97	0.67	12.47
57267	'Apba3'	16.65	23.45	25.6	7.06	13	15.96	28.2	5.46	0.16	11.1
57274	'Slc16a8'	0	0	1.08	0	0	1.94	0.41	0	0	0
57275	'Lenep'	2.92	6.39	12.8	0	12.6	5.41	2.49	10.02	7.72	10.62
57276	'Vsig2'	0	0	0	0	0.22	0	0	0	0	0.04
57278	'Bcam'	19.42	1.79	16.5	14.9	2.32	0.32	2.67	16.31	31.28	7.52
57279	'Slc25a20'	22.12	22.76	4.18	6.98	20.7	39.37	4.88	14.38	1.26	5.77
57294	'Rps27'	466.7	536.2	631	1059	272	507.8	374	143.8	388.3	295.52
57295	'Icmt'	7.16	4.99	10.6	1.93	5.72	0.1	1.54	4.5	14.68	8.37
57296	'Psmd8'	158.3	138.9	120	186	131	154.4	123	167.9	116.3	139.8
57312	'Mrps31'	32.48	25.7	24.4	20	18.3	23.33	21.5	43.22	25.47	31.11
57314	'Nelfcd'	58.67	39.64	42.3	45	54.1	25.78	29.8	78.36	69.22	40.32
57315	'Wdr46'	16.1	6.12	6.26	8.76	10.6	27.12	14.4	11.36	10.34	14.05
57316	'C1d'	140.7	102.6	108	191	111	157.7	129	113.8	110.5	158.42
57317	'Srsf4'	30.33	22.2	33.1	22.2	21.8	29.36	31.5	12.79	16.26	12.65
57319	'Smpdl3a'	14.2	4.83	7.7	26.3	0.14	24.77	0	7.11	9.2	10.34
57320	'Park7'	403.4	319	331	414	417	582.4	432	482.2	302.6	424.59
57321	'Terf2ip'	41	52.95	46	46.3	39.3	70.05	70.9	45.97	45.96	52.31
57329	'Otor'	0	0	0.77	0	0	0	0	0	0	0
57330	'Gigyf1'	1.77	3.28	5.02	2.65	6.98	5.96	2.2	1.1	7.79	3.5
57339	'Jph1'	0.13	0.64	0.25	0.09	0.22	0.23	0.05	0.2	0.05	0.2
57340	'Jph3'	79.56	76.15	90.4	12.8	67.7	79.07	60.2	97.22	64.76	75.59
57342	'Parva'	4.45	12.26	17.3	1.84	7.93	0.33	0.02	2.99	3	9.89

57344	'As3mt'	85.54	55.1	31.7	59.2	45.9	56.91	6.62	39.8	80.1	55.76
57354	'Cramp1l'	0.7	0.26	0.37	5.53	1.4	0.21	1.97	1.42	0.98	0.2
57355	'BC051019'	3.01	0.06	0	0.2	0.12	0	0.12	0.05	0	0.13
57357	'Srd5a3'	5.8	15.57	23.6	14.3	8.82	27.15	24.2	16.51	5.34	24.44
57370	'B4galt3'	40.46	53.03	39.7	24.1	43.7	30.39	25	32.51	56	53.98
57373	'Akip1'	0	0	0.08	44.8	5.19	33.75	0	9.57	13.84	7.53
57376	'Smarce1'	19.97	14.26	15.7	24.1	13.5	19.94	12.2	16.29	13.8	18.73
57377	'Mogs'	0.32	4.6	4.94	0.05	1.61	0.02	0.77	1.18	3.58	3.35
57385	'P2ry4'	0.01	0.03	0.02	0.04	0.04	0	0.03	0	0	0.01
57423	'Atp5j2'	642.2	480.8	553	508	507	812.5	603	430.4	643.2	466.86
57431	'Dnajc4'	9.39	18.62	35.2	9.82	19.1	24.46	32.5	26.74	33.85	28.21
57432	'Zc3h8'	9.1	6.29	6.97	2.3	12.1	4.42	3.55	3.27	3.48	8.39
57434	'Xrcc2'	2.19	2.45	6.44	0.04	4.39	7.47	5.36	1.02	0.09	3.84
57435	'Plin4'	0.01	1.4	0.02	0.01	0	0.01	0.01	0	0.01	0.29
57436	'Gabarapl1'	519.8	471.2	671	471	567	629.6	571	623.3	473.2	436.71
57437	'Golga7'	59.33	43.21	45.9	68.4	94.1	96.8	60.5	58.09	50.85	48.25
57438	'March7'	15.94	17.82	15.5	33.6	6.58	2.48	10.6	10.72	9.84	21.34
57439	'Tmem183a'	27.46	23	24.8	15.9	16.4	19.24	25.8	24.94	32.95	12.94
57440	'Ehd3'	23.06	15.95	45.6	4.11	25.4	24.45	48	36.89	19.15	25.47
574402	'Gpr17'	0	0	21.5	4.44	0	0.01	24	0.54	0	0
574403	'Fam196b'	0.57	0.14	2.27	0	0	3.05	0.01	2.27	0	2.57
574404	'Gm14685'	0	14.71	0	9.54	3.78	0	0	2.8	4.33	5.27
574405	'DXBay18'	0	4.46	2.94	0	0	11.82	0.13	7.1	0.32	8.98
57441	'Gmnn'	0.72	3.77	14.3	8.34	8.15	0	0.05	7.08	0.14	2.73
574417	'Tas2r137'	0	0	0	0	0	0	0	0	0.04	0
574418	'Serinc4'	0.03	0	0.1	0	0	0	0	0.17	0	0
574428	'Zmynd15'	0	0.45	0	0	0	0	0	0.03	0.02	0
57443	'Fbxo3'	83.78	72.36	58.2	55.9	53.4	70.22	62.7	69.08	75.41	90.96
574437	'Xlr3b'	16.43	36.34	18.6	0.04	0.09	22.12	36	14.11	30.5	16.35
57444	'Isg20'	0	0	0	0.07	0	0	0	5.38	0	0
57738	'Slc15a2'	2.85	0.54	5.62	0.98	3.78	0	16.2	0	0	0
57740	'Stk32c'	3.59	13.22	0.15	0.03	2.55	0.15	1.68	2.22	4.07	0
57741	'Noc2l'	3.88	3.18	4.61	0	2.05	4.56	5.42	7.21	8.66	5.08
57743	'Sec61a2'	73.68	66.26	73.5	71.9	55	71.76	53.4	71.38	95.41	86.83
57745	'Zfp112'	0	6.35	6.9	0	2.33	0	3.46	5.64	0.26	5.71
57746	'Piwil2'	0	0	0.02	0	0	0	0	0	0	0.01
57748	'Jmy'	1.65	0.55	0.37	0.52	1.9	0.01	0.31	0.29	1.25	0.76
57750	'Wdr12'	41.21	37.43	52.6	55	35.1	36.64	65.4	47.79	54.66	34.34
57751	'Rnf25'	82.83	44.76	32.6	53.1	85.4	74.85	54.4	66.67	52.67	55.06
57752	'Tacc2'	15.62	13.47	13.4	8.63	11.5	11.07	23.8	15.89	12.09	6.33
57753	'Noc3l'	1.66	0.06	2.13	0.03	1.51	1.19	0.28	0.45	0.16	1.1
57754	'Cend1'	275.7	190	402	218	301	281.2	378	399.8	302.8	206.38
57755	'Dnajb7'	0	0	0	0	2.89	0	1.86	0	2.86	0
57756	'Fhl5'	0	0	0.05	0	0	0	0	0	0	0
57757	'Pglyrp2'	0	0	0	0	1.63	0	0	0.15	0	0
57773	'Wdr4'	4.51	5.8	7.68	9.47	8.56	12.33	2.55	3.82	7.06	18.29
57775	'Usp29'	12.78	11.83	14.7	9.84	4.96	11.73	10.4	12.86	8.18	11.28
57776	'Ttyh1'	20.95	12.67	14	3.18	22.9	13.82	32.9	13.13	10.39	3.39
57778	'Fmnl1'	1.39	3.23	0.99	0	3.19	0.06	1.72	2.4	1.97	0.48
57780	'Fxyd7'	340.1	175.1	194	205	192	321.5	165	307.3	106.1	103.01
57782	'Rbak'	1.98	2.14	5.24	13.1	6.54	0.5	0.13	1.92	1.45	1.75
57783	'Tnip1'	8.6	7.81	10.7	0.02	13.1	8.44	3.64	12.45	4.22	7.07
57784	'Bin3'	0	6.52	6.07	10.8	5.52	18.81	9.51	7.96	6.67	13.76
57785	'Rangrf'	22.39	5.7	21.5	13.8	21.4	18.18	20.8	10.67	18.43	6.87
57808	'Rpl35a'	252.6	286.3	239	357	199	270.9	286	144.7	168.3	169.41
57810	'Cdon'	0.13	0	0.61	0	0.02	1.03	0.1	0.11	0	2.13
57813	'Tk2'	48.14	20.31	43.5	63.5	50.1	33.04	51.8	47.89	69.07	20.41
57815	'Spata5'	5.77	4.85	8.25	0.02	2.56	4.66	0.6	5.9	15.19	12.57

57816	'Tesc'	6.22	13.87	8.74	12.3	3.31	0.05	6.95	7.15	17.71	3.17
57837	'Eral1'	11.67	9.24	21.6	5.81	24.3	4.4	6.07	12.24	5.1	16.29
57869	'Adck2'	7.8	13.79	18.3	15.9	0.34	10.58	6.38	26.1	19.57	15.44
57874	'Hacd3'	149.2	120.1	139	62.1	109	166.2	137	145.7	207.4	184.8
57875	'Angptl4'	0.02	10.84	0.03	0	0	6.04	0.03	0	3.75	2.2
57890	'Il17re'	0.51	0.73	0.43	0	1.13	0	2.3	0.58	0	0
57895	'Ccdc126'	8.61	10.56	17.6	5.47	9.53	9.07	13.2	6.51	20.71	7.93
57896	'Krcc1'	1.58	2.53	0.97	5.96	5.12	0	4.9	2.71	2.93	5.34
57905	'Isy1'	12.47	15.98	26.2	10.7	7.45	31.47	32.2	22.52	30.31	23.79
57908	'Zfp318'	2.71	1.23	3.5	4	5.19	1.5	1.09	2.21	2.46	1.86
57911	'Gsdma'	0	0	0	0	0	0.17	0	0	0	0
57912	'Cdc42se1'	17.74	6.71	18.3	19.8	19.1	9.48	11.1	6.08	14.26	15.55
57913	'Pidd1'	0.04	1.43	3.85	0	0.02	0	0.07	2.92	0.03	0.01
57914	'Crlf2'	5.48	8.98	6.59	6.49	6.22	21.41	0.61	4.32	5.48	13.86
57915	'Tbc1d1'	4.43	3.39	5.34	10.8	2.43	7.75	4.67	1.64	1.32	8.15
57916	'Tnfrsf13b'	2.74	1.36	0.26	4.7	0.14	0	0	0	0	0.42
58172	'Sertad2'	3.41	1.95	4.89	2.99	1.02	3.57	3.83	2.23	4.03	0.94
58175	'Rgs20'	23.32	11.66	12.1	24.8	27.4	21.17	15	26.67	7.31	13.46
58178	'Sorcs1'	6.98	4.5	3.61	0.11	8	1.51	5.11	5	7.8	12.11
58180	'Hic2'	0.01	0.85	2.33	2.56	1.45	0	1.6	1.65	0.51	1.24
58182	'Prokr1'	0	0.18	0.8	0	0	0	0	0	0	0
58184	'Cnot9'	18.41	17.55	22	16.6	18.9	8.35	15.6	19.3	9.98	14.37
58185	'Rsad2'	0	1.88	1.97	0.62	3.44	0	0	0	0	0.01
58186	'Rad18'	6.11	5.24	6.17	6.04	9.72	8.08	6.99	11.2	1.8	0.02
58187	'Cldn10'	36.73	46.51	22.9	68.8	10.2	49.79	12.3	24.21	24.89	4.75
58188	'Vstm2b'	15.47	7.3	21.2	29.7	13.1	22.96	14.9	14.85	13.84	10.42
58193	'Extl2'	39.64	39.75	67.6	64.6	49.4	37.46	31.1	60.74	65.26	56.02
58194	'Sh3kbp1'	9.69	16.12	12.7	11.4	6.56	8.19	15	11.21	12.54	10.43
58198	'Sall1'	0.92	0	1.53	1.43	0	0.27	0	0	0	0.3
58200	'Ppp1r1a'	21.86	23.01	22.4	24.9	30.2	32.46	35.3	24.75	20.98	11.34
58202	'Nelfb'	31.59	46.79	67	17.5	50.4	46.16	58.4	69.31	42.17	32.16
58203	'Zbp1'	0	0	0	0	0	14.57	0	0	0	0
58206	'Zbtb32'	0	2.28	0	0	0	0	0	0	0	2.63
58207	'Slc43a3'	0	0	0.02	0	0	0	0	0	0	0.02
58208	'Bcl11b'	0	0	0	0	1.67	0.12	1.01	0.28	0	0
58212	'Srrm3'	10.98	25.93	24	25.6	39.8	41.9	31.4	10.41	22.23	16.48
58218	'Trem3'	0	0	1.05	0	0	0	0	0	0	0
58220	'Pard6b'	0	0	1.92	0	0	0.01	0	0	1.64	0
58222	'Rab37'	0	2.62	1.45	0	0.35	0	0	0	0	0.41
58223	'Mmp19'	0	0	0.84	0	0.02	0	0.09	0.64	0	0
58226	'Cacna1h'	0.96	1.96	1.65	5.63	2.29	0.65	1.01	2.17	0.03	0.23
58227	'Fam184b'	4.14	3.04	3.5	1.59	1.78	2.24	0	0.55	1.26	3.91
58229	'Efcc1'	5.44	13.43	12.3	9.45	21.7	10.94	21.1	15.99	7.38	7.17
58230	'Rnf8'	42.18	37.67	45.1	51.4	40.8	33.35	38.3	37.16	43.55	20.24
58231	'Stk4'	5.68	3.17	5.38	2.09	4.97	12.84	8.69	7.65	1.53	20.05
58233	'Dnaja4'	4.79	5.94	0.94	9.44	6.83	5.08	5.91	3.51	16.65	6.85
58234	'Shank3'	0.71	1.38	0.84	1.36	0.1	0.62	0.04	1.21	1.01	0.25
58235	'Nectin1'	1.44	1.84	3.31	0.95	0.52	2.15	4.59	0.65	0.73	1.15
58237	'Nkain4'	62.4	32	44.4	76.8	24.6	101.4	28.9	19.05	48.48	9.92
58238	'Fam181b'	5.62	1.54	7.5	2.17	0	0	0	1.55	0	0
58239	'Dexi'	5.92	6.5	5.03	10.1	9.2	0.33	14	8.23	8.76	2.53
58240	'Hs1bp3'	4.47	6.21	10.7	8.2	6.27	4.49	6.38	3.33	0.11	5.7
58242	'Nudt11'	35.04	24.88	29.5	27.1	7.8	9.89	20.8	8.8	20.15	3.43
58243	'Nap1l5'	1998	2303	1677	1968	1445	2103	2067	2485	2008	1841.75
58244	'Stx6'	13.66	24.74	22.2	10.8	23.4	13.66	20.7	13.31	15.92	0.09
58245	'Gpr180'	21.79	16.55	26.3	22.5	7.59	7.15	21.9	14.05	34.94	9.48
58246	'Slc35b4'	33.13	23.25	26.1	19.1	18.5	19.63	13.2	34.17	17.44	33.92
58248	'1700123O20Rik'	30.19	24.8	16.6	6.38	20.7	21.93	35	12.3	15.1	15.22

58249	'Fibp'	78.88	83.73	58.5	75.9	94.6	118	77.9	78.8	50.93	108.55
58250	'Chst11'	0.67	2.38	1.64	2.37	1.36	0	1.12	2.91	0.21	0
58251	'Cep295nl'	0.09	0.98	0.22	1.29	0	0.32	0	0.09	0.17	0.35
58520	'Erg28'	44.03	46.52	51.8	14.9	53.8	47.62	53.9	56.34	34.66	26.79
58521	'Eid1'	17.76	17	8.25	14.3	10.8	26.54	16.8	19.09	20.32	17.93
58522	'Trim54'	0.03	0.03	0.1	0.04	0.1	0.09	0.07	0	0	0.08
58523	'Elp2'	70.92	56.7	82.7	65.6	47.3	70.15	79.9	65.27	98.5	102.91
58799	'Crbn'	48.04	29.68	11.5	31.6	50.9	20.32	22.2	33.84	37.72	30.16
58800	'Trpm7'	6.62	3.84	6.03	4.78	3.67	0.41	5.69	5.45	3.73	7.09
58801	'Pmaip1'	11.09	0	29.1	0	0	23.69	0	0	12.68	0
58802	'Kcnmb4'	0	7.62	1.17	17.3	0.17	0	0	0.19	0.04	4.42
58804	'Cdc42ep5'	0	0	3.14	0	0	0	7.5	0	0	0
58805	'Mlxipl'	0.42	0.02	0.1	0	0	2.02	0	0	0	0.02
58807	'Slco1c1'	1.31	0.2	0	0	0	0.05	0	3	1.68	0.05
58809	'Rnase4'	3.67	3.45	2.65	0	0	14.32	0.07	0	0.6	9.97
58810	'Akr1a1'	519.6	554.6	537	414	522	678.4	579	556.5	444.7	519.45
58859	'Efemp2'	12.74	2.73	5.35	3.3	5.45	6.47	6.59	8.23	7.75	3.62
58861	'Cysltr1'	1.92	0	0	0	0	2.12	0	0	0	0
58864	'Tssk3'	0	0	0	0	0.44	0	0.75	0	0	0
58865	'Tdh'	0	0	0	0	0	0	0	0	0	0.68
58866	'Treh'	0.49	0	0	0	0	0	1.11	0	0	0
58867	'Syngr4'	0	0.26	0	0	0	0	0	0	0.04	0
58869	'Pex5l'	10.08	7.07	0.62	3	11.2	10.26	9.02	2.42	0.88	2.45
58875	'Hibadh'	61.88	68.97	55.3	99	34.6	42.92	38.1	42.88	59.55	51.76
58887	'Repin1'	16.11	24.27	23.8	6.03	20.6	13.52	26.4	13.73	20	13.66
58909	'Fam13a'	6.37	4.65	4.07	13.4	7.05	3.47	8.12	6.27	8.82	2.84
58911	'Sumf1'	9.17	8.15	4.96	16.1	9.04	3.99	11.1	6.6	13.73	12.84
58916	'Myot'	0.02	2.17	0	0	0	0.04	0.02	0	0	0.02
58988	'Rps6kb2'	12.49	13.58	13.3	9.97	37.6	24.47	22.9	9.15	10.93	26.07
58991	'Ghrl'	0	0	0	0.84	0	0	0	0	0	0
58992	'F12'	0	0	0	4.17	1.15	1.76	0	2.55	3.79	0.06
58994	'Smpd3'	8.32	4.83	8.88	1.7	7.02	1.07	0.04	3.73	3.15	4.89
58996	'Arhgap23'	4.06	2.95	2.41	2.67	4.01	3.25	4.77	2.91	6.79	1.37
58998	'Nectin3'	2.91	0.76	0.17	2.26	2.23	0.02	5.54	0.89	0.03	1.45
59001	'Pole3'	7.13	14.1	15.1	26.2	17.9	12.26	15.6	9.74	11.81	11.47
59002	'Wrap73'	5.46	15.16	29.7	11.5	13.6	26.58	12.8	25.01	29.49	17.29
59003	'Maea'	86.17	59.31	109	71.5	85.6	38.74	74.6	92.46	79.38	81.83
59004	'Pias4'	23.17	10.94	11.9	0	32.1	6.61	16.6	14.72	3.47	8.56
59005	'Trappc2l'	352.1	306.6	325	248	291	536.5	289	312.2	356.7	300.62
59006	'Myoz2'	0.2	0	0.83	0.25	0.22	0.28	0.1	0.04	0.31	0.27
59007	'Ngly1'	12.95	15.17	19.2	15.1	14.6	13.22	10.1	10.21	21.07	19.45
59008	'Anapc5'	122	175.8	119	140	124	116	112	145.5	103.2	137.2
59009	'Sh3rf1'	3.61	3.47	3.16	0.42	0.26	0.08	1.74	4.27	6.59	0.29
59010	'Sqor'	8.97	0	5.15	0	0	9.96	6.79	0	13.09	0.03
59012	'Moxd1'	0.17	10.15	0.09	2.67	0.03	0	0	1.12	0.01	0
59013	'Hnrnph1'	117.8	129.8	166	129	115	97.58	160	111.1	82.02	85.83
59014	'Rrs1'	10.42	11.74	15.4	17.9	14.2	11.62	10.7	9.79	6.87	1.89
59015	'Nup160'	0.48	2.28	3.3	5.32	2.57	0.02	2.63	2.13	4.43	0.3
59016	'Thap11'	10.39	17.1	11.1	55	11.5	4.17	8.16	8.37	21.75	7.02
59020	'Pdzk1'	0	0	0	0	0	0	1.95	0	0	0.18
59021	'Rab2a'	64.09	96.95	61.6	70.5	73.1	63.09	99.1	129.3	104.7	64.06
59022	'Edf1'	422	300.9	343	408	350	462.5	447	383.8	397.1	410.54
59024	'Med12'	0.38	0.99	3.49	1.56	1.66	0.35	0.32	0.09	0.31	1.07
59025	'Usp14'	168.5	164.1	159	104	80.1	130.2	163	158.5	140.1	175.8
59026	'Huwe1'	5.91	6	7.83	8.43	3.61	8.24	8.33	4.26	6.42	3.84
59027	'Nampt'	12.84	10.06	10.4	7.84	14.6	2.38	8.58	5.48	14.48	12.4
59028	'Rcl1'	28.65	45.89	12.5	5.06	19.8	27.33	56.7	32.65	18.04	25.37
59029	'Psmd14'	181.7	211.1	191	198	177	274	160	181.5	215.7	282.9

59030	'Mkks'	19.24	4.63	18.9	5.58	6.38	21.35	25.2	15.67	15.15	9.08
59031	'Chst12'	34.38	45.7	42.3	11.6	18.5	36.12	44.3	62.79	81.19	30.49
59032	'Ppp2r3c'	21.8	26.52	4.79	26.5	11.6	27.81	14	23.62	19.64	18.27
59033	'Slc4a8'	6.21	3.91	7.26	4.47	4.26	3.79	3.5	6.4	7.42	13.23
59035	'Carm1'	0.5	0	0	0	1.19	0.02	1.45	2.02	5.45	1.11
59036	'Dact1'	0.02	1.08	1.27	6.68	0.59	0	0	0	3.54	0
59038	'Pxmp4'	1.98	7.37	0.54	9.7	16.2	2.67	5.17	14.54	1.04	4.7
59040	'Rhot1'	25.99	17.24	16.2	5.02	12.5	12.84	24.4	13.51	16.07	6.68
59041	'Stk25'	181.7	218.5	238	171	230	331.4	275	285.1	255.9	242.44
59042	'Cope'	268.3	254.1	325	446	345	415.1	309	395.1	347.4	396.83
59043	'Wsb2'	22.29	18.86	5.61	33.5	33.6	21.98	10.9	14.28	11.08	26.31
59044	'Rnf130'	10.12	9.02	7.35	1.4	10.3	1.35	11.4	7.31	6.02	3.73
59045	'Stard3'	15.73	25.14	12.9	11.1	20.1	24.94	5.07	12.76	24.07	17.02
59046	'Arpp19'	52.39	41.54	27.5	38.8	35.2	57.74	44.4	49.51	33.78	40.82
59047	'Pnkp'	11.1	12.77	13.9	17.7	41.8	32.22	16.5	19.24	20.55	20.64
59048	'C1galt1c1'	41.76	38.31	15.2	20.7	45.4	47.97	24	49.59	46.15	37.21
59049	'Slc22a17'	198	178.4	150	238	218	255.8	229	265.1	250.1	153.06
59050	'Nsa2'	11.18	7.92	7.64	9.85	6.02	7.81	17.7	12	4.35	10.21
59052	'Mettl9'	31.19	19.32	30.2	51.2	27.8	25.52	7.65	39.42	25.3	28.12
59053	'Hgh1'	20.83	9.46	15.5	2.04	6.87	20.18	16.9	16.98	23.85	8.42
59054	'Mrps30'	20.57	18.71	23.5	54.2	29.4	7.55	44	11.59	11.92	15.23
59056	'Evc'	0.02	1.81	0.05	2.08	0	0	0	0.23	2.35	0.06
59057	'Zfp24'	4.21	13.09	11	18.6	6.43	6.02	22.8	6.79	13.73	8.7
59058	'Bhlhe22'	0	0	0	0	0	2.12	0.25	0.42	1.59	0
59069	'Tpm3'	139.2	209.9	116	182	146	192.2	113	123.6	117.1	150.73
59079	'Erbin'	0.63	0.95	1.71	1.59	2.48	1.08	0.49	0.24	0.03	0.03
59083	'Fetub'	0	3.03	0	0	0	0	0	0	0	0
59090	'Midn'	0.6	1.36	1.99	4	0.23	0.66	1.91	1.08	1.62	0.45
59091	'Jph2'	0	0.72	2.67	3.48	0	0	0.65	2.3	0.02	0
59092	'Pcbp4'	65.61	44.18	54.9	30	55.1	49.94	38.7	54.96	50.47	41.42
59093	'Pcbp3'	41.97	74.98	79.4	64	32.4	40	56.5	60.69	47.07	54.18
59095	'Fxyd6'	295.2	246.4	433	298	269	488.8	466	382.5	442.6	358.39
59125	'Nek7'	2.49	4.56	4.17	20.5	8.37	1.87	0.02	0.47	2.92	2.23
59126	'Nek6'	15.56	37.01	18.4	18.4	12.7	29.35	33.5	23.06	12.77	39.16
59287	'Ncstn'	36.18	36.33	30.9	58.9	44.8	34.2	44.4	50.02	35.45	76.34
59288	'Dctn5'	146.1	122.4	101	113	109	151.1	142	159.7	164.2	141.51
59308	'Emcn'	0.03	0	0	0	0	0	0	0	0	0
594844	'Tceal3'	284.6	159.8	217	267	161	194.2	228	201.6	234.7	141.01
595136	'Ndufs5'	651.6	510.8	638	537	513	765	643	491.5	584.8	502.81
60315	'Myg1'	42.35	42.17	61.1	73.9	66.4	64.64	50.1	35.38	60.03	63.08
60321	'Wbp11'	24.49	18.66	34.9	25.2	37	14.62	31.7	29.19	43.88	44.83
60322	'Chst7'	1.74	0	7.36	0	0	0	8.95	0	0.86	2.01
60344	'Fign'	0	0.3	0	1	0	0	2.08	0	0	0
60345	'Nrip2'	47.28	77.21	63.7	44	89.2	24.23	64.7	76.18	49.99	34.65
60363	'Cldn15'	0	0	2.45	0.87	0	0	0	0	0	0
60364	'Donson'	5.23	1.46	4.84	1.75	4.38	0.17	2.58	0.73	1.46	0.18
60365	'Rbm8a'	53.06	52.8	52.3	76.3	58.3	62.17	30.7	58.82	29.78	57.7
60367	'Il1rapl2'	1.45	0	3.9	7.16	0	0	0.43	0	0	2.38
60406	'Sap30'	0.92	2.5	0	5.89	6.05	0.28	0	2.3	0.07	0
60409	'Trappc4'	238.4	183.4	152	252	191	218.1	215	245.6	246.7	248.79
60411	'Cenpk'	0	0	0.06	0	7.82	0.2	0.12	0	0.04	0.04
60425	'Doc2g'	5.36	4.47	4.85	0.17	20.5	3.61	5.53	2.36	4	4.81
60440	'Iigp1'	0.02	0	0.07	3.9	0	0	0	0	1.58	0
60441	'Mrpl38'	99.22	65.52	86.3	84	66.7	85.7	45.3	97.5	85.18	84.27
60455	'Rxylt1'	0.09	2.43	1.62	0	2.41	0.45	0	2.47	1.77	3.12
60507	'Qtrt1'	19.81	18.76	16.2	28.4	36.3	45.89	20.3	18.14	9.92	19.18
60510	'Syt9'	2.12	2.3	0.91	8.16	1.25	6.57	4.47	0.25	5.39	1.25
60525	'Acss2'	19.14	16.1	15.5	3.68	16.5	29.79	23.8	16.29	11.95	15.17

60527	'Fads3'	8.2	4.53	4.71	5.53	0.54	6.57	5.48	8.83	12.34	2.37
60530	'Fignl1'	0	0.98	0	0	2.34	0.35	0	0	0.27	0
60532	'Wtap'	36.35	34.73	30.4	59.8	50.3	29	27.2	40.47	60.36	55.12
60533	'Cd274'	1.9	3.65	0	1.33	3.34	1.09	0.01	0.01	3.52	5.03
60534	'Fancg'	0.6	1.96	8.9	0	3.5	5.7	12	5.78	0.04	6.94
60594	'Capn12'	0	0	0	0	0	3	0	0.06	0	0.99
60595	'Actn4'	16.64	22.17	20	1.65	18.7	12.35	5.91	23.22	27.93	15.96
60596	'Gucy1a1'	5.81	8.54	8.71	0	8.57	11.19	20.1	7.34	15.72	7.54
60597	'Mapk8ip2'	43.41	45.4	78.1	32.3	30.7	27.95	31.5	42.6	34.84	37.46
60599	'Trp53inp1'	1.48	0	2.72	2.07	0.07	0.01	0.02	0.04	1.2	0.01
60611	'Foxj2'	0.01	0.52	0.01	7.82	0.02	0	0.33	0.37	0.65	1.97
60613	'Kcnq4'	1.98	1.05	0	0	0.32	2.57	4.77	5.45	3.07	0.72
606496	'Gsk3a'	1.94	1.29	4.24	5.62	3.29	1.14	2.73	1.09	4.1	1.18
619287	'Rtl4'	0.76	1.45	1.05	3.71	7.42	2.98	2.46	1.22	4.71	8.41
619297	'Ccdc194'	0	0.01	0	0.01	0.02	0.02	0	0.01	0	0
619301	'Tmem253'	1.78	0	0.77	0	0	0	1.23	0	0	0
619326	'9130409I23Rik'	0	0	0	1.93	0	0	0	0	0	0
619329	'F420015M19Rik'	0.03	0.2	0.03	0.14	0.11	1.2	0.11	0.12	0.27	0.06
619331	'Zfp551'	0.08	0.29	3.6	0.19	8.89	6.9	0.04	2.72	11.22	5.05
619332	'4933416C03Rik'	0	0	0	0	0	0	2.28	0	0	0
619441	'Tnfsfm13'	0	0	0.06	0	0	0	0	0	2.61	0
619547	'Rpl34-ps1'	0.27	0.48	0.52	0.97	0.17	0.66	0.35	2.04	1.07	0.3
619597	'Gal3st2c'	0	0.18	0.02	0	0	0	0	0	0	0
619605	'Zcchc17'	143.4	133.4	97.5	123	131	190.9	108	148	210.7	191
619883	'Gm6109'	0	0	0	0.14	0	0.22	0.43	0	0	0
620078	'C130026I21Rik'	0	0	0	0	0	0	0	0.03	0	0
620235	'Siglec15'	0	0.15	0	0	0	0	0	0	0	0
620246	'Gpr52'	1.6	4.69	0.27	0	0.88	2.55	3.75	1.88	2.35	2.65
620292	'Cntnap5c'	0.81	1.7	6.93	11.4	3.31	8.38	31.9	6.83	28.69	20.89
620419	'Zfp963'	2.22	7.55	2.63	0	0	0	4.95	5.34	4.59	5.51
620592	'Tmem28'	0	1.93	4.86	0	1.48	0	0.43	1.24	1.6	0
620631	'Ttc30a2'	0	0	0	0	0	0	0	0.01	0	0
620695	'Gm13889'	7.64	5.09	6.84	9.57	1.52	0.72	2.73	1.4	3.26	0
620807	'Mup6'	0	0	0	0	0	0	0	2.14	0	0
620913	'Gm12185'	0.03	0.11	0.06	0.03	0.14	0.02	0.05	0.04	0.1	0.02
621080	'AI429214'	0	0	0	0	0	0	0	0	0	0.02
621407	'Gm9970'	0.04	0	0	0	0	0	0.99	0.18	0	0.35
621561	'Vmn1r127'	0	0	0	0.02	0	0	0	0	0	0
621580	'Gm21953'	0	0	0.27	2.48	0.2	0	0	0	0.29	1.68
621603	'Aldh3b2'	4.05	12.92	1.09	6.48	25.8	37.32	7.36	11.52	9.22	4.96
621697	'Rpl32l'	0	0.18	0	0	0	0	0.46	0.22	0	0.95
621823	'Psme2b'	0.91	0.35	0.78	2.79	1.27	2.95	1.41	0.29	1.23	0.59
621893	'Hist2h2ab'	0	1.24	0	0	0	0	0	0	0	0
621976	'Tmem170b'	8.01	6.42	3.99	2.14	7.41	1.89	6.9	8.79	7.96	8.7
622282	'Gm6306'	0	0	0	0	0.24	1.53	0	0	0	0.08
622307	'5830473C10Rik'	1.51	0	0	0	0	0	0	0	0	0
622320	'Kctd21'	3.43	1.68	0	0	0	0	0	3.74	0	0
622402	'Akr1c12'	0	0	0.2	0	0	0	0	0	0	0
622404	'Ccdc107'	75.81	65.32	76.6	168	123	149.8	87.2	107.2	132.4	82.67
622408	'Mcidas'	0	0	0	0	1.21	0	0	0	0	0
622434	'Arhgef26'	3.92	6.22	3.57	4.27	0.01	0.02	1.33	0.68	0	0
622474	'Smok3b'	0.01	0	0	0	0	0	0	0	0	0
622480	'Spocd1'	0	0	0.12	0	0.02	0	0	0	0	0.01
622486	'Smok3c'	0.01	0	0.11	0.1	0.26	0	0	0	0	0
622554	'Majin'	0	0	2.6	0	0	0	0	0	0	0
622640	'Gm6337'	0	0	0.21	0	0	0	0	0	0	0.06
622645	'Tmem200c'	1.41	0	0	0	0	0.06	0.51	0.55	0.03	0
622665	'Ccdc17'	2	1.25	0.26	0	8.22	14.91	1.15	3.99	0.49	1.81

622675	'Zfp827'	3.15	4.52	0.85	4.5	1.68	3.04	0.22	1.52	3.34	0.76
622699	'Gm12569'	0	0	0	0	0	0	0	0	0.01	0
622744	'Gm6351'	0	0	0	0	0	0	0	0	0.01	0
623121	'Ifi213'	0	0.04	0	0	0	0.02	0	0	0	0.02
623131	'Prr19'	5.54	9.46	3.62	0	0	2.82	0	2.63	5.16	7.14
623230	'Tmem200b'	0	0	0.12	0	0	0	0	0.57	0	0
623279	'Dok6'	2.07	0.1	6.31	0	0.15	0	0	0	0	1.69
623474	'Rad54b'	0	0	0	0.81	1.57	0.53	0	0.22	0	0.01
623503	'Prlh'	0	0	0	1.14	0	0	0	0	0	0
623534	'Nme9'	0	0	0	0	0	0	0.27	0	0	0
623661	'Lipt1'	10.45	2.25	9.04	0.08	6.71	5.7	2.93	4.08	7.6	1.5
623734	'Vmn2r85'	0	0	0.09	0	0.02	0	0	0.1	0	0.38
623781	'Gm14137'	0	0	0.47	0	0	0	0	0	0.43	0.77
623898	'Gm6460'	0	0	0	0	0	0	2.87	0	0	0
624219	'Angptl8'	0	0	0.7	0	0	0	0.12	0	0	0.26
624245	'Speer4e'	0	0	0	0	0	0	0.11	0	0	0
624681	'Btnl6'	0	0	0.15	0	0	0	0	0.47	0	0
624860	'Gm12253'	0	0.04	0	0	0	0.03	0	0	0	0.03
624866	'Lekr1'	0	1.97	0.6	0.81	2.55	0.37	0	4.33	0.09	2.3
625018	'C4a'	0.01	0.81	2	0	0	0	0	0	0	1.07
625029	'Vmn2r83'	0	0	0	2.25	0	0	0	0	0	0
625068	'Vmn2r84'	0	0	0	0	0	0	0	0.76	0	0
625098	'Slc38a6'	3.18	3.71	6.87	5.26	3.62	0.84	0.39	4.5	2.38	0.04
625109	'Vmn2r86'	0.01	0	0.02	0	0	0	0	0	0	0
625123	'Gm6557'	0	0	0.28	0	0	0	0	0	0	0
625131	'Vmn2r87'	0.01	0	0.64	0	0	0.04	0.02	0.24	0.08	0
625249	'Gpx4'	700.8	517	725	681	796	922.5	816	662.9	618.7	557.86
625281	'Gm6570'	0	0	0.16	0.77	0	0	0	0	0	0.13
625464	'Gm6588'	0	0	0	0.12	0.04	1.53	0	0	0	0
625580	'Vmn2r48'	0	0	0	0	0	0	0	0	0	0
625591	'Cldn34c2'	1.2	0	0	3.21	1.06	0	0	2.08	1.05	1.14
625638	'Fam43b'	6.99	4.98	4.98	6.11	5.31	0	2.82	3.66	10.05	2.51
625662	'Ankrd31'	0	0	0	0	2.01	2.37	0	0	1.75	0
626067	'Gm6650'	0	0	0.03	0	0	0	0	0	0	0
626215	'Gm6657'	0	0	0	0	0	0	0	0	1.06	0
626316	'Zfp987'	0	0	0	0	0	0.13	0	0	0	0
626359	'Wdr93'	0	1.89	0	0	0	0	0	0	0	5.31
626391	'Zfp951'	0.16	0.53	4.11	0.74	3.16	0	2.7	1.83	1.29	0.86
626415	'4930467E23Rik'	2.43	3.89	3.2	0.68	2.47	1.1	1.25	1.55	2.07	4.79
626578	'Gbp10'	0	0	0	0	2.55	0	0	0	0	0
626596	'Rgs22'	0.06	0.04	0	0.06	1.15	0	0	0	0.01	1.12
626682	'Defa28'	0	0	0	0	0	0	0.43	0	0	0
626802	'Gm14322'	2.24	4.26	4.47	1.88	7.1	2.66	3.77	3.64	8.72	2.46
626832	'Gm6710'	0.26	0.52	0.95	0.5	0.65	3.64	2.5	0.3	4.61	0.52
626848	'Zfp971'	22.12	34.83	39.1	70.3	48.2	57.06	24.8	37.96	26.94	31.13
626858	'Gm6713'	0.27	0.19	0.27	0.09	0.08	0	0.04	0.26	0.19	0.09
626870	'Gm11992'	0	0.08	0	0	0	0	0.08	0.05	0	0.16
627035	'Gm6729'	1.04	0.34	0.31	0.07	0.08	0	0.07	0.39	0.11	0.07
627049	'Zfp800'	3.2	1.46	1.47	1.56	1.27	3.33	0.41	2.27	0.16	2.08
627191	'Syndig1l'	2.98	1.78	0.27	1.45	2.25	1.09	0	1.96	15.39	0.9
627214	'Fam196a'	0.95	4.98	2.16	8.8	4.56	6.16	2.89	3.36	3.82	0.81
627352	'Morf4l1b'	23.58	24.22	23.1	25.7	22.1	27.23	18.5	28.43	28.57	25.09
627470	'Gm6760'	0	0	0	0	2.56	0	0	0	0	0
627626	'Ptchd4'	1.22	0.8	0	1.74	0.53	1.69	3.3	0.83	1.46	0.95
627872	'Dnah7a'	0.44	0.33	0.5	0.23	0.58	0.76	0.07	0.16	0.14	0.76
628304	'Gm6866'	0	0	0	0	0	0	0	0.01	0	0
628308	'Zfp970'	13.78	7.48	5.38	10.4	14.5	9.98	8.31	11.32	6.98	10.02
628475	'Gm6882'	1.79	0	0	0	0	0	0	0	1.23	0

628518	'Gm6890'	0	0	0	0.08	0	0	0	0	0	0
628779	'Hs3st4'	5.23	0.31	1.51	7.79	2.27	0.03	0.07	1.12	0	0.31
628813	'Gm11437'	0	0.08	0	0	0	0	0	0.2	0	0.07
628870	'Otogl'	0	0.09	0	0	0	0	0	0	0	0.43
628900	'Serpina3i'	0	0	0.68	0	0	0	0	0	0	0
628946	'Vmn1r77'	0	0	0	0	0	0	0	0	0	0.05
629016	'Zfp953'	0	0	0	0	0.32	0	0	0	0	0
629055	'Gm6943'	0.06	0	0.1	0.03	0.08	0	0	0.03	0	0
629059	'Fam124a'	12.83	6.39	7.01	10.1	5.56	2.58	6.86	9.19	8.14	4.49
629114	'Defb23'	0	0	0	0.7	0	0	0	0	0	0
629147	'Ctxn3'	6.99	0	16.2	25.6	0	5.72	0	1.41	9.32	1.67
629242	'Gm6958'	0.3	1.25	2.14	0.37	0.3	1.79	0.58	0.45	0.66	0.28
629303	'Heatr9'	0	0.03	0	0	0	0	0	0	0	0
629378	'Dact3'	2.9	0.19	0.02	0.06	1.74	1.6	1.26	1.11	1.44	0.22
629499	'Mroh8'	0.03	0	0.16	0	0.12	0	1.27	0	0	0
629756	'Wfdc10'	0	1.76	0	0	0	0	0	0	0	0
630294	'Gm7030'	0	0	0	0	0.06	0.01	0	0.02	0	0.07
630579	'Zfp808'	0.02	0.11	0.09	1.37	2.56	0.04	0.09	0.59	1.04	1.03
630751	'LOC630751'	0	0	0	0	0	0.04	0	0	0	0
630836	'2010315B03Rik'	5.49	6.87	5.05	11.3	11.2	4.72	7.08	6.8	3.32	8.94
631145	'Fam90a1b'	0.18	0.38	0.45	0.68	0	0	0	0.43	0.34	0.09
631323	'Gm12250'	1.7	0	0	0	0	0	0	0	0	0
631584	'Catspere1'	0	0	0	0	0	0.03	0	0	0	0
631797	'Fer1l6'	0	1.11	0	0	0	0	0	0	0	0
631990	'Cdr1'	4.54	1.64	4.08	0.47	10.2	3.56	4.29	0.67	3.64	2.83
632126	'Btnl4'	0	0	0.03	0	0	0	0	0.1	0	0
632687	'March10'	2.27	0.15	0	0	0	0.01	0.04	0	0.17	0
632778	'Erich4'	0	0	0	0	0	0.16	0	0	0	0
633057	'Gm7102'	0.23	0.87	1.11	2.68	0.6	1.21	0.27	0.27	0.56	0.27
633285	'Rbm46'	0	0	0	0	0	0	0	0	0.03	0
633640	'Tmem267'	0.05	3.04	2.95	0.13	2.49	6.57	2.5	5.77	9.25	15.69
633979	'Ak9'	0.26	0	0	0	0	0.25	0.07	0.13	0	0
634104	'Olfr287'	0.27	1.99	0.26	0	0	0	0	3.91	0	0
634731	'Susd1'	0.18	0.03	1.08	0.07	1.04	0.04	0.01	0	0.54	0.08
635169	'CK137956'	0	4.48	0.02	0	3.65	2.09	10.5	0	0	4.37
635253	'Usp51'	7.62	4.76	3.24	18.4	5.74	0.89	0.65	4.12	2	6.86
635668	'Fbxw23'	0.02	0	0.03	0.1	0.08	0	0	0.05	0.02	0
635702	'Naaladl2'	0.27	0.83	0.01	0	0.35	0	0.04	0	0	0
636104	'Gm7173'	0	0	0	0	1.38	0	2.77	2.55	1.35	0.33
636741	'Zfp964'	1.64	0.36	0.37	0.08	0.14	0.35	3.6	0.2	0.31	0.31
636808	'Cntnap5a'	2.28	1.54	1.75	0.78	3.09	1.01	5.9	1.05	2.85	0.37
636931	'Trim71'	0	0	0	0	0	0	0	0	1.54	0
637004	'Vmn2r3'	0	0.31	0.32	0	0	0.49	0	0	0	0
637053	'Vmn2r4'	0	0	0	0	0	0	0.02	0	0	0
637079	'Iqcn'	0	0.52	0.51	1.79	0	1.54	0	0.38	0	0.71
637277	'Sycp2l'	0	0.19	0.02	0	0	0	0	1.06	0	0
637515	'Nlrp1b'	0.89	0.95	1.21	2.3	2.08	1.49	1.11	0.48	0.89	1.05
637896	'Vmn2r78'	0	0	0	0	0	0	0	0	0	0.28
638102	'Vmn2r115'	0	0	0	0.02	0	0	0	0	0	0
638262	'Gm7233'	0	0	0	0.68	0.84	0	0	0	0	0
63828	'Fn3k'	19.1	50.16	47.4	25.9	40.3	50.79	24.3	44.36	30.41	33.86
63856	'Taf8'	8.38	17.18	7.86	6.65	8.25	2.08	0.02	9.5	17	12.01
63857	'Bco1'	0	0.04	0	0.37	0	0	0.02	2.21	0	0.55
638580	'Gm7244'	0	0.21	0	0	0	0	0	0	0	0
63859	'Impg1'	0	1.76	0	0	1.22	0	0	0.42	0	0
63872	'Zfp296'	0	0	0	0	0	0	0	0	0	0.73
63873	'Trpv4'	0	0.1	0	1.04	0	0.29	0	0	0	0
63913	'Fam129a'	0	0	0	0	0	0.36	0	0	0	0

63953	'Dusp10'	1.93	6.12	12.1	4.5	3.05	0.23	7.37	6.69	4.22	1.87
63954	'Rbp7'	0.08	0	0	0	0	0	0	0	0	0
63955	'Cables1'	0.12	1.12	2.26	6.69	1.12	2.97	6.31	0	3.13	0.46
63958	'Ube4b'	8.53	11.92	19.8	10.2	9.48	4.87	12.8	6.18	11.49	4.89
63959	'Slc29a1'	31.67	34.33	47.4	16.8	39.5	35.08	94.8	10.38	56.11	52.43
639774	'Skint8'	1.49	1.31	1.82	3.19	1.9	1.39	2.03	0.66	2.05	1.33
63985	'Gmfb'	46.16	41.26	35.7	32.7	36.2	22.52	43	48.1	37.83	49.21
63986	'Gmfg'	0	0.02	0	0	2.28	0	0	0	0	2.35
639910	'Gm20767'	0	0.03	0	0	0	0	0	0	0	0
63993	'Slc5a7'	0.19	0	0	0	0.19	0	0	1.23	0.3	0
64008	'Aqp9'	0	0	0	0	0	0	3.68	0	0	0
64009	'Syne1'	13.32	17.67	9.46	32.2	17.8	22.23	23.5	18.52	51.25	46.4
64010	'Sav1'	0.4	0	0	0.07	0.16	0.07	0.04	0.13	0	0
64011	'Nrgn'	14.66	6.03	2.2	0.5	38.1	6.86	1.9	28.69	12.86	2.06
64050	'Yeats4'	13.69	31.88	24.6	34.6	17.8	20.07	6.28	17.19	15.56	15.46
64051	'Sv2a'	105.8	77.84	136	31.8	95.2	63.8	93.4	104.6	116.8	100.25
640524	'Sptbn5'	0	0	0.58	0	0.54	1.32	0	0.19	0.21	0.33
640543	'Tgm7'	0.17	0	0	0	0	0	0	0.03	0	0
64058	'Perp'	3.12	0.96	4.85	0	0	2.14	0	0	3.83	1.26
64059	'Oxct2a'	0.17	0.17	0	0	0.14	0.1	0.12	0.04	0.03	0.22
64074	'Smoc2'	2.79	0	21.1	0	0	0	0	0	0	0
64075	'Smoc1'	21.85	42.68	25.5	1.83	22.3	3.85	14.7	5.73	9.74	14.71
64082	'Popdc2'	0.04	0	0.18	0.06	0.11	0.04	0.02	0	0.06	0
64085	'Clstn2'	2.45	1	7.87	4.89	1.67	2.75	1.28	8.65	2.68	3.17
64095	'Gpr35'	0	0.65	2.25	1.95	0	0	0	0	0.02	0
64103	'Tnmd'	2	0	3.22	0	0	2.46	0	1.51	0	1.64
64113	'Moap1'	32.65	17.85	35.3	18.9	25.9	33.75	38.9	42.58	23.22	25.78
641340	'Nrbf2'	17.65	21.93	25.7	28.4	15.6	16.9	33.8	27.6	32.24	13.07
64136	'Sdf2l1'	72.77	76.01	36.3	17.1	29.3	65.29	19.9	38.42	131.5	26.4
641361	'Pinlyp'	0	0	0	0	0	0	0	0	0	0.93
641376	'Tomm40l'	26.53	18.6	25	8.91	24.5	44.42	43	18.62	16.77	21.51
64138	'Ctsz'	81.76	53.14	103	99.7	40.1	293.5	58.8	112.9	156.2	270.16
64143	'Ralb'	6.15	9.57	10.1	14.5	11.9	0	9.99	5.62	1.52	2.61
64144	'Mllt1'	1.71	3.67	2.15	0	0	0	6.61	3.69	0	3.48
64176	'Sv2b'	6.08	3.24	9.73	0.66	2.5	1.35	4.15	15.31	4.76	11.22
64177	'Trpv6'	0	0	0	0	0	0	0.41	0.01	0	0.13
64209	'Herpud1'	137.8	174.6	92.3	180	93	78.66	153	160.2	111.1	145.49
64213	'St7'	26.97	27.9	12.8	11.2	12.4	20.87	22.1	24.18	9.13	21.75
64242	'Ngb'	401.4	520.9	170	342	304	561.2	293	259.2	222	439.14
64290	'Foxb1'	1.74	0.06	0	0	0.82	0	0	0.32	0	0
64291	'Osbpl1a'	23.65	17.84	41.8	15.6	29.9	27.41	18.5	11.12	10.88	11.01
64292	'Ptges'	1.75	3.39	1.57	0	0	2.6	1.08	0.69	0	5.21
64293	'Stk32b'	0	4.25	0.9	0	3.66	3.6	0	1.91	0.01	1.42
64294	'Itm2c'	2090	2130	1429	1524	1504	1766	1195	1898	1455	2095.34
64295	'Tmub1'	43.99	27.18	29.7	24.8	62	53.97	31.2	41.05	73.34	76.8
64296	'Abhd8'	185.2	197.6	186	147	167	177.9	163	142.8	165.3	100.12
64297	'Gprc5b'	2.19	4.29	11.3	5.54	5.81	5.73	1.49	1.03	7.68	1.41
64337	'Gng13'	7.81	14.98	47.1	43.1	11.2	13.91	2.55	20.52	40.43	0.24
64339	'Fndc4'	23	5.47	11.5	15	26.6	15.59	15	18.71	37.92	1.71
64340	'Dhx38'	2.74	7.29	8	7.54	4.65	3.59	3.92	8.57	0.07	16.48
64378	'Gpr88'	0	0.04	0	13.4	0	1.59	0.01	0.31	0.49	0.02
64383	'Sirt2'	60.68	101	92.9	60	100	82.25	77.8	57.22	80.58	82.62
64384	'Sirt3'	50.06	46.38	99.7	53	46.9	76.34	50.1	65.64	48.57	73.85
64385	'Cyp4f14'	1.44	7.09	0	0	0	0	0	2.04	2.25	0
64424	'Polr1e'	2.22	7.69	6.49	4.56	4.43	4.87	7.26	5.2	3.32	5.72
64436	'Inpp5e'	6.14	9.5	10.9	12.1	10.6	4.47	11.7	13	10.17	2.27
64450	'Gpr85'	35.2	22.13	43.3	37	41.8	9.88	28.2	31.21	34.01	32.56
64451	'Dip2a'	0.22	1.07	0.99	0.2	1.11	1.29	2.45	1.98	5.03	1.89

64453	'Zfp280b'	4.53	3.63	10.5	4	5.45	5.26	6.07	10.7	12.31	3.71
64540	'Tspan4'	12.13	8.87	34.8	9.48	32.1	41.91	22.6	29.15	8.72	0
64580	'Ndst4'	6.75	1.52	6.72	4.49	3.86	34.13	1.01	1.05	2.01	3.59
64602	'Ireb2'	4.01	4.09	3.76	10.8	4.94	2.02	3.5	4.18	5.36	3.2
64652	'Nisch'	68.22	63.88	106	37	44.6	25.1	82.5	48.07	44.36	54.35
64654	'Fgf23'	1.02	0.73	0.62	0	0.27	0	0.02	1.43	3.95	0
64655	'Mrps22'	32.34	46.17	40.9	47.4	21.4	50.51	31.9	38.79	19.91	38.44
64656	'Mrps23'	36.51	31.33	60	41.1	40.9	59.16	26.8	53.54	46.97	26.75
64657	'Mrps10'	57.22	68.77	74.4	101	107	141.5	48.2	78.83	98.18	104.12
64658	'Mrps25'	12.52	14.12	17.1	12.4	12.4	20.97	17.2	16.93	8.62	10.93
64659	'Mrps14'	134	118.7	79	85.1	160	156.4	106	96.23	127.9	133.81
64660	'Mrps24'	73.24	84.82	83.2	73.5	60.3	82.68	97.7	78.04	62.28	61.36
64685	'Nmi'	0.54	2.6	11.2	14.2	3.81	6.04	4.68	0	3.62	3.82
64704	'Htra2'	41.77	35.81	45.5	16.2	37.1	47.27	37.4	45.91	44.89	48.23
64705	'Dpys'	0	0	0	0	0.91	0	2.25	0	0.09	0
64706	'Scube1'	1.27	0.58	2.21	0	0.69	0.35	0.98	0.01	0	0.01
64707	'Suv39h2'	0.01	0.12	0	0	0.1	0	0	0	1.37	0
64833	'Acot10'	0	0.03	1.65	0	0.03	4.91	0	0.48	0	0
64898	'Lpin2'	11.99	11.96	17.8	9.75	7.68	11.79	12.3	10.43	7.64	10.68
64899	'Lpin3'	0	0	0	0	0	2.36	0	0	2.51	1.03
64930	'Tsc1'	6.78	5.88	6.17	8.6	3.5	2.94	4.03	3.34	4.01	4.75
64933	'Ap3m2'	33.82	49.27	55.8	57.7	50.1	35	28.2	54.09	61.21	50.34
64934	'Pes1'	14.73	19.78	30.3	7.18	14.2	23.08	36	23.68	19.98	23.57
64945	'Cldn12'	8	3.76	13.2	14.2	14	0.01	3.11	1.27	12.15	8.61
65019	'Rpl23'	78.05	84.98	68.9	100	76.4	100.4	75	62.27	97.7	79.51
65020	'Zfp110'	0	2.93	8.06	1.28	5.01	4.4	0.68	5.32	1.05	0.72
65079	'Rtn4r'	0	1.39	0.57	0	3.88	0	6.54	0.02	0	0
65086	'Lpar3'	0	0	0	0	0	0	0	1.09	0	0
65098	'Zfand6'	30.72	49.06	17.8	48.1	64.8	44.56	57.5	23.65	73.99	38.26
65099	'Irak1bp1'	19.06	11.64	13.1	27.2	15.5	15.64	8.11	8.63	17.78	7.16
65100	'Zic5'	0.63	1.06	0	0	3.19	2.25	6.26	0	1.77	5.74
65102	'Nif3l1'	26.44	21.7	29.6	20	43.3	49.21	53.4	33.39	40.45	26.36
65103	'Arl6ip6'	8.23	2.02	0.88	2.64	6.16	3.84	0	2.08	0	6.48
65105	'Arl6ip4'	71.18	68.02	48.2	49.2	106	35.77	70.8	62.85	78.51	43.5
65106	'Arl6ip5'	318.2	256	149	148	239	307.3	226	353.3	300.4	310.87
65107	'Lrp10'	4.27	4.45	3.04	11.6	1.87	4.29	0	3.01	15.06	1.06
65111	'Dap3'	55.05	81.31	62.5	54.1	66.7	97.29	76.8	54.35	70	58.55
65112	'Pmepa1'	1.86	1.93	1.36	6.69	1.36	2.4	0.11	1.95	0.88	0.08
65113	'Ndfip1'	1757	1885	1404	1623	1259	2009	1610	2052	1981	2021.29
65114	'Vps35'	114.3	96.37	81.8	53.9	50.3	46.06	111	92.75	90.53	111.63
65115	'Bean1'	3.27	0.42	1.64	0.02	12.1	0.02	6.54	1.06	1.25	0
65116	'Prrg2'	0.98	14.29	1.58	1.34	35.2	2.61	4.78	2.9	4.06	7.58
65221	'Slc15a3'	0	1.1	0	0.27	0	0	0	0	0	0
65246	'Xpo7'	3.75	6.49	3.64	4.76	5.97	1.56	11.2	4.71	7.33	5.83
65247	'Asb1'	1.7	3.43	3.11	0.75	1.98	0	2.42	1.72	2.98	0.95
65254	'Dpysl5'	12.79	15.43	5.22	0.71	2.56	2.69	13.2	12.62	11.85	19.92
65255	'Asb4'	32.71	36.17	9.93	43.2	0.3	47.39	5.74	10.3	51.88	48.59
65256	'Asb2'	0.06	1.06	0.14	0	2.29	2.52	0.04	0.01	3.37	1.45
65257	'Asb3'	21.92	16.63	17.7	13.9	13.4	18.9	14	17.44	35.58	10.87
652925	'Tmem243'	0.14	0	0.05	0	1.39	0	0.05	0.72	0	0
654309	'Nrp'	5.92	8.29	5.93	13.3	5.45	4.26	6.8	2.46	2.3	4.28
654459	'Defb25'	8.2	1.21	1.83	0	3.51	0	0	4.13	1.12	3.13
654462	'Kncn'	0	0	0.73	0	0	0	0	1.65	0	0.62
654470	'Tctn1'	9.85	9.21	7.4	2.73	6.91	2.9	0.06	9.86	7.87	15.77
654795	'Sdr39u1'	124.6	111.1	96.2	185	124	218.9	172	138.3	118.9	121.81
654801	'Zfp784'	0.23	3.78	7.35	0	10.2	4.19	0.02	4.24	15.19	3.63
654812	'Angptl7'	0.28	0	0	0.03	0	0	0	0	0	0
654818	'Smco3'	6.79	6.02	0	0	5.24	2.96	0	0	0	0.96

654821	'Gcnt7'	0	0	0	0	0.02	2.46	4.59	0	0.29	0.02
654824	'Ankrd37'	24.39	15.04	8.66	1.19	30.2	35.44	8.72	9.62	12.35	7.41
65945	'Clstn1'	25.14	26.43	53.2	45.1	37.6	32.59	37.6	20.59	31.66	29.75
65960	'Twsg1'	5.98	4.71	5.22	15.5	4.48	0	0.26	2.12	6.37	1.74
65961	'Utp3'	41.28	23.42	29.4	19.7	38.6	49.17	20.1	42.1	19.99	20.13
65962	'Slc9a3r2'	23.75	17.03	40.6	11.5	56.4	17.18	22.1	18.54	32.72	9.57
65963	'Tmem176b'	47.58	220.7	106	106	114	110.4	56.5	62.32	82.98	169.4
65964	'Map3k20'	0.77	0.73	0.34	0.01	2.54	0	0	0.11	0.02	0.07
65967	'Eefsec'	20.62	22.2	10.7	30.1	27.2	7.26	15.3	18.71	13.14	23.49
65969	'Cubn'	0	0	0.02	0	0	0	0	0	0.09	0.1
65970	'Lima1'	0	1.62	0	2.48	0.09	0.13	5.83	1.4	1.36	1.36
65971	'Tbata'	0	2.98	0	0	0	0	0	0	0	0
65972	'Ifi30'	0	5.64	2.24	1.87	0.21	0.1	8.2	2.72	0	1.22
65973	'Asph'	42.41	74.24	25.7	18	49.7	68.54	48.6	52.43	80.38	74.05
66011	'Ranbp17'	0.51	2.03	0.1	3.6	1.03	0.02	1.49	0.69	2.17	0.36
66039	'Tmem254a'	2.57	3.08	4.19	0.08	6.28	0	14.3	6.7	17.98	5.55
66042	'Sostdc1'	2.31	0	16.1	0	0	6.12	0	0.46	0	0.32
66043	'Atp5d'	337	387.9	316	343	348	548.8	503	397.1	349.4	413.81
66044	'Dtd1'	167.9	156.6	202	192	270	203.4	262	238.7	150	166.47
66046	'Ndufb5'	312.9	349.2	377	289	314	350.8	336	346.6	304.8	345.12
66047	'Mrpl54'	127.4	111.7	125	130	131	182.3	134	106.1	127.5	120.67
66048	'Emc6'	90.34	79.66	71.4	135	54.6	71.89	79.7	94.17	60.16	77.64
66049	'Rogdi'	425.3	425.9	417	438	515	458.7	384	434.5	246.2	364.43
66050	'0610009B22Rik'	99.49	73.88	64	106	70.1	79.85	61	77.56	75.64	65.86
66052	'Sdhc'	217.4	202	208	281	188	357.8	290	278.6	227.1	175.36
66053	'Ppil2'	72.82	76.71	78.6	91	90.2	89.3	67.2	80.91	67.02	75.8
66054	'Cndp2'	28.2	31.99	29.8	29.6	41.2	42.57	31.6	31.2	44.9	29.6
66055	'Sf3b6'	62.77	73.57	120	92.8	54.6	97.2	65.3	89.58	98.68	80.68
66056	'Zfp524'	4.12	6.26	7.84	0	6	8.37	0	3.48	7.7	3
66058	'Tmem176a'	42.22	72.69	44.4	21.3	38.6	52.62	35.9	16.09	58.45	107.58
66059	'Krtcap2'	178.1	155.4	137	121	129	139.4	123	116.8	165.9	87.77
66060	'Cystm1'	72.2	78.15	51	144	189	119.5	79.8	57.28	76.38	146.87
66061	'Tctex1d2'	49.91	47.89	24.8	36.5	46.4	69.23	40.1	32.6	70.53	43.79
66066	'Gng11'	10.46	1.75	10.9	12.6	13.2	7.28	12.1	4.45	6.4	2.64
66067	'Gtpbp8'	16.9	17.73	15.1	13.8	19.1	16.77	12.5	25.89	21.64	16.22
66069	'Snupn'	14.14	18.13	11	13.4	38.1	32.34	15.3	21.21	31.86	15.83
66070	'Cwc15'	92.36	68.59	87.1	64.1	63.8	111.6	73.8	54.75	55.54	83.64
66071	'Ethe1'	51.2	71.86	44.1	81.3	14	63.08	20.3	83.09	47.93	67.21
66072	'Sdhaf2'	17.85	31.38	20.7	22.4	32	13.47	18.1	25.09	33.65	45.7
66073	'Txndc12'	24.92	58.38	35.9	66.5	17.3	101.3	63	56.88	75.02	57.92
66074	'Tmem167'	7.77	5.37	6.8	3.35	7.33	8.75	9.71	6.85	7.98	6.51
66075	'Chchd3'	54.26	29.6	47.1	33.1	48.8	50.14	36.8	45.32	37.61	50.71
66077	'Aurkaip1'	97.28	114.6	123	211	131	169.2	145	148.9	129	135.94
66078	'Tsen34'	76.47	106.2	160	301	98.9	77.93	104	76.42	83.13	20.93
66079	'Tmem42'	20.32	32.75	5.94	68.3	3.99	5.03	12.2	30.97	10.13	17.54
66082	'Abhd6'	18.97	36.52	23.5	23.9	23.5	27.94	15.7	25.44	19.94	21.35
66083	'Setd6'	5.44	6.74	0.02	20.7	6.3	0.03	3.91	9.38	7.32	16.43
66084	'Rmnd1'	33.69	25.77	23.6	4.51	14.2	0.79	24	23	13.92	13.59
66085	'Eif3f'	20.57	28.18	65.4	95.5	15.8	20.25	36.6	8.81	41.88	8.77
66086	'Fopnl'	65.88	88.8	74	60.6	83.4	96.2	101	83.03	74.93	94.9
66087	'Emc3'	71.13	88.11	54.8	79.8	71.6	52.47	56.1	66.58	64.09	48.01
66089	'Rmnd5b'	26.51	32.2	36.8	12.2	37.3	36.55	46.3	41.98	31.13	18.29
66090	'Ypel3'	48.56	40.81	38.7	174	54.3	82.71	62	84.03	73.16	30.08
66091	'Ndufa3'	320.6	372.4	521	866	389	842.9	375	216.2	630.1	294.58
66092	'Ghitm'	224.6	216	213	155	190	225	202	226	195.9	209.91
66094	'Lsm7'	241.4	233	317	434	258	388.7	251	233.6	364.2	234.14
66096	'Lamtor4'	74.46	70.21	50.4	56.3	135	117.9	77.9	80.09	69.33	108.38
66098	'Chchd6'	204.2	172.8	179	161	206	278.1	161	247.6	233.6	244.94

66101	'Ppih'	22.64	13.47	27.5	19.6	23.1	21.92	32.1	11.45	27.45	15.45
66102	'Cxcl16'	0.07	0.67	0	0	0.25	4.03	0	0	3.62	0
66104	'Tceal6'	79.1	58.89	90	54.4	42	45.61	78.3	58.1	80.51	33.37
66105	'Ube2d3'	75.71	85.01	66.9	115	61.9	41.9	92.8	74.78	77.72	62.15
66106	'Smpx'	0.15	0.06	0.8	0.29	0.05	0.39	0.1	0	0.08	0.33
66107	'Wfdc21'	0.23	0	0	0	0	0	0	0	0	0
66108	'Ndufa9'	130	140.8	173	134	132	161.7	169	193.1	162	141.9
66109	'Tspan13'	202.1	246.1	153	194	214	202.9	151	192.1	257.3	278.75
66111	'Tmed3'	94.91	175.4	69.5	117	96.6	88.42	75.6	78.38	147	138.42
66112	'Marc1'	0.25	0	0	0	0.08	0	0	0	0	0.1
66114	'Dnajc30'	62.21	89.93	52.8	56.3	96.2	119.1	85	69.27	42.37	64.47
66116	'Nat8f1'	38.15	13.93	27.8	33.3	38.7	37.74	43	17.32	34.46	13.45
66117	'Fmc1'	82.52	91.82	143	87.7	103	218.6	132	69.98	154.6	136.7
66118	'Sarnp'	121.2	129.9	91.8	176	113	102.5	95	130.7	110.4	143.71
66119	'Tomm6'	259.6	238.9	235	321	276	354.1	185	235.7	271.5	232.03
66120	'Fkbp11'	10.82	39.39	12.4	69.3	0.17	24.61	0	23.12	20.28	29.13
66121	'Chchd1'	193	130.5	144	134	213	162.7	118	124.9	116.6	193.99
66124	'Josd2'	43.4	63.97	28.1	50.5	57.3	105	63.9	19.01	31.59	49.33
66125	'Sf3b5'	120.3	67.94	68	195	129	186.1	89.5	112.3	109.1	115.73
66126	'Elof1'	189.3	141.4	126	81.2	188	208	171	185.3	182.2	204.24
66128	'Mrps36'	64.85	67.79	71.7	87.8	84.6	61.6	74.9	60.4	70.56	72.95
66129	'Aaed1'	6.8	7.49	3.75	1.89	6.25	18.98	14.4	4.38	10.08	9.46
66131	'Tipin'	11.58	12.73	24.2	20.9	23.3	23.32	37.2	12.22	11.15	16.67
66132	'1110008L16Rik'	2.37	5.92	9.02	0	5.81	4.4	13.6	6.37	0	4.87
66136	'Znrd1'	31.51	25.47	26.4	30.1	23.7	87.69	46	26.31	19.87	30.29
66138	'Bud23'	71.33	37.79	54.9	33.8	52.6	87.09	81	85.95	69.34	50.69
66139	'Mymk'	0.04	6.56	7.19	0	0	0	0	0	0	3.95
66140	'Ska2'	28.41	13.59	4.9	4.22	2.71	16.34	18.5	16.2	18.39	22.19
66141	'Ifitm3'	4.06	0	18.2	31.5	31.2	5.98	0.08	6.02	0.65	10.84
66142	'Cox7b'	242.1	264.9	280	208	207	357.8	256	192.8	204.2	191.32
66143	'Eef1e1'	94.45	74.6	104	105	105	71.96	86.2	117.9	113.9	119.46
66144	'Atp6v1f'	782.3	696.3	627	832	747	1086	678	773.3	888.3	897.91
66146	'Maco1'	8.93	9.22	3.23	13.1	6.69	11.29	10.9	4.54	7.74	8.3
66147	'Necap2'	21.11	17.51	24.3	0	15	16.88	9.7	21.45	17.5	7.04
66148	'Dnajc15'	134.3	106.7	126	144	89.2	167.4	42	88.5	87.99	125.55
66151	'Prr13'	181.1	190.5	89.3	159	201	221.9	154	183.4	423.8	373.2
66152	'Uqcr10'	559.8	463.4	614	714	502	898.1	476	385.6	606.2	386.28
66153	'Fbxo36'	16.58	9.3	2.4	10.3	8.57	4.9	5.86	4.34	9.31	8.89
66154	'Tmem14c'	43.54	77.72	49.4	54	43.3	43.41	70.8	52.57	22.64	69.52
66155	'Ufc1'	113.4	91.83	114	179	129	54.97	99.9	122.1	99.76	107.14
66156	'Anapc11'	70.91	50.54	44.2	44.6	83.1	85.65	65.5	63.77	86.32	93.59
66158	'Rtl8a'	116.8	77.38	129	90.7	115	82.08	87.9	137.8	222.4	120.98
66161	'Pop4'	18.34	25.97	24.4	41.2	29.3	23.58	6.92	18.3	31.94	16.4
66162	'Bola2'	100	109.3	136	270	89.4	193.3	153	73.3	108.1	58.15
66163	'Mrpl4'	114.1	86.83	122	92.5	163	120.8	174	123.2	120.3	77.49
66164	'Nip7'	11.01	16.11	14.3	21.3	9.23	7.76	10.4	15.93	15.61	11.41
66165	'Bccip'	68.08	111.4	70.4	84	110	96.64	57.7	89.48	73.32	60.03
66167	'Tma7'	285.9	300.4	286	400	269	409.8	363	220.2	258.8	254.81
66168	'Grina'	426.1	337.9	549	243	433	534.3	660	563.9	454.3	473.75
66169	'Tomm7'	40.39	60.83	59.8	72.9	30.3	96.16	68.5	27.69	63.95	45.37
66170	'Chchd5'	12.96	15.3	6.14	27	13.8	16.84	25.3	18.62	11.14	5.75
66171	'Pgls'	54.97	53.66	51.7	67.4	52.1	84.43	28.5	62.96	43.69	89.59
66172	'Med11'	3.07	6.65	16.9	0	17.4	7.45	9.05	19.21	32.82	28.15
66174	'Nudt14'	17.52	9.52	8.86	20.9	21.1	32.54	20.9	17.68	21.28	27.69
66175	'Mustn1'	2.27	0	0	0	0	0.56	0	0	0	0
66176	'Nat9'	19.94	26.6	6.81	9.76	30.5	23.04	17.5	14.05	7.71	18.07
66177	'Ubl5'	263.6	262.7	267	299	200	303.4	274	156	222.8	210.79
66179	'Ogfod3'	26.47	33.2	25.8	11.9	44.3	31.14	13.9	22.15	23.76	42.66

66180	'P3h4'	11.81	38.68	31.4	52.2	14.8	26.05	0	16.92	22.28	21.68
66181	'Nop10'	271.4	277.5	268	176	177	280.6	205	181.1	199.4	205.27
66183	'Sptssb'	0	4.3	0.03	0.03	2.94	0.72	0	1.04	0	3.01
66185	'Virma'	2.75	2.82	4.71	3.59	2.53	1.79	7.77	5.06	1.78	2.07
66190	'Acer3'	4.17	6.17	12.2	7.77	5.53	5.06	3.43	4.82	8.18	10.36
66191	'Ier3ip1'	109.5	157.7	141	227	138	156.8	103	102.9	145.1	116.13
66192	'Lage3'	23.91	13.04	17.3	21.5	16.7	19.38	33.4	20.63	21.8	33.16
66193	'Pithd1'	82.68	79.28	99.7	193	74.3	84.28	72.7	57.04	108.4	31.91
66194	'Pycrl'	42.29	63.67	29.4	67.3	41.7	104.5	81.8	64.73	54.23	99.15
66196	'Myo19'	0.86	0	2.53	0	2.04	0.21	5.1	0.48	1.99	0
66197	'Cks2'	0.07	2.07	3.26	0	3.03	21.38	0.08	5.45	0	0
66199	'Commd4'	112.5	110.9	95.5	143	98.5	133.8	177	146.3	86.21	143.7
66200	'Commd6'	78.77	44.63	34.5	53	84	72.18	41.7	55.4	23.17	67.74
66201	'Vta1'	54.38	61.26	86.8	103	72.3	143.1	54.4	82.29	81.72	67.59
66202	'1110059G10Rik'	5.41	3.36	3.51	14.4	4.75	2.3	2.71	3.24	4.03	6.33
66204	'Acyp1'	33.71	17.09	21.4	11.5	43.2	61.17	22.3	19.8	33.44	72.47
66205	'Cd302'	12.71	10.32	11.9	0	0.04	11.32	19.7	7.26	7.55	4.31
66206	'1110059E24Rik'	45.32	53.09	55.3	78.5	79.9	101.7	41.4	42.38	58.21	51.75
66208	'Nenf'	325.9	374.3	268	515	171	131.9	293	139.4	395.4	139.05
66209	'Inip'	3.12	7.21	2.78	1.12	2.04	2.29	0.61	2.55	1.64	0.33
66211	'Rpl3l'	0	1.77	0.12	0	0.06	0.06	0.05	0.07	0.06	0.04
66212	'Sec61b'	208.1	216.7	139	301	181	169.4	127	175.2	237.8	272.39
66213	'Med7'	34.05	15.77	17.4	20.1	4.41	22.73	9.68	14.9	24.24	20.18
66214	'Rgcc'	8.48	23.17	24.7	0	13.4	6.5	55.3	22.97	0	11.43
66218	'Ndufb9'	489.8	376.6	527	713	621	593	617	532.8	434.3	444.39
66220	'Zdhhc12'	14.73	0.39	4.93	11.3	4.78	33.13	10.5	0	5.14	21.17
66222	'Serpinb1a'	9.78	10.85	0	0	1.03	0	0.21	2.05	0	0
66223	'Mrpl35'	16.93	15.15	23.2	26.9	10.7	5.48	11.5	21.66	19.9	18.56
66225	'Llph'	27.07	17.22	27.9	22.5	81.6	48.1	40.9	53.65	54.72	30.9
66226	'Trappc2'	110	122.4	93.3	116	78.1	124.1	74.9	96.95	104.8	98.93
66229	'Rpl7l1'	30.63	32.14	40.6	18.2	27.8	49.4	23.5	44.54	32.16	42.86
66230	'Mrps28'	67.99	72.93	55.5	116	44.3	70.33	57.5	67.99	51.38	57.04
66231	'Thoc7'	82.99	80.71	80.4	72.2	62	94.61	46.8	57.61	69.28	78.23
66233	'Dmap1'	30.74	43.83	30.3	49.7	23	38.98	35.6	39.86	30.11	57.65
66234	'Msmo1'	102.3	78.6	82.1	94.8	59.4	85.02	99.9	160.6	97.66	75.92
66235	'Eif1ax'	31.51	24.2	36.5	19.9	31.8	34.89	44.8	20.39	18.73	31.88
66237	'Atp6v1g2'	349.4	386	543	327	591	546.9	545	612.4	266.8	252
66240	'Kcne1l'	9.22	2.66	0	6.61	0.03	11.88	5.97	2.05	0	2.19
66241	'Tmem9'	252.9	261.1	200	162	243	346.9	287	287.7	369.7	353.98
66242	'Mrps16'	49.26	111.3	134	86.2	104	144.9	107	106.9	111.7	83.88
66244	'Nemf'	32.32	29.18	28.9	45.9	44.3	52.38	41.4	39.18	36.66	38.55
66245	'Hspbp1'	95.86	100.7	137	110	90	127.9	94.5	146.9	96.33	79.17
66246	'Osgep'	31.29	27.57	32.7	16.3	57.8	44.72	52.9	34.68	16.8	59.46
66248	'Alg5'	32.44	31.55	31	38.2	22.4	39.59	15.8	29.94	50.89	32.2
66249	'Pno1'	21.45	10.74	13.6	24	19.1	31.01	7.5	12.77	10.82	14.27
66251	'Arfgap3'	11.9	18.36	17.9	7.53	26.9	15.71	24.1	17.99	9.06	18.34
66253	'Aig1'	54.79	41.99	46.2	13.5	49.7	52.36	64.3	29.1	29.45	33.08
66254	'Dimt1'	15.47	26.3	14.2	14	13.8	1.86	5.98	7.49	14.29	20.67
66255	'Hsbp1l1'	1.9	1.29	3.01	0	0.68	0	0	0.2	0	0.83
66256	'Ssr2'	116.8	109.7	98.1	128	101	160.7	104	177.3	200.3	175.42
66257	'Nicn1'	127.2	106.7	93.9	160	126	189.4	107	125.9	119	143.73
66258	'Mrps17'	100.8	96.62	95.3	125	95.3	145.7	97.1	101.7	65.55	101.19
66259	'Camk2n1'	15.79	8.26	10.6	11.8	19.5	6.23	14	13.11	13.65	7.31
66260	'Tmem54'	0	0	0	0	0	0	0	0.11	0	0
66262	'Ing5'	1.02	2.8	0.63	8.99	3.98	0.03	0	2.93	4.61	4.94
66264	'Ccdc28b'	19.93	16.44	23.3	14.6	31.8	42.54	21.4	19.8	43.59	30.94
66266	'Eapp'	28.21	44.18	33.3	18	46.3	44.19	36.1	43.3	30.67	33.13
66268	'Pigyl'	80.32	97.6	88.8	105	77.1	89.91	101	107	113.8	80.8

66269	'Tmed6'	0	0	0	0	0	0	0	0	0.1	0
66270	'Retreg1'	3.83	10.47	6.81	9	5.96	8.58	5.74	2.42	1.91	6.1
66271	'Tmem126a'	96.82	101.8	79.8	71.8	99.5	102.8	72.6	89.44	104.6	134.57
66272	'Cox16'	78.47	40.13	47.7	76.3	48.2	46.38	34	57.75	66.97	64.4
66273	'Aamdc'	55.82	51.34	54.2	45.2	62.4	103.4	75.6	57.88	123.3	78.89
66274	'Lyrm9'	18.33	13.43	17.5	2.69	11.1	13.68	17.2	14.97	16.07	12.73
66276	'1810009A15Rik'	91.54	68.54	47.1	81.5	87.1	111.2	43	60.98	81.6	95.81
66277	'Klf15'	0.69	2.75	6.33	6.04	2.89	0.03	0	4.78	6.18	0.03
66278	'Smim20'	47.62	33.85	39.9	90.8	91.2	105.8	49.8	74.48	50.26	54.6
66279	'Tmem218'	13.09	4.18	12	21.9	6.19	26.26	0	18.73	7.98	13.47
66282	'Tma16'	4.82	7.16	7.5	12.6	7.85	5.03	8.05	14.22	6.73	6.59
66286	'Sec11c'	165.6	167.7	167	203	123	345.9	134	219.3	169.5	213.59
66290	'Atp6v1g1'	279	248.1	248	430	376	316.6	231	333.7	295.4	304.05
66291	'Smim8'	38.17	29.61	25.2	48.3	29.9	19.59	23.3	44.71	12.06	30.62
66292	'Mrps21'	153.4	179.1	155	205	151	320.1	150	125.4	269.5	153.29
66294	'Fam3a'	28.89	16.95	22.6	14.2	26.8	28.51	24.5	31.48	14.41	27.25
66296	'Haus2'	12.81	17.58	17.7	14.9	15.5	12.21	7.74	10.31	10.08	10.3
66300	'Inafm1'	52.72	22.8	48.3	73.4	36.6	15.93	39.2	15.01	58.12	4.17
66302	'Rmdn1'	8.02	14.03	7.56	0.85	4.77	11.33	7.82	9.46	18.51	16.1
66306	'Fam53c'	9.03	2.02	11.8	2.14	8.74	2.63	11.1	5.05	5.06	10.33
66307	'Isoc1'	4.73	6.9	10.9	20.8	4.53	1.61	7.38	5.42	4.59	6.31
66308	'Mplkip'	1.61	3.02	3.33	0.4	1.26	0.76	3.62	2.97	3.98	0.95
66309	'Tmem128'	58.59	29.26	45.5	24	58.8	5.26	18.3	35.45	41.51	42.07
66310	'Dpy30'	130.4	135.2	123	106	115	124.5	135	129.3	121.1	113.08
66311	'Cenpw'	1.81	1.38	0.74	0	0	0	0.03	0	0	1.39
66313	'Smurf2'	1.55	0.1	1.74	0.66	1.25	0.03	4.5	1.81	0	0.65
66314	'Tpd52l2'	58.52	55.9	73.5	36.7	48.8	61.95	83.5	48.14	46.08	50.83
66315	'Senp7'	9.25	12.02	2.05	4.98	11.1	6.32	8.7	7.68	19.36	14.09
66317	'Wdr61'	107.2	84.53	68.2	103	46.9	85.2	63.5	81.29	93.02	104.72
66320	'Tmem208'	79.89	72.62	98	103	116	80.44	99.1	60.22	79.05	49.9
66323	'1700001K19Rik'	0	0.21	0.73	0.69	0.11	0.5	0	0	0	0
66328	'Scp2d1'	0.06	0	0	0	2.22	0	0	0	0.67	0.12
66329	'Susd3'	0	0.17	0	0	4.06	34.63	0	3.79	0	0
66333	'Aqp11'	7.86	2.67	0.11	11.1	0.8	0.25	9.88	14.14	8.75	7.67
66335	'Atp6v1c1'	282.3	270.2	254	191	239	261.8	291	331.1	282	320.7
66336	'Cenpp'	4.14	0	3.38	2.9	3.72	2.04	0	0.96	0	1.37
66337	'Fam229b'	27.36	35.43	12.5	15	27.3	60.75	17	11.94	24.25	49.42
66338	'Cdrt4'	0	0	0.14	0	0	0	0	0	0	0
66340	'Psenen'	214.4	259.7	197	264	249	355.9	317	250.9	254.4	266.55
66343	'Tmem177'	9.89	0.2	2.06	0	0	2.91	0.01	2.27	17.96	6.4
66349	'Dmac2'	41.37	41.11	63	28.4	49.1	25.25	83.5	50.61	42.99	51.67
66350	'Pla2g12a'	13.88	17.05	7.83	50.3	6.8	4.35	11.5	8.34	16.46	7.68
66352	'Blzf1'	10.23	12.08	6.08	11.6	20.6	9.31	6.02	13.57	11.63	4.86
66353	'Riiad1'	35.61	23.51	11.2	84.6	30	51.66	19.5	25.56	17.56	29.06
66354	'Snw1'	44.56	53.08	33.5	25.4	47.8	52.72	28.6	66.02	78.6	45.4
66355	'Gmpr'	35.89	26.75	49.8	58.3	77.1	45.2	42.3	71.25	32.42	26.3
66356	'Knop1'	6.05	4.36	4.6	10.9	3.67	4.98	3.65	5.82	7.35	5.83
66357	'Ostc'	187.7	161.8	92.2	127	97.5	179.9	152	161.3	238.4	219.43
66358	'Adprm'	20.35	6.73	11.3	15.4	11.2	12.82	10.9	12.57	6.71	9.55
66359	'Cox20'	93.95	40.16	64.2	83.1	39.9	74.12	57.5	37.19	70.58	51.3
66361	'Zfand1'	7.34	2.75	21.2	0.04	30.3	23.18	24.4	31.04	20.66	10.68
66362	'Exosc3'	21.33	23.74	12.2	11.1	27.5	10.59	0.1	19.94	3.23	29.34
66364	'2310009A05Rik'	30.12	18.92	23.8	19.5	38.7	14.7	12.5	17.46	14.17	19.22
66365	'Ccdc90b'	11.25	7.93	9.49	16.8	23.2	13.66	3.67	9.26	20.9	17.6
66366	'Ergic3'	302.8	323.3	291	299	278	349	228	392.6	315.1	345.62
66367	'2310022A10Rik'	16.3	28.41	15.9	39.7	6.37	25.38	33	25.82	14.49	12.97
66368	'Rtca'	68.4	60.38	65.6	75.6	84	45.99	67.9	42.53	51.52	50.06
66369	'Dus2'	26.7	32.94	27.7	15.6	18.3	13.2	11.3	38.97	45.23	33.02

66371	'Chmp4c'	0	0	0	0	0	0	0.03	0	0	0
66373	'Lsm5'	33.83	48.3	30.9	40.7	15.9	35.35	34.7	38.53	50.33	23.82
66374	'2310011J03Rik'	25.67	25.11	11.3	19.6	30.1	26.11	24.6	22.33	28.02	24.52
66375	'Dhrs7'	38.32	39.62	41.2	57.8	47.4	48.47	28.2	35.49	41.99	51.32
66377	'Ndufc1'	384.2	340	409	488	390	540.1	446	282.7	387.9	352.36
66379	'Cox14'	261.7	251.5	236	232	193	299.8	233	221.5	293.7	257.97
66381	'Rnf113a2'	29.9	21.85	25.1	44.4	23.3	33.37	8.3	22.31	10.33	12.72
66383	'Iscu'	271.9	163.4	357	307	188	140.8	303	181.2	279.5	69.32
66384	'Srp19'	182.3	180.3	125	152	182	176.1	138	197.5	199.5	154.22
66385	'Ppp1r7'	16.23	16.33	13.7	27.2	29.1	27.56	21.3	19.41	15.13	13.95
66387	'Nudt8'	23.75	22.95	45.9	18.4	48.8	70.01	31.5	45.72	36.5	45.14
66388	'Cutc'	1.24	12.85	4.24	10.6	11.6	0.64	3.95	15.6	11.42	7.45
66390	'Prelid3b'	76.99	68.68	72.8	37.4	65	69.42	77.6	89.19	82.77	73.87
66394	'Nosip'	47.39	50.84	35	2.88	47	38.22	47.3	45.57	51.72	28.2
66395	'Ahnak'	0.11	0.07	3.45	0	1.49	0.88	0.05	0	0.04	0.01
66396	'Ccdc82'	19.12	22.26	28.1	47.1	17.6	15.53	21.3	19.75	36.05	28.41
66397	'Sar1b'	162.7	127.3	124	113	103	111.7	104	151.7	120.8	131.24
66398	'Commd5'	25.16	25.23	22.5	0	33.7	61.34	39.2	52.77	28.27	18.03
66399	'Tsfm'	41.3	35.09	41	36.9	48.6	67.17	41.9	47.2	48.37	38.16
66400	'Alkbh7'	28.34	66.91	83.5	33.4	63.9	107.2	31.3	68.37	62.31	56.3
66401	'Nudt2'	42.42	60.77	59.4	9.7	65.9	115.5	41.4	45.12	59.75	29.78
66403	'Asf1a'	12.45	4.89	4.84	8.34	8.81	3.1	2.44	0.86	2.73	3.51
66404	'Rtf2'	22.36	37.9	16.6	18.5	33.5	67.45	67.9	43.68	34.72	37.59
66405	'Mcts2'	27.01	15.83	23.5	1.51	28	26.85	24.6	33.44	16.64	22.85
66406	'Sac3d1'	15.84	12.3	30.7	0	9.98	15.34	11.4	22.33	11.83	6.98
66407	'Mrps15'	87.63	66.32	74.9	88.3	102	106.3	99.3	74.25	69.51	85.99
66408	'Aptx'	18.41	11.15	8.01	16.2	15.5	6.1	17.2	9.12	21.57	11.82
66409	'Rsl1d1'	55.92	95.23	70	83.2	57.6	101.9	51.9	81.35	66.69	89.27
66410	'Mterf3'	20.32	32.81	11.7	11	19.1	19.05	37.4	22.45	22.83	31.39
66411	'Tbcb'	226.3	224	218	204	214	280.7	234	239.8	233.3	200.79
66412	'Arrdc4'	2.03	1.17	7.73	38.5	7.64	0.09	6.34	7.54	7.5	0
66413	'Psmd6'	110.9	113.4	156	104	86.2	160.4	127	157	113.5	117.96
66414	'Ndufa12'	516.9	450.3	418	630	604	772.9	575	479.2	470.3	555.2
66416	'Ndufa7'	405.4	348.1	429	588	382	632.7	377	391.5	641.9	485.08
66419	'Mrpl11'	29.75	25.27	33.6	44.4	38.1	45.78	26.6	32.05	15.96	23.92
66420	'Polr2e'	146	121.8	99.1	115	162	164.8	138	162.1	132.4	108.35
66421	'2410004B18Rik'	64.84	44.63	40.4	50.9	45.2	36.62	26.3	40.15	50.24	22.64
66422	'Dctpp1'	11.79	2.37	14.9	16.5	27	41.59	12.7	6.79	13.35	5.78
66423	'Coprs'	248.3	186.9	225	176	187	303.6	222	125.3	240.3	99.04
66425	'Pcp4l1'	7.67	22.69	33.6	32.3	35.4	9.18	16.3	65.14	19.7	8.32
66427	'Cyb5b'	56.43	41.66	45	31.6	36.1	12.05	44	63.84	41.74	44.31
66431	'Oxld1'	9.44	25.59	13.5	17	17.6	0	22.9	1.36	12.97	22.47
66432	'Slc7a6os'	13.43	13.66	12.1	22.4	4.94	2.88	1.06	5.63	20.22	11.75
66433	'Chchd7'	56.5	82.93	109	52.7	66.1	108.4	77.4	38.77	48.37	77.94
66435	'Uggt2'	3.27	5.74	5.65	0.83	3.72	9.35	4.4	7.77	7.33	5.56
66437	'Fis1'	427.8	355.6	308	447	383	492	361	366.5	273.9	442.56
66439	'Borcs7'	26.59	34.67	33.6	39.5	31.5	34.08	46.8	28.95	26.15	33.3
66440	'Cdc26'	59.83	87.93	59.8	71	73.6	71.71	84.8	98.58	86	128.26
66441	'Magohb'	13.53	11.27	9.24	3.48	31	10.37	4.29	21.44	13.67	6.28
66442	'Spc25'	0	3.08	0	0	0	0	0	0	0	0
66443	'Tnfaip8l1'	0	2.89	0	0	0	0	0	2.45	0	3.7
66445	'Cyc1'	311.9	210	349	312	267	307.3	351	351.2	183.2	244.44
66446	'Exosc7'	65.52	84.04	43.7	56.7	56.7	101.8	57.3	88.06	98.65	90.39
66447	'Mgst3'	135.5	80.92	310	215	376	310.8	114	245.8	157.3	173.17
66448	'Mrpl20'	236.3	227.1	217	201	243	306.8	139	282.2	181.9	201.84
66449	'Pam16'	300.3	222.1	244	181	185	447	248	237.1	348.1	299.93
66454	'Nmnat1'	4.5	7.73	8.9	0	12.2	3.16	0.16	9.78	0	6.69
66455	'Cnpy4'	18.64	15.46	6.87	3.86	13.5	16.12	7.77	16.19	15.22	20.2

66459	'Pyurf'	16.22	9.73	11.3	7.17	10.6	12.22	9.18	18.26	12.24	14.22
66460	'Sys1'	48.5	63.1	92.8	37	44.7	39.87	71.5	26.17	68.84	32.21
66461	'Ptpmt1'	24.93	11.17	5.05	19.3	16.7	18.96	29	17.39	22.82	17.12
66462	'Rex1bd'	108.4	107.4	115	138	135	104.8	103	123.4	131.5	162.3
66464	'Taf12'	24.11	23.18	25.9	14.5	35.7	26.21	25.5	24.49	24.79	26.77
66467	'Gtf2h5'	119	123.5	82.5	102	112	133.7	125	113.7	126.5	134.18
66468	'Ska1'	0	0.02	0	0	0	0	0	0	0	0
66469	'Fam213b'	171.3	131.1	193	248	305	200.5	183	143	165.9	239.96
66471	'Anp32e'	30.39	41.3	23.2	31.1	36	35.52	31.8	10.85	41.94	29.84
66473	'Ctrb1'	0	0	0.34	0	0	0	0	0	0	0
66475	'Rps23'	276.3	317.1	250	270	232	266	243	154.9	251.1	237.83
66477	'Usmg5'	828.8	1231	1220	1413	766	2002	1095	574.1	1279	682.59
664799	'Ctcfl'	0.38	0.78	0.79	1.31	1.07	0.93	0.85	0.35	0.78	0.41
66480	'Rpl15'	289.5	378.7	229	440	250	243.8	181	217.6	200.2	232.87
664804	'Gm7347'	0	0	0	0	0	0	0	0.48	0	0.68
66481	'Rps21'	111.3	167.9	164	320	79	265	158	79.26	188	103.19
66482	'Exoc2'	11.05	9.38	21.4	10.1	8.03	0.9	12.6	12.33	16.91	20.41
66483	'Rpl36al'	234.9	333.4	243	271	221	269.9	276	186.6	292	283.94
664837	'Gm7361'	0	0	0	0	0	0.01	0	0.17	0	0.25
66487	'Smim4'	21.67	24.82	18.8	29.2	24.7	47.44	14.7	9.38	36.68	32.81
66488	'Fam136a'	66.89	61.46	45.7	24.3	49	73.63	40.4	67.56	67.17	41.27
664883	'Nova1'	16.33	11.58	26.1	7.8	9.38	9.66	24	15.37	21.12	7.53
66489	'Rpl35'	547.6	444.5	489	914	415	727	568	297.1	500.1	397.01
66491	'Polr2l'	46.85	46.67	45.4	52.9	52.9	70.42	37.1	35.87	38.51	39.68
66492	'Zmat2'	101.6	78.22	84.3	71.3	72.9	102.4	78.8	63.58	62.83	47.6
66493	'Mrpl51'	88.2	59.1	61	86.7	68.2	85.34	57	64.84	66.46	68.24
66494	'Prelid1'	307.9	202.7	225	174	190	243.6	232	222.4	188.8	147.97
66495	'Ndufb3'	246.2	225.2	277	233	279	359.9	212	175	248.5	224.2
66496	'Ppdpf'	154	130.6	130	175	100	103	194	97.52	112.5	158.42
664968	'Tmem238'	0.26	0.28	0	3.43	0.1	0	0	0.83	0	2.23
664969	'Gm7429'	0	0	0.56	0.14	0.26	0.11	0	0	0	0
66497	'Cmss1'	23.93	14.7	18.9	22.1	9.48	10.48	11.9	15.06	13.62	26.56
66498	'Dda1'	120.5	103.4	97	148	139	157.7	140	110	165.6	137.42
664994	'Isoc2a'	0	0	0	0	0.05	0	3.74	1.64	0	0
66500	'Slc30a7'	2.57	1.08	0.41	1.05	2.96	0.54	17.6	0.02	1.95	1.54
665001	'Gm14391'	22.52	32.66	13.5	17.3	31.7	32.25	27.4	21.85	22.25	17.09
66501	'1700029H14Rik'	0	0.45	0	0	0	0	0	0	0	0
665033	'Col6a5'	0.43	3.12	0	0.01	0	0	0.06	0.15	0.57	0
66505	'Zmynd11'	18.87	26.19	31.4	26.3	25.1	13.02	23.8	16.8	20.95	20.69
66506	'Psmg3'	38.41	45.84	31.5	22.6	56.2	36.55	73.2	30.31	29.25	46.96
66508	'Lamtor1'	104.3	51.96	104	124	81.8	76.03	109	58.25	57.97	78.61
665095	'Cyp2j8'	1.91	0	0	0	0	0	0	0	0	0
66510	'Rnf181'	145.1	107.5	137	180	152	93.63	120	136.2	113	146.35
66511	'Chtop'	64.22	83.97	69.9	58.9	56.9	60.88	50.4	56.04	39.5	51.89
665113	'Tnik'	13.5	17.09	16.1	18.9	26.4	13.84	11.7	18.54	20.28	11.1
665119	'Sec14l5'	1.22	0.57	0	0	0.21	0	0	0	0	0.57
66513	'Tab1'	16.16	13.61	14.3	0	11.6	12.18	6.32	4.64	33.87	4.39
66514	'Asrgl1'	38.75	21.77	33.8	35.3	24.8	28.7	47.9	22.04	43.62	27.21
66515	'Cul7'	4.24	5.65	13	1.43	3.1	1.28	9.51	0.67	3.44	6.74
665155	'Srp54b'	44.97	53.21	56.6	37.2	34.9	47.37	39.7	61.39	65.38	59.24
665180	'Clec2l'	5.77	10.99	10.5	6.45	18.6	4.97	4.62	10.52	8.79	8.37
66521	'Rwdd1'	78.94	75.84	64.4	48.3	67.5	75.01	57.2	65.27	72.14	63.28
665211	'Gm14326'	22.39	23.26	38.4	35.5	38.4	43.72	5.98	32.15	20.93	26.3
66522	'Pgpep1'	7	7.78	5.67	0.03	3.78	0.13	1.5	6.05	4.29	4.57
66523	'2810004N23Rik'	32.66	10.53	19.6	22.3	22.7	41.98	38.3	11.75	13.91	23.83
66525	'Timm50'	68.76	88.09	74.6	76.1	68.7	94.34	49.2	55.08	61.69	83.08
665255	'Vmn2r28'	0	0	0.05	0	0	0	0	0	0	0
66526	'Tceanc2'	10.9	7.97	0.57	10.6	6.13	5.47	2.24	9.2	10.02	2.43

66528	'Smim5'	0	0.11	0.06	0	7.72	0	0	2.72	0.05	0.05
66530	'Ubxn6'	71.18	57.8	51.4	58.5	42.8	64.89	66	46.69	45.06	85.92
66531	'Cmc2'	10.73	10.18	15.2	17.1	19	18.6	0.85	17.34	22.15	11.13
66532	'Rep15'	0	0	2.9	0	0.05	0	0	0	0.41	0
66536	'Nipsnap3b'	23.48	36.51	28.9	35.8	19.8	41.84	34.2	45.5	25.31	39.35
66537	'Pomp'	380.4	272.7	283	405	265	362.3	279	409.1	427.6	395.07
665378	'Gm7609'	0.96	1.13	1.1	0.57	1.29	0.68	0.56	0.47	1.01	0.82
66538	'Rps19bp1'	54.9	48.2	33.3	48.7	64.5	102.6	33.1	44.86	53.79	27.17
66540	'Fam107b'	0	0	1.67	4.36	1.67	0.38	0	0	3.57	0.05
66541	'Immp1l'	50.56	57.22	28.7	56.9	58.1	12.8	89.1	67.18	34.02	58.54
665433	'Hist1h2ao'	16.31	26.46	2.4	42.8	0	25.05	21.9	7.17	5.44	5.48
66548	'Adamtsl5'	0	0	0.05	0	0.12	0.06	0	0	0	0
66549	'Aggf1'	9.69	2.44	6.43	25	7.99	3.97	9.14	0.51	8.35	2
66552	'Sppl2a'	5.09	4.34	10.8	1.51	2.9	6.86	5.39	5.65	9.05	15.41
66556	'Drap1'	51.15	39.91	44.3	72.4	44.2	41.71	21.5	37.23	23.55	21.19
665562	'Rpl31-ps12'	1.73	11.43	1.76	40.6	1.17	7.83	0.56	1.05	0.86	1.54
665563	'Mthfd2l'	8.31	5.71	4.96	1.18	4.08	1.08	3.13	2.94	6.22	2.49
665574	'Gm7694'	1.47	1.95	4.64	0	0	0	0	0	0	0
66559	'Metap1d'	45	26.04	8.64	10.6	32	23.78	23.4	25.52	15.77	18.82
665596	'Hist1h2bq'	1.63	0	0	0	3.12	0	2.37	0.59	0.65	0
665622	'Hist1h2br'	1.63	0	0	0	3.12	0	2.37	0.59	0.65	0
66566	'Ntpcr'	36.74	34.22	30.2	65.6	61.6	46.1	28.4	56.22	40.9	44.89
66568	'Rwdd3'	11.99	8.66	11.6	35.9	13.3	9.6	8.74	18.39	10.81	21.72
665687	'Gm20777'	0	0	0	0	0	0.01	0	0	0	0
66569	'Gdpd1'	81.99	61.84	70.7	34.7	64.1	49.8	35.8	59.59	45.61	70.73
66570	'Cenpm'	2.97	2.13	0.12	0	12.4	4.67	1.92	0	0.05	5.28
665700	'Hmcn2'	0	0.39	0	0.01	0	0	0	0	0	0.01
66573	'Dzip1'	17.35	10.2	14.7	10.8	9.04	4.81	15.4	9.11	11.29	10.61
665738	'Gm7762'	0	0	0	0	0	0.02	0	0	0	0
665755	'LOC665755'	0	0	0.03	0	0	0	0	0	0	0
66576	'Uqcrh'	513.5	503.3	627	586	564	593.2	564	382.1	375.7	458.26
665775	'Bod1l'	2.96	4.4	4.93	9.95	7.7	3.75	3.63	3.52	5.31	5.42
66578	'Mis18a'	2.98	2.45	3.02	16.8	1.43	0	0	3.59	0	0
66580	'Esf1'	9.61	14.98	10.8	13	8.09	10.15	14.3	5.35	2.99	7.28
66583	'Exosc1'	13.42	15.54	10.6	21.5	24.5	22.11	13.2	19.47	28.33	21.3
66585	'Snrnp40'	40.57	37.99	35.7	28.6	52	77.5	13.3	48.97	19.19	34.26
665858	'Gm7827'	0	0	0	0	0.02	0.76	0.04	0.01	0.05	0
66586	'Crls1'	5.63	1.74	3.04	0.11	5.78	3.09	0.92	3.27	4.79	1.45
66587	'Fastk'	18.8	20.96	19.3	16.7	43	14	45.8	43.85	42.77	40.95
66588	'Cmpk1'	44.51	52.71	53.2	53.1	69.6	47.62	41.4	70.37	67.01	57.8
66589	'Ube2v1'	232.7	255.9	161	240	256	278.4	339	356.5	354	310.01
66590	'Farsa'	35.87	24.43	41	64.4	39.7	55.08	32	28.76	44.26	29.83
66591	'Mad2l1bp'	9.5	6.59	3.9	12.1	36.1	12.98	2.21	13.63	15.08	8.06
66592	'Stoml2'	69.99	71.18	56.2	28.5	77.2	92.13	38.6	70.96	79.71	99.36
66593	'Diablo'	42.74	21.43	14.9	19.6	20	14.33	20	11.45	26.55	27.46
66594	'Uqcr11'	699.2	497.7	720	764	714	1206	673	624.2	833.4	674.92
66595	'Aste1'	9.4	8.28	6.13	12.9	4.03	4.31	4.04	6.77	5.1	7.66
66596	'Gtf3a'	34.82	39.61	37.8	79.2	6.09	45.55	27.5	46.96	63.89	88.69
66597	'Trim13'	5	5.29	0.03	14.6	6.49	6.7	7.34	8.53	0	10.84
66598	'66598'	0.97	0	10	0	5.89	6.93	5.4	12.12	3.87	0
66599	'Rdm1'	17.3	11.47	32.7	36	6.38	19.82	8.52	9.72	26.85	18.62
66603	'Gemin2'	16.72	8.22	6.45	0.15	7.79	9.76	6.41	4.17	8.67	4.61
666048	'Trabd2b'	0.01	0.02	0.02	0.01	0.02	0.02	0.02	0	0	0.01
66606	'Lrrc57'	10.01	15.69	8.1	14.5	11.6	5.1	27.4	20.93	11.43	11.03
666060	'Frmpd1'	0.74	0	2.88	0	0	0	3.94	1.91	0.24	0
66609	'Cryzl1'	61.5	65.74	44.3	37	83.8	113.7	65	66.55	59.93	92.43
66610	'Abi3'	4.55	0	0	0	0.05	3.69	0	0.02	1.78	0
66611	'Ribc1'	0	0	0	0	0	0	1.23	0	0	0

66612	'Ormdl3'	88.41	108.1	60.9	92.3	103	63.3	72.6	115.3	82.34	103.21
66614	'Gpatch4'	9.55	11.9	15.6	16.9	15.8	35.91	27.9	18.06	20.21	17.85
66615	'Atg4b'	65.59	56.38	34.4	40.9	44.5	67.38	45.8	37.1	27.11	60.17
66616	'Snx9'	0.03	1.07	0	8.06	1.36	0.04	5.17	5.16	0	1.89
666168	'Cyp4a31'	0.02	0.02	0	0	0	0	0.02	0	0	0.02
66617	'Ntmt1'	88.81	65.95	51.9	57.3	78.2	58.23	43.8	88.93	52.24	85.2
666173	'Vps13b'	0.95	3.57	3.7	6.37	2.09	1.58	8.02	2.88	2.83	3.17
66618	'Snrnp27'	84.9	83.7	51.7	70	63.9	68.31	56.2	66.33	56.89	47.46
666190	'Gm7972'	0	0	0.01	0.01	0	0.11	0	0.23	0.01	0
666209	'Gm7982'	0	0	0	0	0	0	0	0	0.01	0
66622	'Ubr7'	6.43	6.03	4.25	4.2	7.22	6.58	0.05	11.49	0.05	5.61
66624	'Spcs2'	286.5	240.1	165	345	209	332.4	184	300.6	288.3	324.73
666244	'Tmsb15b1'	3.65	10.74	16.7	8.76	13.6	0.5	7.33	1.05	0	2.71
66625	'Pnisr'	39.05	43.24	36.2	57.8	49.6	51.51	43.8	38.04	40.95	46.78
666253	'Gm8005'	0	0	0.53	0	1.07	0	0	0	0	0
66626	'Cdip1'	170.2	237.5	259	125	157	195.3	129	208.5	235.3	214.13
66627	'Ogfod2'	47.11	35.38	19.3	36.6	36.5	57.37	43.1	29.31	28.76	55.81
66628	'Thg1l'	3.6	4.91	3.33	0	8.93	2.21	0.02	3.38	11.55	0.65
66629	'Golph3'	15.39	10.27	17.6	22.1	4.99	12.17	9.84	4.18	6.91	8.31
66631	'Mfsd14b'	17	12.15	13.6	11.7	20.7	6.59	24	13.98	18.57	15.48
666311	'Zscan25'	0.09	0.09	0.05	0	0.77	0.05	0.08	0	0	0.13
66632	'Dph6'	18.4	25.09	15.7	32.8	15.9	8.23	13.2	27.92	17.39	15.86
666329	'Gm3317'	5	8.04	11.5	0	2.52	1.83	2.49	6.88	2.87	3.38
66634	'Mcm8'	0.6	1.21	0.46	1.92	0.02	1.08	0.08	2.03	0.06	0.12
666348	'Apol7e'	0.1	0.02	0	0.03	0.02	0	0.09	0	0	0.01
66637	'Tsen15'	38.6	50.84	49	21.1	63.7	44.04	46.9	41.78	19.44	52.22
66641	'Sike1'	8.05	9.34	3.27	4.78	5.29	4.48	5.24	8.24	4.02	9.06
66642	'Ctnnbl1'	31.19	37.41	13.5	45.4	55.5	15.4	23.5	41.43	8.63	19.99
666420	'Gm8094'	0	1.01	0.55	0	1.77	0	3.28	0	0	0
66643	'Lix1'	21.63	5.07	7.55	6.26	13.8	14.78	5.15	3.21	2.29	2.32
66645	'Pspc1'	11.93	22.29	22.5	16.9	33.8	48.36	22.7	37.99	21.61	31.78
66646	'Rpe'	2.42	6.97	8.51	11.7	14.2	12.22	6.43	7.87	12.31	4.45
666468	'Atg4a'	1.48	8.99	5.24	4.75	0.68	7.17	0.21	1.87	0.04	0.83
66647	'Nsmce3'	29.44	11.06	10	29.3	14.5	15.11	26.3	5.28	7.16	4.49
66648	'Tpgs2'	21.47	23	13.2	27.7	16.3	11.24	4.12	18.73	23.44	12.79
666485	'Gm8127'	0	0	0.1	0	0.06	0	0	0	0	0
666528	'Zfp541'	0	0.32	0	0	0	0	1.85	0	0.24	0
66653	'Brf2'	3.47	4.19	5.08	2.93	6.36	5.43	4.36	4.09	2.09	4.46
666532	'Zfp991'	1.43	0	0	0.16	0.84	2.92	0	0.06	0.9	1.7
66656	'Eef1d'	44.22	64.83	63	52.9	50.4	78.52	119	63.63	63.91	68.34
666561	'Gm8165'	0	0	0.16	0	0.62	0	0	0	0	0
66658	'Ccdc51'	3.75	2.04	8.59	6.51	0	2.88	0.64	1.9	0	0
666584	'BC024063'	0	0	2.5	0	2.27	2.26	0	7.3	2.56	0
66659	'Acp6'	31.33	8.11	7.09	26.8	8.63	8.37	0	8.17	13.55	11.35
66660	'Sltm'	9.32	22.45	21.5	37.5	24.2	23.62	23.2	15.37	16.63	14.99
66661	'Srp72'	26.07	29.15	29	34.4	30.6	23.58	44.5	26.3	36.32	36.96
66663	'Uba5'	14.43	19.45	9.8	57.7	16.4	19.99	28.7	21.53	56.3	5.52
66664	'Tmem41a'	42.3	22.15	44.3	18.6	24.1	42.84	67.1	24.31	34.14	41.68
66665	'Msantd3'	0.86	5.16	2.85	0	4.22	4.61	9	0.82	2.77	0.33
666660	'Gm8220'	0	0	0.57	0	0.23	0	0	0	0	0.06
66667	'Hspbap1'	1.81	0.02	0	0	2	6.11	0	1.1	0	1.47
666675	'Gm8229'	0	0	0.14	0	0	0	0	0	0	0
666678	'Gm8232'	0	0	0.2	0	0	0	0	0	0	0
666704	'Samd1'	1	0	0	0	0.86	1.76	0	1.59	0	1.71
66671	'Ccnh'	42.59	24	31.6	27.3	36.8	46.14	23.4	47.29	36.14	26.5
666723	'Gm8256'	0	0	0.42	0	0.78	0	0	0	0	0
66673	'Sorcs3'	7.1	8.48	2.25	5.74	6.41	2.51	1.95	9.34	3.5	3.11
666731	'Trim43c'	0	0.02	0.11	0	0	0	0	0	0	0

66674	'Spryd7'	50.7	64.36	66.6	44	37.5	55.17	51.4	51.86	65.42	45.06
666747	'Trim43b'	0	0	0.02	0	0	0	0	0.28	0.02	0.1
666750	'Gm8271'	0	0	0	0.19	0.44	0	0	0	0	0.07
66676	'Tmed7'	6.9	3.44	3.73	37.8	3.27	1.72	6.88	2.66	0.81	1.84
66679	'Rae1'	75.56	63.33	91.9	52.7	66.6	65.27	62.2	52.02	47.97	24.52
666794	'Rbm24'	2.28	8.68	1.39	1.73	1.62	3.51	2.23	3.3	0.01	2.04
66680	'Oser1'	25.55	46.09	22.8	77.7	41.9	34.18	13.8	22.95	31.77	32.15
666808	'Gm10410'	0.04	0.88	2	0.35	1.65	1.49	0.05	0	0.92	0.78
66681	'Pgm1'	1.8	0.88	0	0.12	6.85	3.7	5.42	2.53	2.65	3.6
66682	'Trappc5'	68.4	34.61	19.3	24.5	41.5	45.64	33.4	34.44	22.69	70.34
66684	'Tceal8'	22.88	27.92	33.8	95.7	34	25.53	45.9	20.61	20.57	21.25
66686	'Dcbld1'	0	1.71	1.4	1.46	1.32	0	15.6	3.21	7.97	0
66687	'Tbc1d15'	3.8	24.92	8.93	44.1	13.3	13.73	21.3	19.06	30.38	15.64
66689	'Klhl28'	0.1	4.52	0.12	9.74	9.84	4.75	14.2	0.85	0.94	5.18
66690	'Tmem186'	22.12	13.41	19	0.04	4.16	0.52	5.52	20.02	27.53	7.24
66691	'Gapvd1'	11.7	13.52	9.79	9.94	9.29	13.87	13.8	4.66	6.88	14.69
666938	'Bend4'	3.62	0.03	2.92	0	0.86	2.23	2.05	1.89	1.6	2.09
66694	'Uqcrfs1'	213.2	183.4	238	151	230	254.7	213	210.3	242.2	237.06
66696	'Snx31'	0	0	0	3.24	0	0	0	0	0	0
66700	'Chmp3'	36.65	37.19	59.8	60.4	57.9	59.16	66.2	52.91	63.76	56.21
66701	'Spryd4'	15.33	1.58	10.2	11.8	10.5	13.63	45.8	4.98	7.88	2.86
667034	'Pnp2'	10.96	5.39	13.6	21.7	16.9	39.78	0.05	15.43	4.77	15.64
66704	'Rbm4b'	51.77	27.4	27.2	23	39.3	42.79	50.7	42.36	59.57	45.8
66705	'Dnase1l2'	0	0.32	0.06	0	0.06	8.44	0	4.57	0.12	0
667055	'Unc93a2'	0	0	0	0	0	0	0	0	0.08	0
66706	'Ndufaf3'	99.36	91.97	74	65.5	104	112.6	73.2	85.82	53.71	99.94
667060	'Vmn2r5'	0	0.06	0.16	0	0	0.15	0	0	0	0
667069	'Vmn2r6'	0	0.02	0	0	0	0	0	0	0	0
66707	'Nkapl'	3.57	2.01	1.44	0	0	10.97	0	1.85	3.62	4.05
667094	'Gm8453'	0	0	0.02	0	0	0	0	0	0	0
667103	'Gm13570'	0	0.07	0	0	0	0	0	0.5	0	0
66711	'Sbds'	81.71	78.73	90.4	128	105	115.5	64.3	102.7	122	113.72
667118	'Zbed6'	1.13	0.54	0.55	1.81	0.13	0.02	0.14	0.44	0.38	0.72
66713	'Actr2'	49.81	31.94	44.5	34.1	70.3	24.76	33.9	60.76	53.51	57.84
66714	'4921524J17Rik'	19.91	11.65	17.1	25.9	8.7	19.22	5.64	14.93	15.58	16.5
66715	'Henmt1'	0.13	0	0	0.02	3.83	0	4.61	1.67	4.33	0.01
66717	'Ccdc96'	2.89	2.61	0	10.1	1.55	0	0	0	0.54	4.16
66720	'Klhl10'	21.71	24.23	29.1	29.5	19.4	19.97	36	19.68	30.43	26.98
667214	'9930111J21Rik1'	0.34	0.23	0.08	0.81	0.14	1.49	0.37	0.09	0.15	0.23
66722	'Spag16'	6.18	10.28	0.03	11.3	7.34	3	0.08	0.36	3.93	0.99
66724	'Tab3'	0	0.16	0.06	0	1.24	0	0	0.28	0.01	0
667240	'Vmn1r121'	0	0	0	0.02	0	0	0	0	0	0
66725	'Lrrk2'	0.01	0.01	1.84	0.01	0.25	0	2.54	0.09	1.29	1.07
667250	'Gm12657'	2.03	3.6	3.15	3.05	1.46	2.05	5.52	1.53	1.07	1.4
667259	'Vmn1r118'	0	0	0.02	0	0	0	0	0	0	0
66729	'Ankrd61'	0	0	1.4	0	0	0	0	0	0	0
667292	'Vmn1r111'	0	0	0.02	0	0	0	0	0	0	0
66732	'4921530L21Rik'	0	0.53	0	0	0	0	0	0	0	0
66733	'Kcng4'	0	0.28	6.12	0	6.75	0	0	0	0.01	2.33
66734	'Map1lc3a'	404.9	227.6	398	393	266	229.3	339	287.6	307.5	88.85
66736	'Emc2'	76.68	74.01	73.3	79.7	104	54.83	31.6	74.05	57.84	62.56
667370	'Ifit3b'	0	0	14.5	31.5	2.3	0	0.03	2.88	2.8	0
667373	'Ifit1bl1'	4.88	0	0.04	0	0.07	6.77	5.46	1.52	0	0
667378	'Gm8600'	0	0	0	0	0	0	0	0	1.01	0
66740	'4931417E11Rik'	0	0	0.05	0	0	0	0	0	0	0
66743	'Rnf220'	30.69	29.08	14.3	9.3	33.9	5.19	22.2	18.13	13.57	30.84
66748	'Erich2'	0	0	0.23	0	0	0	0	0.07	2.36	0
66753	'Erlec1'	15.24	27.83	16.8	21.2	23.8	20.59	7.69	18.22	14.45	22.65

66756	'Cfap97'	3.62	3.8	3.37	8.38	4.37	2.25	9.43	6.46	9.21	3.76
66757	'Adat2'	2.25	0.07	0.1	0.05	6.51	0.03	4.98	0	3.21	0.03
667570	'Gm8708'	0	0	0	0	0	0	0	0	1.01	0
66761	'4933417A18Rik'	0	0.05	0	0	0	0	0	0	0	0
66763	'4933425L06Rik'	0	0	0	0	0	0	0	0	0	0.32
66765	'4933411K16Rik'	0	0	0	0	0	0	0	0	0	3.13
667663	'Myo3a'	0	0	0.68	0	0	0	0	0	0	0
66768	'Pacrgl'	3.14	4.76	2.7	29.3	1.53	0.03	0.83	0	4.47	0
66771	'Gid4'	9.49	6.26	5.57	6.54	4.87	4.32	7.04	4.14	2.97	2.34
66773	'Gm17019'	0.11	0.13	0.14	0	0.17	0.56	0.28	0	0	0.06
667742	'Piezo2'	1.31	0	0	0.09	0.16	0	0.01	0	0	0.04
66775	'Hacd4'	0	0	3.03	3.82	0	0	0	0.79	0	1.84
667772	'Myh15'	0	0.18	0.01	0	0	0	0	0	0.01	0
667803	'H2-T-ps'	0.18	6.08	4.06	1.76	0	0.19	0	0.08	1.58	6.9
667823	'Trim5'	0	2.35	0	0	0	0.01	0	0	0	0
66784	'Fam187a'	0	0	0.03	0	0	0	0	0	0	0
66787	'Gskip'	56.66	52.48	43.8	15.6	48.6	38.48	39.3	63.94	30.02	49.82
66789	'Alg14'	19.1	49.93	8.17	32.6	16.4	26.88	24.4	27.53	42.29	73.53
66790	'Grtp1'	5.78	10.8	6.11	0.27	0.04	0	3.66	15.03	0.62	7.44
66793	'Efcab1'	39.57	31.42	16.6	18.7	23.9	35.64	18.8	28.21	19.37	39.6
66795	'Atg10'	19.7	11.97	23.5	33	17.6	18.11	11.1	16.54	9.18	36.19
667962	'Zfp966'	2.12	0.9	0	1.8	1.28	0.09	0.51	1.25	0.54	1.11
66797	'Cntnap2'	101	33.93	58.3	55	25.6	62.99	39	109.9	11.15	20.2
667977	'Gm8909'	0.02	0.08	0.18	2.68	1.92	0.1	0	0.09	1.19	0.07
66799	'Ube2w'	35.56	45.55	19.2	38.3	31.2	24.46	35.1	28.87	36.23	32.27
66801	'Prkrip1'	13.31	20.56	19	2.5	17.8	16.25	20.5	24.4	25.23	25.07
668035	'Olfr687'	0	0	0	0	0.09	0	0	0	0	0
668039	'Gm14434'	0	0.4	0	0	0.97	0	0	0.26	1.92	0.3
66808	'9030624G23Rik'	0.36	0.53	1.46	1.12	0.73	0.54	3.06	3.39	8.15	0.39
66809	'Krt20'	1.56	2.74	0	0	2.99	0	0	0.14	0	0
66810	'Rbm22'	6.89	11.93	8.21	33	9.69	6.37	1.54	9.85	15.1	17.26
668110	'Syce1l'	0	0	1.12	0	0.47	0	0	0	0	0
66812	'Ppcdc'	10.07	8.63	7.82	14.6	5.87	28.43	18.8	18.85	20.39	5.57
668137	'Gm8994'	0	0	0	0	0	0	0	0	0	0
66815	'Mcub'	3.04	0	0	0	34.4	0.04	0	0	4.02	10.63
668158	'Ccdc85c'	5.08	2.92	8.64	8.14	4.18	1.11	4.37	2.79	8.94	7.38
66816	'Thap2'	0.41	1.11	2.99	2.41	1.8	1.18	0.23	3.11	0.42	0.51
668166	'Zxdb'	0.33	1.15	0.21	0	0	0.17	0	0.2	1.32	1.31
66817	'Tmem170'	0.76	3.34	0.75	1.99	6	4.65	1.7	2.59	6.08	2.27
668173	'Pex10'	12.99	12.47	11.4	11.3	13.2	3.64	17.2	12.85	17.64	15.33
66818	'Smim7'	27.27	24.35	32.9	38.4	38.5	36.28	31.6	38.3	17.2	36.72
668208	'Gm13288'	0	0	0	0	0	0	0	0	0	0.01
66821	'Bcs1l'	15.95	28.02	9.65	19	6.52	40.72	29.7	31.21	21.54	15.73
668212	'Efr3b'	7.19	4.17	15.8	10.7	6.55	5.07	13.9	4.97	7.94	8.18
668218	'Bin2'	5.25	2.82	0.05	18.9	9.7	42.15	6.61	0.76	0	3.11
66822	'Fbxo25'	70.84	51.93	39.4	33.5	47.1	53.69	40.5	113.5	52.06	77
668225	'Fignl2'	0	0	0	0	0	0	0	0.02	0.27	0
66824	'Pycard'	2.66	1.48	1.87	2.48	0.62	6.32	1.29	0.11	9.53	1.89
66826	'Taz'	60.68	50.28	50.4	51.1	105	105.7	51.4	77.88	63.13	106.81
66827	'Ttc1'	82.84	68.8	71.5	108	67	64.4	82.2	62.81	114.7	69.27
66830	'Nacc1'	1.34	0.17	1.32	0.01	1.8	0.34	1.05	1.23	2.18	1.63
668303	'Kif26a'	0.4	0.27	0.01	1.4	0.37	0.57	0.86	0.14	0.01	0.05
668310	'Cc2d2b'	0	0.24	0	0	0	0	0	0.01	0	0
66832	'Rsph3a'	16.85	30.05	33.9	25	18.4	35.66	25.3	27.39	28.31	20.74
668339	'Gm9112'	0	7.1	0	0	0	0	0	0	0	0
66834	'Acot13'	152.9	160.3	130	302	127	233.1	160	227.8	196.2	272.21
66836	'Tmem223'	137.1	86.02	49.9	72.4	50	112.1	127	77.48	92.62	113.95
66839	'Dele1'	29.55	12.5	26.7	3.85	18.2	11.77	9.91	12.45	23.26	33.59

66840	'Wdr45b'	44.9	45.29	48.3	55.8	54.6	54.64	44	58.99	50.95	70.14
66841	'Etfdh'	29.13	26.51	11.5	34.3	25.1	9.7	36	19.1	9.3	28.95
66844	'Ormdl2'	17.9	24.97	12.1	9.36	12.2	6.49	15.8	7.46	21.43	10.56
66845	'Mrpl33'	121.5	134.7	137	305	117	144.1	143	96.59	134.9	132.03
66847	'Hint3'	7.82	10.29	5.5	0.09	9.12	0	9.23	8.22	8.95	21
66848	'Fuca2'	30.24	35.41	40.9	30.7	28.9	53.6	20.7	39.32	40.87	59.13
66849	'Ppp1r2'	29.76	41.42	47	49.1	22.8	27.98	24.6	28.02	51.99	26
668501	'Zfp507'	2.73	1.12	1.01	4.04	0.07	1.2	0	0.31	3.01	0.59
66853	'Pnpla2'	8.38	19	12.8	18	6.74	2.73	3.47	16.85	6.25	4.72
66854	'Trim35'	80.8	89.72	66.5	75	67.9	37.69	46.8	118.2	100	95.37
66855	'Tcf25'	273.4	285	376	130	286	234.9	373	332.6	277.8	246.84
66857	'Plbd1'	0.16	0	0.03	0	0	0	0	0.02	0	0
668588	'Gm9257'	0.54	0.07	1.09	0	0.15	0.37	0.22	0.51	2.56	0.33
66859	'Slc16a9'	0	0.18	0	0	0	0	0	0	0	0
66860	'Tanc1'	0.12	0	0	1.07	0.02	0.1	0.06	0.48	0.68	0
668605	'Gm9265'	0.05	0.09	0	0	0.82	0	0.09	0.26	0.12	0.02
66861	'Dnajc10'	13.31	34.93	44.3	14.9	35.2	8.45	26.4	33.85	46.17	33.51
668620	'Zfp936'	0.03	0.02	0.03	0	0.05	0	0	0.09	0	0.1
66863	'Lztr1'	40.86	36.7	39.3	54.6	48.9	22.23	40.8	62.8	56.37	44.44
66864	'Clec14a'	0	0	0	0	0	0	2.73	0	0	0
66865	'Pmpca'	32.86	32.97	35.3	56.4	32.9	31.72	28.4	30.56	48.79	24.4
66866	'Nhlrc2'	4.41	2.01	4.7	0.68	1.58	6.33	1.59	0.21	2.97	4.77
668661	'2410002F23Rik'	97.19	122.8	138	71.3	70.2	107.3	176	117.7	138.9	136.41
66867	'Hmg20a'	44.11	35.28	42.4	23.2	25.1	21.18	36.6	19.62	15.64	32.93
66868	'Mfsd1'	12.21	12.56	11.6	5.28	14.6	9.99	5.1	5.56	41.55	18.83
66869	'Zfp869'	15.03	13.81	16.2	18.1	12.5	3.36	13	6.9	15.34	11.55
66870	'Serbp1'	18.47	21.17	16	40.5	14	20.43	31.5	20.18	19.12	14.9
66871	'Cpne8'	5.73	12.24	11.7	43.6	56.7	24.74	20.3	11.22	32.9	14.8
668725	'Mrgpra9'	0	0	0.09	0	0	0	0	0	0	0
66873	'Tril'	1.56	6.83	5.04	0.01	1.41	5.56	12.6	7.66	3.99	1.19
66874	'Ncbp3'	1.92	3.76	1.71	2.55	4.16	1.81	1.28	6.15	0.39	2.01
66875	'Swt1'	4.43	3.92	1.33	3.95	2.44	1.25	6.33	2.36	3.48	0.88
66877	'Crnkl1'	4.31	4.46	5.55	13.8	2.32	1.7	10.1	5.43	2.42	3.51
66878	'Riok3'	20.56	15.88	11.9	25.9	12.5	11.91	10.2	10.86	12.91	24.75
66880	'Rsrc1'	19.74	22.49	38.2	48.9	28.9	17.91	35.4	29.46	31.96	22.21
66881	'Pcyox1'	41.03	66.31	46.2	17.8	51.8	29.08	31.9	42.78	66.79	53.07
66882	'Bzw1'	32.51	44.63	41.6	26.5	39.3	9.77	36.4	25.02	27.44	23.31
66884	'Appbp2'	20.49	16.5	11.3	29.3	18	64.79	17.5	25.05	30.46	25.22
66885	'Acadsb'	39.94	30.92	30	19.7	37.3	29.13	34.4	24.21	26.61	26.55
66887	'Lonp2'	48.71	54.86	74.3	37.2	55.1	74.73	74.8	65.03	115.6	107.38
668880	'Stard9'	0	0.12	0.48	0.38	0.04	0.6	0.55	0.21	0.24	0.1
66889	'Rnf128'	18.31	56.01	49	30.1	39.3	60.25	42.1	16.32	118.7	19.62
668894	'Gm14025'	2	0.31	0.45	0.97	0.08	0.07	0.08	0.03	0.76	0.02
66890	'Lman2'	38.49	29.87	26.9	22.7	17.7	30.73	34.7	27.15	65.19	41.6
66892	'Eif4e3'	9.57	7.84	1.33	2.03	6.8	6.28	3.37	3.43	1.95	8.55
668923	'Zfp442'	1.38	4.44	2.01	1.43	3.92	2.15	0.41	0.55	4.06	2.77
668929	'Rad21l'	0.97	5.07	0.87	0	1.12	2.39	2.97	2.47	2.69	0.92
66894	'Wwp2'	13.5	16.59	8.87	8.44	16.2	15.04	7.5	17.19	28.57	10.88
668940	'Myh7b'	1.89	1.47	2.35	0.12	2.09	2.43	4.78	1.75	1.44	0.9
66895	'Pxdc1'	0	0	3.49	0	0	0	0	1.4	0.05	0
66897	'Naa16'	10.53	4.04	2.09	0.61	6.25	4.83	0.78	6.09	3.69	10.46
66898	'Baiap2l1'	0	0	0.54	3.28	0	0.67	0	0	0	0.21
66899	'Fip1l1'	19.02	13.06	14.6	8.18	6.17	9.7	8.12	14.87	16.28	12.54
66901	'Proz'	1.77	0.26	0	0	0.04	0	0.09	0.04	0.2	0.25
66902	'Mtap'	3.47	2.76	7.42	6.46	0.77	6.68	15.5	5.72	3.03	14.5
66904	'Pccb'	8.76	22.64	34.2	24.2	29.8	16.49	14.6	17.36	9.77	11.75
66905	'Plin3'	1.65	3.38	1.27	0.16	3.92	0.11	1.58	1.97	0.48	0.04
66910	'Tmem107'	25.85	21.87	15.9	39	20.1	68.41	23.1	28.11	16.79	65.62

66911	'Nudt16l1'	60.68	129.1	103	161	107	112.2	116	101.8	91.98	77.4
66912	'Bzw2'	17.14	31.34	37	12.4	14.1	24.04	23.6	32.25	16.67	14.24
66913	'Kdelr2'	71.53	71.51	37.4	101	62.9	23.33	46.7	44.7	45.41	59.03
66914	'Vps28'	528.4	359.9	501	400	467	644	289	569.7	485.3	575.37
66915	'Cops9'	297.8	349.3	319	383	296	595.6	193	160.6	339.2	199.33
66916	'Ndufb7'	340	313.2	404	469	325	540.1	435	426.6	466.5	369.78
66917	'Chordc1'	86.13	117.7	37.4	40.5	30.8	60.13	55.7	52.02	58.29	81.42
66921	'Prpf38b'	18.32	20.6	22.6	30.2	10.2	7.8	20.5	15.53	10.1	9.4
66922	'Rras2'	0	0.15	0.77	0	0.89	0	0.02	0	0.08	0.04
66923	'Pbrm1'	3.57	5.31	3.56	4.86	6.59	3.89	3.06	6.22	4.55	4.43
66925	'Sdhd'	288	238.7	277	218	257	391.2	217	245.8	201.6	320.19
66926	'Trmt6'	14.58	4.15	13.3	12.9	9.74	6.77	11.5	8.46	12.29	10.24
66928	'Dmac1'	74.86	78.66	43.2	53.1	75.1	85.89	68.4	62.29	53.55	37.56
66929	'Asf1b'	0	0	3.62	11.4	9.7	0	0	0	0.05	0
66930	'Fank1'	10.49	11.91	19.4	20.5	27.3	6.23	4.09	6.99	19.04	30.26
66931	'1700010I14Rik'	14.59	8.01	2.88	9.07	0.19	0.27	3.58	9.4	2.9	4.18
66932	'Rexo1'	2.62	2.69	4.65	8.42	7.96	4.9	6.92	1.76	5.62	0.07
66934	'Dsn1'	1.83	6.74	0.06	0	1.85	1.32	0.11	0.19	0.02	1.77
66935	'Cir1'	13.6	18.62	24.1	14.3	24.2	16.76	14.3	9.09	9.09	9.82
66938	'Sh3d21'	0	0.86	0.83	0	2.58	2.04	0.09	0	0	0
66939	'Aagab'	52.2	18.92	17.3	16.9	42.8	14.74	46.4	48.24	42.84	58.02
66940	'Shisa5'	69.47	94.16	63.3	157	61.5	153.2	95.9	153.7	205.2	194.49
66942	'Ddx18'	13.24	14.95	14.5	26.1	12.7	12.73	23.9	9.37	19.31	11.04
66943	'Pqlc1'	20.53	25	15.5	42.5	13.6	19.87	38.4	10.02	46.63	25.87
66945	'Sdha'	284.1	328.6	371	216	296	404.9	353	361	373.6	412.68
66948	'Acad8'	25.09	21.46	10.9	20.7	25.3	21.81	41.2	21.39	22.48	18.58
66949	'Trim59'	0.03	5.66	0	0.29	0.22	0.32	0	0.83	0.01	1.23
66950	'Tmem206'	9.56	22.16	15.2	19	11.6	18.35	15.6	13.09	19.49	19.21
66952	'2310030G06Rik'	0	3.48	2.97	0	1.42	4.6	0	7	10.12	2.64
66953	'Cdca7'	0	0	0	0	0	0	3.78	0	0	2.59
66958	'Tmx2'	456	396.5	419	275	395	524.1	531	500.6	414.4	527.6
66959	'Dusp26'	171.6	219.3	162	167	193	225.3	239	149.9	219.3	141.97
66960	'Mindy3'	10.22	10	14.7	27.6	11.6	25.79	4.69	12.45	16.19	12.03
66962	'Swsap1'	19.11	19.71	3.48	6.64	11	12.33	0.16	14.81	18.88	4.17
66964	'Golt1b'	69.27	48.85	35.7	73.3	63	57.13	24.4	69.5	50.9	75.64
66965	'Ctu2'	17.23	16.71	21.6	6.67	7.19	14.13	11.4	31.52	26.44	20.93
66966	'Trit1'	10.44	10.52	4.09	9.31	1.68	1.67	8.7	11.53	11.6	12.45
66967	'Edem3'	1.64	2.69	3.3	5.18	1.63	1.23	0.58	1.34	2.29	1.19
66968	'Plin5'	3.44	2.42	3.81	5.35	0	15.77	0.03	3.32	0.03	0
66970	'Ssbp2'	76.34	48.78	96.7	33.2	92.7	60.6	40.5	38.7	51.29	63.19
66971	'Cdk5rap1'	14.65	8.82	2.32	2.87	11.8	6.19	4.86	6.79	11.96	11.48
66972	'Slc25a23'	5.58	5.22	8.7	3.58	10.2	2.32	6.62	3.9	8.3	1.95
66973	'Mrps18b'	36.65	52.66	55.8	61.2	60.2	89.67	26.6	44.85	32.76	55.66
66975	'Trappc13'	36.27	33.9	25.2	15.7	24	11.17	23.3	33.22	25.93	45.12
66977	'Nuf2'	0.96	0	0	0	0.02	0	0	0	0	0
66978	'Luc7l'	23.4	17.59	22.1	16.7	22.5	26.61	18.1	15.7	24.7	24.7
66979	'Pole4'	20.13	12.58	13.1	35.9	35.2	35.43	14.9	16.62	40.44	9.09
66980	'Zdhhc6'	19.3	16.35	17.7	5.75	38.8	5.41	19.6	41.08	44.74	21.22
66983	'Zfp830'	4.15	4.52	7.35	3.8	8.44	1.61	2.09	4.15	7.38	1.34
66985	'Rassf7'	0	2.03	0	0	5.16	0.03	0	3.41	4.95	4.5
66988	'Lap3'	13.22	20.36	13.8	26.3	14	17.59	49.7	17.7	16.96	15.78
66989	'Kctd20'	27.86	18.59	22.2	20.9	22.9	7.83	16.6	18.44	17.09	20.72
66990	'Tmem134'	20.56	21.73	10.8	11.4	14.9	27.16	21.9	10.27	10.78	10.42
66991	'Khdc3'	0.29	1.15	0	3.13	0	0	0	0	0.57	0
66993	'Smarcd3'	14.68	4.43	8.57	12.1	17.4	12.82	10.3	16.77	12.13	7.13
66994	'Cep19'	65.07	43.28	41.7	81.9	40	54.1	71.3	64.22	46.09	78.77
66995	'Zcchc18'	825.2	993.1	812	981	754	795.7	880	1085	832.9	711.76
66997	'Psmd12'	114.2	122.4	125	138	142	151.5	86	137.9	108.5	119.46

66998	'Psmd5'	55.73	77.78	69.5	69.7	69.8	83.38	73.9	79.23	64.96	83.29
66999	'Med28'	53.84	52.84	40.2	76.5	63.2	66.37	43.2	63.05	54.91	67.69
67003	'Uqcrc2'	141.9	183.2	200	136	153	184.3	211	224.5	158.8	197.75
67005	'Polr3k'	18.67	32.06	22	30.9	29.4	51.67	31.7	25.3	33.68	31.69
67006	'Cisd2'	35.8	34.23	24.9	28.3	16.8	18.71	29.5	25.71	26.27	29.19
67008	'Yae1d1'	9.86	11.6	5.41	0.02	12	29.11	12.1	11.79	8.68	21.03
67009	'Ttc23'	0	0	0	0	0	0	0	0	0	0.56
67010	'Rbm7'	23.74	15.57	8.04	35.3	21.3	22.93	15.1	27.8	13.81	23.58
67011	'Mettl6'	0.02	7.97	11.3	32.1	16.3	4.11	6.29	12.03	5.47	10.7
67013	'Oma1'	8.84	2.14	10	0.02	8	12.36	0.21	7.72	3.77	4.57
67014	'Riox2'	8.99	19.75	2.94	3.55	7.63	11.57	9.78	8.43	11.76	17.91
67015	'Ccdc91'	28.67	22.34	19.9	9.22	20.6	23.51	19.1	38.82	49.65	31.39
67016	'Tbc1d2b'	1.54	0.09	0	0.13	0.19	0	0.04	0.45	2.12	0
67017	'Fam210b'	6.39	3.52	5.59	14.7	8.55	10.98	4.32	9.23	4.22	6.43
67019	'Actr6'	25.9	35.12	20.2	31.2	25.6	10.51	15.1	24.07	38.01	40.36
67020	'Tmem88'	0.05	0.28	1.82	2.6	0.18	0.27	0.07	1.35	0.09	0.23
67023	'Use1'	104.7	87.48	72.9	118	75	94.48	68.5	92.63	109.9	120.56
67025	'Rpl11'	249.3	270.4	245	342	247	322.8	365	221.6	148.3	251.92
67026	'Thap4'	12.87	11.49	15.8	21.9	29.2	46.46	24	19	15.01	19.24
67027	'Mkrn2'	17.85	16.31	14.3	12.6	8.38	8.52	10.8	9.34	19.44	5.14
67028	'2610002M06Rik'	4.86	3.39	6.03	6.42	6.13	1.59	7.53	5.59	1.51	4.35
67030	'Fancl'	7.51	12.08	7.13	0.14	13.1	6.87	0.6	4.23	6.2	5.44
67031	'Upf3a'	4	1.84	10.6	15.2	13.8	11.51	8.42	5.67	6.48	5.47
67035	'Dnajb4'	49.49	45.59	28.3	52.6	42.9	41.52	35.6	39.69	45.17	55.77
670358	'Gm9484'	0	1.82	0	0	0	0.25	0	1.35	0	0
67036	'Mrpl45'	22.86	23.18	40.6	34.4	31.4	33.37	35.4	43.62	35.97	51.22
67037	'Pmf1'	25.28	4.64	19.8	30.8	20.9	27.5	13.3	15.03	10.68	15.98
67038	'2010109I03Rik'	0	0	0	0	0	0	0	0	0.02	0
67039	'Rbm25'	41.24	33.51	30.9	40.9	41.3	48.08	47.6	34.4	32.28	26.68
67040	'Ddx17'	22.77	25.51	29	31.3	25	33.78	30.5	20.14	26.05	28.97
67041	'Oxct1'	47.07	36.63	61.1	41.9	44.9	37.8	55.1	41.26	29.68	32.59
67042	'Ift27'	48.7	55.19	58.1	63.8	62.6	60.85	53.2	64.3	70.98	80.42
67043	'Syap1'	5.46	4.84	1.01	12	2.16	3.85	2.09	2.93	1.96	0.57
67044	'Higd2a'	316.6	300.6	293	240	223	351.1	274	293.5	223.5	208.14
67045	'Riok2'	13.07	9.89	7.4	5.83	10.9	4.37	2.56	8.3	4.6	16.49
67046	'Tbc1d7'	32.28	26.07	34.9	53.6	54.1	22.63	30.2	16.81	28.67	17.19
67048	'Vma21'	12.27	18.68	16.4	27	12.3	18.14	12.3	19.94	15.12	17.9
67049	'Pus3'	10.2	3.63	6.32	0.6	7.31	0.77	6.03	8.92	11.99	5.03
67050	'Nkap'	3.77	4.46	3.76	7.04	2.66	7.1	2.35	4.35	2.83	4.17
67052	'Ndc80'	0	0	0.02	0	0	0.06	0	0.09	0	0
67053	'Rpp14'	21.97	11	23.7	33.8	18.8	16.45	2.12	15.99	9.68	15.89
67054	'Paics'	93.23	84.53	82.8	82.6	71.5	83.35	91.6	89.63	74.28	74.85
670558	'H60c'	0	0	0	0	0	0.52	0.09	0	0	0
67057	'Yaf2'	32.69	29.76	30.7	70.4	37.8	40.74	24.3	18.18	18.17	24.94
67059	'Ola1'	36.92	51.64	36.1	51.2	47.1	64.66	31.3	34.83	41.69	31.05
670593	'Gm9495'	0.03	0	0.02	0	0	1.24	0	0	0.04	0.01
67062	'Slc25a53'	0.9	1.06	1.28	1.36	1.5	1.47	1.35	1.31	2.62	1.4
67063	'Tmem246'	99.57	98.67	92.8	89.2	45.6	67.64	48.3	68.84	115.5	132.98
67064	'Chmp1b'	12.53	11.6	4.6	8.71	14.5	13.07	10.8	8	12.82	10.87
67065	'Polr3d'	19.45	24.31	11	11.4	16.7	20.58	12.5	14.34	16.54	23.17
67067	'Romo1'	286	305.7	337	514	194	427.7	295	153.6	418.2	236.6
67068	'Dynlrb1'	801	534.4	720	643	710	942.8	774	807.9	865.8	799.36
67070	'Lsm14a'	8.53	3.83	5.87	13	3.77	2.82	2.67	10.56	4.45	7.46
67071	'Rps6ka6'	7.12	2.26	2.77	0.14	0	0.63	3.81	1.73	4.63	11.21
67072	'Cdc37l1'	31.82	14.2	18.1	9.79	30.9	11.9	28.1	13.98	11.98	5
67073	'Pi4k2b'	0	0	0	0	0.44	0	0	0	0	0
67074	'Mon2'	0.76	5.03	8.12	5.4	7.51	3.5	2.19	4.65	0.44	1.37
67075	'Magt1'	2.98	8.24	1.92	0.52	8.03	0.03	0.2	1.77	2.5	0.89

67077	'Catsperz'	0	0	1.17	0	0	0	0	0	0	0
67078	'Pgp'	88.21	51.44	102	143	65.3	65.73	93.1	62.79	67.76	34.14
67080	'1700019D03Rik'	1.77	6.71	17.4	11.2	1.9	22.32	8.52	4.51	21.1	9.18
67082	'ccdc198'	0	1.23	0.32	0	0	0	1.28	0	0	0.37
67087	'Ctnnbip1'	3.7	7.35	11.9	31.1	5.43	0.42	4.23	2.85	6.54	5.35
67088	'Cand2'	0.78	1.16	2.42	2.2	0.17	0.11	0.03	1.65	6.73	4.94
67089	'Psmc6'	72.63	89.98	80.5	31.9	73.3	97.82	124	74.33	67.67	55.09
67091	'Trappc6a'	28.82	15.54	8.15	17.5	27.4	33.69	15.3	11.31	25.9	12.7
67092	'Gatm'	37.18	20.72	37.9	13.8	54.5	74.06	35.1	14.64	15.4	8.32
67095	'Trak1'	5.63	4.76	11.7	4.9	7.33	0.02	4.77	5.3	3.92	4.57
67096	'Mmachc'	22.49	2.62	5.75	18.6	23.6	24.6	1.96	18.52	13.23	19.29
67097	'Rps10'	177.7	182.9	173	157	236	247.3	130	134.5	125.6	203.06
67099	'Mettl21a'	12.7	15.61	8.99	0	12.6	5.52	8	8.81	13.04	5.5
67101	'2310039H08Rik'	26.62	18.84	19.7	25.2	27.2	30.55	10.7	29.72	9.87	18.88
67102	'D16Ertd472e'	0	0.11	0	4.07	1.16	0.01	0.02	0.01	0	0.69
67103	'Ptgr1'	0	0	0	0	0	0	0	0	0	0.03
67105	'Timm21'	27.42	8.34	20.3	13.3	6.16	10.89	26	16.34	21.44	17.05
67106	'Zbtb8os'	39.58	7.58	13	42.8	47.9	34.89	31.8	21.37	14.19	41.36
67109	'Zfp787'	0.19	0.28	0.1	1.36	0.39	4.17	1.02	0.96	2.55	1.58
67111	'Naaa'	23.4	28.6	20.8	14.4	12.6	20.72	15.9	26.2	9.77	18.44
67112	'Fgf22'	8.25	7.89	11.8	15.9	23.5	44.53	4.96	8.95	15.84	6.89
67115	'Rpl14'	212.2	182.8	195	194	178	174	187	136.3	199.5	189.16
67116	'Cuedc2'	162.4	118.1	191	159	157	135.6	209	193.8	173.2	157.41
67117	'Dynlt3'	64.24	77.61	68.6	68.1	84.7	49.24	65	61.11	40.33	61.9
67118	'Bfar'	28	24.31	15	17.3	36.4	11.44	16.6	24.2	30.06	34.25
67119	'Ccdc159'	7.79	12.05	6.4	5.58	10.5	5.42	21.6	12.41	6.12	19.34
67120	'Ttc14'	18.71	26.34	18.9	11.3	18.3	14.73	19	10.32	22.76	6.43
67121	'Mastl'	0	0.05	0	0.01	0	0	0	0	0	0
67122	'Nrarp'	0	2.77	1.04	2.08	0	2.3	4.28	0.03	0	1.13
67123	'Ubap1'	25.94	21.93	26.3	31.9	22.4	20.8	28	30.5	8.45	28.93
67125	'Tspan31'	115.3	89.18	67.6	165	80.1	120.7	103	60.29	80.66	124.17
67126	'Atp5e'	364.6	367.9	485	561	337	472.4	385	235.6	335.8	326.83
67128	'Ube2g1'	2.82	3.78	1.37	8.51	2.75	3.5	3.09	5.18	0.02	1.65
67130	'Ndufa6'	476.7	393.8	489	513	418	763.7	512	386.6	499.5	428.34
67131	'Acbd4'	8.94	11.67	21.2	9.48	15.7	24.29	7.46	16.67	15.34	5.46
67134	'Nop56'	124.9	143.7	137	268	134	184.6	98	196.8	150.5	141.58
67136	'Kbtbd4'	39.76	42.25	54.3	27.3	40.6	37.88	31	26.61	27.2	51.15
67138	'Herc6'	0.11	0.13	0.31	2.34	0.09	0.02	0	0.01	2.89	1.23
67139	'Mis12'	9.28	11.36	9.41	18	19.6	11.82	0	3.82	14.16	1.59
67141	'Fbxo5'	2.6	1.58	0.35	6.85	2.27	0	1.95	0	0	3.01
67143	'Ikzf5'	0.68	0.66	4.4	5.38	1.66	6.04	0.01	3.6	1.53	8.43
67144	'Lrrc40'	12.32	17.72	19.1	14.4	17.6	14.6	23.8	10.45	17.54	15.51
67145	'Tomm34'	162.7	129	105	65.9	87.1	101.8	129	111.3	109.6	162.28
67148	'Ramac'	217.5	149.4	136	134	190	160.7	164	154.8	163.8	202.44
67149	'Nkain1'	1.22	6.35	0.47	0.67	7.79	0.02	4.31	6.65	0	1.86
67150	'Rnf141'	3.5	6.95	3.53	17.2	13.2	3.9	10.6	10.38	5.11	7.5
67151	'Psmd9'	33.74	30.22	32.9	18	16.3	28.19	25.5	31.83	23.02	21.55
67153	'Rnaseh2b'	10.5	13.51	16.7	7.07	14.4	27.41	14.4	21.36	14.8	13.96
671535	'Parp10'	0	0	0	5.92	0.53	0	0	0	0.58	3.08
67154	'Mtdh'	22.48	19.32	17.6	17.5	18.6	13.62	10.3	20.3	25.04	12.9
67155	'Smarca2'	76.91	60.84	72.4	115	58.6	58.2	52.6	63.53	60.31	46.43
67157	'2610301B20Rik'	65.03	71.63	56.1	89.2	70.4	69.21	53.9	59.39	33.03	67.96
67158	'Sft2d3'	1.6	1.54	0.27	9.23	0.85	0.02	1.03	0.21	0.98	0.23
67160	'Eef1g'	154.1	172.6	162	391	153	126.1	185	172.4	130	160.53
67161	'Sclt1'	5.22	0.69	2.08	2.24	5.59	2.46	3.56	0.19	1.75	0.22
67163	'Ccdc47'	57.85	42.74	63	54.1	64.2	60.53	58.1	54.2	75.78	65.26
67164	'Lipt2'	3.34	9.99	9.33	0	0.04	4.65	0	4.78	1.61	0
671650	'Gm20783'	0.23	0	0	0	0.03	0	0	0.08	0.04	0

67166	'Arl8b'	86.45	77.72	89.6	49.1	69.2	62.57	105	71.99	56.95	60.83
67168	'Lpar6'	4.62	18.99	5.82	18	1.07	6.73	2.95	0	0.02	0.03
67169	'Nradd'	9.74	3.51	3.05	11.1	9.28	0	1.24	2.76	0.08	10.96
67171	'Dram2'	8.29	19.92	11.1	16.5	9.65	10.17	16.6	24.27	7.96	18.93
67177	'Cdt1'	0	3.53	0.15	0	0	5.55	0	2.05	0	0
67178	'Zmat5'	42.25	31.99	23.8	80.6	41.6	86.17	43.7	67.88	59.76	46.57
67179	'Ccdc25'	21.69	32.88	33.2	50.3	51.5	39.33	4.89	20.3	13.05	20.73
67180	'Yipf5'	45.48	44.36	35.7	46.6	29.2	34.72	33.5	33.65	36.76	31.26
67181	'Ctdnep1'	8.41	18.14	10.6	2.42	16.5	4.23	4.72	4.04	1.42	8.67
67182	'Pdzk1ip1'	0	6.53	1.9	0	0	0	0	0	0	0
67184	'Ndufa13'	469.3	531.4	533	575	586	887.6	626	412.9	656.9	656.55
67186	'Rplp2'	312	338.7	275	326	229	365.5	323	178.6	366.7	221.84
67187	'Zmynd19'	0.82	0	1.53	0	1.03	0	2.46	0.38	0	1.48
67196	'Ube2t'	2.72	3.44	0	0	0	0	0	0	5.38	0
67197	'Zcrb1'	83.03	79.05	104	110	128	104.1	135	113.2	132.6	126.08
67198	'Spats2l'	12.02	12.89	22.1	0.03	7.97	17.31	14.4	16.66	7.64	2.3
67199	'Pfdn1'	148.7	107.9	95.5	152	128	172.2	126	158.9	182.4	121.5
67200	'Ccdc77'	0.08	6.02	0	2.64	5.48	2.33	0	2.29	0.02	0.3
67201	'Glod4'	80.55	56.2	61.8	102	38	35.84	31.8	54.48	60.64	60.77
67203	'Nde1'	1.33	1.63	12.7	0.09	0	0.03	5.61	5.85	4.4	1.24
67204	'Eif2s2'	39.66	40.05	39.9	34.9	40.6	31.03	30.2	34.33	44.01	40.52
67205	'Utp11'	23.82	27.3	22.6	28.2	19.2	19.81	37.8	32.83	43.33	33.94
67207	'Lsm1'	29.11	33.85	31.3	32.3	38	43.23	25.2	37.16	38.82	27.15
67210	'Gatad1'	31.33	24.31	23.4	41.5	44.8	19.28	18.9	36.8	43.09	31.12
67211	'Armc10'	2.69	14.88	9.18	1.5	11.8	11.39	15.9	16.28	5.37	5.74
67212	'Mrpl55'	76.29	102.2	76.7	92.5	123	158.5	43	141.6	71.04	93.5
67213	'Cmtm6'	4.69	0.75	9.36	7.97	8.94	12.52	7	5.7	6.58	1.93
67216	'Mboat2'	16.23	25.55	18.5	11.9	4.62	10.46	14.9	14.19	7.45	9.46
67217	'L3hypdh'	0.03	8.59	1.22	2.73	6.08	2.61	2.17	7.52	7.08	9.89
67219	'Med18'	2.49	5.26	1.97	0.14	2.49	0	0	2.1	9.57	2
67220	'Plekho1'	11.05	0	0.05	0	8.58	2.63	7.62	0	9.28	4.84
67222	'Srfbp1'	13.6	6.38	8.54	12.5	4.78	1.82	13.8	21.78	5.13	14.55
67223	'Rrp15'	20.31	2.59	8.52	1.21	13	5.14	0.04	8.33	17.35	7.89
67224	'Med29'	36.49	27.13	28.2	18.5	16.4	34.2	40.6	27.43	27.48	32.99
67225	'Rnpc3'	24.91	20.59	26.6	36.5	26.7	19.24	23.6	19.35	31.35	21.23
67226	'Tmem19'	24.13	18.46	13	3.28	16.1	2.21	13.1	16.28	11.82	11.9
67228	'Dph7'	26.48	11.1	27.6	17	30.1	36.33	18.1	33.72	13.54	26.88
67229	'Prpf18'	27.45	20.95	24.7	18.5	20.7	11.63	11.2	15.94	17.94	20.4
67230	'Zfp329'	6.82	4.23	3.05	1.17	7.52	2.54	2.75	2.79	4.23	4.8
67231	'Tbc1d20'	5.51	7.72	4.35	15.4	9.94	0.01	0.03	8.79	5.09	6.58
67235	'Zkscan14'	3.71	10.03	12.9	21.5	4.23	7.97	9.9	18.85	5.25	11.53
67236	'Cinp'	39.4	25.73	14.9	39.3	29.2	37.48	47.5	20.53	13.47	25.81
67238	'Fam220a'	4.26	2.68	1.64	22.6	5.3	6.86	0.45	5.32	4.03	5.88
67239	'Rpf2'	10.72	22.33	26.4	37.6	24.9	53.28	22.5	37.06	33.92	30.73
67241	'Smc6'	15.26	15.4	13.4	7.48	18.3	7.49	9.05	18.96	7.95	17.73
67242	'Gemin6'	4.15	12.9	14.1	13	29.3	16.94	29.5	10.49	22.85	16.4
67245	'Peli1'	10.82	9.87	15.2	20.2	4.31	1.72	9.25	5.04	9.25	1.8
67246	'Resf1'	8.9	4.35	1.58	1.51	5.9	3.55	3.43	3.59	2.29	2.01
67247	'Marc2'	46.56	42.33	50.7	64.9	15	105.3	46.4	39.08	34.23	32.76
67248	'Rpl39'	66.07	126	158	104	73.3	166.9	71.8	44.4	105	50.41
67249	'Tbc1d19'	24.11	25.44	33.2	24.9	17.1	37.1	19.2	10.28	24.48	22.91
672511	'Rnf213'	0.22	0.02	0.09	1.33	0.54	0	1.39	0	0.46	0.13
67252	'Cap2'	40.59	38.33	43.5	28	36.8	29.19	19	37.5	52.49	29.57
67254	'2900011O08Rik'	157.1	172.5	279	179	257	337.8	238	228.5	177.4	224.43
67255	'Zfp422'	15.95	18.75	13.3	15.5	14.6	24.94	23.4	20.82	14.27	11.02
67260	'Cers4'	48.48	47.6	63.4	37.6	51.9	60.12	58.3	58.43	85.84	58.9
67263	'Zswim6'	0.01	1.05	0.86	5.53	0.05	0	2.38	0.89	0	0.01
67264	'Ndufb8'	729.5	657.8	871	1176	1001	1168	932	985.8	970.5	935.79

67266	'Fam69a'	25.15	10.31	28.9	5.44	15	5.5	6.79	21.46	13.47	17.88
67267	'Uqcc2'	318.6	282.3	338	299	281	374.6	367	276.2	391	318.54
67268	'Myl12a'	18.51	23.85	12.2	20.3	36.9	20.55	14.7	20.9	4.86	11.47
672682	'Gm9573'	0	0	0	0	0	0.01	0	0	0	0
67269	'Agtpbp1'	10.61	10.82	8.03	17.7	2.4	1.35	3.62	5.76	3.27	7.55
67270	'Mrpl42'	180	152.1	272	200	188	383.1	259	190.6	318.9	211.92
67272	'Cmtm5'	51.39	60.14	17.2	21.5	70.8	46.95	35.3	31.98	16.21	5.18
67273	'Ndufa10'	196.2	169.6	222	119	178	231.4	167	231.7	186.9	187.85
67276	'Eri1'	0.39	2.45	1.05	1.4	0.41	3.17	0.01	0.42	1.91	1.82
67278	'Pagr1a'	45.86	61.33	45	47.6	18.3	39.69	15.2	54.92	43.76	67.91
67279	'Med31'	46	54.58	45.2	45.7	51.7	59.14	24.4	26.67	56.13	55.05
67281	'Rpl37'	73.62	86.33	105	125	50.6	92.94	78.5	34.09	54.8	43.3
67282	'Washc3'	27.55	22.75	24.9	44.5	24.9	33.58	23.5	33.27	39.79	28.08
67283	'Slc25a19'	27.64	31.51	18.8	68.7	21.8	23.49	24.1	39.03	23.86	22.58
67285	'Cwc27'	25.48	9.48	17.3	50.8	26.9	25.86	15.6	19.45	23.51	20
67286	'Ift22'	206.6	155.7	129	169	210	312.2	200	159.5	111	218.13
67287	'Parp6'	16.56	19.18	37.2	15.3	19.7	6.87	16.8	18.79	38.59	28.21
67288	'Srek1ip1'	7.85	15.47	5.84	0	6.73	3.99	13.5	9.32	11.95	15.71
67290	'3110040N11Rik'	32.37	25.16	36	23.5	30.7	31.99	17.4	38.73	15.51	23.78
67291	'Ccdc137'	9.49	30.33	17.9	15.7	20.6	28.32	2.27	10.45	27.68	12.53
67292	'Pigc'	4.94	6.7	1.25	0	10	5.09	6.16	2.37	2.53	1.74
67295	'Rab3c'	129.8	130.6	57	24.7	65	49.52	37.9	96.09	75.32	92.46
67296	'Socs4'	0	0	0	0	1.87	0	0	0.39	0	0
67298	'Gprasp1'	169.6	135	197	220	92.9	55.77	164	123.7	205.7	213.04
67299	'Dock7'	3.31	2.27	3.5	3.03	1.77	1.22	4.76	1.41	2.4	4.06
67300	'Cltc'	29.13	18.39	69.1	37.9	29.7	16.29	30.1	20.71	40.21	44.13
67302	'Zc3h13'	14.03	15.74	9.59	25.2	18.9	9.04	16.9	14.51	11.71	16.59
67305	'Gpx7'	3.31	14.13	6.85	14.4	3.69	8.42	10.7	9.71	8.41	16.32
67306	'Zc2hc1a'	36.89	25.74	28.3	15.1	40.4	29.23	23.1	32.38	40.36	30.49
67307	'Pbld2'	0.02	0	3.46	0	0	3.74	0	0.52	0	0
67308	'Mrpl46'	75.44	53.95	74.3	39	90.6	89.32	76.1	84.57	49.91	70.73
673094	'Cd99'	0.31	0	0	0	0	0	0.33	0	0.3	0
67311	'Nanp'	29.86	38.01	32	0.13	35.7	30.06	10.9	50.71	20.5	27.18
67313	'Inava'	0	0	1.27	0.07	0	0	7.1	0	0	0
67317	'1700022I11Rik'	0	0	0.18	0	0	0	3.59	0	0	0
67323	'1700042G07Rik'	0	0	0	0	0	0	0	0	0	0.26
67326	'1700037H04Rik'	259.7	187.9	227	384	209	209.8	175	214.6	282.6	240.07
67331	'Atp8b3'	1.73	0	0.03	0	2.15	0	0	0	1.14	1.87
67332	'Snrpd3'	205.6	188.2	189	36.6	209	265.6	137	241	256	247.71
67333	'Stk35'	2.47	1.42	0.81	0.66	1.89	0.76	0	0.07	0.17	0.91
67337	'Cstf1'	15.7	14.22	9.91	0	11.9	16.09	17.4	10.69	20.49	18.95
67338	'Rffl'	4.03	4.76	6.11	0	2.77	0.03	0	1.52	3.36	2.43
67341	'Ascl4'	0	0	0	0	0	0.28	4.44	0	0	0
67343	'Tex43'	0	0	0	9.7	0	0	0	0	0	0
67344	'Tctex1d1'	1.72	0	0.73	12.1	0	0	0	1.93	0	0
67345	'Herc4'	2.7	2.29	6.08	16.6	7.33	1.08	3.95	1.13	3.57	6.78
67356	'Tmco5'	0	0	0.62	0	0	0	4.83	0	0	1.81
67358	'1700093K21Rik'	0	0	1.24	0	0	0	0.57	0	0	0
67367	'Paxbp1'	6	2.56	9.3	10.2	6.25	4.65	6.98	7.23	12.31	8.15
67369	'Qpctl'	26.91	34.41	20.7	13	25.5	12.69	19.1	30.23	18.76	22.46
67370	'Zfp606'	1.14	3.68	4.5	6.05	4.99	4.82	0.04	7.12	7.59	9.31
67371	'Gtf3c6'	33.31	36.9	33.9	16.1	54.7	51.21	30.2	32.69	53.17	18.3
67374	'Jam2'	12.93	5.16	7.4	1.03	0.14	3.16	10.2	7.03	0	0
67375	'Qprt'	0	5.3	0	0	0	0	0	0	0	0
67378	'Bbs2'	14.53	13.99	11.6	8.27	5.18	9.09	7.49	16.05	18.56	25.37
67379	'Dedd2'	12.97	6.46	0.08	13.9	11.1	10.81	10.4	16.93	3.4	6.1
67381	'Med4'	55.71	26.67	24.5	32.8	16	48.01	39.7	46.45	11.77	18.26
67382	'Brd3'	29.91	23.69	25.2	38.1	26.4	20.52	20.2	19.93	19.46	19.59

67383	'Carnmt1'	0.04	1.06	0.29	3.08	1.49	0.7	0.77	1.26	0.72	0
67384	'Bag4'	9.45	5.7	7.41	8.04	1.22	3.47	6.74	6.52	7.4	4.09
67387	'Unc50'	99.58	91.16	61.8	42.1	72	108.2	112	134.3	67.39	82
67388	'Rab5if'	9.99	8.73	4.77	48.7	9.33	0.05	15.1	15.59	0.89	8.53
67389	'C1qtnf12'	3.44	5.78	4.04	0.66	14.5	4.91	0.19	14.49	10.81	0
67390	'Mrm3'	29.03	17.94	23.2	37.2	22.3	38.35	53.1	31.95	37.99	30.73
67391	'Fundc2'	9.51	18.52	15.9	21.8	10.3	8.61	17.2	8.96	2.87	10.48
67392	'4833420G17Rik'	10.84	8.63	14.4	26.6	10.9	9.88	29.5	22.05	11.11	4.3
67393	'Cxxc5'	8.35	7.59	1.72	19.5	20.3	10.15	7.52	10.87	16.23	21.04
67397	'Erp29'	196.5	286.9	185	185	180	250.7	143	140.3	246	294.88
67398	'Srpr'	57.88	69.93	55.4	33.7	52.2	65.14	51.9	47.97	76.33	79.89
67399	'Pdlim7'	96.92	156.2	80	145	216	145.8	131	184.2	104.6	156.7
67405	'Nts'	576.8	349.5	171	207	0.3	0.28	18.1	1960	93.77	0.37
67412	'Soga3'	49.73	47.18	43.6	39	54.5	54.31	49	33.47	48.36	58.72
67414	'Mfn1'	2.63	7.9	3.68	7.95	3.97	13.13	4.24	5.67	0.9	5.25
67416	'Armcx2'	38.8	47.03	58.8	26	47.7	20.64	84.1	33.99	39.86	33.84
67417	'Ears2'	2.47	3.81	2.64	4.45	0.33	3.55	0.36	1.74	4.34	1.3
67418	'Ppil4'	28.22	25.28	20.1	58.1	31	43.44	38	32.27	35.21	54.2
67419	'Armh4'	7.94	3.26	16.2	5.44	50.1	30.37	63.4	27.67	22.94	25.79
67420	'Far1'	21.38	16.37	21	20.8	9.79	10.92	12.4	17.05	19.01	18.36
67422	'Dhdds'	26.13	28.78	18	16.5	9.14	28.37	20.7	20.39	22.89	26.03
67425	'Eps8l1'	5.6	10.31	2.56	1.71	5.19	4.48	5.98	0	3.87	4.66
67426	'Coq8a'	22.86	6.78	21.5	23.9	29.1	53.39	26.6	19.19	13.65	25.44
67427	'Rps20'	13.58	20.45	20.2	20.2	15.3	21.75	11.7	8.41	11.9	13.5
67429	'Nudcd1'	11.51	10.04	3.35	2.27	11.5	6.97	15.3	8.13	14.96	5.28
67430	'4921536K21Rik'	0	0	0	6.07	0	0.42	0	0.36	0.21	0.05
67432	'Hoga1'	1.58	1.22	1.25	32.5	0.03	5.36	8	0.02	0	0
67433	'Ccdc127'	25.88	19.15	12.9	16.4	17.7	20.94	11.6	10.93	24.64	16.79
67434	'Ankrd33b'	0	0	0.01	0	0	0.88	4.25	0	0	0
67437	'Ssr3'	65.18	90.43	49.4	42.5	45.6	29.89	43.5	54.74	53.26	56.4
67439	'Xab2'	27.84	35.83	41.5	9.77	24.6	31.25	20	40.93	28.82	18.77
67440	'Mtpap'	12.14	17.11	9.03	11.4	3.93	15.4	14.9	16.15	12.57	18.83
67441	'Isoc2b'	3.68	1.63	0	0	9.53	12.4	2.12	4.44	3.67	3.98
67442	'Retsat'	5.88	8.75	8.69	6.43	0	0	0	2.76	0.02	3.24
67443	'Map1lc3b'	283.1	304	253	282	277	404.4	264	315.4	302.6	269.1
67444	'Ilkap'	56.24	73.27	19.7	38.7	71.6	24.61	67.3	40.96	53.6	26.54
67445	'C1qtnf4'	18.31	28.74	33.4	40.9	23.6	40.18	37.5	17.18	31.09	34.46
67446	'Dusp28'	37.76	27.38	48.9	29	16.7	30.55	26.8	24.64	26.06	24.24
67448	'Plxdc2'	8.44	6.94	6.37	1.99	10.9	8.6	3.48	5.19	3.98	3.13
67451	'Pkp2'	0.03	2.95	3.91	0.64	2.89	0	0.02	1.54	0.03	1.36
67452	'Pnpla8'	20.26	24.34	23.7	18	10.1	8.94	14.9	11.35	31.44	22.17
67453	'Slc25a46'	50.31	50.43	27.9	27.9	33.2	24.43	43.7	44.98	50.71	50.93
67454	'Ikbip'	7.02	20.54	6.93	7.95	10.1	23.48	11.6	20.66	27.01	15.48
67455	'Klhl13'	88.31	25.32	72.8	62.5	31.6	31.22	10.2	49.63	36.23	15.01
67456	'Ergic2'	44.45	44.86	28.3	23.4	27.5	20.49	41.6	46.48	32.98	34.99
67457	'Frmd8'	3	3.16	7.34	0	3.14	14.17	0.47	5.1	2.24	2.62
67458	'Ergic1'	124.7	84.95	83.2	43.5	58.3	47.26	46.8	92.76	106.8	98.75
67459	'Nvl'	18.27	10.4	11.4	20.1	21.6	10.53	8.31	15.41	13.06	14.69
67460	'Decr1'	7.18	2.64	5.41	3.72	8.3	2.37	4.22	8.03	7.21	0.26
67463	'Poc5'	6.45	6.85	4.92	6.58	14	14.21	20.8	21.79	4.54	9.83
67464	'Entpd4'	9.58	16.09	35	10	14	17.89	29.1	14.03	22	3.93
67465	'Sf3a1'	18.26	19.46	12.3	12.5	7.57	10.34	13.6	13.53	24.18	24.58
67466	'Pdcl'	18.81	4.33	6.59	5.12	7.13	6.8	10.8	1.67	9.22	8.54
67467	'Gpalpp1'	3.2	7.03	7.31	9.34	6.11	0.33	0.12	10.33	7.58	5.03
67468	'Mmd'	23.09	31.66	20.7	49.1	13.7	10.32	18.7	32.73	15.41	17.83
67469	'Abhd5'	0	3.16	2.67	0	8.78	0.31	2.89	2.95	5.81	1.73
67471	'Gpatch1'	11.46	22.18	17.3	7.84	22	14.6	9.21	23.3	14.48	13
67472	'Mtfr1'	10.49	9.36	5.87	5.3	9.66	6.82	2.41	2.21	5.5	6.77

67473	'Slc47a1'	0.02	0	0	0	0	0	0	0	0	0.02
67474	'Snap29'	5	3.73	4.39	0	4.92	1.11	0	6.44	3.28	7.16
67475	'Ero1lb'	9.75	14.06	16.2	16.7	11.2	0.34	24.7	2.31	16.3	8.68
67477	'Abhd15'	0	0	0	0	0	2.22	0	1.89	0.01	0
67480	'Cwc25'	4.75	14.01	7	9.8	6.28	4.53	6.13	3.1	10.76	5.76
67483	'1700028P14Rik'	0	0	0	4.76	0.89	7.04	0	0	0	3.36
67484	'Eepd1'	4.82	13.4	5.86	0	6.79	1.07	8.12	6.24	4	3.69
674842	'Gm9631'	0.22	0	0.12	0.71	0.35	1.09	0	0.21	0.84	0.49
67486	'Polr3g'	0.8	0	0.84	6.85	0.69	2.27	0	2.48	3.38	3.11
67487	'Dhx40'	8.14	10.78	9.18	5.02	3.61	0	0.03	5.85	8.33	9.13
67488	'Calcoco1'	19.07	12.83	20.4	30.6	10.9	10.17	19.9	21.12	24.88	21.25
67489	'Ap4b1'	6.28	22.28	10	6.32	12.1	4.11	0.5	8.19	7.11	12.76
674895	'Nek10'	1.15	2.77	3.66	4.66	0.51	0.98	0.39	1.53	0.01	3.57
67490	'Ufl1'	19.27	16.9	19	25.8	20.8	13.89	10.9	20.55	20.82	25.2
67492	'Zfand4'	3.75	1.89	0.61	10.7	0	2.28	1.38	3.92	2.45	2.11
67493	'Mettl16'	34.64	20.38	22.8	15.7	20.8	23.24	20.2	29.04	31.35	34.53
67495	'Tmem167b'	14.81	19.56	19.9	41.8	16.4	28.19	28.1	26.63	16.83	13.29
67498	'Kcnv1'	1.53	4.25	0.95	0.01	0.03	0.02	0.01	0.01	0.01	0.02
67500	'Ccar1'	29.21	24.14	23.4	16.9	9.27	11.29	21.5	13.59	16.89	14.8
67501	'Ccdc50'	4.68	3.85	4.81	1.78	5.7	2.45	5.5	5.87	2.4	2.17
67504	'Rnf151'	0	0	0	0	0	0	2.5	0	0	0
67509	'Saysd1'	12.84	18.5	4.54	7.22	16.1	24.37	28.4	16.53	37.8	21.93
67510	'Tvp23b'	53.82	36.99	42.2	24.4	47.4	28.74	23.5	17.99	74.49	67.16
67511	'Tmed9'	187.5	151.8	136	179	204	252.4	200	217.4	248.4	253.97
67512	'Agpat2'	0	0	1.2	0	5.57	0	0	0	1.13	0.96
67513	'Faap20'	43.99	46.47	33.6	112	38.8	61.71	20.9	40.61	32.8	46.07
67515	'Ttc33'	85.53	88.87	60.5	69.6	68.8	73.12	42.1	64.86	103.5	45.04
67516	'Kctd4'	6.55	1.7	3.3	4.58	5.5	5.6	8.75	1.37	0.34	1.05
67525	'Trim62'	1.78	0.13	1.62	13.3	3.98	0.17	3.7	5.56	0	0
67526	'Atg12'	38.49	47.61	24.7	49.6	27.1	63.03	29.3	36.99	26.08	21.38
67528	'Nudt7'	14.98	2.88	9.96	19.9	19.3	9.09	0.06	9.65	9.53	16.8
67529	'Fgfr1op2'	44.82	49.79	30.6	18.7	48.2	31.82	29.7	38.89	85.8	54.07
67530	'Uqcrb'	396.5	398.4	507	502	337	479.8	379	353.6	507.5	392.45
67532	'Mfap1a'	29.01	26	23.3	28.4	21.7	21.02	16.7	24.86	23	18.08
67533	'Ppfibp1'	1.73	5.26	2.1	1.94	0.96	0.02	11	0.49	2.74	3.96
67534	'Ttll4'	0.2	0	0.01	3.19	0	0.1	0	0.14	1	0.56
67538	'Zswim3'	4.19	1.81	6.64	0	0.29	5.62	5.93	11.7	3.39	3.55
67542	'Cog6'	19.75	27.46	30.9	14.8	10.3	12.25	19.4	24.78	27.78	23.56
67543	'Pabpc6'	0	0	0	0	0	0	0	0	0	0.01
67544	'Fam120b'	35.26	34.2	32	17.6	40.9	16.56	30.5	27.4	30.77	34.69
67547	'Slc39a8'	0	0	0	0	0.76	0	0.56	1.9	0.82	1
67549	'Gpr89'	20.35	29.62	24.5	8.68	13.2	9.98	18	17.7	16.88	16.46
67553	'Gstcd'	2.06	0.51	0.21	0	1.75	0.27	2.81	2.47	1.39	0
67554	'Slc25a30'	7.81	4.34	3.56	0	1.08	0	0	1.92	0	0.04
67556	'Pigm'	0.01	0.72	0.5	7.24	0.09	0.04	0.1	0.02	0.89	0.46
67557	'Larp6'	3.1	4.77	5.76	13.4	2.04	4.04	0.28	10.04	4.35	8.76
67561	'Wdr48'	18.09	23.63	19.7	8.3	18.8	12.89	12.8	24.36	21.21	28.94
67563	'Narfl'	31.8	20.65	16.4	17.3	22.6	24.79	18.3	28.23	25.77	14.5
67564	'Tmem35a'	151.2	97.57	25.3	21.8	22.8	34.74	74.1	78.99	10.9	3.21
67568	'Mrfap1'	632	588.9	422	704	437	652.3	600	444.5	454.2	335.56
67569	'Mgat4c'	3.25	13.54	14.8	3.31	3.2	1.74	10.2	11.52	1.42	1.93
67574	'Alg13'	1.22	1.06	6.13	0	0.27	0	1.63	1.9	10.48	2.26
67578	'Patl2'	0	0	0	0	0	0	0	0	0.19	0
67579	'Cpeb4'	1.25	4.7	2.15	11.8	3.69	2.51	2.92	1.85	4.22	5.09
67581	'Tbc1d23'	11.7	8.29	8.81	11.4	11.9	14.78	6.05	7.55	11	4.69
675812	'Zfp605'	1.87	6.11	3.61	2.05	5.15	2.86	3.45	7.16	8.01	3.48
67582	'Slc25a26'	3.21	7.52	9.73	9.22	7.66	6.61	12.6	12.51	6.03	11.56
67586	'Ubxn11'	14.31	6.05	2.27	0	42.2	16.91	25.6	10.52	4.36	23.26

67588	'Rnf41'	48.3	38.88	42.7	25.6	58.7	35.22	53	51.72	69.23	47.04
67590	'Tctn3'	11.51	16.37	14	10	18.4	11.73	20.5	17.77	18.06	16.08
67592	'4930524B15Rik'	0	0	1.87	0	0	0	0	0	1.82	0
67593	'4930519G04Rik'	0	0.07	0.17	0.18	0.08	0.05	0.05	0.1	0.04	0.03
67602	'Necap1'	182	162.6	207	179	143	124.7	276	230.4	182.8	187.49
67603	'Dusp6'	16.52	23.06	27.9	44.5	0.07	16.49	21.2	19.08	4.89	10.28
67604	'Get4'	59.6	39.35	73.6	72.8	42.2	55.63	65.8	22.71	69.53	6.88
67605	'Akt1s1'	29.06	35.38	20.6	33.2	27.6	29.21	30.1	26.29	30.07	23.06
67606	'Fibin'	4.11	8.53	0.02	0	0	0	4.09	4.75	6.3	0
67607	'Zfp788'	7.33	6.72	6.36	6.32	5.01	3.71	0.04	13.35	7.03	10.99
67608	'Narf'	16.49	5.3	10.3	0.86	19.3	13.7	15.1	10.36	11.52	9.69
67609	'4930453N24Rik'	22.67	6.33	6.74	15.9	14.9	21.15	0.5	27.51	0.66	23.31
67610	'Rspry1'	9.9	16.36	13.6	15.6	18.7	1.99	4.58	13.88	25.95	11.94
67615	'Ube2r2'	15.15	18.07	9.4	15.2	8.54	14.96	16.6	13.1	13.57	14.06
67618	'Aasdhppt'	26.02	22.18	19.9	41	14.8	23.76	12.2	37.36	12.42	23.18
67619	'Nob1'	43.61	45.43	52.9	44.5	32.5	39.41	49.5	44.26	26	37.98
67620	'Lrp2bp'	0	0.67	0	0.09	1.5	0	0.03	0	0.06	0
67621	'Bend5'	13.55	3.56	13.1	2.34	5.29	5.25	12.7	11.07	4.65	6.53
67622	'Mxra7'	5.32	2.99	0.74	15.7	4.6	5.41	7.19	1.74	4.18	4.18
67623	'Tm7sf3'	12.66	11.02	13.5	0.02	5.31	3.92	22	11.24	18.38	12.5
67628	'Anp32b'	17.05	17.26	17.1	55.1	21.9	10.89	15.1	15.01	19.45	6.2
67629	'Spc24'	8.47	9.92	14.3	29.2	26.9	19.36	9.53	13.48	11.41	11.32
67630	'Samd8'	3.02	4.45	2.58	3.27	2.47	2.2	7.78	1.75	1.31	2.32
67636	'Etfrf1'	33.83	26.17	56.5	44.7	49.2	11.59	48.6	36.05	52.22	42.25
67647	'4930523C07Rik'	0.06	2.59	0	2.44	0	1.66	0	0	0	0
67655	'Ctdp1'	2.05	0.34	0.42	0	4.02	3.72	4.35	2.14	4.86	0.46
67656	'4930548H24Rik'	0	0	0	0	0	0	0	0.77	0	0
67657	'Rabl3'	11.3	11.33	2.62	0	5.12	18.45	5.95	20.87	3.95	11.44
67661	'Ift172'	3.76	6.77	10.4	0.6	8.62	2.72	10.4	2.59	8.54	8.46
67664	'Rnf125'	0.39	0.32	0.35	0.3	2.15	0.18	0.15	0.38	0.73	0.35
67665	'Dctn4'	44.45	38.04	41.6	50.6	35.7	23.56	39.6	29.65	40.54	43.06
67666	'Hapln3'	0	0	0	0	0	0	5.71	0	0	0
67667	'Alkbh8'	10.16	10.77	15.3	4.54	6.98	7.59	5.04	20.64	16.33	4.7
67669	'Hikeshi'	76.51	82.83	72.3	90.5	102	114.2	77.7	84.67	81.03	95.55
67671	'Rpl38'	204.8	291.5	297	464	178	460.3	241	106.9	344.6	155.79
67673	'Elob'	637.7	697.4	733	889	650	1080	751	569.2	787.1	641.5
67674	'Trmt112'	141.7	121.3	137	94.6	108	211.6	117	109.8	124.1	132.95
67675	'Cuta'	99.92	85.65	112	71.7	96.1	132.8	74.3	135.7	116.7	141.05
67676	'Rpp21'	117.4	83.92	89.7	94.5	116	273	87.7	118.1	85.02	105.66
67678	'Lsm3'	42.71	41.69	82.7	82.3	61.6	86.71	67.7	58.58	53.3	79.16
67680	'Sdhb'	466.5	459.5	485	500	472	521.9	403	549.8	451.9	511.38
67681	'Mrpl18'	71.82	64.62	86.8	108	117	77.14	108	82.28	81.85	61.65
67683	'Pbdc1'	28.83	14.81	21.2	44.3	37.6	36.05	45.5	36.19	26.74	43.2
67684	'Luc7l3'	36.88	46.8	23	25.3	58.1	38.76	61	43.95	49.19	40.69
67685	'Dyx1c1'	5.12	3.12	3.78	0	11.1	0.02	1.17	4.76	0.87	2.18
67687	'1700011L22Rik'	0	0	0	0	2.67	0	0	0.07	0	0
67689	'Aldh3b1'	0	0	0	0	1.86	0	0	1.61	0	0
67690	'Prss37'	0.35	0	0	0	0	0	0	0	0	0
67693	'Hypk'	206.1	167.5	186	299	238	342.9	148	230.3	323.9	264.54
67694	'Ift74'	25.49	15.48	14.1	21.9	12.7	27.54	9.2	17.58	19.65	23.41
67695	'Ost4'	197.2	148.7	186	186	173	259.8	188	137.7	187.4	154.82
67698	'Fam174a'	22.97	36.32	39.1	55.4	19.6	19.47	34	17.4	12.31	9.58
67702	'Rnf149'	14.26	6.11	4.47	10.2	6.74	2.58	5.47	8.72	1.11	3.88
67703	'Kirrel3'	9.29	2.38	6.28	4.49	13.1	15.18	24.8	12.16	12.84	25.59
67704	'1810037I17Rik'	70.08	71.63	89.4	90.3	102	125.7	103	58.28	101.1	128.65
67706	'Tmem179b'	4.05	24.03	15.8	13.4	21.1	28.63	16.1	14.37	18.37	5.25
67707	'Mrpl24'	54.42	62.1	17.5	67.8	43.6	49.94	75	33.74	30.71	46.41
67708	'Pcnx4'	23.67	31.69	22.9	17.9	18.6	7.57	25.2	21.77	18.25	19.8

67710	'Polr2g'	171.6	195	196	279	212	228.3	145	184.4	155.9	195.77
67711	'Nsmce1'	57.61	73.56	43.6	76.2	64	46.35	49.5	59.08	39.68	59.27
67712	'Slc25a37'	0.71	0.1	0.79	1.87	1.09	0	0.42	1.22	0.01	0.22
67713	'Dnajc19'	115.8	67.76	69.9	113	103	123.3	62.7	96.95	125.2	78.53
67722	'Actl11'	0	0	0	0	0	0	0	0	0.01	0
67724	'Pop1'	6.96	3.03	0	0.02	3.16	1.32	4.9	2.18	2.17	1.57
67725	'Nudt13'	7.17	5.81	9.75	17.3	8.01	0.02	0.02	4.18	5.69	3.75
67726	'Fam114a2'	15.97	22.49	31.1	27.3	35.1	40.17	20.1	31.4	16.92	23.12
67727	'Stx17'	1.48	1.63	2.85	2.33	0.46	1.14	3.13	0.21	1.59	1.09
67728	'Dph2'	20.09	6.02	8.65	16.6	13.4	10.83	5.48	10.73	15.98	14.05
67729	'Mansc1'	0	0.08	0.18	0	0.06	0	0	0	2.37	1.45
677296	'Fcrl6'	0	0	0	0	0	0	0	1.59	0.98	0
67731	'Fbxo32'	1.01	2.72	0.06	0.13	4.85	0.07	0.05	0.5	0.48	0.61
67732	'Iah1'	11.86	18.15	32.8	41.8	23.4	42.6	33.6	39.1	13.97	17.56
67733	'Itgb3bp'	4.37	5.4	1.18	3.18	1.8	3.86	1.73	2.03	1.74	9.58
67736	'Ccdc130'	20.84	14.86	19.6	14.4	16.1	18.84	3.56	9.51	20.48	6.51
67738	'Ppid'	134.6	121.9	97.1	62.6	90.8	134.5	127	105.4	131.1	112.43
67739	'Slc48a1'	65.64	46.94	44.5	67.7	98.5	98.48	50.5	50.32	69.17	55.65
67742	'Samsn1'	0	0.18	0	0	0	2.34	0	0	0	0
67747	'Ribc2'	0	0	0.97	0	3.68	2.19	0	0	0	5.58
67749	'Mgarp'	0	0	0	0.05	6.58	0	0	0	0	0
67752	'Ppp1r32'	15.03	26.03	4.65	0	7.37	23.44	0.03	9.63	14.52	24.87
67753	'Eqtn'	0.4	0	0	0	0	0	0	0	0	0
67755	'Ddx47'	49.07	47.21	53.7	72.4	60.3	53.2	51.3	59.45	27.43	50.41
67759	'Plgrkt'	10.79	43.49	25.6	4.35	23.2	10.53	7.16	13.19	1.92	25.27
67760	'Slc38a2'	11.63	16.79	13.8	8.5	6.3	6.33	11.3	14.56	14.88	12.56
67763	'Prpsap1'	74.5	72	39.1	61.4	42.5	88.15	80.8	61.26	90.33	97.81
67767	'Jagn1'	109.3	86.68	82.4	51.7	78.6	116.5	66.6	147.4	76.62	93.14
67768	'N6amt1'	26.84	32.12	37.9	22.5	30.4	28.89	13.5	38.41	31.06	33.51
67769	'Gpatch2'	4.1	13.59	5.92	2.43	7.88	6.7	3.63	5.88	9.29	3.7
67770	'Caap1'	4.06	2.9	1.86	6.79	0.23	0.83	1.28	5.29	5.16	3.2
67771	'Arpc5'	152.3	152.3	48.5	176	136	146.1	82.9	111.4	141.3	144.01
67772	'Chd8'	3.86	2.59	4.76	2.32	6.29	6.32	5.34	5.79	6.06	5.72
67773	'Kat8'	39.36	17.1	22.3	16.7	15.8	12.39	17.4	13.12	23.44	38.9
67774	'Borcs5'	30.75	30.49	32.5	13.7	27	45.44	41.9	38.83	40.06	35.17
67775	'Rtp4'	10.18	0.62	14.7	14.8	0.19	7.16	5.24	1.75	3.16	2.11
67776	'Vwa5a'	1.35	0	6.51	0	0.01	5.27	0	1.95	2.98	13.35
67778	'Zfp639'	17.51	16.2	14.7	23.6	12.6	14.51	19.4	9.51	1.01	0.56
67781	'Ilf2'	89.85	71.22	79.8	65.9	49.2	75.35	119	92.53	62.08	71.57
67784	'Plxnd1'	0	0	1.11	0.17	0	0	0.89	0	0	0
67785	'Zmym4'	2.94	1.94	4.42	2.15	1.46	1.78	0.16	2.03	1.66	2.4
67788	'Sfr1'	146.1	160.9	140	171	160	162.1	149	136	145.7	125.34
677884	'Pakap'	3.43	1.18	4.51	2.99	3.31	3.23	0	7.03	1.86	4.21
67789	'Dalrd3'	75.6	80.73	54.4	10.1	66.2	77.42	95.9	57.36	66.38	64.64
67790	'Rab39b'	11.36	10.3	21.5	3.87	0.47	5.94	12.1	9.71	14.08	11.2
67792	'Rgs8'	1.11	0.53	5.45	0	2.84	1.67	13.2	3.82	0.01	0.05
67795	'Rnls'	2.83	1.98	1.35	3.82	3.88	3	2.97	1.48	6.54	4.14
67797	'Snrnp48'	16.44	21.23	13.3	18.6	6.04	20.47	7.07	9.87	26.29	15.32
67800	'Dgat2'	28.48	26.56	25.8	45.7	41.3	41.77	22	35.65	26.96	39.12
67801	'Pllp'	7.61	18.86	77.4	33	14.6	52.45	51.4	16.46	0.02	0.05
67803	'Limd2'	37.4	36.64	32	27.7	37.7	48.08	19	24.16	54.77	26.35
67804	'Snx2'	69.74	84.31	59.7	67.8	49.6	61.79	51.7	74.8	67.97	75.41
67808	'Tprgl'	28.77	26.84	37	75	40.4	56.39	35.2	22.54	24.5	18.99
67809	'Rmdn3'	18.84	51.02	57.2	73.1	43.3	48.49	52.3	52.26	65.82	60.24
67811	'Poldip2'	66.94	53.36	116	79.3	37.7	79.83	77.8	63.2	46.74	48.92
67812	'Ubxn4'	30.68	36.41	22.3	33.4	29.5	24.18	20.2	18.46	38.17	21.62
67815	'Sec14l2'	0	0.04	1.83	0	0.12	0	4.49	0	0	0.03
67819	'Derl1'	63.72	78.57	41.3	49.7	62.7	49.25	48.6	53.72	53.49	99.45

67824	'Nmral1'	10.99	21.5	6.49	1.57	9.69	0	9.07	28.49	19.87	8.53
67826	'Snap47'	738.5	751.8	738	624	560	678.6	715	801.4	573.7	558.46
67830	'Rer1'	76.84	81.67	65.5	86.5	77.3	143.8	151	107.3	77.44	91.37
67832	'Brix1'	8.19	15.92	15.4	26	16.7	24.9	11	19.78	13.95	15.86
67834	'Idh3a'	157.3	172.2	206	106	169	159	193	209.5	169.9	160.77
67836	'Wdr83'	59.07	16.61	26.6	44.9	40.1	45.31	70.1	54.35	41.2	64.24
67838	'Dnajb11'	20.1	42.5	21.2	6.64	11.2	12.62	19.5	12.62	44.69	16.29
67839	'Gpsm1'	18.02	21.06	28.6	18.3	15.2	15.07	10.3	16.88	11.77	6.4
67840	'Mrpl57'	25.91	35.03	15.1	36.9	15.6	39.82	29.2	29.6	30.62	35.98
67841	'Atg3'	35.35	26.79	34.9	57.7	14.9	15.92	21.4	22.74	37.43	8.6
67842	'Nop9'	3.77	2.63	1.02	0	2.27	0.03	0.61	1.76	0	1.18
67843	'Slc35a4'	25.66	17.05	37.9	21.9	32.8	37.08	28.2	29.68	7.85	23.7
67844	'Rab32'	9.66	0	0	0	0	0	0	0	3.76	0
67845	'Rnf115'	15.03	9.97	9.32	49.8	12.6	10.73	3.28	19.61	18.79	7.32
67846	'Tmem39a'	14.94	2.32	4.19	6.16	3.86	1.3	10.4	5.3	5.11	9.41
67847	'Sncaip'	4.04	1.65	0.31	6.27	0.61	0.89	0.03	1.95	1.94	0.25
67848	'Ddx55'	17.61	3.17	8.75	13.6	8.63	21.62	6.82	14.52	10.02	6.54
67849	'Cdca5'	0	0	1.62	0	0	0	0	0	0	0
67851	'1700021F05Rik'	17.2	22.46	14.9	27.5	20.5	27.97	22.4	44.48	22.02	23.6
67855	'Asprv1'	0.06	0	2.03	0	0	0	7.84	0	0	1.99
67856	'Echdc3'	0	0	0.05	7.05	0	0	4.05	1.51	0	0
67857	'Ppp6c'	23.61	23.95	25.3	33.8	13.7	19.96	28.3	14.57	16.62	35.04
67859	'Cysrt1'	0	0	0	0	0	8.91	0	0	0	0
67860	'S100a16'	34.11	36.69	160	83.6	43.7	55.84	81.4	35.62	18.31	34.67
67861	'Akr1b10'	21.85	10.28	33.2	0	16.2	24.5	41.4	25.07	18.1	30.29
67862	'2310033P09Rik'	43.51	19.04	28.4	33.6	57.8	78.92	25	37.04	22	55.22
67863	'Slc25a11'	234.9	182.7	321	163	192	303.4	252	240.1	171	263.33
67864	'Yipf4'	33.23	30.46	29.1	52.9	25.7	9.39	28.6	19.23	24.37	8.11
67865	'Rgs10'	132.7	144.5	315	124	30.9	172.7	157	331.1	272	75.65
67866	'Wfdc1'	0	0.08	4.81	0	89.1	6.6	0.04	1.44	4.23	3.11
67867	'Lrrc28'	5.54	7.25	5.91	0.02	8.48	2.26	8.39	5.92	8.64	13.78
67869	'Paip2'	262.7	248.6	246	227	208	269.8	239	272.6	270.5	295.57
67870	'Enoph1'	49.03	52.81	52.1	26.1	97	60.58	55.9	40.45	34.41	38.57
67871	'Mrrf'	16.07	19.22	16	21	12.6	26.71	24.3	21.23	24.42	9.99
67872	'Nsmce4a'	2.87	8.28	8.84	27.1	11.3	18.92	4.6	8	6.73	8.51
67873	'Mri1'	9.69	7.38	13.7	19.8	3.21	11.6	12.9	12.62	6.59	8.09
67874	'Rprm'	169.6	56.15	50.2	48.3	86.8	17.89	102	42.63	4.39	9.22
67876	'Coq10b'	18.77	26.65	18	78.2	19.6	26.6	20.9	29.12	22.91	30.04
67877	'Naa20'	130.9	106.8	102	141	170	133.7	110	155.3	190.6	103.22
67878	'Tmem33'	13.43	11.66	9.98	13	8.33	4.68	4.7	9.22	11.02	7.78
67880	'Dcxr'	9.84	17.77	3.71	15.4	15.2	6.24	12.9	18.19	14.46	21.39
67881	'Mdp1'	29.76	41.47	32.3	46.6	64.9	34.64	29.5	18.84	54	41.44
67883	'Uxs1'	19.43	32.83	11.3	25.9	15.3	26.04	24.3	10.98	35.35	40.46
67884	'1810043G02Rik'	69.31	118.3	104	95.8	75.2	150.7	113	139.1	76.25	87.83
67886	'Camsap2'	3.94	2.75	6.87	7.09	3.2	2.57	1.17	5.42	4.03	4.06
67887	'Saraf'	672.2	835	501	883	528	736.9	471	535.4	608.9	783.85
67888	'Tmem100'	4.92	7.74	31.2	27.9	2.27	5.82	13	10.78	0	10.22
67889	'Rbm18'	35.05	49	41.2	60.9	44.7	42.27	30	67.95	60.48	35.68
67890	'Ufm1'	4.81	5.42	5.06	3.42	6.33	3.48	3.57	4.6	5.77	8.64
67891	'Rpl4'	255.2	379.9	287	478	308	240.1	300	220.9	209.7	276.23
67892	'Coa6'	67.66	45.82	35.1	52.8	50.5	47.48	38	65.15	47.07	39.86
67893	'Tmem86a'	7.25	9.17	17.3	8.9	7.55	43.7	25.9	19.51	21.66	15.1
67894	'Fam45a'	27.54	28.73	33.7	20.4	50.4	39.68	30.7	49.5	22.42	36.4
67895	'Ppa1'	115.3	158.8	133	155	138	170.6	159	123.8	126.9	132.27
67896	'Ccdc80'	0	1.22	1.41	0	0.01	0	0	0	0.49	0
67897	'Rnmt'	48.53	34.48	34.5	86.7	43.1	46.89	35.4	44.87	57.42	72.95
67898	'Pef1'	68.53	111.5	144	54.2	146	106.9	70.8	131.3	100.3	120.38
67899	'Cmc1'	12.69	22.66	21.3	54.5	12.3	22.19	9.37	16.59	2.85	10.9

67900	'Mtfp1'	87.66	98	85.6	44	94.2	148.6	77.4	127.9	119.7	80.07
67902	'Sumf2'	31.13	29.68	13.2	22.4	10.5	21.83	7.28	3.77	19.3	12.51
67903	'Gipc1'	32.04	53.93	30.1	35.1	39.9	66.25	43.8	42.24	31.38	51.45
67905	'Ppm1m'	12.85	31.14	16.2	15.2	17.5	16.42	17.2	7.73	22.04	14.69
67911	'Zfp169'	2.14	0.85	0.89	0.55	2.17	0	1.91	3.57	2.49	1.38
67912	'1600012H06Rik'	2.11	5.23	4.06	0.04	3.53	4.22	4.51	4.36	5.77	4.29
67914	'Coq9'	70.82	64.22	59.5	12.8	40.4	84.29	95.8	69.19	47.59	77.76
67916	'Plpp3'	38.55	56.68	23.7	15	15.9	40.02	10.1	8.38	28.67	7.95
67917	'Zcchc3'	1.51	4.05	4.25	1.84	3.68	0.61	7.2	2.99	5.64	1.09
67920	'Mak16'	22.62	33.69	20.4	15.6	23.8	20.72	5.22	24.94	5.03	25.12
67921	'Ube2f'	40.44	40.48	36.8	18.5	36.2	23.05	40.1	25.72	50.87	27.75
67922	'Fam32a'	69.84	82.57	102	93.2	57.7	127.9	121	85.55	72.58	58.34
67923	'Eloc'	135.7	127.3	83.2	143	75.6	90.86	92	84.47	99.17	86.35
67933	'Hcfc2'	2.72	4.73	16.7	3.68	9.83	3.14	2.66	7.21	7.13	8.31
67935	'Ces5a'	2.42	0	0	0	1.09	0	7.01	0	6.44	2.49
67936	'Wdr55'	12.87	8.05	9.79	23	10.2	13.78	4.81	19.31	17.65	16.29
67937	'Tmem59l'	911.2	1070	928	1048	736	1069	740	1145	1341	1289.12
67938	'Myl12b'	789.1	622.9	653	536	732	1102	762	960.2	810.2	934
67939	'Prorsd1'	4.78	5.02	5.81	3.91	5.26	11.79	11.1	3.67	12.78	5.81
67941	'Rps27l'	84.52	98.52	71.3	194	71.4	134.8	106	43.27	95.02	109.36
67942	'Atp5g2'	215.9	228.5	233	210	227	331.7	222	188	193.9	232.31
67943	'Mesd'	67.69	137.3	60.3	87.6	60.8	97.57	57.2	72.7	144.7	103.72
67945	'Rpl41'	773.5	787.7	904	1310	542	1031	756	356.8	780.6	632.86
67946	'Spata6'	2.5	3.83	3	1.54	0.89	0.6	0	3.49	0.47	0
67948	'Fbxo28'	2.21	0.52	1.73	3.06	0.57	0.96	2.48	0.44	2.65	0.7
67949	'Nifk'	37	26.55	13	28.4	30.2	37.62	30.6	31.16	30.59	36.47
67951	'Tubb6'	0.03	7.73	3.64	0.03	0	0	0	0.02	0.22	3.37
67952	'Tomm20'	72.11	86.08	109	107	85.6	77.91	102	99.99	99.13	108.19
67955	'Sugt1'	41.06	37.12	12.1	39.4	42.2	37.32	40.2	36.21	41.78	38.17
67956	'Kmt5a'	4.81	1.78	0.8	7.56	2.85	1.95	3.88	2.02	2.3	4.11
67958	'U2surp'	12.92	16.77	17.8	21.3	15.4	15.01	17.5	12.79	14.22	14.88
67959	'Puf60'	181.9	150.5	162	191	213	294.6	195	226.4	222.1	234.53
67963	'Npc2'	23.36	40.45	22.3	39.1	16.7	38.84	16.1	26.04	40.57	32.94
67966	'Zcchc10'	23.15	12.73	9.23	11.1	18.8	0.11	0.16	13.57	17.55	18.95
67967	'Pold3'	19.42	7	21.1	20.2	13.7	15.64	11.8	22.36	29.01	22.87
67968	'Ooep'	4.5	5.34	0.69	12.2	4.72	3.07	0.13	4.94	2.45	9.49
67971	'Tppp3'	489.6	391.1	397	610	492	402	259	314.9	296	367.08
67972	'Atp2b1'	26.09	58.01	26.1	24.4	45.8	37.67	23.1	41.86	42.74	25.94
67973	'Mphosph10'	5.81	8.5	15.5	8.24	11.8	15.48	9.35	9.16	14.35	11.3
67974	'Ccny'	9.39	4.42	10.9	8.02	9.06	4.06	3.91	2.36	7.26	0
67976	'Trabd'	17.95	5.82	16.3	10.7	17.9	5.72	17.9	16.21	6.1	8.39
67978	'Tctn2'	27.65	36.3	29	21.8	11	6.63	22.7	2.49	17.06	34.96
67979	'Atad1'	83.25	59.33	69.1	29.7	26.9	42.42	52.2	70.19	67.8	71.85
67980	'Gnpda2'	56.1	32.02	38.7	35	35.6	62.98	20.6	27.98	43.39	48.53
67983	'Pdzd9'	0.87	0.36	0.27	0.62	1.47	0	1.46	0.26	0.37	0.31
67988	'Tmx3'	20.57	13.15	16.1	15.5	6.9	3.75	4.81	10.41	20.88	22.37
67991	'Nacc2'	3.69	3.89	8.61	0.06	19.6	9.31	6.65	5.31	0.85	4.88
67993	'Nudt12'	10.22	0	1.33	0	10.8	2.06	6.21	0	3.6	0.47
67994	'Mrps11'	76.69	56.13	67.6	60.2	56.4	97.13	61.1	67.95	61.05	71.01
67996	'Srsf6'	45.67	48.63	34.1	39.4	29.1	35.81	38	38.98	48.47	21.75
67997	'Ddx59'	4.34	2.69	12.6	15.8	10.6	10.08	11.7	1.56	9.39	15.19
67998	'Retreg3'	7.15	14.52	3.65	5.14	12.7	6.63	7.76	12.19	24.75	6.77
68001	'Cfap298'	51.92	78.64	63.4	91.7	58.8	57.46	51.5	57.21	39.52	47.31
68002	'Sdhaf4'	128.3	133.5	110	84.6	124	190.1	46	113.5	167.2	177.97
68009	'Defa20'	0	0	0	0	0	0	0.03	0	0	0
68010	'Bambi'	0.53	0.84	1.16	6.58	1.59	0.35	1.69	0.69	2.24	1.04
68011	'Snrpg'	114.3	170.8	128	226	70.1	263.7	126	65.74	161.8	79.25
68014	'Zwilch'	0.06	0	2.57	0	0	0	3.03	0	3.19	0.02

68015	'Trap1'	57.34	49.35	56.3	64.9	32.2	55.35	53.2	49.07	39.7	25.21
68016	'Cavin4'	0	0	0	0	0	0.04	0	0	0.02	0.38
68017	'Mrm2'	11.91	24.08	18.5	25.3	14	32.97	2.01	23.95	6.98	19.62
68018	'Col4a3bp'	5.88	3.5	5.41	0.19	4.26	4.93	4.19	3.41	7.25	5.58
68020	'Apopt1'	51.72	48.83	26.4	58.8	68.2	53.56	45.5	44.04	48.92	54.32
68021	'Bphl'	39.44	23.47	32.5	24.5	30.2	63.57	62.5	53.57	57.15	56.29
68023	'Pdf'	34.34	30.48	79.4	41.1	29.4	35.06	60.6	41.95	40.51	21.37
68024	'Hist1h2bc'	46.88	50.27	22.5	43.2	22.9	20.95	47	22.44	12.59	41.06
68026	'Pclaf'	0.59	0.02	0.12	0	0	0.19	0	0	0.2	0.05
68027	'Tmem178'	1.57	7.45	10	10.7	18.2	14.85	10.4	7.93	4.46	3.04
68028	'Rpl22l1'	308.1	396.3	352	417	200	395.8	338	237.8	276	248.3
68031	'Rnf146'	26.19	25.96	10.2	8.27	21.9	25.9	22.7	30.14	26.1	25.42
68032	'Emc4'	318.3	284.3	267	314	322	407.9	206	386.8	349.8	303.57
68033	'Cox19'	70.2	47.08	50.3	39.7	39.5	73.64	28.3	57.44	74.99	48.56
68034	'Fam122a'	6.05	0.91	1.17	3.18	4.62	2.52	2.41	5.67	8.41	3.37
68035	'Rbm42'	59.92	52.12	63.8	69.3	29.1	36.64	44.7	24.63	53.28	26.44
68036	'Zfp706'	84.38	95.03	75.7	76.8	104	81.28	114	92.1	62.09	85.81
68038	'Chid1'	83.45	115.4	74.6	37	33.2	51.18	64	100.3	59.04	67.79
68039	'Nmb'	23.02	19.46	26.6	43.1	0	0.04	6.68	36.57	3.14	17.47
68040	'Zfp593'	9.99	10.57	9.34	22	0.95	4.51	6.22	6.54	2.67	6.29
68041	'Mid1ip1'	111.5	148.2	122	94.8	107	201.3	72.1	129.1	108.2	191.01
68043	'Eef1akmt1'	98.88	65.77	72.1	60.3	69	138.3	80	92.67	99.48	94.92
68044	'Chac2'	16.07	17.55	7.57	42.3	8.96	3.96	9.64	20.84	16.8	7.05
68045	'Rtraf'	66.22	65.13	53.2	60.1	43.7	62.1	52.3	79.21	62.58	60.31
68046	'2700062C07Rik'	24.98	22.75	39.6	9.91	14	20.68	25.6	12.92	31.46	17.78
68047	'Mpnd'	28.55	10.75	8.69	10.7	12.6	28.8	12.9	36.33	30.93	2.98
68048	'Aen'	20.18	14.14	22.1	20.9	21	20.77	13.9	37.79	25	22.15
68050	'Akirin1'	47.89	71.6	45.1	87.3	34.5	30.01	40.8	38.19	50.09	10.76
68051	'Nutf2'	104.9	119.5	109	104	117	180.5	113	131.5	113.1	129.8
68052	'Rps13'	235.4	280.2	312	287	223	377	284	196	297.9	166.14
68053	'Ubxn2b'	6.21	6.28	6.67	0	5.46	4.67	4.63	7.19	10.89	8.37
68055	'Atp5s'	44.47	27.2	32.5	35.2	42.6	38.79	29.4	25.89	49.31	39
68058	'Chd1l'	1.56	7.38	1.36	0.02	4.28	8.05	0.58	2.87	3.5	4.75
68059	'Tm9sf2'	84.59	110.1	89.7	99.1	74.4	62.15	90.4	109	104.3	95.4
68066	'Slc25a39'	207.4	240.5	119	239	220	212.1	192	152.5	222.1	194.48
68067	'Mrnip'	33.77	50.29	30.3	45.8	31.1	83.07	47	46.71	45.92	54.15
68070	'Pdzd2'	0.85	0.5	1.88	0.49	1.09	0.11	1.57	0.44	1.32	1.35
68073	'Fam173b'	15.48	6	5.66	0	25.6	10.88	6.56	6.68	17.8	17.42
68075	'Lurap1'	12.65	18.59	8.86	1.46	21.4	7.13	4.81	3.48	3.94	8.08
68077	'Nop53'	50.83	40.9	60.4	61.4	49.9	39.05	60.6	69.89	42.92	46.32
68079	'Pdcd2l'	27.27	24.07	15.9	37.6	32.8	34.39	23.5	34.46	48.26	12.53
68080	'Gpn3'	9.87	38.13	19.2	16.5	23.8	32.68	11.4	22.44	20.02	35.55
68082	'Dusp19'	2.38	9.72	12.5	0.04	10.2	2.85	5.24	4.59	5.18	4.11
68083	'Pak1ip1'	63.74	51.18	50.6	44.2	45.8	55.31	60.9	62.33	38.48	34.09
68087	'Dcakd'	42.35	47.54	18.7	24.3	40	45.1	20.7	18.64	31.6	19.75
68089	'Arpc4'	83.09	91.11	84.7	86.3	111	162.6	112	97.11	84.37	91.89
68090	'Yif1a'	18.47	31.22	22.6	39.8	22.5	29.42	9.88	46.81	21.71	14.71
68092	'Ncbp2'	56.06	44.78	56	57.2	49.4	67.81	27.7	54.46	36.58	41.5
68094	'Smarcc2'	22.96	17.64	31.1	17.2	26.6	8.23	20.1	24.79	27.56	22.46
68095	'Ociad1'	458.5	542.4	413	377	414	518.1	461	506.7	348	385.74
68097	'Dynll2'	27.83	14.29	21.7	15.4	43.2	23.4	32.7	21.01	28.71	15.7
68098	'Rchy1'	55.85	33.32	28.2	53.2	56.9	59.11	42.7	47.45	58.14	37.15
68099	'Fam92a'	26.64	41.43	43.5	32.6	27.2	21.11	49.3	41.85	21.09	40.74
68106	'Nt5c3b'	35.31	45.98	49.7	127	36.3	69.28	57.4	62.39	37.57	48.39
68107	'Cntd1'	0	0	0	3.03	1.17	0	2.35	0	0	0
68112	'Entr1'	14.37	25.46	26.7	53.3	12.7	13.82	18	21.08	34.4	8.94
68114	'Mum1'	24	27.32	33.2	0.18	16.3	31.16	18.4	34.84	22.81	29.46
68115	'Maip1'	16.34	31.32	17.5	4.61	3.56	2.6	5.81	16.6	15.83	11.49

68117	'Apool'	8.98	2.88	0.18	0.05	4.93	4.8	0	12.43	5.58	8.95
68118	'Atg101'	80.02	29.13	48.9	106	55.7	93.73	63.3	63.35	33.23	46.76
68119	'Cmtm3'	4.86	2.85	6.08	0	3.47	6.89	6.23	4.42	0	4.32
68121	'Cep70'	18.7	20.96	20.8	20.3	39.9	17.75	12	22	20.85	18.64
68126	'Fahd2a'	10.69	16.71	26.9	75.7	55.3	47.21	27.9	30.28	13.57	48.8
68133	'Gcsh'	68.85	69.62	112	95.8	76.6	100.2	121	48.2	32.77	75.8
68134	'Upf3b'	25.06	20.07	19.7	23.3	16.6	25.15	23	10.41	17.4	21.17
68135	'Eif3h'	127.3	131.5	156	92.2	76.9	132.3	132	104.6	95.52	151.4
68137	'Kdelr1'	49.5	83.14	42	56.3	49.5	59.72	48.3	39.5	36.28	55.72
68140	'Tigd2'	9.91	13.79	9.24	14.3	9.64	10.18	1.86	10.71	8.43	3.56
68142	'Ino80'	1.62	2.68	1.09	3.64	1.36	0.23	0.68	1.71	3.18	2.48
68145	'Etaa1'	0.83	0	0	0	0.19	0.68	0	0	0.93	2.47
68146	'Arl13b'	3.38	2.6	1.09	4.53	1.44	2.88	5.39	0.88	1.12	1.98
68147	'Gar1'	0.64	5.65	2.9	4.27	7.12	1.77	0	2.45	0.86	1.2
68149	'Otub2'	6.55	8.49	8.24	6.18	9.78	7.36	3.41	4.48	7.76	2.39
68151	'Wls'	16.93	4.06	6.07	0.32	2.84	1.51	0	0	0	8.64
68152	'Fam133b'	10.01	5.91	14.1	11.2	6.78	16.66	4.16	6.66	14.08	6.36
68153	'Gtf2e2'	14.49	26.78	10.3	9.16	24.7	17.89	6.56	27.42	18.07	30
68159	'Stx19'	0.15	0	0	0	0	0	0	0	0	0.92
68165	'Fdx1l'	75.43	56.02	114	51.5	107	88.5	60.1	69.77	58.77	31.9
68166	'Spire1'	3.94	2.95	2.51	5.41	12.3	5.2	11.4	7.57	6.73	7.86
68169	'Ndnf'	0.01	0	0	0.01	0	0.28	0	0	0	0.01
68170	'B230118H07Rik'	35.96	52.43	39.3	26.4	53.3	75.11	58.2	41.34	42.24	69.06
68172	'Rpl39l'	0	9.36	0	0	0	0	0	0	0	0
68176	'Inka1'	7.9	2.43	0.05	5.94	0	1.83	0	17.02	13.06	19.48
68177	'Ebpl'	21.66	20.66	18.1	50.8	17.4	25.24	21.2	27.29	31.35	25.24
68178	'Cgnl1'	0.1	0	0.59	0	0	0	0	0.01	0.05	0
68183	'Bcas2'	147.1	165.1	165	168	138	191.4	155	140.6	152.4	151.57
68184	'Denr'	85.56	80.51	93.9	116	87.1	119.2	80.1	100.2	98.43	77.4
68185	'Coa4'	19.6	3.87	0.07	0	32.5	43.65	3.97	31.61	17.35	19.31
68187	'Fam135a'	3.27	1.68	4.23	4	2.07	0.39	3.46	3.26	2.05	3.43
68188	'Sympk'	2.17	3.36	10.3	11.1	2.09	2.28	5.2	4.44	5.06	1.69
68192	'Leprotl1'	61.06	40.3	25.6	32.1	37.6	44.94	25.4	18.89	19.97	51.18
68193	'Rpl24'	285.2	272.7	311	431	242	306.1	252	214.8	195.2	229.78
68194	'Ndufb4'	387.3	364.6	402	539	412	682.4	467	318.1	646.2	471.35
68195	'Rnaset2b'	27.91	34.61	33.9	18.9	19.1	53	46.7	22.98	32.79	13.2
68196	'Hsbp1'	687.3	560.1	504	720	693	728.2	396	582.7	523	459.47
68197	'Ndufc2'	496.7	516.5	526	580	421	498.9	436	403.3	389.5	435.78
68198	'Ndufb2'	330.6	358.3	468	400	319	619.7	418	270.2	472.4	333.57
68201	'Ccdc34'	14.43	12.78	16.7	11.6	8.57	17.95	9.1	6.03	8.16	13.15
68202	'Ndufa5'	313	383.4	368	391	302	616.3	358	219.4	534.5	292.64
68203	'Diras2'	6.67	0.71	5.45	0	5.36	0.05	6.24	4.42	4.5	2.61
68205	'Urm1'	7.76	3.25	6.08	0	4.13	4.85	8.68	11.44	17.11	12.79
68209	'Rnaseh2c'	23.31	27.34	24.8	14.4	37.3	38.25	60	28.19	63.82	45.17
68212	'Tmbim4'	73.4	99.68	97.9	145	104	159.3	149	123	174.3	92.67
68215	'Fam98b'	2.09	9.63	1.37	11.8	8.36	16.7	9.23	2.67	10.9	7.69
68219	'Nudt21'	47.15	25.75	65.8	29.9	44.4	36.05	49.2	69.81	61.64	33.53
68222	'Fam166a'	0	0	0.1	0	0	0.46	0	0	0	0.59
68226	'Efcab2'	7.04	5.48	9.66	7.96	5.88	5.68	12.5	3.03	3.27	14.38
68229	'Spindoc'	1.2	2.94	3.2	7.93	11.2	3.88	2.99	2.71	15.01	3.32
68233	'Fam229a'	1.92	1.22	3.21	2.94	1.93	9.55	2.55	1.11	9.72	12.51
68235	'Mturn'	11.46	14.83	11.1	8.99	4.31	1.65	7.52	5.39	12.68	10.58
68236	'Gtsf1l'	0.11	0.06	0.06	0	0	0	0	0	0	0
68240	'Rpa3'	10.88	13.05	5.69	0	19.3	21	0	13.89	14.64	13.21
68241	'Mcrip2'	9.3	9.45	29.7	11.7	25.3	1.84	9.92	10.62	14.47	13.59
68250	'Ciao2a'	43.17	34.94	34.6	27	10.5	17.69	52.2	20.85	29.92	55.52
68251	'Babam1'	63.27	76.2	96	89.2	77.8	98.42	79.5	75.54	61.52	51.14
68255	'Tmem86b'	1.25	5.51	1.36	1.24	4.93	2.27	0.32	0.62	2.75	0.96

68259	'Ift80'	4.92	9.57	4.85	10.8	9.86	0	9.88	3.48	3.83	2.32
68260	'Trmt12'	3.15	4.12	5.65	3.71	5.77	2.89	8.42	7.34	6.64	3.11
68262	'Agpat4'	79.65	78.91	65.4	48.1	54.3	50.98	39	59.05	56.53	59.54
68263	'Pdhb'	205.5	166.5	177	149	239	235.9	179	196.4	153.5	186.42
68267	'Slc25a22'	55.67	71.5	84.2	48.9	49.9	57.57	103	68.39	89.05	63.31
68268	'Zdhhc21'	1.61	1.6	3.41	4.52	2.73	0.31	3.6	2.51	0.7	1.69
68270	'Dnaaf1'	0.03	0	0	0	0	0	0	0	0	0
68272	'Rbm28'	9	12.36	6.01	10.5	7.51	10.95	13.9	7.57	10.98	16.28
68273	'Pomgnt1'	1.89	16.24	5.11	17.7	10.6	10.12	20.1	5.94	25.32	12.2
68274	'Toporsl'	0	0	0	0	0	0.02	0	0	0	0
68275	'Rpa1'	23.38	26.52	14.9	11.4	30.5	42.19	15.3	25.96	26.81	31.47
68276	'Toe1'	11.63	19.73	16.1	0	13.7	4.35	0.06	15.52	26.91	3.67
68277	'2310057M21Rik'	16.46	20.76	16.7	19.9	18.4	2.88	8.4	15.31	5.58	29.89
68278	'Ddx39'	12.88	30.54	29.5	11.1	22.8	39.01	0.03	26.66	25.27	33
68279	'Mcoln2'	0	0	1.32	0	0	0	0	0	0	0
68281	'4930430F08Rik'	9.11	12.9	5.83	13.5	2.44	2.45	1.83	7.71	3.92	4
68283	'9530077C05Rik'	13.81	9.57	9.76	3.92	9.41	9.47	0.26	16.33	10.56	16.63
68291	'Mto1'	19.09	22.82	12.2	17.3	7.58	13.59	20.9	23.39	15.81	20.63
68292	'Stt3b'	2.72	2.36	0.59	5.84	6.61	1.96	3.44	1.8	7.26	3.52
68294	'Mfsd10'	24.79	20.09	23	11.2	29.8	33.83	36.8	21.04	25.53	19.82
68295	'Aar2'	20.68	27.88	32.7	15.5	44.7	25.12	40.3	33.25	18.49	30.48
68298	'Ncapd2'	0.03	2.29	14.1	1.76	1.78	0	0.04	1.7	0	2.45
68299	'Vps53'	30.34	35.8	26.6	35.6	32.8	40.58	22.2	40.97	34.59	20.3
68303	'Fam114a1'	1.94	0	3.2	1.68	0	1.45	13.1	2.23	0.01	1.39
68304	'Kdelc2'	0.18	0.64	0.3	3.41	2.05	3.13	0.18	0.73	1.31	1.83
68307	'Lrriq4'	0	1.4	0	0	0.76	0	0	0	0	2.22
68310	'Zmym1'	20.23	17.94	4.48	0.03	9.77	10.01	0.34	13.81	8.8	5
68311	'Lypd2'	0	0	0.11	0	0	0	0	0	0	0.1
68312	'Gstm7'	139.1	176.8	58.1	137	80.3	89.92	74.3	154	31.7	71.06
68316	'Apoo'	90.37	113.7	93.3	44.4	59	88.18	119	127.7	61.18	74.9
68318	'Aph1c'	2.55	0.92	1.4	1.66	2.35	0.21	2.67	0	1.05	3.24
68323	'Nudt22'	13.59	15.16	18.9	23.8	6.23	18.74	20.3	16.16	27.32	8.84
68327	'Tsr3'	21.77	13.93	48	16.9	36.5	58.79	37.4	37.14	18.74	58.29
68328	'Rab13'	7.17	1.47	3.27	0	7.85	24.7	6.34	1.61	6.75	1.44
68332	'Sdhaf1'	20.85	16.55	42.8	31.3	42.4	89.65	31.9	22.78	44.53	9.92
68337	'Crip2'	187.6	143	245	249	186	247.2	104	162.4	147	232.1
68338	'Golt1a'	0	0	0	4.2	0	0	0	0	0	0
68339	'Ccdc88c'	0.01	1.05	0.39	1.46	2.83	0.31	0.77	0.09	0.54	0.46
68342	'Ndufb10'	510.1	504.6	525	627	717	694.3	607	665.4	554.6	662.41
68344	'Tmem174'	0	0	0	6.49	0	0	2.93	0	1.48	0
68346	'Sirt5'	8.39	9.31	18.6	22.9	16.6	23.74	30.6	24.85	8.54	13.99
68347	'Mettl26'	145.1	115.3	108	238	181	169	114	115.7	121.9	155.49
68349	'Ndufs3'	182.2	214.4	308	231	295	286.3	195	314.6	199.3	219.63
68350	'Mul1'	14.72	18.33	24	23.8	16.6	9.3	19.5	21.63	20.93	13.86
68352	'Aspdh'	0	0	0	0	0	0	0	0	0	3.31
68364	'0610030E20Rik'	3.62	2.13	3.17	2.25	2.11	3	6.01	1.66	2.97	3.06
68365	'Rab14'	165.6	157.1	127	84.3	119	181.2	129	182.6	177.8	165.21
68366	'Tmem129'	23.69	20.34	25.7	5.75	13.6	21.06	21.2	39.07	0	20.53
68371	'Pbld1'	13.62	2.9	6.82	6.89	1.66	2.2	2.08	1.4	3.39	5.59
68375	'Ndufa8'	508	460.2	474	550	446	784.7	539	664.1	733.9	659.25
68379	'Ciz1'	13.27	12.91	16.2	10.8	7.05	15.99	11.8	8.51	24.66	10.35
68385	'Tlcd1'	18.96	18.3	17.9	27.8	4.13	8.71	12.1	9.05	6.32	10.87
68393	'Mogat1'	0.04	0.28	0.73	0	0.48	6.28	0.38	0.34	0.08	1.34
68394	'Ccdc163'	1.37	5.14	5.31	11.2	6.31	6.96	4.36	1.58	1.94	7.21
68396	'Nat8'	8.21	2.78	0	0	0.56	2.36	0	0.57	9.96	0
68401	'G6pc3'	68.84	49.19	45.8	34.3	57.9	77.28	88.1	61.56	58	42.15
68404	'Nrn1'	30.57	5.11	0	0.14	55.6	42.35	73.2	12.13	49.65	62.72
68420	'Ankrd13a'	1.37	5.5	0.09	11.5	1.05	2.53	0.76	0.47	0.01	1.58

68421	'Lmbrd1'	39.85	24.67	41.7	25.5	21.4	14.05	29.3	21.44	35.8	35.88
68423	'Ankrd13d'	65.62	63.33	82.4	30.6	72.7	54.09	65.1	48.02	49.85	34.36
68427	'Slc39a13'	4.89	5.88	1.49	16.2	12.1	4.13	14.8	7.95	12.54	2.86
68428	'Steap3'	1.94	4.94	2.74	14.5	0.04	0	4.82	1.85	2.59	5.8
68431	'Fbxl15'	35.33	20.18	35.5	20.2	34.8	39.65	13.8	32.01	28.79	50.66
68436	'Rpl34'	193.1	221.1	255	302	155	395	172	169.1	267.9	226.26
68440	'Dusp23'	0.19	0.03	1.76	5.44	2.08	0	5.52	3.74	7.08	14.01
68441	'Rraga'	209.7	221	187	98.6	173	190.1	194	208	252.5	176.6
68449	'Tbc1d10b'	1.54	1.08	5.57	0.64	6.08	3.38	0.57	1.44	2.64	2.44
68453	'Gpihbp1'	0	0	0.06	0	0.12	0.11	0	0	0	0
68458	'Ppp1r14a'	6.84	11.55	4.1	0	31.4	28.12	0	2.77	5.33	0
68460	'Dhrs7c'	0	0	0.04	0	0	0	0	0.18	0	0
68463	'Mrpl14'	80.65	123.4	88.6	90.4	82.7	92.7	71.8	116.4	113.3	84.46
68465	'Adipor2'	11.4	9.81	4.14	2.04	13.7	12.91	0.02	4.74	11.56	3.67
68472	'Tmem126b'	76.47	63.5	40.3	53.3	50.5	86.27	27.6	100.3	90.77	73.02
68473	'Mob1b'	0	1.2	1.17	0.52	0.23	2.19	1.86	1.9	3.45	0
68475	'Ssna1'	130.9	115.4	136	157	201	243.9	116	139.5	153.4	149.93
68477	'Rmnd5a'	1.79	1.67	0.85	6.48	5.25	3.37	0.01	2.84	0	0.76
68479	'Phf5a'	59.18	26.49	32.3	41.7	31.6	41.46	31.4	28.42	11.48	49.39
68480	'Card19'	6.7	8.68	3.88	17	4.77	6.27	19.7	5.64	15.02	2.1
68481	'Mpzl1'	5.01	2.12	30.1	9.22	7.56	2.85	35.4	2.4	8.91	1.96
68487	'Tmem140'	0	0.34	0	0	0	0	0.05	0	0	0
68490	'Zfp579'	3.17	1.29	2.76	4.06	3.97	7.41	5.32	1.56	5.1	0
68493	'Ndufaf4'	12.55	6.96	8.99	8.81	11.6	17.3	13.9	11.06	10.19	6.07
68497	'Arel1'	4.41	4.58	11	3.6	6.17	1.39	6.11	12.51	8.47	4.98
68498	'Tspan11'	1.38	0	0	0	1.64	0	0	0	0	0
68499	'Mrpl53'	154.6	94.56	87.3	144	133	133.1	76.6	83.36	51.95	105.86
68501	'Nsmce2'	31.88	28.87	23.3	20.2	35.9	27.64	53.8	48.19	36.15	48.66
68505	'Vps51'	19.47	12.88	34.3	1.61	19.3	28.62	4.65	15.87	23.09	21.24
68507	'Ppfia4'	11.63	4.93	17.4	3.37	10.2	6.06	9.52	10.87	10.95	11.02
68509	'Ptx4'	0.03	13.27	3.73	0	0	0.03	8.15	4.51	0.05	0
68510	'Ints1'	1.46	1.12	5.47	0	4.11	1.51	2.45	2.46	3.27	2.46
68511	'Dcdc2c'	1.16	3.25	4.01	4.29	6.38	2.41	0.66	1.39	3.52	1.44
68512	'Tomm5'	121.9	109.9	164	192	205	138.7	120	159.5	101.5	103.62
68514	'Micu2'	8.02	10.02	20.6	22	4.47	1.42	12.3	5.98	6.83	6.64
68515	'Myadml2'	5.08	0.03	4.43	0	0	10.69	0	1.54	7.19	0
68519	'Eml1'	18.34	7.06	7.22	5.5	18.2	7	8.67	8.45	7.32	9.58
68520	'Zfyve21'	17.14	11.8	13.5	14.7	13.8	36.48	4.1	19.23	12.91	7.74
68521	'Fam189b'	15.98	28.1	11	6.36	24	10.24	15.8	17.86	25.21	26.1
68523	'Ciao2b'	83.02	85.22	108	30.5	82.6	82.68	56.1	65.43	72.18	84.76
68524	'Wipf2'	2.16	1.11	2.32	7.12	3.6	0.87	5.27	2.78	5.93	3.8
68525	'Evc2'	0	0.36	0.01	0	0	0	6.31	1.64	0	0
68526	'Gpr155'	8.33	2.9	10	6.76	6.11	3.35	5.09	10.74	3.03	7.58
68528	'Smim6'	0	0	0.05	0	0	0	0	0	0	0
68533	'Mphosph6'	21.57	8.8	20.6	28.8	29.4	13.66	12.6	20.21	18.53	24.05
68537	'Mrpl13'	85.19	80.98	68.5	126	82.3	102.2	81	68.25	73.64	68.74
68539	'Tmem109'	76.9	93.05	42.7	63.6	54.4	82.2	76.1	71.73	51.28	140.12
68544	'Trir'	16.72	18.58	22.5	50.2	30.1	17.76	37.9	24.75	38.4	32.34
68545	'Ecscr'	0	0	0	0	5.71	11.64	5.12	2.05	0	0
68549	'Sgo2a'	0.07	0.12	0.2	1.04	0.13	0.21	0	0.58	0.04	1.84
68550	'Tefm'	12.46	10.32	8	0	9.32	15.51	0.05	16.65	13.1	13.18
68552	'Smim14'	120.2	109.5	91.3	131	104	128.2	115	126.5	121.5	109.7
68553	'Col6a4'	0.26	0.66	4.67	0	0.28	0	0	0.35	0	1.01
68554	'Cebpzos'	81.77	55.85	63.1	46.2	60.5	87.87	61.9	71.09	68.39	60.92
68556	'Uckl1'	28.78	31.35	42.4	85.5	32	53.82	10.2	38.47	46.94	37.7
68558	'Ankra2'	29.07	31.64	39	37.1	23.3	38.56	23.3	32.83	25.2	14.55
68559	'Pdrg1'	95.2	49.57	89.5	89.9	123	130.4	93.4	119.5	130.3	167.48
68563	'Dpm3'	21.45	30.16	22.3	38.1	17.1	53.47	52	20.91	73.22	23.08

68564	'Nufip2'	5.59	3.97	3.52	4.73	8.82	4.8	6.39	5.99	4.28	4.65
68565	'Mrps18a'	119.5	135.7	148	165	174	195.7	109	162.6	103.6	111.98
68566	'Caly'	1150	1160	1039	1373	994	1379	918	1162	1440	1649.15
68567	'Cgref1'	26.85	25.99	34.4	25	33.7	26.66	28.4	25.71	32.33	32.35
68572	'Mrpl58'	83.69	54.48	91.5	88.6	105	118.6	20.4	87.14	90.22	106.19
68576	'Lamtor5'	140.9	156.4	139	178	174	158	160	117.3	189.9	102.59
68581	'Tmed10'	80.12	79.77	61.3	66.1	77	95.15	56.7	73.87	103.1	126.82
68585	'Rtn4'	96.6	37.64	46	50.6	97.7	96.72	46.7	44.23	36.67	42.26
68588	'Cthrc1'	40.59	25.31	22.2	20.5	21.7	27.32	36.6	35.36	61.25	53.01
68591	'Mocos'	0.03	0.31	0.1	0	0	0.03	0	0	2.65	0
68592	'Syf2'	49.13	74.39	51.6	89.2	47.3	57.96	55.4	61.52	50.67	50.22
68597	'Ccdc167'	10.11	6.33	18.1	12.7	12.6	11.72	13.6	6.15	6.44	12.01
68598	'Dnajc8'	124.4	129.1	127	163	85.5	169.5	93.9	141.8	145.2	106.67
68603	'Pmvk'	84.33	70.58	95.9	141	107	126.5	84.2	107.8	78.51	136.46
68606	'Ppm1f'	20.69	16.32	30.6	6.79	12	9.63	24	19.42	26.14	21.16
68607	'Serhl'	0	3.14	16.4	11.7	0	0	6.07	0	0.74	0.08
68611	'Mrpl28'	148.8	124.8	128	146	167	296.3	131	188.1	194.1	129.97
68612	'Ube2c'	0.11	0.12	0.11	0.32	0.36	0.28	0.06	0.07	0.05	0.1
68614	'Letmd1'	25.22	9.34	9.17	22.3	6.33	10.5	23.9	16.55	4.64	12.88
68616	'Gdpd3'	0	0	0.09	0	0	0	0	0	0.37	0.04
68617	'Mtcl1'	0.85	0.47	1.94	5.44	1.15	1.86	1.91	2.75	0.47	2.19
68618	'1110012L19Rik'	34.49	29.21	43.1	21.8	41.3	45.42	16.8	27.22	58.88	34.07
68625	'Cfap57'	0	0	0	0	0.9	0	0	0.16	0	0
68626	'Elac2'	11.35	6.69	8.78	11.7	11.1	3.93	12.4	1.1	12.35	2.46
68628	'Fbxw9'	19.29	25.15	21.6	15.2	9.48	28.74	4.71	24.16	22.33	23.43
68631	'Cryl1'	11.03	12.18	32.4	14.3	16.9	37.74	27	9.19	12.81	28.78
68634	'Tm2d3'	256.5	234.5	197	268	214	371	222	290.1	304.8	335.91
68636	'Fahd1'	22.81	25.45	23.6	31.4	16.6	34.95	26.5	23.17	9.9	18.73
68642	'Tmem216'	26.68	14.34	11.8	35.7	51.9	9.37	11.6	26.76	34.2	4.18
68644	'Abhd14a'	35.09	50.68	39.3	48.5	14.3	13.19	12.8	18.93	38.68	44
68646	'Nadk2'	8.64	5.44	2.83	1.1	1.59	1.73	9.62	2.26	2.32	2.17
68652	'Tab2'	2.16	3.16	1.92	8.08	4.01	1.42	7.76	3.72	2.19	6.23
68653	'Samm50'	82.46	84.29	71.8	92.3	79.7	101.6	73.1	96.85	89.68	95.23
68655	'Fndc1'	1.76	0	0.77	0	1.22	0	2.1	0	0	0.69
68659	'Fam198b'	0	0.01	0.03	0.07	0.01	0	0.03	0	0.03	0.02
68666	'Svop'	86.25	54.97	74	6.35	78.9	25.47	78.9	84.09	93.01	69.29
68667	'Trpm4'	0.85	1.28	4.81	0.01	1.62	6.61	1.79	1.19	6.81	7.05
68671	'Pcyt2'	102.2	106	71.1	69.4	133	127.7	132	99.26	153.1	108.08
68675	'Fam172a'	10.77	11.22	14.6	29.5	8.29	2.93	7.11	9.57	4.77	4.02
68678	'Smtnl1'	0	0	0.02	0	0	0	0	0	0.56	0
68680	'Fitm1'	0.1	0	2.04	0	0	0.11	0.11	0	0.05	0
68682	'Slc44a2'	10.98	8.65	9.59	12.4	11.7	10.84	12.4	11.45	15.2	12.18
68691	'Kansl1l'	0.14	0.91	1.11	3.45	1.27	1.82	0.42	0.34	0.95	1.01
68693	'Hnrnpul2'	2.67	2.98	3.25	3.9	4.17	3.69	2.01	4.29	6.51	3.54
68695	'Hddc3'	2.56	24.36	17.3	29.6	12.3	12.28	40.1	29.26	28.79	14.38
68703	'Rere'	5	5.79	7.35	9.69	3.37	6.45	3.92	4.21	2.21	3.63
68705	'Gtf2f2'	18.23	14.47	11	2.92	0.61	14.29	2.35	10.87	9.68	10.41
68708	'Rabl2'	59.92	69.37	45.2	49.6	52.6	58.57	80.1	58.5	53.15	42.44
68709	'Cilp2'	0.27	0.06	0	0	0	0	0	0	0.7	0
68713	'Ifitm1'	0	0	0	0	0	0.18	0	0	0.09	5.24
68718	'Rnf166'	5.25	15.69	5.55	0.03	4.05	18.11	14.3	11.39	18.2	5.87
68721	'1110032A03Rik'	46.48	38.84	28.5	40.1	45.5	42.63	33.1	38.63	26.34	43.48
68724	'Arl8a'	19.34	16.69	17.3	53.4	47.2	16.01	35	9.07	16.64	26.7
68725	'1110032F04Rik'	0.07	0	0	0	1.48	0	0	1.51	0	1.35
68728	'Trp53inp2'	15.78	2.21	13.3	15.2	84.2	5.46	13	11.93	19.92	18.92
68729	'Trim37'	19.17	13.96	32.5	22.8	21.1	26.79	14.8	22.81	26.29	16.73
68730	'Dus1l'	21.97	28.58	40.5	18.6	39	33.5	25.8	27.84	19.77	24.62
68731	'Rbfa'	49.24	30.28	23.5	14.9	38.3	27.78	30.2	49.62	23.24	40.42

68732	'Carmil1'	4.03	8.96	4.58	4.16	7.6	8.57	6.44	5.11	2.67	6.29
68734	'Ppp4r3a'	3.51	3.25	1.09	6.91	2.47	1.58	1.53	1.63	4.01	2.21
68735	'Mrps18c'	147	129.9	152	212	183	261.1	136	140.3	154.6	140.41
68736	'Tyw5'	18.73	34.57	15.7	22.7	7.78	18.45	31	18.69	11.72	14.24
68737	'Angel1'	4.36	0.74	0	7.23	0.01	5.97	6.8	5.27	1.42	4.99
68738	'Acss1'	12.17	9.21	3.17	0.02	2.9	3.45	4.62	2.25	4.85	2.84
68742	'Tmem219'	27.01	41.48	37.1	19	24.3	18.84	18.7	34.01	30.89	24.01
68743	'Anln'	2.33	0.93	0	0	5.7	0	0.02	0	2.62	0
68744	'Zfp740'	4.99	2.17	6.99	24.2	5.88	2.36	5.13	3.97	8.53	2.84
68750	'Rreb1'	0.85	1.6	0	6.03	0.42	0.68	0	0.48	0.2	0.01
68753	'Mybphl'	0	0	0	0	0	0	0	0	0	0.31
68755	'Cgrrf1'	37.75	53.44	54.8	27.5	28.3	45.39	24.7	56.7	20.71	33.44
68758	'Abhd11'	40.69	32.67	43.7	70	51.8	126.3	43	52.21	37.95	69.38
68760	'Synpo2l'	0.12	0	0.01	0.02	1.24	0	0	0.01	0	0
68764	'Cdhr3'	0	1.7	0	0	0	0.02	0	2.12	0	0.78
68767	'Washc1'	8.32	14.25	9.86	6.73	13.5	15.14	14.6	7.31	30.86	16.87
68770	'Phtf2'	2.18	2.09	3.32	4.15	0.47	2.84	3.35	2.01	1.26	2.97
68774	'Ms4a6d'	0	0	0	0	0	0.54	0	0	0	0
68775	'Atp6v1c2'	3.83	0	1.72	10.4	3.59	10.83	0.03	3.12	0	0
68776	'Taf11'	41.6	54	54.9	34.1	43.9	42.72	47.2	40.15	30.66	39.36
68777	'Tmem53'	16.83	10.01	8.33	8.72	3.67	7.73	25.2	9.26	10.5	12.74
68778	'Gucd1'	2.84	6.69	6.6	0	0.02	3.03	6.51	0.1	23.27	3.47
68792	'Srpx2'	0	0	0	0	0	0	3.2	0	0	0
68794	'Flnc'	0	0	0.02	0	0.58	0.06	0.04	0	0	0.05
68795	'Ubr3'	3.77	4.27	5.35	4.73	6.22	3.15	4.84	6.93	3.19	4.38
68796	'Tmem214'	25.84	25.59	47.1	24.3	27.5	19.72	22.7	5.04	36.45	18.63
68797	'Pdgfrl'	0	0	0	0	0	0	0	0	0	0.84
68799	'Rgmb'	7.49	5.03	1.92	14.1	4.06	1.3	8	5.87	5.25	2.26
68801	'Elovl5'	45.34	20.13	24.9	14.6	22.3	23.3	16.8	23.07	30.05	57.89
68802	'Mypn'	0.99	0	0	0	0	0.01	0	2.23	0	0
68810	'Nexn'	0.1	0.05	0.18	0	0.35	0.1	0.24	0.05	0.63	0.06
68813	'Dock5'	0.03	0.76	0	0	0.26	0	0	0.75	0	0.53
68815	'Btbd10'	19.3	42.08	53.7	37.2	39.2	25.9	29.6	37.4	20.06	23.6
68816	'Ppil1'	31.85	21.57	32	23.9	23.4	58.16	30	67.13	68.67	16.19
68817	'Ddi2'	0.16	1.51	0	0.27	0.1	0	0.04	0	0	0
68818	'Zfand2b'	21.12	31.45	32.7	20.8	13.7	73.72	0	37.92	25.29	46.62
68828	'Sync'	0.02	1.67	3.46	8.22	3.44	0	0	1.21	0	0
68832	'Ldah'	26.88	25.57	19.7	17.1	30.8	21.74	18.1	24.94	28.29	24.72
68833	'Pdcl3'	22.7	25.95	18.9	41.2	32.2	50.19	17.9	29.24	35.68	19.11
68836	'Mrpl52'	102	75.01	91.6	128	76.7	164.4	103	55.24	105.8	78.12
68837	'Foxk2'	3.52	2.1	2.11	2.98	3.41	1.66	1.72	1.11	0.2	1.7
68839	'Ankrd46'	80.1	124.1	155	113	109	100.8	143	161.3	88.48	99.09
68842	'Tulp4'	12.81	8.88	5.8	10.8	8.17	8.47	1.92	2.59	8.07	10.65
68845	'Pih1d1'	17.73	12.8	31.4	9	32.7	26.5	31.3	10.55	10.04	38.69
68846	'Rnf208'	66.34	67.97	78.4	25.6	78.2	69.39	114	82.57	79.48	72.61
68852	'Lrrn4cl'	6.02	0.39	5.87	0.95	0.63	7.36	4.56	0.24	6.7	0.31
68854	'Asb11'	0.09	0.04	0.3	0.09	0.04	0.13	0.04	0.06	0.21	0.03
68857	'Dtwd2'	0.62	0.15	0.86	0	1.55	0	0	0.01	0	0
68859	'Smim1'	11.1	8.54	18.4	61.6	15.3	11.2	17.4	28.87	14.85	25.27
68861	'1190002N15Rik'	0.6	1.82	1	0	1.28	0.06	2.12	4.4	2.52	1.22
68865	'Arv1'	3.13	11.62	2.16	0	10.1	1.17	2.8	4.47	5.59	8.7
68867	'Rnf122'	2.12	3.05	4.39	7.69	0.71	1.59	0	1.26	1.63	1.95
68870	'Ak8'	5.34	3.54	3.57	7.46	3.86	13.35	4.92	4.56	4.26	9.91
68874	'Klhdc9'	26.76	15.84	21.6	35.8	28.3	25.51	21.3	13.85	14.66	14.56
68875	'Tmcc2'	5.58	5.71	3.02	6	1.12	2.49	18.3	3.92	2.53	2.25
68876	'Atp23'	5.36	1.91	7.19	0.06	7.12	0.47	1.19	9.25	8.47	0.15
68877	'Maf1'	98.29	92.98	100	49.8	73.8	102.2	126	96.08	96.04	107.81
68879	'Prpf6'	37.86	22.24	26.3	24.9	51.4	20.44	31.7	29.26	34.49	40.25

68888	'Gkn3'	0	0	0	0	0	0	0	0	0	0.29
68889	'Ubac2'	44.27	47.09	39.1	9.52	46.2	64.44	25	46.87	28.59	45.57
68895	'Rasl11a'	11.58	12.19	6.06	13.3	0.04	0	5.53	4.36	3.65	6.87
68897	'Disp1'	1.67	1.31	0.58	0.78	0	1.42	5.22	1.92	0.01	4.52
68904	'Abhd13'	0.01	1.12	1.09	0.44	0.86	0	0	0	1.89	1.31
68910	'Zfp467'	31.15	18.54	28	39.5	20.6	32.92	20.7	13.76	9.84	10.01
68911	'Pygo2'	0.83	7.79	3.57	2.69	7.85	5.39	0	1.42	1.8	2.21
68915	'Vars2'	2.37	1.4	1.45	4.01	4.33	5.27	1.22	0.8	4.2	3.29
68916	'Cdkal1'	4.69	5.3	5.74	1.05	8.61	5.26	3.87	6.06	4.76	5.52
68917	'Hint2'	148.3	139.9	171	192	247	252	221	145.7	181.8	211.52
68918	'1190005I06Rik'	1.68	8.06	0	0	9.25	14.57	0	2.23	11.32	6.69
68920	'1110065P20Rik'	37.01	28.29	73.7	28.4	55	62.98	18.9	40.51	53.23	21.47
68922	'Dnaic1'	1.16	5.96	1.34	0	3.19	7.58	0.19	0.83	0.1	1.85
68925	'Rpap1'	5.42	8.78	6.71	0.04	0.9	1.47	0.2	3.57	1.49	2.57
68926	'Ubap2'	5.86	9.58	6.35	3.23	3.62	6.2	4.65	7.84	11.02	9.1
68927	'Ptcd2'	35.21	32.26	31.2	38.6	18.4	70.33	64.4	27.01	28.73	45.84
68929	'Mospd3'	11.32	10.3	3.67	15.9	19.1	6.19	23.5	11.97	5.78	2.39
68936	'Smim11'	35.3	33.8	54.5	36.8	27.7	35.12	46.6	39.24	45.31	49.39
68938	'Aspscr1'	45.22	71.99	79.9	74.4	86.3	94.87	36.6	102.3	93.22	68.32
68939	'Rasl11b'	6.14	12.52	8.16	64.5	9.66	14.35	0.06	5.59	2.91	1.33
68942	'Chmp2b'	46.48	58.1	41.4	37.2	35.4	35.52	37	59.88	24.27	24.16
68943	'Pink1'	23.74	26.6	24.5	25.4	22.8	24.64	47.8	30.11	25.74	14.22
68944	'Tmco1'	25.16	22.98	16.5	19.9	15.2	21.57	22.2	20.34	12.06	21.46
68947	'Chst8'	6.74	26.29	4.97	1.59	27	47.99	53.5	4.58	0	28.54
68948	'Fam216a'	219	162.8	215	310	126	110.8	130	175.5	139.9	88.57
68952	'Fam57b'	8.25	12.27	7.5	7.33	20.5	21.11	5.97	7.73	9.98	4.93
68953	'Chmp2a'	235	191	230	254	237	197.7	152	216.1	205.4	163.38
68955	'Srrm4'	3.21	2.82	5.29	10.1	7.2	4.84	3.37	5.42	8.46	9.62
68957	'Paqr6'	31.63	15.12	3.66	10.5	1.46	18.24	4.22	12.77	15.5	0.81
68961	'Phkg2'	29.29	48.55	48.8	70.9	52.1	60.99	61.8	64.8	76.5	36.04
68964	'Ctc1'	11.39	10.25	16.9	13.7	8.36	6.23	4.23	10.48	10.4	19.49
68966	'Ngdn'	34.43	38.79	47.3	38.3	36.5	47	48.7	26.16	26.97	21.53
68968	'Cdan1'	1.22	0.85	3.73	2.76	1.44	0.15	0.84	0	1.85	0.44
68969	'Eif1b'	423.5	270.4	281	308	267	259	198	312.9	196.5	163.99
68970	'Dcaf12'	32.56	18.92	14.3	21.7	10.1	14.99	11.6	13.35	13.34	9.47
68971	'Tamm41'	16.33	11.09	37.1	21.1	30.2	28.79	55.5	30.81	28.25	20.36
68972	'Tatdn3'	4.52	13.7	17.7	25.1	7.91	12.73	30.7	13.82	21.37	9.73
68975	'Med27'	23.21	25.39	38.1	37.3	11.8	45.89	27.3	31.76	43.23	37.43
68977	'Haghl'	190.8	138.9	235	209	368	302.9	321	323.7	264.9	255.93
68979	'Nol11'	11.91	13.59	8.54	33.5	11.8	1.82	23.3	14.71	2.77	17.58
68980	'Wdr53'	4.89	5	17.7	27.8	7.47	10.19	11.7	19.7	9.33	8.19
68981	'Snrpa1'	29.89	46.99	34.4	33.6	38.5	39.44	21.3	39.82	38.65	42.32
68988	'Prpf31'	10.38	5.42	17.4	22.2	22	14.29	15.3	14.01	5.61	11.56
68991	'Ssu72'	123.9	118.3	126	185	152	112.9	138	109.4	154.6	133.33
68992	'Zfp580'	2.48	15.07	6.47	0.05	6.76	24.42	11	4.76	9.81	19.81
68995	'Mcts1'	103.1	130.3	74.8	129	68.4	72.7	94.7	137.7	92.04	105.95
68999	'Anapc10'	21.59	38.2	22.8	44.8	27.7	17.2	24.1	26.01	10.24	28.58
69008	'Cab39l'	21.5	14.06	9.68	8.83	24.1	15.64	4.77	11.92	26.44	16.74
69009	'Thap7'	26.84	34.3	14.8	35.3	17.1	31.57	14.1	30.45	31.47	27.11
69010	'Anapc13'	75.11	56.12	77.3	121	78.5	136.6	63.6	35.99	83.6	49.42
69017	'Prrt2'	77.92	59.68	64.9	10.9	39.1	51.71	107	64.66	33.42	36.04
69019	'Spcs1'	325.2	264.3	238	290	262	320.1	246	314.2	307.4	340.13
69020	'Zfp707'	7.06	4.95	2.58	0	3.25	0	0	9.37	7.65	5.13
69024	'Snx15'	58.8	41.97	33.5	23.3	47.4	30.9	62.1	25.92	24.27	40.75
69028	'Mitd1'	4.26	4.53	6.52	14.7	11.8	17.56	4.87	10.83	8.38	8.71
69029	'Smdt1'	355.9	249.4	287	220	415	323.5	201	273.3	347.1	238.52
69034	'Nupr1l'	0	0	0	0	5.57	0	4.04	2.84	0	6.65
69035	'Zdhhc3'	12.43	4.52	34.3	0	2.46	7.25	22.2	7	10.35	5.35

69038	'Tmem258'	188.1	230.4	117	115	124	187.9	160	142.2	242.1	122.5
69046	'Isca1'	189.6	190	185	155	145	160	138	170.2	133.7	208.88
69047	'Atp2c2'	0	5.03	1.61	0	0	0.01	0	1.83	0	0
69048	'Slc30a5'	2.3	2.78	3.55	2.82	7.88	0.86	2.56	1.06	10.26	4.66
69049	'Nat8f5'	0.05	0	0	0	0	0	0	0	0	0
69051	'Pycr2'	44.51	51.88	42.6	21.9	85.1	62.31	38.5	59.71	86.38	79.91
69053	'1810013L24Rik'	2.93	2.44	4.38	7.41	5.17	0.41	0.13	3.58	3.7	4.35
69060	'Pnlip'	0	0	0.03	2.53	0	0	0	0	0	0
69064	'Fuom'	9.37	23.94	17.1	20.9	13.6	24.68	14.3	13.54	16.72	15.44
69065	'Chac1'	4.52	3.68	4.52	0.18	19.9	5.15	12.3	13.67	10.04	0.27
69066	'1810010H24Rik'	3.76	4.04	7.23	12.6	1.02	1.16	0.05	0	5.36	6.2
69068	'Tcim'	0	0	0	0	0	0.04	0	0	5.03	0
69071	'Tmem97'	24.45	19.23	35.4	36.3	15.7	33.12	42.1	30.32	53.54	37.51
69072	'Ebna1bp2'	31.49	20.27	26.6	35.2	19.8	38.7	28.6	25.86	15.61	18.86
69076	'Triap1'	21.15	35.83	26.9	14.2	25.5	43.49	35.2	24.44	21.16	25.55
69077	'Psmd11'	119.4	137.7	88.4	113	136	77.91	120	144.5	104.5	108.91
69080	'Gmppa'	33.19	45.76	19.3	40.9	26.5	35.48	34.7	30.58	28.36	35.92
69082	'Zc3h15'	87.52	88.79	68.1	108	48.1	67.06	55.4	69.37	99.26	74.67
69085	'Zcchc9'	13.22	7.85	20.9	43	14.1	6.55	15.7	9.17	20.06	15.48
69089	'Oxa1l'	22.12	10.49	23	35.6	9.43	20.89	11.3	12.62	10.01	16.38
69090	'Ascc1'	30.94	40.6	41.7	46.5	32.3	25.15	67.5	29.51	37.33	41.14
69091	'Vps26b'	9.67	15.71	17.2	7.26	2.01	10.61	12.9	8.61	9.37	5.68
69094	'Tmem160'	13.79	6.57	16.8	122	17.3	12.06	0.19	5.97	18.51	0.89
69097	'Trim15'	1.71	0.51	0	0	5.49	0	0	0	0	10.98
69101	'Ydjc'	44.87	27.74	23	17.8	16.2	30.23	32.4	28.57	23.32	40.01
69104	'March5'	4.87	6.55	8.34	42.2	8.11	2.39	13.6	2.95	18.05	2.3
69106	'Stoml1'	70.91	85.6	124	97.3	78.7	83.03	131	86.82	138.5	92.48
69109	'Ccnq'	44.04	22.24	44.5	12.7	46.4	52.08	26.2	37.31	22.08	40.74
69113	'Alkbh3'	21.46	11.42	11.2	2.03	10.8	0.85	28.7	5.47	11.06	19.29
69116	'Ubr4'	3.98	4.39	5.61	5.71	6.87	4	2.71	5.05	6.17	2.69
69121	'Chrdl2'	0	0	3.07	0	0	0	0	0	0	0
69123	'Eci3'	0	0	0	0	2.94	0	0	0	0	0
69125	'Cnot8'	35.45	66.46	45.8	59.7	64.7	75.13	88.6	54.47	62.1	41.49
69126	'1810022K09Rik'	191.9	235.5	204	225	113	260.7	170	126.5	172.7	137.69
69129	'Pex11g'	2.72	0	0.1	0	6.49	8.75	2.05	3.3	1.52	2.33
69131	'Cdk12'	0.57	2.68	2.39	11.7	2.37	0.45	2.98	1.15	2.78	2.05
69136	'Tusc1'	0	0	0.11	4.13	0.26	0	0	0.66	0.35	0
69137	'Vstm5'	0.82	6.42	25.1	8.76	12.6	6	5.69	25.37	10.63	11.42
69146	'Gsdmd'	0	0	0	0	0	0	0	0	0	2.97
69147	'2200002J24Rik'	0	0	0.14	0.08	0	0.07	0.07	0	0	0.06
69149	'Kbtbd3'	7.25	3.55	7.8	2.45	4.29	7.16	7.11	8.25	8.13	3.81
69150	'Snx4'	45.8	60.94	52.8	26.7	50.9	34.81	51.3	41.26	52.93	57.73
69151	'Lzic'	8.99	9.34	0	4.57	0.97	9.62	0	11.1	8.84	0.6
69155	'1810030O07Rik'	17.84	8.25	17.6	26.2	18.3	15.78	28.6	25.82	14.18	16.23
69156	'Comtd1'	17.22	17.5	23.8	20.6	24.7	37.39	28.7	26.48	21.57	46.8
69159	'Rhebl1'	9.62	9.03	0.04	0	26	16.47	0.09	4.69	5.89	8.17
69161	'Manbal'	89.69	95.01	86.2	81.3	104	126.2	113	111.3	107.4	135.15
69162	'Sec31a'	11.19	7.58	18.5	4.64	11.6	7.67	7.79	7.75	35.93	12.68
69163	'Mrpl44'	56.18	26.57	29.4	34.3	30.1	32.65	43	48.17	22.33	47.56
69168	'Bola1'	37	37.3	45.3	43.5	63.9	52.69	49.4	65.33	47.54	33.34
69169	'Fcmr'	0	0.85	0	0	0	0.12	0	0	0	0.64
69171	'Cnppd1'	8.93	13.22	10.2	9.95	6.88	9.6	12.2	6.1	8.61	12.75
69178	'Snx5'	22.58	29.62	30.9	27.3	16.9	14.63	18	19.25	13	36.61
69179	'Tmem110'	2.33	0.27	3.8	0	4.58	0.16	4.98	0.51	2.34	0.7
69181	'Dyrk2'	0.01	2.95	1.13	4.49	0.12	0.84	1.86	0.04	0.54	1.52
69183	'C1qtnf2'	4.37	1.14	2.56	0	4.99	0	0.93	0	4.04	0
69185	'Dtwd1'	15.99	24.86	16.4	11.9	2.28	6.04	5.35	11.85	10.21	7.38
69186	'Tmem256'	113.9	111.3	143	192	113	173.6	151	99.79	87.36	72.53

69188	'Kmt2e'	34.3	28.89	16.3	13.7	29.7	52.11	24.6	20.22	28.14	30.79
69189	'Mcemp1'	0	0	0	0	0	0.04	0	2.92	0	4.81
69190	'Dym'	30.18	46.93	25.8	9.05	31.6	31.58	23.4	17.22	25.39	30.28
69191	'Pdia2'	0	0	0.03	0	0	0	0	0	0	0
69192	'Dhx16'	9.02	7.36	6.78	0.08	1.84	2.73	2.34	6.77	9.59	5.08
69195	'Tmem121'	10.72	5.19	24.2	4.13	9.39	6.71	12.9	6.46	11.19	1.99
69202	'Ptms'	20.26	17.24	22.7	25.9	44.9	20.9	25.3	22.62	24.52	15.19
69207	'Srsf11'	52.62	59.71	61.8	64.8	38.9	59.86	59.9	40.8	74.38	36.49
692132	'Trappc3l'	0	0	0	1.43	0	0	0	0	0	0.11
69215	'Sat2'	10.25	19.76	21.4	0	46	35.12	24.3	20.98	26.53	13.37
69216	'Svbp'	43.46	41.6	38	17.5	25.8	54.59	44.4	51.4	31.64	34.35
69217	'Plekha4'	0.06	0	0.53	0	0	5.91	4.31	0	0	0.02
69219	'Ddah1'	24.17	44.47	25.3	31.2	26.9	42.41	56.8	22.35	38.67	18.29
69225	'Naxd'	65.97	60.76	72.8	38.4	75.4	149.3	97.4	70.72	66.36	73.03
69226	'Snx24'	13.45	8.55	2.62	2.45	15.9	0.36	0.03	6.1	0.1	2.28
69227	'Selenot'	10.08	13.93	20.3	13.2	14.5	6.87	15.1	17.52	11.81	15.28
69228	'Zfp746'	2.9	1.24	9.27	0.03	1.13	0	0.01	0.24	1.61	0.62
69232	'Qrich1'	34.56	45.15	47.1	43.2	43.2	8.79	17.1	40.12	48.17	25.78
69234	'Zfp688'	20.9	13.03	21.2	27.4	29.9	20.78	15.3	24.48	44.23	31.72
69237	'Gtpbp4'	20.25	34.59	23	22.5	18.2	29.89	26.8	35.49	52.15	18.47
69239	'Pdzph1'	0	0	0.96	4.59	1.77	2.09	2.42	2.01	0	1.75
69241	'Polr2d'	32.89	51.6	43.2	60.6	45.8	67.04	67.3	71.56	26.42	65.18
69253	'Hspb2'	0	0	0.42	0	0	0.11	0	0.08	0	0.05
69256	'Zfp397'	3.26	5.05	1.52	0.01	5.4	1.85	3.16	2.92	2.25	4.84
69257	'Elf2'	9.25	4	1.56	0.07	3.04	2.59	5.06	4.4	8.3	13.64
69259	'Kctd5'	4.41	4.05	10.4	13.8	7.38	0	0	1.53	4.7	1.93
69260	'Ing2'	0	0.75	0.56	4.9	1.55	0.08	0	2.26	1.6	1.08
69263	'Rfc3'	21.43	5.87	4.13	12.3	35.6	43.3	18.9	11.33	15.15	26.49
69269	'Scnm1'	75.29	53.41	71.4	151	117	52.48	33.6	51.35	65.56	78.05
69270	'Gins1'	0	4.42	0	11.2	0	0	0	2.02	0	0
69274	'Ctdspl'	2.74	1.5	2	5.9	3.49	2.27	2.13	3.39	0.81	1.47
69276	'Sec62'	50.03	67.17	57.4	78	46.4	83.13	66.6	37.74	83.85	53.29
69277	'3300002I08Rik'	5.47	6.44	0.1	4.28	8.68	3.21	1.5	0.07	7.43	10.77
69281	'Spata4'	0.04	0	0	0	0.1	0	0.44	0	0	0
69282	'1700001J03Rik'	0	0	0.07	0	0	0	0	0	0	0
69286	'Glipr1l1'	0	0	0	0	0	0	0.58	0	0	0
69288	'Rhobtb1'	0.01	0	0.04	0	0.36	0.01	0.04	0.36	0.92	0.03
69297	'Lrrc46'	0.17	0.04	3.67	10.8	0	3.27	0.43	2.81	0	2.78
69301	'Tescl'	0	0	1.53	0	0	0	0	0	0	0
69305	'Dcps'	30.62	17.64	23	44.3	23.3	15.37	43	36.96	24.92	53.49
69306	'Efcab9'	0	0	0	0	0	0	0	0.13	0	0
69309	'Slc16a13'	1.34	0.02	1.7	0	0	6.39	4.35	7.33	0	2.97
69310	'Pacrg'	35.44	24.9	13.7	14.4	31.9	25.03	18.8	10.61	11.83	7.47
69312	'Ppp1r42'	0	0	0	0	2.07	4.1	0	0	0	0.79
69315	'1700001L19Rik'	46.5	32.15	20.7	12.4	52.7	50.74	20.3	38.07	22.52	29.81
69318	'Fam24b'	0	0	0.15	0	0	0	0	0	0	0
69325	'1700012B09Rik'	13.38	3.8	3.81	0	19.4	0	0	17.52	0	0
69327	'1700007K13Rik'	4.59	0	7.56	26.7	12.8	2.29	0	7.91	7.3	5.39
69329	'Cfap206'	1.31	0.28	2.4	11.7	13.3	0	0.03	0	1.23	3.45
69349	'1700008O03Rik'	0.06	1.95	3.23	0	1.46	3.34	4.4	2.25	6.05	2.49
69352	'Necab1'	0.03	3.22	8.19	3.04	13.7	12.15	2.86	12.68	8.37	0.62
69354	'Slc38a4'	0	1.67	0.02	1.14	1.35	0.96	0	2.41	0.48	1.02
69358	'Lrrc51'	15.13	8.11	0.1	17.9	28.1	29.18	0	11.24	7.83	4.4
69367	'Glrx2'	31.99	62.7	49.1	17.8	33.1	32.57	29.6	39.58	46.8	24.84
69368	'Wdfy1'	9.1	15.25	11.2	8.39	6.82	1.78	0.72	8.09	13.68	6.82
69371	'Smco2'	0	0.04	0	0	0	0	0	0	0	0
69372	'Mocs3'	2.67	1.12	0.35	0.07	1.83	2.68	0.54	0.05	0	1.46
69376	'Zpbp2'	0	0	0	0	0	0	0	0	0.37	0

69379	'C8g'	9.4	4.23	0.55	26.5	10.4	0.29	8.53	0.05	0.11	8.95
69384	'Tmem89'	0	0	0	0	0	0	0	0	0	4.58
69386	'Hist1h4h'	3.55	9.79	5.48	20.2	16.1	14.46	8.78	3	13.41	13.23
69387	'Dnajb13'	0	0	0	0	0	0	0	0	0	4.26
69399	'1700025G04Rik'	2.57	8.24	5.45	1.31	1.69	3.32	2.56	3.27	12.13	1.49
69408	'Dnajc17'	27.08	39.07	18.3	42.8	21.2	23.72	3.65	20.3	6.15	24.65
69428	'1700016C15Rik'	0	0	0.1	1.02	0	0	0	0	0	0
69440	'Dennd6b'	40.7	22.8	40.3	30.2	40.6	33.66	29.3	23.2	41.33	38.02
69441	'1700023F06Rik'	5.06	10.6	7.7	20.6	6.92	16.24	9.38	9.15	13.07	9.79
69453	'Prss56'	0	0	0.29	0	0	2.72	3.43	0.76	0	1.85
69454	'Clic3'	0	0	0.57	0	0	0	0	0	0	0.45
69456	'Commd10'	27.15	37.63	26.5	67.7	34	29.59	31.5	41.17	48.73	36.79
69459	'Ubl7'	95.14	116.9	122	173	83.1	158.8	92.6	109.4	111.7	90.79
69462	'Slurp2'	0	4.65	0	0	0	0	0	0	0	0
69470	'Tmem127'	9.46	10.59	20.1	3.94	9.17	7.97	4.76	9.99	7.87	6.44
69478	'2300009A05Rik'	11.39	18.56	16.3	24.7	16	6.13	8.99	11.2	40.02	1.48
69479	'1700029J07Rik'	1.42	2.92	4.44	4.52	3.67	1.62	6.93	3.17	9.76	3.73
69480	'Ttc9'	4.4	1.64	3.43	0	0	3.17	4.93	5.99	5.76	0.54
69482	'Nup35'	23.01	10.04	7.06	17.6	0.25	10.65	19.7	21.7	7.02	12.1
69487	'Ndufaf5'	80.68	53.25	71.4	49.9	47.6	62.15	86	40.46	62.71	64.05
69496	'Dydc1'	0	0	0	0	0.11	0	0	0	0.1	0
69499	'Tsr2'	66.8	50.61	68	73.8	46.2	40.02	30.9	74.15	54.51	39.29
69504	'Zfp932'	22.63	15.17	21.3	34.9	17.7	11.81	35.4	20.39	26.22	24.99
69519	'Rwdd2a'	180	204.4	138	162	99.7	126.9	198	282.5	143.1	166.58
69524	'Esam'	2.2	0	0	0	6.67	0	0	0	0	4.92
69527	'Mrps9'	34.78	33.61	21.7	10.9	25.4	36.86	32.1	45.05	39.79	38.98
69528	'1700030J22Rik'	4.41	1.97	6.04	0	4.94	4.55	0.04	1.38	2.61	4.88
69534	'Avpi1'	35.93	44.21	38.9	104	51.5	46.04	35.7	28.24	78.04	11.14
69535	'Ten1'	75.38	44.11	66.3	75.8	70.2	64.4	53.3	59.96	92.68	56.91
69536	'Hemk1'	22.65	27.16	4.53	6.35	9.81	13.57	6.47	16.32	10.79	12.31
69537	'Dnase1l1'	0	0	0	13.9	9.18	0	0.04	2.32	0.27	0
69538	'Antxr1'	1.24	0	0.68	0	0.74	0	1.14	0	0	0.01
69539	'Trnp1'	0	0	1.44	1.81	7.75	6.63	4.48	0	3.25	0.03
69541	'Lyg1'	0.13	0	0	0	0	0	0	0	0	0
69543	'Capns2'	1.98	0	1.08	7.17	0	0.14	0	0	0	0
69544	'Wdr5b'	0	3.52	0.03	0	0	0	9.84	1.78	6.94	0
69546	'Mapk1ip1'	38.25	47.76	35.5	56.9	30.9	15.5	50.7	34.01	47.52	31.56
69547	'Nkpd1'	2.73	3.05	2.48	0	0.04	3.91	1.26	2.17	5.42	1.5
69549	'2310009B15Rik'	20.41	9.81	31.4	27.7	16	42.4	15.5	6.4	26.64	29.58
69550	'Bst2'	0.06	2.24	4.9	27.8	7.3	14.57	0	3.65	9.27	8.67
69551	'2310022B05Rik'	3.37	2.07	0.59	12.7	1.28	0.35	2.05	1.11	3.48	1.95
69553	'Ripor3'	0	0	0	0	2.04	0	0	0.03	0	0
69554	'Klhdc2'	57.38	66.03	46	138	44	55.06	52.7	36.34	69.45	31.77
69556	'Bod1'	9.88	25.65	15	32.1	15.2	17.64	15.8	17.07	19.43	17.26
69562	'Cdk13'	1.6	1.71	1.02	5.73	2.33	1.02	2.83	2.65	1.48	0.9
69563	'Mrln'	0	5.11	0	0	0.3	6.74	0	0	4.99	1.71
69564	'Nmrk2'	0.14	0.13	1	18.4	0.05	0.05	0.12	0.06	0.23	0.16
69568	'Vkorc1l1'	3.85	3.66	7.22	0.33	6.66	0.95	2.87	1.12	5.86	5.5
69572	'Mfsd3'	2.03	4.18	12.7	4.27	8.94	16.6	8.9	11.94	7.44	14.78
69573	'Hilpda'	2.6	18.5	15.5	16.2	31.1	34.08	29.1	17.73	17.56	7.79
69574	'Cmbl'	30.72	61.09	24.6	9.21	3.15	22.04	36.6	58.63	2.95	8
69576	'Smco1'	2.13	3.4	3.97	7.47	5.74	3.65	2.73	1.8	2.89	5.09
69577	'Fastkd3'	9.57	9.59	9.14	0.03	15.9	10.03	20.3	8.52	6.41	0.69
69579	'2310034P14Rik'	0	0	0	0	1.05	0	0	0	0	0
69581	'Rhou'	1.82	3.05	1.89	0.85	6.06	1.19	0	0.59	3.12	1.35
69582	'Plekhm2'	4.56	0.85	0.53	8.45	3.87	4.19	1.61	1.51	7.15	2.32
69585	'Hfe2'	5.41	3.52	4.77	0	5.15	7.15	2.85	3.71	3.19	13.4
69587	'Pcgf3'	5.58	0.61	2.4	7.64	0.26	0	1.05	0.38	0.68	0.44

69590	'Gpx8'	11.64	2.8	0	0	0.32	0.05	1	0	0	1.61
69596	'Ap5s1'	13.13	11.71	12.2	14.1	13.6	23.6	6.15	23.56	14.15	19.24
69597	'Afg3l2'	20.91	24.41	42	28.6	19.7	10.69	27.4	16.72	30.6	6.38
69601	'Dab2ip'	3.29	5.69	3.98	9.91	9.53	6.92	1.44	3.14	2.87	5.81
69605	'Lnpk'	6.33	4.31	4.29	2.33	7.16	3.31	5.17	4.15	6.87	5.08
69606	'Mtfmt'	2.32	12.95	10.4	11.9	13.1	15.71	13.1	18.52	9.25	14.33
69608	'Sec24d'	0.16	13.25	8.59	14.2	2.08	0.17	3.38	7.97	2.95	3.86
69612	'Kansl2'	14.65	8.24	15.9	43.1	16.5	16.42	30.4	9.71	17.16	6.94
69617	'Pitrm1'	19.58	15.55	9.08	0	3.15	13.77	15	13.68	5.8	14.84
69623	'Zfp33b'	4.3	0	0.65	0.03	5.71	0.18	4.56	2.58	0.12	2.9
69627	'Fam89a'	2.87	0.07	0	15.6	0	0	2.41	0.03	0	0
69632	'Arhgef12'	1.81	2.39	1.58	3.28	1.56	2.14	0.7	4.15	4.57	1.87
69634	'Clybl'	23.07	21.6	23.1	25.7	50.4	53.14	48.4	37.34	57.73	22.79
69635	'Dapk1'	6.75	7.57	1.64	2.47	5.83	3.81	1.91	10.76	3.17	4.55
69638	'Enho'	30.26	11.81	23.8	20.6	6.78	41.78	23	18.65	25.56	6.02
69639	'Exosc8'	8.45	26.33	8.43	20	9.06	8.84	5.39	16.07	26.62	23.3
69640	'Fam83g'	0	0	0	0	0.01	0	0	0	0	0
69641	'Wdr20'	3.05	7.16	1.9	7.16	6.64	5.44	3.83	3.33	3.78	4.56
69642	'Mlip'	1.28	2.22	2.25	0.05	0	0.05	0.87	0	1.02	0.01
69654	'Dctn2'	174.8	166.2	169	161	136	192.5	173	147.6	125.7	174.88
69655	'Cd164l2'	0	0.45	2.33	4.69	0	0	0	0	0	10.84
69656	'Pir'	4.75	17.88	1.17	13.2	0	0	4.46	4.74	0.04	6.54
69660	'Tmbim1'	3.12	5.15	2.51	6.45	0.19	21.28	0	5.29	1.96	4.12
69662	'2310061I04Rik'	39.82	33.47	56.1	43.9	50.8	51.11	63.5	56.32	68.67	42.59
69663	'Ddx51'	3.88	2.84	1.95	0	2.36	0	0.41	0	1.28	2.74
69665	'Upk3bl'	0	0	0	0	0	0	3.24	0	0	0
69666	'Psmg4'	33.79	21.02	20.3	43.9	21.3	28.08	4.68	10.77	20.73	20.29
69668	'Ccdc115'	29.33	29.46	34.5	18.4	53.5	22.14	40.2	55.41	32.13	38.12
69671	'Tmem52'	0	0	0	0	2.94	0	0	0.05	0	2.06
69672	'Txndc15'	198.4	182.3	174	132	153	162.2	143	171	157.8	146.15
69674	'Mif4gd'	7.46	7.9	12	13.6	4.23	11.66	6.72	9.03	13.65	3.26
69675	'Pxdn'	1.83	1.96	2.1	9.58	2.35	0.33	4.44	0.46	3.01	0.43
69683	'Emc10'	60.43	53.73	94.8	189	61.1	100.2	77.8	49.39	97.86	45.39
69684	'Aarsd1'	113.6	84.16	108	51.6	86.4	130.2	66.6	106.2	128.5	107.79
69692	'Hddc2'	58.77	70.58	78.4	141	59.7	79.59	37.2	112.6	41.57	78.9
69694	'Tatdn1'	5.93	4.83	4.36	6.72	3.79	12.07	15.5	6.54	10	8.34
69697	'Camsap3'	0.36	0.47	7.28	5.24	2.75	1.81	3.95	2.53	7.1	1.44
69700	'Col22a1'	0	1.22	0	0	0	0	0	0	0.01	0
69702	'Ndufaf1'	18.27	25.74	18.9	8.55	24	21.14	37	30.06	31.55	28.28
69707	'Iqcg'	3.76	3.86	0	0.13	0.45	1.86	0	0.18	0.45	1.31
69709	'Ptrhd1'	6.42	3.98	8.39	6.67	4.67	3.45	4.78	3.17	5.09	4.64
69710	'Arap1'	4.18	1.2	1.93	1.41	0.21	0.57	3.92	2.61	0	4.48
69713	'Pin4'	142.1	85.53	71.1	136	96	106.9	113	67.08	149.8	103.96
69714	'Tfpt'	21.03	28.31	30.6	40.8	28.8	29.17	58.9	26.67	99.63	14.17
69716	'Trip13'	0	3.53	0	0	0	4.62	1.36	0	0.83	0
69717	'Gm10499'	0	0.04	0.15	0.09	0	0	0	0	0.1	0
69718	'Ipmk'	2.57	1.45	1.94	1.88	1.12	0.83	1.67	0.61	0.22	0.55
69719	'Cad'	0.9	1.67	0.98	0.43	0	0	1.3	0.45	0.07	2.36
69721	'Nkiras1'	150.3	137.3	109	217	144	239.1	63.5	149.4	120.7	133.86
69723	'Rpain'	18.93	30.49	22.5	23.7	13.3	9.9	22.6	16.56	14.25	27.15
69724	'Rnaseh2a'	39.69	20.2	21.6	5.63	30.2	16.05	65.6	16.52	6.34	38.13
69726	'Smyd3'	42.4	41.76	38.8	57.7	23.7	73.39	62.8	38.54	72.09	50.58
69727	'Usp46'	16.68	14.45	21.6	10.8	11.8	3.49	35.6	15.38	25.84	17.28
69731	'Gemin7'	139.8	78.65	115	118	117	95.86	138	89.88	102.4	40.09
69736	'Nup37'	8.19	2.59	1.27	4.03	8.37	4.37	10.8	5.96	1.14	4.87
69737	'Ttl'	7.82	1.65	6.33	11.8	5.82	1.32	6.51	7.93	4.57	5.06
69740	'Dph5'	33.39	23.13	20.5	46.3	28.5	23.43	20.3	35.38	34.64	19.14
69742	'Tm2d2'	204	160.3	224	222	145	214	145	210	195.2	173.96

69743	'Casz1'	0.43	0.89	0.71	1.23	2.19	1.36	1.22	0.87	1.27	0.33
69745	'Pold4'	20.78	51.79	12.3	19.7	33.6	53.31	21.6	35.1	29.1	47.58
69747	'Zswim7'	12.31	9.61	10.3	29.5	21.1	22.93	19.2	22.24	32.54	42.19
69748	'Aldh16a1'	1.13	1.82	12.6	0	5	6.47	0.04	2.65	4.66	9.78
69752	'Zfp511'	21.39	17.07	17.6	15	12.7	43.36	13.2	23.26	13.67	34.66
69754	'Fbxo7'	23.23	33.04	40.9	22.4	24	52.99	65.3	29.89	22.09	32.94
69757	'Leng1'	9.61	5.06	8.43	8.73	5.69	16.87	4.06	13.5	3.62	2.96
69761	'1600015I10Rik'	0	1.04	0	0	0	0	0	0	0	0
69769	'Tnfaip8l2'	3.89	0	0.66	0	0	16.45	0	0	0	0
69770	'1600002K03Rik'	25.24	2.54	2.3	0	8.68	8.24	4.55	8.78	7.09	3.76
69772	'Bdh2'	2.2	0	9.72	0	0	5.98	0	2.75	11.56	3.85
69773	'Timm29'	19.73	8.05	8.54	30.4	12.4	4.84	15.7	5.66	38.46	8.67
69780	'Smap2'	20.26	12.05	14.9	10.7	13.4	31.08	13	11.78	11.97	11.26
69784	'1500009L16Rik'	74.87	128.4	45.2	119	57.3	71	23.4	85.81	53.92	37.75
69786	'Tprkb'	45.08	40.11	31.9	25.4	56	29.34	73.8	26.02	35.05	27.08
69787	'Anxa13'	0	0.25	0	0	0	0	0	0	0	0
69790	'Med30'	37.56	37.67	59.5	30.3	38.7	31	70.7	29.28	27.74	18.01
69792	'Med6'	23.36	24.83	11.7	41.7	14.5	24.66	20.6	36.89	32.58	41.3
69802	'Cox11'	4.02	4.14	2.54	14.6	6.2	0	2.41	0.07	1.86	1.82
69804	'Tmem147'	255.1	118.8	258	238	272	288.7	228	267.8	292.5	258.21
69806	'Slc39a11'	14.26	18.89	15.9	18.3	22.9	13.24	19.1	11.83	19.88	54.52
69807	'Trim32'	88.74	91.52	70.6	38.9	71.1	59.97	136	109	65.05	71.37
69814	'Prss32'	0	0	0	0	0	0	0	0	0.06	0
69815	'Krtcap3'	12.99	3.49	1.84	9.14	14.6	0	0	0.74	2.99	7.68
69821	'Mterf4'	16.16	12.93	12.1	24.1	9.24	33.31	17.2	10.97	7.11	0.75
69823	'Fyttd1'	53.32	52.93	56.6	82.9	50.8	27.67	48.1	53.56	69.19	47.8
69824	'Glod5'	0	0	6.98	0	0	0	0	0	4.28	3.97
69833	'Polr2f'	154.5	128.5	152	174	116	120.6	110	119.9	147.6	134.78
69834	'Rab43'	0.36	1.31	2.06	0.19	0.05	0.09	0	0.62	0.36	0.03
69837	'Pcgf1'	30.48	30.69	10.3	12.2	61.6	56.81	30.7	37.21	43.71	37.94
69847	'Wnk4'	0	0.07	0.58	0	0.88	0.18	0.57	0.13	0	0.04
69852	'Tcf23'	0	0.01	0	0	0	0	0	0	0	0
69860	'Eif1ad'	5.18	12.26	29.2	26.2	15.2	20.37	25.8	30.2	33.28	12.17
69863	'Ttc39b'	1.3	0.77	2.79	0.86	1.35	0.15	2.4	1.9	0.01	2.14
69865	'A1cf'	0	0.03	0.01	0	0	0.01	0	0	0.01	0
69870	'Polr3gl'	22.82	23.79	24	38.7	52.6	37.59	39.3	20.55	39.1	46.85
69871	'Ppp1r35'	12.16	8.59	12.4	29.1	13.4	3.31	8.49	8.52	9.51	4.89
69875	'Ndufa11'	109.3	123.9	157	174	145	151.6	164	119.7	113.6	119.96
69876	'Thap3'	28.12	36.74	19.1	42.7	35.6	50.9	26.8	17.91	26.87	53.1
69878	'Snrpf'	51.22	56.64	66.3	55.2	43.3	79.07	67.1	36.9	74.81	71.25
69882	'Ints14'	10.21	15.56	3.92	8.2	7.6	7.11	1.58	11.79	4.32	3
69885	'Aunip'	0	1.51	0	0	0	0	0	0	0	0
69890	'Zfp219'	10.58	12.97	18.7	10.5	13.7	1.57	12.5	11.1	14.35	11.08
69893	'Coa7'	8.51	8.53	2.9	15.7	8.42	12.16	7.73	19.43	18.31	3.42
69894	'Fam241b'	101.6	112.7	84	60	112	112.1	97.7	89.47	117.7	136.68
69900	'Mfsd11'	29.91	31.99	12.7	27.8	14.6	15.45	22.3	33.12	23.8	27.8
69902	'Mrto4'	64.05	39.21	74.4	20.9	53.2	69.95	36.5	54.55	37.05	27.23
69903	'Rasip1'	2.96	1.51	4.8	1.39	1.05	2.29	1.49	2.64	1.65	5.77
69906	'Slc25a32'	1.92	1.86	0.85	2.24	4.22	0.02	9.54	1.39	4.78	0.23
69908	'Rab3b'	90.37	198	65.3	82.7	268	158.3	40.8	27.78	136.6	228.2
69912	'Nup43'	4.68	0.61	16.2	11.8	21.9	8.22	19.2	5.08	6.97	26.51
69917	'Nabp2'	120.6	118.2	87.4	103	66.1	126.4	111	133.4	111.1	96.77
69920	'Polr2i'	50.05	49.66	51.6	42	31.7	58.04	56.2	25.95	38.35	30.19
69922	'Vrk2'	0.03	2.42	0	0	0	0	0	0	0	0
69923	'Agk'	41.9	26.94	30.8	19.4	49.1	35.59	61.5	48.96	53.85	23.75
69926	'Dnah17'	0	0.27	0	0.01	0	0.25	0	0	0.43	0
69928	'Cenps'	6.09	2.04	5.66	15.3	11.4	5.56	0.06	9.81	8.93	14.81
69930	'Zfp715'	3.48	4.99	0.5	11	2.84	0.96	10.1	12.85	10.03	7.26

69934	'Trmt10b'	21.13	17.43	13.9	0.04	15.9	18	9.58	23.48	21.03	19.65
69938	'Scrn1'	80.95	74.23	90.6	94.1	63.6	30.24	73.7	77.94	104.4	118.94
69940	'Exoc1'	11.49	14.35	29.8	12.8	15	9.9	17.9	22.7	30.18	15.53
69942	'Rnf113a1'	8.51	25.02	7.62	0	9.2	8	21.5	15.34	14.54	14.15
69944	'2810021J22Rik'	1.65	0.11	2.7	0.05	4.72	4.5	3.39	3.75	3.67	2.4
69955	'Fars2'	20.47	19.65	9.1	11.9	21.3	23.44	13	20.16	13.93	28.56
69956	'Ptcd3'	41.59	49.24	45.1	31.8	40.9	32.12	49.9	32.83	33.56	61.42
69957	'Cdc16'	54.96	21.41	48.3	76.1	30.6	5.87	54.6	32.1	54.63	7.38
69961	'Rpp25l'	26.83	49.3	29.2	51.7	28.7	29.3	46.1	38.65	53.53	52.32
69962	'Mettl18'	8	8.35	4.32	0	3.74	1.91	5.78	1.67	8.52	5.97
69976	'Galk2'	34.85	52.26	57.2	32.9	44.9	56.45	21.1	43.38	37.28	50.6
69981	'Tmem30a'	88.98	51.54	83.2	117	55.4	26.96	83.7	56.03	89.29	41.11
69987	'Spaca9'	5.25	0.08	0.08	0	0	8.92	6.39	7.21	0	1.58
69993	'Chn2'	20.47	2.46	5.02	0	4.73	9.55	17.1	2.3	38.06	12.47
69994	'Rsc1a1'	0	0	4.4	0	0	0.04	1.73	1.04	0.06	3.79
70009	'Ssty2'	0	0	0	0	0	0	0	0	0.12	0
70012	'Cep85'	2.33	1.5	5.21	0	4.3	0.44	0.4	6.58	0	2.23
70020	'Ino80b'	0.09	1.1	0	2.22	0	2.92	0	1.72	4.16	2.85
70021	'Nt5dc2'	0	0	0.03	0.04	2.02	0	6.46	0.03	0	0
70024	'Mcm10'	0.02	0.02	0.05	0.02	0	0	0	0.03	0.04	0.63
70025	'Acot7'	691.9	565.4	546	588	571	735.9	629	570.8	521.6	640.45
70028	'Dop1b'	11.13	4.97	15.8	8.34	3.99	3.7	6.04	2.08	8.45	4.59
70031	'Cmtm8'	0.64	12.85	0.06	0.8	3.01	0	0.05	1.56	0.11	6.63
70044	'Tut1'	13.79	12.23	12.9	12.6	24.1	10.46	8.46	18.51	16.05	18.65
70045	'2610528A11Rik'	0	0.07	0	0	0	0	0	0	3.94	4.51
70047	'Trnt1'	24.97	50.99	18.6	35.5	26.1	23.23	34.4	39.4	28.12	25.45
70052	'Prpf4'	8.39	12.4	5.64	0.7	11.7	13.71	9.15	5.6	10.44	4.1
70054	'Ccdc89'	0	2.2	0	0	0	0.54	0	0	3.05	2.07
70059	'Degs2'	0	1.2	0.27	2.18	4.73	8.38	0	0.47	2.56	1.93
70062	'Ctag2'	0	0	0	0	0	0	0	0.05	0	0
70078	'Nol7'	23.55	19.68	25.9	18.7	9.44	20.88	19.5	18.47	17.27	6.12
70080	'Igsf23'	0	0	0	0	0	0.02	0	0	0.34	0
70081	'Zfp995'	1.15	1.29	9.92	3.76	0.26	1.35	4.73	4.01	12.08	2.32
70082	'Lysmd2'	88.04	51.02	43	80.1	38	39.53	42.3	54.23	60.43	38.35
70083	'Metrn'	16.01	10.58	9.55	1.28	20.3	6.18	1.46	6.68	3.53	0.7
70088	'Meaf6'	80.21	79.19	77.2	38.2	72.5	68.73	78.8	64	82.83	92.67
70093	'Ube2q1'	6.5	9.57	2.94	7.23	8.02	1.52	12.4	3.81	4.14	6.65
70097	'Sash1'	0.38	0.14	0.35	0.62	0.38	1.09	0.32	0.08	0.65	0.34
70099	'Smc4'	1.04	0.68	0.5	0.01	0.05	1.36	2.73	0.62	0.98	0.71
70101	'Cyp4f16'	2.4	0	7.21	8.9	2.79	0	0	0	4.15	3.26
70103	'Znhit1'	169.6	117	92.3	166	193	177.4	114	176.5	164.6	140.58
70110	'Ifi35'	1.52	2.98	0	42.9	4.82	0	0.07	0.03	0	2.81
70113	'Odf3b'	5.42	2.48	10.5	0	6.52	0	10.3	0.04	4.81	2.38
70118	'Srrd'	0	1.47	6.45	4.37	14.1	11.47	6.15	4.73	0.21	0
70120	'Yars2'	9.01	4.4	0.67	8.82	5.27	17.9	0	4.95	5.66	7.66
70122	'Mllt3'	2.98	1.08	4.26	0.07	3.19	3	8.26	3.01	7.95	1.68
70123	'Nbdy'	136.9	171.2	169	238	204	312.3	221	170.9	143.9	231.32
70127	'Dpf3'	2.91	0.26	1.8	0	0	0.31	0.19	0	6.82	0.77
70129	'Slc44a4'	0	0	1.34	0	4.29	0.02	0	0	0	0
70134	'2210011C24Rik'	0	0	3.43	0	2.38	0	3.34	0	0	0
70144	'Lrch3'	10.36	6.5	11.8	11.4	8.17	1.38	10.9	5.89	7.54	7.38
70152	'Mettl7a1'	4.13	14.24	9	7.5	7.12	13.86	7.18	8.77	9.41	10.99
70153	'2210016F16Rik'	26.67	16.55	15.8	22.7	11.2	27.38	8.87	35.73	35.3	31.98
70155	'Ogfrl1'	5.25	3.08	2.8	8.08	1.51	2.62	3.52	1.31	6.18	0.43
70160	'Vps36'	9.95	7.14	6.31	21.8	18.7	48.35	10.7	12.74	12.27	7.44
70178	'Abhd17c'	2.1	0	2.53	6.55	0	5.49	0	0.02	0	0
70186	'Fam162a'	72.97	60.96	95.6	61.9	65.1	51.95	118	89.99	106.2	83.27
70190	'2610036A22Rik'	0	0.02	0.05	0	0.45	0	0	0	0	0.6

70207	'Taco1'	6.27	5.36	0.96	0	2.85	6.02	2.07	1.33	0	1.11
70208	'Med23'	9.52	14.11	3.4	3.25	9.26	1.88	9.83	13.36	4.48	6.31
70209	'Tmem143'	3.03	0	5.93	0	2.56	2.29	5.12	6.88	3.74	3.52
70223	'Nars'	142.1	151.5	211	163	148	201	146	209.4	212.5	157.32
70225	'Ppil3'	53.35	35.75	23.8	44.4	51.1	61.52	13.5	54.46	27.54	25.12
70227	'Zfp619'	0	1.18	0.01	2.45	0	1.05	0	0.15	0	0
70231	'Gorasp2'	74.88	107.5	87.8	154	78.3	93.16	127	109.7	84.9	87.18
70233	'Cd2bp2'	24.44	30.17	32.7	27.1	40.2	33.45	38.8	19.62	35.8	29.16
70235	'Poc1a'	0	0.37	5.36	0	4.82	0	1.68	8.14	11.3	8.43
70237	'Bhlhb9'	56.58	56.19	32.6	40.4	34.5	27.21	56.7	46.07	26.49	43.53
70238	'Rnf168'	6.19	4.26	5	6.01	6.37	4.13	5.31	5.74	8.08	2.21
70239	'Gtf3c5'	19.02	28.33	13.6	25.5	12.9	35.4	14.7	10.4	29.78	20.28
70240	'Ufsp1'	36.03	16.74	23.8	10.3	15.6	27.51	30	34.66	33.94	7.83
70247	'Psmd1'	143.5	108.4	112	114	75	103	146	142.8	112.9	132.54
70248	'Dazap1'	4.17	3.49	3.4	1.87	6.18	0.35	0.51	1.73	5.64	4.64
70257	'Atp5mpl'	304	367.7	381	464	332	591.8	323	226.1	354.2	289.72
70261	'Chp2'	0	0	1.52	0	0	0	0	0	0	0
70266	'Kyat1'	18.31	6.41	14.7	8.74	10.7	34.5	5.49	7.22	7.93	15.21
70274	'Ly6g6e'	0.15	0	0	0	0	0	0	0	0	0
70285	'Rpf1'	9.05	9.32	17.6	40.7	6.46	28.86	9.56	13.25	6.13	17.67
70291	'Mkrn2os'	0	0	0.02	1.45	0	0	0	0	0	0
70292	'Afap1'	4.64	3.62	2.17	5.04	3.08	0.83	2.65	4.04	4.02	2.92
70294	'Rnf126'	11.98	9.25	13.5	21	11.7	8.64	19.7	9.11	26.96	5.33
70296	'Tbc1d13'	0.92	0.75	1.37	5.74	1.37	1.97	2.1	0.21	1.5	0
70297	'Gcc2'	13.78	6.36	9.06	14.6	11.6	12	8.42	6.16	11.38	11.81
70300	'Fuz'	41.06	30.39	17.5	23.1	10.4	43.31	9.96	51.88	47.53	38.92
70310	'Plscr3'	5.26	10.81	14.5	0	16.8	5.94	3.07	14.46	12.69	20.42
70312	'Cactin'	13.18	3.45	2.05	17.2	5.01	5.58	11.2	12.16	10.07	2.23
70314	'Rabep2'	9.56	6.33	8.32	9.69	13.6	14.3	9.95	10.89	24.85	7.29
70315	'Hdac8'	5.11	16.62	10.3	16.5	3.49	33.64	20.6	8.77	9.43	25.32
70316	'Ndufab1'	467.3	428.7	432	362	405	616.2	512	528.3	611	574.36
70317	'Arl16'	47.58	51.07	28.4	27.6	30.1	38.72	34.9	42.19	51.78	37.87
70325	'Pigw'	2.37	0	0	0	0.07	0	0	0	0	0
70333	'Cd3eap'	14.84	9.43	14.1	14.1	14.6	24.33	17.2	12.87	15.53	12.88
70335	'Reep6'	17.67	4.18	7.28	0.03	8.89	6.8	0.05	4.77	2.79	7.01
70348	'Ube2cbp'	2.62	5.83	8.76	0	0	2.54	4.22	4.66	0	2.79
70349	'Copb1'	50.11	36.28	37.1	43.7	24.5	33.09	26.3	48.9	44.8	55.99
70350	'Basp1'	32.09	36.43	39.2	51.2	21.6	30.78	38	18.25	10.24	24.04
70351	'Ppp4r1'	4.27	7.02	5	1.36	3.45	0.64	6.17	4.93	9.93	10.51
70354	'Secisbp2l'	6.24	5.45	6.9	1.12	5.79	1.84	2.25	4.7	6.78	1
70356	'St13'	61.74	40.51	51.3	13.5	40.3	35.13	60.1	43.77	71.82	58.82
70357	'Kcnip1'	75.92	67.97	53.7	31.8	22.7	74.55	62.2	88.05	34.33	5.27
70359	'Gtpbp3'	5.45	8.67	11.1	6.45	0.84	16.63	4.55	2.74	2.15	3.54
70361	'Lman1'	15.54	22.97	12.7	16.8	12.3	7.7	8.7	9.36	25.65	25.11
70362	'Actl10'	0	1.23	0	0	0	0	0	0	0	0
70363	'Fam135b'	5.1	5.23	4.33	3.52	3.98	5.12	3.67	4.48	4.43	5.73
70369	'Bag5'	42.37	60.4	42.2	12.3	41.8	34.58	31.3	35.94	40.75	42.1
70370	'Fbln7'	0	0	0	0	0.44	0	0	2.1	0	0
70373	'Gpatch2l'	12.11	14.54	8.34	7.6	8.98	5.22	9.04	12.76	9.16	18.03
70375	'Ica1l'	2.41	15.21	10.9	29.9	5.24	2.2	4.68	11.81	11.48	7.6
70377	'Derl3'	2.14	0	0	0	0	0	0	0	0	0.04
70380	'Mospd1'	2.33	6.6	18	25.5	12.8	20.2	17.7	10.33	13.45	10.75
70381	'Tecpr1'	20.1	15.6	19.3	4.73	20	7.67	25.6	16.54	15.2	14.13
70382	'Kctd2'	0.03	4.2	3.4	8.17	12.5	11.69	15.2	10.25	6.67	12.26
70383	'Cox10'	8.81	9.16	12.1	11.4	15.4	2.16	12.5	12.42	4.48	8.78
70385	'Spdl1'	0	0	1.79	0	0.08	0	0	0.67	0	2.7
70387	'Ttc9c'	11.37	13.39	8.63	11.7	4.91	1.96	12.5	14.69	13.5	8.12
70392	'Asb12'	0	0	0	0	0	0	0	0	0.03	0

70394	'Kptn'	35.44	26.8	30.8	41.4	34.5	35.65	25.9	10.52	10.48	29.48
70396	'Asnsd1'	10.1	12.36	7	21.6	9.22	16.79	3.55	11.34	3.98	27.75
70397	'Tmem70'	47.86	65.58	46	31.8	38.4	59.39	34.2	60.71	43.48	67.84
70408	'Polr3f'	4.39	1.02	0.43	4.4	5.68	0.3	3.02	0.5	5.71	2.67
70415	'Stk26'	2.66	3.94	1.35	0	2.63	2.84	0.89	1.19	1.67	3.24
70417	'Megf10'	1.06	0.06	0	2.57	2.69	0	0.01	0	0	0.27
70420	'Arpin'	14.52	8.5	6.44	18	6.35	12.67	18.2	4.75	6.27	10.87
70422	'Ints2'	4.17	3.48	1.41	4.62	1.4	0.93	2.42	3.13	1.22	2.75
70423	'Tspan15'	15.62	4.86	10.6	4.5	6.82	0.14	13.2	6.75	0.18	0.06
70425	'Csnk1g3'	1.99	3.18	1.31	4.84	6.61	2.17	5.25	1.38	0.64	2.35
70426	'Tekt5'	6	13.75	7.34	0	9.71	0	6.05	34.87	7.37	2.39
70427	'Mier2'	7.67	11.7	17.4	7.19	3.67	2.84	13	8.87	19.41	9.33
70428	'Polr3b'	5.14	5.65	3.33	6.81	1.03	2.19	0.18	3.07	6.57	1.49
70430	'Tbce'	47.48	53.97	41.5	48.5	61.4	58.98	42.6	71.08	61.84	30.36
70432	'Rufy2'	21.98	24.35	36.1	17.9	36.2	34.87	11.6	31.99	54.43	32.67
70433	'Draxin'	0	0	0	0	0	0	0	0	0	0.13
70435	'Inf2'	0.84	1.5	0.8	0.05	1.36	3.4	0.04	0.05	0.75	0.01
70439	'Taf15'	17.1	6.72	7.02	3.05	16	13.81	1.4	16.93	9.9	16.5
70445	'Cd248'	0	0	0	0	1.39	0.02	2.31	0	0	0
70450	'Unc13d'	0	0.94	0	0	0	0	0	0	0	0.35
70451	'Dhrs13'	13.16	15.8	8.33	0.36	9.69	6.14	15.3	18.52	6.96	9.47
70454	'Cenpl'	0	0.48	0	0.07	0	0.02	0	0	0	0.46
70456	'Mpc2'	219.9	172.2	250	202	253	172.1	196	163.7	219.3	168.19
70458	'2610318N02Rik'	3.92	2.36	11.1	0	4.02	0	0	1.83	6.31	7.73
70461	'Crtc3'	2.86	2.47	1.61	6.96	2.79	3.97	2.38	1.03	1.48	2.5
70465	'Wdr77'	39.64	23.06	19.5	18.6	43.8	29.24	19.1	31.96	47.13	32.72
70470	'Rprd1b'	8.36	6.91	12.5	16.3	16.2	5.21	22.1	6.29	11.59	6.07
70472	'Atad2'	1.99	0.75	2.45	0.18	0.69	1.52	1.98	0.07	0.13	3.09
70478	'Mipep'	1.11	1.37	2.51	3.58	3.53	0.68	5.33	4.08	3.73	0.02
70481	'Pnma1'	12.52	11.81	23.5	22.2	18.7	7.3	13.3	16.82	3.19	9.82
70484	'Slc35d2'	0.02	0.04	0.18	0.18	4.18	0.04	0.3	0.17	0.26	0.15
70495	'Atp6ap2'	262.4	372.9	313	274	228	298.6	247	327.6	245.8	355.96
70497	'Arhgap17'	0.02	4.52	1.11	0	0.5	1.84	0	2.23	2.51	0.55
70503	'Ddo'	12.72	13.68	1.85	3.62	12.2	13.15	27.5	13.06	3.06	19.34
70508	'Bbx'	9.47	10.95	11.2	11.9	5.57	3.25	13.2	5.51	10.13	18.54
70510	'Rnf167'	84.17	78.92	117	79.7	80.2	113.2	127	89.82	98.4	88.07
70511	'Eef2kmt'	1.64	2.93	2.11	0	6.24	0.05	0.93	4.74	5.52	3.28
70527	'Stambp'	41.84	44.66	47	45.6	31.7	39.15	37.2	49.52	47.52	41.82
70530	'Lrfn2'	5.31	4.27	11.7	0.79	3.59	2.59	3.96	9.57	8.67	0
70533	'Btf3l4'	41.75	35.04	34.2	14.1	37.8	37.76	26.1	50	27.6	40.74
70536	'Qpct'	34.55	74.46	44.9	48.1	31.6	50.48	33.3	47.83	57.19	69.48
70544	'Tmem242'	118.2	55.34	99.8	145	98.8	134.4	93.2	112.9	112.7	103.15
70546	'Zdhhc2'	9.58	4.71	5.41	15.2	11.2	6.06	6.57	6.9	5.45	5.54
70549	'Tln2'	4.19	3.82	3.32	1.3	2.84	4.38	5.11	3	2.52	3.14
70551	'Tmtc4'	4.68	4.68	8.44	1.43	4.49	1.49	6.85	7.56	5.44	5.8
70552	'Lrrc56'	8.35	0.71	1.71	2.37	6.39	0.02	0	2.09	0	3.11
70556	'Slc25a33'	3.56	16.78	4.48	2.56	5.42	6.67	7.33	2.04	13.9	0.07
70560	'Wars2'	1.89	1.72	0.03	0.05	3.07	5.9	0	3.1	0.93	3.36
70561	'Txndc16'	18.57	24.15	24.7	29.6	11.6	16.98	24	19.73	25.55	21.01
70564	'Fam213a'	155	113.2	110	141	125	151.7	106	121.3	142.8	132.58
70567	'Fra10ac1'	30.64	19.55	33.6	17	24.5	20.49	26.7	16.17	24.89	8.87
70568	'Cpne3'	1.53	2.04	4.54	0	3.23	0.8	0.49	1.29	2	1.35
70571	'Tcerg1l'	1.93	9.76	1.88	18.3	1.52	3.18	6.12	6.56	0	2.08
70572	'Ipo5'	19.32	25.41	23.5	41.5	14	5.94	9.84	18.18	6.87	15.1
70573	'Tbccd1'	5.13	14.42	5.97	2.95	4.7	7.71	5.92	2.4	12.03	5.82
70574	'Cpm'	0.34	0.28	2.22	1.43	1.07	2.36	0	0.76	0.12	1.07
70575	'Gfod2'	45.55	39.33	27	9.81	38.5	24.81	23.2	39.93	42.54	23.35
70579	'Zc3h11a'	31.87	24.86	19.7	26	23	10.51	27.8	26.6	19.36	33.7

70584	'Pak4'	3.15	4.01	0.72	0	1.72	2.42	0	7.66	2.44	0
70591	'5730455P16Rik'	34.22	33.43	24.7	13.7	34.8	30.86	47.7	31.97	35.75	35.34
70598	'Filip1'	0.01	1.07	0	1.96	0	1.04	0.9	3.46	0	0
70599	'Itprid2'	0.89	0.92	0.01	5.69	0.3	2.24	0.47	1.75	1.58	1.51
70601	'Ecd'	39.91	34.85	19.8	37.6	34.2	39.37	25.7	34.89	19.05	52.11
70603	'Mutyh'	3.34	1.77	0	0	5.38	0	0	1.4	0.58	0.25
70604	'Dnajb14'	15.62	15.23	14	30.1	13.3	22.17	18.7	14.41	12.16	17.69
70605	'Zdhhc24'	18.38	6.69	7.09	0	9.79	16	24.3	12.15	0.25	6.93
70611	'Fbxo33'	3.52	6.56	2.86	13.7	8.67	6.35	3.27	4.02	5.94	3.96
70612	'Tmem230'	33.99	37.7	27.4	32.8	45.5	49.31	73.5	30.65	41.74	36.53
70615	'Ankrd24'	9.35	8.44	14	13.9	9.48	12.35	8.05	9.37	17.51	14.41
70616	'Sugp1'	25.41	39.33	39.6	39.2	48.6	11.6	36.9	18.24	38.81	31.62
70617	'Fam241a'	0	0	0	0	0	0	0	0.81	0	0
70620	'Ube2v2'	47.34	45.5	47.6	45.2	35.5	47.92	45.9	50.46	47.63	51.43
70625	'Med26'	3.31	11.79	4.92	14.3	3.24	4.23	8.76	2.13	7.97	3.27
70638	'Fam189a1'	0.01	0.93	6.17	2.52	2.65	0.02	0.01	0.77	6.81	2.66
70640	'Dcp2'	0.95	2.15	1.96	3.57	1.95	0.68	0.68	0.58	1.19	0.14
70645	'Oip5'	0	0	1.5	0	0	0	0	0	0	0
70646	'Naa30'	1.09	0.47	1.03	0.32	1.06	0	0.01	1.79	0.09	0.04
70650	'Zcchc8'	3.54	13.92	5.06	6.35	2.69	7.62	10.7	8.2	4.98	3.39
70652	'Tmem144'	2.14	1.99	3.58	0.03	2.64	3.42	5.44	0	1.06	7.55
70661	'Sik3'	3.67	3.65	3.53	5.47	3.16	6.01	2.51	3.15	2.2	0.8
70673	'Prdm16'	3.05	5.06	4.34	2.19	0.83	1.49	3.55	3.68	0.04	3.12
70675	'Vcpip1'	5.71	3.54	6.32	2.88	3.59	6.06	2.17	4.29	2.86	4.99
70676	'Gulp1'	1.22	1.42	1.19	0	1.08	0	0.05	1.33	3.86	2.86
70681	'Abraxas1'	4.15	3.54	2.85	5.07	2.02	7.51	0.08	5.12	4.39	5.95
70683	'Utp20'	0.34	0.95	0.5	2.48	0.32	0.02	0.69	1.35	0.5	1.18
70686	'Dusp16'	0.2	0.68	5.49	3.31	0.5	0.05	2.01	1.42	1.54	4.47
70691	'3830403N18Rik'	0	0.44	0	0.12	0	0	0	0.04	0	0
70693	'Adgra3'	1.86	1.16	2.1	4.56	0.01	0	0	0.01	3.05	0.1
70699	'Nup205'	4.29	1.65	1.01	13.1	3.11	4.07	5.34	0.52	1.86	1.88
70713	'Gpr137c'	4.3	2.31	8.24	0.31	3.56	3.04	3.97	5.73	1.64	1.39
70717	'Medag'	7.75	10.52	0.63	0.07	2.08	1.64	11.2	5.35	4.03	8.24
70719	'Arhgap45'	1.67	0	0.02	0	0	0.95	0	0	0	0
70726	'Angptl6'	12.77	19.92	15.3	14.4	20.1	8.4	12.1	13.74	13.84	27.51
70727	'Rasgef1a'	14.43	20.02	11	39.1	36.3	18.78	15.6	15.08	22.21	25.68
70729	'Nos1ap'	22.48	11.23	16.4	26.2	7.51	4.37	19	4.96	5.46	1.85
70730	'6330409D20Rik'	0.32	0	0.22	0	3.62	2.74	0	1.7	0	0
70737	'Cgn'	10.67	8.3	14.2	18.8	8.31	8.21	5.75	9.33	21.61	12.44
70747	'Tspan2'	11.07	6.32	8.51	5.01	6.83	6.98	9.03	6.55	10.26	4.04
70750	'Kdsr'	7.28	13.91	20.6	6.04	11.1	8.12	21.1	11.76	19.99	14.21
70757	'Hacd2'	9.02	5.92	4.33	17.5	4.67	1.63	6.99	4.71	5.77	0.05
70762	'Dclk2'	7.66	5.34	12.4	11.4	4.89	3.73	12.4	3.32	9.74	1.8
70767	'Prpf3'	21.75	11.64	5.47	16.4	16.2	7.34	20.2	3.1	4.68	7.24
70769	'Nolc1'	28.7	22.75	27.8	38.7	27.2	26.76	37.3	20.24	26.69	23.7
70771	'Gpr173'	0.35	0	0.01	0.41	1.67	0.27	2.27	0.49	0.57	2.1
70772	'Ggnbp1'	0	1.58	0	10.3	0	0.35	0	1.6	0	0.19
70779	'Prdm5'	3.06	2.86	0.07	0	0	1.23	0	0.53	0.98	1.87
70784	'Rasl12'	0.22	0.07	2.29	0	3.11	0	0	3.22	0.02	0
70785	'Dennd1c'	0	0	1.9	0.02	0	2.47	1	0.34	0.66	0.78
70788	'Klhl30'	0	0.02	0	0.02	0	0.01	0	0	0	0.03
70789	'Kynu'	2.14	0	0	0	0	4.43	0	0	0	2.6
70790	'Ubr5'	5.65	4.58	1.06	15.6	4.98	2.44	2.04	2.78	1.4	5.19
70791	'Hars2'	19.67	19.44	10.7	9.54	18.4	14.26	12	2.37	17.55	31.57
70796	'Zdhhc1'	26.08	24.25	38.7	30.4	15.4	16.38	31.7	7.7	23.12	11.1
70797	'Ankib1'	0.97	2.31	2.42	3	3.61	1.01	2.65	0.87	5.25	0.82
70799	'Cep192'	0.11	0.67	0.42	2.25	0.89	0.01	0	1.18	0.55	0.24
70802	'Pwwp2a'	5.43	3.46	5.48	9.19	6.73	2.83	4.52	2.38	3.51	5.44

70804	'Pgrmc2'	6.52	6.92	7.19	12.3	3.46	4.88	15.4	6.78	7.22	3.91
70807	'Arrdc2'	0.17	6.07	3.05	2.14	3.83	1.95	2.19	5.09	1.9	3.75
70810	'Krt25'	0.03	0.68	0.08	0	5.32	7.63	0	1.37	0	0
70821	'4921507P07Rik'	2.74	0.03	0.79	0	2.1	1.83	5.27	0	0.07	2.6
70823	'Hmgxb4'	1	2.07	2.29	3.24	5.69	3.26	1.3	4.33	4.13	1.44
70827	'Trak2'	0.26	1.78	2.32	2.67	9.78	0.03	0.85	1.33	0.7	1.84
70829	'Ccdc93'	2.29	2.69	2.03	1.74	0.01	0.06	1.26	0.67	1.08	0.67
70834	'Spag9'	14.39	15.34	14.7	15.1	14.5	11.06	6.28	10.44	10.85	9.88
70835	'Prss22'	0	0	0	0	0	0	0	0	0.1	0
70839	'P2ry12'	8.57	6.89	0.38	18.9	9.39	31.74	0	6.14	0	0
70843	'Krt28'	0	0	0	0	0	0	0	0	0.5	0
70853	'Vwa3b'	0.38	2.77	0.72	1.93	0	1.14	1.47	0.06	0.3	2.17
70859	'Lrrc63'	0	0.05	0	0	0.13	0	0	0.04	0.02	0
70866	'Slco6d1'	0	0	0	0	0	0	0	0	0	1.35
70873	'Cnbd2'	8.31	5.41	9.76	5.55	5.62	14.05	3.16	4.4	5.11	12.36
70881	'Nt5c1b'	0	0.29	0	0	0.27	0	0	0	0	0
70882	'Armc3'	0	0	1.5	0.02	0	0	0	2.31	0	1.24
70884	'Ccdc81'	2.61	0.14	0.09	0.13	0	0	0	0.09	1.73	0.02
70885	'Ints10'	19.81	20.53	24.5	13.7	9.01	9.35	11	10.1	17.81	20.46
70887	'Dmrtc1a'	39.7	61.21	39.4	54.4	15.2	21.3	7.62	32.95	15.87	15.43
70891	'Spdya'	4.47	6.13	14.9	13.5	5.54	22.42	6.99	1.33	6.39	3.05
70892	'Ttll7'	11.05	5.99	8.96	8.96	20.1	23.26	11.5	7.13	13.67	7.8
70894	'Efcab3'	0	0	0	0	0	0	0	0	0	0.15
70896	'Speer1'	0	0	0.04	0	0	0	0.63	0	0	0
70897	'Fam71d'	0.02	0.18	0.12	0.08	0	0.04	0	0.86	0.04	0.12
70900	'4921517D22Rik'	0	0.03	0	0	0.02	0.01	0	0	0	0
70901	'4921524L21Rik'	0.18	0.26	0.22	0.41	0.1	0.2	0.29	0.09	0.04	0.12
70909	'4921504E06Rik'	0	0.05	0	0	0	0	0	0	0	0
70911	'Phyhipl'	308.8	377.8	221	396	210	278.3	311	357.6	199.8	145.66
70918	'Nsun7'	0.45	2.32	1.68	9.02	6.36	4.09	1.8	2.46	4.7	6.86
70920	'Tex47'	0.02	0	0.05	0	0	0.23	0	0	0	0
70925	'Cdkn2aip'	6.74	12.25	6.81	31	3.66	1.55	3.32	8.46	7.42	8.8
70930	'Nol8'	3.56	5.14	1.28	2.16	0.87	4.47	3.16	3.35	2.09	4.53
70945	'Mmrn1'	0	0.35	0	0	0	0	0	0	0	0
70948	'Wdr20rt'	0	0	0.02	0	0	0	0	0	0	0
70951	'Spata1'	0	0.03	0	0	2.5	0	0.6	0	4.23	1.45
70967	'Eva1c'	0	2.65	5.82	0.7	0	0	0.04	1.31	0	0
70974	'Pgm2l1'	5.09	7.22	4.83	4.97	6.5	15.01	6.46	4.02	2.94	3.08
70976	'Ccdc105'	0	0.34	1.61	0	0.17	0.03	0.06	0.12	0	0.52
70981	'4931423N10Rik'	0	0.23	0	4.53	0	0	0.27	0.01	0	0.02
70984	'4931406C07Rik'	5.45	2.15	4.68	3.8	2.89	9.66	3.11	0.39	3.98	3.31
70989	'Jhy'	0	0	0	0	3.45	0	0	0	0	0
70997	'Spef1'	0	1.78	1.76	5.55	3.32	0	4.52	5	0	5.01
70998	'Phf6'	6.52	7.61	6.68	4.42	4.86	2.93	0.93	6.2	9.15	4.72
70999	'Naa40'	8.88	3.37	4.66	7.23	8.98	2.09	4.77	3.49	7.18	8.76
71003	'Prss41'	0	3.27	0	0	0	0	0	0	0	1
71007	'D7Ertd443e'	0.03	0	0	0	1.48	3.19	0	1.65	0	0
71020	'Spats1'	4.18	3.97	0	8.84	3.24	1.49	0	2.65	0.53	3.97
71030	'4933403O08Rik'	0.39	0.15	0.21	1.02	0	0	0	1.2	0	1.4
71041	'Pcgf6'	5.83	2.2	4.84	23	5.47	3.13	2.35	5.46	4.65	3.23
71046	'4933405L10Rik'	3.25	0	0.53	0	4.27	0	0	1.28	0.3	0
71059	'Hexim2'	43.13	55.7	26.7	10.7	23.1	12.24	73.9	56.28	26.09	46.62
71063	'Zfp597'	0.67	0.04	0.25	4.23	0.61	0.16	2.63	1.19	2.33	2.7
71069	'Stox2'	3.8	2.37	3.29	0.9	2.65	2.36	1.2	2.5	1.69	1.64
71085	'Arhgap19'	0.82	0.09	4.05	0	1.05	0.06	0.07	1	0.26	0
71089	'Sept12'	0	0	0.65	0	0	0	0	0	0	0
71091	'Cdkl1'	12.73	12.64	9.54	10.9	17.2	17.46	4.56	13.57	5.06	22.35
71093	'Atoh8'	0	0	0	0	2.31	0	0	0	0	0

71096	'Sntg1'	5.06	13.69	15.4	6.03	8.98	5.48	9.33	3.6	5.81	8.69
71099	'Tssk4'	0.33	0	0.56	0	0	0	0	0	0	0.21
71101	'Uvssa'	2.98	3.2	3.07	1.85	4.01	2.44	1.44	2.42	4.35	1.86
71111	'Gpr39'	3.76	15.92	25.2	5.11	3.42	7.95	22.3	13.61	21.15	11.53
71116	'Stx18'	35.9	38.68	47.4	37.7	19.4	14.78	31.3	20.07	27.75	40.36
71130	'Sh2d6'	4.84	1.66	0	0	3.31	9.48	3.28	0	0	0
71131	'Zfp689'	5.35	5.29	4	11.5	2.73	0.01	7.68	5.36	0.03	12.88
71132	'Cabyr'	0.82	2.53	2.65	7.05	2.43	0	0	1.41	3.63	1.04
71137	'Rfx4'	1.81	4.4	3.01	1.09	0.43	0.09	0.02	0.57	0.27	1
71145	'Scara5'	0	3.77	0	0	0.13	2.3	0	0	0.05	0
71146	'Golga7b'	23.49	24.6	71.6	83.6	34.6	23.11	20.9	36.13	71.83	44.9
71147	'Oxsm'	1.45	0.87	0.9	0.02	0.34	0.01	0.01	3.42	4.49	4.19
71148	'Mier1'	5.74	4.21	3.74	9.22	2.95	5.56	3.6	2.02	3.29	0.88
71149	'Tex52'	3.21	0	0	0	2.55	0	0	0	0	1.37
71151	'Eri2'	1.56	2.09	0.64	0	2.78	2.1	2.45	0.96	6.9	2.7
71156	'Lrrc72'	0	0	0.08	0	0	0.15	0	0	0	0
71159	'4933416I08Rik'	0	0	1.94	0	0	0	0	1.01	0	0
71163	'Zfp626'	1.02	1.79	0.08	2.25	2.69	0.04	1.56	0.31	0.56	0.28
71164	'Zdhhc11'	0	0	0.03	0	0	0	0	0	0	0
71169	'Nbas'	1.67	0.93	3.95	0.05	3.37	2.35	0.4	2.06	1.21	3.25
71175	'Nipbl'	4.37	3.48	2.31	4.55	2.91	1.53	4.89	1.61	4.6	2.42
71176	'Fbxo24'	0	0	0	0	0.28	0	0	0.79	0	0.6
71177	'Ints13'	15.71	10.96	14.1	16.1	4.66	20.48	13.1	10.72	12.3	8.2
71198	'Otud1'	0.42	0.28	2.08	0.29	0	0	0	1.31	0	0.05
71200	'Dydc2'	3.8	0	2.37	0.07	8.38	13.78	5.85	0	1.7	3.3
71206	'Katnal2'	11.78	9.26	13.1	8.81	9.22	7.41	11.9	9.43	6.12	13.96
71207	'Nudt4'	7.74	3.45	16.6	4.21	10.8	2.61	10.1	2.06	3.63	3.67
71213	'Cage1'	3.27	1.71	0.39	5.26	1.09	2.85	0	1.68	2.87	0.22
71227	'Daw1'	0	0	0	0	0	0	0	2.13	0	0.67
71228	'Dlg5'	2.48	1.1	2.72	1.14	2.89	0.65	0.03	0.16	5.29	0.6
71233	'Enkur'	7.62	10.01	0	16.8	1.3	24.54	0	3.4	6.28	7.52
71236	'Rsph14'	0	5.04	2.16	1.73	4.85	0	0	1.52	1.71	1.57
71238	'Sdhaf3'	25.14	18.47	24.3	24.8	34	30.42	20.1	22.22	17.25	29.81
71240	'Osbpl7'	1.66	7.11	1.67	0	4.11	3.8	3.79	3.23	2.55	0
71242	'Spata24'	0	12.63	3.36	19.4	3.22	11.14	6.68	12.41	2.87	9.65
71254	'Naif1'	1.03	1.59	1.09	0	2.81	1.16	3.36	1.21	2.64	3.21
71263	'Mro'	2.61	3.65	1.31	6.86	4.11	6.85	4.36	3.93	0.84	0.02
71268	'Lrrfip2'	16.74	10.37	18.4	25.8	5.26	1.9	11.8	2.81	12.21	2.78
71275	'Noxred1'	0	0	0	0	0.35	0	0.11	0	0	0
71276	'Ccdc57'	3.9	8.87	1.84	12.2	0.51	2.24	1.36	8.23	3.38	8.78
71279	'Slc29a3'	2.45	0.22	0.07	0	1.05	2.54	0.05	0.13	0.96	3.36
71281	'Apobec4'	0	0	0.71	0	0.13	0	0.03	0	0	0.03
71287	'Cpvl'	0	0	1.87	0	0	0	0	0	0	0
71302	'Arhgap26'	17.78	14.47	21.7	22.7	20.7	7.74	26.8	21.85	25.99	18.7
71306	'Mfap3l'	0.88	0.1	1.66	0	0.59	1.96	0	0.85	1.81	0.01
71310	'Tbc1d9'	4.45	6.01	0.97	4.97	3.2	3.79	5.02	3.11	5.76	4.51
71313	'Fsip1'	2.52	0	0.03	0.1	0	3.86	0	2.45	2.95	1.35
71323	'Rassf8'	1.67	2.21	3.1	0.91	3.37	2.58	10.2	1.74	3.22	2.07
71330	'Rcbtb1'	10.62	7.44	5.35	12.8	5.74	5.61	0	6.3	13.29	6.93
71336	'Rbks'	0.05	0.05	4.25	19.6	9.18	7.59	1.21	2.79	0.72	0
71338	'Tprg'	0	0	0	0	0	0	0	1.03	0	0
71340	'Riok1'	11.34	7.08	13.5	13	7.51	23.7	4.72	6.26	9.92	5.07
71354	'Wdr31'	40.5	28.44	40	48.2	57.4	40	27.8	36.63	48.93	42.36
71355	'Col24a1'	0	0	1.2	0	0	0	0	0	0	0
71361	'Aifm2'	13.64	9.72	4.61	0.04	7.39	7.33	11.5	8.06	12.62	7.87
71365	'Pdss2'	12.64	12.23	6.78	30.7	20.8	19.22	5.7	2.8	7.29	5.72
71367	'Chst9'	0	0	3.01	0	0	0	0	0	0	0
71371	'Arid5b'	9.42	4.65	4.05	8.69	8.58	4.02	5.69	3.36	5.16	1.2

71373	'Prr16'	3.64	0	1.49	0	0	5.43	0	0	0	1.19
71375	'Foxn3'	3.45	5.32	8.51	3.26	2.83	6.01	8.72	1.84	4.4	1.59
71382	'Pex1'	7.62	6.6	4.71	4.02	6.65	8.56	2.78	5.36	14.3	6.12
71386	'Krtap28-13'	0	0	2.76	0	0	0	0	0	0	1.79
71389	'Chd6'	4.99	4.66	5.48	4.97	5.44	5.18	5.63	6.87	3.15	5.07
71393	'Kctd6'	4.27	0.89	2.12	2.67	0	0.03	6.75	4.59	3.62	7.92
71395	'5430419D17Rik'	0	0.34	0	0	0	0	0	0	0	0
71398	'5430427O19Rik'	0	0	0	0.04	0	0	0	0	0.01	0.01
71405	'Fam83c'	0	0.02	0	0	0	0	0	0	0	0
71409	'Fmnl2'	0.52	1.5	1.72	0.33	0.67	1.82	0	1.7	0.28	2
71412	'Dhrs2'	0.04	0.26	0	0	10.7	0	0	0	0	0
71435	'Arhgap21'	3.93	3.37	4.24	6.78	2.58	3.4	5.23	4.09	4.94	5.44
71436	'Flrt3'	23.28	9.33	23.7	12.8	7.83	23.39	32.1	23.72	12.1	24.34
71446	'Wrb'	147.7	184.6	171	72.8	160	174.7	176	201	240	214.21
71448	'Tmem80'	36.3	35.81	14.7	11.6	21	42.57	21.7	20.5	10.76	31.72
71449	'Mettl13'	6.54	5.02	0.02	11	3.87	0.37	7.81	4.33	3.53	2.33
71452	'Ankrd40'	3.15	6.96	4.62	3.76	11.8	4.22	4.51	11.99	4.9	1.49
71458	'Bcor'	0.59	1.59	0.03	0.06	0.13	0.01	0.01	0.71	0.05	0.57
71461	'Ptk7'	0.97	0.66	3.43	1.36	3.49	1.2	2.2	5.22	4.24	3.45
71472	'Usp19'	10.98	15.33	19.4	14	12.3	5.43	14	14.66	14.08	9.77
71474	'Ppp6r2'	21.2	22.08	28.5	38.3	30.9	9.71	17.8	16.58	40.3	29.89
71481	'Alpk1'	0	0	0	0	0.07	0	0.92	0	0	0
71492	'Bbs7'	7.52	22.02	12.9	43.3	16.8	8.83	9.58	21.87	14.21	19.75
71508	'Zfp935'	3.43	7.35	4.87	15.3	7.02	2.08	4.21	1.95	13.35	3.3
71514	'Sfpq'	11.71	10.83	6.99	20.3	15.7	7.11	7.8	6.64	9.16	10.36
71517	'Vsp35l'	32.73	31.05	31.4	44.7	42.9	28.99	28.4	43.46	26.58	41.98
71519	'Cyp2u1'	3.78	2.88	6.26	0	6.01	8.09	2.25	3.12	0.03	4.56
71520	'Grap'	0	3.16	0	0	4.07	0	0	0	0	3.8
71521	'Pds5a'	0.64	0.21	1.34	6.79	2.01	6.06	1.86	1.96	0.99	0.76
71529	'Kazn'	9.53	3.54	4.76	2.55	10.3	6	2.02	7.1	10.23	3.7
71532	'Fam217b'	8.21	8.49	4.17	0	10.4	1.72	13	6.23	7.76	1.14
71538	'Fbxo9'	107.9	104.7	119	65.9	86.5	88.3	93.8	60.11	84.69	27.75
71544	'Arhgap42'	0.18	0.43	1.81	0	0.13	0	0.45	2.72	0	0
71562	'Afmid'	2.24	5.21	8.47	11	7.17	9.63	12.3	0.74	19.55	4.35
71564	'Izumo4'	92.76	101.8	114	107	130	168.8	137	109.3	155	193.32
71566	'Clmp'	12.66	11.65	3.34	9.01	2.88	1.83	0.02	2.74	5.1	7.09
71567	'Mcm9'	1.36	0.17	0	0.63	0.08	0	0	0.04	0.04	0.09
71583	'9130008F23Rik'	0	0	0.45	0	0	0	0	0	0	0
71584	'Gdpd2'	29.84	87.16	14	7.42	60.3	32.3	40.6	31.87	47.55	65.27
71586	'Ifih1'	0	2.05	1.66	0.28	0.03	0	0	0.61	2.29	3.28
71591	'Zfp251'	21.69	3.87	8.13	5.58	9.07	3.55	9.19	7.47	13.23	15.58
71592	'Pogk'	1.91	1.16	4.28	0	2.36	2.05	0.27	0.88	1.41	2.96
71599	'Senp8'	6.46	11.21	6.09	4.57	1.76	5.05	5.23	5.56	7.27	3.38
71602	'Myo1e'	1.03	0.14	0.33	0.22	0.17	0.3	0.09	0.06	0.17	0.08
71607	'Snx20'	0.84	0.59	0	0	2.47	0.55	0.5	0.31	2.52	0.51
71609	'Tradd'	0	2.29	2.22	0	11.5	0.54	5.33	6.69	2.46	3.26
71617	'Armh3'	4.88	9.98	9.41	7.11	14.2	6.01	7.54	6.29	11.31	10.23
71640	'Zfp949'	7.87	2.14	17.9	0.24	12.8	8.03	6.69	3.52	4.69	10.31
71643	'Zgrf1'	0	0.86	0.01	0	0	0.09	1	0	0.49	0.34
71648	'Optn'	15.31	15.83	12.6	6.82	25.8	35.36	17.9	22.44	4.61	30.86
71653	'Shtn1'	4.47	2.8	8.52	1.24	9.75	3.6	1.34	7.08	4.62	4.83
71660	'Rarres2'	1.4	7.23	7.93	0.09	18	0	0	30.14	4.05	6.78
71665	'Fuca1'	36.79	59.21	59.6	51.6	35.9	37.36	58.6	31.34	76.11	67.64
71667	'Tmem248'	9.23	9.3	13.1	26.8	13.3	2.15	15.6	4.38	11.13	8.58
71670	'Acy3'	0	3.2	0	0	0.05	0	8.02	2.78	5.19	0
71673	'Rnf215'	4.69	10.64	6.92	0.19	10	7.16	11.6	10.68	20.45	6.3
71675	'0610010F05Rik'	17.98	6.86	7.24	20.3	10.3	2.91	9.52	2.67	13.76	7.47
71678	'Brox'	27.42	20.8	14.1	29.5	31.3	4.38	18.9	21.94	26.63	26.29

71679	'Atp5h'	885	705.9	914	801	938	999.5	836	816.5	781.6	910.3
71682	'Wdr27'	0.09	0.05	0	0	1.93	0	0.04	0	0	0.83
71683	'Gypc'	0.02	0	0	0	0	0	0	0	0.02	0
71684	'Rbm43'	8.06	17.78	20.5	16.5	17.9	18.19	11.4	23.83	25.15	7.19
71685	'Galnt14'	42.96	19.49	5.62	46.7	0	1.45	0.03	63.78	13.14	1.74
71687	'Tmem25'	49.07	50.92	46.4	10.1	66.4	56.48	63.6	80.17	68.44	60.8
71690	'Esm1'	0.4	0	0.09	5.53	0	0	0	0.19	0	0.81
71691	'Pnmal1'	194.4	207.3	156	153	241	204	142	162.1	190.8	257.97
71693	'Colec11'	0	2.67	0	0	1.07	0	0	0	0	0
71699	'Slc41a3'	6.25	18.03	22.5	9.04	7.2	0.04	0.02	26.32	27.79	30.1
71701	'Pnpt1'	9.7	12.65	13.1	37.3	7.99	4.24	16.8	18.82	12.62	26.26
71702	'Cdc5l'	23.48	37.06	28.6	43.4	24.5	38.45	44.3	31.15	26.44	36.65
71703	'Armcx3'	40.95	25.4	32	16	14.6	6.81	42.7	27.33	27.03	33.08
71704	'Arhgef3'	5.07	7.27	2.78	4.76	8.33	1.01	3.36	2.24	1.89	11.77
71706	'Slc46a3'	1.32	4.47	0.34	9.49	11.7	4.25	0	3.42	11.67	4.4
71707	'Ubiad1'	0.85	8.59	0	2.77	1.74	14.46	0	2.69	7.84	0.01
71709	'Syde1'	0	0.6	0	0	0.05	0	0	0.26	0	0.03
71710	'Lrrcc1'	1.89	6.59	9.47	1.23	4.32	3.91	3.23	3.23	7.69	4.23
71711	'Mus81'	13.09	28.43	14.7	40.3	27.1	16.41	11.7	14.87	28.7	18.74
71712	'Dram1'	0.07	1.88	1.62	0	4.63	0	0	0.54	0	1.62
71713	'Cdc40'	2.84	3.7	8.56	3.88	8.19	8.63	11	6.22	3.95	4.18
71715	'Dhx35'	7.42	6.07	5.98	17.7	6.41	12.31	2.6	2.16	6.65	9.91
71718	'Telo2'	4.97	14.34	5.94	21.8	8.71	6.91	7.78	6.76	9.69	13.37
71720	'Osbpl3'	0.18	0	0.4	0	0	3.17	0	0.58	0.08	2.71
71721	'Fam13c'	12.29	8.07	5.06	11	6.46	2.57	18.3	9.66	9.87	4.93
71722	'Cic'	3.27	2.2	2.26	1.46	4.91	2.66	2.93	0.22	4.83	1.93
71723	'Dhx34'	0	7.05	0	0	3.84	2.01	0	0.43	7.47	5.64
71724	'Aox3'	0	0	0	0	0	0	0	0	0	1.66
71726	'Smug1'	0.21	4.69	2.41	0	4.96	3.66	4.72	1.11	0	3.66
71728	'Stk11ip'	5.11	6.86	4.65	0.1	4.46	2.08	0	2.41	3.28	6.59
71729	'Rgs12'	1.24	4.69	2.3	2.96	1.89	2.23	1.06	5.05	7.26	3.77
71732	'Vps11'	24.92	50.49	49.4	32.5	36.2	20.58	35.6	42	46.28	54.73
71733	'Susd2'	19.85	35.77	28	7.62	12.1	23.09	19.9	46.39	23.89	14.53
71735	'Lrwd1'	5.07	2.75	19.3	0	1.44	6.38	16.4	7.75	0.59	7.34
71738	'Mamdc2'	0	0	0.6	0.34	0	0	0	2.05	0	0
71740	'Nectin4'	0	0.69	1.73	0	0	0	0	0.18	0.58	0
71742	'Ulk3'	8.74	7.9	8.68	0.09	2.55	9.63	3.91	4.6	6.8	6.21
71743	'Coasy'	46	40.11	30.4	25.8	48.8	55.17	30.9	37.16	30.43	22.9
71745	'Cul2'	55	53.17	44.4	23.5	48.2	28.98	55.9	56.64	68.6	35.49
71746	'Rgl3'	0	2.81	0.06	0.02	0	0.02	1.4	0	0	0
71750	'R3hdm2'	4.03	4.2	5.55	11.1	15.6	8.93	7.44	7.15	10.17	17.3
71751	'Map3k13'	2.07	0.58	2.04	0.71	0.91	1.53	3.1	0.58	0.53	2.53
71752	'Gtf3c2'	12.78	12.78	14.3	15.4	10.4	4.18	4.79	5.79	12.12	7.43
71753	'Tmprss6'	0	0.15	0	0	9.54	0	3.12	1.07	0	2.43
71754	'Cyp2d40'	0.24	0	0	0	0	0	0	0	0	0
71755	'Dhdh'	0	3.31	0	7.66	6.03	2.19	0	0	4.08	10.53
71760	'Etnppl'	11.58	7.52	5.84	5.23	1.64	15.88	0	6.81	6.59	3.93
71761	'Amdhd1'	0	1.03	0	0	0	1.06	0	0	0	3.42
71764	'C2cd2l'	14.03	12.84	17.8	18.8	20.1	11.48	13.7	8.55	2.68	15.02
71765	'Klhdc3'	84.88	68.18	115	51.1	62.6	102.7	128	91.43	94.76	54.78
71766	'Raver1'	7.08	5.72	8.36	6	8.35	4.49	6.11	6.11	8	11.82
71767	'Tysnd1'	4.15	2.58	13.8	0.03	0.02	3.62	12.4	1.77	2.77	0.26
71768	'Vwce'	1.89	0	0	0	2.24	0.05	0	0	0	0.84
71769	'Bbs10'	4.68	6.24	0.02	19.3	0.05	0.03	0	1.46	6.13	0
71770	'Ap2b1'	21.33	11.62	43.6	7.92	29.4	7.99	18.2	16.88	49.75	32.6
71772	'Plbd2'	23.15	21.4	56.2	48.3	13.5	17.01	30.7	13.71	33.33	13.64
71774	'Shroom1'	0	0	2.51	4.69	2.31	0.41	0	0.13	0	0
71775	'1300017J02Rik'	0	0	0	0	0	0	0	0.12	0	0

71776	'Tha1'	11.33	9.76	8.02	10.9	2.45	5.45	0.03	2.73	14.64	3.13
71777	'Ing3'	6.63	6.22	9.69	14.7	14.3	5.97	4.06	7.21	0.11	9.46
71778	'Klhl5'	8.15	5	5.36	13.6	2.72	4.46	11.3	0.65	0.13	0.14
71779	'March8'	2.8	2.8	10.3	1.58	3.5	4.02	7.93	4.11	5.61	3.46
71780	'Isyna1'	84.83	108	49.1	99.2	86.5	110.9	103	99.34	101.1	98.5
71781	'Slc16a14'	7.31	0	5.43	0	4.63	7.19	0.02	1.71	4.37	10.12
71782	'Ankle2'	5.44	3.31	4.7	2.39	2.56	1.84	0.07	3.47	3.76	1.53
71785	'Pdgfd'	0	1.19	0	0	6.15	0.19	4.78	1.87	0	0
71787	'Trnau1ap'	55.28	38.15	40	66.6	37	38.51	30.5	35.07	40.72	29.96
71790	'Anxa9'	1.74	0	0	0	0	0.08	0	0	0.47	0
71791	'Cpa4'	2.42	0	6.98	0	0	4.83	0	0.02	4.5	0
71793	'Ints12'	5.32	3.87	7.51	8.58	1.41	8.54	5.65	8.03	8.31	3.96
71795	'Pitpnc1'	10.23	13.18	11.6	13.7	8.68	5.61	9.68	12	9.61	5.44
71799	'Ptcd1'	17.72	14.39	18	3.53	15.4	5.67	13.3	9.96	18.86	14.5
71801	'Plekhf2'	3.45	0	0.41	0.17	0	1.56	0	0	0	0
71803	'Slc25a18'	29.52	20.77	4.57	23.8	1.51	39.35	16.5	23.75	11.87	4.82
71804	'Mtfr2'	0.27	0.19	0.54	5.98	0.02	0.06	0.52	0.47	0.22	0.29
71805	'Nup93'	8.83	7.8	14.7	16.2	8.69	7.01	25.6	10.43	6.35	5.66
71807	'Tars2'	34.78	28.24	17	24.5	17.8	27.99	23.2	21.84	10.85	24.34
71810	'Ranbp3'	85.13	68.85	70.7	68.7	68.3	92.2	130	68.77	94.31	79.54
71816	'Rnf180'	10.49	10.55	15.1	12.2	6.43	0.07	9.65	14.44	8.16	13.67
71817	'Tmem50a'	263.4	262.3	164	306	206	285.5	133	267.1	241.6	298.97
71819	'Kif23'	0	0	0	0	0	0	0	0.03	0	0
71820	'Wdr34'	73.53	67.58	46.1	71.8	50	62.91	55.7	53.93	27.69	95.5
71827	'Lrrc34'	0.12	0	0.63	0	2.34	0	0.11	1.6	0	0
71828	'Gtf2a1l'	0	1.31	0	0	0.64	0	0	0	0	0
71829	'Ddi1'	0	0	0.03	0	0	0	0	0	0	0
71833	'Dcaf7'	29.57	30.02	55.2	23.5	21.5	21.59	32.8	28.47	48.63	42.17
71834	'Zbtb43'	2.21	1.5	1.9	4.02	0.3	6.28	1.88	2.48	2.54	3.37
71835	'Lancl2'	21.96	13.69	17.2	21.1	8.88	9.65	22.6	25.41	18.12	14.03
71836	'Shcbp1l'	1.17	0.31	3.06	4.51	1.35	3.57	0.47	0	2.74	0
71837	'1700003E16Rik'	9.31	21.76	9.51	11.8	29.6	23.73	8.94	8.71	16.91	23.19
71838	'Phf7'	0	4.26	2.84	8.03	10.6	3.46	5.22	4.04	0.88	7.44
71839	'Osgin1'	0	0	0	0	0	0	2.25	0	0	0
71840	'Tekt4'	1.49	5.04	0.1	0	0	1.12	0	1.38	0	0
71843	'R3hcc1'	12.24	16.43	26.4	5.57	28.9	17.91	14.4	15.48	13.35	26.42
71844	'Nupl1'	13.74	11.47	5.08	5.02	11.4	2.71	15.4	9.29	4.39	14.96
71846	'Syce2'	5.62	6.76	8.57	17.5	0.34	9.63	8.94	3.64	26.59	9.31
71853	'Pdia6'	263	323.3	129	185	176	310.7	108	206.5	257.2	290.13
71856	'Wfdc3'	0	0	0	0	0.08	4.02	0	0.89	0	0
71860	'Cfap52'	0	0	0	0.19	0	3.74	0	0	0.09	4.58
71862	'Gpr160'	0	0	0	0.03	0	9.06	0	0	0	0
71865	'Fbxo30'	1.12	0.99	1.08	5.27	0.82	0	0.04	0	0.01	0.76
71868	'Thegl'	0	0	1.63	3.54	0	0	0.03	0.03	0.15	1.62
71870	'Cfap45'	12.32	5.38	3.32	0	0.03	0.38	19.3	1.61	1.4	4.65
71872	'Aox4'	0	0	0	0	0	0	0	1.77	0	0
71874	'2310007B03Rik'	0	0	0	0	0	0	0	0	0	1.73
71876	'Cenpu'	2.71	0	0	0	0.89	0	4.64	5.24	0.04	1.23
71877	'Efhc1'	0.02	1.91	0	0.42	0	2.81	0	7.38	0.02	0
71878	'Fam83d'	0	1.59	0	0.22	0.03	0	0	1.13	0	0
71881	'Apmap'	78	94.62	97.9	95.1	69.8	104.2	105	114.7	74.11	88.36
71883	'Coq2'	15.69	6.87	6.08	42.8	22.7	4.33	18.6	14.32	12.68	11.16
71885	'Faap100'	0.56	10.72	1.81	8.09	3.15	3.98	12.8	7.42	6.12	0.21
71886	'2310002L09Rik'	0	0	0.05	0	0.03	0	0	0	0	0
71887	'Ppm1j'	0	0.37	0	17.4	0	0	0	0	0	0
71889	'Epn3'	0	0.04	3.97	0	0.46	0.2	0.03	0	0	3.14
71890	'Mad2l2'	49.12	46.76	17.8	66.3	6.34	23.87	39.8	24.84	29.49	29.4
71891	'Cdadc1'	76.1	49.23	33.9	76.6	44	72.5	30.8	42.55	64.98	72.35

71893	'Noxo1'	0	1.81	0	0	5.56	0.09	0	0.71	1.05	0.03
71897	'Lypd6b'	5.4	0	0	0	12.2	14.61	0.34	6.31	12.72	8.48
71900	'Tmem106b'	9.85	5.58	8.92	13.5	10.5	3.99	11.7	9.35	5.78	7.35
71901	'Fam219a'	12.6	7.87	12	2.23	8.01	11.25	14.1	6.55	11.62	7.03
71902	'Cand1'	11.49	7.5	14.6	1.31	5.87	3.71	19.8	3.98	12.55	10.8
71903	'Ces2f'	0.29	0.29	0.29	0.21	0.25	0.25	0.32	0.1	0.37	0.3
71904	'Paqr7'	39.62	23.98	22.2	18.9	21.3	35.72	25.4	25.73	23.78	33.11
71907	'Serpina9'	0	0	0	0	0	0	0	1.53	0	0
71908	'Cldn23'	2.89	2.95	0	3.37	0	0	0	0	0	4.9
71909	'Haus5'	5.27	2.97	3.83	14.5	0.04	7.78	0	2.86	7.41	7.48
71910	'Plpp5'	25.76	19.35	16.2	0.94	19.9	19.75	12.2	21.76	16.68	18.51
71911	'Bdh1'	17.04	18.05	32	26.2	27.3	18.69	19.5	31.16	16.39	18.91
71912	'Jsrp1'	2.04	0.4	2.22	0	3.99	0	3.23	2.55	0	0
71913	'Tmem79'	0.9	0	3.13	0	0	0	0	0	0.04	0
71914	'Antxr2'	0.01	0	0.01	0	0.01	0.01	0	0	0	0
71916	'Dus4l'	0	0	1.79	0	0	5.7	0.03	0.28	4.05	4.83
71918	'Zcchc24'	2.17	1.92	2.14	5.9	3.2	0.28	12.9	1.32	0.32	1.21
71919	'Rpap3'	23.41	20.46	28.9	13.8	14.5	24.01	10.9	31.72	31.92	9.99
71923	'Borcs6'	9.59	9.97	5.63	5.12	4.64	3.39	4.22	1.14	3.97	5.61
71924	'Tube1'	5.31	1.45	3.57	0.54	0.29	1.63	0.03	2.63	1.94	2.43
71927	'Itfg1'	142.7	161.2	174	167	87.3	95.18	151	118.5	176.2	105.51
71929	'Tmem123'	0.05	1.36	0	0	1.21	0.02	0	0	0	0
71932	'Ephx3'	0	0	0	0	0	0.04	0	0	0	0
71934	'Car13'	0	0.08	0	0	0	0	0	0	0	0
71939	'Apol6'	0.04	0.02	2.32	0.08	2.7	0.09	0.63	1.86	0.02	0.23
71941	'Cars2'	7.69	22.74	17.5	10.2	12.2	8.75	13.8	9.88	6.66	0.23
71943	'Tom1l1'	0.24	7.68	2.21	0.2	0.14	0.85	8.62	4.89	1.5	2.77
71946	'Endod1'	26.3	17.53	33.4	14.2	31.2	18.8	28	20.81	44.29	19.95
71947	'Tmem94'	8.94	3.69	4.06	0	5.1	0.72	9.6	1.82	4.9	5.46
71949	'Cers5'	30.42	27.17	33.5	74.6	36.1	9.32	38.4	29.59	41.58	28.83
71950	'Nanog'	0.49	0.28	0.65	0.56	0.65	0.41	0.12	0.1	0.95	0.32
71951	'Gpc2'	2.72	1.44	0.89	0	0.19	0	0	0	0	0
71952	'Riox1'	0.07	0	0	0	0	0	0	0.9	2.57	0
71954	'Suds3'	2.58	4.95	2.87	12.7	7.01	10.28	4.47	2.06	4.78	2
71955	'Ist1'	61.34	60.33	37.9	51.8	44.3	62.67	59.6	63.16	51.02	38.68
71956	'Rnf135'	3.9	6.46	2.53	0	2.95	5.56	0	1.91	2.57	6.06
71957	'Ints11'	28.33	32.36	30.8	18.2	49.6	57.65	20.9	53.26	29.06	25.5
71960	'Myh14'	2.3	0.29	1.06	0.01	2.3	0	0	0.93	0	0.02
71962	'Castor1'	1.7	0	0	2.5	0	0	11.2	2.96	0	1.9
71963	'Cdca4'	0	4.24	0	17.8	7.3	4.2	0.02	2.79	8.79	7.82
71966	'Nkiras2'	9.23	9.61	11.7	0.02	3.18	7.58	20.1	4.83	3.98	3.69
71967	'Mageb16'	0	0	0	0	1.12	0	0	0.05	0.5	0
71968	'Wdr73'	13.54	34.57	19.8	8.71	28.9	7.07	15.4	20.85	32.83	14.4
71970	'Zbed5'	9.57	7.24	4.24	8.01	1.63	5.52	14.1	8.95	1.49	7.94
71971	'Zswim1'	40.68	19.36	39.3	16.3	28.2	26.31	27.1	37.52	33.68	54.54
71972	'Dnmbp'	2.25	1.42	1.68	2.18	0.22	0.34	2.39	1.64	0.01	3.23
71973	'Rbpms2'	3.26	0	2.04	0	2.1	0	0	2.43	0	0.03
71974	'Prmt3'	9.11	16.75	7.43	13.5	9.17	1.86	5.59	3.66	13.17	2.72
71978	'Ppp2r2a'	37.09	47.67	48.3	45.2	36.9	41.53	62	40.23	36.94	30.37
71981	'Tdrd12'	0.76	0.01	0	0	0	0	2.96	0	0	0.76
71982	'Snx10'	106.9	112.8	103	80.7	112	65.03	98.6	87.37	114.2	97.23
71983	'Tmco6'	8.47	4.52	3.9	9.53	8.1	11.24	5.74	0.27	6.02	5.34
71984	'Sars2'	17.3	14.94	26.9	6.02	18.1	24.11	23.7	10.59	29.16	25.02
71985	'Acad10'	0	0.66	0.73	0	0	2.79	0	0.04	0.09	0.09
71986	'Ddx28'	20.74	5.83	18.9	8.01	4.73	11.66	5.88	22.99	19.84	6.27
71989	'Rpusd4'	12.62	1.24	5.22	10.9	6.8	1.3	1.12	1.64	13.99	9.44
71990	'Ddx54'	17.39	10.75	2.74	2.32	11.9	0.02	3.34	7.92	23.67	7.55
71991	'Ercc8'	7.12	6.73	3.45	0.46	10	0	9	14.82	9.65	2.35

71994	'Cnn3'	14.78	43.53	44.4	41.7	27.4	31.73	54.4	27.78	16.58	17.83
71997	'Smg9'	20.36	17.93	17.7	29.5	30.5	11.87	3.18	16.19	39.21	18.76
71998	'Slc25a35'	2.42	0	0	0	1.5	0	0.03	0.05	4.93	0
71999	'Fbxo22'	55.76	36.82	68.6	53.7	42.6	19.92	40.4	38.11	45.96	47.5
72002	'Slc39a5'	0	0	0	0	0	0.95	0	0	0	0
72003	'Synpr'	14.95	48.46	119	45.7	3.5	12.41	115	52.22	0.05	41.91
72007	'Fndc3b'	0.54	1.33	0.57	0.66	1.47	0.01	0	1.43	1.14	3.71
72008	'Zfyve19'	27.11	29.27	20.2	82.6	24.8	41.1	24.2	20.87	37.48	39.86
72014	'Btbd17'	5.76	9.44	1.65	0.07	12.4	3.31	6.53	7.57	13.33	0.18
72016	'Tedc2'	4.03	1.65	3.64	0.16	2.17	4.44	0.5	0.6	9.05	4.66
72017	'Cyb5r1'	19.96	22.28	12.2	18.2	34.4	28.13	9.42	31.44	42.84	31.03
72018	'Fundc1'	62.81	37.92	67.1	56.5	54.3	89.86	62.2	73.52	56.26	73.21
72020	'Zfp654'	1.51	0.16	0.18	2.79	0.2	0.01	3.01	3.36	0.03	0.29
72022	'Slc35f2'	0	2.2	0	0	0.02	4.95	5.29	3.95	0	0
72023	'Cyb561d1'	1.36	1.19	1.86	0	8.22	1.8	0	3.69	3.41	4.09
72026	'Trmu'	51.24	34.57	41.3	2.51	39.5	48.64	46.7	17.29	45.09	41.66
72029	'Cnpy3'	49.69	82.06	71.9	46.4	59.9	78.64	72.4	65.63	97.06	95.75
72033	'Tsc22d2'	2.2	2.03	7.49	2.7	2.54	1.54	0	1.95	1.87	2.68
72039	'Mccc1'	10.22	18.14	22.5	4.65	37.9	12.59	23.3	20.63	12.12	15.92
72041	'Alkbh4'	5.98	2.92	6.37	0	6.5	12.24	11	4.63	4.8	4.44
72042	'Cotl1'	103.7	79.1	98.7	41.4	104	116.9	105	118	61.5	146.29
72043	'Sulf2'	2.68	5.8	20.5	0	13.1	8.6	21.6	13.65	9.56	13.3
72046	'Urgcp'	35.82	24.8	33.5	1.36	27.9	14	24.2	43.08	26.07	22.44
72047	'Ddx42'	23.44	21.87	27.4	40.8	25.6	12.68	23.4	30.05	32.51	35.13
72049	'Tnfrsf13c'	0	0.53	0	0	0	0	0	0	0	0
72050	'Kdelc1'	10.49	5.23	5.52	0	7.9	11.46	8.51	8.53	6.06	14.07
72053	'Tmub2'	54.14	50.34	65.9	29.9	55.7	89.75	50.4	56.9	29.09	52.44
72054	'Cyp4f18'	0	0	0.08	0	0	0	0	0.19	0	0.45
72055	'Slc38a10'	34.31	26.87	40.4	19.1	29.8	39.46	19.6	46.11	58.44	78.37
72056	'1810055G02Rik'	0.1	5.33	7.75	22.8	6.22	6.41	20.8	2.78	0.02	7.38
72057	'Phf10'	6.78	2.98	1.34	1.12	0.31	4.27	0	1.1	0.94	0
72061	'2010111I01Rik'	2.11	2.81	5.14	0.02	3.54	6.09	4.1	4.23	8.42	2.86
72065	'Rap2c'	0.88	1.67	2.45	0	0.27	0.05	1.91	2.99	0	0.24
72068	'Cnot2'	10.8	23.6	22.3	16.3	14.9	19.79	8.78	20.03	24.84	25.37
72075	'Ogfr'	10.53	10.64	27.3	16.8	14.2	15.35	9.18	16.35	17.11	7.55
72077	'Gcnt3'	0	0	0	0	0	0.01	0	0	0	0
72080	'Sapcd2'	0	0	8.72	0	0.39	0	10.6	0	0	0
72083	'Mzt2'	31.03	34.91	15	50.9	11.4	50.9	32.8	35.37	31.67	23.22
72084	'Pigx'	35.78	30.51	25.9	53	36.1	29.36	18.5	36.72	27.89	25.48
72085	'Osgepl1'	16.65	17.47	7.1	13.7	12.9	15.36	10.8	17.92	5.07	10.02
72088	'Ush1c'	0	4.56	0.02	0	0	0	0	1.94	0	0.75
72096	'Eef1akmt2'	25.84	14.79	17.9	6.71	31.7	42.77	22.5	21.51	21.97	26.49
72097	'2010300C02Rik'	3.16	3.42	1.93	2.98	4.55	0.13	8.28	0.25	5.13	1.73
72098	'Tmem68'	23.43	24.33	15.4	20.4	7.42	13.95	10.1	18.63	12.25	22.94
72102	'Dusp11'	5.98	4.68	2.89	3.19	3.28	5.43	6.45	3.7	2.21	4.73
72103	'Aplf'	0.63	1.02	0.49	0.41	1.74	1.42	0.27	2.1	0.09	1.5
72106	'Jmjd8'	65.31	60.65	66.6	24.4	33.2	58.62	33	41.58	35.6	44.76
72108	'Ddhd2'	32.41	33.44	30.9	34.5	39.5	52.89	49.2	25.68	39.2	42.78
72112	'Ppp1r14d'	0	0	0	0	4.96	0	0	0	0	0
72113	'Adck1'	8.78	6.29	43.1	21.9	20.3	2.72	35.6	25	15.86	20.79
72114	'Zbed3'	1.54	3.59	8.67	4.18	10.7	0.35	2.83	5.64	14.93	8.93
72117	'Naa50'	13.55	21.37	16.1	32.8	10.9	11.31	27	8.39	21.98	9.41
72119	'Tpx2'	0	0	0	4.69	0	0	0	0.01	0	0
72121	'Dennd2d'	0	0.04	0	2.05	2.68	0	1.49	0.06	1.68	2.94
72123	'Ccdc71l'	0	0.14	0.11	0	1.17	0	1.39	0.16	0.21	0.26
72124	'Seh1l'	43.85	30.53	22.2	33.6	27.1	23.63	51.4	47.97	25.42	27.07
72125	'Amer2'	3.83	1.77	6.77	2.91	2.29	8.68	1.42	1.19	0.95	2.13
72128	'2610008E11Rik'	6.67	2.43	7.96	2.8	2.97	2.73	3.06	3.36	2.4	5.6

72129	'Pex13'	9.45	5.42	12.1	10.6	12.2	14.45	13.4	11.8	7.98	9.62
72133	'Trub1'	11.8	4.37	15.5	6.15	10.7	8.66	6.93	13.75	6.24	5.78
72135	'Pygo1'	2.49	0	1.78	2.66	0.45	0	3.58	0	0	0
72136	'Chst14'	2.7	2.9	0	0	0.02	0	3.67	0.02	0	0
72137	'Wdsub1'	9.29	5.97	9.95	18.4	10.5	2.1	9.14	10.66	20.61	4
72139	'2610044O15Rik8'	16.06	12.82	9.94	22.2	7.94	27.79	4.14	15.64	8.72	14.35
72140	'Cep89'	9.82	7.53	15	11	6.83	10.46	1.14	5.07	10.09	19.33
72141	'Adpgk'	29.59	26.85	36.7	32.4	15.3	40.31	65.5	35.41	48.5	16.7
72144	'Slc37a3'	11.86	13.39	7.4	9.24	4.79	4.51	6.71	8.92	24.49	26.23
72145	'Wdfy3'	7.45	11.38	8.46	11.3	9.85	7.29	5.52	7.05	9.72	9.08
72147	'Zbtb46'	0.94	3.04	0.5	1.73	4.14	6.04	11.2	5.79	3.45	0.01
72148	'Tdrp'	17.56	28.08	15	24.2	4.7	10.14	14.6	13.72	17.02	5.8
72149	'Strada'	4.09	6.49	8.77	9.83	19.3	13.23	3.84	9.53	1.24	6.48
72151	'Rfc5'	0.54	10.9	0	1.26	2.29	5.29	0.03	8.59	3.42	6.3
72154	'Zfp157'	4.42	3	8.57	7.51	1.25	1.25	7.71	5.15	3.1	2.92
72155	'Cenpn'	4.49	0.03	0	4.45	0.77	0	0	3.81	0	0
72157	'Pgm2'	97.11	124.8	107	105	70.5	126.6	114	97.87	94.96	147.01
72160	'Tmem163'	0.6	10.67	18.5	25.4	0.06	12.39	12.1	12.61	25.82	17.09
72162	'Dhx36'	5.83	3.35	4.2	7.27	8.67	4.69	7	5.5	5.03	6.28
72167	'Thumpd2'	1.49	4.99	5.59	0.39	2.77	4.77	7	6.62	10.26	1.14
72168	'Aifm3'	29.78	43.81	14.8	26.7	63.5	66.11	14.5	17.65	27.4	92.1
72169	'Trim29'	0	0	0	0	0	0	0	1.05	0	0
72170	'Chchd4'	122.5	95.66	136	222	95.5	78.22	80.8	99.89	140.6	64.77
72171	'Shq1'	4.94	3.02	5.7	5.47	4.09	6.18	14.6	4.81	3.92	2.3
72175	'Mfsd8'	7.9	1.92	1.79	7.5	0	2.12	3.65	4.21	6.33	6.7
72179	'Fbxl2'	67.96	57.39	38.6	34	39.2	14.74	33.8	30.24	53.87	84.19
72180	'Zfp661'	4.94	2.37	0.02	0	0.35	0.11	2.7	4	0	1.66
72181	'Nsun4'	3.9	10.89	17.1	0.02	7.34	10.96	9.57	5.47	15.13	9.03
72183	'Snx6'	48.65	56.35	38.6	55.8	43.1	35.52	30.3	45.19	40.04	49.45
72184	'Klhl35'	0	0.61	0.17	0	0.17	0	0.03	0	0.64	0.83
72185	'Dbndd1'	41.85	35.95	24.4	33.3	45.7	67.7	32.2	50.65	58.72	29.7
72190	'2510009E07Rik'	3.72	4.21	5.85	2.23	3.55	1.96	3.84	1.2	2.54	1.15
72193	'Scaf11'	4.75	5.41	4.28	5.16	4.32	1.9	0.68	3.42	3.73	2.67
72194	'Fbxl20'	0.56	1.44	2.13	9.01	1.78	1.41	0.83	1.6	1.23	1.92
72195	'Supt7l'	6.87	9.13	5.74	8.61	4.05	0.07	3.11	10.82	25.34	11.73
72198	'Mtrex'	18.91	19.66	9.17	10.8	15.5	14.29	25.2	21.75	20.05	26.32
72199	'Mms19'	12.64	5.53	21.3	5.46	10.9	10.2	15.7	0.25	6.92	17.8
72201	'Otud6b'	22.65	24.89	23.3	10.8	27.8	19.95	24.6	25.35	10.26	31
72205	'Eml2'	11.29	11.86	5.45	1.91	27.3	16.32	19.9	14.96	16.31	9.46
72215	'1700001P01Rik'	0	0	0	0	0	0	0	0	0.53	0
72230	'Zfp558'	0.56	2.08	1.63	0	2.47	0.01	6.01	3.06	2.87	0.06
72236	'Tsnaxip1'	0	0	0.02	0	0	0	0	0.18	0.02	0
72238	'Tbc1d5'	0.94	3.14	4.64	0	3.12	1.05	1.48	0.97	4.45	4.05
72244	'1600014C10Rik'	24.05	11.58	17.9	26	21.1	28.27	12.9	11.84	21.51	19.38
72254	'1700030K09Rik'	12.46	8.85	5.19	2.52	10.5	10.83	21.3	4.12	13.53	7.49
72258	'Kcnk10'	0.01	1.75	4.54	0	2.11	0	5.27	0.44	1.57	0.16
72265	'Tram1'	8.21	10.34	3.14	19.7	9.62	13.38	11	6.16	24.31	13.04
72267	'Lrrc8e'	0.09	0.1	0.11	0.03	0.1	0.08	0.03	0.06	0.15	0.08
72269	'Cda'	4.84	6.38	10.5	41.3	11.4	20.41	44.7	20.51	8.66	0
72273	'Smim24'	4.22	1.73	11.3	9.17	0	0	6.26	8.49	26.28	4.41
72275	'2200002D01Rik'	0	1.26	0	0	0	0	0	0	0	0
72278	'Ccpg1'	69.59	74.82	51.1	45.1	42.1	54.52	52.4	49.48	50.28	71.87
72281	'Sh2d4a'	0	0	0	0	0.02	0	5.98	0	0	0
72284	'LTO1'	15.78	13.38	14.9	13.8	10.9	16.15	29.7	17.68	23.89	18.35
72287	'Plekhf1'	3.63	2.87	5.37	0	0	0	0	2.76	0	0
72290	'Lsm11'	0.05	0.13	1.16	2.58	0.4	0.06	1.04	0.12	0.48	0.17
72293	'Nkd2'	0	0.01	0	0	0	0	0	2.45	8.34	0.01
72296	'Rusc1'	33.69	28.25	53.7	12.7	30.6	20.55	45.6	28.38	36.58	14.1

72297	'B3gnt3'	0	0	0	0	0	0	0.02	0	0	0
72301	'Shisal1'	3.02	3.02	8.72	5.33	14.5	4.02	12.1	5.93	8.94	2.13
72303	'Cyp2c65'	0.02	0	0	0	0	0	0	0	0	0
72306	'Zfp777'	3.38	3.82	8.95	2.17	7.11	3.34	4.37	9.04	8.13	8.85
72307	'2510002D24Rik'	34.39	33.41	57.8	69.6	60.1	74.27	70.4	47.09	49.99	49.96
72308	'Brf1'	2.8	7.87	2.83	4.12	1.66	3	5.36	4.08	11.29	7.16
72309	'Tmem158'	12.55	7.37	10.5	54.6	13.2	4.22	6.8	2.8	5.71	7.88
72310	'Nkg7'	0	3.17	0	0	0	0	0	0	0	0
72313	'Fryl'	1.53	3.14	2.07	2.33	3.73	1.71	4.51	1.67	4.41	1.37
72315	'Ccdc74a'	3.42	3.91	3.65	27.7	3.69	6.07	0	5.79	0.43	3.58
72318	'Cyth4'	0.02	1.69	0	0	0.25	1.05	0	0	0.02	2.29
72320	'Kif1bp'	20.01	17.1	22.1	13.9	11.3	20.91	40.9	43.1	40.8	16.69
72322	'Xpo5'	3.91	9.94	17.9	6.17	3.22	3.59	6.27	9.73	10.48	9.56
72323	'Asb6'	23.79	29.54	6.64	17.7	17.8	13.94	23.6	33.17	25.49	32.94
72324	'Plxdc1'	8.83	8.34	20.4	0.02	7.52	2.23	12.5	5.69	3.58	8.4
72325	'Vps9d1'	7.5	6.25	11.9	2.76	7.89	6.77	16.9	10.55	10.18	13.61
72330	'Klhl40'	0.07	0	0.02	0.07	0	0.02	0.02	0	8.36	0
72333	'Palld'	1.66	8.01	6.71	4.77	0.9	1.93	5.44	5.51	6.71	3.12
72338	'Wdr89'	4.18	3.96	4.84	0.31	0.28	1.8	7.74	1.29	3.98	4.96
72341	'Elp6'	30.46	32.13	36.4	35.8	15.1	18.48	27.6	17.03	10.33	15.49
72344	'Usp36'	4.66	5.24	7.77	13.9	5.6	3.68	9.22	2.52	7.83	6.85
72345	'Amer1'	2.55	0.95	1.56	2.34	2.02	3.62	1.07	1.55	0.71	0.9
72349	'Dusp3'	57.13	44.92	34.8	72.4	103	72.74	34.2	67.44	75.27	85.97
72350	'Zc2hc1c'	3.42	3.36	7.55	4.98	2.01	2.01	1.66	0	4.62	1.1
72351	'Ptar1'	1.19	0.53	2.18	0	3.13	4.37	0	0.58	0.82	0.26
72354	'Ttc4'	39.99	47.35	58.3	34.8	81.6	50.63	41.3	68.08	55.73	55.05
72355	'Cdpf1'	34.16	26.27	27.5	17.2	38.1	72.96	30.3	12.15	41.06	11.95
72357	'2210016L21Rik'	260.4	179.8	257	246	252	300.9	313	267.7	289.5	239.69
72368	'Borcs8'	78.74	64.86	28.1	39.2	46.9	87.39	49.8	67.12	73.8	93.06
72371	'2210408I21Rik'	4.59	6.4	6.45	0	5.36	8.61	4.72	6.27	4.05	5.37
72388	'Ripk4'	0	0	0	0	1.05	0	0	0	0	0
72391	'Cdkn3'	7.85	0	0	0	0.94	0	0	1.01	0	0
72392	'Tmem175'	35.9	52.38	33.6	42.9	38.9	73.12	51.5	79.25	64.94	68.3
72393	'Faim2'	333.5	330.1	297	189	318	390	386	377	365.6	327.64
72397	'Rbm12b1'	3.06	2.17	1.36	6.31	1.01	5.12	8.04	0.99	3.29	5.51
72399	'Brap'	6.27	5.19	10.3	13.8	18.9	3.74	1.66	1.87	4.51	4.4
72400	'Pinx1'	6.92	5.49	2.28	12.6	3.76	0	5.41	4.71	2.42	0
72404	'Wdr44'	1.84	1.66	0.59	6.01	1.75	3.35	3.13	2.2	1.36	3.81
72413	'Kcnmb2'	0	2.02	0	0	23.1	0	3.82	0	0	0
72415	'Sgo1'	0	0	0	0	0	0	0	0	3.36	0.63
72416	'Lrpprc'	6.96	12.4	33.2	28.8	13.5	8.88	32.3	16.39	26.79	28.12
72421	'Ttc30b'	40.31	29.32	24.1	27.4	20.8	12.12	14.9	29.33	17.61	18.66
72425	'Katnbl1'	7.91	8.82	2.86	0.02	1.59	0.5	0.06	11.26	4.2	6.5
72429	'Dnajc25'	0.7	0.03	0.06	3.04	0.82	0	0	0.71	1.25	1.97
72433	'Rab38'	1.16	0	0	0	0	0	0	0	0	3.61
72434	'Lypd3'	0	0	1.79	0	0	6.68	0	0.99	1.7	4.62
72440	'Rhno1'	19.79	6.31	5.49	27.2	14.3	21.96	18.2	8.91	18.77	27.03
72446	'Prr5l'	1.2	1.87	0.06	0.06	0.06	0.42	0.02	0	0	2.06
72454	'Ccdc71'	35.38	15.91	14	6.21	7.44	15.66	10	22.56	24.86	15.89
72459	'Htatsf1'	41.98	31.93	30.5	25.6	27	23.99	28.1	33.53	32.6	5.24
72461	'Prcp'	3.9	3.97	5.79	0	4.15	2.62	11.7	2.88	2.58	3.87
72462	'Rrp1b'	6.79	19.33	9.48	0.39	5.66	22.23	15.7	11.01	4.99	9.23
72465	'Zfp131'	8.46	7.99	5.82	21.5	3.25	0.46	1.83	10.3	7.04	5.79
72469	'Plcd3'	0	6.24	2.42	0.17	2.34	0.19	1.61	3.04	0.28	0.07
72472	'Slc16a10'	0.8	0.1	0.25	0.64	1.51	0.04	0.19	0.14	0.31	1
72475	'Ssbp3'	28.49	30.26	30.3	81.2	24	23.75	29.6	17.26	11.05	31.56
72477	'Tmem87b'	16.16	8.73	10.4	6.69	12.8	12.68	10.5	8.39	8.49	11.72
72479	'Hsdl2'	9.9	8.9	16.2	5.86	15.2	7.5	14	13.47	4.44	6.58

72480	'Tspyl4'	214.4	185.1	247	113	154	91.06	210	245.5	234.8	151.06
72482	'Acbd6'	27.43	11.25	30.5	45	20.8	15.36	23.1	15.51	21.86	4.99
72486	'Rnf219'	3.85	6.97	3	1.58	9.54	7.52	8.12	5.43	0.7	1.44
72500	'Ier5l'	0.03	0	2.6	0	1.62	0	2.55	0	0	0
72502	'Cwf19l1'	0.03	3.83	5.08	4.68	3.83	0.79	16.3	9.36	7.89	2.21
72503	'2610507B11Rik'	3.76	8.78	15.5	5.58	6	4.24	7.59	9.71	6.42	5.05
72504	'Taf4b'	0.48	0	0	0	0.7	0	0.02	1.75	0	0
72507	'Dzip1l'	3.72	2.31	3.89	0	4.43	2.51	1.5	3.19	1.8	1.78
72508	'Rps6kb1'	13.81	25.19	20.5	16.6	21.2	10.94	16.6	24.29	9.96	28.66
72512	'Tmem173'	0	0	0	0	0.02	26.37	0	0	0.02	0
72514	'Fgfbp3'	1.84	0	0	0	0.99	1.41	4.22	0.34	0	0
72515	'Wdr43'	20.14	14	9.25	9.94	12.3	7.51	9.02	12.44	11.84	16.3
72519	'Pip4p2'	30.63	36.59	24.3	19.5	15.3	15.59	9.09	26.08	26.46	23.01
72522	'Atxn7l2'	6.15	7.39	5.72	0.02	4.08	0.13	8.82	10.32	1.45	4.6
72535	'Aldh1b1'	11.81	4.77	2.98	4.81	7.62	28.02	20.3	12.06	24.43	17.69
72536	'Tagap'	0.06	0.4	0.06	0	0.41	0.89	0.02	0.36	0.04	1.98
72542	'Pgam5'	28.38	36.38	34.9	68.1	26.8	24.04	32.1	28.06	24.78	54.97
72543	'Mvb12b'	5.55	6.75	2.72	3.57	4.41	0.23	7.27	4.68	4.6	1.44
72544	'Exosc6'	1.9	0.26	0	18.9	0.49	0	11	0.62	0	0
72549	'Reep4'	5.84	0.06	2.99	0	0	4.75	0.03	2.36	4.95	0
72552	'Hsdl1'	70.7	57.94	80.1	24.8	109	48.91	78.4	67.35	76.47	83.36
72554	'Utp14a'	4.86	6.53	2.5	3.02	6.68	0.04	2.42	8.01	2.74	9.21
72555	'Shisa9'	4.14	4.85	7.35	13.6	3.72	3.71	25	7	7.17	5.65
72556	'Zfp566'	2.15	4.63	0.63	15	10.2	9.06	0	5.01	8.59	4.77
72560	'Naalad2'	0	0	0.71	0	0.28	0	0.02	0	0	0.54
72562	'Pcbd2'	8.87	10.17	20	58.1	8.71	9.34	6.23	9.8	41.32	15.87
72565	'Uaca'	0	2.02	0.01	1.68	1.16	0.87	4.24	1.1	0.01	2.1
72567	'Bclaf1'	39.38	32.49	35.8	41.8	34.9	39.41	29.3	36.89	33.06	31.36
72568	'Lin9'	4.44	1.56	1.04	8.16	0.02	3.15	5.83	3.5	1.96	1.36
72569	'Bbs5'	25.81	30.22	12.9	11.6	26.7	31.92	7.58	20.55	15.78	16.21
72572	'Spats2'	2.57	0.52	0.15	0.2	2.62	1.27	3.98	4.06	3.33	2.75
72580	'Zup1'	2.02	5.05	8.28	6.66	15.6	21.94	17.7	7.36	15.59	4.66
72584	'Cul4b'	7.96	3.48	6.03	4.12	1.53	0.8	3.98	1.03	5.73	6.48
72585	'Lypd1'	4.4	13.37	24.4	24.7	2.98	4.37	18.1	9.73	14.86	8.1
72587	'Pan3'	7.7	5.91	13.1	9.87	10.5	4.5	6.3	6.55	20.28	11.35
72590	'Ppme1'	192	180.5	188	116	214	154.4	207	186.3	214.7	204
72599	'Pdia5'	1.72	2.56	0	0	0.07	0.38	3.07	0.03	10.51	2.93
72605	'Car10'	6.67	20.95	26.9	10.3	32.3	27.88	72.5	26.99	43.63	48.38
72607	'Usp13'	6.92	9.15	6.39	6.67	3.79	2.57	4.04	9.13	9.01	3.86
72611	'Zfp655'	22.05	24.07	20.8	21.4	7.9	13.31	17.8	20.69	21.08	15.23
72612	'Hpf1'	63.64	59.86	35	99	86.6	76.79	90.2	70.36	59.08	57.9
72614	'Pih1d2'	3.35	3.71	0.69	0.88	2.54	1.93	1.19	1.29	0.7	4.75
72615	'Anks3'	31.39	30.54	17.5	14.5	37.8	50.98	28.6	40.73	51.74	50.91
72621	'Pdzd11'	70.13	75.21	67.7	79.1	99.5	162.9	107	104.9	111.9	62.89
72630	'Hspa12b'	0.47	0.44	1.5	0.03	0.08	0	8.1	1.55	1.09	4.12
72632	'Smim18'	81.48	108.2	93.8	106	77.5	123.1	55.9	102.3	69.68	87.42
72634	'Tdrkh'	14.56	16.34	10.2	7.26	11.2	11.2	10.9	19.06	14.19	11.47
72635	'Lins1'	2.82	4.25	6.25	9.08	7.75	2.31	8.5	1.99	1.73	4.86
72640	'Mex3a'	0.48	0.01	0.17	0	0.5	0.75	0.37	1.7	0	0.42
72649	'Tmem209'	15.64	19.25	6.28	32.4	15.5	4.95	9.59	12.01	18.28	36.79
72650	'2810006K23Rik'	9.34	5.61	17.9	0.55	5.5	9.22	12.5	5.34	8.14	3.93
72654	'Ccdc12'	94.15	84.07	63	90.3	138	136.6	81.6	91.2	107.2	106.21
72656	'Ints8'	3.59	3.62	2.56	7.99	2.62	1.02	8.09	5.37	2.19	6.26
72657	'Selenoh'	96.76	84.71	150	57.8	50.7	103.9	111	106.6	77.84	98
72658	'2700097O09Rik'	15.84	30.14	9.88	47.4	23.5	18.07	20.1	14.67	10.99	19.83
72661	'Serp2'	174.8	117.7	217	127	157	124.4	170	149.1	155.3	123.36
72662	'Dis3'	2.92	2.01	7.8	3.32	0.24	1.89	1.11	1.43	2.36	0.41
72667	'Zfp444'	22.38	32.09	35.3	19.1	25.3	19.96	42.9	50.36	39.84	33.47

72668	'Skida1'	0.07	0.01	0.56	0	1.46	0.33	0.03	1.65	1.81	0.16
72672	'Zfp518a'	0.01	0.18	0	0	1.63	0.35	0.06	0.01	0.24	1.33
72674	'Adipor1'	23.22	16.48	17.1	18.6	14.8	31.97	11.7	14.15	8.5	9.18
72685	'Dnajc6'	19.69	27.62	31.1	30.3	25.5	9.72	29.4	19	40.3	31.16
72690	'Grrp1'	0.15	0	0.22	0	0.62	0	0	0	4.99	0.07
72691	'Calhm2'	0	0	3.29	0	3.61	0	0	0	0	0
72692	'Hnrnpll'	5.9	2.78	5.2	21.4	3.92	0.01	6.6	8.79	5.67	2.27
72693	'Zcchc12'	982.1	1034	757	1033	641	706.2	635	1311	775.3	809.67
72699	'Lime1'	12.71	14.87	23.7	3.18	19.6	8.41	21.5	12.76	12.71	13.37
72701	'Zfp618'	1.9	2.27	2.91	0.18	5.55	0	10.1	1.37	0.98	0
72709	'C1qtnf6'	0	0	0.59	0	2.06	0	0.81	0	0.06	0
72713	'Angptl1'	0.04	0	1.4	2	0	0	0	0	0	0
72720	'Zfp248'	7.21	3.55	3.6	1.49	4.65	0.09	5.71	5.44	2.91	8.83
72722	'Fam98a'	8.18	7.09	8.28	1.45	13.2	9.83	1.83	10.46	5.34	10.72
72723	'Zfp74'	7.46	4.06	4.25	0	4.98	4.99	0	5.72	4.95	5.44
72726	'Tbcc'	41.89	24.69	21.3	50.6	27	51.3	50.7	24.67	38.65	9.85
72727	'B3gat3'	30.18	20.16	11.9	24.6	34.2	52.08	18.2	23.25	48.83	29.39
72729	'Cdc42se2'	47.4	54.09	56.3	60.8	40.2	36.5	65.6	65.26	42.2	56.35
72736	'Tmx1'	82.14	98.05	82.2	98.7	42.8	60.3	38.4	91.21	128.1	123.33
72739	'Zkscan3'	10.04	18.65	15.6	5.05	1.89	10.69	7.3	6.53	10.35	21.21
72745	'Tmem161b'	8.28	3.8	6.78	5.85	3.09	3.15	4.62	10.68	9.03	8.22
72747	'Ttc39c'	6.53	4.31	4.06	9.09	1.67	10.06	0.02	1.07	0.36	3.43
72748	'Hdhd3'	7.78	3.43	0.09	10.9	38.2	25.05	6.81	16.78	27.82	15.17
72749	'Tonsl'	1.82	2.65	4.06	0.01	1.18	7.81	7.09	2.51	8.88	4.3
72750	'Fam117b'	1.73	8.59	4.11	14.6	2.24	2.11	4.94	4.04	4.56	4.48
72754	'Arhgef10l'	0.72	2.16	6.75	0.03	5.56	1.11	4.85	1.49	1.63	2.96
72759	'Tmem135'	12.63	22.87	29.6	7.44	19.1	14.52	23.4	16.21	24.85	32.26
72772	'Rint1'	12.36	5.89	12.6	6.36	6.65	6.41	10	4.41	5.57	7.82
72774	'Neil1'	0.04	11.46	3.22	8.38	4.34	11.22	10.8	8.44	11.06	5.91
72775	'Fance'	6.85	4.81	10.4	4.09	1.96	7.08	4.54	1.97	2.28	3.98
72776	'Sass6'	1.98	0.9	0.36	2.04	3.07	1.3	0.41	0.79	1.25	1.25
72780	'Rspo3'	0	8.29	1.18	0	0.31	0.82	0	0	5.48	7.13
72787	'Ndc1'	1.06	8.65	1.56	5.85	2.41	7.64	6.53	5.33	0.07	4.59
72789	'Veph1'	0.05	0.23	0.19	0.3	0.2	0.16	0.13	0.12	0.23	0.16
72792	'2810459M11Rik'	0.81	3.07	2.41	5.9	0.25	0	2.6	2.33	3.54	0
72795	'Ttc19'	7.91	17.8	3.38	10.8	9.12	2.73	10.1	10.5	12.63	5.75
72805	'Zfp839'	0.43	0.3	0.2	0.03	1.23	0.03	0	1.69	1.03	0.95
72807	'Zfp429'	0	0	3.3	0	0	0	0	0	3.99	0
72821	'Scn2b'	105.3	98.01	88.4	68.8	100	98.61	110	113.5	95.41	74.6
72823	'Pard3b'	0.03	0.45	0.01	0	1.83	0	0	0	1.07	0
72825	'Mon1a'	17.17	6.44	7.01	4.15	13.5	12.29	13.5	11.72	10.97	6.82
72826	'Fam76b'	2.66	2.46	2.87	3.45	0.37	0.48	3.22	0.63	8.32	1.06
72828	'Ubash3b'	3.47	0.54	0.41	0.02	7.31	2.27	2.02	8.64	3.13	2.86
72831	'Dhx30'	28.8	42.35	46.4	21	14.7	20.13	27.6	28	27.98	34.22
72832	'Crtac1'	36.42	11	15	30	9.87	19.45	36.2	13.47	12.9	9.38
72836	'Pot1b'	0.8	1.71	1.02	3.4	2.43	0.76	1.43	0.8	1.28	2.26
72843	'Prdm4'	1.97	3.67	2.87	2.55	0.01	0.02	8.25	6.16	1.04	0
72844	'Kctd17'	10.4	13.2	21.2	8.89	35.2	20.75	9.79	15.63	15.9	9.01
72852	'Mblac2'	29.27	30.5	35.2	25	13.8	27.18	37.8	30.58	28.48	18.22
72865	'Rtl8c'	966.9	781.4	881	1009	839	1254	1041	1126	920.1	1326.96
72873	'Bbof1'	0.93	4.51	4.2	3.48	5.2	12.43	2.9	2.9	4.98	2.28
72881	'Zdhhc4'	28.81	12.15	51.7	14.5	29.1	30.9	55.7	31.73	16.2	26.81
72886	'Yju2'	12.95	14.53	9.08	20.3	6.8	20.32	5.61	15.12	0.7	8.64
72895	'Setd5'	8.43	7.41	5.24	5.25	7.06	4.92	8.89	6.24	4.97	8.15
72898	'Asphd2'	20.94	20.26	6.06	11	15.8	28.17	12.3	20.13	13.41	3.62
72899	'Macrod2'	7.1	9.93	9.61	12.8	10.6	5.67	10.9	15.77	5.55	3.16
72900	'Ndufv2'	318.6	272.5	303	271	324	393.7	372	366	312.1	329.87
72902	'Spock3'	179.1	60.34	52.4	49.5	42	42.9	39.5	117.5	46.4	95.85

72925	'March1'	22.52	19.83	15.3	9.79	11.7	5.46	7.36	5.66	11.94	13.6
72927	'Hepacam'	23.18	15.43	33.7	7.28	9.38	23.74	10	11.09	8.1	7.04
72930	'Ppp2r2b'	63.05	75.86	48.5	64.9	49.6	64.43	85.3	101.2	97.23	85.75
72931	'Swi5'	545.4	476.8	470	730	515	701.9	602	539.6	496.5	512.6
72935	'Ddx41'	43.53	17.19	7.38	16.9	19.5	22.85	17.4	19.8	21.9	14.37
72938	'Hspb11'	29.06	27.15	22.6	39.7	34.9	53.22	37.3	30.42	35.71	40.75
72946	'Lrrc47'	0.23	2.32	3.32	4.89	0.48	0	5.53	1.34	12.55	0.02
72947	'Phykpl'	5.72	1.75	3.54	11.8	4.98	22.71	0	9.9	3.99	12.65
72948	'Tppp'	35.68	28.95	52.3	32.1	25.7	8.75	79.2	17.59	35.76	46.04
72949	'Ccnt2'	7.27	12.85	9	6.36	9.27	4.75	11.1	7.64	6.89	12.89
72958	'Zfp493'	0.29	0.01	0.32	0	0	0.45	0.26	1.56	0.13	0.76
72960	'Top1mt'	14.21	30.54	21	18.6	17.5	23.58	30.3	21.09	15	33.01
72961	'Slc17a7'	0	0	0.02	0.44	0	0	0	0.46	0.19	0.01
72962	'Tymp'	2.21	1.26	1.9	1.66	1.72	2.46	1.33	1.3	3.23	1.25
72972	'Ccser2'	6.61	11.97	11.6	7.23	11.5	8.81	6.89	8.21	5.83	5.41
72973	'Fbxo47'	0	0	0	0.19	0	0	0	0	1.11	1.85
72978	'Cnih3'	6.43	0	0	0	0.42	0	0	1.42	5.99	0
72981	'Thap12'	2.34	1.51	5.44	5.75	1.13	4.61	3.61	3.72	1.39	2.85
72982	'Tmem138'	29.79	32.52	21	35.5	18.8	38.8	22.3	29.7	24.75	41.69
72993	'Appl1'	33.14	21.38	21	14.7	25.8	20.84	28.2	19.69	25.17	20.88
72999	'Insig2'	41.54	38.25	32.6	37.3	40.2	60.7	26.8	47.45	49.08	54.8
73010	'Gpr22'	5.33	4.42	5.76	0	5.96	2.12	4.58	5.59	3.08	11.47
73016	'Kremen2'	0	3.32	7.56	1.92	0	8.73	0	1.45	3.63	0.92
73024	'Emc7'	207.8	242.2	188	148	172	218.2	188	233.1	226.8	278.96
73032	'Ttc9b'	38.78	16.74	57.4	90	80.2	14.17	46.2	29.05	63.04	33.9
73046	'Glrx5'	20.57	16.73	28	48.3	12.8	17.51	28.5	16.32	50.69	14.37
73047	'Camk2n2'	8.58	8.99	7.54	3.41	13.2	7.04	11.4	5.5	8.28	5.71
73061	'Cldn34c1'	1.5	0.17	0.22	3.18	5.73	1.41	1.91	1.41	1.04	0.32
73062	'Ppp1r16a'	31.47	9.98	43.7	6.45	24.3	9.8	16.3	15.17	29.81	13.46
73067	'Tmem192'	29.74	9.04	19.9	54.1	37	28.88	21.8	44.98	65.17	19.26
73068	'Fut11'	6.12	5.33	8.88	10.2	4.83	2.29	9.44	5.04	10.01	6.04
73072	'Prr36'	6.16	4.4	9.46	20.9	6.4	8.91	8.06	6.53	2.35	2.64
73075	'Ppil6'	1.86	14.51	3.62	0.05	10.8	8.29	7.28	7.2	5.22	6.32
73078	'Pmpcb'	56.26	49.55	42.4	77.3	62.3	48.69	35.3	61.07	33.8	39.06
73086	'Rps6ka5'	1.41	0.1	0.09	0	1.36	0.12	0.28	0.74	0.36	0.02
73094	'Sgip1'	14.58	13.36	15.1	7.47	16.7	10.43	15.9	11.7	25.18	13.69
73095	'Slc25a42'	10.34	5.62	9.17	4.42	11.4	5.61	8.79	14.32	8.72	6.15
73102	'Slc22a23'	1.34	1.53	1.44	0.03	1.64	2.66	2.3	1.5	2.58	0.77
73103	'3110009E18Rik'	2.96	9.9	4.61	3.67	0.1	12.98	4.88	1.57	8.33	8.8
73106	'Prss57'	0	0	0	1.29	4.33	0	0	0.53	0	1.62
73112	'Abracl'	8.58	6.05	5.21	7.15	12.1	20.45	12	12.86	13.37	15
73121	'Rflna'	0	0	3.09	3.73	2.05	0	0	0	0	0
73122	'Tgfbrap1'	2.15	0.88	0.3	1.26	2.86	1.1	0.56	0.42	1.71	0.26
73124	'Golim4'	0.07	2.08	3.73	0	1.58	1.48	2.79	0.7	0.44	1.4
73130	'Tmed5'	21.24	33.28	11.1	8.98	9.9	22.66	24.8	12.11	18.01	27.27
73132	'Slc25a16'	0.1	0	0	10.7	2.64	0.08	1.97	0.07	6.22	0.2
73137	'Prrc1'	5.87	4.42	6.88	8.59	3.11	4.11	1.86	5.4	10.34	5.75
73139	'Cenpv'	9.98	3.5	1.97	8.59	3.81	4.95	0.05	1.86	1.42	2.65
73149	'Clec4a3'	0	0	0	0	0	3.54	0	0	0	0
73158	'Larp1'	7.15	3.32	3.73	4.57	6.11	2.57	5.25	3.49	4.78	3.39
73162	'Otud3'	0	0.14	1.68	4.99	0	0	0	0	0.03	0
73166	'Tm7sf2'	7.83	6.45	15.2	0.04	20.2	8.43	22.5	24.24	5.06	13.83
73167	'Arhgap8'	0	0	0	0	0	0	5	0	3.63	0
73172	'Exo5'	3.64	6.25	1.45	0	5.19	8.41	13.6	2.02	9.05	12.21
73173	'Pcdh18'	0.03	6.45	2.3	0.01	2.48	0	13.1	3.79	3.43	2.14
73174	'Tbkbp1'	3.43	0.53	1.58	5.23	0.5	1.46	3.62	0.75	3.58	1.16
73176	'3110040M04Rik'	0	0	0	4.36	0	0	0.26	0	0	1.13
73178	'Wasl'	19.01	19.27	23.6	26.4	20.1	31.55	12.7	12.14	16.43	7.38

73181	'Nfatc4'	0	0	0	0	0	0	0	0	0	0.77
73182	'Pear1'	0	0.16	0	0	0	0	0	0	0	0
73191	'Fezf1'	22.34	7.21	0.02	0.02	23.6	38.29	33.7	0.11	3.36	11.82
73192	'Xpot'	8.99	10.39	9.2	2.71	4.94	3.45	6.36	5.68	7.56	8.69
73205	'C9orf72'	7.45	8.49	8.05	3.79	14.6	9.74	22.4	18.74	10.51	14.58
73212	'3110082I17Rik'	46.88	23.93	21.4	28.5	31.5	29.65	20.3	21.03	25.22	20.92
73218	'Sppl2b'	15.32	8.59	23	10.1	8.04	12.96	18.2	13.34	0.21	4.18
73225	'Fam118a'	22.31	20.66	9.86	59.9	34.2	15.78	21.7	24.2	12.88	23.58
73229	'Zfp983'	8.57	5.35	10.1	0.01	11.2	10.27	5.98	10.88	11.02	13.13
73230	'Bmper'	0.19	0	8.33	0	0.01	0.4	2.88	2.36	5.86	0
73233	'Zfp942'	2.93	3.44	4.47	5.91	3.38	0.8	7.67	5.55	9.69	8.28
73234	'Snorc'	0	0	0	0	0	0	0	0	0	0.21
73242	'Atat1'	54.01	35.76	57	68.5	54	57.38	43.4	56.51	40.17	40.15
73246	'Rassf6'	0	0	0	0	4.54	7.1	0	0	2.24	0.84
73247	'Mrgbp'	5.44	0.97	10.8	9.49	1.73	0.68	0.11	7.84	4.94	0.02
73251	'Setd7'	0.98	0.96	1.05	2.21	1.68	0.07	0.16	0.4	0.73	0.41
732521	'Gt(pU21)140Imeg'	0	0.04	0.01	0.06	0.01	1.28	0	0	0.02	0.06
73254	'Ccdc18'	0	0	0.37	0	1.34	0	0	0	0	0
73259	'Cib4'	0	0	0.07	0	0	0	0	0	0	0
73261	'1700037C18Rik'	8.09	7.21	12.9	9.01	10	24.35	10.7	9.53	20.65	26.18
73274	'Gpbp1'	32.01	41.33	18.8	23.5	20.9	14.39	41.5	25.51	29.72	28.22
73284	'Ddit4l'	1.67	2.03	0	0	0	0	0	0	0	0.02
73287	'Cabcoco1'	6.68	2.58	0	0.05	3.54	0	4.84	2.32	0	2.4
73288	'Vps50'	15.71	23.1	22.2	22.8	19.9	6.92	33.4	15.47	20.77	30.69
73293	'Ccdc103'	1.87	2.55	4.3	2.39	5.66	4.92	4.7	4.59	7	3.26
73296	'Rhobtb3'	1.58	0.74	1.88	1.61	3.86	1.19	2.77	2.13	3.33	1.14
73314	'Lrrc69'	1.02	1.39	1.14	1.78	1.51	1.17	0.46	0.89	1.57	0.86
73316	'Calr3'	4.81	3.07	4.35	0	0.92	0.81	0	2.02	2.3	6.93
73318	'Ube2d2b'	0	0	0	0	0	0	0	0	0	0.13
73324	'Clhc1'	0	0.02	0.13	0.08	0	0	0	0	0	0
73327	'Pradc1'	0	9.15	7.49	0	16.9	0	15.8	6.66	0	0.05
73332	'Ccdc30'	19.22	20.75	23.7	17.1	14.1	32.67	8.07	12.31	8.53	13.23
73338	'Itpripl1'	0	0	10.6	2.46	1.02	3.73	0	2.19	4.18	1.36
73340	'Nptxr'	1.81	0.6	0	0	0.64	1.42	0.1	0.55	0.07	0
73341	'Arhgef6'	0	0.85	4.93	3.2	1.42	0.15	3.13	2.21	0.01	1.6
73363	'1700056E22Rik'	0	0	0	0	0	0	4.78	0	0	0
73368	'Col20a1'	0	0	0	0.22	0.01	0.03	1.26	0	0	0.01
73373	'Phospho2'	20.07	39.03	13.5	36.9	17.1	23.66	12.9	22.98	24.66	39.56
73379	'Dcbld2'	1.38	1.86	0.03	1.49	1.86	1.28	4.45	0.39	0.3	0.1
73381	'Cmtm2a'	0.17	0	0	0	0.8	0	0	0	0	0.41
73385	'Fam177a'	37.37	39.97	30.8	25.4	27	26.81	25.2	28.63	27.96	16.73
73389	'Hbp1'	14.69	17.74	16.7	31.5	15.6	18.85	24.9	9.37	14.94	36.15
73390	'Msl3l2'	1.46	3.14	3.37	10.7	5.61	0.23	7.67	3.56	3.26	9.57
73398	'Nipsnap3a'	0	0	0	0.11	0	0	0	0	0	0
73407	'Tepp'	5.28	1.11	5.43	3.88	1.63	6.54	0	3.63	0	0.08
73410	'1700065D16Rik'	0	0.1	0	0	0.1	1.68	0	0	0.28	0
73419	'Armt1'	6.14	5.84	9.9	6.87	0.09	11.43	32.6	6.93	4.16	3.11
73420	'Ccsap'	1.95	3.11	1.95	2.43	1.34	0.51	0.71	1.35	0.45	0.21
73422	'Prox2'	0.05	0.04	0	0	0.53	0	0	0	0.03	1.29
73430	'Zfp974'	1	0.14	0.1	2.49	1.45	1.96	0.3	1.53	1.71	2.29
73442	'Hspa12a'	6.78	7.88	18.2	10.9	8.01	7.64	28.4	15.33	6.55	7.42
73447	'Wdr13'	7.85	14.11	16.8	20.1	14.1	5.13	11.8	7.55	24.68	17.27
73449	'1700066B19Rik'	0	0	0.04	0	3.09	4.06	0	0.49	1.31	3.05
73451	'Zfp763'	8	1.53	8.48	10.4	7.49	1.42	0.03	14.05	5.55	5.28
73453	'1700067K01Rik'	0	0.4	0	0	0	0.04	0	0.08	0	0
73458	'Aldh3b3'	0	0.42	0	0	0	0	0	0	0	0
73467	'1700066M21Rik'	9.53	8.59	8.68	4.56	4.67	4.21	24.8	4.01	14.57	2.66
73469	'Rnf38'	2.45	1.97	2.97	1.08	4.49	1.12	0.77	1.91	5.07	5.05

73470	'Kif2b'	0	0	1.25	0	0	0	0	0	0	0
73473	'Iws1'	13.39	7.86	9.62	23.4	7.36	18.31	7.76	8.77	8.9	5.88
73490	'Mipol1'	2.07	2.74	5.18	0	6.89	1.7	3.2	3.97	5.6	0.33
73523	'Pebp4'	1.67	0	0	0	0	0.3	0	0	2.23	0
73526	'Speer4b'	0	0.2	0	0	1.3	0.09	0	0.32	0.02	0.16
73533	'1700080G18Rik'	0	0.6	0	0	2.11	0	0	0	0.6	0
73545	'1700094D03Rik'	2.83	5.21	3.26	0.1	0.64	3.66	2.28	1.72	6.08	0.55
73569	'Vgll3'	0	0	0	0	0	0	0	0.69	0	0.01
73582	'Camkmt'	21.39	28.27	21.7	3.55	20.1	17.31	10.2	19.2	31.71	42.63
73598	'1700001O22Rik'	0	0	2.41	0	1.1	4.62	3.03	3.01	6.31	4.32
73603	'Trp53tg5'	0	0.32	0	0	1.53	0	0.02	0	0.71	0
73608	'Marveld3'	0	2.23	0	0	1.94	0	1.87	0.65	4.78	0
73610	'Zfp433'	5.28	1.98	0	8.28	9.65	0	0	6.82	0.7	4.44
73634	'1700125H20Rik'	0.01	0	1.3	0.04	0.02	0.03	0.86	0.01	0.04	0.02
73635	'Ptges3l'	2.94	1.5	0.38	1.66	0.03	2.69	8.51	3.88	4.17	4.62
73647	'Capn9'	0	0	0	0	0	0	0	0.08	0	0
73649	'Cybrd1'	0.21	0.43	0.42	0.72	0.26	0.4	0.38	0.15	0.3	0.26
73656	'Ms4a6c'	4.29	1.72	3.59	3.45	3.03	1.89	0.8	0.82	1.95	1.1
73658	'Spns1'	22.33	33.36	48.4	6.4	27.8	36.31	29.7	34.64	24.61	24.49
73660	'Cabp4'	0	2.01	0	0	0	0	0	0	0	0.3
73666	'Thoc3'	63.62	40.17	37.1	89.5	49	54.14	50.5	40.37	39.73	44.43
73667	'2410004P03Rik'	0.03	13.66	3.54	6.12	3.25	0	0.06	5.72	0.02	2.35
73668	'Ttc21b'	5.32	4.17	5.31	0	6.67	5.38	0.4	4.33	2.98	2.4
73671	'Sult6b1'	0	0	0	0	0	0.02	0	0	0.02	0
73673	'Rec114'	0	0	6.09	2.07	2.27	0	5.22	0	0	0
73674	'Wdr75'	13.3	10.97	17.7	17.9	24	21.51	8.79	32.23	35.28	19.11
73677	'Psma8'	0.34	0	0	0	0	0	0	0	0	0
73680	'Zbtb8a'	3.16	0	0.94	0.08	1.23	0	0.29	0.05	2.44	4.07
73681	'Trmt11'	11.07	5.91	5.47	10.4	8.23	9.64	4.87	11.48	14.11	8.66
73683	'Atg16l2'	10.98	14.08	29.4	18.5	12	33.6	20.1	21.06	10.42	27.55
73689	'Bloc1s2'	125.7	95.81	90	53.4	63.1	114.6	108	113.8	78.94	91.24
73690	'Glipr1'	0	0.1	0	0	0.06	7.37	0	1.51	0	0
73692	'Cplane1'	2.57	3.07	3.48	9.88	4.11	2.87	0.58	3.35	3.09	5.39
73694	'Ndufaf7'	39.85	54.37	36.5	33.6	34	47.78	58.6	54.88	61.02	46.58
73699	'Ppp2r1b'	4.24	2.56	13.6	6.46	0	0.06	2.97	1.26	11.97	1.1
73703	'Dppa2'	0	0	0	0	0	1.08	0	0	0	0
73707	'Gucy2g'	0	0.61	0	0	0	0	0	0	0	0
73710	'Tubb2b'	46.45	32.9	47.4	14.1	39.3	48.42	37.1	48.16	44.26	44.09
73711	'Mvb12a'	71.26	33.68	63.9	54.8	46.2	66.05	44	44.27	47.07	43.71
73712	'Dmkn'	0	6.14	0	10	0	0	0	5.36	0	0
73713	'Rbm20'	1.75	0.17	0.04	0.02	0.94	0.01	0	0.01	0.01	0.01
73720	'Cst6'	3.16	2.53	2.82	0	2.09	2.35	5.12	0.56	3.51	4.67
73721	'1110017D15Rik'	31.57	24.05	2.88	61.7	10.4	42.12	10.5	14.95	26.77	13.1
73723	'Sh3bgrl3'	291	276.5	263	249	372	530.3	386	345.9	241.2	270.67
73724	'Mcee'	67.43	54.14	59.1	121	58.1	48.09	11.2	53.78	22.79	41.3
73728	'Psd'	31.92	26.03	20.5	24.8	19.5	20.07	34.4	11.99	22.56	4.66
73729	'Zfp383'	7.2	1.55	5.72	0	3.36	3.41	3.63	5.13	7.26	6.14
73730	'Lce1l'	0	1.84	0	0	0	0	0	0	0	0
73736	'Fcf1'	35.1	51.25	36.8	117	63.4	84.5	42.7	47.42	55.59	66.95
73737	'1110008P14Rik'	142.7	121.5	117	144	106	103.6	66.8	154	127.9	175.6
73738	'Haus7'	13.57	13.95	16.9	24.4	10.9	1.45	6.64	30.73	11.35	24.03
73739	'Cby1'	97.63	53.33	43.9	51.3	80.7	93.33	59.4	94.78	44.62	59.62
73744	'Man2c1'	8.65	22.91	25.6	33.4	23.3	2.84	16.3	8.65	20.18	14.08
73747	'Shld1'	6.34	6.48	4.85	0	10.6	4.17	25.9	0.04	24.34	7.92
73750	'Whrn'	8.66	0.35	1.12	0	8.98	0.01	4.16	5.86	3.32	0.51
73754	'Thap1'	8.93	1.95	1.9	2.6	7.76	6.99	6.75	7.71	3.56	2.1
73804	'Kif2c'	0	0	0.46	0	0	0	0	0	0	0
73813	'Fam83e'	0.68	0	0	0	0	0	0	0	0.01	1.53

73822	'Mfsd12'	5.81	5.72	7.45	0.2	2.71	1.69	8.07	8.27	7.06	2.14
73825	'Ppp1r21'	9.28	6.83	8.94	24.7	3.13	4.86	9.77	12.19	11.3	7.39
73826	'Poldip3'	26.43	24.66	33.9	21.9	21.7	21.66	23.6	19.96	23.82	19.73
73827	'Tmem198b'	2.82	0.34	7.72	2.47	2.01	4.56	5.32	5.18	4.19	0.14
73828	'Dcaf4'	11.51	24.38	29.5	18.4	23.1	37.39	33.6	32	18.16	16.07
73830	'Eif3k'	155.6	192.9	182	261	226	280.4	163	186.1	184	187.03
73833	'Fam98c'	15.74	24.64	29.7	0	23.1	20.74	16	12.54	10.07	2.82
73834	'Atp6v1d'	299.4	316.8	325	295	327	296.7	294	391.5	349.1	343.79
73836	'Slc35b2'	30.63	21.59	46.6	9.68	20.9	15.01	45.8	20.21	30.16	13.68
73844	'Ankrd45'	11.26	18.39	8.21	6.9	8.69	5.78	4.65	6.77	8.22	9.26
73845	'Ankrd42'	6.82	4.69	7.77	8.63	10.3	11.22	23.9	7.63	12.09	20.66
73847	'Fam110a'	17.83	25.07	28.2	10.9	4.01	28.14	24.2	22.26	22.35	23.17
73852	'D3Ertd751e'	11.99	14.21	21.4	0.96	21	11.43	22.1	14.26	15.61	27.09
73863	'4930415O20Rik'	0	0.28	0	0	0	0.27	0	0	0.08	0
73873	'Fam161a'	3.94	3.87	0.73	1.17	0.71	3.62	5.13	1.46	1.9	3.6
73884	'Zdbf2'	3.11	2.24	2.41	3.38	1.34	3.46	2.7	0.98	5.54	2.76
73893	'Tmem202'	3.95	6.34	0	10.1	0	14.44	7.89	4.44	4.72	1.51
73902	'Psmb11'	0.01	0.01	0.09	0.2	0.06	0.1	0.03	0.03	0.1	0.05
73910	'Arhgap18'	0.07	5.34	0.88	7.4	0.51	1.49	2.33	4.53	0.32	3.05
73916	'Ift57'	11.6	4.21	4.35	17.3	9.62	7.98	3.57	9.1	10.42	3.37
73919	'Lyrm1'	28.33	17.31	17.6	14.1	35	9.46	24.4	30.88	30.91	18.68
73936	'Ccdc175'	0	0	0	0	2.49	0	0	0	0	0
73940	'Hapln2'	3.65	6.19	2.3	0.03	10.1	25.88	2.93	0	1.48	1.87
73942	'Fam151b'	0	0	0	0	0	0	0	0.03	0	0
73945	'Otud4'	2.38	1.39	2.43	3.99	4.72	0.77	3.24	1.34	1.64	0.65
73991	'Atl1'	91.09	80.86	69.1	27.5	49.1	54.25	48.1	72.27	70.3	47.14
73998	'Herc3'	11.58	8.67	3.05	13.5	14.2	7.48	8.42	10.84	6.84	12.77
74002	'Psd2'	7.97	11.21	19.5	17.5	15.7	7.45	9.65	5.84	10.77	3.45
74004	'Jakmip3'	1.06	5.06	1.55	0.07	0.67	3.1	3.31	1.72	2.05	2.64
74006	'Dnm1l'	65.41	40.27	77	80.3	34.7	42.1	65.7	54.89	73.04	72.2
74007	'Btbd11'	13.33	7.25	5.22	6.09	5.82	0.58	12.8	7.53	15.88	8.01
74008	'Arsg'	0	8.95	4	7.69	4.05	9.36	4.63	0.38	6.57	1.14
74011	'Slc25a27'	14.36	20.44	24.9	9.13	17.8	9.41	11.8	20.89	35.05	2.65
74012	'Rap2b'	10.86	10.55	9.1	14.8	13.5	9.74	10.4	10.6	4.22	5.34
74013	'Rftn2'	5.65	0	4.26	6.5	0.02	3.53	10	6.09	6.39	0.13
74015	'Fcho1'	33.02	24.32	18.8	4.61	35.9	21.91	35.2	32.24	50.69	21.02
74016	'Phf19'	0	0	0.01	0	0	0	0	0	0	0
74018	'Als2'	1.47	4.24	4.69	8.95	1.9	3.37	4.56	0.72	3.02	1.3
74019	'Traf3ip1'	1.58	5.31	1.9	9.54	2.4	0.02	1.4	3.08	4.92	0.92
74020	'Cpne4'	11.04	5.48	18.9	6.45	8.78	7.59	0	4.84	0.01	2.55
74022	'Glyr1'	61.84	52.97	86.3	36.3	35.5	29.75	72.2	49.78	54.54	59.57
74023	'Rd3'	0	1.56	0	0	24.3	0	0	0.12	0	0
74025	'Nphp3'	0.86	0.05	0.58	3.32	2.27	4.01	0.77	0.38	1.36	0.76
74026	'Msl1'	5.01	13.57	5.53	12.1	8.94	13.99	3.42	5.18	9.84	5.76
74030	'Rin2'	2.85	1.53	2.62	3.03	4.09	0.8	17.6	2.03	0.28	0
74032	'Sdr42e1'	0	13.66	9.28	0	4.81	11.45	10	16.82	0	6.3
74035	'Nol9'	3.24	1.51	3.03	0.07	0.11	8.37	3.47	2.61	4.86	1.56
74039	'Nfam1'	0.02	0	0.04	0.04	0	1.55	0	0	0	0.01
74041	'Ddias'	0	0	0	0	0	0.63	0	0	0	0
74043	'Pex26'	1.67	4.84	1.89	0.02	2.09	0	4.42	3.33	5.24	1.66
74044	'Ttf2'	0	0	0	0	0	0	0	0.64	1.89	0
74048	'Vsir'	0	0.83	3.07	0	1.19	4.31	0	0	1.07	0
74051	'Steap2'	1.13	0.29	5.5	2.27	0.09	0.51	1.82	2.08	0.48	1.46
74052	'Ttc21a'	1.14	0	0	2.17	0	0.97	0.19	0.34	0	0
74053	'Grip1'	1.74	2.81	3.5	0.72	4.54	2.94	4.59	4.44	4.96	0.89
74055	'Plce1'	0.02	0.83	0	0	0	0	0.82	0	0	0.32
74071	'Lmntd1'	0.02	0.03	1.81	0	0	0	0	0	0	1.29
74075	'Syce1'	0	0	0	8.63	0	0.41	0	0	0	0

74080	'Nmnat3'	10.86	3.14	0	0.39	0	4.21	0	5.25	6.45	2.6
74081	'Cep350'	0	1.26	1.28	3.14	1.66	0.12	1.23	0.85	1.06	0.8
74090	'Paqr5'	0	0	0	5.97	0	0	0	0	0.79	0.41
74091	'Npl'	2.78	6.02	0	1.32	0	5.24	3.84	3.81	0	3.4
74094	'Tjap1'	7.22	5.78	5.53	0.02	2.3	7.35	9.11	8.04	4.36	0.77
74096	'Hvcn1'	0.02	0.21	0	0	1.46	4.75	0	0	0.09	0
74097	'Pop7'	54.96	55.5	47.4	41.1	75	46.67	49.3	62.38	25.67	68.69
74098	'0610037L13Rik'	45.06	40.53	53.8	41.8	60.9	34.79	76.9	50.49	33.62	68.28
74100	'Arpp21'	15.07	13.95	8.48	18.1	56	28.35	23.4	29.72	8.24	4.62
74102	'Slc35a5'	8.95	6.67	6.91	4.56	5.46	6.75	7.64	13.26	17.49	16.03
74103	'Nebl'	18.23	8.37	20.2	20.8	8.45	6.07	14.4	5.61	11.7	4.67
74104	'Abcb6'	5.96	14.88	17.7	0	11.2	19.36	8.22	13.42	8.14	16.91
74105	'Gga2'	2.16	3.44	1.39	4.96	4.98	4.45	5.09	7.12	7.01	3.99
74106	'Dcaf6'	5.97	8.05	4.95	0.56	6.17	1.59	4.51	6.73	1.09	1.23
74107	'Cep55'	0	0	0.09	0	0	0	0	0	0	0
74108	'Parn'	8.24	24.36	24.1	19.1	14.3	10.43	20.7	16.5	19.53	24.61
74111	'Rbm19'	1.6	2.46	1.21	4.37	3.39	2.01	7.36	1.48	9.77	4.02
74112	'Usp16'	6.73	4.5	5.22	13.2	5.71	5.74	9.77	5.54	7.3	12.65
74114	'Crot'	10.06	5.24	15.9	0	3.07	14.84	10.9	9.9	20.49	10.55
74116	'Pi16'	19.56	21.69	14.5	11.3	12.5	32.12	3.45	12.38	14.06	32.65
74117	'Actr3'	52.62	44.93	46.7	53.7	49	32.24	45.6	58.19	50.22	74.21
74120	'Zfp263'	6.52	22.86	18.5	5.76	30.3	7.55	5.02	16.61	23.99	11.58
74121	'Acoxl'	0	0	0	0.43	0	0	0	0.31	0	0
74122	'Tmem43'	21.79	34.84	31.6	33.7	20.5	17.91	25.4	39.05	30.68	52
74123	'Foxp4'	6.73	10.18	16.6	5	4.44	4.29	7.6	10.2	23.87	1.5
74125	'Armc8'	53.13	45.82	37.1	42.2	18.1	34.65	16.3	39.41	55.52	43.86
74126	'Syvn1'	7.24	11.05	9.24	10.8	13.4	6.17	9.46	13.49	18.87	15.97
74129	'Dmgdh'	0	1.37	0	0	0	0	0.27	0.11	0	0.2
74131	'Sash3'	1.13	0	0	0	0	0	0	0	0	0
74132	'Rnf6'	33.95	47.21	28.8	28.5	27.6	54.66	35	42.74	34.47	37.64
74133	'Smg8'	1.82	6.15	5.26	0	4.73	4.53	7.07	3.37	3.5	8.22
74134	'Cyp2s1'	2.84	0	8.41	0.02	0	6.05	0	1.97	0	0
74136	'Sec14l1'	4.61	8.25	4.5	3.85	1.5	5.68	7.81	7.26	2.2	7.04
74137	'Nuak2'	0	1.78	0	0	0	2.15	0	1.93	5.49	0
74140	'Tm9sf1'	15.54	22.94	39	4.77	26.5	19.88	21.3	18.28	33.69	22.8
74142	'Lonp1'	8.83	9.47	6.39	21.4	24.6	8.2	8.39	10.54	7.26	17.3
74143	'Opa1'	9.5	6.54	13.7	2.45	4.6	2.37	12.9	7.48	7.92	14.08
74147	'Ehhadh'	0.02	0	0	0	0	0.02	4.85	0	0	0.94
74148	'Cluh'	8.02	5.34	19.8	8.66	6.82	5.07	0.44	5.16	7.8	2.95
74149	'Zfp946'	6.7	6.14	7.84	0	6.46	3.16	6.78	13.38	2.14	8.06
74150	'Slc35f5'	19.36	15.54	9.64	10.4	4.2	13.02	16.1	22.47	4.57	18.57
74152	'Stra6l'	0	0	0	0.02	0	0	8.23	0	0	0.01
74153	'Uba7'	0	0	0	0	0	10.29	0.2	0	0	2.62
74154	'Unkl'	20.31	10.23	11.4	5.86	14.5	3.2	10.9	13.71	19.42	26.12
74155	'Errfi1'	10.79	15.55	16.1	10.4	14.5	15.12	19.4	11.04	3.23	5.48
74156	'Acot12'	0	1.87	0	0	0	0	0	0	0	0
74157	'Cmtr1'	42.06	44.58	52.6	42.7	26.9	23.29	42.3	36.34	54.36	47.28
74158	'Josd1'	4.66	1.92	1.29	4.36	7.92	1.01	5.26	5.41	5.38	10.01
74159	'Acbd5'	15.31	12.64	16.8	5.12	13.4	9.76	12.7	7.53	11.26	14.18
74164	'Nfx1'	30.45	37.69	38.4	32.7	23.6	22.01	50.9	25.63	32	31.74
74165	'Fbxl22'	3.01	0.36	0.06	0	3.61	0.04	0.03	0.5	0.01	0.5
74166	'Tmem38a'	16.94	42.04	70.9	6.78	26	36.14	37	42.78	36.3	40.4
74167	'Nudt9'	39.6	22.03	19	40.8	18.1	12.81	17.7	17.32	26.79	25.15
74168	'Zdhhc16'	27.48	14.94	15.8	25.8	24.8	21.55	42.7	29.54	9.31	34.85
74174	'Gtsf1'	0	0	0.29	0	0	0	3.4	0	0	0
74178	'Stk40'	0.14	3.4	4.26	5.34	1.9	0	1.45	0.31	0.01	0.19
74180	'Muc5b'	0	0	0	0	0	0	0	0	0	0
74182	'Gpcpd1'	13.99	15.19	22.2	8.95	18.5	11.69	5.22	22.29	21.33	31.03

74183	'Perm1'	0.3	0.22	1.19	0.01	1.55	0.24	0.08	0	0.46	1.15
74185	'Gbe1'	1.88	7.33	4.83	0	2.35	0.8	2.74	4.69	2.21	9.5
74186	'Ccdc3'	0	0	0	0	0	0	0	4.96	0	0.02
74187	'Katnb1'	36.72	24.3	46.9	44	17.2	22.62	36.5	32.1	57.47	44.83
74189	'Phactr3'	11.95	17.94	12.5	26	10.7	9.97	10.7	18.99	13.13	10.65
74190	'Exoc3l4'	1.54	2.88	0	2.77	0	2.82	2.4	1.01	0.38	5.08
74191	'P2ry13'	0	0	0	0	0	17.05	0	0	0	0
74192	'Arpc5l'	123.2	143.9	122	195	215	237.5	164	206.6	175.3	188.6
74194	'Rnd3'	9.85	10.18	9.87	36	2.33	6.57	7.01	11.61	10.07	3.9
74195	'Elp3'	45.42	51.88	53.6	43.6	40.7	38.36	49.5	46.23	52.6	46.16
74196	'Ttc27'	2.14	12.9	8.6	0.71	12.1	7.08	4.69	9.72	0.03	7.21
74197	'Gtf2e1'	2.58	9.69	7.36	13.3	14.9	1.26	7.2	3.83	8.7	7.67
74198	'Dtx2'	3.23	1.05	6.53	6.74	0	0.32	0	0	0	0.73
74199	'Vit'	2.25	1.58	0.01	0.04	0	0	4.24	0	0.59	0
74200	'Khdc4'	18.12	19.74	16	6.63	6.94	8.89	15	23.68	23.26	29.2
74201	'Cep97'	3.49	0.68	1.02	0.51	2.8	0	0.46	1.8	0	1.13
74202	'Fblim1'	0.03	0.19	0.03	0.02	0.02	0.01	0.02	0	0.91	0.01
74203	'Eif4enif1'	6.43	9.03	4.11	4.91	13.8	2.83	4.98	6.68	9.75	6.65
74204	'Xpo6'	30.17	98.04	9.7	41.1	90.4	16.55	7.34	10.61	10.86	23.16
74205	'Acsl3'	10.8	19.52	7.47	22.4	11.4	13.54	9.27	17.72	22.16	7.51
74206	'Sipa1l3'	0.02	0.82	0.2	3.4	0.97	0.01	0.02	0.51	1.09	0.22
74211	'1700017B05Rik'	3.13	1.13	0.08	0	5.24	6.69	5.47	3.04	2.12	7.24
74213	'Rbm26'	10.59	19.42	10.2	15.7	21.2	8.79	7.67	10.01	17.4	12.64
74229	'Paqr8'	10.83	6.3	3.79	6.27	5.72	4.66	5.17	2.89	3.03	2.76
74230	'1700016K19Rik'	0	0.06	0	5.57	1.39	5.87	0	0.38	0	4.6
74237	'Tubgcp2'	22.72	27.89	30.1	29.6	24	20.33	17.9	20.14	22.32	23.77
74238	'Mterf2'	12.58	18.88	14.2	29.2	10.7	7.66	14.5	26.57	12.35	8.2
74239	'Iqce'	4.27	9.83	11.4	2.49	12.5	10.5	11.8	3.89	8.78	13.14
74241	'Chpf'	13.34	14.09	12.9	5.65	23.6	13.49	8.78	8.85	23.89	4.56
74243	'Slx4ip'	4.04	5.12	0.46	1.97	7.83	2.43	0.35	1.65	1.12	1.63
74244	'Atg7'	9.84	9.82	20.2	4.94	2.28	0.95	4.47	6.58	10.6	9.15
74245	'Ctbs'	1.84	0.05	1.05	5.92	1.85	1.51	1.97	0.01	0	1.29
74246	'Gale'	18.26	28.25	21.1	8.64	17.8	19.8	11.9	29.39	25.47	23.87
74249	'Lrrc2'	1.62	0.48	0.16	0.14	0.2	0.07	2.81	0.01	0.1	1.17
74251	'Ankrd9'	4.81	7.13	10.3	0.04	6.39	22.56	14.3	7.87	8.57	9.1
74252	'Armc1'	44.6	38.11	37.2	20	24.5	46.13	28.2	36.6	40.4	28.27
74253	'Klrg2'	0	0	0	0	0	0	0	0	0	0.45
74254	'Gpn1'	46.58	65.42	68.5	69.4	38.1	67.61	60.1	64.77	52.25	86.26
74255	'Smu1'	62	68.8	54.8	74.1	36.5	22.42	48.6	49.07	46.61	76.02
74256	'Cyld'	13.47	14.36	13.8	13.4	11.3	6.83	21.9	8.09	13.69	12.21
74257	'Tspan17'	35.61	20.44	31	54.6	28.9	22.16	17.9	30.18	44.02	19.52
74264	'Rnf138rt1'	12.51	9.89	10.7	78.1	5.32	16.63	19	11.26	12.51	31.53
74268	'Aven'	0.34	0.1	0	0	3.18	3.79	0	3.32	0.98	0
74270	'Usp20'	37.43	42.2	48.8	6.31	32.4	15.27	29.7	46.02	44.69	26.3
74277	'Chic2'	0	0.3	0.47	9.37	0	0.07	1.5	0.61	0	0
74281	'Spatc1'	0	0	0	0	0	0	0	0	0	0.08
74286	'Tbc1d21'	0	0	0	0	0	0.22	0	0	0	0
74287	'Kcmf1'	4.53	2.21	4.43	7.73	9.99	1.34	6.88	1.99	3.55	5.43
74302	'Mtmr3'	1.05	4.72	7.1	11	8.64	9.02	3.96	0.34	2.35	5.64
74307	'1700092M07Rik'	0	0	0	0	0	0	0	0	0	1.06
74309	'Osbp2'	0.65	3.72	11.4	3.72	2.86	0.01	6.31	7.22	4.34	2.52
74315	'Rnf145'	17.45	13.99	5.35	32.3	20.5	6.69	13.5	17.33	21.59	7.05
74316	'Isca2'	132.6	81.39	99.6	105	147	197.1	48.7	145.3	113.8	102.07
74318	'Hopx'	0	0	1.24	0	4.17	0.09	1.68	0	0.07	0.84
74319	'Mettl23'	21.59	21.01	12.6	23.4	6.44	11.55	30.8	6.79	9.68	22.29
74320	'Wdr33'	20.86	37.56	29.9	43.5	30.2	23.37	20.1	16.72	27.63	43.25
74322	'Cxxc1'	26.81	16.19	22.1	13	20.5	8.73	8.2	17.54	31.12	14.97
74325	'Cltb'	183.1	155.4	179	124	190	154.6	182	166.5	229.8	130.73

74326	'Hnrnpr'	74.48	84.17	76.5	80.3	54.7	56.17	54.8	71.97	46.76	74.07
74330	'Dnajc14'	14.06	10.54	17.3	6.2	8.4	10.92	4.8	16.4	13.43	31.44
74334	'Ranbp10'	8.4	4.84	1.5	5.97	0.6	2.26	3.32	0.35	2.62	1.18
74335	'Xrcc3'	14.72	6.28	16.2	0.02	26.3	0	0.04	35.34	11.42	0
74337	'Palm3'	0	1.5	0.66	0	0	1.5	0	1.32	0	1.51
74338	'Slc6a19'	0	0	0	0	0	0.01	0	0	0	0
74340	'Ahcyl2'	9.87	4.88	2.66	9.96	2.94	0.99	9.75	2.39	1.1	4.85
74342	'Lrrtm1'	20.48	24.17	47.9	7.43	58.8	41.26	43.4	56.65	41.73	13.56
74343	'Crtc2'	10.22	6.79	16.4	17.4	4.48	5.44	6.2	6.89	8.29	0.12
74347	'Tldc1'	4.34	4.75	1.06	2.62	0	0.02	0	1.07	2.55	0.02
74349	'Fam160a2'	3.64	3.8	3	2.59	3.28	1.94	6.51	2.23	3.74	3.73
74351	'Ddx23'	7.66	9.09	4.75	2.01	0.88	0.9	5.02	4.32	4.34	10.65
74352	'Zfp84'	1.03	0	1.5	4.15	3.01	2.98	1.87	1.39	3.43	3.97
74354	'Lrguk'	2.05	1.89	3.44	4.77	0.37	1.62	0	0.49	0.26	0.6
74355	'Smchd1'	2.77	2.88	5.32	10.1	1.92	5.77	2.01	1	3.31	2.64
74356	'4931428F04Rik'	12.86	6.19	12.5	3.76	2.34	4.86	18.4	13.15	20.7	10.67
74359	'4931414P19Rik'	0.87	1.29	3.36	2.99	1.76	1.99	0.52	0.45	4	0.45
74360	'Cep57'	13.9	6.19	14.1	12.1	5.06	11.57	17.1	8.86	13.81	12.67
74361	'4931429L15Rik'	0	0	0	0.24	0	0	0	0	0	0
74362	'Spag17'	0	0	0	0	0	0	0	0.06	0.21	0
74365	'Lonrf3'	0.64	0.99	1.06	2.96	1.77	0.92	2.1	0.01	0.01	0
74369	'Mei1'	0	0	0	0	0	0	0	0	0	0.03
74370	'Rptor'	1.75	2.28	6.49	0.43	9.23	1.96	5.82	3.71	2.5	8.74
74374	'Clec16a'	38.13	46.62	51.8	60.9	24.3	28.74	33	40.79	30.68	39.45
74375	'Gcc1'	2.65	10.16	5.21	9.22	8.9	2.71	11.1	4.85	2.27	1.93
74376	'Myo18b'	1.03	0	0.41	1.56	0	0.02	1.6	0	0.01	0.01
74377	'Hsf2bp'	0	4.7	0.06	0	0	0	3.28	0	0	0
74383	'Ubap2l'	32.28	25.84	26	33.4	39.4	20.09	29.3	42.15	33.81	44.63
74385	'Ap5m1'	6.32	4.81	7.11	5.69	7.28	1.95	12.6	10.23	14.84	6.57
74386	'Rmi1'	9.04	6.21	10.3	0.62	6.86	3.16	0.01	6.11	7.1	7.05
74387	'4932438H23Rik'	1.55	4.7	0.08	1.72	0	0.02	0.98	0.02	1.84	5.56
74388	'Dpp8'	5.61	1.31	2.41	5.88	1.72	0.47	12.6	3.87	3.97	3.57
74392	'Specc1l'	0.91	1.6	5.49	3.69	7.59	3.63	3.85	5.25	8.42	0.76
74393	'Map10'	2.97	3.29	3.91	0.35	0	0.01	1.1	1.48	5.05	2
74400	'Zfp819'	0	7.02	0.05	0	0.02	4.95	0	0	0	3.64
74405	'Efhc2'	0.08	2.14	0.28	4.96	1.7	1.22	0	2.1	3.75	7.27
74407	'Ttc25'	0	0	0	0.19	0.7	2.5	0.17	0	5.93	2.37
74410	'Ttll11'	1.53	1.83	4.2	0	3.76	0.01	4.19	0.05	4.45	0.73
74411	'Plpp6'	2.83	3.34	2.83	1.65	3.34	1.74	6.2	1.94	3.09	5.33
74412	'Gle1'	35.84	20.64	33.6	23.6	26.2	12.57	17.6	18.68	29.15	29.52
74413	'Tc2n'	0.23	0.03	0.04	0.13	0.14	0.34	0.2	0.25	0.16	0.46
74414	'Polr3c'	26.49	19.44	18.5	25	9.35	5.39	28.1	12.7	12.73	22.32
74419	'Tktl2'	0.01	0	0	1.69	0	0	0	0	0	0
74426	'4933402D24Rik'	0	0.28	0	0	0	0	0	0	0	0
74427	'Eaf1'	3.36	0.85	6.38	0.22	0.07	0.3	2.02	1.92	8.13	3.39
74430	'4930452B06Rik'	0.64	3.37	0.7	0	0	0.44	4.84	7.4	0	0
74435	'Lrriq3'	0	0	0.31	0	2.05	0.04	0	0	0	0
74438	'Clvs1'	12.32	13.73	15.2	1.89	18.8	7.75	0.07	19.62	24.89	14.6
74440	'Cmip'	87.64	118.9	66.5	89	84.9	112.8	117	92.12	83.02	91.35
74442	'Sgms2'	0.25	1.15	0.64	0.29	0.01	0	0	0.01	0	0.13
74443	'P4htm'	99.26	74.91	124	91.6	87.5	86.76	96.4	76.4	74.41	82.06
74446	'Slc9b1'	0	0	0	0	0	0	0	0	0	0.19
74448	'Arl13a'	0	0	0	0	0	0	0	0	0	0.1
74450	'Pank2'	0.6	2.27	0.06	3.04	4.31	3.15	1.06	5.26	0	0.48
74451	'Pgs1'	19.1	25.14	32.3	44.3	31	25.54	38.2	13.41	27.12	10.08
74453	'Cfap53'	0	0	0	0	0	0	0	0.55	0	0
74455	'Nsun6'	6.12	6.9	3.97	0.49	2.65	3.7	0	4.92	6.8	0.4
74464	'Zswim5'	0	0.09	2.33	2.92	0	1.22	0	0.33	0	0.11

74466	'Mfsd13b'	0	1.91	0	0	0	1.27	0	0	0	0
74467	'Pus10'	1.08	3.32	2.67	3	4.78	1.44	1.14	2.4	6.48	8.02
74469	'Taf7l'	0.1	0.07	0	0	0	0	0	0	0	0
74470	'Cep72'	0	0.63	0	6.76	0	0	0.2	0	0	0.24
74471	'4933440N22Rik'	0	0.22	0.05	0	0	1.24	0	0	0.28	0
74477	'4933427D14Rik'	8.89	6.74	5.54	13	2.36	6.22	14.5	4.2	4.93	15.37
74478	'Snx29'	2.05	2.79	2.47	4.61	5.88	10.78	6.91	5	5.78	3.46
74479	'Snx11'	16.14	5.61	8.54	7.62	9.41	6.94	3.73	19.79	14.61	6.97
74480	'Samd4'	3.87	2.06	4.44	7.26	6.9	6.65	4.46	2.52	8.21	3.31
74482	'Ifitm7'	1.87	1.86	3.64	13	2.78	5.22	2.2	2.76	2.74	2.59
74485	'Lrrc71'	2.23	0.72	0	0	0.73	0	0	0.2	1.8	0
74486	'Osbpl10'	0.57	0	5.55	4.74	6	0.79	0.02	4.38	7.18	3.23
74490	'Mamstr'	0	0	1.28	2.97	0	0	0	0	0	1.74
74492	'Kbtbd13'	0	0	0.24	0	0	0	0	0	0	0
74493	'Tnks2'	3.61	2.42	0.66	2.27	2.82	1.39	2.09	0.59	2.72	2.93
74498	'Gorasp1'	13.97	27.48	29	1.82	11.7	22.43	29.5	28.03	19.22	17.05
74504	'Fam53a'	19.23	30.08	27.3	13.7	22.1	11.49	25.4	28.3	27	25.6
74513	'Neto2'	3.39	1.15	0.74	3.95	3.06	1.7	0.55	0.97	0.65	1.07
74519	'Cyp2j9'	14.74	14.15	17.7	28.9	5.64	36.32	10.8	11.68	7.79	7.01
74521	'Ppp4r4'	1.44	4.17	2.27	7.21	2.01	2.15	0	3.01	3.14	4.64
74522	'Morc2a'	1.48	4.72	6.6	10.9	1.07	1.17	9.38	2.88	1.33	4.12
74525	'Fam234b'	60.1	61.98	93	10	62.8	66.48	79	75.52	88.89	104.66
74528	'Mgme1'	2.23	7.64	0	6.75	3.26	5.87	3.56	5.38	7.49	3.42
74533	'Gzf1'	14.85	5.39	8.2	0	4.08	17.64	15.5	8.44	3.47	10.19
74548	'Gsdmc4'	0	0.02	0.02	0	0	2.19	0	0	0	0.1
74549	'Mau2'	24.98	29.31	35.1	20.7	14.8	18.37	42.7	25.96	10.78	43.81
74551	'Pck2'	6.66	13.73	13.2	9.1	12.6	22.46	23.6	23.74	24.22	13.97
74552	'Nipal3'	6.83	11.15	6.89	2.66	10.7	8.49	16	9.22	10.91	6.07
74558	'Gvin1'	0	0	0	0	0.36	0.21	0	0	0	0
74559	'Elovl7'	0	1.26	0.03	0	0.94	2.89	0	0.65	0	0.31
74561	'Nkx6-3'	0	0	0	0.33	0	0	0	0	0	1.12
74563	'Rasgef1c'	9.94	5.17	3.19	3.22	1.68	0	9.55	3.97	0.02	3.43
74568	'Mlkl'	0	0	0	0	0	0.16	0	0	0	0
74569	'Ttc17'	3.97	2.61	12.2	2.52	6.11	12.5	9	6.71	7.32	12.75
74570	'Zkscan1'	3.43	3.48	4.21	1.86	3.68	5.16	2.5	2.04	6.31	1.92
74577	'Glb1l'	6.3	1.95	2.55	2.86	0.81	1.05	0.62	1.37	5.52	0.03
74580	'Pyroxd2'	0	0	0	0	0	0.96	0	0	0.12	1.3
74585	'Sppl3'	7.13	5.02	7.45	6.66	4.46	1.11	18.4	5.39	17.41	7.97
74589	'Kbtbd12'	0	0	0	0	0	0.02	0	1.63	0.46	1.84
74596	'Cds1'	1.4	0	0.43	7.33	3.46	0.98	1.57	3.82	6.44	5.48
74600	'Mrpl47'	21.95	36.76	39.8	0.21	33.5	55.68	32.4	28.81	18.59	23.28
74610	'Abcb8'	32.75	21.07	23.3	14.3	39.3	33.74	27.7	26.96	23.44	33.38
74616	'Scrn3'	5.88	6.2	4.47	3.31	3.72	5.22	14.2	2.09	5.81	2.29
74617	'Scpep1'	24.65	29.85	22.5	21.6	16.8	16.89	32.3	26.66	57.85	65.09
74637	'Shpk'	0.25	0	0	0	2.48	0	9	4.99	0.02	3.29
74644	'4930426D05Rik'	0	0	0.95	0	0	0.8	0	0	0	0
74645	'Tent5c'	0	2.94	0	8.46	0	1.76	0	0	0	0
74646	'Spsb1'	1.17	1.23	1.82	15	0.08	0.3	0	2.15	0	0.01
74648	'S100pbp'	7.51	6.26	4.71	10.6	7.96	4.19	7.91	2.92	7.53	11.78
74649	'Cpa5'	0	0	3.2	0	0	4.66	0	0	0	0
74653	'Pomk'	29.33	24.24	24.4	25.5	20.9	33.74	39.9	22.7	23.14	24.71
74665	'Drc3'	18.96	5.83	13.4	0	19.3	13.15	6.83	6.24	1.23	5.28
74666	'4930432K21Rik'	7.47	5.21	3.83	0	0.02	1.62	7.56	2.91	0.06	18.18
74670	'Zfp943'	2.72	7.59	3.14	2.07	8.85	6.01	3.51	5.54	4.84	5.05
74682	'Wdr35'	26.93	18.25	18.7	12.8	17.6	3.92	13	26.01	21.78	17.71
74685	'Lrrc74b'	0	0	0.07	0.25	0	0	0	0.02	0.04	0
74691	'Tdrd9'	0	0	0.98	0	0.31	0	0	0	0	0
74694	'Tbc1d30'	3.03	6.14	4.41	1	0.19	2.36	0	0.64	4.37	1.48

74711	'Ttll9'	2.34	4.96	2.4	8.95	5.22	11.98	0.03	2.84	2.51	3.6
74717	'Spata17'	0.38	0	0	0	0.54	0.14	0	0.66	0	0
74718	'Snx16'	39.56	22.04	17.9	33.9	18.8	32.64	27.6	39.71	15.83	20.12
74720	'Tmem114'	0	0	0	0	3.31	0	0	2.52	0	0
74729	'Setmar'	3.3	2.55	0	0	4.27	2.74	0.03	4.78	0	7.08
74732	'Stx11'	0	0	0	0	0	0	0	0	0	2.38
74734	'Rhoh'	0.01	0.01	0.01	0.02	0.01	7.51	0	0	0.67	2.36
74735	'Trim14'	0.26	3.46	0.03	0.03	0.03	0	0.02	0	2.27	0
74737	'Pcf11'	3.63	1.46	1.25	3.95	1.71	1.09	0.32	2.75	2.4	0.98
74741	'C2cd5'	11.31	11.21	15.8	5.95	5.07	7.9	11	6	8.22	25.83
74747	'Ddit4'	1.58	1.44	5.61	2.39	3.21	1.2	3.62	2.09	0	1.96
74748	'Slamf8'	0	0.05	0.07	0.08	0.04	0.02	0	0.04	0	0
74753	'Trmo'	5.31	0.34	0.24	0.54	0.15	0.07	3.89	4.11	1.17	2.41
74754	'Dhcr24'	3.63	8.35	8.83	2.74	8.6	12.11	12.6	9.86	3.57	5.49
74760	'Rab3il1'	11.7	8.22	16.6	27.7	7.92	23.51	9.96	4.37	13	9.68
74761	'Mxra8'	8.48	7.76	0.06	4.97	10.7	30.3	53.5	0	35.45	8.07
74762	'Mdga1'	3.98	3.23	13.8	0	7.87	0.87	5	2.92	0.7	4.98
74763	'Naa60'	35.98	32.58	58.5	36.8	38.6	19.51	34.4	33.72	33.14	32.1
74764	'Klc4'	17.57	34.89	20.1	7.61	20.7	24.09	30.1	17.57	36.46	32.76
74766	'Yipf2'	20.77	25.52	16.4	6.06	59.1	34.96	33.5	36.79	42.86	30.87
74769	'Pik3cb'	0	0.34	1.38	1.46	0.05	0	0.85	0.33	0.15	0.06
74770	'Hhatl'	15.09	0.02	0.08	0	5.36	0.03	0	0	3.76	1.13
74772	'Atp13a2'	14.15	8.56	13.3	7.39	6.55	6.51	8.21	13.3	18.03	5.79
74775	'Lmbr1l'	0	1.53	0.02	2.97	6.81	0	0.69	2.57	0.1	2.61
74776	'Ppa2'	31.74	57.7	48	45.4	31.4	25.63	50.2	57.55	76.65	17.57
74777	'Selenon'	2.32	5.15	0.81	7.93	3.16	0.01	0.46	1.01	2.21	2.67
74778	'Rrp7a'	89.37	71.28	59.4	74.4	74.7	87.48	50.3	81.11	110	88.24
74781	'Wipi2'	38.39	34.14	44.2	31.8	15.2	19.28	57	29.78	33.84	11.39
74782	'Glt8d2'	3.21	0	9.98	0.03	6.99	0.25	0.16	2.77	0	0
74838	'Naa15'	7.61	2.38	3.74	2.42	4.83	0.5	0.78	1.36	4.08	1.83
74840	'Manf'	57.49	78.2	37.4	87.7	39.7	53.08	38.6	44.6	57.97	51.94
74841	'Usp38'	2.57	3.03	2.83	8.92	1.06	0.32	10.5	1.64	2.58	5.16
74843	'Mss51'	0	0.11	0	0	0	0.03	0	0	0.49	0
74854	'4930402F06Rik'	0.07	0	0	0	0	0	0	0	0	0
74855	'Fam228a'	1.02	0.25	0	0.3	0.19	0	0	1.33	0.06	0.23
74868	'Tmem65'	1.26	0.61	3.41	4.95	7.24	0.48	9.29	0.74	1.39	0.39
74890	'Morn3'	2.95	0	0.16	0	0	0	0	0	0	0
74892	'4930447A16Rik'	0	0.18	0	0	0	0	0	0	0	0
74895	'Ccdc181'	19.05	13.12	7.97	15.8	23.4	38.28	14.7	17.52	14.19	19.78
74901	'Kbtbd11'	8.65	3.45	4.92	2.39	5.36	7.62	8.2	2.27	5.3	3.37
74918	'Iqca'	0.3	0	0	0.21	0	0	0.19	0.3	0	0.09
74919	'Slc35f6'	25.08	23.19	17.6	6.02	13.6	8.43	9.68	14.22	22.16	25.44
74934	'Armc4'	0	0	0	0	0	0	0	0.18	0	0
74978	'Lrriq1'	4.32	2.32	3.46	11	0.46	4.34	0	1.25	1.28	1.33
74989	'Csmd2os'	0	0	0	0	0.06	0	0.02	1.47	1.73	0.01
74996	'Usp47'	5.53	7.46	13.9	22.1	11.1	6.93	17.4	7.78	16.18	9.97
74998	'Rab11fip2'	2.66	1.59	5.16	6.18	3.53	3.47	0	4.07	2.79	2.89
75007	'Mindy1'	7.96	7.13	6.94	8.13	6.74	1.97	10.6	1.52	14.33	16.01
75011	'4930488N24Rik'	0	0	0	0	0	0	0.02	0	0	0
75013	'4930502E18Rik'	1.23	0	1.31	0	0	0	0	4.63	0	0
75015	'4930503B20Rik'	0.41	0	0	0	0	0	0	0	0	2.05
75029	'Purg'	0.25	0	4.86	4.7	2.73	0	0	1.26	1.97	0.06
75033	'Mei4'	3.5	0	1.18	0.15	0	0.88	0	3.72	0	0.18
75040	'Efcab10'	32.62	40.37	37.3	74.5	28.7	82.2	17	43.02	68.52	65.49
75050	'Kif27'	0.52	0.26	1.5	0.06	0.06	2.38	0.01	0.7	0.02	0.04
75051	'Ccdc173'	7.55	3.96	0.2	0.83	0.47	0.17	1.73	0.63	2.46	0.35
75062	'Sf3a3'	52.37	44.1	56.6	68.9	35.6	47.5	45.5	61.77	49.08	49.79
75079	'Zbtb49'	1.69	2.58	1.46	0	2.73	6.47	0	0.98	7.24	2.83

75083	'Usp50'	0	0	0	0.13	0	0	0	0	0	0
75087	'4930505A04Rik'	0	0	0	0	1.83	0	0.06	0	0.15	0
75089	'Uhrf1bp1l'	5.72	8.93	18.2	7.14	4.35	4.52	3.57	5.78	9.79	9.03
75099	'Lysmd4'	9.54	5.59	11.9	3.09	17.2	14.63	23.9	14.7	23.96	8.74
75104	'Mmd2'	91.38	85.63	74	68.1	40.1	87.5	57.8	38.21	40.79	47.38
75106	'4930519F16Rik'	0	0.4	0.25	0	0.36	0	0.09	0	0	0
75124	'Nxnl2'	4.1	2.27	0	4.55	3.05	0	0	1.83	3.73	0.04
75137	'Rprd2'	1.27	2.4	3.91	1.41	3.96	1.85	1.9	2.45	2.14	3.95
75141	'Rasd2'	17.14	24.27	10.8	10.2	12.2	5.92	3.06	15.54	17.55	14.37
75146	'Mfsd13a'	44.76	18.52	21.3	4.71	20.7	11.2	28.1	23.01	11.72	14.17
75172	'Ccdc146'	0.01	0	0	0	0.42	0.01	0	0.71	0	1.44
75173	'Tex38'	0	0.92	0	0	0	3.3	0	0	0	0
75178	'Meiob'	0.03	0	0	0	0	0	0	0	0.31	0
75180	'Tmem269'	0	0	6.45	4.89	2.46	5.21	0	5.05	2.01	0.62
751865	'Sap25'	1.41	2.23	4.94	0	8.2	0	6.11	1.09	2.92	0
75196	'Ankrd7'	0.44	0.26	0.25	0.01	0.52	0.04	0.02	0.55	0.01	0.43
75202	'Spaca6'	5.23	3.69	1.64	12.6	3.94	5.05	0.44	5.2	4.36	5.44
75209	'Sv2c'	5.45	5.91	3.12	0.95	0	0	1.39	4.4	1.14	2.12
75210	'Prr3'	35.31	27.35	32.4	34.8	15.3	18.43	33.9	52.53	29.68	21.63
75212	'Rnf121'	23.81	23.91	30.5	2.46	19.6	24.12	44.1	35.18	35.09	32.59
75216	'Cep128'	0.01	0.93	0.03	0	0	4.51	0	0	0	0.01
75219	'Dusp18'	1.22	6.89	15.8	7.28	3.81	7.35	7.42	2.06	0	9.49
75221	'Dpp3'	34.24	13.59	25.2	20.7	34.6	40.57	16.2	33.52	35.07	37.82
75234	'Rnf19b'	4.05	5.87	3.55	0.07	0.15	0.14	2.48	5.2	0.05	3.08
75258	'4930563M21Rik'	0	0.98	0.71	1.44	0	0	0	0	0	0
75266	'Tomm20l'	0	3.85	0	0	0	0	0	0	0	0
75273	'Pelp1'	8.32	6.07	5.52	0.18	5.98	4.08	6.56	1.78	10.8	7.94
75275	'Tmco5b'	0	0	0	0	0	2.74	0	0	0	0
75284	'Bcdin3d'	19.65	13.89	8.27	7.75	7.67	6.25	6.71	3.29	10.92	17.53
75288	'Slc35f4'	25.63	14.23	4.03	10.9	6.83	27.66	21.4	21.37	24.81	15.49
75291	'Zbtb3'	6.76	3.07	2.66	0	0.03	0	4.4	3.33	1.89	0
75292	'Prkd3'	0.45	0	1.9	2.71	0.66	0	0	0	1.57	0.21
75296	'Fgfr1op'	13.09	7.75	6.67	6.77	16.9	4.9	11.9	11.1	4.09	13.11
75302	'Asxl2'	1.39	3.28	2.16	2.92	2.76	2.89	3.38	0.92	0.99	3.44
75304	'4930563E22Rik'	4.04	5.95	4.56	5.68	5.59	4.95	1.75	5.71	9.35	5.86
75305	'Ankrd53'	0.51	0.25	0	0	0	0	0	0	0	0
75311	'4930550C14Rik'	0.72	0.12	0	0	0.3	0	0.47	0.83	1.28	0
75316	'Taf1d'	17.19	8.61	17.5	32.4	11.8	12.37	12.3	7.06	17.25	6.8
75317	'Parpbp'	0	0	1.29	0	1.21	0	0	1.39	0	0
75320	'Etnk1'	10.18	4.4	12.2	20.9	10.1	12.92	23.9	4.28	6.7	13.14
75329	'Atf7ip2'	0	0	2	0	0	0	0	0.55	0	0
75339	'Mphosph8'	20.45	10.06	14.9	26.4	29.9	32.01	24.9	13.66	19.28	24.91
75345	'Slamf7'	0	0	0	2.26	0	0	0	0	0	0
75368	'4930558K02Rik'	0	0	0.03	0	1.67	0	0.03	0.04	0	0
75387	'Sirt4'	15.42	17.7	17.4	13.4	9.22	40.87	16.6	10.55	13.7	23.73
75396	'Spp2'	0	0	0.1	0	9.32	0	0	0	0	2.49
75398	'Mrpl32'	55.39	63.54	38.8	45.5	79.4	38.58	65.7	65.68	45.37	76.25
75404	'Arhgap36'	187	304.2	39.9	5.16	31.8	50.14	10	162.4	48.14	124.27
75406	'Ndufs7'	560.6	471.1	596	520	676	976.1	528	550.3	535.9	564.25
75409	'Slitrk5'	10.49	3.12	0.23	2.42	7.82	0.81	0.7	7.8	4.92	4.77
75410	'Kmt2b'	1.37	0.82	0.67	2.15	0.84	0.03	4.48	0.58	2.2	0.31
75415	'Arhgap12'	12.87	9.54	14.7	14.1	9.17	12.84	17.8	10.65	19.34	16.37
75416	'Nop14'	5.32	17.9	10.1	10.6	18	6.95	16.4	13.78	14.1	14.42
75420	'Secisbp2'	14.82	9.3	12.3	16.9	5.12	10.51	19.6	8.8	14.63	7.04
75422	'Mettl5'	32.78	17.79	24.3	48.5	39.5	49.88	32.8	33.31	36.93	34.13
75423	'Arl5a'	2.13	2.23	7.43	0.22	4.17	3.11	1.46	3.6	0.99	0.83
75424	'Zfp820'	0.08	0.17	0.11	0.06	0.07	0.19	0.2	0.16	0.03	1.11
75425	'Tti1'	2.91	3.42	7.9	3.82	5.8	3.88	0.6	5.87	5.56	8.06

75426	'Igfbpl1'	0	7.46	0	0	2.9	1.06	5.12	0	0	0
75429	'Fam183b'	214.1	152.5	32.5	234	104	181.9	59.5	53.72	60.63	186.83
75430	'Anapc15'	13.46	12.79	33.6	29.8	4.85	5.23	18.6	12.83	6.46	8.07
75434	'1700001C02Rik'	0	0	0	0	2.83	0	0	4.48	0	0
75444	'Ccdc192'	0	0	0.06	6.87	0	0	0	0.24	2.41	0
75452	'Ascc2'	6.86	7.93	5.11	7.33	2.45	8.45	11.2	6.48	3.45	4.13
75454	'Phpt1'	141.6	114	186	202	96.2	126.2	154	133.3	109.4	177.39
75458	'Cklf'	4.2	4.22	6.72	9.6	8.42	0.02	3.86	2.82	0	4.93
75459	'Syce3'	0	0	0.12	0	0	0	0	0	0	0
75465	'Dynlrb2'	0	0	0	9.9	16.8	26.05	0	2.6	6.36	12.23
75469	'Spata19'	0	0	0.08	0	0	5.18	0	1.32	0	2.89
75470	'Iqcf5'	0	0	0	0	4.16	0	0	0	0	0
75472	'Cfap126'	0.1	14.53	0	17.7	4.24	14.21	0	5.75	0	7.16
75475	'Oplah'	3.58	4.65	7.82	0	0.98	0	5.67	5.11	2.64	2.11
75477	'Pfn3'	0	0	3.1	0	0	8.04	0	3.25	0	2.69
75480	'1700003F12Rik'	0	1.6	6.6	0	2.41	3.63	3.04	3.55	28.07	12.77
75495	'Morn5'	0	9.29	0	0	0	0.29	0	6.43	0	2.86
75497	'Fabp12'	0	0	0	0	0	0	0	0	1.06	0
75504	'1700013F07Rik'	8.98	3.26	3.18	7.6	1.98	1.02	6.34	4.89	2.63	9.08
75507	'Pou5f2'	0.03	0	0	0	0	0	0	0	1.91	0
75516	'Ttc32'	4.38	1.39	3.91	0	2.29	7.42	4.42	3.85	5.49	10.18
75526	'Eppin'	0	5.32	0	0	0	0	0	0	0	0
75528	'Tex29'	0	0.12	0.16	0	0	0.12	0	0	0	0
75530	'Lyrm7'	11.05	10.07	8.33	6.03	9.3	8.78	11.8	9.15	13.78	4.93
75533	'Nme5'	35.52	19.88	26.2	101	46.9	69.19	8.3	31.53	6.86	35.31
75538	'Fam71e1'	12.74	6.78	11.2	19.7	14.4	9.05	5.43	11.77	9.59	5.41
75540	'Fpgt'	3.77	2.11	2.99	8.77	0.71	0.26	18.9	7.44	0.43	2.21
75541	'Nat8f4'	1.76	8.33	3.58	0	9.65	7.78	0.02	1.79	0.01	8.13
75547	'Akap13'	0.47	1.08	0.69	0.21	1.3	0.73	0	0.88	1.9	1.79
75552	'Paqr9'	4.34	10.29	5.26	3.62	4.45	6.91	11	0.88	1.5	3.24
75553	'Zc3h14'	10.6	10.3	8.16	16.7	6.04	8.36	11.6	11.14	7.06	15.84
75555	'1700020D05Rik'	0	0	0	0	0	0	0	2.2	0	0
75556	'Cfap161'	17.16	12.04	2.37	16.5	1.05	21.08	6.81	8.92	5.08	3.3
75558	'Spata45'	0	0	0	0	0.65	0	0	0	0	0
75560	'Ep400'	6.06	4.21	6.13	4.21	9.06	3.38	7.6	3.79	3.51	8.64
75563	'Dnali1'	0	0	0	0	3.78	0	0	0.02	0.53	3.72
75564	'Rsph9'	121.1	154.7	142	155	194	140	78.4	98.3	104.4	193.05
75565	'Sgf29'	13.6	12.32	28.1	50.7	29.3	25.12	31.5	23.13	57.96	28.33
75568	'Capsl'	2.58	3.78	2.7	6.73	0.22	16.3	0	5.61	0	4.08
75570	'Nhej1'	2.99	6.9	1.78	0	2.23	0.24	5.71	8.66	4.87	9.58
75571	'Spata9'	0.98	3.47	0.46	0.91	0	13.57	0.15	0	2.48	0.25
75572	'Acyp2'	40.64	59.65	44.4	57.3	66.3	105.5	64.5	80.64	74.95	67.36
75578	'Fggy'	13.4	12.85	18.4	31.4	14.5	27.35	28.6	29.93	40.79	14.63
75580	'Zbtb4'	3.23	1.09	3.57	1.37	3.47	4.71	1.95	4.02	2.41	2.88
75581	'Yipf7'	0.04	0	0.44	0	0.08	0.15	0.04	0.03	0.04	0.07
75590	'Dusp9'	0	0	0	0	0	0	0	0.38	0	0
75593	'Malsu1'	23.48	23.19	13.3	17	20.2	16.92	25.5	16.22	13.69	25.06
75597	'Ndufaf2'	67.01	43.62	77.3	23	35.3	26.7	61.6	26.01	50.72	56.02
75599	'Pcdh1'	3.91	0.46	2.05	2.69	2.38	3.82	0.27	0	1.1	0.48
75600	'Calml4'	0.2	0	0	0	0.21	0	0	0.6	0	0
75604	'Tm4sf5'	22.36	3.32	2.46	24.2	8.22	15.64	3.78	10.48	13.99	6.92
75605	'Kdm5b'	4.89	2.83	8.44	10.5	9.04	3.21	4.71	3.38	2.24	1.62
75607	'Wnk2'	1.53	2.55	3.39	7.06	1.62	1.68	6.69	4.2	5.42	1.81
75608	'Chmp4b'	6.85	7.07	3.63	9.97	5.79	13.94	11.9	3.96	8.13	6.62
75610	'2010109A12Rik'	0.27	0.38	0	0	0	4.74	0	0	0	0
75612	'Gns'	12.9	16.12	23	23.4	19.8	3.99	17.2	9.55	20.51	4.46
75613	'Med25'	3.66	20.04	16.7	9.14	9.46	15.68	11.4	6.02	20.73	4.91
75616	'Smim15'	12.65	11.41	6.48	11.3	9.2	19.89	19.6	5.2	11.59	6.48

75617	'Rps25'	375.9	401.6	447	451	325	464.3	372	224.5	490.2	329.69
75619	'Fastkd2'	34.59	12.63	19.6	7.7	2.51	1.58	4.81	14.14	10.38	9.84
75620	'Kxd1'	51.82	40.83	52.2	60.2	19.4	15.42	27.9	43.42	31.1	45.63
75623	'Tex30'	8.92	19.98	11.4	16.7	3.19	0.04	9.66	15.85	10.16	5.39
75624	'Metap1'	24.33	52.41	39	41.8	28.3	17.86	29.5	17.97	9.69	17.09
75625	'Mageh1'	93.46	96.92	52.6	17.8	44.4	49.06	94.7	78.65	127.5	97.91
75627	'Snapc1'	38.85	28.79	23.4	31.9	18.5	22.76	43.7	36.47	26.23	59.14
75641	'1700029I15Rik'	0.29	0.15	0	0.19	1.58	0	0.81	0	2.27	0.14
75646	'Rai14'	0.83	0	0	0	0	0	0.08	0.94	0	0.05
75647	'Ssmem1'	0	0	0	0	0	0	1.03	0	0	0
75657	'Speer4a'	0	0.17	0	2.31	0.03	0	0	0	0	1
75659	'Wdr54'	32.88	30.57	47.8	38.6	45	34.78	35.7	30.78	29.47	59.3
75660	'Lin37'	67.21	33.74	38.1	65.4	47.6	42.67	24.6	42.75	74.84	61.44
75665	'Bicdl1'	5.93	11.25	7.23	6.8	6.27	11.02	4.12	10.6	13.76	0.49
75668	'Rasl10a'	0	1.05	0	0	1.23	0	0	6.9	0	0
75669	'Pik3r4'	6.86	9.19	12.7	4.87	3.71	0.37	10.1	2.76	5.75	2.86
75671	'Tex22'	0	0	0	0	0	0	0	0.05	0	0.14
75677	'Cldn22'	0	0	0.85	0	0	0	0	0	0	0
75678	'Ippk'	7.72	9.31	9.69	9.92	4.49	8.03	13.3	16.33	3.13	3.92
75686	'Nudt16'	20.88	19.72	22.4	15.2	42.1	33.89	16.6	45.83	28.16	39.17
75687	'Ripor1'	11.54	14.79	37	29	14.9	6.26	9.96	13.25	27.6	16.95
75689	'Higd1b'	0	0	0	1.94	0	0	0	0	0	0
75690	'Vsig10l'	2.4	6.37	1.07	0	4.24	0	0	1.5	5.24	0.89
75691	'Anks6'	0.06	0.08	1.67	3.61	0	0.33	0.54	0	1	0.42
75692	'Nr2c2ap'	28.83	17.88	29.1	0	19.9	27.41	2.94	40.35	40.54	69.06
75695	'Rilpl1'	18.17	3.71	9.29	5.69	7.76	8.85	2.18	14.18	13.73	6.46
75697	'C2cd4b'	0	1.05	0.23	9.58	0	0.37	0	0.37	0	0.14
75698	'Shld2'	0	0	0	0	1.26	1.91	0	0.75	0	0.69
75705	'Eif4b'	22.63	27.97	37.9	40.5	31.9	18.87	20.8	18.86	30.27	22.05
75710	'Rbm12'	2.25	5.32	2.76	3.56	6.34	3.01	9.62	8.58	3.66	6.15
75712	'Tmem14a'	53.9	30.57	33.2	61.8	38.4	56.05	48.8	30.78	22.33	67.02
75717	'Cul5'	16.19	11.5	12.9	12.3	9.67	10.82	10.4	5.18	9.48	8.43
75718	'Vwa5b1'	2.49	2.47	4.35	0.01	5.59	4	2.72	3.28	4.09	3.54
75723	'Amotl1'	8.09	16.01	13.8	9.6	9.57	3.92	7.64	11.1	10.16	13.17
75725	'Phf14'	7.7	6.08	3.31	10.5	1.37	0.39	9.97	6.36	5.37	2.86
75729	'Fam227a'	1.61	3.37	3.9	8	5.05	4.19	6.05	0.66	0.47	0
75731	'Idnk'	33.19	48.2	25.6	28.3	17.5	52.26	11.2	37.08	41.42	39.33
75732	'Iqcd'	0	0.61	1.08	2.38	0	2.18	0	1.31	0.28	0.01
75734	'Mff'	249	260.1	245	296	192	237.7	295	290.9	223.8	234.55
75735	'Pank1'	1.82	0.46	0.94	1.8	2.19	0.04	1.99	0.98	0.63	2.54
75736	'Bcl2l12'	5.77	0	3.25	0	0	0	5.23	0	0	0.9
75739	'Mpp7'	2.89	5.15	0.35	0.1	0.12	0.08	0.02	2.21	0.11	2.88
75740	'Egfem1'	7.48	4.05	0.3	9.63	12.5	2.28	8.11	6.12	1.47	2.7
75744	'Svip'	14.68	19.97	9.48	8.8	27.8	6	11.9	9.7	11.88	11.94
75746	'Morc4'	1.74	0.04	0.35	0	1.58	1.29	0.82	1.14	0.87	0
75747	'Sesn3'	9.2	2.27	4.94	0.82	6.02	3.42	7.69	5.44	2.22	0.1
75750	'Slc10a6'	0	0.43	0	0	0	0	1.01	0	0	0
75751	'Ipo4'	29.31	41.53	44.3	32	38.4	23.07	55.7	39.18	25.64	46.27
75758	'9130401M01Rik'	33.73	21.17	31	28.5	50.9	50.6	68.9	28.13	30.44	38.71
75763	'Dcaf17'	8.71	8.06	4.67	7.75	13.9	9.1	8.16	1.5	13.91	14.11
75764	'Slx1b'	3.71	4.67	5.68	6.56	4.83	11.85	13	5.41	4.98	2.26
75766	'Dcstamp'	0	0	0	0	0	0	0	2	0	0
75767	'Rab11fip1'	0.04	0.01	0.09	0.06	0.75	0.01	0.06	0.01	0.55	0.06
75769	'Plppr5'	4.17	1.52	3.49	1.25	0.65	0.08	0	2.76	2.5	0.38
75770	'Brsk2'	1.05	3.08	7.44	5.1	4.51	4.39	7.01	3.94	1.7	3.51
75778	'Them4'	47.85	25.93	30.8	19.5	17.4	11.45	15.8	23.31	42.56	14.79
75782	'Lca5'	1.67	0.98	3.5	2.75	1	0.01	0	2.05	0.56	3.35
75785	'Klhl24'	2.3	7.34	5.49	7.58	6.98	6.24	1	5.32	4.6	10.83

75786	'Ckap5'	12.56	6.06	9.91	12.1	16.1	7.4	7	6.79	5.76	13.78
75788	'Smurf1'	0.27	1.98	2.49	2.8	2.58	0.39	1.47	0.93	0.01	0.17
75796	'Cdyl2'	4.71	2.8	2.57	5.23	8.13	3.2	0.17	4.8	5.42	11.88
75799	'4930444P10Rik'	2.28	1.34	0	0	0.3	0	1.8	0.34	14.32	1.42
75801	'4930447C04Rik'	6.7	3.26	1.29	5.73	6.01	7.89	1.71	2.55	6.18	6.64
75805	'Nln'	8.22	11.43	15.2	5.48	12.8	1.02	0.82	11.93	6.36	2.36
75812	'Tasp1'	9.71	17.48	19.9	20.8	27.6	28.47	6.67	38.38	39.94	25.78
75820	'Wdr64'	0.71	0	0	0	0	0	0	0	0	0.71
75823	'Fam227b'	0	0	0.06	4.17	0	0	0	0	0.06	0
75826	'Senp2'	5.83	11.2	6.82	12	1.9	3.75	6.03	9.47	10.74	7.52
75841	'Rnf139'	3.77	9.34	8.26	7.84	1.73	4.02	0.13	1.14	3.48	3.96
75847	'Ispd'	6.86	15.83	12	12.3	4.08	13.41	4.92	16.23	17.5	4.13
75860	'Tex26'	3.93	0	5.01	0	4.46	0	0.03	0	0	4.31
75869	'Arl5b'	3.06	1.72	3.17	0.02	0.45	0.73	0.88	1.15	0.73	0.16
75870	'Tcam1'	0	0	0	0	0	0	0	0	0.01	0
75871	'Zfp821'	2.33	5.84	2.85	41.6	14.4	15.15	7.4	12.68	7.98	5.54
75894	'Adal'	15.21	5.39	12.2	7.44	20.5	9.56	21.5	15.97	9.25	9.82
75901	'Dcp1a'	0	2.91	5.39	4.36	1.29	3.38	0.01	1.86	8.82	2.19
75906	'Fam184a'	1.71	3.19	2.16	7.41	0.42	3.4	0.08	0.34	2.65	1.9
75909	'Vmp1'	70.84	83.9	74.2	57.6	82.4	92.74	62.6	75.59	81.76	69.6
75914	'Exoc6b'	11.55	8.35	30.8	19.8	10.3	5.98	11.8	7.36	16.51	20.08
75939	'4930579G24Rik'	0.14	0.24	0.13	7.09	0.35	0.21	0.16	1.31	0.33	4.39
75953	'Samd7'	1.43	2.33	0	0	0	2.68	0	0	0.01	0
75956	'Srrm2'	54.77	45.75	71.1	86.1	113	27.3	44.5	24.9	86.69	49.64
75964	'Trappc8'	0.92	1.2	0.69	1.3	0.96	0	0.4	1.11	1.04	0.01
75965	'Zdhhc20'	34.74	22.28	16.6	27	17	15.39	15.2	27.24	25.83	17.64
75973	'Ccdc162'	0.04	0.01	0.13	0	0	0	0	0	0.07	0
75974	'Dock11'	0.59	2.97	1.68	1.67	0.72	0.88	0.01	0.52	0.05	4.15
75985	'Rab30'	1	5.28	5.61	14.5	3.68	14.61	10.2	1.3	3.24	0.16
75991	'Slain2'	0.02	0.04	1.17	0.03	1.43	0.35	0	1.4	0.2	0.65
76007	'Zmym2'	5.43	4.77	6.21	14.2	4.62	4.98	5.39	3.15	11.31	6.26
76014	'Zc3h18'	4.03	1.91	1.84	4.87	3.8	3.06	3.84	6.16	1.21	2.8
76022	'Gon4l'	9.36	9.63	7.77	11.2	9.06	16.53	10.7	7.17	12.37	12.92
76025	'Cant1'	19.52	10.88	26.1	13.3	20.7	21.57	36.5	31.53	29.14	22.06
76041	'Ccdc125'	0.06	0.16	0	0	0	0	0.75	0	1.31	0
76044	'Ncapg2'	0	0.01	0	0	0	0	0	0	0	0.6
76051	'Ganc'	2.44	1.63	1.17	0	3.29	1.12	0	1.19	1.6	0.22
76055	'Mgea5'	11.44	12	14.3	24.9	4.99	14.65	11.2	7.92	28.63	7.99
76071	'Jakmip1'	38.83	37.24	43.6	43.3	32	31.76	65.1	23.72	29.91	22.71
76072	'Rnf183'	2.69	0	0	0	0	0	0	0	0	0
76073	'Pcgf5'	18.49	10.97	4.16	15.6	7.63	19.58	11.7	5.41	6.18	23.11
76074	'Gbp8'	0	0	0	0.02	0.31	0	0	0	0	0
76080	'Ttpal'	5.45	3.02	4.33	0.01	8.7	2.66	3.49	3.7	6.31	14.94
76088	'Dock8'	0	0	0	0	0.01	0.02	0	0	2.12	1.52
76089	'Rapgef2'	2.67	4.97	5.61	2.3	3.9	3.4	4.66	2.65	3.95	3.16
76108	'Rap2a'	5.77	6.01	0.81	13.8	7.08	1.04	6.66	2.39	4.07	4.4
76113	'Lpo'	3.62	1.07	0.02	0	0	0	0	3.54	0	2.44
76117	'Arhgap15'	23.57	18.19	28.2	48.4	39	41.18	31.6	14.2	32.1	23.81
76123	'Gpsm2'	0	1.35	0	5.23	0	0.05	0.02	0.03	0	1.73
76130	'Las1l'	23.28	31.22	54	62.7	48.6	25.73	33.4	40.41	29.75	26.6
76131	'Depdc1a'	0	0	0.58	0	0	0	0	0	0	0.32
76132	'Faxc'	1.36	0.98	1.6	0.47	0.31	1.76	0.48	0.58	2.23	1.52
76137	'Mcur1'	4.66	1.33	2.5	0	3.38	0.02	1.93	0.54	0.04	0.06
76138	'Ccdc138'	5.96	1.57	3.45	3.54	3.98	5.13	3.11	2.69	3.61	0.63
76142	'Ppp1r14c'	10.78	15.24	16.3	24	4.81	8.24	27	11.65	16.88	9.2
76156	'Fam131b'	0.18	0	0.01	0	1.09	3.16	0.2	0.73	0	0
76157	'Slc35d3'	0	3.85	0	0	0	0	0	1	0	0
76161	'Lamp5'	27.31	74.64	0.75	18.3	0.08	0.07	74.6	167.9	0.03	0.67

76166	'Rsg1'	1.64	0	6.57	8.01	9.47	10.76	5.65	7.78	1.98	8.27
76167	'Snrnp35'	10.6	14.97	9.03	11	14.6	1.6	17.6	12.71	3.73	19.44
76178	'Coa5'	28	29.89	41.2	23.3	31.9	12.1	32.4	21.31	22.38	34.69
76179	'Usp31'	0.86	2.47	1.38	1.17	1.88	1.81	0.7	0.21	1.12	0.72
76183	'Celf6'	91.73	57.97	30.6	40.6	64	31.86	22	40.06	75.58	75.54
76184	'Abca6'	0.01	0.25	2.18	0.38	0.02	0.01	0	0.01	0.04	0.02
76187	'Adhfe1'	2.57	2.97	4.06	7.91	7.05	7.9	0	3.85	0	5.14
76192	'Abhd12'	108.1	102.3	140	113	136	128.6	106	119.4	150.4	79.62
76199	'Med13l'	0.89	1.59	1.24	2.61	2.48	0.71	1.22	0.73	2.65	1.02
76205	'Stard3nl'	128.1	110.9	92.7	54.1	91.9	147.9	107	101.2	65.2	121.95
76206	'Gpr165'	16.83	17.93	28	6.03	1.02	2.48	16.4	35.49	57.33	36.83
76217	'Jakmip2'	25.03	26.45	17.8	6.16	30.8	23.98	15.2	27.72	26.56	24.64
76219	'Arxes1'	138.6	182.9	70.4	88.7	75.9	107.9	90.7	120	74.47	76.34
76223	'Agbl3'	4.99	6.85	9.96	9.43	8.57	18.38	5.57	1.6	5.34	5.05
76229	'Vmn2r29'	0.09	0.76	0.69	0	0.78	0.27	0.47	0.33	0.12	0.5
76233	'Dnttip1'	8.34	5.56	3.59	0	12.8	12.03	9.49	4.09	16.38	3.51
76237	'6430628N08Rik'	5.21	0	0	0	4.13	0	4.84	2.65	0	1.93
76238	'Grhpr'	60.92	70.1	59.5	63.5	34.3	80.85	72.7	66.58	52.28	46.61
76246	'Rtf1'	5.27	4.63	6.67	6.72	13.6	7.36	4.95	7.55	7.38	6.29
76251	'Ercc6l2'	1.21	3.61	3.38	3.71	1.82	2.49	4.08	2.29	2.44	0.03
76252	'Atp6v0e2'	319.4	300.9	403	273	302	525.4	419	466	407	367.18
76257	'Slc38a3'	11.9	4.46	13.9	6.11	1.06	6.48	13	5.39	0.02	3.84
76260	'Ttc8'	26.26	25.68	27.2	16.7	22.8	41.04	10	24.99	19.19	33.82
76261	'0610040J01Rik'	3.97	0	7.62	9.49	0	0	5.56	2.58	0	3.52
76263	'Gstk1'	11.15	1.94	7.49	30.2	10.2	36.88	6.95	9.59	0	15.86
76265	'Tsen54'	2.51	1.78	0	2.55	6.59	4.96	3.41	2.14	0	3.29
76267	'Fads1'	29.03	32.27	40.4	7.87	25.7	20.25	25.1	22.13	17.36	16.26
76273	'Ndfip2'	34.93	14.04	37.3	38.1	17.8	12.41	14.7	34.26	33.55	15.92
76281	'Tax1bp3'	5.22	8.09	2.1	16	8.57	8.59	14.2	13.28	3.45	14.89
76282	'Gpt'	0	2.08	0	0	0.5	0	0	0	2.87	0.02
76294	'Asb5'	0.03	0	0.07	0.65	0	0	0	0.03	0.03	0
76295	'Atp11b'	7.75	3.36	12.5	13	5.99	2.35	4.14	5.32	19.85	3.92
76299	'Erp44'	43.65	44.29	19.7	24.6	27.1	38.97	19.9	36.68	47.37	48.58
76302	'Pcnp'	184.1	173.9	166	176	174	169.3	107	159.8	213.1	194.25
76303	'Osbp'	2.25	1.22	0.83	2.68	2.17	2.1	1.96	1.08	1.82	0.34
76306	'Slc18b1'	12.01	2.31	6.09	8.33	6.88	6.22	3.06	4.14	13.31	10.45
76308	'Rab1b'	146.3	224.9	145	161	175	217.5	163	162.9	139.2	186.67
76332	'Cog2'	9.72	7.64	16.1	0.12	9.91	4.11	12.2	7.8	26.59	7.26
76338	'Rab2b'	37.02	20.43	29.9	4.37	10.9	18.98	49.6	34.46	20.34	34.2
76355	'Tgds'	8.32	2.2	7.16	7.38	3.47	4.8	5.95	1.61	9.4	5.93
76357	'Trmt5'	3.99	16.05	9.69	0	3.1	0.08	11.9	14	0	15.02
76366	'Mtif3'	13.3	9.35	12.5	23.7	16.2	7.49	17	13.23	6.8	18.13
76367	'Trp53rkb'	0.27	1.55	0.19	3.11	3.18	2.88	6.02	1.41	0.02	0
76373	'Zfp773'	0.1	0.03	2.52	2.67	0.05	0.04	0.13	0	2.73	0
76375	'Det1'	0	1.74	0	0	5.75	0.76	0	0.08	0	3.59
76376	'Slc24a2'	4.59	0.88	6.26	2.01	5.83	3.37	3.12	2.64	1.54	1.12
76380	'Cep112'	4.88	4.03	2.5	9.7	5.13	6.3	5.14	2.7	3.67	10.18
76405	'1700018B08Rik'	0	0	0	0	1.9	0	0	0	0	0
76406	'Tex45'	0	2.64	0	4.18	0	0	0	0	2.32	0
76408	'Abcc3'	0	0	0	0	0	0.73	0	0.49	0.24	0
76411	'Ift43'	23.76	14.39	12	25.4	51.7	22.16	15.8	31.78	30.7	25.18
76413	'1700016D06Rik'	0.11	0.23	0	0	0	0	0.52	0.03	0	0
76415	'Fam187b'	0	0	0	0	3.77	0	0	0	0	0
76416	'Znrd1as'	15.6	10.86	4.6	9.42	7.31	7.75	0	0.17	0	16.36
76421	'1700028K03Rik'	0.21	0.19	1.65	0	0.26	0	1.26	0.01	0	0.22
76425	'Gid8'	27.07	37.28	34.6	25.6	51.7	39.37	31.8	48.05	39.42	23.05
76426	'Fam209'	0	0.16	0	3.33	0	0.31	0	0	0	1.28
76429	'Lhpp'	6.59	2.65	11.2	0.04	13.9	12.24	24	13.65	0	13.43

76438	'Rftn1'	15.58	20.56	4.08	23.6	17	29.01	13.8	11.14	26.52	19.37
76441	'Daam2'	2.04	0.56	0.01	3.27	5.81	0	1.75	0	0.06	1.42
76448	'Ppp1r18'	0.02	4.45	0.25	0	0.04	0	0	1.52	0	3.28
76453	'Prss23'	3.24	0	0	0	19.3	0	0	3.66	0	0
76454	'Fbxo31'	8.17	2.43	6.13	8.43	7.87	4.54	15.7	4.53	4.62	0.33
76457	'Ccdc134'	3.22	0.55	11.6	8.89	4.05	0	2	3.86	0	1.95
76459	'Car12'	1.25	0	6.42	0	0	0.12	0.01	2.56	0.05	0
76464	'Knl1'	0	0	0.01	0.01	0	0.01	0	0	0.01	0
76467	'Msrb2'	64.04	21.37	23.3	52.2	21.2	25.85	20.2	29.14	19.37	26.46
76469	'Cmya5'	0	0.23	0.02	0.22	0.01	0.01	0.02	0.01	0	0.02
76477	'Pcolce2'	3.49	24.3	1.81	4.05	5.38	0	16.2	1.53	1.34	2.91
76478	'Haus8'	24.01	13.47	29.8	0.04	23	46.77	9.61	21.32	8.16	27.44
76479	'Smndc1'	21.59	22	17.2	23.6	14.1	6.82	17.9	19.35	17.01	13.18
76482	'Rmc1'	24.39	18.79	32.2	26.6	19.2	63.03	30.7	33.61	14.81	40.39
76483	'Lmf1'	11.22	30.23	13.6	49.8	13.4	0.46	13.4	14.37	13.36	28.42
76484	'Kndc1'	1.93	3.36	2.93	7.59	3.48	1.23	3.79	4.95	3.07	4.02
76485	'Glt8d1'	19.22	24.84	10.5	20.5	37.8	20.11	21.6	8.12	27.29	20.84
76487	'Ppp1r3g'	0	2.12	0	0	0	0	0	2.43	0	0
76491	'Abhd14b'	9.49	19.2	19.3	11	56.7	37.39	26.2	8.66	29.48	22.1
76497	'Ppp1r11'	122	103.9	109	111	146	112.1	96	110.1	116.1	111.11
76498	'Paqr4'	23.76	9.2	36.6	26.4	18.7	3.31	18.4	13.3	18.92	10.63
76499	'Clasp2'	30.52	29.19	32.9	39.4	23	21.81	32.3	25.04	53.43	42.01
76500	'Ip6k2'	56.97	86.41	96.9	147	61.9	87.35	92.6	124.3	97.69	67.66
76501	'Commd9'	98.39	68.2	81.6	157	86.5	167.7	53	108.4	122.4	91.29
76507	'Aoc1'	0	1.82	0	0	0	0	0	0	0	0
76509	'Plet1'	0	0.03	0	0	0	0	0	1.46	0	0
76510	'Trappc9'	27.2	38.32	48.2	29.4	24.1	25.84	27.9	38.18	40.25	29.28
76522	'Lsm8'	42.19	45.26	27.1	13.1	27	46.7	25.6	28.96	34.96	38.19
76524	'Cln6'	3.19	0	0	0	0.65	4.4	0	2.33	0	0
76527	'Il34'	41.45	39.65	44.2	11.1	20.6	62.17	34.1	39.09	18.51	26.45
76539	'Fam204a'	15.74	12.02	16	36.6	27.2	25.74	16.4	16.29	23.31	16.23
76547	'Tmem101'	14.37	20.12	18.6	24.3	25	26.94	41.3	31.55	38.87	34.08
76551	'Ccdc6'	7.55	15.14	8.7	5.63	9.87	12.14	6.05	7.93	12.28	12.11
76559	'Atg2b'	4.13	4.48	8.91	7	4.53	3.53	5.56	7.62	6.95	4.48
76560	'Prss8'	0	2.96	0	0	0	0	0	0	0.05	0
76561	'Snx7'	10.91	13.04	7.5	9.83	12.5	8.29	17.2	8.92	11.06	1.35
76563	'Qrsl1'	11.18	12.83	5.05	1.2	1.04	16.57	8.43	9.52	12.76	18.95
76566	'Rflnb'	2.4	1.72	0.16	0	3.64	0.86	1.51	0.87	3.35	0.44
76568	'Ift46'	30.11	52.27	37.8	46	34.3	47.33	59.6	33.52	44.7	64.72
76571	'Styxl1'	0	1.04	0.03	0	3.83	0	1.38	4.19	0.49	0.62
76574	'Mfsd2a'	0	3.01	30.7	0	0.75	8.69	23.1	9.14	0	0
76577	'Faf2'	21.5	17.33	25.1	16.8	13.8	26.01	14.3	22.82	24.33	28.41
76580	'Mib2'	7.33	16.03	38.1	21.2	22.7	11.4	44.9	14.54	45.36	16.6
76582	'Ipo11'	7	2.83	8.64	7.39	3.76	4.12	6.03	3.85	3.93	5.93
76589	'Unc5cl'	0	0	0	0	0	0	0	0	1.38	0
76594	'Dnajc18'	17.75	20.38	19.3	4.48	23.1	19.23	11.5	20.78	21.33	16.56
76608	'Hectd3'	10.15	10.56	17.4	3.98	3.71	0.08	16.8	3.92	13.02	4.22
76612	'Lrrc27'	7.36	12.31	3.63	4.91	6.2	8	6.8	8.12	10.08	11.1
76614	'Immt'	67.98	91.35	108	73	79.7	53.31	63.8	75.56	72.16	93.86
76615	'Got1l1'	0	0	9.06	0	3.79	4.88	20.7	7.18	8.18	0
76626	'Msi2'	5.35	5.39	4.67	10.1	3.58	1.96	3.13	0.86	2.22	1.99
76630	'Stambpl1'	30.52	39.54	36.5	40.8	14.5	21.65	18.1	19.55	24.73	31.18
76645	'Pkd1l2'	0	0	0	0.04	0	0	0.15	1.38	0	0.15
76650	'Srxn1'	21.95	23.76	19.4	5.95	20.5	12.04	6.48	27.99	17.28	24.34
76652	'Actrt3'	2.2	1.02	2.45	0	0	0	0	3.38	7.6	0
76654	'Upp2'	0	0.3	1.16	0.76	3.4	4.05	5.33	1.85	0.06	2.91
76658	'1700123K08Rik'	0	0	0.13	0	0	0	0	0	0	0
76668	'Mdh1b'	1.45	1.1	0	7.99	5.22	0	5.42	2.2	0	13.13

76670	'Cfap70'	0.07	2.11	1.16	2.73	0.1	0	0	0.43	0	2.37
76681	'Trim12a'	0	0	0	0	0	0	6.1	0	0	0
76686	'Clip3'	34.95	35.22	110	31.6	48	24.46	40.8	62.35	35.68	32.29
76687	'Spcs3'	25.56	27.17	7.81	16.1	20.6	8.87	12.4	17.88	28.73	49.96
76688	'Arfrp1'	34.1	50.67	43.7	52	33.3	41.36	49.6	50.35	57.89	53.74
76707	'Clasp1'	6.59	6.01	12.3	3.17	4.28	11.11	4.58	3.98	4.07	7.4
76709	'Arpc2'	122.1	118.6	101	125	107	99.69	120	110.9	174.8	88.48
76718	'Catsperg2'	0	0.85	0.11	0	3.44	0	0.03	0	0	0
76719	'Kansl1'	10.02	4.75	12.3	8.25	4.57	8.53	12	7.37	15.32	6.84
76722	'Ckmt2'	1.55	3.16	3.84	0.74	0.82	1.41	0.75	0.45	1.05	1.34
76737	'Creld2'	59.97	60.54	41.9	38.8	32.9	43.45	46.1	26.67	35.45	67.12
76740	'Efr3a'	22.87	16.39	27.8	3.28	14	9.76	20.1	16.57	20.6	24.61
76742	'Snx27'	9.73	10.1	4.43	7.5	5.18	3.49	8.45	5.56	7.04	5.42
76743	'Gje1'	0.06	0.19	0.39	0.55	0.46	0.1	0.39	0.07	0.4	0.16
76757	'Trdn'	0.02	0	0.15	0.05	0.04	0.06	0.01	0.01	0.03	0.03
76758	'Gsdma2'	0	0	0.12	0	0	0	0	0	0	0
76763	'Mospd2'	4.65	0.6	0.27	0.16	0.22	1.57	0.78	1.59	0.19	0
76768	'Alpi'	0	0.02	0	0	0	0	0	0	0	0
76770	'2010005H15Rik'	22.69	0.78	16.3	148	0	4.43	6.06	37.75	26.34	0.99
76773	'Wdyhv1'	19.5	24.06	15.2	42.8	12.5	38.25	0	35.69	21.01	57.57
76775	'Slc10a7'	2.08	0.67	1.5	5.4	3.86	0.2	6.75	4.11	2.69	3.64
76779	'Cluap1'	23.79	31.42	44.2	25.5	33.9	28.19	26.1	21.05	8.35	46.5
76781	'Mettl4'	6.13	1.67	1.26	1.56	0.01	0.44	4.78	1.65	4.26	1.53
76784	'Mtif2'	19.59	9.22	6.05	10.7	18.7	9.75	18.4	12.51	16.99	10.38
76787	'Ppfia3'	6.69	3.37	6.8	9.44	7.35	3.71	12.1	7.27	2.34	5.4
76788	'Klhdc10'	4.38	4.2	4.9	9.22	3.98	1.87	5.37	5.47	5.76	4.39
76789	'Mzt1'	41.19	38.67	49.2	34.5	28.1	40.65	41.4	42.34	24.36	39.75
76792	'2410131K14Rik'	21.22	22.36	25.8	15.4	17.8	27.56	18.4	28.13	26.29	27.99
76793	'Snip1'	12.99	8.84	6.75	24.5	6.5	7.4	8.46	6.1	19.69	2.42
76795	'Tbc1d9b'	11.47	18.51	20.7	7.07	23.1	6.73	15.5	11.37	8.26	11.22
76797	'2410137M14Rik'	0	0	0	0	0	0	1.58	1.51	0	0
76799	'Tmem234'	62.59	60.12	50.3	39.6	95.9	81.6	89.8	73.45	70.75	43.46
76800	'Usp42'	2.99	1.25	4.75	7.73	3.71	1.59	0.24	1.86	1.67	4.33
76803	'2410141K09Rik'	0.1	0.03	0	0	0	0	0	0	0	0
76804	'Kdm4c'	3.31	5.51	0.79	10.3	4.29	0.69	1.91	1.89	5.91	12.77
76808	'Rpl18a'	238.7	276.7	291	423	224	310.4	278	227.7	290.5	233.64
76809	'Bri3bp'	5.57	4.15	6.76	3.43	3.34	2.98	12	4.84	5.03	4.1
76813	'Armc6'	30.12	43.57	30.6	2.6	26.2	24.45	17.5	43.53	37.67	13.56
76815	'Calcoco2'	0	0	0.02	0	0	0	0	0	0.23	0.02
76816	'Sdccag8'	11.27	17.96	1.98	11.5	18.7	5.45	7.68	10.07	6.8	11.18
76820	'Fam49a'	17.62	9.06	10.1	11.4	23.4	17.22	26.2	18.52	7.96	14.77
76824	'Mtfr1l'	97.96	82.15	66.5	114	57.8	117.1	82.5	34.8	93.2	45.95
76826	'Nubpl'	0.3	2.27	6.16	0	12.3	13.18	3.27	7.38	0	11.81
76829	'Dok5'	0.65	4.17	6.87	7.95	12.4	20.31	9.08	3.36	3.6	10.9
76832	'Hyls1'	3.33	8.49	6.47	0	0	0.81	0	2.86	8.94	5.08
76843	'Dtl'	0	0	0.01	0.09	0	0.03	0	0.91	0	0
76846	'Rps9'	421.4	439.6	335	536	361	387.9	280	327.8	249.1	356.03
76850	'Ago4'	1.29	1.31	0	5.5	0.75	2.58	2.14	0.06	0	1.49
76854	'Gper1'	0	0	0	0	0	0.02	0	0	0	3.89
76856	'Catsper3'	0	0	0	0	0	0	0	0	2.83	0
76857	'Spopl'	0.28	0.19	0.1	0.27	0.64	1.95	0	3.02	5.06	0.27
76863	'Dcun1d5'	24.91	17.01	7.84	26.9	14.3	22.47	3.4	19.8	22.57	9.31
76866	'Morn1'	7.45	10.33	12.3	12.4	15	21.15	11.5	5.32	7.57	16.14
76867	'Rhbdd1'	1.73	1.53	1.05	0	0.24	3.57	4	1.08	0.35	3.57
76872	'Ccdc116'	7.53	0.63	1.9	7.71	3.97	0.06	0.31	3.37	3.3	5.55
76877	'Rab36'	31.38	43.46	32	17.8	38.6	39.86	23.6	37.47	29.16	51.01
76884	'Cyfip2'	40.61	31.76	78.7	32.9	20.3	16.4	62.7	31.82	37.32	34.01
76886	'Fam81a'	21.19	23.31	5.39	20.1	47.7	25.81	22.4	17.05	7.27	17.8

76889	'Coq8b'	15.72	27.8	15.1	14.3	28.7	14.79	31.9	25.56	51.56	35.65
76890	'Memo1'	8.8	5.25	10.3	9.56	6.52	7.73	18.1	6.34	10.12	8.17
76892	'Rnft1'	1.78	1.34	1.63	2.06	1.66	1.18	0	1.62	3.17	2.16
76893	'Cers2'	35.21	38.44	28	2.52	38.9	16.57	17.7	32.66	26.17	23.06
76894	'Mettl15'	8.11	12.79	3	0	9.41	6.17	3.55	2.96	0	6.96
76895	'Bicd2'	4.49	0.85	2.62	4.68	2.6	1.38	1.2	2.76	1.49	0.02
76897	'Ralyl'	99.42	80.23	72.3	121	162	209	79.2	99.28	194.2	101.24
76898	'B3gat1'	1.01	0.73	2.51	1.03	1.95	1.33	1.3	1.38	2.04	0.53
76899	'Golga1'	3.54	2.57	3.98	0.21	8.27	5.14	9.96	5.79	1.18	9.31
76900	'Ssbp4'	69.16	63.31	92.2	72.1	68.4	52.27	68.5	54.85	106.6	36.92
76901	'Jade2'	1.43	1.31	3.97	1.86	2.25	1.41	5.89	0.57	3.56	2.9
76905	'Lrg1'	1.59	0	0	0	0	0.1	0	0.03	0.03	0
76915	'Mnd1'	2.6	0	2.82	0	3.59	0	4.37	1.93	0	3.9
76916	'Timmdc1'	31.36	26.1	29.7	33.3	18.6	22.1	4.68	16.14	19.72	37.29
76917	'Flywch2'	82.62	58.66	71.1	139	84.8	163.3	101	79.41	149.4	135.86
76925	'Spata46'	1.79	2.01	0	0	2.35	0	0	0	0	0
76927	'Tsacc'	5.27	1.32	3.68	6.34	3.54	0.17	7.98	2.85	0.31	0.5
76932	'Arfip2'	37.07	29.43	52	23.9	36.8	27.03	51.1	35	34.33	25.27
76933	'Ifi27l2a'	6.91	9.71	0.08	36.9	8.2	0	0	0	12.16	10.26
76936	'Hnrnpm'	41.18	53.36	43.7	39.6	46.5	47.98	76.6	52.39	56.13	46.71
76938	'Rbm17'	25.22	24.08	45.1	39.3	17	6.21	49.9	24.34	41.02	7.43
76947	'Ndufaf6'	21.42	39.05	31.3	51.6	34.1	19.02	15.3	28.79	23.37	6.29
76952	'Nt5c2'	21.83	26.38	23.8	27.3	14.2	25.76	12.8	18.37	27.76	22.03
76954	'St5'	2.22	3.39	9.3	19.3	0.76	5.1	2.22	0.95	6.57	2.01
76959	'Chmp5'	117.8	131.9	103	51.2	104	121	71.4	93.43	159.6	115.02
76960	'Bcas1'	3.83	2.58	8.89	0	200	16.83	17.5	0	5.29	2.22
76964	'2610028H24Rik'	0	0	0	0	0	0	0	0	0.42	0
76965	'Slitrk1'	21.35	6.7	12.7	6.91	19.2	7.37	14.6	20.86	5.51	3.65
76967	'2700049A03Rik'	2.86	1.81	1.99	6.15	3.02	3.59	0	1.64	0.33	1.93
76969	'Chst1'	51.41	46.5	43.1	37.6	58.6	62.52	29.3	32.37	41.4	29.47
76974	'Urah'	0	0.7	0.08	0	0	0	0	0	0	0
76976	'Arxes2'	362.3	505.4	240	268	263	444.8	312	450.2	258.5	378.34
76980	'Ube2ql1'	6.6	18.61	9.6	14.7	19	13.69	19.8	17.35	9.52	9.62
76982	'Vxn'	0.04	6.65	10.8	0.12	0.9	0.01	8.48	15.31	4.74	7.74
76983	'Scfd1'	56.73	53.66	46.4	82.5	51.5	64.09	47.6	70.12	48.37	75.88
76987	'Hdhd2'	95.96	75.66	144	69.9	78	99.28	47.9	98.76	84.97	81.3
77006	'Ddrgk1'	83.43	88.88	91.2	122	105	133.8	94.7	102.7	119.4	121.59
77015	'Mpped2'	10.93	9.67	9.63	7.39	16.1	19.94	7.09	1.65	5.07	5.75
77018	'Col25a1'	5.91	6.31	7.53	0.96	3.48	4.4	6.98	6.17	5.07	3.36
77031	'Slc9a8'	13.65	8.65	21.2	3.46	5.16	2.97	20.9	17.43	5.65	21.14
77032	'Tstd3'	28.84	37.05	14.3	0.04	16.5	6.6	9.02	15.58	25.18	13.46
77034	'2510039O18Rik'	3.22	0.51	0.33	4.02	0.02	0.61	0	3.17	5.11	0.53
77035	'Kdm8'	0.48	2.67	2.72	0	0.4	0	4.04	0	3.37	3.14
77036	'1700109H08Rik'	0	0	0	0	0	0	0	0.06	0	0.19
77037	'Mrap'	0	2.06	0	0	0	0	0	0	0	0
77038	'Arfgap2'	32.89	38.34	35.2	24.4	18.4	39.04	32.2	32.08	53.49	21.17
77040	'Atg16l1'	30.07	27.02	27.3	33.9	27.6	19.05	17.8	30.77	26.55	32.13
77041	'Arsk'	3.01	0.39	0.21	0.34	2.16	1.62	0.7	0.14	0.95	2.23
77044	'Arid2'	2.63	1.96	0.95	3.95	0.53	0.56	3.02	0.86	1.45	1.92
77045	'Bcl7a'	2.15	3.46	5.63	2.59	10	5.92	3.07	7.46	6.09	7.6
77048	'Cep83'	0.62	1.7	0	4.66	0.25	0.18	0	1.38	0.53	0.9
77053	'Sun1'	20.62	29.54	30.4	17.8	16.2	1.54	17.8	17.86	20.69	26.65
77056	'Tmco4'	0	0	0	0	0.13	0	0	0.4	1.35	0
77058	'Ccdc183'	0.1	0.17	0.03	0	0.09	0.11	0	0.12	0	0
77065	'Ints7'	8.37	6.51	4	4.66	3.55	5.9	1.33	5.37	4.22	9.6
77087	'Ankrd11'	4.23	5.75	5.05	10.9	9.56	5.69	6.62	6.26	3.68	7.13
77090	'Ocel1'	0	0	0.46	2.61	0	0.37	0	1.15	0	2.47
77097	'Tanc2'	7.16	5.77	5.8	11.7	11	6.69	9.95	4.07	5.1	9.04

77106	'Tmem181a'	28.59	47.78	28.5	37.3	40	27.02	21.1	34.36	36.64	34.45
77110	'Gpbp1l1'	2.31	4.36	1.47	1.77	1.62	0.95	1.57	3.54	5.09	4.42
77113	'Klhl2'	5.02	11.16	3.51	8.22	14.4	6.31	3.47	6.38	5.34	8.38
77116	'Mtmr2'	24.6	13.06	23.5	17.1	18.1	10.06	11.3	22.59	18.24	28.91
77117	'Zfp934'	0.03	6.83	3.11	0.27	2.46	0.23	11.2	0.25	3.46	5.6
77125	'Il33'	18.99	4.09	10.2	0.02	8.52	60.8	5.6	0.28	0.02	0
77128	'Crebrf'	7.54	4.26	6.98	9.61	6.04	9.38	9.57	7.22	5.93	11.67
77134	'Hnrnpa0'	11.3	15.61	8.44	11.6	17.2	11.08	11.1	6	11.3	19.5
77219	'Ptgr2'	21.47	22.51	21.5	22.4	15.9	11.2	23.3	25.82	18.76	20.7
77220	'Tmem200a'	14.57	8.07	3.76	3.12	8.66	7.29	4.48	4.97	12.25	5.67
77252	'9430038I01Rik'	2.22	1.56	4.51	0	0	0.84	0	4.57	0	5.12
77254	'Yif1b'	70.11	60.3	72.1	39.2	122	126.1	85.5	91.97	81.88	79.87
77264	'Zfp142'	1.65	0.02	7.51	0.98	0.13	0.27	4.4	1.51	0.01	0
77286	'Nkrf'	10.69	11.49	12.9	11.9	7.74	1.28	8.17	15.41	12.06	23.12
77300	'Raph1'	3.07	3.31	3.34	3.88	1.6	2.3	1.28	2.59	2.32	2
77305	'Wdr82'	33.34	33.91	46	20.5	31.8	28.66	51.4	26.59	24.54	19.02
77318	'Ankrd55'	31.2	10.77	10.9	0.53	9.54	4.84	18.4	21.47	24.28	11.64
77352	'Axdnd1'	0	0	0	0.12	0	0	0	0	0	0
77371	'Sec24a'	2.3	2.6	5.51	0.53	3.09	1.68	0.24	1.92	2.94	2.54
77407	'Rab35'	6.14	6.48	2.2	0.03	11.6	13.3	4.07	4.52	3.58	4.53
77422	'C330018D20Rik'	12.95	17.1	17.5	10.6	15.9	14.01	12.1	12.75	14.13	10.97
77462	'Tmem116'	0	2.43	0.05	0.93	0	0	0	0	6.81	0
77480	'Kidins220'	11.63	14.22	28.5	19.2	11.3	15.61	15.5	16.25	12.75	21.71
77505	'Dnhd1'	0.74	0.52	0.15	2.59	2.25	1.99	0	2.17	1.32	0.11
77519	'Zfp266'	5.23	5.32	4.65	5.59	3.95	4.38	1.95	4.8	2.5	3.86
77521	'Mtus2'	4.76	4.09	6.96	12.4	1.74	9.08	9.15	12.32	9.08	5.11
77531	'Anks1b'	40.03	43.12	42.1	26.7	38.5	20.83	83.8	32.52	18.21	15.23
77532	'Jrkl'	1.23	0.1	0.92	1.49	1.67	2.65	0.18	2.46	2.52	0.23
77552	'Shisa4'	67.76	53.59	102	70.4	82.6	87.5	39.7	68.52	58.53	26.01
77559	'Agl'	1.79	0.56	3.02	0.49	3.66	0.86	1.7	1.78	3.79	2.13
77569	'Limch1'	9.1	10.37	3.88	7.23	12.2	11.15	12.3	6.16	9.07	3.75
77573	'Vps33a'	10.07	12.92	28.6	1.21	27.7	17.83	34.8	18.14	18.08	14.98
77574	'Tcaf1'	49.93	41.1	59.1	36.2	35.9	31.46	52.9	44.84	54.08	42.91
77577	'Spns3'	0	0	0	0	0.58	0	0	0	0	0
77578	'Bcl9'	8.91	8.58	6.53	6.26	3.92	6.29	5.86	3.09	4.68	3.6
77579	'Myh10'	14.33	10.58	10.8	15	13.1	6.57	7.88	5.39	11.85	9.6
77582	'Mboat7'	73.37	89.78	114	53.2	53.9	76.48	54	65.69	94.09	49.54
77590	'Chst15'	0.09	0.09	1.15	0	0	0.07	4.14	5.42	2.16	0.83
77591	'Ddx10'	9.89	14.73	10.2	11.4	22	14.5	6.46	3.4	11.23	6.56
77593	'Usp45'	4.51	2.66	2.31	0.23	0.97	8.38	3.7	1.97	2.62	1.86
77595	'Nup210l'	0.09	0.03	0	0	0	0.01	0.09	0.68	0.01	0.06
77604	'Rbm12b2'	2.65	2.2	4.82	0.97	6.53	0.55	3.13	1.7	7.69	2.49
77605	'H2afv'	82.16	30.25	46.9	24.9	45.3	69.6	38.6	54.95	38.3	44.55
77609	'Ccdc151'	3.78	6.24	3.83	10.4	5.98	9.3	0	0	8.11	2.71
77613	'Prss36'	2.38	5.91	0.91	6.53	16.1	7.16	11.3	7.37	5.19	9.13
77619	'Prelid2'	0	0	0.07	0	0	0	0	1.54	0	0.13
77622	'Apex2'	4.58	16.44	6.17	16.1	4.61	11.16	13	20.89	13.69	13.67
77626	'Smpd4'	15.38	19.46	13.5	8.65	15.1	10.93	25.3	16.4	9.69	15.99
77627	'Efcab6'	0.01	0	0	0	0.35	0	0	0	0	0
77629	'Sphkap'	8.18	7.88	15.2	9.94	6.16	5.1	13.3	19.2	8.2	9.06
77630	'Prdm8'	0	0	0	0	0.34	0	0	0	0	0
77634	'Snapc3'	40.11	23.66	14.8	30.8	38.8	20.59	22.6	24.42	28.53	43.78
77644	'C330007P06Rik'	4.37	12.17	3.61	8.79	10.1	6.77	14.8	11.78	7.27	6.99
77652	'Zfp955a'	5.63	5.61	5.8	0.04	4.44	1.65	7.88	3.72	6.67	2.89
77669	'Arhgef38'	0.02	0	0	0	0	0	0	0	0	0
77683	'Ehmt1'	5.22	10.71	4.12	4.06	13.7	3.58	9.97	4.04	6.5	10.52
77697	'Mmab'	13.34	8.78	13.2	0	10.1	25.41	20.4	16.49	9.49	11.19
77701	'Lcn12'	0	0	0	0	0	0	0	0	0	2.36

77705	'9230104L09Rik'	0.79	0	0	0	0	0	0	0	0	0
77721	'Mrps5'	29.16	53	43.8	46.7	46.5	26.58	61	42.05	30.68	52.95
77733	'Rnf170'	8.41	9.75	10.3	11.9	8.21	9.91	24	7.42	9.96	9.85
77739	'Adamtsl1'	0	0.19	0.88	0	0	0.38	0.23	0	0	0
77744	'Bora'	6.17	8.02	12	18.3	12.8	11.3	11.1	5.51	10.76	8.05
77766	'Elp4'	3.35	1.48	4.62	3.43	0.11	2	3.28	2.7	3.39	4.43
77767	'Ermn'	4.87	9.34	0.5	0	12.6	13.55	0	1.04	0.01	0.01
77771	'Csrnp3'	11.55	13.41	7.58	7.76	5.31	5.63	13.1	9.08	6.91	5.99
77772	'Dcst1'	0	0	0.05	0	0.15	0	0.32	0.05	0	0
77777	'Ulbp1'	0	0	0	0	0	0.03	0	0	0	0
77781	'Epm2aip1'	12.69	19.81	15	28.1	9.94	10.2	14.4	7.37	8.52	13.21
77782	'Polq'	0	0	0	0	0.01	0.04	0.05	0.12	0.17	0.01
77794	'Adamtsl2'	1.53	4.47	0	7.71	0	0.01	0	0.01	0	2.86
77798	'A930009A15Rik'	0	2.35	9.12	0	0	4.77	1.83	0	0	0
77799	'Sla2'	0	0	0	0	0	1.35	0	0	0	0
77803	'Shisal2b'	0	2.73	0	0	4.4	3.71	0	1.06	11.48	11.96
77805	'Esco1'	4.42	7.59	7.81	5.39	9.34	7.53	2.14	3.5	5.78	3.73
77809	'Lrrc42'	10	24.99	15	9.08	21.8	2.09	0.52	11.26	8.75	5.55
77827	'Krba1'	9.07	11.97	13.9	4.71	10.3	5.54	13	7.63	16.09	9.77
77832	'Tchp'	5.64	11.13	8.86	0.07	3.38	2.36	0.05	4.89	0.05	3.84
77836	'Mlana'	0	0.12	0	0	0	0	0	0.48	0	0
77853	'Msl2'	0.46	1.55	0.7	7.52	0.91	0.27	3.23	0.65	0.4	1.03
77862	'Thyn1'	100.1	83.41	137	85.8	94.2	90.03	95.8	119	67.76	54.7
77864	'Ypel2'	6.18	7.88	3.7	9.06	8.31	9.22	3.33	4.17	1.14	1.2
77877	'6030458C11Rik'	9.05	6.21	8.67	9.45	6.35	3.65	13.2	11.69	6.99	4.81
77883	'6030498E09Rik'	0	0	0	0	0	1.43	0	0	0	0
77889	'Lbh'	6.3	4.53	4.56	0	8.54	4.26	9.87	12.21	7.16	7.07
77891	'Ube2s'	26.27	33.11	23.7	15.4	20.8	29.05	24.2	47.9	69.47	32.77
77914	'Krtap17-1'	0	19.98	3.54	31.7	0	0	3.54	4.3	0	2.56
77929	'Yipf6'	4.89	1.44	5.8	8.16	3.48	4.62	1.59	3.27	2.17	8.21
77938	'Fam53b'	2.65	3.24	0.14	0.01	0.91	0.33	1.33	2.4	1.52	0.99
77945	'Rpgrip1'	7.01	5.73	2.62	6.24	14.6	0.82	11	9.54	13.43	5.92
77951	'Cyp20a1'	9.56	12.25	17.4	16.2	10.9	13.36	10.4	0.8	5.87	14.72
77963	'Hook1'	18.2	15.75	12.7	8.29	16.4	21.67	18.4	21.93	26.97	11.44
77974	'Rdh12'	1.38	0	3.52	0	2.25	10.07	0	3.42	10.19	4.76
77975	'Tmem50b'	139.6	91.97	81.4	78.6	66.2	89.68	81.9	102.7	67.57	52.7
77976	'Nuak1'	0.11	0	2.58	0.75	3.37	0.24	2.01	0.02	1.14	1.58
77980	'Sbf1'	10.42	9.57	13.9	1.42	12.5	5.45	18.1	10.14	10.22	4.09
77987	'Ascc3'	2.08	2.51	1.93	2.6	1.24	2.38	2.64	0.68	4.32	1.58
77996	'Cutal'	0	0	0	0	0	0	0	0	0.13	0
77998	'Grifin'	0	0	0	0	0	0	8.38	0	0	0
78004	'Prr15'	0.06	0.11	0	0.84	0.4	0	1.14	0.06	1.3	0.19
78016	'Ccdc150'	0	0	0	0	0	0	0.04	0	0.41	0
78038	'Mccc2'	28.56	21.75	22.2	26.8	22.7	33.27	21.5	26.8	19.61	22.92
78070	'Cpt1c'	78.15	98.67	30.6	48.3	62.5	49.72	43.7	84.32	89.32	81.76
78088	'Sowahb'	0	0	0	6.04	0	0	0	0.98	0	0
78100	'Msantd4'	27	21.31	30.7	28.1	30.4	30.82	25.9	30.52	22.89	15.84
78134	'Lpar4'	0.03	0	0	0	0	0	0	0.07	0	0
78174	'Cox7b2'	2.25	1.65	1.66	0	0	1.69	0	0	2.11	0
78177	'Ninl'	1.24	0.04	0	3.29	2.35	0.01	0	0	0.09	1.22
78232	'Trappc6b'	78.3	45.24	87.3	112	54.6	55.75	21.6	49.93	60.36	48.02
78244	'Dnajc21'	4.75	7.99	2.63	16.2	2.9	4.87	2.39	2.21	0.17	0.17
78245	'Acbd7'	14.58	4.05	13.4	14.2	65.7	0	4.53	4.07	0	3.46
78246	'Phf23'	20.13	35.23	14.9	24.2	27.4	10.42	25.7	28.55	19.99	20.17
78248	'Armcx1'	47.87	51.86	59.5	64.1	50.9	57.65	73.9	61.21	51.1	68.2
78249	'Adgrf4'	2.48	0	0	0	0	0	0	0	0	2.42
78250	'Iqch'	0.51	0.4	0.61	0.44	0.58	0.45	0.46	0.39	0.67	0.31
78251	'Zfp712'	0.32	3.61	1.44	2.75	4.17	3.47	1.55	0.37	0.4	0.67

78252	'Nxpe2'	0	0	0.66	0	0	0	0	0	0	0
78255	'Ralgps2'	4.28	11.89	2.47	6.57	6.39	4.8	3.28	5.68	2.98	4.91
78257	'Lrrc9'	1.84	0.68	2.09	2.27	2.18	1.37	0	0.25	3.52	2.16
78266	'Zfp687'	1.61	4.96	6.01	7.83	3.3	0.36	1.71	1.93	11.11	4.56
78267	'Klhdc8b'	98.16	125.5	54.4	92.1	168	66.5	42.9	37.21	37.48	73.41
78283	'Map7d2'	28.96	29.87	32.3	34.5	35.2	29.05	25.9	36.62	23.95	23.37
78284	'Creb3l4'	0	0.04	0	7.31	0	0	0	0	0	0
78286	'Nav2'	2.24	4.83	2.41	6.02	1.09	1.84	3.32	2.82	5.12	2.77
78287	'Rbsn'	7.35	8.58	8.27	22.9	10.1	1.28	3.01	11.28	9.39	8.58
78294	'Rps27a'	271.2	315	342	366	310	344.8	327	233	188.3	283.56
78303	'Hist3h2ba'	147.9	130.4	135	75.5	162	127.4	120	156.4	117.8	95.83
78304	'Naa38'	122.4	158.5	151	157	110	184.7	164	76.06	124.2	118.7
78308	'Gpr108'	49.14	18.37	22.9	34.1	32.2	37.91	27.4	36.32	37.85	28.06
78309	'Cul9'	4.27	1.82	3.75	1.88	0.44	0.9	2.03	0.98	1.7	3.57
78317	'Ccdc88b'	0.62	0	0.06	0	0	0	0	0	0	0.61
78321	'Ankrd23'	1.49	14.18	6.69	4.36	2.41	11.08	6.69	6.89	11.24	8.82
78323	'Fam219b'	9.22	11.5	12.8	23.8	30.6	13.6	7.06	9.2	17.91	9.31
78330	'Ndufv3'	384.7	303.3	429	510	328	570.7	316	228.5	400.1	382.97
78334	'Cdk19'	2.58	0.3	3.5	0	0.11	0.05	0	1.44	0.13	0
78339	'Ttyh3'	0.74	0.54	2.8	0.84	1.68	0.88	3	1.13	2.31	2.51
78369	'Icam4'	0	0	0	0	3.56	0	0	1.16	2.33	0.49
78372	'Snrnp25'	28.15	29.36	57.3	48	106	95.79	60.8	57.41	23.19	53.11
78373	'Nudt17'	33.82	37.68	26	29.7	23.5	23.97	33.2	23.63	52.55	26.9
78376	'Sapcd1'	0	0	1.43	0	0	1.1	0	0	0	0
78388	'Mvp'	0	0.15	0	0	0.91	4.17	0	0	3.99	0
78390	'Pla2g4d'	0	0	0	0	0	0	0	0	0.57	0
78394	'Ddx52'	5.55	14.6	11	7.47	7.5	12.74	4.94	7.56	7.88	6.14
78405	'Ntf5'	0.95	0	0	0	0	0	0	0	0	0
78408	'Fam131a'	69.99	72.6	43.9	28.8	56.6	52.86	38.2	62.6	34.33	47.66
78412	'Cyren'	3.12	3.19	0.02	0.05	1.79	17.43	0.6	0.54	1.85	4.04
78416	'Rnase6'	11.69	6.64	0.02	0	4.99	0	4.33	4.75	7.25	0
78428	'Pym1'	8.32	9.58	16.2	14.2	8.2	7.72	4.12	19.27	16.06	16.85
78444	'Pgpep1l'	0	0.89	1.95	0	0.8	0.2	0	2.11	0.36	0.33
78455	'Helz'	8.52	3.94	9.18	4.41	8.57	3.22	9.82	5.63	17.85	5.8
78465	'Ccdc190'	0.48	0.71	1.86	0	5.06	0	5.11	0	0	3.16
78473	'Skap1'	2.67	4.28	2.13	0	0.03	3.65	3.71	11	0.04	9.38
78506	'Micu3'	65.78	72.69	49.6	110	63.2	55.23	64.4	58.44	59.12	61.85
78514	'Arhgap10'	0.62	0	0	0	0	0	0	0.02	2.6	0
78521	'B230219D22Rik'	6.98	4.73	4.82	7.37	4	1.31	1.26	5.58	8.56	5.87
78523	'Mrpl9'	36.29	56	57.1	29.4	27.9	23.02	57.6	44.95	14.12	54.34
78541	'Asb8'	41.67	43.67	49	40.2	26.9	56.1	47.7	36.71	40.65	54.49
78558	'Htra3'	0	0	1.25	0.03	0	1.29	0	1.71	0	0
78560	'Adgra2'	0	0.05	0.97	0	0.11	0	0.22	0	0	0.04
78581	'Utp23'	6.1	7.97	8.38	11.7	10.9	2.45	2.95	8.05	8.76	6.72
78586	'Srbd1'	2.61	2.83	1.91	0	3.88	1.63	1.62	0.86	4.07	1.48
78593	'Nrip3'	20.22	22.26	39.6	24.8	47.7	41.44	58.8	24.38	72.03	57.66
78600	'Pde6h'	0	2.35	0.85	0	0	0	0	0	0	0
78610	'Uvrag'	5.21	0.83	0.08	11.1	3.78	3.16	13.6	5.26	3.52	0.05
78617	'Cstad'	4.68	8.78	4.15	0	4.12	0	0	9.48	0	7.28
78618	'Acap2'	0.89	1.72	2.44	1.56	1.77	5.39	2.55	1.62	1.86	1.65
78619	'Zfp449'	2.17	1.99	0	0.71	1.14	0.01	0.09	4.08	1.39	5.35
78625	'1700061G19Rik'	0.15	0	0.74	0	0	0	0.66	0	0.51	0
78651	'Lsm6'	34.19	32.98	28.3	31.2	33.4	44.03	40	33.02	46.62	36.32
78653	'Bola3'	99.04	76.58	66.5	112	83.9	82.19	65.1	79.37	79.73	103.64
78654	'1700066C05Rik'	0.78	0.14	2.5	3.29	1.84	0	0	0.23	0	0.38
78655	'Eif3j1'	5.8	6.84	2.35	4.55	0.89	16.05	1.18	0.5	2.85	3.3
78656	'Brd8'	42.91	35.85	38.9	59.5	56.2	36.13	32.1	30.86	46.97	35.72
78658	'Ncapd3'	1.55	3.71	2.3	3.67	5.48	5.54	7	5.95	2.1	4.07

78670	'Plekhj1'	48.26	57.69	39.3	42.5	56.9	45.65	46.7	61.02	46.98	56.62
78672	'9530057J20Rik'	5.88	4.64	2.83	15.6	8.61	3.13	8.99	4.68	1.27	2.95
78688	'Nol3'	11.7	17.25	17	11.4	24.6	20.96	14	24.37	8.27	19.52
78689	'Naa35'	19.17	25.77	21.9	44.6	20.9	18.86	41.7	25.67	23.78	40.08
78697	'Pus7'	3.16	6.97	6.33	10.1	7.34	0.06	4.77	5.84	2.24	2.68
78703	'Zfp972'	0.89	0.63	3.76	10.6	0.99	0.54	4.98	0.4	2.09	3.4
78748	'Rassf10'	0.22	1.38	0	0	0	0	0	0	0	0
78749	'Filip1l'	0	0.53	0	1.56	0	0	0.02	0	0	0.08
78751	'Zc3h6'	0.15	2.62	1.19	4.1	2.67	0.51	0.31	0.28	0.72	0.68
78752	'Csgalnact2'	0.93	1.51	0	7.42	0	3.62	0.02	1.4	0.05	3.93
78754	'Galnt15'	0	0.08	0	0	0	0	0	0	0	0
78755	'Fam122b'	1.25	2.27	0	0	0.01	0	2.32	4.01	0	3.86
78757	'Rictor'	12.91	14.98	34.4	13.6	26.1	15	28.4	9.16	19.93	24.66
78767	'Efcab11'	0	0	0	0	0	0	0.17	0	0	0
78771	'Mctp1'	0.22	1.46	0.55	0	0.74	0.32	1.19	0.69	0.34	1.18
78772	'Hhipl2'	1.43	0.86	0.29	1.41	0.13	0.25	0.04	0.81	0	0
78774	'Cfap61'	2.09	2.3	0.37	0	3.95	0	0	0.91	0	0.3
78777	'Tepsin'	5.24	7.25	16.9	4.84	2.45	12.71	14.9	15.09	5.88	6.78
78779	'Spata2l'	14.07	10.6	13.8	45.3	11	6.11	9.59	13.75	2.44	4.4
78781	'Zc3hav1'	2.6	1.08	4.78	6.21	1.83	2.64	2.69	0.87	0.08	0.07
78783	'Brpf1'	3.07	3.37	5.93	11.5	0	4.77	1.39	1.24	0.42	0.04
78784	'Celf3'	71.83	94.21	44	53.5	89.7	86.97	56.4	72.88	86.4	98.16
78785	'Clip4'	0.04	5.27	3.92	0.66	2.71	1.27	3.62	0.79	0.72	0.59
78787	'Usp54'	1.5	2.35	4.42	3.93	5.31	0.35	2.27	1.8	3.26	1.98
78789	'Vsig1'	0	0	0	0	7.26	15.93	0	0.02	32.46	5.98
78795	'Armc9'	25.14	21.75	25.8	25.6	30.9	16.04	29.5	40.22	23.06	41.58
78796	'Zcchc4'	6.45	6.73	2.56	6.95	3.93	3.79	7.24	7.13	8.21	14.43
78797	'Ndor1'	10.39	4.81	6.56	8.68	15.3	4.65	15.5	8.02	6.36	11.16
78798	'Eml4'	0.62	1.46	0.28	4.06	1.62	2.15	2.22	0.35	7.53	0.58
78801	'Ak7'	0.47	0	0	0	0	0	0	0.04	0	0
78802	'Ttc30a1'	7.02	6.03	4.67	5.88	0	0	4	2.67	4.03	10.51
78803	'Fbxo43'	0.05	0.04	0.07	0.03	0.08	0.09	0.06	0.01	0.07	0.09
78806	'Stpg1'	0.27	0	0.11	0	0	0	0.77	1.81	0.64	0.24
78808	'Stxbp5'	1.73	1.34	3.39	9.23	3.96	2.08	2.78	1.06	0.98	2.5
78809	'4930562C15Rik'	0	0.28	0	0.7	0	0	0	0	0.36	0
78816	'Gmip'	0.96	6.71	4.88	8.92	4.9	5.69	0	6.33	9.69	13.36
78825	'Desi2'	1.5	0.22	0.64	0.24	0.6	0.13	3.25	2.07	1.9	1.18
78829	'Tsc22d4'	14.1	19.01	9	13	24.9	29.32	34.7	10.11	16.93	12.85
78830	'Slc25a12'	110	117.7	152	87.4	60.5	62.33	126	143.7	89.18	87.4
78832	'Cacul1'	9.75	4.3	11.7	12	5.17	7.38	7.98	7.24	12.39	2.46
78833	'Gins3'	2.74	0	1.09	0	2.07	2.26	0	0	2.85	2.06
78834	'Zfp623'	9.81	10.71	8.72	0.27	15	6.24	7.98	15.85	7.85	1.98
78885	'Coro7'	9.69	8.68	8.8	13.6	23.5	14.88	20.2	23.81	22.48	12.02
78887	'Sfi1'	2.42	9.23	12.1	0.07	5.84	11.74	6.79	8.3	7.8	2.5
78889	'Wsb1'	101.7	134.7	92.5	113	103	69.3	97.4	101.2	210.1	116.8
78890	'Trmt44'	2.25	3.72	3.51	7.06	2.04	0	0	3.34	0.83	0.08
78891	'Scyl1'	20.31	44.88	32.5	21.4	18.8	25.48	47.3	45.34	38.41	29.49
78892	'Crispld2'	0	0	0.01	0.37	4.02	0.04	0	0	0	0
78893	'Cnot10'	22.44	27.69	30.6	61.2	26.7	34.4	22.5	41.14	27.36	40.58
78894	'Aacs'	22.33	21.84	13.8	6.58	20.6	12.35	13.2	18.71	30.16	25.17
78895	'Pus7l'	0	0	0	0	0	0	0	0	2.33	2.03
78896	'1500015O10Rik'	3.59	0	0	0	0	0.05	0	0	0	0
78903	'Wrnip1'	14.25	7.41	11	17	10.1	16.92	6.65	10.54	15.88	3.83
78906	'Misp'	1.93	0	0	0	0	0	0	0.35	0	0
78908	'Igsf3'	4.33	4.06	7.87	8.31	1.89	4.98	0.01	5.49	10.45	1.99
78910	'Asb15'	0	0	0.01	0	0	0.01	0	0	0.02	0
78912	'Sp2'	3.16	2.1	2.28	0	8.16	0.83	2.39	2.11	6.08	4.39
78913	'Ltn1'	1.99	1.45	12.4	2.3	2.61	0.62	2.01	2.77	4.85	4.31

78914	'Nadsyn1'	5.82	8.17	4.34	13.1	6.06	2.27	5.61	3.85	5.5	12.61
78919	'Fndc8'	0	1.15	0	0	0.76	0	2.41	0	1.42	0.39
78920	'Dlst'	15.86	18.2	45.8	12.8	12.3	8.36	40.2	26.08	16.08	17.11
78921	'9130019O22Rik'	2.4	0.12	3.82	0.01	4.24	0.22	0.01	6.95	1.83	1.58
78923	'Chsy3'	1.08	1.02	0	0	0	0	0	0	0.22	1.34
78925	'Srd5a1'	12.79	6.28	1.46	5.09	11.2	2.68	11.7	15.17	2.22	0
78926	'Gas2l1'	4.46	1.16	0.44	5.99	4.74	0.16	0.75	2.71	10.21	0.95
78928	'Pigt'	31.39	25.22	46.9	1.12	14.9	24.43	48.1	24.9	40.14	14.04
78929	'Polr3h'	60.56	63.74	65	45.5	68.1	51.62	76.9	78.27	55.1	20.41
78933	'Agbl4'	14.51	10.83	16.4	19.5	17	11.01	5.52	10.98	3.95	12.8
78935	'Saal1'	3.01	10.17	6.28	8.2	7.73	1.32	22.9	10.52	6.46	4.72
78937	'Avl9'	2.2	1.79	2.05	2.25	3.3	1.32	4.17	2.92	2.08	3.48
78938	'Fbxo34'	13.49	17.48	8.55	40.9	3.38	16.31	19	18.76	23.83	19.82
78943	'Ern1'	0.61	0.05	0.12	0	0.18	0	1.28	0.07	0	0.47
78977	'Popdc3'	0	1.93	0	0	0	0	0	0	0.05	0
79043	'Spsb3'	47.74	41.25	51.4	9.61	33.4	49.01	46.9	46.81	46.23	32.48
79044	'Mrps34'	109	84.36	119	155	121	119.2	95.5	115.5	128.5	100.48
79059	'Nme3'	90.21	82.16	100	147	92.3	172.9	74.1	134.5	59.87	95.82
79196	'Osbpl5'	3.19	5.19	0.04	2.49	1.86	0.17	15.6	0.74	7.11	4.49
79202	'Tnfrsf22'	2.71	1.63	6.42	3.33	4.16	1.73	2.19	1.91	2.63	0.98
79221	'Hdac9'	1.11	0.79	0.41	0.07	2.59	4.87	0	1.69	2.25	0.52
79233	'Zfp319'	0.81	3.99	3.04	0.15	0.83	0.84	2.7	1.27	0	0.92
79263	'Trim39'	5.38	1.52	1.89	4.92	13	0.25	5.8	4.34	3.8	9.22
79264	'Krit1'	7.94	5.52	5.82	3.5	3.99	4.69	7.85	6.73	11.39	5.81
79362	'Bhlhe41'	42.43	24.59	30.4	59.1	41.4	17.38	31.2	28.19	31.23	39.87
79401	'Spz1'	0	0	0	0	0	0	0	1.87	0	0
79456	'Recql4'	0	0	0	2.3	0.05	0	0	0	0	0.51
79464	'Lias'	18.02	18.16	32.6	28.2	35.1	42.17	13.7	19.12	25.24	25.56
79554	'Cptp'	15.01	21.53	39.3	33.4	14.5	29.13	20.2	43.87	37.15	10.98
79555	'BC005537'	5.87	1.93	8.81	7.53	2.36	2.81	3.42	2.06	10.52	2.14
79560	'Ublcp1'	38.99	16.78	46.9	18.3	42.5	47.2	50.9	35.35	46.63	32.81
79565	'Mettl27'	0	5.32	5.75	9.65	6.47	21.39	4.58	2.25	0.37	6.46
79566	'Sh3bp5l'	20.12	21.23	23.3	0.02	24.3	10.24	28.3	26.72	11.65	24.67
80280	'Cdk5rap3'	38.18	43.52	39.7	32.7	28.4	65.28	38.1	44.47	33.54	47.49
80281	'Cttnbp2nl'	1.12	0.39	0.94	3.52	2.87	0.5	0.01	1.29	0.63	3.23
80283	'Abtb1'	16.79	11.69	22.8	21.1	30.8	22.4	14.4	17.64	26.14	10.12
80284	'Smim12'	60.73	53.66	79.6	0	92.6	70.23	67.1	68.07	37.15	33.64
80285	'Parp9'	0.02	0.02	0.11	0.59	0.69	1.08	4.76	0	0	0
80286	'Tusc3'	281.9	359.9	227	318	253	286.5	350	214	265.4	223.06
80287	'Apobec3'	1.33	0.75	0.91	0	1.45	1.37	4.18	1.98	0.48	2.88
80288	'Bcl9l'	2.06	1.26	3.44	0.84	1.4	1.94	2.85	0.18	0.52	0.17
80289	'Lysmd3'	0	4.41	0.02	0	2.07	0.83	3.51	2.36	2.93	4.23
80290	'Gpr146'	0.74	2.39	1.76	3.31	0.01	1.94	4.85	1.22	1.34	2.06
80291	'Rilpl2'	25.7	15.96	15.4	24.5	28.7	10.88	14	15.03	25.17	8.59
80292	'Zxdc'	0.92	2.07	0.83	8.18	2.56	0.03	4.51	0.87	0	1.63
80294	'Pofut2'	33.88	42.01	18.6	28.8	43.6	29.62	9.91	34.57	43.25	43.71
80297	'Sptbn4'	0.89	2.48	0.97	0.45	4.41	0.2	3.6	1.83	3.46	2.11
80334	'Kcnip4'	107.1	59.31	65.9	35.5	41.6	70.39	27.7	29.72	60.73	74.08
80384	'Tex21'	0	0	0.22	0	0	0	0	0	0	0
80385	'Tusc2'	63.22	38.29	28.4	71.7	40.4	30.58	56.3	41.66	45.32	4.82
80509	'Med8'	16.8	19.54	27	13.8	8.73	16.77	9.78	29.86	44.98	21.62
80517	'Herpud2'	1.5	4.58	4.14	6.73	6.89	6.15	2.61	4.44	0	0
80707	'Wwox'	10.05	20.32	17.5	12.7	7.38	16.54	25.3	30.14	27.93	20.71
80708	'Pacsin3'	13.8	4.56	10.7	0.57	3.54	5.07	8	8.7	0.24	3.62
80718	'Rab27b'	9.65	13.85	6.2	12.4	39.3	14.84	9.2	13.45	28.02	36.34
80719	'Igsf6'	1.49	0	0.71	0.83	0	0	0.13	0	0	0
80720	'Pbx4'	20.28	17.7	21	15.2	24.1	21.99	29.5	12.11	14.95	36.68
80721	'Slc19a3'	0	0	0	0	0.08	0	0	0	0.08	0

80732	'Mynn'	4.81	3.81	0.87	2.58	2.38	0.45	2.15	4.42	7.34	6.16
80733	'Car15'	1.91	0.33	0	0	3.57	0.03	0	0.11	1	1.34
80743	'Vps16'	38.86	30.57	52.9	15.7	69.9	39.71	67.2	46.4	67.25	65.78
80744	'Cwc22'	0.73	7.25	5.29	2.5	6.24	1.74	0.31	3.63	1.5	3.82
80748	'BC004004'	91.8	95.78	63.9	113	72.7	115.2	78	114.5	82.54	119.29
80749	'Lrfn1'	2.6	0	0.1	0.42	0.62	0.52	0.02	2.27	0.87	1.1
80750	'N4bp1'	4.67	6.24	3.27	6.44	4.16	4.47	2.36	2.83	4.34	7.17
80751	'Rnf34'	25.88	40.87	53.9	29.1	29	36.46	41.5	31.67	44.94	32.01
80752	'Fam20c'	32.19	24.46	20.8	31.5	18.2	14.89	47.4	28.64	15.33	10.63
80794	'Cblc'	0	0.03	1.31	0	0	0	0	0	0	0
80795	'Selenok'	400	464.9	246	475	425	469.8	323	363.3	382.9	481.93
80797	'Clca3a2'	0	1.04	0	0.55	0.1	0.39	0	1.46	0	0.02
80837	'Rhoj'	2.25	1.3	0.87	1.2	1.37	1.48	3.45	0.12	1.05	0
80859	'Nfkbiz'	0	12.36	7.5	9.9	0.59	3.39	0.34	0.02	0.02	3.01
80860	'Ghdc'	1.39	3.96	8.86	12.5	11.2	14.6	0.1	3.33	2.1	6.76
80861	'Dhx58'	0.04	2.1	2.56	2	0	1.77	0	0.55	0	0.02
80876	'Ifitm2'	0	1.24	0.5	30.1	0.34	13.61	0	1.6	0.9	5.77
80877	'Lrba'	0.45	0.4	1.49	0.02	8.69	1.64	0.3	0.55	1.43	1.52
80879	'Slc16a3'	0	0	0	0	0	0	0	3.04	1.28	0
80880	'Kank3'	1.38	6.4	3.12	1.98	7.26	2.75	5.74	4.32	6.4	9.76
80883	'Ntng1'	15.3	6.27	0.44	14.2	6.56	2.11	9.7	4.89	0.03	0
80884	'Maged2'	98.63	134.4	131	150	86.3	116.3	141	160.2	115.6	130.53
80886	'Senp3'	23.06	13.55	15.4	8.52	24.3	10.23	6.25	17.22	14.64	14.64
80888	'Hspb8'	3.29	10.34	19.7	10.7	25	1.67	5.29	2.97	4.16	43.69
80889	'Tlnrd1'	1.14	2.23	0.06	3.78	0.35	0	1.21	0.16	0.78	1.33
80890	'Trim2'	36.98	38.94	43.1	23.4	30.8	24.08	33.7	34.92	28.64	35.2
80891	'Fcrls'	1.44	5.29	3.78	5.94	6.89	18.72	0	0	0	0
80892	'Zfhx4'	3.4	1.57	3.5	3.47	3.33	5.05	2.59	3.93	3.97	4.23
80893	'Tmprss5'	0	0	0	0	0	0	0	0	0	0.13
80898	'Erap1'	1.24	0.01	0.83	0	0	0	1.2	1.55	8.74	1.74
80902	'Zfp202'	3.11	5.74	0.57	3.05	1.9	6.31	1.08	4.53	0.57	1.48
80904	'Dtx3'	81.51	76.86	78.4	36.6	135	55.08	119	97.9	128.1	108.51
80905	'Polh'	1.64	1.98	2.71	0.55	1.48	0.01	0.94	0.88	0.48	0.56
80906	'Kcnip2'	8	15.55	14.1	7.48	21.3	35.24	2.9	8.25	12.06	51.66
80907	'Lactb'	0	1.47	3.91	1.88	2.07	0	2.45	1.83	1.36	0.18
80909	'Castor2'	11.78	11.73	7.59	4.34	4.64	2.95	3.76	4.54	8.94	6.35
80910	'Gpr84'	0	0.98	0	10.4	0	8.74	0.03	0	0	0
80911	'Acox3'	1.74	4.32	11.1	3.16	7.41	1.15	2.54	8.34	3.51	2.89
80912	'Pum1'	23.04	20.9	21.8	28.5	17.8	14.52	28.4	18.38	26.78	26.32
80913	'Pum2'	9.37	9.55	6.46	16.2	11.9	6.2	5.98	5.77	7.7	7.88
80914	'Uck2'	2.15	1.1	4.39	4.02	4.23	0	7.58	4.42	3.53	0.3
80915	'Dusp12'	13.1	14.5	7.56	0	2.22	20.67	20.7	32.28	30.37	15.03
80976	'Syt13'	53.51	30.96	64.7	17.4	41.7	16.84	36.3	34	61.59	18.39
80979	'Slc26a5'	0	0	0.05	0	0	1.18	1.18	0	0	0.08
80981	'Arl4d'	10.88	0	3.08	0	6.08	0	7.81	4.93	0.03	0.03
80982	'Cemip'	0	0.45	0	0	0	1.07	0	0.89	0.01	0
80985	'Trim44'	10.99	6	11.3	16.8	4.4	11.85	8.24	3.69	5.07	5.33
80986	'Ckap2'	0	1.61	0	0	0	0	0	0	0	0
80987	'Nckipsd'	48.8	26.69	53.3	23.4	36.9	41.31	102	47.09	42.69	40.72
81000	'Rad54l2'	2.01	1.07	2.37	0.61	3.39	2.74	1.76	0.72	0	0.97
81003	'Trim23'	12.06	13.82	14.5	18	8.54	6.48	6.63	13.95	10.79	12.93
81004	'Tbl1xr1'	0.48	0.83	1.75	0	1.51	0.77	1.87	0.45	1.21	0.06
81011	'Vmn1r148'	0	0	0.02	0	0	0.29	0	0	0	0
81013	'Vmn1r65'	0.38	0.22	0.49	1.1	0.85	0.28	0.7	0.43	0.37	0.33
81014	'Vmn1r58'	10.98	13.26	14.5	20.4	16.4	10.67	15.9	9	16.23	11.73
81018	'Rnf114'	39.67	41.61	32.3	21.4	51.8	29.24	52	44.28	36.85	39.85
81489	'Dnajb1'	72.17	128.2	118	227	36	60.04	54.8	44.32	67.91	114.97
81500	'Sil1'	86	95.84	52.1	55.7	52.2	80.7	66.4	61.9	112	97.09

81535	'Sgpp1'	1.2	4.8	4.46	1.96	5.01	4.67	2.22	4.85	1.36	0
81600	'Chia1'	0	3.22	0.03	9.84	23.7	7.01	0.37	0	0	2.94
81601	'Kat5'	41.97	34	30.7	20.9	27.9	45.2	33.2	54.23	35.85	33.52
81630	'Zbtb22'	0.02	7.25	9.39	0	6.31	2.24	5.34	0.81	1.83	0
81701	'Egfl8'	0	0.04	0.3	0	0	1.47	0.29	0	0	0
81702	'Ankrd17'	12.98	15.62	12.7	13.8	7.79	7.77	7.58	9.55	17.69	8.35
81703	'Jdp2'	0.9	0.25	0.19	2.63	0.47	1.09	0.13	0.23	0.96	0
81799	'C1qtnf3'	0	0	0	0	0	0	0	0	0	0.05
81840	'Sorcs2'	0.63	1.54	3.35	0.02	1.47	4.05	0	0.85	5.01	4.48
81845	'Gpank1'	9.84	13.47	24.3	1.43	26	15.84	12.1	35.39	14.62	23.01
81877	'Tnxb'	0.9	1.74	4.06	6.46	1.18	0.29	0.71	0.02	1.54	0.26
81879	'Tfcp2l1'	0.55	0	0	0	0	0.01	0.01	0.69	0.03	0
81896	'Ift122'	6.56	5.75	2.96	14.5	13.3	6.34	10.2	8.45	5.2	14.62
81898	'Sf3b1'	119.4	86.25	103	136	97.7	99.69	85.9	97.97	107.5	120.65
81904	'Cacng7'	0	0.09	1.11	0.22	0	0.67	0	0	0	0.21
81905	'Cacng8'	0.03	0.9	0.23	0.24	0	0.13	0	0.25	2.49	1.01
81906	'Cyp4x1'	5.27	17	9.58	7.92	1.44	2.49	7.71	8.1	7.53	16.43
81907	'Tmem108'	12.7	5.25	19.1	6.47	17.7	12.04	13	13.43	17.07	24.5
81909	'Zfpl1'	71.17	70.29	67.3	63.4	63.6	81.66	56.3	51.98	99	85.28
81910	'Rrbp1'	2.48	5.51	5.15	25.6	8	5.55	4.27	3.66	6.6	2.41
83383	'Tfap4'	0	1.79	0.05	0	0.21	1.79	2.67	3.96	1.69	2.72
83396	'Glis2'	0.25	1.29	3.47	0	4.26	0.14	0.27	1.67	1.51	1.03
83397	'Akap12'	0.97	2.4	4.35	11.4	4.16	1.88	1.11	2.23	2	5.96
83398	'Ndst3'	2.13	0.97	2.83	3.09	3.49	6.44	8.58	3.72	1.74	2.89
83408	'Gimap3'	0	0.02	0	0	0.05	0	0	0	0	0
83409	'Lamtor2'	219	242	222	303	254	377.6	296	310.4	375.8	265.93
83410	'Cstf2t'	36.73	26.28	40	22.8	17.1	4.37	40.6	28.51	41.78	35.73
83428	'Ucn3'	0	0.03	0.07	0.04	0.03	111.8	0	0	207.1	27.12
83429	'Ctns'	8.3	12.53	10.8	10.4	5.31	11.52	0.06	12.02	6.05	7.97
83430	'Il23a'	0	2.01	0	0	0	0	0	0.03	0	0
83431	'Ndel1'	71.49	78.89	63.5	102	41	42.06	37.9	65.46	53.81	63.52
83433	'Trem2'	0	7.21	0.05	10.8	6	86.16	6.52	4.55	0.04	4.12
83434	'Rsph6a'	0	0	0.47	0	0	0.55	0	0	0	0.19
83435	'Plekha3'	7.29	2.98	0.65	7.03	1.94	9.13	2.65	0.92	2.59	3.18
83436	'Plekha2'	0.01	0.04	0.33	3.53	0.08	0	3.31	0.05	3.67	0
83453	'Chrdl1'	0	0	1.98	0	0	0	0	0	0	0
83454	'Nxf2'	0	0	0	4.13	0	0	0	0	0	1.03
83456	'Mov10l1'	0.03	0	0.09	0.03	0	0.03	0.06	0	0	0.08
83485	'Ngrn'	44.81	50.42	62.7	67.2	64.7	53.32	55.1	54.08	50.87	49.47
83486	'Rbm5'	68.49	64.06	61.5	73.7	49.8	47.98	48.2	59.88	49.46	84.65
83490	'Pik3ap1'	0	0	0	0	0.22	0	0	0.56	0	0
83493	'Sacm1l'	37.6	20.06	19.8	23.4	18	16.66	36.1	31.39	43.01	39.95
83553	'Tktl1'	0	1.95	0.06	0	0	0	0	0.03	0	0
83554	'Fstl3'	0	9.59	1.41	0	4.5	0	0.31	0	0	1.84
83555	'Tex13b'	0.02	0	0	0	0	0	0	0	0	0
83556	'Tex16'	0	0	0.01	0	0	0	0	0.13	0.02	1.45
83557	'Lin28a'	0	0	0	0	0	0	0	0.02	0	0
83560	'Tex14'	0.01	0	0	0	0	0	0	0	0.04	3.33
83602	'Gtf2a1'	7.78	7.51	8.01	7	6.34	1.2	7.96	4.7	4.24	7.28
83603	'Elovl4'	25.68	29.68	23.1	9.93	24.5	10.86	33.1	35.38	24.72	21.37
83669	'Wdr6'	271.5	223.9	358	233	307	128.2	235	291.4	229.5	310.53
83671	'Sytl2'	3.28	0	0.65	0	8.46	5.38	10.8	6.14	6.63	2.11
83674	'Cnnm1'	5	1.93	8.22	6.84	8.26	7.89	6.12	7.51	5.06	9.41
83675	'Bicc1'	0	0	0	1.59	0.01	0.8	0	0.11	0	0.01
83679	'Pde4dip'	12.03	13.1	19.2	14.2	10.3	7.61	4.71	9.99	8.5	8.24
83691	'Crispld1'	0	2.7	0	0	2.69	0.98	3.11	0.97	0	0.33
83701	'Srrt'	26.14	16.13	17.2	15	25.4	28.68	19.8	29.35	29.59	17.22
83703	'Dbr1'	12.61	10.76	16.9	21.9	3.29	3.06	15.6	12.36	12.23	12.32

83704	'Slc12a9'	0.73	11.22	9.68	5.11	6.13	1.14	12	3.6	0.12	0.79
83762	'Otof'	0.01	2.12	9.94	2.21	2.23	1.76	11.6	2.02	1.34	0.51
83766	'Actl6b'	64.08	82.35	64.3	74.6	94.2	51.15	62.6	75.44	75.02	104.53
83767	'Wasf1'	17.55	12.32	16.3	25.3	24.9	2.92	14.4	12.62	21.3	6.03
83768	'Dpp7'	53.01	35.03	16.1	45.4	40.5	62.56	30	32.42	20.79	52.93
83770	'Tas1r2'	0	0	0	1.44	0	0	0	0	0	0
83771	'Tas1r3'	0	0	0	0	1.04	0	0.01	0	0	0
83796	'Smarcd2'	2.39	4.27	0	0	2.26	0	2.67	0	0.8	0
83797	'Smarcd1'	6.03	8.26	6.28	11.4	7.74	3.03	2.83	5.05	5.16	3.61
83813	'Tnk1'	0.02	0	0	0	0	0	1.67	0	0	0
83814	'Nedd4l'	7.77	7.31	4.73	8.98	7.94	8.44	3.83	6.51	10.87	6.93
83815	'Cenpq'	4.63	1.18	6.19	0	3.08	0.39	0	2.4	0	0.81
83885	'Slc25a2'	0.67	0	0	0	0	0	0	0	0	1.87
83921	'Cemip2'	2.51	2.4	1.4	2	1.06	1.39	1.52	0.7	0.22	0.84
83922	'Cep41'	5.68	8.65	1.06	0.15	1.71	2.16	13.1	3.16	8.54	17.37
83924	'Gpr137b'	7.44	1.64	7.14	16.1	5.55	13.26	10.5	6.93	20.17	14.25
83925	'Trps1'	0.51	4.69	0.14	4.67	0.84	0.33	1.47	0.04	0.88	0.94
83945	'Dnaja3'	51.95	45.16	51.4	43.2	31.1	44.23	66	63.36	56.05	72.69
83946	'Phip'	3.57	6.14	4.97	11.2	8.54	4.47	2.89	4.33	8.15	8.58
83961	'Nrg4'	1.08	0.31	1.15	0.9	0	2.41	0.57	2.14	1.17	0
83962	'Btbd1'	8.26	15.71	4.16	9.78	8.36	8.45	4.34	7.11	2.39	9.73
83964	'Jam3'	6.05	15.81	17.9	0.93	14.9	15.93	9.96	9.71	5.74	7.28
83965	'Enpp5'	160.9	154.3	127	128	155	170.1	113	171.6	200.6	181.63
83984	'Tssk6'	2.47	6.84	0	0	1.84	6.97	3.88	0	4.83	0
83997	'Slmap'	2.57	2.74	5.96	5.78	4.09	0.55	1.62	0.9	2.35	1.78
84004	'Mcam'	0.08	0.1	6.8	0	0.07	0.02	0	0	0	0
84035	'Kremen1'	0.01	0.84	0	0	0	0	0	0.01	0	0
84036	'Kcnn1'	1.52	0.01	5.2	0	0	0	1.5	3.8	0	0.01
84092	'Usp8'	31.73	16.34	28.4	35.5	17.5	21.83	42.7	36.16	35.81	27.01
84094	'Plvap'	11.79	16.44	32.3	3.31	10.5	36.25	27.5	15.77	19.87	15.97
84095	'Pi4k2a'	2.66	3.81	6.1	23.5	14.4	0.24	3.26	5.39	3.4	2.36
84111	'Gpr87'	1.3	0	0.44	0	0	0	0	0	0	0.69
84113	'Ptov1'	38.97	47.27	53.1	75.1	55.7	86.51	79.2	58.22	43.59	46.84
84505	'Setdb1'	2.26	9.33	8.1	3.41	6.64	2.04	2.88	5.01	5.97	9.8
84544	'Cd96'	0	0.68	0	0	0	0	0	0	0	0
84585	'Rnf123'	10.2	10.2	13.8	2.4	6.2	1.87	14.2	10.61	15.44	12.85
84652	'Fam126a'	2.46	3.7	5.42	0.04	1.19	1.32	1.28	0.3	1.67	3.18
84653	'Hes7'	0.02	0.05	0.26	0.06	0	0.21	0	0.04	0	0
84682	'Cox4i2'	0	0	0	0	0	0	0	0	0	2.32
84704	'Snurf'	55.05	74.86	71.4	61.7	88.8	101.1	74	74.75	59.81	113.08
85031	'Pla1a'	1.05	0.24	0	3.78	0	0	0	1.12	0	0
85305	'Kars'	67.74	60.91	80.5	51.8	94.4	63.94	104	71.73	91.64	67.69
85308	'Emc9'	187.1	110.4	128	139	161	301.5	140	201.3	111	143.82
93670	'Tac4'	1.05	0	0.59	0	0	0	0	0	0	0
93671	'Cd163'	0.42	0	0	0	0	0	0	0	0	0
93674	'Nat8f3'	0	0	0	0	0.4	0	0	0	0	0
93675	'Clec2i'	0	0	0	0.1	0	0	0.04	0	0.1	0
93677	'Lmod2'	0.02	0	0.1	0.03	0.05	0.02	0.03	0	0.09	0.02
93679	'Trim8'	12.82	14.33	11	15.6	9.41	6.53	16.3	5.63	10.72	8.31
93681	'Zkscan8'	0.11	3.68	1.58	0	3.15	0.13	0	3.52	0	1.76
93683	'Glce'	7.09	2.8	2.14	1.25	7.14	0.2	5.49	3.38	4.8	9.82
93684	'Selenof'	271.2	206.8	238	318	291	289.7	247	254.2	176.9	294.92
93685	'Entpd7'	2.1	3.58	3.91	0.9	1.06	1	0.84	0.81	0.12	2.86
93686	'Rbfox2'	20.09	14.77	14.4	14.2	22.7	11.17	30.9	17.9	15.03	18.48
93687	'Csnk1a1'	99.64	134.4	110	91.4	85.8	89.9	123	103.9	88.09	105.15
93688	'Klhl1'	21.39	2.96	0.02	0.83	6.77	2.95	19.9	13.45	6.17	4.56
93689	'Lmod1'	0.1	0.36	0	0	0	0	0	0	0.14	0.12
93690	'Gpr45'	0.89	1.85	0.01	0	0	0	1.19	0.97	2.41	0.82

93691	'Klf7'	29.81	18.39	20	45.9	15.8	40.58	2.91	6.87	28.7	42.85
93692	'Glrx'	38.36	34.32	63.1	26.9	47.8	55.31	69.3	67.62	64.42	7.19
93695	'Gpnmb'	1.63	0	0	0	0	0	0	0	0	0
93696	'Chrac1'	88.24	61.05	59.9	23.5	98.8	84.16	86.5	62.23	54.62	61.17
93697	'Ice2'	5.71	3.71	1.77	1.32	2.21	1.48	1.62	3.28	4.95	2.49
93699	'Pcdhgb1'	0	1.93	0.59	0	0	0.1	0	1.2	0	0.1
93700	'Pcdhgb2'	0	0.9	4.4	15.3	2.27	0.46	0.02	2.06	0	4.31
93701	'Pcdhgb4'	0.75	1.89	4	0.04	0	0	0	0	1.08	0
93702	'Pcdhgb5'	0	0	0.74	0	0	0	0	0.65	3.06	0.56
93703	'Pcdhgb6'	3.61	10.28	8.26	0	1.53	3.86	6.67	1.16	14.56	7.24
93704	'Pcdhgb7'	1.87	0.64	4.79	0	3.85	0.88	6.27	2.96	0	2.78
93705	'Pcdhgb8'	1.35	0.21	1.62	0	0	0	3.71	0	0	0
93706	'Pcdhgc3'	1.73	3.65	5.83	0	0.13	0.06	1.85	0	0.05	1.65
93707	'Pcdhgc4'	20.63	13.67	10.5	7.19	11.2	7.97	21.9	17.88	20.99	23.47
93708	'Pcdhgc5'	7.02	0	4.33	0.86	3.58	0.03	0	0.84	0.02	6.87
93709	'Pcdhga1'	0	2.53	3.48	0	0	0	0	0	0	0
93710	'Pcdhga2'	0.95	1.66	12	0	3.84	0	0.09	1.49	2.58	0
93711	'Pcdhga3'	2.95	3.74	0.21	0	8.51	1.26	1.1	0.23	4.23	4.35
93712	'Pcdhga4'	2	2.27	8.84	0	7.92	0.36	2.65	5.3	2.79	4.92
93713	'Pcdhga5'	0.14	2.53	2.14	4.62	2.52	4.95	0	2.05	0	6.58
93714	'Pcdhga6'	2.85	2.52	13.8	0.56	6.29	0.09	0.66	7.67	6.16	1.67
93715	'Pcdhga7'	9.71	12.21	9.8	5.36	6.76	8.5	13.4	6.24	4.58	7.64
93716	'Pcdhga8'	0.48	1.49	8.92	0	0	0.22	0	5.51	2.98	9.38
93717	'Pcdhga9'	2.57	1.29	1.19	4.1	0	1.13	0	0	0	0.56
93721	'Cpn1'	0	0.03	0	0	0	0	0	0	0	0
93722	'Pcdhga10'	0.35	2.86	2.26	0	0	1.33	0	3.55	0	0
93723	'Pcdhga11'	9.85	1.45	2.64	3	2.33	0.11	3.22	2.93	2.82	4.92
93724	'Pcdhga12'	2.47	3.29	1.73	0	0.44	2.69	0	5.26	10.22	1.18
93728	'Pabpc5'	0	0	2.03	0	1.93	4.1	0	0	0.14	0
93730	'Lztfl1'	9.79	7.05	9.7	5.5	10.9	10.63	8.24	8.38	3.01	10.2
93734	'Mpv17l'	37.2	35.9	20.9	42.6	31	26.37	19.9	36.42	30.87	30.22
93735	'Wnt16'	0	0	0	0	1.27	0	0	0	0	0
93736	'Aff4'	9.15	5.39	7.28	9.78	5.2	1.11	5.18	4.16	2.9	4.88
93737	'Pard6g'	0.69	0.47	1.67	8.24	0.46	4.03	0.18	2.59	2.7	0
93739	'Gabarapl2'	541.3	682.7	555	653	498	714.1	571	458.3	530.2	651.81
93742	'Pard3'	5.41	2.6	1.56	0.04	3.14	0.83	1.1	2.97	0.46	0.49
93746	'Gprc5d'	0	0.99	0	0.22	0	0.39	0	0	0	0
93747	'Echs1'	36.78	38.61	48.5	28.1	43.8	74.74	49.2	57.73	48.07	32.96
93757	'Immp2l'	7.04	2.62	10.5	6.34	17.1	13.26	14.9	1.44	0	8
93759	'Sirt1'	1.09	0.35	1.53	0	0.15	1.49	2.36	2	2.17	1.58
93760	'Arid1a'	5.25	8.59	7.6	10.3	4.66	5.3	1.54	3.42	5.99	2.89
93761	'Smarca1'	15.19	16.62	7.61	23.1	9.3	7.91	10.9	15.26	7.88	20.06
93762	'Smarca5'	6.15	7.24	4.39	14.4	3.12	1.11	6.45	5.78	2.89	3.68
93765	'Ube2n'	168.2	136.4	134	163	124	168.6	168	203.3	195.5	176.77
93790	'Nipa2'	7.93	10.41	7.84	20.4	6.17	4.25	5.74	5.56	7.63	5.06
93834	'Peli2'	0.05	0.03	0	2.54	0	0	0.05	1.46	0	0
93835	'Amn'	5.07	0.34	22.1	4.09	6.92	20.77	2.79	5.13	19.69	12.81
93836	'Rnf111'	7.53	1.88	5.08	3.59	2.74	1.58	9.05	1.06	5.21	2.42
93837	'Dach2'	0	0	0	0	0	2.01	0	0	0	0.01
93838	'Dqx1'	0.06	0	0.05	0.11	0.03	0.03	0.02	0.97	0.01	0.93
93840	'Vangl2'	2.21	7.6	18.3	0.01	6.04	13.52	3.88	19.78	20.51	11.1
93841	'Uchl4'	0	0.26	0.04	9.1	0	2.4	0.05	0	0.94	0
93842	'Igsf9'	2.47	3.31	4.87	0	2.8	2.83	0.03	1.99	2.65	1.23
93843	'Pnck'	672	657.3	558	703	496	824.1	645	667.7	382.9	529.14
93871	'Brwd1'	40.49	41.36	66.5	28.1	24.7	15.62	84.1	27.26	41.28	25.11
93873	'Pcdhb2'	2.8	0	0	0	0	0	0	0	0	0
93874	'Pcdhb3'	1.24	2.74	2.02	0	0.47	0	0.01	0.83	0	0
93875	'Pcdhb4'	0	0	0	1.33	1.35	0	0	1.13	0	1.13

93876	'Pcdhb5'	0.79	4.98	5.82	0.03	1.25	0	0	0.74	2.7	0
93877	'Pcdhb6'	0	1.02	0	0	0.02	0	0	0.59	0	0
93878	'Pcdhb7'	5.34	2.29	3.41	6.92	0	0	0.01	0	5.03	10.56
93879	'Pcdhb8'	0	4.62	0	0.02	0	0	0	1.5	0	0
93880	'Pcdhb9'	20.2	3.06	20.8	14.8	33.8	0	10.9	36.25	3.64	0.76
93881	'Pcdhb10'	1.64	2.5	0	0	0	0	0	4.35	5.31	0
93882	'Pcdhb11'	0.38	0	0	1.88	1.68	0	0	0.25	0	0
93883	'Pcdhb12'	9.64	0.94	15.5	0.02	1.74	0.36	23.3	7.29	0	0
93884	'Pcdhb13'	0.63	0	2.22	0	0	0	4.25	0.56	0.03	0
93885	'Pcdhb14'	3.48	6.24	9.2	6.36	0.57	6.98	0	1.08	1.59	0.76
93887	'Pcdhb16'	0	0	0.27	3.21	3.53	0.17	0	0.01	0.65	0
93888	'Pcdhb17'	10.02	0	2.86	2.63	0.13	1.07	4.34	12.29	9.15	2.08
93889	'Pcdhb18'	0.33	0.25	0	0	0	0	0	0	0	0
93890	'Pcdhb19'	2.75	0	0.02	0	0	0.01	4.98	1.12	0	0.08
93891	'Pcdhb20'	2.17	0.43	0.98	0	3.4	0.04	2.74	6.8	1.71	0
93892	'Pcdhb21'	0	0	0	0	0	0	0	0.04	0	0
93893	'Pcdhb22'	1.24	0	4.84	0	0	0	0	0	0	0
93896	'Glp2r'	0	0	0	1.91	0	0	0	0	0	0
93898	'Cers1'	18.36	45.91	39.4	45	19.8	22.52	35.5	19.71	39.19	19.54
93960	'Nkd1'	0.04	0	0.01	1.74	0	0.93	1.31	0.01	1.47	0
93961	'B3galt5'	6.63	6.03	7.65	0	4.31	5.54	3.03	4.43	11.86	7.71
94040	'Clmn'	0.63	0.82	1.28	0.01	0.14	1.24	1	1.11	0.92	0.3
94041	'Allc'	0	5.7	0	0	0.67	0	0	0	0	0
94043	'Tm2d1'	54.97	99.09	92.7	158	92.2	61.13	70.5	96.85	120.7	96.82
94044	'Bcl2l13'	1.41	5.58	4.15	0.01	6.3	0.66	2.19	6.32	6.06	1.12
94045	'P2rx5'	0	0	0	5.22	3.94	1.09	1.7	0	0	0
94047	'Tmem121b'	0	1.83	1.43	0	1.71	0	0	0.01	0	0.56
94061	'Mrpl1'	10.51	12.24	11.4	14.5	10.9	3.16	10.4	15.37	14.41	15.68
94062	'Mrpl3'	32.68	30.89	47.3	26.7	29.7	49.32	32.6	48.67	13.6	14.44
94063	'Mrpl16'	38.2	27.64	32.4	22.1	35.2	32.03	22.9	22.53	31.91	20.22
94064	'Mrpl27'	217.9	130.6	158	125	251	293.8	230	270.8	159.1	168.83
94065	'Mrpl34'	78.45	81.31	76.2	83.9	41.4	113.2	131	63.1	80.93	67.29
94066	'Mrpl36'	50.4	60.12	36.6	17.6	61.7	95.2	48.1	70.85	32.7	48.55
94067	'Mrpl43'	85.45	68.58	37.9	29.2	86.5	78.05	64.3	81.56	64.37	53.63
94088	'Trim6'	0	0	0	0	0	0	0	0	0	1.92
94089	'Trim7'	0.02	0.09	0	0	0	1.15	0	0.03	1.52	0.63
94090	'Trim9'	22.06	13.19	20.3	16.4	6.28	7.63	15.2	6.62	16.46	12.2
94091	'Trim11'	14.14	0.93	7.32	14.2	4.7	15.94	6.24	10.58	19.5	10.7
94093	'Trim33'	0.6	1.25	0.12	0.86	1.93	1.09	6.06	0.93	1.79	2.4
94094	'Trim34a'	0	0.82	0	0	0	0	0	2.95	0	0.54
94109	'Csmd1'	0.23	0.98	2.79	0.29	1.44	1.45	1.82	0.44	0.39	0.78
94112	'Med15'	9.86	10.99	5.69	16.3	8.75	3.39	28.9	12.22	22.92	15.11
94176	'Dock2'	0	0.66	0.11	0	0	0	0	0	0	0.31
94178	'Mcoln1'	62.95	25.13	93.6	40.8	37.3	43.1	79.5	26.61	82.49	20.54
94180	'Acsbg1'	39.64	36.95	28.5	44	19.2	53.03	57.8	25.38	26.11	22.19
94181	'Nans'	6.41	24.86	11	7.99	6.28	31.38	20.8	28.83	37.25	18.82
94184	'Pdxdc1'	27.16	31.27	35.2	8.11	28.8	24.05	35.7	31.32	34.45	21.55
94185	'Tnfrsf21'	60.57	35.82	55.6	28.4	21.8	18.65	57.9	76.3	47.54	42.24
94186	'Strn3'	5.87	13.42	12.8	28.6	6.81	11.79	3.48	3.69	5.35	9.34
94187	'Zfp423'	5.29	9.68	0.07	0.72	2.18	5.24	10.1	2.79	1.85	7.43
94190	'Ophn1'	3.47	8.54	9.07	2.36	11	1.72	5.15	6.53	2.33	3.97
94191	'Adarb2'	19.02	2.7	4.63	0.68	0.86	1.39	3.54	0.66	1.36	1.37
94212	'Pag1'	0.05	2.28	0.07	0	0.48	0.13	0.47	0.18	0.05	0.56
94213	'Ddx50'	40.96	52.7	28.9	33.6	25.3	29.85	17.9	29.95	35.07	35.65
94214	'Spock2'	46.12	55.98	51.3	18.2	47.8	35.04	60.4	51.93	53.14	52.33
94216	'Col4a6'	0	0.01	0	0	0.49	0	0	0	0	0.07
94217	'Lrp1b'	5.08	6.56	2.49	11.5	0.86	5.63	7.22	5.4	3.61	3.92
94218	'Cnnm3'	3.84	1.2	1.36	0.12	1.01	0.01	1.65	0.99	0.21	3.81

94219	'Cnnm2'	6.18	6.48	12.1	28.9	4.78	3.79	5.95	4.77	3.59	3.71
94220	'Cnnm4'	6.2	6.46	4.82	3.17	9.86	1.57	1.53	1.73	3.61	3.31
94221	'Gopc'	2.64	1.11	3.99	11	1.73	1.44	0	2.29	0.8	2.81
94223	'Dgcr8'	9.98	7.24	4.34	3.21	8.52	1.74	11.7	4.62	14.9	2.48
94226	'S1pr5'	5.6	3.47	3.91	0	12.9	11.02	4.95	0.3	3.02	0.07
94227	'Pi15'	0.17	0.33	0.29	0.37	0.35	0.22	0.35	0.16	0.18	0.14
94229	'Slc4a10'	7.51	7.46	23.3	16.9	7.2	5.2	19.2	9.92	23.6	16.68
94230	'Cpsf1'	8.8	12.79	22	11.7	16.1	12.76	36.7	11.77	6.57	11.87
94232	'Ubqln4'	11.19	5.28	6.27	7.49	6.28	1.8	5.49	4.32	8.77	2.81
94242	'Tinagl1'	0	3.52	0	0	0	0	0	0	4.2	0
94245	'Dtnbp1'	21.13	29.87	38.4	24.4	30.1	86.23	16.7	38.48	19.4	29.48
94246	'Arid4b'	23.01	20.2	16.8	32	19.7	17.83	16	20.08	31.07	28.36
94249	'Slc24a3'	7.34	6.31	11.1	4.67	22.8	14.29	17.1	6.46	5.92	2.02
94253	'Hecw1'	2.52	0.8	2.41	5.81	2.47	3.39	1.32	4.23	2.28	1.24
94254	'Rcc1l'	8.23	16.43	7.8	10.4	13.7	12.02	11.2	5.07	9.83	2.76
94275	'Maged1'	1211	1051	893	955	608	553.1	835	1415	912.9	942.94
94279	'Sfxn2'	1.54	1.11	0.47	0.02	1.29	1.98	1.34	0.81	0.2	0.05
94280	'Sfxn3'	32.55	23	43.4	6.57	33.9	10.18	25.1	26.69	31.2	18.57
94281	'Sfxn4'	13.51	14.53	17.6	35.8	8.09	42.07	6.45	18.93	30.28	18.56
94282	'Sfxn5'	64.76	45.46	37.8	35	77.8	74.15	75.9	54.31	56.16	50.09
94284	'Ugt1a6a'	0	0	0	0	0	0	0	0.22	0	2.08
94315	'Prcc'	0.64	2.44	2.93	2.11	3.91	0.38	0.15	4.47	10.47	1.37
94332	'Cadm3'	56.75	43.82	64.1	16.1	49.3	20.23	62.1	77.46	173.7	57.75
94346	'Tmem40'	3.05	0	2.8	0.05	0	0	0	0	0	0
94352	'Loxl2'	0.8	3.68	3.82	16.1	2.62	2.2	0.77	0.92	1.15	2.99
94353	'Hmgn3'	75.05	90.61	49.5	122	112	85.23	60.7	82.03	61.08	80.9
96875	'Prg4'	0	0	0	0.03	0	0.05	0.7	0	0.14	0
96935	'Susd4'	96.5	108.1	155	55.2	52.4	99.83	71.6	117.3	100.3	131.14
96957	'Tmem62'	9.89	11.39	9.15	19.3	10.5	10.97	3.19	10.44	32.45	8.7
96979	'Ptges2'	15.16	0.15	13.7	3.42	12.8	9.59	18.3	23.55	9.14	15.12
97031	'Tprn'	9.3	5.86	4.88	1.79	5.19	10.5	20.4	0.03	0	0
97064	'Wwtr1'	0.01	0.32	0.06	0.07	0.18	0.01	0	0	0	1.76
97086	'Slc9b2'	4.06	5.19	0.72	2.65	1.6	0	0	4.67	2.57	2.06
97112	'Nmd3'	23.17	18.69	33.8	52.5	20	51.78	13.7	33.65	32.39	36.16
97114	'Hist2h3c2'	0	0	1.06	0	1.82	0.15	0.25	1.24	0	0
97122	'Hist2h4'	2.04	8.41	4.11	0	3.56	5.81	8.02	4.29	5.31	0
97130	'C77080'	0.01	1.63	0.84	0.7	0.02	0.01	3.49	1.76	0	2.71
97159	'A430005L14Rik'	15.75	51.06	32.4	29.1	31.3	77.58	46.5	60.26	50.73	42.73
97165	'Hmgb2'	0.89	22.91	12.7	32	8.69	7.53	10	6.56	3.25	0.37
97212	'Hadha'	12.53	21.11	15.1	10.6	8.38	14.8	21.3	11.1	12.71	16.16
97287	'Mtmr14'	12.69	14.34	9.39	9	7.02	0.02	5.68	10.21	15.28	7.01
97387	'Strn4'	7.55	6.68	5.12	18.9	5.69	5.08	28.6	6.54	8.52	5.74
97440	'B3gnt9'	0	1.73	4.48	0	1.81	0.76	3.52	0	3.15	6.12
97484	'Cog8'	22.59	18.47	14.6	27.5	16.8	22.17	13.1	25.41	11.35	28.41
97487	'Cmtm4'	2.89	2.19	2.99	0.38	1.66	4.05	3.98	0.42	2.18	1.16
97541	'Qars'	5.36	7.93	20.2	23.8	11.6	16.16	6.11	15.73	29.88	25.29
97761	'Sgsm2'	10.12	9.22	8.03	10.2	7.13	2.05	2.32	5.78	1.77	5.86
97820	'4833439L19Rik'	151.1	114.1	102	138	96.8	109.4	157	160.6	126.1	109.54
97827	'Exd2'	13.04	14.15	12.7	15.2	12.7	12.64	17.7	7.35	18.34	13.85
97848	'Serpinb6c'	0	0	0	0	0	0	2.35	0	0	0
97863	'Fam8a1'	9.16	6.29	3.38	14.2	4.91	4	9.35	2.79	4.49	1.16
97884	'B3galnt2'	3.26	1.23	0.82	0.48	5.58	4	1.66	4.07	1.03	0
97895	'Nlrp4f'	0.01	0.11	0.15	0	0.04	0.06	0.13	0.07	0	0
97908	'Hist1h3g'	1.5	0	0.89	0	0	0	0	1.74	3.15	0
97961	'Nol12'	20.69	25.08	21.6	39.5	27.2	33.24	25.8	26.65	14.03	41.61
97998	'Deptor'	0.86	15.93	0.02	6.02	3.02	3.29	0.04	1.11	5.44	10.15
98053	'Gtf2f1'	54.14	38.61	44.3	11.4	53.9	60.83	59.4	40.66	44.32	33.73
98170	'Tmem132a'	16.23	24.13	31.4	0	23.1	19.98	34.7	15.41	33.31	18.59

98193	'Dcaf8'	61.09	53.61	54.8	33	48.4	36.66	35.7	50.74	32.03	54.62
98221	'Eif3m'	115.2	112.6	131	79.7	113	184.1	102	108.4	106.1	119.1
98238	'Lrrc59'	6.75	11.59	10.7	15	10	8.77	9.69	11.07	12.15	4.92
98258	'Txndc9'	49.99	39.38	24.1	55.9	36.5	42.8	45.4	48.47	39.64	52.09
98267	'Stk17b'	0	1.2	0	0	0	0	1.09	2.17	0	0.76
98303	'D630023F18Rik'	7.12	26.62	7.69	13.2	2.95	9.96	16	13.96	9.67	13.33
98314	'D2hgdh'	10.7	11.33	2.82	3.8	0	9.28	4.71	4.23	5.83	6.47
98363	'Efhd1'	5.85	1.2	5.02	0	0.89	16.06	0	2.04	0	0
98365	'Slamf9'	0	1.98	0.04	0	11.9	3.54	0	0	0	0
98366	'Smap1'	15.32	2.8	2.86	11.3	14.5	13.11	5.41	5.54	8.39	14.45
98376	'Gorab'	5.01	7.14	14.6	9.87	1.52	0.21	4.11	3.85	12.24	8.43
98386	'Lbr'	0.21	7.57	2.8	0	3.93	2.77	3.42	5.89	6.59	6.39
98388	'Chst10'	22.21	4.73	13.9	17.8	4.63	0.58	6.84	16	25.53	2.88
98396	'Slc41a1'	4.84	2	11.6	2.35	6.83	0	5.26	3.48	4	5.84
98402	'Sh3bp4'	1.36	0.04	6.29	8	3.66	0.02	12.3	1.59	0.97	0
98403	'Zfp451'	9.91	1.8	6.1	27.9	25.7	5.94	8	14.62	11.59	14.19
98404	'AI597479'	15.42	11.78	11.4	5.77	27	5.82	33.7	15.14	16.62	28.15
98415	'Nucks1'	5.83	7.13	8.33	12.2	8.15	3.72	7.43	6.39	4.15	5.47
98417	'Cnih4'	4.56	13.64	9.02	8.23	22.2	2.98	2.24	8.92	7.6	8.3
98432	'Phlpp1'	1.04	2.64	0.67	5.75	1.7	0.01	0.17	1.03	1.56	0.88
98488	'Gtf3c3'	11.41	5.58	13.7	7.63	6.88	11.32	5.25	10.12	18.89	6.24
98496	'Pid1'	30.94	16.41	28.1	4.68	16.4	11.42	39.4	35.85	36.77	45.62
98558	'Mael'	0	0	0	0	0.03	0	0	0	0	0
98660	'Atp1a2'	20.84	28.8	15.1	25.8	15.5	20.71	18.1	20.83	23.97	12.02
98682	'Mfsd6'	8.46	4.34	3.02	13.1	11.5	6.51	10.9	2.92	10.08	2.56
98685	'Trmt1l'	22.81	22.35	14.6	14.3	9.39	7.72	21.4	13.99	10.11	13.59
98710	'Rabif'	28.54	37.12	46	20.9	23.5	44.1	33.6	37.82	52.66	27.83
98711	'Rdh10'	0.08	3.32	3.62	1.19	3.23	0	0	1.18	0	0.11
98732	'Rab3gap2'	5.65	8.29	17.2	12.2	12	6.18	17.2	6.12	15.46	6.36
98733	'Obsl1'	2.16	4.65	1.97	0.62	5.75	2.71	8.24	1.45	5.02	1.67
98741	'Kcnb2'	1.93	2.18	2.46	7.25	1.54	0.01	2.98	0.87	0.73	5.05
98758	'Hnrnpf'	48.82	34.3	26.9	16.4	25.1	45.75	47.6	37.17	35.19	38.33
98766	'Ubac1'	15.12	17.58	7.7	16.1	13.7	36	1.06	11.42	27.9	9.87
98828	'Cdc123'	100.1	139.7	165	200	175	310.9	96.6	190.7	139.8	176.49
98845	'Eps8l2'	0	0.14	0	0	8.79	5.26	0	0	4.25	9.2
98870	'AI182371'	0	0	0	0	0	0	0	0	0	0.06
98878	'Ehd4'	16.45	14.24	12.3	16.2	14.9	27.83	9.61	3.38	18.34	7.04
98910	'Usp6nl'	1.27	0.31	0.59	2.11	0.37	0.08	0.14	0.71	1.21	0.83
98932	'Myl9'	0	3.66	5.13	0	6.65	10.91	0	4.55	7.33	2.24
98952	'Fam102a'	3.77	3.9	2.82	8.35	1.68	3.62	8.99	9.19	2.26	2.04
98956	'Nat10'	2.13	1.55	10.4	0	3.12	3.39	9.69	1.82	8.72	6.94
98970	'Fibcd1'	0.42	1.56	1.85	1.51	0.37	11.32	0.01	0.35	4.58	5.78
98985	'Clp1'	2.85	14.72	10.9	33.1	4.14	3.39	0	1.36	4.52	3.44
98999	'Znfx1'	0.43	0.18	0	10.1	0.34	4.52	0	0.57	0.55	2.92
99003	'Qser1'	0.91	0	0.01	0	0.16	0.51	0	0.29	1.85	0.04
99010	'Lpcat4'	18.92	9.88	18.8	17.6	12.8	3.32	3.06	5.05	23.78	15.76
99011	'Pomt1'	4.36	13.25	5.72	12.5	5.8	10.81	14.1	9.07	9.29	13.53
99031	'Osbpl6'	17.76	11.25	11	10.2	13.9	8.28	9.52	11.3	19.02	8.82
99035	'Olah'	0	0	0	0	1.82	0	0	0	3.11	0
99045	'Mrps26'	155	125.1	147	81.9	174	126.1	161	187.6	88.26	102.34
99100	'Cep152'	0	1.3	0	0.35	1.52	2.13	0.9	0.81	0.22	0.01
99138	'Stard7'	31.11	32.19	19.3	34	25.9	32.34	47.8	39.73	44.42	24.48
99151	'Cercam'	3.33	2.11	4.21	9.11	9.32	11.81	7.98	5.02	0.25	3.24
99152	'Anapc2'	54.33	55.54	67.1	45.5	64.2	36.43	85.2	42.22	93.35	44.31
99167	'Ssx2ip'	8.49	8.04	7.96	4.26	9.97	8.62	5.09	13.35	4.57	10.1
99237	'Tm9sf4'	27.71	47.37	48.8	13.4	29.6	27.87	57.7	48.17	40.24	57.05
99296	'Hrh3'	9.91	4.43	15.8	8.58	21.6	12.38	4.92	10.14	4.26	2.95
99311	'Commd7'	34.93	44.04	41.9	48	31.9	62.12	25	36.63	55.91	55.93

99326	'Garnl3'	16.18	11.47	22.1	26.8	15.4	5.9	44.6	21.67	17.4	8.09
99334	'Zscan29'	0.11	0.08	2.11	0	2.66	2.62	0	0.22	0.5	4.02
99349	'Dnajc24'	13.71	12.28	13.8	27.7	9.26	6.54	7.7	14.45	2.39	7.01
99371	'Arfgef2'	0.26	0.01	2.05	1.7	1.57	0.03	4.75	1.09	1.38	2.11
99375	'Cul4a'	15.07	14.03	15.3	20.6	26.3	8.22	16.7	17.38	13.22	12.7
99377	'Sall4'	0	0	0	0	0	0	0.02	0	0	0
99382	'Abtb2'	2.97	1.19	3.95	3.34	0.01	0	0	0.01	0.01	0
99412	'Golga2'	7.29	12.41	7.06	11.6	15.3	4.9	17.1	9.96	8.03	16.41
99439	'Duox1'	0.01	0	0	0	0	0	0	0	0	0
99470	'Magi3'	3.8	1.49	1.03	1.03	2.9	0.01	1.12	2.61	0.01	0.9
99480	'Dnttip2'	26.84	27.46	18.2	28.4	29.5	17.96	35	28.4	18.79	27.24
99512	'Wdr47'	31.25	25	38.1	26.3	16.3	11.58	43.7	35.1	51.71	29.73
99526	'Usp53'	2.16	1.69	2.78	0	1.79	1.21	0.6	0.77	2.79	0.31
99543	'Olfml3'	3.63	2.56	4.24	0.03	6.05	48.19	22.5	6.3	0.27	0
99586	'Dpyd'	1.93	2.92	2.2	10.5	0.85	4.54	1.71	4.59	4.05	4.76
99633	'Adgrl2'	5.14	19.55	13.6	3.34	10	2.27	3.49	6.25	1.57	10.14
99650	'4933434E20Rik'	91.31	77.9	43.5	100	114	93.04	67.4	95.91	101.7	108.34
99662	'Eps8l3'	1.63	1.64	3.12	1.6	2.77	1.78	4.29	1.46	2.25	0.11
99663	'Clca4a'	0	0.11	0	0	0	0	0	0	0	0
99681	'Tchh'	0.63	4.42	2.48	0	0.8	3.42	3.45	1.52	1.51	0.94
99683	'Sec24b'	3.44	6.96	6.3	13.4	3.01	9.03	4.45	4.86	9.99	10.38
99696	'Ankrd50'	1.73	1.35	1.2	2.08	1.13	1.18	1.3	1.13	0.74	2.28
99712	'Cept1'	54.5	65.83	34.9	9.17	35.2	60.48	31.1	48.86	50.9	82.11
99730	'Taf13'	11.18	21.48	9.63	37.3	38.3	51.07	11.9	21.28	28.08	16.7
99738	'Kcnc4'	20.45	2.66	12.3	0.02	4.42	13.56	16.7	11.04	19.4	1.76
99887	'Tmem56'	2.74	2.72	1.34	4.59	1.6	3.54	2.72	1.65	1.11	1.75
99889	'Arfip1'	7.98	8.05	4.39	3.14	12	4.51	5.57	3.43	6.38	4.27
99890	'Prmt6'	1.12	5.95	0.7	3.55	0.44	0	0	0	5.41	4.09
99929	'Tiparp'	7.87	21.87	3.51	49	1.35	4.06	5.84	7.51	6.24	19.53
99982	'Kdm1a'	1.54	6.8	4.79	4.62	2.36	23.65	2.69	4.5	6.07	2.81

Supplementary Table 2 Differentially expressed genes (in FPKM) and their test statistical P value of ARC and PVH-projecting apMPOA neurons from RNA-seq analysis. Values from each biological replicate are all included.ARC-projecting apMPOA replicates: A5, A6, A8, A10, A12; PVH-projecting apMPOA replicates: P11, P12, P15, P16, P17

Gene ID	Gene Symbol	A_10	A_12	A_5	A_6	A_8	P_11	P_12	P_15	P_16	P_17	Pvalue(P/A)
100040048	'Ccl27b'	0	0	0	5.43	0	12.44	0	2.3	7.92	13.7	0.0894044
100041546	'Ly6c2'	0	0	0	0	0	0	0	0	5.51	0	1.04E-11
100042514	'Sprr2a3'	0	0	0	0	0	0	0	0	6	0	6.97E-12
100042784	'Prdm11'	0	0	1.94	0	0	0	0	0	0	0	2.44E-12
100043757	'Zfp831'	0.38	0	2.37	0	0	0	0	0.01	0.01	0	0.0040358
100043861	'Klrb1'	0	2.95	0	0	0	0	0	0	0	0	0.0359111
100045778	'Rnf223'	0	0	1.14	0	0	0	0	0	0	0	6.10E-13
100503545	'Nuggc'	0	0	0	0	0	0	4.8	0	0	0	1.55E-12
100504180	'LOC100504180'	0	0	0	0	0	0.78	0	0	0	0	1.53E-11
100504234	'Ccdc170'	0	0	0	0	1.68	0	0	0	0	0	8.01E-13
100861647	'Gm21104'	0	0	0	0	0	0	0	0	9.99	0	4.28E-12
101056205	'Gm29797'	0	0	0	0	0	2.26	0	0	0	0	8.86E-13
102638268	'1700014D04Rik'	0	0	0	0	0	0	0	0	0	1.68	1.64E-12
102640920	'Pvrig'	0	0	0	0	0	0	0	0	2.23	0	1.14E-11
104443	'Npffr2'	0	0	0	0	0	0	0	2.1	0	0	1.05E-11
104709	'Pik3r6'	0	0	0	0	1.59	0	0	0	0	0	5.76E-13
104759	'Pld4'	0	0	0	0	0.05	13.97	12.9	0	5.17	0	2.14E-05
105243944	'Gm39653'	0	0	0	0	0	0	0	3.56	0	0	5.63E-12
105247125	'105247125'	0	0	0	0	0	0	1.54	0	0	0	2.42E-12
105732	'Fam83h'	0	0	0	0	1.04	0	0	0	0	0	7.72E-13
105855	'Nckap1l'	0	0	0	0	1.33	0	0	0	0	0	2.80E-13
106757	'Catsperd'	0	0	0	0	0	2.61	5.09	1.64	0.1	0.37	6.13E-07
107321	'Lpxn'	0	0	0	0	0	12.78	4.83	0	0	0	1.13E-14
107527	'Il1rl2'	0	0	0	0	0	2.04	0	0	0	0	8.90E-12
107751	'Prrxl1'	0	0	2.09	0	0	0	0	0	0	0	3.98E-13
107995	'Cdc20'	0.02	0	0	0	3.97	0	0	0	0	0	2.00E-13
108012	'Ap1s2'	13.1	46.54	38.42	48	78	77.77	168	115	115	183	5.04E-05
108043	'Chrnb3'	1.29	0	0	0	0	0	0	0	0	0	3.85E-13
108167560	'Gm46058'	0	0	2.8	2.83	0	0	0	0	0	0	0.0117877
108168756	'Gm46741'	0	0	0	0.02	0	0	11.9	0	0.02	0	0.0022382
108802	'Calr4'	2.24	0	0	0	0	0	0	0	0	0	7.80E-13
108897	'Aif1l'	1.08	0.98	3.66	0	0	0	0	0	0	0	0.0024934
108912	'Cdca2'	0	0	0	0	0	0	0	1.26	0	0	9.75E-12
108995	'Tbc1d10c'	0	0	0	5.73	0	0	0	0	6.26	0	0.9538081
110454	'Ly6a'	0	0	0	0	0	5.54	0	0	0	0	4.44E-12
114230	'Aipl1'	0	0	0	0	0	0	0	0	0	2.06	1.87E-11
114249	'Npnt'	0	0	0.01	0	0	0	2.74	0.01	6.95	0.92	4.34E-06
11471	'Actl7b'	0	3.43	0	0	0	0	0	0	0	0	7.63E-13
11541	'Adora2b'	0	2.17	0	0	0	0	0	0	0	0	1.40E-12
11552	'Adra2b'	0	0	0	0	0	0	0	0	2.12	0	6.84E-11
11576	'Afp'	0	0	0	0	0	2.54	0	0	0	0	3.86E-12
11609	'Agtr2'	0	0	5.05	0	0	0	0	0	0	0	1.85E-14
11614	'Nr0b1'	0	0	0	0	0	0	0	0	0	3.1	3.81E-12
11630	'Crybg1'	0	0	0	0	0	0	1.28	0	0	0	6.29E-13
11658	'Alcam'	43.9	35.14	24.96	7.53	38	50.39	131	43.3	148	159	4.62E-05
116872	'Serpinb7'	0	0	0	0	0	0	0	1.83	0	0	1.71E-11
11810	'Apobec1'	0	0	0	0	0	0	0	2.63	3.76	7.61	2.35E-04
11830	'Aqp5'	0	0	2.41	0.04	0	0	0	0	0	0	0.0326133
118453	'Mmp28'	0	0	0	0	1.13	0	0	0	0	0	1.41E-12
11997	'Akr1b7'	0	0	4.21	0	0	0	0	0	0	0	6.72E-13
12029	'Bcl6b'	0	0	2.63	0	0	0	0	0	0	0	9.56E-14
12062	'Bdkrb2'	0	0	0	0	0	0	0	0	0	1.46	3.91E-12
12142	'Prdm1'	0	0	1.49	0	0	0	0	0	0	0	1.60E-13
12145	'Cxcr5'	0	0	0	0	0	0	0	0	3.74	0	2.11E-11

12159	'Bmp4'	0	0	8.41	0	0	0	0	0	0	0	7.94E-14
12164	'Bmp8b'	0	0	0	0	0	0	0	0	1.99	2.5	2.10E-13
12268	'C4b'	0	0	3.43	5.43	24.3	0	0.01	0	0.01	0.01	5.11E-06
12310	'Calca'	0	0	0	0	0	20.55	0	0	0	11.3	9.47E-15
12316	'Aspm'	0	0	0	0.02	0	0	1.78	0	0	0	0.0268953
12490	'Cd34'	0	0	0	75.8	0	4.04	4.97	2.73	0	0	0.3663714
12493	'Cd37'	0	0	0	0	0	25.75	0	0	0	4.31	4.07E-15
12508	'Cd53'	0	0	0.05	0	0	10.14	3.49	0.55	0	2.53	9.08E-06
12532	'Cdc25c'	0	0	0	0	0	5.2	0.03	0	0	0	4.36E-13
12566	'Cdk2'	0	0	0	0	0	0	4.69	0	0	0	4.43E-13
12609	'Cebpd'	0.65	1.56	1.47	8.72	1.09	0	0	0	0	0	5.42E-09
12677	'Vsx2'	0	0	0	0	2.71	0	0	0	0	0	1.01E-13
12767	'Cxcr4'	0	0	0	0	2.41	0	0	0	0	0	1.21E-12
12922	Crhr2'	96	120.6	86.78	50	0	0	0	0	0	0	6.53E-15
12931	'Crlf1'	0	0	3.05	0	0	0	0	0	0	0	7.15E-13
12959	'Cryba4'	0	0	4.2	0	0	0	0	0	0	0	0.0391199
13013	'Cst9'	0	0	0	0	0	0	15.4	0	0	0	1.96E-11
13089	'Cyp2b13'	0	0	0	0	4.31	0	0	0	0	0	2.45E-13
13179	'Dcn'	0	0	0	0	3.04	0	0	0	0	0	4.11E-13
13195	'Ddc'	118	81.17	29.82	94.9	31.2	12.03	4.26	12.8	16.3	7.03	8.78E-09
13214	'Defb1'	0	0	0	0	0	0	35.1	0	0	0	4.08E-14
13490	Drd3'	36.3	0	64.02	0	40.5	190.2	0	114	154	267	4.93E-09
13605	'Ect2'	0	0	0	0	0	0	0	1.46	0	0	2.84E-12
13608	'Edar'	2.35	0	0	1.22	0	0	0	0	0	0	2.55E-14
13639	'Efna4'	0	0	1.92	0	0	0	0	0	0	0	0.042011
13797	'Emx2'	0	0	0	0	0	3.04	0	0	11.9	3.61	3.37E-05
13806	'Eno1'	1030	928.3	999.8	851	876	1503	1320	1291	1390	1216	5.20E-07
13846	'Ephb4'	0	0	0	1.51	0	0	0	0	1.5	0	0.9989031
13861	'Epx'	0	2.06	0	0	0	0	0	0	0	0	1.74E-12
13866	'Erbb2'	0	0	0	0	0	0	0.01	0.05	2.4	0	2.86E-13
14063	'F2rl1'	0	0	1.4	0	0	0	0	0	0	0	1.80E-12
140806	'Il25'	0	0	0	0	0	0	0	0	0	3.06	1.87E-11
14089	'Fap'	0	0	0	0	0	0	2.91	0	0	0	1.50E-12
14107	'Fat1'	0.88	1.16	0.76	0.29	0.48	0	0	0.02	0.01	0.11	3.80E-05
14119	'Fbn2'	2.16	1.41	1.14	0	1.38	0	0.01	0.03	0.14	0	0.0014945
14129	'Fcgr1'	0	0	0	0	0	6.74	0	1.46	0	0.74	8.47E-04
14233	'Foxi1'	0	0	0	0	0	1.64	0	0	0	0	1.06E-11
14254	'Flt1'	0	0	0	0	0.56	0	0	0	0	0	0.0327819
14264	'Fmod'	0	0	0	0	0	0	1.93	0	0	0	4.07E-12
142681	'Slc34a3'	0	0	0	0	1.97	0	0	0	0	0	1.36E-12
14427	Galr1'	8.2	18.8	23.8	0	0	75.9	45.5	51.7	49.6	60.3	1.833E-05
14555	'Gpd1'	0.45	0.51	6.8	10.1	0.19	0.03	0	0.09	0.15	0.02	4.36E-06
14587	'Gfra3'	1.94	0	0	0	0	0	0	0	0	0	1.62E-12
14872	'Gstt2'	9.08	12.35	6.78	16.2	5.88	39.35	28.7	9.83	19.1	36.5	0.0022469
14961	'H2-Ab1'	0	0	0	0	0	9.44	0	0	0	0	3.56E-13
14964	'H2-D1'	70	41.7	82.19	133	64.5	223.8	83.5	34.4	340	363	0.0044216
15000	'H2-DMb2'	0	0	10.72	0	0	0	0	0	0	0	1.57E-13
15214	'Hey2'	0	0	0	0	0	0	0	0	4.16	0	8.89E-13
15450	'Lipc'	0	0	0	0	3	0	0	0	0	0	5.58E-13
15485	'Hsd17b1'	0	0	0	0	0	0	0	0	1.63	0	2.21E-11
15486	'Hsd17b2'	0	0	0	0	0	0	0	4.32	0	0	3.90E-12
15557	'Htr1f'	54.4	33.2	0	74	0	0	0	0	0	0	4.99E-15
15894	'Icam1'	0	0	0	0	0	6.16	0	0	0	0	9.74E-14
15945	'Cxcl10'	0	0	0.04	13.3	5.7	0	0	0	0	0	0.0027536
16145	'Igtp'	2.59	0	0.15	31.3	4.14	0	0.02	0	0	0	1.23E-07
16172	'Il17ra'	0	0	0	0	0	1.51	0.01	0	5.92	4.38	3.68E-06
16177	'Il1r1'	0.92	0	0	0	0	0	0	0	0	0	2.42E-12
16398	'Itga2'	0	0	0.85	0	0	0	0	0	0	0	2.38E-12
16414	'Itgb2'	0	0	0	0	0	5.89	0	0	0	0.08	6.94E-14
16416	'Itgb3'	0	0	1.16	0	0	0	0	0	0	0	2.58E-13
16419	Itgb5'	20	5.2	10.09	0	30.8	58.03	80.8	101	50	40	2.07E-04

16470	'Ush1g'	1.04	0	0	0	0	0	0	0	0	0	2.80E-12
16499	'Kcnab3'	4.72	0.25	5.41	0	15.7	0.23	0.04	0	0.24	0.17	3.07E-04
16518	'Kcnj2'	1	0	0	0	0	0	0	0	0	0	4.79E-13
16542	'Kdr'	0	0	0	0	0	0	0	0	1.05	0	3.74E-12
16612	'Klk1'	4.48	0	0	0	0	0	0	0	0	0	1.56E-12
16675	'Krt27'	0	0	0	0	10.3	0	0	0	0	0	1.19E-14
16681	'Krt2'	0	0	0	0	0	0	3.27	0	0	0	9.46E-13
16768	'Lag3'	0.77	0.67	2.16	0	6.38	0	0	0.02	0	0	4.50E-05
16775	'Lama4'	0	0	0.75	0.01	0	0	0	0	0	0	0.0250248
170676	'Peg10'	2.95	1.72	1.58	0.07	0.2	2.24	7.16	3.79	10.6	13.9	0.0017794
170732	'Trhr2'	0	0	0	0	0	0	0	2.34	0	0	1.46E-12
170735	'Arr3'	0	0	0	0	0	0	0	0	0	2.64	6.67E-12
170741	'Pilrb1'	0	0	0	0	6.39	0	0	0	0	0	2.53E-13
17075	'Epcam'	0	0	2.03	0	22.2	0	0	0	0	0.04	8.30E-04
171207	'Arhgap4'	0	0.14	0.11	0	0	5.12	4.75	1.51	0.87	2.38	3.15E-06
17121	'Mxd3'	0	0	0	0	5.16	0	0	0	0	0	2.53E-13
171285	'Havcr2'	0	0	0	0	0	8.3	0	0	4.65	0	5.69E-15
17286	'Meox2'	5.59	0	0	0	0	0	0	0	0	0	2.89E-14
17288	'Mep1b'	0	0	0	0	0	0	0	0	0	1.8	1.00E-11
17773	'Mtnr1a'	0	0	0	0	0	0	0	2.2	0	0	1.63E-11
17831	'Muc2'	0	0	0	0	0	0	0	0	0	0.57	1.10E-11
17880	'Myh11'	0	0.67	0	0	0	0	0	0	0	0	0.0353146
17882	'Myh2'	0	0	1.01	5.32	0	0	0	0	0	0	5.52E-16
17883	'Myh3'	0	0	0	0.1	0	0	0	0	0	0.59	0.3699065
17910	'Myo15'	0.27	0.17	1.03	0	0	0	0	0	0	0	0.0016451
17916	'Myo1f'	0	0	0	0	0	3.36	0	0	0	0	1.93E-13
17926	'Myoc'	2.8	0	0	0	0	0	0	0	0	0	4.24E-13
17927	'Myod1'	0	1.87	0	0	0	0	0	0	0	0	0.0347349
17937	'Nab2'	0.61	1.21	1.69	3.33	8.54	0.1	0.02	0.03	0.07	0	3.06E-08
17996	'Neb'	0.49	0.79	0	0.01	0.01	0	0	0	0	0	8.17E-06
18119	'Nodal'	0	0	0	5.58	0	11.29	0	0	11	0.1	0.448316
18133	'Nov'	0	0	0	0	0	0.5	4.62	0.1	0.51	2.87	2.27E-06
18166	Npy1r'	110	43	41.6	0	5.6	71.9	296	255	40.7	177	0.0435343
18168	'Npy5r'	9.9	0	22.1	0	19.7	40.5	105	86.8	26.8	28.6	0.0225195
18419	'Otog'	0	0	0.62	0	0	0	0	0	0	0	3.48E-13
18442	'P2ry2'	0	0	0	0	0	0	0.02	0	2.07	0	2.84E-12
18519	'Kat2b'	1.06	0.62	0.42	2.33	1.65	0.02	0	0.11	0	0.01	2.18E-05
18613	'Pecam1'	0	0	0	4.53	0	1.95	0	0	0	0	0.7130513
18700	'Piga'	0	2.26	2.16	0	5.27	0	0	0	0	0	1.22E-04
18772	'Pkp1'	0	0	1.79	0	0	0	0	0	0	0	1.25E-12
18784	'Pla2g5'	0	0	0	0	4.29	0	0	0	0	0	1.39E-13
18933	'Prrx1'	0	0.01	0	0	0	0.97	0.95	0.91	0.38	0.01	1.19E-05
19059	'Ppp3r2'	2.04	0	0	0	0	0	0	0	0	0	3.50E-13
19126	'Prom1'	2.08	0	0	0	0	0	0	0	0	0	1.80E-13
19130	'Prox1'	2.1	1.32	2.3	3.45	0	0.07	0.01	0	0	0.01	2.79E-05
19144	'Klk6'	0	1.45	0	0	17.5	0	0	0	0	0	2.44E-15
19216	'Ptger1'	0	0	0	0	0	1.8	0	0	0	0	1.30E-11
19220	'Ptgfr'	19.8	0	0	0	40.4	70.4	31.8	0	21	55.4	0.0171128
19256	'Ptpn20'	0	0	0	0	0	0.79	0	0	0	3.52	5.12E-13
19260	'Ptpn22'	0	0	0	0.02	0	0	5.59	0	0	0	0.0062989
19285	'Cavin1'	1.34	3.1	0	4.39	0.74	0	0	0	0.01	0	2.49E-06
19289	'Igdcc3'	1.97	0	0	0	0	0	0	0	0	0	3.29E-13
19331	'Rab19'	0	0	0	0	0	0	0	0	0	6.29	1.06E-12
19383	'Raly'	21.4	24.41	32.4	41.9	25.5	53.33	48.1	51	44.5	46.5	1.90E-04
19661	'Rbp3'	0	0	0	0	0	0	0	2.98	0	0.01	9.68E-14
19707	'Reps1'	1.67	3.3	4.75	4.65	2.32	9.99	15.5	7.36	11.2	4.26	9.38E-04
19727	'Rfxank'	1.04	0.79	5.74	6.72	2.21	11.73	10.9	6.88	7.53	9.7	0.0098868
19733	'Rgn'	3.26	0	0	0	0	0	0	0	0	0	6.73E-13
19737	'Rgs5'	0	0	0	0	0	0	0.01	0	0	4.26	7.22E-14
19752	'Rnase1'	0	0	0	0	0	13.17	0	0	0	0	2.27E-13
19824	'Trim10'	0	0	1.84	0	0	0	0	0	0	0	1.38E-12

20197	'S100a3'	0	0	1.79	0	0	0	0	0	0	0	0.0412546
20276	'Scnn1a'	0	0	0	7.07	0	0	0.04	1.28	3.71	2.24	0.9776893
20277	'Scnn1b'	0	6.09	0	0	0	0	0	0	0	0	1.10E-14
20299	'Ccl22'	5.89	0	0	0	0	0	0	0	0	0	5.54E-14
20495	'Slc12a1'	0	0	0	0	0	0	0.89	0	0	0	1.01E-11
20526	'Slc2a2'	0	0	0	0.02	0	0	2.32	0	0	0	0.0242788
20657	'Sod3'	2.89	1.88	2.17	0	0	0	0	0	0	0	0.004283
20669	'Sox14'	0	0	0	0	0	0	0	3.09	0	0.02	2.22E-12
20700	'Serpina1a'	2.66	0	0	0	0	0	0	0	0	0	9.14E-13
20703	'Serpina1d'	4.49	0	0	0	0	0	0	0	0	0	7.80E-13
207742	'Rnf43'	0	0	1.19	0	0	0	0	0	0	0	6.52E-13
208595	'Mterf1b'	0	0	0	0	0	11.24	0	0	2.52	0	2.90E-13
208760	'Aqp12'	0	0	3.28	0	0	0	0	0	0	0	0.0359563
209707	'Lcorl'	3.54	7.87	5.36	4.72	4.55	1.44	1.58	2	2.23	2.34	1.00E-04
211488	'Ado'	14.6	11.43	9	8.59	11.9	4.31	4.67	4.3	4.1	1.74	1.83E-05
212541	'Rho'	0	1.37	0	0	0	0	0	0	0	0	8.39E-13
212989	'Best2'	0	0	0	0	0	0	0	0.02	0	2.76	4.84E-12
21337	'Tacr2'	0	0	0	0	0	0	0	0	2.82	0	2.80E-12
213409	'Lemd1'	0.52	0	0	0.3	2.23	15.08	6.08	2.99	25.9	11.1	2.99E-04
213527	'Pth2r'	0	1.5	3.22	5.24	8.12	0	0	0	0	0	2.28E-07
213696	'Duoxa1'	0	0	3.8	0	0	0	0	0	0	0	5.82E-13
214058	'Megf11'	6.8	3.2	30.17	2.55	12.5	4.41	2.17	4.1	0.97	2.04	0.0035413
214111	'Slc24a1'	0.25	1.13	1.25	2.16	1.58	0	0	0	0.01	0	6.05E-11
214359	'Tmem51'	4.86	2.57	0.03	10.2	4.35	0	0	0	0	0	1.41E-08
214425	'Cilp'	0	0	0	0	0	0	0	0	0	1.69	1.64E-12
215632	'Psd4'	0	0	0	0	2.17	0	0	0	0	0	1.04E-13
216049	'Zfp365'	21	17.2	11.25	9.18	29	8.23	9.44	7.07	11.9	8.89	0.0094139
216225	'Slc5a8'	0	0	0.73	0	0	0	0	0	0	0	1.64E-12
217216	'BC030867'	0	0	3.6	0	0	0	0	0	0	0	9.95E-13
217262	'Abca9'	0.84	0	0	0	0	0	0	0	0	0	6.93E-13
217304	'Cd300lb'	0	0	0	0	0	0	0	0	0	2.4	1.76E-12
217333	'Trim47'	0	0	0	4.1	1.48	0	0	0	0	0	3.47E-14
217369	'Uts2r'	40.2	77.2	11.5	0	59.3	216.1	195	134	168	154	5.31E-04
21815	'Tgif1'	0	0	0	0	0	5.03	0	3.95	0	0	1.05E-13
21824	'Thbd'	0	0	0	0	0	3.82	0	0	0.4	0	9.33E-14
21828	'Thbs4'	18.6	0	0	2.95	0	0	0	0	0	0	5.47E-19
218630	'Ccno'	0	0	1.6	0	0	0	0	0	0	0	0.0427915
218877	'Sema3g'	0	0	0	0	0	0	0	0	3.07	0	2.91E-13
219103	'Cenpj'	0.55	1.57	0.99	0	0.97	0	0	0.01	0	0	4.31E-05
22178	'Tyrp1'	0	0	0	0	0	0	0	0	0	4.12	3.35E-13
22242	'Umod'	0	0	0	0	0	0	0	0	6.3	0	1.14E-13
22262	'Uox'	2.95	0	0	0	0	0	0	0	0	0	5.74E-13
22269	'Upk2'	3.55	0	0	0	0	0	0	0	0	0	0.0425297
22359	'Vldlr'	6.15	12.78	13.18	7.09	8.79	3.59	3.76	5.36	4.96	3.98	9.19E-05
22361	'Vnn1'	0	0	2.58	0	0	0	0	0	0	0	5.93E-13
223915	'Krt73'	1.04	2.09	2.57	9.38	0.02	0	0.02	0	0	0	2.19E-07
224694	'Zfp81'	3.3	6.43	6.97	3.13	8.22	0.61	0.02	1.28	1.2	0.09	7.95E-04
225471	'Ticam2'	0	0	0	0	0	0	0	1.66	0	0	3.75E-12
226421	'Rab7b'	0	0	0	0	1.56	0	0	0	0	0	2.03E-13
226564	'Fmo4'	0	0	0	0	2.02	0	0	0	0	0	1.66E-12
227231	'Cps1'	0	0	0	0	0	2	0	0	0	0.02	2.98E-13
227358	'Erfe'	0	0	0	0	0	0	0	0	4.65	0	2.40E-13
228993	'Slc17a9'	0	0	0	0	0	3.64	0	0	0	0	9.56E-13
229003	'Helz2'	0	0	0	0	0	0	0	0	0	0.84	1.00E-10
229055	'Zbtb10'	0.2	0.21	0.76	2.36	0.03	0	0.01	0.01	0.02	0.01	1.94E-06
229302	'Tm4sf4'	2.31	0	0	0	0	0	0	0	0	0	0.0391328
229499	'Fcrl1'	0	0	0	0	0	6.99	0	0	0	0	1.61E-13
230579	'Fam151a'	0	3.58	0	0	0	0	0	0	0	0	2.45E-13
230824	'Grhl3'	2.73	0	0	4.35	0	0	0	0	0	0	3.68E-13
232345	'A2m'	0	0	0	0	1.31	0	0	0	0	0	3.27E-13
232406	'BC035044'	0	0	0	0	0	7.48	0	0	2.95	0.02	1.92E-16

232431	'Gprc5a'	0	0	0	0	5.94	0	0	0	0	0	5.20E-14
232493	'Gys2'	0	0	1.11	0	0	0	0	0	0	0	0.0427915
232974	'Gm4881'	0	0	0	0	0	0	19.2	0	0	0	1.03E-14
234582	'Ccdc102a'	2.36	0	0	0	0	0	0	0	0	0	5.55E-13
234724	'Tat'	0	1.59	0	0	0	0	0	0	0	0	1.74E-12
235135	'Tmem45b'	0	0	5.4	0	20.5	0	0	0	0.04	0	3.41E-04
235415	'Cplx3'	0	0	5.3	0	0.02	0	0	0	0	0	1.58E-14
235587	'Parp3'	0	0	0	6.43	0	3.41	2.57	1.06	0.02	3.1	0.7474219
236573	'Gbp9'	0	0	0	0	3.16	0	0	0	0	0	3.65E-14
237523	'Ptprq'	0.81	0	0	0	0	0	0	0	0	0	7.12E-13
23796	'Aplnr'	21.2	11.4	30.5	25.2	13.1	151.1	113	253	102	233	3.364E-08
23855	'Defa17'	0	0	0	0	0	0	34.3	0	0	0	5.74E-12
238663	'Spata31d1d'	0	0	0	0	0	1.64	0	0	0	0	2.06E-12
23923	'Aadat'	0	0	0	0	0	0	0	0	0	5.37	5.05E-13
239845	'Gpr156'	0	0	1.24	0	0	0	0	0	0	0	1.18E-12
240754	'Lax1'	1.29	0	0	0	0	0	0	0	0	0	0.0425297
24088	'Tlr2'	0	0	0	0	0	2.98	0	0	0	1.92	1.49E-13
24102	'Trex2'	0	3.33	0	0	0	0	0	0	0	0	0.0371568
241327	'Olfml2a'	0	0	0	0	0	0	0	0	2.94	0	1.95E-12
241633	'Atp8b4'	0	0.89	0	0	0	0	0	0	0	0	6.70E-13
242505	'Rasef'	0	0	0	0	0	0.6	0.88	0.01	0.31	0.46	6.86E-05
242646	'Tctex1d4'	0	0	0	0	0	0	0	7.89	0	0	1.21E-12
242702	'Myom3'	0.76	0	0	0	0	0	0	0	0	0	0.0397741
243653	'Clec1a'	0	0	2.13	0	0	0	0	0	0	0	2.58E-13
243911	'Kirrel2'	0	1.57	0	12.7	0	0	0	0	0	0	1.80E-15
244548	'Elmod2'	2.02	0.9	4.21	3.63	0.98	3.72	7.22	3.09	9.99	7.69	0.0017617
244813	'Bsx'	0	0	0	0	5.48	0	0	0	0	0	1.77E-13
245026	'Col6a6'	0	0	0	0	0	0	0	0	1.81	0.45	1.10E-13
245424	'Gpr101'	20.9	7.67	13.19	0	14.5	21.75	35.6	44.2	156	88.1	0.0296404
245532	'Awat2'	0	0	2.72	0	0	0	0	0	0	0	9.95E-13
245671	'Klf8'	0	0.03	0.02	0.02	0	0.35	0.02	1.57	5.11	1.22	2.06E-06
245695	'Tceanc'	2.2	1.06	0.97	0.17	0.57	0	0.01	0.08	0.08	0.03	2.01E-04
245877	'Map7d1'	22.9	22.73	16.77	31.4	27.2	9.72	11.5	12.2	16.6	11.2	8.20E-05
257947	'Olfr543'	0	0	0	0	0	0	0	0	3.33	0	1.75E-11
258470	'Olfr91'	0	0	0	4.75	0	4.22	0	0	0	0	0.9876207
258745	'Olfr689'	0	0	0	0	3.24	0	0	0	0	0	0.0404979
258938	'Olfr1417'	0	0	0	0	0	0	6.81	0	0	0	2.84E-12
259051	'Olfr658'	0	0	0	0	0	3.1	0	0	0	0.06	5.84E-13
260408	'Prss45'	0	0	0	0	0	0	0	0	2.96	0	1.90E-11
26366	'Ceacam10'	0	0	0	0	0	0	0	6.72	0	0	3.01E-12
266632	'Irak4'	69.3	20.5	50.3	0	12.5	399.5	0	301	223	195	0.021491
268288	'Samd3'	8.9	3.2	10.41	0	5.24	87.08	3.24	4.25	56.7	82	0.0157809
268379	'Abca13'	0	0	0	0	0	0	1.23	0	0	0	7.94E-14
269053	'Gpr152'	0	0	0	0	0	0	0	0	0	1.08	8.79E-12
27220	'Cartpt'	341	616.6	449.3	260	153	26.1	8.57	28.8	332	74.5	0.0268095
27411	'Slc14a2'	0	0	0	0	0	0	0	0	0	8.34	3.08E-13
27643	'Ubl4a'	28.5	32.74	39.59	79.6	50.6	71.55	73.1	63.7	74.7	60.6	0.0294218
27883	'Tango2'	104	113.6	172.7	126	175	273.4	250	249	248	179	6.24E-05
279706	'Nup62cl'	0	0	0	21.4	0	0	0	0	0	31.1	0.9062676
29870	'Gtse1'	0	0	0	8.11	2.14	0	0	0	0	0	1.92E-15
30806	'Adamts8'	0	0	0	0	0	0	0	0	0	0.94	8.79E-12
30878	'Apln'	1.12	2.9	2.78	0	3.41	0	0	0	0	0	1.38E-06
30941	'Usp21'	54.1	31.8	25.28	5.05	38.1	16.96	18.4	24	15.3	12.8	0.117979
317757	'Gimap5'	0	0	0	0	0	0	0	0	0	3.52	3.81E-12
319259	'Bricd5'	0	0	0	0	0	0	13.8	0	0	0	2.19E-13
319433	'Serpine3'	0	0	0	0	0	0	0.04	0	3.64	0	6.01E-12
319776	'Tmem72'	0	0	0	0	3.09	0	0	0	0	0	3.65E-14
320309	'1520401A03Rik'	0	0	0	0	0	2.59	0	0	0	0	1.84E-12
320701	'Fam19a4'	0	12.71	10.75	0	0	0	0.02	0	0.02	0	2.25E-04
320747	'Lingo4'	0	1.46	1.26	0	2.45	0	0.02	0	0	0	0.0020198
320981	'Enpp6'	0	0	0	0	5.12	0	0	0	0	0	2.02E-14

328258	'Slc25a48'	0	0	0	0	0	0	0	0	0	3.23	5.91E-12
328643	'Vwa5b2'	6.9	7.38	15.62	0.97	13.1	2.22	3.66	5.78	4.61	2	0.0406949
328779	'Hs3st6'	3.53	0	0	0	0	0	0	0	0	0	5.25E-12
329064	'Pkd2l1'	1.15	0	0	0	0	0	0	0	0	0	1.62E-12
329738	'Aknad1'	0	2.89	0	0	0	0	0	0	0	0	3.01E-13
330050	'Fam185a'	20.5	15.79	15	15.2	9.61	5.7	8.69	5.32	6.8	7.33	7.70E-06
333669	'Gm5134'	0	0	0	0	2.29	0	0	0	0	0	2.78E-13
338403	'Cndp1'	0	0	1.6	0	0	0	0	0	0	0	0.0377994
353344	'Opn5'	0	0	0.03	0	0	0	0	0.02	25	22.8	4.07E-06
380924	'Olfm4'	0	0	4.66	0	0	0	0	0	0	0	3.56E-14
381201	'Ap5b1'	0.02	0	0	0	3.48	0	0	0	0	0	6.27E-14
381293	'Kif14'	0	0	0	0	0	0	0	0	0.53	0	1.45E-11
381319	'Batf3'	0	0	0	0	0	6.21	0	0	0	0	8.90E-12
381546	'Ccdc24'	0	0	0	7.87	0	0	0	0	0	3.5	0.7031542
384071	'Slc25a34'	0	0	0	0	0	0	0	0	0	3.31	7.66E-13
384198	'Fam47e'	0	0	0	0	0	0	0	0	3.76	0	2.01E-12
385354	'Frmd7'	0	0	0	0	0	0	1.67	0	0	0	1.29E-12
386753	'Dbpht2'	41.2	72.72	34.37	107	67.7	14.74	29.3	23.4	15.1	28	1.36E-05
399599	'Ccdc87'	0	0	0	0	0	2.35	0	1.11	0	0	0.0079868
403180	'Ccdc121'	0	0	0	0	0	0	0	0.02	0	5.82	3.74E-11
406219	'Krt87'	0	0	0	0	0	0	0	0	4.67	0	6.84E-11
408059	'BC049352'	0	5.32	0.06	0	0	0	0	0	0	0	9.39E-14
432677	'Vrtn'	0	0	0	0	2.79	0	0	0	0	0	1.24E-13
434215	'Lrrc32'	0	0	0	0	0	0	0	0	3.77	0	5.19E-13
435529	'Adgrf2'	0	0	0	0	0	0	2.05	0	1.19	0	4.83E-14
503491	'Defa24'	0	0	0	0	0	0	43.5	0	0	0	1.15E-13
50706	'Postn'	0	0	0	0	0	0	0	0	7.12	0	3.51E-14
52020	'Umodl1'	0.68	0	0	0	0	0	0	0	0	0	2.54E-12
52551	'Sgta'	137	156.9	152.7	201	137	184.1	248	230	163	234	0.0067916
52570	'Ccdc69'	0	0	3.24	0	0	0	0	0	0	0	1.03E-12
53419	'Corin'	0.03	3.01	0.02	0	0.01	0.01	0	0	0.01	0	0.0024889
53867	'Col5a3'	0	0	1.67	0	0	0	0	0	0	0	6.26E-14
53868	'Rab25'	0	0	0	0	16.8	0	0	0	0	0	9.16E-15
53945	'Slc40a1'	0	0	0.01	0	0.03	0.12	3.31	0	1.83	1.94	9.81E-05
54485	'Dll4'	0	0	0	0	0	0	0	0	2.23	0	1.44E-12
545055	'Cma2'	4.03	0	0	0	0	0	0	0	0	0	1.48E-12
545758	'Gm5868'	0	2.87	0	0	0	0	0	0	0	0	4.90E-12
54624	'Paf1'	52	64.97	44.88	32.4	57	104.6	132	69.4	97.6	77.2	2.15E-04
546519	'Tmem235'	0	0	0	0	0	0	0	0	4.9	2.49	2.16E-13
56050	'Cyp39a1'	0	0	0	0.64	0	0	0	1.03	0	0	0.7885752
56183	'Nmu'	0	0	0	0	0	0	0	17.9	0	0	1.63E-13
56212	'Rhog'	3.14	19.37	26.86	0	17.9	1.22	0.04	1.36	3.61	3.42	0.0266861
56318	'Acpp'	0	0	0	0	2.03	0	0	0	0	0	7.99E-14
56460	'Pkp3'	0	0	0	0	0	0	0	0	2.51	5.03	4.21E-14
56485	'Slc2a5'	0	0	0	0	0	6.24	0	0	2.72	1.71	3.63E-04
56643	'Slc15a1'	0	0	0	0	0	0	0	1.07	0	0	1.83E-11
56720	'Tdo2'	0	0	0	0	0	0	0	0	3.23	0	5.44E-12
56735	'Krt71'	0	0	0	0	0	0	9.5	0	0	0	5.33E-14
56811	'Dkk2'	0	0	0	0	0	0	0	0	1.61	1.77	2.53E-13
57444	'Isg20'	0	0	0	0.07	0	0	0	5.38	0	0	0.0221525
58185	'Rsad2'	0	1.88	1.97	0.62	3.44	0	0	0	0	0.01	2.53E-06
58203	'Zbp1'	0	0	0	0	0	14.57	0	0	0	0	3.64E-13
59058	'Bhlhe22'	0	0	0	0	0	2.12	0.25	0.42	1.59	0	5.05E-04
59083	'Fetub'	0	3.03	0	0	0	0	0	0	0	0	1.40E-12
60363	'Cldn15'	0	0	2.45	0.87	0	0	0	0	0	0	0.0178771
619332	'4933416C03Rik'	0	0	0	0	0	0	2.28	0	0	0	5.23E-12
620292	'Cntnap5c'	0.81	1.7	6.93	11.4	3.31	8.38	31.9	6.83	28.7	20.9	0.001538
622307	'5830473C10Rik'	1.51	0	0	0	0	0	0	0	0	0	1.88E-12
622554	'Majin'	0	0	2.6	0	0	0	0	0	0	0	1.67E-13
626215	'Gm6657'	0	0	0	0	0	0	0	0	1.06	0	1.87E-11
626578	'Gbp10'	0	0	0	0	2.55	0	0	0	0	0	2.14E-13

631323	'Gm12250'	1.7	0	0	0	0	0	0	0	0	0	9.14E-13
631797	'Fer1l6'	0	1.11	0	0	0	0	0	0	0	0	3.13E-13
636931	'Trim71'	0	0	0	0	0	0	0	0	1.54	0	1.22E-12
64008	'Aqp9'	0	0	0	0	0	0	3.68	0	0	0	2.01E-11
64009	'Syne1'	13.3	17.67	9.46	32.2	17.8	22.23	23.5	18.5	51.3	46.4	0.0120642
64074	'Smoc2'	2.79	0	21.14	0	0	0	0	0	0	0	7.97E-19
64075	'Smoc1'	21.9	42.68	25.45	1.83	22.3	3.85	14.7	5.73	9.74	14.7	0.0514525
64899	'Lpin3'	0	0	0	0	0	2.36	0	0	2.51	1.03	0.0014822
654821	'Gcnt7'	0	0	0	0	0.02	2.46	4.59	0	0.29	0.02	6.52E-04
65971	'Tbata'	0	2.98	0	0	0	0	0	0	0	0	1.25E-12
66442	'Spc25'	0	3.08	0	0	0	0	0	0	0	0	1.08E-12
665095	'Cyp2j8'	1.91	0	0	0	0	0	0	0	0	0	0.0411123
665574	'Gm7694'	1.47	1.95	4.64	0	0	0	0	0	0	0	2.01E-04
66765	'4933411K16Rik'	0	0	0	0	0	0	0	0	0	3.13	8.79E-12
667663	'Myo3a'	0	0	0.68	0	0	0	0	0	0	0	0.0353757
66864	'Clec14a'	0	0	0	0	0	0	2.73	0	0	0	1.35E-12
66940	'Shisa5'	69.5	94.16	63.33	157	61.5	153.2	95.9	154	205	194	0.0038767
66953	'Cdca7'	0	0	0	0	0	0	3.78	0	0	2.59	1.36E-13
67109	'Zfp787'	0.19	0.28	0.1	1.36	0.39	4.17	1.02	0.96	2.55	1.58	0.0229686
67375	'Qprt'	0	5.3	0	0	0	0	0	0	0	0	3.42E-13
67498	'Kcnv1'	1.53	4.25	0.95	0.01	0.03	0.02	0.01	0.01	0.01	0.02	5.93E-05
67666	'Hapln3'	0	0	0	0	0	0	5.71	0	0	0	1.55E-12
67749	'Mgarp'	0	0	0	0.05	6.58	0	0	0	0	0	1.33E-13
67849	'Cdca5'	0	0	1.62	0	0	0	0	0	0	0	0.0412546
67859	'Cysrt1'	0	0	0	0	0	8.91	0	0	0	0	1.93E-12
68021	'Bphl'	39.4	23.47	32.5	24.5	30.2	63.57	62.5	53.6	57.2	56.3	8.65E-06
68172	'Rpl39l'	0	9.36	0	0	0	0	0	0	0	0	2.07E-13
68279	'Mcoln2'	0	0	1.32	0	0	0	0	0	0	0	1.14E-12
68375	'Ndufa8'	508	460.2	473.6	550	446	784.7	539	664	734	659	3.04E-04
68792	'Srpx2'	0	0	0	0	0	0	3.2	0	0	0	7.38E-13
68857	'Dtwd2'	0.62	0.15	0.86	0	1.55	0	0	0.01	0	0	0.0020127
69068	'Tcim'	0	0	0	0	0	0.04	0	0	5.03	0	2.51E-12
69121	'Chrdl2'	0	0	3.07	0	0	0	0	0	0	0	9.62E-13
69123	'Eci3'	0	0	0	0	2.94	0	0	0	0	0	1.66E-12
69146	'Gsdmd'	0	0	0	0	0	0	0	0	0	2.97	4.57E-12
69387	'Dnajb13'	0	0	0	0	0	0	0	0	0	4.26	6.40E-12
70316	'Ndufab1'	467	428.7	431.8	362	405	616.2	512	528	611	574	2.64E-04
70866	'Slco6d1'	0	0	0	0	0	0	0	0	0	1.35	1.27E-11
70989	'Jhy'	0	0	0	0	3.45	0	0	0	0	0	4.53E-14
71093	'Atoh8'	0	0	0	0	2.31	0	0	0	0	0	5.08E-13
71355	'Col24a1'	0	0	1.2	0	0	0	0	0	0	0	4.08E-13
71367	'Chst9'	0	0	3.01	0	0	0	0	0	0	0	1.03E-11
71724	'Aox3'	0	0	0	0	0	0	0	0	0	1.66	2.22E-12
71839	'Osgin1'	0	0	0	0	0	0	2.25	0	0	0	8.76E-12
71862	'Gpr160'	0	0	0	0.03	0	9.06	0	0	0	0	0.0055067
71872	'Aox4'	0	0	0	0	0	0	0	1.77	0	0	7.26E-13
71874	'2310007B03Rik'	0	0	0	0	0	0	0	0	0	1.73	1.10E-11
71951	'Gpc2'	2.72	1.44	0.89	0	0.19	0	0	0	0	0	5.83E-04
72112	'Ppp1r14d'	0	0	0	0	4.96	0	0	0	0	0	0.0338858
72169	'Trim29'	0	0	0	0	0	0	0	1.05	0	0	2.41E-11
72293	'Nkd2'	0	0.01	0	0	0	0	0	2.45	8.34	0.01	1.54E-04
72388	'Ripk4'	0	0	0	0	1.05	0	0	0	0	0	1.72E-12
72392	'Tmem175'	35.9	52.38	33.58	42.9	38.9	73.12	51.5	79.3	64.9	68.3	1.86E-05
72415	'Sgo1'	0	0	0	0	0	0	0	0	3.36	0.63	1.66E-13
72512	'Tmem173'	0	0	0	0	0.02	26.37	0	0	0.02	0	2.88E-04
72535	'Aldh1b1'	11.8	4.77	2.98	4.81	7.62	28.02	20.3	12.1	24.4	17.7	1.06E-04
73149	'Clec4a3'	0	0	0	0	0	3.54	0	0	0	0	9.47E-12
73167	'Arhgap8'	0	0	0	0	0	0	5	0	3.63	0	2.45E-13
73363	'1700056E22Rik'	0	0	0	0	0	0	4.78	0	0	0	1.10E-11
73713	'Rbm20'	1.75	0.17	0.04	0.02	0.94	0.01	0	0.01	0.01	0.01	1.25E-05
73936	'Ccdc175'	0	0	0	0	2.49	0	0	0	0	0	2.47E-13

74044	'Ttf2'	0	0	0	0	0	0	0	0.64	1.89	0	0.0089332
74131	'Sash3'	1.13	0	0	0	0	0	0	0	0	0	0.0411123
74152	'Stra6l'	0	0	0	0.02	0	0	8.23	0	0	0.01	0.0026536
74153	'Uba7'	0	0	0	0	0	10.29	0.2	0	0	2.62	1.07E-04
74156	'Acot12'	0	1.87	0	0	0	0	0	0	0	0	4.62E-13
74186	'Ccdc3'	0	0	0	0	0	0	0	4.96	0	0.02	1.84E-13
74191	'P2ry13'	0	0	0	0	0	17.05	0	0	0	0	2.52E-15
74204	'Xpo6'	30.2	98.04	9.7	41.1	90.4	16.55	7.34	10.6	10.9	23.2	3.18E-04
74732	'Stx11'	0	0	0	0	0	0	0	0	0	2.38	4.99E-12
74734	'Rhoh'	0.01	0.01	0.01	0.02	0.01	7.51	0	0	0.67	2.36	1.54E-05
75526	'Eppin'	0	5.32	0	0	0	0	0	0	0	0	1.74E-12
75766	'Dcstamp'	0	0	0	0	0	0	0	2	0	0	1.48E-11
76072	'Rnf183'	2.69	0	0	0	0	0	0	0	0	0	1.14E-11
76405	'1700018B08Rik'	0	0	0	0	1.9	0	0	0	0	0	0.0369408
76415	'Fam187b'	0	0	0	0	3.77	0	0	0	0	0	5.27E-13
76507	'Aoc1'	0	1.82	0	0	0	0	0	0	0	0	5.89E-13
76589	'Unc5cl'	0	0	0	0	0	0	0	0	1.38	0	1.40E-11
76681	'Trim12a'	0	0	0	0	0	0	6.1	0	0	0	7.64E-13
76854	'Gper1'	0	0	0	0	0	0.02	0	0	0	3.89	5.58E-13
76856	'Catsper3'	0	0	0	0	0	0	0	0	2.83	0	2.43E-11
77799	'Sla2'	0	0	0	0	0	1.35	0	0	0	0	1.17E-11
77998	'Grifin'	0	0	0	0	0	0	8.38	0	0	0	6.56E-12
78088	'Sowahb'	0	0	0	6.04	0	0	0	0.98	0	0	0.3835219
78895	'Pus7l'	0	0	0	0	0	0	0	0	2.33	2.03	2.66E-13
80986	'Ckap2'	0	1.61	0	0	0	0	0	0	0	0	1.16E-12
83428	'Ucn3'	0	0.03	0.07	0.04	0.03	111.8	0	0	207	27.1	2.24E-07
83453	'Chrdl1'	0	0	1.98	0	0	0	0	0	0	0	1.41E-13
84004	'Mcam'	0.08	0.1	6.8	0	0.07	0.02	0	0	0	0	4.48E-04
93695	'Gpnmb'	1.63	0	0	0	0	0	0	0	0	0	3.24E-13
93709	'Pcdhga1'	0	2.53	3.48	0	0	0	0	0	0	0	1.83E-15
93837	'Dach2'	0	0	0	0	0	2.01	0	0	0	0.01	3.38E-12
93873	'Pcdhb2'	2.8	0	0	0	0	0	0	0	0	0	1.55E-13
94088	'Trim6'	0	0	0	0	0	0	0	0	0	1.92	1.44E-12
94220	'Cnnm4'	6.2	6.46	4.82	3.17	9.86	1.57	1.53	1.73	3.61	3.31	0.0031149
99382	'Abtb2'	2.97	1.19	3.95	3.34	0.01	0	0	0.01	0.01	0	2.97E-09
