# Supplementary material for: A temperature-regulated circuit for feeding behavior
Source: Nat Commun. 2022 Jul 22;13:4229. doi: 10.1038/s41467-022-31917-w (PMC9307622; doi:10.1038/s41467-022-31917-w)
Supplement: Supplementary file 7 — Reporting Summary [file 41467_2022_31917_MOESM7_ESM.pdf]

nature portfolio

Corresponding author(s): Yi Zhou, Ying Xiong, Shaowen Qian

Last updated by author(s): Jul 7, 2022

Reporting Summary

Nature Portfolio wishes to improve the reproducibility of the work that we publish. This form provides structure for consistency and transparency in reporting. For further information on Nature Portfolio policies, see our Editorial Policies and the Editorial Policy Checklist.

Statistics

For all statistical analyses, confirm that the following items are present in the figure legend, table legend, main text, or Methods section.

n/a	Confirmed	
☐	☒	The exact sample size (*n*) for each experimental group/condition, given as a discrete number and unit of measurement
☐	☒	A statement on whether measurements were taken from distinct samples or whether the same sample was measured repeatedly
☐	☒	The statistical test(s) used AND whether they are one- or two-sided<br>*Only common tests should be described solely by name; describe more complex techniques in the Methods section.*
☒	☐	A description of all covariates tested
☐	☒	A description of any assumptions or corrections, such as tests of normality and adjustment for multiple comparisons
☐	☒	A full description of the statistical parameters including central tendency (e.g. means) or other basic estimates (e.g. regression coefficient) AND variation (e.g. standard deviation) or associated estimates of uncertainty (e.g. confidence intervals)
☐	☒	For null hypothesis testing, the test statistic (e.g. *F*, *t*, *r*) with confidence intervals, effect sizes, degrees of freedom and *P* value noted<br>*Give P values as exact values whenever suitable.*
☒	☐	For Bayesian analysis, information on the choice of priors and Markov chain Monte Carlo settings
☒	☐	For hierarchical and complex designs, identification of the appropriate level for tests and full reporting of outcomes
☒	☐	Estimates of effect sizes (e.g. Cohen's *d*, Pearson's *r*), indicating how they were calculated

Our web collection on statistics for biologists contains articles on many of the points above.

Software and code

Policy information about availability of computer code

Data collection	We listed all softwares used in the experiments and for analysis in the Methods section. We used a FLIR ONE to collect infrared images. Confocal images were captured using ZEISS Viewer (ZEN 3.3).
Data analysis	Matlab 2017b, GraphPad Prism 7, SPSS 18.0, HISAT2 (v2.0.4), Dr. Tom system, ZEISS Viewer (ZEN 3.3). Customized Matlab code were used to analyze the fiber photometry signal and can be accessible (https://github.com/qswenwen/Fiber-Photometry-preprocessing).

For manuscripts utilizing custom algorithms or software that are central to the research but not yet described in published literature, software must be made available to editors and reviewers. We strongly encourage code deposition in a community repository (e.g. GitHub). See the Nature Portfolio guidelines for submitting code & software for further information.

Data

Policy information about availability of data

All manuscripts must include a data availability statement. This statement should provide the following information, where applicable:

- Accession codes, unique identifiers, or web links for publicly available datasets
- A description of any restrictions on data availability
- For clinical datasets or third party data, please ensure that the statement adheres to our policy

The RNA-seq data have been deposited in the database of the NCBI Sequence Read Archive (https://www.ncbi.nlm.nih.gov/sra/) under the accession number PRJNA820979. All raw data generated from RNA-sequencing are freely available from the NCBI. Source data are provided with this paper.The mouse genome

(mm10, build name GRCm38) can be accessible through NCBI Assembly (https://www.ncbi.nlm.nih.gov/assembly/). RefSeq assembly accession: GCF_000001635.26. The version of KEGG pathway database is 93.0, and can be accessible through KEGG databases (https://www.genome.jp/kegg/pathway.html).

Human research participants

Policy information about studies involving human research participants and Sex and Gender in Research.

Reporting on sex and gender	*Use the terms sex (biological attribute) and gender (shaped by social and cultural circumstances) carefully in order to avoid confusing both terms. Indicate if findings apply to only one sex or gender; describe whether sex and gender were considered in study design whether sex and/or gender was determined based on self-reporting or assigned and methods used. Provide in the source data disaggregated sex and gender data where this information has been collected, and consent has been obtained for sharing of individual-level data; provide overall numbers in this Reporting Summary. Please state if this information has not been collected. Report sex- and gender-based analyses where performed, justify reasons for lack of sex- and gender-based analysis.*
Population characteristics	*Describe the covariate-relevant population characteristics of the human research participants (e.g. age, genotypic information, past and current diagnosis and treatment categories). If you filled out the behavioural & social sciences study design questions and have nothing to add here, write "See above."*
Recruitment	*Describe how participants were recruited. Outline any potential self-selection bias or other biases that may be present and how these are likely to impact results.*
Ethics oversight	*Identify the organization(s) that approved the study protocol.*

Note that full information on the approval of the study protocol must also be provided in the manuscript.

Field-specific reporting

Please select the one below that is the best fit for your research. If you are not sure, read the appropriate sections before making your selection.

☒ Life sciences ☐ Behavioural & social sciences ☐ Ecological, evolutionary & environmental sciences

For a reference copy of the document with all sections, see nature.com/documents/nr-reporting-summary-flat.pdf

Life sciences study design

All studies must disclose on these points even when the disclosure is negative.

Sample size	Our sample-size are comparable with sample sizes in the literatures (e.g. Zheng-Dong Zhao et al., Proc Natl Acad Sci 2017, Chan Lek Tan et al., Cell 2016, Amber L. Alhadeff et al., Cell 2018, Tohru M. Takahashi et al., Nature 2020). Sample size was determined to be adequate based on the magnitude and consistency of measurable differences between groups. The sample size (n) of each experiment is provided in the corresponding figure captions in the main manuscript. Sample sizes were chosen to support meaningful conclusions in accordance with ethical committee requirements to limit as much as possible the use of animals.
Data exclusions	If postmortem studies showed evidence of missed injection, death, optical fiber falling off or implantation, mice were excluded from further analysis. Specifically, in the optogenetic experiment, 3 mice for terminal activating ARC, 5 mice for terminal activating PVH, 4 mice for terminal activating DMH, were excluded due to missed injection, death or fiber falling off. In the genetic ablating experiment, 4 mice for PVH ablating, 3 mice for ARC ablating were excluded due to missed injection or death. In the chemogenetic experiment, 2 mice for PVH inhibiting, 2 mice for ARC inhibiting were excluded due to missed injection or death. In the pharmacological injection experiment, 6 mice were excluded due to inaccurate cannula implantation.
Replication	All attempts at replication were successful. Feeding behavior tests in the optogenetic, chemogenetic, genetic ablating and pharmacological injecting experiments, were repeated by 3 times. For feeding test under ambient temperature, each group typically has 15 mice. For C-Fos staining experiment, each group typically has 4 mice. In the fiber photometry experiment, each group has 5 mice or more. In the optogenetic, genetic ablating, and chemogenetic inhibiting experiments, each group has 6 mice or more. In the experiment of rabies tracing, each group has 4 mice. In the RNA-sequencing, each group has 5 mice. In the experiment of pharmacological injection, each group has 14 mice.
Randomization	Animals were randomly assigned to control and treatment groups. For animals with multiple treatments, the sequence of treatments was randomized.
Blinding	Investigators were blinded to the group allocations or data collections in the feeding behavior tests, immunohistochemistry, RNA scope and RNA-sequencing experiments, but were not blinded in the calcium signal experiment, as the calcium signals under different thermal conditions were obviously different, and were automatically displayed in real time by a computer software.

Reporting for specific materials, systems and methods

We require information from authors about some types of materials, experimental systems and methods used in many studies. Here, indicate whether each material, system or method listed is relevant to your study. If you are not sure if a list item applies to your research, read the appropriate section before selecting a response.

Materials & experimental systems

n/a	Involved in the study
☐	☒ Antibodies
☒	☐ Eukaryotic cell lines
☒	☐ Palaeontology and archaeology
☐	☒ Animals and other organisms
☒	☐ Clinical data
☒	☐ Dual use research of concern

Methods

n/a	Involved in the study
☒	☐ ChIP-seq
☒	☐ Flow cytometry
☒	☐ MRI-based neuroimaging

Antibodies

Antibodies used

Primary antibody: anti-cFos (rabbit, ab190289, Abcam)Secondary antibody: goat anti-rabbit 488, ab150077, Abcam; goat anti-rabbit 568, ab175471, AbcamFISH probes: Aplnr (GenBank: NM_011784.3, RNAscope®Probe-Mm-Aplnr, Advanced Cell Diagnostics), GalR1 (GenBank: NM_008082.2, RNAscope®Probe-Mm-Galr1-C2, Advanced Cell Diagnostics) and Fos (GenBank: NM_010234.2, RNAscope®Probe-Mm-Fos-C3, Advanced Cell Diagnostics)

Validation

The primary antibody we used in this study have been validated in mouse brain slices in the following published papers:anti-cFos (rabbit, ab190289, Abcam): Reacts with Mouse, Rat and Human. Validated for use in IHC-FrFl, ICC, WB, and IHC-P.References:Jarvie BC et al. Satb2 neurons in the parabrachial nucleus mediate taste perception. Nat Commun 12:224 (2021). IHC; tested species: mouse.Clawson BC et al. Causal role for sleep-dependent reactivation of learning-activated sensory ensembles for fear memory consolidation. Nat Commun 12:1200 (2021). IHC; tested species: mouse.

Animals and other research organisms

Policy information about studies involving animals; ARRIVE guidelines recommended for reporting animal research, and Sex and Gender in Research

Laboratory animals

Ai9 (Cre-dependent tdTomato reporter) mice (Jackson Laboratories, Stock No. 007909), vGluT2-IRES-Cre mice (Jackson Laboratories, Stock No. 016963), and GAD2-IRES-cre mice (Jackson Laboratories, Stock No. 010802). Mice were housed in a 12h light-dark cycle with ad libitum access to food and water, 50-70% humidity and 18-22°C ambient temperature?. Experiments were performed in both adult male and female mice (6-12 weeks old).

Wild animals

The study did not involve wild animals.

Reporting on sex

Sex was not considered in the study design. Experiments were performed in comparable adult male and female mice.

Field-collected samples

The study did not involve data collected from the field.

Ethics oversight

All experimental procedures were approved by the Animal Care and Use Committee of Army Medical University.

Note that full information on the approval of the study protocol must also be provided in the manuscript.
